# An Integrated Consensus Machine Learning and Structure-Based Workflow for the Discovery of Novel Tankyrase 1 Inhibitors

**DOI:** 10.3390/ph19081310

**Published:** 2026-08-19

**Authors:** Marina Bilotta, Adriana Gargano, Roberta Rocca, Valentina Maggisano, Stefania Bulotta, Stefano Alcaro

**Affiliations:** 1 Dipartimento di Scienze della Salute, Università “Magna Græcia” di Catanzaro, Campus “S. Venuta”, 88100 Catanzaro, Italy; marina.bilotta@studenti.unicz.it (M.B.); a.gargano@unicz.it (A.G.); vmaggisano@unicz.it (V.M.); bulotta@unicz.it (S.B.); alcaro@unicz.it (S.A.); 2 Associazione CRISEA Centro di Ricerca e Servizi Avanzati per l’Innovazione Rurale, Località Condoleo, 88055 Belcastro, Italy

**Keywords:** TNKS1, machine learning, virtual screening, molecular docking, anticancer drug discovery

## Abstract

**Background:** Tankyrase 1 (TNKS1) is a poly(ADP-ribose) polymerase involved in Wnt/β-catenin signaling, telomere maintenance, and genomic stability, making it an attractive therapeutic target in oncology. This study aimed to develop and apply an integrated computational workflow to identify novel TNKS1 inhibitor candidates. **Methods:** A curated dataset of experimentally validated TNKS1 inhibitors and property-matched DUD-E decoys was used to develop a *consensus* supervised machine learning (ML) model prioritization framework for ligand-based virtual screening, integrating Morgan fingerprints with three complementary classifiers. The model screened more than 700,000 compounds, and prioritized hits were evaluated by structure-based virtual screening (SBVS), Prime MM-GBSA binding free-energy refinement, and 500 ns molecular dynamics simulations (MDs). The top candidates were subsequently tested in an in vitro TNKS1 enzymatic inhibition assay. **Results:** The *consensus* ML framework prioritized 670 compounds, yielding five candidates for experimental testing. **Compound 3** displayed the most favorable computational profile and was experimentally confirmed as a TNKS1 inhibitor candidate, exhibiting approximately 80% TNKS1 inhibition at 0.1 μM, whereas the remaining candidates showed only limited activity. **Conclusions:** The proposed workflow efficiently reduced a large chemical space to a focused set of TNKS1 inhibitor candidates while substantially reducing the experimental screening burden. **Compound 3** represents a promising starting point for future structure–activity relationship studies and lead optimization in the context of TNKS1 inhibition. Moreover, this work highlights the value of integrating *consensus* ML, SBVS, and experimental validation to accelerate early-stage hit discovery for TNKS1 and other therapeutic targets.

## 1. Introduction

Tankyrase 1 (TNKS1) is a poly(ADP-ribose) polymerase belonging to the PARP superfamily that plays a pivotal role in the regulation of cellular homeostasis, genomic integrity, and oncogenic signaling pathways [1,2,3]. Through its poly(ADP-ribosyl)ation activity, TNKS1 regulates protein stability and turnover, thereby controlling multiple cellular processes, including telomere maintenance, mitotic progression, DNA damage response, and intracellular signaling regulation [4,5,6]. Among its diverse functions, the modulation of the Wnt/β-catenin pathway is particularly relevant in cancer biology [7,8]. By promoting the PARylation-dependent destabilization of AXIN proteins, TNKS1 contributes to the disassembly of the β-catenin destruction complex, thereby sustaining aberrant Wnt signaling associated with tumor initiation and progression in several malignancies, including colorectal, gastric, ovarian, and hepatocellular carcinomas [9,10,11]. 

The therapeutic relevance of TNKS1 has stimulated considerable interest in the development of catalytic tankyrase inhibitors capable of modulating Wnt-driven oncogenic processes [12,13]. XAV939, one of the most extensively characterized tankyrase inhibitors, demonstrated the feasibility of targeting the catalytic PARP domain and established the structural basis for subsequent TNKS1-directed drug discovery campaigns [14,15]. Nevertheless, despite the growing number of reported tankyrase inhibitors, the identification of novel TNKS1 scaffolds with favorable interaction profiles and suitable pharmacological properties remains a major challenge in medicinal chemistry [6,16]. Despite the increasing number of reported tankyrase inhibitors, most compounds still belong to a limited number of scaffold classes and frequently exhibit suboptimal selectivity profiles and/or inadequate physicochemical and pharmacokinetic properties, underscoring the need for alternative and more innovative discovery strategies to efficiently explore broader regions of chemical space and improve drug-like characteristics [6,17]. 

Recent advances in artificial intelligence (AI) and machine learning (ML) have profoundly reshaped early-stage drug discovery by enabling the rapid exploration of large molecular datasets and the prioritization of candidate compounds with increased efficiency [18,19]. In particular, supervised learning algorithms have demonstrated remarkable capability to capture complex, nonlinear structure–activity relationships that are often challenging to resolve using conventional virtual screening (VS) methods alone, enabling more accurate and data-driven prioritization of active compounds [20,21,22]. Among these methodologies, supervised machine learning algorithms such as Extreme Gradient Boosting (XGBoost), Support Vector Machines with a radial basis function kernel (SVM-RBF), and Logistic Regression have demonstrated excellent predictive performance in cheminformatics applications owing to their robustness, scalability, and ability to capture complex molecular representations [23,24,25]. Although data-driven methodologies provide powerful prioritization tools, structural characterization remains essential for validating ligand recognition mechanisms and assessing the reliability of predicted binding modes [26,27]. Structure-based computational techniques, including molecular docking, energetic refinement, and molecular dynamics (MD) simulations, provide complementary insights into ligand binding modes, the stability of ligand–target interactions over time, and conformational stability within the binding site, thereby improving the reliability of structure-based drug design workflows [28,29]. The integration of predictive ML models with atomistic structure-based analyses therefore represents a particularly attractive strategy for supporting compound prioritization during computational screening campaigns [18]. 

**Figure 1:**
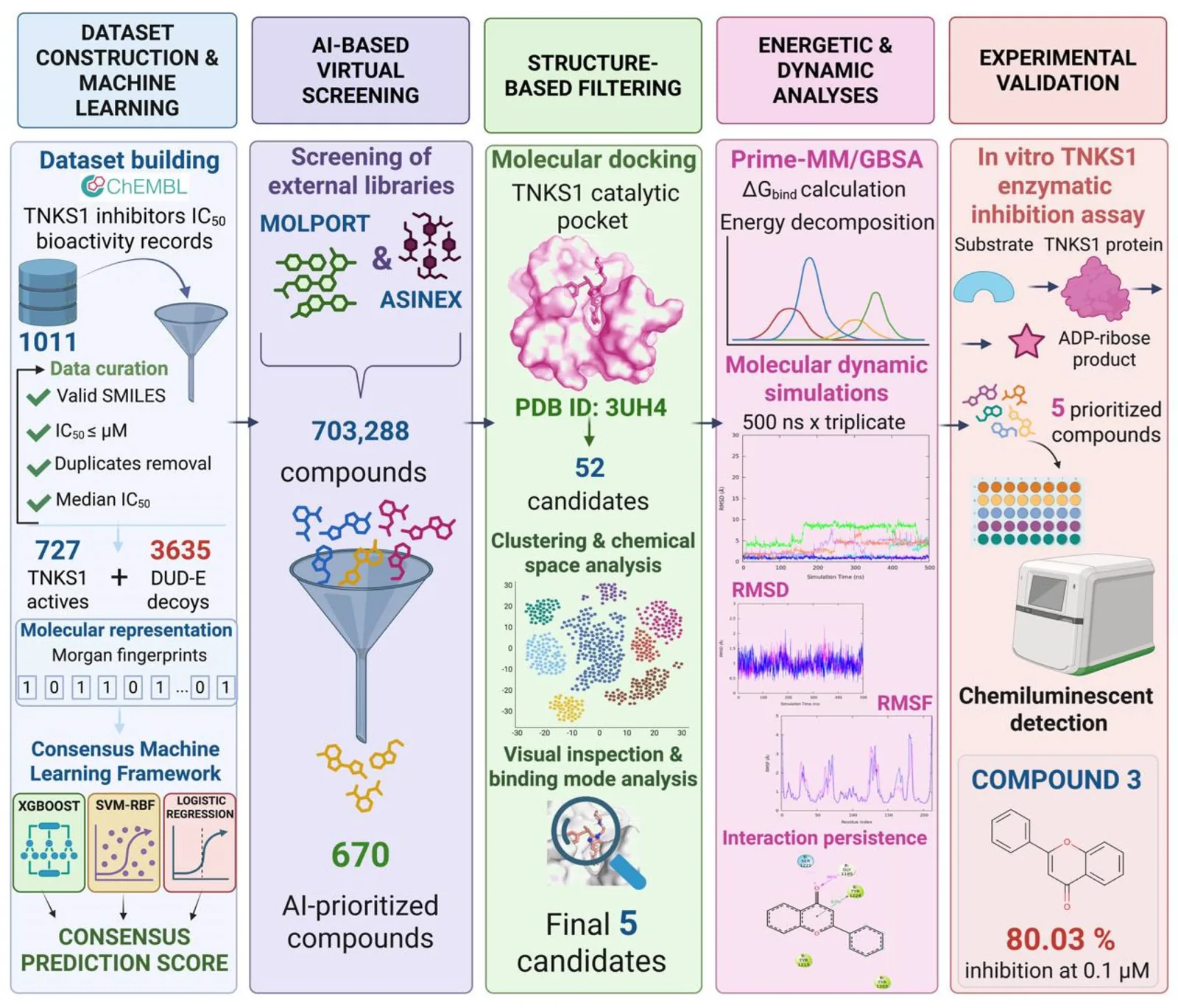
**Overview of the integrated workflow for TNKS1 inhibitor discovery.** The approach combines dataset curation, consensus ML-based VS, structure-based computational analyses, MD simulations, and in vitro enzymatic validation for the identification and prioritization of novel TNKS1 inhibitor candidates.

In the present study, we developed a multi-stage computational workflow integrating supervised machine learning, structure-based virtual screening (SBVS), binding pocket characterization, energetic refinement, and MDs to identify novel TNKS1 inhibitor candidates. A curated dataset of TNKS1 inhibitors and property-matched decoys was used to train three supervised machine learning classifiers based on Morgan fingerprints. The resulting framework was applied to large-scale VS to prioritize compounds predicted to display molecular features consistent with known TNKS1 inhibitors. Selected candidates were subsequently evaluated through molecular docking, Prime MM-GBSA refinement, and MD simulations to assess binding stability within the TNKS1 catalytic pocket. Finally, the top-ranked compounds were experimentally evaluated using an in vitro TNKS1 enzymatic assay, enabling the identification of a promising inhibitor candidate. The overall study design, integrating ML-based prioritization, SBVS, MDs, and experimental enzymatic validation is summarized in Figure 1.

## 2. Results

### 2.1. Dataset Construction and Machine Learning Model Development

To develop a robust ligand-based framework for the identification of novel TNKS1 inhibitors, a carefully curated dataset was assembled and used to train and evaluate supervised machine learning models. Following data curation and preprocessing, the final dataset comprised 727 experimentally validated TNKS1 inhibitors and 3635 property-matched decoys, corresponding to an active-to-decoy ratio of 1:5. The resulting collection captured substantial chemical diversity while minimizing redundancy and structural inconsistencies, thereby providing a reliable basis for model development. To ensure rigorous performance assessment, the curated dataset was partitioned using a stratified splitting strategy that preserved the active-to-decoy ratio across the training, validation, and independent test sets. A scaffold-based partition was not applied, as the primary objective of this work was prospective hit prioritization rather than benchmarking model generalization across distinct chemical scaffolds.

This resulted in 3053 (509 actives and 2544 decoys), 654 (109 actives and 545 decoys), and 655 compounds (109 actives and 546 decoys) for the training, validation, and independent test sets, respectively. 

Molecular structures were encoded as circular Morgan fingerprints [30] to capture topological and substructural features relevant to TNKS1 inhibitory activity. Three supervised classification algorithms, XGBoost [26], Logistic Regression [31], and a SVM-RBF [32], were trained and evaluated to assess their predictive performance. Because the negative class consisted of property-matched DUD-E decoys rather than experimentally confirmed inactive compounds, the resulting models should be interpreted as prioritization tools for ligand-based virtual screening rather than direct predictors of TNKS1 inhibitory activity. Model configurations were defined during model development and consistently applied across the training, validation, and independent test stages. The complete hyperparameter settings and implementation details are reported in Tables S1 and S2.

**Figure 2:**
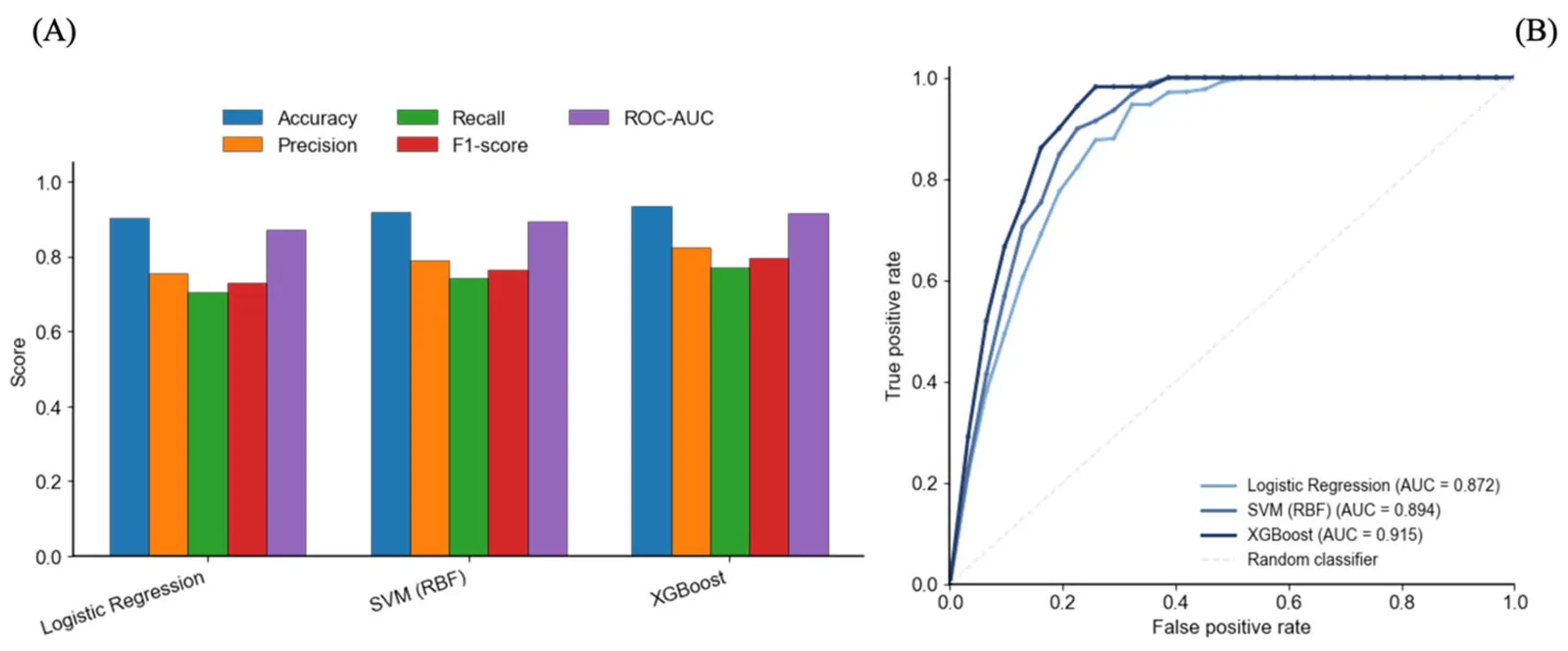
**Performance comparison of the machine learning models.** (**A**) Bar plot summarizing the predictive performance of the evaluated classifiers, Logistic Regression, Support Vector Machine with radial basis function kernel (SVM-RBF), and XGBoost, in terms of accuracy, precision, recall, F1-score, and ROC-AUC. (**B**) Receiver operating characteristic (ROC) curves for the three models, illustrating their discriminative ability.

As summarized in Figure 2A, all three models exhibited consistently strong predictive performance on the independent test set, achieving accuracies between 0.91 and 0.93 and receiver operating characteristic–area under the curve (ROC-AUC) values ranging from 0.87 to 0.92. Logistic Regression achieved an accuracy of 0.913, a precision of 0.755, a recall of 0.706, an F1-score of 0.730, and a ROC-AUC of 0.872. The SVM-RBF model further improved predictive performance, yielding an accuracy of 0.924, a precision of 0.786, a recall of 0.743, an F1-score of 0.764, and a ROC-AUC of 0.894. XGBoost delivered the highest overall performance, with an accuracy of 0.934, a precision of 0.824, a recall of 0.771, an F1-score of 0.796, and a ROC-AUC of 0.915 (Table S3). The corresponding confusion matrices and precision–recall curves for the three classifiers on the independent test set are provided in Figures S5 and S6, respectively. The ROC curves shown in Figure 2B further confirmed the discriminative ability of all three classifiers, with each model exhibiting a clear separation from the random-classification baseline and rapidly approaching the upper-left corner of the ROC space. Although XGBoost achieved the highest predictive performance across all evaluated metrics, the relatively small differences among the three algorithms suggest that each classifier captured complementary aspects of the structure–activity relationships underlying TNKS1 inhibition.

Collectively, these results justified retaining all three for the subsequent consensus-based virtual screening workflow. Each classifier was assessed independently on the validation and independent test sets using a probability threshold of 0.5 for binary classification. The consensus strategy was subsequently applied only during external virtual screening as a decision-level prioritization procedure and was not implemented as an additional independently trained classifier.

### 2.2. AI-Driven Virtual Screening and Consensus-Based Prioritization

Following model validation, the consensus ML framework was applied to the large-scale screening of two chemically diverse external libraries comprising 276,517 natural products from the ASINEX database [33] and 426,771 commercially available compounds from the MolPort dataset [34]. In total, 703,288 molecules were screened, covering a broad and structurally heterogeneous chemical space. The primary objective of this stage was to efficiently reduce the searchable chemical space while retaining high-confidence candidates predicted to inhibit TNKS1. 

To maximize prioritization confidence, the three independently validated classifiers (Logistic Regression, SVM-RBF, and XGBoost) were applied in parallel. For each compound, the arithmetic mean of the three predicted probabilities was calculated and used as the consensus score for ranking. Compound retention was governed by a separate stringent agreement rule: only molecules assigned an active-class probability greater than 0.90 by each of the three individual classifiers were retained for subsequent structure-based evaluation. This stringent agreement-based filtering strategy yielded 670 AI-prioritized compounds, corresponding to approximately 0.095% of the screened libraries, thereby substantially reducing the searchable chemical space and generating a focused collection of candidates consistently supported by all three classifiers. The complete prediction results for the 670 AI-prioritized compounds, including the individual model probabilities and consensus scores, are reported in Table S4.

To further characterize the AI-prioritized compounds, the maximum Morgan fingerprint-based Tanimoto similarity relative to the training actives was calculated together with the maximum similarity to previously reported TNKS1 inhibitors. These analyses were performed solely to assess the structural relationship of the prioritized compounds to known active chemotypes and were not used as selection criteria. 

**Figure 3:**
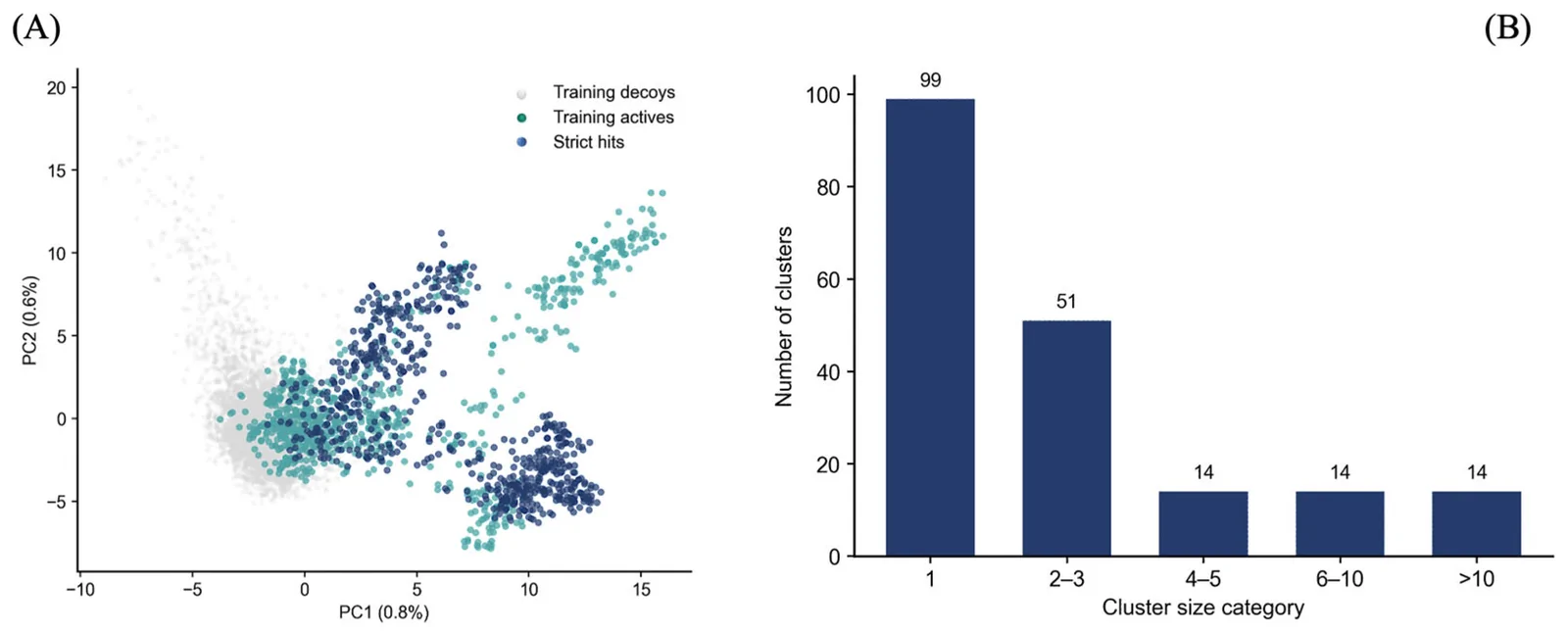
**Structural characterization of the AI-prioritized compound collection.** (**A**) Principal component analysis (PCA) of the chemical space occupied by training compounds and AI-prioritized compounds (670). (**B**) Butina clustering analysis of the 670 AI-prioritized compounds using Morgan fingerprints and a Tanimoto distance threshold of 0.4. The clustering analysis was performed exclusively to describe the structural organization of the retained compounds and was not used as a selection criterion during hit prioritization.

The structural diversity of the AI-prioritized compounds was subsequently assessed by Butina clustering, which identified 192 clusters with a mean cluster size of 3.5 compounds, a median cluster size of 1, and a maximum cluster size of 54 (Figure 3B), indicating the presence of multiple distinct chemotypes. Principal component analysis (PCA) further showed that the prioritized compounds largely overlapped with the region occupied by the training actives while remaining within the chemical space represented by the training set (Figure 3A).

Collectively, these results demonstrate that the consensus machine learning workflow efficiently reduced the initial chemical space to a focused set of structurally diverse, high-confidence candidates suitable for subsequent structure-based evaluation. The SMILES representations, consensus prediction scores, and source information for the five compounds ultimately selected for experimental evaluation are provided in Table S5.

### 2.3. Molecular Docking

To establish a reliable structural model for structure-based screening, the crystallographic structure of TNKS1 was prepared and validated using the Schrödinger Maestro suite (Schrödinger, LLC, New York, NY, USA) [35]. The docking protocol was validated through redocking of the co-crystallized inhibitor XAV939, a well-characterized reference ligand for tankyrase inhibition. Both crystallographic chains (A and B) were independently evaluated to identify the optimal receptor configuration for subsequent virtual screening. Chain B provided a more accurate reproduction of the experimental binding mode, showing a lower redocking RMSD value compared with chain A (0.167 Å vs 0.664 Å), and was therefore selected as the preferred docking model (Figure S1).

The reliability of the docking protocol was confirmed according to the widely accepted criterion of RMSD ≤ 2.0 Å between predicted and crystallographic poses [36], which was satisfied by both chains, with chain B exhibiting superior pose recovery. Based on these results, chain B was selected for all subsequent structure-based analyses. The Glide G-Score obtained from XAV939 redocking (−9.12 kcal/mol) was adopted as the docking score threshold for the VS campaign, and the AI-prioritized compounds identified by the consensus machine learning workflow were docked into the TNKS1 catalytic site.

Compounds with Glide docking scores ≤−9.12 kcal/mol and plausible binding orientations within the catalytic pocket were retained for further evaluation. Their predicted binding modes showed favorable shape complementarity and interaction patterns consistent with the XAV939 reference ligand, including hydrogen bonds, π–π stacking, and hydrophobic contacts. The docking-based filtering reduced the dataset to 52 candidates (Table S6), which were subsequently subjected to visual inspection to assess steric compatibility, binding orientation, and pocket occupancy. The integration of docking score filtering and structural evaluation ultimately led to the identification of five final candidates, displaying G-Scores ranging from −10.02 to −10.57 kcal/mol and consistently occupying favorable regions of the TNKS1 catalytic site (Table 1). 

Despite sharing related core scaffolds, these compounds differ in their peripheral substituents, which may contribute to differences in binding accommodation within the TNKS1 pocket, although the overall interaction pattern is largely conserved. The five selected compounds are characterized by rigid, polyaromatic scaffolds containing diaryl systems and carboxylic acid–bearing functionalities, structural features commonly observed among TNKS1 inhibitors.

Among the selected molecules, **compounds 1**, **2**, **4**, and **5** display a high degree of structural similarity, sharing a common diaryl or biphenyl benzamide scaffold and a conserved terminal carboxylic acid group, which acts as a key anchoring element within the TNKS1 active site. **Compound 1** features a methoxy-substituted phenyl ring connected to the benzamide core, whereas **compound 2** corresponds to a more compact analogue that retains the same carboxylate-mediated binding motif. **Compounds 4** and **5** maintain the same overall structural framework while incorporating subtle peripheral modifications, including para-chloro substitution in **compound 4** and altered methoxy substitution patterns in **compound 5**. These changes are expected to modulate electronic distribution, hydrophobic interactions, and steric properties without disrupting the conserved pharmacophoric arrangement. Collectively, these structural variations preserve the overall binding mode while fine-tuning the local interaction network within the TNKS1 catalytic pocket.

In contrast, **compound 3** exhibits a distinct structural architecture, featuring a flavone-like (dibenzopyran-4-one) scaffold characterized by increased rigidity and planarity. This fused-ring system extends the π-surface and may promote enhanced shape complementarity within the catalytic pocket, representing an alternative binding mode compared with the remaining candidates. Unlike the other compounds, compound 3 does not possess carboxylic acid or amide moieties; instead, its hydrogen-bond accepting potential is exclusively mediated by the carbonyl oxygen atom and the ether oxygen atom. Overall, while all five final compounds share key aromatic and hydrogen-bonding features, they display differences in substitution patterns and scaffold rigidity. 

**Figure 4:**
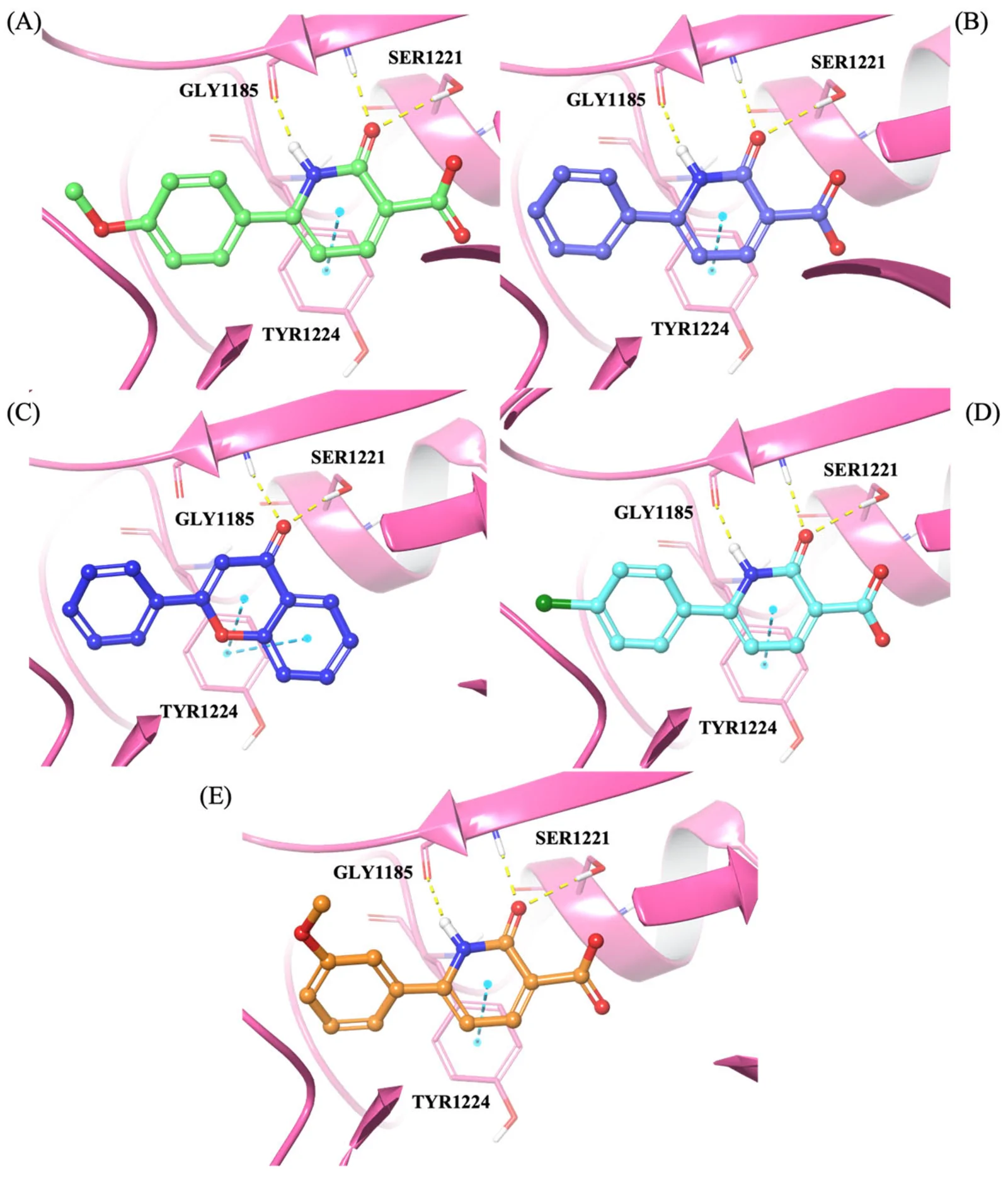
Binding modes of the five final TNKS1 inhibitor candidates within its catalytic pocket: (**A**) **compound 1** (faded yellow-green), (**B**) **compound 2** (faded violet), (**C**) **compound 3** (blue), (**D**) **compound 4** (cyan), and (**E**) **compound 5** (orange). The TNKS1 backbone is shown as a magenta ribbon, with key interacting residues depicted as magenta sticks and ligands represented as colored sticks. Hydrogen bonds and π–π stacking interactions are indicated by yellow and cyan dashed lines, respectively.

As shown in Figure 4A–E, the five compounds occupy comparable regions of the TNKS1 catalytic pocket, although their specific polar interaction patterns differ. **Compounds 1**, **2**, **4**, and **5** share a conserved binding mode in which the terminal carboxylic acid acts as a primary anchoring group through hydrogen bond (H-bond) interactions with SER1221 and GLY1185. Compound 3, which lacks a carboxylic acid moiety, adopts a distinct polar interaction pattern. In the initial static docking pose, its carbonyl oxygen is positioned in proximity to the backbone of GLY1185, although the distance and geometry do not satisfy the criteria for a formal hydrogen bond. Across the series, the aromatic moieties occupy a hydrophobic region near TYR1224, enabling favorable π–π interactions. Thus, while **compounds 1**, **2**, **4**, and **5** display closely related binding poses modulated mainly by peripheral substitutions, **compound 3** adopts a slightly different orientation consistent with its more rigid architecture. Overall, the series is characterized by favorable polar and aromatic interactions that stabilize ligand recognition within the TNKS1 catalytic site.

### 2.4. Prime MM-GBSA Energetic Refinement and Final Hit Selection

To further refine the docking-derived ranking and obtain a more realistic estimation of protein–ligand binding energetics, Prime Molecular Mechanics/Generalized Born Surface Area (MM-GBSA) calculations were performed on the best docking pose of each of the five potential hits identified during the structure-based screening stage in complex with TNKS1. Energetic refinement was carried out using the Schrödinger Prime MM-GBSA workflow [37] on the docking poses generated within the validated chain B catalytic pocket. Unlike docking scoring functions, which primarily evaluate geometric complementarity and empirical interaction terms, MM-GBSA calculations incorporate energetic contributions associated with electrostatic interactions, van der Waals contacts, lipophilic stabilization, molecular packing, and solvation effects, thereby providing a more comprehensive assessment of binding favorability. The energetic refinement revealed appreciable differences among the selected candidates (Table 2). While all five compounds maintained favorable calculated binding free energies, their relative ranking differed from that obtained through docking alone. In particular, compound 3 exhibited the most favorable MM-GBSA binding free energy (ΔG_bind_ = −63.11 kcal/mol), although its Glide docking score was comparable to those of the other candidates. These findings highlight the complementary role of docking and MM-GBSA analyses, indicating that docking scores alone may not fully capture the energetic determinants of protein–ligand binding. Energy decomposition revealed distinct energetic profiles among the selected candidates. **Compounds 1**, **2**, **4**, and **5** showed highly favorable Coulombic contributions (approximately −68 to −70 kcal/mol), together with comparable van der Waals and lipophilic terms, indicating a major contribution of electrostatic and hydrophobic interactions to the overall binding free energy. These effects were partially counterbalanced by unfavorable solvation contributions (approximately 80–85 kcal/mol).

In contrast, **compound 3** exhibited a different energetic profile, characterized by weaker Coulombic interactions but the lowest solvation penalty and the most favorable packing contribution within the series. The combined contribution of these energetic terms resulted in the most favorable predicted binding free energy, suggesting that its ranking was driven by a balanced interplay of electrostatic, desolvation, and packing effects rather than by a single dominant interaction type.

Overall, MM-GBSA refinement provided additional energetic discrimination beyond docking-based ranking, revealing differences among candidates that were not apparent from docking scores alone. 

### 2.5. Molecular Dynamics (MD) Simulations

Triplicate MD simulations of 500 ns were performed to assess the conformational behavior and binding mode of the five TNKS1–ligand complexes selected after docking and MM-GBSA refinement. The time evolution of the ligand root mean square deviation (RMSD), calculated by fitting the ligand trajectories to the TNKS1 protein structure, across the three independent replicates is reported in Figure S2.

The comparative analysis of the triplicate simulations revealed clear differences in the stability profiles of the investigated complexes. While **compound 3** and the co-crystallized ligand consistently maintained a highly stable baseline below 1.5 Å across all three replicates, the remaining candidates (**compounds 1, 2, 4,** and **5**) exhibited marked structural instability. Specifically, **compound 1** underwent abrupt conformational transitions away from its initial docked pose, adopting a distinct binding conformation with an RMSD plateau of approximately 10 Å in replicates A and C. **Compound 4** exhibited pronounced structural instability, ultimately dissociating from the binding pocket in replicate C, where its RMSD exceeded 25 Å during the final 100 ns of the simulation. **Compounds 2** and **5** also failed to establish a stable binding plateau, fluctuating continuously between 2 Å and 6 Å throughout the runs. Given the clear instability of the other candidates, further conformational and contact characterizations were focused exclusively on **compound 3**.

**Figure 5:**
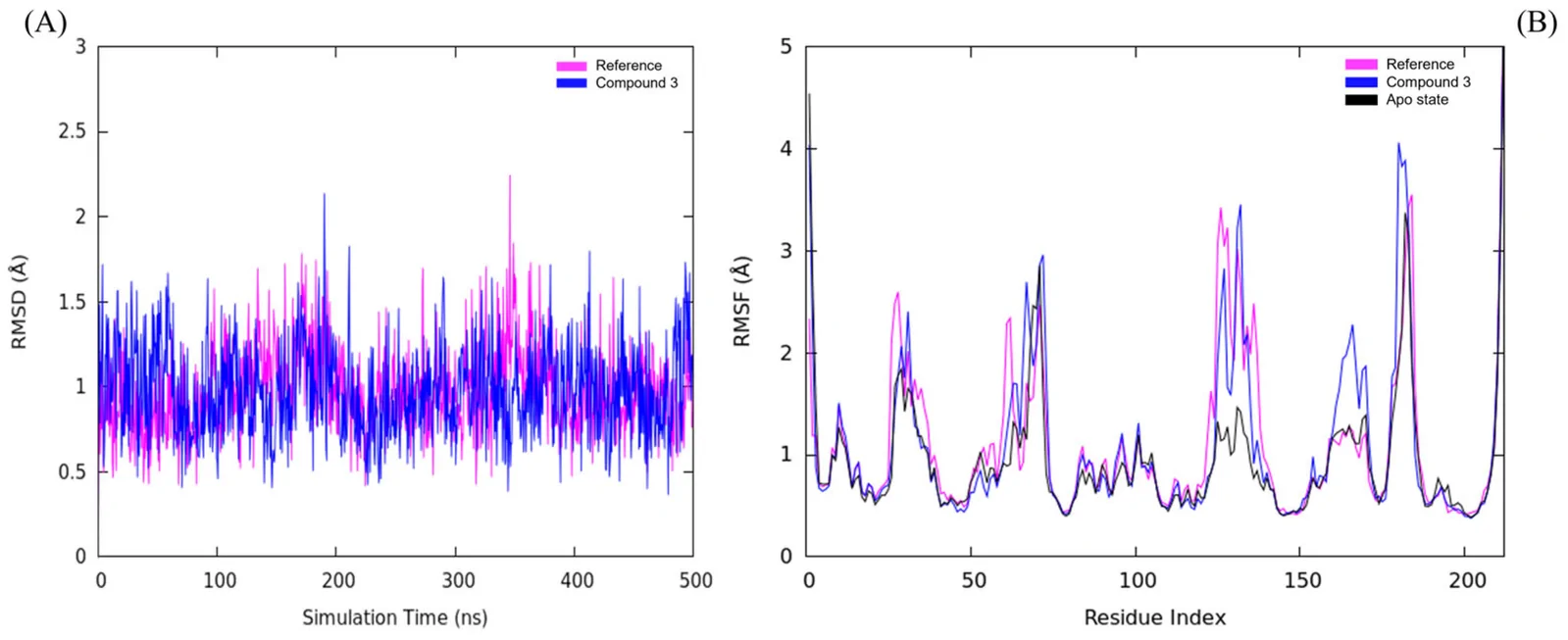
(**A**) Ligand RMSD plot of **compound 3** and the co-crystallized TNKS1 reference ligand during 500 ns MD simulations. RMSD values were calculated after fitting the ligand trajectories to the TNKS1 protein structure. **Compound 3** is represented by the blue line, whereas the co-crystallized reference ligand is shown in magenta. The profiles are representative of three independent simulation replicas (Figure S3). (**B**) RMSF profiles of the TNKS1 C-α atoms during the 500 ns MD simulations. The profiles of the unliganded protein (apo state, black), the co-crystallized reference inhibitor complex (magenta), and the compound 3 complex (blue) are shown to compare residue-level protein flexibility in the absence and presence of ligand binding. The similarity of the ligand-bound profiles provides a comparative assessment of the dynamic behavior of compound 3 relative to the reference complex. Data shown in both panels are representative of three independent simulation replicas (Figure S4).

As shown in Figure 5A, the RMSD trajectory of **compound 3** closely mirrored that of the co-crystallized reference ligand throughout the 500 ns simulation. Both ligands achieved rapid equilibration within the initial nanoseconds and maintained comparable, consistently low RMSD values. The average ligand RMSD values remained below 1.5 Å, with fluctuations confined to a narrow range of approximately 0.5–1.3 Å, confirming the structural integrity of the predicted binding pose under dynamic conditions. 

To further characterize the effect of ligand binding on local protein flexibility and compare it with the intrinsic dynamics of the unbound protein, the root mean square fluctuation (RMSF) of TNKS1 Cα atoms was analyzed across independent MD simulations of the apo protein, the co-crystallized reference complex, and the **compound 3** complex (Figure 5B). Overall, all three systems exhibited highly consistent RMSF profiles, indicating that ligand binding does not substantially perturb the intrinsic conformational dynamics of the protein domain. The secondary-structure elements and catalytic pocket remained particularly rigid throughout the simulations, with residues involved in ligand recognition (80–125) showing consistently low RMSF values (<1.2–1.5 Å) in both ligand-bound systems and the apo protein. These findings suggest that the TNKS1 binding cavity is intrinsically stable and structurally preorganized for ligand accommodation.

The largest fluctuations were confined primarily to solvent-exposed loop regions and terminal segments. In particular, increased mobility was observed in the loops spanning residues 25–35, 60–72, 120–140, and 175–185, with peak RMSF values ranging from 2.4 to 3.6 Å. Minor replica-dependent differences were observed in selected regions, including transient fluctuations within residues 120–140 and 155–170; however, these variations are consistent with baseline thermal motions rather than ligand-induced destabilization. The pronounced mobility observed at the C-terminus (>5.0 Å) is likewise consistent with the expected flexibility of an unconstrained protein terminus.

Collectively, the RMSF analysis indicates that **compound 3** preserves the dynamic characteristics of TNKS1 observed in both the apo and XAV939-bound states, without introducing additional flexibility within the catalytic region. The close similarity between the RMSF profiles of the XAV939- and **compound 3**-bound complexes further supports a binding mode that is dynamically compatible with that of the known active inhibitor and maintains the conformational stability of residues critical for ligand recognition throughout the simulations.

**Figure 6:**
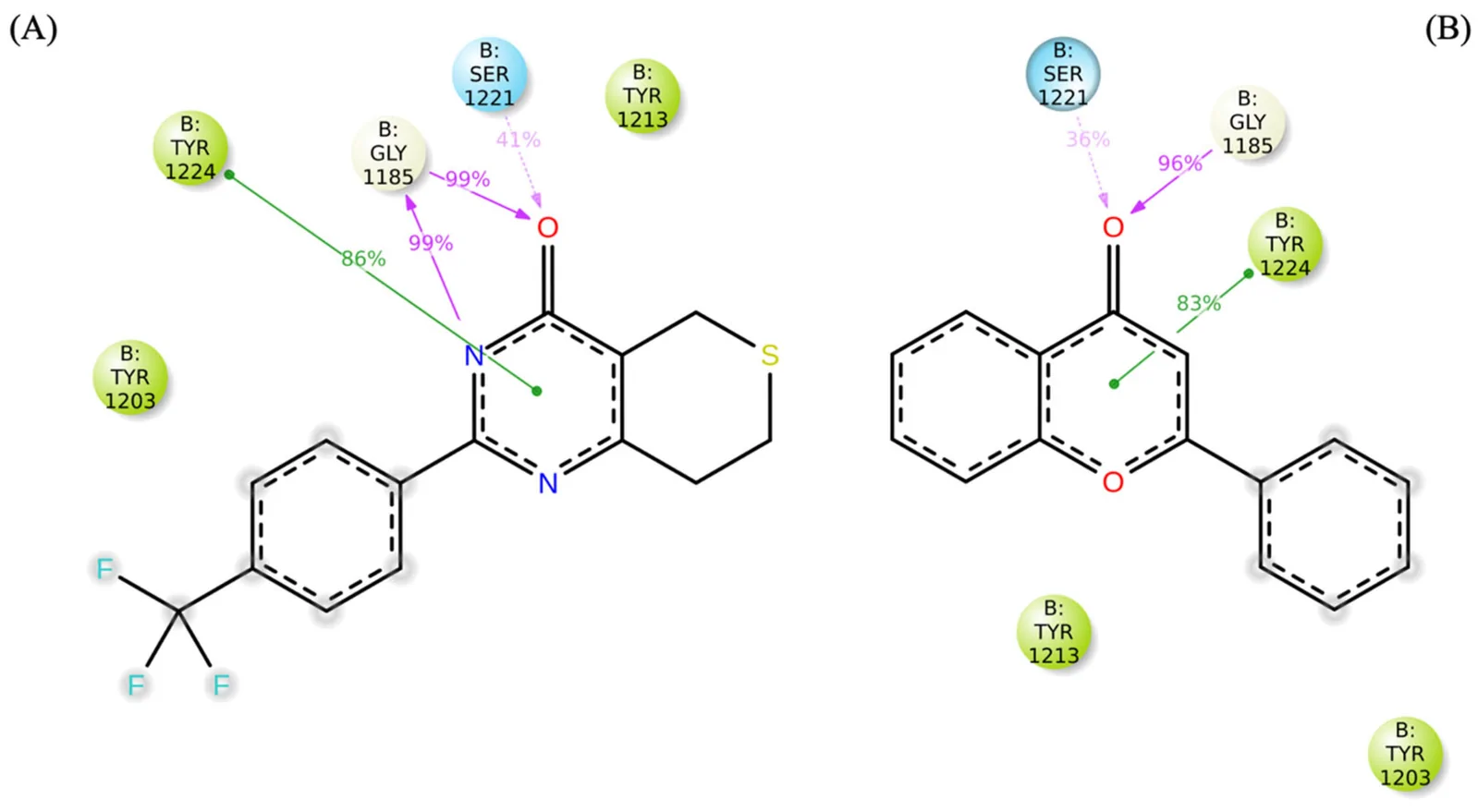
Two-dimensional ligand–protein interaction diagrams derived from MDs trajectories of the TNKS1 complexes with (**A**) the co-crystallized reference ligand and (**B**) **compound 3**. The diagrams summarize the predominant interactions maintained during the simulations, including hydrogen bonds (purple arrows) and π–π stacking interactions (green lines), together with the corresponding percentage occupancies. Hydrophobic residues are highlighted in green, polar residues in blue, and glycine residues in ivory.

To further characterize the dynamic behavior observed during the MD simulations, protein–ligand interaction patterns were analyzed throughout the trajectories. This analysis revealed that both the co-crystallized reference ligand and **compound 3** preserved the key recognition features within the TNKS1 catalytic pocket. The trajectory-averaged two-dimensional interaction maps generated from the 500 ns MD simulations (Figure 6) provided a comparative overview of the predominant contacts maintained by the two ligands, including H-bonds, hydrophobic interactions, and other non-covalent interactions associated with binding mode persistence. In the reference complex, this interaction was highly persistent, forming H-bonds with the backbone amide of Gly1185 (99% occupancy) and the side chain of Ser1221 (41% occupancy). **Compound 3** maintained a remarkably similar profile. During the MD simulation, dynamic relaxation and induced-fit adjustments allowed its carbonyl oxygen to form a stable hydrogen bond with the backbone amide of Gly1185 (96% occupancy), alongside key H-bond contacts with Ser1221 (36% occupancy).

In addition, the reference ligand established an extra H-bond through its ring nitrogen atom with Gly1185 (99% occupancy). A further conserved recognition feature shared by both complexes was the aromatic π–π stacking interaction with Tyr1224, which remained highly preserved throughout the simulations, with occupancies of 86% for the reference ligand and 83% for **compound 3**. The hydrophobic environment of the binding cavity was also maintained in both systems, with Tyr1203 and Tyr1213 providing persistent van der Waals and hydrophobic contacts around the ligand aromatic scaffolds. Overall, the comparable interaction occupancies observed for the two complexes provide a molecular basis for the similar ligand RMSD profiles and indicate that **compound 3** closely reproduces the key binding determinants of the crystallographic inhibitor.

### 2.6. In Vitro TNKS1 Enzymatic Inhibition

**Figure 7:**
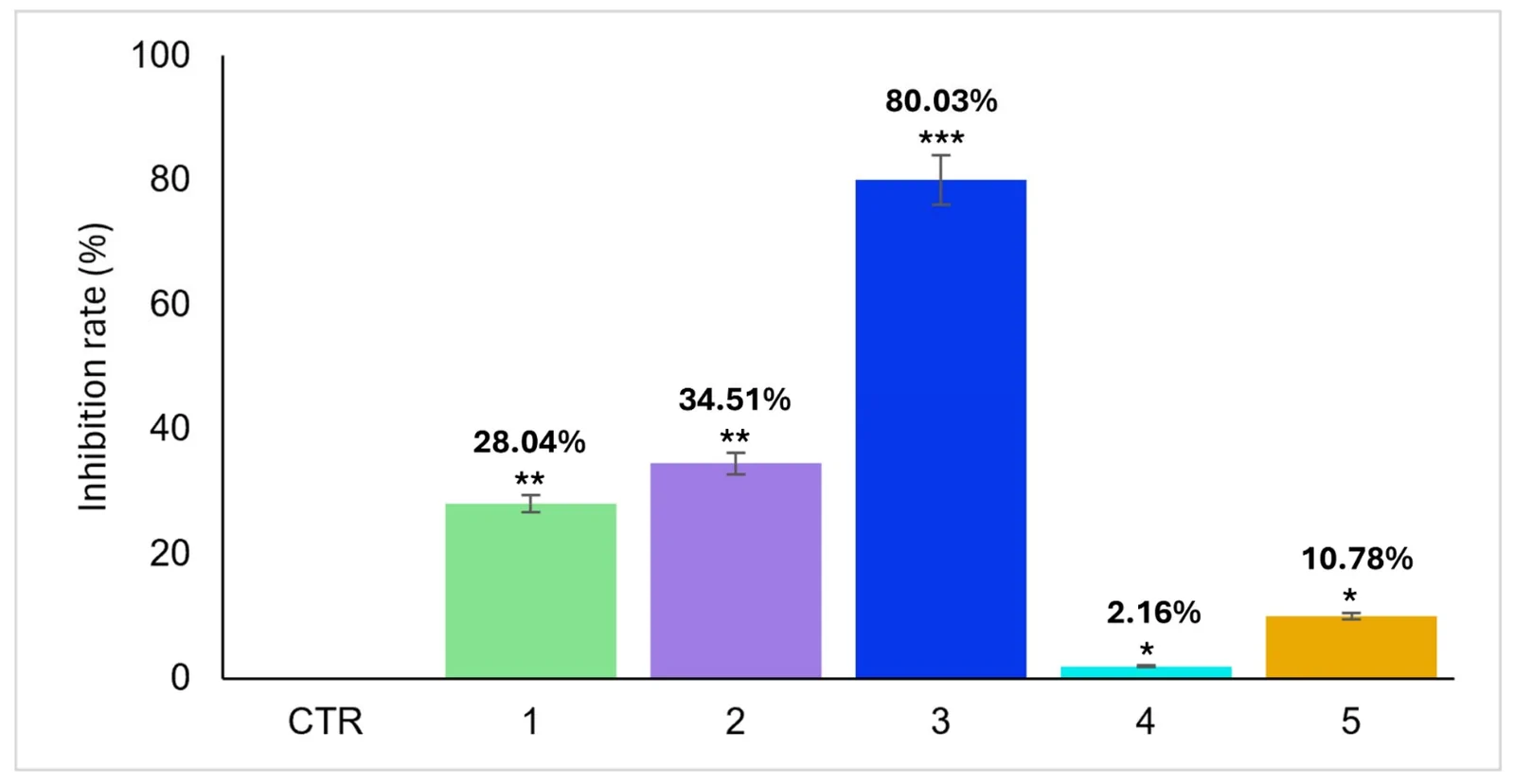
In vitro TNKS1 percentage inhibition rates of the five selected candidate compounds tested at a concentration of 0.1 µM. Data are presented as mean ± standard deviation (*n* = 2). * *p* < 0.05 ** *p* < 0.01, *** *p* < 0.001 vs CTR. **Compounds 1**, **2**, **3**, **4** and **5** are represented by green, violet, blue, cyan, and orange bars respectively.

The five selected candidate compounds were evaluated in an *in vitro* chemiluminescent assay to determine their direct inhibitory activity against purified TNKS1 at a screening concentration of 0.1 µM (Figure 7). The corresponding raw luminescence signals (RLU) for the blank, positive control (CTR+), and tested compounds across the two experimental replicates, together with their mean values, are reported in Table S7. The compounds displayed a wide range of inhibitory activities, highlighting distinct structural efficacy profiles among the prioritized candidates. Specifically, **compound 3** showed the highest inhibitory activity within the series, achieving an inhibition rate of 80.03% at 0.1 µM. This result is consistent with the stability and the persistent H-bonding network observed during the MD simulations, supporting the predicted compatibility of compound 3 with the TNKS1 catalytic pocket. More moderate inhibitory activities were observed for **compound 1** and **compound 2**, which reduced TNKS1 activity by 28.04% and 34.51%, respectively. Conversely, the remaining candidates showed substantially reduced efficacy; **compound 5** displayed weak inhibitory activity, achieving only 10% suppression, while **compound 4** exerted a negligible effect on the enzyme with a 2% inhibition rate. Taken together, these biological findings provide experimental support for the computational prioritization and identify **compound 3** as a promising TNKS1 inhibitor candidate for further structural optimization.

## 3. Discussion

In the present study, we developed and applied an integrated computational workflow combining supervised machine learning, structure-based virtual screening (SBVS), molecular docking, Prime MM-GBSA energetic refinement, and molecular dynamics (MD) simulations to identify novel TNKS1 inhibitor candidates. TNKS1 represents an emerging therapeutic target in oncology due to its key role in regulating Wnt/β-catenin signaling, a pathway frequently dysregulated in cancer development and progression [30,31]. Despite the growing interest in tankyrase inhibition, the discovery of novel chemical entities capable of modulating TNKS1 activity remains an important challenge [6].

SBVS represents a well-established approach in modern drug discovery and has been successfully applied in our laboratory for the identification of bioactive molecules against several therapeutically relevant targets [32,33,34,35,36,37]. In this study, SBVS was integrated with consensus machine learning to support the exploration of chemical space and early-stage hit prioritization [18].

Although several TNKS1 inhibitors have been described, including the widely used reference inhibitor XAV939 [38,39,40] and other reported chemical classes such as IWR-1 [41,42] and JW55 [43], many available compounds remain primarily research tools, and the identification of new chemotypes with improved potency, isoform selectivity, and drug-like properties is still required. The proposed ligand-based and structure-based workflow enabled the systematic exploration of a screening space comprising more than 700,000 compounds, progressively reducing the candidate pool to a focused set of high-confidence molecules suitable for experimental evaluation.

The consensus ML framework successfully prioritized 670 AI-selected compounds for subsequent structure-based investigation. Docking-based screening within the experimentally validated TNKS1 catalytic pocket identified candidates capable of reproducing key recognition features observed for known tankyrase inhibitors while maintaining chemically plausible binding orientations. Integration of docking analysis, binding mode inspection, and structural compatibility reduced the candidate pool to five compounds for further energetic and dynamic evaluation. Prime MM-GBSA calculations provided an additional refinement step, revealing differences in predicted binding free energies that were not fully captured by docking scores alone. Among the selected candidates, **compound 3** emerged as the most favorable hit, showing the most balanced energetic profile despite displaying docking performance comparable to the other prioritized molecules.

MD simulations further supported the prioritization of **compound 3** by demonstrating preservation of the predicted binding mode over 500 ns trajectories, together with maintenance of key interactions within the TNKS1 catalytic pocket and reduced ligand conformational fluctuations. Importantly, biochemical evaluation provided experimental support for the computational prioritization, identifying **compound 3** as the most promising candidate with measurable TNKS1 inhibitory activity.

Interestingly, **compound 3** corresponds to CHEMBL275638, a compound for which previous experimental binding data toward the nucleotide-binding domain 2 (NBD2) of the ABCB1/P-glycoprotein transporter have been reported (K_d_ = 33.9 μM) [44]. Although this previously described interaction involves a different biological target and does not provide evidence of TNKS1 modulation, our findings identify this chemical scaffold as a TNKS1 inhibitor candidate. To further contextualize its chemical novelty, the structural similarity of **compound 3** to previously reported TNKS1 inhibitors was evaluated using Morgan fingerprint-based Tanimoto similarity; the corresponding similarity values and 2D molecular structures are reported in Table S8. Therefore, the relevance of **compound 3** in the present study derives from its newly identified TNKS1 inhibitory activity and from its potential as a starting scaffold for further tankyrase-focused medicinal chemistry optimization.

From a medicinal chemistry perspective, **compound 3** represents a valuable starting point for further optimization rather than a final therapeutic entity. Its chemically distinct framework and experimentally supported TNKS1 inhibitory activity provide a foundation for structure–activity relationship (SAR) exploration aimed at improving potency, physicochemical properties, pharmacological performance, **and isoform selectivity**. Because the present study focused on the identification of TNKS1 inhibitor candidates, the activity of **compound 3** against **TNKS2** and other members of the **PARP family** remains to be established. Comprehensive selectivity profiling will therefore represent an important component of future lead optimization studies. Subsequent optimization campaigns may exploit the identified binding interactions within the TNKS1 catalytic pocket to generate derivatives with improved potency and isoform selectivity.

Overall, these findings highlight the potential of integrating consensus machine learning, SBVS, molecular docking, Prime MM-GBSA refinement, molecular dynamics simulations, and biochemical validation for the prioritization of TNKS1 inhibitor candidates from large chemical libraries. Although the present study was not designed to formally benchmark each computational component independently or to evaluate model generalizability using scaffold-aware validation schemes, and the negative class consisted of property-matched DUD-E decoys rather than experimentally confirmed inactive compounds, the integrated workflow enabled the identification of a promising chemical scaffold supported by experimental evidence. Future studies incorporating alternative screening strategies, appropriate control datasets, and scaffold-based validation protocols will further define the contribution of individual computational steps and the broader applicability of the proposed approach. Importantly, the experimentally observed TNKS1 inhibitory activity of compound 3 underscores the value of combining data-driven prioritization with structure-based methods and biochemical validation to support reliable early-stage hit discovery.

## 4. Materials and Methods

### 4.1. Dataset Construction and Curation

Experimentally validated TNKS1 inhibitors were retrieved from the ChEMBL database [45] based on reported IC_50_ measurements against TNKS1. The initial dataset comprised 1011 IC_50_ bioactivity records. Entries lacking valid IC_50_ values or canonical chemical structures, as well as records flagged with data validity comments, were excluded during data curation. Molecular structures were standardized and converted into canonical SMILES representations using RDKit, and compounds exhibiting IC_50_ values ≤10 μM were retained as active molecules. When multiple IC_50_ measurements were available for the same compound, duplicate bioactivity records were merged using the median IC_50_ value, resulting in a final curated dataset of 727 unique experimentally validated TNKS1 inhibitors.

To construct the supervised classification framework, the active compounds were complemented with property-matched decoys obtained from the DUD-E platform (Database of Useful Decoys: Enhanced) [46]. Structural integrity was verified through automated quality-control procedures, and duplicate compounds were removed before descriptor generation. The initial decoy collection comprised 55,526 molecules. To mitigate class imbalance during supervised learning, random undersampling was applied to the decoy class, resulting in a final dataset composed of 727 active compounds and 3635 property-matched decoys (1:5 ratio). The complete machine learning dataset, including the curated TNKS1 active compounds and the selected DUD-E decoys, is provided in Table S9. This curated dataset was subsequently partitioned using a stratified splitting strategy into training (3053 compounds), validation (654 compounds), and independent test (655 compounds) subsets while preserving class proportions across all partitions. The compound-level assignment to the training, validation, and independent test sets is provided in Table S10. This strategy was selected to preserve the class distribution across all subsets. No Bemis–Murcko scaffold split was applied in the present study. The resulting dataset served as the basis for molecular descriptor generation, machine learning model development, and subsequent external virtual screening campaigns. The complete list of curated TNKS1 active compounds, together with their corresponding ChEMBL identifiers and curated IC_50_ values, is provided in Table S11.

### 4.2. Molecular Descriptor Generation

Molecular representations were derived from curated SMILES structures and encoded into circular fingerprints for supervised machine learning. During data curation, structures were standardized (including salt removal, normalization, and duplicate filtering) prior to descriptor generation. Compounds were then transformed into numerical feature vectors using Morgan circular fingerprints, selected for their ability to capture local atomic environments, molecular connectivity, and relevant substructural patterns associated with biological activity [47]. Fingerprints were computed with a radius of 2 (ECFP4) and a bit vector length of 2048 using the RDKit cheminformatics toolkit implemented in Python [48]. These parameters were chosen to balance structural resolution and computational efficiency while minimizing bit collision effects. The resulting binary fingerprint vectors were directly used as input features for all machine learning models without additional scaling. A consistent descriptor framework was applied across training, validation, test, and external screening datasets to ensure uniform molecular representation and comparability across chemically diverse compounds.

### 4.3. Machine Learning Model Development

Supervised machine learning models were developed to discriminate experimentally validated TNKS1 inhibitors from DUD-E decoy compounds using the molecular fingerprint representations described above. Because the negative class consisted of computationally generated decoys rather than experimentally confirmed inactive TNKS1 compounds, the resulting classifiers were designed as prioritization tools for ligand-based virtual screening rather than as direct predictors of TNKS1 inhibitory activity.

Three complementary classification algorithms were independently trained and evaluated, namely Extreme Gradient Boosting (XGBoost) [26], Logistic Regression [49], and Support Vector Machine (SVM-RBF) [50]. Logistic Regression and SVM-RBF were implemented using scikit-learn (version 1.5.1), whereas XGBoost was implemented using the XGBoost library (version 2.1.3). The complete hyperparameter configuration adopted for each classifier is reported in Table S1. Logistic Regression was configured using the LBFGS solver with L2 regularization, C = 1.0, balanced class weights, and a maximum of 5000 iterations. The SVM-RBF classifier employed an RBF kernel with C = 1.0, gamma = scale, balanced class weights, and probability estimation enabled. XGBoost was configured using the binary logistic objective function, 300 boosting estimators, a learning rate of 0.05, a maximum tree depth of 5, subsample = 0.8, colsample_bytree = 0.8, and log-loss as the evaluation metric. Parameters not explicitly specified were retained at the default values of the corresponding software implementations. The curated dataset was partitioned using a stratified strategy into training, validation, and independent test sets while preserving class proportions. A fixed random seed of 42 was used throughout dataset partitioning and model development. Random undersampling without replacement was applied to the DUD-E decoy class to obtain the final active-to-decoy ratio of 1:5. Balanced class weights were applied to Logistic Regression and SVM-RBF, whereas the default class-weighting configuration was retained for XGBoost. Model performance was evaluated on the independent test set using accuracy, precision, recall, F1-score, and ROC-AUC. A probability threshold of 0.50 was used to assign binary class labels during model evaluation. No additional post hoc probability-calibration procedure, such as Platt scaling or isotonic regression, was applied; ranking and filtering procedures were based on the probability estimates generated directly by the trained classifiers. For external virtual screening, the three classifiers were applied independently to each compound. A consensus score was calculated as the arithmetic mean of the three prediction probabilities and used for compound ranking. Only compounds assigned an active-class probability greater than 0.90 by all three individual classifiers were retained for subsequent structure-based analyses. The consensus procedure was applied exclusively during external virtual screening as a decision-level ranking and filtering strategy. It was not trained or benchmarked as a fourth supervised classifier; consequently, the independent test-set performance reported in Section 2.1 refers to the three individual models. 

The complete computational environment and implementation settings, including software versions, molecular representation, dataset balancing, data partitioning, decision thresholds, and probability handling, are summarized in Table S2. The workflow was implemented in Python 3.9.6 using RDKit 2022.03.4, scikit-learn 1.5.1, XGBoost 2.1.3, NumPy 1.26.4, and pandas 2.2.2.

### 4.4. AI-Driven Virtual Screening Workflow

Following model development, the consensus machine learning pipeline was applied to the large-scale screening of external chemical libraries, including both natural products and commercially available compounds. All molecular structures were processed using the same fingerprint-based descriptor pipeline employed during model training to ensure consistency between the screened compounds and the training set. For each compound, each classifier generated an independent probability estimate of belonging to the active class. A consensus score was calculated as the arithmetic mean of the prediction probabilities generated by the three independently trained classifiers. For external virtual screening, only compounds simultaneously predicted as active by all three models, each assigning a prediction probability greater than 0.90, were retained for subsequent analyses. The arithmetic-mean consensus score and the >0.90 three-model agreement rule served distinct purposes: the former was used for ranking, whereas the latter was used as the retention criterion. To characterize the relationship between the AI-prioritized compounds and the chemical space represented by the training set, the maximum Morgan fingerprint-based Tanimoto similarity relative to the training actives was calculated [51]. In addition, fingerprint-based Tanimoto similarity relative to previously reported TNKS1 inhibitors was determined to contextualize the retained compounds with respect to known active scaffolds and was not used as an exclusion criterion. The resulting AI-prioritized compound collection (670 compounds) was subsequently subjected to Butina clustering analysis, molecular docking, Prime MM-GBSA energetic refinement, and molecular dynamics simulations. 

### 4.5. Butina Clustering Analysis of the AI-Prioritized Collection

A Butina clustering analysis was performed using the RDKit implementation of the algorithm [52,53]. Molecular similarity was calculated from Morgan fingerprints using the Tanimoto coefficient as the pairwise similarity metric. A Tanimoto distance threshold of 0.4 was applied to group structurally related compounds and to describe the structural organization of the AI-prioritized compound collection. The clustering analysis was performed exclusively to assess structural redundancy within the retained compounds and was not used as a criterion for compound ranking or selection. Final candidate prioritization relied on structure-based evaluation, including molecular docking, binding-mode inspection, Prime MM-GBSA energetic refinement, and molecular dynamics simulations.

### 4.6. Protein Preparation and Binding Pocket Characterization

The crystallographic structure of TNKS1 (PDB: 3UH4) [54] was retrieved from the Protein Data Bank [55,56] and prepared using the Protein Preparation Wizard implemented in Schrödinger Maestro [57]. This structure was selected because it provides a high-resolution (2.00 Å) X-ray structure of the human TNKS1 catalytic PARP domain in complex with the well-characterized inhibitor NVP-XAV939, thereby offering an experimentally validated binding site for subsequent structure-based studies. Hydrogen atoms were added, bond orders were assigned, and protonation states were optimized under physiological pH conditions. Water molecules not directly involved in structural stabilization were removed during preprocessing procedures. Protein minimization and local structural optimization were performed using the OPLS4 force field [58] while preserving the crystallographic geometry of the receptor. Redocking experiments involving the co-crystallized inhibitor XAV939 were performed to validate the docking protocol and to identify the most reliable receptor configuration for subsequent structure-based analyses. Both crystallographic chains A and B were independently evaluated for their ability to reproduce the experimental ligand binding pose. Among the tested configurations, chain B provided the most accurate reconstruction of the native ligand orientation and was therefore selected for all downstream calculations. For both chains A and B, receptor grids were generated by centering on the co-crystallized XAV939 ligand within the catalytic cavity, using an inner docking box of 10 × 10 × 10 Å and an outer box of 30 × 30 × 30 Å to ensure full coverage of the binding pocket and its surrounding residues.

### 4.7. Molecular Docking Simulations

Molecular docking calculations were performed using the Glide module implemented in Schrödinger Maestro [59]. Compounds retained after the AI-driven prioritization stage were docked into the experimentally validated TNKS1 catalytic pocket employing a flexible ligand docking protocol. Prior to docking calculations, ligand structures were prepared using the LigPrep workflow, including generation of relevant protonation states at physiological pH (7.4) and subsequent energy minimization to obtain low-energy conformations suitable for docking. Docking poses were ranked using Glide scoring (G-Score) functions, and the compounds exhibiting Glide G-Scores lower than the redocking score of the co-crystallized inhibitor XAV939 (−9.12 kcal/mol) were retained for subsequent visual inspection. The structural evaluation focused on the identification of plausible binding modes, appropriate occupation of the catalytic cavity, steric complementarity, and the formation of key intermolecular interactions within the TNKS1 active site. Particular emphasis was placed on hydrogen-bonding (H-bond) networks, hydrophobic contacts, π-π stacking interactions, and the overall orientation of the ligands relative to the binding mode of the co-crystallized inhibitor XAV939.

### 4.8. Prime MM-GBSA Energetic Refinement

To further refine the docking-derived hit selection, Prime MM-GBSA calculations were performed on the top-ranked docking pose of the identified candidates to estimate their relative binding free energies [60]. Energetic refinement was carried out using the Prime MM-GBSA workflow implemented in Schrödinger Maestro. Binding free energies (ΔG_bind_) were estimated using the minimized protein–ligand complexes generated from the docking stage. The analysis incorporated energetic contributions associated with intermolecular stabilization and solvation effects, providing a more realistic assessment of binding favorability than docking scores alone. MM-GBSA refinement was subsequently integrated with consensus AI prediction scores and docking results to support the final prioritization of the most promising TNKS1 inhibitor candidates prior to molecular dynamics (MD) simulations.

### 4.9. Molecular Dynamics (MD) Simulations

Molecular dynamics (MD) simulations were performed using the Desmond Molecular Dynamics package implemented in Schrödinger Maestro [61] to evaluate the dynamic stability of TNKS1 in both the unliganded (apo) state and in complex with the selected ligands. Each system was embedded in an explicit solvent environment using the TIP3P water model and neutralized by the addition of appropriate counterions. Periodic boundary conditions were applied throughout all simulations. Prior to production runs, each system underwent energy minimization and a multistep equilibration protocol to remove unfavorable steric contacts and ensure system stability. Production simulations were subsequently carried out in the NPT ensemble at 300 K and 1.01325 bar using the OPLS4 force field. A simulation length of 500 ns was employed for each complex to allow adequate sampling of protein–ligand interactions and conformational stability. Trajectory analyses were performed using the simulation analysis tools available in the Schrödinger suite. Structural stability was primarily assessed through root mean square deviation (RMSD) calculations relative to the initial minimized structures. In addition, protein–ligand interaction persistence, ligand accommodation within the catalytic cavity, hydrogen-bond occupancy, hydrophobic contacts, and aromatic interactions were monitored throughout the simulations to evaluate the stability of the predicted binding modes under dynamic conditions. The resulting MD trajectories were subsequently used as an additional structural and dynamic assessment following molecular docking and Prime MM-GBSA refinement, supporting the identification of the most dynamically stable TNKS1 inhibitor candidates.

### 4.10. In Vitro TNKS1 Inhibition Assay

The direct inhibitory activity of the candidate compounds against purified Tankyrase 1 (TNKS1) was evaluated using the TNKS1 Chemiluminescent Assay Kit (BPS Bioscience, San Diego, CA, USA; Cat. #78405-1) following the manufacturer’s instructions [62,63]. A 96-well module plate was coated with 50 µL/well of a 1x histone mixture (diluted in PBS) and incubated overnight at 4 °C. The wells were then washed three times with PBST buffer (1x PBS containing 0.05% Tween-20) and blocked with Blocking Buffer 3 for 90 min at room temperature. After a subsequent washing step, 25 µL of a ribosylation Master Mix containing 10x PARP assay buffer, PARP Substrate Mixture 2 (biotinylated NAD^+^), and freshly prepared dithiothreitol (DTT; 1 mM final concentration) was added to each well. The candidate compounds were dissolved in DMSO and tested at final concentrations of 0.1 µM and 1 µM, ensuring that the final DMSO concentration did not exceed 1% (*v*/*v*). Positive control and blank wells received the vehicle only. The enzymatic reaction was initiated by adding 20 µL of purified GST-tagged TNKS1 enzyme (diluted in 1x PARP buffer to a final concentration of 2 nM) to the test and positive control wells, while blank wells received 1x PARP assay buffer alone. Following a 1 h incubation at room temperature, the plate was washed three times with PBST. For detection, 50 µL of Streptavidin-HRP (diluted 1:50 in Blocking Buffer 3) was added to each well and incubated for 30 min at room temperature. After a final washing cycle, 100 µL of a 1:1 mixture of ELISA ECL Substrates A and B was added. Chemiluminescence was immediately measured using a microplate reader in luminescence mode. Luminescence values from blank wells were subtracted from all experimental data to calculate the percentage of residual enzyme activity. Assay performance and baseline stability were validated using internal positive controls (enzyme + vehicle) representing maximum (100%) enzymatic activity and blank wells for background subtraction.

### 4.11. Compounds Sourcing, Specifications, and Handling

All candidate molecules were purchased through MolPort (MolPort-001-791-079, MolPort-001-791-078, MolPort-001-016-955, MolPort-000-148-418, MolPort-002-678-878). For **compound 3** (MolPort-001-016-955), the upstream supplier catalogue number was AS-58464 and the lot/batch number was 118457. 

According to the information provided by the supplier through MolPort, **compound 3** had a purity of 99.7%, as determined by gas chromatography (GC). The identity of the commercial supplier/manufacturer could not be disclosed because of a confidentiality agreement.

Upon receipt, all tested compounds were stored as dry powders at −20 °C protected from light. Primary stock solutions were prepared in 100% high-purity dimethyl sulfoxide (DMSO) at a concentration of 10 mM, thoroughly vortexed to ensure complete dissolution, aliquoted, and maintained at −20 °C until use. Immediately prior to performing the enzymatic inhibition assays, working solutions were freshly prepared by serial dilution of the stock solutions into the assay buffer. The final DMSO concentration in all experimental reaction mixtures was strictly maintained at 1% (*v*/*v*) to avoid solvent interference with enzymatic activity.

### 4.12. Statistical Analysis

Data from the *in vitro* inhibition assay were analyzed by one-way ANOVA followed by the Tukey–Kramer multiple comparisons test. All results are expressed as mean ± standard deviation (SD) and were considered statistically significant at p-values lower than 0.05. All statistical analyses were performed using GraphPad Prism version 9.3.0 statistical software (GraphPad Software Inc., San Diego, CA, USA).

## 5. Conclusions

In conclusion, this study demonstrates the utility of an integrated artificial intelligence- and structure-based drug discovery pipeline for the identification of novel TNKS1 inhibitor candidates. By combining supervised consensus ML, SBVS, molecular docking, Prime MM-GBSA refinement, MD simulations, and biochemical validation, we prioritized a focused set of compounds from a screening space of more than 700,000 molecules, leading to the identification of compound 3 as a promising starting point for further optimization.

Although additional benchmarking studies will be required to further define the contribution of individual computational steps and to evaluate the broader applicability of the approach, the present findings highlight the value of combining data-driven and physics-based methods with experimental validation to support early-stage hit discovery.

Among the identified candidates, **compound 3** consistently emerged as the most promising hit across all computational and experimental evaluations. Its favorable binding energetics, stable interaction pattern within the TNKS1 catalytic pocket, and measurable inhibitory activity support its potential as a promising starting point for further medicinal chemistry optimization. Although additional studies will be required to improve potency and pharmacokinetic properties, and to establish its selectivity profile toward TNKS2 and other PARP family members, the identified scaffold provides a valuable basis for future structure–activity relationship (SAR) investigations.

Beyond the identification of compound 3 as a TNKS1 inhibitor, this work illustrates the potential complementarity of data-driven and physics-based computational approaches with experimental validation in early-stage hit discovery. Further prospective benchmarking across additional datasets and therapeutic targets will be required to evaluate the broader applicability of the proposed strategy.

## Figures and Tables

**Table 1:** Commercial code, 2D structure, and G-Score of the selected compounds.

Compound	Commercial Code	2D Structure	G-Score (kcal/mol)
**XAV939**		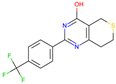	−9.12
**Compound 1**	MolPort-001-791-079	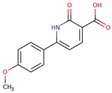	−10.55
**Compound 2**	MolPort-001-791-078	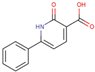	−10.23
**Compound 3**	MolPort-001-016-955	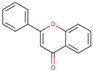	−10.02
**Compound 4**	MolPort-000-148-418	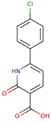	−10.41
**Compound 5**	MolPort-002-678-878	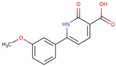	−10.57

**Table 2:** **Docking scores and Prime MM-GBSA energy decomposition of the five final TNKS1 candidates.** The reported MM-GBSA contributions include the overall binding free energy (ΔG_bind_) together with its principal energetic components (Coulomb, van der Waals, lipophilic, packing, and Generalized Born solvation terms), providing insights into the key molecular determinants governing protein–ligand energetics.

Compound	G-Score	ΔG_bind_	Coulomb	vdW	Lipophilic	Packing	Solv GB
**XAV939**	−9.12	−50.97	−4.85	−42.24	−17.36	−3.46	16.68
**Compound 1**	−10.55	−51.11	−70.42	−42.96	−18.91	−3.73	84.69
**Compound 2**	−10.23	−48.01	−68.19	−39.45	−17.61	−3.70	82.19
**Compound 3**	−10.02	−63.11	−16.61	−38.87	−15.12	−6.23	13.88
**Compound 4**	−10.41	−54.09	−69.05	−41.08	−19.13	−3.70	80.36
**Compound 5**	−10.57	−46.57	−68.17	−46.21	−18.72	−3.85	85.09

## Data Availability

The original contributions presented in this study are included in the article and Supplementary Materials. Further inquiries can be directed to the corresponding author.

## Supplementary Materials

The following supporting information can be downloaded at: https://www.mdpi.com/article/10.3390/ph19081310/s1, Table S1. Predictive performance of the three supervised machine learning models evaluated on the independent test set; Table S2. Curated TNKS1 inhibitor dataset used for machine learning model development; Table S3. Hyperparameter configuration of the supervised machine learning models used for TNKS1 inhibitor classification; Table S4. Computational environment and implementation details of the machine learning workflow; Table S5. Machine-learning predictions for the 670 compounds retained after the AI-based screening workflow; Table S6. Machine learning prediction probabilities and consensus scores of the five final TNKS1 candidate compounds selected for experimental validation; Table S7. Complete curated dataset used for machine-learning model development, comprising 727 experimentally validated TNKS1 inhibitors (Label = 1) and 3635 DUD-E property-matched decoys (Label = 0); Table S8. Complete machine learning dataset used for model development and evaluation; Table S9. Raw molecular docking data for the 52 compounds retained following structure-based virtual screening against the TNKS1 catalytic binding site; Table S10. Structural similarity analysis of Compound 3 against representative tankyrase inhibitors; Table S11. Luminescence signal (RLU) of the tested compounds; Figure S1. Redocking validation of the co-crystallized inhibitor XAV939 within the TNKS1 catalytic pocket; Figure S2. Comparative ligand RMSD analysis of selected TNKS1 candidates during independent molecular dynamics (MD) simulations; Figure S3. Reproducibility analysis of ligand RMSD trends across independent molecular dynamics simulations; Figure S4. RMSF analysis of TNKS1 Cα atoms across independent replicate MD simulations; Figure S5. Confusion matrices of the machine-learning classifiers on the independent test set. Confusion matrices obtained for (A) XGBoost, (B) SVM–RBF, and (C) Logistic Regression; Figure S6. Precision–recall curves of the machine-learning classifiers on the independent test set.

## Abbreviations

The following abbreviations are used in this manuscript:

TNKS1	Tankyrase 1
AI	Artificial Intelligence
ML	Machine learning
VS	Virtual Screening
XGBoost	Extreme Gradient Boosting
SVM-RBF	Support Vector Machines with a radial basis function kernel
MD	Molecular dynamics
ROC	Receiver operating characteristic
AUC	Area under the curve
G-Score	Glide Score
H-bond	Hydrogen bond
MM-GBSA	Molecular Mechanics/Generalized Born Surface Area
RMSD	Root mean square deviation
RMSF	Root mean square fluctuation
