# Supplementary material for: An Integrated Consensus Machine Learning and Structure-Based Workflow for the Discovery of Novel Tankyrase 1 Inhibitors
Source: Pharmaceuticals (Basel). 2026 Aug 19;19(8):1310. doi: 10.3390/ph19081310 (PMC13516162; doi:10.3390/ph19081310)
Supplement: Supplementary file 1 [file pharmaceuticals-19-01310-s001.zip › pharmaceuticals-4483169-supplementary.pdf]

Supplementary Informations

An Integrated Consensus Machine Learning and Structure-Based Workflow for the Discovery of Novel Tankyrase 1 Inhibitors

Model	*Accuracy*	*Precision*	*Recall*	*F1*	*ROC-AUC*
Logistic Regression	0.913	0.755	0.706	0.730	0.872
SVM-RBF	0.924	0.786	0.743	0.764	0.894
XGBoost	0.934	0.824	0.771	0.796	0.915

Table S1. Predictive performance of the three supervised machine learning models evaluated on the independent test set. Model performance is reported in terms of Accuracy, Precision, Recall, F1-score, and area under the receiver operating characteristic curve (ROC-AUC).

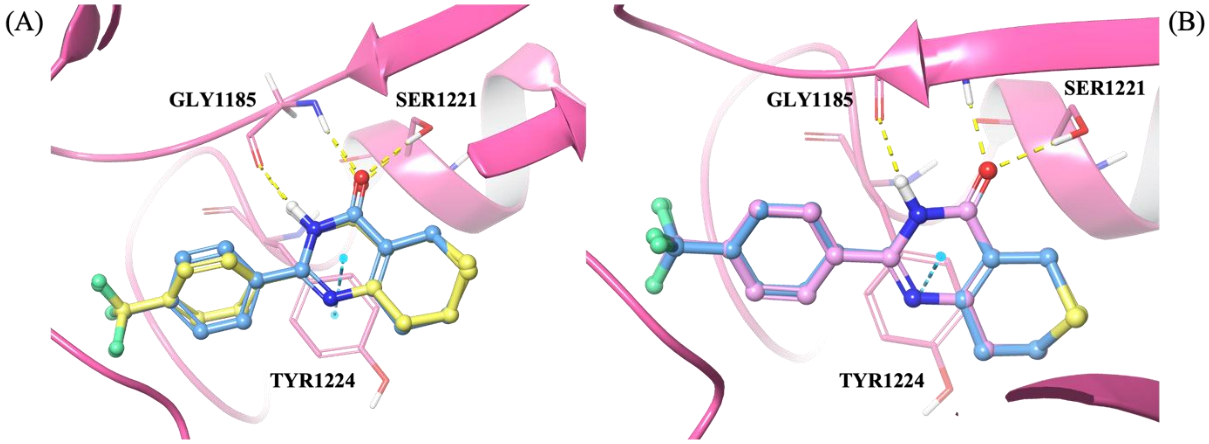

Figure S1. Redocking validation of the co-crystallized inhibitor XAV939 within the TNKS1 catalytic pocket. (A) Redocking validation of XAV939 within the TNKS1 catalytic domain (chain A). The crystallographic binding pose (azure) and the reproduced docking pose (yellow) are shown for structural comparison. **(B)** Redocking validation in chain B, displaying the crystallographic pose (azure) and the corresponding docking pose (mauve). Hydrogen bonding interactions are depicted as yellow dashed lines, while key catalytic residues are represented in stick format.

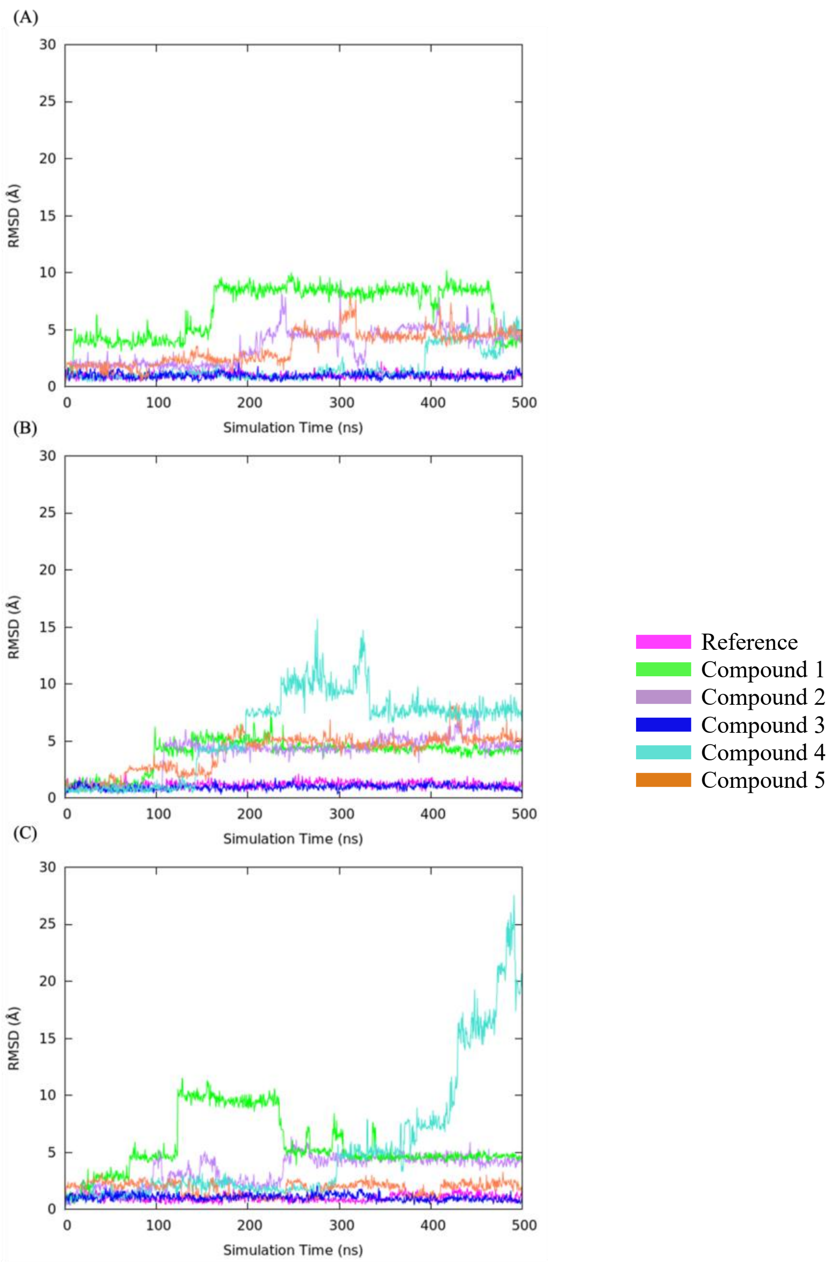

Figure S2. Comparative ligand RMSD analysis of selected TNKS1 candidates during independent molecular dynamics (MD) simulations. (A–C) Root-Mean-Square Deviation (RMSD) trends of the five prioritized compounds and the co-crystallized TNKS1 inhibitor obtained from three independent 500 ns MD simulation replicas. Ligand RMSD values were calculated after least-squares fitting of each trajectory to the TNKS1 protein backbone, providing a measure of ligand conformational stability and binding mode persistence within the catalytic pocket.

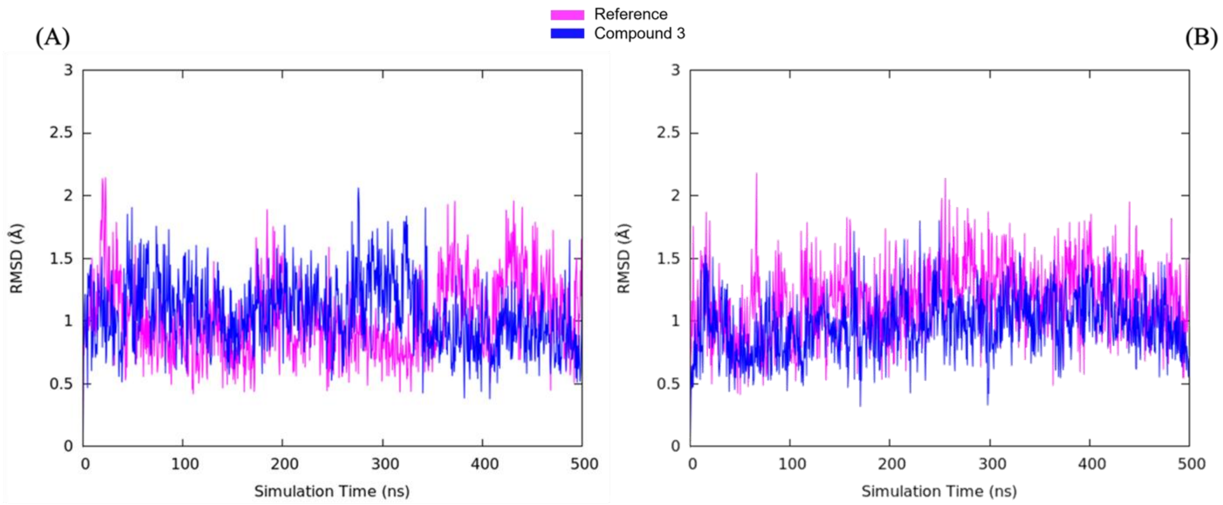

Figure S3. Reproducibility analysis of ligand RMSD trends across independent molecular dynamics simulations. RMSD trajectories of **compound 3** (blue lines) and the co-crystallized TNKS1 inhibitor (magenta lines) obtained from two additional independent 500 ns MD replicas Panels **(A)** and **(B)** display the ligand RMSD trajectories obtained from Replica 2 and Replica 3, respectively. RMSD values were calculated after least-squares fitting of the trajectories onto the TNKS1 protein backbone. The consistent RMSD trends observed across replicates confirm the reproducibility of the ligand stability and conformational behavior reported for the representative simulation shown in Figure 5.

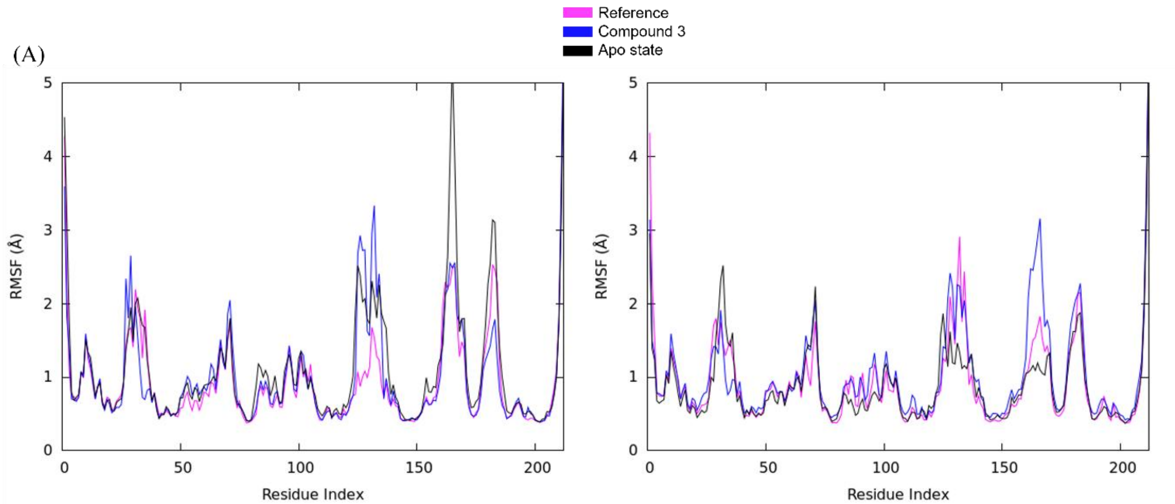

Figure S4. RMSF analysis of TNKS1 Cα atoms across independent replicate MD simulations. Panels **(A)** and **(B)** show the RMSF profiles obtained from the second and third independent simulation replicas, respectively (Replica 2 and Replica 3). In both panels, the unliganded protein (apo state) is represented by the black line, the TNKS1–co-crystallized reference ligand complex by the magenta line, and the TNKS1–compound **3** complex by the blue line. The profiles demonstrate consistent protein flexibility patterns and structural stability across the independent MD runs.

Compound ID	ChEMBL ID	Canonical SMILES	Median IC_{50} (*nM*)	IC_{50} records
TNKS1_active_001	CHEMBL4069447	Cc1cccc2c(=O)[nH]c(CCC(=O)Nc3ccc(C(=O)Nc4cccc5cccnc45)cc3)nc12	0.1	4
TNKS1_active_002	CHEMBL3805393	COc1ccc(C(=O)C2CCN(C(=O)CCCc3cn4cc(F)cc4c(=O)[nH]3)CC2)cn1	0.16	1
TNKS1_active_003	CHEMBL3806163	COc1ccc(C(=O)C2CCN(C(=O)CCCc3cn4cc(F)cc4c(=O)[nH]3)CC2)cc1	0.17	1
TNKS1_active_004	CHEMBL3805896	COc1ccc(C(=O)C2CCN(C(=O)CCCc3cn4c(C)ccc4c(=O)[nH]3)CC2)cc1	0.18	1
TNKS1_active_005	CHEMBL4634919	CCN(C(=O)N1CCC(C(=O)c2ccc3cnn(C)c3c2)CC1)c1ccc(C(=O)O)cc1	0.19	1
TNKS1_active_006	CHEMBL3804939	COc1ccc(C(=O)C2CCN(C(=O)CCCc3nc4c(C)scc4c(=O)[nH]3)CC2)cc1C	0.19	1
TNKS1_active_007	CHEMBL4098188	O=C(CCc1nc2ccccc2c(=O)[nH]1)Nc1ccc(C(=O)Nc2cccc3cccnc23)cc1	0.2	3
TNKS1_active_008	CHEMBL4632401	CCN(C(=O)N1CCC(C(=O)c2ccc3c(C(=O)O)nn(C)c3c2)CC1)c1ccc(OC)cc1	0.25	1
TNKS1_active_009	CHEMBL4640008	CCN(C(=O)N1CCC(C(=O)c2ccc3cc(C(=O)O)n(C)c3c2)CC1)c1ccc(OC)cc1	0.26	1
TNKS1_active_010	CHEMBL4643690	CCN(C(=O)N1CCC(C(=O)c2ccc3ncn(C)c3c2)CC1)c1ccc(C(=O)O)cc1	0.27	1
TNKS1_active_011	CHEMBL3806049	CC(C)n1cc(C(=O)C2CCN(C(=O)CCCc3nc4c(ccn4C)c(=O)[nH]3)CC2)cn1	0.28	1
TNKS1_active_012	CHEMBL4639702	CCN(C(=O)N1CCC(C(=O)c2ccc3c(cnn3C)c2)CC1)c1ccc(C(=O)O)cc1	0.29	1
TNKS1_active_013	CHEMBL4638701	O=c1[nH]c(SCCN2CCN(CCSc3nc4ccccc4c(=O)[nH]3)CC2)nc2ccccc12	0.3	1
TNKS1_active_014	CHEMBL3110106	N#Cc1ccc(O[C@H]2CC[C@H](NC(=O)CCSc3nc4ccccc4c(=O)[nH]3)CC2)cc1Cl	0.4	1
TNKS1_active_015	CHEMBL4645574	CCN(C(=O)N1CCC(C(=O)c2ccc3c(c2)cc(C(=O)O)n3C)CC1)c1ccc(OC)cc1	0.44	1
TNKS1_active_016	CHEMBL4458129	Cc1cc(F)cc2c(=O)[nH]c(-c3ccc(C(C)(C)O)cc3)nc12	0.5	1
TNKS1_active_017	CHEMBL4101890	Cc1cc(N2C[C@@H](C)N[C@@H](C)C2)ncc1-c1ccc(-c2nc3c(ccn3C)c(=O)[nH]2)cc1	0.5393	2
TNKS1_active_018	CHEMBL4567515	Cc1cc(F)cc2c(=O)[nH]c(-c3ccc(C4CCNCC4)cc3)nc12.Cl	0.7	1
TNKS1_active_019	CHEMBL5070513	CCOc1ccc(-c2nnc([C@H]3C[C@H](NC(=O)c4ccnc5cccnc45)C3)n2-c2ccc(C)s2)nc1	0.73	1
TNKS1_active_020	CHEMBL5274873	Cc1ccc2c(=O)[nH]c(-c3ccc(C(C)(C)O)cc3)nn12	0.9	1
TNKS1_active_021	CHEMBL5199558	Cc1ccc2c(=O)[nH]c(-c3ccc(C(C)(C)O)cc3)cn12	0.9	3
TNKS1_active_022	CHEMBL3966355	COc1ccc2c(c1)CC1(CCN(CC(=O)N(Cc3nc4c(c(=O)[nH]3)COCC4)CC3CC3)CC1)C2=O	1	1
TNKS1_active_023	CHEMBL2425812	O=C(CCc1cccc(Cl)c1Cl)Nc1nnc2n1CCS2	1	1
TNKS1_active_024	CHEMBL5206816	N#Cc1ccc(N2CCN(Cc3ccc4c(c3)NC(=O)C3CCCNC43)CC2)nc1	1	1
TNKS1_active_025	CHEMBL5272750	Cc1ccc2c(=O)[nH]c(-c3ccc([C@](C)(O)CCO)cc3)cn12	1	1
TNKS1_active_026	CHEMBL5271127	Cc1ccc2c(=O)[nH]c(-c3ccc(C4(O)CC4)cc3)cn12	1	1
TNKS1_active_027	CHEMBL5086139	CCOc1ccc(-c2nnc([C@H]3C[C@H](NC(=O)c4cccc5nc(C)cnc45)C3)n2-c2ccccc2F)nc1	1.1	1
TNKS1_activ	CHEMBL46	CS(=O)(=O)c1ccc(-	1.1	1

e_028	34146	c2nnc([C@H]3C[C@H](NC(=O)c4ccnc5cccnc45)C3)n2-c2ccccc2F)nc1		
TNKS1_active_029	CHEMBL4588170	Cc1cccc2c(=O)[nH]c(-c3ccc(C(C)(C)O)cc3)nc12	1.4	1
TNKS1_active_030	CHEMBL4551200	Cc1cc(F)cc2c(=O)[nH]c(-c3ccc(C(C)(C)OCCO)cc3)nc12	1.5	1
TNKS1_active_031	CHEMBL5085591	CCOc1ccc(-c2nnc([C@H]3C[C@H](NC(=O)c4cc(F)cc5nccnc45)C3)n2-c2ccccc2F)nc1	1.5	1
TNKS1_active_032	CHEMBL5093078	CCOc1ccc(-c2nnc([C@H]3C[C@H](NC(=O)c4cccc5ncc(C)nc45)C3)n2-c2ccccc2F)nc1	1.5	1
TNKS1_active_033	CHEMBL5279709	Cc1csc2c(=O)[nH]c(-c3ccc(C(C)(C)O)cc3)nc12	1.5	1
TNKS1_active_034	CHEMBL5281076	Cc1ccc2c(=O)[nH]c(-c3ccc(C(C)(C)OCCO)cc3)cn12	1.5	1
TNKS1_active_035	CHEMBL5091214	CCOc1ccc(-c2nnc([C@H]3C[C@H](NC(=O)c4ccnc5cc(F)cnc45)C3)n2-c2ccc(C)s2)nc1	1.6	1
TNKS1_active_036	CHEMBL5093991	Cc1cccc(-c2nnc([C@H]3C[C@H](NC(=O)c4cc(F)cc5nccnc45)C3)n2-c2ccccc2F)n1	1.6	1
TNKS1_active_037	CHEMBL5071436	O=C(N[C@H]1C[C@H](c2nnc(-c3ccccn3)n2-c2ccccc2F)C1)c1cccc2nccnc12	1.6	1
TNKS1_active_038	CHEMBL5280682	Cc1ccc2c(=O)[nH]c(-c3ccc([C@@](C)(O)CCO)cc3)cn12	1.6	1
TNKS1_active_039	CHEMBL5082525	Cc1cccc(-c2nnc([C@H]3C[C@H](NC(=O)c4cccc5nccnc45)C3)n2-c2ccccc2F)n1	1.7	1
TNKS1_active_040	CHEMBL3589282	COc1ccc(-c2cc3c(c(=O)[nH]2)CCCN3C)cc1	1.7	1
TNKS1_active_041	CHEMBL5269584	Cc1ccc2c(=O)[nH]c(-c3ccc([C@@](C)(O)CO)cc3)cn12	1.7	1
TNKS1_active_042	CHEMBL4636903	O=C(N[C@H]1C[C@H](c2nnc(-c3ccccn3)n2-c2ccccc2F)C1)c1ccnc2cc(F)cnc12	1.8	1
TNKS1_active_043	CHEMBL4643373	CS(=O)(=O)c1ccc(-c2nnc([C@H]3C[C@H](NC(=O)c4cccc5cccnc45)C3)n2-c2ccccc2F)nc1	1.8	1
TNKS1_active_044	CHEMBL5275576	Cc1ccc2c(=O)[nH]c(-c3ccc(C4(O)COC4)cc3)cn12	1.8	1
TNKS1_active_045	CHEMBL4584689	Cc1cc(F)cc2c(=O)[nH]c(-c3ccc(N4CCN(C)CC4)cc3)nc12	1.8	1
TNKS1_active_046	CHEMBL5074738	O=C(N[C@H]1C[C@H](c2nnc(-c3ccc(OC(F)F)cn3)n2-c2ccccc2F)C1)c1cccc2nccnc12	1.9	1
TNKS1_active_047	CHEMBL5090849	CCOc1ccc(-c2nnc([C@H]3C[C@H](NC(=O)c4cccc5nccnc45)C3)n2-c2ccccc2F)nc1	1.975	2
TNKS1_active_048	CHEMBL2314698	COc1ccccc1NC(=O)c1ccc(NC(=O)CCc2nc3ccccc3c(=O)[nH]2)cc1	2	2
TNKS1_active_049	CHEMBL4640917	O=C(N[C@H]1C[C@H](c2nnc(-c3ccc(OC(F)F)cn3)n2-c2ccccc2F)C1)c1ccnc2cc(F)cnc12	2	1
TNKS1_active_050	CHEMBL5278616	Cc1ccc2c(=O)[nH]c(-c3ccc([C@](C)(O)CO)cc3)cn12	2	1
TNKS1_active_051	CHEMBL5087403	O=C(N[C@H]1C[C@H](c2nnc(-c3ccccn3)n2-c2ccccc2F)C1)c1cc(F)cc2nccnc12	2	1
TNKS1_active_052	CHEMBL4632685	Cc1cccc(-c2nnc([C@H]3C[C@H](NC(=O)c4ccnc5cc(F)cnc45)C3)n2-c2ccccc2F)n1	2.2	1
TNKS1_active_053	CHEMBL4450725	Cc1cc(F)cc2c(=O)[nH]c(-c3ccc(C4CCN(C)CC4)cc3)nc12	2.2	1
TNKS1_active_054	CHEMBL4638483	Cc1cccc(-c2nnc([C@H]3C[C@H](NC(=O)c4ccnc5cccnc45)C3)n2-c2ccccc2F)n1	2.2	1
TNKS1_active_055	CHEMBL4452246	COc1ccccc1N1CCN(c2nc3c(c(=O)[nH]2)CSCC3)CC1	2.3	1
TNKS1_active_056	CHEMBL2431805	O=c1[nH]c(-c2ccc(C(F)(F)F)cc2)nc2c1CCSC2	2.344	3
TNKS1_active_057	CHEMBL5082704	CCOc1ccc(-c2nnc([C@H]3C[C@H](NC(=O)c4cccc5cccnc45)C3)n2-c2ccc(C)s2)nc1	2.4	1

TNKS1_active_058	CHEMBL5077466	O=C(N[C@H]1C[C@H](c2nnc(-c3ccc(OC(F)F)cn3)n2-c2ccccc2F)C1)c1cc(F)cc2nccnc12	2.4	1
TNKS1_active_059	CHEMBL4644156	O=C(N[C@H]1C[C@H](c2nnc(-c3ccccn3)n2-c2ccccc2F)C1)c1ccnc2cccnc12	2.4	1
TNKS1_active_060	CHEMBL3110119	O=C(CCSc1nc2c(c(=O)[nH]1)CCCC2)N[C@H]1CC[C@H](c2nnc(-c3ccccc3)o2)CC1	2.5	1
TNKS1_active_061	CHEMBL3895038	COc1ccc2c(c1)CC1(CCN(CC(=O)N(Cc3nc4c(c(=O)[nH]3)COCC4)CC(F)F)CC1)C2=O	2.5	1
TNKS1_active_062	CHEMBL4069862	c1ccc(C2=NC3=C(CCSC3)CN2)cc1	2.512	1
TNKS1_active_063	CHEMBL4453770	COc1ccc(N2CCN(c3nc4c(c(=O)[nH]3)CSCC4)CC2)cc1	2.6	1
TNKS1_active_064	CHEMBL4646640	O=C(N[C@H]1C[C@H](c2nnc(-c3ccncn3)n2-c2ccccc2Cl)C1)c1ccnc2cccnc12	2.6	1
TNKS1_active_065	CHEMBL5093410	CCOc1ccc(-c2nnc([C@H]3C[C@H](NC(=O)c4cccc5nc(C)c(C)nc45)C3)n2-c2ccccc2F)nc1	2.6	1
TNKS1_active_066	CHEMBL4454164	O=c1[nH]c(N2CCC3(CC2)CNc2cccc(F)c23)nc2c1CCCC2	2.8	1
TNKS1_active_067	CHEMBL5082990	CCOc1ccc(-c2nnc([C@H]3C[C@H](NC(=O)c4ccnc5cc(F)cnc45)C3)n2-c2ccncc2)nc1	2.9	1
TNKS1_active_068	CHEMBL4647488	O=C(N[C@H]1C[C@H](c2nnc(-c3ccc(OC(F)F)cn3)n2-c2ccccc2F)C1)c1ccnc2cccnc12	2.9	1
TNKS1_active_069	CHEMBL5269897	CC(C)(O)c1ccc(-c2cn3cc(F)cc3c(=O)[nH]2)cc1	2.9	1
TNKS1_active_070	CHEMBL2425810	O=C(CCc1c(Cl)cccc1Cl)Nc1nnc2n1CCS2	3	1
TNKS1_active_071	CHEMBL5190651	O=C(Nc1cccc2c(=O)[nH]ccc12)[C@H]1CCCNC1	3	1
TNKS1_active_072	CHEMBL125244	Cc1cccc2c(O)nc(-c3ccc(O)cc3)nc12	3	1
TNKS1_active_073	CHEMBL3918110	COc1ccc(C(=O)C2CCN([C@H]3CCN(Cc4nc5c(c(=O)[nH]4)COCC5)C3=O)CC2)cc1C	3	1
TNKS1_active_074	CHEMBL3952089	O=c1[nH]nc2c3c(cccc13)NC1=C2CCCC1	3.2	1
TNKS1_active_075	CHEMBL4533577	O=c1[nH]c(N2CCC3(CC2)CNc2cc(F)cc(F)c23)nc2c1CCCC2	3.3	1
TNKS1_active_076	CHEMBL541203	Cc1cccc2c(O)nc(-c3ccc(N)cc3)nc12	3.3	1
TNKS1_active_077	CHEMBL4639869	O=C(N[C@H]1C[C@H](c2nnc(-c3nccs3)n2-c2ccccc2F)C1)c1ccnc2cccnc12	3.3	1
TNKS1_active_078	CHEMBL4585076	Cc1cccc2c(=O)[nH]c(N3CCC4(CC3)C(=O)N(CCO)c3cc(F)cc(F)c34)nc12	3.4	1
TNKS1_active_079	CHEMBL5284314	CC(C)(O)c1ccc(-c2cn3c(F)c(F)cc3c(=O)[nH]2)cc1	3.5	1
TNKS1_active_080	CHEMBL4633567	N#Cc1ccc2c(c1)[nH]c(=O)n2C12CC(c3nnc(-c4ccncn4)n3-c3ccccc3Cl)(C1)C2	3.6	1
TNKS1_active_081	CHEMBL4586580	CC(C)(C)c1ccc(-c2nc3ccc(F)cc3c(=O)[nH]2)cc1	3.6	1
TNKS1_active_082	CHEMBL5277254	Cc1ncc2c(=O)[nH]c(-c3ccc(C(C)(C)C)cc3)cn12	3.8	1
TNKS1_active_083	CHEMBL3805627	Cc1ccc2c(=O)[nH]c(CCCC(=O)N3CCC(Oc4ccc(C#N)cc4)CC3)nn12	3.9	1
TNKS1_active_084	CHEMBL3889537	O=C(CN1CCC2(CC1)Cc1cc(F)ccc1C2=O)N(Cc1nc2c(c(=O)[nH]1)COCC2)CC1CC1	4	1
TNKS1_active_085	CHEMBL1086580	Oc1nc(-c2ccc(C(F)(F)F)cc2)nc2c1CSCC2	4	22
TNKS1_active_086	CHEMBL5286471	CC(C)(C)c1ccc(-c2nc(O)c3c(n2)CCSC3)cc1	4	1
TNKS1_activ	CHEMBL31	O=C(CCSc1nc2ccccc2c(=O)[nH]1)N[C@H]1CC[C@H](c2nnc(-	4.1	1

e_087	10117	c3ccccc3)o2)CC1		
TNKS1_active_088	CHEMBL5176874	COc1ccc(NC(=O)c2ccc(NC(=O)N3CCN(C(=O)c4cc(Cc5n[nH]c(=O)c6ccccc56)ccc4F)CC3)cc2)cc1	4.15	2
TNKS1_active_089	CHEMBL4643964	CCOc1ccc(-c2nnc([C@H]3C[C@H](NC(=O)c4ccnc5cccnc45)C3)n2-c2ccccc2F)nc1	4.31	2
TNKS1_active_090	CHEMBL5077896	CCOc1ccc(-c2nnc([C@H]3C[C@H](NC(=O)c4ccnc5cccnc45)C3)n2-c2cccnc2)nc1	4.5	1
TNKS1_active_091	CHEMBL5078506	CCOc1ccc(-c2nnc([C@H]3C[C@H](NC(=O)c4ccccn4)C3)n2-c2ccc(C)s2)nc1	4.6	1
TNKS1_active_092	CHEMBL4463639	CN1CCCc2c1nc(N1CCC3(CC1)C(=O)N(C)c1cc(OCCO)cc(F)c13)[nH]c2=O	4.7	1
TNKS1_active_093	CHEMBL4449621	O=C1Nc2cccc(F)c2C12CCN(c1nc3c(c(=O)[nH]1)CCCC3)CC2	4.7	1
TNKS1_active_094	CHEMBL4538780	COc1cccc(N2CCN(c3nc4c(c(=O)[nH]3)CSCC4)CC2)c1	4.8	1
TNKS1_active_095	CHEMBL4441837	O=C1Nc2cccc(Cl)c2C12CCN(c1nc3c(c(=O)[nH]1)CCCC3)CC2	4.9	1
TNKS1_active_096	CHEMBL4641515	O=C(N[C@H]1C[C@H](c2nnc(-c3ccncn3)n2-c2ccccc2Cl)C1)c1ccccn1	4.9	1
TNKS1_active_097	CHEMBL3968203	CC1(C)OC(=O)N([C@H]2CC[C@H](n3c(=O)[nH]c4cc(Cl)ccc43)CC2)[C@H]1c1ccccc1	4.98	1
TNKS1_active_098	CHEMBL2425809	O=C(CCc1ccccc1Cl)Nc1nnc2n1CCS2	5	1
TNKS1_active_099	CHEMBL2425813	O=C(CCc1ccc(Cl)cc1Cl)Nc1nnc2n1CCS2	5	1
TNKS1_active_100	CHEMBL3945783	COc1ccc(C(=O)C2CCN([C@H]3CCN(Cc4nc5ccccc(=O)c5[nH]4)C3=O)CC2)cc1	5	1
TNKS1_active_101	CHEMBL3916481	O=C(c1ccccc1)C1CCN(CC(=O)N(Cc2nc3c(c(=O)[nH]2)COCC3)CC(F)F)CC1	5	1
TNKS1_active_102	CHEMBL3763870	COc1cccc2c(=O)[nH]c(-c3ccc(C)cc3)cc12	5.1	1
TNKS1_active_103	CHEMBL5070921	CCOc1ccc(-c2nnc([C@H]3C[C@H](NC(=O)c4ccccn4)C3)n2-c2ccccc2)nc1	5.2	1
TNKS1_active_104	CHEMBL4441713	O=c1[nH]c(N2CCC3(CC2)CNc2cccc(Cl)c23)nc2c1CCCC2	5.3	1
TNKS1_active_105	CHEMBL5072185	CCOc1ccc(-c2nnc([C@H]3C[C@H](NC(=O)c4ccnc5cccnc45)C3)n2-c2ccncc2)nc1	5.4	1
TNKS1_active_106	CHEMBL2381935	CC1(C)OC(=O)N([C@H]2CC[C@H](NC(=O)c3ccnc4cccnc34)CC2)[C@H]1c1ccccc1	5.46	1
TNKS1_active_107	CHEMBL4442053	CN1CCCc2c1nc(N1CCC3(CC1)C(=O)N(CS(C)(=O)=O)c1cc(F)cc(F)c13)[nH]c2=O	5.5	1
TNKS1_active_108	CHEMBL5279214	CC(C)(O)c1ccc(-c2cn3cccc3c(=O)[nH]2)cc1	5.5	1
TNKS1_active_109	CHEMBL3098937	Cc1cccc2c(=O)[nH]c(-c3ccc(Br)cc3)nc12	5.5	2
TNKS1_active_110	CHEMBL5078858	CCOc1ccc(-c2nnc([C@H]3C[C@H](NC(=O)c4ccnc5cc(F)cnc45)C3)n2-c2cccnc2)nc1	5.8	1
TNKS1_active_111	CHEMBL5089832	CCOc1ccc(-c2nnc([C@H]3C[C@H](NC(=O)c4ccnc5cccnc45)C3)n2-c2ccccn2)nc1	5.8	1
TNKS1_active_112	CHEMBL4469043	O=c1[nH]c(N2CCC3(CC2)CNc2cc(Cl)ccc23)nc2c1CCCC2	5.8	1
TNKS1_active_113	CHEMBL3956961	O=C(c1ccc(F)cc1)C1CCN(C2CCN(Cc3nc4c(c(=O)[nH]3)COCC4)C2=O)CC1	6	1
TNKS1_active_114	CHEMBL2419706	COc1ccc(C(=O)C2CCN(CC(=O)N(Cc3nc4c(c(=O)[nH]3)COCC4)CC3CC3)CC2)cc1	6	2
TNKS1_active_115	CHEMBL3956527	COCCOc1ccc(C(=O)C2CCN(CC(=O)NCc3nc4c(c(=O)[nH]3)COCC4)CC2)cc1	6	1
TNKS1_active_116	CHEMBL4532667	CN1CCCc2c1nc(N1CCC3(CC1)C(=O)N(CC#N)c1cc(F)cc(F)c13)[nH]c2=O	6.1	1

TNKS1_active_117	CHEMBL5276381	CC(C)(O)c1ccc(-c2cn3c(F)ccc3c(=O)[nH]2)cc1	6.1	1
TNKS1_active_118	CHEMBL3110101	O=C(CCSc1nc2ccccc2c(=O)[nH]1)N[C@H]1CC[C@H](Oc2ccncc2)CC1	6.2	1
TNKS1_active_119	CHEMBL4470218	COc1cc(F)c2c(c1)N(C)C(=O)C21CCN(c2nc3c(c(=O)[nH]2)CCCN3C)CC1	6.2	1
TNKS1_active_120	CHEMBL4080549	c1ccc(-c2ncc3c(n2)CCSC3)cc1	6.31	1
TNKS1_active_121	CHEMBL4095003	N#Cc1ccc2c(c1)[nH]c(=O)n2[C@H]1C[C@H](c2nnc(-c3ccncn3)n2-c2ccccc2Cl)C1	6.31	7
TNKS1_active_122	CHEMBL4572668	COCCOc1cc(F)c2c(c1)N(C)C(=O)C21CCN(c2nc3c(c(=O)[nH]2)CCCN3C)CC1	6.4	1
TNKS1_active_123	CHEMBL5188771	COc1ccccc1NC(=O)c1ccc(NC(=O)N2CCN(C(=O)c3cc(Cc4n[nH]c(=O)c5ccccc45)ccc3F)CC2)cc1	6.45	1
TNKS1_active_124	CHEMBL4059827	O=C1SCCc2nc(-c3ccc(C(F)(F)F)cc3)[nH]c(=O)c21	6.4785	2
TNKS1_active_125	CHEMBL3098940	Cc1ccc(-c2nc3c(C)cccc3c(=O)[nH]2)cc1	6.7	1
TNKS1_active_126	CHEMBL4483558	O=C1N(CCO)c2cccc(Cl)c2C12CCN(c1nc3c(c(=O)[nH]1)CCCC3)CC2	6.8	1
TNKS1_active_127	CHEMBL4441063	Cn1ccc2c(=O)[nH]c(N3CCC4(CC3)CN(CCO)c3cc(F)cc(F)c34)nc21	7	1
TNKS1_active_128	CHEMBL3925030	O=C(CN1CCC(C(=O)c2ccc(Cl)cc2)CC1)NCc1nc2c(c(=O)[nH]1)COCC2	7	1
TNKS1_active_129	CHEMBL3921853	COc1ccc(C(=O)C2CCN([C@H]3CCN(Cc4nc5ccccc(=O)c5[nH]4)C3=O)CC2)cc1C	7	1
TNKS1_active_130	CHEMBL3901232	CC(C)(C)OC(=O)N1CCC2=C(C1)Nc1cccc3c(=O)[nH]nc2c13	7	1
TNKS1_active_131	CHEMBL4469038	O=C1Nc2cc(Cl)cc(Cl)c2C12CCN(c1nc3c(c(=O)[nH]1)CCCC3)CC2	7.1	1
TNKS1_active_132	CHEMBL4640597	O=C(N[C@H]1C[C@H](c2nnc(-c3ccc(OC(F)F)cn3)n2-c2ccccc2F)C1)c1ccc(F)cn1	7.4	1
TNKS1_active_133	CHEMBL4529394	O=C1Nc2cc(F)cc(F)c2C12CCN(c1nc3c(c(=O)[nH]1)CCCC3)CC2	7.5	1
TNKS1_active_134	CHEMBL2419698	O=C(c1ccc(F)cc1)C1CCN(CC(=O)N(Cc2nc3c(c(=O)[nH]2)COCC3)Cc2cccs2)CC1	7.5	2
TNKS1_active_135	CHEMBL4545573	CC(C)(O)c1ccc(-c2nc3ccc(F)c(F)c3c(=O)[nH]2)cc1	7.5	1
TNKS1_active_136	CHEMBL3589283	CN1CCCc2c1cc(-c1ccc(C(F)(F)F)cc1)[nH]c2=O	7.5	1
TNKS1_active_137	CHEMBL5274581	N#Cc1ccc(Oc2ccc(NC(=O)CCSc3nc4ccccc4c(=O)[nH]3)cc2)cc1	7.6	1
TNKS1_active_138	CHEMBL3110100	N#Cc1ccc(O[C@H]2CC[C@H](NC(=O)CCSc3nc4ccccc4c(=O)[nH]3)CC2)cc1	7.6	1
TNKS1_active_139	CHEMBL4461426	O=c1[nH]c(N2CCC3(CC2)CNc2cc(F)ccc23)nc2c1CCCC2	7.6	1
TNKS1_active_140	CHEMBL5280345	Cc1cc2c(=O)[nH]c(-c3ccc(C(C)(C)O)cc3)cn2c1	7.9	1
TNKS1_active_141	CHEMBL3891789	O=C(c1ccc(OC(F)(F)F)cc1)C1CCN(CC(=O)N(Cc2nc3c(c(=O)[nH]2)COCC3)CC2CC2)CC1	8	1
TNKS1_active_142	CHEMBL2425801	O=C(CCC1CCCCC1)Nc1nnc2n1CCS2	8	1
TNKS1_active_143	CHEMBL3955207	O=C(CN1CCC2(CC1)Cc1cc(F)ccc1C2=O)NCc1nc2c(c(=O)[nH]1)COCC2	8	1
TNKS1_active_144	CHEMBL4437173	CC(C)(O)c1ccc(-c2nc3c(F)cc(F)cc3c(=O)[nH]2)cc1	8.1	4
TNKS1_active_145	CHEMBL5281885	CC(C)(O)c1ccc(-c2cn3c(CO)ccc3c(=O)[nH]2)cc1	8.2	1
TNKS1_activ	CHEMBL45	O=C1N(CCO)c2cccc(F)c2C12CCN(c1nc3c(c(=O)[nH]1)CCCC3)CC2	8.2	1

e_146	77914			
TNKS1_active_147	CHEMBL4566625	Cc1cccc2c(=O)[nH]c(N3CCC4(CC3)CN(CCO)c3cc(F)cc(F)c34)nc12	8.5	1
TNKS1_active_148	CHEMBL5078964	CCOc1ccc(-c2nnc([C@H]3C[C@H](NC(=O)c4ccnc5cc(F)cnc45)C3)n2-c2ccccn2)nc1	8.5	1
TNKS1_active_149	CHEMBL5094240	O=C(N[C@H]1C[C@H](c2nnc(-c3ccc(OC4CC4)cn3)n2-c2ccccc2F)C1)c1ccccn1	8.6	1
TNKS1_active_150	CHEMBL3952773	COc1ccc2c(c1)CC1(CCN([C@H]3CCN(Cc4nc5c(c(=O)[nH]4)COCC5)C3=O)CC1)C2=O	8.6	1
TNKS1_active_151	CHEMBL4290134	CC(C)(C)c1ccc(-c2nc3ccccc3c(=O)[nH]2)cc1	8.7	1
TNKS1_active_152	CHEMBL4636717	O=C(N[C@H]1C[C@H](c2nnc(-c3ccc(OC(F)F)cn3)n2-c2ccccc2F)C1)c1ccccn1	8.8	1
TNKS1_active_153	CHEMBL4529965	NC(=O)c1cccc2c1O/C(=C\c1ccc(C(=O)NCCc3nc4c(C(N)=O)cccc4[nH]3)cc1)C2=O	8.8	1
TNKS1_active_154	CHEMBL3945618	O=C(c1ccc(F)cc1)C1CCN(CC(=O)N(Cc2nc3c(c(=O)[nH]2)COCC3)Cc2ccccc2F)CC1	9	1
TNKS1_active_155	CHEMBL2425800	O=C(CCC1CCCC1)Nc1nnc2n1CCS2	9	2
TNKS1_active_156	CHEMBL2381634	Cc1cc(=O)[nH]c2cc(-c3ccccc3Cl)ccc12	9	1
TNKS1_active_157	CHEMBL2381639	COCCNC(=O)c1ccc(-c2ccc3c(C)cc(=O)[nH]c3c2)c(Cl)c1	9	1
TNKS1_active_158	CHEMBL3903965	CCOc1ccc(C(=O)C2CCN(CC(=O)NCc3nc4c(c(=O)[nH]3)COCC4)CC2)cc1	9	1
TNKS1_active_159	CHEMBL4109152	COc1ccc(C(=O)C2CCN([C@@H]3CCCN(CCC4NC(=O)C5=C(CCOC5)N4)C3=O)CC2)cc1	9	1
TNKS1_active_160	CHEMBL4640220	CS(=O)(=O)c1ccc(-c2nnc([C@H]3C[C@H](NC(=O)c4ccccn4)C3)n2-c2ccccc2F)nc1	9.2	1
TNKS1_active_161	CHEMBL4633531	CCOc1ccc(-c2nnc([C@H]3C[C@H](n4c(=O)[nH]c5cc(C#N)ccc54)C3)n2-c2ccccc2Cl)nc1	9.3	1
TNKS1_active_162	CHEMBL5077379	CCOc1ccc(-c2nnc(C34CC(NC(=O)c5ccnc6cccnc56)(C3)C4)n2-c2ccncc2)nc1	9.4	1
TNKS1_active_163	CHEMBL5280167	CC(C)(O)c1ccc(-c2cn3cc(Cl)cc3c(=O)[nH]2)cc1	9.4	1
TNKS1_active_164	CHEMBL4461149	O=C1N(CCO)c2cc(Cl)cc(Cl)c2C12CCN(c1nc3c(c(=O)[nH]1)CCCC3)CC2	9.5	1
TNKS1_active_165	CHEMBL3897483	COc1ccc(C(=O)C2CCN([C@H]3CCN(Cc4nc5c(c(=O)[nH]4)COCC5)C3=O)CC2)cc1	9.5	1
TNKS1_active_166	CHEMBL5094543	CCOc1ccc(-c2nnc(C34CC(NC(=O)c5ccnc6cccnc56)(C3)C4)n2-c2cccnc2)nc1	9.6	1
TNKS1_active_167	CHEMBL4466181	CN1CCCc2c1nc(N1CCC3(CC1)C(=O)N(C)c1cc(OCC(=O)O)cc(F)c13)[nH]c2=O	9.9	1
TNKS1_active_168	CHEMBL3928915	O=C(CN1CCC(C(=O)c2ccc(OCC3CC3)cc2)CC1)NCc1nc2c(c(=O)[nH]1)COCC2	10	1
TNKS1_active_169	CHEMBL3892864	O=C(c1ccc(F)cc1)C1CCN(CC(=O)N(Cc2nc3c(c(=O)[nH]2)COCC3)C[C@@H]2CCCO2)CC1	10	1
TNKS1_active_170	CHEMBL346886	Cc1cccc2c(O)nc(-c3ccc(C(F)(F)F)cc3)nc12	10	1
TNKS1_active_171	CHEMBL3940284	C[C@@H](C1CC1)N(Cc1nc2c(c(=O)[nH]1)COCC2)C(=O)CN1CCC(C(=O)c2ccc(F)cc2)CC1	10	1
TNKS1_active_172	CHEMBL3983088	O=C(c1ccc(F)cc1)C1CCN(CC(=O)N(Cc2nc3c(c(=O)[nH]2)COCC3)CC(F)F)CC1	10.2	1
TNKS1_active_173	CHEMBL4460022	Cn1ccc2c(=O)[nH]c(N3CCC4(CC3)C(=O)N(CCO)c3cc(F)cc(F)c34)nc21	10.2	1
TNKS1_active_174	CHEMBL4521982	O=c1[nH]c(N2CCC3(CC2)CNc2cc(Cl)cc(Cl)c23)nc2c1CCCC2	10.4	1
TNKS1_active_175	CHEMBL4447252	CC(C)(O)c1ccc(-c2nc3ccc(F)cc3c(=O)[nH]2)cc1	10.5	1

TNKS1_active_176	CHEMBL4446044	CN1CCCc2c1nc(N1CCC3(CC1)C(=O)N(C)c1cc(N4CCOCC4)cc(F)c13)[nH]c2=O	10.5	1
TNKS1_active_177	CHEMBL3905051	COc1ccc(C(=O)C2CCN(CC(=O)NCc3nc4c(c(=O)[nH]3)COCC4)CC2)cc1Cl	10.5	1
TNKS1_active_178	CHEMBL4448872	O=c1[nH]c(N2CCC3(CC2)CN(CCO)c2cc(F)cc(F)c23)nc2c1CCCC2	10.6	4
TNKS1_active_179	CHEMBL4439372	CN1CCCc2c1nc(N1CCC3(CC1)C(=O)N(C)c1cc(F)cc(F)c13)[nH]c2=O	10.7	1
TNKS1_active_180	CHEMBL2419705	O=C(c1ccc(F)cc1)C1CCN(CC(=O)N(Cc2nc3c(c(=O)[nH]2)COCC3)CC2CC2)CC1	10.85	2
TNKS1_active_181	CHEMBL2381960	CC1(C)OC(=O)N([C@H]2CC[C@H](C(=O)Nc3cccc4cccnc34)CC2)[C@H]1c1ccccc1	10.9	1
TNKS1_active_182	CHEMBL2419702	CCCN(Cc1nc2c(c(=O)[nH]1)COCC2)C(=O)CN1CCC(C(=O)c2ccc(F)cc2)CC1	11	2
TNKS1_active_183	CHEMBL2381959	CC1(C)OC(=O)N(c2ccc(C(=O)Nc3ccnc4cccnc34)cc2)[C@H]1c1ccccc1	11	1
TNKS1_active_184	CHEMBL4457037	CN(C)CCOc1cc(F)c2c(c1)N(C)C(=O)C21CCN(c2nc3c(c(=O)[nH]2)CCCN3C)CC1	11.1	1
TNKS1_active_185	CHEMBL4466701	COCCN1C(=O)C2(CCN(c3nc4c(c(=O)[nH]3)CCCN4C)CC2)c2c(F)cc(F)cc21	11.7	1
TNKS1_active_186	CHEMBL4648016	O=c1[nH]c(SCCN2CCN(CCCc3nc(-c4ccccc4)no3)CC2)nc2ccccc12	11.7	1
TNKS1_active_187	CHEMBL4560087	O=c1[nH]c(N2CCC3(CC2)CN(CCO)c2cccc(F)c23)nc2c1CCCC2	11.8	1
TNKS1_active_188	CHEMBL3967549	COc1ccc(C(=O)C2CCN(CC(=O)NCc3nc4c(c(=O)[nH]3)COCC4)CC2)cc1C	12	1
TNKS1_active_189	CHEMBL3099716	CC1(C)OC(=O)N([C@H]2CC[C@H](c3cnc(N)c(-c4ncccn4)c3)CC2)[C@H]1c1ccccc1	12	2
TNKS1_active_190	CHEMBL4514589	CC(C)(O)c1ccc(-c2nc3c(Cl)cccc3c(=O)[nH]2)cc1	12	1
TNKS1_active_191	CHEMBL3986246	COc1ccc(C(=O)C2CCN(CC(=O)NCc3nc4c(c(=O)[nH]3)COCC4)CC2)cc1	12	1
TNKS1_active_192	CHEMBL5077249	CC(C)Oc1ccc(-c2nnc([C@H]3C[C@H](NC(=O)c4ccccn4)C3)n2-c2ccccc2F)nc1	12	1
TNKS1_active_193	CHEMBL4521841	O=C1Nc2cc(F)ccc2C12CCN(c1nc3c(c(=O)[nH]1)CCCC3)CC2	12	1
TNKS1_active_194	CHEMBL3935111	COc1ccc(C(=O)C2CCN(CC(=O)N(Cc3nc4c(c(=O)[nH]3)COCC4)CC(F)F)CC2)cc1	12	1
TNKS1_active_195	CHEMBL3944401	O=C(CN1CCC(C(=O)c2ccc(OCCC3CC3)cc2)CC1)NCc1nc2c(c(=O)[nH]1)COCC2	12	1
TNKS1_active_196	CHEMBL127101	COc1ccc(-c2nc(O)c3cccc(C)c3n2)cc1	12	1
TNKS1_active_197	CHEMBL5078514	CCOc1ccc(-c2nnc([C@H]3C[C@H](NC(=O)c4ccccn4)C3)n2-c2ccc(Cl)s2)nc1	12	1
TNKS1_active_198	CHEMBL2425808	O=C(CCc1ccccc1F)Nc1nnc2n1CCS2	12	1
TNKS1_active_199	CHEMBL3944854	O=C(CN1CCC(C(=O)c2ccc(OCC3CCC3)cc2)CC1)NCc1nc2c(c(=O)[nH]1)COCC2	12	1
TNKS1_active_200	CHEMBL4455904	CN1CCN(c2ccc(-c3nc4ccc(F)cc4c(=O)[nH]3)cc2)CC1	12	1
TNKS1_active_201	CHEMBL4581567	CN1CCCc2c1nc(N1CCC3(CC1)C(=O)N(CC1COC1)c1cc(F)cc(F)c13)[nH]c2=O	12.2	1
TNKS1_active_202	CHEMBL4582280	CN1CCN(c2cc(F)c3c(c2)N(C)C(=O)C32CCN(c3nc4c(c(=O)[nH]3)CCCN4C)CC2)CC1	12.3	1
TNKS1_active_203	CHEMBL4525942	CN1CCCc2c1nc(N1CCC3(CC1)C(=O)N(C)c1cc(OCCN4CCCC4)cc(F)c13)[nH]c2=O	12.4	1
TNKS1_active_204	CHEMBL5402698	O=C(c1cc(Cc2n[nH]c(=O)c3ccccc23)ccc1F)N1CCn2c(nnc2-c2ccc(Br)o2)C1	12.4	1
TNKS1_activ	CHEMBL23	CC1(C)OC(=O)N([C@H]2CC[C@H](NC(=O)c3cccc4cccnc34)CC2)[C@H]	12.44	2

e_205	81934	1c1ccccc1		
TNKS1_active_206	CHEMBL4464007	CN1CCCc2c1nc(N1CCC3(CC1)C(=O)N(CC(O)CO)c1cc(F)cc(F)c13)[nH]c2=O	12.5	1
TNKS1_active_207	CHEMBL3898270	CC[C@H](C)N(Cc1nc2c(c(=O)[nH]1)COCC2)C(=O)CN1CCC(C(=O)c2ccc(F)cc2)CC1	13	1
TNKS1_active_208	CHEMBL3964642	CC[C@H](C)CN(Cc1nc2c(c(=O)[nH]1)COCC2)C(=O)CN1CCC(C(=O)c2ccc(F)cc2)CC1	13	1
TNKS1_active_209	CHEMBL4109734	CC[C@@H](C)N(Cc1nc2c(c(=O)[nH]1)COCC2)C(=O)CN1CCC(C(=O)c2ccc(F)cc2)CC1	13	1
TNKS1_active_210	CHEMBL4442841	CN(C)c1cc(F)c2c(c1)N(C)C(=O)C21CCN(c2nc3c(c(=O)[nH]2)CCCN3C)CC1	13	1
TNKS1_active_211	CHEMBL3110107	O=C(CCc1nc2ccccc2c(=O)[nH]1)Nc1ccc(-c2nnc(-c3ccccc3)o2)cc1	13	1
TNKS1_active_212	CHEMBL5072993	CCOc1ccc(-c2nnc([C@H]3C[C@H](NC(=O)c4ccccn4)C3)n2-c2cccc(Cl)c2)nc1	13	1
TNKS1_active_213	CHEMBL5072404	CCOc1ccc(-c2nnc(C34CC(NC(=O)c5ccnc6cccnc56)(C3)C4)n2-c2ccccn2)nc1	13	1
TNKS1_active_214	CHEMBL3906833	CCCOc1ccc(C(=O)C2CCN(CC(=O)NCc3nc4c(c(=O)[nH]3)COCC4)CC2)cc1	13	1
TNKS1_active_215	CHEMBL4475367	CC(C)(O)c1ccc(-c2nc3ccccc3c(=O)[nH]2)cc1	13	1
TNKS1_active_216	CHEMBL4572625	O=C1Nc2cccc(Br)c2C12CCN(c1nc3c(c(=O)[nH]1)CCCC3)CC2	13.4	1
TNKS1_active_217	CHEMBL4445363	CN1CCCc2c1nc(N1CCC3(CC1)C(=O)N(CCO)c1cc(F)cc(F)c13)[nH]c2=O	13.5	1
TNKS1_active_218	CHEMBL45245	O=c1[nH]c2ccccc2c2ccccc12	13.9	2
TNKS1_active_219	CHEMBL4646010	O=C(N[C@H]1C[C@H](c2nnc(-c3ccccn3)n2-c2ccccc2F)C1)c1ccc(F)cn1	14	1
TNKS1_active_220	CHEMBL5174780	Cc1ccc(NC(=O)c2ccc(NC(=O)N3CCN(C(=O)c4cc(Cc5n[nH]c(=O)c6ccccc56)ccc4F)CC3)cc2)cc1	14	1
TNKS1_active_221	CHEMBL5289848	Cc1ncc2c(=O)[nH]c(-c3ccc(C(C)(C)O)cc3)cn12	14	1
TNKS1_active_222	CHEMBL3933577	O=C(c1ccc(F)cc1)C1CCN(CC(=O)N(Cc2nc3c(c(=O)[nH]2)COCC3)Cc2ccc(F)cc2F)CC1	14	1
TNKS1_active_223	CHEMBL3933931	COc1ccc(C(=O)C2CCN(CC(=O)NCc3nc4c(c(=O)[nH]3)CCCC4)CC2)cc1	14	1
TNKS1_active_224	CHEMBL3965278	C#CCN(Cc1nc2c(c(=O)[nH]1)COCC2)C(=O)CN1CCC(C(=O)c2ccc(F)cc2)CC1	14	1
TNKS1_active_225	CHEMBL3947374	CC(C)Oc1ccc(C(=O)C2CCN(CC(=O)NCc3nc4c(c(=O)[nH]3)COCC4)CC2)cc1	14	1
TNKS1_active_226	CHEMBL3940683	COCCN(Cc1nc2c(c(=O)[nH]1)COCC2)C(=O)CN1CCC(C(=O)c2ccc(OC)cc2)CC1	14	1
TNKS1_active_227	CHEMBL4633637	CCOc1ccc(-c2nnc([C@H]3C[C@H](NC(=O)c4ccccn4)C3)n2-c2ccccc2F)nc1	14	1
TNKS1_active_228	CHEMBL3941313	O=C(c1ccc(F)cc1)C1CCN(CC(=O)N(Cc2nc3c(c(=O)[nH]2)COCC3)CC(F)(F)F)CC1	14	1
TNKS1_active_229	CHEMBL4536612	CC(C)(OCCO)c1ccc(-c2nc3ccc(F)cc3c(=O)[nH]2)cc1	14	1
TNKS1_active_230	CHEMBL3110110	O=C(CCc1nc2ccccc2c(=O)[nH]1)N[C@H]1CC[C@H](c2nnc(-c3ccccc3)o2)CC1	14	1
TNKS1_active_231	CHEMBL4457667	O=C1Nc2cc(Cl)ccc2C12CCN(c1nc3c(c(=O)[nH]1)CCCC3)CC2	14.4	1
TNKS1_active_232	CHEMBL4452289	O=c1[nH]c(N2CCC3(CC2)COc2ccccc23)nc2c1CCCC2	14.6	1
TNKS1_active_233	CHEMBL3929005	COc1ccc(C(=O)C2CCN(C3CCN(Cc4nc5c(c(=O)[nH]4)COCC5)C3=O)CC2)cc1	15	1
TNKS1_active_234	CHEMBL3905010	COc1ccc(C(=O)C2CCN(C3CCN(Cc4ccc(Cl)c(C#N)c4)C3=O)CC2)cc1	15	1

TNKS1_active_235	CHEMBL4303453	COc1ccc(-n2c(SCCCN3C(=O)c4cccc5cccc(c45)C3=O)nnc2-c2ccncc2)cc1	15	1
TNKS1_active_236	CHEMBL3939501	O=c1[nH]nc2c3c(cccc13)NC1=C2CCCCCC1	15	1
TNKS1_active_237	CHEMBL3975140	O=C(c1ccc(OC(F)(F)F)cc1)C1CCN(CC(=O)N(Cc2nc3c(c(=O)[nH]2)COCC3)CC(F)F)CC1	15	1
TNKS1_active_238	CHEMBL3955426	CC#CCOc1ccc(C(=O)C2CCN(CC(=O)N(Cc3nc4c(c(=O)[nH]3)COCC4)CC3CC3)CC2)cc1	15	1
TNKS1_active_239	CHEMBL3827440	O=c1[nH]c(-c2ccc(Br)cc2)nc2c(O)cccc12	15	1
TNKS1_active_240	CHEMBL3938947	COc1ccc(CN(Cc2nc3c(c(=O)[nH]2)COCC3)C(=O)CN2CCC(C(=O)c3ccc(F)cc3)CC2)cc1	15	1
TNKS1_active_241	CHEMBL3930536	COc1ccc(C(=O)C2CCN(C3CCCN(Cc4nc5c(c(=O)[nH]4)COCC5)C3=O)CC2)cc1	15	1
TNKS1_active_242	CHEMBL3893294	O=c1[nH]nc2c3c(cccc13)NC1=C2CCCCC1	15.4	1
TNKS1_active_243	CHEMBL4556736	O=c1[nH]c(N2CCC3(CC2)CNc2cccc(Br)c23)nc2c1CCCC2	15.6	1
TNKS1_active_244	CHEMBL3955370	CC1(C)CCC2=C(C1)c1n[nH]c(=O)c3cccc(c13)N2	15.6	1
TNKS1_active_245	CHEMBL4641728	Cc1cccc(-c2nnc([C@H]3C[C@H](NC(=O)c4ccccn4)C3)n2-c2ccccc2F)n1	16	1
TNKS1_active_246	CHEMBL3946798	COc1cccc(C(=O)C2CCN(CC(=O)N(Cc3nc4c(c(=O)[nH]3)COCC4)CC(F)F)CC2)c1	16	1
TNKS1_active_247	CHEMBL3913243	N#Cc1ccc(CN(Cc2nc3c(c(=O)[nH]2)COCC3)C(=O)CN2CCC(C(=O)c3ccc(F)cc3)CC2)s1	16	1
TNKS1_active_248	CHEMBL3964994	COc1ccc(C(=O)C2CCN([C@@H]3CCN(CC(=O)Nc4nnc5n4CCS5)C3=O)CC2)cc1	16	1
TNKS1_active_249	CHEMBL2419699	O=C(c1ccc(F)cc1)C1CCN(CC(=O)N(Cc2ccccc2)Cc2nc3c(c(=O)[nH]2)COCC3)CC1	16	2
TNKS1_active_250	CHEMBL3964683	CCOc1ccc(C(=O)C2CCN(CC(=O)NCc3nc4c(c(=O)[nH]3)COCC4)CC2)cc1F	16	1
TNKS1_active_251	CHEMBL3961412	COc1ccc(C(=O)C2CCN(CC(=O)N(Cc3nc4c(c(=O)[nH]3)CCCC4)CC3CC3)CC2)cc1	16	1
TNKS1_active_252	CHEMBL4110589	C[C@H](c1ccccc1)N(Cc1nc2c(c(=O)[nH]1)COCC2)C(=O)CN1CCC(C(=O)c2ccc(F)cc2)CC1	16	1
TNKS1_active_253	CHEMBL4591423	O=c1[nH]c(N2CCC3(CC2)CN(CCO)c2cc(Cl)cc(Cl)c23)nc2c1CCCC2	16.4	1
TNKS1_active_254	CHEMBL3986804	CC(c1cccs1)N(Cc1nc2c(c(=O)[nH]1)COCC2)C(=O)CN1CCC(C(=O)c2ccc(F)cc2)CC1	16.5	1
TNKS1_active_255	CHEMBL3929392	O=C(c1ccc(F)cc1)C1CCN([C@H]2CCN(Cc3nc4c(c(=O)[nH]3)COCC4)C2=O)CC1	17	1
TNKS1_active_256	CHEMBL3927023	COc1ccc(C(=O)C2CCN(C3CCN(Cc4nc5c(c(=O)[nH]4)CCC5)C3=O)CC2)cc1	17	1
TNKS1_active_257	CHEMBL3952101	O=C(c1ccc(C(F)(F)F)cc1)C1CCN(CC(=O)N(Cc2nc3c(c(=O)[nH]2)COCC3)CC(F)F)CC1	17	1
TNKS1_active_258	CHEMBL4535636	O=c1[nH]c(N2CCC3(CC2)CN(CCO)c2cccc(Cl)c23)nc2c1CCCC2	17	1
TNKS1_active_259	CHEMBL4544771	CN1CCCc2c1nc(N1CCC3(CC1)C(=O)N(C)c1cc(OCCN4CCOCC4)cc(F)c13)[nH]c2=O	17.1	1
TNKS1_active_260	CHEMBL3978133	O=c1[nH]nc2c3c(cccc13)NC1=C2CCC1	17.5	1
TNKS1_active_261	CHEMBL4554146	O=C1N(CCO)c2cc(F)cc(F)c2C12CCN(c1nc3c(c(=O)[nH]1)CCCC3)CC2	17.85	2
TNKS1_active_262	CHEMBL3960803	O=C(c1ccc(Cl)cc1)C1CCN(CC(=O)N(Cc2nc3c(c(=O)[nH]2)COCC3)CC(F)F)CC1	18	1
TNKS1_active_263	CHEMBL3965681	COc1ccc2c(c1)CC1(CCN(C3CCN(Cc4nc5c(c(=O)[nH]4)COCC5)C3=O)CC1)C2=O	18	1
TNKS1_activ	CHEMBL24	CC(C)CN(Cc1nc2c(c(=O)[nH]1)COCC2)C(=O)CN1CCC(C(=O)c2ccc(F)cc2	18	2

e_264	19701	)CC1		
TNKS1_active_265	CHEMBL3977049	COc1ccc(C(=O)C2CCN(CC(=O)NCc3nc4c(c(=O)[nH]3)COCC4)CC2)cc1OC	18	1
TNKS1_active_266	CHEMBL3943283	Cc1ccc(C(=O)C2CCN(CC(=O)NCc3nc4c(c(=O)[nH]3)COCC4)CC2)cc1	18	1
TNKS1_active_267	CHEMBL3956490	O=C(c1ccc(F)cc1)C1CCN(CC(=O)N(Cc2nc3c(c(=O)[nH]2)COCC3)Cc2nc3ccccc3s2)CC1	18	1
TNKS1_active_268	CHEMBL4575275	O=C1Oc2ccccc2C12CCN(c1nc3c(c(=O)[nH]1)CCCC3)CC2	18.3	1
TNKS1_active_269	CHEMBL4438002	N#Cc1cccc2c1C1(CCN(c3nc4c(c(=O)[nH]3)CCCC4)CC1)CN2	18.5	1
TNKS1_active_270	CHEMBL4441668	CN1CCN(CCOc2cc(F)c3c(c2)N(C)C(=O)C32CCN(c3nc4c(c(=O)[nH]3)CCCN4C)CC2)CC1	18.7	1
TNKS1_active_271	CHEMBL3937313	COc1ccc(C(=O)C2CCN(C3CCN(Cc4nc5ccccc(=O)c5[nH]4)C3=O)CC2)cc1	19	1
TNKS1_active_272	CHEMBL3922251	O=C(c1ccc(F)cc1)C1CCN(CC(=O)N(CCF)Cc2nc3c(c(=O)[nH]2)COCC3)CC1	19	1
TNKS1_active_273	CHEMBL4454483	COc1ccccc1N1CCN(c2nc3c(c(=O)[nH]2)CCCC3)CC1	19.2	2
TNKS1_active_274	CHEMBL4098578	C1=Cc2ccc(-c3ccc(CNCCN4CCOCC4)cc3)cc2NC1	19.95	1
TNKS1_active_275	CHEMBL3929923	CC(C)(C)CN(Cc1nc2c(c(=O)[nH]1)COCC2)C(=O)CN1CCC(C(=O)c2ccc(F)cc2)CC1	20	1
TNKS1_active_276	CHEMBL4786379	Cc1cccc2c1c(=O)n(C[C@H]1CC[C@H](C(=O)Nc3ccncc3)CC1)c(=O)n2Cc1cccc(Cl)c1C#N	20	1
TNKS1_active_277	CHEMBL3939009	O=C(CN1CCC(C(=O)c2ccc3c(c2)CCO3)CC1)NCc1nc2c(c(=O)[nH]1)COCC2	20	1
TNKS1_active_278	CHEMBL4440292	COCCOC(C)(C)c1ccc(-c2nc3ccc(F)cc3c(=O)[nH]2)cc1	20	1
TNKS1_active_279	CHEMBL2425798	O=C(CC1CCCCC1)Nc1nnc2n1CCS2	20	1
TNKS1_active_280	CHEMBL3901978	COc1ccc(C(=O)C2CCN(CC(=O)NCc3nc(C)c(C)c(=O)[nH]3)CC2)cc1	20	1
TNKS1_active_281	CHEMBL3907294	CC(C)N(Cc1nc2c(c(=O)[nH]1)COCC2)C(=O)CN1CCC(C(=O)c2ccc(F)cc2)CC1	20	1
TNKS1_active_282	CHEMBL4539435	C[C@@H]1CN(c2cc(F)c3c(c2)N(C)C(=O)C32CCN(c3nc4c(c(=O)[nH]3)CCCN4C)CC2)C[C@H](C)N1	20.2	1
TNKS1_active_283	CHEMBL4476087	O=c1[nH]c(N2CCC3(CC2)CNc2ccccc23)nc2c1CCCC2	20.7	1
TNKS1_active_284	CHEMBL2381637	Cc1cc(=O)[nH]c2cc(-c3ccc(C(=O)O)cc3Cl)ccc12	21	1
TNKS1_active_285	CHEMBL4442606	O=C1OC2(CCN(c3nc4c(c(=O)[nH]3)CCCC4)CC2)c2ccccc21	21	1
TNKS1_active_286	CHEMBL4639146	Cc1cccc(-c2nnc([C@H]3C[C@H](NC(=O)c4ccc(F)cn4)C3)n2-c2ccccc2F)n1	21	1
TNKS1_active_287	CHEMBL3937250	O=C(c1ccc(F)cc1)C1CCN(CC(=O)N(Cc2cccc(F)c2)Cc2nc3c(c(=O)[nH]2)COCC3)CC1	21	1
TNKS1_active_288	CHEMBL4548930	CC(C)(O)c1ccc(-c2nc3cccc(F)c3c(=O)[nH]2)cc1	21	1
TNKS1_active_289	CHEMBL3897864	COc1ccc(C(=O)C2CCN(C3CCN(CC(=O)Nc4nnc5n4CCS5)C3=O)CC2)cc1	21	1
TNKS1_active_290	CHEMBL4553990	CN1CCC(Oc2cc(F)c3c(c2)N(C)C(=O)C32CCN(c3nc4c(c(=O)[nH]3)CCCN4C)CC2)CC1	21.2	1
TNKS1_active_291	CHEMBL4518341	CN1CCCc2c1nc(N1CCC3(CC1)CN(CCO)c1cc(F)cc(F)c13)[nH]c2=O	21.4	1
TNKS1_active_292	CHEMBL4639723	O=C(N[C@H]1C[C@H](c2nnc(-c3ccccn3)n2-c2ccccc2F)C1)c1ccccn1	22	1
TNKS1_active_293	CHEMBL2419710	CC(C)(O)c1ccc(-c2nc3c(c(=O)[nH]2)COCC3)cc1	22	1

TNKS1_active_294	CHEMBL2381638	Cc1cc(=O)[nH]c2cc(-c3ccc(C(=O)NCCN4CCOCC4)cc3Cl)ccc12	22	1
TNKS1_active_295	CHEMBL3985156	COc1cccc(CN(Cc2nc3c(c(=O)[nH]2)COCC3)C(=O)CN2CCC(C(=O)c3ccc(F)cc3)CC2)c1	23	1
TNKS1_active_296	CHEMBL2419697	O=C(CN1CCC(C(=O)c2ccc(F)cc2)CC1)NCc1nc2c(c(=O)[nH]1)COCC2	23	2
TNKS1_active_297	CHEMBL3941919	O=C(c1ccc(Cl)cc1)C1CCN(C2CCN(Cc3nc4c(c(=O)[nH]3)COCC4)C2=O)CC1	23	1
TNKS1_active_298	CHEMBL4574717	CN1CCCc2c1nc(N1CCC3(CC1)C(=O)N(C)c1cc(N4CCNCC4)cc(F)c13)[nH]c2=O	23.4	1
TNKS1_active_299	CHEMBL3108871	O=c1[nH]c2sccc2c2ccccc12	24	3
TNKS1_active_300	CHEMBL4113551	C[C@H](c1ccc(F)cc1)N(Cc1nc2c(c(=O)[nH]1)COCC2)C(=O)CN1CCC(C(=O)c2ccc(F)cc2)CC1	25	1
TNKS1_active_301	CHEMBL5276118	Cc1ccccc1-n1c(/C=C/c2nnc(-c3ccc(C#N)cc3)o2)nnc1-c1ccc(S(C)(=O)=O)cn1	25	1
TNKS1_active_302	CHEMBL3945237	COc1ccc(C(=O)C2CCN(C3CCN(Cc4nc5c(cnn5C)c(=O)[nH]4)C3=O)CC2)cc1	25	1
TNKS1_active_303	CHEMBL151382	Oc1nc(-c2ccc(C(F)(F)F)cc2)nc2c(O)cccc12	25	1
TNKS1_active_304	CHEMBL4747523	Cc1cccc(Cn2c(=O)n(C[C@H]3CC[C@H](C(=O)Nc4ccncc4)CC3)c(=O)c3c(C)cccc32)c1C#N	25.1	1
TNKS1_active_305	CHEMBL4757303	Cc1cccc2c1c(=O)n(C[C@H]1CC[C@H](C(=O)Nc3ccnnc3)CC1)c(=O)n2Cc1cccc(C(F)(F)F)c1C#N	25.1	1
TNKS1_active_306	CHEMBL4758600	Cc1cccc2c1c(=O)n(C[C@H]1CC[C@H](C(=O)Nc3ccncc3)CC1)c(=O)n2Cc1ccccc1C#N	25.1	1
TNKS1_active_307	CHEMBL4555506	O=C1Nc2ccccc2C12CCN(c1nc3c(c(=O)[nH]1)CCCC3)CC2	25.1	1
TNKS1_active_308	CHEMBL4103924	c1ccc(C2COCN2c2ccc(-c3cncc(-c4ncccn4)c3)cc2)cc1	25.12	1
TNKS1_active_309	CHEMBL3099720	CC1(C)OC(=O)N(c2ccc(-c3cnc(N)c(-c4ncccn4)c3)cc2)[C@H]1c1ccccc1	25.95	2
TNKS1_active_310	CHEMBL4545585	O=C1N(CCO)c2ccccc2C12CCN(c1nc3c(c(=O)[nH]1)CCCC3)CC2	26	1
TNKS1_active_311	CHEMBL3909379	COc1ccc(C(=O)C2CCN(C3CCN(Cc4nc(C)c(C)c(=O)[nH]4)C3=O)CC2)cc1	26	1
TNKS1_active_312	CHEMBL2425802	O=C(CCC1CCCCCC1)Nc1nnc2n1CCS2	26	1
TNKS1_active_313	CHEMBL4462707	N#Cc1cccc2c1C1(CCN(c3nc4c(c(=O)[nH]3)CCCC4)CC1)C(=O)N2	26.8	1
TNKS1_active_314	CHEMBL4579514	O=c1[nH]c(N2CCC3(CC2)CN(CCO)c2ccccc23)nc2c1CCCC2	26.9	1
TNKS1_active_315	CHEMBL3946751	COc1ccc(C(=O)C2CCN(CC(=O)NCc3cc4c(c(=O)[nH]3)COCC4)CC2)cc1	27	1
TNKS1_active_316	CHEMBL4439240	CN1CCc2c1nc(N1CCC3(CC1)C(=O)N(CCO)c1cc(F)cc(F)c13)[nH]c2=O	27.6	1
TNKS1_active_317	CHEMBL3344072	CC(C)(C)c1ccc(-c2nc3c(sc4ccccc43)c(=O)[nH]2)cc1	28.6	1
TNKS1_active_318	CHEMBL3925115	CC(C)C1CC2=NNC(=O)c3cc(F)cc4[nH]c(c2c34)C1	29	1
TNKS1_active_319	CHEMBL3903427	O=C(CN1CCC(C(=O)c2ccc(OC3CCOCC3)cc2)CC1)NCc1nc2c(c(=O)[nH]1)COCC2	29	1
TNKS1_active_320	CHEMBL4635489	O=C(N[C@H]1C[C@H](c2nnc(-c3nccs3)n2-c2ccccc2F)C1)c1ccccn1	29	1
TNKS1_active_321	CHEMBL551505	O=C(Nc1cccc2cccnc12)c1ccc(N2C(=O)[C@@H]3[C@H](C2=O)[C@H]2C=C[C@@H]3C2)cc1	29.2	1
TNKS1_active_322	CHEMBL4456595	O=c1[nH]c(N2CCC3(CC2)NCc2ccccc23)nc2c1CCCC2	29.3	1
TNKS1_activ	CHEMBL44	CN1CCCc2c1nc(N1CCC3(CC1)C(=O)N(CC(=O)O)c1cc(F)cc(F)c13)[nH]c2	29.8	1

e_323	66165	=O		
TNKS1_active_324	CHEMBL4558610	CC(C)(O)c1ccc(-c2nc3c(F)cccc3c(=O)[nH]2)cc1	30	1
TNKS1_active_325	CHEMBL4639158	N#Cc1ccc2c(c1)[nH]c(=O)n2[C@H]1C[C@H](c2nnc(-c3ccncn3)n2-c2ccccc2F)C1	30	1
TNKS1_active_326	CHEMBL127396	Cc1cccc2c(O)nc(-c3ccc([N+](=O)[O-])cc3)nc12	30	1
TNKS1_active_327	CHEMBL1956162	O=C(Nc1cccc2cccnc12)c1ccc(N2C(=O)[C@@H]3[C@@H]4CC[C@@H](C4)[C@@H]3C2=O)cc1	31	3
TNKS1_active_328	CHEMBL2419700	O=C(c1ccc(F)cc1)C1CCN(CC(=O)N(Cc2ccccn2)Cc2nc3c(c(=O)[nH]2)COCC3)CC1	31	2
TNKS1_active_329	CHEMBL4444673	CN1CCC(c2ccc(-c3nc4c(F)cc(F)cc4c(=O)[nH]3)cc2)CC1.O=C(O)C(F)(F)F	31	1
TNKS1_active_330	CHEMBL1594868	CC(C)(C)c1ccc(C(=O)Nc2nnc3n2CCS3)cc1	31	1
TNKS1_active_331	CHEMBL4794878	N#Cc1c(Cn2c(=O)n(C[C@H]3CC[C@H](C(=O)Nc4ccncc4)CC3)c(=O)c3ccccc32)cccc1C(F)(F)F	31.6	1
TNKS1_active_332	CHEMBL4754447	N#Cc1c(Cn2c(=O)n(C[C@H]3CC[C@H](C(=O)Nc4ccncc4)CC3)c(=O)c3c(Cl)cccc32)cccc1C(F)(F)F	31.6	1
TNKS1_active_333	CHEMBL3984137	O=C(c1ccccn1)C1CCN(CC(=O)N(Cc2nc3c(c(=O)[nH]2)COCC3)CC2CC2)CC1	32	1
TNKS1_active_334	CHEMBL4112739	C[C@H](C1CC1)N(Cc1nc2c(c(=O)[nH]1)COCC2)C(=O)CN1CCC(C(=O)c2ccc(F)cc2)CC1	32	1
TNKS1_active_335	CHEMBL3914416	O=C(c1ccc(F)cc1)C1CCN(CC(=O)N(Cc2ccc(F)cc2)Cc2nc3c(c(=O)[nH]2)COCC3)CC1	32	1
TNKS1_active_336	CHEMBL2419889	O=c1[nH]c(-c2ccc(C(F)(F)F)cc2)nc2c1SCCC2	32	1
TNKS1_active_337	CHEMBL4470270	CN1CCCc2c1nc(N1CCC3(CC1)C(=O)N(CC(F)(F)F)c1cc(F)cc(F)c13)[nH]c2=O	32.4	1
TNKS1_active_338	CHEMBL1898239	COc1ccc(-c2noc(CSc3nnc(C)n3-c3ccc(OC)cc3)n2)cc1	33	1
TNKS1_active_339	CHEMBL2425811	Cc1cccc(C)c1CCC(=O)Nc1nnc2n1CCS2	33	1
TNKS1_active_340	CHEMBL3098939	Cc1cccc2c(=O)[nH]c(-c3ccc(Cl)cc3)nc12	33	1
TNKS1_active_341	CHEMBL4471663	CN1CCCc2c1nc(N1CCC3(CC1)C(=O)N(C)c1cc(OCCN4CCCCC4)cc(F)c13)[nH]c2=O	33.3	1
TNKS1_active_342	CHEMBL4638887	Cn1c(=O)n([C@H]2C[C@H](c3nnc(-c4ccncn4)n3-c3ccccc3Cl)C2)c2ccc(C#N)cc21	34	1
TNKS1_active_343	CHEMBL5275231	CC(C)(C)c1ccc(-c2cn3cncc3c(=O)[nH]2)cc1	35	1
TNKS1_active_344	CHEMBL2419703	CCN(Cc1nc2c(c(=O)[nH]1)COCC2)C(=O)CN1CCC(C(=O)c2ccc(F)cc2)CC1	35	1
TNKS1_active_345	CHEMBL4551975	O=c1[nH]c(N2CCC3(CC2)OCc2ccccc23)nc2c1CCCC2	35.7	1
TNKS1_active_346	CHEMBL5284505	Cc1cc2c(=O)[nH]c(-c3ccc(C(C)(C)O)cc3)cn2n1	36	1
TNKS1_active_347	CHEMBL3931845	COc1ccc(C(=O)C2CCN(C3CCN(Cc4ncc(C)c(=O)[nH]4)C3=O)CC2)cc1	36	1
TNKS1_active_348	CHEMBL4285233	COc1cc2ncnc(N3CCC(CN4Cc5ccccc5NC4=O)CC3)c2cc1OC	36	2
TNKS1_active_349	CHEMBL3966085	O=C(CN1CCC(C(=O)c2ccc(F)cc2)CC1)NCc1nc2c(c(=O)[nH]1)CCCC2	36	1
TNKS1_active_350	CHEMBL1257010	Cc1ccnc2c(NC(=O)c3ccc(N4C(=O)[C@@H]5[C@H](C4=O)[C@@H]4C=C[C@H]5C4)cc3)cccc12	36	1
TNKS1_active_351	CHEMBL4461036	COc1ccc(N2CCN(c3nc4c(c(=O)[nH]3)CC(F)(F)CC4)CC2)cc1	36.4	1
TNKS1_active_352	CHEMBL3752492	COc1ccc(C(=O)Nc2ccc(C(=O)N(c3ccncn3)c3ccccc3Cl)cc2)cc1	37.075	2

TNKS1_active_353	CHEMBL3976109	O=C(c1cccc(OC(F)(F)F)c1)C1CCN(CC(=O)N(Cc2nc3c(c(=O)[nH]2)COCC3)CC(F)F)CC1	38	1
TNKS1_active_354	CHEMBL4457148	Cl.O=c1[nH]c(-c2ccc(C3CCNCC3)cc2)nc2c(F)cc(F)cc12	38	1
TNKS1_active_355	CHEMBL4110045	O=C(c1ccc(F)cc1)C1CCN(CC(=O)N(Cc2nc3c(c(=O)[nH]2)COCC3)C[C@H]2CCCO2)CC1	38	1
TNKS1_active_356	CHEMBL3902386	O=C(CN1CCC(n2c(=O)[nH]c3ccccc32)CC1)NCc1nc2c(c(=O)[nH]1)COCC2	39	1
TNKS1_active_357	CHEMBL2381636	Cc1cc(=O)[nH]c2cc(-c3ccc(N)cc3Cl)ccc12	39	1
TNKS1_active_358	CHEMBL4566814	CC(C)(O)c1ccc(-c2nc3ccc(Cl)cc3c(=O)[nH]2)cc1	39	1
TNKS1_active_359	CHEMBL2425803	O=C(CCc1ccccc1)Nc1nnc2n1CCS2	39	1
TNKS1_active_360	CHEMBL4449082	C[C@@H]1CN(c2cc(F)c3c(c2)N(C)C(=O)C32CCN(c3nc4c(c(=O)[nH]3)CCCN4C)CC2)C[C@H](C)O1	39.1	2
TNKS1_active_361	CHEMBL3987175	CC(c1ccc(F)cc1F)N(Cc1nc2c(c(=O)[nH]1)COCC2)C(=O)CN1CCC(C(=O)c2ccc(F)cc2)CC1	39.5	1
TNKS1_active_362	CHEMBL4076368	COc1cccc(N2CCN(c3nc4c(c(=O)[nH]3)CCCC4)CC2)c1	39.7	1
TNKS1_active_363	CHEMBL4759993	Cc1cccc2c1c(=O)n(C[C@H]1CC[C@H](C(=O)Nc3cnn(C)c3)CC1)c(=O)n2Cc1cccc(C(F)(F)F)c1C#N	39.8	1
TNKS1_active_364	CHEMBL4763370	Cc1ccc(NC(=O)[C@H]2CC[C@H](Cn3c(=O)c4c(C)cccc4n(Cc4cccc(C(F)(F)F)c4C#N)c3=O)CC2)cn1	39.8	1
TNKS1_active_365	CHEMBL4791639	N#Cc1ccccc1Cn1c(=O)n(C[C@H]2CC[C@H](C(=O)Nc3ccncc3)CC2)c(=O)c2ccccc21	39.8	1
TNKS1_active_366	CHEMBL3098938	Cc1cccc2c(=O)[nH]c(-c3ccc(F)cc3)nc12	40	1
TNKS1_active_367	CHEMBL4454894	CC(C)(O)c1ccc(-c2nc3c(F)ccc(F)c3c(=O)[nH]2)cc1	40	1
TNKS1_active_368	CHEMBL124749	Cc1cccc2c(O)nc(-c3ccccc3)nc12	41	1
TNKS1_active_369	CHEMBL2419899	CN(C)c1ccc(-c2nc3c(c(=O)[nH]2)COCC3)cc1	41	1
TNKS1_active_370	CHEMBL1867804	COc1ccc(-n2c(C)nnc2SCc2nc(-c3ccc(OC)c(OC)c3)no2)cc1	42	1
TNKS1_active_371	CHEMBL3935482	O=C(c1ccc(F)cc1)C1CCN(CC(=O)N(Cc2cccnc2)Cc2nc3c(c(=O)[nH]2)COCC3)CC1	42	1
TNKS1_active_372	CHEMBL2325500	CS(=O)(=O)c1ccc(-c2nnc(SCc3nnc(-c4ccc(Cl)cc4)o3)n2-c2ccccc2Cl)nc1	43	1
TNKS1_active_373	CHEMBL3939375	O=c1[nH]nc2c3c(cccc13)NC1=C2CCN(Cc2ccccc2)C1	43	1
TNKS1_active_374	CHEMBL3968547	O=C(CN1CCC(C(=O)c2ccc3c(c2)OCO3)CC1)NCc1nc2c(c(=O)[nH]1)COCC2	43	1
TNKS1_active_375	CHEMBL4524437	COc1cccc2c(=O)[nH]c(-c3ccc(C(C)(C)O)cc3)nc12	43	1
TNKS1_active_376	CHEMBL3978546	O=C(CN1CCC(c2noc3cc(F)ccc23)CC1)NCc1nc2c(c(=O)[nH]1)COCC2	43	1
TNKS1_active_377	CHEMBL3098935	Cc1cccc2c(=O)[nH]c(-c3ccc(CNC(=O)OCc4ccccc4)cc3)nc12	44	1
TNKS1_active_378	CHEMBL3827272	Cc1ccc(-c2nc3c(O)cccc3c(=O)[nH]2)cc1	44	1
TNKS1_active_379	CHEMBL435073	Nc1ncnc2c1ncn2C1OC(C(=O)N2CCN(CC(=O)Nc3cccc4c3CNC4=O)CC2)[C@@H](O)[C@H]1O	44.835	2
TNKS1_active_380	CHEMBL3828579	O=C(NCc1ccc(-c2nc3ccccc3c(=O)[nH]2)cc1)OCc1ccccc1	45	1
TNKS1_active_381	CHEMBL4442353	COc1ccc(N2CCN(c3nc4c(c(=O)[nH]3)CCCC4)CC2)cc1	45.5	1
TNKS1_activ	CHEMBL46	N#Cc1ccc2c(c1)oc(=O)n2[C@H]1C[C@H](c2nnc(-c3ccncn3)n2-	48	1

e_382	34877	c2ccccc2Cl)C1		
TNKS1_active_383	CHEMBL3913507	CC1(C)OC(=O)N(c2ccc(-c3cnc(N)c(-c4ncc(F)cn4)c3)cc2)[C@H]1c1ccccc1	48.3	1
TNKS1_active_384	CHEMBL3099681	O=c1[nH]c(-c2ccc(Cl)cc2)nc2c(O)cccc12	49	1
TNKS1_active_385	CHEMBL4467730	CN1CCCc2c1nc(N1CCC3(CC1)C(=O)N(C)c1cc(N4CCCCC4)cc(F)c13)[nH]c2=O	49.1	1
TNKS1_active_386	CHEMBL3912260	CC1(C)OC(=O)N(c2ccc(-c3cnc(N)c(-c4ccc5nncn5n4)c3)cc2)[C@H]1c1ccccc1	49.3	1
TNKS1_active_387	CHEMBL4471982	COc1ccccc1C1=CCN(c2nc3c(c(=O)[nH]2)CCCC3)CC1	49.7	1
TNKS1_active_388	CHEMBL3644587	O=c1[nH]nc2c3c(cccc13)N=C(CN1Cc3ccccc3C1)N2	50	3
TNKS1_active_389	CHEMBL4457607	CC(C)(O)c1ccc(-c2nc3cccc(Cl)c3c(=O)[nH]2)cc1	50	1
TNKS1_active_390	CHEMBL3919581	C[C@@H](c1ccccc1)N(Cc1nc2c(c(=O)[nH]1)COCC2)C(=O)CN1CCC(C(=O)c2ccc(F)cc2)CC1	50	1
TNKS1_active_391	CHEMBL4788947	Cc1c(F)ccc2c1c(=O)n(C[C@H]1CC[C@H](C(=O)Nc3ccncc3)CC1)c(=O)n2Cc1cccc(C(F)(F)F)c1C#N	50.1	1
TNKS1_active_392	CHEMBL4798387	COc1cccc2c1c(=O)n(C[C@H]1CC[C@H](C(=O)Nc3ccncc3)CC1)c(=O)n2Cc1cccc(C(F)(F)F)c1C#N	50.1	1
TNKS1_active_393	CHEMBL4759701	Cc1cccc2c1c(=O)n(C[C@H]1CC[C@H](C(=O)Nc3ccncc3)CC1)c(=O)n2Cc1cccc(C(F)(F)F)c1C#N	50.1	1
TNKS1_active_394	CHEMBL2325497	N#Cc1ccc(-c2nnc(CSc3nnc(-c4ccncc4)n3-c3ccccc3Cl)o2)cc1	51	1
TNKS1_active_395	CHEMBL336718	COc1ccc(-c2nc(O)c3ccccc3n2)cc1	52	3
TNKS1_active_396	CHEMBL2381633	Cc1cc(=O)[nH]c2cc(-c3ccccc3F)ccc12	52	1
TNKS1_active_397	CHEMBL3900374	CCc1cc(=O)[nH]c(CN2CCC(N3CCC(C(=O)c4ccc(OC)cc4)CC3)C2=O)n1	53	1
TNKS1_active_398	CHEMBL2325502	COc1cc(-c2nnc(/C=C/c3nnc(-c4ccc(S(C)(=O)=O)cn4)n3-c3ccccc3Cl)o2)ccn1	54	1
TNKS1_active_399	CHEMBL2419707	O=c1[nH]c(-c2ccc(OC(F)(F)F)cc2)nc2c1COCC2	55	1
TNKS1_active_400	CHEMBL4293376	O=C(Nc1cccc2cccnc12)c1ccc(C2C(=O)[C@@H]3[C@H](C2=O)[C@@H]2C=C[C@H]3C2)cc1	56	1
TNKS1_active_401	CHEMBL562310	O=C(Nc1cccc2cccnc12)c1ccc(N2C(=O)[C@@H]3[C@H](C2=O)[C@@H]2C=C[C@H]3C2)cc1	56	7
TNKS1_active_402	CHEMBL2419704	CN(Cc1nc2c(c(=O)[nH]1)COCC2)C(=O)CN1CCC(C(=O)c2ccc(F)cc2)CC1	57	2
TNKS1_active_403	CHEMBL1609488	Oc1nc(N2CCN(c3ccccc3)CC2)nc2c1CCCC2	58.9	1
TNKS1_active_404	CHEMBL3734971	CC(C)(CO)C1Cc2[nH]c(=O)c3ccccc3c2CO1	60	1
TNKS1_active_405	CHEMBL3735517	CC(C)(C)C1Cc2[nH]c(=O)c3ccccc3c2CO1	60	1
TNKS1_active_406	CHEMBL3734991	Cc1cccc2c(=O)[nH]c3c(c12)COC(C(C)C)C3	60	1
TNKS1_active_407	CHEMBL3736012	Cc1cccc2c(=O)[nH]c3c(c12)COC(C(C)(C)CO)C3	60	2
TNKS1_active_408	CHEMBL3736145	CS(=O)(=O)N1CCC(C2Cc3[nH]c(=O)c4ccccc4c3CO2)C1	60	1
TNKS1_active_409	CHEMBL3736494	CC(C)C1Cc2[nH]c(=O)c3ccccc3c2CO1	60	1
TNKS1_active_410	CHEMBL3735964	O=C(C1COC1)N1CCC(C2Cc3[nH]c(=O)c4ccccc4c3CO2)CC1	60	1
TNKS1_active_411	CHEMBL2419896	Cc1ccc(-c2nc3c(c(=O)[nH]2)COCC3)cc1	62	1

TNKS1_active_412	CHEMBL3098936	COc1cccc2c(=O)[nH]c(-c3ccc(Cl)cc3)nc12	62	1
TNKS1_active_413	CHEMBL2419709	CS(=O)(=O)c1ccc(-c2nc3c(c(=O)[nH]2)COCC3)cc1	63	1
TNKS1_active_414	CHEMBL2016633	O=C(Nc1cccc2cccnc12)c1ccc(N2C(=O)[C@@H]3[C@H](C2=O)[C@H]2C=C[C@H]3C2)cc1	63	1
TNKS1_active_415	CHEMBL4761298	Cc1cccc2c1c(=O)n(C[C@H]1CC[C@H](C(=O)O)CC1)c(=O)n2Cc1cccc(C(F)(F)F)c1C#N	63.1	1
TNKS1_active_416	CHEMBL4799048	N#Cc1c(Cn2c(=O)n(C[C@H]3CC[C@H](C(=O)Nc4ccncc4)CC3)c(=O)c3c(F)cccc32)cccc1C(F)(F)F	63.1	1
TNKS1_active_417	CHEMBL2419718	O=C(CCc1ccccc1F)N(Cc1nc2c(c(=O)[nH]1)COCC2)Cc1cccs1	64	2
TNKS1_active_418	CHEMBL4475497	COc1ccccc1N1CCN(c2nc3c(c(=O)[nH]2)CC(F)(F)CC3)CC1	65.5	1
TNKS1_active_419	CHEMBL3946953	CC1(C)OC(=O)N(c2ccc(-c3cc(-c4ncccn4)c[nH]c3=O)cc2)[C@H]1c1ccccc1	65.9	1
TNKS1_active_420	CHEMBL3909888	O=C(c1ccc(F)cc1)C1CCN(CC(=O)N(Cc2nc3c(c(=O)[nH]2)COCC3)Cc2ccc(S(=O)(=O)N3CCOCC3)s2)CC1	66	1
TNKS1_active_421	CHEMBL4444671	COc1ccccc1C1CCN(c2nc3c(c(=O)[nH]2)CCCC3)CC1	67.6	1
TNKS1_active_422	CHEMBL3959094	C[C@@H](c1ccc(F)cc1)N(Cc1nc2c(c(=O)[nH]1)COCC2)C(=O)CN1CCC(C(=O)c2ccc(F)cc2)CC1	68	1
TNKS1_active_423	CHEMBL2425797	O=C(Nc1nnc2n1CCS2)C1CCCCC1	70	1
TNKS1_active_424	CHEMBL3593715	O=c1[nH]c(N2CCN(Cc3ccncc3)CC2)nc2c1CCCC2	70.895	2
TNKS1_active_425	CHEMBL3099815	Br.Cc1cccc2c(=O)[nH]c(-c3ccc(CN)cc3)nc12	71.5	2
TNKS1_active_426	CHEMBL2431867	CC(C)c1ccc(-c2cc(=O)c3ccccc3o2)cc1	72.22	2
TNKS1_active_427	CHEMBL4555632	CN1CCc2c1nc(N1CCC3(CC1)CN(CCO)c1cc(F)cc(F)c13)[nH]c2=O	73.6	1
TNKS1_active_428	CHEMBL4776820	N#Cc1ccc(N2CCN([C@H]3C=C(c4cc5ncccc5c(=O)[nH]4)CC3)CC2)cc1	74	1
TNKS1_active_429	CHEMBL2419893	O=c1[nH]c(-c2ccc(C(F)(F)F)cc2)nc2c1COCC2	75	1
TNKS1_active_430	CHEMBL2419892	O=c1[nH]c(-c2ccc(C(F)(F)F)cc2)nc2ccccc12	77.25	2
TNKS1_active_431	CHEMBL3941613	O=C(CN1CCC(c2nnc(-c3ccc(F)cc3)o2)CC1)NCc1nc2c(c(=O)[nH]1)COCC2	78	1
TNKS1_active_432	CHEMBL2419692	O=C(CCc1ccc(F)cc1)N(Cc1nc2c(c(=O)[nH]1)COCC2)Cc1cccs1	78	2
TNKS1_active_433	CHEMBL3109648	Cc1c(F)ccc2c1c(=O)n(C[C@H]1CC[C@H](C(=O)O)CC1)c(=O)n2Cc1cccc(C(F)(F)F)c1C#N	79.4	3
TNKS1_active_434	CHEMBL4761086	Cc1ccc2c(c1C)c(=O)n(C[C@H]1CC[C@H](C(=O)O)CC1)c(=O)n2Cc1cccc(C(F)(F)F)c1C#N	79.4	1
TNKS1_active_435	CHEMBL3109647	N#Cc1c(Cn2c(=O)n(C[C@H]3CC[C@H](C(=O)O)CC3)c(=O)c3ccccc32)cccc1C(F)(F)F	79.4	3
TNKS1_active_436	CHEMBL3344071	Cc1ccc(-c2nc3c(sc4ccccc43)c(=O)[nH]2)cc1	79.8	1
TNKS1_active_437	CHEMBL3907218	CC(C)C1CC2=NNC(=O)c3cccc4[nH]c(c2c34)C1	80	1
TNKS1_active_438	CHEMBL3906478	[C-]#[N+]c1c(Cl)cccc1CN1CCC(N2CCC(C(=O)c3ccc(OC)cc3)CC2)C1=O	81	1
TNKS1_active_439	CHEMBL2419898	O=c1[nH]c(-c2ccc(Br)cc2)nc2c1COCC2	81	1
TNKS1_active_440	CHEMBL3926736	COc1ccc(C(=O)C2CCN(C3CCN(Cc4nc5ccsc5c(=O)[nH]4)C3=O)CC2)cc1	83	1
TNKS1_activ	CHEMBL39	COc1ccccc1C(=O)C1CCN(CC(=O)N(Cc2nc3c(c(=O)[nH]2)COCC3)CC(F)F	85	1

e_441	00771	)CC1		
TNKS1_active_442	CHEMBL2325503	CS(=O)(=O)c1ccc(-c2nnc(/C=C/c3nnc(-c4ccc(C#N)cc4)o3)n2-c2ccccc2Cl)nc1	85.5	3
TNKS1_active_443	CHEMBL2419900	COc1ccc(-c2nc3c(c(=O)[nH]2)COCC3)cc1	86	1
TNKS1_active_444	CHEMBL127335	Oc1ccc(-c2nc(O)c3cccc(O)c3n2)cc1	86	1
TNKS1_active_445	CHEMBL123798	COc1cccc2c(O)nc(-c3ccc(C(F)(F)F)cc3)nc12	87	1
TNKS1_active_446	CHEMBL2425816	O=C(CCc1ccco1)Nc1nnc2n1CCS2	88	1
TNKS1_active_447	CHEMBL3935158	COc1ccc(-c2nnc(C3CCN(CC(=O)NCc4nc5c(c(=O)[nH]4)COCC5)CC3)o2)cc1	89	1
TNKS1_active_448	CHEMBL2425804	O=C(CCCC1CCCCC1)Nc1nnc2n1CCS2	89	1
TNKS1_active_449	CHEMBL3736005	CC(C)C1Cc2[nH]c(=O)c3cccnc3c2CO1	93	1
TNKS1_active_450	CHEMBL5087490	O=C(N[C@H]1C[C@H](c2nnc(-c3cnccn3)n2-c2ccccc2F)C1)c1ccccn1	93	1
TNKS1_active_451	CHEMBL4786460	Cc1ccc2c(c1C)c(=O)n(C[C@H]1CC[C@H](C(=O)Nc3ccncc3)CC1)c(=O)n2Cc1cccc(C(F)(F)F)c1C#N	100	1
TNKS1_active_452	CHEMBL3109649	Cc1cccc2c1c(=O)n(C[C@H]1CC[C@@H](C(=O)O)CC1)c(=O)n2Cc1cccc(C(F)(F)F)c1C#N	100	1
TNKS1_active_453	CHEMBL2058057	O=c1[nH]c(-c2ccc(Br)cc2)nc2ccccc12	101	1
TNKS1_active_454	CHEMBL3929705	O=C1NN=C2CCCc3[nH]c4cc(F)cc1c4c32	102	1
TNKS1_active_455	CHEMBL4632484	O=C1CC2(CCN(C(=O)CN3CCN(CCSc4nc5ccccc5c(=O)[nH]4)CC3)CC2)Oc2ccccc21	103	1
TNKS1_active_456	CHEMBL1707591	CC(C)CC(=O)Nc1nnc2n1CCS2	103	1
TNKS1_active_457	CHEMBL3827963	COc1cccc2c(=O)[nH]c(-c3ccc(Br)cc3)nc12	106	1
TNKS1_active_458	CHEMBL2419711	O=C(CCc1ccccc1)N(Cc1nc2ccccc2c(=O)[nH]1)Cc1cccs1	106	1
TNKS1_active_459	CHEMBL3764816	Cn1ncnc1[C@@H]1c2n[nH]c(=O)c3cc(F)cc(c23)N[C@H]1c1ccc(F)cc1	107.575	2
TNKS1_active_460	CHEMBL3932754	COc1ccc(C(=O)C2CCN(CC(=O)NCc3nc4c(c(=O)[nH]3)CCC4)CC2)cc1	110	1
TNKS1_active_461	CHEMBL5172909	Cc1ccccc1NC(=O)c1ccc(NC(=O)N2CCN(C(=O)c3cc(Cc4n[nH]c(=O)c5ccccc45)ccc3F)CC2)cc1	110.5	1
TNKS1_active_462	CHEMBL4112659	COc1ccc(C(=O)C2CCN([C@@H]3CCN(Cc4nc5ccccc(=O)c5[nH]4)C3=O)CC2)cc1C	112	1
TNKS1_active_463	CHEMBL4466835	COc1ccccc1N1CCN(c2nc3c(c(=O)[nH]2)COCC3)CC1	112	1
TNKS1_active_464	CHEMBL3978225	O=c1[nH]nc2c3c(cccc13)NC1=C2CCNC1	120	1
TNKS1_active_465	CHEMBL4749857	COc1cccc2c1c(=O)n(C[C@H]1CC[C@H](C(=O)O)CC1)c(=O)n2Cc1cccc(C(F)(F)F)c1C#N	125.9	1
TNKS1_active_466	CHEMBL4781804	N#Cc1c(Cn2c(=O)n(C[C@H]3CC[C@H](C(=O)O)CC3)c(=O)c3c(Cl)cccc32)cccc1C(F)(F)F	125.9	1
TNKS1_active_467	CHEMBL2419890	O=C1NC(c2ccc(C(F)(F)F)cc2)=NC2CSCC12	127	1
TNKS1_active_468	CHEMBL1163173	O=c1[nH]c(-c2ccc(Cl)cc2)nc2ccccc12	127	2
TNKS1_active_469	CHEMBL340481	COc1ccc(-c2nc(O)c3cccc(OC)c3n2)cc1	128	1
TNKS1_active_470	CHEMBL5077731	O=c1[nH]c2sccc2c2cc(O)ccc12	128.82	1

TNKS1_active_471	CHEMBL3969049	O=C1NN=C2CCCc3c2c2c1cccc2n3CCN1CCCCC1	130	1
TNKS1_active_472	CHEMBL1724183	COc1ccc(-c2noc(CSc3nnc(C)n3-c3cccc(C)c3)n2)cc1	132	1
TNKS1_active_473	CHEMBL3981864	COc1ccc(C(=O)C2CCN(CC(=O)NCc3cc4c(c(=O)o3)COCC4)CC2)cc1	133	1
TNKS1_active_474	CHEMBL2425815	O=C(CCc1ccncc1)Nc1nnc2n1CCS2	134	1
TNKS1_active_475	CHEMBL3589284	CN1CCCc2c1cc(-c1ccc(Cl)cc1)[nH]c2=O	134	1
TNKS1_active_476	CHEMBL2419895	O=c1[nH]c(-c2ccsc2)nc2c1COCC2	136	1
TNKS1_active_477	CHEMBL3902403	COc1ccc(C(=O)C2CCN(C3CCN(Cc4nc(C)cc(=O)[nH]4)C3=O)CC2)cc1	137	1
TNKS1_active_478	CHEMBL2425814	O=C(CCc1cccnc1)Nc1nnc2n1CCS2	138	1
TNKS1_active_479	CHEMBL5084343	COc1ccc2c(=O)[nH]c3sccc3c2c1	138.04	1
TNKS1_active_480	CHEMBL3891329	O=C1NN=C2CCCc3[nH]c4cccc1c4c32	140	1
TNKS1_active_481	CHEMBL64780	O=c1cc(-c2ccc([N+](=O)[O-])cc2)oc2ccccc12	140.625	2
TNKS1_active_482	CHEMBL3967989	COc1ccc(C(=O)C2CCN(C3CCN(Cc4nc5sc(C)cc5c(=O)[nH]4)C3=O)CC2)cc1	141	1
TNKS1_active_483	CHEMBL3973611	COCCN(Cc1nc2c(c(=O)[nH]1)COCC2)C(=O)CN1CCC(c2nnc(-c3ccccc3)o2)CC1	142	1
TNKS1_active_484	CHEMBL3915929	CC1CC2=NNC(=O)c3cccc4[nH]c(c2c34)C1	143	1
TNKS1_active_485	CHEMBL275638	O=c1cc(-c2ccccc2)oc2ccccc12	143.955	2
TNKS1_active_486	CHEMBL2419897	O=c1[nH]c(-c2ccc(Cl)cc2)nc2c1COCC2	148	1
TNKS1_active_487	CHEMBL4446309	COc1ccc(N2CCN(c3nc4c(c(=O)[nH]3)COCC4)CC2)cc1	148	1
TNKS1_active_488	CHEMBL2381632	Cc1cc(=O)[nH]c2cc(-c3cccc(F)c3)ccc12	150	1
TNKS1_active_489	CHEMBL3898162	O=C1NN=C2CCCc3c2c2c1cccc2n3CCN1CCCC1	150	1
TNKS1_active_490	CHEMBL3594137	CC(C)(C)c1ccc(C2NC(=O)c3ccccc3N2)cc1	152.44	2
TNKS1_active_491	CHEMBL3969742	O=C1NN=C2CCCCc3[nH]c4cccc1c4c32	154	1
TNKS1_active_492	CHEMBL3740104	N#Cc1cccnc1N1CCN(C(=O)c2cccc(Cc3n[nH]c(=O)c4ccccc34)c2)CC1	160	1
TNKS1_active_493	CHEMBL2419891	O=c1[nH]c(-c2ccc(C(F)(F)F)cc2)nc2c1CCCC2	163	1
TNKS1_active_494	CHEMBL3892363	O=C1NN=C2CC(c3ccccc3)Cc3[nH]c4cccc1c4c32	164	1
TNKS1_active_495	CHEMBL3827329	COc1cccc2c(=O)[nH]c(-c3ccc(C)cc3)nc12	167	1
TNKS1_active_496	CHEMBL3949450	O=C(CN1CCC(c2nnc(-c3ccccc3)o2)CC1)NCc1nc2c(c(=O)[nH]1)COCC2	170	1
TNKS1_active_497	CHEMBL5189723	O=C(Nc1ccc(F)cc1)c1ccc(NC(=O)N2CCN(C(=O)c3cc(Cc4n[nH]c(=O)c5ccccc45)ccc3F)CC2)cc1	171	1
TNKS1_active_498	CHEMBL222556	O=c1cc(-c2ccc(O)c(O)c2)oc2ccccc12	173.915	2
TNKS1_active_499	CHEMBL1611739	COc1ccc(-c2noc(CSc3nnc(-c4ccncc4)n3-c3ccc(OC)cc3)n2)cc1	175	1
TNKS1_activ	CHEMBL39	O=c1[nH]c(CN(Cc2cccs2)S(=O)(=O)Cc2ccccc2)nc2c1COCC2	177	1

e_500	43146			
TNKS1_active_501	CHEMBL2419694	O=C(CCc1cccc(O)c1)N(Cc1nc2c(c(=O)[nH]1)COCC2)Cc1cccs1	179	2
TNKS1_active_502	CHEMBL341142	Oc1nc(-c2ccccc2)nc2c(O)cccc12	180	1
TNKS1_active_503	CHEMBL2381630	Cc1cc(=O)[nH]c2cc(-c3ccccc3)ccc12	180	1
TNKS1_active_504	CHEMBL3344061	O=c1[nH]c2ccccc2c2c1sc1ccccc12	180	1
TNKS1_active_505	CHEMBL4110631	CC1(C)OC(=O)N([C@H]2CC[C@H](NC(=O)c3cccc4cccnc34)CC2)[C@@H]1c1ccccc1	185	1
TNKS1_active_506	CHEMBL3949925	O=C(CN1CCC(n2cc(-c3ccccc3)cn2)CC1)NCc1nc2c(c(=O)[nH]1)COCC2	188	1
TNKS1_active_507	CHEMBL2381958	CC1(C)OC(=O)N(c2ccc(C(=O)Nc3cccc4cccnc34)cc2)[C@H]1c1ccccc1	189	1
TNKS1_active_508	CHEMBL3589281	CN1CCCc2c1cc(-c1ccccc1)[nH]c2=O	189	1
TNKS1_active_509	CHEMBL3289108	O=c1[nH]c(-c2ccc(O)cc2)nc2ccccc12	189	1
TNKS1_active_510	CHEMBL338088	O=[N+]([O-])c1ccc(-c2nc(O)c3cccc(O)c3n2)cc1	191	1
TNKS1_active_511	CHEMBL4460557	O=c1[nH]c(SCCN2CCN(c3ccccn3)CC2)nc2ccccc12	193	1
TNKS1_active_512	CHEMBL2425789	Cc1n[nH]c(C)c1CCC(=O)Nc1nnc2n1CCS2	195	1
TNKS1_active_513	CHEMBL1364326	COc1ccc(-c2noc(CSc3nnc(C)n3-c3ccc(F)cc3)n2)cc1	197	1
TNKS1_active_514	CHEMBL4474593	NC(=O)c1cccc2c1O/C(=C\c1ccc(C(=O)N3CCN(c4nc5ccccc5[nH]4)CC3)cc1)C2=O	198	1
TNKS1_active_515	CHEMBL4090855	c1ccc(C2=NC3=C(CN2)COCC3)cc1	199.53	1
TNKS1_active_516	CHEMBL4074540	O=c1[nH]c2ccccc2n1[C@H]1C[C@H](c2nnc(-c3ccncn3)n2-c2ccccc2Cl)C1	200	3
TNKS1_active_517	CHEMBL3238252	Cc1cc(NCCc2ccc(O)cc2)c2nncn2n1	200	2
TNKS1_active_518	CHEMBL2419708	COC(=O)c1ccc(-c2nc3c(c(=O)[nH]2)COCC3)cc1	202	1
TNKS1_active_519	CHEMBL2425799	O=C(CC1CCOCC1)Nc1nnc2n1CCS2	202	1
TNKS1_active_520	CHEMBL3911687	CC1(C)CC2=NNC(=O)c3cc(F)cc4[nH]c(c2c34)C1	203	1
TNKS1_active_521	CHEMBL4113362	COc1ccc(C(=O)C2CCN([C@@H]3CCN(Cc4nc5c(c(=O)[nH]4)COCC5)C3=O)CC2)cc1C	206	1
TNKS1_active_522	CHEMBL3924532	O=C(c1ccc(F)cc1)C1CCN(CC(=O)N(Cc2ccncc2)Cc2nc3c(c(=O)[nH]2)COCC3)CC1	206	1
TNKS1_active_523	CHEMBL3589285	CN1CCCc2c1cc(-c1ccc(Br)cc1)[nH]c2=O	214.55	2
TNKS1_active_524	CHEMBL3920028	COc1ccc(C(=O)C2CCN([C@H]3CCCN(CCC4NC(=O)C5=C(CCOC5)N4)C3=O)CC2)cc1	218	1
TNKS1_active_525	CHEMBL2419712	O=C(CCc1ccccc1)N(Cc1nc2c(c(=O)[nH]1)COCC2)Cc1cccs1	219	2
TNKS1_active_526	CHEMBL126167	Nc1ccc(-c2nc(O)c3cccc(O)c3n2)cc1	219	1
TNKS1_active_527	CHEMBL372303	CN(C)CC(=O)Nc1ccc2[nH]c(=O)c3ccccc3c2c1	219	5
TNKS1_active_528	CHEMBL2425796	O=C(Nc1nnc2n1CCS2)C1CCCC1	220	1
TNKS1_active_529	CHEMBL5081642	O=C(N[C@H]1C[C@H](c2nnc(-c3ccccc3)n2-c2ccccc2F)C1)c1ccccn1	220	1

TNKS1_active_530	CHEMBL5395820	C[C@@H](CN1CC(C(=O)N2CCN(c3ncc(C(F)(F)F)cn3)CC2)C1)Nc1cn[nH]c(=O)c1C(F)(F)F	227	1
TNKS1_active_531	CHEMBL3594136	CC(C)c1ccc(C2NC(=O)c3ccccc3N2)cc1	232.21	2
TNKS1_active_532	CHEMBL3934980	O=C(CCc1ccccc1F)N(Cc1ccc[nH]1)Cc1nc2c(c(=O)[nH]1)COCC2	235	1
TNKS1_active_533	CHEMBL1446237	O=C(Cc1ccccc1)Nc1nnc2n1CCS2	239	1
TNKS1_active_534	CHEMBL3930358	O=C(CN1CCC(C(=O)N2CCc3c(Cl)cccc3C2)CC1)NCc1nc2c(c(=O)[nH]1)COCC2	244	1
TNKS1_active_535	CHEMBL1714359	O=C(Nc1nnc2n1CCS2)c1ccccc1	250	1
TNKS1_active_536	CHEMBL4762657	Cc1c(C(N)=O)ccc2c1c(=O)n(C[C@H]1CC[C@H](C(=O)Nc3ccncc3)CC1)c(=O)n2Cc1cccc(C(F)(F)F)c1C#N	251.2	1
TNKS1_active_537	CHEMBL4516906	O=C1NC2(CCN(c3nc4c(c(=O)[nH]3)CCCC4)CC2)c2ccccc21	258	1
TNKS1_active_538	CHEMBL3753510	COc1ccc(C(=O)Nc2ccc(C(=O)NCC3(c4ccc(OC)cc4)CCOCC3)c(Cl)c2)cc1	258.52	2
TNKS1_active_539	CHEMBL1164165	O=c1[nH]c(-c2ccc([N+](=O)[O-])cc2)nc2ccccc12	259	1
TNKS1_active_540	CHEMBL3828676	O=c1[nH]c(-c2ccc(F)cc2)nc2c(O)cccc12	263	1
TNKS1_active_541	CHEMBL2425795	O=C(Nc1nnc2n1CCS2)C1CCC1	264	1
TNKS1_active_542	CHEMBL3952800	O=C(CN1CCC(c2n[nH]c3cc(F)ccc23)CC1)NCc1nc2c(c(=O)[nH]1)COCC2	270	1
TNKS1_active_543	CHEMBL2016634	COc1ccc(-n2c(SCc3nc(-c4ccccc4)no3)nnc2-c2ccncc2)cc1	272	1
TNKS1_active_544	CHEMBL1599148	Cc1nnc(SCc2nc(-c3ccc(Cl)cc3)no2)n1-c1ccc(F)cc1	281	1
TNKS1_active_545	CHEMBL3344065	O=c1[nH]c(-c2ccccc2)cc2c1sc1ccccc12	283	1
TNKS1_active_546	CHEMBL2419691	O=C(CCc1cccc(F)c1)N(Cc1nc2c(c(=O)[nH]1)COCC2)Cc1cccs1	284	2
TNKS1_active_547	CHEMBL4554200	CC(C)n1cc2c(n1)CN(C(=O)c1cc(Cc3n[nH]c(=O)c4ccccc34)ccc1F)C2	289.8	1
TNKS1_active_548	CHEMBL2325499	Cc1ccc(-c2nnc(CCc3nnc(-c4ccncc4)n3-c3ccccc3Cl)o2)cc1	290	1
TNKS1_active_549	CHEMBL5179949	O=C(Nc1ccccc1F)c1ccc(NC(=O)N2CCN(C(=O)c3cc(Cc4n[nH]c(=O)c5ccccc45)ccc3F)CC2)cc1	296	1
TNKS1_active_550	CHEMBL3593716	CN1CCN(c2nc3c(c(=O)[nH]2)CCCC3)CC1	301	2
TNKS1_active_551	CHEMBL2425805	O=C(CCCc1ccccc1)Nc1nnc2n1CCS2	303	1
TNKS1_active_552	CHEMBL2381631	Cc1cc(=O)[nH]c2cc(-c3ccc(F)cc3)ccc12	310	1
TNKS1_active_553	CHEMBL3928549	COc1ccc2c(C3CCN(CC(=O)NCc4nc5c(c(=O)[nH]4)COCC5)CC3)n[nH]c2c1	311	1
TNKS1_active_554	CHEMBL147552	O=c1cc(-c2cccc(O)c2)oc2ccccc12	313.115	2
TNKS1_active_555	CHEMBL4780670	N#Cc1ccccc1Cn1c(=O)n(C[C@H]2CC[C@H](C(=O)Nc3ccncc3)CC2)c(=O)c2ccncc21	316.2	1
TNKS1_active_556	CHEMBL4796484	N#Cc1ccccc1Cn1c(=O)n(C[C@H]2CC[C@H](C(=O)Nc3ccncc3)CC2)c(=O)c2nc[nH]c21	316.2	1
TNKS1_active_557	CHEMBL4764192	N#Cc1c(Cn2c(=O)n(C[C@H]3CC[C@H](C(=O)O)CC3)c(=O)c3c(F)cccc32)cccc1C(F)(F)F	316.2	1
TNKS1_active_558	CHEMBL4076784	C1CCC(CCCCNc2nnc3n2CCS3)CC1	316.23	1
TNKS1_activ	CHEMBL46	O=C(OCc1ccccc1)N1CCN(CCSc2nc3ccccc3c(=O)[nH]2)CC1	318	1

e_559	41894			
TNKS1_active_560	CHEMBL3936070	O=C(CCc1ccccc1)N(Cc1nc2c(c(=O)[nH]1)COCC2)Cc1ccc(Cl)s1	318	1
TNKS1_active_561	CHEMBL4102763	N#Cc1ccc2c(c1)[nH]c(=O)n2[C@H]1CC[C@H](c2nnc(-c3ccncn3)n2-c2ccccc2Cl)CC1	330	3
TNKS1_active_562	CHEMBL3577702	COc1ccc2c(c1)C(CC(=O)N1CCN(C(=O)c3ccccc3N3CCC(=O)NC3=O)CC1)CC2=O	330.565	2
TNKS1_active_563	CHEMBL2419695	O=C(CCc1ccc(O)cc1)N(Cc1nc2c(c(=O)[nH]1)COCC2)Cc1cccs1	343	2
TNKS1_active_564	CHEMBL3589258	Cc1ccc(-c2cc3cnccc3c(=O)[nH]2)cc1	350	1
TNKS1_active_565	CHEMBL2425790	CC(C)(C)c1ccc(C(=O)Nc2nnc3n2CCCS3)cc1	353	1
TNKS1_active_566	CHEMBL3942774	COc1ccc(C(=O)C2CCN(CC(=O)NCc3nc4c(c(=O)[nH]3)CCCCC4)CC2)cc1	353	1
TNKS1_active_567	CHEMBL268379	CC(C)c1ccc(C2NC(=O)c3cccnc3N2)cc1	357.405	2
TNKS1_active_568	CHEMBL5174447	O=C(Nc1ccc(C(F)(F)F)cc1)c1ccc(NC(=O)N2CCN(C(=O)c3cc(Cc4n[nH]c(=O)c5ccccc45)ccc3F)CC2)cc1	366.5	1
TNKS1_active_569	CHEMBL5406267	Oc1ccc2c(c1)sc1nncn12	370.77	2
TNKS1_active_570	CHEMBL3918346	COc1ccc(C(=O)C2CCN(CC(=O)N(Cc3nc4c(c(=O)[nH]3)CCCCC4)CC3CC3)CC2)cc1	371	1
TNKS1_active_571	CHEMBL3958005	COc1ccc(C(=O)C2CCN([C@H]3CCN(CC(=O)Nc4nnc5n4CCS5)C3=O)CC2)cc1	374	1
TNKS1_active_572	CHEMBL2419894	O=c1[nH]c(-c2ccc(C(F)(F)F)cc2)nc2c1CCOC2	378	1
TNKS1_active_573	CHEMBL3933643	COc1ccc(-c2cnn(C3CCN(CC(=O)NCc4nc5c(c(=O)[nH]4)COCC5)CC3)c2)cc1	386	1
TNKS1_active_574	CHEMBL4111286	COc1ccc(C(=O)C2CCN([C@@H]3CCN(Cc4nc5ccccc(=O)c5[nH]4)C3=O)CC2)cc1	390	1
TNKS1_active_575	CHEMBL4761317	N#Cc1ccccc1Cn1c(=O)n(C[C@H]2CC[C@H](C(=O)Nc3ccncc3)CC2)c(=O)c2ncccc21	398.1	1
TNKS1_active_576	CHEMBL1448672	O=C1NC(c2ccc(Cl)cc2)Nc2ccccc21	413.435	2
TNKS1_active_577	CHEMBL2425806	O=C(NCc1ccccc1)Nc1nnc2n1CCS2	444	1
TNKS1_active_578	CHEMBL1173055	CNCc1ccc(-c2[nH]c3cc(F)cc4c3c2CCNC4=O)cc1	452.065	4
TNKS1_active_579	CHEMBL4112930	C[C@]12CCCN1CC1=NNC(=O)c3cc(F)cc4[nH]c2c1c34	453	2
TNKS1_active_580	CHEMBL16751	COc1ccc(-c2cc(=O)c3ccc(O)cc3o2)cc1	453.545	2
TNKS1_active_581	CHEMBL3827770	COc1cccc2c(=O)[nH]c(-c3ccc(F)cc3)nc12	459	1
TNKS1_active_582	CHEMBL3577700	COc1ccc2c(c1)C(CC(=O)N[C@@H](CNC(=O)c1ccccc1N1CCC(=O)NC1=O)C(N)=O)CC2=O	468.87	2
TNKS1_active_583	CHEMBL3929889	CN(C)CCn1c2c3c4c(cc(F)cc41)C(=O)NN=C3CC(C)(C)C2	480	1
TNKS1_active_584	CHEMBL1483505	CCOc1ccc(-n2c(SCc3nc(-c4ccc(OC)cc4)no3)nnc2-c2ccncc2)cc1	509	1
TNKS1_active_585	CHEMBL3289109	Cc1ccc(-c2nc3ccccc3c(=O)[nH]2)cc1	540	1
TNKS1_active_586	CHEMBL4159240	CC(C)(C)OC(=O)N1CCC2(CCN(C(=O)c3cc(Cc4n[nH]c(=O)c5ccccc45)ccc3F)C2)C1	576	1
TNKS1_active_587	CHEMBL3594132	O=C1NC(c2ccccc2OCc2ccccc2)Nc2ccccc21	577.72	2
TNKS1_active_588	CHEMBL4642139	O=c1[nH]c(SCCN2CCN(CCCOc3ccc(-c4ccccc4)cc3)CC2)nc2ccccc12	588	1

TNKS1_active_589	CHEMBL1319888	Cc1ccc(-c2noc(CSc3nnc(C)n3-c3ccc(C)cc3)n2)cc1	589	1
TNKS1_active_590	CHEMBL4107943	COc1ccc(C(=O)C2CCN([C@@H]3CCN(Cc4nc5c(c(=O)[nH]4)COCC5)C3=O)CC2)cc1	600	1
TNKS1_active_591	CHEMBL4077094	O=c1[nH]c(-c2ccc3c(c2)OCO3)nc2ccccc12	620	1
TNKS1_active_592	CHEMBL253786	O=c1cc(-c2ccc(O)c2)oc2cc(O)ccc12	625.48	2
TNKS1_active_593	CHEMBL1418516	COc1ccc(-c2noc(CSc3nnc(C)n3-c3ccc(C)cc3)n2)cc1	629	1
TNKS1_active_594	CHEMBL3969055	COc1ccc(C(=O)C2CCN(C3CCN(CC(=O)Nc4nnc5ccccn45)C3=O)CC2)cc1	636	1
TNKS1_active_595	CHEMBL16861	Cc1ccc(-c2cc(=O)c3ccccc3o2)cc1	645.275	4
TNKS1_active_596	CHEMBL1552719	COc1ccc(-n2c(SCc3nc(-c4ccc(C)cc4)no3)nnc2-c2ccncc2)cc1	650	4
TNKS1_active_597	CHEMBL3827921	Nc1ccc(-c2nc3ccccc3c(=O)[nH]2)cc1	690	1
TNKS1_active_598	CHEMBL4585900	CC(C)(O)c1ccc(-c2nc3ccc(F)cc3c(=O)[nH]2)nc1	700	1
TNKS1_active_599	CHEMBL3984980	CC1(C)CC2=NNC(=O)c3cccc4c3c2c(n4CCN2CCCCC2)C1	700	1
TNKS1_active_600	CHEMBL2144572	CCc1c(C)nc(N2CCN(c3ccccc3F)CC2)nc1O	722.22	2
TNKS1_active_601	CHEMBL3942389	CC(c1nc2c(c(=O)[nH]1)COCC2)N(C)C(=O)CN1CCC(C(=O)c2ccc(F)cc2)CC1	723	1
TNKS1_active_602	CHEMBL3594127	O=C1NC(c2ccc(Cl)cc2)Nc2ncccc21	759.29	2
TNKS1_active_603	CHEMBL3978051	O=C(CN1CCC2(CCc3ccccc3C2=O)CC1)NCc1nc2c(c(=O)[nH]1)COCC2	760	1
TNKS1_active_604	CHEMBL3894926	COc1ccc(C(=O)C2CCN(C3CCN(Cc4nccc(=O)[nH]4)C3=O)CC2)cc1	764	1
TNKS1_active_605	CHEMBL3927814	O=C(c1ccc(OCc2ccccc2)cc1)C1CCN(CC(=O)N(Cc2nc3c(c(=O)[nH]2)COCC3)CC2CC2)CC1	764	1
TNKS1_active_606	CHEMBL2419693	O=C(CCc1ccccc1O)N(Cc1nc2c(c(=O)[nH]1)COCC2)Cc1cccs1	766	2
TNKS1_active_607	CHEMBL2419696	COc1cc(Cl)c(C#N)cc1C(=O)NCCN1CCC(C(=O)c2ccc(F)cc2)CC1	770	1
TNKS1_active_608	CHEMBL1443282	COc1ccc(-c2noc(CSc3nnc(-c4ccncc4)n3-c3ccccc3)n2)cc1	783	1
TNKS1_active_609	CHEMBL3969046	O=C(CN1CCC(C(=O)N2CCc3ccccc3C2)CC1)NCc1nc2c(c(=O)[nH]1)COCC2	815	1
TNKS1_active_610	CHEMBL3972156	CC1(C)CC2=NNC(=O)c3cccc4[nH]c(c2c34)C1	832	1
TNKS1_active_611	CHEMBL2016863	COc1ccc(-c2noc(CSc3nnc(C)n3-c3ccccc3)n2)cc1OC	861	1
TNKS1_active_612	CHEMBL125154	COc1cccc2c(O)nc(-c3ccc([N+](=O)[O-])cc3)nc12	863	1
TNKS1_active_613	CHEMBL301624	O=c1cc(-c2ccc(O)c(O)c2)oc2cc(O)ccc12	870.48	2
TNKS1_active_614	CHEMBL3913122	O=c1[nH]c(CN(Cc2cccs2)S(=O)(=O)CCc2ccccc2)nc2c1COCC2	910	1
TNKS1_active_615	CHEMBL3577627	O=C1CCN(c2ccccc2C(=O)NCCc2ccc(Br)cc2)C(=O)N1	911.005	2
TNKS1_active_616	CHEMBL3907010	CC1CN(C(=O)c2cc(Cc3n[nH]c(=O)c4ccccc34)ccc2F)Cc2nnc(C(F)(F)F)n21	930	1
TNKS1_active_617	CHEMBL5398835	Cc1ccc2sc3nncn3c2c1	952.495	2
TNKS1_activ	CHEMBL41	CC(C)(C)OC(=O)N1CCC2(CC1)CCN(C(=O)c1cc(Cc3n[nH]c(=O)c4ccccc3	953	1

e_618	75833	4)ccc1F)C2		
TNKS1_active_619	CHEMBL2417574	O=C(CCc1ccncc1)N(Cc1nc2c(c(=O)[nH]1)COCC2)Cc1cccs1	960	2
TNKS1_active_620	CHEMBL3951549	COc1ccc(C(=O)C2CCN(C3CCN(CC(=O)Nc4nncc5c4CCCO5)C3=O)CC2)cc1	970	1
TNKS1_active_621	CHEMBL2425792	CC(C)CC(=O)Nc1nnc2n1CCCS2	980	1
TNKS1_active_622	CHEMBL138007	COc1ccc(C2NC(=O)c3ccccc3N2)cc1	995	2
TNKS1_active_623	CHEMBL3951890	O=C(CCc1ccccc1)N(Cc1nc2c(c(=O)[nH]1)COCC2)CC1CCCC1	995	1
TNKS1_active_624	CHEMBL5402663	COc1ccc2sc3nncn3c2c1	1000	1
TNKS1_active_625	CHEMBL5433172	COc1cccc2sc3nncn3c12	1000	1
TNKS1_active_626	CHEMBL4519178	NC(=O)c1cccc2c1O/C(=C\c1ccc(C(=O)N3CCn4c(nnc4C(F)(F)F)C3)cc1)C2=O	1000	1
TNKS1_active_627	CHEMBL126871	COc1cccc2c(O)nc(-c3ccccc3)nc12	1029	1
TNKS1_active_628	CHEMBL2419716	O=C(CCc1ccccc1)N(Cc1ccsc1)Cc1nc2c(c(=O)[nH]1)COCC2	1030	2
TNKS1_active_629	CHEMBL3947278	CC1(C)CCc2[nH]c3cccc4c3c2C1=NNC4=O	1050	1
TNKS1_active_630	CHEMBL3891547	O=C(CCc1ccccc1)N(Cc1nc2c(c(=O)[nH]1)COCC2)Cc1ccccc1F	1050	1
TNKS1_active_631	CHEMBL4084811	O=c1[nH]c2ccccc2n1[C@H]1CC[C@H](c2nnc(-c3ccncn3)n2-c2ccccc2Cl)CC1	1060	3
TNKS1_active_632	CHEMBL1880061	Cc1ccc(-c2noc(CSc3nnc(C)n3-c3ccc(F)cc3)n2)cc1	1070	1
TNKS1_active_633	CHEMBL151728	COc1cccc2c(O)nc(-c3ccc(N)cc3)nc12	1076	1
TNKS1_active_634	CHEMBL151	O=c1cc(-c2ccc(O)c(O)c2)oc2cc(O)cc(O)c12	1085.76	2
TNKS1_active_635	CHEMBL53738	O=c1cc(-c2ccc(F)cc2)oc2ccccc12	1098.24	2
TNKS1_active_636	CHEMBL1315989	COc1ccc(-c2noc(CSc3nnc(-c4ccncc4)n3-c3ccc(C)c(C)c3)n2)cc1	1100	1
TNKS1_active_637	CHEMBL18688	Oc1nc(-c2ccc(F)cc2)nc2ccccc12	1102	1
TNKS1_active_638	CHEMBL3907535	CC(C)N1CC2=NNC(=O)c3cccc4[nH]c(c2c34)C1	1200	1
TNKS1_active_639	CHEMBL5402584	Cc1cc(C)c2c(c1)sc1nncn12	1200	1
TNKS1_active_640	CHEMBL4107982	C[C@]12CCCN1CC1=NNC(=O)c3cccc4[nH]c2c1c34	1220	1
TNKS1_active_641	CHEMBL3975917	CN(C)CCn1c2c3c4c(cccc41)C(=O)NN=C3CC(C)(C)C2	1300	1
TNKS1_active_642	CHEMBL127066	Oc1nc(-c2ccccc2)nc2ccccc12	1370	2
TNKS1_active_643	CHEMBL2381635	COc1ccccc1-c1ccc2c(C)cc(=O)[nH]c2c1	1370	1
TNKS1_active_644	CHEMBL1515576	COc1ccc(-c2noc(CSc3nnc(-c4ccncc4)n3-c3ccc(C)cc3)n2)cc1	1420	1
TNKS1_active_645	CHEMBL3977381	COc1ccc(C(=O)C2CCN(C3CCN(CC(=O)Nc4nnnn4C)C3=O)CC2)cc1	1480	1
TNKS1_active_646	CHEMBL3947623	CC1(C)CC2=NNC(=O)c3cccc4c3c2c(n4CCN2CCCC2)C1	1500	1
TNKS1_active_647	CHEMBL3967400	O=C(CCc1c[nH]c2ccccc12)N(Cc1nc2c(c(=O)[nH]1)COCC2)Cc1cccs1	1580	1

TNKS1_active_648	CHEMBL3947271	O=C(CCc1ccccc1)N(Cc1nc2c(c(=O)[nH]1)COCC2)Cc1ncco1	1700	1
TNKS1_active_649	CHEMBL1527375	CC(C)C1Cc2nc(O)c(C#N)cc2CO1	1700	1
TNKS1_active_650	CHEMBL3892461	COc1ccc2c(c1)CC1(CCN([C@@H]3CCN(Cc4nc5c(c(=O)[nH]4)COCC5)C3=O)CC1)C2=O	1720	1
TNKS1_active_651	CHEMBL2425817	O=C(CCn1cncn1)Nc1nnc2n1CCS2	1790	1
TNKS1_active_652	CHEMBL2325498	Cc1cc2nc(CSc3nnc(-c4ccncc4)n3-c3ccccc3Cl)cc(=O)n2cc1C	1800	1
TNKS1_active_653	CHEMBL4108883	C[C@H](c1ccccc1)N(Cc1nc2c(c(=O)[nH]1)COCC2)S(=O)(=O)Cc1ccccc1	1850	1
TNKS1_active_654	CHEMBL2016635	Cc1ccc(-n2c(SCc3nc(-c4ccccc4)no3)nnc2-c2ccncc2)cc1	1880	1
TNKS1_active_655	CHEMBL3753077	COc1ccc(C2(CNC(=O)c3ccc(NC(=O)c4ccco4)cc3)CCOCC2)cc1	1900	5
TNKS1_active_656	CHEMBL3594126	O=C1NC(c2ccc(OCc3ccccc3)cc2)Nc2ncccc21	1924.92	2
TNKS1_active_657	CHEMBL2425794	O=C(Nc1nnc2n1CCS2)C1CC1	2010	1
TNKS1_active_658	CHEMBL3927114	O=C(CCc1ccccc1)N(Cc1nc2c(c(=O)[nH]1)COCC2)Cc1nccs1	2030	1
TNKS1_active_659	CHEMBL1474898	Cc1ccc(-c2noc(CSc3nnc(C)n3-c3ccc(C)c(C)c3)n2)cc1	2040	1
TNKS1_active_660	CHEMBL3594129	COc1ccc(C2NC(=O)c3cccnc3N2)cc1	2118.98	2
TNKS1_active_661	CHEMBL4106542	O=C(c1ccc(F)cc1)C1CCN([C@@H]2CCN(Cc3nc4c(c(=O)[nH]3)COCC4)C2=O)CC1	2120	1
TNKS1_active_662	CHEMBL3752386	COc1ccc(C2(CNC(=O)c3ccc(NC(=O)c4ccco4)cc3Cl)CCOCC2)cc1	2143.98	2
TNKS1_active_663	CHEMBL3589254	O=c1[nH]c(-c2ccccc2)cc2ccncc12	2200	1
TNKS1_active_664	CHEMBL3753249	COc1ccc(C2(CNC(=O)c3ccc(NC(=O)c4cnc(C)o4)cc3Cl)CCOCC2)cc1	2234.36	2
TNKS1_active_665	CHEMBL3752054	COc1ccc(C2(CNC(=O)c3ccc(NC(=O)c4ccc(C)o4)cc3)CCOCC2)cc1	2295.435	2
TNKS1_active_666	CHEMBL2425807	O=C(CCC1CCNCC1)Nc1nnc2n1CCS2	2320	1
TNKS1_active_667	CHEMBL3593717	CN1c2ncccc2C(=O)NC1c1ccc(NC(=O)C2CCC2)cc1	2322.115	2
TNKS1_active_668	CHEMBL521686	O=C(c1cc(Cc2n[nH]c(=O)c3ccccc23)ccc1F)N1CCN(C(=O)C2CC2)CC1	2340	7
TNKS1_active_669	CHEMBL3577628	O=C1CCN(c2ccccc2C(=O)NCCc2ccc(Cl)cc2)C(=O)N1	2399.415	2
TNKS1_active_670	CHEMBL3926240	CCN(CC)CCn1c2c3c4c(cccc41)C(=O)NN=C3CC(C)(C)C2	2400	1
TNKS1_active_671	CHEMBL5396332	c1ccc2c(c1)sc1nncn12	2500	1
TNKS1_active_672	CHEMBL5284305	Cc1cc2c(=O)[nH]c(-c3ccc(C(C)(C)O)cc3)nn2n1	2600	1
TNKS1_active_673	CHEMBL1729169	CCc1ccc(-n2c(C)nnc2SCc2nc(-c3ccc(OC)cc3)no2)cc1	2630	1
TNKS1_active_674	CHEMBL3965525	O=C(CCc1ccccc1)N(CCO)Cc1nc2c(c(=O)[nH]1)COCC2	2680	1
TNKS1_active_675	CHEMBL3594133	O=C1NC(c2ccc(OCc3ccccc3)cc2)Nc2ccccc21	2695.765	2
TNKS1_active_676	CHEMBL3594134	Cc1ccc(C2NC(=O)c3ccccc3N2)cc1	2695.765	2
TNKS1_activ	CHEMBL24	O=C(Nc1nnc2n1CCCS2)c1ccccc1	2760	1

TNKS1_active_677	CHEMBL25791			
TNKS1_active_678	CHEMBL4177509	CC(C)(C)OC(=O)N1CCC2(CC1)CCN(C(=O)c1cc(Cc3n[nH]c(=O)c4ccccc34)ccc1F)CC2	2800	1
TNKS1_active_679	CHEMBL3422630	COCC(=O)N1CCN(C(=O)[C@H]2CC[C@H](Cn3c(=O)c4cc(OC)ccc4n(C)c3=O)CC2)CC1	2900	1
TNKS1_active_680	CHEMBL28	O=c1cc(-c2ccc(O)cc2)oc2cc(O)cc(O)c12	2925.605	2
TNKS1_active_681	CHEMBL2419715	O=c1[nH]c(CNCc2cccs2)nc2c1COCC2	2930	1
TNKS1_active_682	CHEMBL3964501	CC(C)(C)C(=O)CN(Cc1nc2c(c(=O)[nH]1)COCC2)C(=O)CCc1ccccc1	2990	1
TNKS1_active_683	CHEMBL3740897	C[C@H]1Cn2c(nnc2C(C)(F)F)CN1C(=O)c1cccc(C(F)(F)c2n[nH]c(=O)c3ccccc23)c1	3000	1
TNKS1_active_684	CHEMBL5421878	Oc1ccc(O)c2c1sc1nncn12	3100	1
TNKS1_active_685	CHEMBL3915319	C[C@@H](c1ccccc1)N(Cc1nc2c(c(=O)[nH]1)COCC2)S(=O)(=O)Cc1ccccc1	3120	1
TNKS1_active_686	CHEMBL4436714	COc1ccccc1N1CCN(c2nc3c(c(=O)[nH]2)CC(C)CC3)CC1	3476	1
TNKS1_active_687	CHEMBL3916721	O=C(CCc1ccccc1)N(Cc1nc2c(c(=O)[nH]1)COCC2)Cc1cncs1	3480	1
TNKS1_active_688	CHEMBL3970252	CC(C)(N)C(=O)N1CC2=NNC(=O)c3cccc4[nH]c(c2c34)C1	3800	1
TNKS1_active_689	CHEMBL5427075	Cc1cccc2c1sc1nncn12	3800	1
TNKS1_active_690	CHEMBL5095043	C[C@@H](COCCC(=O)N1CCN(c2ncc(C(F)(F)F)cn2)CC1)Nc1cn[nH]c(=O)c1C(F)(F)F	3973	2
TNKS1_active_691	CHEMBL5416085	COc1ccc(OC)c2c1sc1nncn12	4000	1
TNKS1_active_692	CHEMBL4582338	COc1ccccc1N1CCCN(c2nc3c(c(=O)[nH]2)CCCC3)CC1	4386	1
TNKS1_active_693	CHEMBL3594124	O=C1NC(c2ccccc2OCc2ccccc2)Nc2ncccc21	4483.42	2
TNKS1_active_694	CHEMBL3981506	COc1ccc(C(=O)C2CCN(C3CCN(CC(=O)Nc4noc5ncccc45)C3=O)CC2)cc1	4560	1
TNKS1_active_695	CHEMBL5091585	CCOc1ccc2c(=O)[nH]c3sccc3c2c1	4700	1
TNKS1_active_696	CHEMBL2419713	CCN(Cc1nc2c(c(=O)[nH]1)COCC2)C(=O)CCc1ccccc1	4780	2
TNKS1_active_697	CHEMBL1094636	NC(=O)c1cccc2cn(-c3ccc([C@@H]4CCCNC4)cc3)nc12	5129.305	2
TNKS1_active_698	CHEMBL4064869	N#Cc1ccc2c(c1)[nH]c(=O)n2-c1ccc(-c2nnc(-c3ccncn3)n2-c2ccccc2Cl)cc1	5170	2
TNKS1_active_699	CHEMBL4296925	Cc1ccc2c(c1)sc1nncn12	5300	1
TNKS1_active_700	CHEMBL5395131	CCc1ccc2c(c1)sc1nncn12	5300	1
TNKS1_active_701	CHEMBL4556137	COc1ccccc1N1CCN(c2nc3c(c(=O)[nH]2)C[S+]([O-])CC3)CC1	5345	1
TNKS1_active_702	CHEMBL3594128	Cc1ccc(C2NC(=O)c3cccnc3N2)cc1	5385.16	2
TNKS1_active_703	CHEMBL3974949	CC(C)N(Cc1nc2c(c(=O)[nH]1)COCC2)C(=O)CCc1ccccc1	5390	1
TNKS1_active_704	CHEMBL5427721	Cc1ccc(F)c2c1sc1nncn12	5400	1
TNKS1_active_705	CHEMBL129312	O=C1NC(c2ccccc2)Nc2ccccc21	5497.705	2
TNKS1_active_706	CHEMBL5398837	COc1ccc2c(c1)sc1nncn12	5500	1

TNKS1_active_707	CHEMBL5431108	Cc1ccc2c(c1)sc1nnc(N)n12	5700	1
TNKS1_active_708	CHEMBL3593718	CC(C)C(=O)Nc1ccc(C2NC(=O)c3cccnc3N2C)cc1	6254.785	2
TNKS1_active_709	CHEMBL4438734	COc1ccccc1N1CCC(c2nc3c(c(=O)[nH]2)CCCC3)CC1	6417	1
TNKS1_active_710	CHEMBL4126054	NC(=O)c1ccc(Oc2cccc(C(N)=O)c2)cc1	6500	1
TNKS1_active_711	CHEMBL4445952	COc1ccccc1N1CCN(c2nc3c(c(=O)[nH]2)CNCC3)CC1	6984	1
TNKS1_active_712	CHEMBL1900376	O=C1NC(c2cccc(OCc3ccccc3)c2)Nc2ccccc21	7272.18	2
TNKS1_active_713	CHEMBL5430541	COc1cc(OC)c2sc3nncn3c2c1	7600	1
TNKS1_active_714	CHEMBL3577701	COc1ccc2c(c1)C(CC(=O)NCCNC(=O)c1ccccc1N1CCC(=O)NC1=O)CC2=O	7921.64	2
TNKS1_active_715	CHEMBL4751197	N#Cc1ccccc1Cn1c(=O)n(C[C@H]2CC[C@H](C(=O)NCc3ccccc3)CC2)c(=O)c2ccccc21	7943.3	1
TNKS1_active_716	CHEMBL2419717	O=C(CCc1cccnc1)N(Cc1nc2c(c(=O)[nH]1)COCC2)Cc1cccs1	7960	1
TNKS1_active_717	CHEMBL3577624	O=C1CCN(c2ccccc2C(=O)NCCc2nc(-c3ccccn3)no2)C(=O)N1	8114.155	2
TNKS1_active_718	CHEMBL3593719	CN1c2ncccc2C(=O)NC1c1ccc(NC(=O)C2CCOCC2)cc1	8300	2
TNKS1_active_719	CHEMBL3577625	O=C1CCN(c2ccccc2C(=O)NCc2ccc(-n3cccn3)cc2)C(=O)N1	8308.82	2
TNKS1_active_720	CHEMBL4517609	COc1ccccc1N1CCN(C2=NC3CCCCCC3C(=O)N2)CC1	9336	1
TNKS1_active_721	CHEMBL5439304	CC(C)c1ccc2c(c1)sc1nncn12	9500	1
TNKS1_active_722	CHEMBL2325495	COc1ccc(-n2c(OCc3nc(-c4ccc(C)cc4)no3)nnc2-c2ccncc2)cc1	10000	1
TNKS1_active_723	CHEMBL2325496	COc1ccc(-n2c(NCc3nc(-c4ccc(C)cc4)no3)nnc2-c2ccncc2)cc1	10000	1
TNKS1_active_724	CHEMBL5200521	O=c1[nH][nH]c(=O)c2cc(OCc3ccsc3)ccc12	10000	1
TNKS1_active_725	CHEMBL5404785	COc1ccc(OC)c2c1sc1nnc(N)n12	10000	1
TNKS1_active_726	CHEMBL5395721	Clc1ccc2c(c1)sc1nncn12	10000	1
TNKS1_active_727	CHEMBL3593720	CN1c2ncccc2C(=O)NC1c1ccc(Cl)cc1Cl	10000	1

Table S2. Curated TNKS1 inhibitor dataset used for machine learning model development. The dataset comprises 727 experimentally validated TNKS1 inhibitors retrieved from the ChEMBL database after bioactivity curation, molecular standardization, duplicate removal, and quality-control filtering. For compounds with multiple experimental IC_{50} measurements, a single representative value was obtained by median aggregation. The table reports the median IC_{50} value (nM) for each compound together with the number of experimental IC_{50} records contributing to the reported median value.

Model	*Hyperparameter configuration*
Logistic Regression	solver = lbfgs; penalty = L2; C = 1.0; class_weight = balanced; max_iter = 5000; random_state = 42
SVM-RBF	kernel = rbf; C = 1.0; gamma = scale; probability = True; class_weight = balanced; random_state = 42
XGBoost	objective = binary:logistic; n_estimators = 300; learning_rate = 0.05; max_depth = 5; subsample = 0.8; colsample_bytree = 0.8; eval_metric = logloss; random_state = 42

Table S3. Hyperparameter configuration of the supervised machine learning models used for TNKS1 inhibitor classification. Hyperparameter settings of the three supervised machine learning classifiers used throughout model development and external virtual screening. Parameters not explicitly listed were retained at their default values in the corresponding software implementations.

Item	***Setting***
Python	3.9.6
RDKit	2022.03.4
scikit-learn	1.5.1
XGBoost	2.1.3
NumPy	1.26.4
pandas	2.2.2
Molecular representation	Morgan fingerprints (radius = 2; 2048 bits)
Dataset balancing	Random undersampling without replacement (active:decoy ratio = 1:5)
Dataset partitioning	Stratified split (70% training, 15% validation, 15% independent test)
Random seed	42
Classification threshold	0.50
Consensus score	Arithmetic mean of the prediction probabilities generated by the three classifiers
Virtual screening criterion	Only compounds with prediction probability > 0.90 for all three classifiers were retained
Probability calibration	Not applied

Table S4. Computational environment and implementation details of the machine learning workflow. Summary of the computational environment, software versions, dataset preparation strategy, model evaluation protocol, and consensus prediction procedure adopted throughout machine learning model development and external virtual screening.

SMILES_raw	Source	SMILES	XGBoost_prob	LogReg_prob	SVM_RBF_prob	Consensus_score
**O=C(c1ccccc1F)N1CCc2c(nc(N3CCOCC3)[nH]c2=O)C1**	ASINEX	O=C(c1ccccc1F)N1CCc2c(nc(N3CCOCC3)[nH]c2=O)C1	0.999928	0.9999984048444576	0.9999999984326504	0.9999754669554064
**O=c1[nH]c(N2CCOCC2)nc2c1CN(Cc1cccc3cccnc13)CC2**	ASINEX	O=c1[nH]c(N2CCOCC2)nc2c1CN(Cc1cccc3cccnc13)CC2	0.9998821	0.9999990571812042	0.9999941055270852	0.9999584215736412
**O=C(CCc1ccccc1)N1CCc2c(nc(N3CCOCC3)[nH]c2=O)C1**	ASINEX	O=C(CCc1ccccc1)N1CCc2c(nc(N3CCOCC3)[nH]c2=O)C1	0.99984384	0.9999874463956456	0.9999989490993836	0.999943410441906
**O=C(N1CCc2nc(N3CCOCC3)[nH]c(=O)c2C1)N(c1ccccc1)c1ccccc1**	ASINEX	O=C(N1CCc2nc(N3CCOCC3)[nH]c(=O)c2C1)N(c1ccccc1)c1ccccc1	0.9998735	0.999889624103444	0.9999980670983688	0.9999204033818664
**O=c1[nH]c(N2CCCCC2)nc2c1CCN(Cc1cccc3cccnc13)C2**	ASINEX	O=c1[nH]c(N2CCCCC2)nc2c1CCN(Cc1cccc3cccnc13)C2	0.99971765	0.9999999074808308	0.9999988114104216	0.9999054572296504
**O=c1[nH]c(N2CCCC2)nc2c1CN(Cc1cccc3cccnc13)CC2**	ASINEX	O=c1[nH]c(N2CCCC2)nc2c1CN(Cc1cccc3cccnc13)CC2	0.99971765	0.999998379801878	0.9999962887925568	0.9999041071307112
**O=C(c1ccccc1F)N1CCc2c(nc(N3CCCC3)[nH]c2=O)C1**	ASINEX	O=C(c1ccccc1F)N1CCc2c(nc(N3CCCC3)[nH]c2=O)C1	0.99969244	0.9999972587873583	0.9999999991634466	0.999896565994588
**O=c1[nH]c(N2CCCC2)nc2c1CCN(Cc1cccc3nccnc13)C2**	ASINEX	O=c1[nH]c(N2CCCC2)nc2c1CCN(Cc1cccc3nccnc13)C2	0.9997056	0.9999858714145684	0.999993948768642	0.999895144280888
**O=c1[nH]c(N2CCCCC2)nc2c1CCN(Cc1cccc3nccnc13)C2**	ASINEX	O=c1[nH]c(N2CCCCC2)nc2c1CCN(Cc1cccc3nccnc13)C2	0.9997056	0.9999833152994624	0.999989759322962	0.9998928957606262
**O=C(c1ccccc1-c1ccccc1)N1CCc2nc(N3CCCCC3)[nH]c(=O)c2C1**	ASINEX	O=C(c1ccccc1-c1ccccc1)N1CCc2nc(N3CCCCC3)[nH]c(=O)c2C1	0.99976784	0.9999071673503712	0.9999999996017428	0.9998916689535714
**N#Cc1cccc(NC(=O)N2CCc3nc(N4CCOCC4)[nH]c(=O)c3C2)c1**	ASINEX	N#Cc1cccc(NC(=O)N2CCc3nc(N4CCOCC4)[nH]c(=O)c3C2)c1	0.9996973	0.9997990215020448	0.999989829315566	0.9998287261438138
**CC(C(=O)N1CCc2c(nc(N3CCOCC3)[nH]c2=O)C1)c1ccccc1**	ASINEX	CC(C(=O)N1CCc2c(nc(N3CCOCC3)[nH]c2=O)C1)c1ccccc1	0.99957806	0.9999042595043623	0.9999924069330176	0.9998249083856716
**O=C(NCc1ccccc1)N1CCc2nc(N3CCOCC3)[nH]c(=O)c2C1**	ASINEX	O=C(NCc1ccccc1)N1CCc2nc(N3CCOCC3)[nH]c(=O)c2C1	0.999767	0.9997028362705208	0.9999991333367344	0.9998229916836094
**O=C(c1cccnc1)N1CCc2c(nc(N3CCCC3)[nH]c2=O)C1**	ASINEX	O=C(c1cccnc1)N1CCc2c(nc(N3CCCC3)[nH]c2=O)C1	0.99951434	0.9999396840336954	0.9999881594525885	0.9998140616135514
**CC1CN(c2nc3c(c(=O)[nH]2)CCN(c2ncccn2)C3)CC(C)O1**	ASINEX	CC1CN(c2nc3c(c(=O)[nH]2)CCN(c2ncccn2)C3)CC(C)O1	0.9994161	0.999970958280454	0.9999977483055742	0.9997949398286028
**O=C(c1ccccn1)N1CCc2c(nc(N3CCCC3)[nH]c2=O)C1**	ASINEX	O=C(c1ccccn1)N1CCc2c(nc(N3CCCC3)[nH]c2=O)C1	0.99941015	0.9999701915711736	0.9999999761728064	0.9997934400597608
**CC(CN1CCc2c(nc(N3CCOCC3)[nH]c2=O)C1)c1ccccc1**	ASINEX	CC(CN1CCc2c(nc(N3CCOCC3)[nH]c2=O)C1)c1ccccc1	0.99956447	0.9997530400241929	0.9999815720671908	0.9997663603173368
**O=C(CCc1ccccc1)N1CCc2c(nc(N3CCCC3)[nH]c2=O)C1**	ASINEX	O=C(CCc1ccccc1)N1CCc2c(nc(N3CCCC3)[nH]c2=O)C1	0.9992568	0.9999784272899342	0.9999993083704064	0.9997448417815454
**O=C(Cc1n[nH]c(=O)c2ccccc12)N1CCc2c(nc(N3CCCC3)[nH]c2=O)C1**	ASINEX	O=C(Cc1n[nH]c(=O)c2ccccc12)N1CCc2c(nc(N3CCCC3)[nH]c2=O)C1	0.99922395	0.9999747915777448	0.9999992521373317	0.9997326637467002
**CC1CN(c2nc3c(c(=O)[nH]2)CCN(C(=O)c2ccccc2F)C3)CC(C)O1**	ASINEX	CC1CN(c2nc3c(c(=O)[nH]2)CCN(C(=O)c2ccccc2F)C3)CC(C)O1	0.99920243	0.9999888240276524	0.9999994073040276	0.9997302205266468
**COc1cccc(CN2CCc3c(nc(-c4ccncc4)[nH]c3=O)C2)c1O**	ASINEX	COc1cccc(CN2CCc3c(nc(-c4ccncc4)[nH]c3=O)C2)c1O	0.9991003	0.9999874017876316	0.99999399140458	0.9996939068946572
**O=c1[nH]c(-c2ccncc2)nc2c1CCN(Cc1ccc(OCc3ccccc3F)cc1)C2**	ASINEX	O=c1[nH]c(-c2ccncc2)nc2c1CCN(Cc1ccc(OCc3ccccc3F)cc1)C2	0.99904555	0.9999998092607796	0.9999886891173402	0.9996780164004438
**COc1ccc(-c2cc(-c3ccccc3)[nH]c(=O)c2C#N)cc1**	ASINEX	COc1ccc(-c2cc(-c3ccccc3)[nH]c(=O)c2C#N)cc1	0.99908113	0.999922539653736	0.9999967345426232	0.9996668029975004
**O=c1[nH]c(-c2ccccc2)nc2c1CCNC2**	ASINEX	O=c1[nH]c(-c2ccccc2)nc2c1CCNC2	0.999923	0.9990752112263496	0.999999258308402	0.9996658201112338
**COc1ccc(OC)c(CN2CCc3c(nc(-c4ccncc4)[nH]c3=O)C2)c1**	ASINEX	COc1ccc(OC)c(CN2CCc3c(nc(-c4ccncc4)[nH]c3=O)C2)c1	0.99893945	0.9999955854425548	0.9999925557132894	0.9996425319037852
**N#Cc1cccc(NC(=O)N2CCc3nc(N4CCCC4)[nH]c(=O)c3C2)c1**	ASINEX	N#Cc1cccc(NC(=O)N2CCc3nc(N4CCCC4)[nH]c(=O)c3C2)c1	0.99926025	0.9996546757501872	0.9999927151645428	0.9996358792228076
**N#Cc1cccc(NC(=O)N2CCc3nc(N4CCCCC4)[nH]c(=O)c3C2)c1**	ASINEX	N#Cc1cccc(NC(=O)N2CCc3nc(N4CCCCC4)[nH]c(=O)c3C2)c1	0.99926025	0.9995922248191434	0.9999869362809618	0.999613135951266
**O=C(Nc1ccccc1Cl)N1CCc2nc(N3CCOCC3)[nH]c(=O)c2C1**	ASINEX	O=C(Nc1ccccc1Cl)N1CCc2nc(N3CCOCC3)[nH]c(=O)c2C1	0.99952745	0.9992539393762192	0.999996395606799	0.9995925964530796
**CCOc1ccc(NC(=O)N2CCc3nc(N4CCOCC4)[nH]c(=O)c3C2)cc1**	ASINEX	CCOc1ccc(NC(=O)N2CCc3nc(N4CCOCC4)[nH]c(=O)c3C2)cc1	0.99911374	0.9996617263249088	0.9999997084585572	0.9995917244401
**O=C(Cc1ccc(-c2ccccc2)cc1)N1CCc2nc(N3CCCCC3)[nH]c(=O)c2C1**	ASINEX	O=C(Cc1ccc(-c2ccccc2)cc1)N1CCc2nc(N3CCCCC3)[nH]c(=O)c2C1	0.9992798	0.999486234715131	0.999996663770314	0.9995875651875412
**COc1ccc(CN2CCc3c(nc(-c4ccncc4)[nH]c3=O)C2)c(O)c1**	ASINEX	COc1ccc(CN2CCc3c(nc(-c4ccncc4)[nH]c3=O)C2)c(O)c1	0.9987766	0.9999949444686016	0.9999907299398408	0.9995874296914758
**COc1ccc(CN(Cc2nc3ccccc3c(=O)[nH]2)**	ASIN	COc1ccc(CN(Cc2nc3ccccc3c(=O)[nH]2)	0.99865	0.9999988	0.9999999	0.9995529

**C(=O)c2ccccc2F)cc1**	EX	C(=O)c2ccccc2F)cc1	985	695267368	99941195	062116004
**O=C(Cc1ccc(-c2ccccc2)cc1)N1CCc2nc(-c3ccccc3)[nH]c(=O)c2C1**	ASINEX	O=C(Cc1ccc(-c2ccccc2)cc1)N1CCc2nc(-c3ccccc3)[nH]c(=O)c2C1	0.99938416	0.9992556708905608	0.9999932123875512	0.9995443493627642
**CC(C(=O)N1CCc2nc(N3CCOCC3)[nH]c(=O)c2C1)c1ccccc1**	ASINEX	CC(C(=O)N1CCc2nc(N3CCOCC3)[nH]c(=O)c2C1)c1ccccc1	0.99957806	0.9989160071488266	0.9999899186255456	0.9994946614980024
**O=C(c1ccc(F)c(F)c1)N1CCc2nc(N3CCOCC3)[nH]c(=O)c2C1**	ASINEX	O=C(c1ccc(F)c(F)c1)N1CCc2nc(N3CCOCC3)[nH]c(=O)c2C1	0.99981195	0.998657848985927	0.9999912897612896	0.9994870286976502
**O=c1cc(oc2ccccc12)-c1ccccc1**	MOLPORT	O=c1cc(oc2ccccc12)-c1ccccc1	0.9992899	0.9991664935023016	0.999989678217869	0.9994820139941056
**O=C(O)CCc1n[nH]c(=O)c2ccccc12**	ASINEX	O=C(O)CCc1n[nH]c(=O)c2ccccc12	0.9991122	0.9992715404978576	0.9999929780347796	0.9994589024495693
**O=C(C1CCCCC1)N1CCc2c(nc(N3CCOCC3)[nH]c2=O)C1**	ASINEX	O=C(C1CCCCC1)N1CCc2c(nc(N3CCOCC3)[nH]c2=O)C1	0.9986823	0.9997064282781633	0.9999827260634716	0.9994571581531952
**COc1ccccc1CN(Cc1nc2ccccc2c(=O)[nH]1)C(=O)c1ccccc1F**	ASINEX	COc1ccccc1CN(Cc1nc2ccccc2c(=O)[nH]1)C(=O)c1ccccc1F	0.9983729	0.9999738240124748	0.9999999903683364	0.9994489089292444
**O=C(NCc1ccccc1)N1CCc2nc(N3CCCC3)[nH]c(=O)c2C1**	ASINEX	O=C(NCc1ccccc1)N1CCc2nc(N3CCCC3)[nH]c(=O)c2C1	0.9987451	0.999489444129341	0.999999303315612	0.9994112770846172
**COc1ccc(CN(Cc2nc3ccccc3c(=O)[nH]2)C(=O)c2ccc(Cl)cc2)cc1**	ASINEX	COc1ccc(CN(Cc2nc3ccccc3c(=O)[nH]2)C(=O)c2ccc(Cl)cc2)cc1	0.9981744	0.9999824034683178	0.9999999988817196	0.9993856104299522
**O=C(c1ccc2c(c1)OCCO2)N1CCc2c(nc(N3CCCC3)[nH]c2=O)C1**	ASINEX	O=C(c1ccc2c(c1)OCCO2)N1CCc2c(nc(N3CCCC3)[nH]c2=O)C1	0.9987802	0.9992593193713664	0.9999906307882752	0.9993433803681046
**CC1CN(c2nc3c(c(=O)[nH]2)CCN(CCc2ccccc2)C3)CC(C)O1**	ASINEX	CC1CN(c2nc3c(c(=O)[nH]2)CCN(CCc2ccccc2)C3)CC(C)O1	0.9982338	0.9997285029875658	0.9999891904196412	0.999317162857741
**O=C(C1CCCCC1)N1CCc2c(nc(N3CCCC3)[nH]c2=O)C1**	ASINEX	O=C(C1CCCCC1)N1CCc2c(nc(N3CCCC3)[nH]c2=O)C1	0.9982651	0.999657365235354	0.9999911223130824	0.9993045250509864
**COc1ccc(CN(Cc2nc3ccccc3c(=O)[nH]2)C(=O)c2cccc(OC)c2)cc1**	ASINEX	COc1ccc(CN(Cc2nc3ccccc3c(=O)[nH]2)C(=O)c2cccc(OC)c2)cc1	0.9978483	0.9999866284482606	0.999999999074324	0.9992782999487312
**COc1ccc(CN(Cc2nc3ccccc3c(=O)[nH]2)C(=O)c2ccc(C)cc2)cc1**	ASINEX	COc1ccc(CN(Cc2nc3ccccc3c(=O)[nH]2)C(=O)c2ccc(C)cc2)cc1	0.997677	0.9999881765858322	0.9999999997515056	0.9992217348388356
**O=C(Nc1ccccc1F)N1CCc2nc(N3CCCC3)[nH]c(=O)c2C1**	ASINEX	O=C(Nc1ccccc1F)N1CCc2nc(N3CCCC3)[nH]c(=O)c2C1	0.9990565	0.9985740353422872	0.9999830036578848	0.9992045190260074
**COc1cccc(CNCc2nc3ccccc3c(=O)[nH]2)c1**	ASINEX	COc1cccc(CNCc2nc3ccccc3c(=O)[nH]2)c1	0.9975178	0.9999606217970696	0.9999999787475709	0.9991594749058712
**COc1ccc(CN(Cc2nc3ccccc3c(=O)[nH]2)C(=O)c2ccccc2Cl)cc1**	ASINEX	COc1ccc(CN(Cc2nc3ccccc3c(=O)[nH]2)C(=O)c2ccccc2Cl)cc1	0.99748176	0.9999961016212996	0.9999999968419158	0.9991592872753666
**COc1ccc(CNCc2nc3ccccc3c(=O)[nH]2)cc1**	ASINEX	COc1ccc(CNCc2nc3ccccc3c(=O)[nH]2)cc1	0.9974446	0.9999972281034148	0.9999999999741171	0.9991472859155738
**COc1cc(OC)cc(C(=O)N(Cc2ccc(F)cc2)Cc2nc3ccccc3c(=O)[nH]2)c1**	ASINEX	COc1cc(OC)cc(C(=O)N(Cc2ccc(F)cc2)Cc2nc3ccccc3c(=O)[nH]2)c1	0.9975012	0.9998938448103823	0.9999999885445544	0.999131675944006
**CO[C@H]1CC[C@H](C(=O)N2CCc3nc(-c4ccccc4)[nH]c(=O)c3C2)CC1**	ASINEX	CO[C@H]1CC[C@H](C(=O)N2CCc3nc(-c4ccccc4)[nH]c(=O)c3C2)CC1	0.9976368	0.9997094556772992	0.999997057895358	0.999114436205534
**COc1cccc(C(=O)N(Cc2ccc(F)cc2)Cc2nc3ccccc3c(=O)[nH]2)c1**	ASINEX	COc1cccc(C(=O)N(Cc2ccc(F)cc2)Cc2nc3ccccc3c(=O)[nH]2)c1	0.9973144	0.9999245700476423	0.9999999909111724	0.9990796514930568
**COc1ccc(-c2ccc(C(=O)O)c(=O)[nH]2)cc1**	ASINEX	COc1ccc(-c2ccc(C(=O)O)c(=O)[nH]2)cc1	0.99744296	0.9997739177098742	0.9999999883473004	0.9990722889321034
**O=C(c1ccccc1F)N(Cc1ccc(Cl)cc1)Cc1nc2ccccc2c(=O)[nH]1**	ASINEX	O=C(c1ccccc1F)N(Cc1ccc(Cl)cc1)Cc1nc2ccccc2c(=O)[nH]1	0.9972185	0.999970878697506	0.9999999847081016	0.9990631176841732
**O=C(c1ccc(F)c(F)c1)N1CCc2nc(N3CCCC3)[nH]c(=O)c2C1**	ASINEX	O=C(c1ccc(F)c(F)c1)N1CCc2nc(N3CCCC3)[nH]c(=O)c2C1	0.99935645	0.9976957850598298	0.9999934395002203	0.99901522440347
**CN1CCC(NC(=O)C2CCc3nc(-c4ccc(F)cc4)[nH]c(=O)c3CC2)CC1**	MOLPORT	CN1CCC(NC(=O)C2CCc3nc(-c4ccc(F)cc4)[nH]c(=O)c3CC2)CC1	0.9979646	0.999046364739103	0.9999946464705748	0.9990018772666291
**CN1CCN(CCNC(=O)C2CCc3nc(-c4ccc(F)cc4)[nH]c(=O)c3CC2)CC1**	MOLPORT	CN1CCN(CCNC(=O)C2CCc3nc(-c4ccc(F)cc4)[nH]c(=O)c3CC2)CC1	0.99696666	0.9998682369019264	0.9999992650413498	0.9989447206553372
**O=C(c1ccc(F)c(F)c1)N1CCc2nc(N3CCCCC3)[nH]c(=O)c2C1**	ASINEX	O=C(c1ccc(F)c(F)c1)N1CCc2nc(N3CCCCC3)[nH]c(=O)c2C1	0.99935645	0.9972800380969032	0.9999879254773948	0.998874804074886
**COc1ccccc1CN(Cc1nc2ccccc2c(=O)[nH]1)C(=O)c1ccccc1Cl**	ASINEX	COc1ccccc1CN(Cc1nc2ccccc2c(=O)[nH]1)C(=O)c1ccccc1Cl	0.99668425	0.99990973899311	0.999999595130715	0.998864529113205
**COc1ccccc1-n1c(SCc2nc3ccccc3c(=O)[nH]2)nnc1-c1ccccc1**	ASINEX	COc1ccccc1-n1c(SCc2nc3ccccc3c(=O)[nH]2)nnc1-c1ccccc1	0.99652714	0.9999998955399688	0.9999999983413062	0.9988423430844788
**CO[C@H]1CC[C@H](C(=O)N2CCc3nc(N4CCCC4)[nH]c(=O)c3C2)CC1**	ASINEX	CO[C@H]1CC[C@H](C(=O)N2CCc3nc(N4CCCC4)[nH]c(=O)c3C2)CC1	0.99722254	0.999220672510068	0.9999845109576108	0.9988092420768117
**CO[C@H]1CC[C@H](C(=O)N2CCc3nc(N4CCOCC4)[nH]c(=O)c3C2)CC1**	ASINEX	CO[C@H]1CC[C@H](C(=O)N2CCc3nc(N4CCOCC4)[nH]c(=O)c3C2)CC1	0.9992298	0.9995463495602548	0.9974518104226352	0.9987426495877006
**O=C(c1cccs1)N1CCc2c(nc(N3CCCC3)[nH]c2=O)C1**	ASINEX	O=C(c1cccs1)N1CCc2c(nc(N3CCCC3)[nH]c2=O)C1	0.99938476	0.9994322072856516	0.9973135938456924	0.9987101873293242

**CN1CCN(CCNC(=O)C2CCc3nc(-c4ccccc4)[nH]c(=O)c3CC2)CC1**	MOLPORT	CN1CCN(CCNC(=O)C2CCc3nc(-c4ccccc4)[nH]c(=O)c3CC2)CC1	0.99630594	0.99978095041559	0.999999289410688	0.9986953941205596
**COc1cccc(C(=O)N(Cc2nc3ccccc3c(=O)[nH]2)Cc2ccccc2OC)c1**	ASINEX	COc1cccc(C(=O)N(Cc2nc3ccccc3c(=O)[nH]2)Cc2ccccc2OC)c1	0.99624205	0.9998254642147332	0.9999999274361128	0.998689146002349
**O=C(CN1CCc2nc(N3CCCC3)[nH]c(=O)c2C1)Nc1ccc(OCc2ccccc2)cc1**	ASINEX	O=C(CN1CCc2nc(N3CCCC3)[nH]c(=O)c2C1)Nc1ccc(OCc2ccccc2)cc1	0.9992312	0.9994432928391596	0.9972786429808104	0.9986510517038856
**O=C(c1csc2c1CCCC2)N1CCc2c(nc(N3CCOCC3)[nH]c2=O)C1**	ASINEX	O=C(c1csc2c1CCCC2)N1CCc2c(nc(N3CCOCC3)[nH]c2=O)C1	0.99937636	0.9997961493858626	0.9966045576602272	0.9985923545492684
**COc1ccc(C(=O)N2CCN(Cc3nc4ccccc4c(=O)[nH]3)CC2)cc1**	ASINEX	COc1ccc(C(=O)N2CCN(Cc3nc4ccccc4c(=O)[nH]3)CC2)cc1	0.99763715	0.9981164800550418	0.9999998656674808	0.9985844994647788
**O=c1[nH]c(N2CCCC2)nc2c1CN(Cc1ccc3nccnc13)CC2**	ASINEX	O=c1[nH]c(N2CCCC2)nc2c1CN(Cc1ccc3nccnc13)CC2	0.9997056	0.9997078971134964	0.9961179284819432	0.998510479418298
**O=C(Cc1ccc(F)cc1)N1CCc2c(nc(N3CCCC3)[nH]c2=O)C1**	ASINEX	O=C(Cc1ccc(F)cc1)N1CCc2c(nc(N3CCCC3)[nH]c2=O)C1	0.9990687	0.999049929219362	0.9973512290368148	0.998489945227187
**O=C1NC(CC(=O)N2CCc3c(nc(N4CCCC4)[nH]c3=O)C2)c2ccccc21**	ASINEX	O=C1NC(CC(=O)N2CCc3c(nc(N4CCCC4)[nH]c3=O)C2)c2ccccc21	0.9984352	0.999999887575408	0.9970276779306712	0.9984875882555968
**COc1ccc(CN(Cc2nc3ccccc3c(=O)[nH]2)C(=O)c2ccccc2C)cc1**	ASINEX	COc1ccc(CN(Cc2nc3ccccc3c(=O)[nH]2)C(=O)c2ccccc2C)cc1	0.9953408	0.9999891520703862	0.9999999652075376	0.9984433138017068
**Cc1ccccc1C(=O)N(Cc1ccc(F)cc1)Cc1nc2ccccc2c(=O)[nH]1**	ASINEX	Cc1ccccc1C(=O)N(Cc1ccc(F)cc1)Cc1nc2ccccc2c(=O)[nH]1	0.9955069	0.9998057039157594	0.9999989607067328	0.9984371824300112
**COc1ccccc1CN1CCc2c(nc(-c3ccncc3)[nH]c2=O)C1**	ASINEX	COc1ccccc1CN1CCc2c(nc(-c3ccncc3)[nH]c2=O)C1	0.9992951	0.999960374475924	0.9959042101114598	0.99838656668609
**O=C(c1cccs1)N1CCc2c(nc(N3CCCCC3)[nH]c2=O)C1**	ASINEX	O=C(c1cccs1)N1CCc2c(nc(N3CCCCC3)[nH]c2=O)C1	0.99938476	0.9993295505343388	0.9964370188230026	0.99838377673799
**O=C(c1cccnc1)C1CCN(C(=O)Cc2n[nH]c(=O)c3ccccc23)CC1**	ASINEX	O=C(c1cccnc1)C1CCN(C(=O)Cc2n[nH]c(=O)c3ccccc23)CC1	0.99946004	0.9986957561283508	0.996881147180607	0.9983456482773126
**O=C(c1ccco1)N1CCc2c(nc(N3CCCC3)[nH]c2=O)C1**	ASINEX	O=C(c1ccco1)N1CCc2c(nc(N3CCCC3)[nH]c2=O)C1	0.9995782	0.9994500509243835	0.9958532776584408	0.9982938355039164
**O=C(c1cccc(Cl)c1)N(Cc1ccc(F)cc1)Cc1nc2ccccc2c(=O)[nH]1**	ASINEX	O=C(c1cccc(Cl)c1)N(Cc1ccc(F)cc1)Cc1nc2ccccc2c(=O)[nH]1	0.99525577	0.9995443145424344	0.9999993226642072	0.9982664685019148
**COc1ccc(CN(Cc2nc3ccccc3c(=O)[nH]2)C(=O)c2cccc(Cl)c2)cc1**	ASINEX	COc1ccc(CN(Cc2nc3ccccc3c(=O)[nH]2)C(=O)c2cccc(Cl)c2)cc1	0.9946578	0.9999603715996428	0.9999999730210528	0.9982060530411372
**COc1ccc(-c2cc(-c3ccccc3)nc3sc4c5ccccc5c(=O)[nH]c4c23)cc1**	ASINEX	COc1ccc(-c2cc(-c3ccccc3)nc3sc4c5ccccc5c(=O)[nH]c4c23)cc1	0.9987147	0.9996098073418428	0.9962585630508692	0.9981943519442584
**O=C(Cc1ccccn1)N1CCc2nc(N3CCCC3)[nH]c(=O)c2C1**	ASINEX	O=C(Cc1ccccn1)N1CCc2nc(N3CCCC3)[nH]c(=O)c2C1	0.998689	0.9991423086230432	0.9966201161799444	0.9981504735470508
**O=C(NCCCO)c1ccc(CSc2nc3ccccc3c(=O)[nH]2)cc1**	ASINEX	O=C(NCCCO)c1ccc(CSc2nc3ccccc3c(=O)[nH]2)cc1	0.9953242	0.9990787872950698	0.999998059546825	0.9981336804244
**O=C(Cc1ccc(O)cc1)N1CCc2c(nc(N3CCOCC3)[nH]c2=O)C1**	ASINEX	O=C(Cc1ccc(O)cc1)N1CCc2c(nc(N3CCOCC3)[nH]c2=O)C1	0.99970144	0.999671205200214	0.9950108822667734	0.998127842600436
**O=C(Nc1ccccc1C(F)(F)F)N1CCc2nc(N3CCOCC3)[nH]c(=O)c2C1**	ASINEX	O=C(Nc1ccccc1C(F)(F)F)N1CCc2nc(N3CCOCC3)[nH]c(=O)c2C1	0.9998202	0.9980876239674386	0.9961067687301992	0.99800485516145
**CCn1cc(CN2CCc3c(nc(-c4ccncc4)[nH]c3=O)C2)c2cc(OC)ccc21**	ASINEX	CCn1cc(CN2CCc3c(nc(-c4ccncc4)[nH]c3=O)C2)c2cc(OC)ccc21	0.9974796	0.9999989853557808	0.996446270105719	0.997974957685724
**O=c1[nH]c(-c2ccncc2)nc2c1CCN(Cc1ccc(OC(F)(F)F)cc1)C2**	ASINEX	O=c1[nH]c(-c2ccncc2)nc2c1CCN(Cc1ccc(OC(F)(F)F)cc1)C2	0.9990295	0.9999721788649224	0.9948178915455916	0.9979398625280936
**O=C(CN1CCc2nc(N3CCCCC3)[nH]c(=O)c2C1)Nc1ccc(Oc2ccccc2)cc1**	ASINEX	O=C(CN1CCc2nc(N3CCCCC3)[nH]c(=O)c2C1)Nc1ccc(Oc2ccccc2)cc1	0.9992687	0.9983292908346816	0.996160059365166	0.997919353604366
**O=C(CCCc1nc2ccccc2c(=O)[nH]1)NCc1ccc(F)cc1**	ASINEX	O=C(CCCc1nc2ccccc2c(=O)[nH]1)NCc1ccc(F)cc1	0.99646026	0.9972848067393132	0.999999987359634	0.997915017686557
**O=C(c1cccc(Cl)c1)N(Cc1ccc(Cl)cc1)Cc1nc2ccccc2c(=O)[nH]1**	ASINEX	O=C(c1cccc(Cl)c1)N(Cc1ccc(Cl)cc1)Cc1nc2ccccc2c(=O)[nH]1	0.99428564	0.9993835953020616	0.9999977047106564	0.997888981037819
**CN(C)C(=O)c1ccc(-c2n[nH]c(=O)c3ccccc23)cc1**	ASINEX	CN(C)C(=O)c1ccc(-c2n[nH]c(=O)c3ccccc23)cc1	0.9989416	0.994634119028885	0.9999858511130156	0.9978538568215414
**CCOc1ccc(CN2CCc3c(nc(-c4ccncc4)[nH]c3=O)C2)cc1**	ASINEX	CCOc1ccc(CN2CCc3c(nc(-c4ccncc4)[nH]c3=O)C2)cc1	0.99344206	0.9999722526910352	0.9999957882652452	0.997803366506504
**COc1ccc(CN2CCc3c(nc(-c4ccccn4)[nH]c3=O)C2)cc1**	ASINEX	COc1ccc(CN2CCc3c(nc(-c4ccccn4)[nH]c3=O)C2)cc1	0.9981401	0.9999720541279984	0.9952821881681352	0.997798112986854
**O=c1[nH]c(-c2ccccc2)nc2c1oc1ccccc12**	ASINEX	O=c1[nH]c(-c2ccccc2)nc2c1oc1ccccc12	0.9997873	0.9935778820751804	0.9999858274792432	0.9977836601924066
**O=c1[nH]c(-c2ccncc2)nc2c1CCN(Cc1cccs1)C2**	ASINEX	O=c1[nH]c(-c2ccncc2)nc2c1CCN(Cc1cccs1)C2	0.9991468	0.9998938469332954	0.9943064037434308	0.9977823565971372
**O=c1[nH]c(-c2ccncc2)nc2c1CCN(Cc1ccccc1Cl)C2**	ASINEX	O=c1[nH]c(-c2ccncc2)nc2c1CCN(Cc1ccccc1Cl)C2	0.9984357	0.9999814137720512	0.9949214466087412	0.9977795121595542
**CO[C@H]1CC[C@H](C(=O)N2CCc3nc(N4CCCCC4)[nH]c(=O)c3C2)CC1**	ASINEX	CO[C@H]1CC[C@H](C(=O)N2CCc3nc(N4CCCCC4)[nH]c(=O)c3C2)CC1	0.99722254	0.9990798055484214	0.9969934446290408	0.9977652643134062

**CC1CN(c2nc3c(c(=O)[nH]2)CCN(Cc2cccc4cccnc24)C3)CC(C)O1**	ASINEX	CC1CN(c2nc3c(c(=O)[nH]2)CCN(Cc2cccc4cccnc24)C3)CC(C)O1	0.99927133	0.9999996805837276	0.9940114263696678	0.9977608133903386
**O=c1[nH]c(-c2ccccn2)nc2c1CCN(Cc1ccc(-c3ccccc3)cc1)C2**	ASINEX	O=c1[nH]c(-c2ccccn2)nc2c1CCN(Cc1ccc(-c3ccccc3)cc1)C2	0.9990382	0.9999809302856064	0.9942526010522512	0.997757250263254
**O=C(O)c1ccc(-c2ccccc2)[nH]c1=O**	ASINEX	O=C(O)c1ccc(-c2ccccc2)[nH]c1=O	0.99814415	0.9950954875245248	0.9999996435182128	0.9977464269410036
**O=C(c1cccc(F)c1)N(Cc1ccc(F)cc1)Cc1nc2ccccc2c(=O)[nH]1**	ASINEX	O=C(c1cccc(F)c1)N(Cc1ccc(F)cc1)Cc1nc2ccccc2c(=O)[nH]1	0.9967625	0.9990239329930573	0.9974450909791572	0.9977438460288648
**O=c1[nH]c(-c2ccccn2)nc2c1CCN(Cc1cccc3cccnc13)C2**	ASINEX	O=c1[nH]c(-c2ccccn2)nc2c1CCN(Cc1cccc3cccnc13)C2	0.9995956	0.9999994911785592	0.9936250709281088	0.997740048197289
**O=C(c1ccc2c(c1)OCO2)N1CCc2nc(-c3ccccc3)[nH]c(=O)c2C1**	ASINEX	O=C(c1ccc2c(c1)OCO2)N1CCc2nc(-c3ccccc3)[nH]c(=O)c2C1	0.9992887	0.9939225803994044	0.999990384230513	0.997733880933056
**CC1CCN(CC(=O)N2CCc3c(nc(N4CCOCC4)[nH]c3=O)C2)CC1**	ASINEX	CC1CCN(CC(=O)N2CCc3c(nc(N4CCOCC4)[nH]c3=O)C2)CC1	0.99817824	0.9999269666629772	0.9950529623520226	0.9977193908840284
**O=c1[nH]c(-c2ccncc2)nc2c1CCN(Cc1ccccc1O)C2**	ASINEX	O=c1[nH]c(-c2ccncc2)nc2c1CCN(Cc1ccccc1O)C2	0.9995172	0.9999876661814966	0.9935877119668444	0.9976975268418868
**COc1ccc(NC(=S)N(Cc2ccccc2)Cc2nc3ccccc3c(=O)[nH]2)cc1**	ASINEX	COc1ccc(NC(=S)N(Cc2ccccc2)Cc2nc3ccccc3c(=O)[nH]2)cc1	0.99329823	0.9996486485260052	0.9999974166840914	0.9976480992551618
**NC(=O)c1ccccc1NC(=O)CSc1nc2c(c(=O)[nH]1)CCCC2**	ASINEX	NC(=O)c1ccccc1NC(=O)CSc1nc2c(c(=O)[nH]1)CCCC2	0.99349844	0.9994454887805048	0.9999999910843852	0.99764797480736
**O=c1[nH]c(-c2ccccn2)nc2c1CCNC2**	ASINEX	O=c1[nH]c(-c2ccccn2)nc2c1CCNC2	0.9996675	0.9988967789692286	0.9941977548770738	0.9975873530459152
**O=C(c1ccc2c(c1)OCO2)N1CCc2nc(N3CCOCC3)[nH]c(=O)c2C1**	ASINEX	O=C(c1ccc2c(c1)OCO2)N1CCc2nc(N3CCOCC3)[nH]c(=O)c2C1	0.9995802	0.9957374202026552	0.9974353048419774	0.9975843098438264
**O=C(Cc1n[nH]c(=O)c2ccccc12)N1CCC(c2[nH]ncc2-c2ccncc2)CC1**	ASINEX	O=C(Cc1n[nH]c(=O)c2ccccc12)N1CCC(c2[nH]ncc2-c2ccncc2)CC1	0.99816304	0.9995711327504772	0.995015225489454	0.997583134230866
**O=C(C1CCCC1)N1CCc2nc(-c3ccccc3)[nH]c(=O)c2C1**	ASINEX	O=C(C1CCCC1)N1CCc2nc(-c3ccccc3)[nH]c(=O)c2C1	0.99441564	0.9981985665593984	0.9999981790221638	0.9975374621375188
**COc1ccc(CN(Cc2nc3ccccc3c(=O)[nH]2)C(=O)c2cccc(C)c2)cc1**	ASINEX	COc1ccc(CN(Cc2nc3ccccc3c(=O)[nH]2)C(=O)c2cccc(C)c2)cc1	0.9926725	0.9999306993778068	0.999999881820015	0.9975343613974532
**O=c1[nH]c(-c2ccncc2)nc2c1CCN(Cc1ccnc3ccccc13)C2**	ASINEX	O=c1[nH]c(-c2ccncc2)nc2c1CCN(Cc1ccnc3ccccc13)C2	0.9995153	0.9999579714313552	0.9929443439492954	0.9974725368031124
**COc1ccccc1CN(Cc1nc2ccccc2c(=O)[nH]1)C(=O)c1ccccc1C**	ASINEX	COc1ccccc1CN(Cc1nc2ccccc2c(=O)[nH]1)C(=O)c1ccccc1C	0.99264514	0.9997488714151396	0.9999968858557862	0.9974636339111704
**O=c1[nH]c(N2CCOCC2)nc2c1CCN(Cc1ccccc1F)C2**	ASINEX	O=c1[nH]c(N2CCOCC2)nc2c1CCN(Cc1ccccc1F)C2	0.99990475	0.9999528270088982	0.9925153597165788	0.9974576461677086
**O=c1[nH]c(N2CCOCC2)nc2c1CCN(CC1CCCCC1)C2**	ASINEX	O=c1[nH]c(N2CCOCC2)nc2c1CCN(CC1CCCCC1)C2	0.9991296	0.9999361638005124	0.9929198553044908	0.9973285374924497
**CCc1ccc(C(=O)N2CCc3nc(N4CCCC4)[nH]c(=O)c3C2)cc1**	ASINEX	CCc1ccc(C(=O)N2CCc3nc(N4CCCC4)[nH]c(=O)c3C2)cc1	0.9972324	0.9946838840154356	0.9999991628284892	0.9973051414576896
**CC1CCN(c2nc3c(c(=O)[nH]2)CN(Cc2cccc4cccnc24)CC3)CC1**	ASINEX	CC1CCN(c2nc3c(c(=O)[nH]2)CN(Cc2cccc4cccnc24)CC3)CC1	0.99948776	0.9999959487009558	0.991988163949705	0.9971572901111536
**O=C(c1nccc2ccccc12)N1CCc2c(nc(N3CCCC3)[nH]c2=O)C1**	ASINEX	O=C(c1nccc2ccccc12)N1CCc2c(nc(N3CCCC3)[nH]c2=O)C1	0.9994973	0.999837351953936	0.992062145570241	0.9971322639833804
**O=c1[nH]c(N2CCCCC2)nc2c1CCN(Cc1ccccc1F)C2**	ASINEX	O=c1[nH]c(N2CCCCC2)nc2c1CCN(Cc1ccccc1F)C2	0.999506	0.9999042732405904	0.9919012659336618	0.9971038452927846
**O=C(CCc1ccncc1)N1CCN(Cc2nc3ccccc3c(=O)[nH]2)CC1**	ASINEX	O=C(CCc1ccncc1)N1CCN(Cc2nc3ccccc3c(=O)[nH]2)CC1	0.99591607	0.9994225270964155	0.9958153469423784	0.9970513141975728
**O=c1[nH]c(N2CCOCC2)nc2c1CCN(Cc1cccs1)C2**	ASINEX	O=c1[nH]c(N2CCOCC2)nc2c1CCN(Cc1cccs1)C2	0.9997434	0.999809478606351	0.991335554472062	0.9969628116942184
**O=C(C1CCCO1)N1CCc2nc(-c3ccccc3)[nH]c(=O)c2C1**	ASINEX	O=C(C1CCCO1)N1CCc2nc(-c3ccccc3)[nH]c(=O)c2C1	0.9967776	0.9975322607033708	0.9965010445829886	0.9969369664586226
**O=c1[nH]nc(CCc2n[nH]c(=O)c3ccccc23)c2ccccc12**	ASINEX	O=c1[nH]nc(CCc2n[nH]c(=O)c3ccccc23)c2ccccc12	0.99856347	0.992247930738089	0.999998871837692	0.9969367570106832
**CN(C)c1ccc(C(=O)N2CCc3c(nc(N4CCCC4)[nH]c3=O)C2)cc1**	ASINEX	CN(C)c1ccc(C(=O)N2CCc3c(nc(N4CCCC4)[nH]c3=O)C2)cc1	0.99924076	0.9981156023844422	0.9934383164782296	0.9969315582991743
**O=c1[nH]c(N2CCCC2)nc2c1CCN(Cc1ccnc3ccccc13)C2**	ASINEX	O=c1[nH]c(N2CCCC2)nc2c1CCN(Cc1ccnc3ccccc13)C2	0.99919754	0.9998809199315756	0.9917014689143516	0.996926643837772
**O=c1[nH]nc(Cc2cc(O)n3ncc(-c4ccc(F)cc4)c3n2)c2ccccc12**	ASINEX	O=c1[nH]nc(Cc2cc(O)n3ncc(-c4ccc(F)cc4)c3n2)c2ccccc12	0.9928336	0.997819056045342	0.9999971379666578	0.9968832694537882
**COc1cccc(C(=O)N(Cc2ccc(Cl)cc2)Cc2nc3ccccc3c(=O)[nH]2)c1**	ASINEX	COc1cccc(C(=O)N(Cc2ccc(Cl)cc2)Cc2nc3ccccc3c(=O)[nH]2)c1	0.9907808	0.9998311012136236	0.9999998867738968	0.9968705862553922
**CC1CN(c2nc3c(c(=O)[nH]2)CCN(C(=O)CCc2ccccc2)C3)CC(C)O1**	ASINEX	CC1CN(c2nc3c(c(=O)[nH]2)CCN(C(=O)CCc2ccccc2)C3)CC(C)O1	0.99786925	0.9999120527762134	0.9927551842795784	0.9968454967381204
**O=C(c1cnccn1)N1CCc2c(nc(N3CCCCC3)[nH]c2=O)C1**	ASINEX	O=C(c1cnccn1)N1CCc2c(nc(N3CCCCC3)[nH]c2=O)C1	0.99918824	0.9996839267710989	0.9913735038616688	0.9967485583251904

**O=C(c1nccc2ccccc12)N1CCc2c(nc(N3CCOCC3)[nH]c2=O)C1**	ASINEX	O=C(c1nccc2ccccc12)N1CCc2c(nc(N3CCOCC3)[nH]c2=O)C1	0.9998691	0.9999053461688076	0.990458023932125	0.9967441594336686
**O=c1cc(-c2ccccc2)nc(N2CCN(c3ccccc3F)CC2)[nH]1**	ASINEX	O=c1cc(-c2ccccc2)nc(N2CCN(c3ccccc3F)CC2)[nH]1	0.990747	0.9994032583362478	0.999999996861928	0.9967167433810814
**O=c1[nH]c(-c2ccncc2)nc2c1CCN(Cc1cnc(N3CCOCC3)nc1)C2**	ASINEX	O=c1[nH]c(-c2ccncc2)nc2c1CCN(Cc1cnc(N3CCOCC3)nc1)C2	0.9994869	0.9999893766558692	0.990617159174384	0.9966978196826756
**Cc1cccc(C(=O)N(Cc2ccc(F)cc2)Cc2nc3ccccc3c(=O)[nH]2)c1**	ASINEX	Cc1cccc(C(=O)N(Cc2ccc(F)cc2)Cc2nc3ccccc3c(=O)[nH]2)c1	0.9911664	0.9987599898611484	0.9999950058720688	0.99664046952119
**O=c1[nH]c(-c2ccncc2)nc2c1CCN(Cc1cccc(F)c1)C2**	ASINEX	O=c1[nH]c(-c2ccncc2)nc2c1CCN(Cc1cccc(F)c1)C2	0.9993888	0.9998393447801208	0.9905925466850424	0.9966069018125456
**COc1ccccc1CN(Cc1nc2ccccc2c(=O)[nH]1)C(=O)c1cccc(Cl)c1**	ASINEX	COc1ccccc1CN(Cc1nc2ccccc2c(=O)[nH]1)C(=O)c1cccc(Cl)c1	0.9906923	0.9990831905909954	0.9999968798153492	0.9965907959640514
**O=c1[nH]c(CSc2nnc(-c3ccncc3)n2-c2cccc(Cl)c2)nc2ccccc12**	ASINEX	O=c1[nH]c(CSc2nnc(-c3ccncc3)n2-c2cccc(Cl)c2)nc2ccccc12	0.9896974	0.9999991749054198	0.999999999691906	0.9965655237841814
**O=C(Cc1ccc(Cl)cc1)N1CCc2c(nc(N3CCOCC3)[nH]c2=O)C1**	ASINEX	O=C(Cc1ccc(Cl)cc1)N1CCc2c(nc(N3CCOCC3)[nH]c2=O)C1	0.9994686	0.9987623039137508	0.9914006986175316	0.9965438757077032
**COc1ccccc1CN(Cc1nc2ccccc2c(=O)[nH]1)C(=O)c1cccc(F)c1**	ASINEX	COc1ccccc1CN(Cc1nc2ccccc2c(=O)[nH]1)C(=O)c1cccc(F)c1	0.99460113	0.9987614235186076	0.9959664992107284	0.9964430177382902
**CCn1c(SCc2nc3ccccc3c(=O)[nH]2)nnc1-c1ccc(F)cc1**	ASINEX	CCn1c(SCc2nc3ccccc3c(=O)[nH]2)nnc1-c1ccc(F)cc1	0.9896644	0.9996197923007406	0.9999938956395144	0.996426021240756
**COc1cccc(CN2CCc3c(nc(-c4ccncc4)[nH]c3=O)C2)c1**	ASINEX	COc1cccc(CN2CCc3c(nc(-c4ccncc4)[nH]c3=O)C2)c1	0.999084	0.9998859533829344	0.990303206546629	0.9964243852495516
**COc1ccc(CN(Cc2nc3ccccc3c(=O)[nH]2)C(=O)c2ccc([N+](=O)[O-])cc2)cc1**	ASINEX	COc1ccc(CN(Cc2nc3ccccc3c(=O)[nH]2)C(=O)c2ccc([N+](=O)[O-])cc2)cc1	0.9892273	0.9999782894298505	0.9999999807951132	0.9964018550489464
**O=c1[nH]c(-c2ccncc2)nc2c1CCN(Cc1cccc(Oc3ccccc3)c1)C2**	ASINEX	O=c1[nH]c(-c2ccncc2)nc2c1CCN(Cc1cccc(Oc3ccccc3)c1)C2	0.99957937	0.9999778777050315	0.9895439734032524	0.9963670737100347
**OC(=O)c1ccc([nH]c1=O)-c1ccc(Cl)cc1**	MOLPORT	O=C(O)c1ccc(-c2ccc(Cl)cc2)[nH]c1=O	0.99482036	0.9942083169154676	0.9999949040767454	0.996341192453744
**CC1CCN(c2nc3c(c(=O)[nH]2)CCN(Cc2cccc4nccnc24)C3)CC1**	ASINEX	CC1CCN(c2nc3c(c(=O)[nH]2)CCN(Cc2cccc4nccnc24)C3)CC1	0.9994394	0.9999646721906448	0.9895447973735916	0.9963162959600412
**CC1CN(c2nc3c(c(=O)[nH]2)CCN(Cc2ccc(-c4ccccc4)cc2)C3)CC(C)O1**	ASINEX	CC1CN(c2nc3c(c(=O)[nH]2)CCN(Cc2ccc(-c4ccccc4)cc2)C3)CC(C)O1	0.9977717	0.9999396342767072	0.9911590297853112	0.9962901148056968
**O=C(NCc1nc2ccccc2c(=O)[nH]1)C1COCCO1**	MOLPORT	O=C(NCc1nc2ccccc2c(=O)[nH]1)C1COCCO1	0.9924138	0.9990114538619788	0.9973583932312872	0.9962612219764928
**COc1ccc(O)c(CN2CCc3c(nc(-c4ccncc4)[nH]c3=O)C2)c1**	ASINEX	COc1ccc(O)c(CN2CCc3c(nc(-c4ccncc4)[nH]c3=O)C2)c1	0.9987532	0.9999911231439296	0.9900086658407862	0.9962509930084348
**COc1ccc(C(=O)N(Cc2nc3ccccc3c(=O)[nH]2)C2CCCCC2)cc1**	ASINEX	COc1ccc(C(=O)N(Cc2nc3ccccc3c(=O)[nH]2)C2CCCCC2)cc1	0.9887454	0.9998539056521192	0.9999995361197912	0.9961996110469254
**COc1ccc(-c2cc(=O)[nH]c(N3CCN(c4ccccc4F)CC3)n2)cc1**	ASINEX	COc1ccc(-c2cc(=O)[nH]c(N3CCN(c4ccccc4F)CC3)n2)cc1	0.98847383	0.9999787447957234	0.9999999999273784	0.9961508591101236
**O=c1[nH]c(-c2ccncc2)nc2c1CCN(Cc1cccc(Cl)c1)C2**	ASINEX	O=c1[nH]c(-c2ccncc2)nc2c1CCN(Cc1cccc(Cl)c1)C2	0.9982351	0.9996706408187264	0.9904519768873884	0.9961192413914384
**COc1cccc(CN2CCc3c(nc(-c4ccncc4)[nH]c3=O)C2)c1OC**	ASINEX	COc1cccc(CN2CCc3c(nc(-c4ccncc4)[nH]c3=O)C2)c1OC	0.99940443	0.9999763313144616	0.9889384832195992	0.9961064149744884
**Cn1c(-c2ccccc2)cnc1SCc1nc2ccccc2c(=O)[nH]1**	ASINEX	Cn1c(-c2ccccc2)cnc1SCc1nc2ccccc2c(=O)[nH]1	0.9884376	0.999786174205666	0.9999931219654457	0.9960722963847858
**COc1ccc(CN2CCc3c(nc(-c4ccncc4)[nH]c3=O)C2)c(OC)c1**	ASINEX	COc1ccc(CN2CCc3c(nc(-c4ccncc4)[nH]c3=O)C2)c(OC)c1	0.9990727	0.9999800953720364	0.9889773166517052	0.996010027653442
**O=c1[nH]c(-c2ccncc2)nc2c1CCN(Cc1ccccc1N1CCCC1)C2**	ASINEX	O=c1[nH]c(-c2ccncc2)nc2c1CCN(Cc1ccccc1N1CCCC1)C2	0.99784493	0.9997086883758816	0.990446532093876	0.9960000516444196
**O=c1[nH]c(-c2ccccn2)nc2c1CCN(Cc1ccc(C(F)(F)F)cc1)C2**	ASINEX	O=c1[nH]c(-c2ccccn2)nc2c1CCN(Cc1ccc(C(F)(F)F)cc1)C2	0.99830353	0.9999054174099404	0.9893760083593864	0.99586165278991
**O=C(NCCO)c1ccc(CSc2nc3ccccc3c(=O)[nH]2)cc1**	ASINEX	O=C(NCCO)c1ccc(CSc2nc3ccccc3c(=O)[nH]2)cc1	0.9953242	0.99961974824255	0.9925080785232538	0.9958173403990362
**O=C(C1CCCO1)N1CCc2nc(N3CCCC3)[nH]c(=O)c2C1**	ASINEX	O=C(C1CCCO1)N1CCc2nc(N3CCCC3)[nH]c(=O)c2C1	0.9983614	0.9955111326360242	0.9935633031830916	0.9958119481765316
**O=c1[nH]nc(Cc2cc(O)n3ncc(-c4ccccc4)c3n2)c2ccccc12**	ASINEX	O=c1[nH]nc(Cc2cc(O)n3ncc(-c4ccccc4)c3n2)c2ccccc12	0.99271214	0.9946910012517716	0.999994836091136	0.9957993258087402
**Cc1ccc(C(C)C(=O)N2CCc3c(nc(N4CCOCC4)[nH]c3=O)C2)cc1**	ASINEX	Cc1ccc(C(C)C(=O)N2CCc3c(nc(N4CCOCC4)[nH]c3=O)C2)cc1	0.99169695	0.9996718014893428	0.995952266460604	0.9957736739078152
**COc1ccc(C(=O)N(CCc2ccc(OC)c(OC)c**	ASIN	COc1ccc(C(=O)N(CCc2ccc(OC)c(OC)c2	0.99872	0.9997264	0.9887803	0.9957425

**2)Cc2nc3ccccc3c(=O)[nH]2)cc1**	EX	)Cc2nc3ccccc3c(=O)[nH]2)cc1	094	181077824	418760528	679705332
**O=c1[nH]c(N2CCCCC2)nc2c1CCN(Cc1ccccc1O)C2**	ASINEX	O=c1[nH]c(N2CCCCC2)nc2c1CCN(Cc1ccccc1O)C2	0.9992865	0.9999265891927746	0.9880029834480244	0.995738681812731
**CN1CCC(CCNC(=O)C2CCc3nc(-c4ccc(F)cc4)[nH]c(=O)c3CC2)CC1**	MOLPORT	CN1CCC(CCNC(=O)C2CCc3nc(-c4ccc(F)cc4)[nH]c(=O)c3CC2)CC1	0.9980107	0.9992486277220436	0.9899223828429504	0.9957272351818718
**COc1ccc(CN(Cc2cc3c(C)cc(C)cc3[nH]c2=O)C(=O)c2ccc(F)cc2)cc1**	ASINEX	COc1ccc(CN(Cc2cc3c(C)cc(C)cc3[nH]c2=O)C(=O)c2ccc(F)cc2)cc1	0.99709773	0.9958138895462832	0.994221490090237	0.9957110367577056
**O=c1[nH]c(-c2ccncc2)nc2c1CCN(Cc1cnc(N3CCCCC3)nc1)C2**	ASINEX	O=c1[nH]c(-c2ccncc2)nc2c1CCN(Cc1cnc(N3CCCCC3)nc1)C2	0.9972065	0.9999784415500498	0.9898098757165504	0.9956649421266374
**O=c1[nH]c(-c2ccccc2)nc2c1CN(Cc1ccc(F)cc1)CC2**	ASINEX	O=c1[nH]c(-c2ccccc2)nc2c1CN(Cc1ccc(F)cc1)CC2	0.9995161	0.9944586415318056	0.9929953716022704	0.9956567142092632
**CN(C)c1cccc(C(=O)N2CCc3c(nc(N4CCCC4)[nH]c3=O)C2)c1**	ASINEX	CN(C)c1cccc(C(=O)N2CCc3c(nc(N4CCCC4)[nH]c3=O)C2)c1	0.999355	0.9987837602303892	0.9888130302307792	0.9956506028666846
**CN1CCC(CCNC(=O)C2CCc3nc(-c4ccccc4)[nH]c(=O)c3CC2)CC1**	MOLPORT	CN1CCC(CCNC(=O)C2CCc3nc(-c4ccccc4)[nH]c(=O)c3CC2)CC1	0.9975769	0.9987513936017316	0.9901660738524722	0.9954981199433832
**O=C(C1CCCO1)N1CCc2nc(N3CCOCC3)[nH]c(=O)c2C1**	ASINEX	O=C(C1CCCO1)N1CCc2nc(N3CCOCC3)[nH]c(=O)c2C1	0.9987921	0.9963020774456264	0.9913111271926758	0.9954684388373096
**O=C1COc2ccc(C(=O)C3CCN(C(=O)Cc4n[nH]c(=O)c5ccccc45)CC3)cc2N1**	ASINEX	O=C1COc2ccc(C(=O)C3CCN(C(=O)Cc4n[nH]c(=O)c5ccccc45)CC3)cc2N1	0.9982035	0.9997300748417528	0.988389574203707	0.9954410550173098
**CC(=O)c1cccc(NC(=O)N2CCc3nc(N4CCOCC4)[nH]c(=O)c3C2)c1**	ASINEX	CC(=O)c1cccc(NC(=O)N2CCc3nc(N4CCOCC4)[nH]c(=O)c3C2)c1	0.99938846	0.9975825653809052	0.9893280921787252	0.9954330379680784
**COc1ccc(CCCc2nnc(SCc3nc4ccccc4c(=O)[nH]3)n2C)cc1**	ASINEX	COc1ccc(CCCc2nnc(SCc3nc4ccccc4c(=O)[nH]3)n2C)cc1	0.9864066	0.9998627402997664	0.9999990429117934	0.9954228025095764
**CN(C)c1cccc(C(=O)N2CCc3c(nc(N4CCOCC4)[nH]c3=O)C2)c1**	ASINEX	CN(C)c1cccc(C(=O)N2CCc3c(nc(N4CCOCC4)[nH]c3=O)C2)c1	0.9997149	0.9992918913416434	0.9869575375944718	0.9953214466400516
**COc1ccc(-c2ccc(C(=O)N3CCC(Oc4ccccc4F)CC3)c(=O)[nH]2)cc1**	ASINEX	COc1ccc(-c2ccc(C(=O)N3CCC(Oc4ccccc4F)CC3)c(=O)[nH]2)cc1	0.98973185	0.9999967577749858	0.9962354826955908	0.9953213629034918
**O=c1[nH]c(-c2ccccn2)nc2c1CCN(Cc1cccc3nccnc13)C2**	ASINEX	O=c1[nH]c(-c2ccccn2)nc2c1CCN(Cc1cccc3nccnc13)C2	0.9995653	0.9999682198772296	0.9863659469732352	0.9952998233920392
**COc1cnc(N2CCC3(CCc4c3nc(N3CCOCC3)[nH]c4=O)CC2)nc1**	MOLPORT	COc1cnc(N2CCC3(CCc4c3nc(N3CCOCC3)[nH]c4=O)CC2)nc1	0.9936847	0.999963962435754	0.9922316570822814	0.9952934428633828
**O=C(O)c1n[nH]c(=O)c2ccccc12**	ASINEX	O=C(O)c1n[nH]c(=O)c2ccccc12	0.9991406	0.9867492558687392	0.9999846093889948	0.9952914951631208
**COc1ccc(CNCc2cc3c(C)cc(C)cc3[nH]c2=O)cc1**	ASINEX	COc1ccc(CNCc2cc3c(C)cc(C)cc3[nH]c2=O)cc1	0.98603606	0.999625263769518	0.9999963351770654	0.995219220395728
**NC(=O)CSc1nnc(-c2ccccn2)n1-c1ccccc1Cl**	ASINEX	NC(=O)CSc1nnc(-c2ccccn2)n1-c1ccccc1Cl	0.9920169	0.9961405440061548	0.9973520370660628	0.9951698308596236
**COc1ccc(N(Cc2cccnc2)C(=O)c2ccc(-c3ccccc3)[nH]c2=O)cc1**	ASINEX	COc1ccc(N(Cc2cccnc2)C(=O)c2ccc(-c3ccccc3)[nH]c2=O)cc1	0.9946526	0.99976680194015	0.9909699684323294	0.9951297997570334
**O=c1[nH]c(-c2ccncc2)nc2c1CCN(Cc1ccccc1N1CCCCC1)C2**	ASINEX	O=c1[nH]c(-c2ccncc2)nc2c1CCN(Cc1ccccc1N1CCCCC1)C2	0.99784493	0.9996560021167692	0.9878523539928732	0.9951177635243812
**COc1ccc(-c2n[nH]cc2CN2CCc3c(nc(-c4ccncc4)[nH]c3=O)C2)cc1**	ASINEX	COc1ccc(-c2n[nH]cc2CN2CCc3c(nc(-c4ccncc4)[nH]c3=O)C2)cc1	0.9993926	0.9999541519980594	0.985840932307447	0.9950625709917484
**COc1ccc(OC)c(CN2CCc3c(nc(-c4ccccn4)[nH]c3=O)C2)c1**	ASINEX	COc1ccc(OC)c(CN2CCc3c(nc(-c4ccccn4)[nH]c3=O)C2)c1	0.99877936	0.9999804887239604	0.9864062395305612	0.9950553615780552
**CC1CCN(c2nc3c(c(=O)[nH]2)CCN(CC(C)c2ccccc2)C3)CC1**	ASINEX	CC1CCN(c2nc3c(c(=O)[nH]2)CCN(CC(C)c2ccccc2)C3)CC1	0.9978399	0.9993188420478464	0.98788936583515	0.9950160451854452
**COc1ccc(OC)c2[nH]c(=O)c(CNCc3ccccc3)cc12**	ASINEX	COc1ccc(OC)c2[nH]c(=O)c(CNCc3ccccc3)cc12	0.9921211	0.9995075772805492	0.9933439788979572	0.994990885534742
**CC(=O)c1cccc(NC(=O)N2CCc3nc(N4CCCC4)[nH]c(=O)c3C2)c1**	ASINEX	CC(=O)c1cccc(NC(=O)N2CCc3nc(N4CCCC4)[nH]c(=O)c3C2)c1	0.9984669	0.995852930829656	0.990538558337864	0.9949527993664172
**Cn1cc(CN2CCc3c(nc(-c4ccncc4)[nH]c3=O)C2)c2cc(F)ccc21**	ASINEX	Cn1cc(CN2CCc3c(nc(-c4ccncc4)[nH]c3=O)C2)c2cc(F)ccc21	0.9983359	0.9998796211288108	0.9865378487529782	0.9949177892681016
**O=c1[nH]c(-c2ccncc2)nc2c1CCN(Cc1ccc(F)cc1F)C2**	ASINEX	O=c1[nH]c(-c2ccncc2)nc2c1CCN(Cc1ccc(F)cc1F)C2	0.9992111	0.9999308443871316	0.9855766642035227	0.9949061938374693
**O=C(Cc1ccccc1F)N1CCc2c(nc(N3CCCC3)[nH]c2=O)C1**	ASINEX	O=C(Cc1ccccc1F)N1CCc2c(nc(N3CCCC3)[nH]c2=O)C1	0.99921644	0.9996601319689	0.985837615636311	0.994904728314998
**O=C(C1CCCO1)N1CCc2nc(N3CCCCC3)[nH]c(=O)c2C1**	ASINEX	O=C(C1CCCO1)N1CCc2nc(N3CCCCC3)[nH]c(=O)c2C1	0.9983614	0.9947033038873392	0.9915042194021336	0.9948563106666508
**COc1ccccc1CN(Cc1nc2ccccc2c(=O)[nH]1)C(=O)c1cccc(C)c1**	ASINEX	COc1ccccc1CN(Cc1nc2ccccc2c(=O)[nH]1)C(=O)c1cccc(C)c1	0.9860459	0.9983977713561571	0.9999878687519156	0.9948105123716872
**O=c1[nH]c(-c2ccncc2)nc2c1CCN(Cc1cccc(O)c1)C2**	ASINEX	O=c1[nH]c(-c2ccncc2)nc2c1CCN(Cc1cccc(O)c1)C2	0.99927396	0.9999010745241774	0.9851646728622784	0.9947799010694824

**O=C(c1cccc(F)c1)N(Cc1ccc(Cl)cc1)Cc1nc2ccccc2c(=O)[nH]1**	ASINEX	O=C(c1cccc(F)c1)N(Cc1ccc(Cl)cc1)Cc1nc2ccccc2c(=O)[nH]1	0.991876	0.9986222471876618	0.9937629536759838	0.9947537356633496
**CC(Sc1nc2ccccc2c(=O)[nH]1)C(=O)Nc1ccccc1C(N)=O**	ASINEX	CC(Sc1nc2ccccc2c(=O)[nH]1)C(=O)Nc1ccccc1C(N)=O	0.9839765	0.9999873743441388	0.9999999953323055	0.9946546176738252
**CCn1cc(C(=O)N2CCc3c(nc(N4CCCC4)[nH]c3=O)C2)cn1**	ASINEX	CCn1cc(C(=O)N2CCc3c(nc(N4CCCC4)[nH]c3=O)C2)cn1	0.98440045	0.9995458570668928	0.9999938571499856	0.994646721800066
**CCn1cc(C(=O)N2CCc3c(nc(N4CCCCC4)[nH]c3=O)C2)cn1**	ASINEX	CCn1cc(C(=O)N2CCc3c(nc(N4CCCCC4)[nH]c3=O)C2)cn1	0.98440045	0.9994637371364056	0.9999889385663512	0.9946177089620252
**O=c1[nH]c(-c2ccncc2)nc2c1CCN(Cc1ccc(Cl)cc1Cl)C2**	ASINEX	O=c1[nH]c(-c2ccncc2)nc2c1CCN(Cc1ccc(Cl)cc1Cl)C2	0.9986505	0.9999544372764174	0.9851224049941492	0.9945757778360692
**O=c1[nH]c(-c2ccccc2)nc2c1CN(Cc1ccccc1)CC2**	ASINEX	O=c1[nH]c(-c2ccccc2)nc2c1CN(Cc1ccccc1)CC2	0.99943274	0.9919226696407852	0.9923653498609312	0.9945735873657964
**O=C(CN1CCC(Nc2nc3c(c(=O)[nH]2)CCC3)CC1)Nc1ccccc1C(F)(F)F**	ASINEX	O=C(CN1CCC(Nc2nc3c(c(=O)[nH]2)CCC3)CC1)Nc1ccccc1C(F)(F)F	0.9940532	0.999588821236808	0.9900705493910223	0.9945708518713148
**O=c1[nH]c(-c2ccccn2)nc2c1CCN(Cc1ccccc1)C2**	ASINEX	O=c1[nH]c(-c2ccccn2)nc2c1CCN(Cc1ccccc1)C2	0.9983859	0.9995878927722516	0.9856425155521296	0.9945387715146212
**COc1ccc(-c2noc(CCCC(=O)Nc3ccc(C(N)=O)cc3)n2)cc1**	ASINEX	COc1ccc(-c2noc(CCCC(=O)Nc3ccc(C(N)=O)cc3)n2)cc1	0.98602736	0.9972420778937112	0.9999886506784024	0.9944193628448588
**O=C(O)Cc1n[nH]c(=O)c2ccccc12**	ASINEX	O=C(O)Cc1n[nH]c(=O)c2ccccc12	0.9988838	0.984332423714747	0.9999848804470324	0.99440036265969
**COc1ccc(OCc2nnc(SCc3nc4ccccc4c(=O)[nH]3)n2C)cc1**	ASINEX	COc1ccc(OCc2nnc(SCc3nc4ccccc4c(=O)[nH]3)n2C)cc1	0.9831292	0.9997616889857084	0.999998639134848	0.9942965104800352
**O=c1[nH]c(-c2ccncc2)nc2c1CCN(Cc1ccc3ncccc3c1)C2**	ASINEX	O=c1[nH]c(-c2ccncc2)nc2c1CCN(Cc1ccc3ncccc3c1)C2	0.9993081	0.9999645459914168	0.9834687843841536	0.9942471465530058
**CC1CN(c2nc3c(c(=O)[nH]2)CCN(Cc2cccc4nccnc24)C3)CC(C)O1**	ASINEX	CC1CN(c2nc3c(c(=O)[nH]2)CCN(Cc2cccc4nccnc24)C3)CC(C)O1	0.9992078	0.999942399525976	0.9835867634503772	0.994245652547548
**O=C(Cc1cccc(F)c1)N1CCc2c(nc(N3CCCC3)[nH]c2=O)C1**	ASINEX	O=C(Cc1cccc(F)c1)N1CCc2c(nc(N3CCCC3)[nH]c2=O)C1	0.9988444	0.9983337123746042	0.985383801858773	0.9941872997934906
**COc1cccc(OC)c1C(=O)N(Cc1ccc(F)cc1)Cc1nc2ccccc2c(=O)[nH]1**	ASINEX	COc1cccc(OC)c1C(=O)N(Cc1ccc(F)cc1)Cc1nc2ccccc2c(=O)[nH]1	0.9983785	0.99470721110185	0.9892523710715116	0.994112699138964
**CC(C)n1c(CNC(=O)c2ccccc2F)nnc1SCc1nc2ccccc2c(=O)[nH]1**	ASINEX	CC(C)n1c(CNC(=O)c2ccccc2F)nnc1SCc1nc2ccccc2c(=O)[nH]1	0.985832	0.9999777187185428	0.9964954595809022	0.9941017180787782
**O=C(Cc1cccc(F)c1)N1CCc2c(nc(N3CCOCC3)[nH]c2=O)C1**	ASINEX	O=C(Cc1cccc(F)c1)N1CCc2c(nc(N3CCOCC3)[nH]c2=O)C1	0.99940073	0.9990296856843162	0.9835587556996244	0.993996392095123
**Cc1ccc(C2(CNC(=O)c3ccc(-c4ccccc4)[nH]c3=O)CCOCC2)cc1**	ASINEX	Cc1ccc(C2(CNC(=O)c3ccc(-c4ccccc4)[nH]c3=O)CCOCC2)cc1	0.9819504	0.9999987328959534	0.9999999590955954	0.9939830314171252
**COc1ccc(CCN(Cc2nc3ccccc3c(=O)[nH]2)C(=O)c2ccccc2F)cc1OC**	ASINEX	COc1ccc(CCN(Cc2nc3ccccc3c(=O)[nH]2)C(=O)c2ccccc2F)cc1OC	0.99713147	0.9991287006506192	0.9856437099479808	0.9939679591547133
**Cc1ccccc1CN1CCc2c(nc(N3CCC(C)CC3)[nH]c2=O)C1**	ASINEX	Cc1ccccc1CN1CCc2c(nc(N3CCC(C)CC3)[nH]c2=O)C1	0.9939605	0.9998401934168484	0.9880248331107798	0.9939418420970392
**COc1ccc(-c2ccc(C(=O)N3CCC(Oc4ccc(F)cc4)CC3)c(=O)[nH]2)cc1**	ASINEX	COc1ccc(-c2ccc(C(=O)N3CCC(Oc4ccc(F)cc4)CC3)c(=O)[nH]2)cc1	0.98667127	0.9999865699826268	0.9950417582068488	0.9938998657098158
**O=c1[nH]c(NCCCO)nc2ccccc12**	ASINEX	O=c1[nH]c(NCCCO)nc2ccccc12	0.9954028	0.988510329254392	0.9974622002131682	0.9937917808084412
**CC(=O)c1cccc(NC(=O)N2CCc3nc(N4CCCCC4)[nH]c(=O)c3C2)c1**	ASINEX	CC(=O)c1cccc(NC(=O)N2CCc3nc(N4CCCCC4)[nH]c(=O)c3C2)c1	0.9984669	0.9951063106384668	0.9876581578373156	0.9937437924691714
**O=c1[nH]c(-c2ccccn2)nc2c1CCN(Cc1ccc(OCc3ccccc3F)cc1)C2**	ASINEX	O=c1[nH]c(-c2ccccn2)nc2c1CCN(Cc1ccc(OCc3ccccc3F)cc1)C2	0.9985815	0.999998605560569	0.9826460594317172	0.9937420645519738
**NC(=O)c1ccc(Oc2nccnc2C2CCN(CC3CC3)CC2)cc1**	ASINEX	NC(=O)c1ccc(Oc2nccnc2C2CCN(CC3CC3)CC2)cc1	0.99087256	0.9999540395814208	0.99020918322011	0.9936785949110468
**O=c1cc(-c2ccc(Cl)cc2)nc(N2CCN(c3ccccc3F)CC2)[nH]1**	ASINEX	O=c1cc(-c2ccc(Cl)cc2)nc(N2CCN(c3ccccc3F)CC2)[nH]1	0.9815052	0.999452732786254	0.9999999775690184	0.9936526418410906
**O=c1[nH]c(CCC2CCCC2)nc2ccccc12**	ASINEX	O=c1[nH]c(CCC2CCCC2)nc2ccccc12	0.98216265	0.9987425729151396	0.9999999862606463	0.993635071191886
**O=C(COc1ccc(CN2CCc3c(nc(-c4ccncc4)[nH]c3=O)C2)cc1)N1CCCC1**	ASINEX	O=C(COc1ccc(CN2CCc3c(nc(-c4ccncc4)[nH]c3=O)C2)cc1)N1CCCC1	0.9930393	0.9998903807836604	0.9879316959146702	0.9936204622256052
**O=C(Cn1nnc2ccccc21)N1CCc2c(nc(N3CCCC3)[nH]c2=O)C1**	ASINEX	O=C(Cn1nnc2ccccc21)N1CCc2c(nc(N3CCCC3)[nH]c2=O)C1	0.9987179	0.999750944006178	0.9822187328107416	0.9935625269692668
**COc1cccc(-c2ccc(C(N)=O)c(=O)[nH]2)c1**	ASINEX	COc1cccc(-c2ccc(C(N)=O)c(=O)[nH]2)c1	0.9970293	0.9893208097765694	0.9942720383847448	0.9935407175552364
**O=C(c1cc(C2CC2)nc2ccccc12)N1CCc2c(nc(N3CCOCC3)[nH]c2=O)C1**	ASINEX	O=C(c1cc(C2CC2)nc2ccccc12)N1CCc2c(nc(N3CCOCC3)[nH]c2=O)C1	0.9996474	0.9998273706743764	0.98098167420566	0.9934854746005152
**Cc1c(C(=O)N2CCc3c(nc(N4CCCC4)[nH]c3=O)C2)cnn1C**	ASINEX	Cc1c(C(=O)N2CCc3c(nc(N4CCCC4)[nH]c3=O)C2)cnn1C	0.99412256	0.9979504590225986	0.9881093736460482	0.9933941324870932

**O=C(CN1C[C@@H]2CC[C@H]1C2)N1CCc2c(nc(-c3ccncc3)[nH]c2=O)C1**	MOLPORT	O=C(CN1C[C@@H]2CC[C@H]1C2)N1CCc2c(nc(-c3ccncc3)[nH]c2=O)C1	0.9981521	0.9999952268864712	0.9819041290303094	0.9933504777049365
**O=C(c1n[nH]c2c1CCCC2)N1CCc2c(nc(N3CCCCC3)[nH]c2=O)C1**	ASINEX	O=C(c1n[nH]c2c1CCCC2)N1CCc2c(nc(N3CCCCC3)[nH]c2=O)C1	0.99963117	0.9987792357516216	0.9812521465356108	0.9932208495817876
**CC(C)CN1CCc2c(nc(N3CCC(C)CC3)[nH]c2=O)C1**	ASINEX	CC(C)CN1CCc2c(nc(N3CCC(C)CC3)[nH]c2=O)C1	0.9960757	0.999040415375024	0.9845005514827128	0.99320555221679
**O=C(Cc1cccc(Cl)c1)N1CCc2c(nc(N3CCOCC3)[nH]c2=O)C1**	ASINEX	O=C(Cc1cccc(Cl)c1)N1CCc2c(nc(N3CCOCC3)[nH]c2=O)C1	0.99874204	0.9980124492668532	0.9826604290728328	0.993138307437234
**Cc1ccc(CC(=O)N2CCc3c(nc(N4CCCC4)[nH]c3=O)C2)cc1**	ASINEX	Cc1ccc(CC(=O)N2CCc3c(nc(N4CCCC4)[nH]c3=O)C2)cc1	0.9871443	0.9977767093130052	0.9942758195285416	0.9930656067474852
**O=c1[nH]c(CSc2ncc(-c3ccc(F)cc3)n2Cc2ccco2)nc2ccccc12**	ASINEX	O=c1[nH]c(CSc2ncc(-c3ccc(F)cc3)n2Cc2ccco2)nc2ccccc12	0.98946124	0.9994075418121856	0.9899877339118548	0.9929521729588862
**COc1ccccc1CN(Cc1nc2ccccc2c(=O)[nH]1)C(=O)c1ccc([N+](=O)[O-])cc1**	ASINEX	COc1ccccc1CN(Cc1nc2ccccc2c(=O)[nH]1)C(=O)c1ccc([N+](=O)[O-])cc1	0.978876	0.9997729532734206	0.9999924532746236	0.9928804670767852
**CN(Cc1ccc(-c2nc3c(c(=O)[nH]2)CCCCC3)cc1)CC1CCOCC1**	MOLPORT	CN(Cc1ccc(-c2nc3c(c(=O)[nH]2)CCCCC3)cc1)CC1CCOCC1	0.9948801	0.993526595441064	0.990199224504682	0.9928686333896096
**O=c1[nH]c(-c2cccnc2)nc2c1CCN(Cc1cccc3cccnc13)C2**	ASINEX	O=c1[nH]c(-c2cccnc2)nc2c1CCN(Cc1cccc3cccnc13)C2	0.9994711	0.9999983313357784	0.9791319286814376	0.992867129334708
**Cn1c(Cc2ccccc2)nnc1SCc1nc2ccccc2c(=O)[nH]1**	ASINEX	Cn1c(Cc2ccccc2)nnc1SCc1nc2ccccc2c(=O)[nH]1	0.9813317	0.9972617250220956	0.9999962769744342	0.9928632360145292
**O=C(c1n[nH]c2c1CCC2)N1CCc2c(nc(N3CCCCC3)[nH]c2=O)C1**	ASINEX	O=C(c1n[nH]c2c1CCC2)N1CCc2c(nc(N3CCCCC3)[nH]c2=O)C1	0.99963117	0.9992015058085374	0.9796926008101716	0.9928417576922798
**COCCNC(=O)c1ccc(-c2ccccc2)[nH]c1=O**	ASINEX	COCCNC(=O)c1ccc(-c2ccccc2)[nH]c1=O	0.98213094	0.996308944842776	0.9999965799247243	0.992812156498784
**O=C(CN1CCc2nc(N3CCCC3)[nH]c(=O)c2C1)Nc1ccccc1F**	ASINEX	O=C(CN1CCc2nc(N3CCCC3)[nH]c(=O)c2C1)Nc1ccccc1F	0.9991493	0.99327986305622	0.9859907291137212	0.9928066382265688
**COc1ccccc1CN(Cc1nc2ccccc2c(=O)[nH]1)C(=O)C(C)(C)C**	ASINEX	COc1ccccc1CN(Cc1nc2ccccc2c(=O)[nH]1)C(=O)C(C)(C)C	0.9974045	0.9965216576945516	0.9841468531572334	0.9926910088650136
**O=C(N1CCc2nc(N3CCCC3)[nH]c(=O)c2C1)C1(c2ccccc2)CC1**	ASINEX	O=C(N1CCc2nc(N3CCCC3)[nH]c(=O)c2C1)C1(c2ccccc2)CC1	0.99961	0.9997372473141948	0.9786821786261972	0.9926764775832088
**COc1ccc(OC)c(N2CC(=O)C(c3nc4ccccc4c(=O)[nH]3)=C2N)c1**	ASINEX	COc1ccc(OC)c(N2CC(=O)C(c3nc4ccccc4c(=O)[nH]3)=C2N)c1	0.9892147	0.9993766601523528	0.9891693626654096	0.9925869137198634
**O=C(N1CCc2c(nc(N3CCCC3)[nH]c2=O)C1)C1(c2ccccc2)CC1**	ASINEX	O=C(N1CCc2c(nc(N3CCCC3)[nH]c2=O)C1)C1(c2ccccc2)CC1	0.99961	0.9999768105190832	0.9781719253212492	0.9925862475498558
**O=c1cc(-c2ccccc2)nc(SCc2nc(O)c3ccccc3n2)[nH]1**	ASINEX	O=c1cc(-c2ccccc2)nc(SCc2nc(O)c3ccccc3n2)[nH]1	0.99271303	0.9999762802042168	0.9847656060780522	0.992484973478418
**CCn1cc(C(=O)N2CCc3c(nc(N4CCC(C)CC4)[nH]c3=O)C2)cn1**	ASINEX	CCn1cc(C(=O)N2CCc3c(nc(N4CCC(C)CC4)[nH]c3=O)C2)cn1	0.98673314	0.9992709743013176	0.9909355963100344	0.992313236390867
**Cc1ccc(-c2cc(=O)[nH]c(N3CCN(c4ccccc4F)CC3)n2)cc1**	ASINEX	Cc1ccc(-c2cc(=O)[nH]c(N3CCN(c4ccccc4F)CC3)n2)cc1	0.97744787	0.999427389584863	0.999999990533903	0.9922917491707532
**CCOc1ccccc1CN1CCc2c(nc(-c3ccncc3)[nH]c2=O)C1**	ASINEX	CCOc1ccccc1CN1CCc2c(nc(-c3ccncc3)[nH]c2=O)C1	0.9954383	0.9998202425550902	0.981489137312144	0.992249219196213
**COc1ccc(OC)c(CN2CCc3c(nc(N4CC(C)OC(C)C4)[nH]c3=O)C2)c1**	ASINEX	COc1ccc(OC)c(CN2CCc3c(nc(N4CC(C)OC(C)C4)[nH]c3=O)C2)c1	0.997387	0.9999417466817176	0.979397328398245	0.9922420223525514
**CC(CCN1CCc2c(nc(N3CCOCC3)[nH]c2=O)C1)c1ccccc1**	ASINEX	CC(CCN1CCc2c(nc(N3CCOCC3)[nH]c2=O)C1)c1ccccc1	0.9993399	0.9993398390012476	0.978039650683179	0.992239789414513
**COc1ccc(CN(Cc2nc3ccccc3c(=O)[nH]2)C(=O)Nc2ccc(C)cc2)cc1**	ASINEX	COc1ccc(CN(Cc2nc3ccccc3c(=O)[nH]2)C(=O)Nc2ccc(C)cc2)cc1	0.98087466	0.9993198946472216	0.9963458659590988	0.9921801394124136
**COc1cccc(C(=O)N(Cc2ccc3c(c2)OCO3)Cc2nc3ccccc3c(=O)[nH]2)c1**	ASINEX	COc1cccc(C(=O)N(Cc2ccc3c(c2)OCO3)Cc2nc3ccccc3c(=O)[nH]2)c1	0.97706246	0.9993670732676008	0.9999989746557496	0.9921428372279087
**CC(=O)Nc1ccc(CN2CCc3c(nc(-c4ccncc4)[nH]c3=O)C2)cc1**	ASINEX	CC(=O)Nc1ccc(CN2CCc3c(nc(-c4ccncc4)[nH]c3=O)C2)cc1	0.9972047	0.999697502601631	0.9794413460717802	0.9921145232157932
**CN(C)c1ccc(C(=O)N2CCc3nc(-c4ccccc4)[nH]c(=O)c3C2)cc1**	ASINEX	CN(C)c1ccc(C(=O)N2CCc3nc(-c4ccccc4)[nH]c(=O)c3C2)cc1	0.9995708	0.9845184258401318	0.9922497491937922	0.9921129873289656
**CCOc1ccc(CN2CCc3c(nc(N4CC(C)OC(C)C4)[nH]c3=O)C2)cc1**	ASINEX	CCOc1ccc(CN2CCc3c(nc(N4CC(C)OC(C)C4)[nH]c3=O)C2)cc1	0.9952302	0.999633958100101	0.9812734525080804	0.9920458695048918
**O=C(Nc1ccc(Cl)c(C(F)(F)F)c1)N1CCc2nc(N3CCCC3)[nH]c(=O)c2C1**	ASINEX	O=C(Nc1ccc(Cl)c(C(F)(F)F)c1)N1CCc2nc(N3CCCC3)[nH]c(=O)c2C1	0.997707	0.999477626310876	0.9788100016343342	0.9919982124202336
**O=C(Cn1nnc2ccccc21)N1CCc2c(nc(N3CCCCC3)[nH]c2=O)C1**	ASINEX	O=C(Cn1nnc2ccccc21)N1CCc2c(nc(N3CCCCC3)[nH]c2=O)C1	0.9987179	0.9997058977996918	0.9775611858769268	0.9919949959225
**CC1CN(c2nc3c(c(=O)[nH]2)CCN(CC(C)c2ccccc2)C3)CC(C)O1**	ASINEX	CC1CN(c2nc3c(c(=O)[nH]2)CCN(CC(C)c2ccccc2)C3)CC(C)O1	0.99733907	0.9988898532817984	0.9795083684085344	0.9919124305112084
**C=CCn1c(SCc2nc3ccccc3c(=O)[nH]2)nnc1-c1ccc(F)cc1**	ASINEX	C=CCn1c(SCc2nc3ccccc3c(=O)[nH]2)nnc1-c1ccc(F)cc1	0.98289144	0.9999454139779064	0.9928837543789047	0.9919068695827838

**O=C(NCc1ccccc1)N(Cc1nc2ccccc2c(=O)[nH]1)C1CCCC1**	ASINEX	O=C(NCc1ccccc1)N(Cc1nc2ccccc2c(=O)[nH]1)C1CCCC1	0.9834398	0.9981593281783488	0.9940786081470412	0.991892579816288
**CC(C)CCN1CCc2nc(-c3ccccc3)[nH]c(=O)c2C1**	ASINEX	CC(C)CCN1CCc2nc(-c3ccccc3)[nH]c(=O)c2C1	0.99752563	0.9832835064572116	0.994832010031542	0.9918803829567308
**O=c1[nH]c2ccccc2c(N2CCOCC2)c1N1CCOCC1**	MOLPORT	O=c1[nH]c2ccccc2c(N2CCOCC2)c1N1CCOCC1	0.9950126	0.984998764027322	0.995612568642834	0.9918746380061916
**O=C(Nc1c[nH]c(=O)[nH]c1=O)c1ccc(-c2ccccc2)cc1**	ASINEX	O=C(Nc1c[nH]c(=O)[nH]c1=O)c1ccc(-c2ccccc2)cc1	0.9962268	0.9960750569662858	0.9833177698639176	0.991873204799114
**COc1ccc(CNCc2cc3c(C)ccc(C)c3[nH]c2=O)cc1**	ASINEX	COc1ccc(CNCc2cc3c(C)ccc(C)c3[nH]c2=O)cc1	0.983996	0.998849025344781	0.9926239182868484	0.9918229725651676
**O=c1[nH]c(N2CCCCCC2)nc2c1CN(Cc1ccccc1F)CC2**	ASINEX	O=c1[nH]c(N2CCCCCC2)nc2c1CN(Cc1ccccc1F)CC2	0.999506	0.9980240595512216	0.97780712279663	0.9917790596839844
**COc1ccc(CCN(Cc2nc3ccccc3c(=O)[nH]2)C(=O)c2cccc(Cl)c2)cc1**	ASINEX	COc1ccc(CCN(Cc2nc3ccccc3c(=O)[nH]2)C(=O)c2cccc(Cl)c2)cc1	0.9946578	0.9990103417636572	0.981585624160266	0.991751260142213
**COc1ccc(CN2CCc3c(nc(N4CC(C)OC(C)C4)[nH]c3=O)C2)c(O)c1**	ASINEX	COc1ccc(CN2CCc3c(nc(N4CC(C)OC(C)C4)[nH]c3=O)C2)c(O)c1	0.9973578	0.999933289082506	0.9779330380377244	0.9917413709406606
**O=c1[nH]c(CSc2nnc(-c3ccncc3)n2Cc2ccco2)nc2ccccc12**	ASINEX	O=c1[nH]c(CSc2nnc(-c3ccncc3)n2Cc2ccco2)nc2ccccc12	0.9903842	0.9999297821370636	0.9847590222488394	0.9916910084876228
**CC(C)n1cc(CN2CCc3c(nc(-c4ccncc4)[nH]c3=O)C2)c2ccccc21**	ASINEX	CC(C)n1cc(CN2CCc3c(nc(-c4ccncc4)[nH]c3=O)C2)c2ccccc21	0.9978029	0.9999327481052545	0.9770860172558206	0.9916072261833364
**Cc1c(CN2CCc3c(nc(-c4ccncc4)[nH]c3=O)C2)c2ccccc2n1C**	ASINEX	Cc1c(CN2CCc3c(nc(-c4ccncc4)[nH]c3=O)C2)c2ccccc2n1C	0.9975914	0.9998376998906904	0.9772293976249392	0.991552824606752
**O=C(N1CCc2c(nc(N3CCOCC3)[nH]c2=O)C1)C1(c2ccccc2)CC1**	ASINEX	O=C(N1CCc2c(nc(N3CCOCC3)[nH]c2=O)C1)C1(c2ccccc2)CC1	0.99991024	0.9999865055542624	0.9744354583139851	0.9914440664244054
**C=CCn1c(SCc2nc3ccccc3c(=O)[nH]2)nnc1-c1ccc(OC)cc1**	ASINEX	C=CCn1c(SCc2nc3ccccc3c(=O)[nH]2)nnc1-c1ccc(OC)cc1	0.9740475	0.9999952546993908	0.9999815154525648	0.9913414173885524
**COc1ccc(CN(Cc2nc3ccccc3c(=O)[nH]2)C(=O)Nc2ccccc2F)cc1**	ASINEX	COc1ccc(CN(Cc2nc3ccccc3c(=O)[nH]2)C(=O)Nc2ccccc2F)cc1	0.99506783	0.9995613447801216	0.9792825757463222	0.99130391846019
**Cc1nc(SCc2nc3ccccc3c(=O)[nH]2)nc2c1CCCC2**	ASINEX	Cc1nc(SCc2nc3ccccc3c(=O)[nH]2)nc2c1CCCC2	0.97651345	0.9972781257792762	0.9999810681482756	0.9912575464349672
**O=C(N1CCc2c(nc(N3CCOCC3)[nH]c2=O)C1)C1(c2ccc(Cl)cc2)CC1**	ASINEX	O=C(N1CCc2c(nc(N3CCOCC3)[nH]c2=O)C1)C1(c2ccc(Cl)cc2)CC1	0.9997286	0.9999862556352712	0.97386774489461	0.9911942068607394
**COc1cccc(CNC(=O)COCc2nc3ccccc3c(=O)[nH]2)c1**	ASINEX	COc1cccc(CNC(=O)COCc2nc3ccccc3c(=O)[nH]2)c1	0.99247146	0.999258309056492	0.981743232428092	0.991157666004098
**O=C(C1CC1)N1CCN(Cc2nc3ccccc3c(=O)[nH]2)CC1**	ASINEX	O=C(C1CC1)N1CCN(Cc2nc3ccccc3c(=O)[nH]2)CC1	0.99109143	0.9852715199481002	0.9969797294827124	0.991114226539346
**COc1ccc(-c2ccc(C(=O)N3CCc4[nH]c5c(C)cccc5c4C3)c(=O)[nH]2)cc1**	ASINEX	COc1ccc(-c2ccc(C(=O)N3CCc4[nH]c5c(C)cccc5c4C3)c(=O)[nH]2)cc1	0.99680966	0.999972739043146	0.9763308025152532	0.9910377343155986
**COc1cccc(CNCc2cc3c(C)cc(C)cc3[nH]c2=O)c1**	ASINEX	COc1cccc(CNCc2cc3c(C)cc(C)cc3[nH]c2=O)c1	0.98727304	0.9971567468714558	0.9886250774950852	0.9910182872667218
**O=c1[nH]c(N2CCOCC2)nc2c1CN(Cc1ccccc1Cl)CC2**	ASINEX	O=c1[nH]c(N2CCOCC2)nc2c1CN(Cc1ccccc1Cl)CC2	0.9996669	0.9988738510791196	0.9742207763046722	0.9909205188762624
**O=C1NCCc2c1[nH]c1ccc(OCc3ccccc3)cc21**	ASINEX	O=C1NCCc2c1[nH]c1ccc(OCc3ccccc3)cc21	0.9728095	0.9999046638585734	0.9999964075352858	0.9909035216451922
**O=C1NC2(CCN(C(=O)c3ccccc3F)CC2)Nc2ccccc21**	ASINEX	O=C1NC2(CCN(C(=O)c3ccccc3F)CC2)Nc2ccccc21	0.99890506	0.9999758037953012	0.9738187340843724	0.9908998668517164
**O=C(CN1CCc2nc(N3CCCC3)[nH]c(=O)c2C1)NCc1cccs1**	ASINEX	O=C(CN1CCc2nc(N3CCCC3)[nH]c(=O)c2C1)NCc1cccs1	0.9985189	0.989944317981664	0.9840200195229256	0.9908277405621888
**CC1CN(c2nc3c(c(=O)[nH]2)CCN(C(=O)C(C)C)C3)CC(C)O1**	ASINEX	CC1CN(c2nc3c(c(=O)[nH]2)CCN(C(=O)C(C)C)C3)CC(C)O1	0.9958021	0.9962608479259686	0.9802713959234084	0.990778116107497
**Cn1c(CN2CCOCC2)nnc1SCc1nc2ccccc2c(=O)[nH]1**	ASINEX	Cn1c(CN2CCOCC2)nnc1SCc1nc2ccccc2c(=O)[nH]1	0.97371936	0.9986035015052788	0.9999920757983568	0.9907716452492832
**O=C(CN1CCc2nc(N3CCCC3)[nH]c(=O)c2C1)NC1CC1**	ASINEX	O=C(CN1CCc2nc(N3CCCC3)[nH]c(=O)c2C1)NC1CC1	0.99804854	0.9823439605676682	0.991869812056594	0.9907541055181052
**O=C(CN1CCC(Nc2nc3c(c(=O)[nH]2)CCC3)CC1)NC(c1ccccc1)c1ccccc1**	ASINEX	O=C(CN1CCC(Nc2nc3c(c(=O)[nH]2)CCC3)CC1)NC(c1ccccc1)c1ccccc1	0.99224263	0.9977484601910488	0.982140882330685	0.9907106589393836
**O=c1[nH]c(N2CCOCC2)nc2c1CCC21CCN(CC2CC2)CC1**	MOLPORT	O=c1[nH]c(N2CCOCC2)nc2c1CCC21CCN(CC2CC2)CC1	0.99380374	0.9998408970710267	0.9784562214727712	0.9907002860305092
**O=C(c1ccccc1)N(Cc1nc2ccccc2c(=O)[nH]1)C1CCCC1**	ASINEX	O=C(c1ccccc1)N(Cc1nc2ccccc2c(=O)[nH]1)C1CCCC1	0.9787507	0.99618802739685	0.997034304984605	0.9906576793668164
**O=c1[nH]c(-c2ccccc2)nc2c1CN(C1CCCCC1)CC2**	ASINEX	O=c1[nH]c(-c2ccccc2)nc2c1CN(C1CCCCC1)CC2	0.99858093	0.9906800733226594	0.9825945195369816	0.9906185084922762
**CN(C)c1cccc(C(=O)N2CCc3nc(N4CCCC4)[nH]c(=O)c3C2)c1**	ASINEX	CN(C)c1cccc(C(=O)N2CCc3nc(N4CCCC4)[nH]c(=O)c3C2)c1	0.999355	0.9863869562251673	0.9861102758034886	0.9906174167225136
**CCn1cc(CN2CCc3c(nc(-c4ccccn4)[nH]c3=O)C2)c2cc(OC)ccc21**	ASINEX	CCn1cc(CN2CCc3c(nc(-c4ccccn4)[nH]c3=O)C2)c2cc(OC)ccc21	0.9973048	0.999995515465849	0.9745130686036814	0.9906044604140256

**O=C(CN1CCC(Nc2nc3c(c(=O)[nH]2)CCC3)CC1)NC(c1ccccc1)c1ccccc1**	ASINEX	O=C(CN1CCC(Nc2nc3c(c(=O)[nH]2)CCC3)CC1)NC(c1ccccc1)c1ccccc1	0.99224263	0.9985267560891192	0.981021413686517	0.9905969346906844
**Cc1ccc(-n2c(SCc3nc4ccccc4c(=O)[nH]3)nnc2-c2ccccc2O)cc1**	ASINEX	Cc1ccc(-n2c(SCc3nc4ccccc4c(=O)[nH]3)nnc2-c2ccccc2O)cc1	0.97478163	0.9999814576620606	0.9969633891547286	0.9905754930800632
**CN1CCCc2cc(CN3CCc4c(nc(-c5ccncc5)[nH]c4=O)C3)ccc21**	ASINEX	CN1CCCc2cc(CN3CCc4c(nc(-c5ccncc5)[nH]c4=O)C3)ccc21	0.99545753	0.9999541599847735	0.9762961620286535	0.9905692840116984
**COc1ccc(CN(Cc2nc3ccccc3c(=O)[nH]2)C(=O)C2CCC2)cc1**	ASINEX	COc1ccc(CN(Cc2nc3ccccc3c(=O)[nH]2)C(=O)C2CCC2)cc1	0.98197776	0.999946718457662	0.9897298682568876	0.990551449168776
**CCCNC(=O)N1CCc2nc(N3CCOCC3)[nH]c(=O)c2C1**	ASINEX	CCCNC(=O)N1CCc2nc(N3CCOCC3)[nH]c(=O)c2C1	0.9984484	0.9980629091729498	0.9751419267019862	0.9905510692540476
**O=C(N1CCc2nc(N3CCCC3)[nH]c(=O)c2C1)C1(c2ccc(Cl)cc2)CCC1**	ASINEX	O=C(N1CCc2nc(N3CCCC3)[nH]c(=O)c2C1)C1(c2ccc(Cl)cc2)CCC1	0.9991072	0.9997167707983324	0.9728184786391648	0.9905474771544688
**NC(=O)c1ccc(Oc2nccnc2C2CCNCC2)cc1**	ASINEX	NC(=O)c1ccc(Oc2nccnc2C2CCNCC2)cc1	0.9920888	0.9997490922300264	0.9797146371580132	0.9905175080320971
**CC1CCN(c2nc3c(c(=O)[nH]2)CCN(Cc2cc(F)ccc2F)C3)CC1**	ASINEX	CC1CCN(c2nc3c(c(=O)[nH]2)CCN(Cc2cc(F)ccc2F)C3)CC1	0.9976635	0.9997583169952926	0.973796889512416	0.9904062348108376
**O=C(O)c1ccc2c3c(cccc13)C(=O)NC2=O**	ASINEX	O=C(O)c1ccc2c3c(cccc13)C(=O)NC2=O	0.98913014	0.9883412916948678	0.9936180379935432	0.990363156346434
**Cc1cc(C)c2cc(CNCc3ccc(F)cc3)c(=O)[nH]c2c1**	ASINEX	Cc1cc(C)c2cc(CNCc3ccc(F)cc3)c(=O)[nH]c2c1	0.985446	0.9933291906848218	0.992062367117168	0.9902791780197714
**O=C1NC2(CCN(C(=O)c3ccncc3)CC2)Nc2ccccc21**	ASINEX	O=C1NC2(CCN(C(=O)c3ccncc3)CC2)Nc2ccccc21	0.99792683	0.9998537702567925	0.973048203544281	0.990276268348832
**O=C1NC2(CCN(C(=O)Cc3n[nH]c(=O)c4ccccc34)CC2)Nc2ccccc21**	ASINEX	O=C1NC2(CCN(C(=O)Cc3n[nH]c(=O)c4ccccc34)CC2)Nc2ccccc21	0.9995396	0.9999979090335188	0.9712677961444256	0.9902684396338998
**O=c1[nH]c2c(sc3nc(-c4ccccc4)cc(-c4ccccc4)c32)c2ccccc12**	ASINEX	O=c1[nH]c2c(sc3nc(-c4ccccc4)cc(-c4ccccc4)c32)c2ccccc12	0.99892706	0.990087647298592	0.9816667981098272	0.9902271673992724
**O=C(CN1CCc2nc(N3CCCC3)[nH]c(=O)c2C1)Nc1ccccc1N1CCOCC1**	ASINEX	O=C(CN1CCc2nc(N3CCCC3)[nH]c(=O)c2C1)Nc1ccccc1N1CCOCC1	0.9990281	0.9843123558023378	0.987315414788592	0.9902186190844074
**Cn1c(SCc2nc3ccccc3c(=O)[nH]2)nnc1-c1cccc(F)c1**	ASINEX	Cn1c(SCc2nc3ccccc3c(=O)[nH]2)nnc1-c1cccc(F)c1	0.9708916	0.9995166132029089	0.9999976060441846	0.9901352713779578
**COc1cccc(NC(=O)N(Cc2ccc(F)cc2)Cc2nc3ccccc3c(=O)[nH]2)c1**	ASINEX	COc1cccc(NC(=O)N(Cc2ccc(F)cc2)Cc2nc3ccccc3c(=O)[nH]2)c1	0.99079835	0.9938763424121736	0.9855906498799998	0.9900884488136792
**O=C(c1ccccc1)N(Cc1nc2ccccc2c(=O)[nH]1)C1CCCCC1**	ASINEX	O=C(c1ccccc1)N(Cc1nc2ccccc2c(=O)[nH]1)C1CCCCC1	0.9787507	0.9955014640018744	0.9959978867153152	0.9900833521453944
**CC(Sc1nc2c(c(=O)[nH]1)CCCC2)C(=O)Nc1cccc2ccccc12**	ASINEX	CC(Sc1nc2c(c(=O)[nH]1)CCCC2)C(=O)Nc1cccc2ccccc12	0.9933227	0.9929188779388396	0.983921630098582	0.9900543928323896
**Cc1ccccc1CN1CCc2c(nc(N3CC(C)OC(C)C3)[nH]c2=O)C1**	ASINEX	Cc1ccccc1CN1CCc2c(nc(N3CC(C)OC(C)C3)[nH]c2=O)C1	0.99138945	0.9997394626853124	0.978713616647888	0.9899475109147676
**CC(C)n1cc(CN2CCc3c(nc(N4CCCC4)[nH]c3=O)C2)c2ccccc21**	ASINEX	CC(C)n1cc(CN2CCc3c(nc(N4CCCC4)[nH]c3=O)C2)c2ccccc21	0.99705076	0.9998376631631052	0.9728937137029172	0.9899273796808452
**COc1ccc(CN2CCC(C(=O)c3ccc(Cl)cc3)CC2)cc1**	ASINEX	COc1ccc(CN2CCC(C(=O)c3ccc(Cl)cc3)CC2)cc1	0.9727543	0.9969093524851292	0.9999952081839858	0.9898862867699236
**CC1CCN(c2nc3c(c(=O)[nH]2)CCN(Cc2c(F)cccc2F)C3)CC1**	ASINEX	CC1CCN(c2nc3c(c(=O)[nH]2)CCN(Cc2c(F)cccc2F)C3)CC1	0.99897766	0.9988103825271908	0.9718656802490304	0.9898845746363446
**COc1ccc(CN(Cc2nc3ccccc3c(=O)[nH]2)C(=O)Nc2ccccc2C)cc1**	ASINEX	COc1ccc(CN(Cc2nc3ccccc3c(=O)[nH]2)C(=O)Nc2ccccc2C)cc1	0.9861856	0.9994580752191788	0.983932824430388	0.9898588366479696
**CCOC(=O)c1[nH]c(=O)c2ccccc2c1O**	ASINEX	CCOC(=O)c1[nH]c(=O)c2ccccc2c1O	0.99540013	0.9740968063894871	0.9999835337028512	0.9898268236136956
**CC(Sc1nc2ccccc2c(=O)[nH]1)C(=O)N(c1ccccc1)c1ccccc1**	ASINEX	CC(Sc1nc2ccccc2c(=O)[nH]1)C(=O)N(c1ccccc1)c1ccccc1	0.9792844	0.9981890925565462	0.9919757989702432	0.9898164324117008
**COc1ccc(CN(Cc2nc3ccccc3c(=O)[nH]2)C(=O)/C=C/c2ccccc2)cc1**	ASINEX	COc1ccc(CN(Cc2nc3ccccc3c(=O)[nH]2)C(=O)/C=C/c2ccccc2)cc1	0.9904378	0.9996236120678794	0.978935795777772	0.9896657378327566
**Cc1ccccc1NC(=O)CSc1nnc(-c2ccccn2)n1-c1ccccc1Cl**	ASINEX	Cc1ccccc1NC(=O)CSc1nnc(-c2ccccn2)n1-c1ccccc1Cl	0.9895817	0.986194778950668	0.9930277203457344	0.9896014011453708
**COc1ccc(CN2CCc3c(nc(-c4ccncc4)[nH]c3=O)C2)cc1CN1CCOCC1**	ASINEX	COc1ccc(CN2CCc3c(nc(-c4ccncc4)[nH]c3=O)C2)cc1CN1CCOCC1	0.9990746	0.9999875989307576	0.9697025623087516	0.989588246508242
**COc1ccc2[nH]c(=O)c(CNCc3ccccc3)cc2c1**	ASINEX	COc1ccc2[nH]c(=O)c(CNCc3ccccc3)cc2c1	0.97639465	0.9962141225032052	0.9956558855139822	0.9894215537791666
**CCOc1ccc(NC(=S)N(Cc2ccccc2)Cc2nc3ccccc3c(=O)[nH]2)cc1**	ASINEX	CCOc1ccc(NC(=S)N(Cc2ccccc2)Cc2nc3ccccc3c(=O)[nH]2)cc1	0.9725595	0.9984599556640036	0.9971738780156724	0.9893977817804016
**COc1ccc(C(=O)Nc2c[nH]c(=O)[nH]c2=O)cc1**	ASINEX	COc1ccc(C(=O)Nc2c[nH]c(=O)[nH]c2=O)cc1	0.9946569	0.991590001172374	0.9818461902734952	0.9893643706263046
**C=CCn1c(SCc2nc3ccccc3c(=O)[nH]2)nnc1-c1ccncc1**	ASINEX	C=CCn1c(SCc2nc3ccccc3c(=O)[nH]2)nnc1-c1ccncc1	0.97180027	0.9999754995782388	0.9957917835237752	0.9891891835994648
**CC(C)n1cc(CN2CCc3c(nc(N4CCOCC4)[nH]c3=O)C2)c2ccccc21**	ASINEX	CC(C)n1cc(CN2CCc3c(nc(N4CCOCC4)[nH]c3=O)C2)c2ccccc21	0.9992192	0.9999055272908088	0.9682679152965572	0.9891308739136028
**CCn1c(Cc2ccc(OC)cc2)nnc1SCc1nc2cc**	ASIN	CCn1c(Cc2ccc(OC)cc2)nnc1SCc1nc2ccc	0.96737	0.9998734	0.9999977	0.9890826

**ccc2c(=O)[nH]1**	EX	cc2c(=O)[nH]1	67	535332297	308945128	311373724
**CC1CN(c2nc3c(c(=O)[nH]2)CCN(C(=O)C2CCCCC2)C3)CC(C)O1**	ASINEX	CC1CN(c2nc3c(c(=O)[nH]2)CCN(C(=O)C2CCCCC2)C3)CC(C)O1	0.9953377	0.9982027775371438	0.973568124852166	0.9890362090249928
**O=C(c1cc(F)cc(F)c1)N1CCc2c(nc(N3CCCC3)[nH]c2=O)C1**	ASINEX	O=C(c1cc(F)cc(F)c1)N1CCc2c(nc(N3CCCC3)[nH]c2=O)C1	0.99921274	0.9985839206936628	0.9693078650142692	0.9890348425199128
**CC(C)(C)c1ccc(C(=O)N(Cc2nc3ccccc3c(=O)[nH]2)C2CCCCC2)cc1**	ASINEX	CC(C)(C)c1ccc(C(=O)N(Cc2nc3ccccc3c(=O)[nH]2)C2CCCCC2)cc1	0.9717662	0.9993125815039507	0.9956107325665784	0.9888964959700328
**O=C1Nc2cccc3cccc1c23**	ASINEX	O=C1Nc2cccc3cccc1c23	0.9956118	0.9710646308099248	0.9999872907199	0.9888879027907236
**CN1CCCc2cc(CN3CCc4c(nc(N5CCOCC5)[nH]c4=O)C3)ccc21**	ASINEX	CN1CCCc2cc(CN3CCc4c(nc(N5CCOCC5)[nH]c4=O)C3)ccc21	0.9989617	0.999865558232833	0.967440370055862	0.988755871792236
**O=C(CN1CCc2nc(N3CCCCC3)[nH]c(=O)c2C1)NCc1cccs1**	ASINEX	O=C(CN1CCc2nc(N3CCCCC3)[nH]c(=O)c2C1)NCc1cccs1	0.9985189	0.9881465986745062	0.9795127060036836	0.9887260629533886
**Cc1cccc(CC(=O)N2CCc3c(nc(N4CCCC4)[nH]c3=O)C2)c1**	ASINEX	Cc1cccc(CC(=O)N2CCc3c(nc(N4CCCC4)[nH]c3=O)C2)c1	0.98656076	0.996766788355986	0.9828456046487922	0.9887243849777788
**Cc1cccc(C(=O)N(Cc2ccc(Cl)cc2)Cc2nc3ccccc3c(=O)[nH]2)c1**	ASINEX	Cc1cccc(C(=O)N(Cc2ccc(Cl)cc2)Cc2nc3ccccc3c(=O)[nH]2)c1	0.9730516	0.9972274357968312	0.9958474492335624	0.9887088308797296
**Cc1n[nH]cc1C(=O)N1CCc2c(nc(N3CCC(C)CC3)[nH]c2=O)C1**	ASINEX	Cc1n[nH]cc1C(=O)N1CCc2c(nc(N3CCC(C)CC3)[nH]c2=O)C1	0.98544705	0.999001683081232	0.9813648508632948	0.9886045276951524
**O=C(O)c1ccc(-c2nc3c4ccccc4c4ccccc4c3[nH]2)cc1**	ASINEX	O=C(O)c1ccc(-c2nc3c4ccccc4c4ccccc4c3[nH]2)cc1	0.9887462	0.9999653877001782	0.9766560610725	0.9884558915355236
**COc1cc(OC)cc(C(=O)N(Cc2nc3ccccc3c(=O)[nH]2)C2CCCC2)c1**	ASINEX	COc1cc(OC)cc(C(=O)N(Cc2nc3ccccc3c(=O)[nH]2)C2CCCC2)c1	0.9689696	0.9963130418988784	0.9999947103354668	0.9884257785818992
**Cc1ccc(CN2CCc3nc(-c4ccccc4)[nH]c(=O)c3C2)cc1**	ASINEX	Cc1ccc(CN2CCc3nc(-c4ccccc4)[nH]c(=O)c3C2)cc1	0.99290943	0.9871118669516956	0.9849417498289064	0.9883210160793738
**Cc1cc(C)c2cc(CNCc3cccnc3)c(=O)[nH]c2c1**	ASINEX	Cc1cc(C)c2cc(CNCc3cccnc3)c(=O)[nH]c2c1	0.98248106	0.9973447039610652	0.9850276312119224	0.9882844658617498
**CC(Sc1nc2ccccc2c(=O)[nH]1)C(=O)Nc1ccccc1F**	ASINEX	CC(Sc1nc2ccccc2c(=O)[nH]1)C(=O)Nc1ccccc1F	0.97456795	0.997390745252302	0.9928130830368748	0.988257259353686
**CCOc1ccc2[nH]c(=O)c(CN(Cc3ccc(OC)cc3)C(=O)c3ccc(C(C)(C)C)cc3)cc2c1**	ASINEX	CCOc1ccc2[nH]c(=O)c(CN(Cc3ccc(OC)cc3)C(=O)c3ccc(C(C)(C)C)cc3)cc2c1	0.98318523	0.998103261932645	0.9834826718208312	0.9882570551463716
**Sc1nnc(-c2ccncc2)n1-c1ccccc1Cl**	ASINEX	Sc1nnc(-c2ccncc2)n1-c1ccccc1Cl	0.9835163	0.9852670463809684	0.995950427338349	0.9882445832006792
**COc1ccc(C(=O)C2CCN(Cc3nnnn3CCc3ccccc3)CC2)cc1**	ASINEX	COc1ccc(C(=O)C2CCN(Cc3nnnn3CCc3ccccc3)CC2)cc1	0.9707791	0.9969083942310776	0.9969543169656164	0.9882139440395942
**COC(=O)c1ccc(NC(=O)C(C)Sc2nc3c(c(=O)[nH]2)CCCC3)cc1**	ASINEX	COC(=O)c1ccc(NC(=O)C(C)Sc2nc3c(c(=O)[nH]2)CCCC3)cc1	0.9722848	0.9964633593160676	0.9957016836489558	0.9881499456062612
**Cc1nc2ccccc2n1CC(=O)N1CCc2c(nc(N3CCCC3)[nH]c2=O)C1**	ASINEX	Cc1nc2ccccc2n1CC(=O)N1CCc2c(nc(N3CCCC3)[nH]c2=O)C1	0.99528486	0.9998861462608366	0.968790063134387	0.9879870215869924
**O=C(NC1CCCCC1)N(Cc1ccc(F)cc1)Cc1nc2ccccc2c(=O)[nH]1**	ASINEX	O=C(NC1CCCCC1)N(Cc1ccc(F)cc1)Cc1nc2ccccc2c(=O)[nH]1	0.9725229	0.9993306683526666	0.9918425855105124	0.9878987227576056
**COc1cccc(c1)-c1ccc(C(O)=O)c(=O)[nH]1**	MOLPORT	COc1cccc(c1)-c1ccc(C(O)=O)c(=O)[nH]1	0.98441666	0.9998977479506792	0.9791969580388992	0.9878371232120924
**CCn1c(SCc2nc3ccccc3c(=O)[nH]2)nnc1-c1ccc(OC)c(OC)c1**	ASINEX	CCn1c(SCc2nc3ccccc3c(=O)[nH]2)nnc1-c1ccc(OC)c(OC)c1	0.9764891	0.9989107217349676	0.988032671019163	0.9878108398121374
**CC(C)n1cc(CN2CCc3c(nc(N4CCCCC4)[nH]c3=O)C2)c2ccccc21**	ASINEX	CC(C)n1cc(CN2CCc3c(nc(N4CCCCC4)[nH]c3=O)C2)c2ccccc21	0.99705076	0.999808298651111	0.9665120361509776	0.9877903656595342
**O=C(Nc1ccc(F)cc1)N1CCN(Cc2nc3ccccc3c(=O)[nH]2)CC1**	ASINEX	O=C(Nc1ccc(F)cc1)N1CCN(Cc2nc3ccccc3c(=O)[nH]2)CC1	0.9941559	0.9732599557477296	0.99554165089963	0.9876524968121406
**COc1ccc(C2(C(=O)N3CCC(Nc4nc5c(c(=O)[nH]4)CCCC5)CC3)CC2)cc1**	ASINEX	COc1ccc(C2(C(=O)N3CCC(Nc4nc5c(c(=O)[nH]4)CCCC5)CC3)CC2)cc1	0.9889241	0.9999547274020032	0.9740727728903	0.987650528795402
**Oc1nc(-c2ccncc2)nc2c1CCNCC2**	ASINEX	Oc1nc(-c2ccncc2)nc2c1CCNCC2	0.96449006	0.9983372446515014	0.999999587861438	0.9876089628502808
**COC(=O)c1ccc(NC(=O)C(C)Sc2nc3ccccc3c(=O)[nH]2)cc1**	ASINEX	COC(=O)c1ccc(NC(=O)C(C)Sc2nc3ccccc3c(=O)[nH]2)cc1	0.96273196	0.9994012688163768	0.9999970348462014	0.9873767536993956
**COc1ccc(CCN(Cc2nc3ccccc3c(=O)[nH]2)C(=O)c2ccc(F)cc2)cc1OC**	ASINEX	COc1ccc(CCN(Cc2nc3ccccc3c(=O)[nH]2)C(=O)c2ccc(F)cc2)cc1OC	0.99790907	0.9972692084523485	0.9668507928699006	0.9873430234611762
**O=C1c2ccccc2-c2cc(=O)[nH]c3cccc1c23**	ASINEX	O=C1c2ccccc2-c2cc(=O)[nH]c3cccc1c23	0.9837456	0.9820160379598798	0.9962564387812078	0.9873393505640866
**COc1ccc(CN2CCc3c(nc(-c4ccncc4)[nH]c3=O)C2)cc1CO**	ASINEX	COc1ccc(CN2CCc3c(nc(-c4ccncc4)[nH]c3=O)C2)cc1CO	0.99822825	0.9998042974796384	0.9639346282523836	0.9873223925553578
**CC1CCN(c2nc3c(c(=O)[nH]2)CCN(Cc2ccccc2F)C3)CC1**	ASINEX	CC1CCN(c2nc3c(c(=O)[nH]2)CCN(Cc2ccccc2F)C3)CC1	0.9992036	0.9997973282146146	0.9624252560594596	0.9871420688717434
**COc1ccccc1OCc1nnc(SCc2nc3ccccc3c(=O)[nH]2)n1C**	ASINEX	COc1ccccc1OCc1nnc(SCc2nc3ccccc3c(=O)[nH]2)n1C	0.97563666	0.9913124540118275	0.9943639780963331	0.9871043643869548
**CCn1c(CCNC(=O)c2ccc(OC)cc2)nnc1SCc1nc2ccccc2c(=O)[nH]1**	ASINEX	CCn1c(CCNC(=O)c2ccc(OC)cc2)nnc1SCc1nc2ccccc2c(=O)[nH]1	0.9638777	0.9998711896188388	0.9974775822015268	0.9870754832459486
**COc1cccc(NC(=O)N(Cc2ccccc2)Cc2nc3**	ASIN	COc1cccc(NC(=O)N(Cc2ccccc2)Cc2nc3	0.98795	0.9895527	0.9836640	0.9870567

**ccccc3c(=O)[nH]2)c1**	EX	ccccc3c(=O)[nH]2)c1	354	081552686	585961944	701381626
**COc1ccc(-n2c(SCc3nc4ccccc4c(=O)[nH]3)nc3ccccc3c2=O)cc1**	ASINEX	COc1ccc(-n2c(SCc3nc4ccccc4c(=O)[nH]3)nc3ccccc3c2=O)cc1	0.9787098	0.9984717116170608	0.9838487138701364	0.9870100808066252
**Cc1ccc(-c2cc(C(F)(F)F)c(C#N)c(=O)[nH]2)cc1**	ASINEX	Cc1ccc(-c2cc(C(F)(F)F)c(C#N)c(=O)[nH]2)cc1	0.9957281	0.9715172439094338	0.9936516434184044	0.9869656542773804
**NCCNC(=O)CCc1n[nH]c(=O)c2ccccc12**	ASINEX	NCCNC(=O)CCc1n[nH]c(=O)c2ccccc12	0.9965132	0.9838619308750126	0.9804379006226904	0.9869376731276626
**CC1=C(C(=O)NCc2nc3ccccc3c(=O)[nH]2)OCCO1**	MOLPORT	CC1=C(C(=O)NCc2nc3ccccc3c(=O)[nH]2)OCCO1	0.97019863	0.9950300684494768	0.995435563852958	0.9868880878630186
**O=C(CSc1nnc(-c2ccccn2)n1-c1ccccc1Cl)N1CCOCC1**	ASINEX	O=C(CSc1nnc(-c2ccccn2)n1-c1ccccc1Cl)N1CCOCC1	0.9832659	0.9893425078027894	0.9878705807609603	0.9868263217779232
**COc1ccc(C2(C(=O)N3CCC(Nc4nc5c(c(=O)[nH]4)CCC5)CC3)CC2)cc1**	ASINEX	COc1ccc(C2(C(=O)N3CCC(Nc4nc5c(c(=O)[nH]4)CCC5)CC3)CC2)cc1	0.9889241	0.999970399529892	0.971545970020292	0.9868134852146954
**CC(C)c1ncc(CN2CCc3c(nc(-c4ccncc4)[nH]c3=O)C2)cn1**	ASINEX	CC(C)c1ncc(CN2CCc3c(nc(-c4ccncc4)[nH]c3=O)C2)cn1	0.99565494	0.9998387458655538	0.9648634867249052	0.9867857243985408
**CN(C)CCCOc1ccc(C(=O)C2CCOCC2)cc1**	MOLPORT	CN(C)CCCOc1ccc(C(=O)C2CCOCC2)cc1	0.99012357	0.987879518898766	0.982171926364501	0.98672500507621
**NCc1n[nH]c(=O)c2ccccc12**	ASINEX	NCc1n[nH]c(=O)c2ccccc12	0.99825615	0.9618881752244184	0.9999955646520616	0.986713295594762
**CC(C)CCN1CCc2c(nc(N3CC(C)OC(C)C3)[nH]c2=O)C1**	ASINEX	CC(C)CCN1CCc2c(nc(N3CC(C)OC(C)C3)[nH]c2=O)C1	0.99335444	0.994670302376701	0.9719812965412644	0.986668679551126
**CC1CCN(c2nc3c(c(=O)[nH]2)CCN(C(=O)c2cnccn2)C3)CC1**	ASINEX	CC1CCN(c2nc3c(c(=O)[nH]2)CCN(C(=O)c2cnccn2)C3)CC1	0.99913687	0.9993309774715958	0.9611518135650492	0.9865398853918842
**O=C(c1cccc(Cl)c1)N(Cc1ccc2c(c1)OCO2)Cc1nc2ccccc2c(=O)[nH]1**	ASINEX	O=C(c1cccc(Cl)c1)N(Cc1ccc2c(c1)OCO2)Cc1nc2ccccc2c(=O)[nH]1	0.96750444	0.996187096298118	0.9958559127012288	0.9865158169124918
**COc1ccc(CN(Cc2nc3ccccc3c(=O)[nH]2)C(=O)NC2CCCCC2)cc1**	ASINEX	COc1ccc(CN(Cc2nc3ccccc3c(=O)[nH]2)C(=O)NC2CCCCC2)cc1	0.959561	0.9999417806307556	0.9999839649227176	0.98649557862901
**CC1CCN(c2nc3c(c(=O)[nH]2)CCN(CC2CCCCC2)C3)CC1**	ASINEX	CC1CCN(c2nc3c(c(=O)[nH]2)CCN(CC2CCCCC2)C3)CC1	0.99507964	0.9997441922174384	0.9645793515502452	0.9864677267804915
**CC(C)c1ccc(NC(=O)CN2CCC(Nc3nc4c(c(=O)[nH]3)CCCC4)CC2)cc1**	ASINEX	CC(C)c1ccc(NC(=O)CN2CCC(Nc3nc4c(c(=O)[nH]3)CCCC4)CC2)cc1	0.975575	0.9976856543088516	0.986101258997034	0.986453961183749
**CN1CCC2(CC1)NC(=O)c1ccccc1N2**	ASINEX	CN1CCC2(CC1)NC(=O)c1ccccc1N2	0.9954431	0.9991687672497466	0.964546309228796	0.9863860607253914
**CN(C)c1ncc(CN2CCc3c(nc(-c4ccncc4)[nH]c3=O)C2)cn1**	ASINEX	CN(C)c1ncc(CN2CCc3c(nc(-c4ccncc4)[nH]c3=O)C2)cn1	0.9959437	0.9991853467929308	0.9634217646973252	0.9861836121997856
**O=c1[nH]c(-c2ccccn2)nc2c1CCN(Cc1ccc(OC(F)(F)F)cc1)C2**	ASINEX	O=c1[nH]c(-c2ccccn2)nc2c1CCN(Cc1ccc(OC(F)(F)F)cc1)C2	0.9985092	0.999877047154585	0.9595933141337742	0.9859931965059606
**COc1ccc(C2(O)CCN(CC(=O)Nc3ccccc3C(N)=O)CC2)cc1**	ASINEX	COc1ccc(C2(O)CCN(CC(=O)Nc3ccccc3C(N)=O)CC2)cc1	0.9684096	0.994604620667658	0.9949268597502168	0.9859803594305208
**COc1cc2c(cc1CN1CCc3c(nc(-c4ccncc4)[nH]c3=O)C1)OCO2**	ASINEX	COc1cc2c(cc1CN1CCc3c(nc(-c4ccncc4)[nH]c3=O)C1)OCO2	0.99755305	0.999883385623066	0.960300442575992	0.985912292905698
**O=C(c1cnn2cccnc12)N1CCc2c(nc(N3CCCC3)[nH]c2=O)C1**	ASINEX	O=C(c1cnn2cccnc12)N1CCc2c(nc(N3CCCC3)[nH]c2=O)C1	0.99914646	0.9995116943215676	0.9586748868464994	0.9857776808849612
**COc1ccc(OC)c2[nH]c(=O)c(CNc3ccc(C)c4ncccc34)cc12**	ASINEX	COc1ccc(OC)c2[nH]c(=O)c(CNc3ccc(C)c4ncccc34)cc12	0.9826805	0.9980376730608987	0.9764447492049074	0.9857209739398288
**CCC(=O)N(Cc1ccc(OC)cc1)Cc1cc2c(C)cc(C)cc2[nH]c1=O**	ASINEX	CCC(=O)N(Cc1ccc(OC)cc1)Cc1cc2c(C)cc(C)cc2[nH]c1=O	0.97048044	0.9978909534657696	0.9882979811553576	0.9855564588894152
**O=c1[nH]c(N2CCOCC2)nc2c1CN(C1CCCC1)CC2**	ASINEX	O=c1[nH]c(N2CCOCC2)nc2c1CN(C1CCCC1)CC2	0.99918884	0.9877123589338742	0.9692357413561464	0.985378980226424
**COc1ccc(CCN2CC(=O)C(c3nc4ccccc4c(=O)[nH]3)=C2N)cc1OC**	ASINEX	COc1ccc(CCN2CC(=O)C(c3nc4ccccc4c(=O)[nH]3)=C2N)cc1OC	0.9666821	0.998586200936611	0.9903892694753238	0.9852191822886992
**COc1ccccc1CN1CCc2c(nc(-c3ccccn3)[nH]c2=O)C1**	ASINEX	COc1ccccc1CN1CCc2c(nc(-c3ccccn3)[nH]c2=O)C1	0.998664	0.9997428238130284	0.9571893784218783	0.9851987412423036
**O=C1NC2(CCN(Cc3ccccc3)CC2)Nc2ccccc21**	ASINEX	O=C1NC2(CCN(Cc3ccccc3)CC2)Nc2ccccc21	0.99611735	0.9998707787923946	0.9595249135763648	0.9851710152693636
**CCOc1ccc(C(=O)N(Cc2ccc(F)cc2)Cc2cc3c(C)cc(C)cc3[nH]c2=O)cc1**	ASINEX	CCOc1ccc(C(=O)N(Cc2ccc(F)cc2)Cc2cc3c(C)cc(C)cc3[nH]c2=O)cc1	0.99132466	0.9818865166745266	0.982159318977335	0.9851234996046976
**OC(=O)c1ccc([nH]c1=O)-c1ccccc1**	MOLPORT	OC(=O)c1ccc([nH]c1=O)-c1ccccc1	0.97059834	0.9846720810985002	0.9999893470458536	0.9850865893929462
**C=CCn1c(SCc2nc3ccccc3c(=O)[nH]2)nc1-c1ccccc1F**	ASINEX	C=CCn1c(SCc2nc3ccccc3c(=O)[nH]2)nnc1-c1ccccc1F	0.9751403	0.9999835559453196	0.979922873048576	0.9850155675216364
**CCn1c(COc2ccc(OC)cc2)nnc1SCc1nc2ccccc2c(=O)[nH]1**	ASINEX	CCn1c(COc2ccc(OC)cc2)nnc1SCc1nc2ccccc2c(=O)[nH]1	0.9596987	0.999571303833753	0.9957136148952692	0.9849945319306702
**COc1cccc(C(=O)N(CCc2ccc(Cl)cc2)Cc2**	ASIN	COc1cccc(C(=O)N(CCc2ccc(Cl)cc2)Cc2	0.99078	0.9957950	0.9682337	0.9849365

**nc3ccccc3c(=O)[nH]2)c1**	EX	nc3ccccc3c(=O)[nH]2)c1	08	449801336	5752256	244271164
**C=CCn1c(Cc2ccccc2)nnc1SCc1nc2ccccc2c(=O)[nH]1**	ASINEX	C=CCn1c(Cc2ccccc2)nnc1SCc1nc2ccccc2c(=O)[nH]1	0.9677683	0.99900303492205	0.9880233700726968	0.9849315721650987
**COc1ccc(C(=O)C2CCN(C(=O)NCc3ccccc3)CC2)cc1**	ASINEX	COc1ccc(C(=O)C2CCN(C(=O)NCc3ccccc3)CC2)cc1	0.9599607	0.9944704158554948	0.9999860135073386	0.9848057094814182
**O=c1[nH]c(-c2cccnc2)nc2c1CCN(CC1CC1)C2**	ASINEX	O=c1[nH]c(-c2cccnc2)nc2c1CCN(CC1CC1)C2	0.9984479	0.9998653559259232	0.9559359860563216	0.9847497456774312
**O=C(N1CCOCC1)N1CCc2nc(N3CCOCC3)[nH]c(=O)c2C1**	MOLPORT	O=C(N1CCOCC1)N1CCc2nc(N3CCOCC3)[nH]c(=O)c2C1	0.99984276	0.9781640466861836	0.9761955164421292	0.9847341086917986
**CC1CCN(c2nc3c(c(=O)[nH]2)CCN(Cc2ccc(OCC(=O)O)cc2)C3)CC1**	ASINEX	CC1CCN(c2nc3c(c(=O)[nH]2)CCN(Cc2ccc(OCC(=O)O)cc2)C3)CC1	0.9991041	0.999934107966861	0.9550003654929766	0.984679518681406
**COc1ccc(CN2CCc3c(nc(-c4ccncc4)[nH]c3=O)C2)cc1CN1CCCC1**	ASINEX	COc1ccc(CN2CCc3c(nc(-c4ccncc4)[nH]c3=O)C2)cc1CN1CCCC1	0.9945749	0.9999262599489634	0.9595051948069732	0.9846687863992568
**Cc1cc(C)c2cc(CNc3cc(-c4ccccc4)ccc3O)c(=O)[nH]c2c1**	ASINEX	Cc1cc(C)c2cc(CNc3cc(-c4ccccc4)ccc3O)c(=O)[nH]c2c1	0.9882434	0.9909593352907756	0.9743927896626058	0.9845318420013164
**CCOc1ccc(C(=O)C2CCN(C(=O)CCC(=O)O)CC2)cc1**	ASINEX	CCOc1ccc(C(=O)C2CCN(C(=O)CCC(=O)O)CC2)cc1	0.96229154	0.9950143863026556	0.9961161371573872	0.9844740207251352
**COc1ccc(NC(=O)N2CCC(C(=O)c3ccc(F)cc3)CC2)cc1**	ASINEX	COc1ccc(NC(=O)N2CCC(C(=O)c3ccc(F)cc3)CC2)cc1	0.96778965	0.9854557753340646	0.9999915037220068	0.98441230967315
**COc1ccc(CNCc2cc3cc(OC)ccc3[nH]c2=O)cc1**	ASINEX	COc1ccc(CNCc2cc3cc(OC)ccc3[nH]c2=O)cc1	0.95919544	0.998236347441877	0.9956654534493784	0.9843657453128016
**COc1ccc(-c2ccccc2)cc1NCc1cc2c(C)cc(C)cc2[nH]c1=O**	ASINEX	COc1ccc(-c2ccccc2)cc1NCc1cc2c(C)cc(C)cc2[nH]c1=O	0.98751706	0.9856537200999648	0.979665288769567	0.984278689239622
**COc1ccc(C(=O)N(Cc2ccc(F)cc2)Cc2cc3cc(OC)ccc3[nH]c2=O)cc1**	ASINEX	COc1ccc(C(=O)N(Cc2ccc(F)cc2)Cc2cc3cc(OC)ccc3[nH]c2=O)cc1	0.99085367	0.9805730926526112	0.9812702796218477	0.984232346511225
**O=C(CN1CCc2nc(N3CCCC3)[nH]c(=O)c2C1)NC1CCCCC1**	ASINEX	O=C(CN1CCc2nc(N3CCCC3)[nH]c(=O)c2C1)NC1CCCCC1	0.99804854	0.9725175173622006	0.981992066337848	0.9841860425433672
**O=C(c1ccco1)N(Cc1nc2ccccc2c(=O)[nH]1)C1CCCC1**	ASINEX	O=C(c1ccco1)N(Cc1nc2ccccc2c(=O)[nH]1)C1CCCC1	0.96615267	0.994668162118526	0.9911999086365368	0.9840069129181104
**O=c1[nH]c(-c2ccccn2)nc2c1CCN(Cc1cccs1)C2**	ASINEX	O=c1[nH]c(-c2ccccn2)nc2c1CCN(Cc1cccs1)C2	0.9982262	0.999584319211192	0.9541078071508488	0.9839727839127234
**Cn1c(CC(=O)Nc2ccccc2Cl)nnc1SCc1nc2ccccc2c(=O)[nH]1**	ASINEX	Cn1c(CC(=O)Nc2ccccc2Cl)nnc1SCc1nc2ccccc2c(=O)[nH]1	0.9653986	0.9993170035848208	0.9871657391664184	0.9839604507964844
**CC1CCN(c2nc3c(c(=O)[nH]2)CCN(Cc2ccccc2Cl)C3)CC1**	ASINEX	CC1CCN(c2nc3c(c(=O)[nH]2)CCN(Cc2ccccc2Cl)C3)CC1	0.9984628	0.9997657967229644	0.953634033785966	0.9839542089067244
**Cc1ccc(CNCc2cc3c(C)cc(C)cc3[nH]c2=O)cc1**	ASINEX	Cc1ccc(CNCc2cc3c(C)cc(C)cc3[nH]c2=O)cc1	0.9712715	0.9923710342287386	0.9882099189305176	0.9839508226839448
**CC1CN(c2nc3c(c(=O)[nH]2)CCN(Cc2cc(F)ccc2F)C3)CC(C)O1**	ASINEX	CC1CN(c2nc3c(c(=O)[nH]2)CCN(Cc2cc(F)ccc2F)C3)CC(C)O1	0.99642545	0.9996059975142038	0.9557664290021882	0.9839326254548556
**CC1CCN(c2nc3c(c(=O)[nH]2)CCN(Cc2cccs2)C3)CC1**	ASINEX	CC1CCN(c2nc3c(c(=O)[nH]2)CCN(Cc2cccs2)C3)CC1	0.9965848	0.9990747665626564	0.9559559631971802	0.9838718342744986
**CCC(C(=O)N1CCc2c(nc(N3CCOCC3)[nH]c2=O)C1)c1ccccc1**	ASINEX	CCC(C(=O)N1CCc2c(nc(N3CCOCC3)[nH]c2=O)C1)c1ccccc1	0.99728477	0.9971549728899683	0.9571295252525952	0.9838564227181552
**CC1CCN(c2nc3c(c(=O)[nH]2)CCN(Cc2ccnc4ccccc24)C3)CC1**	ASINEX	CC1CCN(c2nc3c(c(=O)[nH]2)CCN(Cc2ccnc4ccccc24)C3)CC1	0.999243	0.9997022932266324	0.9526135334342384	0.9838529492240744
**O=C1OC(=O)c2ccc(-c3nnc(-c4ccccc4)o3)c3cccc1c23**	MOLPORT	O=C1OC(=O)c2ccc(-c3nnc(-c4ccccc4)o3)c3cccc1c23	0.96327156	0.9975577660006756	0.9899988140338328	0.983609379439756
**O=c1[nH]c(-c2cccnc2)nc2c1CCN(Cc1ccc(C(F)(F)F)cc1)C2**	ASINEX	O=c1[nH]c(-c2cccnc2)nc2c1CCN(Cc1ccc(C(F)(F)F)cc1)C2	0.9983315	0.9994369467553184	0.9529306719575676	0.9835663686305628
**COc1ccc(CN(Cc2cc3cc(OC)ccc3[nH]c2=O)C(=O)c2ccc(OC)cc2)cc1**	ASINEX	COc1ccc(CN(Cc2cc3cc(OC)ccc3[nH]c2=O)C(=O)c2ccc(OC)cc2)cc1	0.987546	0.979866334096824	0.982828633167784	0.983413664656854
**Cn1c(COc2ccc3c(c2)CCCC3)nnc1SCc1nc2ccccc2c(=O)[nH]1**	ASINEX	Cn1c(COc2ccc3c(c2)CCCC3)nnc1SCc1nc2ccccc2c(=O)[nH]1	0.9550706	0.9988835988095126	0.996082209473392	0.9833454743658876
**C=CCn1cc(CN2CCc3c(nc(N4CCOCC4)[nH]c3=O)C2)c2ccccc21**	ASINEX	C=CCn1cc(CN2CCc3c(nc(N4CCOCC4)[nH]c3=O)C2)c2ccccc21	0.99953556	0.9999565066313444	0.9504467297222308	0.9833129323204952
**NC(=O)c1ccc(Oc2nccnc2C2CCCNC2)cc1**	ASINEX	NC(=O)c1ccc(Oc2nccnc2C2CCCNC2)cc1	0.9607488	0.9999969560490702	0.9891843666564438	0.9833100381333852
**O=c1[nH]c(-c2ccccc2)nc2c1C1(CCCC1)Cc1ccccc1-2**	ASINEX	O=c1[nH]c(-c2ccccc2)nc2c1C1(CCCC1)Cc1ccccc1-2	0.9966897	0.9597315323147376	0.9933640209970565	0.9832617435167528
**COc1ccc2c(CN3CCc4c(nc(-c5ccncc5)[nH]c4=O)C3)cn(C)c2c1**	ASINEX	COc1ccc2c(CN3CCc4c(nc(-c5ccncc5)[nH]c4=O)C3)cn(C)c2c1	0.9988834	0.9998548917389892	0.9510261939127488	0.9832548372803868
**Cn1c(COc2ccc(Cl)cc2)nnc1SCc1nc2ccccc2c(=O)[nH]1**	ASINEX	Cn1c(COc2ccc(Cl)cc2)nnc1SCc1nc2ccccc2c(=O)[nH]1	0.9551997	0.9978902563734456	0.99662652713765	0.9832388339954792
**Cc1ccc(-c2cnn3c(O)cc(Cc4n[nH]c(=O)c5ccccc45**	ASINEX	Cc1ccc(-c2cnn3c(O)cc(Cc4n[nH]c(=O)c5ccccc45	0.9545748	0.9949047168884528	0.999986229910202	0.983155256726057

**)nc23)cc1**		)nc23)cc1				
**CCOc1cc(CN2CCc3c(nc(N4CCC(C)CC4)[nH]c3=O)C2)ccc1OC**	ASINEX	CCOc1cc(CN2CCc3c(nc(N4CCC(C)CC4)[nH]c3=O)C2)ccc1OC	0.99650323	0.998993564291202	0.953886797402234	0.9831278652010144
**O=c1[nH]c(-c2ccncc2)nc2c1CCN(Cc1c[nH]nc1-c1ccc(O)cc1)C2**	ASINEX	O=c1[nH]c(-c2ccncc2)nc2c1CCN(Cc1c[nH]nc1-c1ccc(O)cc1)C2	0.99938273	0.9997599127479976	0.9501245077320936	0.9830890515929326
**COCc1cc(=O)[nH]c(N2CCN(c3ccccc3F)CC2)n1**	ASINEX	COCc1cc(=O)[nH]c(N2CCN(c3ccccc3F)CC2)n1	0.94949955	0.9995856091914962	0.9999999318447303	0.9830283628392544
**CCOc1ccc2[nH]c(=O)c(CNCc3ccccc3)cc2c1**	ASINEX	CCOc1ccc2[nH]c(=O)c(CNCc3ccccc3)cc2c1	0.95382375	0.9977749222364878	0.9968503422421348	0.9828163365764416
**O=C(CSc1nc2ccccc2c(=O)[nH]1)Nc1ccc(F)cc1F**	ASINEX	O=C(CSc1nc2ccccc2c(=O)[nH]1)Nc1ccc(F)cc1F	0.9823416	0.9686343421660708	0.9973479288484912	0.98277461951935
**Cc1ccc(-c2nnc(SCc3cc(=O)[nH]c4ccccc34)n2-c2ccccc2)cc1**	ASINEX	Cc1ccc(-c2nnc(SCc3cc(=O)[nH]c4ccccc34)n2-c2ccccc2)cc1	0.94797844	0.9999851575827724	0.9999999158447752	0.9826545034581388
**COc1cccc(NC(=O)N(Cc2nc3ccccc3c(=O)[nH]2)C2CCCCC2)c1**	ASINEX	COc1cccc(NC(=O)N(Cc2nc3ccccc3c(=O)[nH]2)C2CCCCC2)c1	0.9639457	0.9922808481368296	0.9916858697163096	0.982637468223436
**COc1ccc(CCNCc2cc3c(OC)ccc(OC)c3[nH]c2=O)cc1**	ASINEX	COc1ccc(CCNCc2cc3c(OC)ccc(OC)c3[nH]c2=O)cc1	0.9789338	0.997082691980406	0.9718430360027844	0.9826198463903116
**Cc1ccc(NC(=O)N(Cc2ccc(F)cc2)Cc2nc3ccccc3c(=O)[nH]2)cc1**	ASINEX	Cc1ccc(NC(=O)N(Cc2ccc(F)cc2)Cc2nc3ccccc3c(=O)[nH]2)cc1	0.98026216	0.9879550305020156	0.9796230258514772	0.9826134055515672
**COc1ccc(C2(NC(=O)Cc3n[nH]c(=O)c4ccccc34)CCCCC2)cc1**	ASINEX	COc1ccc(C2(NC(=O)Cc3n[nH]c(=O)c4ccccc34)CCCCC2)cc1	0.9821348	0.997399403817672	0.9682279954411804	0.982587406096538
**COc1cccc(N2CC(=O)C(c3nc4ccccc4c(=O)[nH]3)=C2N)c1**	ASINEX	COc1cccc(N2CC(=O)C(c3nc4ccccc4c(=O)[nH]3)=C2N)c1	0.98806494	0.9954900268205557	0.964053293593844	0.98253608838617
**CC1CN(c2nc3c(c(=O)[nH]2)CCN(Cc2c(F)cccc2F)C3)CC(C)O1**	ASINEX	CC1CN(c2nc3c(c(=O)[nH]2)CCN(Cc2c(F)cccc2F)C3)CC(C)O1	0.998464	0.9980617904083746	0.9510523241727884	0.9825260342951004
**O=C(O)Cn1cc(CN2CCc3c(nc(N4CCCC4)[nH]c3=O)C2)c2ccccc21**	ASINEX	O=C(O)Cn1cc(CN2CCc3c(nc(N4CCCC4)[nH]c3=O)C2)c2ccccc21	0.9996319	0.9999672409251972	0.947910045829664	0.982503056156243
**O=c1cc(-c2ccc(F)cc2)nc(N2CCN(c3ccccc3)CC2)[nH]1**	ASINEX	O=c1cc(-c2ccc(F)cc2)nc(N2CCN(c3ccccc3)CC2)[nH]1	0.9778176	0.9694250709705527	0.9999869092880418	0.98240985842121
**O=c1[nH]c(-c2cccnc2)nc2c1CCN(Cc1cccc3nccnc13)C2**	ASINEX	O=c1[nH]c(-c2cccnc2)nc2c1CCN(Cc1cccc3nccnc13)C2	0.9993357	0.9998319539827676	0.9475580577759068	0.9822419059975508
**O=C(c1cc(F)cc(F)c1)N1CCc2nc(N3CCCC3)[nH]c(=O)c2C1**	ASINEX	O=C(c1cc(F)cc(F)c1)N1CCc2nc(N3CCCC3)[nH]c(=O)c2C1	0.99921274	0.9841824643168352	0.9632068745049954	0.982200693557879
**O=C(c1cccc(Cl)c1)N(Cc1nc2ccccc2c(=O)[nH]1)C1CCCC1**	ASINEX	O=C(c1cccc(Cl)c1)N(Cc1nc2ccccc2c(=O)[nH]1)C1CCCC1	0.9716091	0.9843576822989888	0.9903999331755003	0.9821222436916918
**COc1ccc2[nH]c(=O)c(CNCc3cccnc3)cc2c1**	ASINEX	COc1ccc2[nH]c(=O)c(CNCc3cccnc3)cc2c1	0.96314096	0.99795722060651	0.985078523556104	0.9820589028902236
**COc1cccc(OC)c1C(=O)N(Cc1ccc(Cl)cc1)Cc1nc2ccccc2c(=O)[nH]1**	ASINEX	COc1cccc(OC)c1C(=O)N(Cc1ccc(Cl)cc1)Cc1nc2ccccc2c(=O)[nH]1	0.994216	0.9862211600569076	0.965226844565726	0.9818880098327584
**COc1ccc(-c2csc(NC(=O)CSc3nc4ccccc4c(=O)[nH]3)n2)cc1**	ASINEX	COc1ccc(-c2csc(NC(=O)CSc3nc4ccccc4c(=O)[nH]3)n2)cc1	0.9835504	0.9861973861547148	0.9758636807581572	0.9818704987523498
**COc1ccc(CN2CCc3c(nc(N4CC(C)OC(C)C4)[nH]c3=O)C2)cc1OC**	ASINEX	COc1ccc(CN2CCc3c(nc(N4CC(C)OC(C)C4)[nH]c3=O)C2)cc1OC	0.99653035	0.9983096858334964	0.9506988496594756	0.9818462965053172
**CC1CCN(c2nc3c(c(=O)[nH]2)CCN(Cc2ccccc2O)C3)CC1**	ASINEX	CC1CCN(c2nc3c(c(=O)[nH]2)CCN(Cc2ccccc2O)C3)CC1	0.99832004	0.9998445714611373	0.9473435962886894	0.981836070278944
**COc1ccc(cc1)-c1ccc(C(O)=O)c(=O)[nH]1**	MOLPORT	COc1ccc(cc1)-c1ccc(C(O)=O)c(=O)[nH]1	0.97518104	0.9944281674544784	0.975816443124086	0.9818085512422016
**O=c1[nH]c(N2CCOCC2)nc2c1CN(C1CCCCC1)CC2**	ASINEX	O=c1[nH]c(N2CCOCC2)nc2c1CN(C1CCCCC1)CC2	0.99918884	0.985521453812316	0.9605917681685936	0.9817673541233872
**O=C(Cc1n[nH]c(=O)c2ccccc12)N1CCCCC1CN1CCCC1**	ASINEX	O=C(Cc1n[nH]c(=O)c2ccccc12)N1CCCCC1CN1CCCC1	0.9782428	0.9965632291272262	0.9702748030181996	0.9816936155620964
**COc1ccc(CN(Cc2cc3c(C)cc(C)cc3[nH]c2=O)C(=O)c2ccc(Br)cc2)cc1**	ASINEX	COc1ccc(CN(Cc2cc3c(C)cc(C)cc3[nH]c2=O)C(=O)c2ccc(Br)cc2)cc1	0.9888854	0.9793407943679492	0.9766073663996826	0.9816111878156918
**Cn1c(CC(=O)Nc2ccccc2F)nnc1SCc1nc2ccccc2c(=O)[nH]1**	ASINEX	Cn1c(CC(=O)Nc2ccccc2F)nnc1SCc1nc2ccccc2c(=O)[nH]1	0.97579443	0.9992398925890096	0.9697230487519792	0.9815857919628044
**O=C(Cc1n[nH]c(=O)c2ccccc12)N1CCC(n2nnc(-c3ccc(F)cc3)n2)CC1**	ASINEX	O=C(Cc1n[nH]c(=O)c2ccccc12)N1CCC(n2nnc(-c3ccc(F)cc3)n2)CC1	0.98304915	0.9869034849509436	0.9747714622485774	0.9815747004937124
**Cc1ccc(C)c2[nH]c(=O)c(CNCc3ccccc3)cc12**	ASINEX	Cc1ccc(C)c2[nH]c(=O)c(CNCc3ccccc3)cc12	0.98542386	0.9850341727279098	0.9741472019791328	0.9815350792137184
**CN(Cc1cc(-c2ccc(F)cc2)c[nH]c1=O)Cc1cccc2ncccc12**	ASINEX	CN(Cc1cc(-c2ccc(F)cc2)c[nH]c1=O)Cc1cccc2ncccc12	0.9964824	0.996421150101096	0.951698100027801	0.981533874137611
**O=C(CN1CCc2nc(N3CCCC3)[nH]c(=O)c2C1)N1CCCCC1**	ASINEX	O=C(CN1CCc2nc(N3CCCC3)[nH]c(=O)c2C1)N1CCCCC1	0.99900705	0.9662472608028227	0.978868558716528	0.9813742885806792

**O=C(CN1CCc2nc(N3CCCC3)[nH]c(=O)c2C1)N1CCCCCC1**	ASINEX	O=C(CN1CCc2nc(N3CCCC3)[nH]c(=O)c2C1)N1CCCCCC1	0.99900705	0.9662472608028227	0.978868558716528	0.9813742885806792
**CCOc1ccc2[nH]c(=O)c(CN(Cc3cccnc3)C(=O)c3ccccc3F)cc2c1**	ASINEX	CCOc1ccc2[nH]c(=O)c(CN(Cc3cccnc3)C(=O)c3ccccc3F)cc2c1	0.9712607	0.9980819290415086	0.9746468365298108	0.9813298306743976
**COc1ccc(CN(Cc2nc3ccccc3c(=O)[nH]2)C(=O)Nc2cccc(C)c2)cc1**	ASINEX	COc1ccc(CN(Cc2nc3ccccc3c(=O)[nH]2)C(=O)Nc2cccc(C)c2)cc1	0.9733815	0.997107069729769	0.9731105004382224	0.981199696495206
**C=CCOc1ccc(C(=O)N2CCc3c(nc(N4CC(C)OC(C)C4)[nH]c3=O)C2)cc1**	ASINEX	C=CCOc1ccc(C(=O)N2CCc3c(nc(N4CC(C)OC(C)C4)[nH]c3=O)C2)cc1	0.99755186	0.9991490110689112	0.94675908404962	0.9811533178478904
**O=C(Nc1ccccc1Cl)N(Cc1nc2ccccc2c(=O)[nH]1)C1CCCCC1**	ASINEX	O=C(Nc1ccccc1Cl)N(Cc1nc2ccccc2c(=O)[nH]1)C1CCCCC1	0.96723014	0.9951395349130876	0.980931193855758	0.9811002899772392
**Cc1nnc(SCc2nc3ccccc3c(=O)[nH]2)n1C**	ASINEX	Cc1nnc(SCc2nc3ccccc3c(=O)[nH]2)n1C	0.9436338	0.9993669151543582	0.9999997401603674	0.9810001500330906
**O=C(CN1CCc2nc(N3CCCC3)[nH]c(=O)c2C1)Nc1cc(C(F)(F)F)ccc1Cl**	ASINEX	O=C(CN1CCc2nc(N3CCCC3)[nH]c(=O)c2C1)Nc1cc(C(F)(F)F)ccc1Cl	0.9981589	0.9826852240556012	0.9616840255529848	0.980842707245373
**O=c1[nH]c(-c2ccncc2)nc2c1CCN(Cc1cc(Cl)ccc1O)C2**	ASINEX	O=c1[nH]c(-c2ccncc2)nc2c1CCN(Cc1cc(Cl)ccc1O)C2	0.99780935	0.9999420050682516	0.9447277042767576	0.9808263532785264
**Cc1nc(N2CCN(C)CC2)[nH]c(=O)c1Cc1cccc2ccccc12**	ASINEX	Cc1nc(N2CCN(C)CC2)[nH]c(=O)c1Cc1cccc2ccccc12	0.9702228	0.9921080847979138	0.979805089746502	0.9807119819040572
**N#Cc1ccc(-c2ccccc2)[nH]c1=O**	ASINEX	N#Cc1ccc(-c2ccccc2)[nH]c1=O	0.99937266	0.9472533277401224	0.9953473129803908	0.9806577672780842
**COc1ccc(CCN(Cc2nc3ccccc3c(=O)[nH]2)C(=O)c2cccc(F)c2)cc1**	ASINEX	COc1ccc(CCN(Cc2nc3ccccc3c(=O)[nH]2)C(=O)c2cccc(F)c2)cc1	0.9966641	0.9986630415912237	0.9465323377242713	0.98061982872045
**O=c1[nH]c(-c2ccncc2)nc2c1CCN(Cc1ccc3nccnc3c1)C2**	ASINEX	O=c1[nH]c(-c2ccncc2)nc2c1CCN(Cc1ccc3nccnc3c1)C2	0.9991308	0.9994000495710172	0.9431060532940626	0.98054562944344
**O=c1[nH]c(N2CCOCC2)nc2c1CCC21CCN(CC2CCCCC2)CC1**	MOLPORT	O=c1[nH]c(N2CCOCC2)nc2c1CCC21CCN(CC2CCCCC2)CC1	0.9796682	0.999656832007706	0.96206443286371	0.9804631549624792
**NC(=O)c1ccc(Oc2ncncc2C2CCN(Cc3ccncc3)CC2)cc1**	ASINEX	NC(=O)c1ccc(Oc2ncncc2C2CCN(Cc3ccncc3)CC2)cc1	0.9768447	0.9999103086232816	0.9643834673308804	0.9803795013157244
**O=c1[nH]c(-c2ccccc2)nc2c1C1(CCCCC1)Cc1ccccc1-2**	ASINEX	O=c1[nH]c(-c2ccccc2)nc2c1C1(CCCCC1)Cc1ccccc1-2	0.9966897	0.9527900648111678	0.9912813742693308	0.9802537054396544
**CCc1ccc(CN2CCc3c(nc(-c4ccccn4)[nH]c3=O)C2)cc1**	ASINEX	CCc1ccc(CN2CCc3c(nc(-c4ccccn4)[nH]c3=O)C2)cc1	0.9902782	0.998534131196245	0.9514038409992256	0.9800720522031268
**O=C(NC1CCCCC1)N(Cc1nc2ccccc2c(=O)[nH]1)C1CCCC1**	ASINEX	O=C(NC1CCCCC1)N(Cc1nc2ccccc2c(=O)[nH]1)C1CCCC1	0.9679605	0.985685590720518	0.9864128827366704	0.9800196501108313
**O=C(NC1CCCCC1)N(Cc1nc2ccccc2c(=O)[nH]1)C1CCCCC1**	ASINEX	O=C(NC1CCCCC1)N(Cc1nc2ccccc2c(=O)[nH]1)C1CCCCC1	0.9679605	0.985685590720518	0.9864128827366704	0.9800196501108313
**O=c1[nH]c(CSc2nnc(-c3ccccc3O)n2Cc2ccccc2)nc2ccccc12**	ASINEX	O=c1[nH]c(CSc2nnc(-c3ccccc3O)n2Cc2ccccc2)nc2ccccc12	0.9765636	0.9998644444442744	0.963441322275276	0.9799564465343856
**O=C1NC2(CCN(C(=O)c3ccccn3)CC2)Nc2ccccc21**	ASINEX	O=C1NC2(CCN(C(=O)c3ccccn3)CC2)Nc2ccccc21	0.99815613	0.9997951080943168	0.9419173527711524	0.9799561970597808
**O=c1[nH]c(-c2ccncc2)nc2c1CCN(Cc1ccccc1)C2**	ASINEX	O=c1[nH]c(-c2ccncc2)nc2c1CCN(Cc1ccccc1)C2	0.9987185	0.997863835792972	0.9432757599037812	0.9799526986113608
**O=c1[nH]c(NC2CCN(C3CCCCC3)CC2)nc2c1CCCC2**	ASINEX	O=c1[nH]c(NC2CCN(C3CCCCC3)CC2)nc2c1CCCC2	0.99159616	0.984151360950612	0.9640968386411636	0.9799481206369864
**COc1cc(CN2CCc3c(nc(-c4ccncc4)[nH]c3=O)C2)ccc1OC(C)C**	ASINEX	COc1cc(CN2CCc3c(nc(-c4ccncc4)[nH]c3=O)C2)ccc1OC(C)C	0.99046046	0.9982958013801276	0.9504898770525008	0.9797487112834204
**O=c1[nH]c(-c2ccncc2)nc2c1CCN(Cc1ccccc1OCc1ccc(F)c1)C2**	ASINEX	O=c1[nH]c(-c2ccncc2)nc2c1CCN(Cc1ccccc1OCc1cccc(F)c1)C2	0.9991309	0.999945465082524	0.9401651299736168	0.9797471665768902
**COc1ccc(C(=O)N/N=C2\C(=O)Nc3ccccc32)cc1**	ASINEX	COc1ccc(C(=O)N/N=C2\C(=O)Nc3ccccc32)cc1	0.99403125	0.9624483208400908	0.982585536764441	0.9796883693604564
**COc1cc(C)c(CN2CCc3c(nc(-c4ccncc4)[nH]c3=O)C2)cc1OC**	ASINEX	COc1cc(C)c(CN2CCc3c(nc(-c4ccncc4)[nH]c3=O)C2)cc1OC	0.989181	0.999741074520124	0.950057372287336	0.9796598096401148
**COc1ccc(OC)c(CN2CCc3c(nc(-c4ccncc4)[nH]c3=O)C2)c1**	ASINEX	COc1ccc(OC)c(CN2CCc3c(nc(-c4ccncc4)[nH]c3=O)C2)c1	0.9979432	0.9998838055283638	0.9410406073292854	0.9796225451267948
**Cc1cccc(C)c1NC(=O)CSc1nnc(-c2ccccn2)n1-c1ccccc1Cl**	ASINEX	Cc1cccc(C)c1NC(=O)CSc1nnc(-c2ccccn2)n1-c1ccccc1Cl	0.99284744	0.96391059759836	0.9818501507287296	0.9795360636513476
**CN1CCOC(C(=O)N2CCC3(CCc4c3nc(-c3ccccc3)[nH]c4=O)CC2)C1**	MOLPORT	CN1CCOC(C(=O)N2CCC3(CCc4c3nc(-c3ccccc3)[nH]c4=O)CC2)C1	0.993707	0.9997833246652168	0.9451109568345488	0.979533760903008
**O=c1[nH]c(CSc2nnc(-c3ccccn3)o2)nc2ccccc12**	ASINEX	O=c1[nH]c(CSc2nnc(-c3ccccn3)o2)nc2ccccc12	0.99311066	0.9983644550770724	0.9468319324827024	0.9794356814326852
**CCC(CC)C(=O)N(Cc1nc2ccccc2c(=O)[nH]1)Cc1ccccc1OC**	ASINEX	CCC(CC)C(=O)N(Cc1nc2ccccc2c(=O)[nH]1)Cc1ccccc1OC	0.9695654	0.9955011043897872	0.9731672400914422	0.9794112453405855
**Cc1ncc(CN2CCc3c(nc(-c4ccncc4)[nH]c3=O)C2)cn1**	ASINEX	Cc1ncc(CN2CCc3c(nc(-c4ccncc4)[nH]c3=O)C2)cn1	0.99049044	0.9997971184513852	0.9477421445381734	0.9793432331811712

**OC(=O)c1ccc([nH]c1=O)-c1ccc(Cl)cc1**	MOLPORT	OC(=O)c1ccc([nH]c1=O)-c1ccc(Cl)cc1	0.987354	0.9841084259871922	0.9662661862329128	0.979242864253778
**CN(C)CCOc1ccc(C(=O)C2CCOCC2)cc1**	MOLPORT	CN(C)CCOc1ccc(C(=O)C2CCOCC2)cc1	0.98935145	0.9566479818752852	0.9915940047206244	0.979197812664284
**CCOc1ccc2[nH]c(=O)c(CN(Cc3ccccc3)C(=O)c3ccccc3OC)cc2c1**	ASINEX	CCOc1ccc2[nH]c(=O)c(CN(Cc3ccccc3)C(=O)c3ccccc3OC)cc2c1	0.9690373	0.9847783762934968	0.98364683222951	0.9791541676369414
**CCn1c(SCc2nc3ccccc3c(=O)[nH]2)nnc1-c1cccc(F)c1**	ASINEX	CCn1c(SCc2nc3ccccc3c(=O)[nH]2)nnc1-c1cccc(F)c1	0.9534611	0.9977188908585406	0.985942589616671	0.9790408636890334
**CCOc1ccc(C(=O)N(Cc2ccccc2)Cc2cc3cc(OCC)ccc3[nH]c2=O)cc1**	ASINEX	CCOc1ccc(C(=O)N(Cc2ccccc2)Cc2cc3cc(OCC)ccc3[nH]c2=O)cc1	0.9795593	0.972642751583402	0.9849128334691812	0.9790382957942002
**O=c1[nH]c(-c2ccccn2)nc2c1CCN(Cc1cnc(N3CCOCC3)nc1)C2**	ASINEX	O=c1[nH]c(-c2ccccn2)nc2c1CCN(Cc1cnc(N3CCOCC3)nc1)C2	0.99940693	0.9999471240842396	0.9377473254853296	0.9790337944513516
**O=c1[nH]c(-c2ccncc2)nc2c1CCN(Cc1cccn1-c1ccccc1F)C2**	ASINEX	O=c1[nH]c(-c2ccncc2)nc2c1CCN(Cc1cccn1-c1ccccc1F)C2	0.99978036	0.9999571324182552	0.937054725768552	0.9789307383569366
**O=C1NC2(CCN(C(=O)c3cccc4cccnc34)CC2)Nc2ccccc21**	ASINEX	O=C1NC2(CCN(C(=O)c3cccc4cccnc34)CC2)Nc2ccccc21	0.99968195	0.99999835657716	0.937004574806131	0.9788949603329232
**O=c1[nH]nc(-c2ccccc2)c2ccccc12**	ASINEX	O=c1[nH]nc(-c2ccccc2)c2ccccc12	0.9996754	0.9369810855745382	0.9999996680453096	0.9788853822414668
**CC1CN(c2nc3c(c(=O)[nH]2)CCN(Cc2ccccc2F)C3)CC(C)O1**	ASINEX	CC1CN(c2nc3c(c(=O)[nH]2)CCN(Cc2ccccc2F)C3)CC(C)O1	0.9988034	0.9996695872298412	0.9381076324423516	0.9788601989412274
**O=C1Nc2ccccc2/C1=N/NC(=O)c1ccc(-c2ccccc2)cc1**	ASINEX	O=C1Nc2ccccc2/C1=N/NC(=O)c1ccc(-c2ccccc2)cc1	0.98164314	0.9740663787399044	0.9807367927618176	0.9788154372725772
**CCn1c(CCNC(=O)c2ccccc2F)nnc1SCc1nc2ccccc2c(=O)[nH]1**	ASINEX	CCn1c(CCNC(=O)c2ccccc2F)nnc1SCc1nc2ccccc2c(=O)[nH]1	0.94824076	0.9996972484684586	0.9883370714518211	0.9787583589696016
**O=C(COCc1nc2ccccc2c(=O)[nH]1)Nc1ccc(C2CCCCC2)cc1**	ASINEX	O=C(COCc1nc2ccccc2c(=O)[nH]1)Nc1ccc(C2CCCCC2)cc1	0.9778977	0.997558440644944	0.9607202021637778	0.9787254488187784
**COc1ccc(CN2CCc3c(nc(-c4cccnc4)[nH]c3=O)C2)c(O)c1**	ASINEX	COc1ccc(CN2CCc3c(nc(-c4cccnc4)[nH]c3=O)C2)c(O)c1	0.9976279	0.9998669367715242	0.9385805703066894	0.9786918070100292
**CC(C)c1ccc2[nH]c(=O)c(CNCc3ccccc3)cc2c1**	ASINEX	CC(C)c1ccc2[nH]c(=O)c(CNCc3ccccc3)cc2c1	0.9719792	0.9921680827568734	0.9718483689243343	0.978665217507068
**O=C(CCn1nnc2ccccc21)N1CCc2c(nc(N3CCCC3)[nH]c2=O)C1**	ASINEX	O=C(CCn1nnc2ccccc21)N1CCc2c(nc(N3CCCC3)[nH]c2=O)C1	0.9990785	0.9991217174638732	0.9377094884805556	0.978636572712067
**COc1ccc2oc(C(=O)N3CCN(Cc4nc5ccccc5c(=O)[nH]4)CC3)cc2c1**	ASINEX	COc1ccc2oc(C(=O)N3CCN(Cc4nc5ccccc5c(=O)[nH]4)CC3)cc2c1	0.984219	0.9886204095204136	0.9630317821972488	0.9786237354540952
**CCOc1ccc(CN2CCc3c(nc(-c4cccnc4)[nH]c3=O)C2)cc1**	ASINEX	CCOc1ccc(CN2CCc3c(nc(-c4cccnc4)[nH]c3=O)C2)cc1	0.9873476	0.999270101190646	0.949213657542772	0.9786104538592189
**NC(=O)c1ccc(Oc2ncnc2C2CCN(C3CCOCC3)CC2)cc1**	ASINEX	NC(=O)c1ccc(Oc2ncnc2C2CCN(C3CCOCC3)CC2)cc1	0.9935889	0.9989722755176068	0.9432376173914974	0.9785996057723544
**COc1ccc(CCCc2nnc(SCc3nc4ccccc4c(=O)[nH]3)n2C)cc1C**	ASINEX	COc1ccc(CCCc2nnc(SCc3nc4ccccc4c(=O)[nH]3)n2C)cc1C	0.9476686	0.9980806704008583	0.9900110196298654	0.9785867673446834
**CC(Oc1ccccc1F)C(=O)N1CCN(Cc2nc3ccccc3c(=O)[nH]2)CC1**	ASINEX	CC(Oc1ccccc1F)C(=O)N1CCN(Cc2nc3ccccc3c(=O)[nH]2)CC1	0.98518074	0.9938539170399884	0.9561989718529248	0.978411208160329
**O=c1[nH]cc(-c2ccccc2)cc1CNc1cccc2ncccc12**	ASINEX	O=c1[nH]cc(-c2ccccc2)cc1CNc1cccc2ncccc12	0.989956	0.9960727874739634	0.9491522862804914	0.9783936983544512
**O=C(CO)N1CCC2(CCc3c2nc(N2CCOCC2)[nH]c3=O)CC1**	MOLPORT	O=C(CO)N1CCC2(CCc3c2nc(N2CCOCC2)[nH]c3=O)CC1	0.99420255	0.999081579625223	0.9417791495339184	0.978354427795021
**Cc1cc(CN2CCc3c(nc(-c4ccncc4)[nH]c3=O)C2)c(C)n1-c1ccccc1F**	ASINEX	Cc1cc(CN2CCc3c(nc(-c4ccncc4)[nH]c3=O)C2)c(C)n1-c1ccccc1F	0.9978484	0.9999984852473456	0.9371867060253316	0.9783445276018584
**COc1cccc(CN2CCc3c(nc(-c4cccnc4)[nH]c3=O)C2)c1O**	ASINEX	COc1cccc(CN2CCc3c(nc(-c4cccnc4)[nH]c3=O)C2)c1O	0.9982936	0.9995755228230756	0.9371382865026402	0.9783357959834804
**COc1ccc(F)c(CN2CCc3c(nc(-c4cccnc4)[nH]c3=O)C2)c1**	ASINEX	COc1ccc(F)c(CN2CCc3c(nc(-c4cccnc4)[nH]c3=O)C2)c1	0.99868387	0.999934986239824	0.9362445963907826	0.9782878174897736
**CCOc1ccc2[nH]c(=O)c(CN(Cc3ccccc3OC)C(=O)CC)cc2c1**	ASINEX	CCOc1ccc2[nH]c(=O)c(CN(Cc3ccccc3OC)C(=O)CC)cc2c1	0.96452326	0.9892350196745155	0.980935251371412	0.9782311756236568
**CN(Cc1cc(-c2ccccc2)c[nH]c1=O)Cc1cccc2ncccc12**	ASINEX	CN(Cc1cc(-c2ccccc2)c[nH]c1=O)Cc1cccc2ncccc12	0.99471855	0.992411492354514	0.9470544282856884	0.9780614907586482
**CCOc1ccc2[nH]c(=O)c(CN(Cc3ccccc3)C(=O)CC)cc2c1**	ASINEX	CCOc1ccc2[nH]c(=O)c(CN(Cc3ccccc3)C(=O)CC)cc2c1	0.9630488	0.9917881470971222	0.9792427843087452	0.9780265823777003
**CC1CN(c2nc3c(c(=O)[nH]2)CCN(Cc2ccc(C#N)cc2)C3)CC(C)O1**	ASINEX	CC1CN(c2nc3c(c(=O)[nH]2)CCN(Cc2ccc(C#N)cc2)C3)CC(C)O1	0.99728644	0.999025812026021	0.9376687594865002	0.9779936701514924
**CCc1ccc(NC(=S)N(Cc2ccccc2)Cc2nc3ccccc3c(=O)[nH]2)cc1**	ASINEX	CCc1ccc(NC(=S)N(Cc2ccccc2)Cc2nc3ccccc3c(=O)[nH]2)cc1	0.9671659	0.9846941237954604	0.9817819999238012	0.9778806703583554
**O=c1[nH]c(C2CCCCC2)nc2ccccc12**	ASINEX	O=c1[nH]c(C2CCCCC2)nc2ccccc12	0.98374635	0.9497786969951664	0.9999973566408564	0.9778408011491924

**CC1CCN(c2nc3c(c(=O)[nH]2)CCN(Cc2ccccn2)C3)CC1**	ASINEX	CC1CCN(c2nc3c(c(=O)[nH]2)CCN(Cc2ccccn2)C3)CC1	0.99918824	0.999688221590491	0.9346399636878918	0.9778388098737288
**O=C1Nc2ccccc2/C1=C/c1nc2ccccc2c(=O)n1-c1ccc(C(=O)O)cc1**	ASINEX	O=C1Nc2ccccc2/C1=C/c1nc2ccccc2c(=O)n1-c1ccc(C(=O)O)cc1	0.96793526	0.9998017869938088	0.9655080357757012	0.9777483622933584
**O=C1NC2(CCN(C(=O)c3cccc4ncccc34)CC2)Nc2ccccc21**	ASINEX	O=C1NC2(CCN(C(=O)c3cccc4ncccc34)CC2)Nc2ccccc21	0.99947244	0.9999814742348744	0.9337307574739144	0.9777282236659608
**Cc1oc2ccccc2c1CN1CCc2c(nc(N3CCCC3)[nH]c2=O)C1**	ASINEX	Cc1oc2ccccc2c1CN1CCc2c(nc(N3CCCC3)[nH]c2=O)C1	0.993298	0.9994513775450764	0.940267030570322	0.9776721340840694
**CC(=O)c1ccc(NC(=O)C(C)Sc2nc3c(c(=O)[nH]2)CCCC3)cc1**	ASINEX	CC(=O)c1ccc(NC(=O)C(C)Sc2nc3c(c(=O)[nH]2)CCCC3)cc1	0.9694448	0.9857915929239616	0.9777703861042596	0.9776689301241226
**O=C(N1CCOCC1)N1CCc2nc(N3CCCC3)[nH]c(=O)c2C1**	ASINEX	O=C(N1CCOCC1)N1CCc2nc(N3CCCC3)[nH]c(=O)c2C1	0.99950695	0.964297220871988	0.96897553257311	0.9775932346078386
**CCn1c(SCc2nc3ccccc3c(=O)[nH]2)nnc1-c1cccc(OC)c1**	ASINEX	CCn1c(SCc2nc3ccccc3c(=O)[nH]2)nnc1-c1cccc(OC)c1	0.9408843	0.998761451782779	0.9930651871285372	0.9775703103456728
**O=C(C1CC1)N(Cc1nc2ccccc2c(=O)[nH]1)C1CCCC1**	ASINEX	O=C(C1CC1)N(Cc1nc2ccccc2c(=O)[nH]1)C1CCCC1	0.9799652	0.990814054597989	0.9618981045781294	0.9775591230456854
**O=c1[nH]c(CSc2nnc(Cc3ccccc3)o2)nc2ccccc12**	ASINEX	O=c1[nH]c(CSc2nnc(Cc3ccccc3)o2)nc2ccccc12	0.9963965	0.9922692710952496	0.9439904033682254	0.9775520521514296
**O=c1[nH]c(-c2ccncc2)nc2c1CCN(Cc1cn(CCCO)c3ccccc13)C2**	ASINEX	O=c1[nH]c(-c2ccncc2)nc2c1CCN(Cc1cn(CCCO)c3ccccc13)C2	0.99967456	0.9999904886080276	0.9329860727874631	0.9775503733450058
**CC1CN(c2nc3c(c(=O)[nH]2)CCN(CC2CCCCC2)C3)CC(C)O1**	ASINEX	CC1CN(c2nc3c(c(=O)[nH]2)CCN(CC2CCCCC2)C3)CC(C)O1	0.9944659	0.9996087393960328	0.9384616625448606	0.9775120964958108
**Cc1cc2c(cc1C(=O)NCCc1nc3ccccc3c(=O)[nH]1)OCCO2**	MOLPORT	Cc1cc2c(cc1C(=O)NCCc1nc3ccccc3c(=O)[nH]1)OCCO2	0.93246585	0.9999053388444602	0.9999827848412086	0.977451324997528
**CCN(CC)C(=O)CSc1nnc(-c2ccccn2)n1-c1ccccc1Cl**	ASINEX	CCN(CC)C(=O)CSc1nnc(-c2ccccn2)n1-c1ccccc1Cl	0.98575264	0.9854066279494864	0.961187222409708	0.9774488308379626
**Cc1c(C(=O)N2CCc3c(nc(N4CCC(C)CC4)[nH]c3=O)C2)cnn1C**	ASINEX	Cc1c(C(=O)N2CCc3c(nc(N4CCC(C)CC4)[nH]c3=O)C2)cnn1C	0.98845184	0.9967130922572	0.9466409310970728	0.9772686206158732
**CC(C)n1cc(CN2CCc3nc(N4CCCC4)[nH]c(=O)c3C2)c2ccccc21**	ASINEX	CC(C)n1cc(CN2CCc3nc(N4CCCC4)[nH]c(=O)c3C2)c2ccccc21	0.99705076	0.9966535087400314	0.9380482408332106	0.9772508372499186
**O=c1[nH]c(-c2cccnc2)nc2c1CCN(Cc1ccc(F)cc1)C2**	ASINEX	O=c1[nH]c(-c2cccnc2)nc2c1CCN(Cc1ccc(F)cc1)C2	0.99836284	0.998088914217598	0.9352990297872068	0.9772502610755864
**O=C(Cc1n[nH]c(=O)c2ccccc12)N1CCCCC1CN1CCOCC1**	ASINEX	O=C(Cc1n[nH]c(=O)c2ccccc12)N1CCCCC1CN1CCOCC1	0.9681571	0.998720165826979	0.9646392921017042	0.9771721901757008
**Cc1cc(C)c2cc(CNc3cccnc3O)c(=O)[nH]c2c1**	ASINEX	Cc1cc(C)c2cc(CNc3cccnc3O)c(=O)[nH]c2c1	0.94319487	0.9972715490149672	0.9906990272462528	0.9770551474805466
**CCOc1ccc2[nH]c(=O)c(CN(Cc3ccc(OC)cc3)C(=O)CC)cc2c1**	ASINEX	CCOc1ccc2[nH]c(=O)c(CN(Cc3ccc(OC)cc3)C(=O)CC)cc2c1	0.9501715	0.9976114585292044	0.9831133410186396	0.9769654233966446
**CC1CCCN(c2nc3c(c(=O)[nH]2)CN(Cc2cccc4cccnc24)CC3)C1**	ASINEX	CC1CCCN(c2nc3c(c(=O)[nH]2)CN(Cc2cccc4cccnc24)CC3)C1	0.99877506	0.9999603487708308	0.9320365552011212	0.9769239896390576
**N=C1CNc2ccccc2C(=O)O1**	ASINEX	N=C1CNc2ccccc2C(=O)O1	0.96960336	0.9989921069913004	0.9618251385947268	0.9768068684284256
**Cc1oc2ccccc2c1CN1CCc2c(nc(N3CCOCC3)[nH]c2=O)C1**	MOLPORT	Cc1oc2ccccc2c1CN1CCc2c(nc(N3CCOCC3)[nH]c2=O)C1	0.9980526	0.999717648778444	0.9326007654610484	0.9767903370951304
**CC(=O)N(Cc1nc2ccccc2c(=O)[nH]1)C1CCCC1**	ASINEX	CC(=O)N(Cc1nc2ccccc2c(=O)[nH]1)C1CCCC1	0.9473962	0.9898625539761958	0.9929668961433212	0.9767418896320732
**CC(Sc1nc2c(c(=O)[nH]1)CCCC2)C(=O)Nc1ccc(Cl)cc1**	ASINEX	CC(Sc1nc2c(c(=O)[nH]1)CCCC2)C(=O)Nc1ccc(Cl)cc1	0.9510708	0.9870206521199562	0.9919566326785076	0.9766826901069748
**O=c1cc(-c2ccccc2)c2cc3c(cc2[nH]1)OCCO3**	MOLPORT	O=c1cc(-c2ccccc2)c2cc3c(cc2[nH]1)OCCO3	0.9927061	0.9420917743180566	0.9952092541890076	0.976669049507085
**O=C(C1COCCO1)N1CCc2c(nc(-c3ccccn3)[nH]c2=O)C1**	MOLPORT	O=C(C1COCCO1)N1CCc2c(nc(-c3ccccn3)[nH]c2=O)C1	0.99972767	0.9997652836084184	0.9304734780858442	0.9766554760240947
**CC1CCN(c2nc3c(c(=O)[nH]2)CN(Cc2cc(F)ccc2F)CC3)CC1**	ASINEX	CC1CCN(c2nc3c(c(=O)[nH]2)CN(Cc2cc(F)ccc2F)CC3)CC1	0.9976635	0.995025571012728	0.9372370925857704	0.9766420538411008
**Cn1c(CC(=O)Nc2ccccc2)nnc1SCc1nc2ccccc2c(=O)[nH]1**	ASINEX	Cn1c(CC(=O)Nc2ccccc2)nnc1SCc1nc2ccccc2c(=O)[nH]1	0.94753724	0.997477183511625	0.9846785422057224	0.9765643230278628
**COc1ccc(CN2CCc3c(nc(-c4ccccn4)[nH]c3=O)C2)c(OC)c1**	ASINEX	COc1ccc(CN2CCc3c(nc(-c4ccccn4)[nH]c3=O)C2)c(OC)c1	0.99893266	0.9999120310192068	0.930709992793151	0.9765182278127884
**Cc1ccc(CN2CCc3c(nc(N4CCOCC4)[nH]c3=O)C2)o1**	ASINEX	Cc1ccc(CN2CCc3c(nc(N4CCOCC4)[nH]c3=O)C2)o1	0.9978136	0.998546441713902	0.9327295004750494	0.9763631748697668
**O=C(c1cccc(F)c1)N(Cc1nc2ccccc2c(=O)[nH]1)C1CCCC1**	ASINEX	O=C(c1cccc(F)c1)N(Cc1nc2ccccc2c(=O)[nH]1)C1CCCC1	0.9814556	0.9789765438458836	0.9685559229424192	0.9763293636306164
**O=C(CN1CCc2nc(N3CCCC3)[nH]c(=O)c2C1)Nc1ccc(C2CCCCC2)cc1**	ASINEX	O=C(CN1CCc2nc(N3CCCC3)[nH]c(=O)c2C1)Nc1ccc(C2CCCCC2)cc1	0.9986235	0.9679651969281174	0.9623170009624477	0.9763018960747072

**O=C(NCCN1CCOCC1)c1c(O)c2ccccc2[nH]c1=O**	ASINEX	O=C(NCCN1CCOCC1)c1c(O)c2ccccc2[nH]c1=O	0.97232705	0.9731105694864408	0.9834383523986804	0.9762919918106756
**CCc1ncc(CN2CCc3c(nc(-c4ccncc4)[nH]c3=O)C2)cn1**	ASINEX	CCc1ncc(CN2CCc3c(nc(-c4ccncc4)[nH]c3=O)C2)cn1	0.994468	0.999895321357334	0.934470807789909	0.9762780342854498
**CCOc1ccc2[nH]c(=O)c(CNCc3ccc(F)cc3)cc2c1**	ASINEX	CCOc1ccc2[nH]c(=O)c(CNCc3ccc(F)cc3)cc2c1	0.93609893	0.997452407947304	0.9952404571397075	0.9762639327689738
**COc1ccc(-c2ccc(C(=O)Nc3cccc(OC(C)C)c3)c(=O)[nH]2)cc1**	ASINEX	COc1ccc(-c2ccc(C(=O)Nc3cccc(OC(C)C)c3)c(=O)[nH]2)cc1	0.94876415	0.9854562740703952	0.9945585892420492	0.9762596695622476
**O=C(NC1CCCCC1)N(Cc1ccc(Cl)cc1)Cc1nc2ccccc2c(=O)[nH]1**	ASINEX	O=C(NC1CCCCC1)N(Cc1ccc(Cl)cc1)Cc1nc2ccccc2c(=O)[nH]1	0.9519382	0.9985023699773438	0.9783001728785692	0.97624691825793
**Cc1nn(C)cc1C(=O)N1CCc2c(nc(N3CCC(C)CC3)[nH]c2=O)C1**	ASINEX	Cc1nn(C)cc1C(=O)N1CCc2c(nc(N3CCC(C)CC3)[nH]c2=O)C1	0.9722448	0.9952351802673493	0.9610358093268128	0.9761719295770924
**CC(C)(C)C(=O)N(Cc1nc2ccccc2c(=O)[nH]1)C1CCCCC1**	ASINEX	CC(C)(C)C(=O)N(Cc1nc2ccccc2c(=O)[nH]1)C1CCCCC1	0.95707464	0.9910176358414924	0.9801193882390002	0.9760705554206296
**COc1ccc(CN(Cc2cc3c(C)ccc(C)c3[nH]c2=O)C(=O)c2ccc(OC)c(OC)c2)cc1**	ASINEX	COc1ccc(CN(Cc2cc3c(C)ccc(C)c3[nH]c2=O)C(=O)c2ccc(OC)c(OC)c2)cc1	0.9943252	0.9876295451380688	0.946223749423836	0.9760595050489248
**O=C(CN1CCc2nc(N3CCCC3)[nH]c(=O)c2C1)Nc1cccc(F)c1**	ASINEX	O=C(CN1CCc2nc(N3CCCC3)[nH]c(=O)c2C1)Nc1cccc(F)c1	0.99886703	0.979476504086726	0.9494330281511728	0.9759255223833362
**Cc1cc(C)c2cc(CNCc3ccc(Cl)cc3)c(=O)[nH]c2c1**	ASINEX	Cc1cc(C)c2cc(CNCc3ccc(Cl)cc3)c(=O)[nH]c2c1	0.97163683	0.9851841679610436	0.9708082309071744	0.9758764102095414
**Cc1ccc(CN2CCc3c(nc(N4CCCC4)[nH]c3=O)C2)o1**	ASINEX	Cc1ccc(CN2CCc3c(nc(N4CCCC4)[nH]c3=O)C2)o1	0.99306244	0.9971787809503012	0.9373740252473216	0.9758717475927604
**O=c1[nH]c(-c2ccccn2)nc2c1CCN(Cc1cnc(N3CCCCC3)nc1)C2**	ASINEX	O=c1[nH]c(-c2ccccn2)nc2c1CCN(Cc1cnc(N3CCCCC3)nc1)C2	0.9956999	0.999892701093655	0.9319113015478444	0.9758346283829412
**Oc1nc(-c2ccncc2)nc2c1CCN(Cc1ccc(OC(F)(F)F)cc1)CC2**	ASINEX	Oc1nc(-c2ccncc2)nc2c1CCN(Cc1ccc(OC(F)(F)F)cc1)CC2	0.9417041	0.997077941610484	0.9886266653029712	0.9758029004411326
**O=C1NC2(CCN(C(=O)c3cccc4nccnc34)CC2)Nc2ccccc21**	ASINEX	O=C1NC2(CCN(C(=O)c3cccc4nccnc34)CC2)Nc2ccccc21	0.9996269	0.9999779003426132	0.9277608576526244	0.9757885443063148
**O=C(O)c1ccc(OCc2ccc(F)cc2)cc1**	ASINEX	O=C(O)c1ccc(OCc2ccc(F)cc2)cc1	0.94999427	0.9770198038087572	0.9999938362196766	0.9756693020205356
**CCCCCn1c(=O)c(-c2nc3ccccc3c(=O)[nH]2)c(O)c2ccccc21**	ASINEX	CCCCCn1c(=O)c(-c2nc3ccccc3c(=O)[nH]2)c(O)c2ccccc21	0.9632971	0.9980218631113998	0.9656220335086444	0.9756470084324708
**CCOc1ccc(C(=O)N2CCc3nc(-c4ccncc4)nc(O)c3CC2)cc1**	ASINEX	CCOc1ccc(C(=O)N2CCc3nc(-c4ccncc4)nc(O)c3CC2)cc1	0.92903304	0.9976111681979188	0.9999992884979856	0.9755478325654235
**O=C(Cc1n[nH]c(=O)c2ccccc12)N1CCCC(c2[nH]ncc2-c2ccncc2)C1**	ASINEX	O=C(Cc1n[nH]c(=O)c2ccccc12)N1CCCC(c2[nH]ncc2-c2ccncc2)C1	0.98572123	0.9956614845043846	0.9450708245100616	0.9754845131737812
**O=c1[nH]c(-c2ccccn2)nc2c1CCN(Cc1ccnc3ccccc13)C2**	ASINEX	O=c1[nH]c(-c2ccccn2)nc2c1CCN(Cc1ccnc3ccccc13)C2	0.99925417	0.9996928223589804	0.9274109144289256	0.9754526346226106
**Cc1ccsc1CN1CCc2c(nc(N3CCOCC3)[nH]c2=O)C1**	ASINEX	Cc1ccsc1CN1CCc2c(nc(N3CCOCC3)[nH]c2=O)C1	0.9956975	0.9997839763617884	0.930670984995712	0.9753841532263444
**COc1ccc(C2c3c([nH]c(=O)[nH]c3=O)Nc3ccc4ccccc4c32)cc1**	ASINEX	COc1ccc(C2c3c([nH]c(=O)[nH]c3=O)Nc3ccc4ccccc4c32)cc1	0.9969408	0.9934469962184548	0.9357114621104988	0.9753664166452856
**CCc1ncnc(N2CCC3(CCc4c3nc(N3CCOCC3)[nH]c4=O)CC2)c1F**	MOLPORT	CCc1ncnc(N2CCC3(CCc4c3nc(N3CCOCC3)[nH]c4=O)CC2)c1F	0.98617095	0.999929560442976	0.939838505788918	0.9753130045945404
**CC1CCN(c2nc3c(c(=O)[nH]2)CCN(Cc2cccc(C#N)c2)C3)CC1**	ASINEX	CC1CCN(c2nc3c(c(=O)[nH]2)CCN(Cc2cccc(C#N)c2)C3)CC1	0.9990466	0.999856822236735	0.9268783796993296	0.9752606085476274
**O=C(Cc1n[nH]c(=O)c2ccccc12)N1CCC(c2onc3c2CCCC3)CC1**	ASINEX	O=C(Cc1n[nH]c(=O)c2ccccc12)N1CCC(c2onc3c2CCCC3)CC1	0.98357207	0.9748276524908572	0.9673501994808908	0.9752499726006596
**COc1ccc(C(=O)N(Cc2ccccc2)Cc2cc3c(C)cc(C)cc3[nH]c2=O)cc1OC**	ASINEX	COc1ccc(C(=O)N(Cc2ccccc2)Cc2cc3c(C)cc(C)cc3[nH]c2=O)cc1OC	0.98841	0.9708640503525344	0.9664044068209195	0.975226151068723
**COc1ccc(-n2c(C)cc(CN3CCc4c(nc(-c5ccncc5)[nH]c4=O)C3)c2C)cc1**	ASINEX	COc1ccc(-n2c(C)cc(CN3CCc4c(nc(-c5ccncc5)[nH]c4=O)C3)c2C)cc1	0.9937186	0.999995606971334	0.931800421721836	0.9751715509360532
**COc1ccc2[nH]c(=O)c(CNc3cc(-c4ccccc4)ccc3OC)cc2c1**	ASINEX	COc1ccc2[nH]c(=O)c(CNc3cc(-c4ccccc4)ccc3OC)cc2c1	0.9692495	0.9833918192884844	0.972857124684188	0.9751661436319634
**COc1ccc(C(=O)C2CCN(C(=O)Nc3ccccc3F)CC2)cc1**	ASINEX	COc1ccc(C(=O)C2CCN(C(=O)Nc3ccccc3F)CC2)cc1	0.95083946	0.9846938745241692	0.9896787628423048	0.9750706990875204
**COc1ccccc1C(=O)N(Cc1ccccc1)Cc1cc2c(C)cc(C)cc2[nH]c1=O**	ASINEX	COc1ccccc1C(=O)N(Cc1ccccc1)Cc1cc2c(C)cc(C)cc2[nH]c1=O	0.977242	0.9719403348606124	0.9758545706201368	0.9750122994770628
**CC(C)n1c(SCc2nc3ccccc3c(=O)[nH]2)nnc1-c1ccco1**	ASINEX	CC(C)n1c(SCc2nc3ccccc3c(=O)[nH]2)nnc1-c1ccco1	0.93865716	0.9996588865787578	0.9865361143645563	0.9749507218389946
**O=C(O)c1c[nH]c(=O)[nH]c1=O**	ASINEX	O=C(O)c1c[nH]c(=O)[nH]c1=O	0.9835324	0.9608980828945396	0.9803430069173332	0.9749245061844426
**CC1CCCN(c2nc3c(c(=O)[nH]2)CN(C(=O)C(C)(C)c2ccc(F)cc2)CC3)C1**	ASINEX	CC1CCCN(c2nc3c(c(=O)[nH]2)CN(C(=O)C(C)(C)c2ccc(F)cc2)CC3)C1	0.9963425	0.967053901931254	0.9612038760630018	0.9748667527256346

**CC1CN(c2nc3c(c(=O)[nH]2)CCN(Cc2ccnc4ccccc24)C3)CC(C)O1**	ASINEX	CC1CN(c2nc3c(c(=O)[nH]2)CCN(Cc2ccnc4ccccc24)C3)CC(C)O1	0.9988732	0.9995146821911576	0.925835850725034	0.9747412357022376
**Cc1cc(C)c2cc(CNCc3ccccc3Cl)c(=O)[nH]c2c1**	ASINEX	Cc1cc(C)c2cc(CNCc3ccccc3Cl)c(=O)[nH]c2c1	0.9909741	0.9748066508180264	0.9583337548129216	0.9747048446257516
**CCOc1ccc2[nH]c(=O)c(CNCc3ccc(OC)cc3)cc2c1**	ASINEX	CCOc1ccc2[nH]c(=O)c(CNCc3ccc(OC)cc3)cc2c1	0.92697877	0.9993555610929156	0.9974271002253372	0.9745871427454784
**CCOc1ccccc1N1CC(=O)C(c2nc3ccccc3c(=O)[nH]2)=C1N**	ASINEX	CCOc1ccccc1N1CC(=O)C(c2nc3ccccc3c(=O)[nH]2)=C1N	0.94167	0.992223571495536	0.9898282426854678	0.9745739382447116
**C=CCn1c(COc2ccc(OC)cc2)nnc1SCc1nc2ccccc2c(=O)[nH]1**	ASINEX	C=CCn1c(COc2ccc(OC)cc2)nnc1SCc1nc2ccccc2c(=O)[nH]1	0.9387254	0.9997988793126924	0.9849801382943868	0.9745014764996722
**COc1cccc(CNCc2cc3c(C)ccc(C)c3[nH]c2=O)c1**	ASINEX	COc1cccc(CNCc2cc3c(C)ccc(C)c3[nH]c2=O)c1	0.9854109	0.9913116014893228	0.9462821043310384	0.9743348781821858
**COc1ccc2c3c([nH]c2c1)C(=O)NCC3**	LOTUS	COc1ccc2c3c([nH]c2c1)C(=O)NCC3	0.9333192	0.9930218153847948	0.996079253381654	0.9741400932575378
**COc1ccc(-c2ccc(C(=O)N3CCC(Oc4cccc(F)c4)CC3)c(=O)[nH]2)cc1**	ASINEX	COc1ccc(-c2ccc(C(=O)N3CCC(Oc4cccc(F)c4)CC3)c(=O)[nH]2)cc1	0.98158616	0.9997852191299867	0.9408285180168636	0.9740666318074848
**O=C(CCn1nnc2ccccc21)N1CCc2c(nc(N3CCCCC3)[nH]c2=O)C1**	ASINEX	O=C(CCn1nnc2ccccc21)N1CCc2c(nc(N3CCCCC3)[nH]c2=O)C1	0.9990785	0.9989629824941038	0.924044378767904	0.9740286244845936
**O=C(c1ccc(Br)cc1)N(Cc1nc2ccccc2c(=O)[nH]1)C1CCCC1**	ASINEX	O=C(c1ccc(Br)cc1)N(Cc1nc2ccccc2c(=O)[nH]1)C1CCCC1	0.93686056	0.9938299494209554	0.9911548471231966	0.973948452638334
**O=C(NCCc1nc2ccccc2c(=O)[nH]1)c1scc2c1OCCO2**	MOLPORT	O=C(NCCc1nc2ccccc2c(=O)[nH]1)c1scc2c1OCCO2	0.9715398	0.997346565410346	0.9525807680074628	0.9738223762721736
**COc1ccc2[nH]c(CN3CCc4c(nc(-c5ccncc5)[nH]c4=O)C3)c(C)c2c1**	ASINEX	COc1ccc2[nH]c(CN3CCc4c(nc(-c5ccncc5)[nH]c4=O)C3)c(C)c2c1	0.98347497	0.9999974693048708	0.9379606952253904	0.9738110447980508
**O=C(Cc1n[nH]c(=O)c2ccccc12)NCc1cccc(Oc2ccccc2)c1**	ASINEX	O=C(Cc1n[nH]c(=O)c2ccccc12)NCc1cccc(Oc2ccccc2)c1	0.9940507	0.9686280823468666	0.9587033960722956	0.973794053336732
**CC(=O)Oc1ccc(CN2CCc3c(nc(N4CC(C)OC(C)C4)[nH]c3=O)C2)cc1**	ASINEX	CC(=O)Oc1ccc(CN2CCc3c(nc(N4CC(C)OC(C)C4)[nH]c3=O)C2)cc1	0.99386024	0.997895555318507	0.929552062972291	0.9737692876805916
**O=C(Cc1n[nH]c(=O)c2ccccc12)N1CCC(n2nnc(-c3ccccc3)n2)CC1**	ASINEX	O=C(Cc1n[nH]c(=O)c2ccccc12)N1CCC(n2nnc(-c3ccccc3)n2)CC1	0.9844832	0.9686120708936165	0.9681975358104148	0.9737642630447662
**CN1CCN(c2nc(-c3ccc4ccccc4c3)cc(=O)[nH]2)CC1**	ASINEX	CN1CCN(c2nc(-c3ccc4ccccc4c3)cc(=O)[nH]2)CC1	0.9352333	0.9855488203975156	0.999997861828234	0.973593325729862
**CCOc1ccc2[nH]c(=O)c(CN(Cc3ccc(F)cc3)C(=O)c3ccccc3Cl)cc2c1**	ASINEX	CCOc1ccc2[nH]c(=O)c(CN(Cc3ccc(F)cc3)C(=O)c3ccccc3Cl)cc2c1	0.98062253	0.9870718013960385	0.9529384669137504	0.9735442660977696
**Cc1oc2ccccc2c1CN1CCc2c(nc(N3CCCCC3)[nH]c2=O)C1**	ASINEX	Cc1oc2ccccc2c1CN1CCc2c(nc(N3CCCCC3)[nH]c2=O)C1	0.993298	0.99935218452429	0.9273316665939147	0.9733272817516716
**COc1ccccc1CN(Cc1nc2ccccc2c(=O)[nH]1)C(=O)C1CCC1**	ASINEX	COc1ccccc1CN(Cc1nc2ccccc2c(=O)[nH]1)C(=O)C1CCC1	0.9752876	0.99876769539281	0.9458932372058164	0.9733161829505084
**CC1CN(c2nc3c(c(=O)[nH]2)CCN(Cc2ccc(OC(F)(F)F)cc2)C3)CC(C)O1**	ASINEX	CC1CN(c2nc3c(c(=O)[nH]2)CCN(Cc2ccc(OC(F)(F)F)cc2)C3)CC(C)O1	0.99738353	0.9994285831040716	0.9230286479773512	0.973280255329906
**COc1ccc(CN(Cc2cc3c(C)cc(C)cc3[nH]c2=O)C(=S)NCc2ccccc2)cc1**	ASINEX	COc1ccc(CN(Cc2cc3c(C)cc(C)cc3[nH]c2=O)C(=S)NCc2ccccc2)cc1	0.9684995	0.9847770477445886	0.9665227280304952	0.973266419151031
**COc1ccc(C(=O)C2CCN(Cc3nnnn3Cc3cccs3)CC2)cc1**	ASINEX	COc1ccc(C(=O)C2CCN(Cc3nnnn3Cc3cccs3)CC2)cc1	0.9717637	0.9885676244539277	0.9594636792237932	0.9732649913740696
**CC1CN(c2nc3c(c(=O)[nH]2)CCN(Cc2cccs2)C3)CC(C)O1**	ASINEX	CC1CN(c2nc3c(c(=O)[nH]2)CCN(Cc2cccs2)C3)CC(C)O1	0.995509	0.9986666827326468	0.9256105472858042	0.973262086151068
**CC1CN(c2nc3c(c(=O)[nH]2)CCN(Cc2ccccc2Cl)C3)CC(C)O1**	ASINEX	CC1CN(c2nc3c(c(=O)[nH]2)CCN(Cc2ccccc2Cl)C3)CC(C)O1	0.9979103	0.9996181895017796	0.9220248426089336	0.9731844509565112
**CCOc1ccc2[nH]c(=O)c(CN(Cc3ccc(F)cc3)C(=O)CC)cc2c1**	ASINEX	CCOc1ccc2[nH]c(=O)c(CN(Cc3ccc(F)cc3)C(=O)CC)cc2c1	0.95881265	0.9906060484478316	0.9701329188683048	0.9731838737781794
**Cc1ccc(-c2n[nH]c(CCNC(=O)CCc3n[nH]c(=O)c4ccccc34)n2)cc1**	ASINEX	Cc1ccc(-c2n[nH]c(CCNC(=O)CCc3n[nH]c(=O)c4ccccc34)n2)cc1	0.9917857	0.9999691565236154	0.927759276761767	0.9731713794583172
**O=C(CCCc1nc2ccccc2c(=O)[nH]1)N1CCC2(O)CCCCC2C1**	MOLPORT	O=C(CCCc1nc2ccccc2c(=O)[nH]1)N1CCC2(O)CCCCC2C1	0.9400016	0.9920529280807616	0.9874313256973432	0.9731619535731094
**O=c1[nH]c(-c2ccncc2)nc2c1CCN(Cc1c[nH]nc1-c1ccc(Cl)cc1)C2**	ASINEX	O=c1[nH]c(-c2ccncc2)nc2c1CCN(Cc1c[nH]nc1-c1ccc(Cl)cc1)C2	0.99869746	0.9990056667482275	0.9215992144248786	0.9731007802902764
**O=C1NC2(CCN(Cc3cc(F)ccc3F)CC2)Nc2ccccc21**	ASINEX	O=C1NC2(CCN(Cc3cc(F)ccc3F)CC2)Nc2ccccc21	0.9967102	0.9999734264292468	0.9225253304265676	0.973069646030694
**CCOc1ccc(C(=O)N(Cc2ccc(OC)cc2)Cc2cc3cc(OC)ccc3[nH]c2=O)cc1**	ASINEX	CCOc1ccc(C(=O)N(Cc2ccc(OC)cc2)Cc2cc3cc(OC)ccc3[nH]c2=O)cc1	0.96600527	0.9785204201433596	0.9743833814710476	0.9729696891090484
**Cc1nn(C)cc1CN1CCc2c(nc(N3CCOCC3)[nH]c2=O)C1**	ASINEX	Cc1nn(C)cc1CN1CCc2c(nc(N3CCOCC3)[nH]c2=O)C1	0.99438643	0.9980361197417946	0.9264684019000696	0.9729636520656392
**O=c1[nH]c(-c2ccccn2)nc2c1CCN(Cc1cccc(Oc3ccccc3)c1)C2**	ASINEX	O=c1[nH]c(-c2ccccn2)nc2c1CCN(Cc1cccc(Oc3ccccc3)c1)C2	0.9992613	0.9998564088578435	0.9196804600144872	0.9729327295032096

**CCOc1ccc2[nH]c(=O)c(CN(Cc3cccs3)C(=O)c3ccc(C(C)(C)C)cc3)cc2c1**	ASINEX	CCOc1ccc2[nH]c(=O)c(CN(Cc3cccs3)C(=O)c3ccc(C(C)(C)C)cc3)cc2c1	0.93938524	0.9994068215361344	0.9796938499366232	0.9728286355941196
**Cc1cc(=O)[nH]c(SCc2nc(O)c3ccccc3n2)n1**	ASINEX	Cc1cc(=O)[nH]c(SCc2nc(O)c3ccccc3n2)n1	0.93443936	0.9998281768788484	0.9841738415734208	0.972813793182566
**O=C(Nc1ccc(Cl)cc1)N(Cc1nc2ccccc2c(=O)[nH]1)C1CCCCC1**	ASINEX	O=C(Nc1ccc(Cl)cc1)N(Cc1nc2ccccc2c(=O)[nH]1)C1CCCCC1	0.9473302	0.9876088006146736	0.98347849619907	0.9728058245480116
**CCOc1ccc2[nH]c(=O)c(CNc3cc(-c4ccccc4)ccc3OC)cc2c1**	ASINEX	CCOc1ccc2[nH]c(=O)c(CNc3cc(-c4ccccc4)ccc3OC)cc2c1	0.9421527	0.9934737653804304	0.9827003343453456	0.9727755928974328
**O=c1[nH]c(-c2ccncc2)nc2c1CCN(Cc1cccn1-c1ccccc1Cl)C2**	ASINEX	O=c1[nH]c(-c2ccncc2)nc2c1CCN(Cc1cccn1-c1ccccc1Cl)C2	0.9990088	0.9998651359466988	0.9191043244026094	0.9726594117022312
**CCC(=O)N(Cc1ccc(OC)cc1)Cc1cc2c(C)ccc(C)c2[nH]c1=O**	ASINEX	CCC(=O)N(Cc1ccc(OC)cc1)Cc1cc2c(C)ccc(C)c2[nH]c1=O	0.9706731	0.9935454160945288	0.953737681085337	0.9726520604796328
**O=C(c1ccc(Br)cc1)N(Cc1nc2ccccc2c(=O)[nH]1)C1CCCCC1**	ASINEX	O=C(c1ccc(Br)cc1)N(Cc1nc2ccccc2c(=O)[nH]1)C1CCCCC1	0.93686056	0.9927217823360576	0.9882958777585644	0.972626073821824
**Cc1cc(C(=O)N2CCc3nc(N4CCCC(C)C4)[nH]c(=O)c3C2)nc2ccccc12**	ASINEX	Cc1cc(C(=O)N2CCc3nc(N4CCCC(C)C4)[nH]c(=O)c3C2)nc2ccccc12	0.98496443	0.9949819096840818	0.9378373791146308	0.9725945730436322
**COc1cccc(CN2CCc3c(nc(-c4ccccn4)[nH]c3=O)C2)c1**	ASINEX	COc1cccc(CN2CCc3c(nc(-c4ccccn4)[nH]c3=O)C2)c1	0.99880004	0.9993548893185876	0.9193425865515464	0.9724991717205052
**COc1ccc(C(=O)C2CCN(Cc3nnnn3Cc3ccccc3)CC2)cc1**	ASINEX	COc1ccc(C(=O)C2CCN(Cc3nnnn3Cc3ccccc3)CC2)cc1	0.9515811	0.9793743144887204	0.9863240621522816	0.97242649904401
**COc1ccc(C(=O)C2CCN(Cc3nnnn3Cc3ccc(F)cc3)CC2)cc1**	ASINEX	COc1ccc(C(=O)C2CCN(Cc3nnnn3Cc3ccc(F)cc3)CC2)cc1	0.95822036	0.9764476768580194	0.9819246879901709	0.972197575837153
**O=C(N1CCCC1)N1CCc2nc(N3CCOCC3)[nH]c(=O)c2C1**	ASINEX	O=C(N1CCCC1)N1CCc2nc(N3CCOCC3)[nH]c(=O)c2C1	0.99950695	0.9494144936571256	0.9675298753514118	0.972150439795652
**O=C(Nc1cccc(Cl)c1)N(Cc1nc2ccccc2c(=O)[nH]1)C1CCCC1**	ASINEX	O=C(Nc1cccc(Cl)c1)N(Cc1nc2ccccc2c(=O)[nH]1)C1CCCC1	0.9636376	0.9814038845560012	0.9713943523292604	0.9721452757645288
**O=C(Cc1ccc2c(c1)OCO2)N1CCc2nc(-c3ccccc3)[nH]c(=O)c2C1**	ASINEX	O=C(Cc1ccc2c(c1)OCO2)N1CCc2nc(-c3ccccc3)[nH]c(=O)c2C1	0.99794644	0.9560857876910162	0.962249224993506	0.972093817952692
**COc1cc2c(cc1CN1CCc3c(nc(-c4ccncc4)[nH]c3=O)C1)CCCC2**	ASINEX	COc1cc2c(cc1CN1CCc3c(nc(-c4ccncc4)[nH]c3=O)C1)CCCC2	0.9952538	0.9996320569962518	0.9210682840157486	0.9719847141192752
**CCc1cccc2c(CN3CCc4c(nc(-c5ccncc5)[nH]c4=O)C3)c[nH]c12**	ASINEX	CCc1cccc2c(CN3CCc4c(nc(-c5ccncc5)[nH]c4=O)C3)c[nH]c12	0.9941239	0.9998934382745986	0.921822162739813	0.9719464923697432
**CC1CCC(N2CCc3nc(-c4ccccc4)[nH]c(=O)c3C2)CC1**	ASINEX	CC1CCC(N2CCc3nc(-c4ccccc4)[nH]c(=O)c3C2)CC1	0.9988096	0.9868155024654398	0.9301830792986224	0.971936052599536
**CN(C)C(=O)CN1CCC(c2nccnc2Oc2ccc(C(N)=O)cc2)CC1**	ASINEX	CN(C)C(=O)CN1CCC(c2nccnc2Oc2ccc(C(N)=O)cc2)CC1	0.9661569	0.9996955867824509	0.9495271900497456	0.971793225587081
**COc1ccc(CN2CCC3(CC2)NC(=O)c2ccccc2N3)cc1OC**	ASINEX	COc1ccc(CN2CCC3(CC2)NC(=O)c2ccccc2N3)cc1OC	0.99385095	0.999822264654418	0.9215831298554612	0.9717521136454236
**O=c1[nH]c(-c2ccccn2)nc2c1CCN(Cc1cccc(F)c1)C2**	ASINEX	O=c1[nH]c(-c2ccccn2)nc2c1CCN(Cc1cccc(F)c1)C2	0.9988965	0.9990914425196012	0.9170714239417748	0.9716864486893348
**COc1ccc2[nH]c(=O)c(CN(Cc3ccc(F)cc3)C(=O)c3ccc(F)cc3)cc2c1**	ASINEX	COc1ccc2[nH]c(=O)c(CN(Cc3ccc(F)cc3)C(=O)c3ccc(F)cc3)cc2c1	0.9895477	0.9518196458300232	0.9736665281296116	0.971677967817274
**COc1ccc(CCn2c(SCc3nc4ccccc4c(=O)[nH]3)nc3ccccc3c2=O)cc1OC**	ASINEX	COc1ccc(CCn2c(SCc3nc4ccccc4c(=O)[nH]3)nc3ccccc3c2=O)cc1OC	0.9594315	0.9888404830368204	0.9667498534723958	0.9716739551847738
**O=C(c1nc2ccccc2s1)C1CCN(C(=O)Cc2n[nH]c(=O)c3ccccc23)CC1**	ASINEX	O=C(c1nc2ccccc2s1)C1CCN(C(=O)Cc2n[nH]c(=O)c3ccccc23)CC1	0.9556844	0.9902231163663848	0.9690525268083722	0.9716533555404708
**CC1CN(c2nc3c(c(=O)[nH]2)CCN(Cc2ccc(C(C)C)cc2)C3)CC(C)O1**	ASINEX	CC1CN(c2nc3c(c(=O)[nH]2)CCN(Cc2ccc(C(C)C)cc2)C3)CC(C)O1	0.9928852	0.9969902769465354	0.925067066771457	0.971647505361696
**CC(=O)c1ccc(S(=O)(=O)N2CCc3nc(N4CCOCC4)[nH]c(=O)c3C2)cc1**	ASINEX	CC(=O)c1ccc(S(=O)(=O)N2CCc3nc(N4CCOCC4)[nH]c(=O)c3C2)cc1	0.9490312	0.9824016761674152	0.9834279603785958	0.9716202702429676
**O=C1NC2(CCN(C(=O)Cc3n[nH]c(=O)c4ccccc34)CC2)Oc2ccccc21**	ASINEX	O=C1NC2(CCN(C(=O)Cc3n[nH]c(=O)c4ccccc34)CC2)Oc2ccccc21	0.98718137	0.9944718980228234	0.933201945785294	0.9716184030993592
**Cn1c(CCNC(=O)c2ccc(Cl)cc2)nnc1SCc1nc2ccccc2c(=O)[nH]1**	ASINEX	Cn1c(CCNC(=O)c2ccc(Cl)cc2)nnc1SCc1nc2ccccc2c(=O)[nH]1	0.93012196	0.9948334911199288	0.989832242338952	0.9715958972382164
**CC1CCN(c2nc3c(c(=O)[nH]2)CCN(CCC(C)c2ccccc2)C3)CC1**	ASINEX	CC1CCN(c2nc3c(c(=O)[nH]2)CCN(CCC(C)c2ccccc2)C3)CC1	0.99651825	0.9981804849518878	0.9199279957934242	0.9715422450084676
**CCOc1ccc2[nH]c(=O)c(CN(Cc3cccs3)C(=O)c3ccccc3Cl)cc2c1**	ASINEX	CCOc1ccc2[nH]c(=O)c(CN(Cc3cccs3)C(=O)c3ccccc3Cl)cc2c1	0.9396614	0.9970808742788368	0.977799124042068	0.97151379398325
**Cc1cc(C)c2cc(CN(Cc3ccccc3)C(=O)c3ccc(F)cc3)c(=O)[nH]c2c1**	ASINEX	Cc1cc(C)c2cc(CN(Cc3ccccc3)C(=O)c3ccc(F)cc3)c(=O)[nH]c2c1	0.9930687	0.9474844273516132	0.973646811811301	0.9713999780770912
**CCOc1cc(CN2CCc3c(nc(N4CC(C)OC(C)C4)[nH]c3=O)C2)ccc1OC**	ASINEX	CCOc1cc(CN2CCc3c(nc(N4CC(C)OC(C)C4)[nH]c3=O)C2)ccc1OC	0.99454474	0.9983600539334678	0.9212486059267936	0.9713844681172796
**O=C(Nc1ccccc1F)N(Cc1ccc(F)cc1)Cc1nc2ccccc2c(=O)[nH]1**	ASINEX	O=C(Nc1ccccc1F)N(Cc1ccc(F)cc1)Cc1nc2ccccc2c(=O)[nH]1	0.99602544	0.9929213548940478	0.9251329965951745	0.9713599315221032
**COc1ccc(CCNC(=O)C2CCCCC2c2nc3ccccc3c(=O)[nH]2)cc1**	ASINEX	COc1ccc(CCNC(=O)C2CCCCC2c2nc3ccccc3c(=O)[nH]2)cc1	0.9533277	0.9873729101650834	0.973194753454638	0.9712984596722009
**Cc1ccccc1NC(=O)N(Cc1ccc(F)cc1)Cc1nc2ccccc2c(=O)[nH]1**	ASINEX	Cc1ccccc1NC(=O)N(Cc1ccc(F)cc1)Cc1nc2ccccc2c(=O)[nH]1	0.9864184	0.9903800419669878	0.9370620825404798	0.971286850181434

**COc1ccc(NC(=O)CSc2nnc(-c3ccccn3)n2-c2ccccc2C)cc1**	ASINEX	COc1ccc(NC(=O)CSc2nnc(-c3ccccn3)n2-c2ccccc2C)cc1	0.9310959	0.988033617612575	0.9946018343773336	0.9712437833804356
**O=C(CN1CCc2nc(N3CCCC3)[nH]c(=O)c2C1)NC(c1ccccc1)c1ccccc1**	ASINEX	O=C(CN1CCc2nc(N3CCCC3)[nH]c(=O)c2C1)NC(c1ccccc1)c1ccccc1	0.9981041	0.947011257543464	0.9684379088850334	0.9711844206291604
**O=C(O)c1ccc(-c2nc3sc4c(c3c(=O)[nH]2)CCCC4)cc1**	ASINEX	O=C(O)c1ccc(-c2nc3sc4c(c3c(=O)[nH]2)CCCC4)cc1	0.9357351	0.9945538255292856	0.9824449300853034	0.97091128736093
**O=C1NC2(CCNCC2)Nc2ccccc21**	ASINEX	O=C1NC2(CCNCC2)Nc2ccccc21	0.9975126	0.9976290312657534	0.9174429015125788	0.9708615039141884
**Cc1ccc(CN2CCc3c(nc(N4CCCCC4)[nH]c3=O)C2)o1**	ASINEX	Cc1ccc(CN2CCc3c(nc(N4CCCCC4)[nH]c3=O)C2)o1	0.99306244	0.9966700628477164	0.9226455833100592	0.9707926942461444
**CC1CCN(c2nc3c(c(=O)[nH]2)CCN(Cc2cccc(Cl)c2)C3)CC1**	ASINEX	CC1CCN(c2nc3c(c(=O)[nH]2)CCN(Cc2cccc(Cl)c2)C3)CC1	0.99616086	0.9958646856311388	0.9203193582358332	0.9707816362323296
**O=C(c1ccccc1F)N1CCC(c2cc3cccnc3c(O)n2)CC1**	ASINEX	O=C(c1ccccc1F)N1CCC(c2cc3cccnc3c(O)n2)CC1	0.92593956	0.9968208021014544	0.9895779515414408	0.970779437859806
**CCOc1ccc(CN2CCC3(CC2)NC(=O)c2ccccc2N3)cc1**	ASINEX	CCOc1ccc(CN2CCC3(CC2)NC(=O)c2ccccc2N3)cc1	0.95060444	0.999961556585292	0.961597566661103	0.970721187342711
**COc1ccccc1CNCc1cc2c(C)cc(C)cc2[nH]c1=O**	ASINEX	COc1ccccc1CNCc1cc2c(C)cc(C)cc2[nH]c1=O	0.96771955	0.9658255521915164	0.9783944255472038	0.9706465108799476
**NC(=O)c1cc(Cl)c(C(=O)O)[nH]c1=O**	ASINEX	NC(=O)c1cc(Cl)c(C(=O)O)[nH]c1=O	0.9668374	0.9767990306259244	0.968259220495116	0.9706318857598292
**CC1CN(c2nc3c(c(=O)[nH]2)CCN(Cc2ccccc2O)C3)CC(C)O1**	ASINEX	CC1CN(c2nc3c(c(=O)[nH]2)CCN(Cc2ccccc2O)C3)CC(C)O1	0.9975368	0.9997465996386948	0.914559536699581	0.9706143049294296
**Cc1cccc(CN2CCc3c(nc(N4CCC(C)CC4)[nH]c3=O)C2)c1**	ASINEX	Cc1cccc(CN2CCc3c(nc(N4CCC(C)CC4)[nH]c3=O)C2)c1	0.98655975	0.9974790015457892	0.9273501695562916	0.9704629732505592
**COCCn1c(SCc2nc3ccccc3c(=O)[nH]2)nc2ccccc2c1=O**	ASINEX	COCCn1c(SCc2nc3ccccc3c(=O)[nH]2)nc2ccccc2c1=O	0.9512929	0.9806232893112758	0.979367983434188	0.9704280483914528
**CCOc1ccccc1-n1c(SCC(=O)Nc2ccccc2Cl)nnc1-c1ccncc1**	ASINEX	CCOc1ccccc1-n1c(SCC(=O)Nc2ccccc2Cl)nnc1-c1ccncc1	0.94514257	0.9851742871031572	0.9807156044806116	0.970344152913838
**COc1ccc2c(c1)C1(NC(=O)c3ccccc3N1)C(=O)N2**	ASINEX	COc1ccc2c(c1)C1(NC(=O)c3ccccc3N1)C(=O)N2	0.9920872	0.99980183100593	0.9187571012395866	0.9702153725427866
**CCOc1cc2c(cc1CN1CCc3c(nc(-c4ccncc4)[nH]c3=O)C1)OCO2**	ASINEX	CCOc1cc2c(cc1CN1CCc3c(nc(-c4ccncc4)[nH]c3=O)C1)OCO2	0.9866153	0.9996484950889774	0.9243526408254084	0.970205478697638
**NC(=O)c1ccc(Oc2nccnc2C2CCN(Cc3ccccn3)CC2)cc1**	ASINEX	NC(=O)c1ccc(Oc2nccnc2C2CCN(Cc3ccccn3)CC2)cc1	0.99200493	0.999886789662686	0.9187010353321092	0.970197585322616
**CCOc1ccc2[nH]c(=O)c(CN(Cc3ccccc3)C(=O)c3ccccc3Cl)cc2c1**	ASINEX	CCOc1ccc2[nH]c(=O)c(CN(Cc3ccccc3)C(=O)c3ccccc3Cl)cc2c1	0.9755922	0.9834813153632356	0.9514425677771264	0.9701720263760212
**O=c1[nH]c(-c2cccnc2)nc2c1CCN(Cc1ccc(O)cc1)C2**	ASINEX	O=c1[nH]c(-c2cccnc2)nc2c1CCN(Cc1ccc(O)cc1)C2	0.9976896	0.9989665378992522	0.9138577735791704	0.9701713054125464
**COc1cccc(CN2CCc3nc(-c4ccncc4)nc(O)c3CC2)c1F**	ASINEX	COc1cccc(CN2CCc3nc(-c4ccncc4)nc(O)c3CC2)c1F	0.9175605	0.9974400508348444	0.9954429202031287	0.9701478296086355
**O=c1[nH]c(-c2ccccn2)nc2c1CCN(Cc1cccc(Cl)c1)C2**	ASINEX	O=c1[nH]c(-c2ccccn2)nc2c1CCN(Cc1cccc(Cl)c1)C2	0.9972059	0.9981388287363748	0.9149044407841976	0.9700830608624788
**CCN(CC)c1ccc(CN2CCc3c(nc(N4CCCCC4)[nH]c3=O)C2)c(O)c1**	ASINEX	CCN(CC)c1ccc(CN2CCc3c(nc(N4CCCCC4)[nH]c3=O)C2)c(O)c1	0.99491876	0.9992222648271714	0.9161027623725152	0.9700812636124098
**COc1cc(CN2CCc3c(nc(N4CC(C)OC(C)C4)[nH]c3=O)C2)ccc1O**	ASINEX	COc1cc(CN2CCc3c(nc(N4CC(C)OC(C)C4)[nH]c3=O)C2)ccc1O	0.99544024	0.9984095984029464	0.9162446288892856	0.970031490655638
**O=C(C1CCC1)N1CCN(Cc2nc3ccccc3c(=O)[nH]2)CC1**	ASINEX	O=C(C1CCC1)N1CCN(Cc2nc3ccccc3c(=O)[nH]2)CC1	0.9446218	0.9723301409705494	0.9931254996169478	0.9700258139879466

Table S5. Machine-learning predictions for the 670 compounds retained after the AI-based screening workflow. Predicted probabilities obtained with the three classifiers (XGBoost, Logistic Regression, and SVM-RBF) are reported for each of the 670 compounds retained after the AI-based screening and filtering procedure. The Consensus_score represents the final consensus prediction calculated from the individual model probabilities. The original and standardized SMILES strings and the source library of each compound are also provided.

Compound	***SMILES_raw***	***Source***	***XGBoost_prob***	***LogReg_prob***	***SVM_RBF_prob***	***Consensus_score***
1	COc1ccc(cc1)-c1ccc(C(O)=O)c(=O)[nH]1	Molport	0.97518	0.99443	0.97582	0.98181
2	OC(=O)c1ccc([nH]c1=O)-c1ccccc1	Molport	0.97060	0.98467	0.99999	0.98509
3	O=c1cc(oc2ccccc12)-c1ccccc1	Molport	0.99929	0.99917	0.99999	0.99948
4	OC(=O)c1ccc([nH]c1=O)-c1ccc(Cl)cc1	Molport	0.98735	0.98411	0.96627	0.97924
5	COc1cccc(c1)-c1ccc(C(O)=O)c(=O)[nH]1	Molport	0.98442	0.99990	0.97920	0.98784

Table S6. Machine learning prediction probabilities and consensus scores of the five final TNKS1 candidate compounds selected for experimental validation. Prediction probabilities assigned by the three independently trained machine learning classifiers (XGBoost, Logistic Regression, and SVM-RBF) together with the corresponding arithmetic-mean consensus score for the five TNKS1 candidate compounds selected after the structure-based screening workflow. All compounds satisfied the consensus selection criterion requiring an active-class probability > 0.90 from each individual classifier prior to docking.

SMILES	Label
**O=C(c1ccc(F)cc1)C1CCN(CC(=O)N(Cc2nc3c(c(=O)[nH]2)COCC3)CC2CC2)CC1**	1
**CCc1cc2c(cc1Cl)c(cc(=O)o2)CSc3n[nH]c(=O)n3C[C@@H]4CC**	0

**CO4**	
**CC(C)[C@H]1CC(=Nc2c1c(=O)[nH]n2C3CCCCC3)O**	0
**CCn1ccnc(c1=O)N2C[C@@H](N[C@H](C2)C)C**	0
**CC[C@H](C(=O)Nc1ccccc1OCC)Sc2nc3ccsc3c(=O)n2Cc4cccc[nH+]4**	0
**CCC[C@H](C(=O)N[C@H](Cc1ccc(cc1)Cl)C(=O)[O-])NC(=O)CCc2c(c3cc4c(c(oc4cc3oc2=O)C)C)C**	0
**c1cc(c(c2c1nsn2)/N=C(/c3ccc(nn3)Cl)\[O-])Cl**	0
**C[C@H]1CC[C@@H]([C@@H](C1)[NH+]2CCCCc3c2cccc3)C(=O)[O-]**	0
**Cc1c(c(n[nH]1)C)C(=O)NC23CC4CC(C2)CC(C4)C3**	0
**C[C@@H](C(=O)NC1=c2cc(ccc2=[NH+][C@@H]1C(=O)OC)Cl)N3CCN(CC3)CCO**	0
**Cc1cccc(c1)OCCCCn2c3ccccc3[nH+]c2CCCNC(=O)c4cccc(c4)Br**	0
**C[C@@H]1CCCN(C1)S(=O)(=O)c2cc(ccc2Cl)C(=O)Nc3ccc4c(c3)OCCCO4**	0
**c1ccc2c(c1)CCN(C2)S(=O)(=O)c3ccc(c(c3)C(=O)Nc4ccc5c(c4)OCO5)Cl**	0
**c1cc2c(cc1NC(=O)Cc3ccsc3)OC4(O2)CCCC4**	0
**C[C@@H]1CCC[C@@H]1NC(=O)c2cc(c(c(c2)Cl)Cl)[N+](=O)[O-]**	0
**COc1cc(cc(c1OC)OC)C[C@@]2(C(=O)NC(=O)N(C2=O)c3ccccc3F)CCc4cccc[nH+]4**	0
**CC(C)(O)c1ccc(-c2nc3c(F)ccc(F)c3c(=O)[nH]2)cc1**	1
**C[C@@H](C1CC1)N(C)C(=O)N[C@@H]2CCCCC[C@@H]2C(=O)[O-]**	0
**Cc1ccc(cc1)n2c(c(c(n2)c3ccccc3)C#N)N**	0
**Cc1cc(F)cc2c(=O)[nH]c(-c3ccc(C4CCNCC4)cc3)nc12.Cl**	1
**c1ccc(cc1)N2CCN(CC2)[C@@H]3[C@H]([C@@H]([C@H]4CO[C@@H]3O4)[NH2+]Cc5ccccn5)O**	0
**COc1ccc(cc1)[C@H]2c3ccc4ccccc4c3Oc5c2c([n+](cn5)CCc6ccc(c(c6)OC)OC)N**	0
**CC1(C[C@](CCO1)([C@H]2CCCC[NH2+]2)O)C**	0
**CC(C)(CNC(=O)CSc1nnc(n1C)[C@H]2COc3ccccc3O2)[NH+]4CCOCC4**	0
**Cc1cc(n(n1)C)NC(=O)c2cc3c(s2)n(c([nH+]3)N(C)C)c4ccc(cc4)Br**	0
**C[C@@H](C(=O)NC)Oc1cc2c(cc1C=O)OCO2**	0
**COc1ccc2c(c1)CC1(CCN([C@H]3CCN(Cc4nc5c(c(=O)[nH]4)COCC5)C3=O)CC1)C2=O**	1
**O=C(N[C@H]1C[C@H](c2nnc(-c3ccc(OC(F)F)cn3)n2-c2ccccc2F)C1)c1ccccn1**	1
**CN1CCCc2c1nc(N1CCC3(CC1)C(=O)N(C)c1cc(N4CCOCC4)cc(F)c13)[nH]c2=O**	1
**Cc1ccc(cc1)S(=O)(=O)CNC(=O)[C@@H](c2cc(ccc2Cl)[N+](=O)[O-])N(C(=O)COC)C(C)(C)C**	0
**C[C@@H]1Cc2cc(ccc2N1C(=O)C)S(=O)(=O)Nc3cccc(c3)c4ccc(nn4)N5CCOCC5**	0
**c1cc2c(cc1N)oc(n2)N3CCc4c(ccs4)C3**	0
**Cc1ccc(c(c1)[C@@H](c2cc[nH+]cc2)NC(=O)Cc3c([nH]c4ncnn4c3=O)C)C**	0
**Cn1c(c(c(=O)n(c1=O)C)C(=O)COC(=O)c2c(c(c(cn2)Cl)Cl)Cl)N**	0

Cc1c(c(=O)n(n1C)c2ccccc2)N\3C(=O)CS/C3=N/N=C\4/c5ccccc5NC4=O	0
Cc1ccc(cc1)c2csc3c2c(ncn3)OCC(=O)NC4CC4	0
CC[C@H](CO)Nc1nc(c(s1)C)C	0
C[C@@H](c1ccccc1)N2C(=O)/C(=C\c3cn(nc3c4ccc(cc4)Cl)c5ccccc5)/C(=NC2=O)[O-]	0
Cc1cccc(-c2nnc([C@H]3C[C@H](NC(=O)c4ccnc5cc(F)cnc45)C3)n2-c2ccccc2F)n1	1
COc1ccccc1-c1ccc2c(C)cc(=O)[nH]c2c1	1
CC(C)NC(=O)Nc1ccccc1C(=O)OCc2cc(=O)n3cc(ccc3n2)Br	0
C[C@H](c1cc(ccc1O)F)Nc2ccc3c(c2)nccn3	0
CC(=O)Nc1cc(ccc1F)NC(=O)c2cccc(c2)S(=O)(=O)N[C@H]3CCS(=O)(=O)C3	0
c1cc(ccc1S(=O)(=O)N2CCC[C@H](C2)C(=O)Nc3nc4ccc(cc4s3)F)Cl	0
[H]/N=C\1/C(=C/c2cccn2CCOc3ccccc3)/C(=O)N=C4N1N=C(S4)N5CCC(CC5)C	0
C1C[C@@H]([NH2+]C1)CC(=O)N2C[C@H]3CC[C@@H](C2)O3	0
c1cc(ccc1S(=O)(=O)N=C2NCN(CN2)C3CC3)Cl	0
Cc1ccccc1Cc2c(c(=O)cc(n2Cc3cc[nH+]cc3)C)C(=O)NCCn4ccnc4	0
c1cc(nc(c1Cl)C[NH+]2CCN(CC2)c3nccs3)N	0
Cc1cc(=O)n2c([nH]1)cc(n2)[C@@H]3CCCN(C3)C(=O)CCN4Cc5ccccc5C4=O	0
Cc1[nH+]c(cn1Cc2csc(n2)C)c3nc(c4n3cccc4)C(=O)NCCc5ccncc5	0
CN1CCCc2c1nc(N1CCC3(CC1)CN(CCO)c1cc(F)cc(F)c13)[nH]c2=O	1
C[C@H](c1cccnc1)Nc2[nH]c3cc(ccc3n2)N	0
C[C@H]1CN(C[C@@H](O1)C)S(=O)(=O)CCNC(=O)Cc2ccc3c(c2)CCCC3	0
c1cc(c(cc1NC(=O)N2CCCCCC2)F)O	0
CC(C)Cc1nnc(s1)NC(=O)CCC(=O)N2CCC3(CC2)OCCO3	0
COCCNC(=O)[C@@H](c1cc[nH+]cc1)N(Cc2ccccc2Cl)C(=O)Cn3c4ccccc4nn3	0
CC(C)c1ccccc1n2cc(c(n2)C(=O)[O-])O	0
CCOc1ccc(-c2nnc([C@H]3C[C@H](NC(=O)c4ccnc5cc(F)cnc45)C3)n2-c2cccnc2)nc1	1
COc1ccc(cc1NC(=O)CSc2ncc(c(n2)N)S(=O)(=O)c3cccs3)Cl	0
c1ccc(cc1)CN2C(=O)[C@@](C(=O)NC2=O)(Cc3cccc4c3cccc4)CN5C[C@H]6C[C@@H](C5)c7cccc(=O)n7C6	0
Cc1nccn1Cc2c(snn2)NC	0
CC(=O)N1CCN(CC1)Cc2[nH+]c3cc(ccc3n2C)NC(=O)COc4ccc(cc4)Cl	0
C[C@@H]1CC=C2[C@@H](C1)[C@H](C(C(=C2C#N)N)(C#N)C#N)c3ccccc3OC(F)F	0
C=CCN1c2c(ccc(c2Cl)Cl)/C(=N/O)/C1=O	0
c1c[nH]nc1NC(=O)c2cc(cc(c2)Br)F	0
Cc1cc(no1)NC(=O)CSc2nnc(s2)N3CCN(CC3)C(=O)Cc4ccc(cc4)OC	0
CN1c2ccc(cc2-c3c(cnc(n3)SCC(=O)Nc4nccs4)S1(=O)=O)Cl	0

**c1cc(ccc1S(=O)(=O)N2CCN(CC2)C(=O)CSc3nnc(n3N)C4CC4)Cl**	0
**C[C@H]1CCC[C@H](C1)c2nc(on2)C=O**	0
**CC(C)c1ccc(cc1)[C@@]2(CC(OC2(C)C)(C)C)O**	0
**CC1(C)OC(=O)N(c2ccc(-c3cnc(N)c(-c4ncccn4)c3)cc2)[C@H]1c1ccccc1**	1
**Cc1ccc(c(c1)C(=O)[C@H]2[C@@H](N(C(=O)C2=O)c3nc(c(s3)C(=O)OC)C)c4cccc(c4)[N+](=O)[O-])C**	0
**CCc1c(C)nc(N2CCN(c3ccccc3F)CC2)nc1O**	1
**CC(C)(C#CC(=O)[O-])C#CC(C)(C)C#CC(C)(C)O**	0
**CC(C)(C(C(F)F)(F)F)O**	0
**[H]/N=C\1/NC[C@H](N1c2ccc(c(c2)Br)Cl)C3CCOCC3**	0
**O=C(c1ccc(Cl)cc1)C1CCN(C2CCN(Cc3nc4c(c(=O)[nH]3)COCC4)C2=O)CC1**	1
**c1cc(cnc1)[C@@H](c2nc(no2)c3ccc4c(c3)nc[nH]4)[NH+]5CCCC5**	0
**Cc1ccc(s1)S(=O)(=O)N2CCC[C@@H]2C(=O)NCc3ccc(cc3)N(C)C**	0
**COc1ccc2c(c1)C(CC(=O)N1CCN(C(=O)c3ccccc3N3CCC(=O)NC3=O)CC1)CC2=O**	1
**COc1ccc2c(=O)[nH]c3sccc3c2c1**	1
**CC[C@H]1CCC[C@@]2(CC1)C[NH2+]C[C@@H]2C(=O)[O-]**	0
**O=c1[nH]nc2c3c(cccc13)N=C(CN1Cc3ccccc3C1)N2**	1
**Cc1cc(ccc1c2nnnn2c3cc(ccc3F)Br)N**	0
**C(=O)N[C@@H]1C(=C(C(=O)O1)Cl)Cl**	0
**CCn1c(c(c(n1)C)N)Oc2cc(ccc2C(C)C)C**	0
**CC1CCN(CC1)c2ccc(c[nH+]2)CNC(=O)NCC3(CCCCC3)N4CCOCC4**	0
**CC(C)(C)[C@H]1CCc2c(sc(c2C#N)NC(=O)Cn3nc(nn3)c4cccs4)C1**	0
**CCc1ccsc1CNc2cc(ccc2C)[N+](=O)[O-]**	0
**c1cc(c2c(c1S(=O)(=O)NCCNC(=O)C3=C[C@@H]4C(=CC=N4)C=C3)non2)Cl**	0
**c1cc(c2nc(nn2c1)N[C@@H]3C[C@H]4CC[C@@H]3C4)Br**	0
**c1ccc2c(c1)[C@H](CCO2)NC(=O)CN3CCN(CC3)c4c5ccsc5ncn4**	0
**C[C@@H]1CCC[C@H]([C@@H]1C)NC(=O)[C@@H]2CCCC[C@@H]2C(=O)[O-]**	0
**O=c1[nH]c(N2CCC3(CC2)CN(CCO)c2cc(Cl)cc(Cl)c23)nc2c1CCCC2**	1
**CCN1CCN(CC1)CCCN2CC3(CCN(CC3)Cc4[nH]ccn4)OC2=O**	0
**CC(C)C(=O)N1CC(c2c1ccc(c2)S(=O)(=O)[N-]c3ccc4c(c3)OCO4)(C)C**	0
**Cc1c(nn(c(=O)c1c2nc(c(s2)C(=O)NC[C@@H](C)N3CCN(CC3)c4ccccc4)C)C)C**	0
**c1cc(c(cc1Cl)C#N)Sc2nc(cc(n2)N)N**	0
**C[C@@H]1CO[C@@](O1)(C)c2ccccc2**	0
**c1cc(c(cc1/C=C(/C#N)\C(=O)Nc2nccs2)Cl)[O-]**	0
**O=c1cc(-c2ccc(O)c(O)c2)oc2ccccc12**	1
**O=c1[nH]c(-c2ccc([N+](=O)[O-])cc2)nc2ccccc12**	1
**COc1cc(cc(c1OC)OC)c2c3c(n[nH]2)OC(=C([C@@H]3c4ccc(cc4)Br)C#N)N**	0
**Cc1cc(nc(n1)Cl)NC23CC4C[C@@](C2)(C[C@](C4)(C3)C)C**	0

CCOc1ccc(C(=O)C2CCN(CC(=O)NCc3nc4c(c(=O)[nH]3)COCC4)CC2)cc1	1
O=C(CN1CCC(n2cc(-c3ccccc3)cn2)CC1)NCc1nc2c(c(=O)[nH]1)COCC2	1
Cc1ccc(cc1)CNc2c3c(nc4n2nc(n4)C)CCCC3	0
COCc1c(cnn1c2nccc(n2)c3cccs3)C(=O)N[C@@H](c4ccc[nH+]c4)C(F)(F)F	0
CCCCNC(=O)c1cn(cc(c1=O)C(=O)N2CC[C@H](C2)[NH+](CC)CC)C3CCCCC3	0
Cc1ccc(cc1)S(=O)(=O)N2CCCC[C@@H]2C(=O)Nc3cc4ccccc4[nH+]c3	0
Cn1c(cc(=O)c(c1CN2CCN(CC2)CCOc3ccccc3Cl)O)CN4C[C@H]5C[C@@H](C4)c6cccc(=O)n6C5	0
C[C@H]1CCN(C1)C(=O)NC(=O)C[C@@H](C)CC(=O)O	0
c1cc(ccc1c2cc(c(nn2)[O-])O)Cl	0
CC(=O)N1CCc2c1cc(c(c2)Br)S(=O)(=O)N3CCC(CC3)C(=O)NCc4ccco4	0
CN1CCc2c1nc(N1CCC3(CC1)C(=O)N(CCO)c1cc(F)cc(F)c13)[nH]c2=O	1
[H]/N=C\1/[C@@]2([C@@H](O[C@](O1)([C@@H](C2(C#N)C#N)c3ccccc3)C)c4ccccc4)C#N	0
C/C(=C\c1cc2c(c(c1)Cl)OCCO2)/C(=O)O	0
c1ccc(cc1)[C@@H](C(=O)Nc2ccccc2F)NS(=O)(=O)c3ccc4c(c3)CCC(=O)N4	0
Cn1c2ccc(cc2n(c1=O)C)S(=O)(=O)CC(=O)Nc3cc(c(cc3OC)OC)Cl	0
COc1ccc(cc1C(=O)C2=C(C(=O)N([C@@H]2c3cccc(c3)F)CCCn4cc[nH+]c4)[O-])F	0
Cc1ccc(-c2cc(=O)c3ccccc3o2)cc1	1
CCCN1CCN(CC1)CCCNC(=O)c2cc3n(n2)C[C@@](N(C3=O)C)(C)C(=O)NCc4ccccc4	0
C[C@H]1CCCN([C@@H]1C)C(=O)c2c(cnc(n2)C(C)C)NC	0
Cc1cccc2c1OCC/C(=C\c3cc4c(c(c3)Cl)OCO4)/C2=O	0
c1cc(c(cc1S(=O)(=O)Nc2ccc(nc2)N)F)F	0
Cc1cc(=O)oc2c1ccc(c2)OCC(=O)N3Cc4c(c5ccccc5[nH]4)C[C@@H]3C(=O)OC	0
CC1(CC[NH+](CC1)Cc2cc(ccc2[O-])Cl)C(=O)[O-]	0
Cc1c(c(n(n1)c2ccccc2)Cl)/C=N/NC(=O)CNC(=O)c3ccccc3Cl	0
C[C@H]1[C@@H](O[C@@H]([C@@H]1c2nc([nH]n2)C3CCCCC3)C)C	0
O=C(Nc1cccc2c(=O)[nH]ccc12)[C@H]1CCCNC1	1
[H]\N=C\1/C(=C/c2cc(n(c2C)c3ccccc3OCC)C)/C(=O)N=C4N1C(=NS4)S(=O)(=O)C	0
CC(C)(O)c1ccc(-c2nc3cccc(F)c3c(=O)[nH]2)cc1	1
c1ccc2c(c1)N3C(=O)CC[C@]3(S2)C(=O)Nc4nc5c(s4)CCC5	0
C[C@@H](C(=O)N1CCN(CC1)S(=O)(=O)c2ccccc2)OC(=O)c3ccccc3NCCO	0
Cc1ccc(s1)S(=O)(=O)N2CCC[C@H]2C(=O)NCc3ccccc3Cn4cc[nH+]c4	0
CC(C)NS(=O)(=O)c1ccc(c(c1)C(=O)NNC(=O)c2cc(cn2C)Br)Cl	0
Cc1cccc(c1OCCCn2c3ccccc3[nH+]c2CCCNC(=O)c4ccc(cc4)Cl)C	0

O=C(CN1CCC2(CCc3ccccc3C2=O)CC1)NCc1nc2c(c(=O)[nH]1)COCC2	1
C[C@@H]1C(=c2ccccc2=[NH+]1)C(=O)[C@@H](C)OC(=O)CCNC3=NS(=O)(=O)c4c3cccc4	0
c1ccc(cc1)c2nnc(o2)[C@H]3CC34CCN(CC4)S(=O)(=O)c5c[nH]c6c5cccn6	0
C[C@@H]1CC[C@]([C@@H](C1)C)([C@@H]2CCOC2)O	0
c1c(c(n[nH]1)N)[C@@H]([C@H]2CCOC3(C2)CCCC3)O	0
CCc1ccc2c(c1)cc(c(=O)[nH]2)[C@H](c3nnnn3CCOC)[NH+](Cc4cccnc4)Cc5ccco5	0
c1csc2c1[C@H](NCC2)c3[nH]c4cc(cnc4n3)Br	0
CNc1c(cnc(n1)c2ccccc2)I	0
c1cc(c(cc1[C@H](C(=O)[O-])[NH+]2CCCCCC2)Cl)[O-]	0
Cc1cccc(c1C)NS(=O)(=O)c2cc(ccc2OC)N3CCCCS3(=O)=O	0
CCOC(=O)c1c2c(n(n1)Cc3cccc[nH+]3)CCN(C2)C(=O)Cc4c[nH]c5c4cccc5	0
CCCN1[C@@H](c2c([nH]nc2C1=O)c3cc(ccc3O)Cl)c4cc(c(c(c4)OC)OC)OC	0
Cc1ccc(cc1S(=O)(=O)N2CCCCCC2)NC(=O)c3ccc(cc3)n4cncn4	0
CC(C)(O)c1ccc(-c2nc3ccc(F)c(F)c3c(=O)[nH]2)cc1	1
CC/N=C\1/N(C(=O)[C@H](S1)CC(=O)Nc2ccc(cc2)N3CCOCC3)CC	0
C[C@H]1CCc2c(sc3c2c(nc(n3)CN4CCOCC4)NCc5ccc(nc5)n6ccnc6)C1	0
Cc1cccc2c1nc(s2)c3csc(n3)N	0
COC(=O)[C@]1([C@@H]2[C@@H]([C@H]([NH2+]1)c3ccccc3F)C(=O)N(C2=O)c4ccccc4)CO	0
c1ccc2c(c1)ccc3c2[C@@H]4[C@H](CO3)CO[C@H]([NH2+]4)c5ccc(cc5)Cl	0
C[C@H]1CCC[C@H](N1C(=O)CSc2nc3c(c4cc(ccc4[nH]3)Br)nn2)C	0
N#Cc1ccccc1Cn1c(=O)n(C[C@H]2CC[C@H](C(=O)Nc3ccncc3)CC2)c(=O)c2nc[nH]c21	1
Cc1c2c(c(cnc2n(n1)c3ccccc3)C(=O)Nc4ccc5c(c4)OCCO5)Cl	0
c1ccc(C2=NC3=C(CCSC3)CN2)cc1	1
c1cc(nnc1n2c(c(cn2)I)N)Cl	0
C[C@@H]1[C@H](CC[C@@H]1Cl)C[C@@H]2Cc3cc(ccc3O2)F	0
O=c1[nH]c(-c2ccc(C(F)(F)F)cc2)nc2ccccc12	1
Cc1cscc1[C@@H](c2cc3c(cc2Cl)OCCCO3)O	0
C[C@H](c1[nH+]c2ccccc2n1CCCCOc3ccccc3OC)NC(=O)c4cc(c(c(c4)OC)OC)OC	0
C1COC[C@@H]1Oc2c(nsn2)Cl	0
C[C@H](C(=O)Nc1ccc(cc1)N2CCOCC2)N3CCN(CC3)Cc4ccc5c(c4)OCO5	0
Cc1c(sc2c1c(=O)n(c(=S)[nH]2)[C@H]3CC[C@@H]([C@@H]3C)C)C	0
COc1ccc(C2NC(=O)c3ccccc3N2)cc1	1
CC1(C)OC(=O)N([C@H]2CC[C@H](n3c(=O)[nH]c4cc(Cl)ccc43)CC2)[C@H]1c1ccccc1	1
O=C(CN1CCC(C(=O)c2ccc(OC3CCOCC3)cc2)CC1)NCc1nc2c(c(=O)[nH]1)COCC2	1

**CC(C)(O)c1ccc(-c2cn3c(CO)ccc3c(=O)[nH]2)cc1**	1
**c1cc(c(cc1S(=O)(=O)N2CCCC2)NC(=O)c3c(ccs3)S(=O)(=O)N4CCCCC4)[O-]**	0
**Cc1c(c(n(n1)c2ccc(cc2)C(=O)N[C@@H](C)c3[nH]c4ccccc4n3)C)Cl**	0
**O=C1CCN(c2ccccc2C(=O)NCCc2ccc(Cl)cc2)C(=O)N1**	1
**Cc1cc[nH+]cc1NC(=O)N2C[C@@H](O[C@H](C2)c3ccc(cc3)F)C**	0
**CC(C)C(=O)N1CCN(CC1)c2[nH+]c3c(s2)C[C@@H](CC3)C(=O)NC4CC[NH+](CC4)C**	0
**Cc1c(c(nc(n1)c2cc3c(c(c2)Br)OCCO3)N)Br**	0
**Cn1cc[nH+]c1[C@@H](c2cccc(c2)OC)NS(=O)(=O)c3ccc(cc3)S(=O)(=O)N4CCCC4**	0
**c1cncc(c1NC2C[NH2+]C2)Cl**	0
**Cc1cccc(c1C)N2C(=O)C(C(=O)NC2=O)(CCc3cc[nH+]cc3)CCc4cc[nH+]cc4**	0
**Cc1csc(n1)c2ccc(cc2)NC(=O)NCc3cc4c(c(c3)Br)OCO4**	0
**COc1cc2cc3c(c(sc3nc2cc1OC)C(=O)N4CCCCCC4)N**	0
**Cn1c2ccccc2c(=O)c3c1nc(n(c3=O)CCc4ccc(cc4)OC)c5cc(c(c(c5)OC)OC)OC**	0
**CCOC(=O)C[C@@H]1C(=O)NCCN1C(=O)COc2ccc(cc2C)Br**	0
**COc1cccc(c1OC)[C@H]2c3c(c4ccccc4s3)OC(=C2C#N)N**	0
**C[C@H]1C[C@H]2[C@@H]([C@@H](C(=O)O2)C[NH+]3CCCC3)[C@@H]([C@]4([C@@H]1[C@@H](C[C@@H]4O)OC(=O)C)C)OC(=O)C**	0
**Cc1cc(nc2c1N(CCS2)C(=O)Nc3cccc(c3)C(=O)NCc4cccs4)C**	0
**Cc1cc(n(n1)c2ccc(cn2)CNCc3c(nn(c3C)C)C)C**	0
**CC[C@@](C)(C(=O)NC1CCC(CC1)O)[NH3+]**	0
**Cc1ccc(cc1S(=O)(=O)N2CCOCC2)NC(=O)[C@H](C)OC(=O)Cc3c(noc3C)C**	0
**Cc1nc2c(s1)ccc3c2sc(n3)NC(=O)[C@H]4CCCN(C4)S(=O)(=O)c5ccc(cc5)Cl**	0
**Cc1ccc(cc1)N2C(=O)[C@@H]3[C@@H](N[C@@]4([C@@H]3C2=O)c5cccc(c5NC4=O)C)CC6=c7ccccc7=[NH+]C6**	0
**Cc1ccc(c(c1)C)c2nnc(s2)NC(=O)c3ccc[nH+]c3N(C)C**	0
**CN(C)CC[NH+]1CCC(CC1)NC(=O)/C(=N/c2cccc(c2)C#N)/[O-]**	0
**CC(C)(C)c1ccc(-c2nc3ccccc3c(=O)[nH]2)cc1**	1
**Cc1ccccc1Cn2ccc(n2)NC(=O)CCS(=O)(=O)c3nc(cc(n3)C(F)(F)F)c4ccc5c(c4)OCO5**	0
**COc1ccc(CN(Cc2nc3c(c(=O)[nH]2)COCC3)C(=O)CN2CCC(C(=O)c3ccc(F)cc3)CC2)cc1**	1
**Cc1c2cc(sc2n(n1)c3ccccc3)C(=O)Nc4ccncc4**	0
**Cc1cc2c(c(=O)n1CCc3ccc(c(c3)OC)OC)[C@@H](C(=C(O2)N)C#N)c4ccc(cc4)F**	0
**C[C@H]1Cc2ccccc2N1c3cc(=O)n(c(=O)[nH]3)C**	0
**CC(C)c1ccc(C2NC(=O)c3ccccc3N2)cc1**	1
**C1CCCC(CCC1)C(=O)N2CCC[C@@H]2/C(=N\O)/N**	0
**C[C@H]1CC[C@](C1)([C@@H]2CCOC3(C2)CCCCC3)C(=O)O**	0
**Cc1nnc(s1)N2[C@@H](c3c(=O)c4cc(ccc4oc3C2=O)Cl)c5ccc(c(c5)OC)O**	0
**c1cc(oc1C(=O)[O-])Sc2ncns2**	0

**CC(C)(C)OC(=O)N1CCC2(CC1)CCN(C(=O)c1cc(Cc3n[nH]c(=O)c4ccccc34)ccc1F)CC2**	1
**O=C1N(CCO)c2ccccc2C12CCN(c1nc3c(c(=O)[nH]1)CCCC3)CC2**	1
**C1CCCC(CC1)(/C(=N\O)/N)C(=O)N2CCSCC2**	0
**c1ccc2c(c1)sc1nncn12**	1
**Cc1c(c(n(n1)c2ccccc2)C)CN(C)C(=O)CCNC3=NS(=O)(=O)c4c3cccc4**	0
**CCN(Cc1cccc(c1)[N+](=O)[O-])C(=O)c2ccc(cc2)S(=O)(=O)Nc3cc[nH+]cc3**	0
**Cc1c(c(n(n1)C)Nc2c(cc(c3c2nsn3)Cl)Cl)C#N**	0
**CC(C)c1n[nH]c(=S)n1C2(CCCC2)C**	0
**CNC(=O)N1C=CC=C[C@@H]1Br**	0
**Cc1c2c(n(n1)c3ccccc3)C[C@H](C2)C(=C)C**	0
**Cc1c(c(n(n1)c2ccccc2)Cl)CN3C(=O)[C@@]4(CCCc5c4ccs5)NC3=O**	0
**c1cc(ccc1NC(=O)N2CCN(CC2)S(=O)(=O)c3ccc(c(c3)F)F)F**	0
**N#Cc1ccc2c(c1)[nH]c(=O)n2[C@H]1C[C@H](c2nnc(-c3ccncn3)n2-c2ccccc2Cl)C1**	1
**CC(C)[C@H]1CCC(=O)OCC1**	0
**Cc1c(scn1)CCCn2ccnc2c3cc4n(n3)CCNC4**	0
**Cc1c(c(n(n1)C)C)S(=O)(=O)NCc2ccc(s2)C(=O)c3cccs3**	0
**Cc1c(n(c(c1C#N)N)c2ccc(c(c2F)F)F)C**	0
**CC(C)NC(=O)[C@@]1(CCN(C1)C(=O)Cn2cncn2)Cc3cccc(c3)c4cc[nH+]cc4**	0
**c1cc(ccc1c2csc(n2)c3[nH]ccn3)Br**	0
**C[C@@H]1[C@@H]2C[C@@H]([C@H]3[C@]45CCC[C@]([C@H]4C[C@H]([C@]3([C@@H]2OC(=O)C)C1=O)OC(=O)C)(CO[C@H]5OC(=O)C)C)OC(=O)C**	0
**c1ccc2c(c1)cccc2CN3CCN(CC3)C(=O)CNS(=O)(=O)c4ccc5c(c4)OCCCO5**	0
**Cc1ccc(c(c1)[N+](=O)[O-])NC(=S)NCc2ccc(cc2)Cl**	0
**c1c(cc(c(c1Cl)NNC(=O)c2csnn2)Cl)Cl**	0
**CCn1c(ncn1)CN2CCc3c([nH]cn3)[C@@H]2c4cn[nH]c4c5ccccc5**	0
**COc1cc(ccc1O)/C=N/NC(=O)c2ccn(n2)Cc3ccc(cc3)Br**	0
**Cc1cc(cc[nH+]1)N2CCC(CC2)(C(=O)[O-])n3c[nH+]c4c3cccc4**	0
**CC(=O)c1cc(nc(c1)Br)Cl**	0
**CCc1c(cnn1c2ccc(cc2)C)C(=O)NCCS(=O)(=O)N3C[C@@H](O[C@H](C3)C)C**	0
**Cc1c(scn1)Cc2[nH]c3c(n2)CC(CNC3=O)(C)C**	0
**[H]/N=C\1/C(=C\c2cc(c(c(c2)Cl)OC(=O)c3ccco3)OC)/C(=O)N=C4N1N=C(S4)CC**	0
**Cc1cccc2c1c(=O)n(C[C@H]1CC[C@H](C(=O)O)CC1)c(=O)n2Cc1cccc(C(F)(F)F)c1C#N**	1
**CCc1ccc(cc1)[C@@H]2C(=C(C(=O)N2CCC[NH+](C)C)[O-])C(=O)c3cccc(c3)S(=O)(=O)N(C)C**	0
**CN(C)C(=O)c1ccc(cc1)NC(=O)COC(=O)c2cccnc2SCC(F)(F)F**	0
**Cc1cc(sc1C)C(=O)Nc2ccc(cn2)Br**	0
**Cc1cc2c(cc1S(=O)(=O)N3CCN(CC3)C(=O)c4ccc(cc4)Cl)O[C@H](C(=O)N2)C**	0
**Oc1ccc(O)c2c1sc1nncn12**	1
**CCOc1ccc(cc1S(=O)(=O)Nc2nc3ccc(cc3s2)Cl)NC(=O)C**	0

C[C@H]1C[C@@H](CCO1)Nc2ccc3c(c2)CCO3	0
C[C@]1(CCCO1)Cn2c(nnn2)c3ccccc3N	0
C/C=C\1/CN2CC[C@]34[C@@H]2C[C@@H]1C(=C3Nc5c4cccc5O)C=O	0
Oc1ccc(-c2nc(O)c3cccc(O)c3n2)cc1	1
CCc1ccc(o1)CNc2cc3c(c(n(c3[nH+]c2)CCOC)C(=O)OC)NC(=O)c4ccco4	0
CC(C)(C)c1csc(n1)[C@@H](c2cc3c(c(c2)Cl)OCO3)N	0
C[C@H]1CCCN([C@@H]1C[NH3+])C2CCC3(CC2)OCCO3	0
Cc1cccc(c1)Nc2nnc(s2)SCCCn3c(=O)c4c(ncn4C)n(c3=O)C	0
Cc1cccc(c1)/C=N/NC(=O)c2ccccc2NS(=O)(=O)c3cccs3	0
Cc1cc(n(n1)C)C(=O)Nc2ccc(nc2)Oc3ccc(cc3)Cl	0
COc1cc(ccc1O)[C@@H]2[C@@H](C(=O)C(=O)N2CCCn3cc[nH+]c3)C(=O)c4ccc(cc4)Cl	0
c1ccc2c(c1)CC[C@]23C[NH2+]C[C@H]3C(=O)[O-]	0
Cc1ccc(c(c1)C)S(=O)(=O)Nc2ccc(c(c2)[N+](=O)[O-])F	0
Cn1c2ccc(cc2c(=O)n(c1=O)C)S(=O)(=O)N[C@@H](c3ccccc3)C(=O)N4CCc5ccccc5C4	0
c1ccc(cc1)C(c2ccccc2)N3CCN(CC3)C4=NC(=O)[C@@H](C5(N4)CCCC5)C#N	0
O=C(CCc1c(Cl)cccc1Cl)Nc1nnc2n1CCS2	1
c1ccc2cc(ccc2c1)C(=O)NNC(=O)c3ccc(cc3)C#N	0
Cc1cc(cc2c1[C@@H](C[C@@]3(O2)CC(NC(=S)N3)(C)C)c4ccc(cc4)OC)OC(=O)C	0
COc1ccc(-n2c(OCc3nc(-c4ccc(C)cc4)no3)nnc2-c2ccncc2)cc1	1
CC(C)c1[nH]c2c(n1)cc(cc2Br)C(=O)[O-]	0
CC(C)(C)CN(Cc1nc2c(c(=O)[nH]1)COCC2)C(=O)CN1CCC(C(=O)c2ccc(F)cc2)CC1	1
CC(C)(C)[C@@H]1C(=O)N(C2(CCCC2)C(=O)N1)C	0
CN1CCCc2c1nc(N1CCC3(CC1)C(=O)N(C)c1cc(OCC(=O)O)cc(F)c13)[nH]c2=O	1
Cc1c(n2ccccc2n1)C(=O)C3=C(C(=O)N([C@@H]3c4cccc(c4)O)CC[NH+](C)C)O	0
COc1ccc2c(c1)sc1nncn12	1
C[C@](c1nc(no1)C2Cc3ccccc3C2)(C(F)(F)F)N	0
CC(C)NC(=O)N1CCC(CC1)Oc2ccc(cn2)Cl	0
CC1(Cc2c(cnc(n2)c3ccncc3)[C@@H](C1)NC(=O)[C@@H]4CC(=O)N(C4)C5CCCCC5)C	0
C[C@@H](C1CCCC1)N2CCCN3CCCC[C@@H]3C2	0
NC(=O)c1cccc2c1O/C(=C\c1ccc(C(=O)N3CCn4c(nnc4C(F)(F)F)C3)cc1)C2=O	1
CC(=O)N1CCc2c(c(n3c(n2)cc(n3)[C@H]4CCCCN4C(=O)CCC5CCCC5)O)C1	0
CCc1nnc(s1)NS(=O)(=O)c2ccc(cc2)N3C(=O)C[C@@H](C3=O)SCC	0
C[C@H]1CN(C[C@@H]1C)c2c(cc(cn2)N)C#N	0
COC(=O)C1=C2CCN(C[C@H]2S[C@@H]1S(=O)(=O)NCC=C)C[C@@H]3C=NN=C3c4ccc(cc4)F	0
CN1CCCc2c1nc(N1CCC3(CC1)C(=O)N(C)c1cc(N4CCNCC4)cc(F)c13)[nH]c2=O	1
Cc1c(n2ccccc2n1)C(=O)C3=C(C(=O)N([C@H]3c4ccc(cc4)Cl)c5nnc(s5)C)O	0

c1cc(c(cc1[N+](=O)[O-])C(=O)Nc2ccc(cn2)Br)N3CCCCC3	0
NC(=O)c1cccc2c1O/C(=C\c1ccc(C(=O)NCCc3nc4c(C(N)=O)cccc4[nH]3)cc1)C2=O	1
COc1cccc2c(O)nc(-c3ccccc3)nc12	1
Cc1n[nH]c(C)c1CCC(=O)Nc1nnc2n1CCS2	1
Cc1nc2cc(c(cc2s1)N)N(C)CC(=O)N(C)C	0
COc1ccc(cc1)NC(=O)c2nnc(s2)[C@H]3CCCN(C3)S(=O)(=O)c4ccc(s4)Cl	0
C[C@H](C(=O)N1CCN(CC1)C(=O)c2ccco2)N3C(=O)[C@](NC3=O)(C)c4ccc5c(c4)CCC5	0
COc1ccc(cc1OC)C(=O)C2=C(C(=O)N([C@H]2c3cc4c(c(c3)OC)OCO4)CCCn5cc[nH+]c5)O	0
CCN(C(=O)N1CCC(C(=O)c2ccc3cc(C(=O)O)n(C)c3c2)CC1)c1ccc(OC)cc1	1
CC(=O)N1CCN(CC1)S(=O)(=O)c2cccc(c2)C(=O)Nc3ccnn3Cc4ccccc4	0
Cc1nnc(s1)SCCn2c3c([nH+]c2N4CCC(CC4)C(=O)N)n(c(=O)n(c3=O)C)C	0
C[C@]1(C(=O)N(C(=O)N1)C2Cc3ccccc3C2)C4CCN(CC4)C(=O)C5(CCCCC5)C	0
C[C@@H]1C[C@@H](CN(C1)S(=O)(=O)Nc2ccccc2C(=O)[O-])C	0
CC1(Cc2c(c(nc3c2c4c(s3)c(nnn4)NC[C@@H]5CCCO5)N6CCOCC6)CS1)C	0
Cc1ccc(cc1)C[NH+](Cc2cc3cc(c(cc3[nH]c2=O)OC)OC)Cc4nnnn4C[C@@H]5CCCO5	0
Cc1ccc2c(c1)c(n[nH]2)C(=O)N[C@@H]3CCC[C@H](C3)C	0
C/C=C\[C@H](C(=O)NCS(=O)(=O)c1ccc(cc1)C)N(Cc2ccc(o2)C)C(=O)COC	0
CCCCSc1nc2c(c3c(s2)CO[C@@](C3)(C)CC)c(n1)SCC(=O)Nc4nccs4	0
O=c1[nH]c(-c2ccc(Br)cc2)nc2c1COCC2	1
Cc1ccc(c(c1)[O-])C(=O)N2CCC[C@H](C2)[C@H]3CCC[NH2+]3	0
Cc1c(c(n(n1)C)C)N[C@@H]2CCCC[C@H]2C	0
Cc1cc(c(nc1)Nc2cc3ccccc3nc2)Br	0
COc1ccc(-c2nc(O)c3cccc(OC)c3n2)cc1	1
Cc1c(F)ccc2c1c(=O)n(C[C@H]1CC[C@H](C(=O)O)CC1)c(=O)n2Cc1cccc(C(F)(F)F)c1C#N	1
CCO[C@H]1[C@@H](c2ccccc2C13CCN(CC3)C(=O)C4=CN=NC4)NC(=O)c5cccnc5	0
COCCN1C(=O)C2(CCN(c3nc4c(c(=O)[nH]3)CCCN4C)CC2)c2c(F)cc(F)cc21	1
Cc1ccc(-c2nc3c(c(=O)[nH]2)COCC3)cc1	1
C1C=Cc2c(=C1)n3c(=C4CC(=C3)C=C4)n2	0
C[C@H]1CN2CCCC[C@@H]2CN1C3CCCCCC3	0
Cc1ccc(cc1C)NC(=O)c2c3c(on2)-c4ccsc4CC3	0
CCn1cc(c(n1)C)/C=c\2/c(=O)n3c(=NC(=C([C@H]3c4ccc(cc4)OC)C(=O)OCC)C)s2	0
Cc1cccc2c1c(=O)n(C[C@H]1CC[C@H](C(=O)Nc3ccncc3)CC1)c(=O)n2Cc1ccccc1C#N	1
Cc1c2cc(c(cc2oc(=O)c1CC(=O)Nc3nnc(s3)C(C)(C)C)OC)Cl	0

O=c1[nH]c(-c2ccc(Br)cc2)nc2c(O)cccc12	1
c1ccc2c(c1)CCN[C@H]2c3nc(no3)c4ccoc4	0
Cc1c[nH+]ccc1N2CCN(CC2)C(=O)Nc3cccc(c3)[N-]S(=O)(=O)C	0
CN(C)c1c(cc(cn1)Br)C(=O)NNc2cc(cc(n2)Cl)C#N	0
Cc1cc(n(n1)c2nc(cs2)CCNC(=O)C(=O)c3c([nH]c4c3cccc4)C)C	0
CCOc1ccc(-c2nnc([C@H]3C[C@H](NC(=O)c4ccccn4)C3)n2-c2cccc(Cl)c2)nc1	1
CCc1ccc(cc1)[C@@H]2C(=C(C(=O)N2CCCN3CCOCC3)[O-])C(=O)c4ccc5c(c4)C[C@@H](O5)C	0
Oc1nc(-c2ccc(C(F)(F)F)cc2)nc2c1CSCC2	1
CCOc1cc(ccc1OCC(=O)OC)/C=C/2\C(=O)NC(=S)N(C2=O)c3ccccc3F	0
c1cc(c(c2c1nsn2)N[C@@H]3CCSC3)Cl	0
C[C@H](c1cccc(c1)Cl)N2CCN(CC2)c3ccc(nn3)[O-]	0
Cc1c(c(sn1)Nc2ccc3c(c2)C(=O)N(C3=O)C)C#N	0
c1ccc(cc1)N2CCN(CC2)C(=O)c3ccccc3NC(=O)c4cccc(c4)S(=O)(=O)N5CCCCC5	0
CC1(C)CC2=NNC(=O)c3cccc4c3c2c(n4CCN2CCCCC2)C1	1
C[C@@H]1c2c(sc(n2)NC(=O)c3ccc(cc3)CN4CCN(CC4)C)C[C@]5([C@H]1[C@H]([C@H](CC5)[C@H](C)C(=O)NCCN6CCOCC6)O)C	0
Cc1cnc(c(c1OC)C)C[S@@](=O)c2[nH]c3ccc(cc3n2)OC	0
c1cc(ccc1NS(=O)(=O)c2ccc(cc2)N3C(=O)CCS3(=O)=O)Br	0
COc1ccc(cc1OC)CC(=O)N(CCCn2cc[nH+]c2)c3nc4c(ccc(c4s3)OC)OC	0
Cc1ccc(cc1)C2=NO[C@H](N2)[C@@H]3C=C(N=N3)c4ccc(cc4)Cl	0
C[C@@H](CCC(=O)N1CCc2c(sc(c2C#N)N)C1)c3ccccc3	0
CC(=O)Nc1nnc(s1)SCc2cc(=O)c(co2)OC(=O)[C@H]3CC(=O)N(C3)c4ccccc4	0
COc1ccc(cc1)CC/N=C/2\[C@@H](C(=O)C2=O)N3CCN(CC3)c4cccc[nH+]4	0
CN(CCc1cccc[nH+]1)C(=O)c2cnn3c2N[C@@H](C[C@@H]3C(F)(F)F)c4ccc(c(c4)OC)OC	0
C[C@@H](c1ccccc1)NC(=O)CN2CN(C3(C2=O)CCN(CC3)C(=O)N(C)C)c4ccccc4	0
Cc1ccc(c(c1)S(=O)(=O)N(CCC(=O)N2CCNCC2)CC[NH+]3C[C@@H](C[C@@H](C3)C)C)C	0
Cc1cc(ccc1NC2(CCCCC2)C(=O)[O-])Br	0
O=C(CCc1ccccc1)N(Cc1nc2ccccc2c(=O)[nH]1)Cc1cccs1	1
Cc1nc(cc(n1)[O-])C2CC[NH+](CC2)Cc3ccc4c(c3)non4	0
Cc1cc(c(o1)C)[C@@H](C)Nc2c3nnc(n3ccn2)C	0
N#Cc1ccc(N2CCN(Cc3ccc4c(c3)NC(=O)C3CCCNC43)CC2)nc1	1
Cn1c(nnc1SCC(=O)Nc2ccc3c(c2)OC(O3)(F)F)[C@@H]4COc5ccccc5O4	0
CC1(C)OC(=O)N([C@H]2CC[C@H](c3cnc(N)c(-c4ncccn4)c3)CC2)[C@H]1c1ccccc1	1
CC1=C([C@@H](C2=C(N1)C[C@@H](CC2=O)c3cccs3)c4cc(c(c(c4)OC)OC)OC)C(=O)OC[C@@H]5CCCO5	0
CCn1c(c(c(n1)C)Br)C[NH2+]CC2(CCC(CC2)(C)C)O	0
CC1(CCC[C@@](CC1)(C#N)N2CCNCC2)C	0
C[C@@H]1C[C@@]2(CC(C1)(C)C)C(=O)N(C(=O)N2)CC(=O)	0

N(Cc3ccco3)[C@@H]4CCS(=O)(=O)C4	
COc1ccc(C(=O)C2CCN(C3CCN(Cc4nc5ccccc(=O)c5[nH]4)C3=O)CC2)cc1	1
CC(C)Cc1ccc(cc1)S(=O)(=O)N(CCN2CCCCC2)CC(=O)N3CCNCC3	0
O=C(N[C@H]1C[C@H](c2nnc(-c3ccc(OC(F)F)cn3)n2-c2ccccc2F)C1)c1cccc2nccnc12	1
CN1CC[C@H](C1)Nc2nc(nc(n2)Cl)N3CCCC3	0
Cc1ccc2c(c1C)nc(s2)N(CCCn3ccnc3)C(=O)c4ccc(c(c4)[N+](=O)[O-])C	0
[H]/N=c\1/c(cc2c(n1CCc3ccccc3)nc4c(cccn4c2=O)C)S(=O)(=O)c5ccc(cc5)Br	0
C[C@@H]1C[C@H](CN(C1)C(=O)N[C@@H](C)CC(=O)[O-])C	0
c1cc(ccc1F)S(=O)(=O)N2CCCN(CC2)C/C(=N/N3C(=O)C4(CCCCC4)NC3=O)/[O-]	0
CS(=O)(=O)c1ccc(-c2nnc(SCc3nnc(-c4ccc(Cl)cc4)o3)n2-c2ccccc2Cl)nc1	1
COc1ccc(cc1OC)c2ccc(c(n2)SCC(=O)Nc3ccc4c(c3)OCCO4)C#N	0
O=c1[nH]nc2c3c(cccc13)NC1=C2CCNC1	1
COc1cccc2c(=O)[nH]c(-c3ccc(C)cc3)nc12	1
C[C@@H](c1ccccc1)n2c(n[nH]c2=S)CSc3nnc(n3C[C@H]4CCCO4)c5ccn(n5)C	0
COc1ccc(cc1OC)[C@@H]2c3cc(c(cc3CC(=O)N2NC(=O)c4ccc(cc4)[N+](=O)[O-])OC)OC	0
Cc1cccn2c1nc(c2C)C(=O)C3=C(C(=O)N([C@@H]3c4cccc(c4)Br)CCC[NH+](C)C)O	0
O=C1Nc2cc(F)cc(F)c2C12CCN(c1nc3c(c(=O)[nH]1)CCCC3)CC2	1
CC[NH2+][C@@H](c1ccc(cc1)Cl)c2ccc3c(c2)oc(n3)[O-]	0
Cc1ccc(cc1C)C(=O)NCC(=O)N2CCN(CC2)C/C=C/c3ccccc3	0
c1ccc2c(c1)c3ccnc(c3[nH]2)c4ccco4	0
O=C(Nc1nnc2n1CCCS2)c1ccccc1	1
C[C@@H]1CN([C@@H](CO1)c2ccccc2)Cc3c[nH]nc3c4ccc5c(c4)OCCO5	0
CC(C)CNS(=O)(=O)c1ccc(cc1)S(=O)(=O)N2CCC[NH+](C2)Cc3ccccc3	0
CNC(=O)[C@H]1CN(c2ccccc2O1)CC(=O)N3CCN(CC3)S(=O)(=O)c4ccc5c(c4)CCC5	0
CCn1c(=O)c2c3c(sc2nc1NCCn4ccnc4)CCCC3	0
Cc1cc(cc(c1OCc2cn3ccccc3n2)N)Br	0
c1cc(c(cc1c2nnc(s2)N)F)Br	0
c1cc(c(nc1)N)[C@H](c2cc3c(c(c2)Cl)OCO3)O	0
C[C@@H](C(C)(C)C)N(C)C(=O)N(C)c1ccccc1C(=O)[O-]	0
Cn\1c2ccc(cc2s/c1=N\C(=O)c3ccc(cc3)S(=O)(=O)N(C)C)C(=O)OC	0
CCc1c(c(nc(n1)[C@H]2COc3ccccc3O2)NC)I	0
CCS(=O)(=O)c1ccc(cc1)c2nnc(o2)NC(=O)c3ccc(cc3)S(=O)(=O)N(CC=C)CC=C	0
CCOc1ccc(-c2nnc([C@H]3C[C@H](NC(=O)c4ccccn4)C3)n2-c2ccc(Cl)s2)nc1	1
c1ccc(cc1)c2cc(n(n2)c3ccccc3)C(=O)OCC(=O)N[C@@H]4CCS(=O)(=O)C4	0
Cc1ncc2c(=O)[nH]c(-c3ccc(C(C)(C)O)cc3)cn12	1

**c1csc(c1Cl)c2[nH]nc(n2)C3CC3**	0
**c1cc(c(cc1S(=O)(=O)N[C@@H]2CCN3[C@@H]2CCC3)N)O**	0
**CC1(CCC[C@]1(C2(CCCC2)C#N)O)C**	0
**O=C1Nc2cc(F)ccc2C12CCN(c1nc3c(c(=O)[nH]1)CCCC3)CC2**	1
**CCn1c2ccc(cc2nc1C[NH+]3CCN(CC3)C(=O)C)NC(=O)COc4ccccc4**	0
**c1ccc(cc1)c2nnnn2/C(=C\c3ccco3)/C(=O)OCC(=O)c4ccc(c(c4)Cl)Cl**	0
**Cc1cc2c(c(=O)n1CCc3ccc(c(c3)OC)OC)[C@H](C(=C(O2)N)C#N)c4ccc[nH+]c4**	0
**c1cn(c[nH+]1)CCCNC(=O)c2cc(c(cc2Cl)Cl)S(=O)(=O)N3CCOCC3**	0
**c1ccc2c(c1)c(c3c(n2)sc(n3)Cl)Cl**	0
**COc1ccc(C(=O)C2CCN(CC(=O)NCc3cc4c(c(=O)[nH]3)COCC4)CC2)cc1**	1
**Cc1cccc(c1)C(=O)N[C@H](C(=O)N2CCN(CC2)C(=O)c3cccs3)S(=O)(=O)c4ccccc4**	0
**Cc1c(nc(c(n1)N[C@H]2CCC[C@@H]2C)Cl)C**	0
**Cc1c(c(on1)C)S(=O)(=O)[N-]c2ccc3c(c2)N(CCC3)S(=O)(=O)c4ccc(cc4)F**	0
**Cc1cc(cc-2c1N(S(=O)(=O)c3c2cccc3)C)C(=O)NC4CC4**	0
**C[C@@H]1CN(CCN1S(=O)(=O)N2CCc3ccccc3C2)S(=O)(=O)N4CCc5ccccc5C4**	0
**c1ccc2c(c1)cc(cn2)NC(=O)c3ccc(cc3[O-])Cl**	0
**C[C@H]1C(=O)N[C@@](C(=O)N1C2CCCCCC2)(C)C3CC3**	0
**c1cc2c(cc1S(=O)(=O)N3CCCCC3)nnn2CCCC(=O)NCCc4c[nH]c[nH+]4**	0
**Cc1cc(cc(c1NS(=O)(=O)c2ccc(=O)[nH]c2)C)Br**	0
**CCc1c2cc(ccc2nc-3c1Cn4c3cc5c(c4=O)COC(=O)[C@@]5(CC)O)OC(=O)N6CCC(CC6)NC(=O)OCc7ccccc7**	0
**c1ccc(cc1)c2nc(cs2)C(=O)Nc3cccc(c3)[N+](=O)[O-]**	0
**CC1(C)OC(=O)N([C@H]2CC[C@H](C(=O)Nc3cccc4cccnc34)CC2)[C@H]1c1ccccc1**	1
**O=c1[nH]c(-c2ccccc2)cc2c1sc1ccccc12**	1
**Cc1ccc(cc1)CSc2nnc(s2)N3[C@H]([C@@H](C(=O)C3=O)C(=O)c4ccc(cc4)F)c5cc[nH+]cc5**	0
**Cc1c(nc(s1)/N=C(/c2cccs2)\[O-])c3ccc4c(c3)N(C(=O)CO4)CC=C**	0
**Cc1cc(c(c(c1C)S(=O)(=O)Oc2ccc(cc2OC)/C=C/C(=O)[O-])C)C**	0
**C/C(=N/NC(=O)c1cc(c[nH]1)Br)/[C@H]2C[C@H]2c3ccccc3**	0
**CN1CCCc2c1nc(N1CCC3(CC1)C(=O)N(CC(O)CO)c1cc(F)cc(F)c13)[nH]c2=O**	1
**Cc1cc(ccc1N[C@@H]2CCOC(C2)(C)C)I**	0
**Cc1c(sc(n1)N2[C@@H](/C(=C(\c3ccc(cc3)F)/O)/C(=O)C2=O)c4cccc(c4)[N+](=O)[O-])C**	0
**CC(C)n1ccc(n1)Cn2ccc3c2cc(cc3)C(=O)[O-]**	0
**c1cc(cnc1)c2nnc(s2)NC(=O)c3cc4cc(ccc4oc3=O)Br**	0
**Cc1c(c(sn1)Nc2ccc(c(c2)Cl)F)C#N**	0
**Cn1cc(c2c1cccc2)[C@H](CNS(=O)(=O)c3ccc(cc3)N4CCCCS4(=O)=O)N(C)C**	0
**COc1ccc(-c2noc(CSc3nnc(C)n3-c3cccc(C)c3)n2)cc1**	1
**Cc1cccc(c1)NC(=O)c2ccccc2NC(=O)Cn3c[nH+]c4c3c(=O)n(c(=O**	0

)n4C)C	
CCc1cccc(c1)N2C[C@H](CC2=O)C(=O)NNC(=O)c3c(nc(s3)N4CCOCC4)C	0
CC(=O)O[C@H]1[C@@H](c2c(ccc(c2O)/C=C\C(=O)N3CCOCC3)OC1(C)C)[NH+]4CCOCC4	0
CC1=C([C@H]([N+]2=C(CSC2=N1)CC(=O)NC(C)C)c3cccc(c3)Oc4ccccc4)C(=O)OCCOC	0
c1ccc(cc1)/C=C\2/CCCC3=C2Nc4ncnn4[C@H]3c5ccccc5	0
COc1ccc(C(=O)C2CCN([C@@H]3CCN(Cc4nc5ccccc(=O)c5[nH]4)C3=O)CC2)cc1C	1
c1ccc2c(c1)[C@H](CCS2(=O)=O)Nc3cccc(c3F)F	0
c1ccc(cc1)c2csc3c2[nH]c(c3)C(=O)N4CCC(CC4)C(=O)N5CCOCC5	0
CC1(C(=O)N(C(=O)N1C)C[C@H]([C@H](CC2CCCC2)C(=O)N3CCCCC3)C(=O)NO)C	0
Cc1ccc2c(=O)[nH]c(-c3ccc(C(C)(C)OCCO)cc3)cn12	1
Cc1cc(c2ccc(cc2[nH+]1)C(F)(F)F)Nc3ccc(cc3)S(=O)(=O)[N-]c4c(nccn4)OC	0
Cc1c(cccc1Cl)NC(=S)N2CCN(CC2)S(=O)(=O)c3ccc(cc3)OC	0
c1cc2c(cc1Br)nc([nH]2)/C(=C\c3csnn3)/Cl	0
Cc1ccn(n1)CCC(=O)N(Cc2ccc(c(c2)OC)OCCc3cccc[nH+]3)[C@H]4CCCCNC4=O	0
Nc1ccc(-c2nc3ccccc3c(=O)[nH]2)cc1	1
Cc1c(c(n(n1)c2ccc(cn2)NC(=O)c3cc(=O)c4ccccc4o3)C)Cl	0
c1cc(c(c(c1)Br)O)/C=N\c2[nH]cnn2	0
C[C@@H]1Cc2cc(ccc2O1)/C(=C/3\[C@@H](N(C(=O)C3=O)CCCn4cc[nH+]c4)c5ccc(cc5)OCC=C)/O	0
CCCN1C(=O)/C(=C/c2c(c(c(=O)n(c2N3CCN(CC3)CCO)CC)C#N)C)/SC1=S	0
COc1cccc(c1)[C@H]2C[C@@H](n3c(nnn3)N2)c4cc(ccc4F)Br	0
Cc1cccc2c(=O)[nH]c(-c3ccc(CNC(=O)OCc4ccccc4)cc3)nc12	1
C[C@H](C12CC3CC(C1)CC(C3)C2)NC(=O)[C@@H]4CCCN(C4)S(=O)(=O)N5CCOCC5	0
Cc1cc(nc2c1c3c(s2)[C@H](C(=C(O3)N)C#N)c4ccc(c(c4)F)Br)C	0
Oc1ccc2c(c1)sc1nncn12	1
C/C=C/C1OCC2(CO1)[C@H](CC(=C[C@@H]2C)C)C	0
c1c[nH+]c([nH]1)[C@@H]2CC(=O)N[C@H]3C2=C(N[NH+]3C4CCCCC4)[O-]	0
c1cc(c(c(c1N)[N+](=O)[O-])F)Br	0
C[C@H]1Cc2cc(ccc2N1C(=O)C3CC3)S(=O)(=O)N4CCC[C@@H](C4)C(=O)NCc5ccco5	0
Cc1cc(=O)[nH]c2cc(-c3ccc(F)cc3)ccc12	1
c1cc(cc(c1)NC(=O)c2ccc(cc2)S(=O)(=O)N3CCOCC3)c4ccc([nH+]n4)N5CCCC5	0
C[C@@H]1CCC[C@@H](C1)[C@@]2(CCCOC2)O	0
CCCCCc1nc2c(c3c(s2)CCC3)c(=O)n1N	0
CC1=C[C@H](N=N1)[C@H]2N=N[C@@H](N2/N=C/c3cccc(c3Cl)Cl)[S-]	0
O=C(c1ccc(F)cc1)C1CCN([C@H]2CCN(Cc3nc4c(c(=O)[nH]3)COCC4)C2=O)CC1	1
COc1cc(cc(c1OC)OC)[C@@H]2C(=C(C(=O)N2CCCn3cc[nH+]c3)O)C(=O)c4ccco4	0

**c1cc(sc1)c2n[nH]c(=S)n2/N=C/c3cc(cc(c3O)Br)Br**	0
**Cc1ncc(n1CCSc2nc3c(n2Cc4ccc(cc4)Br)c(=O)n(c(=O)n3C)C)[N+](=O)[O-]**	0
**c1ccc2c(c1)nc(s2)CN3CCN(CC3)C(=O)CSc4nnc(o4)c5ccc6c(c5)OCO6**	0
**Cc1nc(no1)c2cc(sc2)S(=O)(=O)N3CCC(CC3)C(=O)Nc4ccc(c(c4)Cl)OC**	0
**C[C@@H](C(=O)Nc1cccc(c1)S(=O)(=O)N(C)C)Oc2cccc(c2)Br**	0
**CN(C)c1ccc(cc1)c2c3c(nc(c2C#N)N)CCC3**	0
**C[C@H]1CCCCN1S(=O)(=O)c2ccc(cc2)C(=O)N(Cc3cccc[nH+]3)c4nc5ccc(cc5s4)F**	0
**c1cc(ccc1S(=O)(=O)N2CCC(CC2)c3cc(on3)N)Cl**	0
**CN([C@@H]1CCCc2c1cccc2)C(=O)Nc3ccc4c(c3)COC4=O**	0
**Cc1ccc(cc1)S(=O)(=O)Nc2ccc(=O)n3c2[C@@H]4C[C@H](C3)C[NH+](C4)C5CC[NH+](CC5)C**	0
**Cc1c(sc2c1c(=O)n(c(n2)C(C)C)[C@@H]3CCCN(C3)C(=O)c4c5c(n[nH]4)CCC5)C**	0
**CC1(C(C1(C)C)Nc2c3nncn3ccn2)C**	0
**C[C@@H]1CCC[C@@](C1)(C#N)[C@@]2(CCO[C@@H](C2)C)O**	0
**Cc1ccccc1NC(=O)c2cc3c(nn(c3nc2)C)C**	0
**CC(C)(C)c1ccc(-c2nc3ccc(F)cc3c(=O)[nH]2)cc1**	1
**Cc1ccc(cc1)S(=O)(=O)N[C@@H](C(C)C)C(=O)Nc2nc3ccc(cc3s2)NC(=O)C**	0
**CN1CCc2c1nc(N1CCC3(CC1)CN(CCO)c1cc(F)cc(F)c13)[nH]c2=O**	1
**CC(C)COc1ccc(cc1)/C(=C/2\[C@@H](N(C(=O)C2=O)Cc3ccc[nH+]c3)c4cccs4)/O**	0
**COc1cccc(N2CCN(c3nc4c(c(=O)[nH]3)CSCC4)CC2)c1**	1
**COc1ccc(C(=O)Nc2ccc(C(=O)N(c3ccncn3)c3ccccc3Cl)cc2)cc1**	1
**CCNC(=O)[C@]1(CCN(C1)C(=O)Cn2c(nc(n2)C)C)Cc3ccc(cc3)c4ccc[nH+]c4**	0
**Cc1cc(cc(c1)NS(=O)(=O)c2cc(sc2C)c3nc(on3)C(=O)N4CCCC4)C**	0
**CC(=O)N[C@@H]1c2ccccc2C3([C@H]1OCc4cc[nH+]cc4)CCN(CC3)C(=O)CCCC(=O)OC**	0
**CC1(CCC[C@]2(C1)CC(=O)NC2(C)C)C**	0
**Cc1c2c(oc1C(=O)NC(C)(C)C)CC(C[C@@H]2O)(C)C**	0
**c1ccc2c(c1)c(=O)n(cn2)CCC(=O)NC[C@H]3CCCN3c4[nH+]ccs4**	0
**CC1(CC(=C/C(=N\C)/C1)NC)C**	0
**C1C[C@@H](C[C@@H](C1)NC(=O)N2CC[NH+]3CCC[C@@H]3C2)C(=O)[O-]**	0
**COc1cc(cc(c1OC)OC)NC(=O)[C@@H]2c3cc(c(cc3C(=O)N4[C@H]2c5ccccc5CC4)OC)OC**	0
**O=c1[nH]c2sccc2c2ccccc12**	1
**Cc1ccc(cc1C)c2c(c(no2)N)Br**	0
**CC[C@@H](C)NC(=O)[C@@H](C)NC(=O)c1cc2n(n1)CCCN(C2=O)Cc3cccc(c3)C**	0
**CN1CCN(CC1)c2ccc(c[nH+]2)NC(=O)c3ccc4c(c3)OCO4**	0
**[H]/N=C\1/[C@@H]([NH+]=C(N1CC#C)C)C2CCC(CC2)C**	0
**CC(C)c1ccc(cc1)Nc2ncc(cn2)Br**	0
**CCc1c2c(n(n1)C)n(c(=S)[nH]2)[C@@H]3CCC[C@H](C3)C**	0
**c1cc(c(cc1O)O)C(=O)OCc2cc3c(cc2Br)OCCO3**	0

**C[C@@H]1C[C@H]1c2nc3c(c(n2)NN)CCCC3**	0
**Cc1c(c2c(s1)ncn(c2=O)CC(=O)NCCC[NH+]3C[C@H](C[C@@H](C3)C)C)S(=O)(=O)N4CCC(CC4)C**	0
**CC(=O)N1CCN(CC1)S(=O)(=O)c2cccc(c2)C(=O)Nc3cccc(c3)N4CCCC4=O**	0
**Cc1ccc(cc1)S(=O)(=O)N(CC(=O)N/N=C(\C)/CC(C)(C)c2ccccc2)c3cccc(c3)Cl**	0
**c1cc(cc(c1)Cl)[C@H]2C(=C(C(=O)N2CCCn3cc[nH+]c3)[O-])C(=O)c4ccncc4**	0
**CC(C)(C)OC(=O)NCCC(=O)/N=C/1\N([C@@H]2CS(=O)(=O)C[C@@H]2S1)c3cc(ccc3Cl)Cl**	0
**CC[C@]1(CCN(C[C@H]1O)Cc2cc(no2)C3CCCCC3)O**	0
**CC1(COCCN1[C@@H]2CCCCC[C@@H]2C#N)C**	0
**c1c(cc(c(c1C(=O)[O-])NC=O)Br)Br**	0
**C[C@@H](c1ccc(cc1)Cl)N(C)c2c(cc(cn2)CN)Cl**	0
**CC1(CCCN(C1)c2cc3c(cc2N)c(=O)[nH]cn3)C**	0
**CC(C)OCCCN1CC(=O)N2C[C@H](C3=c4ccccc4=[NH+][C@@H]3[C@]2(C1=O)C)c5ccc(c(c5)OC)OC(C)C**	0
**c1ccc(c(c1)[N-]S(=O)(=O)c2cccc(c2)C(=O)N3CCC[C@H]3c4ccc5c(c4)OCCCO5)F**	0
**C[C@]1(C(=O)Nc2ccccc2O1)C(=O)Nc3nc(cs3)c4ccc(cc4)Cl**	0
**C1CC[C@H]2C(=NN([C@]2(C(F)(F)F)O)C(=O)C3CC3)CC1**	0
**Cc1cc(c(c2c1C(=O)/C(=C/c3ccc4c(c3)OCO4)/O2)C[NH+]5C[C@H]6C[C@@H](C5)c7cccc(=O)n7C6)[O-]**	0
**c1ccc2c(c1)[nH+]cn2c3ccc(cc3)CNC(=O)C(=O)Nc4ccc(cc4)C#N**	0
**c1cc2c(cc1/C=C/c3csc(n3)N)OCCO2**	0
**COc1ccc(cc1OC)NC(=O)CS[C@H]2N=C3C(=CN=N3)C(=O)N2c4ccc(c(c4)Cl)F**	0
**Cc1ccc(c(c1)C(=O)[O-])NC(=O)N(C)[C@H](C)C(C)C**	0
**C[C@H](C(=O)NC(=O)N[C@H]1CCc2ccccc2C1)Cl**	0
**COc1ccc(cc1)S(=O)(=O)[C@@H](CNS(=O)(=O)c2ccc(cc2)OC)c3ccc[nH+]c3**	0
**CC(=O)OC[C@@H]1[C@H]([C@@H]([C@H]([C@@H](O1)O[C@H]2CC[C@@]3([C@H](C2)CC[C@@H]4[C@@H]3CC[C@@]5([C@@H]4CCC5=O)C)C)OC(=O)C)OC(=O)C)OC(=O)C**	0
**Cc1cc(no1)NC(=O)[C@H](c2ccccc2)N3CCC(CC3)C(=O)c4ccc5c(c4)OCCO5**	0
**Cn1cc(cc1C(=O)N)S(=O)(=O)N2CCC(CC2)C(=O)Nc3ccc(cc3)Br**	0
**Cc1c(sc2c1c3nnc(n3cn2)SCC(=O)Nc4ccccc4N5CCOCC5)C**	0
**O=C(c1ccccn1)C1CCN(CC(=O)N(Cc2nc3c(c(=O)[nH]2)COCC3)CC2CC2)CC1**	1
**Cc1ccc(cc1)N(Cc2nnc(n2C)SCC(=O)NCc3ccccc3)S(=O)(=O)C**	0
**c1cc(c(cc1[N+](=O)[O-])/C(=N/c2cnc(cn2)Br)/[O-])I**	0
**COc1ccc(-c2noc(CSc3nnc(-c4ccncc4)n3-c3ccc(C)cc3)n2)cc1**	1
**CC1CCN(CC1)C(=O)c2cn(cc(c2=O)C(=O)NC[C@H]3COc4ccccc4O3)C(C)C**	0
**COc1ccccc1NC(=O)/C=C/c2ccc3c(c2)OCO3**	0
**CC(=O)Nc1ccc(cc1)S(=O)(=O)NC[C@@H](c2ccc[nH+]c2)S(=O)(=O)c3cccs3**	0
**Cc1ccc(c(c1)Nc2nn3c(=O)c4ccc(cc4nc3s2)C(=O)NC[C@@H]5CCCO5)C**	0

**C1CCC2(CC1)CN(CCCN2)[C@@H]3CCOC3**	0
**c1cc2c(cc1N)oc(n2)Cc3ccsc3**	0
**CCCc1cc(c(s1)NC(=O)/C(=C/c2cccc(c2)OCc3cccc(c3)Cl)/C#N)C(=O)OC**	0
**c1cc(ccc1Cc2nc(on2)[C@@H]3C[C@H](C=C3)[NH3+])O**	0
**C[C@@H](c1ccccc1)N2CCn3c4ccc(cc4[nH+]c3C2)NC(=O)C**	0
**Cc1ccc(cc1N[C@@H]2[C@H]3CCO[C@@H]3C2(C)C)Br**	0
**c1cc2c(cc[nH+]c2cc1Cl)N3[C@H]4CC[C@@H]3[C@@H](C4)C(=O)[O-]**	0
**Cc1cc(c(o1)C)c2c(c(no2)N)Br**	0
**c1cc(c(cc1S(=O)(=O)N2CCOCC2)NC(=O)c3cc(ccc3Cl)Cl)N4CCCCC4**	0
**Cc1c(cc(s1)c2nc(on2)C3CCC3)S(=O)(=O)N4CCC(CC4)C(=O)NC5CCCCCC5**	0
**C[C@@H]1C[C@@H]2CCCC[C@@H]2N1C(=O)C3CC3**	0
**CCN(C(=O)N1CCC(C(=O)c2ccc3c(c2)cc(C(=O)O)n3C)CC1)c1ccc(OC)cc1**	1
**c1ccc(cc1)c2ccc(cc2)CN[C@H]3CO[C@H]4[C@@H]3OC[C@@H]4n5c(nnn5)Oc6ccc(cc6)n7ccnc7**	0
**c1cc(ccc1[C@H]2Cc3c(c[nH]n3)[C@H](C2)Cl)Cl**	0
**COc1cc2c(cc1OC)C(=[NH+]CC2)C[C@H]3CN(CC[C@H]3CC(=O)[O-])C(=O)N4CCOCC4**	0
**CC(c1ccc(F)cc1F)N(Cc1nc2c(c(=O)[nH]1)COCC2)C(=O)CN1CCC(C(=O)c2ccc(F)cc2)CC1**	1
**C[C@@H]1CCC[C@@H]([C@@H]1C)NS(=O)(=O)c2ccc(cc2)[O-]**	0
**CS(=O)(=O)c1cc(c(cc1Cl)Cl)C(=O)NCCCN2CCCCCC2=O**	0
**Cc1ccc(-c2nc3ccccc3c(=O)[nH]2)cc1**	1
**Cc1ccc2c(c1)sc(n2)N3CCN(CC3)C(=O)Cc4ccc(cc4)S(=O)(=O)C(C)C**	0
**CC(C)(C)[C@H]1CC[C@@H]([C@@H](C1)[C@@H]2CCO[C@@]3(C2)CCOC3)O**	0
**CCCNC(=O)[C@@H](C)NC(=O)c1ccc(c(c1)S(=O)(=O)N2CCc3c2cccc3)Cl**	0
**Cc1ccc(cc1)CNC(=O)CSc2c3c(nc(n2)c4ccccc4)n(c(=O)n(c3=O)C)C**	0
**c1cscc1n2c(=O)c3c4c(sc3[nH]c2=S)CCCC4**	0
**O=C1NN=C2CCCCc3[nH]c4cccc1c4c32**	1
**c1c(cc(c2c1OCCCO2)Cl)CSc3nnc(n3C4CC4)N**	0
**COCCn1c2c(c(n1)C(=O)NCc3ccc[nH+]c3)CN(CC2)C(=O)CCc4ccccc4Cl**	0
**CNc1c(ccc(n1)c2ccc(cc2)C#N)C#N**	0
**COc1ccc(C(=O)C2CCN([C@@H]3CCN(Cc4nc5c(c(=O)[nH]4)COCC5)C3=O)CC2)cc1**	1
**Cc1ccc(cc1)n2c(nnc2S[C@H](C)C(=O)N[C@H]3CCS(=O)(=O)C3)N4CCCC4**	0
**c1cc(ccc1[C@@H]2C(=C(c3ccc(cc3)S(=O)(=O)N4CCCC4)O)C(=O)C(=O)N2c5nccs5)F**	0
**CC(C)C(=O)N1CCc2c(nc3cc(nn3c2[O-])C4CC[NH+](CC4)C[C@H]5CCC=CC5)C1**	0
**Cc1ccc(c(c1)[C@@H]2CC(=O)Nc3c2c(nn3c4nc(cnn4)c5cccc(c5)OC)C)C**	0

**CC1(C)OC(=O)N(c2ccc(C(=O)Nc3cccc4cccnc34)cc2)[C@H]1c1ccccc1**	1
**N#Cc1ccc(-c2nnc(CSc3nnc(-c4ccncc4)n3-c3ccccc3Cl)o2)cc1**	1
**Cc1cccc(c1C)n2c3c(nc2C(F)(F)F)CNCC3**	0
**C#CCN(CC(=O)Nc1cc(nn1c2ccccc2)c3ccc4c(c3)CCC4)[C@H]5CCS(=O)(=O)C5**	0
**Cc1cc(n(n1)C)C[C@@H](c2cc3c(s2)CCCCC3)N[NH3+]**	0
**CC[NH+]1CCC[C@@H](C1)NC(=O)N2CCC(CC2)(C)C(=O)[O-]**	0
**CC(C)(C)Nc1nn(c(=S)s1)C[NH+]2CCN(CC2)S(=O)(=O)c3ccc(cc3)Cl**	0
**c1c(cc(cc1F)F)c2nc(on2)[C@]3(CCOC3)N**	0
**C[C@H]1CCC[C@H](C1)NC(=O)N2CCC[C@@H](C2)NC(=O)C**	0
**CCC(=O)N1CCN(CC1)C(=O)[C@@H](NC(=O)c2cccs2)S(=O)(=O)c3ccc(cc3)C**	0
**CCCN1C(=C([C@](C1=O)(C(F)(F)F)NS(=O)(=O)c2ccc(cc2)NC(=O)C)C(=O)OC)C**	0
**CC[C@H](C)NC(=O)CN1c2c(c(nn2c3cccc(c3)C)c4ccccc4)[C@@H](SCC1=O)c5ccc6c(c5)OCO6**	0
**O=C1CCN(c2ccccc2C(=O)NCc2ccc(-n3cccn3)cc2)C(=O)N1**	1
**C[C@@H]1C[C@H](CN(C1)S(=O)(=O)c2cccc(c2)C(=O)NCc3ccccc3n4cc[nH+]c4)C**	0
**Cc1ccc(cc1)NC(=O)C2CCN(CC2)S(=O)(=O)c3cc(sc3C)c4nc(on4)C**	0
**Cc1ccc2c(c1C)c(=O)n(C[C@H]1CC[C@H](C(=O)O)CC1)c(=O)n2Cc1cccc(C(F)(F)F)c1C#N**	1
**CNc1cc(ccc1Cl)S(=O)(=O)N**	0
**CC(=O)N1CCc2c1ccc(c2)S(=O)(=O)Nc3ccc(c(c3)S(=O)(=O)N4CCCCC4)Cl**	0
**C[C@H](c1ccc(cc1)F)N(C)C(=O)C[NH+]2CCC(CC2)NC(=O)Nc3ccc(cc3)F**	0
**Cc1ccc(cc1Cl)NS(=O)(=O)c2cc(ccc2OC)N3C(=O)C(CS3(=O)=O)(C)C**	0
**C[C@@H](c1cccs1)NC(=O)c2cc(ccc2[N+](=O)[O-])F**	0
**c1cc2c(cc1[C@@H]3COC(=O)N3)OCCCO2**	0
**c1cc2cn[nH]c2cc1OC(=O)c3c(ccs3)C#N**	0
**c1ccc(cc1)C(=O)CCC(=O)N2CCc3c(sc(n3)NC(=O)NC4CCCC4)C2**	0
**C[C@@H](c1[nH]c2ccccc2[nH+]1)NC(=O)c3ccc(=O)n(c3)CC(=O)N(C)c4ccccc4**	0
**CCN(CC)C(=O)C(=O)N/N=C\c1cc(n(c1C)c2cccc(c2)Br)C**	0
**CC(C)c1ccc2c(c1)sc1nncn12**	1
**c1ccc2c(c1)CC[C@H](C2)Nc3ccc(c([nH+]3)C(=O)[O-])Cl**	0
**CCN(CC)C(=O)CN1CCN(CC1)C(=O)c2c[n+](cc3c2nn(c3O)c4ccccc4)C(C)C**	0
**Cc1c(sc2c1c(nc(n2)SCc3nc(no3)c4cccc(c4)Br)N)C**	0
**CCOc1ccc(cc1)S(=O)(=O)N2CCN(CC2)c3cc(=O)n4c(n3)sc(n4)c5ccccc5**	0
**Cc1ccc(c(c1)S(=O)(=O)[N-]c2ccc(c(c2)NS(=O)(=O)c3ccc(cc3)F)OC)C**	0
**Cc1cnc(s1)NC(=O)c2ccc(cc2[O-])Br**	0

**C[NH+]1CCN(CC1)[C@H](CNC(=O)C(=O)Nc2ccccc2OC)c3ccc(cc3)N(C)C**	0
**C[C@H]1CC[C@H]([C@@H](C1)O)C(C)(C)c2ccco2**	0
**N#Cc1c(Cn2c(=O)n(C[C@H]3CC[C@H](C(=O)Nc4ccncc4)CC3)c(=O)c3ccccc32)cccc1C(F)(F)F**	1
**CC1([C@H]([C@H]2[C@@H]1OCCC2)Nc3ccc4cn[nH]c4c3)C**	0
**c1cc(oc1)[C@H]2C[C@@H](n3c(c(cn3)C(=O)Nc4ccc(cn4)Cl)N2)C(F)(F)F**	0
**c1cc(c(cc1[C@@H]([C@H]2CCO[C@@]3(C2)CCOC3)O)Cl)Cl**	0
**c1cc(c2nc(c(n2c1)N)C3CC3)Br**	0
**CCc1c(c2nnc3c(n2n1)ccn(c3=O)NC(=O)c4ccc5c(c4)OCCO5)c6cccc(c6)Cl**	0
**Cc1ccc(c(c1)NC(=O)/C(=C\c2c([nH]c3c2cccc3)C)/C#N)C**	0
**CN1C(=O)C(=Cc2ccc(c(c2)Cl)O)C(=O)N(C1=O)C**	0
**CC[NH+]1CCN(CC1)c2nc3c(n2C[C@H](C)CSc4nnc(s4)C)c(=O)[nH]c(=O)n3C**	0
**Cc1ccc2c(cc(=O)oc2c1C)CN3C(=O)[C@@](NC3=O)(C)c4ccc(cc4)C#N**	0
**C[C@]1(C(=O)N(C(=O)N1)NC(=O)c2cccc(c2)S(=O)(=O)N3CCCCCC3)c4ccccc4**	0
**CN1C(=O)C(=CNCCN(C)C)C(=O)N(C1=O)C**	0
**Cc1csc2c(=O)[nH]c(-c3ccc(C(C)(C)O)cc3)nc12**	1
**C[C@H]1CC[C@]2(C[C@@H]1C)C[C@H](c3ccc(cc3O2)F)O**	0
**COc1ccc(cc1OC)c2c(non2)NC(=O)COc3c(c(c(c3F)F)F)F)F**	0
**c1cc(ccc1NC(=O)c2c3c(n(n2)c4ccc(cc4)F)CCCC3)[N+](=O)[O-]**	0
**c1c(cc(c2c1[C@H](CCO2)N[C@@H]3CCC[C@@H]3C#N)Cl)Cl**	0
**c1ccc2c(c1)C[C@H](O2)/C(=N/N=C/C3=c4ccccc4=[NH+]C3)/[O-]**	0
**C[C@@H]1[C@H](CCN1C(=O)N2CCC(CC2)C(C)(C)C)C(=O)[O-]**	0
**c1cc(c(c(c1)F)C(=O)[O-])Oc2ccc3c(c2)OCO3**	0
**Cc1nc2cc(ccc2n1c3cc(ccc3F)Cl)N**	0
**CC(C)(CNC(=O)C#Cc1ccc2c(c1)OCO2)c3cc[nH+]cc3**	0
**Cc1c(c(no1)C(=O)NNS(=O)(=O)c2ccc(cc2)Cl)c3csc(n3)c4csc(n4)C**	0
**Cc1ccc(cc1C)S(=O)(=O)N2CC(C2)c3[nH]c4ccccc4n3**	0
**O=C(CCc1nc2ccccc2c(=O)[nH]1)N[C@H]1CC[C@H](c2nnc(-c3ccccc3)o2)CC1**	1
**CC[C@@H]1c2ccsc2CCN1C(=O)N[C@H]3CCCCNC3=O**	0
**Cc1ccc2c(c1)[nH]c(n2)Sc3ccc(o3)C(=O)[O-]**	0
**CC(C)C1Cc2[nH]c(=O)c3ccccc3c2CO1**	1
**C[C@@H](C1CC1)N(Cc1nc2c(c(=O)[nH]1)COCC2)C(=O)CN1CCC(C(=O)c2ccc(F)cc2)CC1**	1
**CC/C(=C(\c1cc(ccc1Br)C)/Cl)/C#N**	0
**Cc1ccccc1n2cc(c(n2)c3cc4ccccc4o3)C[NH3+]**	0
**c1cc(cc(c1)F)c2nc(on2)c3ccc[nH+]c3N4CCC(CC4)C(=O)NC[C@H]5CCCO5**	0
**c1ccc(cc1)[C@@H]2C[C@H]2Nc3cncc(n3)NN**	0
**CCOC(=O)N1CCN(CC1)C(=O)Cc2csc(n2)N**	0
**c1cc(cc(c1)Cl)C2=Nc3c(cnn3[C@@H]2NC4CCCCC4)C#N**	0
**Cc1cc(cc(c1C)S(=O)(=O)N)C(=O)N2CCCCC[C@H]2c3ccc(cc3)OC**	0

C[C@@H]1CC(=C(N1c2[nH]ncn2)C)C(=O)CSc3nnc(s3)NC[C@H]4CCCO4	0
Cc1c2c(cc(nc2n(n1)c3ccccc3C(F)(F)F)c4ccco4)C(=O)NCc5ccc[nH+]c5	0
Cc1ccc(cc1)n2c(=O)n(c(=O)c(n2)C(=O)NCc3cccnc3)Cc4cccc(c4)C	0
c1ccc(cc1)COc2cccc(c2)[C@@H]3C(=C(C(=O)N3CCCn4cc[nH+]c4)[O-])C(=O)c5ccco5	0
CCOc1ccc(cc1Cl)C(=O)C2=C(C(=O)N([C@H]2c3cc(c(c(c3)OC)OC)OC)CCCn4cc[nH+]c4)O	0
CN1C(=O)c2ccccc2N3[C@]1(CCC3=O)C(=O)OCC(=O)c4ccc(cc4)NC(=O)c5ccccc5	0
CC1(OC(=O)C(=CNc2ccc(c(c2)F)F)C(=O)O1)C	0
Cc1ccc2c(=O)[nH]c(-c3ccc(C4(O)COC4)cc3)cn12	1
CNC(=O)c1ccc(cc1)c2nc3cc(ccc3n2C)NS(=O)(=O)c4cc(ccc4F)F	0
CC[C@H]1CCC[C@]2(C1)CC(=O)NC(=O)[C@@H]2C(C)C	0
COc1cc(ccc1NC(=O)c2ccco2)NC(=O)/C=C/c3ccc(cc3)Cl	0
[H]/N=C/1\NC[C@@]2(N1c3ccc(cc3)Cl)CCCOC2	0
c1ccc(c(c1)N[C@]2(COc3c2ccc(c3)O)C#N)Br	0
Cn1c2ccc(cc2n(c(=O)c1=O)C)S(=O)(=O)CCC(=O)Nc3cccc(c3)Cl	0
Cc1cc[nH+]c(c1)N[C@H](c2ccc(c(c2)OC)OC)c3ccc4ccc(nc4c3O)C	0
COc1ccc(C(=O)C2CCN(C3CCN(CC(=O)Nc4noc5ncccc45)C3=O)CC2)cc1	1
C[C@H](c1ccccc1)N(Cc1nc2c(c(=O)[nH]1)COCC2)C(=O)CN1CCC(C(=O)c2ccc(F)cc2)CC1	1
Cc1coc2c1cc3c(c2)OC(=O)[C@H]([C@@H]3C)CCC(=O)NC4=CC5=CC=N[C@@H]5C=C4	0
Cc1cccn2c1nc(c2)CSc3[nH]ncn3	0
CC(=O)Nc1ccc(cc1)C[NH+]2CCc3c(c(nn3CC4CC4)C(=O)NCc5nccs5)C2	0
c1ccc2c(c1)C[C@](CO2)([C@H]3CCCC[C@@H]3O)C(=O)O	0
Cc1c(c(n(n1)c2ccccc2)C)C(=O)Nc3cccc(c3)S(=O)(=O)/N=C\4/CCCN4	0
C[C@H]1CCCN(C1)S(=O)(=O)c2ccc(cc2)NS(=O)(=O)c3ccc4c(c3)CC(=O)N4	0
CC(C)(C)[C@@H](C(=O)[O-])NC(=O)N(C)C1CC1	0
Cc1cccc2c(O)nc(-c3ccc([N+](=O)[O-])cc3)nc12	1
CCS(=O)(=O)N1CCc2ccc(cc2C1)S(=O)(=O)NCCc3ccccn3	0
c1c(cc(c2c1n[nH]c2)Br)C#N	0
c1cc2c(cc1Nc3nnc(s3)Br)OCCCO2	0
Cc1c(sc(n1)C)C(=O)C2=C(C(=O)N([C@H]2c3ccc(cc3)C(C)C)c4nc(c(s4)C(=O)C)C)[O-]	0
CC(C)N1[C@@H]([C@@H](c2ccccc2S1(=O)=O)O)Cc3cc(c(c(c3)OC)OC)OC	0
CN(C)S(=O)(=O)c1ccc(cc1)c2csc(n2)NC(=O)/C=C\c3ccsc3	0
CC(C)CC(=O)Nc1nnc2n1CCCS2	1
CN(CCCC(=O)Nc1cccc(c1)S(=O)(=O)N2CCCCC2)S(=O)(=O)c3cccs3	0
c1cc(c(c(c1)Cl)C(=O)NC[C@H](c2cccs2)O)Cl	0
[H]/N=C\1/C(=C/c2ccc(cc2)OCCCOc3ccccc3Cl)/C(=O)N=C4N1N=C(S4)c5cccnc5	0

**Cc1ccc2c(c(cnc2n1)C(=O)N3CCCC[C@H]3C)Nc4cccc(c4)C(=O)C**	0
**C1CC[C@@H]([C@H](C1)C(=O)N[C@@H]2CCCC[C@@H]2O)[NH3+]**	0
**C[C@H]1CCC[C@](C1)(C#N)N2CCNCC2**	0
**CC1(C[C@H](C(O1)(C)C)Nc2ccc(cc2Br)C#N)C**	0
**COc1ccc(C(=O)C2CCN([C@H]3CCN(Cc4nc5ccccc(=O)c5[nH]4)C3=O)CC2)cc1**	1
**CCc1c(cc2cccc(c2[nH+]1)C(=O)[O-])C**	0
**c1ccc2c(c1)c(c[nH]2)CCN3CCN([C@@H](C3)C(=O)N4CCOCC4)C(=O)c5ccco5**	0
**Cc1ccc(cc1Cl)S(=O)(=O)/N=c\2/cc(cc[nH]2)Cl**	0
**CC1(CC2=C([C@H](C(=C(O2)N)C#N)c3ccc(s3)Br)C(=O)C1)C**	0
**C[C@@H]1CC[NH2+]C2(CCCC2)CN1CCF**	0
**c1cc[nH+]c(c1)N2CCN(CC2)C(=O)c3ccc(cc3)[N-]S(=O)(=O)c4ccc5c(c4)OCCCO5**	0
**O=C(CCc1ccccc1)N(Cc1nc2c(c(=O)[nH]1)COCC2)Cc1nccs1**	1
**O=C(CN1CCC(c2nnc(-c3ccccc3)o2)CC1)NCc1nc2c(c(=O)[nH]1)COCC2**	1
**C[C@@H](c1ccc(c(c1)Cl)F)NS(=O)(=O)c2cc([nH]c2)C(=O)N3CCCC3**	0
**CC[C@@H]1CCCCN1C(=O)NCC(=O)[O-]**	0
**Cc1c(cno1)NC2CCN(CC2)c3ccc(cc3)N(C)C**	0
**CC(C)OCCCNC(=O)[C@]1(Cn2cnc(c2C(=O)N1C3CC3)C(=O)Nc4cc(ccc4F)F)C**	0
**Cc1ccc2c(c1)c(c(o2)c3nnc(o3)C(=O)NCCN4CCN(CC4)c5cc(ccc5C)Cl)C**	0
**Oc1nc(-c2ccccc2)nc2c(O)cccc12**	1
**COc1ccccc1N1CCCN(c2nc3c(c(=O)[nH]2)CCCC3)CC1**	1
**CCOc1ccc(cc1F)S(=O)(=O)NC[C@H](c2ccc3c(c2)CCN3C)N4CC[NH+](CC4)C**	0
**Cc1cccc(c1)C(=O)N2[C@H](COC23CCN(CC3)C(=O)C(C)(C)C)C(=O)NC4CC4**	0
**Cc1cc(n2c(n1)ncn2)N[C@H]3CC[C@@H]([C@@H](C3)C)C**	0
**O=C(CCc1ccccc1O)N(Cc1nc2c(c(=O)[nH]1)COCC2)Cc1cccs1**	1
**Cc1cccc(c1)N(CC(=O)N/N=C\c2cc(n(c2C)c3ccc(cc3)Br)C)S(=O)(=O)C**	0
**c1cc(cc(c1)F)n2c(c(nn2)c3nc(cs3)c4ccc(cc4)F)N**	0
**Cc1c(c(n(n1)c2nc3ccccc3s2)O)[C@H]4CC(=O)N(C4=O)c5ccccc5F**	0
**Cc1cc2c(s1)nc(n(c2=O)NC(=O)c3cnn4c3nc(cc4C(F)(F)F)c5ccco5)C**	0
**CCCNS(=O)(=O)c1ccc(cc1)NC(=O)[C@H]2CC(=O)N(C2)C3CCCCC3**	0
**c1cc(cc(c1)n2c(cc(n2)c3cccc(c3)Cl)N)C#N**	0
**Cc1ccc(cc1)COc2ccc(cc2)/C=C\3/C(=[NH2+])N4C(=[NH+]SC4=NC3=O)SC**	0
**Cc1c(c(n(n1)C)C)CN2CCN(CC2)C(=S)Nc3ccc(c(c3)OC)OC**	0
**CC(C)(O)c1ccc(-c2nc3ccc(F)cc3c(=O)[nH]2)cc1**	1
**Cc1ccc(c(c1)C)[C@H]2C(=C(N(C3=C2C(=O)CCC3)c4ccc(cc4[N+](=O)[O-])Cl)N)C#N**	0
**O=c1[nH]c2ccccc2c2ccccc12**	1

**c1ccc(cc1)/C=N/N2CC(=O)N3[C@@H](C2=O)Cc4c5ccccc5[nH]c4[C@@H]3c6ccccc6**	0
**c1ccc(cc1)C(c2ccccc2)C(=O)/[NH+]=C/3\C=CC4=NC(=O)NC4=C3**	0
**C[C@H](C(=O)Nc1nc(cs1)c2ccc(cc2)S(=O)(=O)N3CCOCC3)Oc4ccccc4Cl**	0
**c1cc(c(cc1N)Oc2c(cc3c(n2)CCC3)C#N)Cl**	0
**c1cc(cc(c1)[N-]S(=O)(=O)c2c(cc(cc2Br)F)F)c3nnnn3C4CC4**	0
**O=C(Nc1ccc(C(F)(F)F)cc1)c1ccc(NC(=O)N2CCN(C(=O)c3cc(Cc4n[nH]c(=O)c5ccccc45)ccc3F)CC2)cc1**	1
**Cc1cnc(cn1)C(=O)Nc2c(c(c(s2)C)C)C#N**	0
**COc1ccc(cc1)CNS(=O)(=O)c2cc(ccc2OC)N3C(=O)CCS3(=O)=O**	0
**COc1cc(ccc1NC(=S)NC(=O)c2cc3ccccc3c(c2OC)Br)[N+](=O)[O-]**	0
**Cc1ccc(cc1C)S(=O)(=O)C2(CCCC2)C(=O)Nc3ncc(s3)C(=O)OC**	0
**CC1(C[C@H](c2cc3c(cc2O1)OCO3)O)C**	0
**C[NH+]1CCN(CC1)[C@H](CNC(=O)c2cc(on2)c3ccccc3)c4ccc5c(c4)OCO5**	0
**CCOC(=O)c1cc2c(nc3ccccn3c2=O)[n+](c1NC(=O)C(c4ccccc4)c5ccccc5)C(C)C**	0
**Cc1nc2n(n1)c(c(s2)[C@@H](c3ccccc3Cl)[NH+]4CCN(CC4)c5ccccc5F)[O-]**	0
**c1cc(nc2c1cc(cc2)S(=O)(=O)N3CCCC3)N4CCC[C@@H](C4)C(=O)Nc5ccc6c(c5)OCCO6**	0
**C[C@]12CCC[C@@]3([C@@H]1C[C@H]4[C@@H](C2)OC(=O)[C@@H]4C[NH2+]Cc5ccc[nH+]c5)CO3**	0
**CC(C)Cn1c2cc(c(cc2nc1N)Cl)Cl**	0
**O=C(CCc1ccncc1)N(Cc1nc2c(c(=O)[nH]1)COCC2)Cc1cccs1**	1
**O=C(NCc1ccccc1)Nc1nnc2n1CCS2**	1
**c1cc2c(cc[nH]2)cc1NC(=O)[C@H]3CC=CC[C@H]3C(=O)O**	0
**O=C(CCn1cncn1)Nc1nnc2n1CCS2**	1
**c1cc2c(cc1NC(=O)c3ccc(o3)Br)nc(o2)c4ccncc4**	0
**COCCNC(=O)c1ccc(-c2ccc3c(C)cc(=O)[nH]c3c2)c(Cl)c1**	1
**CCc1c(c(n(n1)C)C[C@@H](C(CC)(CC)N(C)C)[NH3+])Br**	0
**COc1cc(cc(c1OC)OC)C(=O)NN[C@@H](c2ccccc2)C(=O)C3=c4ccccc4=[NH+]C3**	0
**c1cc(oc1)c2cc(n3c(n2)cc(n3)C(=O)Nc4ccc(cc4F)F)C(F)(F)Cl**	0
**Cc1c(n2ccccc2[nH+]1)/C(=C/3\[C@H](N(C(=O)C3=O)CCCn4cc[nH+]c4)c5ccc(cc5)Cl)/O**	0
**Cc1c(c(n(n1)c2ccc(cc2)F)Cl)C(=O)Nc3cc4c(cc3Cl)OCCO4**	0
**Cc1cc(=O)[nH]c2cc(-c3ccc(C(=O)NCCN4CCOCC4)cc3Cl)ccc12**	1
**CC(=O)Nc1ccc(cc1)NC(=O)c2ccc(cc2)S(=O)(=O)N3CCN(CC3)C=O**	0
**O=C(N[C@H]1C[C@H](c2nnc(-c3ccncn3)n2-c2ccccc2Cl)C1)c1ccccn1**	1
**Cc1c(sc(n1)C)C(=O)C2=C(C(=O)N([C@@H]2c3ccccc3OC)CC[NH+](C)C)[O-]**	0
**COc1ccc(C(=O)C2CCN(C3CCCN(Cc4nc5c(c(=O)[nH]4)COCC5)C3=O)CC2)cc1**	1
**C1CC(C1)Oc2nc(nc(n2)Cl)Cl**	0
**COc1ccc(cc1OC)c2c(c(n(n2)Cn3ccc(n3)[N+](=O)[O-])c4ccc(c(c4)OC)OC)Br**	0

c1cc(cc(c1)S(=O)(=O)N2CCOCC2)NC(=O)Cn3cnc4c(c3=O)cc(cc4Cl)Cl	0
C1CCc2c(c(c(s2)N)c3nc(no3)[C@@H]4CCCOC4)C1	0
CN(c1ccc(cc1)C#N)C(=O)c2c3ccccc3[nH]n2	0
Cc1ccn2c(c1)nc(n2)N3CCCNCC3	0
c1cc(cc(c1)[N+](=O)[O-])/C=N/c2ccc(cc2)S(=O)(=O)[N-]c3ccc(cc3)I	0
Cc1cccc(c1)OCCCn2cc(c3c2cccc3)/C=C\4/C(=[NH2+])N5C(=[NH+]SC5=NC4=O)S(=O)(=O)C	0
Cc1ccn\2c(c1)n[nH]/c2=N\C(=O)[C@@H]3C[C@H]3c4ccc(s4)C	0
CCc1c(c(nc([nH+]1)c2cc3c(c(c2)Cl)OCCO3)N)Br	0
N#Cc1c(Cn2c(=O)n(C[C@H]3CC[C@H](C(=O)O)CC3)c(=O)c3c(Cl)cccc32)cccc1C(F)(F)F	1
Cc1ccc2c(c1)N(C[C@@H](O2)C(=O)Nc3c(n(n(c3=O)c4ccccc4)C)C)S(=O)(=O)C	0
C[C@H](c1ccc2c(c1)OCCO2)NC(=O)c3cccc4c3C(=O)N(C4)CCOC	0
Cc1ccc(cc1N[C@H]2CCO[C@@]3(C2)CCOC3)F	0
c1ccc(cc1)C2=C([C@@H](NC(=O)N2)c3ccccc3[N+](=O)[O-])c4nc5ccccc5s4	0
CCCOC(=O)CCc1cc2c(cc(=O)oc2c(c1[O-])C[NH+]3C[C@H]4C[C@@H](C3)c5cccc(=O)n5C4)C	0
Cc1cc(F)cc2c(=O)[nH]c(-c3ccc(C(C)(C)OCCO)cc3)nc12	1
c1cc(ccc1NC(=O)[C@@H]2Cc3cc(ccc3O2)Cl)N4CCCS4(=O)=O	0
c1ccc(cc1)c2cc(c3cnn(c3n2)Cc4cccc(c4)F)C(=O)NNC(=O)C5CC5	0
Cn1cc[nH+]c1[C@@H](c2ccccc2OC)NC(=O)NCc3coc(n3)c4cccs4	0
c1cc(c(cc1F)C(=O)NC2(CCCC2)C#N)Br	0
O=c1[nH]c(N2CCC3(CC2)CN(CCO)c2cc(F)cc(F)c23)nc2c1CCCC2	1
Cc1ccccc1n2c3c(cn2)c(nc(n3)SCC(=O)N4CCN(CC4)c5ccccc5)O	0
c1cc2c(cc1Br)nc(o2)[C@@]3(CCOC3)N	0
c1ccc2c(c1)[nH]c(n2)/C(=C/c3cccc(c3)O)/C#N	0
CC(=O)c1ccc(c(c1)OC)OCC(=O)NCCN2C(=O)/C(=C\c3ccc(cc3)Cl)/SC2=O	0
CC1(CCCN1C(=O)NC(=O)CC(C)(C)C(=O)O)C	0
Cc1c2c(c(sc2n([nH+]1)C)C(=O)N[C@@H]3CC[C@@H]([C@@H]3C)C)N	0
CCC[C@@H](c1nnc2n1cccc2)NC(=O)NC3(CC3)c4ccccc4F	0
C[C@]12CC[C@](C1(C)C)(CC2=O)C(=O)NCCN(CCNC(=O)[C@]34CC[C@](C3(C)C)(C(=O)C4)C)CCNC(=O)[C@@]56CC[C@@](C5(C)C)(C(=O)C6)C	0
O=c1[nH]nc2c3c(cccc13)NC1=C2CCCC1	1
O=C(CCc1ccco1)Nc1nnc2n1CCS2	1
c1ccc2c(c1)cc(o2)C(=O)C3=C(C(=O)N([C@@H]3c4cccnc4)c5nc6ccc(cc6s5)F)[O-]	0
C[C@@H]1C[C@@](CC(C1)(C)C)(C(=O)[O-])Nc2ccccc2Br	0
Cc1ccc(c(c1)C)N2C[C@@H](CC2=O)C(=O)Nc3cccc([nH+]3)C	0
CC[C@@H](C)c1cnc2c(c1SCC(=O)Nc3nc4ccc(cc4s3)C)c(=O)n(c(=O)n2C)C	0
CCOc1ccc(-c2nnc([C@H]3C[C@H](NC(=O)c4cc(F)cc5nccnc45)C3)n2-	1

c2ccccc2F)nc1	
CCOc1ccc(cc1OC)[C@H]2CC3=C([C@@H](CC(=O)N3)c4cc5c(cc4Br)OCO5)C(=O)C2	0
Cc1c(c(on1)C)S(=O)(=O)N2CCC[C@H](C2)C(=O)NCc3ccc(cc3)c4csc(n4)C	0
c1cc2c(cc1N)sc(n2)N3CCN4CCCC[C@@H]4C3	0
Cc1cc(F)cc2c(=O)[nH]c(-c3ccc(C4CCN(C)CC4)cc3)nc12	1
Oc1nc(N2CCN(c3ccccc3)CC2)nc2c1CCCC2	1
c1cc(c(cc1CSc2ccnc(c2)C(=O)[O-])F)[N+](=O)[O-]	0
CC(C)C1CC2=NNC(=O)c3cccc4[nH]c(c2c34)C1	1
Cc1c(nc2c(c1c3cccs3)c(ncn2)N)C	0
C1CCN(CC1)[C@@]2(CCC[C@H](C2)C3CC3)C#N	0
CCc1c(nnn1c2cc(ccc2F)Cl)N	0
CC(C)(C)c1ccc(C(=O)Nc2nnc3n2CCCS3)cc1	1
c1cc2c(cc1N[C@H]3CCC[C@H]3O)OCCO2	0
CC[NH+]1CCN(CC1)[P@](=S)(/C(=N\c2ccccc2)/N3CCN(CC3)CC)N(C)C	0
CC(C)Cc1nc2c(ccs2)c(n1)Cl	0
CCOc1ccc(-c2nnc([C@H]3C[C@H](NC(=O)c4cccc5nc(C)cnc45)C3)n2-c2ccccc2F)nc1	1
C[C@@H]1[C@@H](CCO1)[C@@H](c2ccc3c(c2)CCCC(=O)N3)Cl	0
CCc1cc(n[nH]1)C(=O)N2CCOC(C2)(C)C	0
CC(C)(O)c1ccc(-c2nc3ccccc3c(=O)[nH]2)cc1	1
Cc1ccc2c(c1C)c(=O)n(C[C@H]1CC[C@H](C(=O)Nc3ccncc3)CC1)c(=O)n2Cc1cccc(C(F)(F)F)c1C#N	1
Cc1ccc(cc1)C(=O)N2[C@@H](COC23CCN(CC3)C(=O)c4cccc(c4)[N+](=O)[O-])C(=O)NC[C@@H]5CCCO5	0
CCOc1cc(ccc1O)[C@@H]2c3c(n[nH]c3C(=O)N2CCOC)c4c(cc(cc4O)C)C	0
CC1(CC2=C([C@H](C(=C(O2)N)C#N)c3ccc(cc3)C#N)C(=O)C1)C	0
c1cc2c(ccc3c2OC(=C([C@H]3c4cccnc4)C#N)N)c(c1)N	0
COc1ccc(c(c1)[C@H]2/C(=C(\c3ccco3)/O)/C(=O)C(=O)N2CCCn4cc[nH+]c4)OC	0
Cc1cc2ccc3cccnc3c2nc1N	0
Cc1ccc(cc1)S(=O)(=O)N2CCN(CC2)CC(=O)Nc3ccc4c(c3)OC(O4)(F)F	0
CN(c1ccccc1OC)C(=O)c2cc(c(c(c2)Cl)Cl)N	0
O=c1[nH]c(N2CCC3(CC2)NCc2ccccc23)nc2c1CCCC2	1
c1c2c(cc3c1c(c(nn3)Cl)[O-])CCCC2	0
Cc1c(non1)CN[C@H]2CCc3ccccc3C2	0
O=C1NC(c2ccc(OCc3ccccc3)cc2)Nc2ncccc21	1
Cc1ccc(cc1/N=C/c2cccc(c2O)OC)S(=O)(=O)N(C)C(C)C	0
[H]/N=C/1\C(C2=CC[NH+](C[C@@H]2[C@@H](C1(C#N)C#N)[C@@H]3CCCC=C3)CC)C#N	0
Cc1c(c([nH]c1C(=O)[C@H](C)N2CCC(CC2)c3nc4ccccc4s3)C)C(=O)OC	0
Cc1cccc(c1)NC(=O)N2CCC[C@@]3(C[NH+](C[C@@H]3C2)Cc4cnn(c4)C)C(=O)N(C)C	0
CC(C)c1ncc(c(n1)CN)N2CCC[C@@H]2C(C)C	0

**Cc1ccc(cc1C(F)(F)F)n2c(c(nn2)N)C**	0
**O=C(Nc1nnc2n1CCS2)C1CCCC1**	1
**COc1cccc(c1)[C@H]2C[C@H](n3c(ncn3)N2)c4cccc(c4)F**	0
**COc1ccccc1NC(=O)c1ccc(NC(=O)N2CCN(C(=O)c3cc(Cc4n[nH]c(=O)c5ccccc45)ccc3F)CC2)cc1**	1
**CC[C@H](C)c1nc(c(c(n1)N2CCN(CC2)C(=O)C(C)C)Cc3ccc(cc3)[N+](=O)[O-])C**	0
**C[C@H]1CCN([C@@H](C1)C)c2[nH+]cc(s2)C(=O)[O-]**	0
**Cc1cc(F)cc2c(=O)[nH]c(-c3ccc(N4CCN(C)CC4)cc3)nc12**	1
**CN1CCC(Oc2cc(F)c3c(c2)N(C)C(=O)C32CCN(c3nc4c(c(=O)[nH]3)CCCN4C)CC2)CC1**	1
**Cc1c(c(n(n1)c2ccccc2)C)NC(=O)CCc3nc4cc(ccc4n3C)S(=O)(=O)N(C)C**	0
**c1cc(sc1)/C=C/2\CCC[C@@H]3C2=NN([C@H]3c4cccs4)C(=O)c5ccc(c(c5)S(=O)(=O)N6CCOCC6)Cl**	0
**Cc1ccsc1c2nnc(n2N)S**	0
**c1cc(c(cc1Cl)OCC(F)(F)F)NC(=O)N2CCC[C@H](C2)O**	0
**O=C(c1ccc(F)cc1)C1CCN(CC(=O)N(Cc2nc3c(c(=O)[nH]2)COCC3)C[C@H]2CCCO2)CC1**	1
**Cn1c2ccccc2nc1CN3CCOC[C@H]3c4[nH]cc([nH+]4)c5ccc(cc5)S(=O)(=O)C**	0
**CC1(CCC[C@]1(C#N)N(C)C)C**	0
**Cc1c(cnc(n1)C)/C(=C\2/[C@@H](N(C(=O)C2=O)CCCn3ccnc3)c4cccc(c4)F)/O**	0
**CC(C)(CO)NC(=O)[C@@H]1CCCC[C@@H]1[NH3+]**	0
**Cc1ccc(cc1)c2cn3c4c([nH+]c3n2c5c(n(n(c5=O)c6ccccc6)C)C)n(c(=O)n(c4=O)C)C**	0
**Cc1c(nc2c(c(nn2c1NCCCN3CCCC3=O)C)c4ccccc4)C**	0
**C[C@H](C(=O)Nc1ccc2c(c1)OCCO2)NC(=O)[C@H]3Cc4ccccc4CN3C(=O)OC(C)(C)C**	0
**O=C(Nc1nnc2n1CCS2)c1ccccc1**	1
**[H]/N=C(/N1[C@H](CC(=N1)c2ccccc2)c3ccccc3)\S[C@@H]4CC(=O)N(C4=O)c5cccc(c5)Br**	0
**COc1cccc(c1)N2[C@@H](SCC2=O)c3cccc(c3)[N-]S(=O)(=O)c4ccc(cc4)Br**	0
**C[C@H]1CCCCC[C@H]1NC(=O)NC[C@@H](C)C(=O)[O-]**	0
**CC(C)Nc1cc([nH]n1)c2ccc(cc2Cl)F**	0
**c1ccc(c(c1)[C@H]2c3cn[nH]c3N=C(CS2)O)Cl**	0
**O=C(c1ccc(C(F)(F)F)cc1)C1CCN(CC(=O)N(Cc2nc3c(c(=O)[nH]2)COCC3)CC(F)F)CC1**	1
**CN(C[C@H]1CC=C(S1)Cl)C(=O)CSc2nnc(n2C3CC3)C4=c5cccc c5=[NH+]C4**	0
**c1cc(cc(c1)N)c2nc3cccc(c3o2)Cl**	0
**CN1CCO[C@@H](C1)c2nc(c(c(n2)N)Br)C3CC3**	0
**Cc1ccc(cc1)/C=C/c2c(c(no2)C)S(=O)(=O)N3CCC(CC3)C(=O)Nc4c(cc(cc4C)C)C**	0
**O=C(CCc1ccccc1)N(Cc1nc2c(c(=O)[nH]1)COCC2)Cc1ncco1**	1
**c1cc(c(cc1F)C#CC[NH3+])[N-]S(=O)(=O)c2c[nH+]c[nH]2**	0
**CCCn1c(c(c(c(c1=O)C#N)C)/C=C/2\C(=O)N(C(=S)S2)[C@H](C)CC)N3CCC(CC3)C(=O)OCC**	0
**C[NH2+][C@H]1CCCc2c1sc(n2)c3[nH]ncn3**	0
**CCOC(=O)c1c(nc(s1)N2[C@H]([C@@H](C(=O)C2=O)C(=O)c3**	0

cccs3)c4ccc(cc4)[N+](=O)[O-])C	
CCOc1ccc(cc1Cl)C(=O)[C@H]2[C@H](N(C(=O)C2=O)CCCn3cc[nH+]c3)c4ccc(cc4)[N+](=O)[O-]	0
Cc1cnc(c(n1)N2CCNCC23CCCCC3)C	0
O=C(N[C@H]1C[C@H](c2nnc(-c3ccccn3)n2-c2ccccc2F)C1)c1ccccn1	1
c1cc(cc(c1)[N+](=O)[O-])c2ccc(o2)/C=C(\C#N)/c3[nH]c4cc(c[nH+]c4[nH+]3)Br	0
c1ccn2c(c1)nc(c(c2=O)/C=C\3/C(=O)N(C(=S)S3)C4CCCC4)NCCCn5ccnc5	0
Cc1c(sc2c1c(ncn2)N[C@H]3CCCc4c3cccc4)C	0
C[C@@H]1CCC[C@]2(C1)CS/C(=N\c3ccc(cc3)CC#N)/N2	0
c1cc(ccc1c2nc3ccc(cc3c(n2)N)F)F	0
c1cc2c(c(c1)Cl)[nH]c(n2)[C@H]3CCCCS3	0
CC(C)[C@H](C(=O)N1CCN(CC1)S(=O)(=O)c2ccccc2C#N)NC(=O)Cc3ccccc3	0
C[C@@H](C(=O)Nc1ccc(cc1)S(=O)(=O)N2CCCC2)Sc3nnc(n3C)c4ccncc4	0
CC(C)CN(Cc1nc2c(c(=O)[nH]1)COCC2)C(=O)CN1CCC(C(=O)c2ccc(F)cc2)CC1	1
Cn1c(c(cn1)OC)[C@@H](c2cc3c(s2)CCCC3)NN	0
CC(C)[C@H]1[C@H](CC(=O)NC1=O)C2CCCCC2	0
CCCNC(=O)N1CCc2c1ccc(c2)S(=O)(=O)NC3CCCCCCC3	0
c1cc(sc1)c2cc([nH]n2)c3nc(no3)[C@H]4CC(=O)N(C4)c5ccc6c(c5)CCC6	0
CC1(CCC(CC1)Nc2cc(ncn2)Cl)C	0
CCOc1ccc(-c2nnc([C@H]3C[C@H](NC(=O)c4cccc5cccnc45)C3)n2-c2ccc(C)s2)nc1	1
Cc1c(c(n(n1)C)N[C@@H]2CCC[NH2+]C2)C#N	0
Cn1cc(cc1C(=O)N(C)C2CCCCCC2)N	0
Cn1c(nnc1SCc2cc3c(c(c2)Cl)OCCO3)N	0
C[C@@H](c1ccc2c(c1)OCCO2)NC(=O)CSc3ccc(c[nH+]3)S(=O)(=O)N4CCCC4	0
O=c1[nH]c(CNCc2cccs2)nc2c1COCC2	1
Cc1c(c([nH]c1C(=O)OC)C)C(=O)[C@H]2[C@H](N(C(=O)C2=O)CC[NH+](C)C)c3ccc(cc3)C(=O)OC	0
O=C(c1cc(Cc2n[nH]c(=O)c3ccccc23)ccc1F)N1CCn2c(nnc2-c2ccc(Br)o2)C1	1
CC[NH+]1CCN(CC1)[C@@H](C)CNc2c3c(csc3nc(n2)CN4CCOCC4)c5ccccc5	0
CC[C@@H]1CC[C@@H]([C@@H]1C)Nc2cnc(cn2)C(=O)[O-]	0
C[C@@H]1CCN([C@@H](C1)C)Cc2ccc(c(c2)NC)[N+](=O)[O-]	0
CC(=O)N1CCOc2c1cc(cc2)S(=O)(=O)N3CCC(CC3)C(=O)Nc4cccc(c4)F	0
Cc1c(c2c(o1)CCCC2)C(=O)N3CC[C@@H](C3)c4c5c(nccn5)n(n4)CC[NH3+]	0
Cn1c(cc(n1)C(=O)NCCn2c[nH+]c3c2cccc3)NC(=O)Cc4ccccc4	0
C[C@H]1Cn2c(nnc2C(C)(F)F)CN1C(=O)c1cccc(C(F)(F)c2n[nH]c(=O)c3ccccc23)c1	1
c1cc(ccc1c2csc(n2)N3CCN(CC3)S(=O)(=O)c4ccc5c(c4)NC(=O)CO5)Cl	0

COc1ccc(cc1NC(=O)CSc2ccc(cn2)C(F)(F)F)S(=O)(=O)N3CCOCC3	0
c1ccc(cc1)[C@H]2C=C3C(=NC(=NC3=O)CSC4=NN[C@@H](O4)CN5CCCC5=O)S2	0
C[C@@H]1CCc2c(sc3c2c(ncn3)SCC4=NC(=O)C5=C([C@H](SC5=N4)C(=O)[O-])C)C1	0
COc1ccc(C(=O)C2CCN(CC(=O)N(Cc3nc4c(c(=O)[nH]3)COCC4)CC3CC3)CC2)cc1	1
[H]/N=C/1\[C@@H]([NH+]=C(N1CC#C)C2CC2)C3CC3	0
COc1cccc(c1)C(=O)/C=C\Nc2ccc(cc2)S(=O)(=O)Nc3cnc4ccccc4n3	0
c1ccc(cc1)CNC(=O)[C@@H](c2ccccc2)N(Cc3ccc[nH+]c3)C(=O)c4csnn4	0
c1cc(sc1)[C@@H](c2cc3c(cc2Cl)OCCCO3)O	0
O=c1[nH]c(SCCN2CCN(CCSc3nc4ccccc4c(=O)[nH]3)CC2)nc2ccccc12	1
CCc1ccc(cc1)[C@@H]2Nc3ccccc3S(=O)(=O)N2	0
Cc1c(sc2c1c(ncn2)N[C@@H]3CCCCC[C@H]3C)C	0
CN1CCCc2c1nc(N1CCC3(CC1)C(=O)N(CC(=O)O)c1cc(F)cc(F)c13)[nH]c2=O	1
COc1cccc(c1)[C@H]2c3c(c4ccccc4[nH]3)C[C@@H]5N2C(=O)CN(C5=O)CCc6ccc(c(c6)OC)OC	0
CCOC(=O)C1=C(Oc2c(c(nn2c3ccccc3)C)[C@H]1c4ccc(cc4)N(C)C)N	0
Cc1nc2c(s1)cc(c3c2scc3)O[C@H]4CCCC[C@@H]4O	0
c1c(c(nc(n1)C2CCCCCC2)N)Br	0
Cc1cnc(s1)Nc2cc(nc(n2)C)[C@@H]3CCC[NH+]3Cc4cnc(nc4)CCOC	0
C[NH+]1CCN(CC1)[C@H](CNS(=O)(=O)c2ccccc2Br)c3ccc4c(c3)OCO4	0
C[C@@H]1CC[C@H](C[C@@H]1C)n2c(c(cn2)Br)N	0
Cc1cccn2c1nc(c(c2=O)/C=C\3/C(=O)N(C(=S)S3)CCCCCC(=O)[O-])N4CC[NH+](CC4)C	0
c1ccc2c(c1)[C@@H](CS2(=O)=O)NC(=O)c3c(n(nn3)c4cccc(c4)F)C(F)(F)F	0
CCN\1C(=O)/C(=C/c2c(cccc2Cl)F)/S/C1=N\c3nnc(s3)C	0
Cc1cc(nc(n1)S[C@H]2[C@H](C[C@@H](C[C@@H]2[NH2+]C)C)C)[O-]	0
c1cc(c(cc1[N+](=O)[O-])Cl)O[C@H]2[C@@H]([C@H]([C@@H]([C@H](O2)CO)O[C@H]3[C@@H]([C@H]([C@@H]([C@H](O3)CO)O)O)O)O)O	0
C[C@H]1CCc2ccccc2N1C(=O)c3cc(cn3C)N	0
C[C@H](Cn1cncn1)NC(=O)c2coc(n2)C[NH+]3CCN(CC3)C/C=C/c4ccccc4	0
Cc1ccc2cc(ccc2n1)[C@@H]3C(=[NH2+])N(C=[NH+]3)C4CC4	0
CC(C)C1=NO[C@@H](C1)c2nc(no2)c3ccc(cc3)F	0
Cc1cc(cc(c1)N(C)c2c(cc(cn2)N)C)C	0
CC1=C([C@@H](C2=C(N1)CC(CC2=O)(C)C)c3ccc(c(c3)[N+](=O)[O-])Cl)C(=O)Nc4nc5ccc(cc5s4)Br	0
CCCOC(=O)C1=C(N=C2C[C@H](CC(=O)C2[C@H]1c3cc(c(c(c3)Br)O)OC)c4ccc(c(c4)OC)OC)C	0
CC1(CCC(=O)N(CC1)CC#C)C	0

**C/C(=C/c1ccco1)/CN2CCc3c(c(n[nH]3)c4ccc5c(c4)OCO5)C2**	0
**CC[C@H]1CC[C@H]([C@@H](C1)n2cnc3c2cc(c(c3)C)C)O**	0
**COc1ccc(OC)c2c1sc1nncn12**	1
**Cc1cc(c(n1c2ccc(cc2)Br)C)/C=N\N=C(\[C@@H]3COc4ccccc4O3)/[O-]**	0
**Cc1nnc(s1)NC(=O)CSc2nnc3n2cnc4c3c(cs4)c5ccc(cc5)Cl**	0
**CS(=O)(=O)NCc1cc2n(n1)CCC[NH+](C2)Cc3ccc(cc3)C(=O)[O-]**	0
**Cc1ccc2nc(c(n2c1)C(=O)N/N=C/c3ccccc3[O-])C**	0
**c1ccc(cc1)c2cc(c(n[nH+]2)[O-])N3CCCCC3**	0
**[H]/N=C\1/C(=C/c2cc(n(c2C)c3cc(ccc3OC)Cl)C)/C(=O)N=C4N1N=C(S4)S(=O)(=O)CC**	0
**Cn1cc[nH+]c1[C@H](c2ccccc2F)NC(=O)C3CCN(CC3)S(=O)(=O)c4ccccc4**	0
**CC(=O)Nc1ccc2c(c1)[nH]cc2[C@H](C(=O)[O-])[NH+]3CCc4c(sc(n4)c5ccc(cc5)OC)C3**	0
**C[C@H]1[C@H](Oc2ccccc2O1)C(=O)N(CCCn3cc[nH+]c3)c4nc5c(ccc(c5s4)Cl)OC**	0
**O=C(CCc1ccccc1F)N(Cc1nc2c(c(=O)[nH]1)COCC2)Cc1cccs1**	1
**CCS(=O)(=O)N1CCc2c(sc(n2)NC(=O)Cc3ccc(cc3)S(=O)(=O)C(C)C)C1**	0
**CC1CC[NH+](CC1)Cc2nnc(n2C[C@@H]3CCCO3)SCC(=O)NC(=O)Nc4ccccc4**	0
**O=C(c1ccc(F)cc1)C1CCN(CC(=O)N(Cc2nc3c(c(=O)[nH]2)COCC3)CC(F)(F)F)CC1**	1
**COc1cccc(c1Oc2cc(c(cc2N)Cl)Cl)OC**	0
**CC(C)C[C@@H]1C(=O)N[C@H](C(=O)N[C@@H](C(=O)N2CCC[C@H]2C(=O)N[C@@H](C(=O)N1)C(C)C)CO)Cc3c[nH]c4c3cccc4**	0
**C1C[C@H](OC1)COc2c(nnc(n2)Cl)Cl**	0
**CCC(=O)N([C@H]1NN=C(S1)SCC2=NC(=O)C3=C([C@H](SC3=N2)C(=O)C)C)C4CC4**	0
**C[C@H]1CCCCCN1c2c(cnc(n2)NN)Cl**	0
**Cc1c(c2nnc3c(n2n1)ccn(c3=O)NC(=O)c4ccc(cc4)Cl)c5ccc(cc5)Cl**	0
**Cc1cc(n2c(n1)nc(n2)C(=O)Nc3ccc(cc3)S(=O)(=O)N4C[C@@H](C[C@@H](C4)C)C)C**	0
**C[C@@]12CC[C@@H](C(=C)C1)[C@](C2)(O)[S@@](=NC)(=O)c3ccccc3**	0
**O=C(CCC1CCNCC1)Nc1nnc2n1CCS2**	1
**c1cc(c(c(c1[N+](=O)[O-])N[C@H]2CC[C@H](C2)C(=O)[O-])F)F**	0
**CNc1c2ccsc2nc(n1)[C@H]3CCCc4c3cccc4**	0
**c1ccc2c(c1)C[C@H](CO2)C(=O)Nc3c[nH]c4c3cccc4**	0
**c1cc(c(cc1NC(=O)COC(=O)c2cc(ccc2Cl)S(=O)(=O)N3CCOCC3)Cl)F**	0
**CC(=O)Nc1ccc(cc1)n2ccc3c(c2=O)cc4c(n3)ccn(c4=O)Cc5ccc[nH+]c5**	0
**Cc1cc(n2c(n1)ncn2)[C@@H]([C@H](C)O)C(C)C**	0
**C#CCN1c2ccccc2[C@@]3(C1=O)CC(=Nc4c3cnn4Cc5ccccc5Cl)O**	0
**CC1(Cc2c(c(nc(c2C#N)N3CCN(CC3)C(=O)C(=O)NCc4ccccc4)c5ccco5)CO1)C**	0
**CN(C)CCOc1cc(F)c2c(c1)N(C)C(=O)C21CCN(c2nc3c(c(=O)[nH]2)CCCN3C)CC1**	1

**Cc1ccc(cc1)S(=O)(=O)Oc2ccc(c(c2)OS(=O)(=O)c3ccc(cc3)C)[C@@]4(CC(NC(=S)N4)(C)C)C**	0
**c1cc(ccc1C(=O)NCC2CCN(CC2)C(=O)c3cnccn3)S(=O)(=O)N4CCCCC4**	0
**COc1ccc(N2CCN(c3nc4c(c(=O)[nH]3)COCC4)CC2)cc1**	1
**Cc1c(ccc(n1)N)c2cn(nc2C)C(C)C**	0
**Cc1cccc(c1C)OC[C@H](C[NH+](Cc2cc(c(c(c2)OC)OC)OC)[C@@H]3CCS(=O)(=O)C3)O**	0
**c1c2c(c(c(c1F)F)F)nc3c(c2[O-])CCCC3**	0
**Cc1cccn2c1nc(c(c2=O)/C=C\3/C(=O)N(C(=S)S3)Cc4ccc5c(c4)OCO5)NCCCOC**	0
**Cc1cc2c(=O)[nH]c(-c3ccc(C(C)(C)O)cc3)cn2c1**	1
**C[C@H]1CN[C@H](CN1c2c(ccc3c2nsn3)Cl)C**	0
**C1C[C@@H](C[C@H](C1)NC(=O)N2CCC3(CC2)OCCO3)C(F)(F)F**	0
**Cc1cc([nH+]c(c1C(=O)[O-])N2C[C@H](CC[C@@H]2C)C)C**	0
**O=C(c1ccc(F)cc1)C1CCN(CC(=O)N(Cc2nc3c(c(=O)[nH]2)COCC3)Cc2nc3ccccc3s2)CC1**	1
**c1cc(oc1)[C@H]2[C@@H]3C[C@H]([C@H]2[N+](=O)[O-])C=C3**	0
**CCCNc1cc(cc(n1)Cl)C(=O)NC2(CCCC2)C**	0
**c1ccn2cc([nH+]c2c1)CNC(=O)c3ccc(cc3)NC(=O)C[C@H]4C(=O)N=C(S4)N5CCCC5**	0
**O=C(N[C@H]1C[C@H](c2nnc(-c3ccncn3)n2-c2ccccc2Cl)C1)c1ccnc2cccnc12**	1
**C[C@@H](c1ccc(cc1)N2CCCC(CC2)(C)C)O**	0
**C[C@@]1([C@@]2(O1)CCCC(C2)(C)C)C(=O)OC**	0
**C[C@@H]1C[C@H]2CCCC[C@@H]2N1C(=O)Nc3cnn(c3)C[C@@H]4CCCO4**	0
**c1ccc(cc1)c2ccc(cc2)C(=O)NCCS(=O)(=O)N3CCN(CC3)C(=O)c4ccco4**	0
**c1cc(cc(c1)Cl)[C@H]2C(=C(C(=O)N2c3nnc(s3)SCc4ccc(cc4)Cl)O)C(=O)c5ccc6c(c5)OCCO6**	0
**c1cc(ncc1c2c[nH]c3c2cc(cn3)Br)Cl**	0
**CC(C)(C)NC(=O)[C@H](c1cccs1)N(c2ccc[nH+]c2)C(=O)C(=O)NCc3ccc4c(c3)OCO4**	0
**CCN1c2ccc(cc2C=CC1=C3C(=O)NC(=S)S3)C**	0
**Cc1ccc(cc1C)NC(=O)c2c(=O)c3cc(ccc3n(n2)C)Cl**	0
**CC(C)CSC1=[NH+]SC2=NC(=O)/C(=C\c3ccc(c(c3)OC)OCC=C)/C(=[NH2+])N12**	0
**CCOC(=O)[C@H]1CCCCN1C(=O)c2cc(cc(c2)n3cnnn3)c4cccc5c4scc5**	0
**c1cc(sc1S(=O)(=O)N2CCN(CC2)C(=O)CCCN3C(=O)C4(CCCC4)NC3=O)Cl**	0
**C[C@@H]1CCCCN1C(=O)Cn2cc(c3c2cccc3)/C=C/4\C(=O)NC(=O)N(C4=O)Cc5ccco5**	0
**c1cc(nc(c1)Cl)N/N=C/c2c[nH]c3c2cc(cc3)Br**	0
**CC[C@H](C)NC(=O)c1ccc(nc1)Oc2ccc(cc2)[N-]S(=O)(=O)c3cc(ccc3C)C**	0
**O=c1[nH]c(N2CCC3(CC2)CNc2cccc(Cl)c23)nc2c1CCCC2**	1
**CCn1c2ccc(cc2[nH+]c1CNc3ccc(c(c3)C)N4CCCC4=O)S(=O)(=O)N5CCCCC5**	0

**C[C@H]1CCCCC[C@@H]1N2CCCN3CCC[C@@H]3C2**	0
**C1CCC(CC1)c2nc([nH]n2)[C@H]3CC[C@@H]4CCCC[C@@H]4N3**	0
**C[C@H](/C(=N/c1cccc2c1non2)/[O-])N3C(=O)/C(=C/c4ccccc4)/SC3=S**	0
**O=c1[nH]c(-c2ccc(O)cc2)nc2ccccc12**	1
**c1c(cc(c(c1F)F)F)[N-]S(=O)(=O)c2c[nH+]c[nH]2**	0
**COc1ccc(cc1OC)[C@@H]2c3c4ccccc4ccc3Oc5c2c(=[NH2+])n(cn5)CCCN6CCOCC6**	0
**Cc1c(sc2c1c(ncn2)Nc3ccc4c(c3)OCCCO4)C**	0
**c1ccc2c(c1)ccc3c2cc(c(=O)o3)c4csc(n4)/C(=C/Nc5cccc(c5)[N+](=O)[O-])/C#N**	0
**Cn1cccc1C(=O)NC(=O)CSc2nnc(n2Cc3ccccc3)c4ccncc4**	0
**C[C@@H]1CCC[C@H](C1)Nc2ccc(cc2)n3nccn3**	0
**CC(C)c1nc2n(n1)C[C@H](CC2)[NH2+]Cc3cccc4c3OCO4**	0
**COc1ccc(C(=O)C2CCN(CC(=O)NCc3nc(C)c(C)c(=O)[nH]3)CC2)cc1**	1
**CC(C)(C)c1cc(c(nn1)Cl)C(F)(F)F**	0
**Cc1nc(on1)CN[C@H]2CCO[C@H](C2)C**	0
**Cc1ccc(o1)[C@@H](C(=O)NCc2ccccc2)N(c3ccccc3Br)C(=O)c4csnn4**	0
**C[C@H]1CCC[NH+](C1)[C@@H]2CCCCC[C@@H]2C(=O)[O-]**	0
**Cc1ccsc1CNc2c(cnc(n2)NN)C**	0
**O=C(CCc1ccccc1)Nc1nnc2n1CCS2**	1
**CC(C)[C@@H](CO)Nc1c2cc(ccc2ncn1)N**	0
**C[C@H](c1ccc(F)cc1)N(Cc1nc2c(c(=O)[nH]1)COCC2)C(=O)CN1CCC(C(=O)c2ccc(F)cc2)CC1**	1
**Cc1ccc(c(c1)Br)NC(=O)N2CCC[C@@H]([C@@H]2CO)C**	0
**CCN1CCN(CC1)c2ccc(cc2Cl)[N-]S(=O)(=O)c3c([nH+]c4n(c3=O)ccs4)C**	0
**C[C@@H](c1ccc(cc1O)O)[NH+]2CCC[C@@H](C2)N**	0
**CC1(c2c(c3ccccc3[nH]2)C[C@@H]4N1C(=O)CN(C4=O)/N=C/c5ccc(cc5OC)OC)C**	0
**C[C@H]1CN(C[C@@H](O1)C)Cc2csc(n2)NC(=O)c3cnn(c3)c4ccc(cc4)Cl**	0
**Cc\1ccc[nH]/c1=N\S(=O)(=O)c2ccc3c(c2)CCO3**	0
**C[NH+](C)CCCn1c(nnc1S)c2cc3n(n2)[C@@H](C[C@H](N3)c4ccc(cc4)Cl)C(F)(F)F**	0
**Cc1ccc(c(c1)c2c3c([nH]n2)C(=O)N([C@H]3c4ccc(cc4)F)Cc5cccnc5)O**	0
**N#Cc1c(Cn2c(=O)n(C[C@H]3CC[C@H](C(=O)Nc4ccncc4)CC3)c(=O)c3c(F)cccc32)cccc1C(F)(F)F**	1
**Cl.O=c1[nH]c(-c2ccc(C3CCNCC3)cc2)nc2c(F)cc(F)cc12**	1
**Cc1c(c(on1)C)c2ccc(s2)C(=O)NNC(=O)c3ccc(cc3)Br**	0
**COc1cc(cc(c1OC)OC)[C@H]2C(=C(C(=O)N2CCCn3cc[nH+]c3)O)C(=O)c4cccs4**	0
**c1ccc(cc1)C(=O)C2=C(C(=O)N([C@@H]2c3ccc(cc3)Br)c4cccc(c4)[N+](=O)[O-])[O-]**	0
**CCC(CC)(C(=O)NC(=O)NC(=O)C)Br**	0
**c1ccc2c(c1)nc(s2)CCCC(=O)OCC(=O)N3CCN(CC3)S(=O)(=O)c4ccc(cc4)Cl**	0

**COc1ccccc1C(=O)NNC(=O)CCN2C(=O)/C(=C\c3ccccc3)/SC2=S**	0
**COc1cc(cc(c1OC)OC)C(=O)N(Cc2ccc(cc2)S(=O)(=O)C)c3cccc[nH+]3**	0
**CCn1c(c(c(n1)C)[C@H](c2cc3c(o2)ccc(n3)C)[NH3+])C**	0
**c1ccc(c(c1)Oc2ccc(cc2)S(=O)(=O)N3CCc4cnc(nc4C3)N)Cl**	0
**Cc1ccc(cc1[N+](=O)[O-])S(=O)(=O)N/N=C(/C)\c2ccc3c(c2)Nc4ccccc4S3**	0
**c1ccnc(c1)C(=O)NCC(=O)N/N=C/c2cc(ccc2OC(=O)/C=C/c3ccco3)Br**	0
**C[C@H](C(=O)OC)n1c2ccc(cc2[nH+]c1SCC(=O)[O-])I**	0
**Cc1cc(cc(c1)[C@H]2CN[C@H]([C@@H](O2)C)C)C**	0
**Cc1cc(n(n1)CCN(c2nc3c(cccc3s2)C(C)C)C(=O)c4cccc(c4)[N+](=O)[O-])C**	0
**Cc1ccc(cc1)S(=O)(=O)N2CCC[C@@H](C2)C(=O)Nc3nnc(o3)Cc4ccc(cc4)S(=O)(=O)C**	0
**Cc1c(non1)COc2c(cc(cc2Cl)Cl)C(=O)[O-]**	0
**CC(C)(C)OC(=O)N1CCC[C@@H](C1)CNC(=O)c2ccc(cc2I)[N+](=O)[O-]**	0
**CC(C)[C@H](c1nnc2n1CCN(CC2)Cc3ccccc3Cl)NC(=O)c4ccoc4**	0
**Cc1cccc2c(=O)[nH]c(-c3ccc(Br)cc3)nc12**	1
**Cn1c(=O)c(c(n(c1=O)Cc2ccccc2)N)C(=O)COC(=O)c3cc(cnc3)Br**	0
**Cc1cc(cc(c1)N2C[C@H](CC2=O)C(=O)Nc3nc4ccc(cc4s3)S(=O)(=O)C)C**	0
**c1cc(oc1C(=O)[O-])[NH+]2CCC3(CCCCC3)CC2**	0
**CCOC(=O)c1c(c2cc(ccc2[nH]1)F)NC(=O)[C@H](C)N3CCN(CC3)Cc4ccc5c(c4)OCO5**	0
**O=C(N[C@H]1C[C@H](c2nnc(-c3ccccn3)n2-c2ccccc2F)C1)c1ccnc2cccnc12**	1
**O=C(N[C@H]1C[C@H](c2nnc(-c3nccs3)n2-c2ccccc2F)C1)c1ccnc2cccnc12**	1
**CC1(C(C1(C)C)C(=O)N2CCCC[C@@H]2[C@H]3CCC[C@@H]3O)C**	0
**CN(C)c1ccc(-c2nc3c(c(=O)[nH]2)COCC3)cc1**	1
**c1cc(cc(c1)S(=O)(=O)N2CCCC2)NC(=O)CN3CC[NH+](CC3)Cc4ccc(cc4)C#N**	0
**Cc1c(nn(c1C)c2ccc(nn2)Nc3ccc(cc3)NC(=O)c4c(nc(s4)C)C)C**	0
**CN1CCN(CC1)CCNC(=O)CCc2nnc3n2nc(cc3)NCc4ccccc4Cl**	0
**CCCn1c2ccc(cc2[nH+]c1CCC(=O)Nn3c[nH+]c4c3cccc4)S(=O)(=O)N5CCCC5**	0
**CCOc1ccc(cc1OCC)[C@H]2C(=C(C(=O)N2CCc3ccccc3)O)C(=O)c4c(nc(s4)C)C**	0
**c1ccc2c(c1)C[C@H]([C@@H]3N2CCN(C3)c4ncccn4)C(=O)NCCC[NH+]5CCOCC5**	0
**Cc1c(sc2c1c(=O)[nH]c(n2)[C@H](C)OC(=O)c3ccc(c(c3)S(=O)(=O)NC)OC)C**	0
**Cc1nn(c(=O)c2n1c3ccoc3c2)CCCC(=O)NCc4ccc(cc4)Cl**	0
**Cc1ccc(o1)[C@@H](C)N2CC(=O)N[C@H](C2=O)C(C)(C)C**	0
**CCCc1cnc2c(c1NOCC(=O)Nc3c(cc(cc3C)C)C)c(=O)n(c(=O)n2C)C**	0
**c1cc2cn[nH]c2cc1C(=O)Nc3c(cc(cc3F)F)F**	0
**Cc1ccc(-c2nc3c(C)cccc3c(=O)[nH]2)cc1**	1
**CN1[C@H]([C@H](c2cc(c(cc2C1=O)OC)OC)C(=O)Nc3cc(ccc3O**	0

C)OC)c4ccc[nH+]c4	
C[C@H](C(=O)NC(=O)NC(C)(C)C)Sc1ccc(cn1)S(=O)(=O)N2CCCCC2	0
c1cc(ccc1c2nc(c(o2)SCC(=O)NC[C@@H]3CCCO3)S(=O)(=O)c4ccc(cc4)Br)F	0
Cc1ccc(c(c1C)S(=O)(=O)Nc2cccc(n2)C)N	0
Cc1ccc(c(c1)C)/C=N\NC(=O)c2cccc(c2)S(=O)(=O)[N-]c3ccc(cc3)Cl	0
COc1ccc(-c2nc3c(c(=O)[nH]2)COCC3)cc1	1
Cc1cc(sc1Br)c2[nH]nc(n2)C3CCCC3	0
c1cc(ccc1[C@@H]2CC(=NN2C3=NC(=O)[C@H](S3)CC(=O)[O-])c4ccc(cc4)F)Cl	0
CC(C)NC(=O)[C@@H]1COC2(N1C(=O)c3ccc(cc3)F)CCN(CC2)C(=O)C(C)(C)C	0
C[C@H](c1ccccc1)NC(=O)CSc2nnc(n2C)CN(c3ccccc3)S(=O)(=O)C	0
N#Cc1ccc2c(c1)[nH]c(=O)n2[C@H]1C[C@H](c2nnc(-c3ccncn3)n2-c2ccccc2F)C1	1
CN(Cc1ccccc1Br)c2ccc(c(c2)N)C#N	0
C[C@H]1c2ccsc2CC[NH+]1CC(=O)N3C[C@@H](Oc4c3cccc4)C(=O)N	0
CCOC(=O)C1=c2n(c(=O)/c(=C/c3ccc(cc3Cl)Cl)/s2)C(=C([C@@H]1c4ccc(cc4Cl)Cl)C(=O)OCC)N	0
CC[C@H]1[C@@H](c2c(ccc(c2O1)C)C(C)C)O	0
Cn1c2c(c(=O)n(c1=O)C)n(cn2)CC(=O)Nc3nnc(s3)SCc4ccccc4F	0
Cn1c2ccc(cc2nc1CN3CCN(CC3)S(=O)(=O)c4ccc(o4)C(=O)N)Cl	0
C[C@H]1CC[C@H]([C@@](C1)(c2nccn2C)O)C(C)C	0
c1ccc(c(c1)NC(=O)c2c(c3c(o2)ccc[nH+]3)NC(=O)c4ccc(c(c4)F)F)F	0
CC1=C[C@H](N=N1)NC(=O)CN2[C@H]3C=CS[C@@H]3C(=O)N(C2=O)CCC(=O)NCCc4ccc(cc4)Cl	0
Cc1c(sc(n1)[S-])CC(=O)NC2CCCCCCC2	0
O=C(CCc1nc2ccccc2c(=O)[nH]1)Nc1ccc(C(=O)Nc2cccc3cccnc23)cc1	1
Cc1cc(ccc1Br)n2c(c(nn2)N)C(C)C	0
COc1ccc(C(=O)C2CCN(C3CCN(Cc4nc5ccsc5c(=O)[nH]4)C3=O)CC2)cc1	1
c1ccc(c(c1)/C=C(/C#N)\c2[nH]c3ccccc3n2)N4CCCC4	0
Cc1ccccc1NC(=O)[C@H]2[C@@H]([C@@H]([C@@](CC2=O)(C)O)C(=O)Nc3ccccc3C)c4ccccc4OC	0
Cc1ccccc1[C@H]2C[C@@H]([NH+]=C3N2NC=N3)c4ccccc4OC(F)F	0
c1ccc2=[NH+]CC(=c2c1)/C=N/NC(=O)COc3ccc(cc3Br)[N+](=O)[O-]	0
Cc1c(c(=O)n(n1C)c2ccccc2)N3[C@@H](SCC3=O)c4ccccc4O	0
CC1=C([C@H](n2c(=O)/c(=C/c3ccc(c(c3)OC)OC(=O)C)/sc2=N1)c4ccc(cc4)Cl)C(=O)OC(C)C	0
Cc1ccnc2n1nc(n2)C(=O)Nc3ccc(cc3C(F)(F)F)NC(=O)c4ccc(cc4)F	0
O=C(CN1CCC(C(=O)c2ccc(OCCC3CC3)cc2)CC1)NCc1nc2c(c(=O)[nH]1)COCC2	1
CC[NH+]1CCN(CC1)c2nc3c(n2C[C@H](COc4ccc(c(c4)C)C)O)c(=O)n(c(=O)n3C)C	0

**CCc1c(c(nc(n1)c2cnn(c2)c3ccccc3)N)Br**	0
**Cc1cnc(nc1N[C@@H]2CCCCC[C@H]2C)Cl**	0
**Cc1cc(sc1C[NH3+])S(=O)(=O)N2C[C@@H](CC[C@@H]2C)C**	0
**CC1(C[C@@H]2CC(=C)CN1N2)C**	0
**Cc1cc(c(c(c1)C)C(=O)NC2(CCCCC2)C#N)C**	0
**c1ccc2c(c1)OC[C@@H](O2)C/[NH+]=C(/c3cc(ccc3F)S(=O)(=O)N4CCOCC4)\O**	0
**COc1ccc(C(=O)C2CCN(CC(=O)NCc3nc4c(c(=O)[nH]3)CCCC4)CC2)cc1**	1
**Cc1c(c(sc1C(=O)OC)NC(=O)c2cc3n(n2)[C@H](C[C@@H](N3)c4ccc(c(c4)Cl)Cl)C(F)(F)F)C(=O)OC**	0
**COc1ccc(C(=O)C2CCN([C@@H]3CCN(Cc4nc5ccccc(=O)c5[nH]4)C3=O)CC2)cc1**	1
**c1cc(ccc1c2cc(=O)n(cn2)CCNS(=O)(=O)c3ccc(cc3)F)F**	0
**Cc1c2cc3c(cc2oc(=O)c1CC(=O)N[C@@H]4CS(=O)(=O)C[C@H]4O)OC5(CCCCC5)CC3**	0
**CC1(C)OC(=O)N(c2ccc(-c3cc(-c4ncccn4)c[nH]c3=O)cc2)[C@H]1c1ccccc1**	1
**c1c(cc(cc1Cl)Cl)NC(=O)C2(CCCCCC2)C#N**	0
**O=c1[nH]c(N2CCC3(CC2)CN(CCO)c2cccc(Cl)c23)nc2c1CCCC2**	1
**O=C(CN1CCC(C(=O)c2ccc(F)cc2)CC1)NCc1nc2c(c(=O)[nH]1)COCC2**	1
**c1cc(c(cc1Cl)N)C(=O)N2CCO[C@H]3[C@@H]2CCCC3**	0
**Cc1c(c(sn1)N[C@@H]2CCCCC2(C)C)C#N**	0
**Cc1ccc(cc1C)[N-]S(=O)(=O)c2ccc(c(c2)C(=O)N3CCN(CC3)Cc4ccccc4)Cl**	0
**c1ccn2c(c1)c(nc2c3ccc(cc3)Br)CO**	0
**Cc1ccc(cc1)S(=O)(=O)NC2CC(N(C(C2)(C)C)O)(C)C**	0
**COc1cc(OC)c2sc3nncn3c2c1**	1
**CC[NH+](CC)CCN1[C@@H](C(=C(C1=O)O)C(=O)c2c(nc(s2)C)C)c3ccc(c(c3)OCC)OCC**	0
**C[C@@H]1C[C@@H](CC(C1)(C)C)NC(=O)/C=C\2/C(=Nc3ccccc3S2)[O-]**	0
**C[C@@H]1CN(CCCO1)c2cc(c(cc2F)[NH3+])C(=O)[O-]**	0
**O=C(N[C@H]1C[C@H](c2nnc(-c3cnccn3)n2-c2ccccc2F)C1)c1ccccn1**	1
**CSc1ccc(cc1)[C@H]2c3ccc(cc3OC(=C2C#N)N)OC(=O)c4ccccc4[N+](=O)[O-]**	0
**CC1=C([C@@H](NC(=S)N1c2ccc3c(c2)OCCO3)c4ccc(cc4)F)C(=O)N(C)C**	0
**C1Cc2c(nc(s2)N3CCC[C@@H](C3)C(F)(F)F)[C@@H](C1)O**	0
**c1c(cnc(c1Cl)N2CCC(CC2)C(=O)Nc3nn4cnnc4s3)C(F)(F)F**	0
**COc1ccc(C2(CNC(=O)c3ccc(NC(=O)c4ccco4)cc3)CCOCC2)cc1**	1
**Cc1ccc(cc1Cl)n2c(c(nn2)/C(=N/C(C)C)/O)C**	0
**CCOc1ccc(cc1CN2C[C@H]3C[C@@H](C2)c4cccc(=O)n4C3)/C=N/NC(=O)c5ccoc5C**	0
**COc1cccc2c(=O)[nH]c(-c3ccc(Cl)cc3)nc12**	1
**Cc1c(sc2c1c(=O)n(c(=S)[nH]2)[C@@H]3CC[C@@H](C[C@@H]3C)C)C**	0
**CC1(Cc2c(c(nc(c2C#N)SCc3ccc(o3)C(=O)OC)N4CC[NH2+]CC4)CO1)C**	0
**Cc1c(nnc(c1C#N)N[C@@H](C)c2ccccc2)C**	0

**c1cc(oc1)CNC(=O)C2=C(C3=[NH+][C@@H](C[C@H](N3N2)C(F)(F)F)c4ccc(cc4)F)Cl**	0
**C1CCC2(CC1)CN(C2)[C@@H]3CCC[C@@H]3C#N**	0
**CC1=C[C@@H]2[C@@](CC[C@@H]3[C@@]2(CC[C@@H](C3(C)C)OC(=O)C)C=O)([C@@]4([C@@]1(C(=O)[C@@](C4=O)(C)O)C)C(=O)OC)C**	0
**c1ccc2c(c1)CCCN2CCNC(=O)c3c4c([nH]n3)-c5ccccc5OC4**	0
**O=C(CN1CCC(c2nnc(-c3ccc(F)cc3)o2)CC1)NCc1nc2c(c(=O)[nH]1)COCC2**	1
**CC[C@H]1CC[C@@H](C1)c2nc(c(c(n2)N)Br)C3CC3**	0
**CCC(C)(C)C1CCC2(CC1)C(=O)N(C(=O)N2)CC(=O)N3C[C@@H](C[C@@H](C3)C)C**	0
**CC1=C(C[C@H](CC1)C(=O)N2CCc3c([nH+]c(nc3NC)C4CCN(CC4)C(=O)C)C2)C**	0
**Cc1ccc(c(c1)C)S(=O)(=O)N2CCN(CC2)Cc3[nH]c4ccsc4c(=O)n3**	0
**Cc1ccccc1OC[C@@H](Cn2c3c([nH+]c2N/N=C/4\c5cc(ccc5NC4=O)Br)n(c(=O)n(c3=O)C)C)O**	0
**CCOc1ccc(-c2nnc([C@H]3C[C@H](NC(=O)c4ccccn4)C3)n2-c2ccccc2)nc1**	1
**c1cc(sc1/C=N/O)N2CCCCC2**	0
**c1cc(ccc1CN[C@H]2CCc3c(sc4c3c(=O)n(cn4)CCn5ccnc5)C2)F**	0
**[H]/N=C/1\C(C2=CC[NH+](C[C@H]2[C@@H](C1(C#N)C#N)CCc3ccccc3)C)C#N**	0
**c1ccc2c(c1)c(cc(n2)c3ccco3)C(=O)Nc4ccc(cc4)S(=O)(=O)N5CCOCC5**	0
**CCOC(=O)C1=C(OC(=C([C@H]1c2cccnc2)C#N)N)CSc3c(c(cc(n3)c4cccs4)c5ccc(cc5)Cl)C#N**	0
**Cc1ccc(cc1Cl)NC2CCC3(CC2)OCCO3**	0
**CCc1c(nnn1c2ccc(cc2)C(C)C)C(=O)[O-]**	0
**c1cc(ccc1N)Oc2c(cncn2)I**	0
**c1ccc(cc1)N2CCN(CC2)[C@@H](CNC(=O)C(=O)NCCCn3ccnc3)c4cccnc4**	0
**Cc1cc(c(n1C2CC2)C)C(=O)O[C@@H](C)C(=O)NC34CC5CC(C3)CC(C5)C4**	0
**Cc1c(sc2c1c(=O)n(c(n2)S[C@@H](C)C(=O)NC(=O)NC)C3CCCC3)C**	0
**CC1CCC(CC1)NC(=O)c2cc(ccc2Cl)[N+](=O)[O-]**	0
**CC(=O)c1ccc(cc1)S(=O)(=O)[N-]c2ccc(c(c2)C(=O)[O-])N3CCC(CC3)[NH+]4CCCCC4**	0
**C[C@@H](C(=O)Nc1c(c2c(s1)CCCCC2)C#N)N3CCN(CC3)CC(=O)NC(C)C**	0
**Cc1ccccc1N2CC[NH+](CC2)[C@@H]3CCCN(C3)C(=O)c4cc(n[nH]4)c5ccsc5**	0
**C[C@H]1CCCC[C@H]1Nc2c3ccsc3ncn2**	0
**C[C@@H](C(=O)NC(C)(C)C)N1CC(C1)C(=O)[O-]**	0
**Cn1c2c(c(=O)n(c1=O)C)n(c(n2)N/N=C\c3ccc(cc3)Br)Cc4ccc(cc4)Cl**	0
**Cc1ccc(cc1)C[n+]2c(c(cc3c2nc4ccc(cn4c3=O)C)S(=O)(=O)c5ccc(cc5)Cl)N**	0
**c1cc(ccc1C2=NN=C[C@@H]2CN3CC[C@H]([C@@H](C3)[O-])[NH+]4CCCCCC4)Cl**	0
**C[C@@H](C(=O)Nc1cccc(c1)S(=O)(=O)N(C)C)OC(=O)[C@]23**	0

CCC(=O)N2c4ccccc4S3	
c1cc2=C(C=c3ccc(=C(C=c1cc2)C#N)cc3)C#N	0
Cc1cccc(c1)OCC(=O)c2ccc3c(c2)N(C(=O)CO3)Cc4ccc(o4)C(=O)N5CC[NH+](CC5)C	0
CCCCNC(=O)[C@@H]1Cc2cc(ccc2N1C(=O)C)S(=O)(=O)N3CCCC3	0
CCOC(=O)c1ccc(cc1)n2c(cc(c2C)/C=C\3/C(=O)NC(=O)N(C3=O)c4ccc(cc4)F)C	0
c1cc(cc(c1)[N+](=O)[O-])c2ccc(o2)/C=C\3/C(=NC(=S)N(C3=O)c4ccc(cc4)Cl)[O-]	0
Cc1c(c(n([nH+]1)C)O[C@H]2CC[C@@H]([C@@H](C2)C)C)C(=O)[O-]	0
N#Cc1ccccc1Cn1c(=O)n(C[C@H]2CC[C@H](C(=O)Nc3ccncc3)CC2)c(=O)c2ccncc21	1
Cn1c2c(c(n3c2[C@H](OCC3)c4c5ccccc5ccc4O)c6ccc(cc6)OC)c(=O)n(c1=O)C	0
Cc1ccccc1S(=O)(=O)Nc2ccc(c(c2)C(=O)N(C)C)N3CC[NH2+]CC3	0
Cc1ccc(c(c1)Br)Nc2nc(nc(n2)N3CCOCC3)N/N=C/c4cc(ccc4O)Br	0
Cn1cc(c(=O)n(c1=O)C)S(=O)(=O)NCc2ccccc2c3ccc(cc3)CN4CCCC4=O	0
c1ccc(cc1)n2c3nc(=O)c(nn3c(n2)C(=O)c4cccs4)Cc5ccc(cc5)Cl	0
CC(C)C(=O)N/N=C/1\c2cc(ccc2N(C1=O)C)Br	0
c1cc(c2c(c1)oc(n2)CSc3ccc(cn3)Br)N	0
Cc1c(sc(c1[C@@H](C)NC(=O)Nc2cc(c(c(c2)OC)OC)OC)n3cccc3)C	0
Cc1ccc(cc1)c2c(sc(n2)C)CC(=O)NCCC[NH+]3CCN(CC3)c4ncccn4	0
CCOC(=O)c1c(c2c3c(c([nH+]c2s1)N4CCOCC4)CSC(C3)(C)C)N	0
Cc1ccc(cc1)C2=NN([C@@H](C2)c3ccco3)C(=O)CN4C(=O)[C@@H](NC4=O)CC5=c6ccccc6=[NH+]C5	0
CC(=O)N1CCc2c1cc(cc2)NC(=O)c3ccc(cc3)S(=O)(=O)N4CCCCC4	0
Cc1ccc(cc1)[C@H](c2cccs2)NC(=O)Cn3c(=O)n(c(=O)n(c3=O)CC=C)CC=C	0
c1cc(nc2c1cc(cc2)N)Oc3cc(ccc3F)F	0
CC1=C[C@@H](N=N1)[C@H]2N=N[C@@H](N2/N=C/c3cc(cc(c3[O-])Br)Br)S	0
Cn1c2c(cn1)c(ncn2)Sc3ccc(cc3)O	0
c1cc[nH+]c(c1)N2CCN(CC2)C(=O)c3cccnc3SCC(=O)Nc4ccc(cc4)Br	0
CC1(CC[C@H]2[C@H](C1)S(=O)(=O)CCCN2)C	0
CN1C(=O)/C(=C/c2ccc(o2)c3ccc(cc3Cl)Cl)/NC1=O	0
c1ccc2c(c1)C(=NS2(=O)=O)NCCC(=O)N3CCN(CC3)Cc4ccc5c(c4)OCO5	0
COc1ccc(C(=O)C2CCN(C3CCN(Cc4ncc(C)c(=O)[nH]4)C3=O)CC2)cc1	1
c1cc(cc(c1)S(=O)(=O)Oc2ccc3c(c2)occ(c3=O)Oc4ccc(cc4)F)C(=O)[O-]	0
Cc1ccc(c(c1)S(=O)(=O)NCC(=O)N2CC(C2)S(=O)(=O)c3ccc(cc3)Cl)C	0
C[C@]12CCCN1CC1=NNC(=O)c3cc(F)cc4[nH]c2c1c34	1

**Cc1nc(nn1c2ccc(cc2)F)C(=O)Nc3ccccc3C(=O)NCc4ccco4**	0
**C[C@@H]1CCC[C@@](C1)(C#N)[C@@]2(CCCOCC2)O**	0
**Cc1c(c(n(n1)Cc2ccccc2Cl)C)NC(=O)c3cc(on3)c4ccc(cc4)F**	0
**Cc1cc(n2c(n1)nnn2)Nc3ccc(cc3F)Br**	0
**Cc1cc(ccc1N2CC[NH+](CC2)C)NC(=O)C(=O)c3c(nn(c3C)c4cccc(c4)C(F)(F)F)C**	0
**CN1CCCc2c1nc(N1CCC3(CC1)C(=O)N(C)c1cc(F)cc(F)c13)[nH]c2=O**	1
**c1ccc(cc1)/N=C/2\C(=C\c3ccc(cc3)O)\SC(=O)N2**	0
**Cc1cc(cc(n1)[C@@H]2CCN(C2)C(=O)Cc3ccc4c(c3)OCCCO4)c5ccc(nc5)N**	0
**c1ccc2c(c1)ncc(n2)Oc3ccc(cc3N)Br**	0
**C/C(=N/N=C/1\NC(=O)C[C@H](S1)C(=O)Nc2cccc(c2)Cl)/c3cccs3**	0
**O=C(c1ccc(OCc2ccccc2)cc1)C1CCN(CC(=O)N(Cc2nc3c(c(=O)[nH]2)COCC3)CC2CC2)CC1**	1
**Cc1c(c([nH]c1C(=O)OC)C)C(=O)[C@@H]2[C@H](N(C(=O)C2=O)CCC[NH+](C)C)c3cccc(c3)OC**	0
**Cc1ccc2c(=O)[nH]c(-c3ccc(C4(O)CC4)cc3)cn12**	1
**Cc1ccc(-c2noc(CSc3nnc(C)n3-c3ccc(C)c(C)c3)n2)cc1**	1
**c1ccc(cc1)CC2CCN(CC2)[C@@H]3[C@H]([C@@H]([C@H]4CO[C@@H]3O4)NC(=O)Nc5cccc(c5)C(F)(F)F)O**	0
**[H]/N=C/1\[C@H](C2=CCN(C[C@@H]2[C@H](C1(C#N)C#N)c3ccc(cc3)CC)C(=O)OCC)C#N**	0
**O=c1[nH]c(N2CCC3(CC2)CNc2cc(Cl)cc(Cl)c23)nc2c1CCCC2**	1
**Cc1ccc(c(c1)Br)Nc2cc(ncn2)Cl**	0
**N#Cc1c(Cn2c(=O)n(C[C@H]3CC[C@H](C(=O)Nc4ccncc4)CC3)c(=O)c3c(Cl)cccc32)cccc1C(F)(F)F**	1
**CC(C)(C)c1ccc(-c2cn3cncc3c(=O)[nH]2)cc1**	1
**C[NH+]1CCN(CC1)c2ccc(nn2)c3ccc(cc3)NS(=O)(=O)c4ccc(cc4)Br**	0
**c1cc(sc1)[C@@H]2[C@@H]3CC=C[C@@H]3c4cc(ccc4N2)S(=O)(=O)N5CCOCC5**	0
**O=C1NN=C2CCCc3[nH]c4cccc1c4c32**	1
**Cc1ccc(c2c1c(cc(n2)c3cnccn3)C(=O)[O-])C**	0
**c1c(cc(c(c1N)S(=O)(=O)N2CC[C@H](C2)O)Cl)Cl**	0
**c1cc(ncc1c2nc(no2)[C@@H]3CSCCS3)N**	0
**c1cc(ccc1c2csc(n2)NC(=O)/C=C/c3ccc(cc3Cl)Cl)[N+](=O)[O-]**	0
**C[C@H]1c2ccsc2CCN1c3ccc(c(n3)OC)N**	0
**Cc1ccc(cc1)c2coc(n2)c3cccc(c3)[N-]S(=O)(=O)c4ccc(cc4)N5CCCC5=O**	0
**CC1(CCCC[C@H]1Nc2c(cc(c[nH+]2)C(=O)[O-])Cl)C**	0
**c1cc(cc(c1)C(F)(F)F)c2cncc(n2)[C@@H]3CCCN(C3)C(=O)c4ccn[nH]4**	0
**Cc1c[nH+]c(s1)N2CCN(CC2)C(=O)c3ccc(cc3)S(=O)(=O)Nc4ccncc4**	0
**CCCCN1c2ccccc2[C@]3(C1=O)C(=C(C(=O)N3CC[NH+]4CCOCC4)O)C(=O)c5cccc(c5)S(=O)(=O)N(C)C**	0
**CS(=O)(=O)c1ccc(cn1)NC(=O)[C@H]2CCCC[C@H]2c3[nH]c4cc(ccc4n3)Cl**	0
**Cc1c(cccc1NC(=O)N(C)c2ccccc2)C(=O)[O-]**	0
**Cc1cc(c(cc1N)S(=O)(=O)N2C[C@H](S[C@@H](C2)C)C)Cl**	0

**CCCc1c2c(cc(nc2on1)c3cccnc3)/C(=N/c4c5ccsc5ccn4)/[O-]**	0
**CN1CCN(CC1)S(=O)(=O)c2ccc(nc2)SCC(=O)Nc3ccc(cc3)N4CCCC4**	0
**C[C@@H]1[C@]2(CCO1)C[C@@H](c3ccc(cc3O2)F)O**	0
**CCN1CCN(CC1)Cc2ccc(cc2)NC(=O)c3c(=O)n(c(=O)n(n3)c4ccc(cc4)C)C**	0
**CCOc1ccc(cc1OC)[C@H]2c3c(n[nH]c3C(=O)N2CCO)c4cc(ccc4O)Cl**	0
**[H]\N=C\1/C(=C\c2ccc(c(c2)OCC)OCCOc3ccccc3)/C(=O)N=C4N1C(=NS4)S(=O)(=O)C**	0
**O=C(N[C@H]1C[C@H](c2nnc(-c3ccc(OC(F)F)cn3)n2-c2ccccc2F)C1)c1ccnc2cccnc12**	1
**c1cc(c2c(c1)OCO2)CNc3nc(ns3)C4CC4**	0
**O=C(c1ccc(F)cc1)C1CCN(CC(=O)N(Cc2nc3c(c(=O)[nH]2)COCC3)Cc2ccc(F)cc2F)CC1**	1
**Cc1cc(cc(c1)[C@H](c2ccc3c(c2)C[C@H](O3)C)O)C**	0
**C[C@@H](c1ccc(cc1)OC(F)(F)F)n2c(ccn2)N**	0
**CC1(Cc2c(cnc(n2)N3CCOCC3)[C@H](C1)NC(=O)c4cc5ccccc5[nH]4)C**	0
**CCOc1ccc(-c2nnc([C@H]3C[C@H](NC(=O)c4ccnc5cccnc45)C3)n2-c2ccccc2F)nc1**	1
**c1ccc2c(c1)ncc(n2)/C=C\C(=O)NCC3CCC4(CC3)OCCO4**	0
**Cc1c(c(n[nH]1)C)S(=O)(=O)N2CCCN(CC2)C(=O)c3cc[nH]c(=O)c3**	0
**c1cc2c(cc1NC(=O)Nc3nc4c(s3)CCCC4)CCC2**	0
**Cn1ccnc1[C@H](c2ccccc2N3CCN(CC3)Cc4cccc(c4)OC)O**	0
**c1cc(cc(c1)F)Cn2c3c(cccc3Cl)nc2N**	0
**CC(C)[C@H](C(=O)N1CCN(CC1)c2cccc[nH+]2)NS(=O)(=O)c3cccc4c3nsn4**	0
**c1ccc(c(c1)n2ccc(n2)C(=O)N[C@]3(CCSC3)C#N)Cl**	0
**c1cc(cc(c1)NC(=O)c2ccc(s2)Cl)c3c[nH+]c4n3CCC4**	0
**C1CCC(CC1)[NH+]2CCN(CC2)CCCNC(=O)[C@@H]3CCCN(C3)S(=O)(=O)N4CCCCC4**	0
**CCn1c2ccc(cc2nc1SCc3cc(=O)n4cc(ccc4n3)C)S(=O)(=O)N5CCOCC5**	0
**COc1cc(-c2nnc(/C=C/c3nnc(-c4ccc(S(C)(=O)=O)cn4)n3-c3ccccc3Cl)o2)ccn1**	1
**Cc1ccc(c(c1)C)NC(=O)CSc2nnc3n2c4c(c5c(s4)COC(C5)(C)C)c(=O)n3C**	0
**CCc1cc(nc(n1)S(=O)(=O)C)C#CC**	0
**CC(C)[C@H]1CCCC[C@H]1NC(=O)c2c(cn[nH]2)Br**	0
**c1ccc(cc1)n2nc3ccc(c(c3n2)Br)N**	0
**c1ccc(c(c1)N[C@H]([C@H]2CCC=CC2)C(=O)[O-])Br**	0
**Cc1ccc(nc1)[C@]2(CC[C@H]3[C@@H]2C[NH+](C3)Cc4ccc5c(c4)C[C@@H](O5)C)O**	0
**CC(C)(C)c1c(nnn1C[C@H]2C[C@H]3CC[C@@H]2C3)C(=O)[O-]**	0
**c1cc(ccc1[N+](=O)[O-])OC(=O)c2cc(c(cc2Cl)Cl)S(=O)(=O)N3CCCCC3**	0
**CCCn1c(c(c(=O)n(c1=O)C)C(=O)CSc2nnc(n2c3ccc(cc3)F)[C@H](C)[NH+](C)C)N**	0

Cc1cc(=O)[nH]c2cc(-c3ccc(C(=O)O)cc3Cl)ccc12	1
[H]/N=C\1/C(=C([C@@H](S1)C)C)c2[nH]c3c(n2)ccc(n3)NC	0
c1cc2n(c1)CCN([C@@H]2c3ccc(cc3)Cl)C(=O)N[C@@H]4CC(=O)N(C4)c5ccc6c(c5)OCCO6	0
C[C@H]1CC[NH+]1c2cc(c(cc2C(=O)[O-])F)F	0
Cc1c(nnc2c1c(c(s2)C(=O)N(C)C[C@H](C)C#N)N)C	0
O=c1[nH]c(CN(Cc2cccs2)S(=O)(=O)CCc2ccccc2)nc2c1COCC2	1
[H]/N=C/1\C(=C\c2cccc3c2cccc3)\C(=O)N=C4N1C(=NS4)S(=O)(=O)CC(C)C	0
c1cc(c2c(c1)OCCCO2)C[NH2+][C@H]3CCCc4c3[nH]nc4	0
c1ccn2c(c1)nc(c(c2=O)/C=C\3/C(=O)N(C(=S)S3)C[C@H]4CCCO4)NCCCn5ccnc5	0
O=c1[nH]c(N2CCN(Cc3ccncc3)CC2)nc2c1CCCC2	1
Cc1c(c(n(n1)C(C)C)N(C)c2cccc(c2)C#N)N	0
Cc1c(ncn(c1=O)CC(=O)N(Cc2cccc[nH+]2)c3nc4ccc(cc4s3)Br)C	0
CC(C)(C)c1cc(n[nH]1)C[NH+]2CC[C@]3(CCN(C[C@H]3C2)S(=O)(=O)C)O	0
CC[NH+]1CCN(CC1)CCC/N=C(/c2cc(nc3c2cc(cc3)S(=O)(=O)N4CCCC[C@@H]4C)[O-])\O	0
O=C(CCc1cccc(O)c1)N(Cc1nc2c(c(=O)[nH]1)COCC2)Cc1cccs1	1
C[C@@H]1CCCC[C@@H]1Nc2c(nsn2)Cl	0
Cn1c(nn(c1=S)CN2Cc3c(ccc(c3[C@@H](C2)O)OC)OC)c4ccc(cc4)Cl	0
CN1CCN(c2nc3c(c(=O)[nH]2)CCCC3)CC1	1
C1CCC(CCCCNc2nnc3n2CCS3)CC1	1
C[C@H]1CN(C[C@@H](O1)C)S(=O)(=O)c2ccc(cc2)NC(=O)CN(c3ccccn3)S(=O)(=O)C	0
c1cc2c(cc1S(=O)(=O)C(F)(F)F)nc[nH]2	0
O=C(N[C@H]1C[C@H](c2nnc(-c3ccccn3)n2-c2ccccc2F)C1)c1ccc(F)cn1	1
c1ccc2c(c1)c(cc(n2)c3ccc4c(c3)OCO4)C(=O)Nc5cccnc5	0
CC(=O)N1CCc2c(c(n3c(n2)cc(n3)[C@H]4CCCC[NH+]4Cc5cc6ccccc6o5)[O-])C1	0
CN(C)c1cc(F)c2c(c1)N(C)C(=O)C21CCN(c2nc3c(c(=O)[nH]2)CCCN3C)CC1	1
CN(C)[C@H](CNC(=O)C1=c2cc(ccc2=[NH+]C(=O)C1)S(=O)(=O)N3CCOCC3)c4cccs4	0
c1cc(ccc1c2c[nH+]c([nH]2)SCC(=O)c3ccc4c(c3)NC(=O)CO4)Br	0
CC(=O)Nc1ccc(cc1)c2csc(n2)NC(=O)[C@@H]3Cc4cc(ccc4OC3)Cl	0
Cc1cccc([nH+]1)CN2CCOc3c(cc(cc3O)c4ccc(s4)C)C2	0
C[C@@H](c1ccc(s1)Cl)NC(=O)c2ccncc2[O-]	0
CC1(Cc2c(cnc(n2)c3cc(cs3)Cl)[C@H](C1)NC)C	0
c1cc(c(cc1F)Br)NC(=O)c2ccc(cc2[O-])Cl	0
O=C(Cc1ccccc1)Nc1nnc2n1CCS2	1
c1cc(oc1)/C=C/C(=O)C2=C(C(=O)N([C@@H]2c3ccc(c(c3)Cl)Cl)CCCn4cc[nH+]c4)O	0
CCN(c1ccccc1C)c2nc3c(s2)[C@H](CCC3)[NH2+]C	0
C[C@H](c1ccc(cc1)Cl)NC(=O)CCc2[nH]c(=O)c3cnn(c3n2)c4ccccc4	0
Cc1ccc(cc1)S(=O)(=O)/N=C(/CN2CCCCC2)\N3CCN([C@@H]3c4ccccc4)S(=O)(=O)c5ccccc5	0

**c1cc2=[NH+][C@H]3CN(CCC3=c2cc1Cl)C(=O)NCCC(=O)[O-]**	0
**C[C@]12CCCN1CC1=NNC(=O)c3cccc4[nH]c2c1c34**	1
**c1ccc2=[NH+]CC(=c2c1)CCNC(=O)N[C@@H]3CC(=O)N(C3)c4ccc5c(c4)OCCO5**	0
**Cc1nnc(s1)[N-]S(=O)(=O)c2ccc3c(c2)CCCN3C(=O)[C@H](C)Oc4ccccc4**	0
**Cc1cccc2c(=O)[nH]c(CCC(=O)Nc3ccc(C(=O)Nc4cccc5cccnc45)cc3)nc12**	1
**Cc1c(c(on1)C)[C@H](C)NC(=O)N2CC[C@H](C[C@@H]2C)C**	0
**CC(C)[C@@]1(C[C@@H]([C@@]2([C@@H]1[C@H](C[C@]3([C@H](C2)O3)C)OC(=O)c4ccc(cc4)O)C)OC(=O)C)O**	0
**C[C@@H]1CCC[C@@H]([C@@H]1C)N2[C@@H](C(=O)NC3(C2=O)CCCC3)C**	0
**Cc1ccc(c(n1)Oc2c(cc(cc2N)Br)C)C#N**	0
**c1cc(c(cc1c2ccc(cc2O)F)[N+](=O)[O-])[O-]**	0
**COc1ccc(C(=O)C2CCN([C@@H]3CCN(CC(=O)Nc4nnc5n4CCS5)C3=O)CC2)cc1**	1
**Cc1c(c(on1)/C=C/c2ccco2)S(=O)(=O)N3CCC(CC3)C(=O)N[C@H]4CCCc5c4cccc5**	0
**C[C@H]1CCC[C@H](C1)[C@@H](c2cn[n-]n2)O**	0
**CC[C@H](C)Nc1c(c(=O)n2ccccc2n1)/C=C\3/C(=O)N(C(=S)S3)Cc4ccc5c(c4)OCO5**	0
**Cc1cc(cc(c1C)S(=O)(=O)N(C)C)C(=O)Nc2ccccc2N3CCCC3=O**	0
**c1ccc2c(c1)c(c[nH]2)CCNC(=O)[C@@H]3[C@H]4C=C[C@@]5([C@H]3C(=O)N(C5)Cc6cc[nH+]cc6)O4**	0
**C[C@H]1Cc2cc(ccc2N1S(=O)(=O)c3ccc(cc3)C(=O)OC)S(=O)(=O)N**	0
**c1cc(ccc1CCl)C(=O)N=c2[nH]cc[nH]2**	0
**CC[C@@H](C(=O)Nc1c(c2c(s1)CN(CC2)C(=O)C)C#N)Sc3c(on[n+]3C)[O-]**	0
**Cc1nc2c(c(n1)SCC(=O)c3ccc(cc3O)O)CCC2**	0
**C[C@]1(CCCN(C1)C(=O)NC2CCC(CC2)(C)C)C(=O)[O-]**	0
**CCOC(=O)c1ccc(cc1)C[C@@H]2[C@@H](c3ccccc3S(=O)(=O)N2Cc4ccc[nH+]c4)O**	0
**c1cc(cc(c1)F)C(=O)c2cc3c(cc2N)OCCO3**	0
**Cc1cc(no1)NC(=O)[C@@]23CC[C@@H](C2)C(C3=C)(C)C**	0
**Cn1c2c(c(=O)n(c1=O)C)n(cn2)CC(=O)N(Cc3cccc[nH+]3)c4nc5ccccc5s4**	0
**O=C(CC1CCCCC1)Nc1nnc2n1CCS2**	1
**Cc1c(c(=O)n(n1C)c2ccccc2)/C=N\NC(=O)c3csc4c3CCCC4**	0
**COc1ccc(cc1Cl)NC(=O)[C@H]2CCCN(C2)c3ccc4nnc(n4n3)C(F)(F)F**	0
**c1cc(cnc1)CN(CCC#N)S(=O)(=O)c2ccc(cc2)S(=O)(=O)NC3CCCCC3**	0
**Cc1ccnc(c1)n2c(c(c(n2)C(F)(F)F)C)O**	0
**c1ccc2c(c1)-c3ccccc3C2COC(=O)N[C@@H](Cc4c[nH+]cn4C(=O)OCC5c6ccccc6-c7c5cccc7)C(=O)[O-]**	0
**Cc1ccc(c(c1C)Oc2c(c(ncn2)Cl)N)C**	0
**COc1ccc(cc1OC)[C@H]2C(=C(C(=O)N2CCCn3cc[nH+]c3)O)C(=O)/C=C/c4ccccc4**	0
**C[C@@H]1CN(C[C@@H](N1)C)c2cc(ccc2[N+](=O)[O-])F**	0

CN(C)C(=O)[C@]1(CCCN(C1)C(=O)Cc2c[nH]nc2O)Cc3cccc(c3)c4ccccc4	0
Cc1c(c([nH]c1C(=O)OC)C)C(=O)C2=C(C(=O)N([C@@H]2c3cccc(c3)OCC=C)CCC[NH+](C)C)O	0
Cc1cc(=O)[nH]c2cc(-c3ccccc3Cl)ccc12	1
Cc1ccn2cc(nc2c1)c3ccc(cc3)NC(=O)c4ccoc4C	0
Cn1cc[nH+]c1[C@H](c2ccccc2F)NC(=O)C3CCN(CC3)S(=O)(=O)c4ccc(c(c4)F)F	0
Cc1cc(c(c2c1C(=O)/C(=C\c3cc(c(c(c3)OC)OC)OC)/O2)CN4CCN(CC4)c5ccccn5)O	0
Cc1cc(nc2c1c3c(s2)[C@H](C(=C(O3)N)C#N)c4ccncc4)C	0
COc1cccc(c1)C2=C[C@H](N3C(=[NH+]2)N=NN3)c4ccc(cc4)SC	0
Cc1cc(nc2n1c(nn2)SCCC(C)C)[O-]	0
Cc1cc(c(c(c1)C)c2cnc(o2)[C@@H](C)Cl)C	0
c1ccc(c(c1)c2ccnc(n2)n3c(c(cn3)C(=O)N4CC[NH+](CC4)CCO)C5CC5)C(F)(F)F	0
Cc1ccc(cc1)c2cc(c3c(noc3n2)C)/C(=N/c4nnc(s4)Cc5ccccc5)/[O-]	0
C=CCNC(=O)N1CCc2c(c3cc(ccc3[nH]2)Cl)C1	0
COc1ccc(cc1S(=O)(=O)Nc2ccc(cc2)C(F)(F)F)N3C(=O)CCCC3=O	0
c1cc(c(cc1S(=O)(=O)N2CCOCC2)C(=O)OCC(=O)NC3CCCCC3)Cl	0
Cc1ccc2c(c1)n(c(n2)N)c3ccc(c(c3)Br)OC	0
COc1ccccc1N1CCN(c2nc3c(c(=O)[nH]2)C[S+]([O-])CC3)CC1	1
c1cc(cc(c1)Br)/N=N/c2ccc(c(c2)/C=N/NC(=O)c3ccncc3)O	0
COc1ccc(C(=O)C2CCN(C3CCN(CC(=O)Nc4nnnn4C)C3=O)CC2)cc1	1
c1cc(ccc1[C@@H]2[C@@H]3CCC=C3C(=C(C2(C#N)C#N)N)C#N)I	0
Cc1cc2nc(cc(n2n1)N3CCC(CC3)O)C(C)C	0
CC[NH+](CC)CCN1[C@H]([C@H](C(=O)C1=O)C(=O)c2ccc3c(c2)C[C@@H](O3)C)c4cccc(c4)O	0
Cc1cccn2c1nc(c2C)C(=O)C3=C(C(=O)N([C@H]3c4ccccc4F)CCCn5cc[nH+]c5)O	0
CC[C@@H](C)N(C)C(=O)Nc1cc(c(c(c1)C(=O)[O-])C)C	0
N#Cc1ccc(CN(Cc2nc3c(c(=O)[nH]2)COCC3)C(=O)CN2CCC(C(=O)c3ccc(F)cc3)CC2)s1	1
COc1ccc(cc1OC)c2ccnc3c2c(nc(n3)N4CCc5c4cccc5)[O-]	0
C[C@@]1([C@H]2[C@@H]([C@@H](N1)c3ccc(cc3)F)C(=O)N(C2=O)c4ccc5c(c4)OCCO5)C(=O)OC	0
C[C@@H](C(=O)NNC(=O)c1cc(nc2c1cccc2)c3ccc(cc3)Cl)NC(=O)c4ccco4	0
CCOc1ccc(cc1OC)[C@@H]2c3c(c4ccccc4[nH]3)C[C@H]5N2C(=O)CN(C5=O)CCCOC(C)C	0
C[C@@H](c1ccc(cc1Cl)Cl)NC(=O)NCc2ccnc(c2)N3CC[NH+](CC3)C	0
CCN(CC)CCn1c2c3c4c(cccc41)C(=O)NN=C3CC(C)(C)C2	1
CC(=O)[C@@H](Cc1ccccc1)NC(=O)CSc2ccc(c[nH+]2)S(=O)(=O)N3CCCC3	0
Cc1c(sc(n1)C(C)(C)C)C(=O)NCCN2C[C@H](O[C@@H](C2)C)C	0
C[NH+](C)CCN(C1CCN(CC1)Cc2ccccc2S(=O)(=O)C)S(=O)(=O)	0

c3cccc4c3nccc4	
c1cc(c(c(c1)Cl)Cl)c2ccc(o2)/C=C(/C#N)\C(=C(C#N)C#N)N	0
Cc1c(c(n(n1)Cc2ccccc2Cl)C)C[NH+](C)Cn3c(=S)n4c(n3)CCCCC4	0
CCn1cc(cn1)[C@@]2(CCC[C@@H]([C@@H]2C)C)O	0
CC(C)C1Cc2nc(O)c(C#N)cc2CO1	1
Cc1nnc(SCc2nc(-c3ccc(Cl)cc3)no2)n1-c1ccc(F)cc1	1
c1cc(c(cc1NC(=O)c2ccnc(c2)Cl)[N+](=O)[O-])Br	0
Cc1cc(n2c(n1)ncn2)N3CCO[C@@H](C3)CO	0
c1cc(cc(c1)OCC(=O)NC[C@@H](c2ccco2)[NH+]3CCCCC3)NC(=O)c4ccco4	0
O=c1[nH]nc2c3c(cccc13)NC1=C2CCC1	1
CCOC(=O)C1=C(NC(=O)N[C@@H]1c2ccc(cc2)Cl)CSc3cccc[n+]3[O-]	0
CCCn1c2c(c(=O)n(c1=O)C)C(=O)/C(=C/c3cc4c(cc3Br)OCO4)/N2	0
Cc1ccc(cc1)n2c3c(c(n2)C)c(=O)c(c[nH]3)C(=O)NCc4ccc(cc4)Cl	0
O=c1cc(-c2ccc(O)c(O)c2)oc2cc(O)ccc12	1
C[C@@H]1C[C@H](CC(C1)Nc2ccc3c(n2)nccn3)C	0
c1ccc2c(c1)CCC3=C2OCO3	0
CC1CC[NH+](CC1)[C@H](c2cc3cc4c(cc3nc2O)OCO4)c5nnnn5C[C@H]6CCCO6	0
Cc1ccc(NC(=O)c2ccc(NC(=O)N3CCN(C(=O)c4cc(Cc5n[nH]c(=O)c6ccccc56)ccc4F)CC3)cc2)cc1	1
C1CCCC(CC1)c2nc(on2)[C@@H]3C[C@H](C=C3)N	0
O=c1[nH]c(-c2ccc(Cl)cc2)nc2c1COCC2	1
Cc1cccc(c1)OCC(=O)NNC(=O)c2cc(ccc2N3CCCC3)S(=O)(=O)N(C)C	0
c1cc(sc1)/C=C\2/C(=N\c3ccc(cc3)O)/NC(=O)S2	0
CN1CCCc2c1nc(N1CCC3(CC1)C(=O)N(C)c1cc(OCCN4CCOCC4)cc(F)c13)[nH]c2=O	1
CC(=O)N[C@H](Cc1ccccc1F)C(=O)N2CC[NH+](CC2)Cc3ccc(cc3)c4nc(on4)c5ccco5	0
COc1ccccc1C1=CCN(c2nc3c(c(=O)[nH]2)CCCC3)CC1	1
C[C@H]1CC[C@@H](C[C@@H]1C)Nc2c(cc(cn2)Br)N	0
COc1cccc2c1O[C@@H](N3[C@@H]2CC(=N3)c4ccccc4[O-])c5cccc(c5OC)OC	0
c1cc(cc(c1)O)/C=C(/C#N)\c2[n-]c3cc(cnc3n2)Br	0
COC(=O)C1=C(N2C(=O)CCSC2=C([C@H]1c3cccc(c3)[N+](=O)[O-])C#N)N	0
CC[C@]1(C(=O)N(C(=O)N1)NC(=O)CN[C@@H](C)c2ccc(cc2Cl)Cl)c3ccccc3	0
C[C@H]1C[C@@H]1NC(=O)c2cc(ccc2[N+](=O)[O-])F	0
Cc1ccc(c(c1)[C@H](C)NC(=O)C(=O)Nc2ccc(nc2)n3cc[nH+]c3C)OC	0
C[C@@H]1CN(C[C@H](O1)C)C(=O)c2ccccc2NC(=O)c3cc(c(c(c3)OC)OC)OC	0
c1cc(ccc1S(=O)(=O)N2CCC[C@H](C2)C(=O)NCCCn3ccnc3)Cl	0
CNC(=O)NCC(=O)N[C@@H]1CCc2c1ccc(c2)Cl	0
COc1cc(ccc1OCc2ccccc2)/C=C\3/C(=O)NC(=O)N(C3=O)c4ccc(cc4)Br	0
CCCN1CCN(CC1)C(=O)Cn2cnc3c(c2=O)c(c(s3)C)S(=O)(=O)N4	0

C[C@@H](C[C@@H](C4)C)C	
c1ccc2=[NH+]C(=O)CC(=c2c1)C(=O)OCC(=O)Nc3ccc(cc3)S(=O)(=O)N4CCCC4	0
Cc1csc(n1)CNCc2cccc(c2[O-])Br	0
CC(C)(C)[C@H]1CCc2c(c(no2)CO)C1	0
c1cc(ccc1C(=O)N/N=C\c2ccc(o2)c3cc(ccc3Cl)C(=O)[O-])Br	0
Cc1c(n(c([nH+]1)SCC(=O)Nc2c(c3c(n2C[C@H]4CCCO4)CCCC3)C#N)C5CCCCC5)C	0
C[C@@]1(CC[C@@H]2[C@H](C1)C2(C)C)C(=O)O	0
CN1CCCc2c1cc(-c1ccccc1)[nH]c2=O	1
CCN(C(=O)N1CCC(C(=O)c2ccc3c(C(=O)O)nn(C)c3c2)CC1)c1ccc(OC)cc1	1
CCCOc1ccc(cc1C)S(=O)(=O)N2CCC3(CC2)N(CCO3)S(=O)(=O)c4ccc(cc4)Cl	0
c1cc(oc1)c2c3c(c(=O)n(n2)CC(=O)Nc4cc(ccc4Cl)Cl)nc(s3)N5CCCCC5	0
CC(=O)Nc1ccc(cc1)C(=O)CSc2c3c(nc(n2)C(C)(C)C)n(c(=O)n(c3=O)C)C	0
C[C@@H]1CCc2c(sc(c2C#N)NC(=O)CN3CCN(CC3)S(=O)(=O)c4ccc5c(c4)OCCO5)C1	0
CC1=C([C@H](n2c(=O)/c(=C/c3ccc(cc3OC(=O)C)OC(=O)C)/sc2=N1)c4ccc(cc4)OC)C(=O)OC	0
c1ccc(c(c1)Cc2nc(no2)c3cc[nH+]cc3)NC(=O)C[C@H]4CSc5n4c(=O)c6c(n5)CCC6	0
Nc1ncnc2c1ncn2C1OC(C(=O)N2CCN(CC(=O)Nc3cccc4c3CNC4=O)CC2)[C@@H](O)[C@H]1O	1
C[C@H](C(=O)Nc1ccc(cc1)N2CCOCC2)Nc3cccc(c3)N4CCCC4=O	0
COCC(=O)N1CCN(C(=O)[C@H]2CC[C@H](Cn3c(=O)c4cc(OC)ccc4n(C)c3=O)CC2)CC1	1
CCn1c2ccc(cc2n(c(=O)c1=O)C[C@H](COCc3ccccc3Cl)O)S(=O)(=O)N(C)C	0
Cc1ccc(cc1Cl)NC(=O)CN2c3ccc(cc3C(C2=O)(C)C)S(=O)(=O)N4CCOCC4	0
c1cc2c(cc1NC(=O)c3ccc(cc3[N+](=O)[O-])Cl)CCC2	0
c1cc(oc1)C[NH+]2CCC(CC2)CNS(=O)(=O)c3ccc(s3)c4cc(on4)C(F)(F)F	0
Cc1csc(n1)c2cc(cn2CC(=O)N3CCOCC3)S(=O)(=O)Nc4ccccc4F	0
Cc1c(n2ccsc2n1)CNc3c(ccc4c3nsn4)Cl	0
O=c1[nH]c(N2CCC3(CC2)CNc2cccc(F)c23)nc2c1CCCC2	1
Cc1ccc(c(c1)c2cc3c(nccn3n2)N4CC[C@@H](C4)O)C	0
c1cc(c(cc1n2c(c(nn2)N)C3CC3)Cl)Cl	0
Cc1c(snn1)C(=O)Nc2c(c3c(s2)CCCC3)C(=O)NCc4cccnc4	0
CC[C@H](C)[NH+]1CCN(CC1)S(=O)(=O)c2ccsc2C(=O)[O-]	0
O=c1cc(-c2ccc(O)cc2)oc2cc(O)cc(O)c12	1
Cc1c(non1)C[NH+]2[C@H]3CCCC[C@@H]3C[C@@H]2C(=O)[O-]	0
Cc1c(c(n(n1)c2ccc(c(c2)N)C#N)C)Cl	0
Cc1ccc(cc1)S(=O)(=O)N(Cc2ccco2)CC(=O)NNC(=O)c3cn(c4c3cccc4)C	0
CCc1ccc(cc1S(=O)(=O)N2CCOCC2)C(=O)Nc3ccc(c(c3)C(=O)N)Cl	0

**O=C(CCc1ccccc1)N(Cc1ccsc1)Cc1nc2c(c(=O)[nH]1)COCC2**	1
**CSc1ccc(cc1)NC(=O)C2CCN(CC2)S(=O)(=O)[C@@H]3C=C[C@@H](O3)[C@@H]4C=CN=N4**	0
**Cc1cc(c(n1c2nccs2)C)C(=O)NCc3ccc(cc3)Cn4ccnc4**	0
**[H]/N=C\1/C(=C/c2ccc(o2)c3cccc(c3)Cl)/C(=O)N=C4N1N=C(S4)c5ccncc5**	0
**CN(C)c1ccc(cc1Cl)NC(=O)Cc2ccc[nH]2**	0
**CCn1cc(c(n1)C)[C@@H](C)NC(=O)[C@@H](C)n2c(c(c(n2)C(F)F)Br)C**	0
**Cc1ccc(cc1n2c3ccccc3nn2)/C=C/C(=O)[O-]**	0
**COc1c(cc2ccccc2n1)[C@H]3CC(=O)Nc4c3c([nH]n4)C5CCC5**	0
**CC1(C)OC(=O)N(c2ccc(-c3cnc(N)c(-c4ncc(F)cn4)c3)cc2)[C@H]1c1ccccc1**	1
**c1ccc(cc1)n2c[nH+]cc2C(=O)NNS(=O)(=O)c3ccc4c(c3)OCCO4**	0
**c1cc(sc1)S(=O)(=O)[N-]c2ncc(s2)C#CCO**	0
**c1cc2c(cc1NC(=O)N[C@H]3CCOC4(C3)CCCC4)COC2**	0
**CCOc1cccc2c1oc(=O)c(c2)c3cc(=O)oc4c3ccc(c4CN5CCN(CC5)C)O**	0
**CCOc1ccc(-c2nnc([C@H]3C[C@H](NC(=O)c4cccc5ncc(C)nc45)C3)n2-c2ccccc2F)nc1**	1
**c1c(cc(c2c1n[nH]c2C(=O)[O-])F)Br**	0
**COC(=O)[C@H](CC1=c2ccccc2=[NH+]C1)NC(=O)[C@H]3CC(=O)N(C3)c4ccccc4F**	0
**Cc1ccc(cc1)[C@H](C)NC(=O)N2CCN(CC2)c3[nH+]ccs3**	0
**COc1ccc(-c2noc(CSc3nnc(C)n3-c3ccccc3)n2)cc1OC**	1
**COc1ccc(C(=O)C2CCN([C@H]3CCN(CC(=O)Nc4nnc5n4CCS5)C3=O)CC2)cc1**	1
**c1ccc(cc1)CN2CCN(CC2)S(=O)(=O)CCNC(=O)CCCN3C(=O)c4ccccc4C3=O**	0
**CCN(Cc1nc2c(c(=O)[nH]1)COCC2)C(=O)CN1CCC(C(=O)c2ccc(F)cc2)CC1**	1
**Cc1c2c(ncn(c2=O)C)sc1C(=O)N3CCC(CC3)c4c[nH]c5c4cccc5**	0
**C[NH2+][C@H]1c2ccccc2C[C@H]1[NH+]3CCO[C@@H]4[C@@H]3CCCC4**	0
**Cc1cc(c(c(c1)C)c2nnc3n2C[C@H](CC3)CO)C**	0
**C1CCc2nnc(n2CC1)c3c4c(sc3N)CCC4**	0
**C[NH+]1CCN(CC1)[C@@H](CNC(=O)Cn2c3ccccc3oc2=O)c4ccc5c(c4)CCCN5C**	0
**c1cc2c(cc1CN)nc([nH]2)c3ccc4c(c3)nccn4**	0
**c1ccn2c(c1)c(nc2n3cc(nc3)C(=O)NCc4cccnc4)CNc5cccnc5**	0
**Cc1c(sc(n1)C)C(=O)C2=C(C(=O)N([C@H]2c3cccc(c3OC)OC)c4nc(c(s4)C(=O)C)C)O**	0
**CC1(CN(C1)c2ccc(cc2)C#N)C**	0
**c1ccc(c(c1)C(=O)N/N=C/c2c(cccc2Cl)Cl)NC(=O)c3ccco3**	0
**c1cc(c(cc1c2c(c(no2)N)Br)Cl)Cl**	0
**c1cn(cn1)CCCNC(=O)CC2(CCCC2)CC(=O)N3CCn4c(nnc4C(F)(F)F)C3**	0
**Cc1cc(cc(c1OC)C)S(=O)(=O)Nc2ccc3c(c2)N(CC3)C(=O)c4ccco4**	0
**c1cc(ccc1C#N)S(=O)(=O)N[C@@H]2CCCC[C@@H]2O**	0
**CO[C@@H]1CCC[C@@H](C1)Oc2cc3c(cc2[NH3+])OCCO3**	0
**Cc1cccn2c1nc(c2C)C(=O)C3=C(C(=O)N([C@H]3c4ccc(cc4)OCC**	0

=C)c5nccs5)[O-]	
O=C(N[C@H]1C[C@H](c2nnc(-c3ccccc3)n2-c2ccccc2F)C1)c1ccccn1	1
COc1ccc(C(=O)C2CCN(CC(=O)NCc3nc4c(c(=O)[nH]3)COCC4)CC2)cc1Cl	1
O=C(Nc1nnc2n1CCS2)C1CCCCC1	1
Cc1cc(ccc1F)[N-]S(=O)(=O)c2ccc(nc2)N	0
Cc1cccc2n1c(=O)cc([nH+]2)CS[C@@H]3N=C4C(=CN=N4)C(=O)N3c5ccc(cc5)Br	0
C[C@@H](c1ccc2c(c1)OCO2)Nc3cccc(c3)COC	0
CC(C)/C(=N/S(=O)(=O)c1c(n(c[nH+]1)C)Cl)/[O-]	0
COc1ccc(-c2cnn(C3CCN(CC(=O)NCc4nc5c(c(=O)[nH]4)COCC5)CC3)c2)cc1	1
Cc1ccc(c(c1)C)c2nc(co2)C[NH+]3CCN(CC3)c4ccccc4O	0
COc1ccc(C(=O)C2CCN(C3CCN(CC(=O)Nc4nnc5n4CCS5)C3=O)CC2)cc1	1
c1cc(oc1)c2nc(on2)c3cc(ccc3N)I	0
C[C@@]12CC[C@H]([C@@]([C@H]1Cc3c(nc(s3)NC(=O)C4CCCC4)[C@H]2CC(=O)NCCN5CCOCC5)(C)CO)O	0
Cc1cc2c(cc1C)n(cn2)[C@@H]3CCCC([C@@H]3O)(C)C	0
CC(C)n1c2c(cn1)c(cc(n2)c3ccco3)C(=O)NC(C)(C)C#N	0
Cn1c2c(c(=O)n(c1=O)C)[nH]c(n2)N[C@H]3CCC[C@H]3C[NH3+]	0
CCc1c(c2c3ncnn3c(nc2s1)N4CCN(CC4)C(=O)Nc5cccc(c5)Cl)C	0
Cc1c2c(c(cnc2sn1)C(=O)N3CCC(CC3)C)N4CCC(CC4)C(=O)Nc5ccc(cc5)OC	0
COc1ccc(cc1NC(=O)N[C@H]2CCOC3(C2)CCCC3)F	0
C[C@H]1CC(=O)N[C@H](C(=O)N1C[C@@H]2CCOC2)C(C)C	0
c1c[nH+]ccc1c2cc(nc3c2cncc3)C(=O)NCCS(=O)(=O)N4CCCC4	0
Cc1ccc(c(c1)S(=O)(=O)[N-]c2cccc(c2)C#C)O	0
O=c1[nH]c(-c2ccc(Cl)cc2)nc2c(O)cccc12	1
Cc1c(c(n(n1)C)N2C[C@@H](S[C@H](C2)C)C)N	0
O=C(c1ccc(F)cc1)C1CCN(CC(=O)N(Cc2cccnc2)Cc2nc3c(c(=O)[nH]2)COCC3)CC1	1
Cc1ccc(cc1)C(=O)C2=C(C(=O)N([C@@H]2c3ccncc3)CCCn4cc[nH+]c4)[O-]	0
Cc1c(c(n([nH+]1)C)C)CCN(C)c2c(cc(c[nH+]2)NC(=O)c3ccc(cc3)Br)C(=O)[O-]	0
CCOC(=O)c1c(c(c(s1)NC(=O)[C@@H]2CCCCN2S(=O)(=O)C)C#N)C	0
Cc1ccsc1[C@@H](C)Nc2ccc(c(c2)C(=O)[O-])Cl	0
c1ccc(c(c1)c2cnn(c2)c3c(cncn3)Cl)N	0
c1ccc2c(c1)c(cn2CC(=O)N3CCOCC3)S(=O)(=O)CC(=O)Nc4cccc(c4)Cl	0
c1ccnc(c1)c2ccc(cc2)C#N	0
CCOc1cccc2c1nc(s2)N3CCN(CC3)CCNC(=O)CS(=O)(=O)c4ccc(cc4)C	0
O=C(CCC1CCCCC1)Nc1nnc2n1CCS2	1
Cc1ccc(cc1)C[n+]2c(c(cc3c2nc4c(cccn4c3=O)C)C(=O)NC(C)(C)C)N	0
N#Cc1cccnc1N1CCN(C(=O)c2cccc(Cc3n[nH]c(=O)c4ccccc34)c2)	1

CC1	
c1c(cncc1Br)S(=O)(=O)/N=c/2\[nH]cc(s2)Br	0
O=C(CN1CCC(C(=O)c2ccc(OCC3CCC3)cc2)CC1)NCc1nc2c(c(=O)[nH]1)COCC2	1
CC1(O[C@@H]2[C@H]([C@H](O[C@@H]2O1)CN(C)C)O)C	0
Cc1nc([nH]n1)c2ccccc2N[C@H]3CSCCC3(C)C	0
C[C@]1(C2(O1)CCC(CC2)(C)C)C(=O)OC	0
c1ccc(c(c1)C#N)S(=O)(=O)N2CCC(CC2)C(=O)NNC(=O)c3ccccc3n4cccc4	0
Cc1cc(c([nH+]c1)N(C)[C@@H](C)C/C(=N/O)/N)Br	0
c1cc(sc1)[C@@H](C(=O)NC2CCCC2)N(c3ccc(cc3)O)C(=O)c4cnccn4	0
CCN(CC)C(=O)c1c2c(sc1NC(=O)c3ccc(cc3)S(=O)(=O)N(CC)CC)C[NH+](CC2)C	0
C#CCOC(=O)c1cc2c(c(c1)Cl)[nH]nn2	0
COc1ccc(-c2nc(O)c3cccc(C)c3n2)cc1	1
c1cc(c(cc1N)Oc2ccc(c(c2)Cl)C#N)Cl	0
C[C@H](c1ccc(cc1)S(=O)(=O)N)N(C)CC(=O)Nc2cc(ccc2OC)S(=O)(=O)N(C)C	0
Cc1c(c2ccccc2[nH]1)C(=O)C(=O)Nc3ccc4ccccc4n3	0
Cc1cc2c(cc1C)O[C@H](CC2=O)c3cc(cc4c3OCOC4)C(=O)[O-]	0
Cc1cc(c2cnn(c2n1)C)C(=O)Nc3nc(c(s3)C)c4ccc(cc4)Cl	0
CCNC(=O)c1c2c(sc1NC(=O)[C@@]3(C(=O)N(c4cc(ccc4O3)C)C)C)CCCC2	0
COc1ccc2c(c1)C(CC(=O)N[C@@H](CNC(=O)c1ccccc1N1CCC(=O)NC1=O)C(N)=O)CC2=O	1
Cc1cc2c(c(=O)n1CC=C)[C@]3(c4ccccc4N(C3=O)CC=C)C(=C(O2)N)C#N	0
Cc1c2c(n(n1)c3ccc(=O)[nH]n3)NC(=O)C[C@@H]2c4ccc(c(c4)OC)OC(F)F	0
c1cc2c(cc1C(=O)[O-])[nH]c([nH+]2)C3=CC=CC3	0
c1ccc2c(c1)c(n(c(=O)n2)C3CCCCC3)N	0
Cc1ccc(-c2noc(CSc3nnc(C)n3-c3ccc(F)cc3)n2)cc1	1
C[C@H](c1nnc2n1CC[NH+](CC2)Cc3ccc4c(c3)ccc[nH+]4)NC(=O)CCc5ccccc5	0
c1cc2c(cc1[N+](=O)[O-])C(=O)CCC2	0
CC(C)(O)c1ccc(-c2cn3cc(Cl)cc3c(=O)[nH]2)cc1	1
Cc1cc(c2ccc(cc2[nH+]1)C(=O)[O-])N3CC[NH2+]CC3	0
CC1(CC2=C([C@H](c3c4nc(nn4cnc3O2)CSc5nc6ccccc6o5)c7ccc(c(c7)OC)OC)C(=O)C1)C	0
c1ccc(cc1)S(=O)(=O)c2ccc(o2)C(=O)NC[C@@H](c3ccccc3Cl)[NH+]4CCOCC4	0
CCn1cc(cn1)Cn2cc(cn2)NC(=O)CCn3cc(c(n3)C)Br	0
CC(C)(C)OC(=O)N1CCC2(CC1)CCN(C(=O)c1cc(Cc3n[nH]c(=O)c4ccccc34)ccc1F)C2	1
C[C@H]1CCC[C@](CC1)(C#N)NC2CCCC2	0
CC[C@@H](C)OC(=O)C1=C(NC2=C([C@@H]1c3cc4c(cc3Cl)OCO4)C(=O)CCC2)C	0
c1c(c([nH]c1C(=O)N2CCCCCCC2)Cl)Cl	0
C1C[C@@H](CN(C1)c2nc(nc(n2)Cl)Cl)O	0
Cc1ccc(-c2nnc(CCc3nnc(-c4ccncc4)n3-c3ccccc3Cl)o2)cc1	1
CC[NH+]1CCCC[C@H]1C(=O)N2CCc3c(c(nn3CCc4ccccc4)C(=	0

O)N5CCC(CC5)O)C2	
Cc1ccc(o1)/C=C\C(=O)Nc2cc(on2)C	0
c1cc(ccc1N2CCN(CC2)C(=O)CSc3ccc(cn3)S(=O)(=O)N4CCCCC C4)O	0
Cc1cccc(c1C)c2[nH]c3cc(cnc3n2)Br	0
c1cc(cc(c1)Br)c2[nH]nc(n2)CO	0
c1ccc2c(c1)cc(o2)[C@H]3[C@H](CCO3)C#N	0
COc1cccc2c(O)nc(-c3ccc([N+](=O)[O-])cc3)nc12	1
CC[NH+]1CCC[C@H]1CNS(=O)(=O)c2cc(ccc2C)c3ccc(=O)n(n3)c4c(noc4C)C	0
Cc1cnn(c1)c2cnc(nc2CO)C(C)C	0
CN(C)CCn1c2c3c4c(cccc41)C(=O)NN=C3CC(C)(C)C2	1
c1ccc2c(c1)N(C(=O)CS2)CC(=O)OCC(=O)Nc3cccc(c3)Cl	0
CCCCOc1cccc(c1)[C@H]2/C(=C(/c3ccc4c(c3)OCCO4)\O)/C(=O) C(=O)N2CCCn5cc[nH+]c5	0
C[C@]1(C(=O)N(C(=O)N1)CC(=O)C2=CC(=NC2)C(=O)N3CCC C3)c4ccc5ccccc5c4	0
Cc1cc(cc(c1C)NC(=O)CSc2ccc(c[nH+]2)S(=O)(=O)N(C)C)S(=O)(=O)N(C)C	0
CC(C)N1CC2=NNC(=O)c3cccc4[nH]c(c2c34)C1	1
Cc1ccc(cc1)Sc2cc(nc(n2)N)Cl	0
CC(C)c1ccc(c(c1)C(C)C)NCc2cn(nn2)C	0
CN(C)[C@H]1CN(C[C@@H]1O)c2nccc(n2)C3CCCC3	0
CC/C=C(\C)/C(=O)N1CCC[C@@H](C1)S(=O)(=O)N	0
Cc1cc(n(n1)C)C[C@@]2(CCCCC2(C)C)O	0
CN1CCN(CC1)C(=O)c2ccc(c(c2)NS(=O)(=O)c3cccs3)N4CC[NH2 +]CC4	0
O=C(N[C@H]1C[C@H](c2nnc(-c3ccc(OC(F)F)cn3)n2- c2ccccc2F)C1)c1ccnc2cc(F)cnc12	1
[H]/N=C\1/C(=C/c2cccn2c3cc(ccc3Cl)[N+](=O)[O-])/C(=O)N=C4N1N=C(S4)C(F)(F)F	0
CC1(CS(=O)(=O)N(C1=O)c2cccc(c2)C(=O)Nc3nc4ccc(cc4s3)S(= O)(=O)C)C	0
C[C@H](c1[nH+]c2ccccc2n1CCCCOc3ccc(cc3)OC)NC(=O)c4cc(c(c(c4)OC)OC)OC	0
CCc1c(c(n(n1)C)C(=O)N2CC[NH+]([C@H](C2)CC)C)N	0
CC(C)n1cc2c(n1)CN(C(=O)c1cc(Cc3n[nH]c(=O)c4ccccc34)ccc1F)C2	1
Cc1ccc(cc1)S(=O)(=O)[C@@H](C(=O)N2CCN(CC2)C(=O)c3ccc (cc3)F)NC(=O)c4ccco4	0
CC(C)(C)c1cc(c(s1)C(=O)OC)NC(=O)NC(=O)c2ccccc2Cl	0
Cc1c(c(n(n1)C)C)N[C@H]2CCCC2(C)C	0
c1cc(ccc1/C=C\C(=O)N[C@@H]2CS(=O)(=O)C[C@@H]2[NH+] 3CCCCC3)C(=O)NC4CC4	0
CC(C)C1(CN(C1)C(=O)c2cccc3c2OCCO3)N	0
COc1ccccc1N2CC[NH+](CC2)CC[C@@H]3CC[C@@H]([C@H](O3)C(=O)N4CCN(CC4)c5ccc(cc5)F)O	0
Cn1c2c(cn1)[C@@H](CCC2)N(C)C(=O)N[C@@H]3CC[C@@H](C3)[NH+](C)C	0
CCn1cc(c(=O)c2c1ccc(c2)S(=O)(=O)N3CCOCC3)C(=O)NC4CC CCCC4	0
c1ccc2c(c1)CCC[C@]2(c3nc(on3)C(F)(F)F)N	0

C[C@H](C(=O)Nc1ccc(cc1)OC(F)(F)F)OC(=O)/C=C/c2ccc(c(c2)[N+](=O)[O-])Cl	0
CC(=CC/C=C/1\CC[C@H]2[C@H](C1)C(=O)NNC2=O)C	0
CC1(C[C@@H](CCO1)Nc2ccc3c(c2)nccn3)C	0
CC(C)C[C@@H](c1nnc2n1CC[NH+](CC2)Cc3ccc4c(c3)OCCO4)NC(=O)COC	0
CCc1c(nc(nc1Cl)[C@H]2C[C@H]2C)C	0
CC(C)c1ccc(cc1)[C@H]2C(=C(c3ccc(cc3)S(=O)(=O)N(C)C)O)C(=O)C(=O)N2Cc4ccc[nH+]c4	0
CS(=O)(=O)c1ccc2c(c1)sc(n2)N(Cc3ccc[nH+]c3)C(=O)c4ccc5c(c4)OCCO5	0
CC(=O)Oc1c(cc(cc1Br)/C=C(\C#N)/C(=N/c2cccc3c2cccc3)/[O-])OC	0
c1cc2c(cc1N3CC[NH+](CC3)CCCCC4=CN=C5C4=CC(=CC5)C#N)cc(o2)C(=O)N	0
CCc1ccccc1N(C)S(=O)(=O)c2ccc3c(c2)c(=O)c(c[nH]3)C(=O)NC(C)C	0
Cc1ccc2=[NH+]C(=O)/C(=C/N=C(/N)\[S-])/C(=c2c1)O	0
Cc1ccoc1[C@H](c2ccc3c(c2)CC(=O)N3)Br	0
C[C@H]1CC[C@H](C[C@@H]1C)NC(=O)[C@@H]2CCCC[C@@H]2C(=O)O	0
C[C@H](C#N)C1(CCC(CC1)(C)C)O	0
C/C(=N\NC(=O)CSc1nc2ccccc2c(=O)n1c3ccc(cc3)Cl)/c4ccc(cc4)OC(=O)C	0
c1cc2c(c(c1)n3cc(c(n3)C(=O)[O-])O)CCCC2	0
CC[C@@H]1CCCCN1c2nc3c(s2)[C@@H](CC(C3)(C)C)O	0
c1c[nH]c(=NC(=O)COc2cc(c(cc2Cl)Cl)Cl)[nH]1	0
Cc1ccc(c(c1)C)NC(=O)C(=O)Nc2ccccc2C	0
c1cc(c(cc1[N+](=O)[O-])NC(=O)C2CC2)Br	0
C[C@H](C(=O)N)Sc1nc(c2ccsc2n1)N	0
CCCCNC(=O)NC1CCC(CC1)[NH3+]	0
C[C@H]1CCCc2c1c3c4nc(nn4cnc3s2)c5cccc(c5)O	0
CN(CC(=O)N1C[C@H](Oc2c1cccc2)C(=O)N)S(=O)(=O)/C=C/c3ccccc3	0
CC[C@H](C(=O)Nc1ccc(cc1)C(=O)N2CCC(CC2)C)Oc3cccc(c3)C	0
CC(C)Oc1ccc(cc1OC)c2cc([nH]n2)C(=O)NN/C=C\3/C=C(C=CC3=O)Br	0
c1ccc2=[NH+]CC(=c2c1)C3=NN[C@@H](N3C4CC4)SCC(=O)c5ccc(cc5)N6CCCC6=O	0
c1ccc(cc1)CN2CCCC[C@H](C2=O)C3CCN(CC3)S(=O)(=O)c4c[nH]nc4	0
Cc1c(cccc1Br)NC(=O)N2CCCC[C@H]2c3[nH]cc[nH+]3	0
C/C(=N/NC(=O)C[NH+]1CCN(CC1)S(=O)(=O)c2ccc(cc2)Br)/c3ccccc3	0
CC[C@H]1CCc2c(sc(n2)C(=O)C)C1	0
CCn1cc(c(n1)S(=O)(=O)N2CC[NH+](CC2)c3cccc(c3C)C)C(=O)Nc4cccc(c4)C#N	0
Cc1c(c(n[nH]1)C(=O)N2c3ccccc3CC[C@@H]2C)N	0
Cc1csc([nH+]1)N2CCN(CC2)C[C@@H](Cn3c4ccccc4c5c3cccc5)O	0
CCc1[n-]nc(n1)C(=O)N(C)[C@H]2CCc3c2cccc3	0

**COc1cccc(c1OS(=O)(=O)c2ccccc2)/C=N/NC(=O)c3cccc(c3)Br**	0
**Cc1c2c(cc(nc2on1)c3ccccc3)C(=O)NO**	0
**CC[C@H]1CO[C@H](CN1c2[nH+]cc(s2)C(=O)[O-])C**	0
**Cc1nc2c(s1)ccc3c2sc(n3)NC(=O)c4ccn(n4)C(C)C**	0
**COCCCn1c(cnc1S(=O)(=O)C)CN2CCC[C@@H]2c3[nH]c4ccc(cc4n3)Cl**	0
**CN(C)[C@@H](C#N)c1csc2c1cccc2**	0
**C[C@@H]1[C@]([C@@](C1(F)F)(F)Cl)(C)C(=O)[O-]**	0
**CCCCn1c(nnn1)CSc2nc3c(c4c(s3)CCC4)c(=O)n2c5cccc(c5)OC**	0
**O=C(CCc1nc2ccccc2c(=O)[nH]1)Nc1ccc(-c2nnc(-c3ccccc3)o2)cc1**	1
**c1cc2c(cc1/C=C\C(=O)N3CCC[C@@H](C3)Cc4nc(no4)c5ccsc5)OCO2**	0
**Cc1cccc(c1)C(=O)N[C@@H](C(=O)N2CCN(CC2)C(=O)c3ccccc3)S(=O)(=O)c4ccccc4**	0
**O=C(CCSc1nc2c(c(=O)[nH]1)CCCC2)N[C@H]1CC[C@H](c2nnc(-c3ccccc3)o2)CC1**	1
**c1cc(sc1)S(=O)(=O)NC[C@H](c2ccc3c(c2)OCO3)N4CCN(CC4)c5ccc(cc5)F**	0
**CC[NH+]1CCN(CC1)c2c(c(=O)n3ccccc3n2)/C=C(/C#N)\c4[nH]c5ccccc5n4**	0
**C[C@H](c1ccccn1)Nc2ccc(cc2[N+](=O)[O-])C(=O)N3CCC[C@H](C3)O**	0
**COC(=O)c1c(c2ccc(nc2s1)CN3CCN(CC3)c4cccc[nH+]4)NC(=O)c5ccsc5**	0
**C[C@H]1CCCC[C@]12C(=O)N(C(=O)N2)CN3CCN(CC3)S(=O)(=O)c4ccc5c(c4)CCC5**	0
**CCOc1cc(cc(c1O)Br)CC2=Nc3c(c(=O)n(c(=O)n3CC(C)C)C)C2=O**	0
**C[C@@]1(C(=O)N(C(=O)N1)Cc2c(c(c(c(c2F)F)F)F)F)c3ccc4c(c3)OCO4**	0
**CC1=C(C[C@@H]([C@@H](C1)C(=O)Nc2c(c3c(s2)CCCC3)C(=O)N4CCOCC4)C(=O)O)C**	0
**Cc1ccc2c(c1Br)[nH]cn2**	0
**CCc1c(c(no1)C)C(=O)N2CCC[C@@H]2c3cc4n(n3)[C@H](C[C@H](N4)C)C(F)(F)F**	0
**c1cc(ccc1NC(=O)CN2CCN(CC2)CC(=O)N3CCCC3)S(=O)(=O)N4CCCCC4**	0
**c1cc[nH+]c(c1)CNC(=O)c2c3c(n(n2)CC4CC4)CCN(C3)C(=O)[C@H]5CC56CCCCC6**	0
**Cn1c2ccc(cc2nc1CCC(=O)NC3[NH+]=c4ccccc4=[NH+]3)S(=O)(=O)N(C)C**	0
**Cc1ccc2c(c1)sc(n2)N3[C@@H](C(=C(C3=O)O)C(=O)c4c(nc(s4)C)C)c5cccc(c5OC)OC**	0
**CC(C)c1c(cnc(n1)N2CCC(CC2)NC(=O)[C@H]3CC(=O)OC3(C)C)c4cc[nH+]cc4**	0
**CN(C)CCn1c2c3c4c(cc(F)cc41)C(=O)NN=C3CC(C)(C)C2**	1
**C[C@@H]1CN(C[C@@H](O1)C)S(=O)(=O)N[C@@H](C)c2ccccc2OC**	0
**CC[NH+](CCCNS(=O)(=O)c1ccc2c(c1)n(c(=O)c(=O)n2C)C)Cc3ccccc3**	0
**Cc1ccc(cc1)S(=O)(=O)NCCC(=O)N[C@@H](C)c2ccc(cc2)n3cncn3**	0

C[C@H]1C(=c2ccccc2=[NH+]1)C(=O)COC(=O)CN(C)S(=O)(=O)c3ccc(cc3)NC(=O)C	0
CCc1cc(n(n1)CC)c2[nH]c3cc(cnc3n2)Br	0
COc1ccc(C(=O)C2CCN(CC(=O)NCc3nc4c(c(=O)[nH]3)COCC4)CC2)cc1C	1
Cc1ccc(cc1Cl)S(=O)(=O)[N-]c2ccc(c(c2)C(=O)NC(C)C)N3CC[NH2+]CC3	0
c1ccc(cc1)/C=C\S(=O)(=O)N2CCN(CC2)C(=O)CSc3nnnn3c4ccc(cc4)O	0
Cc1cc(n2c(n1)nnc2SCC(=O)NNC(=O)c3ccc(cc3)Br)C	0
CC(C)c1cc(no1)c2n[nH]c(=S)o2	0
Cc1cccn2c1[nH+]c(c2)CNC(=O)c3cn(cc(c3=O)C(=O)N(C)CC(C)C)C4CCCC4	0
Cc1ccc(cc1/[NH+]=c\2/c(cc3ccc(cc3o2)O)C(=O)Nc4nccs4)F	0
c1ccc2=[NH+]CC(=c2c1)C[C@@H]3C(=O)N(C(=O)N3)CC(=O)N([C@@H]4CCS(=O)(=O)C4)C5CCCC5	0
Cc1ccc(cc1F)Nc2ccc(cc2[N+](=O)[O-])S(=O)(=O)C	0
CCOc1ccc(-c2nnc([C@H]3C[C@H](NC(=O)c4ccccn4)C3)n2-c2ccccc2F)nc1	1
CCC[C@@H]1C[C@](CCO1)(c2nccs2)N	0
Cc1c(C(N)=O)ccc2c1c(=O)n(C[C@H]1CC[C@H](C(=O)Nc3ccncc3)CC1)c(=O)n2Cc1cccc(C(F)(F)F)c1C#N	1
c1ccc(cc1)[C@H](C(=O)NC2CC2)N3CCN(CC3)S(=O)(=O)c4ccccc4Cl	0
Cc1c(c(n[nH]1)C)S(=O)(=O)N2CCC[C@H](C2)C(=O)N3CCC(CC3)CC(=O)N	0
Cc1ccc(c(c1C)O)C(=O)Nc2ncc(s2)C	0
CC1(CCN(CC1)[C@H]2CCCCC[C@@H]2C#N)C	0
N#Cc1ccc2c(c1)[nH]c(=O)n2-c1ccc(-c2nnc(-c3ccncn3)n2-c2ccccc2Cl)cc1	1
c1ccnc(c1)N2C[C@@H]3CN(CCC[C@@]3(C2)C(=O)NCC4CC4)C(=O)CC5CC5	0
CSc1ncc(cn1)C[NH+]2CC[C@H]([C@@H](C2)O)N3CCN(CC3)c4ccccn4	0
Cc1ccc(cc1C(=O)N[C@@H](c2ccccc2)c3[nH+]ccn3C)S(=O)(=O)NC4CC4	0
c1ccc(cc1)C(=O)N[C@@H](C(Cl)(Cl)Cl)n2cnc3c2ncn(c3=O)[C@H](C(Cl)(Cl)Cl)NC(=O)c4ccccc4	0
O=C1NC(c2ccccc2OCc2ccccc2)Nc2ccccc21	1
c1ccc2c(c1)c(nc(n2)C(F)(F)F)SCc3nc4ccccc4c(=O)n3CC(=O)N	0
Cc1ccc(cc1)CN2C(=O)/C(=C\c3ccc(cc3C)N4CCOCC4)/NC2=O	0
c1ccc(c(c1)Oc2c(c(=O)n3ccccc3n2)C=O)Cl	0
c1c(cc(c(c1F)F)F)c2[nH]cc(n2)C(=O)[O-]	0
Cc1c(sc2c1c(=O)n(cn2)CCC(=O)N[C@H](C)c3ccc(cc3)n4cc[nH+]c4)C	0
CC[C@@H](C)N(Cc1nc2c(c(=O)[nH]1)COCC2)C(=O)CN1CCC(C(=O)c2ccc(F)cc2)CC1	1
[H]/N=C/1\C(=C\c2cc(c(c(c2)Cl)OCCOc3ccccc3CC=C)OC)\C(=O)N=C4N1OC(=C4)C	0
COc1ccc(C(=O)C2CCN(CC(=O)NCc3nc4c(c(=O)[nH]3)CCCCC4)CC2)cc1	1
O=c1[nH]c(N2CCC3(CC2)CN(CCO)c2ccccc23)nc2c1CCCC2	1

O=C1NC(c2ccc(Cl)cc2)Nc2ncccc21	1
COc1ccc(C(=O)C2CCN(C3CCN(Cc4ccc(Cl)c(C#N)c4)C3=O)CC2)cc1	1
CCc1c[nH+]c2c(c1NOCC(=O)N3CCN(CC3)c4ccccc4)c(=O)n(c(=O)n2C)C	0
COc1ccc2cc([nH]c2c1)C(=O)Nc3ccc4c(c3)CCC4	0
Cc1ccc(nc1)[C@@]2(CC[C@@H]3[C@H]2C[NH+](C3)Cc4cc5c(cc4C)OCO5)O	0
Cc1c(sc2c1c(nc(n2)CC[NH3+])N3CCN(CC3)c4c(cccn4)C#N)C	0
CCOc1ccc(cc1)N2C(=O)[C@](C(=O)NC2=O)(Cc3ccc(cc3)OC)CN4C[C@H]5C[C@@H](C4)c6cccc(=O)n6C5	0
O=c1[nH]c(N2CCC3(CC2)CNc2cc(Cl)ccc23)nc2c1CCCC2	1
[H]/N=C\1/C(=C/c2ccc(cc2)OC(=O)c3ccco3)/C(=O)N=C4N1N=C(S4)[C@H](CC)c5ccccc5	0
COc1ccc(cc1)S(=O)(=O)[C@@H](CNS(=O)(=O)c2ccc(cc2OC)OC)c3ccc[nH+]c3	0
c1cc(ncc1C(=O)/N=C(/c2cc(cc(c2)Cl)Cl)\N)Cl	0
O=C1Nc2cc(Cl)ccc2C12CCN(c1nc3c(c(=O)[nH]1)CCCC3)CC2	1
CCCCN1[C@@H](CC(=O)NC2(C1=O)CCCC2)C	0
CC[C@H](C)[C@@H](C(=O)[O-])NC(=O)[C@H](C(C)C)N1C(=O)[C@@]2([C@@H]3C(=c4ccccc4=[NH+]3)CCN2C1=O)C	0
c1c(cc(c(c1Cl)NC(=O)N2CCOCC2)Cl)Cl	0
COc1ccc(C(=O)C2CCN(CC(=O)NCc3cc4c(c(=O)o3)COCC4)CC2)cc1	1
c1ccc2c(c1)C(c3ccccc3O2)/C(=N/c4nnc(s4)[C@@H]5CC(=O)N(C5)c6ccc(cc6)F)/[O-]	0
COc1ccc2cc(c(nc2c1)OC)C[NH+]3CCc4c(nc(nc4[O-])c5ccc[nH+]c5)C3	0
Cc1ccc(c(c1)C(=O)[O-])[NH+]2C[C@H](OC[C@@H]2C)C	0
c1ccc(cc1)S(=O)(=O)NCc2nnc3n2CCN(CC3)Cc4ccccc4C(F)(F)F	0
CC(C)(C)NC(=O)Nc1cn(c2c1cccc2)C	0
C[C@@]1(C(=O)N(C(=O)N1)Cc2c(c3ccccc3s2)Cl)c4ccc(cc4)S(=O)(=O)C	0
Cc1c(c(=O)c2ccc3c(c2o1)CN(CO3)[C@H]4CCS(=O)(=O)C4)Oc5cccc(c5)OC	0
C1CCc2c(c(c(s2)N)c3nc(no3)[C@H]4CCOC4)C1	0
CC[C@H]1CCCN1C(=O)[C@]2(CCC[C@@H](C2)C)N	0
CCOc1cccc(c1)N2C(=O)/C(=C\c3ccc(o3)I)/C(=O)NC2=O	0
CC[C@@H]1C[C@H](CCO1)NCc2[nH]c3ccccc3n2	0
Cc1c2c(on1)C[C@@H](CC(=N2)c3ccc(cc3)O)c4ccccc4	0
COc1cccc(N2CCN(c3nc4c(c(=O)[nH]3)CCCC4)CC2)c1	1
CN1CCCc2c1cc(-c1ccc(C(F)(F)F)cc1)[nH]c2=O	1
Cc1ccccc1N2C[C@@H](CC2=O)C(=O)Nc3ccc(cc3)S(=O)(=O)NC(C)(C)C	0
C1CCC(C1)N2CCC[C@H]2C#N	0
c1c(c(nc(n1)Cl)NC2CCCCCC2)Br	0
Cc1cc(on1)C(=O)N[C@@H](c2ccccc2OC)c3ccccn3	0
Cc1ccc(c(c1)N2C(=O)/C(=C\c3cc(c(c(c3)Br)O)OC)/C(=O)NC2=O)C	0
Cc1c(nnn1c2c(non2)N)C(=O)N/N=C/c3cc(c(c(c3)Br)OCc4cccc5c4cccc5)OC	0

[H]/N=C/1\C(=Cc2ccc(o2)c3ccc(c(c3)Cl)OC)C(=O)N=C4N1N=C(S4)COc5ccccc5	0
c1[nH]c2c(n1)CN[C@@H](C2)c3nc(no3)C4CCCCC4	0
O=C(CCc1cccc(Cl)c1Cl)Nc1nnc2n1CCS2	1
Cc1c(cnc2n1ncc2c3ccc4c(c3)OCO4)C(=O)NCCC[NH+]5CCCC[C@@H]5C	0
Cc1cccc(c1C)NC(=O)Nc2ccc(c(c2)C(=O)N(C)C)F	0
C[C@@H]1CN(C[C@@H](O1)C)c2ccc(c[nH+]2)CNC(=O)c3ccc(cc3)Cn4cncn4	0
Cc1ccc2c(c1)C3=Nc4ncnn4[C@@H]([C@@H]3[C@@H](O2)c5cc(c(c(c5)OC)OC)OC)c6ccc[nH+]c6	0
CCN(Cc1nc2c(c(=O)[nH]1)COCC2)C(=O)CCc1ccccc1	1
c1cc(ccc1[C@@H]2CC(=O)Nc3c2cc4c(c3)OCO4)Br	0
COc1ccccc1C1CCN(c2nc3c(c(=O)[nH]2)CCCC3)CC1	1
CC(=O)Nc1ccc(cc1)NC(=O)CN(c2cccc3c2cccc3)S(=O)(=O)C	0
Cc1ccccc1NC(=O)c1ccc(NC(=O)N2CCN(C(=O)c3cc(Cc4n[nH]c(=O)c5ccccc45)ccc3F)CC2)cc1	1
COc1cccc(c1)c2c(cn(n2)Cc3ccccc3)C(=O)NCc4nnc5n4CCC5	0
COc1ccc(NC(=O)c2ccc(NC(=O)N3CCN(C(=O)c4cc(Cc5n[nH]c(=O)c6ccccc56)ccc4F)CC3)cc2)cc1	1
Cc1cccn2c1nc(c2C)C(=O)C3=C(C(=O)N([C@@H]3c4cccc(c4)Cl)CCC[NH+]5CCOCC5)O	0
C[C@@H]1C[NH+]2CCC[C@@H]2CN1c3ncc(s3)C(=O)[O-]	0
c1cnc2c(cnn2c1)CN3CCC[C@@H]3c4ccn[nH]4	0
C[C@H](C(=O)NCc1ccc(c(c1)OC)OC)Sc2c3cnn(c3ncn2)c4ccc(cc4)F	0
O=c1[nH]c(N2CCC3(CC2)CNc2ccccc23)nc2c1CCCC2	1
Cc1nc(cs1)c2cccc(c2)NC(=O)c3csc(n3)Nc4ncccn4	0
CC(C)(C)CC(=O)N1CCN(CC1)[C@H](C2CCCC2)C(=O)NCCC[NH+]3CCOCC3	0
Cc1c2ccccc2c(=O)n(n1)CC(=O)Nc3ccc4c(c3)nc(n4C)CN5CCN(CC5)C	0
O=C(Nc1nnc2n1CCS2)C1CCC1	1
Cc1ccc(cc1C)C(=O)Nc2c(ccc(c2C)[N+](=O)[O-])C	0
Cn1c2ccccc2nc1[C@@H]3CNCC4(O3)CCCC4	0
Cc1c2cnn(c(=O)c2c(n1C)C)CN3CCN(CC3)S(=O)(=O)c4ccc5c(c4)OCCCO5	0
O=C(CN1CCC(C(=O)N2CCc3c(Cl)cccc3C2)CC1)NCc1nc2c(c(=O)[nH]1)COCC2	1
C1CCC2(C1)CCN(CC2)c3nc(ns3)N	0
Cc1ccc(cc1)n2c(=O)n(c(=O)c(n2)C(=O)NCc3ccc[nH+]c3)Cc4ccccc4C	0
CCN1[C@H]([C@H](OC1=S)C=C(C#N)C#N)O	0
C[C@H]1CCC[C@]2(C1)CN[C@@H]([C@@H](O2)c3ccco3)C	0
Cc1ccc(s1)[C@H](C)NC(=O)c2cc(ccc2NC)Cl	0
Cc1ccc2c(c1)cc(o2)c3n[nH]c(=S)n3C(C)C	0
Cn1c2c(c(=O)[nH]c1=O)n(c([nH+]2)N3CCCCC3)CCSc4nnnn4c5ccccc5	0
c1ccnc(c1)CNC(=O)c2c(snn2)NC(=O)N3C[C@@H]4C[C@H](C3)c5cccc(=O)n5C4	0
CC1(CCCC[C@@H]1n2ccc3c2CCCC3=O)C	0
Cc1ccc(cc1)S(=O)(=O)Oc2ccc(cc2)/C=C/3\C(=NN(C3=O)c4ccccc	0

4)[O-]	
CC(C)(C)c1ccc(cc1)S(=O)(=O)[C@H]2CS(=O)(=O)C[C@@H]2N[C@H]3CCS(=O)(=O)C3	0
Cc1cc(ncc1Br)NC(=O)NC2CCCCC2	0
CC(C)Oc1c(ccc(n1)N2CC[NH+]3CCC[C@@H]3C2)N	0
COc1ccc(cc1F)c2ccc(c(c2)O)C#N	0
Cc1cc(no1)NS(=O)(=O)c2ccc(cc2)NC(=O)CCCC(=O)Nc3ccc(cc3)S(=O)(=O)Nc4cc(on4)C	0
Cc1c\2c(oc1C(=O)N3CCN(CC3)c4ccccc4)CCC/C2=N\NC(=O)c5ccoc5C	0
CCN(CC)C(=O)COc1ccc(cc1OC)[C@H]2C3=C(CC(CC3=O)(C)C)Nc4n2ncn4	0
c1cc(c(cc1F)Cl)NCc2nc3ccsc3c(n2)[O-]	0
CC(=O)N1CCc2c(sc(c2c3nc4ccccc4s3)NC(=O)c5ccc(s5)[N+](=O)[O-])C1	0
[H]/N=C\1/C(=C/c2cc(n(c2C)c3ccc(cc3OC)OC)C)/C(=O)N=C4N1N=C(S4)CCC	0
Cc1c(c(n(n1)C)C)NC(=O)[C@@H](C)Sc2[nH]c(c([nH+]2)c3cccs3)c4cccs4	0
CCC[C@@H](C)c1nc(ns1)N	0
COc1c(cc(cc1Br)c2nc3cnccc3o2)N	0
COc1cccc(c1)S(=O)(=O)NC[C@H](Cn2ccccc2=O)O	0
Cc1ccc2ccc(c(c2n1)O)[C@H](c3cccc[nH+]3)Nc4ccc(cn4)Cl	0
[H]/N=C\1/C(=C/c2cc(c(c(c2)OC)OS(=O)(=O)c3ccccc3)CC=C)/C(=O)N=C4N1N=CS4	0
COCCOc1cc(F)c2c(c1)N(C)C(=O)C21CCN(c2nc3c(c(=O)[nH]2)CCCN3C)CC1	1
Cc1cc(nc(n1)NS(=O)(=O)c2ccc(cc2)/N=C/c3c4ccccc4ccc3O)C	0
Cc1ccc(c(c1)C)S(=O)(=O)NC(=O)c2cccc3c2OCCO3	0
COc1ccccc1N1CCN(c2nc3c(c(=O)[nH]2)CC(C)CC3)CC1	1
CC[C@H]1CNC[C@]2(O1)CCCC2(C)C	0
Cc1ccc(cc1NC(=O)c2ccc(s2)C)C#N	0
c1c2c(cc(c1Cl)N[C@H]3C[C@H]4CC[C@@H]3C4)OCC(=O)N2	0
c1ccc(cc1)CSc2cc(nc(n2)N)Cl	0
O=C(Nc1cccc2cccnc12)c1ccc(N2C(=O)[C@@H]3[C@H](C2=O)[C@H]2C=C[C@H]3C2)cc1	1
c1cc(ccc1OCCN2CC[NH+](CC2)Cc3c(c(coc3=O)C[NH+]4CCSCC4)[O-])F	0
c1c(cc(c(c1[N+](=O)[O-])F)Cl)F	0
CCCCNC(=O)[C@@H]1[C@@H](Nn2c(nnc2S1)c3ccccc3)c4ccc(c(c4)Cl)OC	0
Cc1csc(n1)c2cc(cn2CC(=O)N3CCCC3)S(=O)(=O)Nc4ccc(c(c4)Cl)OC	0
C[C@@]1(Cn2c(cc(n2)C(=O)NCCC[NH+]3CCCCC3)C(=O)N1C)C(=O)NC4CCCCC4	0
Cc1cn2c3c(=O)[nH]c(=O)n(c3nc2n1CC[NH+]4CCN(CC4)Cc5ccc(cc5Cl)Cl)C	0
Cc1ccc(cc1)NC(=O)[C@H](C)Sc2c3c(c4c5c(c(nc4s3)N6CCOCC6)COC(C5)(C)C)ncn2	0
CC(C)n1c[nH+]cc1c2c(n3cc(ccc3n2)Cl)N	0
Cc1ccccc1COc2ccc(cc2)/C=C\3/C(=NC(=O)N(C3=O)c4ccc(cc4)Br)[O-]	0

**Cc1cc(no1)NS(=O)(=O)c2ccc(cc2)NC(=O)CCCCC(=O)Nc3ccc(cc3)S(=O)(=O)Nc4cc(on4)C**	0
**COc1ccccc1N1CCN(c2nc3c(c(=O)[nH]2)CCCC3)CC1**	1
**COc1ccc(cc1)CN(Cc2cc3cc(c(cc3[nH]c2=O)OC)OC)S(=O)(=O)c4ccc(cc4)Cl**	0
**O=C(c1ccc(F)cc1)C1CCN(CC(=O)N(Cc2ccncc2)Cc2nc3c(c(=O)[nH]2)COCC3)CC1**	1
**CN1CCCc2c1cc(-c1ccc(Br)cc1)[nH]c2=O**	1
**CCC1CCC(CC1)(c2nc(on2)[C@@H]3CCCOC3)[NH3+]**	0
**Cc1cc(ccc1Br)NC(=O)c2cc(cn2C)S(=O)(=O)N3CCOCC3**	0
**Cc1nc2ccc(cc2s1)S(=O)(=O)N3CCN(CC3)c4ccc(c(c4)NCc5ccccc5)[N+](=O)[O-]**	0
**Cc1cccc(c1)n2c(=O)n(c(=O)c(n2)C(=O)NCCc3cccc[nH+]3)Cc4ccc(cc4)F**	0
**COc1ccc(N2CCN(c3nc4c(c(=O)[nH]3)CC(F)(F)CC4)CC2)cc1**	1
**Cc1cc(c(n1c2ccc(cc2)S(=O)(=O)N)C)C(=O)COC(=O)c3ccccc3**	0
**CSCC[C@H](C(=O)OCC(=O)c1ccc(s1)Cl)NS(=O)(=O)c2ccc3c(c2)OCCO3**	0
**CCOC(=O)N1CCN(CC1)Cc2ccc(cc2)OC[C@H]3CCCN(C3)C(=O)c4cccc5c4[nH]c(c5C)C**	0
**C[C@H]1CCCC[C@H]1Oc2c(cnc(n2)N)Cl**	0
**Cn1c[nH+]c(c1Cl)S(=O)(=O)[N-]c2ccc(cc2)[NH+]3CCCCC3**	0
**CC[NH+]1CCN(CC1)c2ccc(cc2NC(=O)Cn3c(csc3=O)C)S(=O)(=O)N(CC)CC**	0
**Cc1cc2nc(CSc3nnc(-c4ccncc4)n3-c3ccccc3Cl)cc(=O)n2cc1C**	1
**CC(C)NC(=O)C(=O)N1CCN(CC1)c2c(c3c(c(n2)c4ccco4)COC(C3)(C)C)C#N**	0
**C[C@H]1CCCCCN1c2ccc(c(c2)N)S(=O)(=O)N**	0
**CC(C)(C)[C@]1(CC(=NN1C(=O)c2ccc(cc2)O)C(F)(F)F)O**	0
**Cc1ccc(cc1F)S(=O)(=O)[N-]c2cc3c(cc2Sc4ccc(cc4)OC)n(c(=O)n3C)C**	0
**CCC[C@@H](c1nnnn1C2CCCC2)N(Cc3ccc(cc3)F)Cc4cc5cc6c(cc5[nH]c4=O)OCCO6**	0
**COc1cc(F)c2c(c1)N(C)C(=O)C21CCN(c2nc3c(c(=O)[nH]2)CCCN3C)CC1**	1
**c1cc2c(cc1CNc3cc4c(cc3Cl)OCO4)OCO2**	0
**c1cc(c[nH+]c1)c2csc(n2)NC(=O)[C@H]3CCCN(C3)S(=O)(=O)c4ccc(cc4)F**	0
**O=C(c1ccc(F)cc1)C1CCN(CC(=O)N(Cc2nc3c(c(=O)[nH]2)COCC3)C[C@@H]2CCCO2)CC1**	1
**c1c2c(nc(c1C#N)Sc3[n-]ncn3)CCC2**	0
**COc1ccc(cc1NC(=O)CSC2=NC(=C)C([C@H]([C@@H]2C#N)c3ccco3)C(=O)N)Cl**	0
**Cc1c(c(=O)n(n1C)c2ccccc2)N3C(=O)/C(=C\c4ccc(c(c4)OC)OC(=O)c5ccccc5)/SC3=S**	0
**CC(C)(C)NC(=O)N1CCC(CC1)CNS(=O)(=O)c2cc(cc(c2)F)F**	0
**COc1ccc(cc1)S(=O)(=O)NCCS(=O)(=O)N2CCN(CC2)c3ccc(cc3)F**	0
**Cc1[nH+]ccn1Cc2cccc(c2)CNC(=O)Nc3ccc(cc3)CN4CCCC4=O**	0
**Cc1c(c(on1)C)[C@@H](C)N2CCCNC3(C2)CCCCC3**	0
**COc1ccccc1N1CCN(c2nc3c(c(=O)[nH]2)COCC3)CC1**	1
**c1cc(ncc1NC(=O)C2=NC=C(C2)S(=O)(=O)N3CCCC3)N4CCCC**	0

C4	
CN1CCC(c2ccc(-c3nc4c(F)cc(F)cc4c(=O)[nH]3)cc2)CC1.O=C(O)C(F)(F)F	1
Cc1cc2ncnn2c(c1)N[C@@H]3CC[C@H]([C@@H](C3)C)C	0
c1ccc(cc1)[C@@](c2cc3ccccc3[nH]2)(c4cc5ccccc5n4S(=O)(=O)c6ccccc6)NS(=O)(=O)c7ccccc7	0
CSc1nc(sn1)NC(=O)N2CC[C@H]3[C@H]2CCCC3	0
C[C@]1(C(=O)N(C(=O)N1)CN2CCN(CC2)Cc3ccc4c(c3)OCO4)c5ccc6c(c5)CCC6	0
[H]/N=C\1/C(=C\c2ccc(c(c2)OC)OCCOc3ccc(c(c3)C)C)/C(=O)N=C4N1N=C(S4)CC	0
Cc1ccc(cc1)c2c(nn(c2NC(=O)C)c3ccc(cc3)C(=O)Nc4cccc(n4)C)C	0
c1cc(ccc1c2c(cnc(n2)N)F)Cl	0
Cc1cccc2c(O)nc(-c3ccccc3)nc12	1
c1ccc(c(c1)Oc2c3cc(ccc3ncn2)N)Cl	0
c1ccc(cc1)S(=O)(=O)c2cn(c3c2cccc3)CC(=O)NCCCN4CCCC4=O	0
c1c2c(sc1c3nc(co3)CCl)CCC2	0
C[C@]1(C(=O)N(C(=O)N1)Cc2cccc(c2)Br)c3ccc4c(c3)OCO4	0
Cc1ccc(nc1)n2c(c(c(n2)C)CC(=O)[O-])C	0
CN1CCN(CC1)C(=O)c2coc(n2)C[NH+](Cc3ccc4c(c3)OCO4)Cc5cccc(c5OC)OC	0
Cc1c(nc(nc1N)c2ccc(cc2)Br)C	0
CN(C)c1ccc2c(n1)[nH]c(n2)c3ccc(cc3)F	0
C[C@@H]1CC[C@@H](C1)c2nc3c(c(n2)N)CCCC3	0
Cc1nc(cs1)COc2ccccc2C(=O)NNC(=O)c3cccc(c3)S(=O)(=O)N(C)C	0
C[C@H](C(=O)Nc1ccc(cc1)N(C)C)OC(=O)CCNC2=NS(=O)(=O)c3c2cccc3	0
Cc1cc(c(c(c1)C)[C@@H](Cc2c(nn(c2Cl)C)C)[NH2+]N)C	0
CCOC(=O)C1=C(N=C([C@@H]1S(=O)(=O)N2CCC(CC2)C(=O)NC3CCC(CC3)C)C)C	0
Cc1cc(nc(n1)C(C)C)N2CCN(CC2)C(=O)[C@H](C)NC3=NS(=O)(=O)c4c3cccc4	0
COc1ccc(-c2noc(CSc3nnc(-c4ccncc4)n3-c3ccc(OC)cc3)n2)cc1	1
Cc1c(c(on1)C)S(=O)(=O)N2CCC(CC2)C(=O)Nc3nc(cs3)CN4C[C@H](O[C@H](C4)C)C	0
CS(=O)(=O)Nc1ccc(cc1)C(=O)CSc2nnc(n2C3CC3)COc4ccccc4Cl	0
Cc1cccc(c1)CN2CCCn3c(cc(n3)C(=O)N[C@H](C)C(=O)NCc4cc[nH+]cc4)C2=O	0
Cc1ccc(-c2nc3c(sc4ccccc43)c(=O)[nH]2)cc1	1
C[C@H]1CCC[C@@]2(C1)CS/C(=N\c3cccc(c3)C#N)/N2	0
Cc1cc(c(cc1Cl)OC)NC(=O)c2cnc3c(c(nn3c2C)C)c4ccccc4	0
Cc1cccc2c(=O)[nH]c(-c3ccc(F)cc3)nc12	1
Cn1cc(cc1C(=O)N2CCC[C@@H]2C3CCCCC3)N	0
O=C(CN1CCC(C(=O)c2ccc3c(c2)CCO3)CC1)NCc1nc2c(c(=O)[nH]1)COCC2	1
Cc1ccoc1C(=O)Nc2cnc(cn2)Br	0
CCO[C@@]1(OCC(=O)[C@@]2(O1)CC[C@@H]3[C@@]2(C[C@@H]([C@@H]4[C@@H]3CCC5=CC(=O)C=C[C@@]45C)O)C)C	0
CC1([C@H]2CC=C([C@@H]1C2)/C=N\N3CCCCC3)C	0

C[C@@H]1CCCCCN1c2cc(nc(n2)N)Cl	0
Cc1cc(c(cc1Cl)Cl)S(=O)(=O)N2CCc3c(sc(n3)NC(=O)c4cnccn4)C2	0
CS(=O)(=O)N1CCC(C2Cc3[nH]c(=O)c4ccccc4c3CO2)C1	1
Cc1ccc(c(c1)NC(=O)[C@H](c2ccccc2)Sc3cc(nc(n3)C)C)OC	0
C[C@H]1CN[C@@H](CN1c2cc(c(cc2[N+](=O)[O-])Br)F)C	0
Cc1c(c2nc(c3ccccc3n2n1)N4CCN(CC4)Cc5ccsc5)Cl	0
C[C@@H]1CC[C@](C1)(C#N)C2(CCCCC2)O	0
Cc1c(snn1)C(=O)Nc2nc(cs2)CCl	0
c1cc(ccc1/C=C/2\COc3cc(ccc3C2=O)O)O	0
Cc1ccc2c(c1)[nH]c([nH+]2)CSc3nnc(n3CC=C)CCc4ccc(cc4)I	0
CNc1cc(ncn1)Nc2nccc(n2)[C@H]3CCCN(C3)Cc4ccc[nH+]c4NC	0
[H]/N=C\1/C(=C/c2ccc(cc2)[NH+](CC)CC)/C(=O)N=C3N1N=C(S3)C4CCCCC4	0
CCOC(=O)C1CCN(CC1)C(=O)[C@@H]2CCCN(C2)S(=O)(=O)c3ccc4c(c3)CCCC(=O)N4	0
O=C(CCSc1nc2ccccc2c(=O)[nH]1)N[C@H]1CC[C@H](c2nnc(-c3ccccc3)o2)CC1	1
Cc1c(sc(n1)[S-])CC(=O)Nc2ccc(cc2)Br	0
c1cc(cc(c1)N[C@H]2CCN(C2)C3CC3)c4[n-]nnn4	0
C[C@H]1CNC[C@@H](O1)c2ccc(cc2)C(C)(C)C	0
Cc1c(c(n(n1)C)Sc2cc3ccccc3[nH]2)C#N	0
Cc1coc2c1cc3c(c(c(=O)oc3c2C)CC(=O)NC(C)C)C	0
CN1CCN(CC1)[C@@]2(CCCNC2)C#N	0
Cc1c(c(=O)n(n1C)c2ccccc2)NC(=O)c3ccc(c(c3)S(=O)(=O)NC(C)C)OC	0
Cc1nc(on1)c2c3c(sc2NC(=O)NCc4cccs4)COCC3	0
CC(C)(O)c1ccc(-c2cn3c(F)c(F)cc3c(=O)[nH]2)cc1	1
CC(=O)O[C@H]1CC[C@]2([C@@H]3CC[C@@]4([C@@H](CC[C@@]4([C@@H]3CC[C@@]2(C1)O)O)C5=CC(=O)OC5)C)C=O	0
CN1CCN(c2ccc(-c3nc4ccc(F)cc4c(=O)[nH]3)cc2)CC1	1
CS(=O)(=O)C1C[C@H]2CC[C@@H](C1)N2C(=O)c3ccc(cc3)NS(=O)(=O)c4ccccc4F	0
c1cc2c(cc1F)nc([nH]2)CCNC(=O)CN3c4ccc(cc4CCC3=O)S(=O)(=O)N5CCCC5	0
c1ccc(cc1)N2CCN(CC2)C(=O)/C(=C/c3cn(c4c3cccc4)CC(=O)NC[C@H]5CCCO5)/C#N	0
Cc1csc(n1)c2cc(cn2CC(=O)N3CCOCC3)S(=O)(=O)Nc4ccc(cc4)SC	0
CN(C)S(=O)(=O)c1ccc(cc1)S(=O)(=O)Nc2ccccc2C(=O)N3CCOCC3	0
CC1(CCc2c(cc(c3c2oc(=O)c4c3CCC4)OCC(=O)NCCCn5cc[nH+]c5)O1)C	0
CN(C)c1ccc(cc1)/C=c\2/c(=O)n(/c(=C(/C#N)\C(=O)NCCN3CCOCC3)/s2)Cc4ccco4	0
O=c1[nH]c(N2CCC3(CC2)COc2ccccc23)nc2c1CCCC2	1
CN(C)CCn1ccc(n1)NC(=O)[C@H]2CSCN2C(=O)c3ccc(cc3)Cl	0
C[NH+](C)c1ccc(cc1)[C@H]2[C@@H]3[C@@H](Oc4ccccc4C3=Nc5n2nc[nH+]5)c6ccc(cc6)OC	0
Cc1c2c(nc(n1)NC(C)C)n(c(=O)c(n2)Br)C3CCN(CC3)C(=O)OC(C)(C)C	0

**C1C[C@@H](NC1)[C@H]2CCCN2S(=O)(=O)C(F)(F)F**	0
**Cc1c(sc2c1c(=O)n(c(n2)SCCNS(=O)(=O)C)C3CCCC3)C**	0
**C[C@@H]1C[C@H]2[C@@H]([C@@H](C(=O)O2)C[NH+]3CCCC3)[C@@H]([C@]4([C@H]1[C@H](C[C@@H]4O)OC(=O)C)C)OC(=O)C**	0
**Cc1c(c(n(n1)C)C[C@@]2(CCCC(C2)(C)C)O)Br**	0
**C[C@H](C(=O)NC(=O)NC(C)C)Sc1nnc(n1C2CC2)C3CC3**	0
**Cc1cc(c(c(c1)C)S(=O)(=O)N2CCC[C@@H]2c3nnc(s3)C(=O)Nc4ccc(cc4)Cl)C**	0
**Br.Cc1cccc2c(=O)[nH]c(-c3ccc(CN)cc3)nc12**	1
**Cc1cccc2c1c(=O)n(C[C@H]1CC[C@H](C(=O)Nc3ccncc3)CC1)c(=O)n2Cc1cccc(C(F)(F)F)c1C#N**	1
**COc1ccc(cc1OC)S(=O)(=O)Nc2cc3c(cc2Br)OCCO3**	0
**Cc1c(nnn1c2cccc(c2)C#N)[C@H](C)NC**	0
**CC(C)Oc1ccc(-c2nnc([C@H]3C[C@H](NC(=O)c4ccccn4)C3)n2-c2ccccc2F)nc1**	1
**CCOc1ccc(-c2nnc([C@H]3C[C@H](NC(=O)c4ccnc5cccnc45)C3)n2-c2ccncc2)nc1**	1
**c1ccc(c(c1)F)S(=O)(=O)NCCC(=O)N2CCC(CC2)[NH+]3CCC(CC3)Oc4nccs4**	0
**c1cc2c(cc1c3cn4cc(ccc4n3)N)OCCO2**	0
**CC(C)[C@@H](c1ccc2c(c1)OCCO2)NC(=O)N3CCN(CC3)S(=O)(=O)c4cccc(c4)F**	0
**c1cc(ccc1NC(=O)N2CC[NH+](CC2)CCNC(=O)c3ccsc3)F**	0
**O=c1cc(-c2cccc(O)c2)oc2ccccc12**	1
**c1cc(sc1)[C@@H]2c3ccsc3CCN2S(=O)(=O)c4ccc5c(c4)[nH]c(=O)[nH]5**	0
**c1ccc2c(c1)cc(c(=O)o2)C(=O)CSc3nnc(s3)SCC(=O)[C@H]4C=C5C=CC=C[C@H]5OC4=O**	0
**c1cc(cc(c1)Cl)CNC(=O)c2cn(cn2)c3ccc(cn3)c4nc(no4)C5CC5**	0
**C[C@H]1CC2=C(C(=O)C1)[C@]3(c4cccc5c4N(C3=O)C(C[C@]5(C)c6ccccc6)(C)C)C(=C(O2)N)C#N**	0
**Cc1cc(cc([nH+]1)[C@H]2CCCN(C2)C(=O)c3c(onc3C(C)C)C)Nc4nc(cc(n4)C)C**	0
**c1ccc2c(c1)ccc(c2/C=N\Nc3c4c5c(sc4ncn3)CCCC5)O**	0
**CCCCn1c(c(c(c(c1=O)C#N)C)/C=C/2\C(=O)N(C(=S)S2)Cc3ccccc3)N4CC[NH+](CC4)C**	0
**Cc1ccc(o1)C(=O)C2=C(C(=O)N([C@]23c4ccccc4N(C3=O)CC=C)CC[NH+]5CCOCC5)[O-]**	0
**[H]/N=C/1\C(=C([C@@H](S1)C)C)c2nc3cc(ccc3n2C(C)C)F**	0
**c1cc(c(cc1NC(=O)c2ccc(s2)Cl)[O-])Cl**	0
**CC(C)(C)C(=O)N1CCN(CC1)C(=O)Nc2cccc(c2F)F**	0
**CCn1c(nnc1S[C@@H](C(C)C)C(=O)NC(=O)N)CSc2nc3ccccc3s2**	0
**c1ccc2c(c1)[C@H](CO2)Cn3cnc4c3ncnc4N**	0
**COCCNC(=O)[C@H](c1ccc[nH+]c1)N(Cc2ccccc2Cl)C(=O)Cn3c4ccccc4nn3**	0
**Cc1csc(n1)[C@H](C)NC(=O)N2CC[C@](C2)(C)C(=O)[O-]**	0
**CC(C)c1csc(n1)NC(=O)c2ccc(cc2Cl)[N+](=O)[O-]**	0
**c1cc2c(cc1Cl)nc([nH]2)c3cc(cnc3)N**	0
**C[C@@H](c1cc[nH]n1)Nc2ccc(c(c2)F)F**	0

**CC(C)N(Cc1nc2c(c(=O)[nH]1)COCC2)C(=O)CCc1ccccc1**	1
**c1cc(c(cc1Cl)Cl)OCc2nc([nH]n2)CC#N**	0
**c1ccc(cc1)[C@H]2CN(CC[NH2+]2)c3cccc(n3)C(=O)N4CCN(CC4)c5cccc[nH+]5**	0
**Cc1cc(c(cc1OC)S(=O)(=O)N2CCN(CC2)c3cc(nc(n3)C)N4CCCCC4)C**	0
**CC[C@@H](C#N)Nc1c(cc(cc1Cl)Br)Cl**	0
**COc1cccc2c1c(=O)n(C[C@H]1CC[C@H](C(=O)O)CC1)c(=O)n2Cc1cccc(C(F)(F)F)c1C#N**	1
**CCc1nnc(s1)NC(=O)CSc2nnc(n2C)CCc3[nH]c4ccccc4[nH+]3**	0
**Cc1cccc(c1C)NC(=O)c2ccc3c(c2)nns3**	0
**c1cc2c(cccn2)c(c1)N3CCNC4(C3)CCCC4**	0
**CC(=O)N1CCCc2c1ccc(c2)S(=O)(=O)Nc3ccccc3c4nnc5n4CCCCC5**	0
**Cc1ccc(o1)[C@H]2C(=C(c3ccc(cc3)S(=O)(=O)N(C)C)O)C(=O)C(=O)N2Cc4ccc[nH+]c4**	0
**Cc1ccnc2n1nc(n2)C(=O)Nc3nc(cs3)c4ccc(cc4Cl)Cl**	0
**Cc1c(nc(nc1Cl)[C@@H]2CCC[C@H](C2)C)C**	0
**c1ccc2c(c1)c(cs2)C(=O)N[C@H]3CCC[C@H]3C(=O)[O-]**	0
**CCOc1ccc(-c2nnc(C34CC(NC(=O)c5ccnc6cccnc56)(C3)C4)n2-c2ccccn2)nc1**	1
**Cc1cc(ccc1C(C)C)OCC(=O)N2CCc3c(sc(c3C#N)N)C2**	0
**Cc1ccc(cc1N2CCN(CC2)S(=O)(=O)c3ccc4c(c3)CCC(=O)N4)Cl**	0
**CC(C)C(=O)N1CCC(CC1)NC(=O)C2CCN(CC2)S(=O)(=O)c3cc(n(c3)C)C(=O)N**	0
**Cc1c(c[nH]n1)S(=O)(=O)[N-]c2ccc([nH+]c2)N(C)C**	0
**CCn1c2ccccc2nc1CNC(=O)c3cc(nc4c3c(no4)C)c5cc(oc5C)C**	0
**Cc1cc(c(c(c1)c2nc3cc(ccc3o2)Cl)N)C**	0
**c1ccc(cc1)C[C@@H](C(=O)N2CCN(CC2)c3cccc[nH+]3)NS(=O)(=O)c4cccc5c4nsn5**	0
**Cc1cc(c(cc1Cl)Cl)S(=O)(=O)N2CCCN(CC2)S(=O)(=O)c3c[nH]cn3**	0
**O=C(c1ccc(F)cc1)C1CCN([C@@H]2CCN(Cc3nc4c(c(=O)[nH]3)COCC4)C2=O)CC1**	1
**c1ccc2c(c1)c(cn2CC(=O)Nc3ccc4c(c3)OCO4)/C=C/5\C(=O)N(C(=S)S5)CC(=O)[O-]**	0
**Cc1cc2c(cc1C)n(cn2)c3c(cnc(n3)Cl)F**	0
**CC(=O)c1ccc(cc1)NS(=O)(=O)c2cccc(c2)S(=O)(=O)[C@@H]3CCS(=O)(=O)C3**	0
**C1CCCC(CCC1)C(=O)N[C@H]2CS(=O)(=O)C[C@H]2[NH+]3CCCC3**	0
**C[C@@H]1CN2CCCC[C@@H]2CN1Cc3c(ccc(n3)N)Cl**	0
**Cc1ccc(cc1NS(=O)(=O)C)C(=O)N[C@@H](c2ccccc2F)c3[nH+]ccn3C**	0
**C[C@H]1CC[C@@H](N(C1)[C@@H]2CCC[C@@H]2C#N)C**	0
**C[C@@H](c1cc(=O)[nH]c(n1)N2CCCC2)NS(=O)(=O)c3ccc4c(c3)OCCO4**	0
**O=[N+]([O-])c1ccc(-c2nc(O)c3cccc(O)c3n2)cc1**	1
**COc1cc(c(cc1OC)[N+](=O)[O-])C(=O)N2CCc3c(sc(n3)NC(=O)c4cccs4)C2**	0
**CC(C)(O)c1ccc(-c2nc3ccc(F)cc3c(=O)[nH]2)nc1**	1
**CCc1cccc(c1NC(=O)c2c3n(cn2)C[C@@](N(C3=O)C4CC4)(C)C(**	0

=O)NCc5ccc(cc5)F)C	
C[C@@H]1CC[C@@H](N(C1)C(=O)N[C@@H](C)CC(=O)[O-])C	0
C[C@@]1(C(=O)N(C(=O)N1)C[C@H](COc2ccc(cc2)Br)O)c3ccco3	0
c1ccc(c(c1)n2c3ccc(cc3nc2N)Br)Cl	0
Cc1ccn(n1)c2cc(c(cc2C)N)Cl	0
c1cc(cc(c1)Cl)[C@H]2C(=C(C(=O)N2CC[NH+]3CCCCC3)[O-])C(=O)c4ccco4	0
O=C(CC1CCOCC1)Nc1nnc2n1CCS2	1
CC1CCC(CC1)(CN[C@H]2CCCC[C@H]2C#N)O	0
Cn1c(nnc1SCc2ccc(cc2)Br)CNS(=O)(=O)c3ccc(cc3)Br	0
c1ccc2c(c1)CN(CC(=O)N3CC[C@H]([C@H](C3)CCCO2)CC(=O)N4CCOCC4)C(=O)C5CCCC5	0
Cc1c(sc(n1)CN2CC3(CCCCC3)NCC2(C)C)C	0
Cc1ccc(c(c1)/C(=C\2/[C@@H](N(C(=O)C2=O)c3nnc(s3)C)c4ccc(cc4)O)/O)C	0
c1cc2c(cc1N)n(cn2)c3cc(cnc3)Br	0
c1cc(c(cc1Cl)Cl)C(=O)Nc2ccc(nc2)n3cc[nH+]c3	0
CCCCc1ccc(cc1)NC(=O)Cn2c(=O)c3c(c(n2)c4cccs4)sc(n3)N5CCOCC5	0
C[C@@H](C(=O)Nc1ccnn1C(C)C)Sc2ccc(cn2)S(=O)(=O)N3CCCC3	0
C[C@@H](c1ccc(F)cc1)N(Cc1nc2c(c(=O)[nH]1)COCC2)C(=O)CN1CCC(C(=O)c2ccc(F)cc2)CC1	1
O=C1SCCc2nc(-c3ccc(C(F)(F)F)cc3)[nH]c(=O)c21	1
CC1=C(C[C@H]([C@H](C1)C(=O)Nc2c(c3c(s2)CCCC3)C(=O)N4CCCCC4)C(=O)O)C	0
c1ccc2c(c1)C[C@@H]2c3nc(on3)c4cc(cnc4)O	0
Cc1c(nc(nc1Cl)c2cc(cs2)Cl)C	0
CC[C@H](C)NS(=O)(=O)c1ccc(cc1)C(=O)N(C)Cc2cnn(c2)c3ccccc3	0
Cc1[nH+]ccn1c2ccc(nn2)N3CCN(CC3)S(=O)(=O)c4cc(ccc4Cl)Cl	0
Cc1c(c(=O)n(n1C)c2ccccc2)NC(=O)[C@@H]3CC(=O)N(C3)c4cccc5c4cccc5	0
O=C(c1ccccc1)C1CCN(CC(=O)N(Cc2nc3c(c(=O)[nH]2)COCC3)CC(F)F)CC1	1
C[C@@H]1c2c(c3ccccc3[nH]2)[C@H](CN1[C@@H]4CC(=O)N(C4=O)c5cccc(c5)[N+](=O)[O-])c6ccccc6	0
c1cc(sc1S(=O)(=O)N2CC[NH2+]CC23CCCCC3)Cl	0
c1cc(cc(c1)[N+](=O)[O-])C[C@@H](Cc2ccc(cc2)Br)[NH3+]	0
CN(C)S(=O)(=O)c1ccc(cc1)N2C[C@@H](CC2=O)C(=O)NCCC3=c4ccccc4=[NH+]C3	0
Cc1c(c(n(n1)Cc2cc(ccc2OC)/C=N\NC(=O)c3cc[nH]n3)C)Br	0
C[C@H]1CN(C[C@@H](N1)C)[C@@H]2CCCCC[C@@H]2O	0
[H]/N=C\1/C(=C/c2cc(ccc2OC)Br)/C(=O)N=C3N1N=C(S3)c4ccccc4	0
[H]/N=c\1/c(cc2c(n1CCc3ccc(c(c3)OC)OC)nc4c(cccn4c2=O)C)S(=O)(=O)c5ccccc5	0
Cc1c(sc(c1C#N)NC(=O)[C@@]2(CC[NH2+]C2)C)C	0
Cc1ccc2c(c1)n(c(=O)c3n2nc(n3)C(=O)NCc4ccc(o4)C)Cc5cccc(c5)Cl	0

Cc1c(sc(n1)c2cc3ccccc3oc2=O)C(=O)[O-]	0
Cc1c(cc(n1C)C#N)CN2CCc3c(c4cc(ccc4[nH]3)Cl)C2	0
Cn1c2ccc(cc2nc1CCC(=O)Nc3cccc(c3)C#C)S(=O)(=O)N4CCOCC4	0
c1ccn2c(c1)[nH+]c3c(c2=O)cc(s3)C(=O)NCCN4C(=O)/C(=C\c5cccnc5)/SC4=O	0
CCOC(=O)C1=C(N=c2n(c(=O)/c(=C\c3ccc(c(c3)OC)OC(=O)C)/s2)[C@H]1c4ccc(c(c4)OC)OC(=O)C)C	0
CN(c1cccc(c1)NC(=O)CN(c2ccc3c(c2)OCCO3)S(=O)(=O)C)S(=O)(=O)C	0
c1cc(ccc1N2CCCC2=O)S(=O)(=O)N3[C@@H]4CCCC[C@@H]4C[C@@H]3C(=O)N	0
Cn1c(nnc1SCC(=O)CC(=O)Nc2ccccc2)Cn3c[nH+]c4c3cccc4	0
CCCCN1[C@H]([C@@H]2C(=NN=C2C1=O)c3cc(cc(c3[O-])C)C)c4ccc(c(c4)OCC)O	0
C/C(=N/O)/c1ccccc1O[C@@H]2CCO[C@@]3(C2)CCOC3	0
COc1cccc2c(=O)[nH]c(-c3ccc(Br)cc3)nc12	1
C[C@H]1C[C@@H](CC(C1)Nc2c(cc(cn2)N)Br)C	0
c1c[nH+]c([nH]1)CN2C[C@H](CN(C(=O)C2)C3CCCCC3)O	0
c1cc(nc(c1)F)Nc2cc(ccc2C#N)Cl	0
C[C@@]1(CC[C@](C1(C)C)(C)C(=O)O)C(=O)N2CCCCCC2	0
C[C@H]1CCCN1c2nc3c(s2)CCC[C@@H]3O	0
Cc1ccccc1S(=O)(=O)[N-][C@H]2C(=C(C(=O)O2)Oc3ccccc3)Br	0
N#Cc1ccc2c(c1)oc(=O)n2[C@H]1C[C@H](c2nnc(-c3ccncn3)n2-c2ccccc2Cl)C1	1
Oc1nc(-c2ccccc2)nc2ccccc12	1
Cc1ccc(c(c1)CC(=O)N2CCC[C@H](C2)/C(=N/O)/N)C	0
C[C@@H]1CCCC(=O)CCC/C=C/c2cc3c(c(c2C(=O)O1)O)[C@H](CC(=O)O3)c4cc(cc(c4)OC)OC	0
C[C@@H]1CC[C@H]([C@@](C1)([C@@H]2CCO[C@@]3(C2)CCOC3)O)C(C)C	0
CC[NH+]1CCN(CC1)C(=O)CC2CCN(CC2)C(=O)NC3CCC(CC3)C	0
c1cc(c(cc1Br)[O-])C(=O)Nc2cc(c(cc2F)F)F	0
Cc1cc(no1)C(=O)Nc2nc(cs2)c3ccc(cc3F)NC(=O)C	0
O=C(c1ccc(F)cc1)C1CCN(CC(=O)N(Cc2cccc(F)c2)Cc2nc3c(c(=O)[nH]2)COCC3)CC1	1
CNC(=O)CN(C)C(=O)Cc1cn(nc1c2ccc(cc2)Cl)c3ccccc3	0
O=c1[nH]c(-c2ccccc2)cc2ccncc12	1
CCC[C@@H](C)NC(=O)[C@H]1CCCN(C1)C(=O)C(=O)Nc2cccc3c2cc[nH+]c3	0
Cc1c(c(n(n1)[C@H]2CCO[C@@H](C2)C(C)C)N)Br	0
Cn1c(=O)c(c(n(c1=O)Cc2ccccc2)N)C(=O)CSc3ccc(cn3)S(=O)(=O)N(C)C	0
C[C@@H]1CCCCC[C@H]1n2c3cc(c(cc3[nH]c2=S)F)OC	0
CCc1nc(sn1)N2C[C@H](N[C@H](C2)C)C	0
Cc1ccc(c(c1)NC(=O)[C@H]2c3cc(c(cc3C(=O)N([C@@H]2c4ccc(c(c4)OC)OC)C)OC)OC)OC	0
Cc1ccc(c(c1)C)NC(=O)[C@H]2CC(=O)[N+](=C(S2)Nc3ccc(cc3)OC)CCc4ccc(cc4)OC	0
c1cc(ccc1NC2=NN=C(CS2)c3ccc4c(c3)OCCCO4)S(=O)(=O)N5CCOCC5	0

CC(=O)N[C@H]1CN([C@@H]2CO[C@@H](O[C@H]2[C@@H]1O)c3ccccc3)C(=O)OC(C)(C)C	0
C[C@@H]1[C@H](CC[C@]2([C@H]1[C@H]([C@@H](CC2)[C@H](C)C(=O)NC3CCCCC3)O)C)NC(=O)c4cncnc4	0
c1ccc(cc1)N2[C@@H](NN=C2c3cccc(c3)S(=O)(=O)N4CCCC4)SCC(=O)C5=NC=CC5	0
CC(C)[C@H](C#N)NC(=O)c1ccc(cc1O)Br	0
Cc1cc2nc(c(c(n2n1)C)CCC(=O)Nc3cccc(c3C)C(=O)N4CCOCC4)C	0
COc1ccc(-n2c(SCc3nc(-c4ccccc4)no3)nnc2-c2ccncc2)cc1	1
CC1CCN(CC1)c2c(c(ncn2)NC34CC5CC(C3)CC(C5)C4)[N+](=O)[O-]	0
CN1CCCc2c1nc(N1CCC3(CC1)C(=O)N(C)c1cc(OCCO)cc(F)c13)[nH]c2=O	1
Cn1cc[nH+]c1[C@H](c2ccc(cc2)F)NC(=O)c3cccc(c3)S(=O)(=O)N4CCCC4	0
Cc1cc(n(n1)[C@@H]2CCS(=O)(=O)C2)NC(=O)/C=C/c3cn(nc3c4ccccc4)c5ccccc5	0
CN1[C@H]2[C@H](C(=O)N=C1[O-])N(C(=[NH+]2)N(C)Cc3ccccc3)CCSC4[NH+]=c5ccccc5=[NH+]4	0
C[C@H]1C[C@H](CC(C1)(C)C)OC[C@@H](C[NH+]2CCN(CC2)CCN3C(=O)c4cccc5c4c(ccc5)C3=O)O	0
Cc1ccc(o1)c2c(c(n[nH]2)NC(=O)[C@@H]3CCn4c[nH+]cc4C3)C	0
Cc1c(c(n(n1)C)Cl)C[NH+]2C[C@H]([C@]3(C2)Cc4ccccc4C(=O)N3)C(=O)NC(C)(C)C	0
CNc1c(cccn1)C[NH+]2CCC[C@H](C2)c3cncc(n3)c4cccc(c4)Cl	0
Cc1c2c(cc(nc2on1)c3ccccc3)C(=O)N[C@@H]4CC[C@@H](C4)[NH+](C)C	0
Cc1cc(cc(c1)C(=O)Nc2ccc(cc2)c3ccc(=O)n(n3)C)C	0
c1cc(c(cc1Cl)Cl)COc2c(cc(cc2[N+](=O)[O-])Br)/C=N/n3cnnc3	0
c1ccc2=[NH+]CC(=c2c1)CCN3[C@H](Nc4c(nccn4)C3=O)SCc5ccc(cc5)F	0
Cc1ccc2c(c1)nc([nH]2)Sc3cccc(n3)C#N	0
C[C@H]1C[C@@H](CC(C1)(C)C)[NH+]2[C@@H]3CCCC[C@@H]3C[C@@H]2C(=O)[O-]	0
Cc1ccc2cc(ccc2n1)[C@H]3C[NH+]=C(N3C(C)C)N	0
COC(=O)[C@@H]1CS[C@H]2CCCCCCCCCC[C@@H]2N1	0
c1ccc2c(c1)nc(n2/N=C\c3cc(ccc3[O-])Br)CO	0
CN1C(=[NH2+])[C@H]([NH+]=C1C2CCCC2)c3cncn3C4CC4	0
c1cc(c[nH+]c1)/C=C\2/C(=O)N(C(=O)S2)CCNC(=O)CCNS(=O)(=O)c3ccc4c(c3)OCCO4	0
CCn1cnnc1CNC(=O)c2cnn(c2C3CC3)c4cccc(c4)C(F)(F)F	0
Cc1c(nnn1Cc2csc(n2)c3ccccn3)N	0
CC1=C([C@H](C2=C(N1)CCCC2=O)c3cc(c(c(c3)Cl)O)OC)C(=O)OC(C)C	0
Cc1cccc(c1)N([C@H](c2cc[nH+]cc2)C(=O)NC(C)(C)C)C(=O)Cn3nc(nn3)c4ccccc4F	0
c1ccc(c(c1)/N=C/c2cccnc2)Cl	0
CC#CCOc1ccc(C(=O)C2CCN(CC(=O)N(Cc3nc4c(c(=O)[nH]3)COCC4)CC3CC3)CC2)cc1	1
Cc1c(ncnc1N)c2ccc(cc2)Br	0
C1C[C@H](CN(C1)C(=O)N[C@H]2CC[NH+](C2)C3CC3)CC(=	0

O)[O-]	
CCc1cccc2c1nc(s2)N(CC[NH+](C)C)C(=O)[C@@H]3CCCN(C3)S(=O)(=O)c4cccs4	0
CN1C[C@@H](C(=O)N(C1=O)C)S(=O)(=O)NC[C@H](c2ccccc2Cl)C3=c4ccccc4=[NH+]C3	0
[H]/N=C\1/C(=C/c2ccc(cc2)OCCOc3ccccc3Cl)/C(=O)N=C4N1N=C(S4)C(F)(F)F	0
O=C1N(CCO)c2cc(Cl)cc(Cl)c2C12CCN(c1nc3c(c(=O)[nH]1)CCCC3)CC2	1
Cc1c(c(on1)/C=C/c2ccc(cc2F)F)S(=O)(=O)N3CCC(CC3)C(=O)NCc4cccnc4	0
CN(CC(=O)Nc1cccc2c1cccc2)C(=O)c3cc(c[nH]3)S(=O)(=O)N4CCOCC4	0
C[C@@H]1C[C@H](CN(C1)C(=O)C(=O)Nc2c3c(nn2c4ccc(cc4)Cl)CS(=O)(=O)C3)C	0
CCOC(=O)c1c(nc(s1)NC(=O)CSc2[nH+]c3c(n2C)c(=O)n(c(=O)n3C)C)C	0
CC(C)(C(=O)NCCS(=O)(=O)N1CCN(CC1)c2cccc[nH+]2)Oc3ccc(cc3)Cl	0
C[C@@H](C(=O)N1CC[NH+](CC1)[C@@H](c2ccccc2Cl)C(=O)OC)NC(=O)c3ccco3	0
CCc1c(nnn1[C@@H]2CCO[C@]3(C2)CCSC3)N	0
c1ccc(cc1)CNc2ncc3c(n2)[C@H]4CN(C[C@H]4S(=O)(=O)C3)Cc5ccc[nH+]c5	0
Cc1ccc(cc1)S(=O)(=O)NCc2cc(n(n2)C)c3ccccn3	0
CC(C)(C)c1cc(cc(c1O)C(C)(C)C)/C=N/NC(=O)CN2CCN(CC2)S(=O)(=O)c3ccc(cc3)Br	0
Cc1nc2cc(ccc2s1)NC(=O)c3cc(n(n3)[C@H]4CCS(=O)(=O)C4)c5ccco5	0
Cc1ccc(c(c1)Br)n2c(c(c3c2nc(nc3N)C)C)C	0
c1cc(c(cc1N[C@@H](C2CCCC2)C(=O)[O-])Br)Cl	0
Cc1ccc(nc1)NS(=O)(=O)c2ccc(c(c2)N)Cl	0
c1cc(sc1)[C@@H]2C[C@@H](Nc3n2ncn3)c4ccc5c(c4)OCO5	0
C[C@@H]([C@H](C)O)C1(CCCCCC1)C(=O)O	0
COc1ccc(C(=O)C2CCN(C3CCN(CC(=O)Nc4nnc5ccccn45)C3=O)CC2)cc1	1
O=C(N[C@H]1C[C@H](c2nnc(-c3ccccn3)n2-c2ccccc2F)C1)c1ccnc2cc(F)cnc12	1
N#Cc1ccc2c(c1)[nH]c(=O)n2[C@H]1CC[C@H](c2nnc(-c3ccncn3)n2-c2ccccc2Cl)CC1	1
Cc1cc2c([nH]c(n2)[C@@H]3CC[C@@H](O3)C)nc1	0
c1cc(nc(c1)Nc2cccc(c2F)Cl)C#N	0
CC1(C)CCC2=C(C1)c1n[nH]c(=O)c3cccc(c13)N2	1
Cc1ccc(c(c1)S(=O)(=O)N)O[C@@H]2CC[C@@H]([C@@H](C2)C)C	0
CC/C=c/1\[nH]n-2c(=[NH2+])/c(=C/c3ccc(c(c3)OC)OS(=O)(=O)c4ccccc4)/c(=O)nc2s1	0
CC(=O)OCC(=O)[C@]1(CC[C@@H]2[C@@]1(CC(=O)[C@@H]3[C@@H]2CC=C4[C@@]3(CCC5(C4)OCCO5)C)C)O	0
C[C@@H](C(=O)NN1CC[NH+](CC1)C)Sc2nc(cc(n2)C(F)(F)F)c3ccccc3	0

O=C(c1ccc(Cl)cc1)C1CCN(CC(=O)N(Cc2nc3c(c(=O)[nH]2)COCC3)CC(F)F)CC1	1
Cc1cc(c(c(c1)S(=O)(=O)Nc2ccc3c(c2)n(c(=O)n3C)C)C)C	0
Cc1c(c(on1)C)[C@H](C)NC(=O)N[C@H]2CCC[C@@H]2CCC(C)C	0
Cc1cc(c2ccc(cc2n1)F)C(=O)Nc3ccc4c(c3)oc(n4)C	0
Cc1c2c(c3c4c(c(ncn4)NCc5ccccc5)oc3n1)CC(OC2)(C)C	0
c1cc(sc1)S(=O)(=O)N2CCCC[C@H]2C(=O)Nc3nnc(o3)c4ccc5c(c4)CCCC5	0
c1cc(ccc1F)S(=O)(=O)[N-]c2ccc3c(c2)[nH]c(=O)o3	0
C[C@H]1CN(C[C@@H](O1)C)S(=O)(=O)c2ccc(cc2)C(=O)Nc3c(c4c(s3)C[NH+](CC4)Cc5ccccc5)C(=O)OC	0
CN1CCCc2c1nc(N1CCC3(CC1)C(=O)N(C)c1cc(OCCN4CCCCC4)cc(F)c13)[nH]c2=O	1
COc1ncc(cn1)c2c(c(nc3c2SCCSC3)N)C#N	0
c1ccc2c(c1)CC[C@@H]2Nc3ccccc3C#N	0
O=c1cc(-c2ccc([N+](=O)[O-])cc2)oc2ccccc12	1
C[C@H](C(=O)Nc1cccc(c1)S(=O)(=O)N)N2CCN(CC2)S(=O)(=O)c3cccs3	0
c1ccc2c(c1)c(cn2CCC#N)c3cn4cccc(c4n3)O	0
c1ccc2=[NH+]CC(=c2c1)C3=NN[C@@H](N3C[C@@H]4CCCO4)SCC(=O)N[C@@H]5CCS(=O)(=O)C5	0
COC(=O)NC(=O)c1c2c(sc1NC(=O)[C@@H]3CCCCN3S(=O)(=O)c4cccs4)CCCC2	0
C[C@H](c1[nH]c2c(n1)ccc(n2)OC)Cl	0
C[C@]1(C(=O)N(C(=O)N1)CC(=O)N([C@@H]2CCS(=O)(=O)C2)C3CC3)c4ccccc4Cl	0
Cc1cccc(c1C)[C@H](Cc2c(c(nn2C)C)Br)N[NH3+]	0
Cc1cc(ccc1Br)[N-]S(=O)(=O)c2ccc(cc2C)N3C(=O)CCS3(=O)=O	0
C[C@H]1CN(C[C@@H]1C)C(=O)c2ccc3c(c2)cc[nH]3	0
CN(c1ccccc1)c2ccc(cc2C#N)N	0
CO[C@@H]1C[C@@H](NC1)c2[nH]c3cc(c(cc3n2)Br)F	0
C[C@@H]1C[NH+](Cc2cc(ccc2O1)Cl)CCC(=O)NCc3ccc4c(c3)ccc[nH+]4	0
CNC(=O)c1c(cn(n1)C)NC(=O)Cn2c(c(c(n2)C(F)(F)F)Br)C3CC3	0
Cc1cc(nc(c1C#N)N[C@@H]2CCC[C@@H](C2)C)C	0
COc1cc2ncnc(N3CCC(CN4Cc5ccccc5NC4=O)CC3)c2cc1OC	1
COc1cc2c(cc1F)nc(n2[C@@H]3C[C@H]4CC[C@@H]3C4)N	0
c1cc(cc(c1)F)n2cc(cn2)CN[C@H]3C[C@H]4C(=O)N[C@H](C(=O)N4C3)Cc5ccc(cc5)O	0
Cc1c2c(cc(nc2on1)C3CC3)C(=O)Nc4ccc5c(c4)OCCO5	0
Cc1cccc([nH+]1)CNC(=O)[C@H]2CC23CCN(CC3)C(=O)c4cc(no4)c5ccccc5	0
CC(C)(C=C)[C@@]12CC(=O)C(=O)N\3[C@]1(NC(=O)/C3=C\c4c[nH+]c[nH]4)N(c5c2cccc5)OC	0
Cc1ccc(cc1C(=O)NC[C@@H](c2cccc(c2)OC)[NH+]3CCCC3)S(=O)(=O)NC(C)(C)C	0
COc1cccc2c(O)nc(-c3ccc(N)cc3)nc12	1
c1ccc(cc1)S(=O)(=O)CCc2nnc(o2)NC(=O)CCCS(=O)(=O)c3ccc(cc3)Cl	0
c1ccc(cc1)/N=C(/C(=C\2/c3ccccc3C(=[NH+]2)N)/C#N)\[O-]	0
c1cc(ccc1NC(=O)Nc2nc(cs2)c3c(ccs3)Br)Cl	0

**CC(=O)N[C@@H](CC(=O)N1CCN(CC1)[C@@H](C#N)c2ccc(cc2)OC)c3ccccc3**	0
**Cc1cccc(c1)[N-]S(=O)(=O)c2ccc(c(c2)OC)N/C=C\3/C(=NN(C3=O)c4ccccc4)C**	0
**CC(C)n1cnc(n1)[C@H]2CN(c3c2cc(cc3)C(=O)NCCc4cccc(c4)F)S(=O)(=O)C**	0
**CN1CCCc2c1nc(N1CCC3(CC1)C(=O)N(CC#N)c1cc(F)cc(F)c13)[nH]c2=O**	1
**C[C@H]1C[C@@H](CN(C1)S(=O)(=O)N2CCC[C@@H](C2)C(=O)NCCC[NH+]3CCN(CC3)C)C**	0
**CC(=O)c1ccc(cc1)NC(=O)N2CCCN(CC2)C(=O)c3cccc(c3)S(=O)(=O)C**	0
**c1ccc(cc1)C(=O)COc2ccc(cc2)NC(=O)[C@H]3[C@H]4C[C@H]5[C@H]3C(=O)O[C@H]5C4**	0
**Cc1nc2c(c3c(s2)CCCC3)c4n1c(nn4)SCC(=O)Nc5ccc6c(c5)OCCO6**	0
**Cc1cc(c(c2c1CC[C@H]3[C@H]2OC(=O)[C@H]3C)C)OC(=O)C**	0
**C[C@@H](C(=O)Nc1ccc(cc1Cl)[N+](=O)[O-])N2CCN(CC2)S(=O)(=O)c3ccc4c(c3)OCCO4**	0
**O=C(N[C@H]1C[C@H](c2nnc(-c3ccc(OC(F)F)cn3)n2-c2ccccc2F)C1)c1cc(F)cc2nccnc12**	1
**Cc1ccc(nc1)NC(=O)c2ccc(c(c2[O-])C)C**	0
**c1c(c([nH+]n1[C@@H]2CCS(=O)(=O)C2)C3CCCCC3)C(=O)[O-]**	0
**Cc1cccn2c1nc(c2C)C(=O)C3=C(C(=O)N([C@H]3c4ccc(cc4)F)CC[NH+]5CCOCC5)[O-]**	0
**CCOc1cc(cc(c1OCC)I)/C=C(/C#N)\c2[n-]c3cc(ccc3n2)OC**	0
**Cn1c2c(c(n1)C(=O)NCCCN3CCOCC3)CN(CC2)C(=O)[C@H]4C=c5ccccc5=[NH+]4**	0
**Cc1c2cnn(c(=O)c2c(n1[C@H](C)C(=O)NCCCN3CCN(CC3)C)C)c4ccccc4**	0
**CC(C)C(=O)Nc1ccc(C2NC(=O)c3cccnc3N2C)cc1**	1
**c1ccc(cc1)c2c(nnn2c3ccccc3)C(=O)/N=c\4/c(nn(s4)c5ccc(cc5)Cl)C(=O)N6CCCC6**	0
**Cc1c\2c(oc1C(=O)N[C@H](C)COC)CCC/C2=N\NC(=O)c3ccc(cc3)Cl**	0
**Cc1cc(c(n1c2cccc(c2)F)C)/C=C\3/C(=[NH2+])N4C(=NC3=O)SN=C4S(=O)(=O)CC(C)C**	0
**COc1ccc(C(=O)C2CCN([C@H]3CCN(Cc4nc5c(c(=O)[nH]4)COCC5)C3=O)CC2)cc1C**	1
**COc1ccc2c(c1)C3=Nc4[nH+]cnn4[C@H]([C@@H]3[C@@H](O2)c5ccc(c(c5)OC)OC)c6ccc(cc6)F**	0
**Cn1c2ccc(cc2oc1=O)S(=O)(=O)NCc3cccc(c3)Cn4ccnc4**	0
**C=C[C@H](CC(=O)NCc1[nH]c(c[nH+]1)c2ccccc2)c3ccccc3**	0
**Cc1cc(c(cc1[N-]S(=O)(=O)c2ccc(c(c2)C(F)(F)F)Cl)[N+](=O)[O-])C**	0
**CNc1c(cc(c[nH+]1)S(=O)(=O)Nc2ccc(c(c2)Cl)Cl)Cl**	0
**Cc1nnnn1[C@H](Cc2cccc(c2)F)C(=O)NCc3ccccc3c4ccc(cc4)Cn5cc[nH+]c5**	0
**Cc1c(sc(n1)N)c2cc(c(c(n2)N)C#N)[C@H]3CCC=CC3**	0
**CC(C)c1ccc(cc1)[C@H]2C(=C(C(=O)N2CC[NH+]3CCOCC3)[O-])C(=O)c4ccc5c(c4)OCCO5**	0

**Cc1ccc(cc1)S(=O)(=O)N[C@@]2(C(=O)N(C(=O)N2)Cc3ccccc3)C(F)(F)F**	0
**c1ccc2c(c1)[C@H](CCO2)Nc3c(nsn3)Cl**	0
**Cc1cc(cc(c1)Cl)c2nnc(s2)CN**	0
**CC(C)C(=O)NCCNC(=O)N[C@H](C[NH+](C)C)c1ccccc1**	0
**C[C@H](C(=O)NC(C)C)NC(=O)[C@H]1CCCN(C1)S(=O)(=O)c2cccc3c2nsn3**	0
**Cc1ccccc1n2c(nnn2)SCC(=O)NCCN3C(=O)/C(=C/c4ccc[nH+]c4)/SC3=O**	0
**c1ccc(cc1)n2nc(c(n2)NC(=O)Cn3nc(nn3)c4ccccc4F)C(=O)N**	0
**CCn1c(nnc1SCC(=O)Nc2nc(cs2)c3ccccc3)[C@H](C)NC(=O)c4ccc(cc4Cl)Cl**	0
**Cc1cc(ncc1Br)N2CCC(CC2)[C@H](C)O**	0
**Cc1ccc2c(c1)c(cc(n2)C)C(=O)NC[C@@H]3Cc4cc(ccc4O3)c5ccc(nn5)OC**	0
**COc1ccccc1C(=O)C1CCN(CC(=O)N(Cc2nc3c(c(=O)[nH]2)COCC3)CC(F)F)CC1**	1
**Cc1c(c(n([nH+]1)[C@H]2CCS(=O)(=O)C2)C)CN(C)Cn3c(=S)sc(n3)Nc4ccc(cc4)F**	0
**CC(C)c1nc(nc(n1)Cl)N(C)C**	0
**COc1ccc2c(c1)CC1(CCN([C@@H]3CCN(Cc4nc5c(c(=O)[nH]4)COCC5)C3=O)CC1)C2=O**	1
**Cc1ccc(cc1)c2ccc(=O)n(n2)[C@H](C)C(=O)NCCOc3cc(ncn3)N4CC[NH+](CC4)C**	0
**Cc1c(c(on1)C)[C@H](C)/[NH+]=C/c2cc(cc(c2[O-])Cl)Cl**	0
**C[C@H]1C[NH+](CC[C@@H]1N(C)C(=O)NC[C@@H]2CCO[C@@H]2C(C)C)C**	0
**O=c1[nH]c2ccccc2n1[C@H]1CC[C@H](c2nnc(-c3ccncn3)n2-c2ccccc2Cl)CC1**	1
**CCc1ccc(-n2c(C)nnc2SCc2nc(-c3ccc(OC)cc3)no2)cc1**	1
**Cc1cc(nc(n1)Sc2cc(nc(n2)Cl)C)C**	0
**COCCN(Cc1nc2c(c(=O)[nH]1)COCC2)C(=O)CN1CCC(c2nnc(-c3ccccc3)o2)CC1**	1
**C[C@@H]1[C@@H](OCCN1C(=O)N[C@@H](c2ccc(cc2)OC)C3CCOCC3)C**	0
**COc1cc2c(cc1OC)nc(nc2N3CCCC3)C[NH+](CCO)[C@H]4CS(=O)(=O)C[C@@H]4O**	0
**CN[C@@H]1COC[C@H]1C(=O)Nc2nc3c(s2)CCC3**	0
**CN(C)S(=O)(=O)c1ccc(cc1)C(=C2[C@H](N(C(=O)C2=O)Cc3ccc[nH+]c3)c4ccc(cc4)Cl)O**	0
**c1cc(ccc1N2CCN(CC2)C(=O)[C@@H]3N[C@@H](NO3)C4=CC5=CC=N[C@@H]5C=C4)Cl**	0
**Cc1ccc2c(=O)[nH]c(-c3ccc([C@](C)(O)CO)cc3)cn12**	1
**c1ccc(cc1)C[NH+]2CCc3c(c(nc(n3)Nc4cc(cc(c4)Cl)Cl)OCC(=O)[O-])C2**	0
**Cc1cc(c(cc1Cl)OC)NS(=O)(=O)c2ccc(cc2)N3C(=O)[C@H](CS3(=O)=O)C**	0
**C[C@H](C(=O)Nc1cccc(c1)S(=O)(=O)N)Sc2ccc(cn2)S(=O)(=O)N3CCCC3**	0
**Cc1c2c(nc3n(c2=O)CCCCC3)sc1C(=O)NCc4ccc(nc4)Cl**	0
**COc1ccccc1N1CCN(C2=NC3CCCCCC3C(=O)N2)CC1**	1
**CCOC(=O)c1c2csc(c2c(=O)n(n1)c3ccc(cc3)OC(F)(F)F)NC(=O)C**	0

Cc1c(cccc1[C@H](C)c2c[nH]cn2)CO	0
CCc1ccc(cc1)c2cc(c3c(nn(c3n2)[C@@H]4CCS(=O)(=O)C4)C)C(=O)NCc5cccc[nH+]5	0
CC1(Cc2c(ccn2c3ccc(c(c3)Cl)C#N)[C@@H](C1)O)C	0
c1nnc2n1CCN(C2)C(=O)[C@H]3CC=CC[C@@H]3C(=O)O	0
C1CC[C@@H]2[C@@H](C1)C[C@@H](N2C(=O)NC3CCCC3)C(=O)[O-]	0
C[NH+]1CCc2c(c(c(c(n2)[S-])C#N)/C=C/c3ccccc3)C1	0
CCN1C2=C([C@H](NC1=O)c3ccc(cc3)C)C(=O)N(C2)C(C)(C)C(=O)NCc4cccc[nH+]4	0
CN1CCc2c(cnc(n2)NCc3ccco3)C1	0
CC(C)(C)[C@H]1CCCN1C(=O)O	0
CCCOc1cc2c(cc1N)sc(n2)C	0
Cc1ccc(cc1S(=O)(=O)NC[C@H](c2ccc(cc2)Cl)[NH+](C)C)N3C(=O)CCS3(=O)=O	0
Cn1c2c(cn1)c(nc(n2)COc3c(cccc3Br)Br)N	0
c1ccc(cc1)CNC(=O)[C@H]2CN(CCN2S(=O)(=O)c3cccc(c3)C#N)Cc4ccco4	0
COc1ccc(c2c1c3c([nH]2)c(ncn3)Cl)C=O	0
C[C@@H]1CCCN(CC1)c2ccc(c(c2)Cl)C#N	0
CN[C@H]1CCCN(C1)c2c(ccc3c2nsn3)Cl	0
COc1ccccc1[C@H]2c3c(c4ccccc4[nH]3)C[C@@H]5N2C(=O)CN(C5=O)CCc6ccc(c(c6)OC)OC	0
Cn1cc(c2c1cccc2)/C=C(\C#N)/C(=O)Nc3cccc(c3)O	0
[H]/N=C\1/C(=C/c2ccc(c(c2)OCC)OCCOc3ccccc3CC=C)/C(=O)N=C4N1N=C(S4)C(C)C	0
c1ccc(cc1)C#CCNC(=O)N[C@@H]2CCOC3(C2)CCCCC3	0
CC(C)Cn1c(c(c(=O)[nH]c1=O)N(CC(C)C)C(=O)c2csc(n2)c3ccsc3)N	0
Cc1ccnc2c(NC(=O)c3ccc(N4C(=O)[C@@H]5[C@H](C4=O)[C@@H]4C=C[C@H]5C4)cc3)cccc12	1
CN(c1ccc(cc1)c2csc(n2)NC(=O)Cc3ccc4c(c3)OCCO4)S(=O)(=O)C	0
Cc1ccc(c(c1)Br)[N-]S(=O)(=O)c2c(cc(cc2C)N3C(=O)CCS3(=O)=O)C	0
CC1(CCCC[C@@H]1Nc2c(nsn2)Cl)C	0
c1ccc2c(c1)ccc(c2/C=N\c3ccc(cn3)Br)O	0
CCNc1c(cnc2c1c(ccc2F)Br)C#N	0
Cc1ccc(c(c1)C(=O)NC[C@H](c2ccc(o2)C)N3CCOCC3)C	0
Cc1ccc(cc1)[C@H]2c3ccsc3CCN2CCC(=O)N4CCN([C@@H](C4)C)C(=O)COCc5ccccc5	0
Cc1ccc(cc1[N+](=O)[O-])C(=O)Nc2ccc(cc2)c3csc4[n+]3CCN4	0
CNC(=O)c1c2ccccc2c(=O)n(n1)C[NH+]3CCN(CC3)Cc4ccc(s4)Br	0
Cc1cc(c(s1)C)[C@H]2C(=C(OC3=C2C(=O)CC(C3)(C)C)N)C#N	0
c1cc2c(cc1N[C@@H]3CCS(=O)(=O)C3)nc(o2)C4CC4	0
CC(C)(C)OC(=O)N1C[C@@H]2C[C@H]3CNC[C@@]3(C1)O2	0
CC1(Cc2c(c(nc3c2c4c(s3)c(ncn4)SCCO)N5CCOCC5)CO1)C	0
Cc1ccn(n1)[C@@H](C)C(=O)N2[C@@](CC(=N2)C)(C(F)F)O	0
O=c1[nH]c2sccc2c2cc(O)ccc12	1
O=C(c1ccc(OC(F)(F)F)cc1)C1CCN(CC(=O)N(Cc2nc3c(c(=O)[nH]2)COCC3)CC2CC2)CC1	1
CC[C@@H]1CCC[C@](CC1)([C@@H]2CCOC3(C2)CCOCC3)	0

O	
CC(C)(O)c1ccc(-c2nc3c(F)cccc3c(=O)[nH]2)cc1	1
c1cc(c(c(c1)Cl)[C@H]2c3ccc(cc3OC(=C2C#N)N)OC(=O)c4ccco4)Cl	0
N#Cc1ccccc1Cn1c(=O)n(C[C@H]2CC[C@H](C(=O)NCc3ccccc3)CC2)c(=O)c2ccccc21	1
[H]/N=C\1/C(=C/c2cn(c3c2cccc3)CCOc4ccc(cc4)Br)/C(=O)N=C5N1N=C(S5)CCC	0
CCS(=O)(=O)N1CCC(CC1)(c2ccccc2)C(=O)N[C@@H](C)c3ccc(cc3)C#N	0
CS(=O)(=O)c1cccc(c1)S(=O)(=O)NCC[NH+]2CCN(CC2)c3c4c(cc[nH+]3)CCCN4	0
C[C@]1(Cn2c(cc(n2)c3cccs3)C(=O)N1c4ccc5c(c4)OCCO5)C(=O)NC(C)(C)C	0
Cc1ccc(c(c1)C)[C@H](c2ccc3c(c2)oc(=O)n3C)N	0
Cc1cccc2c(=O)[nH]c3c(c12)COC(C(C)(C)CO)C3	1
CCOc1cc2c(cc1OCC)[nH]c(=S)n(c2=O)CCC[NH+]3CCN(CC3)c4cccc(c4)Cl	0
c1cc(cc(c1)Br)N2C(=O)[C@@H]3[C@@H](C2=O)N(N=N3)CC(=O)Nc4ccc(cc4)Br	0
CCOC(=O)[C@H]1CCCN(C1)C(=O)CN(C)C(=O)[C@H](C)NC(=O)c2cccc(c2Cl)Cl	0
[H]\N=C\1/C(=C/c2cc(n(c2C)c3cc(ccc3OC)C)C)/C(=O)N=C4N1C(=NS4)S(=O)(=O)C(C)C	0
CC[C@@H](/C(=N/O)/N)N[C@H](C)c1cc(oc1C)C	0
COc1ccc(cc1)n2c(nnn2)SCC(=O)N(Cc3cccs3)c4ccc5c(c4)OCCO5	0
CCOC(=O)c1c(csc1NC(=O)c2c(n(c3[n+]2cc(cc3)C)c4ccccc4C)S)c5cccs5	0
Cc1cccc(Cn2c(=O)n(C[C@H]3CC[C@H](C(=O)Nc4ccncc4)CC3)c(=O)c3c(C)cccc32)c1C#N	1
C[C@@H]1CCCC[C@@H]1NC(=O)[C@@H]2CCc3c([nH]cn3)C2	0
CC\1=NC(=[NH+][C@H](/C1=C(/[O-])\OC)c2cn(nc2c3cccs3)c4ccccc4)O	0
COc1ccc(C2(CNC(=O)c3ccc(NC(=O)c4ccc(C)o4)cc3)CCOCC2)cc1	1
CCN1C(=O)/C(=C\c2cn(nc2C)C)/N=C1SCC(=O)Nc3c(c4c(s3)C[C@H](CC4)C)C(=O)OCC	0
Cc1ccccc1CC(=O)N[C@@H](C)c2[nH+]c3ccccc3n2CCCOc4ccccc4CC=C	0
C1CCC(CC1)(C#N)C2(CCCCC2)C#N	0
Cc1cc(nn1C[C@@H](C)c2nnc(n2CCCN3CCOCC3)[S-])[N+](=O)[O-]	0
CC(C)Cn1c2c(c(n1)C(=O)NCc3ccc[nH+]c3)CN(CC2)C(=O)C4=C[C@@H]5C(=CC=N5)C=C4	0
Cc1ccc(c(c1)Br)Nc2nc(nc(n2)N3CCOCC3)N/N=C/c4ccc(c(c4)[N+](=O)[O-])Br	0
C[C@@]1(CCCO1)c2[nH]c3cc(c(cc3n2)F)Br	0
Cc1ccc2c(c1)c3c([nH]2)c(=O)n(cn3)CCC(=O)N4CCN(CC4)c5ccc(cc5)C(=O)C	0
c1cn(nc1CN)c2cc(cc(c2)Cl)Cl	0
COc1ccc(cc1NC(=O)[C@@H]2Cc3ccccc3CN2C(=O)c4ccco4)S(=	0

O)(=O)N5CCOCC5	
Cc1cccc2c1c(=O)n(C[C@H]1CC[C@H](C(=O)Nc3ccnnc3)CC1)c(=O)n2Cc1cccc(C(F)(F)F)c1C#N	1
c1c([nH]cn1)S(=O)(=O)N2CCCC[C@@H]2[C@@H]3CCCC[C@@H]3O	0
CC[C@H]1CO[C@@H](CN1C(=O)c2cc(c(c(c2)Cl)Cl)N)C	0
Cc1ccc(cc1)n2c(cc(c2C)/C=N\NC(=O)CN(c3ccc(c(c3)OC)OC)S(=O)(=O)C)C	0
c1cc(sc1)[C@H]2C[C@H](n3c(nnn3)N2)c4ccc(cc4)Cl	0
c1cc(cc(c1)Cl)/C=C/C(=C/C2=NCCN2)/O	0
c1ccc(cc1)S(=O)(=O)Nc2ccccc2NC(=O)CN(c3ccc(cc3)Br)S(=O)(=O)c4ccccc4	0
Cn1c(=O)ccc(n1)C(=O)Nc2ccc(cc2)c3nc4ccccc4s3	0
Cc1cc(c2cc(c(=O)[nH]c2c1)[C@H](c3nnnn3Cc4ccco4)N5CCN(CC5)Cc6ccccc6)C	0
CCCCn1c2ccc(cc2nc1C[NH+]3CCN(CC3)CC(=O)N4CCCCCC4)S(=O)(=O)N	0
Cc1ccc(c2c1[nH+]ccc2)NC(=O)N(C)Cc3ccc(cc3)OC	0
C[C@@H]1[C@@H](Oc2ccccc2O1)C(=O)Nc3cc4c(cc3Cl)OCCCO4	0
CCCN1C(=O)/C(=C\c2c(nc3c(cccn3c2=O)C)N[C@@H](CC)CO)/SC1=S	0
CCOc1cc(ccc1O)[C@H]2C(=C(C(=O)N2Cc3ccco3)[O-])C(=O)c4c(nc5n4cccc5)C	0
CCOc1ccc(-c2nnc([C@H]3C[C@H](NC(=O)c4ccnc5cccnc45)C3)n2-c2ccc(C)s2)nc1	1
Cc1c(c(n(n1)c2ccc(cc2CO)[N+](=O)[O-])C)Cl	0
C[C@@]12c3c(c4ccccc4[nH]3)CCN1C(=O)CN(C2=O)/N=C/c5ccc(cc5)Cl	0
O=C1NN=C2CC(c3ccccc3)Cc3[nH]c4cccc1c4c32	1
Cc1cc(ncc1Br)NC(=O)c2ccc(c(c2)F)[N+](=O)[O-]	0
C[C@@H]1CN(C[C@@H]1C)C(=O)N[C@@H]2CCC[C@@H](C2)C(=O)[O-]	0
CC(C)(N)C(=O)N1CC2=NNC(=O)c3cccc4[nH]c(c2c34)C1	1
C[NH+]1CCC(CC1)N2C[C@@H]3C[C@H](C2)c4ccc(c(=O)n4C3)NC(=O)COc5ccccc5	0
C1CN[C@@H](CC12OCCO2)C#N	0
C[C@@H]1Cc2ccccc2N1C(=O)NCc3cn4cc(ccc4[nH+]3)Cl	0
c1c2c(cc(c1Cl)Cl)n(c(n2)N)[C@@H]3CCN4[C@@H]3CCCC4	0
C1CC2(CCC1(C(F)(F)F)[NH3+])OCCO2	0
Cc1c(sc(n1)NC(=O)c2cccc(c2)S(=O)(=O)N3CCCCCC3)c4nccn4C	0
Cc1cc(ccc1F)S(=O)(=O)N2CCOC23CCN(CC3)S(=O)(=O)c4cc(ccc4OC)F	0
Cc1ccc(cc1S(=O)(=O)NC[C@@H](c2ccccc2)[NH+](C)C)N3C(=O)CCS3(=O)=O	0
c1cc(ccc1S(=O)(=O)[C@@H]2CCN(C2)C(=O)c3cc(n[nH]3)c4ccc5c(c4)OCO5)Cl	0
Cc1cc(c(o1)C)C(=O)NCC(=O)Nc2nc(cs2)CN3CCCCCC3	0
c1cc2c(cc1F)nc(n2C3CC3)c4ccnc(c4)N	0
CN1[C@@H]([C@@H](CCCC1=O)C(=O)O)c2ccc(c(c2)Cl)Cl	0
O=c1[nH]c(N2CCC3(CC2)OCc2ccccc23)nc2c1CCCC2	1

**Cn1cc(cn1)Cn2c3ccc(cc3[nH+]c2N)Cl**	0
**CC#CC[C@H]1CCCCC[C@@H]1C(=O)[O-]**	0
**Cc1c(n(c2c1c(nc(n2)C)N)[C@H](C)C(C)C)C**	0
**Cc1cc(c(c(c1)Br)O)/N=N\c2ccccc2**	0
**c1cc(c(c(c1)F)[C@H]2CC(=Nc3c2cn[nH]3)O)F**	0
**CC1(CCC[C@H]1n2c(nnn2)c3ccc(cc3Cl)N)C**	0
**CCOc1ccc(cc1)c2n[nH]c(=S)n2CCC(=O)N3CCCc4c3cccc4**	0
**c1ccc2c(c1)nc(n2c3cc(c(cc3Cl)Cl)Cl)N**	0
**CC(C)(C)c1ccc(-c2nc(O)c3c(n2)CCSC3)cc1**	1
**C[C@H]1CCCC[C@@H]1NC(=O)[C@@]2(Cn3c4ccoc4cc3C(=O)N2CCN(C)C)C**	0
**COc1cc(c2c(c1)occ(c2=O)c3ccc(c(c3)OC)O[C@H]4[C@@H]([C@@H]([C@@H]([C@@H](O4)CO[C@@H]5[C@@H]([C@@](CO5)(CO)O)O)O)O)O)O**	0
**CC[C@@H](C(=O)N1CCN(CC1)c2ncccn2)Sc3[nH+]cc(c(n3)N)C(=O)OCC**	0
**COC(=O)[C@@H]1C[C@H]2Cc3c4ccccc4[nH]c3CN2C1**	0
**C[C@@H](C(=O)c1ccc2c(c1)CCN2C(=O)C)Sc3nnc(s3)N**	0
**COc1ccc(C(=O)C2CCN([C@H]3CCN(Cc4nc5ccccc(=O)c5[nH]4)C3=O)CC2)cc1C**	1
**Cc1ccn2cc(nc2c1)c3ccc(c(c3)NS(=O)(=O)c4ccc(cc4)NC(=O)C)C**	0
**CC1([C@H]([C@H]2[C@@H]1OCC2)Nc3ccc4c(c3)CCO4)C**	0
**Cc1cc(c(c(c1)C)C(=O)N(C)OC)C**	0
**CC1(CCCC1)C(=O)NC[C@H]2CCCC[C@H]2O**	0
**Cc1cccc(c1NS(=O)(=O)c2cc(ccc2OC)N3C(=O)CCS3(=O)=O)C(C)C**	0
**C[C@H]1C(=O)[C@]2([C@]3([C@H](CC(=C)[C@@]2(C(=O)O1)C)[C@]4([C@@H](CC(=O)C(C4=C(C3=O)O)(C)C)O)C)C)C(=O)OC**	0
**c1ccc(C2COCN2c2ccc(-c3cncc(-c4ncccn4)c3)cc2)cc1**	1
**Cc1c2c(nc(nc2n(n1)c3ccccc3Cl)C)NC[C@@H]4CCCO4**	0
**COc1cc(c(cc1OC(F)F)NS(=O)(=O)c2ccc3c(c2)CCC3)C(=O)[O-]**	0
**c1cc(c(cc1Cl)Cl)n2c3c(cc(cn3)Br)nc2N**	0
**c1cc(cc(c1)[N+](=O)[O-])[C@H]2C(=C(C(=O)N2CC[NH+]3CCOCC3)[O-])C(=O)c4ccc(cc4)Br**	0
**CC1(Cc2c(cc(c(=O)n2c3ccc(cc3)OC)C(=O)NCCc4ccc(c(c4)OC)OC)C(=O)C1)C**	0
**c1cc2c(cc1NC(=O)CSc3c4c(ncn3)nc(s4)N5CCOCC5)CCC2**	0
**COC(=O)CCN\1C(=O)[C@H](S/C1=N/c2ccccc2)CC(=O)Nc3ccc(cc3)Br**	0
**CSCC[C@@H](C(=O)N1CCN(CC1)C(=O)N2CCCCC2)NS(=O)(=O)c3ccccc3F**	0
**c1cc(sc1)/C=C/2\CCC[C@@H]3C2=NN([C@H]3c4cccs4)C(=O)CN5C(=O)C6(CCCC6)NC5=O**	0
**c1cc(oc1)CNc2cc(cc(c2)S(=O)(=O)N3CCCC3)C(=O)NCCn4ccnc4**	0
**Cc1ccccc1OCCN2c3c(c(ccc3[C@]4(C2=O)N(N=C(S4)NC(=O)C)C(=O)C)C)C**	0
**Cc1cccc2c1c(=O)n(C[C@H]1CC[C@H](C(=O)Nc3ccncc3)CC1)c(=O)n2Cc1cccc(Cl)c1C#N**	1
**CC(C)(C)c1ccc(C2NC(=O)c3ccccc3N2)cc1**	1
**Cc1cc(c(c(c1)C)/C=C/c2c(c(no2)C)S(=O)(=O)N3CCC(CC3)C(=O**	0

**)NCc4ccc[nH+]c4)C**	
**CCCCCN1C(=O)/C(=C\c2c(nc3ccccn3c2=O)N4CCN(CC4)C(=O)OCC)/SC1=S**	0
**C[C@@H]1[C@H](N(CC[NH2+]1)C(=O)OC(C)(C)C)C**	0
**Cc1c(c2ccccc2[nH]1)CN3CCc4c(c(cc(=O)n4CC3)OC)C(=O)N5CCC[C@H]5C(=O)OC**	0
**Cc1ccc(cc1S(=O)(=O)N2CCOCC2)NC(=O)[C@H](C)Sc3nnc4n3CCCCC4**	0
**c1cc(c2c(c1)nc(cn2)Cl)C(F)(F)F**	0
**CNc1c2c(ncn2C)nc(n1)Cl**	0
**Cc1c(ccc(n1)NC(=O)c2cc(ccc2[N+](=O)[O-])Cl)Br**	0
**C[C@@H]1CN(c2cc(F)c3c(c2)N(C)C(=O)C32CCN(c3nc4c(c(=O)[nH]3)CCCN4C)CC2)C[C@H](C)N1**	1
**Cc1ccoc1[C@H](c2ccc3c(c2)oc(n3)[O-])O**	0
**c1c2c(cc3c1[nH+]cn3)c4cncc2C4**	0
**C1CCc2nnc(n2CC1)S[C@H]3CCCCC[C@H]3O**	0
**COc1ccc(cc1)c2ncc(cn2)C[NH+]3CCc4c(c(nc(n4)c5cccnc5)[O-])C3**	0
**Cc1c(c(c(c(n1)OCC(=O)N/N=C/C2=c3ccccc3=[NH+]C2)C#N)COC)Br**	0
**c1c[nH+]ccc1c2cc(ncn2)N3CCC[C@@H](C3)C(=O)[O-]**	0
**COc1ccc(C(=O)C2CCN(C3CCN(CC(=O)Nc4nncc5c4CCCO5)C3=O)CC2)cc1**	1
**Cc1nc(co1)c2ccc(cc2)NC3CCC(CC3)(C)C**	0
**Cc1cccc(n1)C(=O)N(CCC2=CCCCC2)c3c(n(c(=O)[nH]c3=O)Cc4ccccc4)N**	0
**CC(=O)Nc1ccc(cc1)S(=O)(=O)NCC(=O)N2CCCN(CC2)Cc3ccc(cc3)C#N**	0
**Cc1ccc(cc1C)N(Cc2ccc(cc2)C(=O)NCCCN3CC[NH+](CC3)C)S(=O)(=O)C**	0
**C[C@@H]1CN(c2ccccc2S1)S(=O)(=O)c3c[nH+]c([nH]3)C(C)C**	0
**CC[N+]1=C(S/C(=C\c2cccc(c2)[N+](=O)[O-])/C1=O)Nc3ccc(cc3)C(=O)[O-]**	0
**c1cc2c(nc1)nc([nH]2)/C(=C\c3ccc(cc3)C#N)/C#N**	0
**CCOc1ccc(cc1F)S(=O)(=O)N2CCC3(CC2)N(CCO3)S(=O)(=O)c4ccc(c(c4)C)F**	0
**c1ccc(cc1)c2nc([n-]n2)S[C@@H]3CC(=O)N(C3=O)c4ccc(cc4)Oc5ccccc5**	0
**CC1(CCCC1)Nc2c(nsn2)Cl**	0
**Cc1[nH]c2ccc(cc2n1)S(=O)(=O)c3ccc4c(c3)[nH]c(n4)C**	0
**CN(C)c1nc(nc(n1)Sc2nccs2)N**	0
**O=C1NC(c2ccc(Cl)cc2)Nc2ccccc21**	1
**c1cc(c(cc1C#N)Cl)n2c3cc(ccc3cn2)N**	0
**c1cc2c(cc1/N=C\3/NC4(CCCC4)CS3)OCO2**	0
**CS(=O)(=O)N1CCC[C@@H](C1)C(=O)N/N=C/c2cc(ccc2O)Br**	0
**Cc1nc(cc2n1c(=O)[nH]n2)[C@H]([C@H](C)Cl)C(C)C**	0
**Cc1ccc(c(c1)O)NC(=O)C2CCN(CC2)S(=O)(=O)c3ccc4c(c3)CCC4**	0
**C[C@H]1CCCCN1S(=O)(=O)N2CCCCC[C@@H]2N**	0
**Cc1ccccc1n2c(c(c(n2)c3cccc(c3)C(F)(F)F)C#N)N**	0
**CCN(CC)S(=O)(=O)c1ccc(c(c1)NC(=S)N2CC[NH+](CC2)Cc3ccc4c(c3)OCO4)C**	0

CN1CCN(CCOc2cc(F)c3c(c2)N(C)C(=O)C32CCN(c3nc4c(c(=O)[nH]3)CCCN4C)CC2)CC1	1
O=C(CCc1ccccc1)N(Cc1nc2c(c(=O)[nH]1)COCC2)Cc1ccccc1F	1
CCCCC12C[NH+]3CC(C1=O)(C[NH+](C2)C34c5cc(ccc5NC4=O)Br)C	0
Cc1ccccc1-n1c(/C=C/c2nnc(-c3ccc(C#N)cc3)o2)nnc1-c1ccc(S(C)(=O)=O)cn1	1
COc1ccc(C(=O)C2CCN(CC(=O)NCc3nc4c(c(=O)[nH]3)CCC4)CC2)cc1	1
[H]/N=C/1\C2=C(N=NC2=N[C@@H](N1c3cccc(c3)Cl)SCc4cc(=O)n5ccccc5[nH+]4)C	0
c1cc(ccc1NC(=O)COc2c3cc(ccc3ncn2)Br)S(=O)(=O)N4CCCC4	0
Cn1ccc(n1)NC(=O)c2cc(cc(c2F)S(=O)(=O)N3CCOCC3)Br	0
CCOC(=O)c1ccc(cc1)NC(=O)c2cc(=O)n(/c(=N/Cc3ccc[nH+]c3)/s2)Cc4cccnc4	0
CC1([C@H]2CC[C@@]13CNS(=O)(=O)[C@@H]3C2)C	0
Cc1cc(n(n1)c2ccc(cc2)Cl)NC(=O)c3ccc4c(c3)COCO4	0
c1ccc2c(c1)C[C@H](CO2)C(=O)Nc3ccc4c(c3)OCO4	0
CC1(Cc2cccc(c2O1)OCC(=O)Nc3cccc(c3)S(=O)(=O)/N=C\4/CCCN4)C	0
Cc1cc(ccc1S(=O)(=O)Nc2cccc(c2)CN3C(=O)[C@H]4CCCN4C3=O)N5CCCC5=O	0
c1c(cc(cc1F)F)c2c(onc2N)[C@@H]3C[C@H]4CC[C@@H]3C4	0
Cc1c(sc2c1c(nc(n2)CN(C)c3ccccc3)N)C	0
Cc1cc(c(n1c2ccccc2)C)C(=O)N/N=C(/C)\c3ccc4c(c3)OCCO4	0
O=c1[nH]c2ccccc2c2c1sc1ccccc12	1
CCCn1cc(c(n1)C)C(=O)N2CCCC[C@@H]2c3nc4c(c(n3)[O-])C[NH+](CC4)C(C)C	0
CC(C)NC(=O)CCNC(=O)NC[C@H](c1ccc(cc1)F)[NH+](C)C	0
COc1ccc(N2CCN(c3nc4c(c(=O)[nH]3)CCCC4)CC2)cc1	1
c1cc(cc(c1)Cl)[C@H]2/C(=C(\c3ccc4c(c3)OCCO4)/O)/C(=O)C(=O)N2Cc5cccnc5	0
Cc1ccc(cc1)/C=C\2/C(=O)N(C(=O)S2)CCNC(=O)[C@@H]3CC(=O)N(C3)c4cccc(c4)OC	0
c1ccc(cc1)C(c2ccccc2)C(=O)NCCCS(=O)(=O)N3CCN(CC3)c4ncccn4	0
c1ccc(cc1)S(=O)(=O)CCC(=O)N2CC[NH+](CC2)CCNC(=O)c3ccc4c(c3)OCO4	0
C[C@H]1CN2CCC[C@@H]2CN1C(=O)Nc3ccc(c(c3)F)F	0
COc1cccc2c(=O)[nH]c(-c3ccc(C(C)(C)O)cc3)nc12	1
c1c(cc(c(c1Cl)S(=O)(=O)[N-]c2c[nH]nc2)Cl)Cl	0
C[C@H]1CN(C[C@@H](O1)C)S(=O)(=O)c2ccc(cc2)C(=O)Nc3c(ccs3)C(=O)NC(=O)OC	0
Cc1cc(ccc1n2c3ccc(cc3[nH+]c2CO)N)Br	0
Cc1cc2ncc(c(n2n1)N)c3ccc(cc3)F	0
c1ccc2c(c1)CC[C@@H]2c3nc(on3)c4c(ccc[nH+]4)[O-]	0
C[C@H]1CCc2ccccc2N1C(=O)NC(=O)[C@H](C)Cl	0
Cn1cc(cn1)C[NH+]2CC[C@H]3[C@@H]2C[C@@H](O3)C(=O)NCC4CCOCC4	0
Cc1cc2c(cc1S(=O)(=O)[N-]c3cc(c(c(c3)OC)OC)OC)[nH]c4c2C(=O)CC(C4)(C)C	0
C[C@@H](CN1CC(C(=O)N2CCN(c3ncc(C(F)(F)F)cn3)CC2)C1)	1

**Nc1cn[nH]c(=O)c1C(F)(F)F**	
**CCC(=O)N1CCc2c1ccc(c2)S(=O)(=O)CCC(=O)Nc3c(n(n(c3=O)c4ccccc4)C)C**	0
**Oc1nc(-c2ccc(F)cc2)nc2ccccc12**	1
**c1cc(c(cc1Br)S(=O)(=O)NCCc2nc[nH]n2)[N+](=O)[O-]**	0
**O=c1[nH]c(SCCN2CCN(CCCc3nc(-c4ccccc4)no3)CC2)nc2ccccc12**	1
**Cc1ccc(c(c1)N[C@@H]2COc3c2ccc(c3)O)F**	0
**C[C@H]1CC(=O)NC2(CCCCC2)C(=O)N1C[C@H]3CCCO3**	0
**C[C@H](C12CC3CC(C1)CC(C3)C2)n4c(=O)[nH][nH]c4=S**	0
**Cc1ccc(cc1C)n2c(cc(c2C)/C(=C(/C#N)\C(=O)c3cccc(c3)NC(=O)c4ccco4)/[O-])C**	0
**CCN(CC)S(=O)(=O)c1ccc(c(c1)NC(=O)CSc2nnc(n2C)C(F)(F)F)C**	0
**CN1[C@@H]([C@@H](c2cc(c(cc2C1=O)OC)OC)C(=O)Nc3ccc(c(c3)OC)OC)c4cccs4**	0
**CCOc1ccc(cc1Cl)C(=O)C2=C(C(=O)N([C@@H]2c3cccc(c3)Br)CCCn4cc[nH+]c4)O**	0
**c1ccc(cc1)[C@@H]2CN(CC[NH2+]2)c3cc(ccn3)C(=O)N4CCN(CC4)c5cccc[nH+]5**	0
**COc1ccc(-c2noc(CSc3nnc(-c4ccncc4)n3-c3ccccc3)n2)cc1**	1
**Cc1ccc(cc1)C2=C(C(=O)N([C@H]2c3ccc(cc3)F)Cc4ccc(cc4)F)O**	0
**CC(C)[C@H](C(=O)Nc1ccc(nc1)N2CC[NH+](CC2)C)NC(=O)c3ccc(cc3)Cl**	0
**C[C@@H]1CCc2c(sc(c2C(=O)OC)NC(=O)Cn3c(=O)ccc(n3)C(C)(C)C)C1**	0
**Cc1cc(nc2c1cc(cc2)S(=O)(=O)N(C)C)SCC(=O)Nc3ccc4c(c3)OCO4**	0
**Cc1c(c([nH]c1C(=O)OC)C)/C(=C/2\[C@@H](N(C(=O)C2=O)CCCn3cc[nH+]c3)c4cccs4)/O**	0
**CC(C)(CO)C1Cc2[nH]c(=O)c3ccccc3c2CO1**	1
**Cc1ccc(c(c1)c2cc(n(n2)C)N)C**	0
**CC1(CCCC[C@@H]1N2CCNCC2=O)C**	0
**C[C@H]1CN(C[C@@H]1C)C(=O)C[C@@H]2COCCN2**	0
**COc1ccc(C(=O)C2CCN([C@@H]3CCCN(CCC4NC(=O)C5=C(CCOC5)N4)C3=O)CC2)cc1**	1
**C[C@H]1CCCC[C@]12C[C@@H](CCO2)O**	0
**c1cc(cc(c1)NC(=O)NC[C@@H](c2ccco2)[NH+]3CCCCC3)Cn4cccnc4=O**	0
**Cc1ccccc1OCCOc2ccc(cc2)/C=C\3/C(=[NH2+])N4C(=[NH+]SC4=NC3=O)SC**	0
**O=C(c1cccc(OC(F)(F)F)c1)C1CCN(CC(=O)N(Cc2nc3c(c(=O)[nH]2)COCC3)CC(F)F)CC1**	1
**CC[NH+](CC)CCCN1[C@@H](C(=C(C1=O)O)C(=O)c2c(nc(s2)C)C)c3ccc(c(c3)OC)OC**	0
**c1ccc(c(c1)c2[nH]ccn2)C(=O)N3C[C@H](CN(C(=O)C3)C4CCCCC4)OCc5ccccn5**	0
**c1cc[nH+]c(c1)/N=C/2\NN=C(CS2)c3ccc(s3)Cl**	0
**COc1cccc2c(O)nc(-c3ccc(C(F)(F)F)cc3)nc12**	1
**Cc1c(c(=O)n(n1C)c2ccccc2)C(c3ccc(c(c3)OC)O)c4c(n(n(c4=O)c5ccccc5)C)C**	0
**C1CCN2C[C@H](OC[C@@H]2C1)CNC(=O)N3CC[C@@]4(C3**	0

)CCOC4	
c1ccc(c(c1)c2ccc(o2)/C=C(/c3ccc(s3)S(=O)(=O)N4CCOCC4)\C(=O)[O-])Cl	0
Cc1c(n(c(c1C#N)NC(=O)C[NH+]2CCN(CC2)CC(=O)N3CCCC3)Cc4ccccc4)C	0
O=C(CCC1CCCCCC1)Nc1nnc2n1CCS2	1
O=C(Nc1ccccc1F)c1ccc(NC(=O)N2CCN(C(=O)c3cc(Cc4n[nH]c(=O)c5ccccc45)ccc3F)CC2)cc1	1
CCCCOc1ccc(c(c1)C)C(=O)[C@H]2[C@H](N(C(=O)C2=O)Cc3ccc[nH+]c3)c4ccc(c(c4)OCC)O	0
Cc1cc(c(c(c1)C)c2cc(c[nH+]c2)C(=O)[O-])C	0
O=C1Nc2ccccc2C12CCN(c1nc3c(c(=O)[nH]1)CCCC3)CC2	1
C=CCNC(=O)N1[C@H]2CCCC[C@@H]2S[C@@H]3[C@@H]1CCCC3	0
CCOc1ccc2c(=O)[nH]c3sccc3c2c1	1
Cc1ccc(cc1N[C@H](C)c2ccc(s2)Br)C(=O)[O-]	0
c1cc(cc(c1)[N+](=O)[O-])/C=C\2/C(=O)N(C(=O)S2)CCC(=O)Nc3ccc(cc3)Br	0
c1ccc(cc1)CN2CCN(CC2)c3nnc(s3)n4cccc4CNCC[NH+]5CCCC5	0
c1cc2c(cc1[N-]S(=O)(=O)[C@H]3CCNC3)OC(O2)(F)F	0
Cc1ccc(cc1S(=O)(=O)N2CCc3c(sc(n3)NC(=O)c4cc(on4)C)C2)Cl	0
Cn1c([nH+]nc1SCc2nnc(s2)C(=O)NCc3ccc(cc3)F)[C@H]4CCS(=O)(=O)C4	0
c1ccc2c(c1)cccc2NC(=O)N[C@@H]3CCC[C@@H]3C#N	0
Cc1c(c(on1)C)CSc2c(cccn2)C(=O)Nc3nc4c(s3)CN(CC4)C	0
Cc1cccn2c1nc(c2C)C(=O)C3=C(C(=O)N([C@@H]3c4ccc(cc4)Cl)CCC[NH+]5CCOCC5)[O-]	0
CC(C)(CCCNC(=O)NC[C@H]1C[NH+]2CCCC[C@@H]2CO1)CO	0
N#Cc1ccc(O[C@H]2CC[C@H](NC(=O)CCSc3nc4ccccc4c(=O)[nH]3)CC2)cc1Cl	1
c1cc(sc1)c2cc(n[nH]2)C(=O)N3CCN(CC3)S(=O)(=O)c4ccc(s4)Cl	0
Cc1ccc(c(c1)Cl)S(=O)(=O)NNC(=O)c2c([nH+]c(s2)N3CCOCC3)C	0
c1ccc(C2=NC3=C(CN2)COCC3)cc1	1
Cc1c(ccc2c1NC(=O)[C@@]23C(=C(N(C4=C3C(=O)CC(C4)(C)C)c5nnc(s5)SCc6cccc(c6)C(F)(F)F)N)C#N)Cl	0
O=C(C1COC1)N1CCC(C2Cc3[nH]c(=O)c4ccccc4c3CO2)CC1	1
c1ccc2c(c1)nc(o2)Sc3c4cc(ccc4ncn3)N	0
COc1ccc(cc1OC)[C@H](CC(=O)N2CCN(CC2)c3nc4ccc(cc4c(n3)c5ccccc5)Br)[NH3+]	0
CC[C@H](C)CN(Cc1nc2c(c(=O)[nH]1)COCC2)C(=O)CN1CCC(C(=O)c2ccc(F)cc2)CC1	1
c1cc(c(nc1)Cl)S(=O)(=O)[N-]c2ccc(cc2)O	0
Cn1ncnc1[C@@H]1c2n[nH]c(=O)c3cc(F)cc(c23)N[C@H]1c1ccc(F)cc1	1
C[C@H]1c2ccsc2CCN1c3nc4c(s3)CCC[C@@H]4O	0
Cc1ccc(cc1)CNc2c(c(c(c(=O)n2C)C#N)C)/C=C\3/C(=O)N(C(=S)S3)C[C@H]4CCCO4	0
c1cc(c(c(c1Cl)C(=O)N[C@H]2C[C@H]3CC[C@@H]2O3)Cl)Cl	0
Cc1cc(c(cc1Cl)Cl)S(=O)(=O)N2CC(C2)c3nc(no3)c4ccc[nH]c4=O	0
CC(C)c1ccccc1Nc2ccc(nn2)C(=O)[O-]	0

**Cc1c(sc2c1c(=O)n(cn2)CC3(CCN(CC3)C(=O)[C@@H](C)CN4CCOCC4)O)C**	0
**Cc1cc(sc1C)c2nnc3n2cc(cc3)N**	0
**Cc1nnc2n1nc(s2)c3ccc(cc3)NC(=O)c4cccc(c4)Cl**	0
**CCOc1ccc(cc1)N2/C(=C/c3cc(c(cc3Br)OCCO)OCC)/C(=O)N(C2=S)C**	0
**c1cc(ccc1Oc2coc3c(c2=O)ccc(c3O)[O-])F**	0
**COc1ccc2c(C3CCN(CC(=O)NCc4nc5c(c(=O)[nH]4)COCC5)CC3)n[nH]c2c1**	1
**Cc1ccc(o1)C(=O)C2=C(C(=O)N([C@@H]2c3ccco3)c4nnc(s4)SCc5ccc(cc5)F)O**	0
**Cc1ccc(cc1)/C=C\2/C(=O)N(C(=O)S2)CCC(=O)Nc3c(n(n(c3=O)c4ccccc4)C)C**	0
**Cc1ccc(c(c1)S(=O)(=O)N2CCN(CC2)C(=O)[C@@H](CCSC)NC(=O)c3ccccc3)C**	0
**CC[C@](C)([C@]1(CCOc2c1cccc2)C#N)O**	0
**O=c1cc(-c2ccc(F)cc2)oc2ccccc12**	1
**C1CCCC2(CCC1)C(=O)N(C(=O)N2)CN3CCCC3**	0
**CC(C)(C)c1ccc(-c2nc3c(sc4ccccc43)c(=O)[nH]2)cc1**	1
**CCSC1=[NH+]SC2=NC(=O)/C(=C\c3ccc(cc3)OCCOc4ccccc4)/C(=[NH2+])N12**	0
**Cc1cccc(c1)[N-]S(=O)(=O)c2ccc(c(c2)C(=O)OCC(=O)N3CCC[C@H]3c4cccs4)C**	0
**Cc1cc(nc(n1)N)Oc2ccccc2I**	0
**N#Cc1ccc(N2CCN([C@H]3C=C(c4cc5ncccc5c(=O)[nH]4)CC3)CC2)cc1**	1
**C[C@H]1[C@H](O[C@@H]([C@@H]1C(=O)NC(C)(C)C)C)C**	0
**CC1(C)OC(=O)N([C@H]2CC[C@H](NC(=O)c3ccnc4cccnc34)CC2)[C@H]1c1ccccc1**	1
**O=C(CN1CCC2(CC1)Cc1cc(F)ccc1C2=O)NCc1nc2c(c(=O)[nH]1)COCC2**	1
**Cc1c(sc(c1C(=O)NC(=O)OC)NC(=O)CCCS(=O)(=O)c2ccc(cc2)Cl)C**	0
**COc1cc(c(cc1Nc2c(nc3ccccc3n2)NS(=O)(=O)c4cccs4)Cl)OC**	0
**Cc1cc(c(c(c1)C)NC(=O)c2cc(ccc2[N+](=O)[O-])Cl)C**	0
**Cc1ccc(cc1)/C=C\C(=O)Nc2ccc3c(c2)NC(=O)[C@@H](O3)C**	0
**Cc1ccc(cc1)N2C(=O)/C(=C\c3cc(n(c3C)c4ccc(cc4)C(=O)C)C)/C(=O)NC2=O**	0
**Cc1cc(ccc1Br)N2CC(=O)NC3(C2=O)CCCC3**	0
**Cn1c(=O)ccc(n1)C(=O)N2CCc3c(c(n[nH]3)c4ccc(cc4Cl)Cl)C2**	0
**c1csc2c1[C@@H](N(CC2)C(=O)NC[C@H]3CCCO3)C(=O)[O-]**	0
**C[C@@H]([C@@H]1C=C(N=N1)c2ccccc2)N(C)C(=O)[C@@H]3CN(C=C(C3=O)C(=O)NCc4ccccc4)C5CC5**	0
**COc1ccc(C(=O)C2CCN(C(=O)CCCc3cn4cc(F)cc4c(=O)[nH]3)CC2)cn1**	1
**CCn1cc(c(=O)c2c1cc(c(c2)F)N3CCN(CC3)C(=O)c4ccco4)C(=O)NCc5ccc(cc5)Cl**	0
**Cc1cc(ccc1S(=O)(=O)[N-]c2ccc3c(c2)CCN3)Cl**	0
**c1cc2c(cc1Br)C(=O)N(C2=O)C[C@H]3CCC[C@H]3O**	0
**Cc1cc(no1)NC(=O)CSCC(=O)N2CCN(CC2)Cc3ccc(cc3)Cl**	0
**C[C@@H]1CN(C[C@H](O1)C)c2c3cnn(c3ncn2)CCNC(=O)c4cc5c6c(c4)CCN6CCC5**	0

**Cc1nc2n(n1)c(c(s2)[C@H](c3ccc(c(c3)Cl)Cl)N4CCOCC4)O**	0
**CN(C)c1ccc(cc1)CNC(=O)Cn2c3ccc(cc3oc2=O)S(=O)(=O)N4CCCCCC4**	0
**COc1ccc(cc1OC)C(=O)N/N=C/2\c3cc(ccc3N(C2=O)CN4CCCC[C@@H]4c5cccnc5)Br**	0
**O=c1cc(-c2cccc(O)c2)oc2cc(O)ccc12**	1
**Cn1cnc(c1S(=O)(=O)N2CCC[C@H]2C3CCCC3)N**	0
**Cc1c(nnc(c1C#N)Nc2ccc(cc2)SC)C**	0
**Cc1c(oc(n1)C[NH+]2[C@H]3CC[C@@H]2[C@@H](C3)C(=O)[O-])C**	0
**CCN(CC)S(=O)(=O)c1ccc(cc1)N2C[C@H](CC2=O)C(=O)Nc3ccccc3C(=O)NC4CC4**	0
**CCCOc1ccc(C(=O)C2CCN(CC(=O)NCc3nc4c(c(=O)[nH]3)COCC4)CC2)cc1**	1
**Cc1c(c(no1)c2c(cccc2Cl)Cl)/C(=N/CCCN3CCOCC3)/O**	0
**Cc1ccc(cc1)N2CCN(CC2=O)C(=O)CSc3nnc(s3)N**	0
**CC[C@H]1CO[C@H](CN1S(=O)(=O)c2cc(c(c(c2)Cl)C)N)C**	0
**CC(C)[C@@H](C(=O)NCC[NH+](C(C)C)C(C)C)NS(=O)(=O)c1cccc2c1nsn2**	0
**C[C@@H]1C[C@@H](CC(C1)(C2(CCCCC2)C#N)O)C**	0
**C[NH+](C)C1CCN(CC1)C(=O)N[C@@H]2C[C@H](C=C2)C(=O)[O-]**	0
**c1ccc2c(c1)C[C@H](N2)c3nc(no3)c4ccco4**	0
**CCn\1c2cc3c(cc2s/c1=N\C(=O)c4ccc(cc4)S(=O)(=O)N5C[C@H](O[C@H](C5)C)C)OCCO3**	0
**C[C@H](Cc1nc(on1)c2ccc(cc2)[NH+](C)C)[NH3+]**	0
**CS(=O)(=O)CC[C@H](C(=O)NCCC[NH+]1CCC(CC1)Cc2ccccc2)N3Cc4ccccc4C3=O**	0
**C[C@H]1CCC[C@H](OC1=O)C(C)C**	0
**c1cc(c(cc1F)C(=O)N2CCC[NH+]3CCC[C@@H]3C2)O**	0
**c1ccnc(c1)C(=O)N2C[C@@H]3C[C@H](C2)c4ccc(c(=O)n4C3)NS(=O)(=O)c5cccs5**	0
**Cc1c2c([nH]n1)N=C(CS[C@H]2c3ccc(c(c3)OC)OC4CCCC4)O**	0
**C1=Cc2ccc(-c3ccc(CNCCN4CCOCC4)cc3)cc2NC1**	1
**Cc1cccc2c(O)nc(-c3ccc(O)cc3)nc12**	1
**Cc1ccc2nc(cc(=O)n2c1)CSc3c(nc(s3)N)C**	0
**COc1ccc(-c2nc(O)c3ccccc3n2)cc1**	1
**CCOc1ccc(-n2c(SCc3nc(-c4ccc(OC)cc4)no3)nnc2-c2ccncc2)cc1**	1
**c1c(cc(c2c1OCO2)Cl)Cn3c(c(cn3)Br)N**	0
**CCCNC(=O)[C@H]1CCCN1C(=O)c2cccc(c2)NS(=O)(=O)c3ccc(cc3)C**	0
**C[C@@H]1CCCCN1c2ccc(cc2C#N)Cl**	0
**C[C@@H]1CC[C@H]([C@@H](C1)N2CCO[C@@H](C2)C)O**	0
**Cc1ccc(cc1)C(=O)[C@H]2[C@@H](N(C(=O)C2=O)c3ccc(cc3)C(=O)[O-])c4ccc(cc4C)C**	0
**COc1ccc(cc1S(=O)(=O)[N-]c2cccc(c2)c3ccc(nn3)N4CCCC4)Br**	0
**CC(=O)c1ccc(cc1)NC(=O)C2CCN(CC2)S(=O)(=O)c3ccc(cc3)SC**	0
**Cc1c(cccc1Cl)N(CC(=O)Nc2ccc(cc2)S(=O)(=O)N3CCOCC3)S(=O)(=O)C**	0
**CS(=O)(=O)c1ccc2c(c1)sc(n2)N(CC[NH+]3CCOCC3)C(=O)c4c[nH]c5c4cccc5**	0
**c1ccc(c(c1)C(=O)NNC(=S)NC(=O)/C=C/c2ccc(cc2)[N+](=O)[O-**	0

])Br	
C[C@@H](c1ccccc1Cl)Nc2ccc(c(c2)Cl)C(=O)[O-]	0
CC[C@H](C)NC(=O)CN1c2c(c(nn2c3ccc(cc3)OC)C(C)(C)C)[C@@H](SCC1=O)c4ccc5c(c4)OCO5	0
CN(C)CC(=O)Nc1ccc2[nH]c(=O)c3ccccc3c2c1	1
Cc1ccc(-c2nc3c(O)cccc3c(=O)[nH]2)cc1	1
C[C@@H]1Cc2ccccc2N1C3CCN(CC3)c4ccc(cc4[N+](=O)[O-])S(=O)(=O)NC	0
Cc1ccc(c(c1)C)S(=O)(=O)[N-]c2cc(cnc2N3CC[NH2+]CC3)C(=O)N4CCCC4	0
Cc1c(c(sn1)NC)c2nc(no2)C3CCCCCC3	0
Cc1cc(c(n1c2cccc(c2)S(=O)(=O)N3CCOCC3)C)/C=N/Nc4cccc[nH+]4	0
O=C(CN1CCC(C(=O)c2ccc(F)cc2)CC1)NCc1nc2c(c(=O)[nH]1)CCCC2	1
C1CC[C@@]23[C@@H]4CCC[C@@H]([C@@H]4NC=O)C[C@]2(C1)O3	0
c1cc2c(cc1NC(=O)CN3CCN(CC3)S(=O)(=O)c4ccc(s4)Br)OCO2	0
Cc1nnc(n1Cc2ccccc2)SCC(=O)Nc3cc(ccc3n4cncn4)C(F)(F)F	0
Cc1cc(on1)CNc2c3c(c(sc3ncn2)C)C	0
c1cc(c(c(c1)Cl)[N-]S(=O)(=O)c2ccc(=O)[nH]c2)Cl	0
O=c1[nH]c(-c2ccc3c(c2)OCO3)nc2ccccc12	1
CC1(C)CC2=NNC(=O)c3cc(F)cc4[nH]c(c2c34)C1	1
Cc1cccn2c1nc(cc2=O)CN3CCNC[C@@H]3C#N	0
CN(Cc1[nH]cc[nH+]1)C(=O)Nc2cc(ccc2Cl)Cl	0
Cc1ccc(cc1)[C@@]2(C(=O)N(C(=O)N2)CC(=O)OCc3cc(=O)oc4c3ccc(c4)Br)C	0
Cc1cc(cc(c1)Nc2c(c(nc3n2ncn3)C)C)C	0
C[C@H]1CC(=O)N[C@H](C(=O)N1C(C)C)C2CCCCC2	0
CS(=O)(=O)NC1CCN(CC1)c2cnn(c(=O)c2Cl)c3ccc(cc3)Cl	0
c1ccc2c(c1)ncn2C[C@@H](c3ccc(cc3)F)NC(=O)NC[C@H]4COc5ccccc5O4	0
CC(=O)[C@H]1[C@H]([C@@]2(c3ccccc3NC2=O)[C@@H]4N1c5ccc(cc5C=C4)Cl)C(=O)c6cccc(c6)[N+](=O)[O-]	0
CC[C@@H](CNC(=O)N1CCC[C@H]([C@@H]1C)C)C(=O)[O-]	0
[H]/N=C\1/C(=C\c2cc(c(c(c2)Cl)OCCOc3ccc(cc3)C)OC)/C(=O)N=C4N1N=C(S4)CC	0
C[C@H]1[C@@H]([C@H]([C@H]([C@@H](O1)Oc2ccc3ccc(=O)oc3c2c4c(ccc5c4oc(=O)c(c5)Oc6ccc7ccc(=O)oc7c6)O)O)O)OC(=O)C	0
Cc1ccc(cc1C)S(=O)(=O)Nc2ccc(cc2)C[NH+]3CCN(CC3)c4ccccn4	0
c1cc(ccc1c2c(nc([nH]2)c3ccc(cc3)Br)c4ccncc4)F	0
c1c(csc1c2c3c(nc(c2C#N)N)CCOC3)CN4CCCCC4	0
COc1ccc(cc1OC)S(=O)(=O)N(CC(=O)Nc2ccc(cc2)S(=O)(=O)/N=c/3\[nH]ccs3)c4ccccc4	0
Cn1cc(cc1C(=O)Nc2ccc(c(c2)[O-])Cl)Cl	0
CCn1c(c(c([nH+]1)C)NC(=O)Nc2nnc(s2)C(F)(F)F)C	0
CC(C)C(=O)Nc1nc2cc3c(cc2s1)OCCO3	0
c1ccc(cc1)/N=N\c2ccc(cc2O)[O-]	0
O=C(CN1CCC2(CC1)Cc1cc(F)ccc1C2=O)N(Cc1nc2c(c(=O)[nH]1)COCC2)CC1CC1	1
c1cc(c(cc1F)Br)Nc2cc(ccn2)C#N	0

Cn1c2ccc(cc2sc1=O)S(=O)(=O)NC3CCCCCC3	0
Cc1ccc2c(=O)[nH]c(-c3ccc(C(C)(C)O)cc3)cn12	1
c1ccc2c(c1)cc(o2)Cn3c(c(cn3)I)N	0
COc1cc(cc(c1O)OC)/C=C/2\C(=O)NN(C2=O)c3ccc(cc3)Cl	0
Cc1cc2c(cc1C)S(=O)(=O)[C@@H]([C@H]([C@@H]2NC(C)C)C)C	0
Cc1cc(c(n1c2cccc(c2)Cl)C)/C=C/3\C(=[NH2+])N4C(=NC3=O)SC (=N4)S(=O)(=O)C	0
Cc1ccc2c(c1)CCCN2C(=O)c3c(n[nH]c3N)C	0
Cc1c([nH]c(c1C(=O)C2=C(C(=O)N([C@@H]2c3ccc(cc3)OC)CC [NH+](C)C)[O-])C)C	0
CCOc1ccc(cc1OCC)[C@@H]2C(=C(C(=O)N2CCCn3cc[nH+]c3) [O-])C(=O)c4c(nc(s4)C)C	0
Cc1ccc(c(c1)Br)NC(=O)N[C@@H]2CCS(=O)(=O)C2	0
Cc1c(n2ccccc2n1)CNc3c(nn(c3C)C)C	0
CC1CCC(CC1)(CO)NC(=O)N2CCCCCC2	0
CCc1ccc(cc1)S(=O)(=O)Nc2cc(nn2[C@@H]3CCS(=O)(=O)C3)c4 ccco4	0
c1ccn2c(c1)nc(c2CN)Oc3cc(ccc3Cl)Cl	0
Cc1cccc(c1)NC(=O)c2cnn3c2[NH+]=C(C[C@H]3c4cccc(c4)Br)C(=O)[O-]	0
Cc1ccc2cc(c(nc2c1)C)C(=O)Nc3ccc4c(c3)n(c(=O)n4C)C	0
CCc1ccc(cc1)N2C[C@@H](CC2=O)c3nnc(s3)NC(=O)[C@H]4C C(=O)N(C4)c5ccc(cc5)C	0
Cn1c2c(c(=O)n(c1=O)C)n(cn2)C[C@@H](CN3CCN(CC3)Cc4cc ccc4Cl)O	0
CS(=O)(=O)Nc1cccc(c1)NC(=O)c2nnn(n2)c3ccc(cc3)Br	0
c1ccc2c(c1)c(cn2C(=O)N3CCOCC3)/C=N\NC(=O)c4cc5cc(ccc5o4)Br	0
Cc1cc(cc(c1C)S(=O)(=O)NC)C(=O)N2CCC[C@@H]2c3ccc4c(c3) OCCCO4	0
Cc1c(c(n[nH]1)C)S(=O)(=O)N2CCCN(CC2)S(=O)(=O)c3ccccc3[N+](=O)[O-]	0
CS(=O)(=O)Nc1ccc(cc1)N[C@@H]2CCCCc3c2cccc3	0
Cc1ccc2c(cc(=O)oc2c1)CSc3nc4c(c(c(s4)C)C)c(=O)n3C	0
Cc1ccc(cc1)S(=O)(=O)Nc2c3ccsc3ncn2	0
Cc1ccc(cc1C)NS(=O)(=O)C2=C(N=N[C@@H]2C)C	0
C[C@@H]1C[C@@H](CN(C1)c2cc(c(cc2F)[N+](=O)[O-])C(=O)[O-])C	0
COc1c(cc(cc1Br)Cl)N[C@@H]2C[C@H]3[C@@H]2C=CC3	0
O=C(CCc1cccnc1)N(Cc1nc2c(c(=O)[nH]1)COCC2)Cc1cccs1	1
C[C@H]1CCN([C@@H](C1)C)C(=O)Cl	0
O=C(CCc1ccccc1)N(Cc1nc2c(c(=O)[nH]1)COCC2)Cc1cncs1	1
CCc1nn2c(=O)cc(nc2s1)COc3ccc(cc3OC)c4[nH]c5c(c6c(s5)CCC C6)c(=O)n4	0
c1c[nH]c2c1cc(c(n2)F)Br	0
c1cc(cc(c1)NC(=O)C[C@@H]2C(=O)N/C(=N\N=C/c3ccco3)/S2) C(F)(F)F	0
C[C@H]1CSCCN1c2nc3ccc(cc3o2)N	0
COc1ccc2c(c1)CC1(CCN(CC(=O)N(Cc3nc4c(c(=O)[nH]3)COCC 4)CC3CC3)CC1)C2=O	1
CS(=O)(=O)C1=[NH+]SC2=NC(=O)/C(=C\c3ccc(cc3)OC(=O)c4c	0

cccc4)/C(=[NH2+])N12	
C[NH+]1CCC(=NNC(=O)c2cc(c(c(c2)O)O)O)CC1	0
Cc1ccc(c(c1)OCC(=O)NCc2[nH+]c3ccccc3n2CCCCOc4cccc(c4C)C)C	0
Cc1cc2c(c(c(c(=O)o2)Cc3ccccc3)C)c(c1)OCC(=O)N4Cc5c(c6ccccc6[nH]5)C[C@H]4C(=O)OC	0
c1ccc(cc1)c2cnn(c2)c3ccc(cc3C#N)N	0
Cc1ccc(cc1)CN2c3ccccc3C(=C(S2(=O)=O)/C=N/CCc4ccc(c(c4)OC)OC)[O-]	0
Cc1c(sc(n1)N)C(=O)N(CC(C)C)C(C)C	0
c1ccc(cc1)n\2c(=O)/c(=C/c3ccccc3[N+](=O)[O-])/s/c2=C(/C#N)\c4nnc5n4CCCCC5	0
CC1CC2=NNC(=O)c3cccc4[nH]c(c2c34)C1	1
c1ccc(cc1)c2[nH+]ccn2CCCNC(=O)C(=O)NCc3ccc4c(c3)OCO4	0
Cc1c2c(n(n1)c3ccccc3)NC4=[NH+][C@H](NC(=O)C4=C2)c5cccs5	0
COCCOC(C)(C)c1ccc(-c2nc3ccc(F)cc3c(=O)[nH]2)cc1	1
Cc1ccc(c(c1)F)NC(=O)NCc2ccc(nc2)N3CCO[C@H](C3)C	0
[H]/N=C\1/C(=C\c2ccc(c(c2)OC)OCCOc3cc(cc(c3)CC)C)/C(=O)N=C4N1N=C(S4)C(C)C	0
Cc1ccnc(n1)SCCCn2c3c(=O)[nH]c(=O)n(c3[nH+]c2N4CCN(CC4)C(=O)c5ccco5)C	0
CC(C)Sc1ccc(cc1)Cc2nnc(o2)NC(=O)[C@@H]3CCCN3S(=O)(=O)c4ccc(cc4)Cl	0
O=c1[nH]c(-c2ccc(C(F)(F)F)cc2)nc2c1CCOC2	1
COc1cc(cc(c1O)Br)[C@@H]2c3c(=O)c4cc(ccc4oc3C(=O)N2c5nncs5)F	0
CCc1c(c(n(n1)C)N)c2ccc(cc2)OC(F)F	0
CSc1ccccc1NC(=O)c2cc(n(n2)[C@H]3CCS(=O)(=O)C3)c4ccco4	0
COc1cc2c(cc1OC)nc([nH]2)c3cc4c(s3)CCC4	0
CC(C)(C)OC(=O)N1CCC2(CCN(C(=O)c3cc(Cc4n[nH]c(=O)c5ccccc45)ccc3F)C2)C1	1
CC(C)CC1=C[C@H](N=N1)[C@@H]2Nn3c(nnc3S2)c4ccccn4	0
CCc1nc2n(n1)c(c(s2)[C@H](c3cc(c(c(c3)OC)OC)OC)[NH+]4CCN(CC4)c5ccc(cc5)F)[O-]	0
C[C@H]1[C@@H](Oc2ccc(cc2N1)Br)C	0
Cc1c(cccc1[N+](=O)[O-])C(=O)NC[C@@](C)(c2cc3ccccc3s2)O	0
O=C1NC(c2ccc(OCc3ccccc3)cc2)Nc2ccccc21	1
Cc1c(c(n(n1)c2nc(cs2)c3ccc(cc3)OC)O)[C@@H](c4ccccc4)[C@@H](C#N)C(=O)N	0
O=C(CCCC1CCCCC1)Nc1nnc2n1CCS2	1
Cc1cc(c2c(c1)sc(n2)N(CCC[NH+](C)C)C(=O)C3CCN(CC3)S(=O)(=O)c4cccs4)C	0
Cc1c(n2ccccc2n1)CN3CCN(CC3)C(=O)[C@H](CC4=c5ccccc5=[NH+]C4)NC(=O)C	0
Cc1c(c(=O)n(n1C)c2ccccc2)NC(=O)[C@H](C)Sc3ccc(cn3)S(=O)(=O)N(C)C	0
c1ccnc(c1)/C=N/NC(=O)CSc2nnc(n2c3ccc(cc3)Br)c4ccncc4	0
CCC(=O)Nc1ccc(c(c1)C)S(=O)(=O)NC[C@H](c2ccco2)[NH+]3CCOCC3	0
CCOc1cc(ccc1O)[C@H]2c3c4c(c(n3CCO2)c5ccccc5)c(=O)n(c(=O)n4C)C	0

**c1ccc(cc1)[C@H](CN2CCCC2=O)NC(=O)Nc3ccc([nH+]c3)N4CCCCC4**	0
**c1ccc2c(c1)c(nnc2[O-])C(=O)NCc3ccc(s3)Cl**	0
**Cc1cc(nc(n1)C)SCC(=O)Nc2c(c3c(s2)C[C@@H](CC3)C)C(=O)OC**	0
**COc1cccc(CN(Cc2nc3c(c(=O)[nH]2)COCC3)C(=O)CN2CCC(C(=O)c3ccc(F)cc3)CC2)c1**	1
**Cc1c(c(n(n1)C)C(=O)NNC(=O)c2ccc(s2)Br)[N+](=O)[O-]**	0
**CNC(=O)[C@H](C1CCCC1)N2CCN(CC2)C(=O)Nc3cccc(c3)F**	0
**Cc1ccc(-c2noc(CSc3nnc(C)n3-c3ccc(C)cc3)n2)cc1**	1
**CC[NH+](CC)CCNC(=O)C(=O)Nc1c2c(nn1c3cccc(c3)Cl)CS(=O)(=O)C2**	0
**CC[C@@H](C(=O)Nc1ccc(cc1)S(=O)(=O)N2CCCC2)Sc3cc(ncn3)C**	0
**Cc1csc(n1)[C@@H](C)NC(=O)N2CCC(CC2)(C)C(=O)[O-]**	0
**c1ccc(cc1)CN(Cc2ccccc2Cl)c3c4c([nH+]cn3)nc[nH]4**	0
**CC(C)c1ccc(-c2cc(=O)c3ccccc3o2)cc1**	1
**C1C[C@@H]2CCCN([C@H]2C1)C(=O)Cl**	0
**COc1ccccc1/C=C(/c2ccccc2)\C(=N\S(=O)(=O)c3ccc[nH+]c3)\[O-]**	0
**C[C@@H]1CC[C@H](CN1Cc2nc(no2)c3ccccn3)C[NH3+]**	0
**N#Cc1c(Cn2c(=O)n(C[C@H]3CC[C@H](C(=O)O)CC3)c(=O)c3c(F)cccc32)cccc1C(F)(F)F**	1
**Cc1cc(cc(c1)c2nnc3n2c(nc(c3)Cl)C)C**	0
**c1c(cc(c(c1O)O)O)c2c(c(=O)c3c(cc(cc3o2)O)[O-])O[C@H]4[C@@H]([C@H]([C@@H](O4)CO)O)O**	0
**Cc1cc(c(c(c1)C)NC(=O)CN(C)C(=O)/C=C/c2ccc(c(c2)OC)OCc3c(noc3C)C)C**	0
**Cc1c(c(=O)n(n1C)c2ccc(cc2)S(=O)(=O)Nc3ccc4c(c3)n(c(=O)n4C)C)C(C)C**	0
**CCn1c(c(c([nH+]1)C)CCNC(=O)[C@H](C)C2CCN(CC2)S(=O)(=O)c3ccc(s3)Cl)C**	0
**C[C@H](C[C@@](C)(C#N)N)OC**	0
**C[C@@H]1C[C@@H](C[NH+](C1)c2c(n3ccsc3n2)C(=O)[O-])C**	0
**C1CC[C@H]([C@H](CC1)O)[C@@H]2CCCC2=O**	0
**c1cc[nH+]c(c1)CNC(=O)c2c(=O)n(c(=O)n(n2)c3ccc(cc3)C(F)(F)F)Cc4ccc(cc4)F**	0
**CC(=O)Nc1ccc(c(c1)NCc2cc(cc3c2OCOC3)Cl)F**	0
**c1ccc(c(c1)/C=N\c2cc(cc(c2)F)F)[O-]**	0
**c1cc(ccc1NC(=O)CCn2cc(cn2)Cl)S(=O)(=O)Nc3nccs3**	0
**CCc1c(nc(nc1NC)N2CCN(CC2)C)C**	0
**CC(C)(C)OC(=O)NCC(=O)/[NH+]=C/1\N([C@@H]2CS(=O)(=O)C[C@@H]2S1)CCc3ccc(cc3)F**	0
**CCCc1c2c(n(n1)C)NC3(CCCC3)CN2**	0
**c1cc(c(cc1CNC(=O)c2ccc(c(c2)S(=O)(=O)N3CCCCC3)F)F)n4ccnc4**	0
**CC(C)c1ccc(cc1)n2c(=O)n(c(=O)c(n2)C(=O)NCc3cccc[nH+]3)Cc4ccccc4**	0
**c1cc[nH+]c(c1)CN2CC[NH+](CC2)C[C@@H](CC(=O)Nc3ccc4c(c3)OCO4)C(=O)[O-]**	0
**Cc1ccc(cc1)S(=O)(=O)NCC(=O)NCC(=O)Nc2nc3c(s2)CCCCC3**	0
**c1ccc(cc1)c2cnc(n2c3ccccc3)SCC(=O)Nc4ncc(s4)S(=O)(=O)c5ccc(cc5)[N+](=O)[O-]**	0

**Cc1ccc2c(c1)c(c(o2)C(=O)NCc3ccc4c(c3)n(c(=O)n4C)C)C**	0
**C[C@H](C(=O)NCCN1C(=O)/C(=C\c2ccc(cc2Cl)Cl)/SC1=O)N3C(=O)CCC3=O**	0
**c1cc(ccc1c2ccnc3c2cc[nH]3)[C@@H](CN4CCOCC4)O**	0
**CCS(=O)(=O)C1=NN2C(=[NH2+])/C(=C\c3cc(n(c3C)c4ccc(c(c4)C)C)C)/C(=O)N=C2S1**	0
**Cc1cc(cc(c1Oc2c(cncn2)Cl)N)Br**	0
**COC(=O)CNC(=O)c1cn(cc(c1=O)C(=O)NC2CCCCCCC2)CCc3cccc[nH+]3**	0
**CCn1ccc(=O)c(c1)NC(=O)c2cc(=O)[nH]c3c2cc(cc3)S(=O)(=O)N(CC)CC**	0
**Cc1ccc(cc1)CNC(=O)[C@]2(CN(CC(=O)N2Cc3ccc(cc3)N(C)C)S(=O)(=O)C)C**	0
**CC(C)(C)C1CCN(CC1)c2nc3c(s2)[C@H](CCC3)O**	0
**CN[C@H]1CCCc2c1sc(n2)[C@@H]3CCOc4c3cccc4**	0
**Cc1ccc(cc1)S(=O)(=O)O[C@H]2[C@@H]3[C@H](CC[C@@H](O3)c4ccccc4)O[C@@H]([C@@H]2OS(=O)(=O)c5ccc(cc5)C)OC**	0
**c1cc(ccc1C(=O)Nc2ccc(cc2)S(=O)(=O)/N=C\3/CCCN3)Br**	0
**c1cc(sc1)/C=C\2/C(=O)N(C(=O)S2)CCNC(=O)c3ccc(c(c3)[N+](=O)[O-])N4CCOCC4**	0
**COc1cc(cc(c1[O-])Br)[C@@H](C(F)(F)F)O**	0
**C[C@H]1CCC[C@H]([C@@H]1C)NS(=O)(=O)c2cccc3c2nsn3**	0
**O=C1NN=C2CCCc3[nH]c4cc(F)cc1c4c32**	1
**CC(C)c1ccc(cc1)[C@H]2c3c(c4ccccc4[nH]3)C[C@@H]5N2C(=O)N(C5=O)c6ccccc6C(=O)NC[C@H]7CCCO7**	0
**CCn1c2ccc(cc2nc1CCC(=O)N[C@H](C#N)c3ccc(cc3)F)S(=O)(=O)N(C)C**	0
**c1ccc2c(c1)c3cc(ccc3[nH]2)[C@H]([C@H]4COCCO4)Cl**	0
**C[C@H]1CCCN([C@@H]1C)c2cncc(n2)N**	0
**c1ccc2c(c1)/C(=N\O)/CCN2Cc3ccc(cc3)O**	0
**Cc1ccc2c(=O)[nH]c(-c3ccc([C@@](C)(O)CCO)cc3)cn12**	1
**CC(=O)c1cccc(c1)NC(=O)c2ccc(cc2)C/N=c\3/c(c(c3=O)O)N4CCCC4**	0
**CCc1c(c(on1)CC)CC(=O)N[C@@H]2CCC[C@H]([C@@H]2C)C**	0
**Cc1cc2c(c(c1)Cl)OC[C@H]2NC**	0
**CC(C)S(=O)(=O)c1ccc(cc1)C(=O)/N=c\2/n(c3cc(ccc3s2)[N+](=O)[O-])CC=C**	0
**Cc1c(c(n(c(=O)c1C#N)C)N2CCC(CC2)C(=O)N)/C=C\3/C(=O)N(C(=S)S3)CCc4ccccc4**	0
**COc1ccc(-c2cc3c(c(=O)[nH]2)CCCN3C)cc1**	1
**C[C@@H]1CCC[NH+](C1)CCCNC(=O)CN2c3ccc(cc3CCC2=O)S(=O)(=O)N4CCCCC4**	0
**CC[C@@H]1CCCC[C@H]1NC(=O)N2CC[C@@H](C2)C(=O)[O-]**	0
**Cc1cccc2c(O)nc(-c3ccc(C(F)(F)F)cc3)nc12**	1
**Cc1cccn2c1nc(c(c2=O)/C=C\3/C(=O)N(C(=S)S3)C(C)C)NCCCn4ccnc4**	0
**CCCCn1cc(c(=O)c2c1cc(c(c2)F)N3CC[NH+](CC3)CCC)S(=O)(=O)c4ccc(cc4)C**	0
**C[C@@H](c1ccc(cc1)C#N)NC(=O)N(C)Cc2ccc3c(c2)OCO3**	0
**Cc1cc(sc1Br)c2[nH]c3cc(ccc3n2)Cl**	0

**CCOC(=O)[C@@H]1C(=c2cc(c(cc2=[NH+]1)OC)OC)NC(=O)CCN3CCN(CC3)c4ccccc4OC**	0
**c1csc(c1Br)c2nc(no2)N**	0
**CCOC(=O)C1=C(N2C(=O)[C@@H](SC2=C([C@@H]1c3cc(c(c(c3)OC)OC)OC)C(=O)OC)C)N**	0
**Cc1c(sc2c1c(=O)n(c(n2)SCC(=O)NCCN3C(=O)CSC3=O)C(C)C)C**	0
**C[C@H](c1ccc2c(c1)OCCO2)NC(=O)C3CCN(CC3)S(=O)(=O)c4ccc(cc4)OC**	0
**Cc1c2c(c(n(n2)c3ccccc3)n4cccc4)c(=O)n(n1)[C@@H](C)C(=O)NCC[NH+]5CCCCCC5**	0
**COc1ccc(-c2cc(=O)c3ccc(O)cc3o2)cc1**	1
**COc1cc(cc(c1OC)OC)C(=O)N2CSC[C@@H]2C(=O)NCCc3cscn3**	0
**c1cc(ccc1NC2(C3CC4CC(C3)CC2C4)C(=O)[O-])Br**	0
**C[C@H]1CC[C@@H]([C@@H](C1)N2CCCCC[C@@H]2C)C#N**	0
**CC(=O)c1cc2c(cc1NC(=O)c3nc(n(n3)c4ccccc4)c5cccs5)OCO2**	0
**CS(=O)(=O)c1ccc(-c2nnc([C@H]3C[C@H](NC(=O)c4ccccn4)C3)n2-c2ccccc2F)nc1**	1
**COc1ccc(C(=O)C2CCN(C(=O)CCCc3cn4cc(F)cc4c(=O)[nH]3)CC2)cc1**	1
**CN1[C@H]([C@H]([C@@](N(C1=O)C)(C(F)(F)F)O)C(=O)c2ccccc2)c3ccccc3[N+](=O)[O-]**	0
**C[C@H](c1nnc(n1CC=C)SCC(=O)N2CC[NH+](CC2)C)NC(=O)c3ccccc3**	0
**c1cc(sc1S(=O)(=O)/N=c/2\[nH]ccs2)Cl**	0
**CC12C[C@H]3C[C@@H](C1)CC(C3)(C2)/C(=N/O)/C=N/O**	0
**C[NH+]1[C@@H]2CCN([C@@H]2C(=O)NC[C@@H]1CCC(=O)N3CCC[C@@H]3COC)Cc4cccc(c4)Cl**	0
**CCc1nc2n(n1)C[C@@H](CC2)Nc3c4ccccc4ncn3**	0
**CCSc1nnc(s1)NC(=O)CSc2nnc(s2)NC(=O)c3ccc(c(c3)C)C**	0
**CC(C)CN1[C@H]([C@@]23C=C[C@@H](O2)[C@@H]([C@@H]3C1=O)C(=O)NC4CC4)C(=O)NC5CCCCC5**	0
**Cc1ccc(cc1Cl)[C@@H](c2ccc3c(c2)OCCCO3)O**	0
**[H]/N=C\1/N(C(=O)C[C@@H](S1)C(=O)Nc2ccc(cc2)Br)/N=C\c3ccccc3**	0
**COc1ccc2cc([nH]c2c1)C(=O)N3CCc4c(sc(n4)NC(=O)C5CC5)C3**	0
**Cc1cccc(-c2nnc([C@H]3C[C@H](NC(=O)c4ccnc5cccnc45)C3)n2-c2ccccc2F)n1**	1
**C1CCC2(C1)C[C@@H](CCO2)c3n[nH]nn3**	0
**CCCc1c(nn(c1N)[C@@H]2CCS(=O)(=O)C2)c3cccs3**	0
**COc1ccc(cc1)/N=C\2/N(C(=O)C[C@@H](S2)C(=O)Nc3ccc(cc3)F)Cc4ccc(c(c4)OC)OC**	0
**c1cc[nH+]c(c1)N2CC[C@H](C2)NC(=O)CNS(=O)(=O)c3cccc(c3)Cl**	0
**CCn1c(nnc1SCc2nc3c4c(c(sc4ncn3n2)C(=O)OC)C)c5cc(n(n5)C)C(F)(F)F**	0
**Cc1c(sc(n1)C)C(=O)C2=C(C(=O)N([C@@H]2c3ccccc3Cl)CCCn4cc[nH+]c4)O**	0
**Cc1cccc(c1)NC(=O)N2CCN(CC2)Cc3nc4cc(ccc4n3C)C(=O)N(C)**	0

C	
C[C@H]1[C@@H]([C@@H](C[C@@H](O1)O[C@@H]2CC[C@@]3([C@@H](C2)CC[C@@H]4[C@@H]3CC[C@@]5([C@@]4(C[C@@H]([C@@H]5C6=CC(=O)OC6)OC(=O)C)O)C)C)OC)O	0
C[C@@H]1CCC[NH+]([C@@H]1C)[C@@H]2CC3(CC[C@@H]2O)OCCO3	0
COC(=O)[C@@H](Cc1ccc[nH+]c1)NC(=O)[C@H]2CN(CCN2S(=O)(=O)C)C(=O)Nc3ccccc3	0
C[C@H]1C[C@](CCO1)(C2(CCC(CC2)C(C)C)C#N)O	0
CC(C)c1[nH]nc(n1)c2cc(c(cc2F)F)N	0
Cc1cc(c(o1)C)C(=O)N2CCN(CC2)C(=O)Nc3ccc(cc3)CC(=O)N4CCCC4	0
c1ccc(cc1)c2[nH]cc([nH+]2)CO	0
C[C@@]1(C(=O)N(C(=O)N1)Cc2ccc(cc2Br)F)c3ccc4c(c3)OCO4	0
c1cc(c(c(c1)Cl)Cn2ccc3c2ncnc3N)Cl	0
C[C@@H](c1ccc(cc1)N2CCCCCCC2)O	0
c1cc(c(cc1Br)F)NC(=O)c2ccc(nc2)F	0
c1cc(ccc1N2C[C@@H](CC2=O)C(=O)NCc3cc(on3)c4ccc5c(c4)OCO5)Cl	0
c1cc(cc(c1)Br)c2cc(n[nH]2)C(=O)N3[C@@](CC(=N3)C(F)F)(C(F)F)O	0
c1cc2c(cc1O[C@H]3COCC[C@@H]3O)OCO2	0
c1cc[nH+]c(c1)CCn2cc(c(=O)c(c2)C(=O)N3C[C@@H]4CC[C@H]3C4)C(=O)NC5CCCCCCC5	0
CCc1nc2n(n1)c(c(s2)[C@@H](c3cc(c(c(c3)OC)OC)OC)[NH+]4CCN(CC4)c5ccc(cc5)F)[O-]	0
Cc1cc(ccc1[N-]S(=O)(=O)c2ccc3c(c2)CCC(=O)N3)Cl	0
C[C@H](C(=O)Nc1ccc2c(c1)OCCO2)[NH+]3CCc4c(ccs4)C3	0
Cc1cc2c(cc1Cl)c(cc(=O)o2)CS/C(=N/C)/N	0
Cc1cc(n2c(n1)c(cn2)C(=O)N[C@@H]3CCCc4c3cnn4Cc5ccc(cc5)Cl)C	0
Cc1ccc(c(c1)n2c(c3c(n2)CCC3(C)C)N)C	0
Cc1c(sc(n1)C(C)(C)NC(=O)NCc2cnn(c2)C)C	0
O=c1[nH]c(-c2ccc(OC(F)(F)F)cc2)nc2c1COCC2	1
CCOc1ccc(cc1)/C(=N/NC(=O)c2cc(n[nH]2)c3c(nn(c3C)c4ccccc4)C)/C	0
C[C@H](c1cccs1)Nc2ccc(c(c2F)F)C(=O)[O-]	0
O=C1NC(c2ccccc2)Nc2ccccc21	1
O=C(CCc1ccc(F)cc1)N(Cc1nc2c(c(=O)[nH]1)COCC2)Cc1cccs1	1
CC(C)(O)c1ccc(-c2nc3c(c(=O)[nH]2)COCC3)cc1	1
c1c(cc(c(c1F)NC(=O)Cn2cc(cn2)N)F)Br	0
O=C1N(CCO)c2cccc(F)c2C12CCN(c1nc3c(c(=O)[nH]1)CCCC3)CC2	1
CC(=O)Nc1ccc(cc1OC)NC(=O)C2=Cc3cc(ccc3OC2)F	0
COc1cccc(c1)[C@H]2CN=C(N2c3cccc(c3)Br)N	0
CCOc1cc(ccc1OC(=O)C)[C@@H]2C3C(=NC(=C2C(=O)OCCOC)C)C[C@H](CC3=O)c4ccc(c(c4)OC)OC	0
CCn1ccc2c1ccc(c2)S(=O)(=O)N3CCC(CC3)C(=O)NCc4cccc[nH+]4	0
Cc1ccc(cc1)n2c(nnc2S[C@@H](C)C(=O)Nc3ccc4c(c3)OCCO4)N5CCOCC5	0

Cc1ccsc1[C@H](C)Nc2cccc(c2)c3nnnn3C	0
CC1=C2[C@@H](C3=C(C[C@@H](CC3=O)c4cccs4)N[C@@H]2N=N1)c5ccc(c(c5)O)OC	0
Cc1cccc2c(=O)[nH]c(-c3ccc(Cl)cc3)nc12	1
c1cc(ccc1/C=C\2/C(=O)N(C(=S)S2)[C@@H]3CC(=O)N(C3=O)CCCCC(=O)O)Cl	0
Cc1ccc(C(=O)C2CCN(CC(=O)NCc3nc4c(c(=O)[nH]3)COCC4)CC2)cc1	1
CC(C)Cc1nnc(s1)NC(=O)C2CCN(CC2)S(=O)(=O)c3cc(n(c3)C)C(=O)N	0
c1c(cc(c2c1OCCCO2)Cl)c3ncc(c(n3)N)I	0
Cc1cccc(c1)Cc2c(nc(nc2N3CCN(CC3)C[C@@H](CN4CCOCC4)O)C)C	0
O=C(c1ccc(F)cc1)C1CCN(CC(=O)N(CCF)Cc2nc3c(c(=O)[nH]2)COCC3)CC1	1
NC(=O)c1cccc2cn(-c3ccc([C@@H]4CCCNC4)cc3)nc12	1
Cc1c(c(n([nH+]1)[C@@H]2CCS(=O)(=O)C2)C)N3CCN(CC3)C(=O)Nc4cc(ccc4OC)Cl	0
Cc1cccc(-c2nnc([C@H]3C[C@H](NC(=O)c4ccccn4)C3)n2-c2ccccc2F)n1	1
CC(C)C1CC2=NNC(=O)c3cc(F)cc4[nH]c(c2c34)C1	1
COc1ccc(C(=O)C2CCN(C3CCN(Cc4nc(C)cc(=O)[nH]4)C3=O)CC2)cc1	1
Cc1ccc(cc1)S(=O)(=O)N2CCC(CC2)C(=O)N/N=C/3\CCc4c3cccc4	0
COc1cc2c(cc1O[C@H]3[C@@H]([C@H]([C@@H]([C@H](O3)CO[C@@H]4[C@@H]([C@](CO4)(CO)O)O)O)O)O)occ(c2=O)c5ccc6c(c5)OCO6	0
CC(C)c1nnc(s1)NC(=O)c2cc(nn2C)c3ccc(cc3)Cl	0
C[C@H](c1ccccc1)Nc2c(nc(o2)c3ccc(c(c3)OC)OC)S(=O)(=O)c4ccc5c(c4)OCCO5	0
Cc1c(c(n(n1)C(C)C)NC2(CCCC2)CO)N	0
CN1[C@@H]([C@H](c2cc(c(cc2C1=O)OC)OC)C(=O)Nc3ccc(cc3F)F)c4ccc(c(c4)OC)OC	0
Cc1c/c(=N/S(=O)(=O)c2cc(ccc2Cl)F)/cc[nH]1	0
c1ccc2c(c1)nc(n2CC(=O)NNC(=O)CSc3ccccc3F)c4cscn4	0
C[NH2+][C@H]1COC[C@H]1/C(=N/c2c(cc(c3c2nsn3)Cl)Cl)/[O-]	0
COc1ccc(C(=O)C2CCN(CC(=O)N(Cc3nc4c(c(=O)[nH]3)CCCC4)CC3CC3)CC2)cc1	1
c1cc2c(cc1Cn3ccnc3c4cc5n(n4)CCCNC5)nsn2	0
O=C(CCc1cccc(F)c1)N(Cc1nc2c(c(=O)[nH]1)COCC2)Cc1cccs1	1
Cc1ccc(cc1)S(=O)(=O)/C(=C/c2c(nc3ccccn3c2=O)Oc4ccc(cc4C)C)/C#N	0
COc1ccc(C(=O)C2CCN(C3CCN(Cc4nc(C)c(C)c(=O)[nH]4)C3=O)CC2)cc1	1
c1cc2n(c(=O)c1)C[C@H]3C[C@@H]2CN(C3)C(=O)Nc4ccc(cc4)Cl	0
Cc1c(cnn1c2ccccc2)c3ccnn3C(=O)Nc4ccccc4	0
Cc1ccc(c(c1)Br)Nc2ccnc(n2)Cl	0
Cc1cc(F)cc2c(=O)[nH]c(-c3ccc(C(C)(C)O)cc3)nc12	1
C[C@H]1[C@@H](S(=O)(=O)CCN1C(=O)NC2CCC2)C	0

**C[C@@H]1C[S@@](=O)CC[NH+]1CC(=O)Nc2cccc(c2)S(=O)(=O)/N=C\3/CCCCCN3**	0
**COc1cc(cc(c1OCc2cccc3c2cccc3)Br)/C=C\4/C(=O)NC(=O)N(C4=O)c5ccccc5**	0
**C[C@@H](C(=O)NCc1ccc2c(c1)OCO2)Sc3ccc(cn3)S(=O)(=O)N4CCN(CC4)C**	0
**C[C@@H]1C[NH+](CC[C@@H]1N(C)C(=O)N[C@@H]2CCC(c3c2cccc3)(C)C)C**	0
**Cc1ccc(NC(=O)[C@H]2CC[C@H](Cn3c(=O)c4c(C)cccc4n(Cc4ccc(C(F)(F)F)c4C#N)c3=O)CC2)cn1**	1
**CC1(C)OC(=O)N(c2ccc(C(=O)Nc3ccnc4cccnc34)cc2)[C@H]1c1ccccc1**	1
**CCn1c2ccc(cc2nc1SCc3cc4c(c(c3)Cl)OCCCO4)S(=O)(=O)N**	0
**C[C@H]1CN(C[C@@H](O1)C)c2c(c(=O)n(c(=O)[nH]2)C)Cc3ccc(cc3)Br**	0
**c1c(cc(cc1F)Br)c2nc(on2)[C@@H]3COCC[NH2+]3**	0
**Cc1ccc(cc1C)NC(=O)Nc2cccc(c2)C#N**	0
**COc1cccc2c1N[C@@H]([C@@H]3[C@@H]2C=CC3)c4c(cccc4Cl)Cl**	0
**Cc1[nH+]c2ccccc2c(n1)N3CCN(CC3)C(=O)C(=O)c4c[nH]c5c4c(cnc5n6cnc(n6)C)F**	0
**CC[C@@H]1CCC[C@@](C1)(C(=O)[O-])Nc2ccccc2Cl**	0
**O=C1CCN(c2ccccc2C(=O)NCCc2ccc(Br)cc2)C(=O)N1**	1
**CC(C)(C)c1nc(sn1)N2CCCC[C@H]2CC(=O)[O-]**	0
**CN(Cc1nc2c(c(=O)[nH]1)COCC2)C(=O)CN1CCC(C(=O)c2ccc(F)cc2)CC1**	1
**CC1(CCCC[C@H]1NC(=O)[C@@]2(CCNC2)C(F)(F)F)C**	0
**Cc1cc(c(n1c2cc(ccc2Cl)Cl)C)/C=N\NC(=O)C(=O)NC[C@H]3CCCO3**	0
**CN(C)c1ccc(cc1)[C@H]2Nc3ccc(cc3-c4c(nc(nn4)SC)O2)Br**	0
**CCC[C@@H](c1cccs1)Nc2nc3ccc(cn3n2)F**	0
**Cc1[nH+]ccn1c2ccc(cn2)NC(=O)c3c(ccs3)S(=O)(=O)N4CCCCC4**	0
**CN1c2ncccc2C(=O)NC1c1ccc(Cl)cc1Cl**	1
**C[C@H](C(=O)Nc1c(cc(cn1)Cl)Cl)Sc2ccc(cn2)S(=O)(=O)N3CCN(CC3)C**	0
**COc1ccc(cc1OC)CC/[NH+]=C(/c2ccc(c(c2)[N+](=O)[O-])N3C[C@H]4C[C@@H](C3)c5cccc(=O)n5C4)\O**	0
**COc1ccc(c2c1cccc2)C[C@]3(CCC(=O)N3)CCC(=O)N4CCC(=O)NCC4**	0
**N#Cc1ccc(Oc2ccc(NC(=O)CCSc3nc4ccccc4c(=O)[nH]3)cc2)cc1**	1
**CC1(C)CCc2[nH]c3cccc4c3c2C1=NNC4=O**	1
**O=C(c1ccc(F)cc1)C1CCN(CC(=O)N(Cc2ccc(F)cc2)Cc2nc3c(c(=O)[nH]2)COCC3)CC1**	1
**Cc1c(F)ccc2c1c(=O)n(C[C@H]1CC[C@H](C(=O)Nc3ccncc3)CC1)c(=O)n2Cc1cccc(C(F)(F)F)c1C#N**	1
**CSc1ncc(c(n1)/C(=N/c2nc3ccc(cc3s2)S(=O)(=O)C)/[O-])Br**	0
**Cc1nccn1c2ccc(nn2)C(=O)Nc3ccc(cc3)S(=O)(=O)/N=C(/C)\[O-]**	0
**O=C(Nc1cccc2cccnc12)c1ccc(N2C(=O)[C@@H]3[C@H](C2=O)[C@@H]2C=C[C@H]3C2)cc1**	1
**COc1ccccc1N1CCN(c2nc3c(c(=O)[nH]2)CNCC3)CC1**	1
**CC1(Cc2c(sc(n2)N3CCCOCC3)[C@H](C1)NC)C**	0
**Cc1c(c2ccccc2[nH]1)C(=O)COC(=O)c3ccc(c(c3)S(=O)(=O)N4CC**	0

OCC4)F	
Cc1c(c(=O)n(n1C)c2ccccc2)C(c3cc4c(cc3Br)OCO4)c5c(n(n(c5=O)c6ccccc6)C)C	0
Cc1ccc(cc1NC(=O)N[C@@H](c2ccccc2)c3ccco3)F	0
Cc1c(sc2c1c(nc(n2)C[NH+]3C[C@H]4CC[C@@H]3C4)NN)C	0
Cc1cc(=O)c(nn1c2ccccc2C(F)(F)F)C(=O)Nc3ccccc3C(=O)NC	0
COc1cc(c(cc1N\2[C@H]3CS(=O)(=O)C[C@H]3S/C2=[NH+]\C(=O)CCC(=O)[O-])Cl)OC	0
Cn1c(=O)n(n(c1=O)c2ccc(cc2)C(=O)N[C@H](Cc3ccccc3)c4ccccc4)C	0
C[C@@H]1CCC[C@H](N1C(=O)COc2ccc(cc2OC)/C=N/NC(=O)[C@@H]3COc4ccccc4O3)C	0
C[C@@H]1C[NH+](C[C@@H]1C(=O)[O-])[C@@H]2CCC(c3c2cccc3)(C)C	0
c1cc2c(cc1CN3CCc4c(c(n[nH]4)c5ccc(c(c5)F)F)C3)non2	0
Cc1c(c(n(n1)CC(=O)Nc2ccc3c(c2)OCCO3)C)S(=O)(=O)N(C)Cc4ccco4	0
CC(C)CCn1c(c(c2c1nc3ccccc3n2)S(=O)(=O)c4ccc(cc4)Cl)N	0
[H]/N=C/1\[C@@]2(CCCCC2(C)C)N(C(=O)N1)C3CC3	0
CSc1ccc(cc1)N2C[C@H](CC2=O)c3nnc(o3)/N=C(/c4cccc(c4)F)\[O-]	0
Cc1cccc(C)c1CCC(=O)Nc1nnc2n1CCS2	1
O=C1Nc2ccc(Br)c2C12CCN(c1nc3c(c(=O)[nH]1)CCCC3)CC2	1
CCN1CCN(CC1)S(=O)(=O)c2ccc(cc2)NC(=O)[C@@H]3CC(=O)N(C3)C4CCCC4	0
CC(C)(C#C)NC(=O)N1[C@H]2CC[C@@H]1[C@@H](C2)C(=O)[O-]	0
c1ccc(c(c1)c2c3c(n[nH]2)C(=O)N([C@@H]3c4cccc(c4)Cl)C[C@H]5CCCO5)O	0
Cc1cc(ccc1C(=O)c2ccc3c(c2)CC(O3)(C)C)N	0
Cc1ccc(cc1F)[C@@H](C)Nc2nc(nc(n2)Cl)Cl	0
c1cc(c[nH+]c1)CNC(=O)c2c(n(nn2)c3cc(cc(c3)Cl)Cl)CSc4ncccn4	0
Cc1ccc2c(c1)c(=O)c3c(o2)C(=O)N([C@H]3c4ccc(cc4)OCC(=O)N)[C@H]5CCS(=O)(=O)C5	0
c1ccc2c(c1)N/C(=C(/C#N)\C(=O)c3ccc(s3)[N+](=O)[O-])/S2	0
c1cc(c2c(c1)oc(n2)N3CCCN4CCC[C@@H]4C3)N	0
COc1cccc2c(=O)[nH]c(-c3ccc(C)cc3)cc12	1
Cn1c(cc(=O)c(c1CN2CCN(CC2)CCOc3ccccc3)O)CN4CCCCC4	0
O=C(c1ccc(F)cc1)C1CCN(CC(=O)N(Cc2nc3c(c(=O)[nH]2)COCC3)CC(F)F)CC1	1
CC(C)(O)c1ccc(-c2cn3cccc3c(=O)[nH]2)cc1	1
CCCCc1nc(c(s1)C(=O)N2CCN(CC2)Cc3[nH+]c4ccccc4c(n3)N)C	0
Cc1c(cnn1[C@@H]2CCS(=O)(=O)C2)C(=O)Nc3ccc(c(c3)S(=O)(=O)N4CCCC4)Cl	0
CN1c2ccc(cc2CC1=O)S(=O)(=O)CCC(=O)Nc3ccc(cc3)c4ccno4	0
Cc1ccc(cc1S(=O)(=O)/N=c\2/cc(cc[nH]2)Cl)Cl	0
Cc1c(sc(n1)c2cc(c(n2C)C)S(=O)(=O)Nc3cccc(c3)C(=O)C)C(=O)N4CCCC4	0
Cc1c(c(n(n1)C)O[C@H]2C[C@H](CC(C2)(C)C)C)N	0
CCc1cc(cc(c1)OCCOc2c(cc(cc2Br)/C=C\3/C(=O)NC(=O)N(C3=O)c4ccc(cc4)Cl)OCC)C	0
c1cc2c(cc1NC(=O)[C@H]3CCc4c(cc(s4)C(=O)N5CCCC5)C3)OC	0

**O2**	
**Cc1ccc(cc1)O[C@@H]2[C@H]([C@@H]([C@H]3[C@@H](O2)COC(O3)(C)C)O)NC(=O)C**	0
**CCCS(=O)(=O)Nc1ccc2n(c1=O)C[C@H]3C[C@@H]2CN(C3)C(=O)c4ccc(cc4)N(C)C**	0
**CC[C@@H](C)N(CC)C(=O)c1cc2cc(ccc2o1)N**	0
**Cc1ccn(n1)[C@H](c2ccc(cc2)F)[C@@H](C)N**	0
**c1ccc2c(c1)nc(s2)[C@H]3CCCC[C@@H]3C(=O)NNC(=O)[C@@H]4COc5ccccc5O4**	0
**CC(=O)N1CCN(CC1)C(=O)CN2CC3=C(C2=O)[C@H](NC(=O)N3CC=C)c4ccccc4Cl**	0
**C1CC1[C@H]2C=C(N=N2)Cl**	0
**CC(=O)N[C@H]1[C@@H]([C@@H]2[C@@H](CO[C@@H](O2)c3ccccc3)O[C@@H]1OCc4ccccc4)OC(=O)/C=C\c5ccccc5**	0
**CC(C)OC(=O)c1c(c2c(c3c(nc2s1)CC[NH+](C3)C)c4ccccc4OC)N**	0
**COc1ccc(C(=O)C2CCN(C3CCN(Cc4nc5sc(C)cc5c(=O)[nH]4)C3=O)CC2)cc1**	1
**Cn1cccc1[C@H](CNC(=O)C(=O)Nc2ccc(cc2)C(F)(F)F)[NH+]3CCN(CC3)C**	0
**Cn1cc(cn1)NS(=O)(=O)c2ccnc(c2)Cl**	0
**C[C@@H](C(=O)NNC(=O)c1ccc(cc1)Cn2nc(nn2)c3ccccc3)n4cc[nH+]c4**	0
**c1ccc2c(c1)cnn2[C@H]3CC4(CC[C@@H]3O)OCCO4**	0
**C[C@H]1CC[C@](C[C@@H]1C)([C@@H](C)C#N)O**	0
**c1cc(ccc1C(=O)NCCN2C(=O)/C(=C/c3ccc(c(c3)[N+](=O)[O-])Cl)/SC2=O)OC(F)F**	0
**Cc1cc(nc(n1)[C@@]2(CCCN2C3CCCC3)C)N**	0
**Cc1cccc2c(=O)[nH]c3c(c12)COC(C(C)C)C3**	1
**CCc1ccc2c(c1)sc(n2)NC(=O)c3ccc(nn3)[O-]**	0
**c1conc1CN2CCN(CC2)c3cc(nc(n3)N)Cl**	0
**Cc1cc(ccc1F)[C@H](c2cc3c(c(c2)Cl)OCO3)O**	0
**CC(C)C(=O)/C(=c\1/n(c(=O)/c(=C\c2ccc(cc2)NC(=O)C)/s1)CC=C)/C#N**	0
**O=C(CCc1ccccc1Cl)Nc1nnc2n1CCS2**	1
**CC(C)(C)c1nc2cc(ccc2n1C)NC(=O)c3ccco3**	0
**Cn1cc(cc1C(=O)Nc2cc(ccc2OC)S(=O)(=O)N(C)C)S(=O)(=O)N3CCOCC3**	0
**C[C@H]1c2nnc(n2CC[NH2+]1)c3c(cc(cc3F)F)F**	0
**Cc1ccc(c(c1)CC(=O)Nc2nc3ccc(cn3n2)F)C**	0
**O=c1[nH]c(N2CCC3(CC2)CN(CCO)c2cccc(F)c23)nc2c1CCCC2**	1
**CCC[NH2+][C@@H]1CCCc2c1nc(s2)N3CCS(=O)(=O)CC3**	0
**C[C@@H]1CCC[C@@H](CC1)[C@@H]2CC(=O)NC2=O**	0
**c1ccc(cc1)n2c-3c(cn2)C(=O)N([C@@H](c4n3ccc4)C(=O)NC5CCCCCC5)C6CCCCC6**	0
**c1ccc(cc1)OCC[NH+]2CCN(CC2)Cc3c(c(coc3=O)C[NH+]4CCSCC4)[O-]**	0
**O=C(N[C@H]1C[C@H](c2nnc(-c3ccc(OC4CC4)cn3)n2-c2ccccc2F)C1)c1ccccn1**	1
**Cc1cccc2c(=O)[nH]c(-c3ccc(C(C)(C)O)cc3)nc12**	1
**Cc1cccc(c1)c2c3c(c4n2CCO[C@@H]4c5cccc(c5O)OC)n(c(=O)n(c3=O)C)C**	0

**Cn1cc(cn1)/C=C\2/C(=O)N(C(=N2)SCC(=O)Nc3ccc(cc3Cl)Cl)c4ccccc4**	0
**O=C1NC(c2ccccc2OCc2ccccc2)Nc2ncccc21**	1
**Cc1c2cc(ccc2oc1C(=O)Nc3ccc(cc3N4CCOCC4)N5CCOCC5)F**	0
**CC(C)NC(=O)[C@H]1COC2(N1C(=O)c3ccc(cc3)OC)CCN(CC2)C(=O)C4CCC4**	0
**CCOc1ccc(cc1[N+](=O)[O-])C(=O)OCC(=O)N[C@@H](c2ccc(cc2)F)c3cccs3**	0
**c1cc(c([nH+]c1)N2CCOCC2)CNC(=O)Nc3ccc(cc3)N4CCCS4(=O)=O**	0
**c1ccc(cc1)S(=O)(=O)NC[C@H]2C[C@@H]3CC[NH+]2C[C@@H]3CN4CCN(CC4)c5ccccn5**	0
**Cn1cc(cn1)[C@@H]2c3cnn(c3N=C(CS2)O)c4cc(ccc4Cl)F**	0
**Cc1ccc(C2NC(=O)c3cccnc3N2)cc1**	1
**CCOc1cc(ccc1O)[C@@H]2C(=C(c3ccc4c(c3)OCCO4)O)C(=O)C(=O)N2CCCn5ccnc5**	0
**O=C1CC2(CCN(C(=O)CN3CCN(CCSc4nc5ccccc5c(=O)[nH]4)CC3)CC2)Oc2ccccc21**	1
**c1cc(cc(c1)c2cnc([nH]2)[C@H]3CCCCCN3)C#N**	0
**CC[NH+]1CCC(CC1)CCNC(=O)[C@@H]2CCc3c(sc(n3)N4CCN(CC4)C(=O)C)C2**	0
**CC[C@@H]1C[C@@H](n2c(cc([nH+]2)[C@@H]3CCCN(C3)C(=O)c4ccc(=O)[nH]c4)N1)C(F)(F)F**	0
**CCOC(=O)C1=C(N(C(=O)N[C@@H]1c2ccccc2Cl)C)CN3CCN(CC3)C(=O)C(C)C**	0
**CC1(C)CC2=NNC(=O)c3cccc4[nH]c(c2c34)C1**	1
**O=C(CN1CCC(C(=O)c2ccc(OCC3CC3)cc2)CC1)NCc1nc2c(c(=O)[nH]1)COCC2**	1
**Cc1cc2c(c(=O)n1CCOC)[C@@]3(c4ccccc4N(C3=O)Cc5ccccc5)C(=C(O2)N)C(=O)OC**	0
**CC(=O)N1CCc2c(sc(c2C#N)NC(=O)c3ccc(cc3)S(=O)(=O)N(C)C[C@@H]4CCCO4)C1**	0
**CC(=O)N1CCc2c(sc(c2C(=O)OC)NC(=O)/C=C/c3cc(c(c(c3)OC)OC)OC)C1**	0
**c1cc(c([nH+]c1)N2CCN(CC2)S(=O)(=O)c3ccc(cc3)C(F)(F)F)C(=O)NCc4cccs4**	0
**CC[C@H]1CCC[C@@H](C1)NC(=O)N2CC[NH+](CC2)Cc3nc(no3)C**	0
**O=c1[nH]c(SCCN2CCN(CCCOc3ccc(-c4ccccc4)cc3)CC2)nc2ccccc12**	1
**CCn1c2c(c(n1)C)[nH]c(=S)n2[C@H]3CCC[C@@H]([C@@H]3C)C**	0
**[H]/N=C/1\[C@@H]([C@@H](C(=c2n1c(=O)/c(=C/c3ccccc3)/s2)C(=O)OCC)c4ccccc4)C(=O)N**	0
**CC[C@H]1C(=O)N[C@H](C(=O)N1[C@@H](C)c2c(noc2C)C)C**	0
**Cn1c2c(cn1)c(nc(n2)Cl)N(C)c3ccccc3**	0
**Cc1cc(c(c(=O)o1)/C(=N/c2ccc3c4ccccc4oc3c2)/C)O**	0
**C[NH+](C)CCN(c1nc2ccc(cc2s1)SC)C(=O)[C@H]3CCCN3S(=O)(=O)c4cccs4**	0
**COC(=O)c1c(cc(=O)n2c1CCN(CC2)Cc3c[nH]c4c3cccc4)OCCCc5ccc[nH+]c5**	0
**CC(C)Sc1nc2c(c3c(s2)COC(C3)(C)C)c4n1c(nn4)SCC(=O)Nc5ncc**	0

**s5**	
**Cc1cccn2c1[nH+]c(c2)c3ccc(cc3)NC(=O)C(=O)N[C@H]4CC(=O)N(C4=O)C(C)C**	0
**c1c2c(cc(c1NCc3c[nH+]c[nH]3)Br)[nH]c(=O)[nH]2**	0
**CC(C)N1[C@@H](C[NH+]=C1N)c2cc3c(c(c2)Cl)OCCCO3**	0
**CCCCCCN1C(=O)/C(=C/c2c(nc3c(cccn3c2=O)C)N4CCN(CC4)CCO)/SC1=S**	0
**CS(=O)(=O)c1ccc(-c2nnc(/C=C/c3nnc(-c4ccc(C#N)cc4)o3)n2-c2ccccc2Cl)nc1**	1
**C[C@H]1[C@H](CCN1C(=O)[C@@H]2CC[C@@H]3CCCC[C@@H]3N2)C(=O)[O-]**	0
**C[C@H](C(=O)NC1(CCCCC1)C#N)N2CCN(CC2)S(=O)(=O)c3ccc4c(c3)OCCO4**	0
**Cc1ccc(cc1C)N2C[C@]34C=C[C@H](O3)[C@H]([C@H]4C2=O)C(=O)N(C)Cc5[nH]cc(n5)C**	0
**CCOc1ccc(cc1)[C@@H]\2C(=C(S/C2=N/[O-])N)C#N**	0
**Cc1ccc(cc1NC2CCC3(CC2)OCCO3)Cl**	0
**c1ccc2c(c1)c(nc(n2)CN3CCOCC3)SCC(=O)c4cc([nH]c4)C(=O)N5CCCC5**	0
**O=C(c1ccc(OC(F)(F)F)cc1)C1CCN(CC(=O)N(Cc2nc3c(c(=O)[nH]2)COCC3)CC(F)F)CC1**	1
**Cc1ccc(cc1C(=O)N(C)CC(=O)Nc2c(cc(cc2C)C)C)S(=O)(=O)N3CCCC3**	0
**COc1ccc(C(=O)C2CCN([C@@H]3CCN(Cc4nc5c(c(=O)[nH]4)COCC5)C3=O)CC2)cc1C**	1
**CCOC(=O)C1=C([C@H](N2C(=O)[C@H](SC2=N1)Cc3cccc(c3)[N+](=O)[O-])c4ccccc4)C**	0
**CC[C@@H](C)Oc1cccc(c1)C(=O)NC(=S)Nc2ccc(cc2)S(=O)(=O)N3CC[NH+](CC3)C**	0
**C[C@H]1CCc2c(c(c(s2)/N=C/c3cccc(c3[O-])O)C#N)C1**	0
**CC(=O)c1cccc(c1)N2C(=O)[C@@H]3[C@H]([NH2+][C@@]([C@H]3C2=O)(CCSC)C(=O)OC)c4ccc(cc4OC)OC**	0
**c1ccc2c(c1)n(c(=O)o2)CC(=O)OCC(=O)Nc3nc(cs3)c4ccc(c(c4)F)F**	0
**c1ccc(cc1)N2CC[NH+](CC2)[C@@H](CNC(=O)C(=O)Nc3ccccc3[N+](=O)[O-])c4cccnc4**	0
**CCn1c(c[nH+]c1SCC(=O)Nc2cc3c(cc2Cl)OCCO3)c4ccc5c(c4)OCCO5**	0
**COc1ccc(-c2noc(CSc3nnc(C)n3-c3ccc(C)cc3)n2)cc1**	1
**Cc1ccc(cn1)[C@@H](C)NC(=O)c2cn[nH]c2c3cccc(c3)F**	0
**C1CCC(CC1)(C(=O)NCC2(CCCC2)O)[NH3+]**	0
**C1CC[C@@H]([C@@H](C1)C(=O)NC[C@@H]2CCC[C@@H]2O)[NH3+]**	0
**C1CCC(CC1)[C@H]([C@@H]2CCOC3(C2)CCC3)O**	0
**COc1ccc(c(c1)OC)C(=O)C2=C(C(=O)N([C@H]2c3cccs3)CCCn4cc[nH+]c4)[O-]**	0
**c1cc(ccc1NC(=O)N2CCC3(CC2)c4[nH+]ccn4C[C@@H](O3)C(=O)N5CCOCC5)F**	0
**C[C@H](c1nc2ccccc2n1CC(=O)Nc3ccc(c(c3)S(=O)(=O)N(C)C)Cl)NC(=O)C**	0
**c1cnc(c(n1)Nc2cc(c(c(c2)F)F)F)Cl**	0
**c1cc(ccc1c2cc(no2)/C(=N/c3nnc(s3)N4CCCCCC4)/[O-])Br**	0

CCOc1ccc(cc1OCC)[C@@H]2C(=C(C(=O)N2CC[NH+](C)C)O)C(=O)c3ccc(cc3)Br	0
CCOC(=O)c1c2c(sc1NC(=O)C(=O)N/N=C/c3cn(nc3c4ccc(cc4)C)c5ccccc5)C[C@@H](CC2)C	0
Cc1c(c(n(n1)c2ccccc2)C)S(=O)(=O)Nc3nc4c(s3)C[C@@H](CC4)C	0
C[C@H]1CCc2c(c([nH]n2)C(=O)NC3CCCC3)C1	0
CN1c2ccccc2[C@@]3(C1=O)C(=C(C(=O)N3CCC[NH+](C)C)O)C(=O)c4ccc(cc4)S(=O)(=O)N(C)C	0
C[C@H](C#N)NC1C[C@H]2CCC[C@@H](C1)[NH+]2C	0
Cc1ccc2c(c1)sc1nnc(N)n12	1
Cc1cccn2c1nc(c(c2=O)/C=C/3\C(=O)N(C(=S)S3)CCOC)NC[C@H](c4ccccc4)O	0
c1ccc(cc1)N2CC[NH+](CC2)[C@@H](CNC(=O)C(=O)NCCC3=c4ccccc4=[NH+]C3)c5cccnc5	0
Cc1cc(ccc1OCC=C)C(=O)C2=C(C(=O)N([C@H]2c3ccc(cc3)Br)CCC[NH+]4CCOCC4)[O-]	0
Cc1cc(c(cc1Cl)C)[C@@H](c2ccc(cc2)S(=O)(=O)C)N	0
C[C@@H](c1ccccn1)N(C)C(=O)c2cc(cc(c2)S(=O)(=O)N(C)C)NCc3ccsc3	0
COc1cc(cc(c1OCc2ccc(cc2)Cl)CC=C)/C=C\3/C(=O)NC(=O)N(C3=O)c4ccc(cc4)Br	0
NC(=O)c1cccc2c1O/C(=C\c1ccc(C(=O)N3CCN(c4nc5ccccc5[nH]4)CC3)cc1)C2=O	1
Cc1ncc2c(=O)[nH]c(-c3ccc(C(C)(C)C)cc3)cn12	1
Cc1n[nH]c(=S)n1c2c(cc(cc2Cl)Cl)Cl	0
Cc1cc(no1)/N=C/c2c(c3ccccc3s2)O	0
Cc1c(c(nc(n1)C)N2CCN(CC2)C[C@@H](C[NH+]3CCOCC3)O)Cc4ccccc4	0
C[C@H]1CN(C(=Nc2cccc(c2)S(=O)(=O)N(C)C)S1)C(=O)CCNC(=O)c3cccs3	0
COC(=O)Nc1c(c2c(s1)CCCC2)C(=O)[O-]	0
Cc1c(sc(n1)N2[C@@H](C(=C(c3ccc4c(c3)C[C@H](O4)C)O)C(=O)C2=O)c5ccc[nH+]c5)C(=O)OCC=C	0
C[C@H]1CCCC[C@@H]1NC(=O)[C@@]2(Cn3c4ccoc4cc3C(=O)N2CC[NH+]5CCOCC5)C	0
CC(C)(C)c1ccc(C(=O)Nc2nnc3n2CCS3)cc1	1
Cc1cc(ccc1S(=O)(=O)NC[C@H](c2ccc3c(c2)CCN3C)N4CC[NH+](CC4)C)NC(=O)C	0
COc1ccc(cc1OC)CCNC(=O)/C(=C/N=C\2/N([C@@H]3CS(=O)(=O)C[C@@H]3S2)CC=C)/C#N	0
Cc1ccc-2c(c1)NC(c3c2/c(=N/c4cccc(c4)F)/ss3)(C)C	0
C[C@H]1C[C@@H](CC(C1)OC(=O)C2(CCC2)N)C	0
CC(=O)O[C@@H]1CC[C@@]2([C@@H]3CC[C@@]4([C@@H](CC[C@@]4([C@@H]3CC[C@@]2(C1)O)O)C5=CC(=O)OC5)C)C=O	0
C[C@@H]1CCCC[C@]12C(=O)N(C(=O)N2)CC(=O)N3[C@@H](CC(=N3)c4ccc(cc4)Cl)c5ccco5	0
Cc1ccc(c(c1)[N+](=O)[O-])N2C3=C([C@H](C(=C2n4cccc4)C#N)c5ccc(s5)C)C(=O)CCC3	0
CCCN(Cc1nc2c(c(=O)[nH]1)COCC2)C(=O)CN1CCC(C(=O)c2ccc(F)cc2)CC1	1

COc1ccc(C(=O)C2CCN([C@H]3CCN(Cc4nc5c(c(=O)[nH]4)COCC5)C3=O)CC2)cc1	1
Cc1ccccc1Cn2c(=O)c(nn(c2=O)c3ccc(cc3)Cl)C(=O)NCCc4cccc[nH+]4	0
Cc1cccc2c1c(=O)n(C[C@H]1CC[C@@H](C(=O)O)CC1)c(=O)n2Cc1cccc(C(F)(F)F)c1C#N	1
C[C@@]12CCC[C@@]([C@]1(C[C@H]3[C@@H](C2)OC(=O)[C@@H]3C[NH+]4CCN(CC4)c5ccc(cc5)[N+](=O)[O-])O)(C)OC	0
CC(=O)N1c2ccc(cc2C[C@H]1/C(=N/Cc3cccc(c3OC)OC)/O)S(=O)(=O)N4CCCC4	0
Cc1ccc(c(c1)OCc2nnc(n2N)S[C@@H](C)C(=O)N(C)[C@H]3CCS(=O)(=O)C3)C(C)C	0
Cc1cc/c(=N/S(=O)(=O)c2cc(n(c2C)C)c3nc(c(s3)C(=O)N4CCCCC4)C)/[nH]c1	0
Cc1cc2c(cc1C)oc3c(c2=O)[C@@]4(c5ccccc5N(C4=O)C)N(C3=O)CCCO	0
c1cc(ccc1c2ccc(=O)n(n2)CCNC(=O)C3CCN(CC3)c4cc(ncn4)n5cncn5)F	0
c1ccc2c(c1)OC[C@H](O2)CSc3cc(ccc3N)F	0
c1cc(ccc1C#N)NC(=O)Nc2ccc(cn2)Br	0
O=C(c1ccc(F)cc1)C1CCN(CC(=O)N(Cc2ccccn2)Cc2nc3c(c(=O)[nH]2)COCC3)CC1	1
Cc1cc(c(cc1C)S(=O)(=O)N2C[C@H]3CC[C@@H]2C3)N	0
c1ccc2c(c1)[C@@H](CN2c3nc4ccccn4n3)CC[NH3+]	0
c1c(cnc(c1Br)N[C@H]2CCNC2)[N+](=O)[O-]	0
C[C@@H]1[C@H](C(=O)N(N1C)c2ccccc2)NC3=[NH+]N=C(CS3)C4=CC(=NC4)C(=O)N5CCCC5	0
c1cc2c(cc1NC(=O)c3ccc(cc3Cl)[N+](=O)[O-])CCC2	0
c1ccc(cc1)c2ccc3c(c2)[n+](c(o3)C/C=N/c4ccccc4)CCCS(=O)(=O)[O-]	0
N#Cc1cccc2c1C1(CCN(c3nc4c(c(=O)[nH]3)CCCC4)CC1)C(=O)N2	1
CC[C@@]1(CCCN(C1)C(=O)N[C@@H]2CCC[NH+](C2)C)C(=O)[O-]	0
c1cc(c(cc1Cl)Cl)Cc2nc(on2)c3csc(n3)N	0
Cc1cccc(c1)c2ccc(nn2)N3CCC[C@@H](C3)O	0
C[C@@H]1CCC[NH+](C1)[C@H](c2cc3cc4c(cc3[nH]c2=O)OCCO4)c5nnnn5C6CCCC6	0
c1cc(ccc1C#N)n2c(c(c(n2)C3CC3)Br)O	0
Cc1c(c(n(n1)c2cc(ncn2)N3CCOCC3)C)CCC(=O)NCCc4ccc(cc4)SC	0
Cc1ccc(cc1)n2nc3cc(c(cc3n2)Cl)NC(=S)NC(=O)c4cccc(c4OC)C	0
C[C@@H](c1nnc(n1c2ccc(cc2)Cl)S[C@H](c3ccccc3)C(=O)NC(=O)NC)[NH+](C)C	0
Cc1cc(sc1c2nnc3n2CCCCC3)N	0
CC[C@H]1CC[C@H]([C@@H](C1)Oc2ccc3c(c2)OCO3)O	0
COc1ccccc1N1CCN(c2nc3c(c(=O)[nH]2)CSCC3)CC1	1
Cc1cc2c(c(=O)n1CC[NH+](C)C)[C@@H](C(=C(O2)N)C#N)c3cc(c(cc3OC)OC)OC	0
COc1cccc(c1)NC(=O)/C(=C\[NH+]=C/2\N([C@@H]3CS(=O)(=O)C[C@@H]3S2)c4cccc(c4)OC)/C#N	0
C[C@@H]1CCC[C@H](N1C(=O)Nc2c[nH]c3c2cccc3)C	0

**c1c(cc(c(c1Cl)[C@H]([C@H]2CC=CC[C@@H]2C(=O)[O-])O)Cl)Cl**	0
**C[NH+](CC(=O)NCCNc1ccc(cn1)C(F)(F)F)[C@H]2CCS(=O)(=O)C2**	0
**CCCn1c(c(c(c(c1=O)C#N)C)/C=C/2\C(=O)N(C(=S)S2)CCCCCC(=O)O)N3CCCCC3**	0
**c1cc(c(nc1)SCC(=O)N2[C@H]([C@H]3CCC/C(=C\c4ccco4)/C3=N2)c5ccco5)C(=O)[O-]**	0
**Cc1c(cnn1c2ccccc2)C(=O)C3=C(C(=O)N([C@@H]3c4cccc(c4)Br)CC[NH+]5CCOCC5)[O-]**	0
**CC(C)[C@H](C(=O)N1CCC[NH+](CC1)CC(=O)N2CCCC2)NC(=O)C**	0
**c1cc(ccc1CN2CCN(CC2)C(=O)c3cc4c(cc3[N+](=O)[O-])OCO4)C(F)(F)F**	0
**Cc1cc(cc(c1)S(=O)(=O)NCc2nc3cccnc3n2Cc4ccc(cc4)F)C**	0
**CC[C@@H]1CN[C@@H](CN1c2c(c(nn2C)C)C#N)CC**	0
**CCOC(=O)c1c2c(n(n1)Cc3cccc[nH+]3)CCN(C2)C(=O)Cc4c([nH]c5c4cccc5F)C**	0
**c1cc2c(c(c1)[N+](=O)[O-])sc(c2Cl)C(=O)Nc3nccs3**	0
**Cc1cccc(-c2nnc([C@H]3C[C@H](NC(=O)c4ccc(F)cn4)C3)n2-c2ccccc2F)n1**	1
**COc1cc(ccc1O)[C@@H]2[C@H]([C@@H](CO2)[C@@H](c3ccc(c(c3)OC)O)O[C@H]4[C@@H]([C@H]([C@@H]([C@H](O4)CO)O)O)O)CO**	0
**O=c1[nH]c(-c2ccc(C(F)(F)F)cc2)nc2c1COCC2**	1
**C[C@H]1C[C@@H]([C@@H]2[C@@H](C1)NC[C@@H](S2(=O)=O)C)C**	0
**COc1ccc2c(n1)[nH]c(n2)[C@H]3CSc4c3cccc4**	0
**CCCCOc1ccc(cc1OCC)[C@H]2C(=C(C(=O)N2CCC[NH+](C)C)[O-])C(=O)c3ccc4c(c3)C[C@@H](O4)C**	0
**COc1ccc(-n2c(NCc3nc(-c4ccc(C)cc4)no3)nnc2-c2ccncc2)cc1**	1
**C1CCCC2(CCC1)C[NH2+]C[C@H]2C(=O)[O-]**	0
**C[C@@H]1C[NH+](C[C@@H](O1)C)C(C)(C)CNc2nc3ccccc3o2**	0
**Cc1cc(c(cc1C)S(=O)(=O)NC[C@@H]2CCCC[NH+]2C)N**	0
**COc1cc(cc2c1nc(s2)NC(=O)c3ccccc3)NS(=O)(=O)c4ccc(cc4)F**	0
**CC[C@H]1C(=O)NC(=O)CN1C(=O)C2(CCCC2)C**	0
**Cn1ccnc1C(=O)N2CCC[C@@H]2c3c(cnc(n3)N)c4ccc(cc4)C(F)(F)F**	0
**O=C(Nc1cccc2cccnc12)c1ccc(N2C(=O)[C@@H]3[C@@H]4CC[C@@H](C4)[C@@H]3C2=O)cc1**	1
**Cc1cc(c(cc1Cl)NC(=O)c2nc3nc(cc(n3n2)C)C)Br**	0
**c1ccc(cc1)OCC(=O)/N=C/2\N([C@@H]3CS(=O)(=O)C[C@H]3S2)c4ccccc4C(F)(F)F**	0
**Cc1ccc(cc1)c2cc(c3c(nn(c3n2)[C@H]4CCS(=O)(=O)C4)C)C(=O)NCc5ccc[nH+]c5**	0
**Cc1c2c(ncn(c2=O)C)sc1C(=O)N[C@H]3CCCC[C@@H]3C**	0
**c1cc[nH+]c(c1)CCNC(=O)[C@H]2CC23CCN(CC3)C(=O)C4(CCCCC4)N5CCOCC5**	0
**Cn1c(=O)n([C@H]2C[C@H](c3nnc(-c4ccncn4)n3-c3ccccc3Cl)C2)c2ccc(C#N)cc21**	1
**CCOc1ccc(-c2nnc([C@H]3C[C@H](NC(=O)c4ccnc5cc(F)cnc45)C3)n2-**	1

**c2ccccn2)nc1**	
**CC(C)c1cc(n[nH]1)C(=O)N2C[C@@H](CN(C(=O)C2)Cc3ccc[nH+]c3)OCc4ccccc4**	0
**Cc1c(nc(c(c1c2ccc(cc2)Cl)C#N)N)C**	0
**Cc1c(c(on1)C)c2[nH]c(c(c(=O)n2)I)[O-]**	0
**CC[C@H]1C(=O)N([C@@H](CC(=O)N1)C)Cc2cnn(c2)C**	0
**Cc1c(c(c(s1)C2=C3[C@H](N=C2c4c(cccc4OC)OC)N(C(=O)N(C3=O)C)C)O)C(=O)OC**	0
**Cc1c(c(=O)n(n1C)c2ccccc2)NC(=O)[C@@H](C)N3CCN(CC3)c4ccc(cc4F)C(=O)C**	0
**C[C@@H]1CNC[C@@H](O1)C2CCCC2**	0
**COc1ccc(C2(CNC(=O)c3ccc(NC(=O)c4ccco4)cc3Cl)CCOCC2)cc1**	1
**CN(C)c1ccc(c[nH+]1)NC(=O)c2c(cn(n2)c3ccccc3)O**	0
**C[C@H](C(=O)[O-])C1(CCC(CC1)(C)C)O**	0
**C1CC2=C([C@@H](C(=C(O2)N)C#N)[C@@H]3CCC=CC3)C(=O)C1**	0
**Cc1c(scn1)[C@H](C)Nc2cc3c(cc2Cl)NC(=O)CO3**	0
**CC(C)c1ccc(cc1)NC(=O)O[C@@H]2CO[C@H]3[C@@H]2OC[C@@H]3NS(=O)(=O)c4ccc(cc4)Cl**	0
**c1ccc2c(c1)OC[C@@H](O2)[C@@H](c3ccc(c(c3)Cl)Cl)N[NH3+]**	0
**Cc1c(sc(n1)CNC(=O)N2C[C@H](CC[C@@H]2C)C)C(=O)[O-]**	0
**CC(=O)Nc1ccc(cc1)/C=C\C(=O)NS(=O)(=O)c2ccc3c(c2)CCCC3**	0
**CC(=O)OCC(=O)[C@@]12[C@@H](C[C@@H]3[C@@]1(C[C@@H]([C@]4([C@@H]3CC(=C5[C@@]4(CCC(=C5)OCCCl)C)C=O)F)O)C)OC(O2)(C)C**	0
**Cc1ccc2c(=O)[nH]c(-c3ccc([C@](C)(O)CCO)cc3)cn12**	1
**Cc1cccc(c1C)N2CC[NH+](CC2)CCNC(=O)CN(C)c3nnc(s3)N4CCCC4=O**	0
**Cc1nc2cc(ccc2o1)N[C@@H]3C[C@H](SC3)C**	0
**CC(C)(C)n1c(nnn1)[C@@H](c2ccc(c(c2)OC)OC)N3CCN(CC3)c4nc5ccccc5s4**	0
**c1ccc(cc1)C(=O)N[C@@H](c2c3ccccc3c(=O)[nH]n2)C(=O)N/N=C/c4cc(c(c(c4)Br)O)Br**	0
**Cc1cc([nH+]c2c1c(c(s2)C(=O)N3CCOCC3)NC(=O)c4ccc(cc4)F)C**	0
**CCC(CC)(CNC(=O)NC1CC[NH+](CC1)C[C@@H]2CCOC2)O**	0
**COc1cc(cc(c1OC)OC)C(=O)N/N=C/c2c(c(c(c(c2F)F)F)F)F**	0
**CCN1C(=[NH+][C@@H](C1=[NH2+])c2cc3c(c(c2)Cl)OCO3)C**	0
**Cc1ccc(cc1S(=O)(=O)N2CCOCC2)NC(=O)[C@@H](C)[NH+](C)Cc3cccc(c3)C#N**	0
**C[C@H](C(=O)N1CCN(CC1)S(=O)(=O)c2ccc3c(c2)sc(=O)n3C(C)C)NC(=O)c4ccccc4**	0
**c1ccc(cc1)[C@@H](C(=O)Nc2ccc(c(c2)[N+](=O)[O-])Cl)N3CCC[NH+](CC3)CC(=O)N4CCCC4**	0
**CC1(C)OC(=O)N(c2ccc(-c3cnc(N)c(-c4ccc5nncn5n4)c3)cc2)[C@H]1c1ccccc1**	1
**c1cc(ccc1c2nc(c(o2)NCCCn3cc[nH+]c3)S(=O)(=O)c4ccc(cc4)Br)F**	0
**c1cc(c(cc1C(=O)N[C@@H]2CCS(=O)(=O)C2)S(=O)(=O)NC[C@H]3CCCO3)Cl**	0
**C[C@@H](c1ccc(cc1)N2CCOCC2)NC(=O)Cn3c4cc(ccc4oc3=O)**	0

Cl	
Cn1c2ccc(cc2nc1CC[NH+]3CCN(CC3)c4ccccn4)NC(=O)COc5ccc(cc5)[N+](=O)[O-]	0
Cn1c2ccc(cc2oc1=O)S(=O)(=O)N3CCC[C@@H](C3)C(=O)Nc4ccc(cc4)Cl	0
CCn1c2ccc(cc2nn1)C(=O)Nc3ccccc3Cl	0
c1cc(cc(c1)C(F)(F)F)CNC(=O)c2coc(n2)CN3CC[NH+](CC3)Cc4ccc(cc4)[N+](=O)[O-]	0
COC(=O)c1c(c2cnc3cc(ccc3c2s1)F)O	0
CN1CCN(c2cc(F)c3c(c2)N(C)C(=O)C32CCN(c3nc4c(c(=O)[nH]3)CCCN4C)CC2)CC1	1
CCc1nc2n(n1)c(c(s2)[C@@H](c3ccc(cc3)F)N4C[C@H](O[C@H](C4)C)C)O	0
Cc1ccc2nc(c(c(=O)n2c1)/C=C/3\C(=O)N(C(=S)S3)Cc4ccc(cc4)OC)NCc5ccco5	0
Cc1ccc(cc1)NC(=O)CCc2c(n(c3cc(nn3c2=O)c4cc(c(c(c4)OC)OC)OC)C)C	0
CSc1nc(cs1)C(=O)N2CCc3c(c(nn3Cc4ccccc4)C(=O)N5CCNC(=O)C5)C2	0
CC(C)C[C@H]1C(=O)N(C(=O)N1)C[NH+]2CCN(CC2)S(=O)(=O)c3ccc(c(c3)C(F)(F)F)Cl	0
Cc1ccc(cc1)C(=C2[C@H](N(C(=O)C2=O)c3cccnc3)c4ccc(c(c4)OC)O)O	0
CC(C)c1ccc(C2NC(=O)c3cccnc3N2)cc1	1
C[C@@H]1Cc2cc(ccc2N1S(=O)(=O)c3ccc(cc3)C(=O)OC)S(=O)(=O)N	0
Cc1c(sc(n1)Cc2ccccc2)C(=O)/N=c/3\[nH]nc4n3cccc4	0
CC1=C(C2(CCCCC2)OC1=O)C(=O)NC3CCCCC3	0
COc1cccc2c1oc(c2)C(=O)C3=C(C(=O)N([C@H]3c4ccc(c(c4)OC)OC)CCCn5cc[nH+]c5)[O-]	0
Cc1c(sc(n1)c2cccs2)c3ccc(=O)n(n3)CCNS(=O)(=O)c4cccc(c4)Cl	0
c1ccc(cc1)C[NH+]2CCN(CC2)CCCNC(=O)[C@H]3CCCN(C3)c4nnc(s4)n5cccc5	0
COc1ccc(cc1OC)[C@@H](CC(=O)OC)c2c(cc(c3c2oc(cc3=O)c4ccccc4)O)O	0
Cc1ccc2c(c1)cc(c(=O)[nH]2)[C@H](c3nnnn3C4CCCC4)N(Cc5ccc(cc5)OC)C[C@@H]6CCCO6	0
CC(C)[C@H](C#N)c1nc2ccccc2s1	0
Cc1c(cccn1)[N-]S(=O)(=O)c2c(n(c[nH+]2)C)Cl	0
CC(C)Cn1c(c(cn1)SC)N	0
c1nc(=O)c2c(n1C3CCCCC3)snn2	0
CC(C)[C@@H](c1ccc2c(c1)OCCO2)NC(=O)C(=O)Nc3ccc(cc3)Cl	0
Cn1c2ccc(cc2nc1C[NH+]3CCN(CC3)C(=O)c4ccccc4)NC(=O)c5ccc(cc5)Cl	0
Cc1c(sc2c1c(=O)n(c(=S)[nH]2)C3(CCCCC3)C)C	0
C[C@@H]1Cc2c(cnc(n2)NC3CC3)[C@H](C1)C	0
C[C@H]1CN[C@H](SC1)c2cc3c(c(c2)Br)OCCO3	0
CCCc1c2c(n(n1)C)n(c(=S)[nH]2)[C@@H]3CCCCC3(C)C	0
Cn1ccc2c(=O)[nH]c(N3CCC4(CC3)CN(CCO)c3cc(F)cc(F)c34)nc21	1
COC(=O)c1cccc(c1)NC(=O)c2cccc(c2)S(=O)(=O)[C@H]3CCS(=	0

O)(=O)C3	
CCOC(=O)c1ccc2c(c1)s/c(=N/C(=O)c3ccc(o3)Br)/n2CC(=O)OC	0
CC(=O)N1CCN(CC1)c2cc3c(cc2F)c(=O)c(cn3C4CC4)C(=O)Nc5cc(no5)C(C)(C)C	0
Cc1ccccc1COc2ccc(cc2OC)/C=N/NC(=O)[C@@H](C)Oc3ccc(cc3)c4ccccc4	0
O=C(CN1CCC(n2c(=O)[nH]c3ccccc32)CC1)NCc1nc2c(c(=O)[nH]1)COCC2	1
c1cc2c(cc1NC(=O)c3ccc(o3)Br)OCCCO2	0
Cc1cc(cc(c1)N2C[C@H](CC2=O)c3nnc(s3)NC(=O)[C@@H]4CC(=O)N(C4)C5CCCCC5)C	0
Cc1ccc2cc(c(=O)[nH]c2c1)CN(CCc3ccc(c(c3)OC)OC)Cc4nnnn4CCc5ccccc5	0
Cc1nc([nH]n1)C(=O)NC23CC4C[C@@](C2)(C[C@](C4)(C3)C)C	0
COc1ccc(cc1OC)CC(=O)NCCN2C(=O)/C(=C\c3ccc(cc3)F)/SC2=O	0
C[C@H]1CN([C@@H](CO1)C)c2ccc(cc2N)[N+](=O)[O-]	0
CN1C(=O)[C@H](N(C1=S)Cc2cccc[nH+]2)CC(=O)Nc3ccc(cc3)c4ccccc4	0
CCOc1ccc2c(c1)sc(n2)Sc3ccc(cc3[N+](=O)[O-])[C@H]4N[C@H]5CCCC[C@@H]5C(=O)N4	0
C[C@]1(C(=O)N(C(=O)N1)CC(=O)N2[C@@H](CC(=N2)c3ccco3)c4cccs4)c5ccc6c(c5)CCC6	0
CNC(=O)c1ccc(cc1)CN(C)C(=O)/C=C\c2ccc(cc2)S(=O)(=O)NCc3ccco3	0
Cc1cccc2c1c(=O)n(C[C@H]1CC[C@H](C(=O)Nc3cnn(C)c3)CC1)c(=O)n2Cc1cccc(C(F)(F)F)c1C#N	1
Cc1ccc(cc1F)[C@H](C)NS(=O)(=O)c2c[nH+]ccc2N	0
Cc1cc2c(=O)[nH]c(-c3ccc(C(C)(C)O)cc3)cn2n1	1
C[C@@H]1CCC[C@@H](C1)c2nc(on2)c3ccc(s3)N	0
c1cc(ccc1OCC(=O)N2CCC[C@H](C2)c3ccc(cn3)c4ccc(nc4)N)F	0
O=C1NC(c2cccc(OCc3ccccc3)c2)Nc2ccccc21	1
c1ccc2c(c1)OC[C@H](O2)C(=O)Nc3ccc(cc3)[N-]S(=O)(=O)c4ccc5c(c4)OCCCO5	0
c1ccc(c(c1)/C(=N/c2nncs2)/[O-])N	0
CCCCNC(=O)c1cn(cc(c1=O)C(=O)N2CCN(C(=O)C2)c3ccccc3)C(C)C	0
CC(C)n1cc(C(=O)C2CCN(C(=O)CCCc3nc4c(ccn4C)c(=O)[nH]3)CC2)cn1	1
C[C@@]1(CCS(=O)(=O)C1)NC(=O)c2cc(n(n2)c3ccc(cc3)Cl)c4ccco4	0
Cc1ccc(c(c1)C)c2c3c(c(=O)n(n2)CC(=O)NCc4ccc5c(c4)OCO5)nc(s3)C	0
C1CC[C@H]([C@H](CC1)O)C2(CCCCC2)C(=O)[O-]	0
Cc1cccc([nH+]1)CN2CCS(=O)(=O)[C@H]3[C@@H]2CCN(CC3)C(=O)NCC(C)C	0
CS(=O)(=O)Nc1cn[nH]c1[C@@H]2CCCN(C2)Cc3cn4cc(ccc4n3)Cl	0
C[C@H]1CN[C@@H]2CCCCCCCCCC[C@@H]2[S@@]1=O	0
C[C@@H]1CN2CCCC[C@@H]2CN1c3ccc(cc3Cl)[C@@H](C)O	0

O=C1Nc2cccc(Cl)c2C12CCN(c1nc3c(c(=O)[nH]1)CCCC3)CC2	1
CC(C)(C)OC(=O)N1CCC2=C(C1)Nc1cccc3c(=O)[nH]nc2c13	1
C[C@@H](C(=O)NCc1ccc2c(c1)OCO2)N3CCN(CC3)S(=O)(=O)c4ccc5ccccc5c4	0
C[C@H]1C[C@@H](CN(C1)c2ccccc2C(C)(C)C)C	0
Cc1ccc2cc(c(=O)[nH]c2c1C)C[NH+](Cc3ccc4c(c3)OCO4)Cc5nnnn5CCc6ccccc6	0
CC1=C2C[C@H]3C(=C)CCC[C@]3(C[C@@H]2OC1=O)C	0
CC(=O)c1ccc(cc1)NC(=O)CCN2C(=O)/C(=C/c3cc(c(c(c3)OC)OC)OC)/SC2=S	0
C[C@@H]1C[C@@H](n2c(cc(n2)[C@@H]3CCCN3C(=O)c4cc[nH]n4)N1)C(F)(F)F	0
Cc1ccc(cc1)Oc2c3c(nc(n2)N4CCN(CC4)C)CCN(C3)S(=O)(=O)c5ccccc5C#N	0
Cc1nc(no1)NS(=O)(=O)c2c(cc(cc2Cl)Cl)Cl	0
Cc1ccc(cc1F)S(=O)(=O)N2CCC[C@@H](C2)c3[nH+]c4c(c(n3)NC)C[NH+](CC4)C	0
Cc1cc(nc([nH+]1)N2CCC(CC2)(C)C(=O)[O-])C	0
C[C@H](CCO)CNS(=O)(=O)c1ccc(o1)C[NH2+]C(C)(C)C	0
CS(=O)(=O)N1CCN(CC1)Cc2c(c(coc2=O)C[NH+]3CCN(CC3)c4cccc(c4)Cl)[O-]	0
C[C@H](c1ccccc1)NC(=O)[C@H](C)[NH+]2CCN(CC2)C(=O)[C@@H]3COc4ccccc4O3	0
CC1(c2cc(ccc2NC1=O)S(=O)(=O)N3CCCc4c3c(c(cc4)F)F)C	0
COc1ccc(c(c1)n2c3ccncc3nc2N)Br	0
Cc1cc(NCCc2ccc(O)cc2)c2nncn2n1	1
Cc1c(c([nH]c1C(=O)OC)C)C(=O)C2=C(C(=O)N([C@H]2c3ccc(cc3)Cl)CCCn4cc[nH+]c4)[O-]	0
c1ccc(c(c1)/C=N/Nc2c(cc(cn2)Cl)Cl)O	0
CC1(CCC[C@@]2(CC1)C[C@H](CCO2)O)C	0
C[C@]1(CCCN(C1)C(=O)[C@H]2C[C@@H]3CCCC[C@@H]3N2)C(=O)[O-]	0
Cc1c(sc2c1c(nc(n2)CSC(=[NH2+])N)[O-])C	0
C[C@@H](C(=O)O[C@H](C)C(=O)Nc1ccccc1C#N)N2C(=O)[C@@H]3CC=CC[C@@H]3C2=O	0
c1ccc(cc1)CC2CCN(CC2)S(=O)(=O)c3ccc(nc3)Sc4[nH]nc(n4)c5cncc5	0
c1c2c([nH+]c3c1c(c(s3)C(=O)NCC(F)(F)F)N)CCC2	0
CC(=O)C1=CC[C@H]2[C@@]1(CC[C@H]3[C@@H]2C[C@H]4[C@@]5([C@@]3(CC[C@@H](C5)O)C)O4)C	0
C[C@@H]1Cc2cc(ccc2N1Cc3cccc(c3)S(=O)(=O)N4CCOCC4)S(=O)(=O)N	0
CC(C)(C)[C@H]1CCN(C[C@H]1O)C(=O)NCC#C	0
O=C(c1ccc(F)cc1)C1CCN(CC(=O)N(Cc2nc3c(c(=O)[nH]2)COCC3)Cc2ccc(S(=O)(=O)N3CCOCC3)s2)CC1	1
Cc1cc2c(s1)ncn(c2=O)CC(=O)NC(=O)N[C@@H]3CCCC[C@@H]3C	0
c1ccc2c(c1)CCN2c3c4c(c5ccccc5o4)ncn3	0
O=C(CCc1cccnc1)Nc1nnc2n1CCS2	1
c1c(nc(cn1)N)C2CC2	0
c1cnc(nc1)N2CCC[NH+](CC2)[C@@H](c3c[nH]c4c3ccc(c4)Br)C(=O)[O-]	0

COP(=O)([C@@H](c1nnc(o1)c2ccccc2)NC(=O)c3ccccc3)OC	0
Cc1cc(n2c(n1)c(c(n2)C(=O)Nc3ccc(cc3)F)c4ccc(c(c4)S(=O)(=O)N5CCCC5)OC)C	0
Cn1c2c(c(=O)[nH]c1=O)n(c(n2)N3CC[NH+](CC3)Cc4ccc(cc4)Br)CC=C	0
Cc1ccccc1/N=C/2\NN=C(CS2)c3c([nH]c4c3cccc4)C	0
C[C@@H]1C[S@](=O)[C@@H]2CCCCCCCCCC[C@@H]2N1	0
C[C@@]12C[C@@H](CC(C1)(C)C)N(C2)C(=O)[C@@]3(COC[C@@H]3[NH3+])C	0
c1cc(cc(c1)S(=O)(=O)Nc2ccc(cc2)C(=O)NCc3cc[nH+]c(c3)N4CCCC4)[N+](=O)[O-]	0
CS(=O)(=O)CC[C@H](C(=O)NC[C@H]1Cc2ccccc2OC1)N3C(=O)c4ccccc4C3=O	0
CCNC(=O)[C@@H]1Cc2ccccc2N3[C@@H]1CN(CC3)c4cccc[nH+]4	0
Cc1cc(nn1c2cccc(c2)C(=O)NCCc3cccc(c3)Cl)c4nnc(o4)C	0
C[C@H]1CCCN(C1)S(=O)(=O)c2cccc(c2)c3cs/c(=N\N=C\c4ccco4)/[nH]3	0
c1ccc2c(c1)[C@@H](CCS2)Nc3ccc4c(c3)OCCO4	0
Cc1cc(cc(c1)NC=C2C(=O)N(C(=O)N(C2=O)C)C)C	0
Cc1ccc(cc1F)NC(=O)[C@H]2CCCN(C2)S(=O)(=O)c3ccc4c(c3)CCCC(=O)N4	0
Cc1cc(cc(c1O)C)c2nc(c(c(n2)NC)Br)C	0
COc1ccc(cc1OC)CCN([C@H]2CC(=O)N(C2=O)c3cccc(c3)Cl)C(=S)Nc4ccccc4	0
Cc1nc(on1)CCCNC(=O)c2ccc(c(c2)S(=O)(=O)N3C[C@@H](O[C@H](C3)C)C)Cl	0
Cc1ccc(cc1F)n2c(c(c3c2[nH+]c(nc3N)C)C)C	0
C[C@@H](c1ccc(cc1)n2cncn2)NC(=O)NCCCN3CC[NH+](C[C@@H]3c4ccccc4)C	0
C[C@@H](c1nc(c2c(csc2n1)c3cccs3)[O-])Sc4nnc(o4)c5cccnc5	0
Cc1c(c(on1)C)S(=O)(=O)N2CCC[NH+](CC2)CCC(=O)Nc3c(c4c(s3)CCCC4)C#N	0
c1ccc2c(c1)nc(c3n2nc(n3)C(=O)NCCc4ccncc4)NCc5ccccn5	0
c1cnc(nc1[O-])Sc2cc(c(cc2N)Cl)Cl	0
Cc1cc(ccc1C#CCO)NS(=O)(=O)c2ccccc2	0
CC(C)Oc1cncc(n1)N2C[C@H]3CC[C@@H](C[C@H]3C2)[NH3+]	0
c1ccc2c(c1)ccc3c2cc(o3)C(=O)NC4CCCC4	0
CN1CCN(CC1)S(=O)(=O)c2ccc(cc2)NC(=O)c3cccc(c3C(=O)[O-])[N+](=O)[O-]	0
CC1CN(C(=O)c2cc(Cc3n[nH]c(=O)c4ccccc34)ccc2F)Cc2nnc(C(F)(F)F)n21	1
Cc1[nH+]c(cc(n1)N(C)C)[C@@H](Cc2c[nH]c3c2cccc3)NC(=O)c4cccc(c4)F	0
CC[C@@H]1CCCN(C1)c2nc(cc(n2)C)C	0
Cn1c2c(c(=O)n(c1=O)C)n3cc(n(c3[nH+]2)[C@H]4CCS(=O)(=O)C4)c5ccc6ccccc6c5	0
C#CCNC(=O)N1CCSC[C@H]1c2ccc(cc2)Cl	0
Cc1ccc(c(c1)C)[N-]S(=O)(=O)c2ccc3c(c2)CCN3C(=O)CCC(=O)N4CCCCCC4	0
CCc1cccc(c1n2c(c(c3c2ncnc3N)C)C)C	0

Cc1nc2n(n1)c(c(s2)[C@@H](c3ccco3)N4CCN(CC4)C(c5ccccc5)c6ccccc6)[O-]	0
Cc1c(c(n(n1)C(C)C)N2C[C@H](N[C@@H](C2)C)C)[N+](=O)[O-]	0
O=C(N[C@H]1C[C@H](c2nnc(-c3ccc(OC(F)F)cn3)n2-c2ccccc2F)C1)c1ccc(F)cn1	1
CCn1c[nH+]c2c1c(=O)n(c(=O)n2Cc3ccccc3)CC(=O)Nc4cccc(c4)C(=O)C	0
Cc1cccc2c(=O)[nH]c(N3CCC4(CC3)C(=O)N(CCO)c3cc(F)cc(F)c34)nc12	1
CN[C@@H]1CCCc2c1sc(n2)N3CC[C@@H]4CCCC[C@@H]4C3	0
CC1(CCC[C@H]1n2c3cc(c(cc3[nH]c2=S)F)OC)C	0
Cc1c(c(n[nH]1)C(C)C)Br	0
COc1ccc(OC)c2c1sc1nnc(N)n12	1
Cc1ccc(o1)[C@@H](CNC(=O)[C@@]2(CCCCN2C(=O)c3ccccc3)C)N4CCOCC4	0
Cc1cc(c(c(c1)C)NC(=O)c2cnc(nc2C)N3CCCCC3)C	0
CCOC(=O)c1c([nH]c(c1c2ccc(cc2)OC)C(=O)C(=O)NCc3cccc[nH+]3)C	0
CC(=O)N1CCN(CC1)c2c(cc3ccc(cc3n2)Cl)CNCCCN4CCCC4=O	0
Cc1c(sc(n1)C)C(=O)C2=C(C(=O)N([C@@H]2c3cccc4c3cccc4)CCOC)O	0
C[C@H]1Cc2cc(ccc2N1C(=O)c3ccncc3Cl)N	0
C[C@@H](C(=O)Nc1cccc(c1)C#N)Sc2nc3c(c4c(s3)CCCC4)c(=O)n2CCC[NH+](C)C	0
c1cocc1[C@@H](c2cnccn2)O	0
Cc1cc(cnc1N2CCN3CCC[C@@H]3C2)N	0
C1CCC[NH+](CC1)[C@@H]2CCCC[C@H]2C(=O)[O-]	0
C[C@@H]1CN(C[C@H](O1)C)C(=O)c2ccc(c(c2)S(=O)(=O)NCc3ccco3)Cl	0
C[C@H]1CCC[C@@]1(C#N)N2CCCC2	0
CC1CCC(CC1)(C2(CCC(CC2)(C)C)C#N)O	0
c1cc(cnc1)c2nnc(o2)C3CCCC3	0
O=C(CN1CCC(c2noc3cc(F)ccc23)CC1)NCc1nc2c(c(=O)[nH]1)COCC2	1
CC1(CCN(C1)c2ccc(cc2C#N)Cl)C	0
C[C@H](C1CC1)N(Cc1nc2c(c(=O)[nH]1)COCC2)C(=O)CN1CCC(C(=O)c2ccc(F)cc2)CC1	1
CCn1cc(c(=O)c2c1nc(cc2)C)C(=O)Nc3nc(cs3)c4ccc(cc4)C#N	0
Cn1c2c(c(nc(n2)c3ccc(cc3)Cl)SCC(=O)Nc4ccc(cc4F)F)c(=O)n(c1=O)C	0
Cc1ccc(cc1)N2[C@H]([C@H](CC2=O)C(=O)NNC(=O)c3cc(oc3C)C)c4cccs4	0
CC1=C[C@H](N=N1)C(=O)N/N=C/c2cc(c(c(c2Br)Br)O)OC	0
Cc1ccc(cc1)C2=NN([C@@H](C2)c3ccco3)C(=O)CSc4nnc(n4C)CCC(=O)N	0
Cc1cc(sc1c2nc(no2)C3(CC3)C)N	0
CCNC(=O)c1c(c(c([nH]1)C)C(=O)[C@@H]2[C@H](N(C(=O)C2=O)c3cc(on3)C)c4ccccc4F)C	0
c1c2c(sc1c3c(nco3)CN)CCCCC2	0
CS(=O)(=O)CC[C@@H](C(=O)[O-	0

])NC(=O)c1cc(nn1c2c(cccn2)Cl)Br	
CC(=O)[C@@H]1CC[C@H]2[C@@]1(CC[C@@H]3[C@@H]2CCC4=C/C(=N\OCC(=O)N[C@@H](Cc5c[nH]c[nH+]5)C(=O)OC)/CC[C@]34C)C	0
Cc1c(n(c2c1c(ncn2)N)Cc3ccc(s3)Cl)C	0
c1cc2=NC(=O)C(=c2cc1Cl)c3c(nc(s3)N)O	0
Cc1cc(c2c(c1)cc(c(=O)[nH]2)[C@H](c3nnnn3C4CCCCC4)[NH+](C)C5CCCCC5)C	0
CCOC(=O)c1c2c(n(n1)Cc3cccc[nH+]3)CCN(C2)C(=O)c4ccc5c(c4)cc[nH]5	0
O=C(Nc1cccc2cccnc12)c1ccc(N2C(=O)[C@@H]3[C@H](C2=O)[C@H]2C=C[C@@H]3C2)cc1	1
Cc1c(c(on1)/C=C\c2ccc(c(c2)S(=O)(=O)[N-]c3ccc4c(c3)OCCO4)OC)NC(=O)C	0
C[C@H](C(=O)Nc1ccc(cc1)C(C)(C)C)[NH+]2CCCC3(C2)OCCO3	0
COc1ccc(cc1S(=O)(=O)N)C(=O)N2CCc3c2cccc3	0
Cc1c(nc2cc(ccc2n1)C(=O)N3CCC[C@H](C3)CNC(=O)c4ccc(cc4)F)C	0
C[C@@H](C(=O)NC1(CCCCC1)C#N)Sc2ccc(cn2)S(=O)(=O)N3CCN(CC3)C	0
Cc1ccc(cc1S(=O)(=O)N(C)C)NC(=O)CN2CCN(CC2)S(=O)(=O)c3cccs3	0
CC(C)C1(CN(C1)C(=O)Nc2cccc3c2cccc3)O	0
O=C1N(CCO)c2cc(F)cc(F)c2C12CCN(c1nc3c(c(=O)[nH]1)CCCC3)CC2	1
Cc1c(nc(c(n1)N2CCCC[C@H]2C(=O)NC)Cl)C	0
C[NH+]1CCc2cc3c(c(c2[C@H]1c4c(n(c(=S)n(c4=O)c5ccccc5)c6ccccc6)[O-])OC)OCO3	0
C[C@@H](COCCC(=O)N1CCN(c2ncc(C(F)(F)F)cn2)CC1)Nc1cn[nH]c(=O)c1C(F)(F)F	1
CC[NH2+][C@H]1c2cc(ccc2S(=O)(=O)[C@@H]([C@@H]1C)C)C(C)(C)C	0
Cc1ccc(cc1N2CC[NH+](CC2)[C@H](c3cc4c(cc(cc4[nH]c3=O)C)C)c5nnnn5CCOC)Cl	0
CC1([C@H]2C[NH2+]C[C@H]2CN1C(=O)c3cc(c([n-]3)Cl)Cl)C	0
c1cc2c(cc1[C@H]3CCCN3C(=O)Nc4ccc(c(c4)C#N)F)OCO2	0
O=c1[nH]c(N2CCC3(CC2)CNc2cc(F)ccc23)nc2c1CCCC2	1
CCOC(=O)CCc1cc2c(cc(=O)oc2c(c1O)C[NH+]3C[C@H]4C[C@@H](C3)c5cccc(=O)n5C4)C	0
[H]/N=C\1/C(=C/c2cc(n(c2C)c3cc(c(cc3OC)OC)Cl)C)/C(=O)N=C4N1N=C(S4)CCCC	0
CC(C)C[n+]1c(c(cc2c1nc3ccccn3c2=O)/C(=N/c4nccs4)/[O-])N	0
O=C(N[C@H]1C[C@H](c2nnc(-c3nccs3)n2-c2ccccc2F)C1)c1ccccn1	1
Cc1ccc(-n2c(SCc3nc(-c4ccccc4)no3)nnc2-c2ccncc2)cc1	1
Cc1c2c(n[nH]1)NC3=C([C@@H]2c4ccc(cc4)OC)C(=O)CC(C3)(C)C	0
C[C@H](C(=O)Nc1c(cc(cn1)Cl)Cl)OC(=O)c2cccc(c2)S(=O)(=O)Nc3ccc(cc3)F	0
O=C(OCc1ccccc1)N1CCN(CCSc2nc3ccccc3c(=O)[nH]2)CC1	1
CCOc1cccc2c1oc(=O)c(c2)C(=O)N3CCCc4c3cccc4C	0

O=C(c1ccc(F)cc1)C1CCN(C2CCN(Cc3nc4c(c(=O)[nH]3)COCC4)C2=O)CC1	1
O=C(CCc1ccccc1F)N(Cc1ccc[nH]1)Cc1nc2c(c(=O)[nH]1)COCC2	1
CC(C)Oc1ccc(C(=O)C2CCN(CC(=O)NCc3nc4c(c(=O)[nH]3)COCC4)CC2)cc1	1
O=c1[nH][nH]c(=O)c2cc(OCc3ccsc3)ccc12	1
c1ccc(cc1)CN2CCN(CC2)C(=O)c3c4c([nH]n3)CCN(C4)C(=O)Cc5ccccc5[N+](=O)[O-]	0
Cc1cc(no1)NC(=O)C[NH+](C)CC(=O)N(C)[C@@]2(CCCCC2=O)c3ccccc3Cl	0
c1cc2c(cc1O)OC[C@H]2N3C[C@H]4C[NH2+]C[C@@H]4C3	0
CC[C@@H]1CO[C@@H](CN1Cc2c(cccc2Br)N)C	0
CCN(Cc1ccc(cc1F)C#N)C(=O)Nc2cccc(c2)[N+](=O)[O-]	0
Cc1cc(nc(n1)C)N[C@@H]2CCCCC[C@@H]2C	0
CCS(=O)(=O)c1ccc(c(c1)NC(=O)c2ccc(s2)c3ccc4c(c3)OCCO4)O	0
CCN(CC)S(=O)(=O)c1ccc(c(c1)[N-]S(=O)(=O)c2ccc(cc2Cl)Br)C	0
CCCN(CCC)C(=O)c1cc(c(=O)n(c1)C)NC(=O)c2cccnc2SC	0
CCCCN1C(=O)/C(=C/c2c(nc3c(cccn3c2=O)C)N4CCN(CC4)c5ccccc5)/SC1=S	0
c1ccc2c(c1)CCN2C(=O)COC(=O)CNC(=O)c3ccc(s3)Br	0
Cc1cc(no1)NC(=O)[C@H](c2ccccc2)Sc3nc(ns3)SC	0
COc1ccc(C(=O)C2CCN(CC(=O)N(Cc3nc4c(c(=O)[nH]3)COCC4)CC(F)F)CC2)cc1	1
CCO[C@H]1[C@H]2[C@@H]([C@H](O1)CO)OC(O2)(C)C	0
CCOC(=O)C1=c2n(c(=O)/c(=C/c3ccncc3)/s2)C(=C([C@H]1c4ccncc4)C(=O)OC)N	0
c1cc(oc1)C/N=C(/C2=NN(c3ccc(cc3S2(=O)=O)F)c4ccc(cc4)F)\[O-]	0
Cc1ccc(cc1C)N2C[C@@H](CC2=O)C(=O)NC[C@@H](c3cccn3C)N4CC[NH+](CC4)C	0
COc1cc2c(c(c1)OC)c(=O)c(co2)c3ccc(cc3)O[C@@H]4[C@H]([C@@H]([C@@H]([C@@H](O4)CO)O)O)O	0
C[C@@H](C(=O)Nc1ccc(cc1)NC(=O)/C=C/c2cc(ccc2OC)Cl)n3cncn3	0
[H]/N=C\1/C(=C\c2cn(c3c2cccc3)CCCOc4ccccc4)/C(=O)N=C5N1N=C(S5)c6cccnc6	0
O=C(CCSc1nc2ccccc2c(=O)[nH]1)N[C@H]1CC[C@H](Oc2ccncc2)CC1	1
CC1(C)CC2=NNC(=O)c3cccc4c3c2c(n4CCN2CCCC2)C1	1
CCNS(=O)(=O)c1c(c2c(s1)CN(CC2)C(=O)c3cc(nn3CC)C)C(=O)OC	0
COc1ccc(C(=O)C2CCN(C(=O)CCCc3cn4c(C)ccc4c(=O)[nH]3)CC2)cc1	1
O=c1[nH]c(N2CCC3(CC2)CNc2cc(F)cc(F)c23)nc2c1CCCC2	1
Cc1ccc2c(c1NC(=O)c3ccc(cc3F)C#N)nsn2	0
O=c1[nH]c(-c2ccc(Cl)cc2)nc2ccccc12	1
COc1ccc(C(=O)C2CCN(C(=O)CCCc3nc4c(C)scc4c(=O)[nH]3)CC2)cc1C	1
Cc1ccc(s1)S(=O)(=O)[N-]c2ccc(c(c2)N)F	0
Cc1cc2c(nc1)nc([nH]2)c3c(nns3)C	0
C[C@@H]1CN(C[C@@H](O1)C)Cc2ccccc2NC(=O)c3ccc(o3)S(	0

=O)(=O)N4CCCC4	
CC(C)(OCCO)c1ccc(-c2nc3ccc(F)cc3c(=O)[nH]2)cc1	1
Cn1cc(ccc1=O)N[C@@H]2CCc3c2ccc(c3)Br	0
C[C@@H]1CN2C[C@@]([C@@H]1O)(C[NH2+][C@@H]2c3ccc(c(c3)OC)OC)C	0
O=c1[nH]c(N2CCC3(CC2)CNc2cccc(Br)c23)nc2c1CCCC2	1
[H]/N=C\1/C(=C/c2ccc(c(c2)OC)OCc3ccc(cc3)[N+](=O)[O-])/C(=O)N=C4N1N=C(S4)C	0
CCN(C(=O)CC#N)C(=O)NC(C)C	0
C[C@H]1CN(CCN1C(=O)c2cccc(c2)[N+](=O)[O-])C(=O)CC[NH+]3CCc4c(ccs4)[C@@H]3c5ccccc5	0
Cn1c2ccc(cc2c(=O)n(c1=O)C)S(=O)(=O)N[C@H](c3ccccc3)C(=O)N4CCc5ccccc5C4	0
Cc1c(c(n(n1)C)C)S(=O)(=O)NCc2cc3n(n2)CCN(C3)CC(C)C	0
Cc1ccc(c(c1)S(=O)(=O)C2(CCCC2)C(=O)NCc3ccc[nH+]c3N4CCOCC4)C	0
Cc1cn(nn1)CC(=O)N2CC[C@](C2)(Cc3cccc(c3)c4ccc[nH+]c4)C(=O)NC(C)C	0
C1COCCN1[C@H]2C[C@H]([C@H]3[C@@H]2O3)O	0
Cc1cc(c(c(c1)C)[N-]S(=O)(=O)c2ccc3c(c2)sc(=O)n3CCC(=O)N4CCOCC4)C	0
CCCNC(=O)O[C@@H]1CC[C@@]2([C@@H]([C@]1(C)CO)CC[C@H]([C@H]2CC(=O)N3CCC(CC3)C)O)C	0
c1cc(c(cc1Cl)[N+](=O)[O-])CN2CCC[C@H](C2)O	0
COc1ccc(cc1OC)CC(=O)NC[C@H](c2cccnc2)S(=O)(=O)c3ccccc3	0
c1cc(sc1)[C@H]2CCCN2C(=O)c3ccc(c(c3)[N+](=O)[O-])N	0
c1cc2c(nc1)[C@@H](CCC2)c3cnccc3N	0
C[C@H]1Cc2cc(ccc2O1)c3cc(no3)C(=O)Nc4ccc(cc4)S(=O)(=O)N5CCOCC5	0
Cn1cc(c2c1cccc2)/C=C\3/C(=O)N(C(=S)S3)NC(=O)c4ccccc4[N+](=O)[O-]	0
Cn1c2ccccc2c(=O)c(c1O)C(=O)C[C@@H](c3ccc4c(c3)OCO4)Nc5ccc[nH+]c5	0
Cc1cc(nc(n1)NC)n2cc(cn2)Cl	0
CCCNc1c(c(=O)n2ccccc2n1)/C=C\3/C(=O)N(C(=S)S3)CCOC	0
c1ccc2c(c1)c3c(c(=O)o2)[C@@H](C(=C(O3)N)C#N)c4ccco4	0
Cc1ccsc1C(=O)OCC(=O)Nc2cccc(c2)S(=O)(=O)N3CCOCC3	0
C[C@H]1[C@H]2C[NH2+]C[C@@H]2CN1C(=O)c3cc(c([n-]3)Cl)Cl	0
C1CCC(C1)(CC(=O)N2CCCN3CCC[C@H]3C2)[NH3+]	0
CC1(CCC[C@H]1NC(=O)c2cc(c(cc2[N+](=O)[O-])F)F)C	0
Cn1c(=O)c2cc(sc2n(c1=O)C)C(=O)NCC[NH+]3CCN(CC3)c4ccccc4	0
c1ccc2c(c1)C(=NS2(=O)=O)NCCCC(=O)OCC(=O)Nc3c(c4c(s3)CCC4)C#N	0
CC(=[NH2+])C(C#N)C(=O)COC(=O)c1cccc(c1)S(=O)(=O)N2CCc3ccccc3C2	0
CC1(Cc2c(c([nH+]c3c2c4c(s3)c(ncn4)N(CCO)Cc5ccccc5)N6CCOCC6)CO1)C	0
O=C(CCc1ccccc1)N(Cc1nc2c(c(=O)[nH]1)COCC2)CC1CCCC1	1
CC1=C([C@@]2(c3ccccc3-	0

c4c2nc(o4)N)C(=O)N1CC#C)C(=O)OCCOC	
Cc1ccc(cc1Oc2ccc(cc2Br)[C@H](C)N)[N+](=O)[O-]	0
CCOc1ccc(-c2nnc([C@H]3C[C@H](NC(=O)c4ccnc5cccnc45)C3)n2-c2ccccn2)nc1	1
CC1CCC(CC1)N(C)C(=O)N[C@H](C)CC(=O)[O-]	0
C[C@H]1[C@@H](CC[C@@H]1O)Cc2c3ccccc3n(n2)C	0
CCn1ccc(n1)C(=O)Nc2c(c3c(s2)CCCC3)C(=O)NCc4ccc[nH+]c4	0
c1c(cnc2c1nc([nH]2)CCC(=O)NC[C@@H]3CCC[NH+]4[C@H]3CCCC4)Br	0
COc1ccc(C(=O)C2CCN(C3CCN(Cc4nc5c(c(=O)[nH]4)COCC5)C3=O)CC2)cc1	1
CCOc1ccc(-c2nnc([C@H]3C[C@H](NC(=O)c4ccnc5cc(F)cnc45)C3)n2-c2ccncc2)nc1	1
c1ccc2=[NH+]CC(=c2c1)C3=NN[C@H](O3)SCC(=O)Nc4ccc(cc4)S(=O)(=O)N5CCCC5	0
c1ccc(c(c1)NC(=O)C(=O)NCCc2cn3ccccc3[nH+]2)OC(F)(F)F	0
c1ccc2c(c1)CC[NH2+][C@H]2C3(CCC4(CC3)OCCO4)O	0
Cc1ccc(c(c1)C(=O)N[C@@H]2CCCCC[C@@H]2C(=O)[O-])C	0
O=C1NC2(CCN(c3nc4c(c(=O)[nH]3)CCCC4)CC2)c2ccccc21	1
Cc1ccc(cc1)[C@H]2[C@@H]3C(=c4cc(ccc4=[NH+]3)OC)CCN2C(=O)Nc5ccc(cc5OC)OC	0
O=C1N(CCO)c2cccc(Cl)c2C12CCN(c1nc3c(c(=O)[nH]1)CCCC3)CC2	1
COc1cccc(c1[O-])/C=N/Nc2nc(cc(n2)c3ccc(c(c3)OC)OC)c4ccccc4	0
c1cc(c(cc1C(=O)N2CCC[C@H](C2)C(=O)Nc3ccc(nc3)n4cc[nH+]c4)Cl)Cl	0
Cn1cc(cn1)Cc2c(nc(s2)N)C3CC3	0
CCc1c(c(=O)n(n1CC)[C@@]2(CCS(=O)(=O)C2)C)[NH3+]	0
CC(=O)Nc1ccc2c(c1)s/c(=N/C(=O)CCCS(=O)(=O)c3ccc(cc3)Cl)/n2CCOC	0
Cc1c(nnc(c1C#N)N2CCC(CC2)c3ccc(cc3)O)C	0
CCOc1cc(ccc1O)[C@@H]2c3c(n[nH]c3C(=O)N2CC[NH+]4CCOCC4)c5ccc(cc5)C	0
O=c1cc(-c2ccccc2)oc2ccccc12	1
c1cc(oc1)C(=O)N2CCC(CC2)NC(=O)CCc3ccc(cc3)S(=O)(=O)N4CCOCC4	0
Cc1c2c(n(n1)c3nc4ccccc4s3)NC(=O)C[C@H]2c5cccc(c5)OC6CCCC6	0
C[C@H]1CC[C@@H]([C@@H]1C)NC(=O)NC2CCC(CC2)C(=O)[O-]	0
CC[C@@H]1CS[C@@H](N1)c2cc3ccccc3o2	0
Cc1ccccc1NCc2nc3nc4c(c(n3n2)[O-])CCC4	0
Cc1nc(nn1c2c(cccc2Cl)Cl)C(=O)Nc3ccc4c(c3)COC4	0
C[C@H]1CCC[C@@H](C1)NC(=O)Nc2cc(c(cc2Br)OC)OC	0
Cc1nc(no1)[C@@]2(CC(CSC2)(C)C)N	0
c1cc(ccc1c2nnc(o2)SC[C@H](c3ccc(cc3)Br)O)[N+](=O)[O-]	0
CN1C(=O)C[C@@H](C12CC[NH+](CC2)Cc3ccc(s3)Cl)C(=O)O	0
Cc1c(sc(n1)C)C(=O)N(c2ccc3c(c2)OCCO3)[C@H](c4ccccc4)C(=O)NCc5ccco5	0
c1cc(c(cc1Cl)Cl)C(=O)NNS(=O)(=O)c2cc(c(nc2)Cl)Cl	0

**c1nc([nH]n1)Sc2nc(c(s2)C=O)Cl**	0
**Cc1cccc(c1C)N2C[C@@H](CC2=O)C(=O)Nc3ccc(cc3)S(=O)(=O)Nc4ncccn4**	0
**CC1=NC2=C([C@H](C1C(=O)OCCOC)c3ccc(cc3)[N+](=O)[O-])C(=O)C[C@H](C2)c4ccccc4**	0
**Cc1nc2c(n1[C@@H]3CCS(=O)(=O)C3)cc(nc2C(=O)Nc4cccc(c4)OC)c5cccs5**	0
**Cc1cc(nc(c1NC(=O)c2cc(sc2)Br)C)C**	0
**Cc1c2ccccc2oc1C(=O)NCCN3C(=O)/C(=C/c4ccc5c(c4)OCO5)/SC3=O**	0
**c1ccn2c(c1)nnc2CN[C@H]3CCc4c3ccc(c4)N**	0
**Cc1cccc(c1)CN2CCN(CC2)C(=O)[C@H](C)NS(=O)(=O)c3ccc(cc3)NC(=O)C**	0
**N#Cc1ccccc1Cn1c(=O)n(C[C@H]2CC[C@H](C(=O)Nc3ccncc3)CC2)c(=O)c2ccccc21**	1
**c1ccc(cc1)C[NH+]2CCN(CC2)C(=O)Cc3ccc(cc3)NC4=[NH+][C@H]5CS(=O)(=O)C[C@H]5S4**	0
**Cc1ccc(cc1)NC(=O)COc2ccc(cc2Cl)/C=C/3\C(=O)N(C(=O)S3)[C@H](C)C(=O)OC**	0
**CS(=O)(=O)c1ccc(-c2nnc([C@H]3C[C@H](NC(=O)c4ccnc5cccnc45)C3)n2-c2ccccc2F)nc1**	1
**O=C(CCc1ccncc1)Nc1nnc2n1CCS2**	1
**Nc1ccc(-c2nc(O)c3cccc(O)c3n2)cc1**	1
**Clc1ccc2c(c1)sc1nncn12**	1
**O=c1[nH]c(-c2ccc(C(F)(F)F)cc2)nc2c1CCSC2**	1
**Cc1cc(C)c2c(c1)sc1nncn12**	1
**Oc1nc(-c2ccc(C(F)(F)F)cc2)nc2c(O)cccc12**	1
**[H]\N=C\1/C(=C\c2cc(n(c2C)c3ccc(cc3)OCC)C)/C(=O)N=C4N1C(=NS4)S(=O)(=O)CC(C)C**	0
**Cc1c(cnn1c2cccc(c2)Cl)C(=O)Nc3cc4c(cc3Br)OCCO4**	0
**Cc1ccn2c1nc(c(c2=O)/C=C\3/C(=O)N(C(=S)S3)Cc4ccccc4)NC[C@@H](c5ccccc5)O**	0
**Cc1ccc(cc1)NC(=O)Nc2ccc(cn2)Cl**	0
**COc1cccc2sc3nncn3c12**	1
**c1ccc2c(c1)c(cn2CC(=O)N3CCCCCC3)S(=O)(=O)CC(=O)Nc4ccc(cc4)OC(F)(F)F**	0
**Cc1c(c2c(n1c3ccc(cc3)OC)ccc(c2C[NH+](C)C)O)C(=O)c4cc(ccc4OC)OC**	0
**Cc1cc(c(o1)C)c2c[nH]c(n2)[C@H]3CCCCCN3**	0
**C[NH+](C)CCN(c1nc2c(ccc(c2s1)Cl)OC)C(=O)CCS(=O)(=O)c3ccc(cc3)OC**	0
**CN1CCCc2c1nc(N1CCC3(CC1)C(=O)N(CC(F)(F)F)c1cc(F)cc(F)c13)[nH]c2=O**	1
**COc1ccccc1NC(=O)c1ccc(NC(=O)CCc2nc3ccccc3c(=O)[nH]2)cc1**	1
**CCc1ccc(s1)S(=O)(=O)Nc2ccccc2C#CC[NH3+]**	0
**CN1CCCc2c1nc(N1CCC3(CC1)C(=O)N(CS(C)(=O)=O)c1cc(F)cc(F)c13)[nH]c2=O**	1
**Cc1cc2c(c(=O)n1CCC[NH+](C)C)[C@H](C(=C(O2)N)C#N)c3cc4c(c(c3)OC)OCO4**	0
**Cn1cc(cn1)CNc2c(nsn2)Cl**	0
**C[C@@H](c1ccccc1)Oc2ccc(cc2)/C=C\3/C(=[NH2+])N4C(=NC3**	0

=O)SN=C4S(=O)(=O)C	
c1ccc(cc1)CCCCNC(=O)c2cc(ccc2N3CCOCC3)S(=O)(=O)N4CCOCC4	0
C[C@@H](c1nc(nc(n1)Nc2ccccc2)N)N3CCN(CC3)Cc4ccc(c(c4)F)OC	0
CCOC(=O)N1CCN(CC1)C(=O)c2ccccc2NS(=O)(=O)c3ccc(c(c3)C)F	0
Cc1c2c(n(n1)c3ccccc3)Oc4c(c(=[NH2+])n(cn4)CCCN5CCOCC5)[C@H]2c6ccccc6	0
c1nc2c(c3c(s2)CCCC3)c(=O)n1CC(=O)N/N=C/c4c(c(c(c(c4F)F)F)F)F	0
COC(=O)c1c(ccs1)NC(=O)CN2CCCN(S2(=O)=O)Cc3ccccc3Cl	0
COc1ccc(c(c1)[C@H]2Nc3ccc(cc3-c4c(nc(nn4)SC)O2)Br)OC	0
CCc1cc2c(c(c1)C(C)(C)C)OCCNC2	0
Cc1cccc(c1)c2nc(on2)[C@@H]3Cc4c(nc[nH]4)CN3C	0
Cc1ccc(cc1)[C@H]2c3c(nn(c3OC(=C2C#N)N)c4ccc(cc4)C)C	0
Cc1cc2c(nc1)n(c(n2)N)C[C@H]3Cc4c3cccc4	0
Cc1cc2c(cc1CNC(=O)Cn3c4cc5c(cc4nc3C(F)(F)F)OCCO5)OCCCO2	0
CS(=O)(=O)[C@H]1CCC[C@@H]1Nc2ccc3c(c2)OCO3	0
COc1ccc(C(=O)C2CCN([C@H]3CCCN(CCC4NC(=O)C5=C(CCOC5)N4)C3=O)CC2)cc1	1
CCN(c1ccccc1)C(=O)Cn2c3ccccc3[nH+]c2[C@@H]4CC(=O)N(C4)c5ccccc5F	0
Cc1ccc(cc1)S(=O)(=O)CNC(=O)[C@@H](c2ccco2)N(c3cccc(c3)Cl)C(=O)CC(C)C	0
Cc1cc(=O)n(cc1NC(=O)N[C@H]2[C@H]3[C@@H]2c4ccccc4CC3)C	0
C1CCCC(CC1)(CN[C@@H]2CCOC2)O	0
c1cnn(c1)c2ccc(cc2)c3cnco3	0
c1cc2c(cc1C(=O)N/N=C/c3ccc(o3)I)OCO2	0
COc1ccc(C(=O)C2CCN(C3CCN(Cc4nccc(=O)[nH]4)C3=O)CC2)cc1	1
COc1ccc(c(c1)N2C(=O)c3ccc(cc3C2=O)N)Br	0
Cc1ccc(cc1C)c2ccc(nn2)n3c(c(c(n3)C)S(=O)(=O)NCC4CC4)C	0
c1cc(sc1)CN2Cc3c([nH]c[nH+]3)C[C@H]2c4nc(no4)c5ccncc5	0
CCNc1ccc(c(n1)CSc2nccc(n2)C)Cl	0
COc1ccc2c(c1)CC1(CCN(CC(=O)N(Cc3nc4c(c(=O)[nH]3)COCC4)CC(F)F)CC1)C2=O	1
CC1=NN([C@@](C1)(C(F)(F)F)O)C(=O)c2cc(c(c(c2)Cl)O)Cl	0
Cc1cc2c(=O)[nH]c(-c3ccc(C(C)(C)O)cc3)nn2n1	1
O=C1Nc2cccc(F)c2C12CCN(c1nc3c(c(=O)[nH]1)CCCC3)CC2	1
CN1c2ncccc2C(=O)NC1c1ccc(NC(=O)C2CCC2)cc1	1
Cc1nc(cs1)COc2ccccc2/C=C/C(=O)NCc3ccc([nH+]c3)N4CCOCC4	0
CCC[C@H]1O[C@@H]2C[C@@H]3[C@@H]4CCC5=CC(=O)C=C[C@@]5([C@@H]4[C@H](C[C@@]3([C@@]2(O1)C(=O)CO)C)O)C	0
CCOC(=O)C1=C(N=c2n(c(=O)/c(=C/c3cc(cc(c3O)OC)Br)/s2)[C@@H]1c4ccc(c(c4)OC)OC)C	0
CC(C)N(Cc1nc2c(c(=O)[nH]1)COCC2)C(=O)CN1CCC(C(=O)c2ccc(F)cc2)CC1	1

**C[C@H](c1[nH+]c(c2ccccc2n1)N3CCOCC3)N4CCN(CC4)S(=O)(=O)c5ccc(cc5)n6cccn6**	0
**Cc1cccc(c1)NS(=O)(=O)c2ccc(c(c2)[N-]S(=O)(=O)c3ccc(cc3Cl)C)C**	0
**C[C@@H]1CN(c2cc(F)c3c(c2)N(C)C(=O)C32CCN(c3nc4c(c(=O)[nH]3)CCCN4C)CC2)C[C@H](C)O1**	1
**COc1cc(=O)n2c(c1C(=O)NCc3ccco3)CCN(CC2)C(=O)C#Cc4ccccc4**	0
**C[C@@H](c1cccc(c1)n2ccnc2)NC(=O)Cn3c4ccccc4cn3**	0
**C[C@@H]1C(=C(C(=N1)C(=O)Nc2cc(ccc2Oc3ccccc3)S(=O)(=O)N4CCCCC4)C)C(=O)C**	0
**COc1cc(ccc1OCc2ccc(cc2)Cl)/C=C\3/C(=O)N=C(S3)[N-]c4ccc(cc4)O**	0
**c1ccc(cc1)S(=O)(=O)N(CC(=O)Nc2ccc(cc2)S(=O)(=O)N3CCOCC3)c4ccc(c(c4)Cl)Cl**	0
**Cc1c(c(n(c(=O)c1C#N)C)N2CC[NH+](CC2)CCO)/C=C\3/C(=O)N(C(=S)S3)[C@H](C)c4ccccc4**	0
**CC1(C[C@@H]1Nc2c(nsn2)Cl)C**	0
**C[C@@H](c1ccccc1)NC(=O)C(=O)NCC[NH+]2CCN(CC2)C(=O)c3ccc(cc3)[N+](=O)[O-]**	0
**c1ccc2c(c1)OC[C@@H](O2)C(=O)Nc3cccc(c3)C#Cc4cccc[nH+]4**	0
**Cc1c2cc(ccc2oc1C(=O)OCC(=O)Nc3c(c4c(s3)CCC4)C(=O)N)Br**	0
**CS(=O)(=O)c1ccc(-c2nnc([C@H]3C[C@H](NC(=O)c4cccc5cccnc45)C3)n2-c2ccccc2F)nc1**	1
**Cc1ccc(cc1N2CCCCS2(=O)=O)N**	0
**CC1(C)OC(=O)N([C@H]2CC[C@H](NC(=O)c3cccc4cccnc34)CC2)[C@H]1c1ccccc1**	1
**CC[NH+](CC)CCCN1CC(=O)N2[C@H](C1=O)Cc3c4ccccc4[nH]c3[C@@H]2c5ccccc5OCC**	0
**C[C@@H]1CCCCN1c2c(cc(cc2Cl)C(=O)OC)N**	0
**Cc1c2cc(ccc2oc1/C(=N/c3nnns3)/[O-])Cl**	0
**CC(C)(O)c1ccc(-c2cn3cc(F)cc3c(=O)[nH]2)cc1**	1
**Cc1c(sc2c1c(nc(n2)Cn3ccccc3=O)N)C**	0
**C[C@@H]1CC(O[C@@H](O1)[C@@H]2CCC=CC2)(C)C**	0
**CC(C)N1CCN(CC1)C(=O)N[C@H]2CCC[C@H](C2)C(=O)[O-]**	0
**c1c(ncc(n1)Br)NC(=O)[C@@H]2C[C@H]3CCCC[C@@H]3N2**	0
**Cc1cc(cc(c1)C(=O)Nc2c(c(cc(n2)C)C)c3nc(on3)C)C**	0
**CCn1c(c(c(n1)C)N)NC2(CCCCC2)CO**	0
**N#Cc1ccc(O[C@H]2CC[C@H](NC(=O)CCSc3nc4ccccc4c(=O)[nH]3)CC2)cc1**	1
**CC(C)NC(=O)[C@H]1CCCN(C1)c2nc3c(s2)c(=O)n(cn3)CC(=O)NC4CCCC4**	0
**C[C@]12CC[C@@H]3[C@@H]([C@@H]1CC[C@@H]2O)CCC4=C/C(=N\OCC(=O)NCCc5c[nH]cn5)/CC[C@]34C**	0
**c1cc(c2c(c1Cl)con2)Cl**	0
**COc1ccc(cc1)C2=NN([C@H](C2)c3ccco3)C(=O)CN4C(=O)[C@]5(CCCc6c5cccc6)NC4=O**	0
**CCCn1c(cc(n1)N)C(F)(F)F**	0
**COC(=O)c1ccc(cc1)S(=O)(=O)Nc2cc(n[nH]2)c3ccc(s3)Br**	0
**O=C(CN1CCC(c2n[nH]c3cc(F)ccc23)CC1)NCc1nc2c(c(=O)[nH]1)COCC2**	1

CC(C)CNC(=O)c1ccc2c(c1)n3c(nn(c3=O)CC(=O)c4ccc(cc4)Cl)n(c2=O)C(C)C	0
Cc1ccc(cc1)[C@@H]2[C@@H]3CC=C[C@H]3c4c(ccc(c4N2)C)C(=O)[O-]	0
c1cc(cc(c1)[N+](=O)[O-])/C=C\2/C(=O)c3ccc(c(c3O2)C[NH+]4C[C@H]5C[C@@H](C4)c6cccc(=O)n6C5)O	0
CCOc1ccc(-c2nnc(C34CC(NC(=O)c5ccnc6cccnc56)(C3)C4)n2-c2cccnc2)nc1	1
O=C(c1ccc(F)cc1)C1CCN(CC(=O)N(Cc2ccccc2)Cc2nc3c(c(=O)[nH]2)COCC3)CC1	1
C[C@H](C(=O)Nc1ccc(cc1)Br)Sc2nnnn2Cc3cccs3	0
Cc1c([nH+]c[nH]1)CN2CCc3c(nc[nH]3)[C@H]2c4ccc(c(c4)Cl)Cl	0
COc1ccc(-c2nnc(C3CCN(CC(=O)NCc4nc5c(c(=O)[nH]4)COCC5)CC3)o2)cc1	1
CN(C)C(=O)c1cc[nH+]cc1[O-]	0
C1CCc2c(c(c(c(n2)Cl)C#N)C#N)C1	0
C[C@@H]1CN(C[C@H](O1)c2ccc(cc2)F)C(=O)NCC(C)(C)c3cc[nH+]cc3	0
[H]/N=c\1/c(cc2c(n1CCc3ccc(c(c3)OC)OC)nc4c(cccn4c2=O)C)S(=O)(=O)c5ccc(cc5)Br	0
Cc1cccc(c1)C(=O)N2[C@H](COC23CCN(CC3)C(=O)Cc4ccccc4)C(=O)NCc5cc[nH+]cc5	0
Cc1ccccc1C(=O)N2[C@@H](COC23CCN(CC3)C(=O)c4ccco4)C(=O)NCC(C)C	0
CC(C)C(=O)N1CCC(CC1)c2nc(cc(n2)O)[C@@H]3CCCN(C3)C(=O)[C@@H]4CCCO4	0
c1ccc(cc1)C(=O)[C@@H]2[C@H]3[C@@H]([C@@H]4[NH+]2C=Cc5c4cccc5)C(=O)N(C3=O)c6ccc(cc6)[N+](=O)[O-]	0
Cc1c(c(n(c(=O)c1C#N)C)NCc2ccc3c(c2)OCO3)/C=C/4\C(=O)N(C(=S)S4)C	0
Cc1cc(c(c2c1C(=O)/C(=C/c3ccc4c(c3)OCO4)/O2)CN5CCN(CC5)CCO)O	0
c1cc2cn[nH]c2c(c1)NCc3cc4c(c(c3)Br)OCO4	0
N#Cc1c(Cn2c(=O)n(C[C@H]3CC[C@H](C(=O)O)CC3)c(=O)c3ccccc32)cccc1C(F)(F)F	1
C[C@@]1(CC[C@](C1(C)C)(C)C(=O)[O-])C(=O)NC	0
CNCc1ccc(-c2[nH]c3cc(F)cc4c3c2CCNC4=O)cc1	1
COc1cc(cc(c1OC)OC)/C=C/2\C(=NC(=N2)c3cc(ccc3Cl)I)[O-]	0
Cc1ccc2c(c1)c3c([nH]2)nc(nn3)SC(C)C	0
C[C@H]1CCCCCN1c2c(cc(cn2)Br)CO	0
CCOc1ccc2c(c1)sc(n2)N3C(=O)CNC3=S	0
c1cc(sc1)[C@H]2c3cn[nH]c3N=C(CS2)O	0
C[C@@H](C12CC3CC(C1)CC(C3)C2)NC(=O)CSc4c(c5c(s4)c(=O)[nH]c(=O)[nH]5)C#N	0
COc1ccc(cc1)c2[nH]nc(n2)CNC(=O)[C@H](c3ccccc3)N4CCSCC4	0
Cc1c(c(=O)n(n1C)c2ccccc2)N(C)S(=O)(=O)c3cccc(c3)C(=O)Nc4ccccc4Cl	0
Cn1c2c(cc(cn2)C(=O)NCc3cn(nc3c4ccccc4)Cc5ccccc5)c(=O)n(c1=O)C	0

**COc1cc(cc(c1OC)Cl)/C=N/N=C/2\NC(=O)[C@@H](S2)CC(=O)Nc3ccccc3**	0
**C1C=COC2=C[C@@H]([C@H]3C(=C21)SC=N3)S**	0
**CC(C)(O)c1ccc(-c2nc3cccc(Cl)c3c(=O)[nH]2)cc1**	1
**O=c1[nH]nc2c3c(cccc13)NC1=C2CCCCCC1**	1
**C1CCC(CC1)C(=O)N[C@H](C2CC2)/C(=N/O)/N**	0
**C[C@@H]1CC[C@H](C[C@@H]1C)N[C@@H](C)C(=O)NC(=O)N**	0
**O=C1Oc2ccccc2C12CCN(c1nc3c(c(=O)[nH]1)CCCC3)CC2**	1
**C1CCC[NH+](CC1)[C@H]2CCCC[C@@H]2C(=O)[O-]**	0
**c1c(ncnc1Cl)Nc2cc(ncn2)Cl**	0
**CCc1cc2c(cc1O)occ(c2=O)n3ccccc3=O**	0
**[C-]#[N+]c1c(Cl)cccc1CN1CCC(N2CCC(C(=O)c3ccc(OC)cc3)CC2)C1=O**	1
**O=C(CCc1ccccc1)N(CCO)Cc1nc2c(c(=O)[nH]1)COCC2**	1
**CCOc1ccc(C(=O)C2CCN(CC(=O)NCc3nc4c(c(=O)[nH]3)COCC4)CC2)cc1F**	1
**CSc1ccc(cc1)CC(=O)/N=c/2\n(c3ccc(cc3s2)S(=O)(=O)N)CC#C**	0
**CC(C)(C#Cc1ccc(cc1)[C@@H]2C[C@H]3CN(C(=O)[C@@]34[NH+]2CCC4)Cc5ccccc5OC)O**	0
**C[C@@]1(C(=O)N(C(=O)N1)CC(=O)N2CCN(CC2)S(=O)(=O)Cc3ccccc3)c4ccc(cc4)F**	0
**C[C@@H](c1ccc(cc1)n2cncn2)NC(=O)c3cc(ccc3NS(=O)(=O)C)Cl**	0
**c1cc(cc(c1)Br)C(=O)N2[C@](CC(=N2)C(F)(F)F)(c3ccc(cc3)[N+](=O)[O-])O**	0
**CNc1cc([nH+]c(n1)C2CCN(CC2)S(=O)(=O)N3CCCCC3)[C@H]4CC(=O)N(C4)C**	0
**C[C@H]1CCC[C@H](C1)NC(=O)C(=O)Nc2ccc3c(c2)CCC3**	0
**CCOC(=O)N1CCN(CC1)S(=O)(=O)c2ccc(cc2)C(=O)Nc3ccc4c5c3cccc5CC4**	0
**Cc1c(oc(c1c2nc3cc(ccc3o2)N)C)C**	0
**CC(C)C1Cc2[nH]c(=O)c3cccnc3c2CO1**	1
**Cc1nc2n(n1)c(c(s2)[C@H](c3ccc(cc3)Br)N4CCN(CC4)Cc5ccccc5)[O-]**	0
**O=C1CCN(c2ccccc2C(=O)NCCc2nc(-c3ccccn3)no2)C(=O)N1**	1
**c1ccc(c(c1)c2nc3cc(ccc3o2)O)Br**	0
**c1cc(ccc1C(=O)CSc2nc3c(c(n2)O)nc[nH]3)Br**	0
**Cc1c(c(n(n1)[C@@H]2CCCCC[C@@H]2O)C)O**	0
**C[C@@H](Cn1cccn1)n2cncc2c3c[nH+]ccc3N**	0
**COc1ccc(cc1)[C@@H]2[C@H](O[N-]S2)O**	0
**Cc1c(sc(n1)NC(=O)CCN2C(=O)/C(=C\c3ccc(cc3)OC)/SC2=S)C(=O)OC**	0
**CS(=O)(=O)[N-]c1cccc(c1)C(=S)N**	0
**CCn1c2ccccc2c3c1nc(nn3)SCC(=O)NC[C@@H]4C[NH+](CCO4)Cc5ccccc5**	0
**Cc1ccc2c(=O)[nH]c(CCCC(=O)N3CCC(Oc4ccc(C#N)cc4)CC3)nn12**	1
**Cc1ccc(cc1)N([C@H](C)C(=O)Nc2ccc(cc2)S(=O)(=O)N3CCOCC3)S(=O)(=O)C**	0
**C[C@@H]1CCCN2C1=N[C@@H](C2=[NH2+])c3ccc4c(c3)CCO**	0

4	
CCCc1cc2c(s1)nc(n(c2=O)NC(=O)c3c(cn(n3)C)Br)C	0
CC(C)(C)c1cc(n[nH]1)C(=O)N2CCC(CC2)(C(F)(F)F)O	0
c1cc(c(cc1N)Br)c2nn3cnnc3s2	0
CS(=O)(=O)N1CCCC[C@@H]1C(=O)NCCN2C(=O)/C(=C/c3cccs3)/SC2=O	0
CCn1c2ccc(cc2[nH+]c1CCC(=O)Nc3cnn(c3)Cc4ccccc4)S(=O)(=O)N(C)C	0
CCc1ccccc1NC(=O)[C@]23CC[C@H](C2(C)C)c4c3nc(c(n4)C#N)C#N	0
Cc1cc2c(cn1)nc3cc(ccc3c2N)N(C)C	0
CCOC(=O)NC(=O)c1ccsc1NC(=O)[C@H]2CCCN2S(=O)(=O)c3ccc(cc3)Cl	0
c1ccc2=[NH+]CC(=c2c1)C[C@@H](C(=O)[O-])NC(=O)CCN3C(=O)[C@H]4C=CC=CC4=NC3=O	0
O=c1[nH]c(-c2ccsc2)nc2c1COCC2	1
COC(=O)c1ccc(-c2nc3c(c(=O)[nH]2)COCC3)cc1	1
C[C@H](C(=O)Nc1ccc(cc1)N2CCOCC2)N3C(=O)[C@@H](NC3=O)CC4=c5ccccc5=[NH+]C4	0
CC(C)(C)C1Cc2[nH]c(=O)c3ccccc3c2CO1	1
CN1CCCc2c1nc(N1CCC3(CC1)C(=O)N(C)c1cc(N4CCCCC4)cc(F)c13)[nH]c2=O	1
Cc1c(c(sc1C(=O)N(C)C)NC(=O)c2c(cnn2C)I)C(=O)OC(C)C	0
Cn1cc(ccc1=O)NC(=O)[C@@H]2C[C@H]3CCCC[C@@H]3N2	0
C[C@@H]1CN(C[C@H](O1)C)C(=O)COc2ccc(cc2OC)/C=N/Nc3c(cc(cn3)C(F)(F)F)Cl	0
c1ccc2c(c1)cc(o2)C(=O)C3=C(C(=O)N([C@H]3c4cccc(c4)Br)CCCn5ccnc5)[O-]	0
C[NH+]1CCN(CC1)S(=O)(=O)c2cccc(c2)C(=O)Nc3ccccc3C(=O)N4CCCCC4	0
Cc1cccc2c1sc1nncn12	1
c1ccc(cc1)c2cc([nH]n2)C(=O)N3CC[NH+](CC3)Cc4cc(=O)n5ccsc5n4	0
[H]/N=C/1\CCCN1c2cc(ccc2Cl)C#N	0
c1cc(nnc1N/N=C/2\C[C@@H]3[C@@H]2CC=C3)Cl	0
COc1ccc(N2CCN(c3nc4c(c(=O)[nH]3)CSCC4)CC2)cc1	1
C[C@@H]1C[C@H](CN(C1)S(=O)(=O)c2ccc(cc2)[N-]S(=O)(=O)c3cccc(c3Cl)Cl)C	0
Cc1c(nnn1c2ccc(cc2F)Br)N	0
Cc1c(sc(n1)N)S(=O)(=O)N2CC[C@@H](C2)[NH+](C)C	0
C[C@H]1C[C@@H](C[NH+](C1)Cc2[nH+]c3c(n2Cc4ccc(c(c4)Cl)Cl)c(=O)[nH]c(=O)n3C)C	0
Cc1nc2c(c(n1)Cl)CCCC2	0
C#CCN(Cc1nc2c(c(=O)[nH]1)COCC2)C(=O)CN1CCC(C(=O)c2ccc(F)cc2)CC1	1
Cn1c2ccc(cc2n(c(=O)c1=O)C)S(=O)(=O)Nc3ccc(cc3)Cc4cccc[nH+]4	0
C[C@H](c1ccc(c(c1)F)F)N2CCN(CC2)C3CNC3	0
O=C(CN1CCC(C(=O)c2ccc3c(c2)OCO3)CC1)NCc1nc2c(c(=O)[nH]1)COCC2	1
c1cc(cc(c1)I)Oc2ccc(c3c2non3)N	0
CCCC[NH+]1CCN(CC1)C(=O)c2cn(nc2S(=O)(=O)N3CCN(CC3	0

)c4cccc(c4)Cl)CC	
CCOC(=O)[C@@H](Cc1ccc(cc1)/N=C/c2ccc(cc2[O-])O)N3C(=O)c4ccccc4C3=O	0
C[C@]12CC[C@H](C[C@H]1CC[C@@H]3[C@@H]2C(=O)C[C@@]4([C@@H]3CC[C@@H]4C(=O)CO[C@@H]5[C@H]([C@@H]([C@H]([C@@H](O5)C(=O)OC)O)O)O)C)O	0
[H]/N=C\1/C(=C/c2cc(n(c2C)c3ccc(c(c3)Cl)C)C)/C(=O)N=C4N1N=C(S4)c5ccco5	0
CC(C)[C@@H]1C(=O)NC(C(=O)N1[C@H]2CCCCC2(C)C)(C)C	0
COC(=O)C[C@@H](c1ccc(c(c1)Oc2ccccc2)F)N[C@H]3C4=C(CCCC4)C(=O)N3Cc5ccc6c(c5)OCO6	0
CCN(CC)C(=O)[C@@H]1CCCN(C1)C(=O)c2coc(n2)CN3CC[NH+](CC3)C[C@@H](CCC=C)O	0
c1cc(sc1)S(=O)(=O)[N-]c2ccc(nc2)Oc3c4ccc(cc4ncn3)Cl	0
CCN(C(=O)N1CCC(C(=O)c2ccc3ncn(C)c3c2)CC1)c1ccc(C(=O)O)cc1	1
CCC[C@H]1CC(=O)O[C@]1(C)C(=O)C[n+]2cc(cc(c2)Br)Br	0
c1cc(sc1)CN(CC(=O)NC2CCCC2)C(=O)C[S@](=O)CC(=O)Nc3ccc4c(c3)OCO4	0
Cc1ccc(c(c1)[O-])C(=O)Nc2cccc(c2)Br	0
COc1ccccc1N1CCC(c2nc3c(c(=O)[nH]2)CCCC3)CC1	1
COc1ccc(C(=O)C2CCN(C3CCN(Cc4nc5c(c(=O)[nH]4)CCC5)C3=O)CC2)cc1	1
Cc1c(c(=O)n(n1C)c2ccccc2)NC(=O)[C@H](C)Sc3nnc(n3C)C(F)(F)F	0
c1ccc2c(c1)ncc(n2)Oc3ccc(cc3N)F	0
C[C@@H](c1ccccc1)N(Cc1nc2c(c(=O)[nH]1)COCC2)C(=O)CN1CCC(C(=O)c2ccc(F)cc2)CC1	1
c1ccc(cc1)Oc2ccc(cc2)N\3[C@H]4CS(=O)(=O)C[C@H]4S/C3=[NH+]\C(=O)COCC(=O)[O-]	0
Cc1ccsc1C(=O)c2cc3c(s2)CCCC3	0
C[C@H]1CCCCCN1C(=O)[C@@H]2CCCC[C@@H]2C(=O)O	0
c1ccc(c(c1)CNc2ccc(cn2)S(=O)(=O)N3CCCC3)CN4CCOCC4	0
CC[C@@H](C)N1[C@@H]([C@@]23C=C[C@@H](O2)[C@@H]([C@@H]3C1=O)C(=O)Nc4ccc(cc4)C(C)C)C(=O)NC5CCCCC5	0
CN(C)c1ccc(cc1)[C@@H]2CC(=O)Nc3c2cc4c(c3)OCO4	0
C[NH+](C)CCN1[C@@H](C(=C(C1=O)O)C(=O)c2ccc3c(c2)OCCO3)c4ccc(cc4)Br	0
Cc1cc(c2c(n1)n3c(n2)CCCCC3)C(=O)Nc4ccc5c(c4)sc(n5)N6CCOCC6	0
C/C=C/C(=O)N1C[C@@H](C[C@H](C1)C)C	0
CC[NH+](CCNC(=O)c1c(c2c(ncnc2s1)N3CCN(CC3)C)C)Cc4ccccc4	0
C[C@H]1C=C2C(=C(C([C@H]([C@@]2(CN1C)C)c3ccccc3)(C#N)C#N)N)C#N	0
COc1ccc(cc1)[C@H]2CC(=NN2C(=O)CSc3[nH]c4c(cnn4c5ccc(cc5)S(=O)(=O)N)c(=O)n3)c6cccs6	0
Cc1ccc(c(c1)N2CC3(CCCC3)NC[C@@H]2C)OC	0
CCOc1cccc(c1)[C@H]2C(=C(C(=O)N2CC[NH+](C)C)[O-])C(=O)c3c(c([nH]c3C)C(=O)OC)C	0

**Cc1ccc(cc1)S(=O)(=O)[N-]c2ccc3c(c2)[nH+]c(n3C)CN4CCN(CC4)C(=O)C(C)C**	0
**CC[NH+]1Cc2c(c3ccccc3nc2/C(=C\c4cn(nc4c5ccncc5)C)/C1)C(=O)[O-]**	0
**Cc1ccc2c(=O)[nH]c(-c3ccc([C@@](C)(O)CO)cc3)cn12**	1
**Cc1ccc2c(c1C)[nH]c(n2)CCn3cc[nH+]c3c4ccoc4**	0
**CO/C(=C\1/[C@@H]([C@@H](C(=NC1=O)SCC(=O)Nc2ccc(cc2)Br)C#N)c3ccccc3[N+](=O)[O-])/[O-]**	0
**O=c1[nH]c(-c2ccc(F)cc2)nc2c(O)cccc12**	1
**Cc1cc(ccc1N)Oc2ccc(cc2C#N)Cl**	0
**c1ccc2c(c1)C[C@@H](NC2)c3nncn3C4CCCCC4**	0
**C=CCn1c(nnc1SCC(=O)NC2(CCCCCC2)C#N)[C@@H]3CCS(=O)(=O)C3**	0
**CC(C)(C)CC(=O)N1CCC2(CC1)N([C@H](CO2)C(=O)NCc3ccc[nH+]c3)C(=O)c4ccccc4**	0
**Cc1ccc(cc1)n2c3c(cn2)C(=O)C[C@H](C3)c4ccccc4O**	0
**Cc1c(c(n[nH]1)C)[N-]S(=O)(=O)c2cccc3c2cccn3**	0
**c1cc(nc2c1sc(n2)N)Br**	0
**CC(C)c1cc(c(c(=O)n1c2ccccc2)/C(=N/O)/C)[O-]**	0
**O=c1[nH]nc2c3c(cccc13)NC1=C2CCN(Cc2ccccc2)C1**	1
**O=C(c1cc(Cc2n[nH]c(=O)c3ccccc23)ccc1F)N1CCN(C(=O)C2CC2)CC1**	1
**CC[N+](=c1ccc-2cc(c(oc2c1)O)c3nnc(s3)Nc4cc(ccc4F)[N+](=O)[O-])CC**	0
**c1ccc(cc1)Cn2c3c(c(n2)C(=O)NCc4nccs4)CN(CC3)C(=O)c5cscn5**	0
**CC(C)(O)c1ccc(-c2nc3ccc(Cl)cc3c(=O)[nH]2)cc1**	1
**CC\1=NC(=[NH+][C@@H](/C1=C(/[O-])\OC)c2cn(nc2c3cccs3)c4ccccc4)O**	0
**Cc1cnc(cn1)c2nnc(o2)C3CCC(CC3)CNC(=O)c4ccc(cc4)N(C)C**	0
**CC(C)Oc1ccc(cc1NC(=O)/C=C\c2cccc3c2nccc3)S(=O)(=O)N4CCOCC4**	0
**C[C@@H](C(=O)NCCCn1cc[nH+]c1)N2CC[C@@]3(CCC2=O)CC(=O)c4ccccc4O3**	0
**CC[C@@]1(C[C@H](CCO1)n2c(c(cn2)C)N)C**	0
**c1ccc(cc1)[N-]S(=O)(=O)c2ccc3c(c2)NCCC3**	0
**O=C1NN=C2CCCc3c2c2c1cccc2n3CCN1CCCCC1**	1
**c1cc(oc1)Cn2c3c(c(=O)n(c(n3)SCC(=O)NC[C@H]4CCCO4)c5ccc(cc5)F)sc2=S**	0
**C[C@@H]1CC[C@H](C[C@@H]1C)NC(=O)N(C)C[C@@H](C)C(=O)[O-]**	0
**Cc1ccc(c(c1)NS(=O)(=O)c2ccc3c(c2)[C@@H]4C=CC[C@@H]4[C@@H](N3)c5ccccc5[N+](=O)[O-])OC**	0
**COc1ccc(cc1N(CC(=O)Nc2c(cccc2F)F)S(=O)(=O)c3ccc(c(c3)OC)OC)Cl**	0
**[H]/N=C\1/C(=C\c2ccc(c(c2)Br)N(C)C)/C(=O)N=C3N1N=C(S3)S(=O)(=O)CC(C)C**	0
**c1cc(c(cc1OCC2CCOCC2)Cl)[N+](=O)[O-]**	0
**CC[C@@H]1CC[C@H](C1)c2nc(on2)c3c(cc(s3)N)C**	0
**c1cc(cc(c1)S(=O)(=O)N2CCN(CC2)S(=O)(=O)/C=C/c3ccc(cc3)Cl)F**	0
**C[C@@H]1CC(=C[C@H]([C@@H]1[C@@H]2OCCC(O2)(C)C)C)C**	0

**Cc1cc(sc1C)C(=O)Nc2nc3c(s2)CNCC3**	0
**Cc1cc(cnc1N2CCc3ccccc3C2)/C=N/O**	0
**Cn1c2c(c(=O)n(c1=O)C)n(c(n2)N(C)Cc3ccccc3)C[C@H](CO)O**	0
**c1cc2c(cc1[C@H]([C@@H]3CCCCO3)Cl)CCCC(=O)N2**	0
**C[C@H]1CC2=C([C@@H](/C(=C(\[O-])/OC3CCCCC3)/C(=N2)C)c4ccc(cc4)N(C)C)C(=O)[C@@H]1C(=O)OC**	0
**Cn1c(=O)cc(n(c1=O)C)Oc2ccccc2/[NH+]=C/c3cc(c(c(c3)Br)O)Br**	0
**CN1[C@@H]([C@@H](c2cc(c(cc2C1=O)OC)OC)C(=O)Nc3ccc(cc3OC)OC)c4ccc(cc4)SC**	0
**Cc1c2c(n(n1)c3ccccc3)Oc4c(c5nc(nn5cn4)CSc6nc7ccccc7s6)[C@@H]2c8ccccc8**	0
**CC(=O)N(c1ccccc1)c2nc(cs2)/C=C/C(=O)N[C@H](c3ccccc3F)c4[nH+]ccn4C**	0
**Cc1cc2nc(cc(=O)n2o1)CSc3ccccc3[N-]S(=O)(=O)c4ccc(c(c4)F)F**	0
**c1cc(ccc1/N=C/2\NC(=O)/C(=C/c3ccc(o3)S[C@@H]4N[C@@H]5C=CC=CC5=N4)/S2)F**	0
**CC(C)N1CCN(CC1)C(=O)Nc2c(ccs2)C(=O)[O-]**	0
**c1ccc(cc1)C(=O)N2CCCN(CC2)c3ccc(c[nH+]3)NC(=O)C4CCCCC4**	0
**N#Cc1ccc2c(c1)[nH]c(=O)n2C12CC(c3nnc(-c4ccncn4)n3-c3ccccc3Cl)(C1)C2**	1
**CCCc1c(c(n(n1)C)NCC2CC(C2)O)N**	0
**C1C[C@@H](COC1)Cn2c(=O)[nH]nc2[S-]**	0
**Cc1c2cc(cnc2n(n1)C)N[C@H]3CC[C@@H](C[C@@H]3C)C**	0
**[H]/N=C\1/C(=C/c2ccc(c(c2)Br)OCc3ccccc3[N+](=O)[O-])/C(=O)N=C4N1N=CS4**	0
**CCc1c(nc(nc1Cl)C2CCCC2)Cl**	0
**Cc1cccn2c1nc(c2C)C(=O)C3=C(C(=O)N([C@H]3c4ccccc4F)CCCn5cc[nH+]c5)[O-]**	0
**CC[C@H]1C(=O)N[C@H](C(=O)N1c2cc(cc(c2)Cl)Cl)C**	0
**CC(C)n1cncc1Cn2ccc3c2ccc(c3)C(=O)[O-]**	0
**O=C(c1ccc(F)cc1)C1CCN(CC(=O)N(Cc2nc3c(c(=O)[nH]2)COCC3)Cc2cccs2)CC1**	1
**CCCCn1c(c(c(=O)[nH]c1=O)N(C)C(=O)c2c(nc(s2)c3cccc(c3)Cl)C)N**	0
**COc1cc(cc(c1OC)OC)[C@H]2C(=C(NC3=C2C(=O)CCC3)SCC(=O)Nc4cc(cc(c4)Cl)Cl)C#N**	0
**Cc1nc(cs1)Cc2nnc(o2)SCc3nnc(o3)c4cc5c(s4)CC[C@@H](C5)C(C)(C)C**	0
**Cc1ccc(cc1)[C@@H]2[C@H]3COc4ccccc4[C@@H]3Nc5n2ncn5**	0
**[H]/N=C\1/C(=C/c2cc(n(c2C)c3ccc(c(c3)[N+](=O)[O-])Cl)C)/C(=O)N=C4N1N=C(S4)CCCCC**	0
**C[NH+](C)[C@H](CNC(=O)Nc1cccc(c1)S(=O)(=O)N(C)C)c2cccs2**	0
**CCc1[nH]c(c([nH+]1)C)CNc2cc(cc(c2)S(=O)(=O)N(C)C)C(=O)N(C)[C@H](C)c3ccccn3**	0
**Cc1c2c(nc(nc2N3CCCN(CC3)C(=O)C4CCCC4)Cc5ccccc5)n(n1)c6ccccc6**	0
**CC1(C)OC(=O)N([C@H]2CC[C@H](NC(=O)c3cccc4cccnc34)CC2)[C@@H]1c1ccccc1**	1
**Cc1ccc(cc1N(CC(=O)N2CCN(CC2)C(c3ccccc3)c4ccccc4)S(=O)(=**	0

O)C)[N+](=O)[O-]	
CCN(CC)S(=O)(=O)c1ccc(cc1)C(=O)Nc2c(c3c(s2)CN(CC3)C(C)C)c4nc5ccccc5s4	0
Cc1cc2c(nc1)nc([n-]2)[C@H]3c4ccsc4CC[NH2+]3	0
C[C@H](c1nc2ccccc2s1)N3CCN(CC3)c4ccc(cc4[N+](=O)[O-])S(=O)(=O)NC(C)C	0
CCn1c2ccccc2n(c(=O)c1=O)C/C(=N/CCN3CCN(CC3)c4cccc(c4)Cl)/O	0
CCOc1ccccc1NC(=O)N2CCN(CC2)Cc3nnnn3c4ccc(c(c4)F)F	0
C[C@@H]1CC[C@@H]([C@@H](C1)Nc2ccc(nc2)n3cncn3)C	0
Cc1ccc2c(c1)c3c([nH]2)c(ncn3)N4CCSCC4	0
COc1ccc(cc1O)[C@H]2C[C@@H](c3cc(ccc3O2)Br)O	0
CC(C)CC(=O)Nc1nnc2n1CCS2	1
C=CCOc1ccc(cc1)C(=C2[C@H](N(C(=O)C2=O)CCCn3cc[nH+]c3)c4cccc(c4)Cl)O	0
Cc1c(nnc(c1C#N)N[C@@H]2CCC[C@H](C2)C)C	0
CC(C(=O)O/N=C/1\CCCCCN1)(Cl)Cl	0
CC(=O)Nc1cccc(c1)NC(=O)C2CCN(CC2)c3nc4ccc(cc4s3)F	0
CCCNC(=O)[C@H]1CCCN(C1)S(=O)(=O)c2cc(cs2)c3nc(on3)C4CCC4	0
CCOc1cc(ccc1OCCC(C)C)[C@@H]2C(=C(C(=O)N2CCC[NH+](C)C)O)C(=O)c3ccc4c(c3)C[C@H](O4)C	0
C[C@H](c1ccccc1)N(Cc1nc2c(c(=O)[nH]1)COCC2)S(=O)(=O)Cc1ccccc1	1
Cn1ccc2c(=O)[nH]c(N3CCC4(CC3)C(=O)N(CCO)c3cc(F)cc(F)c34)nc21	1
Cc1ccc(cc1)C(=O)Oc2ccc(cc2OC)[C@H]3Nc4c(c5c(s4)C[NH+](CC5)C(C)C)C(=N3)O	0
CC1(C[C@H](CCO1)Nc2ccc3c(c2)OCO3)C	0
Cc1cccc(-c2nnc([C@H]3C[C@H](NC(=O)c4cccc5nccnc45)C3)n2-c2ccccc2F)n1	1
CCN(CC)C(=O)c1cc(nc2c1c(=O)n(c(=O)n2C)CC(=O)Nc3ccc(c(c3)C)C)C	0
c1cc(cnc1)CN2[C@@H](c3c(n[nH]c3C2=O)c4ccc(cc4)Cl)c5ccc(cc5)[N+](=O)[O-]	0
COc1ccc2c(c1)C(CC(=O)NCCNC(=O)c1ccccc1N1CCC(=O)NC1=O)CC2=O	1
CCOc1ccc(-c2nnc([C@H]3C[C@H](NC(=O)c4ccccn4)C3)n2-c2ccc(C)s2)nc1	1
Cc1ccc(c(c1)C)NC(=O)c2cc(cc3c2[nH]cn3)F	0
C[C@H]1CCN([C@@H]2[C@@H]1CCCC2)C(=O)c3nc([n-]n3)C(C)C	0
CCn1cc(c(n1)C)S(=O)(=O)N2CCN(CC2)C(=O)CCS(=O)(=O)c3ccc(cc3)C	0
Cc1c2c(cc(nc2n(n1)C)c3ccco3)C(=O)Nc4cc(ccc4N5CCOCC5)Cl	0
C[C@]1(CC[C@@H]2[C@@]1(C=C2Br)C)C(=O)[O-]	0
c1cc(c(cc1CC(=O)Nc2cc[nH]n2)Br)F	0
Cc1ccc(cc1)n2c3c(c(c(c([nH+]3)C)CCC(=O)NCCN4CC[NH+](CC4)C)C)c(n2)C	0
Cc1[nH+]ccn1c2ccc(cc2F)CNC(=O)/C=C/c3cn(nc3c4ccc[nH+]c4)c5ccccc5	0

**Cc1ccc(cc1)[C@H](c2ccccc2)NC(=O)c3cccc(c3)S(=O)(=O)N4CC[NH+](CC4)C**	0
**C[C@@H]1CCC[C@@](C1)(C#N)C2(CCC(CC2)C(C)(C)C)O**	0
**Cc1ccc(o1)c2nnn(n2)CC(=O)N(c3ccc4c(c3)OCCO4)[C@H](c5ccc(cc5)F)C(=O)NC6CCCC6**	0
**CN1CCCc2c1nc(N1CCC3(CC1)C(=O)N(CCO)c1cc(F)cc(F)c13)[nH]c2=O**	1
**CCc1nnc(s1)NC(=O)[C@H](C)Sc2nnc(n2CC)CNC(=O)c3ccc4c(c3)OCO4**	0
**O=c1[nH]c(SCCN2CCN(c3ccccn3)CC2)nc2ccccc12**	1
**Cc1cc(c(c2c1nccc2)NC(=O)N(C)Cc3cnccn3)Cl**	0
**O=c1[nH]c(CN(Cc2cccs2)S(=O)(=O)Cc2ccccc2)nc2c1COCC2**	1
**C[C@H]1[C@H]2CC[C@]3([C@@H]2CC(=CC3)C)[C@H]1OC(=O)C**	0
**CC(C)n1c2cc(ccc2nc1c3cccc(c3)N)F**	0
**Cc1ccc2c(c1)c3c([nH]2)CCN(C3)C(=O)C[C@]4(CC(=O)N(C4=O)Cc5ccc[nH+]c5)c6ccccc6**	0
**c1ccc(c(c1)C(=O)Nc2cccc(c2)S(=O)(=O)N3CCOCC3)OCc4cn5ccccc5n4**	0
**CC(C)(C)c1[nH]nc(n1)C(=O)N2CCN(CC2)C(C)(C)C**	0
**c1c(cc(nc1Cl)Cl)N=C=O**	0
**CN(c1ccc(c(c1)Cl)C(=O)Nc2ccccc2C(=O)N3CCOCC3)S(=O)(=O)C**	0
**CC(=O)Nc1ccc(cc1)/C=C\2/C(=O)N(C(=O)S2)CCN3C(=O)/C(=C\c4ccc(cc4)N(C)C)/SC3=O**	0
**CCOc1cc(ccc1OCc2ccccc2)[C@H]3C(=C(C(=O)N3CC[NH+](C)C)O)C(=O)c4ccc(o4)C**	0
**C[C@]12CCCN([C@@H]1CCC[C@@H]2O)C(=O)OC(C)(C)C**	0
**Cc1cnc(cn1)CNC(=O)c2cc(n3c2cccc3)c4cn(c(n4)C)Cc5cccnc5**	0
**CCOc1cc(nc(n1)N2CCNCC2)C(F)(F)F**	0
**CCc1c2c(nc(nc2on1)CCC(=O)NCCN3CC[NH+](CC3)CC)N4CCCCC4**	0
**Cc1ccc2c(c1)C3=Nc4[nH+]cnn4[C@@H]([C@@H]3[C@@H](O2)c5cccs5)c6cccnc6**	0
**Cc1cc(c(cc1C)S(=O)(=O)Nc2cc(cnc2N3CCC[C@H](C3)C[NH+]4CCCC4)C(=O)[O-])C**	0
**COc1ccc(cc1)c2c(nnc(n2)SCC(=O)NCCc3ccc(cc3)S(=O)(=O)N)c4ccc(cc4)OC**	0
**c1cc(c(cc1n2cnnn2)N[C@@H]3C[C@@H]4[C@@H]3C=CC4)Cl**	0
**c1cc(ccc1C#N)NC(=O)[C@@H]2CSCN2S(=O)(=O)c3ccc(cc3)C(=O)NCC(F)(F)F**	0
**CC1(CN(CCCN1)[C@H]2CCOC2)C**	0
**Cc1ccn(n1)CC(=O)N2CCOc3c(cc(cc3O)c4cccc(c4)Cl)C2**	0
**CCN(CC)c1ccc(cc1)C[NH+]2CCc3nnc(n3CC2)[C@H](C)NC(=O)COc4ccccc4**	0
**CC[C@@H]/1CC[C@@H]2C[C@@H](C[C@@](O2)([C@@H]3CSC(=O)N3)O)OC(=O)/C=C(/CC/C=C\C=C1)\CC**	0
**CC[C@]12C[C@H](n3c4ccccc4c5c3[C@H]1N(CC5)CC=C2)O**	0
**CCS(=O)(=O)c1ccc(nn1)c2ccc(cc2)NS(=O)(=O)c3cc(ccc3OC)Br**	0
**C[C@]1(CCCS1)CNc2ccnc(n2)NN**	0
**C[C@@H]1Cc2cc(ccc2N1C(=O)C)S(=O)(=O)N3CCC[C@@H]3C(=O)Nc4cccc[nH+]4**	0

c1cc(c(cc1C#N)NC(=O)N2CCC[C@@H](C2)O)OC(F)F	0
Cc1cc(c(cc1Br)Br)OCC(=O)NN/C(=C\C(=O)Nc2ccc3c(c2)OCCO3)/C	0
Cc1c2cc(cnc2n(n1)C)CN[C@@H]3CCC[C@@H](C3)O	0
CCOCCOC(=O)C1=C(N=C2C[C@H](CC(=O)C2[C@H]1c3cc(c(cc3OC)OC)OC)c4cccs4)C	0
COc1ccc(-n2c(SCCCN3C(=O)c4cccc5cccc(c45)C3=O)nnc2-c2ccncc2)cc1	1
CCc1nnc(s1)NC(=O)c2ccccc2NC(=O)c3c(onc3c4ccccc4)C	0
c1ccc(c(c1)N2CC[NH+](CC2)Cc3c(ccc4c3O/C(=C\c5cccc(c5)[N+](=O)[O-])/C4=O)[O-])F	0
CCCNc1c(nsn1)c2ccc3c(c2)OCCO3	0
Cc1ccc(n1NC(=O)c2ccc(cc2Br)F)C	0
c1cc(nc(c1)Nc2cc(ccc2C#N)Cl)C(=O)[O-]	0
Cc1ccc2c(c1)[nH]c(n2)CN3CCC(CC3)C(=O)Nc4nccs4	0
CC1=C2C[C@@H]3[C@](CCC[C@@]3(C)O)(C[C@@]2(OC1=O)O)C	0
[H]/N=C/1\C(=C/c2cn(c3c2cccc3)CCOc4ccccc4)\C(=O)N=C5N1OC(=C5)C	0
C[C@H]1CC[C@@H]([C@@H]1C)Nc2nc3ccc(cn3n2)Cl	0
C[C@H]1c2ccsc2CCN1C(=O)Nc3cc(ccc3OC)C#N	0
c1ccc2c(c1)c(c3c(n2)/C(=C\c4ccc5c(c4)OCO5)/CC3)C(=O)OCC(=O)N[C@H]6CCS(=O)(=O)C6	0
CSc1ccc(cc1)N2C[C@@H](CC2=O)C(=O)Nc3cccc(c3)OCCn4ccnc4	0
Cc1cc(no1)NC(=O)[C@H](C)OC(=O)CCNS(=O)(=O)c2c(cccc2Cl)Cl	0
CC[NH+](CC)CCN1[C@@H](C(=C(C1=O)O)C(=O)c2c(nc3n2cccc3)C)c4cccc(c4)Oc5ccccc5	0
COc1ccc(cc1NC(=O)c2cccc(c2)F)NC(=O)CCCn3cncn3	0
Cc1ccc(nn1)N[C@H]2CC3(CCN(CC3)C(=O)c4ccc5ccccc5n4)OC2	0
C[C@@H]1Cc2ccccc2N(C1)C(=O)c3c(cn[nH]3)Br	0
Cc1ccsc1[C@@H]2CNC[C@]3(O2)CCC[C@@H](C3)C	0
Cn1c2cc(c(cc2oc1=O)S(=O)(=O)Nc3ccc4c(c3)CCCC4)Cl	0
CCS(=O)(=O)[N-]c1cccc(c1)C2=NN([C@@H](C2)c3ccccc3F)S(=O)(=O)c4ccccc4	0
CC1CCC(CC1)[NH+]2CCN(CC2)C(=O)NC[C@@H]3C[NH+]4CCCC[C@@H]4CO3	0
C[C@H](c1ccc2c(c1)OCCO2)NC(=O)CN3c4cc(nn4CCC3=O)c5cn(c6c5cccc6)C	0
C1CC=C[C@@H](C1)NC(=O)[C@@H]2CC=CC[C@@H]2C(=O)N[C@@H]3CCCC=C3	0
Cc1c2c(n(n1)C)n(c([nH+]2)N)C[C@H]3Cc4c3cccc4	0
Cc1cc(cn(c1=O)CC(=O)Nc2ccc3c(c2)OCO3)I	0
CCO[C@H]1CCCN(C1)S(=O)(=O)c2c(cc3c(c2Cl)oc(=O)[nH]3)Br	0
CC(C)(O)c1ccc(-c2nc3c(F)cc(F)cc3c(=O)[nH]2)cc1	1
C[C@@]12CCC[C@]([C@@H]1CC[C@@]34[C@@H]2CC[C@@H](C3)[C@@H](C4)CO)(C)C(=O)[O-]	0
CC1(CCC[C@@]1(C#N)NC)C	0
Cc1csc(n1)NC(=O)c2cc(nc3c2cc(cc3)S(=O)(=O)Nc4ccc(cc4)F)O	0

CC(C)NC(=O)c1cc(c(c(c1Cl)S(=O)(=O)N)Cl)F	0
C1CC2(CCC1NC(=O)C3C4CC5CC(C4)CC3C5)OCCO2	0
C[C@H]1CCC[C@@H](C1)N(C)C(=O)c2c(ccc(n2)N)Cl	0
CN1c2ncccc2C(=O)NC1c1ccc(NC(=O)C2CCOCC2)cc1	1
c1cc(cc(c1)O)[C@H]2C(=C(Nc3n2ncn3)C(F)(F)F)C(=O)c4ccc(s4)Cl	0
Cc1[nH+]ccn1CC2(CCN(CC2)C(=O)[C@@H]3CC(=O)N(C3)c4ccccc4C(C)C)O	0
COc1ccc(C(=O)C2CCN(CC(=O)NCc3nc4c(c(=O)[nH]3)COCC4)CC2)cc1OC	1
Cc1cccc2c(O)nc(-c3ccc(N)cc3)nc12	1
CC1=C([C@]2(c3cc(ccc3NC2=O)Br)C(=C(O1)N)C(=O)OCC=C)C(=O)OC(C)C	0
Cc1c(c(n(n1)C)C)C[C@@H](C)NC(=O)Cn2cccc(c2=O)C(=O)NC3CCCCC3	0
Cc1cc(=O)[nH]c2cc(-c3ccccc3F)ccc12	1
Cc1c2c(on1)NC3=C([C@@H]2c4cccc(c4)OC)C(=O)C[C@@H](C3)c5ccc(cc5)O	0
C[C@]12CC[C@@H]3C(=CC(=O)[C@]4([C@@]3(C[C@H]([C@H](C4)O)O)C)O)[C@@]1(CC[C@@H]2[C@](C)([C@@H](CCC(C)(C)O)O)O)O	0
Cc1ccc(cc1)C(=O)N2[C@@H](COC23CCN(CC3)C(=O)c4ccco4)C(=O)NC[C@@H]5CCCO5	0
Cc1ccc(cc1Cl)n2c3c(c(n2)C)[C@@H](CC(=O)N3)C(=O)c4cc(sc4C)C	0
CCc1cc(=O)[nH]c(CN2CCC(N3CCC(C(=O)c4ccc(OC)cc4)CC3)C2=O)n1	1
Cn1cccc1/C=N/n2c(nnc2[S-])c3cccs3	0
Cc1ccc(o1)CN([C@H](c2ccc(cc2)O)C(=O)NC3CCCCC3)C(=O)c4ccccc4O	0
CC(=O)N(CCOC)CC(=O)Nc1ccc(cc1)N2CCN(CC2)C(=O)c3ccccc3F	0
COc1ccc(-n2c(SCc3nc(-c4ccc(C)cc4)no3)nnc2-c2ccncc2)cc1	1
Cc1c(n(c(c1C#N)NC(=O)COC(=O)c2ccc(cc2)S(=O)(=O)C)c3ccccc3)C	0
CN1CCc2c(c(c3c(c2S(=O)(=O)N4CCC(CC4)(c5ccc(cc5)Cl)O)OCO3)OC)C1	0
Cc1ccnc2c1[nH+]c(n2c3ccc(cc3)Br)N	0
c1ccc(cc1)c2c(c[nH]n2)C(=O)Nc3cccc(c3)N4CCCCS4(=O)=O	0
Cc1c(nnn1c2cccc3c2ccc[nH+]3)C(=O)N[C@@H](C)c4cccc(c4)N5CCCC5=O	0
c1ccc2c(c1)cc(o2)CN3Cc4c([nH]c[nH+]4)C[C@H]3C(=O)NCc5ccc(cc5)C#N	0
CCn1c2ccccc2c3c1ccc(c3)[C@@H]4[C@@H]5CC=C[C@H]5c6c(ccc(c6N4)O)[N+](=O)[O-]	0
C[NH+]1CCN(CC1)CCCN2c3ccccc3Sc4c2cc(cc4)S(=O)(=O)N(C)C	0
CC(C)[C@H]1CC[C@@H]2[C@@H](C1)S(=O)(=O)CCCN2	0
Cc1cc(ccc1S(=O)(=O)NCc2cc[nH+]cc2)N3C(=O)C(CS3(=O)=O)(C)C	0
O=C(N[C@H]1C[C@H](c2nnc(-c3ccccn3)n2-c2ccccc2F)C1)c1cccc2nccnc12	1

C[C@H]1C[C@@H](CN(C1)C(=O)C[NH+](C)[C@@H](C)c2ccc(cc2)S(=O)(=O)N)C	0
c1ccc2=[NH+]C(=O)C(=c2c1)N[N-]C3=NC(=O)[C@H](S3)CC(=O)c4ccc(cc4)Cl	0
Cc1c2c(n(n1)c3ccccc3)OC(=C([C@@H]2c4ccc(c(c4)Cn5c(c(c(n5)C)[N+](=O)[O-])C)OC)C#N)[NH3+]	0
Cc1c(c(n[nH]1)C)S(=O)(=O)N2CCCC[C@H]2c3[nH+]c(c4c(n3)N(CCC4)CC5CC5)C	0
COc1cccc(c1)/C=C\2/C(=O)Nc3cc(ccc3S2)C(=O)NCC(OC)OC	0
c1ccc(c(c1)C#N)N[C@@H]2CCCc3c2nccc3	0
CN(C)[C@@H](CNC(=O)Nc1ccc(cc1C(F)(F)F)n2cncn2)c3ccsc3	0
C[C@H](Cc1cccs1)NC(=O)c2ccc(cc2[O-])Cl	0
c1cc(ncc1F)c2nc(on2)[C@@H]3CCCC[C@H]3C[NH3+]	0
c1c(cc(c(c1N)Cl)Cl)C(=O)N2CCO[C@H]3[C@H]2CCCC3	0
C[C@@H](c1ccc(cc1)n2cncn2)NC(=O)c3ccc(cc3)NC(=O)CSc4nccn4C	0
COc1ccc(cc1OC)[C@H]2[C@H]3[C@H](n4c(ncn4)N=C3c5cc(ccc5O2)Cl)c6cccs6	0
C[NH+](C[C@@H]1CCCO1)C2CCN(CC2)S(=O)(=O)c3ccc(s3)c4ccc(nn4)[O-]	0
CC1=NN([C@@H](C1)c2ccco2)c3ccc(cc3)S(=O)(=O)N	0
C[C@H]1C(=C2C(=NC(=NC2=O)CS[C@H](C)C(=O)O[C@@H](C)C(=O)N)S1)C	0
c1ccnc(c1)C[NH+]2Cc3c([nH]c[nH+]3)C[C@H]2c4nc(no4)c5ccc(cc5)C(F)(F)F	0
Cc1ccc(C2NC(=O)c3ccccc3N2)cc1	1
c1ccc(cc1)S(=O)(=O)Nc2ccc(=O)n3c2[C@@H]4C[C@H](C3)CN(C4)S(=O)(=O)c5ccccc5	0
CCc1ccc(cc1)c2[nH]c(=S)n(n2)C[NH+]3CCN(CC3)[C@H]4CCS(=O)(=O)C4	0
CN(c1ccc(c(c1)C(=O)[O-])N)C2CCCC2	0
Cc1c(sc2c1c(ncn2)N[C@H]3CC[C@@H]([C@@H](C3)C)C)C	0
CCOC(=O)c1csc(n1)NC(=O)[C@H]2CCCN(C2)S(=O)(=O)c3ccccc3	0
c1cc(c(c(c1N)N[C@@H]2CCS(=O)(=O)C2)F)F	0
CS(=O)(=O)c1ccc(-c2nc3c(c(=O)[nH]2)COCC3)cc1	1
c1ccnc(c1)Cn2cnc3c2nc(nc3N)Cl	0
C[C@@H](c1ccccc1)NC(=O)[C@H](C)NC(=O)[C@H]2CC(=O)N(C2)c3ccc(cc3)OC	0
CC(C)(C)NS(=O)(=O)c1ccc(cc1)c2cc(cc(c2)F)O	0
CCCn1c2c(c(n1)C(=O)N3CCOCC3)C[C@@H](CC2)[NH2+]CC4(CCCC4)N5CCOCC5	0
c1cc(c(cc1Cl)Cl)C(=O)Nc2ccc(nc2)Cl	0
C[C@@H]1CN(CC(O1)(C)C)C(=O)[C@@H]2CCC[C@@H]([C@@H]2C)[NH3+]	0
Cc1ccc2sc3nncn3c2c1	1
COCCOc1ccc(C(=O)C2CCN(CC(=O)NCc3nc4c(c(=O)[nH]3)COCC4)CC2)cc1	1
COc1ccccc1N1CCN(c2nc3c(c(=O)[nH]2)CC(F)(F)CC3)CC1	1
CC[C@H]1CCC[C@@H](C1)c2nc([nH]n2)Cl	0
Cc1cnc(cn1)CNC(=O)c2c(cn(n2)C)Cl	0
Cc1cc2c(cc1C)nc([nH]2)/C(=C\c3ccc4c(c3)n(c(=O)n4C)C)/C#N	0

**C[C@@H]1CCC[C@H]([C@@H]1C)NC(=O)c2cc(c[nH]2)S(=O)(=O)N3CCCCC3**	0
**Cc1ccc(cc1NC(=O)C2CCN(CC2)S(=O)(=O)c3ccc(cc3)F)[N+](=O)[O-]**	0
**CCOc1ccc(-c2nnc([C@H]3C[C@H](NC(=O)c4ccnc5cccnc45)C3)n2-c2cccnc2)nc1**	1
**Cc1csc(n1)Cc2nc(c3c4c(sc3n2)CCCC4)N**	0
**Cc1ccc(cc1[N+](=O)[O-])C(=O)NCC#Cc2ccc(cc2)N(C)C**	0
**Cc1cccn2c1nc(c2/C(=C/3\[C@H](N(C(=O)C3=O)CC[NH+]4CCOCC4)c5ccco5)/O)C**	0
**c1cn2c([nH+]1)CN(CC2)C(=O)N3CC[C@H](C3)CC(=O)[O-]**	0
**c1ccc(cc1)[C@H]2[C@H]3[C@@H](c4cc(ccc4OC3=O)Br)C(=C(S2)N)C#N**	0
**CC(=O)N1CCc2c(sc(c2C(=O)OC)NC(=O)c3ccc4c(c3)scn4)C1**	0
**COc1ccc(cc1OC)N2C[C@@H](CC2=O)NC(=O)N3CCn4cccc4[C@@H]3c5ccc(c(c5)F)F**	0
**c1ccc(c(c1)/C=N\n2c(nnc2[S-])C(F)(F)F)O**	0
**CCN(C(=O)N1CCC(C(=O)c2ccc3c(cnn3C)c2)CC1)c1ccc(C(=O)O)cc1**	1
**CC(C)(C)NC(=O)N(CCN1CCOCC1)CC(=O)N2[C@@H](CC(=N2)c3ccc(cc3)F)c4ccco4**	0
**O=C1OC2(CCN(c3nc4c(c(=O)[nH]3)CCCC4)CC2)c2ccccc21**	1
**C[C@@H](C(=O)Nc1cccc(c1)OC)NC(=O)N2CCN(CC2)C(=O)CCC3=c4ccccc4=[NH+]C3**	0
**c1ccc(c(c1)/C=N/Nc2nc(cs2)c3ccc(cc3)F)O**	0
**C[C@]12CC[C@@H]3c4ccc(cc4CC[C@H]3[C@@H]1CCC25OCCO5)O**	0
**CC1COC(OC1)(C)C(C)C**	0
**C[C@@]12CC[C@@H](C1)C([C@H]2Nc3ccc(nn3)C(=O)[O-])(C)C**	0
**Cc1nnc2n1ccnc2N[C@@H]3CCCCC[C@H]3C**	0
**C[C@H](c1cnn(c1)C)NC(=O)N2CCSC3(C2)CCCCC3**	0
**Cn1cc(cc1C(=O)NNS(=O)(=O)c2cccc(c2Cl)Cl)Br**	0
**CCOC(=O)c1nc(c2c(noc2n1)C)Nc3cccc(c3)F**	0
**Cc1c(c(on1)C)[C@@H](C)Nc2cc(ncn2)OC**	0
**CC[C@@H](CC#C)NC(=O)N1CCN(CC1)[C@H](C)C(=O)N2CCCC2**	0
**Cc1ccc(cc1)n2c(c(cn2)C(=O)[C@H]3CCN(C3)C(=O)c4cnc(cn4)C)N**	0
**Cc1c/c(=N\S(=O)(=O)CC#N)/c2ccccc2[nH]1**	0
**CC1(C(=O)N(C(=O)N1)CCCC(=O)N2CCCN(CC2)c3ccc(cn3)C(F)(F)F)C**	0
**Cc1c2cc(cnc2n(n1)C)N[C@H]3CCCSC3**	0
**[H]/N=C\1/[C@H]([C@H](C2=C(O1)C[C@@H](CC2=O)C)c3cccs3)C#N**	0
**CCc1nc(sn1)NCC2(CCC(CC2)(C)C)O**	0
**Cc1ccc(o1)C[NH2+][C@@H](C)c2cnc(nc2C)N3CCCCCCC3**	0
**CCCc1c(c(nc(n1)c2nc[n-]n2)NC)Br**	0
**Cc1ccc(c2c1[C@H]([C@H]([C@@H](S2(=O)=O)C)C)NC(C)C)C**	0
**O=C(Nc1ccc(F)cc1)c1ccc(NC(=O)N2CCN(C(=O)c3cc(Cc4n[nH]c(=O)c5ccccc45)ccc3F)CC2)cc1**	1

c1cc2c(cc1I)nc(n2C[C@H]3CCCOC3)N	0
c1cc(cc(c1)NC(=O)O[C@@H]2CO[C@H]3[C@@H]2OC[C@@H]3n4c(nnn4)SC5CCCC5)C(F)(F)F	0
O=c1[nH]c(-c2ccc(C(F)(F)F)cc2)nc2c1SCCC2	1
CCc1nnc(s1)NC(=O)c2ccc3c(c2)S(=O)(=O)c4ccccc4C3=O	0
Cc1cc(c(s1)C)[C@@H](C)Nc2c3nnc(n3ccn2)C	0
O=c1[nH]c2ccccc2n1[C@H]1C[C@H](c2nnc(-c3ccncn3)n2-c2ccccc2Cl)C1	1
C[C@@]1(C(=O)NC(=O)N1)CCc2ccc3c(c2)OCO3	0
CC(=O)Nc1ccc(cc1)S(=O)(=O)NCC(=O)N2CCC[C@@H]2c3nc4ccccc4s3	0
CN(C)C(=O)c1ccccc1NC(=O)N2CC3C[C@H]4C[C@@H](C3)CC2C4	0
CC[NH+](CC)CCN1[C@@H](C(=C(C1=O)O)C(=O)c2c(nc(s2)C)C)c3ccc(cc3)O	0
CCc1ccc(cc1)c2cc(c3c(nn(c3n2)[C@@H]4CCS(=O)(=O)C4)C)C(=O)NCc5c(cccc5F)F	0
Cc1c(c(=O)n(n1C)c2ccccc2)NS(=O)(=O)c3ccc(cc3)CN4C(=O)c5ccccc5C4=O	0
Cc1cn2c([nH+]1)CC[C@@H](C2)N[C@@H](C)c3nc4cc(ccc4n3C)Cl	0
C[C@@]1(CCN(C1)C(=O)N2CCC(CC2)C[NH+](C)C)C(=O)[O-]	0
C[C@H]1C[C@@]2(CC(=O)NC2)CC(C1)(C)C	0
Cc1c(sc2c1c(nc(n2)CN3CCSCC3)NN)C	0
Cc1cc(c(c(n1)NN=Cc2cc(c(c(c2)I)OCC#C)OC)C#N)COC	0
Cc1ccc(cc1C)C(=O)[C@@H](C)n2c(=O)n3c4cc(ccc4c(=O)n(c3n2)C(C)C)C(=O)NC(C)C	0
CCCOc1ccccc1[C@@H]2c3c(nn(c3N=C(CS2)O)C4CCCCC4)O	0
C[C@H]1CC[C@]([C@@H](C1)C)(C#N)OC	0
c1cc2c(cc1N[C@H]3CCCC[C@@H]3C#N)OCCO2	0
CCn1c(=O)c(nn(c1=O)c2ccc(cc2)F)c3nc(on3)C(=O)N[C@H](C)CCc4ccccc4	0
Cc1cc(=O)[nH]c2cc(-c3ccc(N)cc3Cl)ccc12	1
CC(=O)Nc1ccc2c(c1)sc(n2)NC(=O)C[C@H]3c4ccccc4C=CN3C(=O)C	0
CC[C@H](C)N1C(=O)/C(=C/c2c(nc3c(cccn3c2=O)C)N4CCN(CC4)CCO)/SC1=S	0
c1ccc(cc1)C[NH+]2CCN(CC2)C(=O)c3c4c(n[nH]3)CCN(C4)C(=O)Cc5cccc(c5)Cl	0
C[C@@H]1CCN([C@H](C1)C)C(=O)NC(=O)CC(C)(C)C(=O)O	0
O=C(CCCc1ccccc1)Nc1nnc2n1CCS2	1
C[C@H](/C(=N/c1nccs1)/O)Sc2nnc(n2C)C/[NH+]=C(/c3ccccc3F)\O	0
C[C@H]1CCC[C@@]([C@@H]1C)(Cc2[nH+]cnn2C)O	0
C[C@H](C[NH3+])c1nc(on1)c2ccc(cc2Cl)Br	0
c1cc(cc(c1)N2CC[NH+](CC2)Cc3nc(co3)C(=O)NCCN4CCOCC4)C(F)(F)F	0
Cc1cc(ccc1NC(=O)c2cccn2C)Br	0
COc1ccc2c(n1)[nH]c(n2)c3ccc4c(c3)CCN4	0
O=c1[nH]c(-c2ccc(Br)cc2)nc2ccccc12	1
C[C@@H]1CC[NH2+][C@@H]([C@@H](S1(=O)=O)C)C2CC2	0

**Cc1nc(no1)c2ccc(c(c2)S(=O)(=O)N3CCC[C@H](C3)C(=O)NCc4ccc(cc4)Cl)Cl**	0
**Cc1cccc(n1)N2CCC(CC2)NS(=O)(=O)c3ccccc3OC(F)(F)F**	0
**CC(C)c1nc2c(n1c3cnn(c3)C)CCNC2**	0
**C[C@@H]1CC[C@@H](N(C1)C(=O)c2cc(nc(c2)Cl)N)C**	0
**Cc1cc(cc(c1)S(=O)(=O)N[C@@H]2COc3c2cccc3)N**	0
**Cc1cc(c(c2c1C(=O)/C(=C/c3cc(c(cc3OC)OC)OC)/O2)CN4CC[NH+](CC4)C)[O-]**	0
**COc1cc(cc(c1O[C@H]2CCCC[C@H]2O)Br)C[NH3+]**	0
**c1cc(ccc1/C=C/C(=O)OC[C@@H]2[C@@H]([C@@H]([C@H]([C@@H](O2)OC(=O)c3cc(c(c(c3)[O-])O)O)O)O)O)O**	0
**C[C@@H](C(=O)Nc1cccc(c1)S(=O)(=O)N)Sc2ccc(cn2)S(=O)(=O)N3CCCC3**	0
**c1[nH+]c(nn1[C@@H]2CCO[C@]3(C2)CCSC3)N**	0
**Cc1c(c(on1)C=CN(C)C)S(=O)(=O)NCC[NH+]2CCN(CC2)c3ccccc3F**	0
**Cc1c(c(=O)n(n1C)c2ccccc2)N/C=C\C(=O)c3ccco3**	0
**Cn1c(nnc1SCC(=O)/[NH+]=C\2/NCCS2)CC(=O)Nc3ccccc3Cl**	0
**O=C(c1ccc(F)cc1)C1CCN(CC(=O)N(Cc2nc3c(c(=O)[nH]2)COCC3)Cc2ccccc2F)CC1**	1
**COc1cccc2c1nc(s2)NC(=O)c3ccc(cc3)S(=O)(=O)N(CCC#N)CCC#N**	0
**c1cc2c(cc1O)c(co2)C(=O)c3ccc(s3)Cl**	0
**C[C@@H](c1nccn1C)Nc2ccc3c(c2)OC(O3)(F)F**	0
**c1cscc1CNC(=O)N2CCC[C@H](C2)C[NH+]3CCOCC3**	0
**COc1cccc2c(=O)[nH]c(-c3ccc(F)cc3)nc12**	1
**COC(CNC(=O)c1ccc2c(c1)N(C(=O)c3ccccc3S2(=O)=O)Cc4ccc(cc4)Cl)OC**	0
**Cc1ccc(F)c2c1sc1nncn12**	1
**Cc1ccc(s1)c2cc([nH]n2)C(=O)N3CCc4c5ccccc5[nH]c4[C@H]3c6cccc[nH+]6**	0
**Cc1c(c(n(n1)C)C[C@]2(CCCOCC2)[NH3+])Cl**	0
**c1cc(cnc1)O[C@H]2C[C@@H](C23CCCCC3)Cl**	0
**C[C@@H]1CC[C@@H]([C@@H](C1)O)C(C)(C)[C@@H]2CCO[C@@]3(C2)CCOC3**	0
**C#CCOc1c(nnc(n1)Cl)Cl**	0
**Cc1cc(=O)[nH]c2cc(-c3cccc(F)c3)ccc12**	1
**C[C@H](C(=O)Nc1ccc2c(c1)OC(O2)(F)F)NC(=O)c3ccc(cc3)Br**	0
**O=C(CCc1ccccc1)N(Cc1nc2c(c(=O)[nH]1)COCC2)Cc1ccc(Cl)s1**	1
**Cc1c(sc(n1)N2[C@@H](C(=C(C2=O)O)C(=O)c3ccc4c(c3)C[C@@H](O4)C)c5ccc(cc5)C(=O)OC)C(=O)C**	0
**CN(Cc1ccc(s1)Cl)C(=O)Nc2ccc(cc2)[N+](=O)[O-]**	0
**CC1(Oc2ccc(cc2O1)NC(=O)c3ccc(cc3)n4ccnn4)C**	0
**C[NH+](C)CCN1C(=O)C[C@](C1=O)(CC(=O)N(C)C[C@H](c2ccccc2)O)c3ccccc3Cl**	0
**Cn1c2c(c(=O)n(c(=O)n2C)C)nc1SCCC(=O)Nc3ccc(cc3F)Cl**	0
**COc1ccc(-c2noc(CSc3nnc(C)n3-c3ccc(F)cc3)n2)cc1**	1
**O=c1cc(-c2ccc(O)c(O)c2)oc2cc(O)cc(O)c12**	1
**c1ccc(cc1)C2=NN[C@H](O2)C3=C[C@H](C=N3)S(=O)(=O)N4CCN(CC4)c5cccc(c5)Cl**	0
**c1ccc2c(c1)c3c([nH]2)[C@H](CCC3)c4[nH]nc(n4)C5CC5**	0
**CCN(CC)C(=O)[C@H]1CCCN(C1)c2ccc(cc2/C=N/n3cnnc3SCc4**	0

**ccccc4)[N+](=O)[O-]**	
**C[C@H](C(=O)Nc1ccc(cc1Cl)F)OC(=O)c2cc(ccc2N3CCCC3)S(=O)(=O)N(C)C**	0
**c1cc(cc(c1)S(=O)(=O)N2CCCCCC2)/C(=N/c3cccc4c3cc[nH+]c4)/O**	0
**c1cc(ccc1n2c3c(c(ncn3)N4CCN(CC4)C(=O)CCC5CCCCC5)nn2)Cl**	0
**CCOc1cc(cc(c1O)I)/C=C\2/C(=O)NC(=O)N(C2=O)C**	0
**Cc1ccc(cc1)n2c3c(c(n2)C(=O)N[C@@H]4CS(=O)(=O)C[C@@H]4[NH+]5CCCCC5)CCC3**	0
**CC(C)(O)c1ccc(-c2cn3c(F)ccc3c(=O)[nH]2)cc1**	1
**c1c(csc1Br)C(=O)NN2CCCCC2**	0
**c1ccn(c1)N[C@H]2CCCCC[C@@H]2C#N**	0
**CC(=O)C(=Cc1ccc(c(c1)[N+](=O)[O-])O)C(=O)C**	0
**c1cc2n(c(=O)c1)C[C@@H]3C[C@H]2CN(C3)c4ccc(cc4NC(=O)c5cccnc5)C(=O)N6CCN(CC6)c7ccc(cc7)[N+](=O)[O-]**	0
**C[C@H]1CCCCN1c2n[nH]c(=S)n2C3CCCC3**	0
**Cc1cc(c(cc1C)c2cnc(cn2)N)C**	0
**c1ccc(cc1)N2C[C@@H](CC2=O)C(=O)Nc3cccc(c3)c4ccc([nH+]n4)N5CCOCC5**	0
**Cc1ccc2c(c1)C(=Nc3cc(ccc3S2)C(=O)NC(C)C)C**	0
**c1ccc(-c2ncc3c(n2)CCSC3)cc1**	1
**O=C(Nc1nnc2n1CCS2)C1CC1**	1
**CC1=CC(=O)C=C2[C@]1([C@@H]3[C@@H](CC2)[C@@H]4CC[C@]([C@]4(C[C@@H]3O)C)(C(=O)COC(=O)C)O)C**	0
**Cc1ccc(cc1)S(=O)(=O)N(CC(=O)N/N=C(\C)/c2ccc(cc2)I)c3cccc(c3)C**	0
**CC(C)c1ccc(cc1)c2c(c([nH]c(=S)n2)[O-])C#N**	0
**COc1ccc(-c2noc(CSc3nnc(-c4ccncc4)n3-c3ccc(C)c(C)c3)n2)cc1**	1
**COc1ccc(-n2c(C)nnc2SCc2nc(-c3ccc(OC)c(OC)c3)no2)cc1**	1
**N#Cc1cccc2c1C1(CCN(c3nc4c(c(=O)[nH]3)CCCC4)CC1)CN2**	1
**Cc1cnn(c1)c2c(ccc(n2)C(F)(F)F)C(=[NH2+])N**	0
**CCOc1ccc(cc1)Nc2[n+](c(cs2)c3ccc(cc3)S(=O)(=O)N4CCCC4)Cc5ccco5**	0
**CCN(CC)S(=O)(=O)c1ccc(cc1)NC(=O)c2ccc(cc2)S(=O)(=O)N(C)Cc3ccccc3**	0
**CCC[NH+]1CCN(CC1)CCCNC(=O)c2cc3n(n2)C[C@@](N(C3=O)CCC)(C)C(=O)NC4CCCC4**	0
**CC(C)(C)C1CCN(CC1)c2cc(ncn2)NN**	0
**CCOc1ccc(-c2nnc([C@H]3C[C@H](NC(=O)c4cccc5nccnc45)C3)n2-c2ccccc2F)nc1**	1
**c1c(c(cc2c1OCCO2)N[C@H]3C[C@H]4CC[C@@H]3C4)N**	0
**CC(C)S(=O)(=O)N1CCc2c(sc(n2)NC(=O)c3cc(n[nH]3)c4ccc(cc4)F)C1**	0
**COc1ccc(cc1OC)[C@@H]2[C@H](S(=O)(=O)[C@@H]([C@@H]([NH2+]2)c3ccc(c(c3)OC)OC)C(=O)OC)C(=O)OC**	0
**CC(=O)N(C)c1ccc2c(c1)[C@H]3C=CC[C@H]3[C@H](N2)c4cccc(c4O)OC**	0
**C[C@H](CNC(=O)N1CCC[C@@H](CC1)C(C)C)C(=O)[O-]**	0
**C[NH+]1CCN(CC1)c2ccc(cc2)NC(=O)[C@@H]3CCCN(C3)C4=NS(=O)(=O)c5c4cccc5**	0

**Cc1cc2c(c3c(/c(=N\N)/o2)CCC3)c4c1c(c(o4)C)C**	0
**CCN(C(=O)N1CCC(C(=O)c2ccc3cnn(C)c3c2)CC1)c1ccc(C(=O)O)cc1**	1
**c1cc(c(cc1Br)N2CCC[C@@H]2CO)[N+](=O)[O-]**	0
**Cc1cc(cc2c1N[C@H]([C@H]3[C@H]2C=CC3)c4cccc(c4)OC)[N+](=O)[O-]**	0
**Cc1cc(cc(c1)F)N[C@@]2(CCCOCC2)C#N**	0
**CN(C)S(=O)(=O)c1cccc(c1)c2c(cn(n2)c3ccccc3)/C=C\4/C(=O)N(C(=S)S4)C[C@@H]5CCCO5**	0
**c1cc(cc(c1)S(=O)(=O)[N-]c2ccc3c(c2)N=[NH+]C3)C#N**	0
**c1c(c(cc(c1S(=O)(=O)N2CCOCC2)Cl)Cl)/C(=[NH+]/CCCN3CCCC3=O)/O**	0
**CCN(CC)S(=O)(=O)c1ccc(cc1)S(=O)(=O)[N-]c2cccc(c2)C#Cc3cccs3**	0
**C[NH+](C)c1ccc(cc1)[C@@H]2CC(=NN2C(=O)CSc3nnc(s3)N)c4ccccc4Cl**	0
**Cc1ccc(cc1)[C@@H]2CC(=NN2C(=O)CSc3nnc(n3C)CNC(=O)c4ccco4)c5cccs5**	0
**Cc1nc(cc(n1)Cl)O[C@H]2CCCC[C@@H]2C**	0
**C[C@@H](c1ccccc1)Nc2c3ccccc3c4nnnn4n2**	0
**c1ccc(cc1)OC[C@@H](C[NH+]2CCN(CC2)S(=O)(=O)c3ccc4c(c3)OCCCO4)O**	0
**CC(C)(C)C(=O)CN(Cc1nc2c(c(=O)[nH]1)COCC2)C(=O)CCc1ccccc1**	1
**COCCN1C(=O)/C(=C\c2cccn2c3ccc(cc3)O)/C(=O)N(C1=O)c4ccccc4**	0
**C[C@H]1C[C@@H]1C(=O)NC2(CCCCCC2)C#N**	0
**CC\1=NN(C(=O)/C1=C\c2ccc(c(c2)OC)OCC(=O)N)c3cccc(c3)C(F)(F)F**	0
**CC1(C[C@]2([C@@H](O2)C(=O)OC)C(O1)(C)C)C**	0
**CC(=O)N1Cc2ccccc2OCCC[C@H]3CN(CC[C@H]3CC(=O)NCc4cccc[nH+]4)C(=O)C1**	0
**c1cc(c(cc1S(=O)(=O)NCC(=O)N2CCNCC2)Cl)Cl**	0
**COc1ccc(cc1OC)[C@@H]2C(=C(c3ccc(cc3)F)O)C(=O)C(=O)N2c4cccc(c4)C(=O)[O-]**	0
**Cc1ccc(cc1)n2c(c(nn2)C(=O)Nc3cccc(c3)Cl)C**	0
**N#Cc1ccccc1Cn1c(=O)n(C[C@H]2CC[C@H](C(=O)Nc3ccncc3)CC2)c(=O)c2ncccc21**	1
**C[C@@H](c1ccccc1NS(=O)(=O)C)NC(=O)c2ccc(c(c2)S(=O)(=O)N(C)C)OC**	0
**C1CCCC(CC1)[C@@H]([C@H]2CCO[C@@]3(C2)CCOC3)O**	0
**O=C(N[C@H]1C[C@H](c2nnc(-c3ccccn3)n2-c2ccccc2F)C1)c1cc(F)cc2nccnc12**	1
**Cc1nc(cc2n1c(=O)[nH]n2)N(C)[C@@H](C)CC#N**	0
**c1ccc(cc1)C2CCC(CC2)(C3(CCCC3)C#N)O**	0
**C[C@]1\2CC[C@@H]3c4ccc(cc4CC[C@H]3[C@@H]1CC/C2=N\NC(=O)CC#N)O**	0
**Cc1ccc(cc1)S(=O)(=O)N(Cc2ccccc2)Cc3ccc(o3)/C=N\NC(=O)c4c(nc5n4ccc(c5)C)C**	0
**Cc1ccc(cc1C)NC(=O)Cn2cnc3c4ccccc4oc3c2=O**	0
**Cc1ccc(cc1)n2c(=O)c(c([nH]c2=O)[O-])[C@@H]3[C@@H]4C(=c5ccccc5=[NH+]4)CCN3C**	0

**Cc1c(c(n(n1)[C@H](C)C2CCCC2)N)Cl**	0
**c1c(cc(c2c1OCO2)Cl)[C@@H]([C@@H]3CCCCCN3)O**	0
**Cc1cccc(c1)n2c(=O)/c(=C/c3ccc(o3)[C@H]4C[C@H]4C)/sc2=C(C#N)C#N**	0
**CC1(CS(=O)(=O)N(C1=O)c2ccc(cc2)S(=O)(=O)NC3CCCCC3)C**	0
**Cc1ccc(c(c1)C(=O)Nc2cc3ccccc3nc2)O**	0
**CCOc1ccccc1N2C[C@H](CC2=O)C(=O)Nc3ccccc3N4CCCC4**	0
**O=C(Nc1cccc2cccnc12)c1ccc(C2C(=O)[C@@H]3[C@H](C2=O)[C@@H]2C=C[C@H]3C2)cc1**	1
**C[C@@H]1C[C@@H]2[C@@H]([C@@H](C(=O)O2)CN3CCCCC3)[C@@H]([C@]4([C@@H]1[C@@H](C[C@@H]4O)OC(=O)C)C)O**	0
**CC(=O)c1ccc(cc1)NC(=O)Cn2c3c(c4c(s3)COC(C4)(C)C)c(=O)n(c2=O)c5cccc(c5)Cl**	0
**c1ccn2c(c1)nc(c2Br)CSc3[nH]c4cc(ccc4n3)[N+](=O)[O-]**	0
**Cc1ccc2c(=O)[nH]c(-c3ccc(C(C)(C)O)cc3)nn12**	1
**C[C@@H](c1ccccc1)N(Cc1nc2c(c(=O)[nH]1)COCC2)S(=O)(=O)Cc1ccccc1**	1
**Cc1cccc(-c2nnc([C@H]3C[C@H](NC(=O)c4cc(F)cc5nccnc45)C3)n2-c2ccccc2F)n1**	1
**c1ccc(c(c1)CNC(=O)C[C@@H]2CCN3C[C@@H]2CCCOc4ccc(cc4CNCC3=O)N5CCOCC5)Cl**	0
**C[C@H]1CCC[C@@H](C12C(=O)N(C(=O)N2)CN3CCCCCCC3)C**	0
**c1ccc(cc1)C2=C([C@@H](C(=C(O2)N)C#N)c3ccco3)C#N**	0
**C[C@H]1CC[C@H]([C@@H](C1)C)n2c3c(nc2C(F)(F)F)CNCC3**	0
**O=C(CCc1ccccc1)N(Cc1nc2c(c(=O)[nH]1)COCC2)Cc1cccs1**	1
**Cc1csc(n1)NC(=O)c2c3c(on2)-c4ccsc4CC3**	0
**CCc1nnc(s1)NC(=O)[C@H](C)Sc2nnc(n2C)CCNC(=O)c3ccccc3F**	0
**Cc1ccc(c([nH+]1)C[NH+]2CCN(CC2)[C@H](C)C(=O)N3CCOCC3)[O-]**	0
**c1cc(ccc1[C@H](CNS(=O)(=O)c2ccc(cc2)N3C(=O)CCS3(=O)=O)[NH+]4CCCC4)F**	0
**COc1ccc(C(=O)Nc2ccc(C(=O)NCC3(c4ccc(OC)cc4)CCOCC3)c(Cl)c2)cc1**	1
**CN(Cc1ccc2c(c1)ccc[nH+]2)C(=O)c3ccc(=O)n(c3)CC(=O)NC4CCCCC4**	0
**c1cc(ccc1c2nc(on2)c3c(n(nn3)c4ccc(c(c4)Cl)C(F)(F)F)N)Cl**	0
**CC[C@H](CO)NC(=O)C(=O)Nc1c2c(nn1c3cccc(c3)Cl)CS(=O)(=O)C2**	0
**C[C@@]1(C(=O)N(C(=O)N1)CC(=O)N(C)Cc2ccc3c(c2)OCCO3)c4ccc(cc4Cl)Cl**	0
**CCc1nnc(s1)N2[C@@H](c3c(=O)c4cc(c(cc4oc3C2=O)C)Cl)c5cccc(c5)O**	0
**COc1cccc(C(=O)C2CCN(CC(=O)N(Cc3nc4c(c(=O)[nH]3)COCC4)CC(F)F)CC2)c1**	1
**Cc1ccc(cc1NC(=O)NCc2cnc(s2)C)S(=O)(=O)N(C)C(C)C**	0
**Cc1cc2ccccc2nc1N[C@@H](C)c3nncn3C**	0
**Cc1c(c2c(s1)ncn(c2=O)CC(=O)NC[C@@H]3COc4ccccc4O3)S(=**	0

O)(=O)N5C[C@H](C[C@@H](C5)C)C	
C[C@@H]1CCc2ccccc2N1c3ncc(cn3)N	0
CC1=C([C@@H](c2c(n(c(=O)n(c2=O)C)C)N1)c3cc(c(c(c3)OC)OC)OC)C(=O)OCC=C	0
c1cc(sc1c2cc3c(s2)c(=O)[nH]cn3)Br	0
Cc1ccc(cc1)N2C(=O)/C(=C/c3c(n(c4c3cccc4)Cc5ccc(cc5)Br)C)/C(=O)N=C2[O-]	0
Cc1ccc(c2c1[C@H](C[C@H]2Nc3cnn(c3)C)C)O	0
O=C(CCC1CCCC1)Nc1nnc2n1CCS2	1
C[C@@]12CCC[C@]([C@@H]1CC[C@@]34[C@@H]2CC[C@@H](C3)C(=O)C4)(C)C(=O)O	0
Cc1ccccc1C(=O)N2[C@@H](COC23CCN(CC3)C(=O)c4cccs4)C(=O)NCc5cccc(c5)C(F)(F)F	0
CCc1c(c(n(n1)c2ccc(cc2F)F)O)Br	0
CC1(CC[C@](C1)(c2cncnc2)O)C	0
Cc1ccc(cc1)N2CCN(C2c3ccc(o3)C)c4ccc(cc4)C	0
C[C@H]1C[C@H]2C[NH2+]C[C@@H]2N1S(=O)(=O)c3cc(cnc3)Br	0
Cc1ccc(c(c1)n2cc(c(n2)c3ccccc3)C[NH+]4CCN(CC4)C(=O)c5[nH]ncn5)C	0
Cc1c(sc(n1)C)C(=O)C2=C(C(=O)N([C@@H]2c3cc(ccc3OC)OC)CCC[NH+](C)C)[O-]	0
COc1ccc(C(=O)C2CCN(CC(=O)N(Cc3nc4c(c(=O)[nH]3)CCCCC4)CC3CC3)CC2)cc1	1
Cc1cccc(c1C)N2C[C@H](CC2=O)C(=O)Nc3nc4ccc(cc4s3)S(=O)(=O)C	0
C[C@@H]1c2cccc(c2O[C@@H]1C)C(=O)O[C@@H](C)C(=O)NCCc3ccc(cc3)S(=O)(=O)N	0
O=C(CN1CCC(C(=O)c2ccc(Cl)cc2)CC1)NCc1nc2c(c(=O)[nH]1)COCC2	1
C[C@H](C(=O)NC(=O)N)Sc1nc2c(c3c(s2)CCCC3)c(=O)n1c4ccccc4C(F)(F)F	0
Cn1c2ccc(cc2[nH+]c1c3cc(ccc3F)Cl)C(=O)[O-]	0
C[C@@H]1CCCCN1C(=O)c2cccc(c2)S(=O)(=O)NCc3ccc4c(c3)OCO4	0
CN(C)c1ccc(cc1)[C@H]2C3=C(N=N[C@@H]3NC4=C2C(=O)CCC4)O	0
Cc1cc(nc(n1)Cl)N[C@@H]2CCC[C@H](C2)C	0
Cc1cccn2c1nc(c2C)C(=O)C3=C(C(=O)N([C@@H]3c4cccc(c4)OCC=C)CCCn5cc[nH+]c5)O	0
COc1ccc(C(=O)C2CCN(CC(=O)NCc3nc4c(c(=O)[nH]3)COCC4)CC2)cc1	1
Cc1ccc2cc(c(=O)[nH]c2c1)[C@@H](c3nnnn3Cc4ccccc4)N5CC[NH+](CC5)Cc6ccccc6	0
CCc1ccc2c(c1)sc1nncn12	1
CCOC(=O)c1c(sc(c1O)C2=C3[C@@H](N=C2c4ccc(c(c4)F)OC)N(C(=O)N(C3=O)C)C)C	0
COc1cc(Cl)c(C#N)cc1C(=O)NCCN1CCC(C(=O)c2ccc(F)cc2)CC1	1
COc1ccc(C(=O)C2CCN(C3CCN(Cc4nc5c(cnn5C)c(=O)[nH]4)C3=O)CC2)cc1	1
Cc1ccc(c(c1)F)/N=C\2/N[C@@]3(CCC[C@H](C3)C)CS2	0

CCC[C@@H]1CCc2c(sc(n2)NC(=O)C3CCN(CC3)C(=O)N4CC CC4)C1	0
Cc1cccc(c1NC(=O)COC(=O)c2cc3c(nn(c3s2)c4ccccc4)C)[N+](=O)[O-]	0
Cc1cc(c(c(c1)C)/N=C\2/NC[C@H](CS2)C)C	0
CC(=O)N[C@H]1[C@H]([C@@H]2[C@@H](CO[C@@H](O2) c3ccco3)O[C@@H]1Oc4cccc5c4cccc5)OCC(=O)[O-]	0
CC1(Cc2c(c(nc3c2c4c(s3)c(ncn4)N(Cc5ccccc5)Cc6ccccc6)N7CC OCC7)CO1)C	0
CNc1c2c(nc(n1)[C@H]3CCCN3S(=O)(=O)Cc4cc(cc(c4)F)F)CC[NH+](C2)C	0
CCC(=O)N(Cc1ccc(o1)C)[C@H](c2ccc(c(c2)OC)O)C(=O)NCS(= O)(=O)c3ccc(cc3)C	0
Cc1c(sc([nH+]1)N)S(=O)(=O)[N-]c2cccc(c2F)F	0
Cc1cc(N2C[C@@H](C)N[C@@H](C)C2)ncc1-c1ccc(- c2nc3c(ccn3C)c(=O)[nH]2)cc1	1
CN1[C@H]([C@@H](c2cc(c(cc2C1=O)OC)OC)C(=O)Nc3ccc(cc 3F)F)c4ccc(c(c4)OC)OC	0
c1ccc2=[NH+]CC(=c2c1)CCNC(=O)[C@@H]3CC34CCN(CC4) C(=O)c5cccc(c5)n6cccn6	0
c1c2c(cc3c1OCO3)/C(=N/O)/C[C@@H]2/N=C(/C(F)(F)F)\[O-]	0
Cc1c(c(on1)C)CN2CC(C2)O	0
COCCN(Cc1nc2c(c(=O)[nH]1)COCC2)C(=O)CN1CCC(C(=O)c2 ccc(OC)cc2)CC1	1
CC(C)[C@@H]1CCC[C@]2(CC1)C[C@@H](CCO2)Cl	0
CN(C1C[C@H]2CC[C@@H](C1)[NH2+]2)C(=O)N3CCCC3	0
O=C(CCc1ccc(O)cc1)N(Cc1nc2c(c(=O)[nH]1)COCC2)Cc1cccs1	1
Cc1ccc2c(c1)c(=O)c3c(o2)C(=O)N([C@H]3c4ccccc4F)CCCN5CC OCC5	0
CC(C)(C)c1ccc(cc1)S(=O)(=O)N2CCN(CC2)CN3C(=O)[C@]4(C [NH+]5CCC4CC5)N=C3[O-]	0
C[C@H](C(=O)Nc1c(cc(cc1Cl)Cl)Cl)n2cnc3c2c(=O)n(c(=O)n3C) C	0
c1cc2c(cc1C(=O)N)nc(s2)Br	0
CC(C)c1[nH]c2c(c3c(s2)CCCC3)c(=S)n1	0
c1ccc(cc1)C#C/C(=N/c2ccc(cc2)[N+](=O)[O-])/c3ccccc3	0
c1cc-2c(cc1[N+](=O)[O-])C[C@H](c3c2cc(cc3Cl)Cl)C#N	0
[H]/N=C\1/C=C[C@@H](S1)c2nc(no2)c3cc(cs3)Br	0
CCc1nc2n(n1)c(c(s2)[C@@H](c3cccc(c3)F)N4C[C@H](O[C@H] (C4)C)C)O	0
C[C@@H](CC#N)N(C)c1nc(nc(n1)Cl)Cl	0
Cc1nnc(o1)SCC(=O)Nc2ccc(cc2N3CCOCC3)C(F)(F)F	0
CCOc1ccc(- c2nnc([C@H]3C[C@H](n4c(=O)[nH]c5cc(C#N)ccc54)C3)n2- c2ccccc2Cl)nc1	1
Cc1cc(n(n1)C)NC(=O)N[C@H](C)c2cc(oc2C)C	0
Cc1cc(n(n1)c2ccc(nn2)OCC(=O)N3CC[C@@H](C3)c4cc5nc(cc(n 5n4)C(F)(F)F)C)C	0
Cc1c(c(n(n1)C)Sc2nccc(n2)[O-])N	0
CC1(O[C@H]2[C@@H](O1)[C@@H](O[C@@H]3[C@H]2OC(O3)(C)C)C(=O)NCc4nc5ccccc5n4Cc6ccccc6)C	0
CCC[NH+](Cc1nnc(o1)c2cccs2)CC(=O)N3CCc4c3ccc(c4)S(=O)(	0

=O)N	
Cc1ccc(c(c1)Cl)S(=O)(=O)Nc2ccsc2C(=O)[O-]	0
c1cc(oc1)c2nc(c(o2)N3CCCC3)C#N	0
C[C@@H]1CCC[C@H](C1)CNC(=O)N2CCC3(CC2)OCCO3	0
O=C(CCc1ccc(Cl)cc1Cl)Nc1nnc2n1CCS2	1
CC(C)c1nc(n(n1)c2cccc3c2nccc3)C4CCC(CC4)O	0
C[C@@H](c1ccc(cc1Cl)Cl)NC(=O)[C@H](C)Sc2nnnn2c3ccc(cc3)[O-]	0
CC(c1cccs1)N(Cc1nc2c(c(=O)[nH]1)COCC2)C(=O)CN1CCC(C(=O)c2ccc(F)cc2)CC1	1
Cc1ccccc1N2C(=O)/C(=C\c3ccc(o3)c4ccc(cc4)F)/C(=NC2=O)[O-]	0
C1CCN(CC1)c2c3c(c(nc(n3)N(CCO)CCO)N4CCCCC4)nc(n2)N(CCO)CCO	0
Cc1ccc2c(c1)sc3n2c(=O)c(c(n3)C)S(=O)(=O)Nc4ccc(cc4)F	0
C[C@H](C(=O)Nc1c2ccccc2oc1C(=O)C)N3CCN(CC3)Cc4cn5ccccc5[nH+]4	0
c1cc(ccc1N[C@H]2CCCC[C@H]2O)N3CCCC3	0
C[NH+](C)c1ccc(cc1)[N-]C2=NC=NC3=NC=N[C@@H]32	0
Cc1ccc(nc1)NC[C@@]2(CCC[C@H](C2)C)O	0
CC[C@@H]1CO[C@H](CN1C(=O)C(C)(C)C#N)C	0
CCOC(=O)c1c2c(sc1NC(=O)[C@H](C)n3cc(nc3C)C)C[C@H](CC2)C	0
CCO[C@H](C[C@@](C(=S)Nc1ccccc1)(n2c3ccccc3nn2)Oc4ccccc4)c5ccccc5	0
Cc1c(oc(c1C#N)NC(=O)[C@H](C)[NH+]2CCc3cc(ccc3C2)F)C	0
C[NH+](C)CCN(c1nc2c(s1)cccc2S(=O)(=O)C)C(=O)CCS(=O)(=O)c3ccc(cc3)F	0
Cc1ccc(c(c1)C)[C@@H](C)NC(=O)c2cc(nc3c2c(no3)C)C	0
Cc1ccc(cc1)C(=O)Nc2nn3c(nnc3s2)c4ccccc4	0
C[C@@H]1[C@@H](O[C@@H]([C@@H]1C(=O)Nc2ccc(cc2)Br)C)C	0
C[C@H]1C[C@@H](n2c(c(cn2)c3n[nH]c(=S)n3[C@@H](C)c4ccccc4)N1)C(F)(F)F	0
COc1ccc(c(c1)OC)[C@H]2C[C@H](Nc3n2nc[nH+]3)c4ccc(cc4)Br	0
COc1ccc2sc3nncn3c2c1	1
COc1ccc2c(c1)CC1(CCN(C3CCN(Cc4nc5c(c(=O)[nH]4)COCC5)C3=O)CC1)C2=O	1
COc1cc(cc(c1OC)OC)/C=C\2/C(=O)N(C(=S)S2)NC(=O)c3cccc(c3)[N+](=O)[O-]	0
c1c(cnc(c1C#N)NCC2(CCCCC2)O)N	0
CC1(O[C@@H]2[C@H]([C@@H](O[C@@H]2O1)[C@@H]3CO3)O)C	0
C[C@H]1CCCC[C@@H]1N(C)C(=O)c2ccc(o2)S(=O)(=O)[NH-]	0
C[C@H](C12CC3CC(C1)CC(C3)C2)NC(=O)N4CCC(CC4)CO	0
Cc1c(sc(n1)C[NH3+])c2nc(no2)C3CCC(CC3)(C)C	0
c1ccc2c(c1)c(cc(n2)CCl)C#N	0
O=C1NN=C2CCCc3c2c2c1cccc2n3CCN1CCCC1	1
COc1ccc(cc1)N2C(=O)/C(=C/c3ccc(o3)N4CCCCC4)/C(=O)NC2=S	0
Cc1nc(cc(n1)Cl)Nc2ccc3c(c2)OCO3	0
C[C@H](CC(=O)N[C@@H]1CCCc2c1cnn2c3ccc(cc3)F)n4c5ccc	0

cc5nn4	
C[C@H]1C(=NN(C1=O)c2nc(cs2)c3cc4ccccc4[nH]3)C	0
Cc1cccc2c(=O)[nH]c(N3CCC4(CC3)CN(CCO)c3cc(F)cc(F)c34)nc12	1
c1cc2c(cc1n3c(n[nH]c3=S)C4CC4)OCO2	0
C[C@H](CC(=O)NCc1c[nH]nc1N)C(C)(C)C	0
C[C@@H](C(=O)N1CCc2c1cccc2)Nc3ccc(nc3)OC	0
COc1ccc(C2NC(=O)c3cccnc3N2)cc1	1
Cc1cc(ccc1N2C(=O)[C@@H]3[C@H]4c5ccccc5C=C[NH+]4[C@@H]([C@@H]3C2=O)C(=O)c6ccco6)[N+](=O)[O-]	0
CC[C@H]1[C@H](NC[C@@]2(O1)CCC[C@@H](C2)C)C	0
CCN(CC)C(=O)c1ccc2c(c1)N(C(=O)[C@H]3N2CCCC3)CC(=O)Nc4ccc5c(c4)OCO5	0
COc1ccccc1[C@H]2c3c(c4ccccc4[nH]3)C[C@H]5N2C(=O)CN(C5=O)CCN6CCOCC6	0
CC[C@]1(C(=O)N(C(=O)N1)CN2CCN(CC2)S(=O)(=O)N3CCCCCC3)c4ccccc4	0
O=C(CN1CCC(C(=O)N2CCc3ccccc3C2)CC1)NCc1nc2c(c(=O)[nH]1)COCC2	1
Cc1ccc(cc1C)Cc2nc(c(c(n2)NC)I)C(C)C	0
c1cc(cc(c1)C(F)(F)F)CNc2ccc(cc2[N+](=O)[O-])S(=O)(=O)N3CCOCC3	0
O=C(CCc1ccccc1F)Nc1nnc2n1CCS2	1
Cc1cc(c2cc(c3nn(c(=O)n3c2c1)C)C[NH2+]C4CCCC4)C	0
c1cc(c[nH+]c1)Cn2c(=O)c(nn(c2=O)c3ccc(cc3)Cl)C(=O)NCc4ccc(cc4)F	0
COc1cc(ccc1OS(=O)(=O)c2ccc(cc2)Cl)/C=N\N=C/3\NC(=O)CS3	0
C[C@H]1CCC[C@@H](C1)c2nc(on2)c3c4c(sc3N)CCC4	0
CN1CCCc2c1nc(N1CCC3(CC1)C(=O)N(C)c1cc(OCCN4CCCC4)cc(F)c13)[nH]c2=O	1
COCCNc1[n+](c(cs1)c2cc(ccc2OC)OC)/N=C/c3c4ccccc4ccc3O	0
Cc1c(c(=O)n2ccsc2n1)S(=O)(=O)NCCC[NH+]3CCN(CC3)c4ccccc4	0
C[C@H](c1cc2ccccc2o1)NC(=O)CN3CCN(CC3)S(=O)(=O)c4ccc5c(c4)OCCCO5	0
c1cc(c(cc1Cl)NC(=O)c2c(ccs2)C#CCO)C#N	0
Cc1c(nc(nc1N2CC(C2)[NH+]3CCCCC3)N)C	0
Cc1ccc(o1)/C=N\NC(=O)c2cc3ccccc3o2	0
c1ccc(cc1)C[NH+]2CC[C@@H](C2)NC(=O)CN3CCN(CC3)S(=O)(=O)c4ccccc4C#N	0
Cc1c(c(n(n1)C)C)S(=O)(=O)N2CCCN(CC2)C(=O)Nc3ccc(cc3)C(C)(C)C	0
Cc1ccc2c(c1)sc1nncn12	1
c1cc(c(c(c1C(=O)[O-])F)F)N[C@@H]2[C@@H]([C@@H]3[C@@H]2OCC3)[NH3+]	0
Cn1c2ccc(cc2oc1=O)S(=O)(=O)N3CCC[C@H](C3)C(=O)Nc4ccc(cc4)Cl	0
C[C@H](c1ccccc1)N2C(=O)/C(=C\c3c(nc4ccccn4c3=O)NCCOCCO)/SC2=S	0
CC(C)[NH+](Cc1cnn(c1)C)CC(=O)Nc2cc(ccc2n3cncn3)C(F)(F)F	0
[H]/N=C\1/[C@H]([C@H](C2=C(O1)CC(CC2=O)(C)C)c3c[nH]c4c3cccc4)C#N	0

**CCOC(=O)N1CCN(CC1)c2c(c(=O)n3cccc(c3n2)C)/C=C\4/C(=O)N(C(=S)S4)C5CCCC5**	0
**Cc1ccc2c(c1)c(=O)c3c(o2)C(=O)N([C@H]3c4ccc(cc4)O)c5nc(c(s5)C(=O)OC)C**	0
**C[C@@H]([C@@H](c1ccc(cc1)F)O)Nc2nccc(n2)Oc3ccc(cc3)F**	0
**C[C@@H]1C[C@@H]1n2c3ccc(cc3nc2N)Br**	0
**C1CCC(CC1)CN2CCN(CC2)C(=O)[C@H]3CCCN(C3)C(=O)N**	0
**CS(=O)(=O)c1ccc(cc1)c2[nH]nc(c(=O)n2)CCC(=O)N3CC[NH+](CC3)Cc4ccccc4**	0
**Cc1cc2c(cc1C(C)C)c(cc(=O)o2)CN3C(=O)C(NC3=O)(C)C**	0
**C=CCOc1cccc(c1)[C@H]2C(=C(C(=O)N2CCCn3cc[nH+]c3)O)C(=O)c4ccncc4**	0
**c1cc(c(cc1C(=O)Nc2ccc3c(c2)OCCCO3)[N+](=O)[O-])Cl**	0
**COc1ccc(C2(CNC(=O)c3ccc(NC(=O)c4cnc(C)o4)cc3Cl)CCOCC2)cc1**	1
**CCc1nnc(n1/N=C/c2ccc(c(c2)O)O)S**	0
**NC(=O)c1ccc(Oc2cccc(C(N)=O)c2)cc1**	1
**Cc1ccc2c(c1)c3c([nH]2)CCN(C3)C(=O)C[C@@]4(CC(=O)N(C4=O)Cc5ccc[nH+]c5)c6ccccc6**	0
**Cc1cc(n(n1)c2cc(ncn2)N3CCC[C@H](C3)C(=O)NCCn4c(=O)ccc(n4)c5ccc(cc5)F)C**	0
**c1ccc(cc1)c2ccccc2C(=O)Nc3ccc(c(c3)S(=O)(=O)N4CCOCC4)N5CCOCC5**	0
**C[C@]1(CCN(C1)C(=O)NC2(CCCCC2)C)C(=O)[O-]**	0
**c1ccc(c(c1)N/N=N/c2ccccc2Cl)Cl**	0
**CCn1c(ccn1)[C@@]2(C[C@@H](CC(C2)(C)C)C)O**	0
**Cc1cc(no1)NC(=O)C2CC[NH+](CC2)Cc3[nH]c4cccc(c4n3)F**	0
**CCOc1ccc(cc1)Nc2[n+](c(cs2)c3ccc(cc3)S(=O)(=O)N4CCCC4)C[C@H]5CCCO5**	0
**COc1ccc(cc1)n2c(c3c(n2)CS(=O)(=O)C3)NC(=O)C(=O)NCCCc4ccccc4**	0
**C[C@H]1CC[C@@H]([C@@H](C1)[NH3+])C(C)(C)c2ccc3c(c2)OCCO3**	0
**CC(C)CN1C(=O)N(C(=O)C12CCN(CC2)C[C@H]3C=NN=C3c4ccccc4)C5Cc6ccccc6C5**	0
**C1C[C@H]2[C@@H](C[C@@H]1[NH+]2c3c(c(c(c(c3F)F)F)F)F)C(=O)[O-]**	0
**Cc1c(c(on1)C)S(=O)(=O)NCC(=O)O[C@@H](C)C(=O)Nc2cc(ccc2Cl)Cl**	0
**Cc1cc(c2c(c1)c(c3c([nH+]2)[C@H](CC[C@@H]3C)C)C(=O)[O-])C**	0
**c1cc(cc(c1)Br)NC(=O)/C(=C/c2ccc(o2)c3ccc(c(c3)Cl)C(=O)[O-])/C#N**	0
**[H]/N=C\1/C(=C/c2ccc(c(c2)Cl)OCc3ccc(cc3)C)/C(=O)N=C4N1N=C(S4)S(=O)(=O)C**	0
**C[C@H](C(=O)Nc1ccc(cc1)S(=O)(=O)N2CCCC2)Sc3ccccn3**	0
**c1cc(cc(c1)S(=O)(=O)N2CCCCC2)NS(=O)(=O)c3ccc4c(c3)OCCCO4**	0
**Cc1c(c(n2c(n1)nc(n2)NCc3ccc(cc3)F)[O-])Cl**	0
**CC(=O)c1cccc(c1)[N-]S(=O)(=O)c2ccc(cc2)N3C(=O)C(CS3(=O)=O)(C)C**	0
**CN(c1ccc(cc1)CN)C(=O)N2CCCCCC2**	0

**COc1ccc(cc1)[C@@H]2[C@H]3CSCC=C3C(C(=C2C#N)N)(C#N)C#N**	0
**COCCCN1C(=O)/C(=C/c2c(nc3ccccn3c2=O)N4CC[NH+](CC4)Cc5ccc6c(c5)OCO6)/SC1=S**	0
**C[C@@H]1CN(C[C@H](O1)C)S(=O)(=O)N(C)Cc2cn(nc2c3cccc c3)Cc4ccccc4Cl**	0
**C[C@H](C(=O)N1CCNC1=O)Sc2ccc(c[nH+]2)S(=O)(=O)N3CCCCCC3**	0
**Cc1ccc(-c2cc3cnccc3c(=O)[nH]2)cc1**	1
**c1ccc(cc1)S(=O)(=O)N2CCN([C@@H](C2)C(=O)Nc3ccc(cc3)Br)S(=O)(=O)c4ccccc4**	0
**Cc1cnc(cn1)CNc2cc3c(cc2[NH3+])NC(=O)CC3**	0
**CO[C@H]1CCC[C@H]1N2CC(=O)NC2=O**	0
**CC[C@H](C)N(Cc1nc2c(c(=O)[nH]1)COCC2)C(=O)CN1CCC(C(=O)c2ccc(F)cc2)CC1**	1
**c1cc2c(cc1C(=O)c3ccc(c(c3)N)Br)OCCCO2**	0
**Cc1cc(=O)[nH]c2cc(-c3ccccc3)ccc12**	1
**C1CCCC(CCC1)c2nc([nH]n2)C3CCCC3**	0
**c1cc(ccc1F)S(=O)(=O)Nc2cc(cc(c2)C(=O)N3CCOCC3)C(=O)N4CCOCC4**	0
**C1C=C([C@H]2[C@@H]1C(=CC2)C#N)C#N**	0
**Cc1ccc(c(c1C)OC(=O)c2cc3ccccc3oc2=O)C**	0
**CC(=O)OC[C@]1(CCC[C@@]2([C@H]1C[C@@H]([C@@]34[C@@H]2C[C@@H]([C@@H]([C@@H]3OC(=O)C)C(=C)C4=O)OC(=O)C)OC(=O)C)C)C**	0
**Cc1cc(cc(c1)Nc2ccc(cc2C=O)Br)C**	0
**Cc1cccc(c1)OCCn2cc(c3c2cccc3)/C=C\4/C(=O)N=C(N(C4=O)Cc5ccco5)[O-]**	0
**CC[NH+]1CCC[C@H]1CN2C[C@@H](CC2=O)C(=O)NNc3cc(cc(n3)Cl)C#N**	0
**CCOC(=O)c1c2c(sc1NC(=O)c3cc4n(n3)[C@@H](C[C@H](N4)c5ccc6c(c5)OCO6)C(F)(F)F)CCCC2**	0
**CCCOc1c(c[nH+]c2c1c(=O)n(c(=O)n2C)CC(=O)Nc3ccccc3C(F)(F)F)C**	0
**CN1CCCc2c1nc(N1CCC3(CC1)C(=O)N(CC1COC1)c1cc(F)cc(F)c13)[nH]c2=O**	1
**C[C@@]1(CC[C@]2(C(=C1)C(=O)C[C@H]3[C@]2(CCCC3(C)C)C(=O)[O-])O)C=C**	0
**C[C@H]1CCCC[C@H]1Cn2c(n[nH]c2=S)N**	0
**c1cn(nc1CN)[C@@H]2CCOC3(C2)CCCCC3**	0
**COc1cccc2c1c(=O)n(C[C@H]1CC[C@H](C(=O)Nc3ccncc3)CC1)c(=O)n2Cc1cccc(C(F)(F)F)c1C#N**	1
**CC(C)(C)Oc1nc2ccc(cc2s1)N**	0
**c1ccc2c(c1)CC[C@@H]2c3nc(on3)C4(CCCCC4)N**	0
**C[C@H]1CN(C[C@@H](O1)C)C(=O)Nc2ccc(c(c2)F)N3CCCC3**	0
**CC(C)(C)c1ccc(cc1)S(=O)(=O)N2CCN(CC2)C(=S)Nc3ccc(cc3)S(=O)(=O)N4CCOCC4**	0
**CCOc1ccc(-c2nnc([C@H]3C[C@H](NC(=O)c4cccc5nc(C)c(C)nc45)C3)n2-c2ccccc2F)nc1**	1
**C[C@H](Cn1cncn1)Nc2c3c(csc3nc(n2)CN(C)C)c4ccccc4**	0
**Cc1cccc(c1)[C@@H]2C3=C(CN(C3=O)[C@@H](Cc4ccccc4)C(=**	0

O)N5CCOCC5)N(C(=O)N2)CC=C	
O=c1[nH]c(-c2ccc(C(F)(F)F)cc2)nc2c1CCCC2	1
O=C(NCc1ccc(-c2nc3ccccc3c(=O)[nH]2)cc1)OCc1ccccc1	1
CC(=O)N(C)Cc1nc(cc(n1)NC)[C@@H]2CCC[NH+](C2)Cc3ccc4c(c3)CCCN4C	0
Cc1c(c(on1)C)CCCNC(=O)N2C[C@H]3CC[C@@H]2C3	0
C[C@H]1CCCC[C@H]1NC(=O)Nc2cc3c(cc2SC)OCCO3	0
COc1ccc(-c2noc(CSc3nnc(C)n3-c3ccc(OC)cc3)n2)cc1	1
COc1cc(cc(c1O)Br)/C=C/C(=O)c2ccc3c(c2)NC(=O)CO3	0
COc1ccc(cc1)c2c(cn(n2)CCC#N)C(=O)Nc3ccccc3Cl	0
C[C@@H]1CN(C[C@H](O1)C)c2cncc(n2)NC	0
CC1(C(C1(C)C)n2c3cc(c(cc3[nH]c2=S)F)OC)C	0
COc1ccc(cc1)N2C(=O)/C(=C/c3ccc(c(c3)OC)OC(=O)c4ccccc4I)/SC2=S	0
C[C@]12CCC(=O)C=C1CC[C@@H]3[C@@H]2[C@H](C[C@]4([C@H]3CC[C@@]4(C(=O)COC(=O)CCC(=O)N5C[C@H](C[C@H]5C(=O)OC)O)O)C)O	0
c1cc(nc(c1)Nc2ccc3c(c2)cccn3)C#N	0
CCOc1ccc(-c2nnc(C34CC(NC(=O)c5ccnc6cccnc56)(C3)C4)n2-c2ccncc2)nc1	1
CN1CCc2c1ccc(c2)[C@@H](CNC(=O)C(=O)Nc3ccc(c(c3)[N+](=O)[O-])F)[NH+](C)C	0
CC[NH+](CC)c1ccc(c(c1)O)/C=C/2\C(=O)NC(=O)N(C2=O)c3ccc(c(c3)C)Br	0
c1cc2c(cc1COc3c(cc(cn3)Br)N)OCO2	0
Cc1c(cn(n1)C)[C@@H](C)Nc2c3ccsc3ncn2	0
c1ccc(cc1)C2(CCOCC2)CNC(=O)[C@H]3CC34CCN(CC4)C(=O)c5ccc6c(c5)no[n+]6[O-]	0
Cc1ccc(cc1)c2nnc(n2N)SCC(=O)Nc3c(cc(cn3)C(F)(F)F)Cl	0
CC(=O)N1C=Cc2ccccc2[C@H]1CC(=O)Nc3cc(ccc3n4cncn4)Cl	0
Cc1cn2c3c([nH+]c2n1CCOCCO)n(c(=O)n(c3=O)Cc4cccc5c4cccc5)C	0
C[C@@H](CNc1cc2c(cc1N)CC(=O)N2)COC	0
COc1cc(cc(c1OC)OC)/C=C\2/C(=O)N(C(=S)S2)CCC(=O)Nc3ccc(cc3)F	0
CCOc1ccc(-c2nnc([C@H]3C[C@H](NC(=O)c4ccnc5cc(F)cnc45)C3)n2-c2ccc(C)s2)nc1	1
Cc1cc(no1)CN2[C@@H](C(=O)N[C@@H](C2=O)C(C)C)C	0
c1ccc2c(c1)cc(o2)C(=O)C3=C(C(=O)N([C@H]3c4ccc(cc4)O)CCC[NH+]5CCOCC5)[O-]	0
COc1ccc(cc1OC)S(=O)(=O)NC[C@@H](c2ccc[nH+]c2)S(=O)(=O)c3ccc(cc3)Cl	0
COc1ccc(c(c1)S(=O)(=O)NC[C@H](c2ccc[nH+]c2)S(=O)(=O)c3ccc(cc3)Cl)OC	0
c1cc(ccc1c2c(cn[nH]2)Cn3ccnc3c4cc5n(n4)CCNC5)F	0
Cc1cc(ccc1Br)N2C(=O)/C(=C\c3ccc(c(c3)OC)OC(C)C)/C(=NC2=O)[O-]	0
Cc1ccc(cc1C)S(=O)(=O)N2CC[NH+](CC2)Cc3nc4cc(ccc4o3)C(=O)N5CCCCC5	0
Cc1nc2c(c(n1)N(C)C[C@@H]3COc4ccccc4O3)CC[NH2+]CC2	0
O=c1[nH]nc2c3c(cccc13)NC1=C2CCCCC1	1

c1ccnc(c1)[C@H]2C(=C(C(=O)N2CC[NH+]3CCCCC3)[O-])C(=O)/C=C/c4ccco4	0
CCn1cc(c(n1)C(=O)N2[C@H](CC(=N2)c3ccc4c(c3)OCCO4)c5ccccc5O)Cl	0
CC(c1nc2c(c(=O)[nH]1)COCC2)N(C)C(=O)CN1CCC(C(=O)c2ccc(F)cc2)CC1	1
COc1cc(cc(c1OC)OC)NC(=O)C2CCN(CC2)c3c4c([nH+]c[nH]4)nc(n3)Cl	0
C[C@@H](C(=O)NC1=c2cc(ccc2=[NH+][C@H]1C(=O)OC)Cl)N3CCN(CC3)c4cccc(c4)C(F)(F)F	0
O=C(CCc1c[nH]c2ccccc12)N(Cc1nc2c(c(=O)[nH]1)COCC2)Cc1cccs1	1
COc1ccc2c3c1O[C@H]4[C@@]3(CCN(C2)c5ccnc(n5)NCc6ccccc6)C=C[C@@H](C4)O	0
Cc1c(n2cc(cc(c2n1)Cl)Cl)N	0
c1cc(sc1)c2ccc([nH+]n2)N3CCC[C@@H](C3)C(=O)Nc4ccc(cc4)S(=O)(=O)Nc5ncccn5	0
C[C@@H](C(=O)Nc1ccccc1Br)Sc2ccc(cc2)NC(=O)c3ccc(cc3)[N+](=O)[O-]	0
c1ccc2c(c1)C[C@@H](CO2)NCc3cccc4c3non4	0
Cc1c(c(=O)n(n1C)c2ccccc2)NC(=O)c3ccc(cc3)S(=O)(=O)NCC=C	0
c1ccc2c(c1)ccnc2CSc3nc(cnn3)O	0
C1CCC(C1)(C#N)N2CCCC2	0
CC(C)(O)c1ccc(-c2nc3c(Cl)cccc3c(=O)[nH]2)cc1	1
Cc1ccc(cc1)/C=C/2\C(=O)N(C(=O)S2)CCNC(=O)[C@@H]3CS[C@@]4(N3C(=O)CC4)C	0
c1ccc2c(c1)nc(s2)[C@H]3CC=CC[C@H]3C(=O)NCc4nc(no4)c5ccncc5	0
C[NH+]1CCC[C@@H](C1)N(C)C(=O)NC2CCC(CC2)C(=O)[O-]	0
Cc1cccc(c1[N+](=O)[O-])C(=O)Nc2ccc(cc2)c3nccs3	0
C[C@H]1CC[C@@H](N(C1)C(=O)Nc2cc(c(cc2OC)OC)F)C	0
CCCN1c2ccccc2[C@]3(C1=O)C(=C(C(=O)N3CC[NH+](C)C)O)C(=O)c4ccc(cc4)S(=O)(=O)N5CCOCC5	0
CN1CCCc2c1cc(-c1ccc(Cl)cc1)[nH]c2=O	1
C[C@H]1c2ccsc2CCN1C(=O)c3cc(c[nH]3)Br	0
Cc1cc(cc(c1N)C(=O)[O-])N2CC[NH+]([C@@H](C2)C)C	0
CCc1cccc(c1NC(=S)N/N=C\2/c3ccccc3N(C2=O)C[NH+]4CCN(CC4)C(=O)OCC)CC	0
CCN(c1cccc(c1)c2ccnc3n2ncc3C(=O)NCCCC(=O)[O-])C(=O)C	0
C[C@@H]1[C@@H](CC[NH+]1c2ccc(cc2)S(=O)(=O)NC)C(=O)[O-]	0
CCOc1cc(cc(c1OS(=O)(=O)C)Br)[C@H]2N(N=C(S2)NC(=O)C)C(=O)C	0
CCc1ccc(cc1)[N-]S(=O)(=O)c2c[nH]cn2	0
Cc1c(ccc(n1)C(=O)Nc2ccc(cc2)/N=N/c3ccccc3)C#N	0
O=C1Nc2cc(Cl)cc(Cl)c2C12CCN(c1nc3c(c(=O)[nH]1)CCCC3)CC2	1
c1ccnc(c1)c2nc3c(c(n2)NCCCN4CCCC4=O)CCNC3	0
Cc1ccn(n1)[C@@H]2CC3(CC[C@H]2[NH3+])OCCO3	0
Cc1cc(cc(c1O)C)C[NH+]2CCN(CC2)c3ccccc3[C@@H](c4nccn4C)O	0

**O=C1NC(c2ccc(C(F)(F)F)cc2)=NC2CSCC12**	1
**C[NH+](C)CCn1cc(cn1)NC(=O)c2cc(c(cc2I)OC)OC**	0
**Cn1cccc1[C@H](CNC(=O)[C@H]2CC(=O)N(C2)c3ccccc3)N4C CN(CC4)C**	0
**Cc1cc(c(cc1C)S(=O)(=O)N)C**	0

Table S7. Complete curated dataset used for machine-learning model development, comprising 727 experimentally validated TNKS1 inhibitors (Label = 1) and 3,635 DUD-E property-matched decoys (Label = 0). The dataset is provided in CSV format and includes the molecular SMILES representation and corresponding class label.

Compound_ID	SMILES	Label	Dataset_Split
TNKS1_ML_0001	CCc1noc2nc(CCC(=O)NCCN3CC[NH+](CC)CC3)nc(N3CC CCC3)c12	0	Train
TNKS1_ML_0002	COc1cccc2c(=O)[nH]c(-c3ccc(C)cc3)nc12	1	Train
TNKS1_ML_0003	Cc1csc(-c2ccc(NC(=O)NCc3cc(Br)c4c(c3)OCO4)cc2)n1	0	Train

TNKS1_ML_0004	CC1CCC([NH+]2CCN(C(=O)NC[C@@H]3C[NH+]4CCCC[C@@H]4CO3)CC2)CC1	0	Train
TNKS1_ML_0005	Cc1cc(=O)n(C)cc1NC(=O)N[C@H]1[C@@H]2CCc3ccccc3[C@H]12	0	Train
TNKS1_ML_0006	C[C@H]1C[C@H](C)CN(c2ccccc2C(C)(C)C)C1	0	Train
TNKS1_ML_0007	COC(=O)NC(=O)c1c(NC(=O)[C@@H]2CCCCN2S(=O)(=O)c2cccs2)sc2c1CCCC2	0	Train
TNKS1_ML_0008	O=C(CSc1nc(CN2CCOCC2)nc2ccccc12)c1c[nH]c(C(=O)N2CCCC2)c1	0	Train
TNKS1_ML_0009	CNCc1ccc(-c2[nH]c3cc(F)cc4c3c2CCNC4=O)cc1	1	Train
TNKS1_ML_0010	CCN1CCN(S(=O)(=O)c2ccc(NC(=O)[C@@H]3CC(=O)N(C4CCCC4)C3)cc2)CC1	0	Train
TNKS1_ML_0011	COc1ccc(CN(Cc2nc3c(c(=O)[nH]2)COCC3)C(=O)CN2CCC(C(=O)c3ccc(F)cc3)CC2)cc1	1	Train
TNKS1_ML_0012	Cc1ccc(C(=O)[C@@H]2C(=O)C(=O)N(c3ccc(CC(=O)[O-])cc3)[C@H]2c2ccc(C)cc2C)cc1	0	Train
TNKS1_ML_0013	O=C(CCNS(=O)(=O)c1ccc2c(c1)OCCO2)NCCN1C(=O)S/C(=C\c2ccc[nH+]c2)C1=O	0	Train
TNKS1_ML_0014	Cc1n[nH]c(C)c1S(=O)(=O)N1CCCC[C@H]1c1nc2c(c(C)[nH+]1)CCCN2CC1CC1	0	Train
TNKS1_ML_0015	O=C(Nc1cccc(-c2ccc(N3CCOCC3)[nH+]n2)c1)[C@@H]1CC(=O)N(c2ccccc2)C1	0	Train
TNKS1_ML_0016	C[C@@H](OC(=O)/C=C/c1ccc(Cl)c([N+](=O)[O-])c1)C(=O)Nc1ccc(OC(F)(F)F)cc1	0	Train
TNKS1_ML_0017	CCSc1nnc(NC(=O)CSc2nnc(NC(=O)c3ccc(C)c(C)c3)s2)s1	0	Train
TNKS1_ML_0018	O=C(N[C@H](n1cnc2c(ncn2[C@@H](NC(=O)c2ccccc2)C(Cl)(Cl)Cl)c1=O)C(Cl)(Cl)Cl)c1ccccc1	0	Train
TNKS1_ML_0019	Cc1cc(C)n2c(SCC(=O)NNC(=O)c3ccc(Br)cc3)nnc2n1	0	Train
TNKS1_ML_0020	Nc1cccc2oc(N3CCCN4CCC[C@@H]4C3)nc12	0	Train
TNKS1_ML_0021	C/N=C(\N)SCc1cc(=O)oc2cc(C)c(Cl)cc12	0	Train
TNKS1_ML_0022	Cc1cc(C)c(NC(=O)c2cc(Cl)ccc2[N+](=O)[O-])c(C)c1	0	Train
TNKS1_ML_0023	CN1CCCc2c1nc(N1CCC3(CC1)C(=O)N(CC#N)c1cc(F)cc(F)c13)[nH]c2=O	1	Train
TNKS1_ML_0024	Cc1nc(C(C)(C)C)sc1C(=O)NCCN1C[C@@H](C)O[C@H](C)C1	0	Train
TNKS1_ML_0025	CNc1cnc(C(C)C)nc1C(=O)N1CCC[C@H](C)[C@H]1C	0	Train
TNKS1_ML_0026	Cc1ccc(C(=O)N2[C@H](C(=O)NC[C@@H]3CCCO3)COC23CCN(C(=O)c2ccco2)CC3)cc1	0	Train
TNKS1_ML_0027	O=C(CN1CCN(CC(=O)N2CCCC2)CC1)Nc1ccc(S(=O)(=O)N2CCCCC2)cc1	0	Train
TNKS1_ML_0028	O=C(NC[C@@H](c1cccnc1)[NH+]1CCN(c2ccccc2)CC1)C(=O)Nc1ccccc1[N+](=O)[O-]	0	Train
TNKS1_ML_0029	O=C(NCCn1ccnc1)c1cc(NCc2ccco2)cc(S(=O)(=O)N2CCCC2)c1	0	Train
TNKS1_ML_0030	CCOc1ccc(C(=O)C2=C(O)C(=O)N(CCCn3cc[nH+]c3)[C@@H]2c2cccc(Br)c2)cc1Cl	0	Train
TNKS1_ML_0031	COc1ccccc1N1CCN(c2nc3c(c(=O)[nH]2)COCC3)CC1	1	Train
TNKS1_ML_0032	O=C([O-])c1cccnc1SCC(=O)N1N=C2/C(=C/c3ccco3)CCC[C@@H]2[C@@H]1c1ccco1	0	Train
TNKS1_ML_0033	Cc1nn2c(nnc3c(=O)n(NC(=O)c4ccc(Cl)cc4)ccc32)c1-c1ccc(Cl)cc1	0	Train
TNKS1_ML_0034	CCOC(=O)C[C@@H]1C(=O)NCCN1C(=O)COc1ccc(Br)cc1C	0	Train
TNKS1_ML_0035	C[C@H]1CCCC[C@]12C[C@H](O)CCO2	0	Train
TNKS1_ML_0036	Cc1nc(C(=O)Nc2ccc3c(c2)COC3)nn1-c1c(Cl)cccc1Cl	0	Train
TNKS1_ML_0037	O=C1N(CCO)c2cc(F)cc(F)c2C12CCN(c1nc3c(c(=O)[nH]1)	1	Train

	CCCC3)CC2		
TNKS1_ML_0038	N#Cc1ccc(NC(=O)C(=O)NCc2ccc(-n3c[nH+]c4ccccc43)cc2)cc1	0	Train
TNKS1_ML_0039	CC(c1nc2c(c(=O)[nH]1)COCC2)N(C)C(=O)CN1CCC(C(=O)c2ccc(F)cc2)CC1	1	Train
TNKS1_ML_0040	COC(=O)c1[nH]c(C)c(C(=O)C2=C([O-])C(=O)N(CCCn3cc[nH+]c3)[C@H]2c2ccc(Cl)cc2)c1C	0	Train
TNKS1_ML_0041	Cc1ccc2c(c1)=C(O)/C(=C\N=C(\N)[S-])C(=O)[NH+]=2	0	Train
TNKS1_ML_0042	Cc1ccn([C@H](c2ccc(F)cc2)[C@@H](C)N)n1	0	Train
TNKS1_ML_0043	N#Cc1ccc2c(c1)[nH]c(=O)n2-c1ccc(-c2nnc(-c3ccncn3)n2-c2ccccc2Cl)cc1	1	Train
TNKS1_ML_0044	N#CC1(N2CCCC2)CCCC1	0	Train
TNKS1_ML_0045	O=C([O-])[C@H]1CC=CC[C@@H]1[C@H](O)c1c(Cl)cc(Cl)cc1Cl	0	Train
TNKS1_ML_0046	Nc1cc(Br)cnc1OCc1ccc2c(c1)OCO2	0	Train
TNKS1_ML_0047	O=C(NCCN1C(=O)S/C(=C\c2ccc(Cl)c([N+](=O)[O-])c2)C1=O)c1ccc(OC(F)F)cc1	0	Train
TNKS1_ML_0048	C[C@H](C(=O)NC1CCCCC1)[C@@H]1CC[C@@]2(C)CC[C@H](NC(=O)c3cncnc3)[C@@H](C)[C@@H]2[C@H]1O	0	Train
TNKS1_ML_0049	O=C(CN1CCC(c2nnc(-c3ccccc3)o2)CC1)NCc1nc2c(c(=O)[nH]1)COCC2	1	Train
TNKS1_ML_0050	C[C@@H](Nc1cccc(N2CCCC2=O)c1)C(=O)Nc1ccc(N2CCOCC2)cc1	0	Train
TNKS1_ML_0051	CN(Cc1[nH]cc[nH+]1)C(=O)Nc1cc(Cl)ccc1Cl	0	Train
TNKS1_ML_0052	CC(Cl)(Cl)C(=O)O/N=C1\CCCCCN1	0	Train
TNKS1_ML_0053	CN(C)S(=O)(=O)c1ccc2c(c1)nc(CCC(=O)NC1[NH+]=c3ccccc3=[NH+]1)n2C	0	Train
TNKS1_ML_0054	Cc1cccn2cc(CNC(=O)c3cn(C4CCCC4)cc(C(=O)N(C)CC(C)C)c3=O)[nH+]c12	0	Train
TNKS1_ML_0055	CC(C)C(=O)NCCNC(=O)N[C@H](C[NH+](C)C)c1ccccc1	0	Train
TNKS1_ML_0056	O=C(c1ccc(F)cc1)C1CCN(CC(=O)N(Cc2nc3c(c(=O)[nH]2)COCC3)Cc2nc3ccccc3s2)CC1	1	Train
TNKS1_ML_0057	[NH3+]C[C@@H]1CCCC[C@H]1c1nc(-c2ccc(F)cn2)no1	0	Train
TNKS1_ML_0058	Cc1cc(Br)cc(C)c1NS(=O)(=O)c1ccc(=O)[nH]c1	0	Train
TNKS1_ML_0059	O=C1c2[nH]nc(-c3ccc(Cl)cc3)c2[C@@H](c2ccc([N+](=O)[O-])cc2)N1Cc1cccnc1	0	Train
TNKS1_ML_0060	COCC(=O)N([C@@H](C(=O)NCS(=O)(=O)c1ccc(C)cc1)c1cc([N+](=O)[O-])ccc1Cl)C(C)(C)C	0	Train
TNKS1_ML_0061	CC(=O)N[C@@H](C(=O)N1CCC[NH+](CC(=O)N2CCCC2)CC1)C(C)C	0	Train
TNKS1_ML_0062	Cc1sc2nc(CC[NH3+])nc(N3CCN(c4ncccc4C#N)CC3)c2c1C	0	Train
TNKS1_ML_0063	CS(=O)(=O)Nc1ccc(N[C@@H]2CCCCc3ccccc32)cc1	0	Train
TNKS1_ML_0064	N#Cc1cccnc1N1CCN(C(=O)c2cccc(Cc3n[nH]c(=O)c4ccccc34)c2)CC1	1	Train
TNKS1_ML_0065	Cc1onc(-c2c(Cl)cccc2Cl)c1/C(O)=N/CCCN1CCOCC1	0	Train
TNKS1_ML_0066	C[C@H](Oc1ccc(/C=C2/C(=[NH2+])N3C(=NC2=O)SN=C3S(C)(=O)=O)cc1)c1ccccc1	0	Train
TNKS1_ML_0067	CC(C)(CNC(=O)C#Cc1ccc2c(c1)OCO2)c1cc[nH+]cc1	0	Train
TNKS1_ML_0068	COc1ccc(C2(CNC(=O)c3ccc(NC(=O)c4ccc(C)o4)cc3)CCOCC2)cc1	1	Train
TNKS1_ML_0069	O=C1NC(c2ccccc2OCc2ccccc2)Nc2ccccc21	1	Train
TNKS1_ML_0070	Cc1ccc(N2C(=O)/C(=C/c3c(C)n(Cc4ccc(Br)cc4)c4ccccc34)C(=O)N=C2[O-])cc1	0	Train
TNKS1_ML_0071	C=CCOC(=O)C1=C(N)OC(C)=C(C(=O)OC(C)C)[C@]12C(=O)Nc1ccc(Br)cc12	0	Train
TNKS1_ML_0072	Cc1noc(C)c1[C@H](C)NC(=O)N1CC[C@@H](C)C[C@@H]1C	0	Train

TNKS1_ML_0073	COc1ccc(-c2noc(CSc3nnc(-c4ccncc4)n3-c3ccc(OC)cc3)n2)cc1	1	Train
TNKS1_ML_0074	CC(C)(O)c1ccc(-c2cn3cccc3c(=O)[nH]2)cc1	1	Train
TNKS1_ML_0075	Cc1cc(C)c(NC(=O)c2csc(Br)c2)c(C)n1	0	Train
TNKS1_ML_0076	CCCCNC(=O)[C@H]1Sc2nnc(-c3ccccc3)n2N[C@H]1c1ccc(OC)c(Cl)c1	0	Train
TNKS1_ML_0077	Cc1ccc(NC[C@]2(O)CCC[C@@H](C)C2)nc1	0	Train
TNKS1_ML_0078	CCCNC(=O)N1CCc2cc(S(=O)(=O)NC3CCCCCCC3)ccc21	0	Train
TNKS1_ML_0079	Cc1cccc(N2C[C@@H](C(=O)Nc3nc4ccc(S(C)(=O)=O)cc4s3)CC2=O)c1C	0	Train
TNKS1_ML_0080	COC(=O)[C@]1(C)O[C@@]12CCCC(C)(C)C2	0	Train
TNKS1_ML_0081	Cc1nnc2c(N[C@@H]3CCCCC[C@H]3C)nccn12	0	Train
TNKS1_ML_0082	Cc1ccc(/C=N\NC(=O)c2cccc(S(=O)(=O)[N-]c3ccc(Cl)cc3)c2)c(C)c1	0	Train
TNKS1_ML_0083	O=C(NCCS(=O)(=O)N1CCCC1)c1cc(-c2cc[nH+]cc2)c2cnccc2n1	0	Train
TNKS1_ML_0084	O=C(NCCC1=c2ccccc2=[NH+]C1)N[C@@H]1CC(=O)N(c2ccc3c(c2)OCCO3)C1	0	Train
TNKS1_ML_0085	O=C(CN1CCC(C(=O)c2ccc3c(c2)CCO3)CC1)NCc1nc2c(c(=O)[nH]1)COCC2	1	Train
TNKS1_ML_0086	CCOc1ccc(-c2nnc([C@H]3C[C@H](NC(=O)c4ccccn4)C3)n2-c2ccccc2F)nc1	1	Train
TNKS1_ML_0087	CN(C)c1ccc(NC(=O)c2nn(-c3ccccc3)cc2O)c[nH+]1	0	Train
TNKS1_ML_0088	COc1ccc(-c2c(C(=O)Nc3ccc(F)cc3)nn3c(C)cc(C)nc23)cc1S(=O)(=O)N1CCCC1	0	Train
TNKS1_ML_0089	C[C@H]1C[C@H](C)CN(C(=O)C[NH+](C)[C@@H](C)c2ccc(S(N)(=O)=O)cc2)C1	0	Train
TNKS1_ML_0090	O=S1(=O)CC[C@H](Nc2cccc(F)c2F)c2ccccc21	0	Train
TNKS1_ML_0091	C[C@H]1CCCC[C@@]12NC(=O)N(CN1CCN(S(=O)(=O)c3ccc4c(c3)CCC4)CC1)C2=O	0	Train
TNKS1_ML_0092	N#Cc1cc(Cl)ccc1Sc1nc(N)cc(N)n1	0	Train
TNKS1_ML_0093	C[C@@H]1CC[C@@H](C[NH3+])CN1Cc1nc(-c2ccccn2)no1	0	Train
TNKS1_ML_0094	O=C(/C=C\c1ccc2c(c1)OCO2)N1CCC[C@H](Cc2nc(-c3ccsc3)no2)C1	0	Train
TNKS1_ML_0095	CC1=C2C[C@H]3[C@](C)(O)CCC[C@]3(C)C[C@]2(O)OC1=O	0	Train
TNKS1_ML_0096	CC(C)[C@H](NC(=O)C(=O)Nc1ccc(Cl)cc1)c1ccc2c(c1)OCCO2	0	Train
TNKS1_ML_0097	Cc1ccc(NC(=O)N(C)[C@H](C)C(C)C)c(C(=O)[O-])c1	0	Train
TNKS1_ML_0098	Cc1nc2c(cc(O[C@H]3CCCC[C@@H]3O)c3ccsc32)s1	0	Train
TNKS1_ML_0099	O=C(Nc1ccc(Cl)cc1)N1C[C@@H]2C[C@H](C1)c1cccc(=O)n1C2	0	Train
TNKS1_ML_0100	COc1ccc(C(=O)C2CCN([C@H]3CCN(Cc4nc5c(c(=O)[nH]4)COCC5)C3=O)CC2)cc1	1	Train
TNKS1_ML_0101	O=C1NN=C2CCCc3c2c2c1cccc2n3CCN1CCCCC1	1	Train
TNKS1_ML_0102	COc1cc(/C=C(C#N)/C([O-])=N/c2cccc3ccccc23)cc(Br)c1OC(C)=O	0	Train
TNKS1_ML_0103	CC(C)C1Cc2[nH]c(=O)c3ccccc3c2CO1	1	Train
TNKS1_ML_0104	Cc1nn(-c2ccccc2)c(C)c1CN(C)C(=O)CCNC1=NS(=O)(=O)c2ccccc21	0	Train
TNKS1_ML_0105	C[C@H]1CC[C@H](Nc2ncc(Br)cc2N)C[C@@H]1C	0	Train
TNKS1_ML_0106	N#Cc1ccccc1Cn1c(=O)n(C[C@H]2CC[C@H](C(=O)Nc3ccncc3)CC2)c(=O)c2ncccc21	1	Train
TNKS1_ML_0107	COc1cccc([C@H]2C[C@H](c3cc(Br)ccc3F)n3nnnc3N2)c1	0	Train

TNKS1_ML_0108	O=C1NC2(CCN(c3nc4c(c(=O)[nH]3)CCCC4)CC2)c2ccccc21	1	Train
TNKS1_ML_0109	C[C@H]1CN(C(=O)c2ccc3[nH]ccc3c2)C[C@@H]1C	0	Train
TNKS1_ML_0110	N#C/C(=C/c1ccc(C#N)cc1)c1nc2ncccc2[nH]1	0	Train
TNKS1_ML_0111	C[C@H]1CCC[C@@H](NC(=O)C(=O)Nc2ccc3c(c2)CCC3)C1	0	Train
TNKS1_ML_0112	Cc1cc(/C=N\N=C(/[O-])[C@@H]2COc3ccccc3O2)c(C)n1-c1ccc(Br)cc1	0	Train
TNKS1_ML_0113	COc1ccc2c(c1)CC1(CCN([C@@H]3CCN(Cc4nc5c(c(=O)[nH]4)COCC5)C3=O)CC1)C2=O	1	Train
TNKS1_ML_0114	C[C@@H]1C[NH+](CCC(=O)NCc2ccc3[nH+]cccc3c2)Cc2cc(Cl)ccc2O1	0	Train
TNKS1_ML_0115	O=C(CCc1c[nH]c2ccccc12)N(Cc1nc2c(c(=O)[nH]1)COCC2)Cc1cccs1	1	Train
TNKS1_ML_0116	C(=C1/CCCC2=C1Nc1ncnn1[C@H]2c1ccccc1)\c1ccccc1	0	Train
TNKS1_ML_0117	Clc1cc2c(cc1NCc1ccc3c(c1)OCO3)OCO2	0	Train
TNKS1_ML_0118	CCCCc1ccc(NC(=O)Cn2nc(-c3cccs3)c3sc(N4CCOCC4)nc3c2=O)cc1	0	Train
TNKS1_ML_0119	CC(=O)N1CCN(Cc2[nH+]c3cc(NC(=O)COc4ccc(Cl)cc4)ccc3n2C)CC1	0	Train
TNKS1_ML_0120	Cc1nn(C)c(C[C@]2(O)CCCC(C)(C)C2)c1Br	0	Train
TNKS1_ML_0121	CC(=O)[C@H]1[C@@H](C(=O)c2cccc([N+](=O)[O-])c2)[C@]2(C(=O)Nc3ccccc32)[C@H]2C=Cc3cc(Cl)ccc3N12	0	Train
TNKS1_ML_0122	Cc1ccc(NC(=O)N2CCC[C@H](C)[C@@H]2CO)c(Br)c1	0	Train
TNKS1_ML_0123	O=C1NN=C2CCCc3c2c2c1cccc2n3CCN1CCCC1	1	Train
TNKS1_ML_0124	Cc1ccc(S(=O)(=O)NNC(=O)c2sc(N3CCOCC3)[nH+]c2C)c(Cl)c1	0	Train
TNKS1_ML_0125	CC[NH+]1C/C(=C/c2cn(C)nc2-c2ccncc2)c2nc3ccccc3c(C(=O)[O-])c2C1	0	Train
TNKS1_ML_0126	CC(C)S(=O)(=O)N1CCc2nc(NC(=O)c3cc(-c4ccc(F)cc4)n[nH]3)sc2C1	0	Train
TNKS1_ML_0127	C[C@H]1C[C@@H](C)CN(c2cc(C(=O)[O-])c([N+](=O)[O-])cc2F)C1	0	Train
TNKS1_ML_0128	COc1ccc(CC/N=C/C2=C([O-])c3ccccc3N(Cc3ccc(C)cc3)S2(=O)=O)cc1OC	0	Train
TNKS1_ML_0129	N#Cc1c(Cn2c(=O)n(C[C@H]3CC[C@H](C(=O)Nc4ccncc4)CC3)c(=O)c3ccccc32)cccc1C(F)(F)F	1	Train
TNKS1_ML_0130	CCNc1c(C#N)cnc2c(F)ccc(Br)c12	0	Train
TNKS1_ML_0131	COc1ccc(CN(C[C@@H]2CCCO2)[C@H](c2cc3cc(C)ccc3[nH]c2=O)c2nnnn2C2CCCC2)cc1	0	Train
TNKS1_ML_0132	Cc1nc(-c2cc(Cl)cs2)nc(Cl)c1C	0	Train
TNKS1_ML_0133	O=C(N[C@H]1C[C@H](c2nnc(-c3ccc(OC(F)F)cn3)n2-c2ccccc2F)C1)c1cccc2nccnc12	1	Train
TNKS1_ML_0134	CCOC(=O)C1=C(CSc2cccc[n+]2[O-])NC(=O)N[C@@H]1c1ccc(Cl)cc1	0	Train
TNKS1_ML_0135	Cc1cc(C(F)(F)F)n2nc([C@H]3CCN(C(=O)COc4ccc(-n5nc(C)cc5C)nn4)C3)cc2n1	0	Train
TNKS1_ML_0136	CCOc1ccc(N2C(=S)N(C)C(=O)/C2=C\c2cc(OCC)c(OCCO)cc2Br)cc1	0	Train
TNKS1_ML_0137	CC(C)Cn1c(N)nc2cc(Cl)c(Cl)cc21	0	Train
TNKS1_ML_0138	COC(=O)c1sc(NC(=O)c2cc3n(n2)[C@@H](C(F)(F)F)C[C@H](c2ccc(Cl)c(Cl)c2)N3)c(C(=O)OC)c1C	0	Train
TNKS1_ML_0139	Cc1cc(N2C(=O)C(C)(C)CS2(=O)=O)ccc1S(=O)(=O)NCc1cc[nH+]cc1	0	Train
TNKS1_ML_0140	CC(C)NC(=O)[C@H]1CCCN(c2nc3ncn(CC(=O)NC4CCCC4)c(=O)c3s2)C1	0	Train
TNKS1_ML_0141	CN(c1ccc(N)c(C(=O)[O-])c1)C1CCCC1	0	Train

TNKS1_ML_0142	CCn1c(SCc2cc(=O)n3cc(C)ccc3n2)nc2cc(S(=O)(=O)N3CCOCC3)ccc21	0	Train
TNKS1_ML_0143	Cc1noc(C)c1-c1ccc(C(=O)NNC(=O)c2ccc(Br)cc2)s1	0	Train
TNKS1_ML_0144	Cn1c(=O)c2cc(C(=O)NCc3cn(Cc4ccccc4)nc3-c3ccccc3)cnc2n(C)c1=O	0	Train
TNKS1_ML_0145	CN(C(=O)N1CCCC1)C1C[C@@H]2CC[C@H](C1)[NH2+]2	0	Train
TNKS1_ML_0146	CCc1nc2sc([C@H](c3cc(OC)c(OC)c(OC)c3)[NH+]3CCN(c4ccc(F)cc4)CC3)c([O-])n2n1	0	Train
TNKS1_ML_0147	C#Cc1cccc(NC(=O)CCc2nc3cc(S(=O)(=O)N4CCOCC4)ccc3n2C)c1	0	Train
TNKS1_ML_0148	O=C1NC(c2ccc(OCc3ccccc3)cc2)Nc2ccccc21	1	Train
TNKS1_ML_0149	COc1ccc(C)cc1N1CC2(CCCC2)NC[C@@H]1C	0	Train
TNKS1_ML_0150	O=S(=O)([N-]c1cccc(-c2nnnn2C2CC2)c1)c1c(F)cc(F)cc1Br	0	Train
TNKS1_ML_0151	O/N=C1/CCN(Cc2ccc(O)cc2)c2ccccc21	0	Train
TNKS1_ML_0152	CN[C@H]1CCCc2nc([C@@H]3CCOc4ccccc43)sc21	0	Train
TNKS1_ML_0153	O=C(Nc1ccc(N2CCOCC2)c(S(=O)(=O)N2CCOCC2)c1)c1ccccc1-c1ccccc1	0	Train
TNKS1_ML_0154	CN(C)C(=O)c1ccccc1NC(=O)N1CC2C[C@H]3CC1C[C@@H](C2)C3	0	Train
TNKS1_ML_0155	C=C1C(C)(C)[C@H]2CC[C@]1(C(=O)Nc1cc(C)on1)C2	0	Train
TNKS1_ML_0156	CC(C)[C@H](C#N)NC(=O)c1ccc(Br)cc1O	0	Train
TNKS1_ML_0157	Cc1ccc(Nc2ccc(S(C)(=O)=O)cc2[N+](=O)[O-])cc1F	0	Train
TNKS1_ML_0158	Cn1c(C(C)(C)C)nc2cc(NC(=O)c3ccco3)ccc21	0	Train
TNKS1_ML_0159	Cn1c(Cn2c[nH+]c3ccccc32)nnc1SCC(=O)CC(=O)Nc1ccccc1	0	Train
TNKS1_ML_0160	CCc1noc(CC)c1CC(=O)N[C@@H]1CCC[C@@H](C)[C@@H]1C	0	Train
TNKS1_ML_0161	CN(C)[C@H]1CN(c2nccc(C3CCCC3)n2)C[C@@H]1O	0	Train
TNKS1_ML_0162	Cc1ccc(-c2onc(N)c2Br)cc1C	0	Train
TNKS1_ML_0163	Cc1cccc2c(O)nc(-c3ccc([N+](=O)[O-])cc3)nc12	1	Train
TNKS1_ML_0164	CC(C)(C)NC(=O)[C@]1(C)Cn2nc(-c3cccs3)cc2C(=O)N1c1ccc2c(c1)OCCO2	0	Train
TNKS1_ML_0165	Cn1c(=O)c2c(ncn2CC(=O)Nc2nnc(SCc3ccccc3F)s2)n(C)c1=O	0	Train
TNKS1_ML_0166	[H]/N=C(\S[C@@H]1CC(=O)N(c2cccc(Br)c2)C1=O)N1N=C(c2ccccc2)C[C@@H]1c1ccccc1	0	Train
TNKS1_ML_0167	Cc1cc(N)cnc1N1CCN2CCC[C@@H]2C1	0	Train
TNKS1_ML_0168	Cc1ccccc1N1CC[NH+]([C@@H]2CCCN(C(=O)c3cc(-c4ccsc4)n[nH]3)C2)CC1	0	Train
TNKS1_ML_0169	COc1ccc(C2=NN(C(=O)CN3C(=O)N[C@@]4(CCCc5ccccc54)C3=O)[C@@H](c3ccco3)C2)cc1	0	Train
TNKS1_ML_0170	O=C(NCc1cccnc1)c1cn(-c2nc(CNc3cccnc3)c3ccccn23)cn1	0	Train
TNKS1_ML_0171	O=C(Cn1cnc2c(Cl)cc(Cl)cc2c1=O)Nc1cccc(S(=O)(=O)N2CCOCC2)c1	0	Train
TNKS1_ML_0172	COc1cccc2c(O)nc(-c3ccc(C(F)(F)F)cc3)nc12	1	Train
TNKS1_ML_0173	O=C(Nc1ccc(C(F)(F)F)cc1)c1ccc(NC(=O)N2CCN(C(=O)c3cc(Cc4n[nH]c(=O)c5ccccc45)ccc3F)CC2)cc1	1	Train
TNKS1_ML_0174	C[C@@H]1CC[C@@H](O)[C@H](N2CCO[C@H](C)C2)C1	0	Train
TNKS1_ML_0175	CC1(C)Cc2c(C#N)c(N3CCN(C(=O)C(=O)NCc4ccccc4)CC3)nc(-c3ccco3)c2CO1	0	Train
TNKS1_ML_0176	COC(=O)[C@@H]1[C@H](c2ccc(OC)c(OC)c2)[NH2+][C@H](c2ccc(OC)c(OC)c2)[C@@H](C(=O)OC)S1(=O)=O	0	Train
TNKS1_ML_0177	Cc1nn2c(nc(N3CCN(Cc4ccsc4)CC3)c3ccccc32)c1Cl	0	Train
TNKS1_ML_0178	O=C(C1=C([O-])C(=O)N(CC[NH+]2CCCCC2)[C@H]1c1cccc(Cl)c1)c1ccco1	0	Train

TNKS1_ML_0179	N#Cc1cccc2c1C1(CCN(c3nc4c(c(=O)[nH]3)CCCC4)CC1)CN2	1	Train
TNKS1_ML_0180	COc1ccc(-c2noc(CSc3nnc(C)n3-c3ccc(C)cc3)n2)cc1	1	Train
TNKS1_ML_0181	CC(C)(C)c1nc(C(=O)N2CCN(C(C)(C)C)CC2)n[nH]1	0	Train
TNKS1_ML_0182	O=C([O-])C[C@@H]1CCN(C(=O)N2CCn3cc[nH+]c3C2)C1	0	Train
TNKS1_ML_0183	CC(=O)c1cccc(NS(=O)(=O)c2cc(-c3nc(C)c(C(=O)N4CCCC4)s3)n(C)c2C)c1	0	Train
TNKS1_ML_0184	NCc1ccn(-c2cc(Cl)cc(Cl)c2)n1	0	Train
TNKS1_ML_0185	CCOc1cc(/C=C2\C(=O)NC(=S)N(c3ccccc3F)C2=O)ccc1OCC(=O)OC	0	Train
TNKS1_ML_0186	Cc1cc(/N=C/c2sc3ccccc3c2O)no1	0	Train
TNKS1_ML_0187	CCN(CC)C(=O)CN1CCN(C(=O)c2c[n+](C(C)C)cc3c(O)n(-c4ccccc4)nc23)CC1	0	Train
TNKS1_ML_0188	C[C@H]1CCCCN1S(=O)(=O)N1CCCCC[C@@H]1N	0	Train
TNKS1_ML_0189	CCn1c(NCCn2ccnc2)nc2sc3c(c2c1=O)CCCC3	0	Train
TNKS1_ML_0190	C/C(=N\c1ccc2c(c1)oc1ccccc12)c1c(O)cc(C)oc1=O	0	Train
TNKS1_ML_0191	CCOc1cc([C@@H]2c3c(-c4ccc(C)cc4)n[nH]c3C(=O)N2CC[NH+]2CCOCC2)ccc1O	0	Train
TNKS1_ML_0192	CS(=O)(=O)CC[C@H](C(=O)NC[C@@H]1COc2ccccc2C1)N1C(=O)c2ccccc2C1=O	0	Train
TNKS1_ML_0193	COc1cc(C(=O)Nc2ccccc2C(=O)N2C[C@@H](C)O[C@H](C)C2)cc(OC)c1OC	0	Train
TNKS1_ML_0194	N#Cc1c(Cn2c(=O)n(C[C@H]3CC[C@H](C(=O)Nc4ccncc4)CC3)c(=O)c3c(Cl)cccc32)cccc1C(F)(F)F	1	Train
TNKS1_ML_0195	C=CCn1c(SCC(=O)NC2(C#N)CCCCCC2)nnc1[C@@H]1CCS(=O)(=O)C1	0	Train
TNKS1_ML_0196	Cn1c([C@@H]2CNCC3(CCCC3)O2)nc2ccccc21	0	Train
TNKS1_ML_0197	CN(c1ccccc1)c1nc(Cl)nc2c1cnn2C	0	Train
TNKS1_ML_0198	O=C(c1ccc(F)cc1)C1CCN([C@H]2CCN(Cc3nc4c(c(=O)[nH]3)COCC4)C2=O)CC1	1	Train
TNKS1_ML_0199	CS(=O)(=O)N1CCC[C@H](C(=O)N/N=C/c2cc(Br)ccc2O)C1	0	Train
TNKS1_ML_0200	C[C@H](c1nc2ccccc2c(N2CCOCC2)[nH+]1)N1CCN(S(=O)(=O)c2ccc(-n3cccn3)cc2)CC1	0	Train
TNKS1_ML_0201	O=C(N[C@@H](Cc1c[nH+]cn1C(=O)OCC1c2ccccc2-c2ccccc21)C(=O)[O-])OCC1c2ccccc2-c2ccccc21	0	Train
TNKS1_ML_0202	C[C@H]1C[C@H](C)CCN1C(=O)NC(=O)CC(C)(C)C(=O)O	0	Train
TNKS1_ML_0203	Cc1cc2c(=O)[nH]c(-c3ccc(C(C)(C)O)cc3)nn2n1	1	Train
TNKS1_ML_0204	C[C@@H]1CCC[C@@H](C)N1C(=O)Nc1c[nH]c2ccccc12	0	Train
TNKS1_ML_0205	COCCN1C(=O)C(O)=C(C(=O)c2sc(C)nc2C)[C@H]1c1cccc2ccccc12	0	Train
TNKS1_ML_0206	[NH3+]N[C@H](c1ccc(Cl)c(Cl)c1)[C@H]1COc2ccccc2O1	0	Train
TNKS1_ML_0207	Cc1ccc(C)c(NC(=O)/C(C#N)=C\c2c(C)[nH]c3ccccc23)c1	0	Train
TNKS1_ML_0208	O=C(NCCS(=O)(=O)N1CCN(C(=O)c2ccco2)CC1)c1ccc(-c2ccccc2)cc1	0	Train
TNKS1_ML_0209	Cc1cccc2c1c(=O)n(C[C@H]1CC[C@H](C(=O)Nc3ccncc3)CC1)c(=O)n2Cc1ccccc1C#N	1	Train
TNKS1_ML_0210	CS(=O)(=O)Nc1ccc(C(=O)CSc2nnc(COc3ccccc3Cl)n2C2CC2)cc1	0	Train
TNKS1_ML_0211	C[C@@H]1CCCN(S(=O)(=O)c2cc(C(=O)Nc3ccc4c(c3)OCCCO4)ccc2Cl)C1	0	Train
TNKS1_ML_0212	CC[C@H]1C(=O)N[C@@H](C)C(=O)N1c1cc(Cl)cc(Cl)c1	0	Train
TNKS1_ML_0213	Cc1ccc(CNc2c3c(nc4nc(C)nn24)CCCC3)cc1	0	Train
TNKS1_ML_0214	Nc1cc(-c2nc3cc(F)ccc3n2C2CC2)ccn1	0	Train
TNKS1_ML_0215	Cc1cc(C)cc(Nc2c(C)c(C)nc3ncnn23)c1	0	Train
TNKS1_ML_0216	C[C@@H](Sc1ccccn1)C(=O)Nc1ccc(S(=O)(=O)N2CCCC2)	0	Train

	cc1		
TNKS1_ML_0217	Cc1cc(-c2nc3ccc(Cl)cc3[nH]2)sc1Br	0	Train
TNKS1_ML_0218	O=C1[C@H]2Cc3c([nH]c4ccccc34)[C@H](c3ccccc3)N2C(=O)CN1/N=C/c1ccccc1	0	Train
TNKS1_ML_0219	COc1ccc(CC(=O)NC[C@H](c2cccnc2)S(=O)(=O)c2ccccc2)cc1OC	0	Train
TNKS1_ML_0220	CC1(C)C[C@@H](Nc2ccc(C#N)cc2Br)C(C)(C)O1	0	Train
TNKS1_ML_0221	Cc1ccc(NC(=O)c2cc(F)cc3nc[nH]c23)c(C)c1	0	Train
TNKS1_ML_0222	CNc1nc([C@H]2CCCN(S(=O)(=O)c3ccc(C)c(F)c3)C2)[nH+]c2c1C[NH+](C)CC2	0	Train
TNKS1_ML_0223	CC1(C)CS(=O)(=O)N(c2cccc(C(=O)Nc3nc4ccc(S(C)(=O)=O)cc4s3)c2)C1=O	0	Train
TNKS1_ML_0224	COc1ccc2c(C3CCN(CC(=O)NCc4nc5c(c(=O)[nH]4)COCC5)CC3)n[nH]c2c1	1	Train
TNKS1_ML_0225	O=C([O-])c1ccc(Cl)c(-c2ccc(/C=N\NC(=O)c3ccc(Br)cc3)o2)c1	0	Train
TNKS1_ML_0226	Cc1ccc(S(=O)(=O)NC(=O)c2cccc3c2OCCO3)c(C)c1	0	Train
TNKS1_ML_0227	CO/C([O-])=C1/C(C)=NC(O)=[NH+][C@@H]1c1cn(-c2ccccc2)nc1-c1cccs1	0	Train
TNKS1_ML_0228	C[C@@H]1C[C@H]1c1nc2c(c(NN)n1)CCCC2	0	Train
TNKS1_ML_0229	Cc1ccc(Nc2cc(Cl)ncn2)c(Br)c1	0	Train
TNKS1_ML_0230	C[C@@H](O)c1ccc(N2C[C@H]3CCCCN3C[C@H]2C)c(Cl)c1	0	Train
TNKS1_ML_0231	CC[NH+](CC)CCN1C(=O)C(O)=C(C(=O)c2sc(C)nc2C)[C@H]1c1ccc(O)cc1	0	Train
TNKS1_ML_0232	[H]/N=C1C(=C\c2cc(Cl)c(OCCOc3ccccc3CC=C)c(OC)c2)\C(=O)N=C2C=C(C)ON2\1	0	Train
TNKS1_ML_0233	CCOC(=O)c1cc2c(=O)n3ccccc3nc2[n+](C(C)C)c1NC(=O)C(c1ccccc1)c1ccccc1	0	Train
TNKS1_ML_0234	Cc1cc(C(=O)NCC(=O)Nc2nc(CN3CCCCCC3)cs2)c(C)o1	0	Train
TNKS1_ML_0235	[H]/N=C1C(=C\c2cc(C)n(-c3ccccc3OCC)c2C)\C(=O)N=C2SN=C(S(C)(=O)=O)N2\1	0	Train
TNKS1_ML_0236	N#Cc1ccc(O[C@H]2CC[C@H](NC(=O)CCSc3nc4ccccc4c(=O)[nH]3)CC2)cc1Cl	1	Train
TNKS1_ML_0237	Cc1cc(C)c(C(=O)NC2(C#N)CCCCC2)c(C)c1	0	Train
TNKS1_ML_0238	O=C(CCSc1nc2ccccc2c(=O)[nH]1)N[C@H]1CC[C@H](Oc2ccncc2)CC1	1	Train
TNKS1_ML_0239	CC[NH+]1CCN(c2nc3c(c(=O)n(C)c(=O)n3C)n2C[C@@H](O)COc2ccc(C)c(C)c2)CC1	0	Train
TNKS1_ML_0240	Nc1ccc(-c2ccc([C@@H]3CCCN(C(=O)COc4ccc(F)cc4)C3)nc2)cn1	0	Train
TNKS1_ML_0241	CO[C@]1(C)CCC[C@@]2(C)C[C@H]3OC(=O)[C@H](C[NH+]4CCN(c5ccc([N+](=O)[O-])cc5)CC4)[C@H]3C[C@@]21O	0	Train
TNKS1_ML_0242	[H]/N=C1C(=C/c2cc(C)n(-c3cc(Cl)ccc3OC)c2C)/C(=O)N=C2SC(S(=O)(=O)CC)=NN2/1	0	Train
TNKS1_ML_0243	CC1CCC(CO)(NC(=O)N2CCCCCC2)CC1	0	Train
TNKS1_ML_0244	CN(C)[C@@H](C#N)c1csc2ccccc12	0	Train
TNKS1_ML_0245	O=S(=O)(c1ccc(F)c(/C(O)=[NH+]/C[C@H]2COc3ccccc3O2)c1)N1CCOCC1	0	Train
TNKS1_ML_0246	Clc1ccc2nsnc2c1N[C@@H]1CCSC1	0	Train
TNKS1_ML_0247	O=C1[NH+]=c2ccccc2=C1N[N-]C1=NC(=O)[C@@H](CC(=O)c2ccc(Cl)cc2)S1	0	Train
TNKS1_ML_0248	C=CCN1C(=O)COc2ccc(-c3nc(/N=C(\[O-])c4cccs4)sc3C)cc21	0	Train
TNKS1_ML_0249	COC(=O)c1c(NC(=O)Cn2nc(C(C)(C)C)ccc2=O)sc2c1CC[C@@H](C)C2	0	Train

TNKS1_ML_0250	[O-]c1ccc(/N=N\c2ccccc2)c(O)c1	0	Train
TNKS1_ML_0251	Cc1cc(NC(=O)C[NH+](C)CC(=O)N(C)[C@]2(c3ccccc3Cl)CCCCC2=O)no1	0	Train
TNKS1_ML_0252	Cc1cc(C(=O)N2CCN(C(=O)Nc3ccc(CC(=O)N4CCCC4)cc3)CC2)c(C)o1	0	Train
TNKS1_ML_0253	CCc1nn(C)c(C(=O)N2CC[NH+](C)[C@@H](CC)C2)c1N	0	Train
TNKS1_ML_0254	Cc1ncc([N+](=O)[O-])n1CCSc1nc2c(c(=O)n(C)c(=O)n2C)n1Cc1ccc(Br)cc1	0	Train
TNKS1_ML_0255	O=C(CCSc1nc2ccccc2c(=O)[nH]1)N[C@H]1CC[C@H](c2nnc(-c3ccccc3)o2)CC1	1	Train
TNKS1_ML_0256	O=c1ncn(C2CCCCC2)c2snnc12	0	Train
TNKS1_ML_0257	CC(C)(CC(=O)NC(=O)N1CCCC1(C)C)C(=O)O	0	Train
TNKS1_ML_0258	COc1ccc(OC)c(S(=O)(=O)NC[C@H](c2ccc[nH+]c2)S(=O)(=O)c2ccc(Cl)cc2)c1	0	Train
TNKS1_ML_0259	O=C(Nc1ccccc1F)c1ccc(NC(=O)N2CCN(C(=O)c3cc(Cc4n[nH]c(=O)c5ccccc45)ccc3F)CC2)cc1	1	Train
TNKS1_ML_0260	O=C1N(CCO)c2cccc(Cl)c2C12CCN(c1nc3c(c(=O)[nH]1)CCCC3)CC2	1	Train
TNKS1_ML_0261	COc1cc(/C=C2/Oc3c(CN4CCN(c5ccccn5)CC4)c(O)cc(C)c3C2=O)cc(OC)c1OC	0	Train
TNKS1_ML_0262	CC(=O)N1CCN(c2cc3c(cc2F)c(=O)c(C(=O)Nc2cc(C(C)(C)C)no2)cn3C2CC2)CC1	0	Train
TNKS1_ML_0263	Cc1ccccc1Cn1c(=O)c(C(=O)NCCc2cccc[nH+]2)nn(-c2ccc(Cl)cc2)c1=O	0	Train
TNKS1_ML_0264	O=c1[nH]c(CN(Cc2cccs2)S(=O)(=O)CCc2ccccc2)nc2c1COCC2	1	Train
TNKS1_ML_0265	Cc1nn(-c2ccc(F)cc2)c(Cl)c1C(=O)Nc1cc2c(cc1Cl)OCCO2	0	Train
TNKS1_ML_0266	Cc1ccc(S(=O)(=O)N2CCC[C@H](C(=O)Nc3nnc(Cc4ccc(S(C)(=O)=O)cc4)o3)C2)cc1	0	Train
TNKS1_ML_0267	CC(C)(CO)CCCNC(=O)NC[C@H]1C[NH+]2CCCC[C@@H]2CO1	0	Train
TNKS1_ML_0268	Cc1cccc(OCCCn2cc(/C=C3/C(=[NH2+])N4C(=NC3=O)S[NH+]=C4S(C)(=O)=O)c3ccccc32)c1	0	Train
TNKS1_ML_0269	Cc1nc2c(C)cccn2c1/C(O)=C1/C(=O)C(=O)N(CC[NH+]2CCOCC2)[C@@H]1c1ccco1	0	Train
TNKS1_ML_0270	Cc1nnc(SCC(=O)Nc2cc(C(F)(F)F)ccc2-n2cncn2)n1Cc1ccccc1	0	Train
TNKS1_ML_0271	[NH3+]CC[C@@H]1CN(c2nc3ccccn3n2)c2ccccc21	0	Train
TNKS1_ML_0272	NNc1cncc(N[C@@H]2C[C@H]2c2ccccc2)n1	0	Train
TNKS1_ML_0273	COc1ccc(S(=O)(=O)NC[C@@H](c2ccc[nH+]c2)S(=O)(=O)c2ccc(Cl)cc2)cc1OC	0	Train
TNKS1_ML_0274	O=C(CCc1ccncc1)N(Cc1nc2c(c(=O)[nH]1)COCC2)Cc1cccs1	1	Train
TNKS1_ML_0275	[O-]c1n[nH+]c(-c2ccccc2)cc1N1CCCCC1	0	Train
TNKS1_ML_0276	Cc1coc2c(C)c3oc(=O)c(CC(=O)NC(C)C)c(C)c3cc12	0	Train
TNKS1_ML_0277	Cc1ccc(CN2C(=O)N/C(=C/c3ccc(N4CCOCC4)cc3C)C2=O)cc1	0	Train
TNKS1_ML_0278	O=C1NC(c2ccccc2OCc2ccccc2)Nc2ncccc21	1	Train
TNKS1_ML_0279	CC(=O)c1ccc(N2CCN(C(=O)CCn3cnc4c([nH]c5ccc(C)cc54)c3=O)CC2)cc1	0	Train
TNKS1_ML_0280	C[C@H](Nc1nccc(Oc2ccc(F)cc2)n1)[C@H](O)c1ccc(F)cc1	0	Train
TNKS1_ML_0281	[H]/N=C1C(=C\c2cn(CCCOc3ccccc3)c3ccccc23)/C(=O)N=C2SC(c3cccnc3)=NN2/1	0	Train
TNKS1_ML_0282	CNc1cc([C@@H]2CCC[NH+](Cc3ccc4c(c3)CCCN4C)C2)nc(CN(C)C(C)=O)n1	0	Train
TNKS1_ML_0283	CN(C)c1ccc([C@@H]2C3=C(CCCC3=O)N[C@H]3N=NC(O)=C32)cc1	0	Train
TNKS1_ML_0284	Cc1sc2nc(Cn3ccccc3=O)nc(N)c2c1C	0	Train

TNKS1_ML_0285	O=C(Nc1cccc([N+](=O)[O-])c1)c1csc(-c2ccccc2)n1	0	Train
TNKS1_ML_0286	Cc1cc(C)c([N+](=O)[O-])cc1[N-]S(=O)(=O)c1ccc(Cl)c(C(F)(F)F)c1	0	Train
TNKS1_ML_0287	CCO[C@H]1CCCN(S(=O)(=O)c2c(Br)cc3[nH]c(=O)oc3c2Cl)C1	0	Train
TNKS1_ML_0288	O=C(O)[C@]1([C@H]2CCCC[C@@H]2O)COc2ccccc2C1	0	Train
TNKS1_ML_0289	Cc1cc(Cl)c(S(=O)(=O)N2C[C@@H](C)S[C@H](C)C2)cc1N	0	Train
TNKS1_ML_0290	Cc1nc2sc([C@@H](c3ccccc3Cl)[NH+]3CCN(c4ccccc4F)CC3)c([O-])n2n1	0	Train
TNKS1_ML_0291	COc1ccc(S(=O)(=O)N2CCOCC2)cc1NC(=O)[C@@H]1Cc2ccccc2CN1C(=O)c1ccco1	0	Train
TNKS1_ML_0292	Cc1ccnc2nc(C(=O)Nc3nc(-c4ccc(Cl)cc4Cl)cs3)nn12	0	Train
TNKS1_ML_0293	O=C(NCCc1cn2ccccc2[nH+]1)C(=O)Nc1ccccc1OC(F)(F)F	0	Train
TNKS1_ML_0294	CCN(C(=O)N1CCC(C(=O)c2ccc3ncn(C)c3c2)CC1)c1ccc(C(=O)O)cc1	1	Train
TNKS1_ML_0295	O=c1[nH][nH]c(=O)c2cc(OCc3ccsc3)ccc12	1	Train
TNKS1_ML_0296	CC[C@@H]1CC[C@H](Nc2cnc(C(=O)[O-])cn2)[C@@H]1C	0	Train
TNKS1_ML_0297	Cc1nn(-c2ccccc2)c(Cl)c1CN1C(=O)N[C@]2(CCCc3sccc32)C1=O	0	Train
TNKS1_ML_0298	CC[C@]1(c2ccccc2)NC(=O)N(NC(=O)CN[C@@H](C)c2ccc(Cl)cc2Cl)C1=O	0	Train
TNKS1_ML_0299	Cc1cc2c(cc1C)C(=O)C[C@H](c1cc(C(=O)[O-])cc3c1OCOC3)O2	0	Train
TNKS1_ML_0300	CS(=O)(=O)N1CCC(C2Cc3[nH]c(=O)c4ccccc4c3CO2)C1	1	Train
TNKS1_ML_0301	C[C@H]1Oc2ccccc2O[C@H]1C(=O)Nc1cc2c(cc1Cl)OCCCO2	0	Train
TNKS1_ML_0302	Cn1c(=O)c2c(ncn2CC(=O)N(Cc2cccc[nH+]2)c2nc3ccccc3s2)n(C)c1=O	0	Train
TNKS1_ML_0303	Cc1ccc(/C=N\NC(=O)c2cc3ccccc3o2)o1	0	Train
TNKS1_ML_0304	Cc1cccn2cc(CSc3ncn[nH]3)nc12	0	Train
TNKS1_ML_0305	Cc1cccc(NC(=O)c2ccc3snnc3c2)c1C	0	Train
TNKS1_ML_0306	CC[C@@H](Oc1cccc(C)c1)C(=O)Nc1ccc(C(=O)N2CCC(C)CC2)cc1	0	Train
TNKS1_ML_0307	Cc1sc2ncn3c(SCC(=O)Nc4ccccc4N4CCOCC4)nnc3c2c1C	0	Train
TNKS1_ML_0308	CC(C)CN(C(=O)c1csc(-c2ccsc2)n1)c1c(N)n(CC(C)C)c(=O)[nH]c1=O	0	Train
TNKS1_ML_0309	COc1cc(/C=C/C(=O)N(C)CC(=O)Nc2c(C)cc(C)cc2C)ccc1OCc1c(C)noc1C	0	Train
TNKS1_ML_0310	Nc1cccc2oc(CSc3ccc(Br)cn3)nc12	0	Train
TNKS1_ML_0311	Cc1cc[nH+]cc1NC(=O)N1C[C@H](C)O[C@@H](c2ccc(F)cc2)C1	0	Train
TNKS1_ML_0312	COc1ccc(C2(CNC(=O)c3ccc(NC(=O)c4cnc(C)o4)cc3Cl)CCOCC2)cc1	1	Train
TNKS1_ML_0313	O=C(/C=C/c1ccco1)C1=C(O)C(=O)N(CCCn2cc[nH+]c2)[C@@H]1c1ccc(Cl)c(Cl)c1	0	Train
TNKS1_ML_0314	C[C@@H](Sc1nc2sc3c(c2c(=O)n1-c1ccccc1C(F)(F)F)CCCC3)C(=O)NC(N)=O	0	Train
TNKS1_ML_0315	Cc1nn(-c2ccccc2)c2c1[C@H](c1ccccc1)c1c(ncn(CCCN3CCOCC3)c1=[NH2+])O2	0	Train
TNKS1_ML_0316	COc1ccc(-c2ncc(C[NH+]3CCc4nc(-c5cccnc5)nc([O-])c4C3)cn2)cc1	0	Train
TNKS1_ML_0317	Cc1c(N/C=C\C(=O)c2ccco2)c(=O)n(-c2ccccc2)n1C	0	Train
TNKS1_ML_0318	CS(=O)(=O)N1CCN(Cc2c([O-])c(C[NH+]3CCN(c4cccc(Cl)c4)CC3)coc2=O)CC1	0	Train
TNKS1_ML_0319	C[C@H](c1ccccc1)N1C(=O)/C(=C\c2c(NCCOCCO)nc3cccc	0	Train

	n3c2=O)SC1=S		
TNKS1_ML_0320	C[C@@H](C(=O)NNC(=O)c1ccc(Cn2nnc(-c3ccccc3)n2)cc1)n1cc[nH+]c1	0	Train
TNKS1_ML_0321	COc1ccc(S(=O)(=O)NCCS(=O)(=O)N2CCN(c3ccc(F)cc3)CC2)cc1	0	Train
TNKS1_ML_0322	CNC(=O)CN(C)C(=O)Cc1cn(-c2ccccc2)nc1-c1ccc(Cl)cc1	0	Train
TNKS1_ML_0323	COc1cc2cc(C[NH+](Cc3ccc(C)cc3)Cc3nnnn3C[C@@H]3CCCO3)c(=O)[nH]c2cc1OC	0	Train
TNKS1_ML_0324	Cc1cccc(C(=O)N2[C@@H](C(=O)NCc3cc[nH+]cc3)COC23CCN(C(=O)Cc2ccccc2)CC3)c1	0	Train
TNKS1_ML_0325	COc1cccc2c(=O)[nH]c(-c3ccc(F)cc3)nc12	1	Train
TNKS1_ML_0326	Cc1ccsc1C(=O)OCC(=O)Nc1cccc(S(=O)(=O)N2CCOCC2)c1	0	Train
TNKS1_ML_0327	COC(=O)c1ccc(S(=O)(=O)Nc2cc(-c3ccc(Br)s3)n[nH]2)cc1	0	Train
TNKS1_ML_0328	CN1CCCc2c1nc(N1CCC3(CC1)C(=O)N(C)c1cc(OCCN4CCOCC4)cc(F)c13)[nH]c2=O	1	Train
TNKS1_ML_0329	C[C@@H](C1CC1)N(C)C(=O)N[C@@H]1CCCCC[C@@H]1C(=O)[O-]	0	Train
TNKS1_ML_0330	COc1cc(OC)c([C@@H]2C(C#N)=C(N)Oc3cc(C)n(CC[NH+](C)C)c(=O)c32)cc1OC	0	Train
TNKS1_ML_0331	CCc1nsc(N2C[C@H](C)N[C@H](C)C2)n1	0	Train
TNKS1_ML_0332	CCn1c(C[NH+]2CCN(C(C)=O)CC2)nc2cc(NC(=O)COc3ccccc3)ccc21	0	Train
TNKS1_ML_0333	CNC(=O)[C@H]1CN(CC(=O)N2CCN(S(=O)(=O)c3ccc4c(c3)CCC4)CC2)c2ccccc2O1	0	Train
TNKS1_ML_0334	COc1cccc(N2CCN(c3nc4c(c(=O)[nH]3)CSCC4)CC2)c1	1	Train
TNKS1_ML_0335	N#CC1=C(N)C(C#N)(C#N)[C@H](c2ccc(I)cc2)[C@@H]2CCC=C12	0	Train
TNKS1_ML_0336	O=C1/C(=C/c2ccc(O)cc2)COc2cc(O)ccc21	0	Train
TNKS1_ML_0337	C[C@@H]1CN2CCCC[C@@H]2CN1Cc1nc(N)ccc1Cl	0	Train
TNKS1_ML_0338	[H]/N=C1C(=C/c2cc(C)n(-c3ccc(Cl)c([N+](=O)[O-])c3)c2C)/C(=O)N=C2SC(CCCCC)=NN2/1	0	Train
TNKS1_ML_0339	C[C@@H](n1c(=O)[nH][nH]c1=S)C12CC3CC(CC(C3)C1)C2	0	Train
TNKS1_ML_0340	O=C(CCc1nc2ccccc2c(=O)[nH]1)Nc1ccc(C(=O)Nc2cccc3cccnc23)cc1	1	Train
TNKS1_ML_0341	Cc1nc2sc([C@H](c3ccc(Cl)c(Cl)c3)N3CCOCC3)c(O)n2n1	0	Train
TNKS1_ML_0342	O=C(NCCN1C(=O)S/C(=C/c2cccnc2)C1=O)c1cc2c(=O)n3ccccc3[nH+]c2s1	0	Train
TNKS1_ML_0343	Cc1cc(Cl)ccc1[N-]S(=O)(=O)c1ccc2c(c1)CCC(=O)N2	0	Train
TNKS1_ML_0344	CC(C)(O)c1ccc(-c2nc3c(F)cc(F)cc3c(=O)[nH]2)cc1	1	Train
TNKS1_ML_0345	Cc1ccc(S(=O)(=O)CNC(=O)[C@@H](c2ccco2)N(C(=O)CC(C)C)c2cccc(Cl)c2)cc1	0	Train
TNKS1_ML_0346	CCc1onc(C)c1C(=O)N1CCC[C@@H]1c1cc2n(n1)[C@@H](C(F)(F)F)C[C@@H](C)N2	0	Train
TNKS1_ML_0347	Cc1nc(NC(C)C)nc2c1nc(Br)c(=O)n2C1CCN(C(=O)OC(C)(C)C)CC1	0	Train
TNKS1_ML_0348	O=C(N[C@H]1C[C@H](c2nnc(-c3ccncn3)n2-c2ccccc2Cl)C1)c1ccccn1	1	Train
TNKS1_ML_0349	CC1(C)OC(=O)N(c2ccc(-c3cnc(N)c(-c4ccc5nncn5n4)c3)cc2)[C@H]1c1ccccc1	1	Train
TNKS1_ML_0350	COc1ccc(CCN2CC(=O)N3[C@@H](Cc4c([nH]c5ccccc45)[C@@H]3c3ccccc3OC)C2=O)cc1OC	0	Train
TNKS1_ML_0351	CCCc1cc(C(=O)OC)c(NC(=O)/C(C#N)=C/c2cccc(OCc3cccc(Cl)c3)c2)s1	0	Train
TNKS1_ML_0352	CCc1c(Cl)nc(C2CCCC2)nc1Cl	0	Train
TNKS1_ML_0353	Cc1ccc(NC(=O)CN2CCN(S(=O)(=O)c3cccs3)CC2)cc1S(=O)(=O)N(C)C	0	Train

TNKS1_ML_0354	CCCN1C(=O)/C(=C/c2c(C)c(C#N)c(=O)n(CC)c2N2CCN(CCO)CC2)SC1=S	0	Train
TNKS1_ML_0355	CCCCN1C(=O)C2(CCCC2)NC(=O)C[C@H]1C	0	Train
TNKS1_ML_0356	CCCCN1C(=O)/C(=C/c2c(N3CCN(c4ccccc4)CC3)nc3c(C)ccn3c2=O)SC1=S	0	Train
TNKS1_ML_0357	CCCCn1c(N)c(N(C)C(=O)c2sc(-c3cccc(Cl)c3)nc2C)c(=O)[nH]c1=O	0	Train
TNKS1_ML_0358	Cc1ccc(Nc2nc(N/N=C/c3ccc(Br)c([N+](=O)[O-])c3)nc(N3CCOCC3)n2)c(Br)c1	0	Train
TNKS1_ML_0359	Cc1cc2nc(/C(C#N)=C\c3ccc4c(c3)n(C)c(=O)n4C)[nH]c2cc1C	0	Train
TNKS1_ML_0360	COc1ccccc1-c1c2c(nc3sc(C(=O)OC(C)C)c(N)c13)CC[NH+](C)C2	0	Train
TNKS1_ML_0361	c1cn(Cc2ccc3nsnc3c2)c(-c2cc3n(n2)CCCNC3)n1	0	Train
TNKS1_ML_0362	O=C(C1=C([O-])C(=O)N(CCC[NH+]2CCOCC2)[C@H]1c1ccc(O)cc1)c1cc2ccccc2o1	0	Train
TNKS1_ML_0363	[O-]c1c2c(nc3c(F)c(F)c(F)cc13)CCCC2	0	Train
TNKS1_ML_0364	Cc1cccc(NS(=O)(=O)c2ccc(C)c([N-]S(=O)(=O)c3ccc(C)cc3Cl)c2)c1	0	Train
TNKS1_ML_0365	Cc1c[nH]c(CN(C)C(=O)[C@@H]2[C@@H]3C=C[C@@]4(CN(c5ccc(C)c(C)c5)C(=O)[C@H]24)O3)n1	0	Train
TNKS1_ML_0366	CCc1sc2nc(N3CCN(C(=O)Nc4cccc(Cl)c4)CC3)n3ncnc3c2c1C	0	Train
TNKS1_ML_0367	O=C(C1=C([O-])C(=O)N(c2cccc([N+](=O)[O-])c2)[C@@H]1c1ccc(Br)cc1)c1ccccc1	0	Train
TNKS1_ML_0368	O=c1[nH]c(SCCN2CCN(CCCc3nc(-c4ccccc4)no3)CC2)nc2ccccc12	1	Train
TNKS1_ML_0369	C[C@@H](NC(=O)N1CCSC2(CCCCC2)C1)c1cnn(C)c1	0	Train
TNKS1_ML_0370	C[C@H]1CCCC[C@@H]1NC(=O)[C@]1(C)Cn2c(cc3occc32)C(=O)N1CCN(C)C	0	Train
TNKS1_ML_0371	N#C/C(=C\c1cccc(O)c1)c1nc2ccccc2[nH]1	0	Train
TNKS1_ML_0372	CC1(C)C[C@@H](O)c2cc3c(cc2O1)OCO3	0	Train
TNKS1_ML_0373	O=C(CCNS(=O)(=O)c1ccccc1F)N1CCC([NH+]2CCC(Oc3nccs3)CC2)CC1	0	Train
TNKS1_ML_0374	Cc1ccc(N(Cc2nnc(SCC(=O)NCc3ccccc3)n2C)S(C)(=O)=O)cc1	0	Train
TNKS1_ML_0375	[H]/N=C1\[C@H](C(N)=O)[C@H](c2ccccc2)C(C(=O)OCC)=c2s/c(=C\c3ccccc3)c(=O)n21	0	Train
TNKS1_ML_0376	COc1ccc(NC(=O)CCCn2cncn2)cc1NC(=O)c1cccc(F)c1	0	Train
TNKS1_ML_0377	O=C1CCS(=O)(=O)N1c1ccc(S(=O)(=O)NC[C@@H](c2ccc(F)cc2)[NH+]2CCCC2)cc1	0	Train
TNKS1_ML_0378	Cc1cccc(OCC(=O)c2ccc3c(c2)N(Cc2ccc(C(=O)N4CC[NH+](C)CC4)o2)C(=O)CO3)c1	0	Train
TNKS1_ML_0379	CCCCn1c(C[NH+]2CCN(CC(=O)N3CCCCCC3)CC2)nc2cc(S(N)(=O)=O)ccc21	0	Train
TNKS1_ML_0380	CC1=C(C)C[C@@H](C(=O)O)[C@@H](C(=O)Nc2sc3c(c2C(=O)N2CCCCC2)CCCC3)C1	0	Train
TNKS1_ML_0381	COc1ccc(CC[N+]2=C(Nc3ccc(OC)cc3)S[C@@H](C(=O)Nc3ccc(C)cc3C)CC2=O)cc1	0	Train
TNKS1_ML_0382	Cc1nnc([N-]S(=O)(=O)c2ccc3c(c2)CCCN3C(=O)[C@H](C)Oc2ccccc2)s1	0	Train
TNKS1_ML_0383	Cc1ccccc1Cc1c(C(=O)NCCn2ccnc2)c(=O)cc(C)n1Cc1cc[nH+]cc1	0	Train
TNKS1_ML_0384	CCOC(=O)c1c(NC(=O)[C@H](C)n2cc(C)nc2C)sc2c1CC[C@H](C)C2	0	Train
TNKS1_ML_0385	Cc1cc(C(=O)NC2CC2)cc2c1N(C)S(=O)(=O)c1ccccc1-2	0	Train

TNKS1_ML_0386	Cc1ccc(S(=O)(=O)N2CCC(C(=O)N/N=C3\CCc4ccccc43)CC2)cc1	0	Train
TNKS1_ML_0387	O=C1NC2(CCCCC2)C(=O)N1/N=C(\[O-])CN1CCCN(S(=O)(=O)c2ccc(F)cc2)CC1	0	Train
TNKS1_ML_0388	O=C1N[C@@H](Cc2ccc(O)cc2)C(=O)N2C[C@@H](NCc3cnn(-c4cccc(F)c4)c3)C[C@@H]12	0	Train
TNKS1_ML_0389	Cc1cc(/C=C2\C(=[NH2+])N3N=C(S(C)(=O)=O)SC3=NC2=O)c(C)n1-c1cccc(Cl)c1	0	Train
TNKS1_ML_0390	Cc1csc(Cc2nc(N)c3c4c(sc3n2)CCCC4)n1	0	Train
TNKS1_ML_0391	C[C@]1(c2ccc3c(c2)OCO3)NC(=O)N(Cc2cccc(Br)c2)C1=O	0	Train
TNKS1_ML_0392	C[C@@H](c1ccccc1)N1C(=O)N=C([O-])/C(=C/c2cn(-c3ccccc3)nc2-c2ccc(Cl)cc2)C1=O	0	Train
TNKS1_ML_0393	Cc1cccc(-n2nc(C(=O)NCCc3cccc[nH+]3)c(=O)n(Cc3ccc(F)cc3)c2=O)c1	0	Train
TNKS1_ML_0394	Cn1c(=O)c(=O)n(C)c2cc(S(=O)(=O)CCC(=O)Nc3cccc(Cl)c3)ccc21	0	Train
TNKS1_ML_0395	Cc1cc(C)c(NC(=O)CN(C)C(=O)c2cc(S(=O)(=O)N3CCCC3)ccc2C)c(C)c1	0	Train
TNKS1_ML_0396	Nc1ccc(-c2nc(O)c3cccc(O)c3n2)cc1	1	Train
TNKS1_ML_0397	O=C(Nc1cccc2cccnc12)c1ccc(N2C(=O)[C@H]3[C@H]4C=C[C@H](C4)[C@H]3C2=O)cc1	1	Train
TNKS1_ML_0398	COc1cc(/C=C2/C(=O)NC(=O)N(c3cc(C)ccc3C)C2=O)cc(Br)c1O	0	Train
TNKS1_ML_0399	N#C[C@H]1CCC[C@H]1NC(=O)Nc1cccc2ccccc12	0	Train
TNKS1_ML_0400	Cc1nc(Cl)c(N[C@H]2CCC[C@@H]2C)nc1C	0	Train
TNKS1_ML_0401	CCc1cc2oc(=O)cc(CSc3n[nH]c(=O)n3C[C@@H]3CCCO3)c2cc1Cl	0	Train
TNKS1_ML_0402	CC1CCC(O)(C2(C#N)CCC(C)(C)CC2)CC1	0	Train
TNKS1_ML_0403	CC(C)(O)c1ccc(-c2cn3cc(Cl)cc3c(=O)[nH]2)cc1	1	Train
TNKS1_ML_0404	COc1ccc2c(c1)C(CC(=O)N[C@@H](CNC(=O)c1ccccc1N1CCC(=O)NC1=O)C(N)=O)CC2=O	1	Train
TNKS1_ML_0405	CNC(=O)N1C=CC=C[C@@H]1Br	0	Train
TNKS1_ML_0406	Cc1cc(C)c2[nH]c(=O)c([C@H](c3nnnn3C3CCCCC3)[NH+](C)C3CCCCC3)cc2c1	0	Train
TNKS1_ML_0407	C[C@H](OC(=O)[C@]12CCC(=O)N1c1ccccc1S2)C(=O)Nc1cccc(S(=O)(=O)N(C)C)c1	0	Train
TNKS1_ML_0408	CC1CCN(c2ccc(CNC(=O)NCC3(N4CCOCC4)CCCCC3)c[nH+]2)CC1	0	Train
TNKS1_ML_0409	O=C(COc1cccc(NC(=O)c2ccco2)c1)NC[C@@H](c1ccco1)[NH+]1CCCCC1	0	Train
TNKS1_ML_0410	CSC1=[NH+]SC2=NC(=O)/C(=C\c3ccc(OCCOc4ccccc4C)cc3)C(=[NH2+])N21	0	Train
TNKS1_ML_0411	CC(C)c1nnc(NC(=O)c2cc(-c3ccc(Cl)cc3)nn2C)s1	0	Train
TNKS1_ML_0412	C[C@H]1CC[C@@H](C(C)(C)c2ccco2)[C@H](O)C1	0	Train
TNKS1_ML_0413	Cc1cc(N[C@@H]2CCCCC[C@@H]2C)nc(C)n1	0	Train
TNKS1_ML_0414	Cc1c(/C=N\NC(=O)c2csc3c2CCCC3)c(=O)n(-c2ccccc2)n1C	0	Train
TNKS1_ML_0415	COc1ccc(-c2noc(CSc3nnc(C)n3-c3ccccc3)n2)cc1OC	1	Train
TNKS1_ML_0416	COc1ccc(C(=O)C2CCN(C3CCN(Cc4nc5c(c(=O)[nH]4)CCC5)C3=O)CC2)cc1	1	Train
TNKS1_ML_0417	Nc1nc(-c2nc(Cc3ccc(Cl)cc3Cl)no2)cs1	0	Train
TNKS1_ML_0418	COc1ccc(S(=O)(=O)N2CCC(C(=O)N[C@H](C)c3ccc4c(c3)OCCO4)CC2)cc1	0	Train
TNKS1_ML_0419	CC(C)(O)c1ccc(-c2nc3c(Cl)cccc3c(=O)[nH]2)cc1	1	Train
TNKS1_ML_0420	N#C[C@H]1CCC[C@H]1N[C@H]1CCOc2c(Cl)cc(Cl)cc21	0	Train
TNKS1_ML_0421	Nc1ccc(F)c(F)c1N[C@@H]1CCS(=O)(=O)C1	0	Train
TNKS1_ML_0422	O=C([C@@H]1N[C@H](C2=CC3=CC=N[C@@H]3C=C2)NO1)N1CCN(c2ccc(Cl)cc2)CC1	0	Train

TNKS1_ML_0423	O=C(NCCCn1ccnc1)C(=O)NC[C@@H](c1cccnc1)N1CCN(c2ccccc2)CC1	0	Train
TNKS1_ML_0424	Brc1cnc2nc([C@H]3NCCc4sccc43)[nH]c2c1	0	Train
TNKS1_ML_0425	NC(=O)c1ccc(Oc2cccc(C(N)=O)c2)cc1	1	Train
TNKS1_ML_0426	c1cnc2c(CN3CCC[C@@H]3c3ccn[nH]3)cnn2c1	0	Train
TNKS1_ML_0427	CCOc1cc([C@@H]2OCCn3c(-c4ccccc4)c4c(=O)n(C)c(=O)n(C)c4c32)ccc1O	0	Train
TNKS1_ML_0428	COc1ccc([C@@H]2C(=C(O)c3ccc(F)cc3)C(=O)C(=O)N2c2cccc(C(=O)[O-])c2)cc1OC	0	Train
TNKS1_ML_0429	Clc1ccccc1CN(Cc1ccccc1)c1nc[nH+]c2nc[nH]c12	0	Train
TNKS1_ML_0430	CCOc1cccc2cc(-c3cc(=O)oc4c(CN5CCN(C)CC5)c(O)ccc34)c(=O)oc12	0	Train
TNKS1_ML_0431	COc1cccc2c(O)nc(-c3ccc([N+](=O)[O-])cc3)nc12	1	Train
TNKS1_ML_0432	CC1(C)CCC2=C(C1)c1n[nH]c(=O)c3cccc(c13)N2	1	Train
TNKS1_ML_0433	Cc1nn([C@@H](C)C(=O)NCC[NH+]2CCCCCC2)c(=O)c2c(-n3cccc3)n(-c3ccccc3)nc12	0	Train
TNKS1_ML_0434	CCOc1ccc(-c2nnc([C@H]3C[C@H](NC(=O)c4cccc5nc(C)cnc45)C3)n2-c2ccccc2F)nc1	1	Train
TNKS1_ML_0435	O=C(Nc1ccc2oc(-c3ccncc3)nc2c1)c1ccc(Br)o1	0	Train
TNKS1_ML_0436	Clc1ccc(N/N=C2\C[C@H]3C=CC[C@H]23)nn1	0	Train
TNKS1_ML_0437	O=C(NCc1ccc(F)cc1)c1nn(-c2ccc(Cl)cc2)c(=O)n(Cc2ccc[nH+]c2)c1=O	0	Train
TNKS1_ML_0438	O=C(CSc1ncccc1C(=O)N1CCN(c2cccc[nH+]2)CC1)Nc1ccc(Br)cc1	0	Train
TNKS1_ML_0439	CO[C@@H]1CCC[C@H](Oc2cc3c(cc2[NH3+])OCCO3)C1	0	Train
TNKS1_ML_0440	C[C@H]1CCN(C(=O)NC(=O)C[C@@H](C)CC(=O)O)C1	0	Train
TNKS1_ML_0441	N#Cc1ccc(C[NH+]2CCN(CC(=O)Nc3cccc(S(=O)(=O)N4CCCC4)c3)CC2)cc1	0	Train
TNKS1_ML_0442	C[C@H](NC(=O)c1ccc(NC(=O)CSc2nccn2C)cc1)c1ccc(-n2cncn2)cc1	0	Train
TNKS1_ML_0443	Cc1noc(CN[C@H]2CCO[C@@H](C)C2)n1	0	Train
TNKS1_ML_0444	O=c1[nH]c(N2CCN(Cc3ccncc3)CC2)nc2c1CCCC2	1	Train
TNKS1_ML_0445	Cc1ccc2c(=O)[nH]c(-c3ccc([C@](C)(O)CCO)cc3)cn12	1	Train
TNKS1_ML_0446	COc1cc(I)c(C(=O)Nc2cnn(CC[NH+](C)C)c2)cc1OC	0	Train
TNKS1_ML_0447	Cc1nn(C)c(C[C@@]2([NH3+])CCCOCC2)c1Cl	0	Train
TNKS1_ML_0448	O=C([O-])COc1nc(Nc2cc(Cl)cc(Cl)c2)nc2c1C[NH+](Cc1ccccc1)CC2	0	Train
TNKS1_ML_0449	Cc1noc(C)c1S(=O)(=O)[N-]c1ccc2c(c1)N(S(=O)(=O)c1ccc(F)cc1)CCC2	0	Train
TNKS1_ML_0450	Cn1c(=O)c2cc(C(=O)NCC[NH+]3CCN(c4ccccc4)CC3)sc2n(C)c1=O	0	Train
TNKS1_ML_0451	Cc1[nH+]c2ccccn2c1/C(O)=C1/C(=O)C(=O)N(CCCn2cc[nH+]c2)[C@@H]1c1ccc(Cl)cc1	0	Train
TNKS1_ML_0452	Cc1ccc(NS(=O)(=O)c2ccc(Cl)c(N)c2)nc1	0	Train
TNKS1_ML_0453	N#CC1=C(N)OC(c2ccccc2)=C(C#N)[C@@H]1c1ccco1	0	Train
TNKS1_ML_0454	O=C(C1=C([O-])C(=O)N(CCCn2ccnc2)[C@H]1c1cccc(Br)c1)c1cc2ccccc2o1	0	Train
TNKS1_ML_0455	Cc1noc(-c2c(NC(=O)NCc3cccs3)sc3c2CCOC3)n1	0	Train
TNKS1_ML_0456	Cc1cc(=O)[nH]c2cc(-c3ccc(C(=O)O)cc3Cl)ccc12	1	Train
TNKS1_ML_0457	Cc1cnc(Nc2cnc3ccccc3c2)c(Br)c1	0	Train
TNKS1_ML_0458	O=C(C1=C(C(F)(F)F)Nc2ncnn2[C@H]1c1cccc(O)c1)c1ccc(Cl)s1	0	Train
TNKS1_ML_0459	Cc1nonc1CN[C@H]1CCc2ccccc2C1	0	Train
TNKS1_ML_0460	C[C@H]1CN(Cc2ccccc2NC(=O)c2ccc(S(=O)(=O)N3CCCC3)o2)C[C@@H](C)O1	0	Train

TNKS1_ML_0461	COC(=O)[C@@H](C)n1c(SCC(=O)[O-])[nH+]c2cc(I)ccc21	0	Train
TNKS1_ML_0462	CC(C)(C)c1ccc(-c2nc3c(sc4ccccc43)c(=O)[nH]2)cc1	1	Train
TNKS1_ML_0463	CC1(C)CC(=O)C2=C(C1)OC(N)=C(C#N)[C@@H]2c1ccc(C#N)cc1	0	Train
TNKS1_ML_0464	Nc1nnc(SCc2cc(Cl)c3c(c2)OCCCO3)n1C1CC1	0	Train
TNKS1_ML_0465	C[C@H]1CCCN1c1nc2c(s1)CCC[C@@H]2O	0	Train
TNKS1_ML_0466	O=C(/C=C/c1ccco1)C1=C([O-])C(=O)N(CC[NH+]2CCCCC2)[C@H]1c1ccccn1	0	Train
TNKS1_ML_0467	C[C@H](Sc1ccc(S(=O)(=O)N2CCN(C)CC2)cn1)C(=O)NCc1ccc2c(c1)OCO2	0	Train
TNKS1_ML_0468	O=C(CCc1ccccc1)N(Cc1nc2c(c(=O)[nH]1)COCC2)CC1CCCC1	1	Train
TNKS1_ML_0469	CCn1[nH+]c(C)c(CCNC(=O)[C@H](C)C2CCN(S(=O)(=O)c3ccc(Cl)s3)CC2)c1C	0	Train
TNKS1_ML_0470	O=C1OC2(CCN(c3nc4c(c(=O)[nH]3)CCCC4)CC2)c2ccccc21	1	Train
TNKS1_ML_0471	CC(C)[C@@H]1CC[C@H](C)C[C@@]1(O)c1nccn1C	0	Train
TNKS1_ML_0472	COc1ccc(-c2ccc(C#N)c(O)c2)cc1F	0	Train
TNKS1_ML_0473	c1cnn(-c2ccc(-c3cnco3)cc2)c1	0	Train
TNKS1_ML_0474	N#Cc1ccc2c(c1)[nH]c(=O)n2[C@H]1CC[C@H](c2nnc(-c3ccncn3)n2-c2ccccc2Cl)CC1	1	Train
TNKS1_ML_0475	O=C(Nc1ccc(O)c(F)c1)N1CCCCCC1	0	Train
TNKS1_ML_0476	C=CCN1C(=O)[C@]2(C(C(=O)c3ccc(C)o3)=C([O-])C(=O)N2CC[NH+]2CCOCC2)c2ccccc21	0	Train
TNKS1_ML_0477	Cc1nc2c(-c3ccccc3)c(C)nn2c(NCCCN2CCCC2=O)c1C	0	Train
TNKS1_ML_0478	C[C@H]1CC[C@@](O)([C@@H](C)C#N)C[C@@H]1C	0	Train
TNKS1_ML_0479	COc1ccc(N2C(=O)CCS2(=O)=O)cc1S(=O)(=O)Nc1c(C)cccc1C(C)C	0	Train
TNKS1_ML_0480	Cc1ccc(S(=O)(=O)Nc2ccsc2C(=O)[O-])c(Cl)c1	0	Train
TNKS1_ML_0481	O=C(c1cccc(S(=O)(=O)[N-]c2ccccc2F)c1)N1CCC[C@H]1c1ccc2c(c1)OCCCO2	0	Train
TNKS1_ML_0482	C[NH2+][C@H]1c2ccccc2C[C@H]1[NH+]1CCO[C@H]2CCCC[C@@H]21	0	Train
TNKS1_ML_0483	O=C(NC[C@@H](c1ccco1)[NH+]1CCCCC1)Nc1cccc(Cn2cccnc2=O)c1	0	Train
TNKS1_ML_0484	O=C(CS[C@H]1NN=C(C2=c3ccccc3=[NH+]C2)N1C1CC1)c1ccc(N2CCCC2=O)cc1	0	Train
TNKS1_ML_0485	Cc1ccc(C2=C(O)C(=O)N(Cc3ccc(F)cc3)[C@H]2c2ccc(F)cc2)cc1	0	Train
TNKS1_ML_0486	CC[C@@H]1CS[C@H](c2cc3ccccc3o2)N1	0	Train
TNKS1_ML_0487	COc1ccc(C(=O)C2CCN([C@H]3CCN(CC(=O)Nc4nnc5n4CCS5)C3=O)CC2)cc1	1	Train
TNKS1_ML_0488	O=C(NCc1cccc(Cl)c1)c1cn(-c2ccc(-c3nc(C4CC4)no3)cn2)cn1	0	Train
TNKS1_ML_0489	CCc1c2c(nc3ccc(OC(=O)N4CCC(NC(=O)OCc5ccccc5)CC4)cc13)-c1cc3c(c(=O)n1C2)COC(=O)[C@]3(O)CC	0	Train
TNKS1_ML_0490	COc1cc(/C=C2/C(=O)NC(=O)N(c3ccc(Br)cc3)C2=O)ccc1OCc1ccccc1	0	Train
TNKS1_ML_0491	C[C@@H](Nc1ccc(C(=O)N2CCC[C@@H](O)C2)cc1[N+](=O)[O-])c1ccccn1	0	Train
TNKS1_ML_0492	COc1cc2cc3c(N)c(C(=O)N4CCCCCC4)sc3nc2cc1OC	0	Train
TNKS1_ML_0493	CC(C)c1nc2n(n1)C[C@@H]([NH2+]Cc1cccc3c1OCO3)CC2	0	Train
TNKS1_ML_0494	N#CC1(NC(=O)c2cc(F)ccc2Br)CCCC1	0	Train
TNKS1_ML_0495	Cc1ccc(C)c(Nc2nn3c(=O)c4ccc(C(=O)NC[C@@H]5CCCO5)cc4nc3s2)c1	0	Train
TNKS1_ML_0496	CC(=O)N1CC(=O)N2CC[C@@H](CC(=O)NCc3cccc[nH+]3)[C@@H](CCCOc3ccccc3C1)C2	0	Train

TNKS1_ML_0497	COCCOC(C)(C)c1ccc(-c2nc3ccc(F)cc3c(=O)[nH]2)cc1	1	Train
TNKS1_ML_0498	C[C@@H]1CC(OC(=O)C2(N)CCC2)C[C@@H](C)C1	0	Train
TNKS1_ML_0499	Cc1nn(-c2ccc(NC(=O)c3cc(=O)c4ccccc4o3)cn2)c(C)c1Cl	0	Train
TNKS1_ML_0500	CCOC(=O)c1csc(NC(=O)[C@H]2CCCN(S(=O)(=O)c3ccccc3)C2)n1	0	Train
TNKS1_ML_0501	O=C(CCC1CCNCC1)Nc1nnc2n1CCS2	1	Train
TNKS1_ML_0502	O=c1occ(C[NH+]2CCSCC2)c([O-])c1CN1CC[NH+](CCOc2ccccc2)CC1	0	Train
TNKS1_ML_0503	Brc1nnc(Nc2ccc3c(c2)OCCCO3)s1	0	Train
TNKS1_ML_0504	O=C(Nc1nnc2n1CCS2)c1ccccc1	1	Train
TNKS1_ML_0505	CC(=O)N1CCc2nc3cc([C@H]4CCCC[NH+]4Cc4cc5ccccc5o4)nn3c([O-])c2C1	0	Train
TNKS1_ML_0506	[O-]c1ccc[nH+]c1-c1nc([C@H]2CCc3ccccc32)no1	0	Train
TNKS1_ML_0507	Nc1cc2c(cc1N[C@H]1C[C@H]3CC[C@@H]1C3)OCCO2	0	Train
TNKS1_ML_0508	O[C@@H]1CCCC[C@@H]1Nc1ccc(N2CCCC2)cc1	0	Train
TNKS1_ML_0509	Cc1cccc2c(=O)[nH]c(N3CCC4(CC3)C(=O)N(CCO)c3cc(F)cc(F)c34)nc12	1	Train
TNKS1_ML_0510	C[C@@H](Nc1ccc(C(=O)[O-])c(F)c1F)c1cccs1	0	Train
TNKS1_ML_0511	CCCNc1cc(C(=O)NC2(C)CCCC2)cc(Cl)n1	0	Train
TNKS1_ML_0512	c1cc(N2CCNC3(CCCC3)C2)c2cccnc2c1	0	Train
TNKS1_ML_0513	CC(=O)OCC(=O)[C@@]1(O)CC[C@H]2[C@H]3CC=C4CC5(CC[C@]4(C)[C@@H]3C(=O)C[C@@]21C)OCCO5	0	Train
TNKS1_ML_0514	CC(C)n1c(=O)sc2cc(S(=O)(=O)N3CCN(C(=O)[C@@H](C)NC(=O)c4ccccc4)CC3)ccc21	0	Train
TNKS1_ML_0515	CC(C)c1ccccc1Nc1ccc(C(=O)[O-])nn1	0	Train
TNKS1_ML_0516	Cc1cc(N2CC[NH2+]CC2)c2ccc(C(=O)[O-])cc2[nH+]1	0	Train
TNKS1_ML_0517	Cc1ccc(CNC(=O)[C@@]2(C)CN(S(C)(=O)=O)CC(=O)N2Cc2ccc(N(C)C)cc2)cc1	0	Train
TNKS1_ML_0518	CCOCCOC(=O)C1=C(C)N=C2C[C@@H](c3cccs3)CC(=O)C2[C@H]1c1cc(OC)c(OC)cc1OC	0	Train
TNKS1_ML_0519	O=C([O-])c1cc(SCc2ccc([N+](=O)[O-])c(F)c2)ccn1	0	Train
TNKS1_ML_0520	Cc1nc2sc3c(c2c2nnc(SCC(=O)Nc4ccc5c(c4)OCCO5)n12)CCCC3	0	Train
TNKS1_ML_0521	CC(=O)N[C@H]1CN(C(=O)OC(C)(C)C)[C@@H]2CO[C@H](c3ccccc3)O[C@H]2[C@@H]1O	0	Train
TNKS1_ML_0522	CN(Cc1ccc(Cl)s1)C(=O)Nc1ccc([N+](=O)[O-])cc1	0	Train
TNKS1_ML_0523	O=C(c1ccccc1-c1ncc[nH]1)N1CC(=O)N(C2CCCCC2)C[C@H](OCc2ccccn2)C1	0	Train
TNKS1_ML_0524	Cc1cnc2c(c1)nc(N)n2C[C@H]1Cc2ccccc21	0	Train
TNKS1_ML_0525	COc1ccc(NC(=O)C2CCN(c3c(C(=O)N4CCC(C)CC4)cnc4snc(C)c34)CC2)cc1	0	Train
TNKS1_ML_0526	O=C([O-])[C@@H]1CCCC[C@H]1[NH+]1CCCCCC1	0	Train
TNKS1_ML_0527	Cc1cc(O)c(CN2CCN(CCO)CC2)c2c1C(=O)/C(=C/c1ccc3c(c1)OCO3)O2	0	Train
TNKS1_ML_0528	CC1(C)[C@@H](Nc2ccc3c(c2)CCO3)[C@@H]2CCO[C@@H]21	0	Train
TNKS1_ML_0529	O=C(Cn1nc(-c2ccco2)c2sc(N3CCCCC3)nc2c1=O)Nc1cc(Cl)ccc1Cl	0	Train
TNKS1_ML_0530	COc1ccc(N2CCN(c3nc4c(c(=O)[nH]3)CC(F)(F)CC4)CC2)cc1	1	Train
TNKS1_ML_0531	COc1ccc(C(=O)N/N=C2/C(=O)N(CN3CCCC[C@@H]3c3cccnc3)c3ccc(Br)cc32)cc1OC	0	Train
TNKS1_ML_0532	O=C1Nc2cccc(Cl)c2C12CCN(c1nc3c(c(=O)[nH]1)CCCC3)CC2	1	Train
TNKS1_ML_0533	O=C(Nc1c(Cl)cc(Cl)cc1Cl)N1CCOCC1	0	Train
TNKS1_ML_0534	Cn1cc[nH+]c1[C@@H](NC(=O)C1CCN(S(=O)(=O)c2ccc(F)c(F)c2)CC1)c1ccccc1F	0	Train

TNKS1_ML_0535	Nc1noc(-c2sccc2Br)n1	0	Train
TNKS1_ML_0536	CN(C)[C@@]1(C#N)CCCC1(C)C	0	Train
TNKS1_ML_0537	COc1cc([C@H]2C(C#N)=C(N)Oc3cc(C)n(CCC[NH+](C)C)c(=O)c32)cc2c1OCO2	0	Train
TNKS1_ML_0538	CS(=O)(=O)NC1CCN(c2cnn(-c3ccc(Cl)cc3)c(=O)c2Cl)CC1	0	Train
TNKS1_ML_0539	Cc1ccc([N+](=O)[O-])cc1N(CC(=O)N1CCN(C(c2ccccc2)c2ccccc2)CC1)S(C)(=O)=O	0	Train
TNKS1_ML_0540	CCOC(=O)C1=C(N)n2c(s/c(=C\c3ccc(Cl)cc3Cl)c2=O)=C(C(=O)OCC)[C@H]1c1ccc(Cl)cc1Cl	0	Train
TNKS1_ML_0541	O=C(NC[C@@H](O)c1cccs1)c1c(Cl)cccc1Cl	0	Train
TNKS1_ML_0542	Cn1c(=O)c2c([nH+]c3n([C@H]4CCS(=O)(=O)C4)c(-c4ccc5ccccc5c4)cn23)n(C)c1=O	0	Train
TNKS1_ML_0543	CC(C)(C)CC(=O)N1CCN([C@@H](C(=O)NCCC[NH+]2CCOCC2)C2CCCC2)CC1	0	Train
TNKS1_ML_0544	CNC(=O)[C@@]1(C)CC[C@](C)(C(=O)[O-])C1(C)C	0	Train
TNKS1_ML_0545	CC(C)NC(=O)c1cc(F)c(Cl)c(S(N)(=O)=O)c1Cl	0	Train
TNKS1_ML_0546	CN1c2ncccc2C(=O)NC1c1ccc(NC(=O)C2CCOCC2)cc1	1	Train
TNKS1_ML_0547	Cc1cc(C)cc(N(C)c2ncc(N)cc2C)c1	0	Train
TNKS1_ML_0548	Cc1[nH+]c(N)sc1S(=O)(=O)[N-]c1cccc(F)c1F	0	Train
TNKS1_ML_0549	C[NH+]1CCN(c2ccc(NC(=O)[C@@H]3CCCN(C4=NS(=O)(=O)c5ccccc54)C3)cc2)CC1	0	Train
TNKS1_ML_0550	COc1c(N)cc(-c2nc3cnccc3o2)cc1Br	0	Train
TNKS1_ML_0551	O=c1[nH]c(N2CCC3(CC2)CNc2cccc(Cl)c23)nc2c1CCCC2	1	Train
TNKS1_ML_0552	CCn1ccnc(N2C[C@H](C)N[C@@H](C)C2)c1=O	0	Train
TNKS1_ML_0553	CCOC(=O)NC(=O)c1ccsc1NC(=O)[C@H]1CCCN1S(=O)(=O)c1ccc(Cl)cc1	0	Train
TNKS1_ML_0554	COc1cc(OC)cc([C@H]2CC(=O)Oc3cc4c(c(O)c32)C(=O)O[C@H](C)CCCC(=O)CCC/C=C/4)c1	0	Train
TNKS1_ML_0555	Cc1cnn(-c2nc(C(F)(F)F)ccc2C(N)=[NH2+])c1	0	Train
TNKS1_ML_0556	O[C@H](CN1CCOCC1)c1ccc(-c2ccnc3[nH]ccc23)cc1	0	Train
TNKS1_ML_0557	O=C(Nc1ccc(N2CCCCC2)nc1)C1=NC=C(S(=O)(=O)N2CCCC2)C1	0	Train
TNKS1_ML_0558	Cn1c(Oc2ccccc2/[NH+]=C/c2cc(Br)c(O)c(Br)c2)cc(=O)n(C)c1=O	0	Train
TNKS1_ML_0559	Cc1cc(Br)cc(N)c1OCc1cn2ccccc2n1	0	Train
TNKS1_ML_0560	O=C(Nc1ccc([N+](=O)[O-])cc1)c1nn(-c2ccc(F)cc2)c2c1CCCC2	0	Train
TNKS1_ML_0561	CC[C@@](C)(O)[C@@]1(C#N)CCOc2ccccc21	0	Train
TNKS1_ML_0562	Cn1c(N)nnc1SCc1cc(Cl)c2c(c1)OCCO2	0	Train
TNKS1_ML_0563	Cc1nc2c(ccc3nc(NC(=O)[C@H]4CCCN(S(=O)(=O)c5ccc(Cl)cc5)C4)sc32)s1	0	Train
TNKS1_ML_0564	CCCCOc1ccc([C@H]2C(C(=O)c3ccc4c(c3)C[C@H](C)O4)=C([O-])C(=O)N2CCC[NH+](C)C)cc1OCC	0	Train
TNKS1_ML_0565	CC/C(C#N)=C(/Cl)c1cc(C)ccc1Br	0	Train
TNKS1_ML_0566	CC1CCN(C(=O)c2cn(C(C)C)cc(C(=O)NC[C@H]3COc4ccccc4O3)c2=O)CC1	0	Train
TNKS1_ML_0567	Cc1ccsc1[C@H](C)Nc1cccc(-c2nnnn2C)c1	0	Train
TNKS1_ML_0568	Cc1ccc2cc([C@H]3[NH+]=CN(C4CC4)C3=[NH2+])ccc2n1	0	Train
TNKS1_ML_0569	CN1CCCc2c1cc(-c1ccc(C(F)(F)F)cc1)[nH]c2=O	1	Train
TNKS1_ML_0570	N#CC1=C(N)Oc2c(c(=O)oc3ccccc23)[C@@H]1c1ccco1	0	Train
TNKS1_ML_0571	O=C(CCCC1CCCCC1)Nc1nnc2n1CCS2	1	Train
TNKS1_ML_0572	O=C(Nc1cnc2ccccc2c1)c1ccc(Cl)cc1[O-]	0	Train
TNKS1_ML_0573	CCN(CC)S(=O)(=O)c1ccc(C)c(NC(=S)N2CC[NH+](Cc3ccc4c(c3)OCO4)CC2)c1	0	Train
TNKS1_ML_0574	Nc1nc2nc(Br)ccc2s1	0	Train
TNKS1_ML_0575	CC(C)Sc1ccc(Cc2nnc(NC(=O)[C@@H]3CCCN3S(=O)(=O)	0	Train

	c3ccc(Cl)cc3)o2)cc1		
TNKS1_ML_0576	O=C(N[C@H]1C[C@H](c2nnc(-c3ccc(OC(F)F)cn3)n2-c2ccccc2F)C1)c1ccc(F)cn1	1	Train
TNKS1_ML_0577	CC1(C)CC[C@@H]2NCCCS(=O)(=O)[C@H]2C1	0	Train
TNKS1_ML_0578	Cc1csc(N2CCN(C[C@H](O)Cn3c4ccccc4c4ccccc43)CC2)[nH+]1	0	Train
TNKS1_ML_0579	Cc1ccc(S(=O)(=O)[N-]c2ccc3c(c2)[nH+]c(CN2CCN(C(=O)C(C)C)CC2)n3C)cc1	0	Train
TNKS1_ML_0580	CC(C)c1ccc(C2NC(=O)c3cccnc3N2)cc1	1	Train
TNKS1_ML_0581	CC1(C)Cc2c(c(N3CCOCC3)nc3sc4c(NC[C@@H]5CCCO5)nnnc4c23)CS1	0	Train
TNKS1_ML_0582	CC[C@@H]1c2ccsc2CCN1C(=O)N[C@H]1CCCCNC1=O	0	Train
TNKS1_ML_0583	CC(C)N(Cc1nc2c(c(=O)[nH]1)COCC2)C(=O)CN1CCC(C(=O)c2ccc(F)cc2)CC1	1	Train
TNKS1_ML_0584	O=c1[nH]c(N2CCC3(CC2)OCc2ccccc23)nc2c1CCCC2	1	Train
TNKS1_ML_0585	Nc1ccc(NS(=O)(=O)c2ccc(F)c(F)c2)cn1	0	Train
TNKS1_ML_0586	Cc1ccc2c(=O)[nH]c(-c3ccc(C4(O)COC4)cc3)cn12	1	Train
TNKS1_ML_0587	O=C(CN1CCN(c2ncnc3sccc23)CC1)N[C@H]1CCOc2ccccc21	0	Train
TNKS1_ML_0588	Cc1nnnn1[C@H](Cc1cccc(F)c1)C(=O)NCc1ccccc1-c1ccc(Cn2cc[nH+]c2)cc1	0	Train
TNKS1_ML_0589	Cc1csc([C@@H](C)NC(=O)N2CCC(C)(C(=O)[O-])CC2)n1	0	Train
TNKS1_ML_0590	C1CCC(CCCCNc2nnc3n2CCS3)CC1	1	Train
TNKS1_ML_0591	COc1cc(OC)c(NC(=O)CS(=O)(=O)c2ccc3c(c2)n(C)c(=O)n3C)cc1Cl	0	Train
TNKS1_ML_0592	O=C([O-])CN1C(=O)/C(=C\c2cn(CC(=O)Nc3ccc4c(c3)OCO4)c3ccccc23)SC1=S	0	Train
TNKS1_ML_0593	Clc1nnc(Cl)c(OC[C@@H]2CCCO2)n1	0	Train
TNKS1_ML_0594	CC1(C)Cc2c(ccn2-c2ccc(C#N)c(Cl)c2)[C@H](O)C1	0	Train
TNKS1_ML_0595	Cc1ccc(Cl)cc1N1CCN(S(=O)(=O)c2ccc3c(c2)CCC(=O)N3)CC1	0	Train
TNKS1_ML_0596	CC(C)NC(=O)[C@@H]1COC2(CCN(C(=O)C(C)(C)C)CC2)N1C(=O)c1ccc(F)cc1	0	Train
TNKS1_ML_0597	CC1=NN(C(=O)c2cc(Cl)c(O)c(Cl)c2)[C@](O)(C(F)(F)F)C1	0	Train
TNKS1_ML_0598	Cc1cnc(NC(=O)c2ccc(Br)cc2[O-])s1	0	Train
TNKS1_ML_0599	CC[C@H]1C(=O)NC(=O)CN1C(=O)C1(C)CCCC1	0	Train
TNKS1_ML_0600	Cn1cc[nH+]c1[C@@H](NC(=O)c1cccc(S(=O)(=O)N2CCCC2)c1)c1ccc(F)cc1	0	Train
TNKS1_ML_0601	Cc1cn2c3c(=O)[nH]c(=O)n(C)c3nc2n1CC[NH+]1CCN(Cc2ccc(Cl)cc2Cl)CC1	0	Train
TNKS1_ML_0602	COc1cccc([C@@H]2C(C#N)=C(N)Oc3c2sc2ccccc32)c1OC	0	Train
TNKS1_ML_0603	CCCNC(=O)[C@H]1CCCN(S(=O)(=O)c2cc(-c3noc(C4CCC4)n3)cs2)C1	0	Train
TNKS1_ML_0604	Cc1c(N(C)S(=O)(=O)c2cccc(C(=O)Nc3ccccc3Cl)c2)c(=O)n(-c2ccccc2)n1C	0	Train
TNKS1_ML_0605	Cn1cc(/C=C2\SC(=S)N(NC(=O)c3ccccc3[N+](=O)[O-])C2=O)c2ccccc21	0	Train
TNKS1_ML_0606	Cc1nc2sc([C@H](c3ccc(Br)cc3)N3CCN(Cc4ccccc4)CC3)c([O-])n2n1	0	Train
TNKS1_ML_0607	CC[C@@H]1CN(c2c(C#N)c(C)nn2C)[C@H](CC)CN1	0	Train
TNKS1_ML_0608	O=C([O-])c1[nH]nc2cc(Br)cc(F)c12	0	Train
TNKS1_ML_0609	CCOC(=O)N1CCN(c2nc3c(C)cccn3c(=O)c2/C=C2\SC(=S)N(C3CCCC3)C2=O)CC1	0	Train
TNKS1_ML_0610	C[NH+](C[C@@H]1CCCO1)C1CCN(S(=O)(=O)c2ccc(-c3ccc([O-])nn3)s2)CC1	0	Train
TNKS1_ML_0611	Cc1cccc(N2CC[NH+](CCNC(=O)CN(C)c3nnc(N4CCCC4=O)s3)CC2)c1C	0	Train

TNKS1_ML_0612	Cc1cccc2c1OCC/C(=C\c1cc(Cl)c3c(c1)OCO3)C2=O	0	Train
TNKS1_ML_0613	C[NH2+][C@H]1C[C@@H](C)C[C@H](C)[C@@H]1Sc1nc(C)cc([O-])n1	0	Train
TNKS1_ML_0614	COc1ccc([N-]S(=O)(=O)c2cc(C)ccc2C)cc1NS(=O)(=O)c1ccc(F)cc1	0	Train
TNKS1_ML_0615	N#Cc1ccc(CNC(=O)[C@@H]2Cc3[nH]c[nH+]c3CN2Cc2cc3ccccc3o2)cc1	0	Train
TNKS1_ML_0616	O=C(Nc1ccc(Cl)cc1)Nc1nc(-c2sccc2Br)cs1	0	Train
TNKS1_ML_0617	CC1CCC(N(C)C(=O)N[C@H](C)CC(=O)[O-])CC1	0	Train
TNKS1_ML_0618	C=CCn1c(=O)/c(=C\c2ccc(NC(C)=O)cc2)s/c1=C(/C#N)C(=O)C(C)C	0	Train
TNKS1_ML_0619	C[C@H](O)c1ccc(N2CCCCCCC2)cc1	0	Train
TNKS1_ML_0620	Cn1c(=O)[nH]c(=O)c2c1[nH+]c(N1CCCCC1)n2CCSc1nnnn1-c1ccccc1	0	Train
TNKS1_ML_0621	Cc1cccc(-c2noc([C@@H]3Cc4[nH]cnc4CN3C)n2)c1	0	Train
TNKS1_ML_0622	Nc1ccccc1/C([O-])=N/c1nncs1	0	Train
TNKS1_ML_0623	COc1ccc(OC)c2c1CN(Cn1nc(-c3ccc(Cl)cc3)n(C)c1=S)C[C@H]2O	0	Train
TNKS1_ML_0624	C[C@@H](C(=O)NC(C)(C)C)N1CC(C(=O)[O-])C1	0	Train
TNKS1_ML_0625	COc1ccc(/C=N\NC(=O)c2cc[nH]n2)cc1Cn1nc(C)c(Br)c1C	0	Train
TNKS1_ML_0626	COc1cc(C[NH+](C[C@H](O)COc2cccc(C)c2C)[C@@H]2CCS(=O)(=O)C2)cc(OC)c1OC	0	Train
TNKS1_ML_0627	COC(=O)CNC(=O)c1cn(CCc2cccc[nH+]2)cc(C(=O)NC2CCCCCC2)c1=O	0	Train
TNKS1_ML_0628	Cn1c(=O)c(=O)n(C)c2cc(S(=O)(=O)Nc3ccc(Cc4cccc[nH+]4)cc3)ccc21	0	Train
TNKS1_ML_0629	CC(C)C(=O)N1CCC(c2nc(O)cc([C@@H]3CCCN(C(=O)[C@@H]4CCCO4)C3)n2)CC1	0	Train
TNKS1_ML_0630	Cc1c(NC(=O)[C@@H]2CC(=O)N(c3cccc4ccccc34)C2)c(=O)n(-c2ccccc2)n1C	0	Train
TNKS1_ML_0631	c1ccc(CN2CCN(c3nnc(-n4cccc4CNCC[NH+]4CCCC4)s3)CC2)cc1	0	Train
TNKS1_ML_0632	CCc1nnc(NC(=O)CSc2nnc(CCc3[nH]c4ccccc4[nH+]3)n2C)s1	0	Train
TNKS1_ML_0633	COc1ccc([C@H]2C[C@H](O)c3cc(Br)ccc3O2)cc1O	0	Train
TNKS1_ML_0634	N#Cc1ccccc1Cn1c(=O)n(C[C@H]2CC[C@H](C(=O)NCc3ccccc3)CC2)c(=O)c2ccccc21	1	Train
TNKS1_ML_0635	Cc1c(Cl)ccc2c1NC(=O)[C@@]21C(C#N)=C(N)N(c2nnc(SCc3cccc(C(F)(F)F)c3)s2)C2=C1C(=O)CC(C)(C)C2	0	Train
TNKS1_ML_0636	Cc1ccc(O[C@@H]2CC[C@H](C)[C@H](C)C2)c(S(N)(=O)=O)c1	0	Train
TNKS1_ML_0637	CC1(C)CCc2[nH]c3cccc4c3c2C1=NNC4=O	1	Train
TNKS1_ML_0638	N#C[C@@H]1CCCN1C1CCCC1	0	Train
TNKS1_ML_0639	Cc1ccccc1-n1nnnc1SCC(=O)NCCN1C(=O)S/C(=C\c2ccc[nH+]c2)C1=O	0	Train
TNKS1_ML_0640	C=CCN1C(=O)N[C@H](c2ccccc2Cl)C2=C1CN(CC(=O)N1CCN(C(C)=O)CC1)C2=O	0	Train
TNKS1_ML_0641	Nc1ccc2nc(N3CCN4CCCC[C@@H]4C3)sc2c1	0	Train
TNKS1_ML_0642	N#Cc1ccc(-c2nnc(CSc3nnc(-c4ccncc4)n3-c3ccccc3Cl)o2)cc1	1	Train
TNKS1_ML_0643	Cc1n[nH]c(C)c1S(=O)(=O)N1CCCN(S(=O)(=O)c2ccccc2[N+](=O)[O-])CC1	0	Train
TNKS1_ML_0644	c1csc(CN2Cc3[nH+]c[nH]c3C[C@H]2c2nc(-c3ccncc3)no2)c1	0	Train
TNKS1_ML_0645	COC(=O)[C@H](c1ccccc1Cl)[NH+]1CCN(C(=O)[C@H](C)NC(=O)c2ccco2)CC1	0	Train
TNKS1_ML_0646	COc1cc(/C=C2\C(=O)NN(c3ccc(Cl)cc3)C2=O)cc(OC)c1O	0	Train

TNKS1_ML_0647	O=C(Nc1ccc(S(=O)(=O)N2CCOCC2)cc1)c1cc(-c2ccco2)nc2ccccc12	0	Train
TNKS1_ML_0648	N#Cc1ccccc1Cn1c(=O)n(C[C@H]2CC[C@H](C(=O)Nc3ccncc3)CC2)c(=O)c2ccncc21	1	Train
TNKS1_ML_0649	Cc1ccsc1-c1nnc(S)n1N	0	Train
TNKS1_ML_0650	CCOc1ccc(-c2nnc([C@H]3C[C@H](NC(=O)c4ccnc5cccnc45)C3)n2-c2ccccn2)nc1	1	Train
TNKS1_ML_0651	CCn1nc(C)c([C@@H]([NH3+])c2cc3nc(C)ccc3o2)c1C	0	Train
TNKS1_ML_0652	Cc1nc(N)sc1C(=O)N(CC(C)C)C(C)C	0	Train
TNKS1_ML_0653	O=S(=O)(NCc1nnc2n1CCN(Cc1ccccc1C(F)(F)F)CC2)c1ccccc1	0	Train
TNKS1_ML_0654	O=C(Nc1ccc(Cl)c([N+](=O)[O-])c1)[C@H](c1ccccc1)N1CCC[NH+](CC(=O)N2CCCC2)CC1	0	Train
TNKS1_ML_0655	COc1ccc(C(=O)C2CCN(CC(=O)N(Cc3nc4c(c(=O)[nH]3)COCC4)CC3CC3)CC2)cc1	1	Train
TNKS1_ML_0656	CS(=O)(=O)N1CCCC[C@@H]1C(=O)NCCN1C(=O)S/C(=C\c2cccs2)C1=O	0	Train
TNKS1_ML_0657	Cc1ccc(CN(C(=O)c2ccccc2O)[C@@H](C(=O)NC2CCCCC2)c2ccc(O)cc2)o1	0	Train
TNKS1_ML_0658	Cc1ccc(S(=O)(=O)Nc2ccc(F)c([N+](=O)[O-])c2)c(C)c1	0	Train
TNKS1_ML_0659	Cc1ccc(NC(=O)Nc2cccc(C#N)c2)cc1C	0	Train
TNKS1_ML_0660	O=c1[nH]c(SCCN2CCN(c3ccccn3)CC2)nc2ccccc12	1	Train
TNKS1_ML_0661	Nn1c(SCC(=O)N2CCN(S(=O)(=O)c3ccc(Cl)cc3)CC2)nnc1C1CC1	0	Train
TNKS1_ML_0662	O=C(C[S@@](=O)CC(=O)N(CC(=O)NC1CCCC1)Cc1cccs1)Nc1ccc2c(c1)OCO2	0	Train
TNKS1_ML_0663	O=C(Nc1cnc(Br)cn1)[C@@H]1C[C@H]2CCCC[C@@H]2N1	0	Train
TNKS1_ML_0664	Clc1ccccc1/N=C/c1cccnc1	0	Train
TNKS1_ML_0665	CC[C@@H](C)OC(=O)C1=C(C)NC2=C(C(=O)CCC2)[C@@H]1c1cc2c(cc1Cl)OCO2	0	Train
TNKS1_ML_0666	Cn1cc(NC(=O)NC(C)(C)C)c2ccccc21	0	Train
TNKS1_ML_0667	CC1(C(=O)[O-])CC[NH+](Cc2cc(Cl)ccc2[O-])CC1	0	Train
TNKS1_ML_0668	CCc1c(C)nc([C@H]2C[C@H]2C)nc1Cl	0	Train
TNKS1_ML_0669	C[NH+]1CCC(=NNC(=O)c2cc(O)c(O)c(O)c2)CC1	0	Train
TNKS1_ML_0670	CC[C@]1(c2ccccc2)NC(=O)N(CN2CCN(S(=O)(=O)N3CCCCCC3)CC2)C1=O	0	Train
TNKS1_ML_0671	CN(Cc1nc2c(c(=O)[nH]1)COCC2)C(=O)CN1CCC(C(=O)c2ccc(F)cc2)CC1	1	Train
TNKS1_ML_0672	COc1cc(/C=C2\C(=O)N(c3ccc(Br)c(C)c3)C(=O)N=C2[O-])ccc1OC(C)C	0	Train
TNKS1_ML_0673	O=C(/C=C/c1ccc(Cl)cc1Cl)Nc1nc(-c2ccc([N+](=O)[O-])cc2)cs1	0	Train
TNKS1_ML_0674	COc1cc(C(C)=O)ccc1OCC(=O)NCCN1C(=O)S/C(=C/c2ccc(Cl)cc2)C1=O	0	Train
TNKS1_ML_0675	Cc1ccc2c(c1)n(Cc1cccc(Cl)c1)c(=O)c1nc(C(=O)NCc3ccc(C)o3)nn12	0	Train
TNKS1_ML_0676	N#CC1=C(N)Oc2cc(OC(=O)c3ccco3)ccc2[C@@H]1c1c(Cl)cccc1Cl	0	Train
TNKS1_ML_0677	CCn1nnc2cc(C(=O)Nc3ccccc3Cl)ccc21	0	Train
TNKS1_ML_0678	CC(C)(C)c1ccc(-c2nc3ccc(F)cc3c(=O)[nH]2)cc1	1	Train
TNKS1_ML_0679	CNc1snnc1Cn1ccnc1C	0	Train
TNKS1_ML_0680	CCCc1nn(C)c(NCC2CC(O)C2)c1N	0	Train
TNKS1_ML_0681	Cc1ccn2nc(N3CCCNCC3)nc2c1	0	Train
TNKS1_ML_0682	CNc1nc(-c2cc(C)c(O)c(C)c2)nc(C)c1Br	0	Train
TNKS1_ML_0683	O=c1[nH]c2ccccc2c2c1sc1ccccc12	1	Train

TNKS1_ML_0684	CCc1c([NH3+])c(=O)n([C@]2(C)CCS(=O)(=O)C2)n1CC	0	Train
TNKS1_ML_0685	O=C1NC(=N/c2ccc(O)cc2)/C(=C/c2cccs2)S1	0	Train
TNKS1_ML_0686	[H]/N=C1C(=C/c2cn(CCOc3ccc(Br)cc3)c3ccccc23)/C(=O)N=C2SC(CCC)=NN2/1	0	Train
TNKS1_ML_0687	Nc1ccc2nc(-c3ccc4c(c3)OCCO4)cn2c1	0	Train
TNKS1_ML_0688	CN1CCN(C(=O)c2ccc(N3CC[NH2+]CC3)c(NS(=O)(=O)c3cccs3)c2)CC1	0	Train
TNKS1_ML_0689	Cn1cc(/C=C2\N=C(SCC(=O)Nc3ccc(Cl)cc3Cl)N(c3ccccc3)C2=O)cn1	0	Train
TNKS1_ML_0690	O=C([O-])c1[nH+]c(N[C@@H]2CCc3ccccc3C2)ccc1Cl	0	Train
TNKS1_ML_0691	O=C(Nc1ccc(F)cc1)N1CCN(S(=O)(=O)c2ccc(F)c(F)c2)CC1	0	Train
TNKS1_ML_0692	Cc1cc(C)c([N-]S(=O)(=O)c2ccc3c(c2)sc(=O)n3CCC(=O)N2CCOCC2)c(C)c1	0	Train
TNKS1_ML_0693	CCN(CC)S(=O)(=O)c1ccc2[nH]c(=O)cc(C(=O)Nc3cn(CC)ccc3=O)c2c1	0	Train
TNKS1_ML_0694	O=C(NN1CCCCC1)c1csc(Br)c1	0	Train
TNKS1_ML_0695	Cc1ccc(-n2c(C)c(C)c3c(N)nc(C)nc32)c(Br)c1	0	Train
TNKS1_ML_0696	Cc1ccc(F)c(N[C@@H]2COc3cc(O)ccc32)c1	0	Train
TNKS1_ML_0697	C[C@H]1C[C@@H]1NC(=O)c1cc(F)ccc1[N+](=O)[O-]	0	Train
TNKS1_ML_0698	Cc1ccccc1C(=O)N1[C@H](C(=O)NCc2cccc(C(F)(F)F)c2)COC12CCN(C(=O)c1cccs1)CC2	0	Train
TNKS1_ML_0699	Cc1ccn2cc(-c3ccc(NC(=O)c4ccoc4C)cc3)nc2c1	0	Train
TNKS1_ML_0700	CCOc1cc(CC2=Nc3c(c(=O)n(C)c(=O)n3CC(C)C)C2=O)cc(Br)c1O	0	Train
TNKS1_ML_0701	C[C@@H]1CCCCN1c1ccc(Cl)cc1C#N	0	Train
TNKS1_ML_0702	CS(=O)(=O)c1ccc(-c2nnc(SCc3nnc(-c4ccc(Cl)cc4)o3)n2-c2ccccc2Cl)nc1	1	Train
TNKS1_ML_0703	O=C(N[C@H]1C[C@H]2CC[C@@H]1O2)c1c(Cl)ccc(Cl)c1Cl	0	Train
TNKS1_ML_0704	Cc1nc(N2C(=O)C(=O)/C(=C(\O)c3ccc(F)cc3)[C@H]2c2cccc([N+](=O)[O-])c2)sc1C	0	Train
TNKS1_ML_0705	Cc1cc(N2CCC([C@H](C)O)CC2)ncc1Br	0	Train
TNKS1_ML_0706	CC(C)(C)c1ccc(S(=O)(=O)[C@H]2CS(=O)(=O)C[C@@H]2N[C@H]2CCS(=O)(=O)C2)cc1	0	Train
TNKS1_ML_0707	CC(C)C1CC2=NNC(=O)c3cc(F)cc4[nH]c(c2c34)C1	1	Train
TNKS1_ML_0708	COc1ccccc1NC(=O)c1ccc(NC(=O)N2CCN(C(=O)c3cc(Cc4n[nH]c(=O)c5ccccc45)ccc3F)CC2)cc1	1	Train
TNKS1_ML_0709	Nc1nnn(-c2ccc(Cl)c(Cl)c2)c1C1CC1	0	Train
TNKS1_ML_0710	[O-]c1ccc(Br)cc1/C=N\n1c(CO)nc2ccccc21	0	Train
TNKS1_ML_0711	CCOc1cc(/C=C2/C(=O)NC(=O)N(C)C2=O)cc(I)c1O	0	Train
TNKS1_ML_0712	COc1ccc(N2C[C@H](NC(=O)N3CCn4cccc4[C@@H]3c3ccc(F)c(F)c3)CC2=O)cc1OC	0	Train
TNKS1_ML_0713	CN1CCCc2c1nc(N1CCC3(CC1)C(=O)N(CC1COC1)c1cc(F)cc(F)c13)[nH]c2=O	1	Train
TNKS1_ML_0714	CCc1nn2c(=O)cc(COc3ccc(-c4nc(=O)c5c6c(sc5[nH]4)CCCC6)cc3OC)nc2s1	0	Train
TNKS1_ML_0715	CCOC(=O)N1CCN(S(=O)(=O)c2ccc(C(=O)Nc3ccc4c5c(cccc35)CC4)cc2)CC1	0	Train
TNKS1_ML_0716	CC(C)Oc1nc(N2CC[NH+]3CCC[C@@H]3C2)ccc1N	0	Train
TNKS1_ML_0717	COc1cccc2c1c(=O)n(C[C@H]1CC[C@H](C(=O)Nc3ccncc3)CC1)c(=O)n2Cc1cccc(C(F)(F)F)c1C#N	1	Train
TNKS1_ML_0718	CCc1cc(-c2nc3ncc(Br)cc3[nH]2)n(CC)n1	0	Train
TNKS1_ML_0719	Cc1ccc(S(=O)(=O)N(Cc2ccccc2)Cc2ccc(/C=N\NC(=O)c3c(C)nc4cc(C)ccn34)o2)cc1	0	Train
TNKS1_ML_0720	Cc1nc(COc2ccccc2C(=O)NNC(=O)c2cccc(S(=O)(=O)N(C)C)c2)cs1	0	Train
TNKS1_ML_0721	C[C@@H](NC(=O)CCc1nc2c(cnn2-	0	Train

	c2ccccc2)c(=O)[nH]1)c1ccc(Cl)cc1		
TNKS1_ML_0722	CC(C)[C@@H](NC(=O)Cc1ccccc1)C(=O)N1CCN(S(=O)(=O)c2ccccc2C#N)CC1	0	Train
TNKS1_ML_0723	Cc1ccc(-n2nc(C(=O)N[C@@H]3CS(=O)(=O)C[C@@H]3[NH+]3CCCCC3)c3c2CCC3)cc1	0	Train
TNKS1_ML_0724	COC(=O)[C@]1(CCSC)[NH2+][C@H](c2ccc(OC)cc2OC)[C@H]2C(=O)N(c3cccc(C(C)=O)c3)C(=O)[C@@H]21	0	Train
TNKS1_ML_0725	Cc1csc2c(=O)[nH]c(-c3ccc(C(C)(C)O)cc3)nc12	1	Train
TNKS1_ML_0726	Nc1ccc2ncn(-c3cncc(Br)c3)c2c1	0	Train
TNKS1_ML_0727	[H]/N=C1C(=C/c2cccn2-c2cc([N+](=O)[O-])ccc2Cl)/C(=O)N=C2SC(C(F)(F)F)=NN2/1	0	Train
TNKS1_ML_0728	COc1ccc([C@@H]2C[C@H](C(F)(F)F)n3ncc(C(=O)N(C)CCc4cccc[nH+]4)c3N2)cc1OC	0	Train
TNKS1_ML_0729	CO[C@H]1CN[C@@H](c2nc3cc(Br)c(F)cc3[nH]2)C1	0	Train
TNKS1_ML_0730	Cc1cc(C)c(S(=O)(=O)N2CCC[C@@H]2c2nnc(C(=O)Nc3ccc(Cl)cc3)s2)c(C)c1	0	Train
TNKS1_ML_0731	Cn1c(=O)c2[nH]c(N[C@H]3CCC[C@H]3C[NH3+])nc2n(C)c1=O	0	Train
TNKS1_ML_0732	CC(=O)c1ccc(S(=O)(=O)[N-]c2ccc(N3CCC([NH+]4CCCCC4)CC3)c(C(=O)[O-])c2)cc1	0	Train
TNKS1_ML_0733	Cc1c(C(=O)N(C)C)sc(NC(=O)c2c(I)cnn2C)c1C(=O)OC(C)C	0	Train
TNKS1_ML_0734	CCN(C(=O)N1CCC(C(=O)c2ccc3c(C(=O)O)nn(C)c3c2)CC1)c1ccc(OC)cc1	1	Train
TNKS1_ML_0735	COc1cccc2c1N[C@H](c1c(Cl)cccc1Cl)[C@H]1CC=C[C@H]21	0	Train
TNKS1_ML_0736	Cc1cn2c3c(=O)n(Cc4cccc5ccccc45)c(=O)n(C)c3[nH+]c2n1CCOCCO	0	Train
TNKS1_ML_0737	Cc1cccn2c(C)c(C(=O)C3=C([O-])C(=O)N(CCCn4cc[nH+]c4)[C@H]3c3ccccc3F)nc12	0	Train
TNKS1_ML_0738	COCc1cccc(N[C@@H](C)c2ccc3c(c2)OCO3)c1	0	Train
TNKS1_ML_0739	Cc1ccc(C)c(CC(=O)Nc2nc3ccc(F)cn3n2)c1	0	Train
TNKS1_ML_0740	CN[C@@H]1COc2c(Cl)cc(C)cc21	0	Train
TNKS1_ML_0741	Nc1ccc(-c2nc3ccccc3c(=O)[nH]2)cc1	1	Train
TNKS1_ML_0742	N/C(=N/O)C1(C(=O)N2CCSCC2)CCCCCC1	0	Train
TNKS1_ML_0743	Cc1cc(Sc2nc(C)cc(C)n2)nc(Cl)n1	0	Train
TNKS1_ML_0744	CCC[C@@H]1C[C@@](N)(c2nccs2)CCO1	0	Train
TNKS1_ML_0745	CN(C)C(=O)c1ccc(NC(=O)COC(=O)c2cccnc2SCC(F)(F)F)cc1	0	Train
TNKS1_ML_0746	Fc1ccc(-c2n[nH]c3c2CN(Cc2ccc4nonc4c2)CC3)cc1F	0	Train
TNKS1_ML_0747	COc1cccc(CN(Cc2nc3c(c(=O)[nH]2)COCC3)C(=O)CN2CCC(C(=O)c3ccc(F)cc3)CC2)c1	1	Train
TNKS1_ML_0748	CNC(=O)c1ccc(-c2nc3cc(NS(=O)(=O)c4cc(F)ccc4F)ccc3n2C)cc1	0	Train
TNKS1_ML_0749	Cc1n[nH]c(C)c1[N-]S(=O)(=O)c1cccc2ncccc12	0	Train
TNKS1_ML_0750	CCOC(=O)C1=C(C)N=c2s/c(=C\c3cc(Br)cc(OC)c3O)c(=O)n2[C@@H]1c1ccc(OC)c(OC)c1	0	Train
TNKS1_ML_0751	Cn1c(SCC(=O)Nc2ccc3c(c2)OC(F)(F)O3)nnc1[C@@H]1COc2ccccc2O1	0	Train
TNKS1_ML_0752	Cc1c(N2C(=O)CS/C2=N/N=C2\C(=O)Nc3ccccc32)c(=O)n(-c2ccccc2)n1C	0	Train
TNKS1_ML_0753	CC(=O)O[C@H]1C[C@H]2[C@@H](C)C(=O)[C@@]3([C@@H]1[C@@]14CCC[C@@](C)(CO[C@H]1OC(C)=O)[C@H]4C[C@H]3OC(C)=O)[C@@H]2OC(C)=O	0	Train
TNKS1_ML_0754	CN(C)S(=O)(=O)c1cccc(-c2nn(-c3ccccc3)cc2/C=C2\SC(=S)N(C[C@@H]3CCCO3)C2=O)c1	0	Train
TNKS1_ML_0755	O=C(CCc1ccccc1F)N(Cc1ccc[nH]1)Cc1nc2c(c(=O)[nH]1)C	1	Train

	OCC2		
TNKS1_ML_0756	C1=Cc2ccc(-c3ccc(CNCCN4CCOCC4)cc3)cc2NC1	1	Train
TNKS1_ML_0757	Cc1ccc(S(=O)(=O)[N-]c2cc(C(=O)N3CCCC3)cnc2N2CC[NH2+]CC2)c(C)c1	0	Train
TNKS1_ML_0758	O=C(CCc1ccc(S(=O)(=O)N2CCOCC2)cc1)NC1CCN(C(=O)c2ccco2)CC1	0	Train
TNKS1_ML_0759	COC[C@@H](C)CNc1cc2c(cc1N)CC(=O)N2	0	Train
TNKS1_ML_0760	C[C@H](Nc1ccc(F)c(F)c1)c1cc[nH]n1	0	Train
TNKS1_ML_0761	O=C(NCCCN1CC[NH+](Cc2ccccc2)CC1)[C@H]1CCCN(c2nnc(-n3cccc3)s2)C1	0	Train
TNKS1_ML_0762	CC(C)/C([O-])=N/S(=O)(=O)c1[nH+]cn(C)c1Cl	0	Train
TNKS1_ML_0763	Cc1cccc(NC(=O)Nc2ccc(F)c(C(=O)N(C)C)c2)c1C	0	Train
TNKS1_ML_0764	CC1(C)CCC[C@@]1(O)C1(C#N)CCCC1	0	Train
TNKS1_ML_0765	Cc1c(C(=O)Nc2ccc(N3CCOCC3)cc2N2CCOCC2)oc2ccc(F)cc12	0	Train
TNKS1_ML_0766	Cn1c(=O)c2cc(S(=O)(=O)N[C@H](C(=O)N3CCc4ccccc4C3)c3ccccc3)ccc2n(C)c1=O	0	Train
TNKS1_ML_0767	CCc1nc2sc([C@@H](c3cccc(F)c3)N3C[C@H](C)O[C@H](C)C3)c(O)n2n1	0	Train
TNKS1_ML_0768	Cc1ccc(S(=O)(=O)N2CCN(Cc3nc(=O)c4sccc4[nH]3)CC2)c(C)c1	0	Train
TNKS1_ML_0769	[H]/N=C1C(=C/c2cc(C)n(-c3ccc(C)c(Cl)c3)c2C)/C(=O)N=C2SC(c3ccco3)=NN2/1	0	Train
TNKS1_ML_0770	COc1ccc(CCn2c(C)cc3c(c2=O)[C@H](c2ccc(F)cc2)C(C#N)=C(N)O3)cc1OC	0	Train
TNKS1_ML_0771	CN1CCC(c2ccc(-c3nc4c(F)cc(F)cc4c(=O)[nH]3)cc2)CC1.O=C(O)C(F)(F)F	1	Train
TNKS1_ML_0772	O=C(NCc1ccc[nH+]c1N1CCOCC1)Nc1ccc(N2CCCS2(=O)=O)cc1	0	Train
TNKS1_ML_0773	Cc1cc(NC(=O)CSCC(=O)N2CCN(Cc3ccc(Cl)cc3)CC2)no1	0	Train
TNKS1_ML_0774	N#Cc1ccsc1C(=O)Oc1ccc2cn[nH]c2c1	0	Train
TNKS1_ML_0775	O=c1[nH]cnc2cc(-c3ccc(Br)s3)sc12	0	Train
TNKS1_ML_0776	C[C@H](c1ccc(F)cc1)N(Cc1nc2c(c(=O)[nH]1)COCC2)C(=O)CN1CCC(C(=O)c2ccc(F)cc2)CC1	1	Train
TNKS1_ML_0777	COc1ccc(-c2noc(CSc3nnc(C)n3-c3cccc(C)c3)n2)cc1	1	Train
TNKS1_ML_0778	O=C1NN=C2CCCc3[nH]c4cc(F)cc1c4c32	1	Train
TNKS1_ML_0779	O=C1NC(c2ccc(Cl)cc2)Nc2ncccc21	1	Train
TNKS1_ML_0780	C#CCNC(=O)N1CCSC[C@H]1c1ccc(Cl)cc1	0	Train
TNKS1_ML_0781	CCOc1ccc(-c2nnc([C@H]3C[C@H](NC(=O)c4cc(F)cc5nccnc45)C3)n2-c2ccccc2F)nc1	1	Train
TNKS1_ML_0782	COc1ccc(C(=O)C2CCN([C@@H]3CCN(Cc4nc5c(c(=O)[nH]4)COCC5)C3=O)CC2)cc1	1	Train
TNKS1_ML_0783	Cc1cccc2c1c(=O)n(C[C@H]1CC[C@H](C(=O)O)CC1)c(=O)n2Cc1cccc(C(F)(F)F)c1C#N	1	Train
TNKS1_ML_0784	CC[C@H]1CCC[C@H](NC(=O)N2CC[NH+](Cc3nc(C)no3)CC2)C1	0	Train
TNKS1_ML_0785	c1coc(-c2nccc3c2[nH]c2ccccc23)c1	0	Train
TNKS1_ML_0786	Cc1ccc(S(=O)(=O)NCCC(=O)N[C@@H](C)c2ccc(-n3cncn3)cc2)cc1	0	Train
TNKS1_ML_0787	O=C(O)[C@H]1CC=CC[C@@H]1C(=O)N1CCn2cnnc2C1	0	Train
TNKS1_ML_0788	C[C@@H]1CCC[C@H]([C@]2(O)CCCOC2)C1	0	Train
TNKS1_ML_0789	CSc1ncc(Br)c(/C([O-])=N/c2nc3ccc(S(C)(=O)=O)cc3s2)n1	0	Train
TNKS1_ML_0790	O=C(NCc1ccc(-n2ccnc2)c(F)c1)c1ccc(F)c(S(=O)(=O)N2CCCCC2)c1	0	Train
TNKS1_ML_0791	Cc1cccc(-n2c(C(F)(F)F)nc3c2CCNC3)c1C	0	Train
TNKS1_ML_0792	CN[C@H]1CC(C)(C)Cc2nc(N3CCCOCC3)sc21	0	Train

TNKS1_ML_0793	COc1ccc([C@@H]2[C@@H](C(=O)Nc3ccc(F)cc3F)c3cc(OC)c(OC)cc3C(=O)N2C)cc1OC	0	Train
TNKS1_ML_0794	O=C(Nc1nnc(-c2cccnc2)s1)c1cc2cc(Br)ccc2oc1=O	0	Train
TNKS1_ML_0795	CCCCOc1cccc([C@H]2/C(=C(\O)c3ccc4c(c3)OCCO4)C(=O)C(=O)N2CCCn2cc[nH+]c2)c1	0	Train
TNKS1_ML_0796	O=C(NCCCn1cc[nH+]c1-c1ccccc1)C(=O)NCc1ccc2c(c1)OCO2	0	Train
TNKS1_ML_0797	COc1ccc(NC(=O)N2CCC3=c4cc(OC)ccc4=[NH+][C@@H]3[C@@H]2c2ccc(C)cc2)c(OC)c1	0	Train
TNKS1_ML_0798	[H]/N=C1C(=C\c2ccc(OCCOc3cc(C)cc(CC)c3)c(OC)c2)/C(=O)N=C2SC(C(C)C)=NN2/1	0	Train
TNKS1_ML_0799	Cc1sc2nc(SCc3nc(-c4cccc(Br)c4)no3)nc(N)c2c1C	0	Train
TNKS1_ML_0800	CC(C)(O)c1ccc(-c2cn3c(CO)ccc3c(=O)[nH]2)cc1	1	Train
TNKS1_ML_0801	Cc1cc(S(=O)(=O)N(C)C)cc(NC(=O)CSc2ccc(S(=O)(=O)N(C)C)c[nH+]2)c1C	0	Train
TNKS1_ML_0802	[H]/N=C1C(=C/c2ccc(OCCOc3ccccc3Cl)cc2)/C(=O)N=C2SC(C(F)(F)F)=NN2/1	0	Train
TNKS1_ML_0803	COc1ccccc1N1CCN(c2nc3c(c(=O)[nH]2)CC(C)CC3)CC1	1	Train
TNKS1_ML_0804	CCN(Cc1nc2c(c(=O)[nH]1)COCC2)C(=O)CN1CCC(C(=O)c2ccc(F)cc2)CC1	1	Train
TNKS1_ML_0805	C[C@H]1c2ccsc2CC[NH+]1CC(=O)N1C[C@H](C(N)=O)Oc2ccccc21	0	Train
TNKS1_ML_0806	O=C([O-])[C@@H](c1ccc([O-])c(Cl)c1)[NH+]1CCCCCC1	0	Train
TNKS1_ML_0807	CN1CCc2c1nc(N1CCC3(CC1)C(=O)N(CCO)c1cc(F)cc(F)c13)[nH]c2=O	1	Train
TNKS1_ML_0808	O=C(CSc1nc2c(sc(=S)n2Cc2ccco2)c(=O)n1-c1ccc(F)cc1)NC[C@H]1CCCO1	0	Train
TNKS1_ML_0809	CS(=O)(=O)C1C[C@@H]2CC[C@H](C1)N2C(=O)c1ccc(NS(=O)(=O)c2ccccc2F)cc1	0	Train
TNKS1_ML_0810	C[C@H]1CCCCC[C@H]1NC(=O)NC[C@@H](C)C(=O)[O-]	0	Train
TNKS1_ML_0811	O=C1C[C@H](c2noc(-c3cc(-c4cccs4)n[nH]3)n2)CN1c1ccc2c(c1)CCC2	0	Train
TNKS1_ML_0812	Cc1cc(=O)[nH]c2cc(-c3ccc(C(=O)NCCN4CCOCC4)cc3Cl)ccc12	1	Train
TNKS1_ML_0813	C[C@H](N[C@@H]1CC[C@@H](C)[C@@H](C)C1)C(=O)NC(N)=O	0	Train
TNKS1_ML_0814	C[C@H]1CCCC[C@H]1Cn1c(N)n[nH]c1=S	0	Train
TNKS1_ML_0815	Cc1nc(N)c(C#N)c(-c2ccc(Cl)cc2)c1C	0	Train
TNKS1_ML_0816	Cc1c(C#N)c(N)n(-c2ccc(F)c(F)c2F)c1C	0	Train
TNKS1_ML_0817	CCCNC(=O)[C@@H](C)NC(=O)c1ccc(Cl)c(S(=O)(=O)N2CCc3ccccc32)c1	0	Train
TNKS1_ML_0818	Cl[C@H](c1ccc2[nH]c3ccccc3c2c1)[C@H]1COCCO1	0	Train
TNKS1_ML_0819	CCCCNC(=O)c1cn(C2CCCCC2)cc(C(=O)N2CC[C@@H]([NH+](CC)CC)C2)c1=O	0	Train
TNKS1_ML_0820	CCn1c(SCC(=O)Nc2nc(-c3ccccc3)cs2)nnc1[C@H](C)NC(=O)c1ccc(Cl)cc1Cl	0	Train
TNKS1_ML_0821	C[C@@H](Sc1ccc(S(=O)(=O)N2CCCCC2)cn1)C(=O)NC(=O)NC(C)(C)C	0	Train
TNKS1_ML_0822	O=C(N[C@@H]1CS(=O)(=O)c2ccccc21)c1nnn(-c2cccc(F)c2)c1C(F)(F)F	0	Train
TNKS1_ML_0823	CN1C(=O)c2cc(C(=O)NCCC[NH+]3CCCCC3)nn2C[C@]1(C)C(=O)NC1CCCCC1	0	Train
TNKS1_ML_0824	Cc1c(C(=O)N[C@H]2CCCC[C@@H]2C)sc2ncn(C)c(=O)c12	0	Train
TNKS1_ML_0825	CC(=O)c1cc(Cl)nc(Br)c1	0	Train
TNKS1_ML_0826	COc1ccc(Cl)cc1NC(=O)CSc1ncc(S(=O)(=O)c2cccs2)c(N)n1	0	Train
TNKS1_ML_0827	O=S1(=O)CC[C@@H](Nc2ccc3oc(C4CC4)nc3c2)C1	0	Train

TNKS1_ML_0828	Cl[C@H]1C[C@H](Oc2cccnc2)C12CCCCC2	0	Train
TNKS1_ML_0829	C[C@H]1CCCN(c2cncc(N)n2)[C@@H]1C	0	Train
TNKS1_ML_0830	COc1cccc2c(=O)[nH]c(-c3ccc(C)cc3)cc12	1	Train
TNKS1_ML_0831	CCc1ccc(-c2cc(C(=O)NCc3c(F)cccc3F)c3c(C)nn([C@@H]4CCS(=O)(=O)C4)c3n2)cc1	0	Train
TNKS1_ML_0832	[H]/N=C1C(=C/c2cc(C)n(-c3ccc(OCC)cc3)c2C)\C(=O)N=C2SN=C(S(=O)(=O)CC(C)C)N2\1	0	Train
TNKS1_ML_0833	COc1cc(/C=C2\SC(=S)N(NC(=O)c3cccc([N+](=O)[O-])c3)C2=O)cc(OC)c1OC	0	Train
TNKS1_ML_0834	Cc1ccc(S(=O)(=O)/N=c2/cc(Cl)cc[nH]2)cc1Cl	0	Train
TNKS1_ML_0835	Cc1cc2ncn([C@@H]3CCCC(C)(C)[C@@H]3O)c2cc1C	0	Train
TNKS1_ML_0836	O=C(NCc1cc(-c2ccc3c(c2)OCO3)on1)[C@@H]1CC(=O)N(c2ccc(Cl)cc2)C1	0	Train
TNKS1_ML_0837	Cc1nc(N)c2c(C)c(C)n(-c3ccc(C)c(F)c3)c2[nH+]1	0	Train
TNKS1_ML_0838	CCN1C(=C2SC(=S)NC2=O)C=Cc2cc(C)ccc21	0	Train
TNKS1_ML_0839	CC1(C)C[C@@H]1Nc1nsnc1Cl	0	Train
TNKS1_ML_0840	O=C1CCN(c2ccccc2C(=O)NCCc2ccc(Cl)cc2)C(=O)N1	1	Train
TNKS1_ML_0841	COc1ccc(C(=O)C2CCN(C(=O)CCCc3cn4c(C)ccc4c(=O)[nH]3)CC2)cc1	1	Train
TNKS1_ML_0842	C[C@@H]1CC(=O)C2=C(C1)OC(N)=C(C#N)[C@]21C(=O)N2c3c1cccc3[C@@](C)(c1ccccc1)CC2(C)C	0	Train
TNKS1_ML_0843	COc1cc2c(cc1F)nc(N)n2[C@@H]1C[C@H]2CC[C@@H]1C2	0	Train
TNKS1_ML_0844	CC[C@H]1CCc2nc(C(C)=O)sc2C1	0	Train
TNKS1_ML_0845	Cc1cc(F)cc2c(=O)[nH]c(-c3ccc(N4CCN(C)CC4)cc3)nc12	1	Train
TNKS1_ML_0846	C[NH+]1CCN([C@H](CNC(=O)c2cc(-c3ccccc3)on2)c2ccc3c(c2)OCO3)CC1	0	Train
TNKS1_ML_0847	CN[C@H]1CC(C)(C)Cc2nc(-c3cc(Cl)cs3)ncc21	0	Train
TNKS1_ML_0848	CC1(C)OC(=O)N([C@H]2CC[C@H](NC(=O)c3ccnc4cccnc34)CC2)[C@H]1c1ccccc1	1	Train
TNKS1_ML_0849	C=CCOc1ccc([C@H]2/C(=C(/O)c3ccc4c(c3)C[C@@H](C)O4)C(=O)C(=O)N2CCCn2cc[nH+]c2)cc1	0	Train
TNKS1_ML_0850	C[C@@H](CCC(=O)N1CCc2c(sc(N)c2C#N)C1)c1ccccc1	0	Train
TNKS1_ML_0851	CCOC(=O)C1=C(C)N=C(C)[C@@H]1S(=O)(=O)N1CCC(C(=O)NC2CCC(C)CC2)CC1	0	Train
TNKS1_ML_0852	Cc1ccc2oc(-c3n[nH]c(=S)n3C(C)C)cc2c1	0	Train
TNKS1_ML_0853	CC(C)NC(=O)NC(=O)[C@@H](C)Sc1nnc(C2CC2)n1C1CC1	0	Train
TNKS1_ML_0854	C=CCN1/C(=N/C=C(\C#N)C(=O)NCCc2ccc(OC)c(OC)c2)S[C@H]2CS(=O)(=O)C[C@H]21	0	Train
TNKS1_ML_0855	O=C(C1CC1)N1N=C2CCCCC[C@@H]2[C@]1(O)C(F)(F)F	0	Train
TNKS1_ML_0856	COc1cc2oc(=O)c(CC(=O)Nc3nnc(C(C)(C)C)s3)c(C)c2cc1Cl	0	Train
TNKS1_ML_0857	C[C@@H](OC(=O)c1cc(S(=O)(=O)N(C)C)ccc1N1CCCC1)C(=O)Nc1ccc(F)cc1Cl	0	Train
TNKS1_ML_0858	C#CCOc1c(I)cc(C=NNc2nc(C)cc(COC)c2C#N)cc1OC	0	Train
TNKS1_ML_0859	O=S(=O)([N-]c1cc(F)c(F)c(F)c1)c1c[nH+]c[nH]1	0	Train
TNKS1_ML_0860	CCN(C(=O)Cn1c([C@@H]2CC(=O)N(c3ccccc3F)C2)[nH+]c2ccccc21)c1ccccc1	0	Train
TNKS1_ML_0861	CC(C)[C@H]1CCCC[C@H]1NC(=O)c1[nH]ncc1Br	0	Train
TNKS1_ML_0862	O=C1CCCN1CCC/[NH+]=C(\O)c1cc(S(=O)(=O)N2CCOCC2)c(Cl)cc1Cl	0	Train
TNKS1_ML_0863	Cc1ccnc2c1[nH+]c(N)n2-c1ccc(Br)cc1	0	Train
TNKS1_ML_0864	Brc1ccc(-c2csc(-c3ncc[nH]3)n2)cc1	0	Train
TNKS1_ML_0865	CO/C([O-	0	Train

	])=C1\C(=O)N=C(SCC(=O)Nc2ccc(Br)cc2)[C@@H](C#N)[C@H]1c1ccccc1[N+](=O)[O-]		
TNKS1_ML_0866	Clc1ccc(-n2cnnn2)cc1N[C@@H]1C[C@H]2CC=C[C@@H]21	0	Train
TNKS1_ML_0867	Cc1c(C(N)=O)ccc2c1c(=O)n(C[C@H]1CC[C@H](C(=O)Nc3ccncc3)CC1)c(=O)n2Cc1cccc(C(F)(F)F)c1C#N	1	Train
TNKS1_ML_0868	Cc1nsc(N[C@@H]2CCCCC2(C)C)c1C#N	0	Train
TNKS1_ML_0869	Cc1ccc([C@@H]2[C@H]3COc4ccccc4[C@@H]3Nc3ncnn32)cc1	0	Train
TNKS1_ML_0870	CC1=Nc2cc(C(=O)NC(C)C)ccc2Sc2ccc(C)cc21	0	Train
TNKS1_ML_0871	CC(=O)OCC(=O)[C@]1(O)CC[C@H]2[C@@H]3CCC4=CC(=O)C=C(C)[C@]4(C)[C@H]3[C@@H](O)C[C@@]21C	0	Train
TNKS1_ML_0872	Clc1cnccc1NC1C[NH2+]C1	0	Train
TNKS1_ML_0873	C=CCOc1cccc([C@@H]2C(C(=O)c3c(C)[nH]c(C(=O)OC)c3C)=C(O)C(=O)N2CCC[NH+](C)C)c1	0	Train
TNKS1_ML_0874	c1ccc(C2COCN2c2ccc(-c3cncc(-c4ncccn4)c3)cc2)cc1	1	Train
TNKS1_ML_0875	O=C(Nc1ccc(Br)cc1)[C@@H]1CN(S(=O)(=O)c2ccccc2)CCN1S(=O)(=O)c1ccccc1	0	Train
TNKS1_ML_0876	COc1ccc([C@@H]2Oc3ccc(Cl)cc3C3=Nc4ncnn4[C@H](c4cccs4)[C@@H]32)cc1OC	0	Train
TNKS1_ML_0877	CNc1cc(CN2CC[C@@H](C)C[C@H]2C)ccc1[N+](=O)[O-]	0	Train
TNKS1_ML_0878	COc1cc(=O)n2c(c1C(=O)NCc1ccco1)CCN(C(=O)C#Cc1ccccc1)CC2	0	Train
TNKS1_ML_0879	Cc1cc(C)c(/C=C/c2onc(C)c2S(=O)(=O)N2CCC(C(=O)NCc3ccc[nH+]c3)CC2)c(C)c1	0	Train
TNKS1_ML_0880	CC(C)NC(=O)N1CCC(Oc2ccc(Cl)cn2)CC1	0	Train
TNKS1_ML_0881	Nc1nsc(N2CCC3(CCCC3)CC2)n1	0	Train
TNKS1_ML_0882	Cc1cc(C)c(-c2cnc([C@@H](C)Cl)o2)c(C)c1	0	Train
TNKS1_ML_0883	Cc1cc(C)cc(S(=O)(=O)NCc2nc3cccnc3n2Cc2ccc(F)cc2)c1	0	Train
TNKS1_ML_0884	O=C(c1ccc(F)cc1)C1CCN([C@@H]2CCN(Cc3nc4c(c(=O)[nH]3)COCC4)C2=O)CC1	1	Train
TNKS1_ML_0885	COc1cc([C@@H]2C(C(=O)c3ccco3)=C(O)C(=O)N2CCCn2cc[nH+]c2)cc(OC)c1OC	0	Train
TNKS1_ML_0886	Cc1ncn(CC(=O)N(Cc2cccc[nH+]2)c2nc3ccc(Br)cc3s2)c(=O)c1C	0	Train
TNKS1_ML_0887	Cn1cc(S(=O)(=O)NCc2ccccc2-c2ccc(CN3CCCC3=O)cc2)c(=O)n(C)c1=O	0	Train
TNKS1_ML_0888	N#CCc1nc(COc2ccc(Cl)cc2Cl)n[nH]1	0	Train
TNKS1_ML_0889	COc1ccc(-n2c3c(cc(C(=O)NCCc4ccc(OC)c(OC)c4)c2=O)C(=O)CC(C)(C)C3)cc1	0	Train
TNKS1_ML_0890	CN1CCN(S(=O)(=O)c2ccc(NC(=O)c3cccc([N+](=O)[O-])c3C(=O)[O-])cc2)CC1	0	Train
TNKS1_ML_0891	Cc1cc([C@H]2C(C#N)=C(N)OC3=C2C(=O)CC(C)(C)C3)c(C)s1	0	Train
TNKS1_ML_0892	CCn1cc(Cn2cc(NC(=O)CCn3cc(Br)c(C)n3)cn2)cn1	0	Train
TNKS1_ML_0893	Cc1cccc2c(O)nc(-c3ccc(O)cc3)nc12	1	Train
TNKS1_ML_0894	O=C(Nc1ccc(N2CCCCC2)[nH+]c1)N[C@@H](CN1CCCC1=O)c1ccccc1	0	Train
TNKS1_ML_0895	Cc1ccc(N2C3=C(C(=O)CCC3)[C@@H](c3ccc(C)s3)C(C#N)=C2n2cccc2)c([N+](=O)[O-])c1	0	Train
TNKS1_ML_0896	CC(=O)N[C@@H]1[C@@H](OCc2ccccc2)O[C@@H]2CO[C@H](c3ccccc3)O[C@@H]2[C@H]1OC(=O)/C=C\c1ccccc1	0	Train
TNKS1_ML_0897	CON(C)C(=O)c1c(C)cc(C)cc1C	0	Train
TNKS1_ML_0898	O=C(NC[C@H]1COc2ccccc2O1)N[C@@H](Cn1cnc2ccccc21)c1ccc(F)cc1	0	Train
TNKS1_ML_0899	COc1ccc(-c2noc(CSc3nnc(C)n3-c3ccc(OC)cc3)n2)cc1	1	Train

TNKS1_ML_0900	Cc1cc(C[NH+]2CCN(c3ccccc3[C@H](O)c3nccn3C)CC2)cc(C)c1O	0	Train
TNKS1_ML_0901	c1ccc2c(c1)C[C@H](NCc1cccc3nonc13)CO2	0	Train
TNKS1_ML_0902	O=c1[nH]c(-c2ccc([N+](=O)[O-])cc2)nc2ccccc12	1	Train
TNKS1_ML_0903	c1ccc(C2=NC3=C(CN2)COCC3)cc1	1	Train
TNKS1_ML_0904	C[C@H](Nc1ccc(C(=O)[O-])c(Cl)c1)c1ccccc1Cl	0	Train
TNKS1_ML_0905	CCCOc1c(C)c[nH+]c2c1c(=O)n(CC(=O)Nc1ccccc1C(F)(F)F)c(=O)n2C	0	Train
TNKS1_ML_0906	CC(=O)Nc1ccc(S(=O)(=O)NCC(=O)N2CCC[C@@H]2c2nc3ccccc3s2)cc1	0	Train
TNKS1_ML_0907	COc1ccc2sc3nncn3c2c1	1	Train
TNKS1_ML_0908	COCCCn1c(CN2CCC[C@@H]2c2nc3cc(Cl)ccc3[nH]2)cnc1S(C)(=O)=O	0	Train
TNKS1_ML_0909	CCS(=O)(=O)c1ccc(O)c(NC(=O)c2ccc(-c3ccc4c(c3)OCCO4)s2)c1	0	Train
TNKS1_ML_0910	CC(C)[C@@H]1C(=O)NC(C)(C)C(=O)N1[C@H]1CCCCC1(C)C	0	Train
TNKS1_ML_0911	O=C(C[C@@H]1CCN2C[C@@H]1CCCOc1ccc(N3CCOCC3)cc1CNCC2=O)NCc1ccccc1Cl	0	Train
TNKS1_ML_0912	Cc1ccc(C)c(C(=O)N[C@@H]2CCCCC[C@@H]2C(=O)[O-])c1	0	Train
TNKS1_ML_0913	CC(=O)N[C@@H]1[C@@H](Oc2cccc3ccccc23)O[C@@H]2CO[C@H](c3ccco3)O[C@@H]2[C@@H]1OCC(=O)[O-]	0	Train
TNKS1_ML_0914	Nc1cc(C(=O)N2CCO[C@@H]3CCCC[C@H]32)cc(Cl)c1Cl	0	Train
TNKS1_ML_0915	COc1ccc(-c2nc(CNC(=O)[C@H](c3ccccc3)N3CCSCC3)n[nH]2)cc1	0	Train
TNKS1_ML_0916	COc1cc(Cl)c(C)cc1NS(=O)(=O)c1ccc(N2C(=O)[C@@H](C)CS2(=O)=O)cc1	0	Train
TNKS1_ML_0917	Cc1ccc(-n2c(S[C@@H](C)C(=O)Nc3ccc4c(c3)OCCO4)nnc2N2CCOCC2)cc1	0	Train
TNKS1_ML_0918	CC1(C)CC[C@@](O)(c2cncnc2)C1	0	Train
TNKS1_ML_0919	C[C@H](NC(=O)Cn1ncc2ccccc21)c1cccc(-n2ccnc2)c1	0	Train
TNKS1_ML_0920	O[C@@H]1CCCCC[C@@H]1Sc1nnc2n1CCCCC2	0	Train
TNKS1_ML_0921	C[C@H]1CC[C@H](C(=O)[O-])[C@H]([NH+]2CCCCc3ccccc32)C1	0	Train
TNKS1_ML_0922	COc1ccc(C)cc1[C@H](C)NC(=O)C(=O)Nc1ccc(-n2cc[nH+]c2C)nc1	0	Train
TNKS1_ML_0923	O=S(=O)([N-]c1ccc(Oc2ncnc3cc(Cl)ccc23)nc1)c1cccs1	0	Train
TNKS1_ML_0924	Cc1cc(/C=N\NC(=O)C(=O)NC[C@H]2CCCO2)c(C)n1-c1cc(Cl)ccc1Cl	0	Train
TNKS1_ML_0925	CC(C)NS(=O)(=O)c1ccc(Cl)c(C(=O)NNC(=O)c2cc(Br)cn2C)c1	0	Train
TNKS1_ML_0926	CC(C)NS(=O)(=O)c1ccc(N2CCN([C@H](C)c3nc4ccccc4s3)CC2)c([N+](=O)[O-])c1	0	Train
TNKS1_ML_0927	COc1ccc(C(=O)C2CCN(C3CCN(Cc4ccc(Cl)c(C#N)c4)C3=O)CC2)cc1	1	Train
TNKS1_ML_0928	Cc1ccc(C)c(-n2cc(C[NH+]3CCN(C(=O)c4ncn[nH]4)CC3)c(-c3ccccc3)n2)c1	0	Train
TNKS1_ML_0929	COc1ccc([C@H]2C(C(=O)/C=C/c3ccccc3)=C(O)C(=O)N2CCCn2cc[nH+]c2)cc1OC	0	Train
TNKS1_ML_0930	C[C@@H]1CCC[C@H](NS(=O)(=O)c2ccc([O-])cc2)[C@@H]1C	0	Train
TNKS1_ML_0931	C[C@H](C(=O)Nc1ccc(C(C)(C)C)cc1)[NH+]1CCCC2(C1)OCCO2	0	Train
TNKS1_ML_0932	Cc1ccc(NC(=O)C2CCN(S(=O)(=O)c3cc(-c4noc(C)n4)sc3C)CC2)cc1	0	Train

TNKS1_ML_0933	N#CC1(C(=O)Nc2cc(Cl)cc(Cl)c2)CCCCCC1	0	Train
TNKS1_ML_0934	Cc1ccc(-c2coc(-c3cccc([N-]S(=O)(=O)c4ccc(N5CCCC5=O)cc4)c3)n2)cc1	0	Train
TNKS1_ML_0935	CSc1ccc(S(=O)(=O)N2CCC(C(=O)Nc3ccc(C(C)=O)cc3)CC2)cc1	0	Train
TNKS1_ML_0936	O=C(c1cnn(-c2nccc(-c3ccccc3C(F)(F)F)n2)c1C1CC1)N1CC[NH+](CCO)CC1	0	Train
TNKS1_ML_0937	Cc1ccc(-n2ncc(C(=O)[C@H]3CCN(C(=O)c4cnc(C)cn4)C3)c2N)cc1	0	Train
TNKS1_ML_0938	COc1cccc([C@@H]2Nc3c(C)cc([N+](=O)[O-])cc3[C@@H]3C=CC[C@H]32)c1	0	Train
TNKS1_ML_0939	Cc1ncn(-c2ncc(F)c3c(C(=O)C(=O)N4CCN(c5nc(C)[nH+]c6ccccc56)CC4)c[nH]c23)n1	0	Train
TNKS1_ML_0940	Cc1ccc(-c2ccc(=O)n([C@H](C)C(=O)NCCOc3cc(N4CC[NH+](C)CC4)ncn3)n2)cc1	0	Train
TNKS1_ML_0941	COCCn1nnnc1[C@@H](c1cc2c(C)cc(C)cc2[nH]c1=O)[NH+]1CCN(c2cc(Cl)ccc2C)CC1	0	Train
TNKS1_ML_0942	CC1(C)OC(=O)C(=CNc2ccc(F)c(F)c2)C(=O)O1	0	Train
TNKS1_ML_0943	CCn1[nH+]c(C)c(NC(=O)Nc2nnc(C(F)(F)F)s2)c1C	0	Train
TNKS1_ML_0944	COc1ccc(CCn2c(C)cc3c(c2=O)[C@@H](c2ccc[nH+]c2)C(C#N)=C(N)O3)cc1OC	0	Train
TNKS1_ML_0945	[NH3+]CC#Cc1cc(F)ccc1[N-]S(=O)(=O)c1c[nH+]c[nH]1	0	Train
TNKS1_ML_0946	CC1(C(=O)NC[C@H]2CCCC[C@H]2O)CCCC1	0	Train
TNKS1_ML_0947	Clc1ccc([C@H]2[NH2+][C@@H]3c4c(ccc5ccccc45)OC[C@@H]3CO2)cc1	0	Train
TNKS1_ML_0948	CN1C[C@H](S(=O)(=O)NC[C@@H](C2=c3ccccc3=[NH+]C2)c2ccccc2Cl)C(=O)N(C)C1=O	0	Train
TNKS1_ML_0949	c1cc2c(cc1/N=C1/NC3(CCCC3)CS1)OCO2	0	Train
TNKS1_ML_0950	CC(C)(O)C(F)(F)C(F)F	0	Train
TNKS1_ML_0951	Cc1cccc(-c2nnc([C@H]3C[C@H](NC(=O)c4ccc(F)cn4)C3)n2-c2ccccc2F)n1	1	Train
TNKS1_ML_0952	CCOC(=O)[C@H]1CCCCN1C(=O)c1cc(-c2cccc3ccsc23)cc(-n2cnnn2)c1	0	Train
TNKS1_ML_0953	Cc1ccc2nc(C)c(C(=O)N/N=C/c3ccccc3[O-])n2c1	0	Train
TNKS1_ML_0954	C[C@H](Sc1ccc(S(=O)(=O)N2CCCC2)cn1)C(=O)Nc1cccc(S(N)(=O)=O)c1	0	Train
TNKS1_ML_0955	COc1ccc(C(=O)C2CCN([C@H]3CCCN(CCC4NC(=O)C5=C(CCOC5)N4)C3=O)CC2)cc1	1	Train
TNKS1_ML_0956	COc1ccc(C(=O)C2CCN(C3CCN(Cc4nc5ccsc5c(=O)[nH]4)C3=O)CC2)cc1	1	Train
TNKS1_ML_0957	O=C(Nc1cccc2cccnc12)c1ccc(C2C(=O)[C@H]3[C@H]4C=C[C@H](C4)[C@H]3C2=O)cc1	1	Train
TNKS1_ML_0958	CC(C)NC(=O)[C@]1(Cc2cccc(-c3cc[nH+]cc3)c2)CCN(C(=O)Cn2cncn2)C1	0	Train
TNKS1_ML_0959	C#CCOC(=O)c1cc(Cl)c2[nH]nnc2c1	0	Train
TNKS1_ML_0960	O=c1[nH]c(-c2ccc(Cl)cc2)nc2c(O)cccc12	1	Train
TNKS1_ML_0961	C[C@@H]1C[C@H]2CCCC[C@@H]2N1C(=O)Nc1cnn(C[C@@H]2CCCO2)c1	0	Train
TNKS1_ML_0962	C[C@@H]1CN(S(=O)(=O)N(C)Cc2cn(Cc3ccccc3Cl)nc2-c2ccccc2)C[C@@H](C)O1	0	Train
TNKS1_ML_0963	COc1ccc(S(=O)(=O)CCC(=O)N(CC[NH+](C)C)c2nc3c(OC)ccc(Cl)c3s2)cc1	0	Train
TNKS1_ML_0964	O=CN[C@H]1OC(=O)C(Cl)=C1Cl	0	Train
TNKS1_ML_0965	C[C@@H]1CCC[C@@](C#N)([C@]2(O)CCO[C@H](C)C2)C1	0	Train

TNKS1_ML_0966	CCN(Cc1cccc([N+](=O)[O-])c1)C(=O)c1ccc(S(=O)(=O)Nc2cc[nH+]cc2)cc1	0	Train
TNKS1_ML_0967	CN1C(=O)[C@@H](C(C)(C)C)NC(=O)C12CCCC2	0	Train
TNKS1_ML_0968	CO[C@@]1(C#N)CC[C@H](C)C[C@H]1C	0	Train
TNKS1_ML_0969	CCCOc1ccc(C(=O)C2CCN(CC(=O)NCc3nc4c(c(=O)[nH]3)COCC4)CC2)cc1	1	Train
TNKS1_ML_0970	Cn1c(=O)c2c(SCC(=O)Nc3ccc(F)cc3F)nc(-c3ccc(Cl)cc3)nc2n(C)c1=O	0	Train
TNKS1_ML_0971	Cc1n[nH]c(C(=O)NC23CC4C[C@](C)(C2)C[C@@](C)(C4)C3)n1	0	Train
TNKS1_ML_0972	O=C(Cl)N1CCC[C@H]2CCC[C@@H]21	0	Train
TNKS1_ML_0973	Cc1ccc2c(c1)N(S(C)(=O)=O)C[C@H](C(=O)Nc1c(C)n(C)n(-c3ccccc3)c1=O)O2	0	Train
TNKS1_ML_0974	CC(C)(O)c1ccc(-c2cn3c(F)c(F)cc3c(=O)[nH]2)cc1	1	Train
TNKS1_ML_0975	C[C@H]1Cc2ccccc2N1c1cc(=O)n(C)c(=O)[nH]1	0	Train
TNKS1_ML_0976	O=C(CN1CCC(n2c(=O)[nH]c3ccccc32)CC1)NCc1nc2c(c(=O)[nH]1)COCC2	1	Train
TNKS1_ML_0977	O=C([O-])COCC(=O)/[NH+]=C1\S[C@@H]2CS(=O)(=O)C[C@@H]2N1c1ccc(Oc2ccccc2)cc1	0	Train
TNKS1_ML_0978	CCCn1c(N)c(C(=O)CSc2nnc([C@H](C)[NH+](C)C)n2-c2ccc(F)cc2)c(=O)n(C)c1=O	0	Train
TNKS1_ML_0979	CC[NH+]1CCC(CCNC(=O)[C@@H]2CCc3nc(N4CCN(C(C)=O)CC4)sc3C2)CC1	0	Train
TNKS1_ML_0980	C=CCc1ccccc1OCCCn1c([C@H](C)NC(=O)Cc2ccccc2C)[nH+]c2ccccc21	0	Train
TNKS1_ML_0981	C[C@H]1CCN(C(=O)Cl)[C@H](C)C1	0	Train
TNKS1_ML_0982	CC(C)[C@@H]1CC[C@@H](C)C[C@@]1(O)[C@@H]1CCO[C@]2(CCOC2)C1	0	Train
TNKS1_ML_0983	Cc1ccc(-n2c(S[C@H](C)C(=O)N[C@H]3CCS(=O)(=O)C3)nnc2N2CCCC2)cc1	0	Train
TNKS1_ML_0984	Cc1ccnc(SCCCn2c(N3CCN(C(=O)c4ccco4)CC3)[nH+]c3c2c(=O)[nH]c(=O)n3C)n1	0	Train
TNKS1_ML_0985	CC(=O)[C@@H](Cc1ccccc1)NC(=O)CSc1ccc(S(=O)(=O)N2CCCC2)c[nH+]1	0	Train
TNKS1_ML_0986	Cc1ccc(S(=O)(=O)Nc2ncnc3sccc23)cc1	0	Train
TNKS1_ML_0987	CC[NH+]1CCC[C@H]1CNS(=O)(=O)c1cc(-c2ccc(=O)n(-c3c(C)noc3C)n2)ccc1C	0	Train
TNKS1_ML_0988	[H]/N=C1C(=C/c2cc(CC=C)c(OS(=O)(=O)c3ccccc3)c(OC)c2)/C(=O)N=C2SC=NN2/1	0	Train
TNKS1_ML_0989	C[C@H](c1ccc(F)c(F)c1)N1CCN(C2CNC2)CC1	0	Train
TNKS1_ML_0990	CC1(C)O[C@H]2O[C@@H]([C@@H]3CO3)[C@H](O)[C@H]2O1	0	Train
TNKS1_ML_0991	c1csc([C@@H]2C[C@H](c3ccc4c(c3)OCO4)Nc3ncnn32)c1	0	Train
TNKS1_ML_0992	Cc1cc(N)nc([C@]2(C)CCCN2C2CCCC2)n1	0	Train
TNKS1_ML_0993	O=c1[nH]c(-c2ccsc2)nc2c1COCC2	1	Train
TNKS1_ML_0994	O=c1[nH]c(N2CCC3(CC2)CNc2cccc(F)c23)nc2c1CCCC2	1	Train
TNKS1_ML_0995	CNc1cc([C@H]2CC(=O)N(C)C2)[nH+]c(C2CCN(S(=O)(=O)N3CCCCC3)CC2)n1	0	Train
TNKS1_ML_0996	C[C@H]1CN2CCCC[C@@H]2CN1C1CCCCCC1	0	Train
TNKS1_ML_0997	O=C(CCC1CCCCCC1)Nc1nnc2n1CCS2	1	Train
TNKS1_ML_0998	COc1cc(/C=N/NC(=O)c2nnn(-c3nonc3N)c2C)cc(Br)c1OCc1cccc2ccccc12	0	Train
TNKS1_ML_0999	COc1cc(NC(=O)N[C@H](C)c2c(-n3cccc3)sc(C)c2C)cc(OC)c1OC	0	Train
TNKS1_ML_1000	CC[C@H](Sc1c([O-])on[n+]1C)C(=O)Nc1sc2c(c1C#N)CCN(C(C)=O)C2	0	Train

TNKS1_ML_1001	CC(=O)NC1=NN(C(C)=O)[C@@]2(S1)C(=O)N(CCOc1ccccc1C)c1c2ccc(C)c1C	0	Train
TNKS1_ML_1002	COc1cccc(NC(=O)/C(C#N)=C\[NH+]=C2/S[C@H]3CS(=O)(=O)C[C@H]3N2c2cccc(OC)c2)c1	0	Train
TNKS1_ML_1003	CC1(C)CCCC[C@@H]1N1CCNCC1=O	0	Train
TNKS1_ML_1004	Cc1cc(-n2nnc(N)c2C(C)C)ccc1Br	0	Train
TNKS1_ML_1005	CNc1[nH+]cc(S(=O)(=O)Nc2ccc(Cl)c(Cl)c2)cc1Cl	0	Train
TNKS1_ML_1006	O=C([O-])c1cn([C@@H]2CCS(=O)(=O)C2)[nH+]c1C1CCCCC1	0	Train
TNKS1_ML_1007	C[C@H](C(=O)NCCN1C(=O)S/C(=C/c2ccc(Cl)cc2Cl)C1=O)N1C(=O)CCC1=O	0	Train
TNKS1_ML_1008	Cc1cccc(C)c1CCC(=O)Nc1nnc2n1CCS2	1	Train
TNKS1_ML_1009	Cc1ccc(S(=O)(=O)Nc2ccc(=O)n3c2[C@@H]2C[C@H](C3)C[NH+](C3CC[NH+](C)CC3)C2)cc1	0	Train
TNKS1_ML_1010	COc1ccc(N2C(=O)/C(=C/c3ccc(N4CCCCC4)o3)C(=O)NC2=S)cc1	0	Train
TNKS1_ML_1011	O=c1[nH]c(-c2ccc(C(F)(F)F)cc2)nc2c1SCCC2	1	Train
TNKS1_ML_1012	O=C(CCSc1nc2c(c(=O)[nH]1)CCCC2)N[C@H]1CC[C@H](c2nnc(-c3ccccc3)o2)CC1	1	Train
TNKS1_ML_1013	C[C@H](C(=O)NC1(C#N)CCCCC1)N1CCN(S(=O)(=O)c2ccc3c(c2)OCCO3)CC1	0	Train
TNKS1_ML_1014	Cc1ccc2nc(CN3CCC(C(=O)Nc4nccs4)CC3)[nH]c2c1	0	Train
TNKS1_ML_1015	COc1cc(OC)c(Nc2nc3ccccc3nc2NS(=O)(=O)c2cccs2)cc1Cl	0	Train
TNKS1_ML_1016	COCc1c(Br)c(C)nc(OCC(=O)N/N=C/C2=c3ccccc3=[NH+]C2)c1C#N	0	Train
TNKS1_ML_1017	O=S(=O)(c1ccc(NCc2ccccc2CN2CCOCC2)nc1)N1CCCC1	0	Train
TNKS1_ML_1018	CC(C)(O)c1ccc(-c2nc3ccc(F)cc3c(=O)[nH]2)cc1	1	Train
TNKS1_ML_1019	C[C@H](Sc1ccc(NC(=O)c2ccc([N+](=O)[O-])cc2)cc1)C(=O)Nc1ccccc1Br	0	Train
TNKS1_ML_1020	C[C@H]1C[C@H](C(F)(F)F)n2ncc(-c3n[nH]c(=S)n3[C@@H](C)c3ccccc3)c2N1	0	Train
TNKS1_ML_1021	CCCN(Cc1nc2c(c(=O)[nH]1)COCC2)C(=O)CN1CCC(C(=O)c2ccc(F)cc2)CC1	1	Train
TNKS1_ML_1022	C[C@@H](Nc1ccc2nccnc2c1)c1cc(F)ccc1O	0	Train
TNKS1_ML_1023	O=c1[nH]c2sccc2c2cc(O)ccc12	1	Train
TNKS1_ML_1024	Cc1cc(C)c(-c2c[nH+]cc(C(=O)[O-])c2)c(C)c1	0	Train
TNKS1_ML_1025	Cc1cccc2c(=O)[nH]c(-c3ccc(CNC(=O)OCc4ccccc4)cc3)nc12	1	Train
TNKS1_ML_1026	NC(=O)[C@H]1C[C@H]2CCCC[C@H]2N1S(=O)(=O)c1ccc(N2CCCC2=O)cc1	0	Train
TNKS1_ML_1027	CN1C(=[NH2+])[C@@H](c2cncn2C2CC2)[NH+]=C1C1CCCC1	0	Train
TNKS1_ML_1028	O=C(c1ccc(OC(F)(F)F)cc1)C1CCN(CC(=O)N(Cc2nc3c(c(=O)[nH]2)COCC3)CC2CC2)CC1	1	Train
TNKS1_ML_1029	COc1cccc(/C=C2\Sc3ccc(C(=O)NCC(OC)OC)cc3NC2=O)c1	0	Train
TNKS1_ML_1030	COc1ccc(C(=O)C2CCN(C3CCN(CC(=O)Nc4noc5ncccc45)C3=O)CC2)cc1	1	Train
TNKS1_ML_1031	O=C(CCN1C(=O)N=C2C=CC=C[C@@H]2C1=O)N[C@@H](CC1=c2ccccc2=[NH+]C1)C(=O)[O-]	0	Train
TNKS1_ML_1032	CN(C(=O)Nc1ccc2c(c1)COC2=O)[C@@H]1CCCc2ccccc21	0	Train
TNKS1_ML_1033	COCCN(Cc1nc2c(c(=O)[nH]1)COCC2)C(=O)CN1CCC(c2nnc(-c3ccccc3)o2)CC1	1	Train
TNKS1_ML_1034	Nc1ncnc2c1ncn2C1OC(C(=O)N2CCN(CC(=O)Nc3cccc4c3CNC4=O)CC2)[C@@H](O)[C@H]1O	1	Train
TNKS1_ML_1035	CC(C)CNS(=O)(=O)c1ccc(S(=O)(=O)N2CCC[NH+](Cc3ccccc3)C2)cc1	0	Train
TNKS1_ML_1036	CC1(C)Cc2nc(N3CCOCC3)ncc2[C@@H](NC(=O)c2cc3ccc	0	Train

	cc3[nH]2)C1		
TNKS1_ML_1037	CC(C)(C)[C@H]1CCc2c(sc(NC(=O)Cn3nnc(-c4cccs4)n3)c2C#N)C1	0	Train
TNKS1_ML_1038	Cc1cccc(NC(=O)N2CCN(Cc3nc4cc(C(=O)N(C)C)ccc4n3C)CC2)c1	0	Train
TNKS1_ML_1039	Cc1ccc(N2CCN(C(=O)CSc3nnc(N)s3)CC2=O)cc1	0	Train
TNKS1_ML_1040	CC(=O)Nc1ccc(S(=O)(=O)NC[C@H](c2ccc3c(c2)CCN3C)N2CC[NH+](C)CC2)c(C)c1	0	Train
TNKS1_ML_1041	Cc1c(C#N)c(NC(=O)C[NH+]2CCN(CC(=O)N3CCCC3)CC2)n(Cc2ccccc2)c1C	0	Train
TNKS1_ML_1042	O=C(CCc1ccccc1)N(Cc1nc2c(c(=O)[nH]1)COCC2)Cc1ccccc1F	1	Train
TNKS1_ML_1043	O=C(CCn1cnc2ccccc2c1=O)NC[C@H]1CCCN1c1[nH+]ccs1	0	Train
TNKS1_ML_1044	C#CCNC(=O)N1CC[C@H](C(C)(C)C)[C@H](O)C1	0	Train
TNKS1_ML_1045	COc1cc2c(cc1F)[nH]c(=S)n2C1C(C)(C)C1(C)C	0	Train
TNKS1_ML_1046	Cc1cc(F)cc(N[C@]2(C#N)CCCOCC2)c1	0	Train
TNKS1_ML_1047	O=C(OCc1ccccc1)N1CCN(CCSc2nc3ccccc3c(=O)[nH]2)CC1	1	Train
TNKS1_ML_1048	COc1cc([C@@H]2N=C(O)c3c(sc4c3CC[NH+](C(C)C)C4)N2)ccc1OC(=O)c1ccc(C)cc1	0	Train
TNKS1_ML_1049	COc1ccc(-c2nnc(SCC(=O)NCCc3ccc(S(N)(=O)=O)cc3)nc2-c2ccc(OC)cc2)cc1	0	Train
TNKS1_ML_1050	Cc1cc(C)c2c3c(sc2n1)[C@@H](c1ccc(Br)c(F)c1)C(C#N)=C(N)O3	0	Train
TNKS1_ML_1051	CCOc1ccc(-c2nnc([C@H]3C[C@H](NC(=O)c4ccccn4)C3)n2-c2ccc(C)s2)nc1	1	Train
TNKS1_ML_1052	Cc1nn(-c2ccc([N+](=O)[O-])cc2CO)c(C)c1Cl	0	Train
TNKS1_ML_1053	[H]/N=C1C(=C/c2ccc(OC(=O)c3ccco3)cc2)/C(=O)N=C2SC([C@H](CC)c3ccccc3)=NN2/1	0	Train
TNKS1_ML_1054	Cc1ccsc1C(=O)c1cc2c(s1)CCCC2	0	Train
TNKS1_ML_1055	Cc1cc(C)cc([C@@H](O)c2ccc3c(c2)C[C@@H](C)O3)c1	0	Train
TNKS1_ML_1056	CC(C)c1ccc(-c2nc(=S)[nH]c([O-])c2C#N)cc1	0	Train
TNKS1_ML_1057	CS(=O)(=O)c1ccc(-c2nc(=O)c(CCC(=O)N3CC[NH+](Cc4ccccc4)CC3)n[nH]2)cc1	0	Train
TNKS1_ML_1058	CC[C@](C)([NH3+])C(=O)NC1CCC(O)CC1	0	Train
TNKS1_ML_1059	C/C=C/C(=O)N1C[C@H](C)C[C@@H](C)C1	0	Train
TNKS1_ML_1060	COc1ccc(OC)c2c1sc1nncn12	1	Train
TNKS1_ML_1061	CC[NH+]1CCN(C(=O)CC2CCN(C(=O)NC3CCC(C)CC3)CC2)CC1	0	Train
TNKS1_ML_1062	CC1(C)CCC[C@@]2(CC1)C[C@@H](O)CCO2	0	Train
TNKS1_ML_1063	Cc1n[nH]c(-c2ccccc2N[C@H]2CSCCC2(C)C)n1	0	Train
TNKS1_ML_1064	O=C(Nc1ccc2c(c1)OCCCO2)c1ccc(Cl)c([N+](=O)[O-])c1	0	Train
TNKS1_ML_1065	CC1(C)CCCC[C@H]1NC(=O)[C@]1(C(F)(F)F)CCNC1	0	Train
TNKS1_ML_1066	COc1cccc2c(=O)[nH]c(-c3ccc(Br)cc3)nc12	1	Train
TNKS1_ML_1067	Cc1ccc([C@@H](C)NC(=O)c2cn[nH]c2-c2cccc(F)c2)cn1	0	Train
TNKS1_ML_1068	COC(=O)[C@@H]1C[C@H]2Cc3c([nH]c4ccccc34)CN2C1	0	Train
TNKS1_ML_1069	CC(=O)Nc1ccc2nc(NC(=O)[C@@H](NS(=O)(=O)c3ccc(C)cc3)C(C)C)sc2c1	0	Train
TNKS1_ML_1070	O=C(Nc1ccc(Br)c([N+](=O)[O-])c1)c1ccnc(Cl)c1	0	Train
TNKS1_ML_1071	CCc1cccc(C)c1NC(=O)c1ncn2c1C(=O)N(C1CC1)[C@](C)(C(=O)NCc1ccc(F)cc1)C2	0	Train
TNKS1_ML_1072	O=C1C[C@@H](Sc2nc(-c3ccccc3)n[n-]2)C(=O)N1c1ccc(Oc2ccccc2)cc1	0	Train
TNKS1_ML_1073	C[C@@H]1O[C@H](C)[C@H](C(=O)Nc2ccc(Br)cc2)[C@@H]1C	0	Train

TNKS1_ML_1074	COc1cccc(OC)c1Oc1cc(Cl)c(Cl)cc1N	0	Train
TNKS1_ML_1075	COc1ccc(C(=O)C2CCN(CC(=O)N(Cc3nc4c(c(=O)[nH]3)CCCCC4)CC3CC3)CC2)cc1	1	Train
TNKS1_ML_1076	CC[C@H](C)NC(=O)c1ccc(Oc2ccc([N-]S(=O)(=O)c3cc(C)ccc3C)cc2)nc1	0	Train
TNKS1_ML_1077	Nc1cnn(CC(=O)Nc2c(F)cc(Br)cc2F)c1	0	Train
TNKS1_ML_1078	Cn1nc(C(=O)Nc2ccc(-c3nc4ccccc4s3)cc2)ccc1=O	0	Train
TNKS1_ML_1079	Cc1cccc(CN2CCCn3nc(C(=O)N[C@H](C)C(=O)NCc4cc[nH+]cc4)cc3C2=O)c1	0	Train
TNKS1_ML_1080	CCOc1ccc([C@H]2c3c(-c4cc(Cl)ccc4O)n[nH]c3C(=O)N2CCO)cc1OC	0	Train
TNKS1_ML_1081	Cc1nc(Cc2ccccc2)sc1C(=O)/N=c1\[nH]nc2ccccn12	0	Train
TNKS1_ML_1082	O=c1[nH]c(-c2ccc(Br)cc2)nc2c1COCC2	1	Train
TNKS1_ML_1083	Cc1ccc2[nH]c3c(c2c1)CN(C(=O)C[C@]1(c2ccccc2)CC(=O)N(Cc2ccc[nH+]c2)C1=O)CC3	0	Train
TNKS1_ML_1084	CC(C)(C)c1ccc(S(=O)(=O)N2CCN(C(=S)Nc3ccc(S(=O)(=O)N4CCOCC4)cc3)CC2)cc1	0	Train
TNKS1_ML_1085	N#C[C@H]1CCCC[C@@H]1Nc1ccc2c(c1)OCCO2	0	Train
TNKS1_ML_1086	CC(=O)N1CCN(c2nc3cc(Cl)ccc3cc2CNCCCN2CCCC2=O)CC1	0	Train
TNKS1_ML_1087	CC[NH2+][C@@H](c1ccc(Cl)cc1)c1ccc2nc([O-])oc2c1	0	Train
TNKS1_ML_1088	COc1ccc(S(=O)(=O)NC[C@@H](c2ccc[nH+]c2)S(=O)(=O)c2ccc(OC)cc2)cc1	0	Train
TNKS1_ML_1089	Cc1ccc(-c2nc3c(O)cccc3c(=O)[nH]2)cc1	1	Train
TNKS1_ML_1090	CCN1CCN(/C(=N/c2ccccc2)[P@](=S)(N(C)C)N2CC[NH+](CC)CC2)CC1	0	Train
TNKS1_ML_1091	Cc1nc(-c2c(C)c(C)nn(C)c2=O)sc1C(=O)NC[C@@H](C)N1CCN(c2ccccc2)CC1	0	Train
TNKS1_ML_1092	C[C@H](NC(=O)c1cc(Cl)ccc1NS(C)(=O)=O)c1ccc(-n2cncn2)cc1	0	Train
TNKS1_ML_1093	CC[NH+]1CCN(c2nc3c(c(=O)[nH]c(=O)n3C)n2C[C@H](C)CSc2nnc(C)s2)CC1	0	Train
TNKS1_ML_1094	N#Cc1c(Cn2c(=O)n(C[C@H]3CC[C@H](C(=O)Nc4ccncc4)CC3)c(=O)c3c(F)cccc32)cccc1C(F)(F)F	1	Train
TNKS1_ML_1095	COc1ccc(S(=O)(=O)N(C)C)cc1NC(=O)CN(C)[C@H](C)c1ccc(S(N)(=O)=O)cc1	0	Train
TNKS1_ML_1096	Nc1nc2cc(Br)ccc2n1-c1ccccc1Cl	0	Train
TNKS1_ML_1097	Cc1ccc(CSc2nnc(N3C(=O)C(=O)[C@@H](C(=O)c4ccc(F)cc4)[C@@H]3c3cc[nH+]cc3)s2)cc1	0	Train
TNKS1_ML_1098	CCC(=O)N(C1CC1)[C@H]1NN=C(SCC2=NC(=O)C3=C(C)[C@@H](C(C)=O)SC3=N2)S1	0	Train
TNKS1_ML_1099	O=[N+]([O-])c1ccc(OCC2CCOCC2)cc1Cl	0	Train
TNKS1_ML_1100	Cc1ccc(-c2nc3c(sc4ccccc43)c(=O)[nH]2)cc1	1	Train
TNKS1_ML_1101	Cc1cccn2c(C)c(C(=O)C3=C([O-])C(=O)N(CCC[NH+]4CCOCC4)[C@@H]3c3ccc(Cl)cc3)nc12	0	Train
TNKS1_ML_1102	CCCN1C(=O)c2cc(C(=O)NCCCN3CC[NH+](CCC)CC3)nn2C[C@@]1(C)C(=O)NC1CCCC1	0	Train
TNKS1_ML_1103	[O-]/C(=N\[C@H]1C/C(=N\O)c2cc3c(cc21)OCO3)C(F)(F)F	0	Train
TNKS1_ML_1104	Cc1ccc(-c2noc(CSc3nnc(C)n3-c3ccc(F)cc3)n2)cc1	1	Train
TNKS1_ML_1105	O=C(NCc1cccc[nH+]1)c1nn(CC2CC2)c2c1CN(C(=O)[C@H]1CC13CCCCC3)CC2	0	Train
TNKS1_ML_1106	Cc1c(CO)cccc1[C@H](C)c1c[nH]cn1	0	Train
TNKS1_ML_1107	O=C(Nc1cccc(S(=O)(=O)N2CCOCC2)c1)c1ccccc1OCc1cn2ccccc2n1	0	Train
TNKS1_ML_1108	Cn1c(CNS(=O)(=O)c2ccc(Br)cc2)nnc1SCc1ccc(Br)cc1	0	Train
TNKS1_ML_1109	O=C(Nc1ccc(F)cc1F)c1cc2nc(-c3ccco3)cc(C(F)(F)Cl)n2n1	0	Train

TNKS1_ML_1110	O=C(c1ccc(F)cc1)C1CCN(CC(=O)N(Cc2nc3c(c(=O)[nH]2)COCC3)CC(F)F)CC1	1	Train
TNKS1_ML_1111	[H]/N=C1C(=C\c2cc(C)n(-c3cc(C)ccc3OC)c2C)\C(=O)N=C2SN=C(S(=O)(=O)C(C)C)N2\1	0	Train
TNKS1_ML_1112	O=C(Nc1cc(S(=O)(=O)N2CCCC2)ccc1[O-])c1sccc1S(=O)(=O)N1CCCCC1	0	Train
TNKS1_ML_1113	CCOc1ccc(/C=N/NC(=O)c2ccoc2C)cc1CN1C[C@@H]2C[C@H](C1)Cn1c2cccc1=O	0	Train
TNKS1_ML_1114	C[C@]12CC[C@@H]3c4ccc(O)cc4CC[C@H]3[C@@H]1CCC21OCCO1	0	Train
TNKS1_ML_1115	C[C@H]1CCC[C@](O)(Cc2[nH+]cnn2C)[C@@H]1C	0	Train
TNKS1_ML_1116	O=C1C(=O)N(Cc2cccnc2)[C@@H](c2cccc(Cl)c2)/C1=C(/O)c1ccc2c(c1)OCCO2	0	Train
TNKS1_ML_1117	CN(C)c1nc(N)nc(Sc2nccs2)n1	0	Train
TNKS1_ML_1118	CC(C)(O)c1ccc(-c2nc3cccc(Cl)c3c(=O)[nH]2)cc1	1	Train
TNKS1_ML_1119	COc1ccccc1[C@H]1c2[nH]c3ccccc3c2C[C@@H]2C(=O)N(CCN3CCOCC3)CC(=O)N12	0	Train
TNKS1_ML_1120	CCn1c2ccccc2c2nnc(SCC(=O)NC[C@@H]3C[NH+](Cc4ccccc4)CCO3)nc21	0	Train
TNKS1_ML_1121	N#C/C(C(=O)c1ccc([N+](=O)[O-])s1)=C1/Nc2ccccc2S1	0	Train
TNKS1_ML_1122	CC1(C)C[C@H](Nc2ccc3nccnc3c2)CCO1	0	Train
TNKS1_ML_1123	[NH3+][C@@H]1[C@H]2CCO[C@@H]2[C@@H]1Nc1ccc(C(=O)[O-])c(F)c1F	0	Train
TNKS1_ML_1124	N#Cc1ccc(N2CCN([C@H]3C=C(c4cc5ccccc5c(=O)[nH]4)CC3)CC2)cc1	1	Train
TNKS1_ML_1125	Nc1cc2c(cc1C(=O)c1cccc(F)c1)OCCO2	0	Train
TNKS1_ML_1126	O=C(CC1CC1)N1CCC[C@]2(C(=O)NCC3CC3)CN(c3ccccn3)C[C@@H]2C1	0	Train
TNKS1_ML_1127	C[NH+](C)C1CCN(C(=O)N[C@H]2C=C[C@H](C(=O)[O-])C2)CC1	0	Train
TNKS1_ML_1128	Nc1ccc2nc(N3CCc4sccc4C3)oc2c1	0	Train
TNKS1_ML_1129	Cc1cccc([N-]S(=O)(=O)c2ccc(C)c(C(=O)OCC(=O)N3CCC[C@H]3c3cccs3)c2)c1	0	Train
TNKS1_ML_1130	CCOc1ccc(-c2nnc([C@H]3C[C@H](NC(=O)c4ccnc5cccnc45)C3)n2-c2ccc(C)s2)nc1	1	Train
TNKS1_ML_1131	CS(=O)(=O)Nc1cccc(NC(=O)c2nnn(-c3ccc(Br)cc3)n2)c1	0	Train
TNKS1_ML_1132	C[C@H]1CCC[C@]2(CS/C(=N\c3cccc(C#N)c3)N2)C1	0	Train
TNKS1_ML_1133	Cc1nc2cc(NC(=O)c3cc(-c4ccco4)n([C@H]4CCS(=O)(=O)C4)n3)ccc2s1	0	Train
TNKS1_ML_1134	O=[N+]([O-])c1cc(Br)cc(/C=N/n2cnnc2)c1OCc1ccc(Cl)cc1Cl	0	Train
TNKS1_ML_1135	COC(=O)CCN1C(=O)[C@@H](CC(=O)Nc2ccc(Br)cc2)S/C1=N/c1ccccc1	0	Train
TNKS1_ML_1136	COc1ccc(CNC(=O)[C@@H](C)Sc2ncnc3c2cnn3-c2ccc(F)cc2)cc1OC	0	Train
TNKS1_ML_1137	Cc1nnc(N2C(=O)C(O)=C(C(=O)c3c(C)nc4ccccn34)[C@@H]2c2ccc(Cl)cc2)s1	0	Train
TNKS1_ML_1138	CCn1c(SCc2cc(Cl)c3c(c2)OCCCO3)nc2cc(S(N)(=O)=O)ccc21	0	Train
TNKS1_ML_1139	CC[C@@H]1CCC[C@](Nc2ccccc2Cl)(C(=O)[O-])C1	0	Train
TNKS1_ML_1140	C[C@H]1CCC[C@H](c2noc(-c3c(N)sc4c3CCC4)n2)C1	0	Train
TNKS1_ML_1141	COc1ccc(C)cc1NS(=O)(=O)c1ccc2c(c1)[C@@H]1C=CC[C@@H]1[C@H](c1ccccc1[N+](=O)[O-])N2	0	Train
TNKS1_ML_1142	Cc1cccc([C@H](Cc2c(Br)c(C)nn2C)N[NH3+])c1C	0	Train
TNKS1_ML_1143	C[NH+](C)c1ccc([N-]C2=NC=NC3=NC=N[C@@H]32)cc1	0	Train

TNKS1_ML_1144	CCOc1cccc2sc(N3CCN(CCNC(=O)CS(=O)(=O)c4ccc(C)cc4)CC3)nc12	0	Train
TNKS1_ML_1145	[H]/N=C1C(=C/c2ccc(OCc3ccccc3[N+](=O)[O-])c(Br)c2)/C(=O)N=C2SC=NN2/1	0	Train
TNKS1_ML_1146	O=C(COc1ccccc1)/N=C1/S[C@@H]2CS(=O)(=O)C[C@H]2N1c1ccccc1C(F)(F)F	0	Train
TNKS1_ML_1147	COc1ccc(C(=O)N2CCc3ccccc32)cc1S(N)(=O)=O	0	Train
TNKS1_ML_1148	C[C@H]1C[C@@H](C)C[NH+](c2nc3sccn3c2C(=O)[O-])C1	0	Train
TNKS1_ML_1149	CC(C)(C)OC(=O)N1CCC[C@H](CNC(=O)c2ccc([N+](=O)[O-])cc2I)C1	0	Train
TNKS1_ML_1150	Cl/C(=C/c1csnn1)c1nc2cc(Br)ccc2[nH]1	0	Train
TNKS1_ML_1151	COc1ccc(C(=O)C2CCN(C3CCN(CC(=O)Nc4nncc5c4CCCO5)C3=O)CC2)cc1	1	Train
TNKS1_ML_1152	CC1(Nc2nsnc2Cl)CCCC1	0	Train
TNKS1_ML_1153	Nc1c(I)cnn1-c1ccc(Cl)nn1	0	Train
TNKS1_ML_1154	CN(Cc1ccccc1)c1nc2c(c(=O)n(C)c(=O)n2C)n1C[C@@H](O)CO	0	Train
TNKS1_ML_1155	Cc1ccc(NC(=O)c2ccc(C)c(C)c2[O-])nc1	0	Train
TNKS1_ML_1156	O=C1N(CCO)c2cc(Cl)cc(Cl)c2C12CCN(c1nc3c(c(=O)[nH]1)CCCC3)CC2	1	Train
TNKS1_ML_1157	CSc1ccc([C@@H]2C(C#N)=C(N)Oc3cc(OC(=O)c4ccccc4[N+](=O)[O-])ccc32)cc1	0	Train
TNKS1_ML_1158	C[C@H]1CCCN(S(=O)(=O)c2ccc(NS(=O)(=O)c3ccc4c(c3)CC(=O)N4)cc2)C1	0	Train
TNKS1_ML_1159	C[C@H](NC(=O)c1ccco1)C(=O)NNC(=O)c1cc(-c2ccc(Cl)cc2)nc2ccccc12	0	Train
TNKS1_ML_1160	CC(C)[C@H]1CCCN(C(=O)NC[C@@H](C)C(=O)[O-])CC1	0	Train
TNKS1_ML_1161	[H]/N=C1\C2=C(C)N=NC2=N[C@H](SCc2cc(=O)n3ccccc3[nH+]2)N1c1cccc(Cl)c1	0	Train
TNKS1_ML_1162	O=C(NNc1c(Cl)cc(Cl)cc1Cl)c1csnn1	0	Train
TNKS1_ML_1163	COC(=O)C1=C(C)N=c2s/c(=C\c3ccc(OC(C)=O)cc3OC(C)=O)c(=O)n2[C@@H]1c1ccc(OC)cc1	0	Train
TNKS1_ML_1164	O=C(NCCC[NH+]1CCOCC1)[C@@H]1Cc2ccccc2N2CCN(c3ncccn3)C[C@H]12	0	Train
TNKS1_ML_1165	Cc1noc(C)c1[C@H](C)NC(=O)N[C@H]1CCC[C@@H]1CCC(C)C	0	Train
TNKS1_ML_1166	Clc1nsnc1N[C@H]1CCOc2ccccc21	0	Train
TNKS1_ML_1167	O=C(c1ccc(F)cc1)C1CCN(CC(=O)N(Cc2ccnc2)Cc2nc3c(c(=O)[nH]2)COCC3)CC1	1	Train
TNKS1_ML_1168	C[C@H]1OCC[C@]12C[C@H](O)c1ccc(F)cc1O2	0	Train
TNKS1_ML_1169	CC[NH+](CC)CCCN1C(=O)C(O)=C(C(=O)c2sc(C)nc2C)[C@H]1c1ccc(OC)c(OC)c1	0	Train
TNKS1_ML_1170	COc1ccc(C(=O)C2CCN(C3CCN(Cc4nc5c(c(=O)[nH]4)COCC5)C3=O)CC2)cc1	1	Train
TNKS1_ML_1171	COC(=O)c1[nH]c(C)c(C(=O)[C@H]2C(=O)C(=O)N(CCC[NH+](C)C)[C@@H]2c2cccc(OC)c2)c1C	0	Train
TNKS1_ML_1172	Cc1cc(C)cc(C(=O)Nc2nc(C)cc(C)c2-c2noc(C)n2)c1	0	Train
TNKS1_ML_1173	CN1C(=O)CCC[C@@H](C(=O)O)[C@H]1c1ccc(Cl)c(Cl)c1	0	Train
TNKS1_ML_1174	CCOc1ccc(Nc2scc(-c3ccc(S(=O)(=O)N4CCCC4)cc3)[n+]2C[C@H]2CCCO2)cc1	0	Train
TNKS1_ML_1175	COC(=O)[C@@H]1C[C@H](O)CN1C(=O)CCC(=O)OCC(=O)[C@@]1(O)CC[C@H]2[C@@H]3CCC4=CC(=O)CC[C@]4(C)[C@H]3[C@@H](O)C[C@@]21C	0	Train
TNKS1_ML_1176	Cc1[nH+]ccn1-c1ccc(N2CCN(S(=O)(=O)c3cc(Cl)ccc3Cl)CC2)nn1	0	Train

TNKS1_ML_1177	COc1ccccc1N1CCN(C2=NC3CCCCCC3C(=O)N2)CC1	1	Train
TNKS1_ML_1178	CN(Cc1ccccc1)C1=[NH+][C@@H]2[C@H](C(=O)N=C([O-])N2C)N1CCSC1[NH+]=c2ccccc2=[NH+]1	0	Train
TNKS1_ML_1179	COc1ccc(C(=O)C2CCN(CC(=O)NCc3nc4c(c(=O)[nH]3)COCC4)CC2)cc1	1	Train
TNKS1_ML_1180	CC1=C[C@@H]([C@H]2N=N[C@H]([S-])N2/N=C/c2cccc(Cl)c2Cl)N=N1	0	Train
TNKS1_ML_1181	CC[N+](CC)=c1ccc2cc(-c3nnc(Nc4cc([N+](=O)[O-])ccc4F)s3)c(O)oc-2c1	0	Train
TNKS1_ML_1182	O=C(N[C@@H]1CCS(=O)(=O)C1)c1ccc(Cl)c(S(=O)(=O)NC[C@H]2CCCO2)c1	0	Train
TNKS1_ML_1183	Cn1nc(C(=O)N2CCc3[nH]nc(-c4ccc(Cl)cc4Cl)c3C2)ccc1=O	0	Train
TNKS1_ML_1184	CCN(CC)C(=O)c1c(NC(=O)c2ccc(S(=O)(=O)N(CC)CC)cc2)sc2c1CC[NH+](C)C2	0	Train
TNKS1_ML_1185	Cc1ccc(/C=C/C(=O)[O-])cc1-n1nnc2ccccc21	0	Train
TNKS1_ML_1186	C[C@@H](NC(=O)CN1CCN(S(=O)(=O)c2ccc3c(c2)OCCCO3)CC1)c1cc2ccccc2o1	0	Train
TNKS1_ML_1187	CC(C)[NH+](CC(=O)Nc1cc(C(F)(F)F)ccc1-n1cncn1)Cc1cnn(C)c1	0	Train
TNKS1_ML_1188	Cc1cc([C@@H](C)Nc2nccn3c(C)nnc23)c(C)o1	0	Train
TNKS1_ML_1189	Nc1ccc(-c2nc([C@@H]3CSCCS3)no2)cn1	0	Train
TNKS1_ML_1190	CC(=O)Nc1ccc(S(=O)(=O)Nc2cc(-c3cn4ccc(C)cc4n3)ccc2C)cc1	0	Train
TNKS1_ML_1191	CC(C)(N)C(=O)N1CC2=NNC(=O)c3cccc4[nH]c(c2c34)C1	1	Train
TNKS1_ML_1192	C[C@@]1(c2ccc3c(c2)OCO3)NC(=O)N(Cc2c(F)c(F)c(F)c(F)c2F)C1=O	0	Train
TNKS1_ML_1193	N#C[C@@H]1CC2(CCN1)OCCO2	0	Train
TNKS1_ML_1194	CC(C)[C@H]1OCC[C@H]1CNC(=O)N(C)[C@H]1CC[NH+](C)C[C@@H]1C	0	Train
TNKS1_ML_1195	CC(C)(C)c1nsc(N2CCCC[C@H]2CC(=O)[O-])n1	0	Train
TNKS1_ML_1196	CC(C)[C@@H]1C(=O)NC(=O)C[C@@H]1C1CCCCC1	0	Train
TNKS1_ML_1197	N[C@@]1(c2nc3cc(Br)ccc3o2)CCOC1	0	Train
TNKS1_ML_1198	O=C(Nc1cccnc1)c1cc(-c2ccc3c(c2)OCO3)nc2ccccc12	0	Train
TNKS1_ML_1199	C[C@H]1CCCN(C2CCC3(CC2)OCCO3)[C@@H]1C[NH3+]	0	Train
TNKS1_ML_1200	Cc1cc(-c2ccc(N)nc2)cc([C@@H]2CCN(C(=O)Cc3ccc4c(c3)OCCCO4)C2)n1	0	Train
TNKS1_ML_1201	COc1cc(-c2[nH]nc3c2[C@@H](c2ccc(Br)cc2)C(C#N)=C(N)O3)cc(OC)c1OC	0	Train
TNKS1_ML_1202	COc1ccc(S(=O)(=O)N2CCN(C(=S)Nc3cccc(Cl)c3C)CC2)cc1	0	Train
TNKS1_ML_1203	C[C@@H]1C[C@H](NC(=O)/C=C2\Sc3ccccc3N=C2[O-])CC(C)(C)C1	0	Train
TNKS1_ML_1204	COc1cccc([C@@H]2C(C(=O)c3sc(C)nc3C)=C(O)C(=O)N2c2nc3ccc(C)cc3s2)c1OC	0	Train
TNKS1_ML_1205	N/C(=N\O)[C@H](NC(=O)C1CCCCC1)C1CC1	0	Train
TNKS1_ML_1206	Cc1noc(/C=C/c2ccc(F)cc2F)c1S(=O)(=O)N1CCC(C(=O)NCc2cccnc2)CC1	0	Train
TNKS1_ML_1207	Cc1ccc(NC(=O)c2nn(C)c3ccc(Cl)cc3c2=O)cc1C	0	Train
TNKS1_ML_1208	O=C(CCc1cccc(Cl)c1Cl)Nc1nnc2n1CCS2	1	Train
TNKS1_ML_1209	CCn1nccc1[C@]1(O)C[C@H](C)CC(C)(C)C1	0	Train
TNKS1_ML_1210	COCCOC(=O)C1C(C)=NC2=C(C(=O)C[C@@H](c3ccccc3)C2)[C@H]1c1ccc([N+](=O)[O-])cc1	0	Train
TNKS1_ML_1211	[NH3+]C1(C(=O)NCC2(O)CCCC2)CCCCC1	0	Train
TNKS1_ML_1212	N#C/C(=C\c1ccc(-c2cccc([N+](=O)[O-])c2)o1)c1[nH]c2cc(Br)c[nH+]c2[nH+]1	0	Train

TNKS1_ML_1213	COc1cc(/C=c2\sc3n(c2=O)[C@H](c2ccc(Cl)cc2)C(C(=O)OC(C)C)=C(C)N=3)ccc1OC(C)=O	0	Train
TNKS1_ML_1214	O=C(CCc1ccccc1F)Nc1nnc2n1CCS2	1	Train
TNKS1_ML_1215	C[C@H]1CN(S(=O)(=O)c2ccc(NC(=O)CN(c3ccccn3)S(C)(=O)=O)cc2)C[C@H](C)O1	0	Train
TNKS1_ML_1216	Cc1ccc(-c2noc(CSc3nnc(C)n3-c3ccc(C)cc3)n2)cc1	1	Train
TNKS1_ML_1217	O=c1ccc(NS(=O)(=O)c2ccccc2)c2n1C[C@H]1C[C@@H]2CN(S(=O)(=O)c2ccccc2)C1	0	Train
TNKS1_ML_1218	N#C/C(=C/c1ccccc1N1CCCC1)c1nc2ccccc2[nH]1	0	Train
TNKS1_ML_1219	Fc1ccc(-c2[nH]c(-c3ccc(Br)cc3)nc2-c2ccncc2)cc1	0	Train
TNKS1_ML_1220	CC1CCC(O)(CN[C@H]2CCCC[C@H]2C#N)CC1	0	Train
TNKS1_ML_1221	[H]/N=c1/c(S(=O)(=O)c2ccc(Br)cc2)cc2c(=O)n3cccc(C)c3nc2n1CCc1ccccc1	0	Train
TNKS1_ML_1222	CNC(=O)[C@H](C)Oc1cc2c(cc1C=O)OCO2	0	Train
TNKS1_ML_1223	COc1cccc([C@@H](NS(=O)(=O)c2ccc(S(=O)(=O)N3CCCC3)cc2)c2[nH+]ccn2C)c1	0	Train
TNKS1_ML_1224	C[C@H]1CN(c2cc(F)c3c(c2)N(C)C(=O)C32CCN(c3nc4c(c(=O)[nH]3)CCCN4C)CC2)C[C@@H](C)O1	1	Train
TNKS1_ML_1225	C[C@H]1CC[C@@](C(=O)O)([C@@H]2CCOC3(CCCCC3)C2)C1	0	Train
TNKS1_ML_1226	C=CCN1C(=O)/C(=N\O)c2ccc(Cl)c(Cl)c21	0	Train
TNKS1_ML_1227	COC(=O)[C@H]1[NH+]=c2ccc(Cl)cc2=C1NC(=O)[C@H](C)N1CCN(CCO)CC1	0	Train
TNKS1_ML_1228	C[C@H](/C([O-])=N/c1cccc2nonc12)N1C(=O)/C(=C/c2ccccc2)SC1=S	0	Train
TNKS1_ML_1229	Cc1ccc(-c2nn(CC(=O)NCc3ccc4c(c3)OCO4)c(=O)c3nc(C)sc23)c(C)c1	0	Train
TNKS1_ML_1230	C[C@H](OC(=O)c1ccccc1NCCO)C(=O)N1CCN(S(=O)(=O)c2ccccc2)CC1	0	Train
TNKS1_ML_1231	Cc1ccc(NC(=O)Nc2ccc(Cl)cn2)cc1	0	Train
TNKS1_ML_1232	Cc1ccc(/N=C2/N[C@@]3(CCC[C@@H](C)C3)CS2)c(F)c1	0	Train
TNKS1_ML_1233	C[C@H](NC(=O)N(C)Cc1ccc2c(c1)OCO2)c1ccc(C#N)cc1	0	Train
TNKS1_ML_1234	CN1CCCc2c1nc(N1CCC3(CC1)C(=O)N(C)c1cc(N4CCNCC4)cc(F)c13)[nH]c2=O	1	Train
TNKS1_ML_1235	Cc1c(-c2ccnn2C(=O)Nc2ccccc2)cnn1-c1ccccc1	0	Train
TNKS1_ML_1236	COCCNc1scc(-c2cc(OC)ccc2OC)[n+]1/N=C/c1c(O)ccc2ccccc12	0	Train
TNKS1_ML_1237	O=C(COC(=O)c1c2c(nc3ccccc13)/C(=C\c1ccc3c(c1)OCO3)CC2)N[C@H]1CCS(=O)(=O)C1	0	Train
TNKS1_ML_1238	NC1(c2nc([C@H]3CCc4ccccc43)no2)CCCCC1	0	Train
TNKS1_ML_1239	Cc1cc(S(=O)(=O)N2CCc3nc(NC(=O)c4cnccn4)sc3C2)c(Cl)cc1Cl	0	Train
TNKS1_ML_1240	O=C(c1cc(Cc2n[nH]c(=O)c3ccccc23)ccc1F)N1CCN(C(=O)C2CC2)CC1	1	Train
TNKS1_ML_1241	Cc1ccc(O)c2c1[C@@H](C)C[C@H]2Nc1cnn(C)c1	0	Train
TNKS1_ML_1242	C[C@@H]1CC[NH2+]C2(CCCC2)CN1CCF	0	Train
TNKS1_ML_1243	C[C@H]1CCc2n[nH]c(C(=O)NC3CCCC3)c2C1	0	Train
TNKS1_ML_1244	CCC[NH+](CC(=O)N1CCc2cc(S(N)(=O)=O)ccc21)Cc1nnc(-c2cccs2)o1	0	Train
TNKS1_ML_1245	Cc1nn(C)c(C)c1S(=O)(=O)NCc1cc2n(n1)CCN(CC(C)C)C2	0	Train
TNKS1_ML_1246	Cc1cc([C@@H](C)Nc2nccn3c(C)nnc23)c(C)s1	0	Train
TNKS1_ML_1247	CC(C)(O)c1ccc(-c2nc3ccc(F)cc3c(=O)[nH]2)nc1	1	Train
TNKS1_ML_1248	O=C(CCc1ccccc1)N(Cc1ccsc1)Cc1nc2c(c(=O)[nH]1)COCC2	1	Train
TNKS1_ML_1249	Cc1ccn([C@@H]2CC3(CC[C@H]2[NH3+])OCCO3)n1	0	Train
TNKS1_ML_1250	O=C(NCCC1=c2ccccc2=[NH+]C1)[C@@H]1CC12CCN(C(=O)c1cccc(-n3cccn3)c1)CC2	0	Train

TNKS1_ML_1251	CC(=O)N1CCOc2ccc(S(=O)(=O)N3CCC(C(=O)Nc4cccc(F)c4)CC3)cc21	0	Train
TNKS1_ML_1252	O=C([O-])[C@H](c1c[nH]c2cc(Br)ccc12)[NH+]1CCCN(c2ncccn2)CC1	0	Train
TNKS1_ML_1253	Cc1cc(NC23CC4C[C@](C)(C2)C[C@@](C)(C4)C3)nc(Cl)n1	0	Train
TNKS1_ML_1254	COc1ccc(N2CCCCS2(=O)=O)cc1S(=O)(=O)Nc1cccc(C)c1C	0	Train
TNKS1_ML_1255	COc1ccc(C(=O)N2[C@@H](C(=O)NC(C)C)COC23CCN(C(=O)C2CCC2)CC3)cc1	0	Train
TNKS1_ML_1256	Cc1ccccc1-n1cc(C[NH3+])c(-c2cc3ccccc3o2)n1	0	Train
TNKS1_ML_1257	O=C(c1cccs1)c1nn(-c2ccccc2)c2nc(=O)c(Cc3ccc(Cl)cc3)nn12	0	Train
TNKS1_ML_1258	[H]/N=C1C(=C\c2ccc(N(C)C)c(Br)c2)/C(=O)N=C2SC(S(=O)(=O)CC(C)C)=NN2/1	0	Train
TNKS1_ML_1259	C[C@@H]1CCCCN1C(=O)Cn1cc(/C=C2\C(=O)NC(=O)N(Cc3ccco3)C2=O)c2ccccc21	0	Train
TNKS1_ML_1260	CCN(CC)S(=O)(=O)c1ccc(C(=O)Nc2sc3c(c2-c2nc4ccccc4s2)CCN(C(C)C)C3)cc1	0	Train
TNKS1_ML_1261	O=C(Nc1ccc2c(c1)OCO2)[C@H]1CCc2sc(C(=O)N3CCCC3)cc2C1	0	Train
TNKS1_ML_1262	CCN1C(=S)O[C@H](C=C(C#N)C#N)[C@@H]1O	0	Train
TNKS1_ML_1263	Cc1ccc2c(c1)sc1nc(C)c(S(=O)(=O)Nc3ccc(F)cc3)c(=O)n12	0	Train
TNKS1_ML_1264	CN1CC[NH+]([C@@H](CNC(=O)C(=O)Nc2ccc(C(F)(F)F)cc2)c2cccn2C)CC1	0	Train
TNKS1_ML_1265	CCCNS(=O)(=O)c1ccc(NC(=O)[C@H]2CC(=O)N(C3CCCCC3)C2)cc1	0	Train
TNKS1_ML_1266	C[C@H]1CN(c2ccc([N+](=O)[O-])cc2N)[C@H](C)CO1	0	Train
TNKS1_ML_1267	Cc1cc([N+](=O)[O-])ccc1N1C(=O)[C@@H]2[C@H]3c4ccccc4C=C[NH+]3[C@H](C(=O)c3ccco3)[C@@H]2C1=O	0	Train
TNKS1_ML_1268	CCc1ccsc1CNc1cc([N+](=O)[O-])ccc1C	0	Train
TNKS1_ML_1269	O=S(=O)([N-]c1ccccc1)c1ccc2c(c1)NCCC2	0	Train
TNKS1_ML_1270	CC(C)(O)c1ccc(-c2cn3cc(F)cc3c(=O)[nH]2)cc1	1	Train
TNKS1_ML_1271	CCOC(=O)c1c[nH+]c(S[C@@H](CC)C(=O)N2CCN(c3ncccn3)CC2)nc1N	0	Train
TNKS1_ML_1272	Cc1ccc(S(=O)(=O)N(CC(=O)NNC(=O)c2cn(C)c3ccccc23)Cc2ccco2)cc1	0	Train
TNKS1_ML_1273	Nc1nc2cc(I)ccc2n1C[C@H]1CCCOC1	0	Train
TNKS1_ML_1274	Cc1ccc(C2NC(=O)c3cccnc3N2)cc1	1	Train
TNKS1_ML_1275	C[C@@H]1CCC[C@H]([C@@H]2CC(=O)NC2=O)CC1	0	Train
TNKS1_ML_1276	C[C@H]1C=C2C(C#N)=C(N)C(C#N)(C#N)[C@@H](c3ccccc3)[C@]2(C)CN1C	0	Train
TNKS1_ML_1277	CC(C)c1csc(NC(=O)c2ccc([N+](=O)[O-])cc2Cl)n1	0	Train
TNKS1_ML_1278	O=C([O-])c1nn(-c2cccc3c2CCCC3)cc1O	0	Train
TNKS1_ML_1279	O=[N+]([O-])c1ccc(I)c(/C([O-])=N/c2cnc(Br)cn2)c1	0	Train
TNKS1_ML_1280	CC(=O)N1CCc2cc(Br)c(S(=O)(=O)N3CCC(C(=O)NCc4ccco4)CC3)cc21	0	Train
TNKS1_ML_1281	N#CC1(C2(O)CCC(c3ccccc3)CC2)CCCC1	0	Train
TNKS1_ML_1282	C=CCOc1ccc(/C=C2/C(=[NH2+])N3C(=NC2=O)S[NH+]=C3SCC(C)C)cc1OC	0	Train
TNKS1_ML_1283	CCN(CC)S(=O)(=O)c1ccc(N2CC[NH+](CC)CC2)c(NC(=O)Cn2c(C)csc2=O)c1	0	Train
TNKS1_ML_1284	C[C@]1(c2ccc3c(c2)CCC3)NC(=O)N(CN2CCN(Cc3ccc4c(c3)OCO4)CC2)C1=O	0	Train
TNKS1_ML_1285	O=C(NCc1cccc(C(F)(F)F)c1)c1coc(CN2CC[NH+](Cc3ccc([N+](=O)[O-])cc3)CC2)n1	0	Train
TNKS1_ML_1286	N#C[C@]1(Nc2ccccc2Br)COc2cc(O)ccc21	0	Train

TNKS1_ML_1287	O=C(Nc1ccc(S(=O)(=O)/N=C2/CCCN2)cc1)c1ccc(Br)cc1	0	Train
TNKS1_ML_1288	Fc1cc(Br)cc(-c2noc([C@@H]3COCC[NH2+]3)n2)c1	0	Train
TNKS1_ML_1289	CCOc1ccc(-c2nnc([C@H]3C[C@H](NC(=O)c4ccnc5cccnc45)C3)n2-c2ccccc2F)nc1	1	Train
TNKS1_ML_1290	O=C(Nc1nnc2n1CCS2)C1CCCC1	1	Train
TNKS1_ML_1291	CCc1nn(C)c(N)c1-c1ccc(OC(F)F)cc1	0	Train
TNKS1_ML_1292	CC(C)c1ccc([C@H]2c3[nH]c4ccccc4c3C[C@H]3C(=O)N(c4ccccc4C(=O)NC[C@H]4CCCO4)C(=O)N32)cc1	0	Train
TNKS1_ML_1293	C[C@@H]1CC[C@@](O)([C@@H]2CCOC2)[C@H](C)C1	0	Train
TNKS1_ML_1294	CCc1ccc(N2C[C@H](c3nnc(NC(=O)[C@H]4CC(=O)N(c5ccc(C)cc5)C4)s3)CC2=O)cc1	0	Train
TNKS1_ML_1295	CCc1[nH+]c2c(C(=O)[O-])cccc2cc1C	0	Train
TNKS1_ML_1296	CCN1C(=[NH2+])[C@@H](c2cc(Cl)c3c(c2)OCO3)[NH+]=C1C	0	Train
TNKS1_ML_1297	Cc1ccc(S(=O)(=O)N[C@@]2(C(F)(F)F)NC(=O)N(Cc3ccccc3)C2=O)cc1	0	Train
TNKS1_ML_1298	O=C(/C=C\c1cnc2ccccc2n1)NCC1CCC2(CC1)OCCO2	0	Train
TNKS1_ML_1299	CCOC(=O)c1sc(N2C(=O)C(=O)[C@@H](C(=O)c3cccs3)[C@@H]2c2ccc([N+](=O)[O-])cc2)nc1C	0	Train
TNKS1_ML_1300	COc1ccc(C(=O)C2CCN(C(=O)CCCc3nc4c(C)scc4c(=O)[nH]3)CC2)cc1C	1	Train
TNKS1_ML_1301	CCCNc1nc2ccccn2c(=O)c1/C=C1\SC(=S)N(CCOC)C1=O	0	Train
TNKS1_ML_1302	O=C(Nc1cc([N+](=O)[O-])ccc1Br)C1CC1	0	Train
TNKS1_ML_1303	CC1=C(C(=O)NC2CCCCC2)C2(CCCCC2)OC1=O	0	Train
TNKS1_ML_1304	O=C(CCC1CCCCC1)N1CCN(c2ncnc3c2nnn3-c2ccc(Cl)cc2)CC1	0	Train
TNKS1_ML_1305	O=S(=O)([O-])CCC[n+]1c(C/C=N/c2ccccc2)oc2ccc(-c3ccccc3)cc21	0	Train
TNKS1_ML_1306	O=C(N[C@H]1C[C@H](c2nnc(-c3ccccn3)n2-c2ccccc2F)C1)c1ccccn1	1	Train
TNKS1_ML_1307	Cc1cc(N)c(S(=O)(=O)N2C[C@H]3CC[C@@H]2C3)cc1C	0	Train
TNKS1_ML_1308	COc1c(C)cc(S(=O)(=O)Nc2ccc3c(c2)N(C(=O)c2ccco2)CC3)cc1C	0	Train
TNKS1_ML_1309	Cc1noc(C)c1S(=O)(=O)NCC(=O)O[C@@H](C)C(=O)Nc1cc(Cl)ccc1Cl	0	Train
TNKS1_ML_1310	Cc1cc2c(cc1CNC(=O)Cn1c(C(F)(F)F)nc3cc4c(cc31)OCCO4)OCCCO2	0	Train
TNKS1_ML_1311	CC(C)C1CCC(C#N)([C@@]2(O)CCO[C@@H](C)C2)CC1	0	Train
TNKS1_ML_1312	[H]/N=C1/C=C[C@H](c2nc(-c3cc(Br)cs3)no2)S1	0	Train
TNKS1_ML_1313	C[C@@H](NC(=O)[C@H]1Cc2ccccc2CN1C(=O)OC(C)(C)C)C(=O)Nc1ccc2c(c1)OCCO2	0	Train
TNKS1_ML_1314	Cc1ccc(N2C[C@H](C(=O)Nc3cccc(C)[nH+]3)CC2=O)c(C)c1	0	Train
TNKS1_ML_1315	O=[N+]([O-])c1ccc(Br)cc1S(=O)(=O)NCCc1nc[nH]n1	0	Train
TNKS1_ML_1316	CC(=O)Nc1ccc(/C=C2\SC(=O)N(CCN3C(=O)S/C(=C/c4ccc(N(C)C)cc4)C3=O)C2=O)cc1	0	Train
TNKS1_ML_1317	CN1CCCc2c1cc(-c1ccc(Br)cc1)[nH]c2=O	1	Train
TNKS1_ML_1318	COC(=O)CCCC(=O)N1CCC2(CC1)c1ccccc1[C@@H](NC(C)=O)[C@@H]2OCc1cc[nH+]cc1	0	Train
TNKS1_ML_1319	Nc1nc2cccc(Cl)c2n1Cc1cccc(F)c1	0	Train
TNKS1_ML_1320	O=C(c1ccc(Cl)c(S(=O)(=O)N2CCOCC2)c1)N1N=C2/C(=C/c3cccs3)CCC[C@H]2[C@@H]1c1cccs1	0	Train
TNKS1_ML_1321	Cc1ccccc1/N=C1\NN=C(c2c(C)[nH]c3ccccc23)CS1	0	Train
TNKS1_ML_1322	CC[C@H]1CCC[C@H](c2n[nH]c(Cl)n2)C1	0	Train
TNKS1_ML_1323	COc1ccc([C@@H]2Oc3ccccc3C3=Nc4[nH+]cnn4[C@@H](c4ccc([NH+](C)C)cc4)[C@@H]32)cc1	0	Train
TNKS1_ML_1324	O=C([O-	0	Train

	])[C@@H]1C[C@@H]2CC[C@H]1N2c1cc[nH+]c2cc(Cl)ccc12		
TNKS1_ML_1325	CC(C)(C)c1ccc(-c2nc(O)c3c(n2)CCSC3)cc1	1	Train
TNKS1_ML_1326	O=C1COc2cc(N[C@H]3C[C@H]4CC[C@@H]3C4)c(Cl)cc2N1	0	Train
TNKS1_ML_1327	Cc1ccc(C(=O)Nc2ccc(-c3csc4[n+]3CCN4)cc2)cc1[N+](=O)[O-]	0	Train
TNKS1_ML_1328	Cc1cc(I)ccc1N[C@@H]1CCOC(C)(C)C1	0	Train
TNKS1_ML_1329	CC(C)Oc1ccc(-c2nnc([C@H]3C[C@H](NC(=O)c4ccccn4)C3)n2-c2ccccc2F)nc1	1	Train
TNKS1_ML_1330	Nc1ccc(Oc2cccc(I)c2)c2nonc12	0	Train
TNKS1_ML_1331	CCn1cc([C@@H](C)NC(=O)[C@@H](C)n2nc(C(F)F)c(Br)c2C)c(C)n1	0	Train
TNKS1_ML_1332	CN(C[C@@H](O)c1ccccc1)C(=O)C[C@]1(c2ccccc2Cl)CC(=O)N(CC[NH+](C)C)C1=O	0	Train
TNKS1_ML_1333	O=C(C1=C([O-])C(=O)N(CC[NH+]2CCOCC2)[C@H]1c1cccc([N+](=O)[O-])c1)c1ccc(Br)cc1	0	Train
TNKS1_ML_1334	Cc1cnc(NC(=O)c2ccc(C)c(C)c2O)s1	0	Train
TNKS1_ML_1335	Cc1ccc2oc3c(c(=O)c2c1)[C@H](c1ccccc1F)N(CCCN1CCOCC1)C3=O	0	Train
TNKS1_ML_1336	CN(C)S(=O)(=O)c1ccc2c(c1)N(CCCN1CC[NH+](C)CC1)c1ccccc1S2	0	Train
TNKS1_ML_1337	N#Cc1ccc(NC(=O)[C@@H]2CSCN2S(=O)(=O)c2ccc(C(=O)NCC(F)(F)F)cc2)cc1	0	Train
TNKS1_ML_1338	O=C(CCc1ccccc1)Nc1nnc2n1CCS2	1	Train
TNKS1_ML_1339	Cc1nc(C)c(C(=O)Nc2ccc(Nc3ccc(-n4nc(C)c(C)c4C)nn3)cc2)s1	0	Train
TNKS1_ML_1340	Cc1cccc2c(O)nc(-c3ccccc3)nc12	1	Train
TNKS1_ML_1341	COc1ccc(N2CCN(c3nc4c(c(=O)[nH]3)COCC4)CC2)cc1	1	Train
TNKS1_ML_1342	C[C@@H]1C[C@@H](C)CN(S(=O)(=O)c2cccc(C(=O)NCc3ccccc3-n3cc[nH+]c3)c2)C1	0	Train
TNKS1_ML_1343	Cc1ccc(-c2nnc(NC(=O)c3ccc[nH+]c3N(C)C)s2)c(C)c1	0	Train
TNKS1_ML_1344	Cc1ccc(C)c(OCC(=O)NCc2[nH+]c3ccccc3n2CCCCOc2cccc(C)c2C)c1	0	Train
TNKS1_ML_1345	CC1CC[NH+](Cc2nnc(SCC(=O)NC(=O)Nc3ccccc3)n2C[C@@H]2CCCO2)CC1	0	Train
TNKS1_ML_1346	CS(=O)(=O)CC[C@H](C(=O)NCCC[NH+]1CCC(Cc2ccccc2)CC1)N1Cc2ccccc2C1=O	0	Train
TNKS1_ML_1347	COc1cc([C@H]2CC(=O)Nc3c2c(C)nn3-c2ccc(=O)[nH]n2)ccc1OC(F)F	0	Train
TNKS1_ML_1348	O=c1cc(-c2ccc(F)cc2)oc2ccccc12	1	Train
TNKS1_ML_1349	CC(=O)Nc1ccc(-c2csc(NC(=O)c3cc(C)on3)n2)c(F)c1	0	Train
TNKS1_ML_1350	COc1ccc(CC(=O)N2CCN(c3nnc(SCC(=O)Nc4cc(C)on4)s3)CC2)cc1	0	Train
TNKS1_ML_1351	Cc1c(C(=O)NCCN2C(=O)S/C(=C\c3ccc4c(c3)OCO4)C2=O)oc2ccccc12	0	Train
TNKS1_ML_1352	O=C(Nc1cccc2cccnc12)c1ccc(N2C(=O)[C@H]3[C@H]4C=C[C@@H](C4)[C@H]3C2=O)cc1	1	Train
TNKS1_ML_1353	O=c1[nH]c(CN(Cc2cccs2)S(=O)(=O)Cc2ccccc2)nc2c1COCC2	1	Train
TNKS1_ML_1354	CNc1nc(Cc2ccc(C)c(C)c2)nc(C(C)C)c1I	0	Train
TNKS1_ML_1355	CC[NH+]1CCN(CCC/N=C(\O)c2cc([O-])nc3ccc(S(=O)(=O)N4CCCC[C@@H]4C)cc23)CC1	0	Train
TNKS1_ML_1356	CS(=O)(=O)c1ccc2nc(N(Cc3ccc[nH+]c3)C(=O)c3ccc4c(c3)OCCO4)sc2c1	0	Train
TNKS1_ML_1357	C=CCn1/c(=N/C(=O)c2ccc(S(=O)(=O)C(C)C)cc2)sc2ccc([N	0	Train

	+](=O)[O-])cc21		
TNKS1_ML_1358	CC#CC[C@H]1CCCCC[C@@H]1C(=O)[O-]	0	Train
TNKS1_ML_1359	Cc1nnc2sc(-c3ccc(NC(=O)c4cccc(Cl)c4)cc3)nn12	0	Train
TNKS1_ML_1360	O=C(CNS(=O)(=O)c1cccc(Cl)c1)N[C@@H]1CCN(c2cccc[nH+]2)C1	0	Train
TNKS1_ML_1361	O=c1cc(-c2ccc(F)cc2)ncn1CCNS(=O)(=O)c1ccc(F)cc1	0	Train
TNKS1_ML_1362	COc1ccc(Br)c(N2C(=O)c3ccc(N)cc3C2=O)c1	0	Train
TNKS1_ML_1363	Cc1nn(-c2ccccc2C(F)(F)F)c2nc(-c3ccco3)cc(C(=O)NCc3ccc[nH+]c3)c12	0	Train
TNKS1_ML_1364	CCCNC(=O)O[C@@H]1CC[C@@]2(C)[C@H](CC(=O)N3CCC(C)CC3)[C@H](O)CC[C@@H]2[C@]1(C)CO	0	Train
TNKS1_ML_1365	C[C@H]1C[C@H](C)CN(S(=O)(=O)N2CCC[C@H](C(=O)NCCC[NH+]3CCN(C)CC3)C2)C1	0	Train
TNKS1_ML_1366	Cc1cc2oc3c(c(=O)c2cc1C)[C@]1(C(=O)N(C)c2ccccc21)N(CCCO)C3=O	0	Train
TNKS1_ML_1367	Cc1cccc(NC(=O)c2ccccc2NC(=O)Cn2c[nH+]c3c2c(=O)n(C)c(=O)n3C)c1	0	Train
TNKS1_ML_1368	Cc1noc(CCCNC(=O)c2ccc(Cl)c(S(=O)(=O)N3C[C@H](C)O[C@@H](C)C3)c2)n1	0	Train
TNKS1_ML_1369	O=C(CCc1ccccc1)N(Cc1nc2c(c(=O)[nH]1)COCC2)Cc1ccc(Cl)s1	1	Train
TNKS1_ML_1370	Cc1cc(N[C@@H]2CC[C@@H](C)[C@H](C)C2)n2ncnc2c1	0	Train
TNKS1_ML_1371	NC(=O)N1CCC[C@H](C(=O)N2CCN(CC3CCCCC3)CC2)C1	0	Train
TNKS1_ML_1372	c1ccc2c(c1)sc1nncn12	1	Train
TNKS1_ML_1373	Clc1ccc(C2=NN/C(=N\c3cccc[nH+]3)SC2)s1	0	Train
TNKS1_ML_1374	C[C@@H](NC(=O)CCc1ccccc1)c1nnc2n1CC[NH+](Cc1ccc3[nH+]cccc3c1)CC2	0	Train
TNKS1_ML_1375	O=C([O-])CCNC(=O)N1CCC2=c3cc(Cl)ccc3=[NH+][C@H]2C1	0	Train
TNKS1_ML_1376	O=S1(=O)Cc2cnc(NCc3ccccc3)nc2[C@H]2CN(Cc3ccc[nH+]c3)C[C@H]21	0	Train
TNKS1_ML_1377	O=C(Nc1nnc2n1CCS2)C1CCCCC1	1	Train
TNKS1_ML_1378	Cc1n[nH]cc1S(=O)(=O)[N-]c1ccc(N(C)C)[nH+]c1	0	Train
TNKS1_ML_1379	Cc1ccc2nc(CSc3sc(N)nc3C)cc(=O)n2c1	0	Train
TNKS1_ML_1380	Cc1cccc(Cc2c(C)nc(C)nc2N2CCN(C[C@H](O)CN3CCOCC3)CC2)c1	0	Train
TNKS1_ML_1381	O=C(Cc1cccc(Cl)c1)N1CCc2n[nH]c(C(=O)N3CC[NH+](Cc4ccccc4)CC3)c2C1	0	Train
TNKS1_ML_1382	CN(C)CCn1ccc(NC(=O)[C@H]2CSCN2C(=O)c2ccc(Cl)cc2)n1	0	Train
TNKS1_ML_1383	Cc1cc(-c2onc(N)c2Br)c(C)o1	0	Train
TNKS1_ML_1384	COC(=O)[C@@]1(C)OC12CCC(C)(C)CC2	0	Train
TNKS1_ML_1385	COCCn1/c(=N\C(=O)CCCS(=O)(=O)c2ccc(Cl)cc2)sc2cc(NC(C)=O)ccc21	0	Train
TNKS1_ML_1386	[H]/N=C1\S[C@@H](C)C(C)=C1c1nc2ccc(NC)nc2[nH]1	0	Train
TNKS1_ML_1387	Cc1cc2nc(C(C)C)cc(N3CCC(O)CC3)n2n1	0	Train
TNKS1_ML_1388	COc1ccc([C@@H]2CCCCCN2C(=O)c2cc(C)c(C)c(S(N)(=O)=O)c2)cc1	0	Train
TNKS1_ML_1389	COc1ccc(C(=O)C2CCN([C@@H]3CCN(Cc4nc5ccccc(=O)c5[nH]4)C3=O)CC2)cc1C	1	Train
TNKS1_ML_1390	Cc1cc(C)cc([C@H]2CN[C@@H](C)[C@H](C)O2)c1	0	Train
TNKS1_ML_1391	COc1ccc(C(=O)C2CCN(CC(=O)NCc3nc(C)c(C)c(=O)[nH]3)CC2)cc1	1	Train
TNKS1_ML_1392	CC(C)C(=O)Nc1nc2cc3c(cc2s1)OCCO3	0	Train
TNKS1_ML_1393	C[C@H]1CN[C@@H]2CCCCCCCCCC[C@@H]2[S@@]1=O	0	Train
TNKS1_ML_1394	Cc1ccc([C@@H](CNC(=O)[C@]2(C)CCCCN2C(=O)c2ccc	0	Train

	cc2)N2CCOCC2)o1		
TNKS1_ML_1395	CC[C@@H]1Oc2c(C)ccc(C(C)C)c2[C@H]1O	0	Train
TNKS1_ML_1396	Cc1cc(C)c(C)c(S(=O)(=O)Nc2ccc3c(c2)n(C)c(=O)n3C)c1	0	Train
TNKS1_ML_1397	CCc1nnc(NC(=O)c2ccccc2NC(=O)c2c(-c3ccccc3)noc2C)s1	0	Train
TNKS1_ML_1398	Cc1ccc([N-]S(=O)(=O)c2c(C)cc(N3C(=O)CCS3(=O)=O)cc2C)c(Br)c1	0	Train
TNKS1_ML_1399	O=C(OCC(=O)c1ccc(Cl)c(Cl)c1)/C(=C/c1ccco1)n1nnnc1-c1ccccc1	0	Train
TNKS1_ML_1400	C[C@]1(c2ccccc2)NC(=O)N(NC(=O)c2cccc(S(=O)(=O)N3CCCCCC3)c2)C1=O	0	Train
TNKS1_ML_1401	Cc1nc(CN2CC3(CCCCC3)NCC2(C)C)sc1C	0	Train
TNKS1_ML_1402	C/C(=N/NS(=O)(=O)c1ccc(C)c([N+](=O)[O-])c1)c1ccc2c(c1)Nc1ccccc1S2	0	Train
TNKS1_ML_1403	O=C([O-])c1ccc([NH+]2CCC3(CCCCC3)CC2)o1	0	Train
TNKS1_ML_1404	CC1(C)OC(=O)N(c2ccc(-c3cnc(N)c(-c4ncccn4)c3)cc2)[C@H]1c1ccccc1	1	Train
TNKS1_ML_1405	COC(=O)c1ccc(-c2nc3c(c(=O)[nH]2)COCC3)cc1	1	Train
TNKS1_ML_1406	CC(C)n1cc(C(=O)C2CCN(C(=O)CCCc3nc4c(ccn4C)c(=O)[nH]3)CC2)cn1	1	Train
TNKS1_ML_1407	CC1(C)NC(=O)N(CCCC(=O)N2CCCN(c3ccc(C(F)(F)F)cn3)CC2)C1=O	0	Train
TNKS1_ML_1408	COc1cc([C@H]2C(C(=O)OC[C@@H]3CCCO3)=C(C)NC3=C2C(=O)C[C@@H](c2cccs2)C3)cc(OC)c1OC	0	Train
TNKS1_ML_1409	C[C@@H](CC#N)N(C)c1nc(Cl)nc(Cl)n1	0	Train
TNKS1_ML_1410	Cn1c(=O)c2c(nc(N/N=C\c3ccc(Br)cc3)n2Cc2ccc(Cl)cc2)n(C)c1=O	0	Train
TNKS1_ML_1411	O=C(Nc1ccc2c(c1)CCC2)Nc1nc2c(s1)CCCC2	0	Train
TNKS1_ML_1412	Cc1nc2cc(N(C)CC(=O)N(C)C)c(N)cc2s1	0	Train
TNKS1_ML_1413	CN(C)c1ccc(/C=c2\s/c(=C(\C#N)C(=O)NCCN3CCOCC3)n(Cc3ccco3)c2=O)cc1	0	Train
TNKS1_ML_1414	Cc1ccc(O)c(-c2n[nH]c3c2[C@H](c2ccc(F)cc2)N(Cc2cccnc2)C3=O)c1	0	Train
TNKS1_ML_1415	CCCCNC(=O)c1cn(C(C)C)cc(C(=O)N2CCN(c3ccccc3)C(=O)C2)c1=O	0	Train
TNKS1_ML_1416	N#Cc1ccc(Cl)cc1Nc1cccc(C(=O)[O-])n1	0	Train
TNKS1_ML_1417	CCc1ccc(-c2nn(C[NH+]3CCN([C@H]4CCS(=O)(=O)C4)CC3)c(=S)[nH]2)cc1	0	Train
TNKS1_ML_1418	Cc1c(C(=O)NC(C)(C)C)oc2c1[C@@H](O)CC(C)(C)C2	0	Train
TNKS1_ML_1419	CNS(=O)(=O)c1ccc([NH+]2CC[C@@H](C(=O)[O-])[C@H]2C)cc1	0	Train
TNKS1_ML_1420	Cc1ccc(S(=O)(=O)Oc2ccc([C@]3(C)CC(C)(C)NC(=S)N3)c(OS(=O)(=O)c3ccc(C)cc3)c2)cc1	0	Train
TNKS1_ML_1421	COc1cc(C(=O)N(Cc2ccc(S(C)(=O)=O)cc2)c2cccc[nH+]2)cc(OC)c1OC	0	Train
TNKS1_ML_1422	CCC[NH2+][C@@H]1CCCc2sc(N3CCS(=O)(=O)CC3)nc21	0	Train
TNKS1_ML_1423	CC(C)CCn1c(N)c(S(=O)(=O)c2ccc(Cl)cc2)c2nc3ccccc3nc21	0	Train
TNKS1_ML_1424	O=C(OCc1cc2c(cc1Br)OCCO2)c1ccc(O)cc1O	0	Train
TNKS1_ML_1425	COc1ccc(CC(=O)NCCN2C(=O)S/C(=C/c3ccc(F)cc3)C2=O)cc1OC	0	Train
TNKS1_ML_1426	Cc1ccc(NC(=O)[C@H]2CCCN(S(=O)(=O)c3ccc4c(c3)CCCC(=O)N4)C2)cc1F	0	Train
TNKS1_ML_1427	COc1cc(C(=O)N2CCc3nc(NC(=O)c4cccs4)sc3C2)c([N+](=O)[O-])cc1OC	0	Train
TNKS1_ML_1428	Cc1cc(C[C@]2(O)CCCCC2(C)C)n(C)n1	0	Train
TNKS1_ML_1429	Cc1nn(C)cc1[C@@H](C)Nc1ncnc2sccc12	0	Train
TNKS1_ML_1430	COc1ccc(Sc2cc3c(cc2[N-	0	Train

	]S(=O)(=O)c2ccc(C)c(F)c2)n(C)c(=O)n3C)cc1		
TNKS1_ML_1431	O=C(CS[C@@H]1NN=C(c2cccc(S(=O)(=O)N3CCCC3)c2)N1c1ccccc1)C1=NC=CC1	0	Train
TNKS1_ML_1432	Cc1nc(C)c(Cc2ccccc2)c(N2CCN(C[C@H](O)C[NH+]3CCOCC3)CC2)n1	0	Train
TNKS1_ML_1433	CCOc1cc([C@@H]2C(C(=O)c3ccc4c(c3)C[C@@H](C)O4)=C(O)C(=O)N2CCC[NH+](C)C)ccc1OCCC(C)C	0	Train
TNKS1_ML_1434	Fc1nc2[nH]ccc2cc1Br	0	Train
TNKS1_ML_1435	O=S(=O)(c1ccc2c(c1)[C@H]1C=CC[C@H]1[C@@H](c1cccs1)N2)N1CCOCC1	0	Train
TNKS1_ML_1436	CN1CCC(Oc2cc(F)c3c(c2)N(C)C(=O)C32CCN(c3nc4c(c(=O)[nH]3)CCCN4C)CC2)CC1	1	Train
TNKS1_ML_1437	Cc1ccc([C@H]2C(C#N)=C(N)N(c3ccc(Cl)cc3[N+](=O)[O-])C3=C2C(=O)CCC3)c(C)c1	0	Train
TNKS1_ML_1438	CCOC(=O)c1[nH]c2ccc(F)cc2c1NC(=O)[C@H](C)N1CCN(Cc2ccc3c(c2)OCO3)CC1	0	Train
TNKS1_ML_1439	O=C1CC[C@@]2(C(=O)Nc3nc4c(s3)CCC4)Sc3ccccc3N12	0	Train
TNKS1_ML_1440	CSCC[C@H](NS(=O)(=O)c1ccccc1F)C(=O)N1CCN(C(=O)N2CCCCC2)CC1	0	Train
TNKS1_ML_1441	C[C@@H]1Cc2cc(S(N)(=O)=O)ccc2N1Cc1cccc(S(=O)(=O)N2CCOCC2)c1	0	Train
TNKS1_ML_1442	O=C(NCCCN1CC[NH+](C2CCCCC2)CC1)[C@@H]1CCCN(S(=O)(=O)N2CCCCC2)C1	0	Train
TNKS1_ML_1443	O=C(NCc1ccc(Cl)s1)c1nnc([O-])c2ccccc12	0	Train
TNKS1_ML_1444	CC1=NN(c2ccc(S(N)(=O)=O)cc2)[C@H](c2ccco2)C1	0	Train
TNKS1_ML_1445	COc1ccc(CN2CCN([C@@H](C)c3nc(N)nc(Nc4ccccc4)n3)CC2)cc1F	0	Train
TNKS1_ML_1446	CC(C)(C)[C@@]1(O)CC(C(F)(F)F)=NN1C(=O)c1ccc(O)cc1	0	Train
TNKS1_ML_1447	Clc1cccc(N/N=C/c2c[nH]c3ccc(Br)cc23)n1	0	Train
TNKS1_ML_1448	O=C(NCc1cccc[nH+]1)c1nn(-c2ccc(C(F)(F)F)cc2)c(=O)n(Cc2ccc(F)cc2)c1=O	0	Train
TNKS1_ML_1449	C[C@@H]1CCCC[C@@H]1NC(=O)[C@@H]1CCc2nc[nH]c2C1	0	Train
TNKS1_ML_1450	CC(C)C[n+]1c(N)c(/C([O-])=N/c2nccs2)cc2c(=O)n3ccccc3nc21	0	Train
TNKS1_ML_1451	CC(C)Oc1ccc(C(=O)C2CCN(CC(=O)NCc3nc4c(c(=O)[nH]3)COCC4)CC2)cc1	1	Train
TNKS1_ML_1452	O=c1[nH]c(-c2ccc(C(F)(F)F)cc2)nc2c1CCSC2	1	Train
TNKS1_ML_1453	CC(C)(C)NS(=O)(=O)c1ccc(-c2cc(O)cc(F)c2)cc1	0	Train
TNKS1_ML_1454	Cc1cccc(C(=O)N2[C@@H](C(=O)NC3CC3)COC23CCN(C(=O)C(C)(C)C)CC3)c1	0	Train
TNKS1_ML_1455	Cc1ccc(C)c(S(=O)(=O)NCC(=O)N2CC(S(=O)(=O)c3ccc(Cl)cc3)C2)c1	0	Train
TNKS1_ML_1456	COc1ccc(NC(=O)[C@H]2CCCN(c3ccc4nnc(C(F)(F)F)n4n3)C2)cc1Cl	0	Train
TNKS1_ML_1457	CN(Cc1ccc2c(c1)OCCO2)C(=O)CN1C(=O)N[C@@](C)(c2ccc(Cl)cc2Cl)C1=O	0	Train
TNKS1_ML_1458	CCCN1CCN(C(=O)Cn2cnc3sc(C)c(S(=O)(=O)N4C[C@@H](C)C[C@@H](C)C4)c3c2=O)CC1	0	Train
TNKS1_ML_1459	O=C1CCc2cc(S(=O)(=O)N[C@H](C(=O)Nc3ccccc3F)c3ccccc3)ccc2N1	0	Train
TNKS1_ML_1460	O=C(CS(=O)(=O)c1cn(CC(=O)N2CCCCCC2)c2ccccc12)Nc1ccc(OC(F)(F)F)cc1	0	Train
TNKS1_ML_1461	O=C(NCCc1c[nH]c2ccccc12)[C@H]1[C@@H]2C(=O)N(Cc3cc[nH+]cc3)C[C@]23C=C[C@H]1O3	0	Train
TNKS1_ML_1462	COc1ccc([C@H]2CC(c3cccs3)=NN2C(=O)CSc2nc(=O)c3cnn(-c4ccc(S(N)(=O)=O)cc4)c3[nH]2)cc1	0	Train

TNKS1_ML_1463	Cc1[nH+]n(C)c2sc(C(=O)N[C@@H]3CC[C@H](C)[C@@H]3C)c(N)c12	0	Train
TNKS1_ML_1464	CCCN1C(=O)[C@](NS(=O)(=O)c2ccc(NC(C)=O)cc2)(C(F)(F)F)C(C(=O)OC)=C1C	0	Train
TNKS1_ML_1465	Nc1ccc2c(c1)CC[C@@H]2NCc1nnc2ccccn12	0	Train
TNKS1_ML_1466	CC1(C)[C@H]2C[NH2+]C[C@H]2CN1C(=O)c1cc(Cl)c(Cl)[n-]1	0	Train
TNKS1_ML_1467	CC1(C)CC2=NNC(=O)c3cccc4[nH]c(c2c34)C1	1	Train
TNKS1_ML_1468	O=c1[nH]c2sccc2c2ccccc12	1	Train
TNKS1_ML_1469	COc1ccc(-n2c(OCc3nc(-c4ccc(C)cc4)no3)nnc2-c2ccncc2)cc1	1	Train
TNKS1_ML_1470	Cc1cccc(-c2nnc([C@H]3C[C@H](NC(=O)c4ccnc5cc(F)cnc45)C3)n2-c2ccccc2F)n1	1	Train
TNKS1_ML_1471	CC[N+]1=C(Nc2ccc(C(=O)[O-])cc2)S/C(=C\c2cccc([N+](=O)[O-])c2)C1=O	0	Train
TNKS1_ML_1472	Cc1sc2ncnc(N[C@H]3CCCc4ccccc43)c2c1C	0	Train
TNKS1_ML_1473	N#Cc1cccc(-c2cnc([C@H]3CCCCCN3)[nH]2)c1	0	Train
TNKS1_ML_1474	Cc1cc(C[C@H](N[NH3+])c2cc3c(s2)CCCCC3)n(C)n1	0	Train
TNKS1_ML_1475	C[C@H](Cl)C(=O)NC(=O)N1c2ccccc2CC[C@@H]1C	0	Train
TNKS1_ML_1476	Cn1cc(Cl)cc1C(=O)Nc1ccc(Cl)c([O-])c1	0	Train
TNKS1_ML_1477	N#C/C(=C\c1ccc(-c2ccc(C(=O)[O-])c(Cl)c2)o1)C(=O)Nc1cccc(Br)c1	0	Train
TNKS1_ML_1478	O=C(N/N=C/c1ccc(I)o1)c1ccc2c(c1)OCO2	0	Train
TNKS1_ML_1479	Cc1ccc([C@@H](NC(=O)c2cccc(S(=O)(=O)N3CC[NH+](C)CC3)c2)c2ccccc2)cc1	0	Train
TNKS1_ML_1480	C[C@H]1CCc2ccccc2N1C(=O)c1cc(N)cn1C	0	Train
TNKS1_ML_1481	Cc1nn([C@H]2CCO[C@H](C(C)C)C2)c(N)c1Br	0	Train
TNKS1_ML_1482	COc1cccc([C@H]2CN=C(N)N2c2cccc(Br)c2)c1	0	Train
TNKS1_ML_1483	CCc1ccc2nc(NC(=O)c3ccc([O-])nn3)sc2c1	0	Train
TNKS1_ML_1484	CC(C)Oc1cncc(N2C[C@H]3CC[C@H]([NH3+])C[C@H]3C2)n1	0	Train
TNKS1_ML_1485	CN1CCCc2c1nc(N1CCC3(CC1)C(=O)N(C)c1cc(F)cc(F)c13)[nH]c2=O	1	Train
TNKS1_ML_1486	Cc1ccoc1[C@@H](O)c1ccc2nc([O-])oc2c1	0	Train
TNKS1_ML_1487	Cc1nn(C(C)C)c(N2C[C@@H](C)N[C@H](C)C2)c1[N+](=O)[O-]	0	Train
TNKS1_ML_1488	COc1ccc([C@H]2[C@H](C(=O)Nc3ccc(F)cc3F)c3cc(OC)c(OC)cc3C(=O)N2C)cc1OC	0	Train
TNKS1_ML_1489	COc1ccc(-c2ccnc3nc(N4CCc5ccccc54)nc([O-])c23)cc1OC	0	Train
TNKS1_ML_1490	COc1cc([C@@H]2[C@H](C(=O)c3ccc(Cl)cc3)C(=O)C(=O)N2CCCn2cc[nH+]c2)ccc1O	0	Train
TNKS1_ML_1491	Cc1ccnc2c(NC(=O)c3ccc(N4C(=O)[C@H]5[C@H]6C=C[C@H](C6)[C@H]5C4=O)cc3)cccc12	1	Train
TNKS1_ML_1492	C[C@@H]1c2nc(NC(=O)c3ccc(CN4CCN(C)CC4)cc3)sc2C[C@]2(C)CC[C@H]([C@H](C)C(=O)NCCN3CCOCC3)[C@H](O)[C@@H]12	0	Train
TNKS1_ML_1493	C1CCC2(C1)C[C@H](c1nn[nH]n1)CCO2	0	Train
TNKS1_ML_1494	N/C(=N\C(=O)c1ccc(Cl)nc1)c1cc(Cl)cc(Cl)c1	0	Train
TNKS1_ML_1495	C[C@@H]1Cc2ccccc2N(C(=O)c2[nH]ncc2Br)C1	0	Train
TNKS1_ML_1496	COc1ccc(C(=O)C2CCN(C3CCN(Cc4nc5ccccc(=O)c5[nH]4)C3=O)CC2)cc1	1	Train
TNKS1_ML_1497	Cc1cccc2c(=O)[nH]c(-c3ccc(Br)cc3)nc12	1	Train
TNKS1_ML_1498	CC[C@@H]1CCC[C@@](O)([C@@H]2CCOC3(CCOCC3)C2)CC1	0	Train
TNKS1_ML_1499	Cc1ccc2c(c1C)c(=O)n(C[C@H]1CC[C@H](C(=O)O)CC1)c(=O)n2Cc1cccc(C(F)(F)F)c1C#N	1	Train
TNKS1_ML_1500	C=CCNS(=O)(=O)[C@H]1S[C@@H]2CN(C[C@@H]3C=	0	Train

	NN=C3c3ccc(F)cc3)CCC2=C1C(=O)OC		
TNKS1_ML_1501	Cc1ccc(N2CCN(c3ccc(C)cc3)C2c2ccc(C)o2)cc1	0	Train
TNKS1_ML_1502	Cc1ccc(N2C(=O)CCS2(=O)=O)cc1S(=O)(=O)NC[C@@H](c1ccccc1)[NH+](C)C	0	Train
TNKS1_ML_1503	COc1ccc([C@@H]2C(C(=O)c3c(C)[nH]c(C)c3C)=C([O-])C(=O)N2CC[NH+](C)C)cc1	0	Train
TNKS1_ML_1504	C[C@H]1[NH+]=c2ccccc2=C1C(=O)[C@@H](C)OC(=O)CCNC1=NS(=O)(=O)c2ccccc21	0	Train
TNKS1_ML_1505	C[NH+]1CCc2nc([S-])c(C#N)c(/C=C/c3ccccc3)c2C1	0	Train
TNKS1_ML_1506	O=c1[nH]c(N2CCC3(CC2)CN(CCO)c2ccccc23)nc2c1CCCC2	1	Train
TNKS1_ML_1507	O=C(CCNC1=NS(=O)(=O)c2ccccc21)N1CCN(Cc2ccc3c(c2)OCO3)CC1	0	Train
TNKS1_ML_1508	CCN(CC)C(=O)COc1ccc([C@H]2C3=C(CC(C)(C)CC3=O)Nc3ncnn32)cc1OC	0	Train
TNKS1_ML_1509	C[NH+](C)CCN(C(=O)CCS(=O)(=O)c1ccc(F)cc1)c1nc2c(S(C)(=O)=O)cccc2s1	0	Train
TNKS1_ML_1510	O=C(/C=C/c1ccc([N+](=O)[O-])cc1)NC(=S)NNC(=O)c1ccccc1Br	0	Train
TNKS1_ML_1511	CCCN1C(=O)c2n[nH]c(-c3cc(Cl)ccc3O)c2[C@H]1c1cc(OC)c(OC)c(OC)c1	0	Train
TNKS1_ML_1512	COc1ccc(C(=O)C2CCN([C@@H]3CCCN(CCC4NC(=O)C5=C(CCOC5)N4)C3=O)CC2)cc1	1	Train
TNKS1_ML_1513	Cc1cc(Cl)c(NC(=O)N(C)Cc2cnccn2)c2cccnc12	0	Train
TNKS1_ML_1514	COc1ccc(OC)c([C@H]2/C(=C(/O)c3ccco3)C(=O)C(=O)N2CCCn2cc[nH+]c2)c1	0	Train
TNKS1_ML_1515	CCOc1cc([C@@H]2SC(NC(C)=O)=NN2C(C)=O)cc(Br)c1OS(C)(=O)=O	0	Train
TNKS1_ML_1516	COc1cc([C@@H]2C(=C(O)c3ccc(C)cc3)C(=O)C(=O)N2c2cccnc2)ccc1O	0	Train
TNKS1_ML_1517	CCc1ccccc1N(C)S(=O)(=O)c1ccc2[nH]cc(C(=O)NC(C)C)c(=O)c2c1	0	Train
TNKS1_ML_1518	CC[C@H](C)[NH+]1CCN(S(=O)(=O)c2ccsc2C(=O)[O-])CC1	0	Train
TNKS1_ML_1519	CC(C)c1nc(=S)c2c3c(sc2[nH]1)CCCC3	0	Train
TNKS1_ML_1520	COc1ccc(-n2c(N)nc3ccc(C)cc32)cc1Br	0	Train
TNKS1_ML_1521	Cc1cc([C@@H](O)c2cc(Cl)c3c(c2)OCO3)ccc1F	0	Train
TNKS1_ML_1522	Nc1ccc2nc(Cc3ccsc3)oc2c1	0	Train
TNKS1_ML_1523	O=C(NNS(=O)(=O)c1ccc2c(c1)OCCO2)c1c[nH+]cn1-c1ccccc1	0	Train
TNKS1_ML_1524	O=c1[nH]nc2c3c(cccc13)NC1=C2CCC1	1	Train
TNKS1_ML_1525	C[C@H](NC(=O)CSc1sc2c(=O)[nH]c(=O)[nH]c2c1C#N)C12CC3CC(CC(C3)C1)C2	0	Train
TNKS1_ML_1526	Cc1ccc2c(=O)[nH]c(-c3ccc(C(C)(C)OCCO)cc3)cn12	1	Train
TNKS1_ML_1527	Cc1cc(=O)[nH]c2cc(-c3ccccc3F)ccc12	1	Train
TNKS1_ML_1528	O=c1[nH]nc2c3c(cccc13)NC1=C2CCCCC1	1	Train
TNKS1_ML_1529	CC1CCC(NC(=O)c2cc([N+](=O)[O-])ccc2Cl)CC1	0	Train
TNKS1_ML_1530	N#Cc1cc2c(nc1Oc1cc(N)ccc1Cl)CCC2	0	Train
TNKS1_ML_1531	COc1ccc(N2C[C@@H](C(=O)N[C@@H](C)C(=O)N[C@@H](C)c3ccccc3)CC2=O)cc1	0	Train
TNKS1_ML_1532	COc1ccc([C@H]2C(C#N)=C(N)C(C#N)(C#N)C3=CCSC[C@@H]32)cc1	0	Train
TNKS1_ML_1533	COc1ccc(Br)c(-n2c(N)nc3cnccc32)c1	0	Train
TNKS1_ML_1534	CC(=O)C(=Cc1ccc(O)c([N+](=O)[O-])c1)C(C)=O	0	Train
TNKS1_ML_1535	Cc1ccc(Oc2nc(N3CCN(C)CC3)nc3c2CN(S(=O)(=O)c2ccccc2C#N)CC3)cc1	0	Train
TNKS1_ML_1536	O=C(c1ccc(F)cc1)C1CCN(CC(=O)N(CCF)Cc2nc3c(c(=O)[nH]2)COCC3)CC1	1	Train

TNKS1_ML_1537	CCOC(=O)N1CC[NH+](CN2C(=O)/C(=N\NC(=S)Nc3c(CC)cccc3CC)c3ccccc32)CC1	0	Train
TNKS1_ML_1538	O=C1C[C@@H](NC(=O)N2CCn3cccc3[C@H]2c2ccc(Cl)cc2)CN1c1ccc2c(c1)OCCO2	0	Train
TNKS1_ML_1539	C[C@@H]1CN(C(=O)N[C@@H]2CCC[C@H](C(=O)[O-])C2)C[C@@H]1C	0	Train
TNKS1_ML_1540	O=C1N[C@H](c2ccc3c(c2)OCCCO3)CO1	0	Train
TNKS1_ML_1541	[NH3+][C@H]1C=C[C@H](c2nc(Cc3ccc(O)cc3)no2)C1	0	Train
TNKS1_ML_1542	O=C(CCc1cccnc1)N(Cc1nc2c(c(=O)[nH]1)COCC2)Cc1cccs1	1	Train
TNKS1_ML_1543	O=[N+]([O-])[C@@H]1[C@@H]2C=C[C@@H](C2)[C@@H]1c1ccco1	0	Train
TNKS1_ML_1544	[H]/N=C1\C(C#N)C2=CC[NH+](CC)C[C@@H]2[C@H]([C@H]2C=CCCC2)C1(C#N)C#N	0	Train
TNKS1_ML_1545	CSc1cnn(CC(C)C)c1N	0	Train
TNKS1_ML_1546	Oc1ccccc1/C=N/Nc1nc(-c2ccc(F)cc2)cs1	0	Train
TNKS1_ML_1547	Cc1ccn2/c(=N/C(=O)[C@@H]3C[C@H]3c3ccc(C)s3)[nH]nc2c1	0	Train
TNKS1_ML_1548	COc1cc([C@@H]2c3c(oc4ccc(Cl)cc4c3=O)C(=O)N2c2nnc(C)s2)ccc1O	0	Train
TNKS1_ML_1549	CCn1cc([C@]2(O)CCC[C@H](C)[C@@H]2C)cn1	0	Train
TNKS1_ML_1550	Cc1ccc([C@@H]2C(C#N)=C(N)Oc3c2c(C)nn3-c2ccc(C)cc2)cc1	0	Train
TNKS1_ML_1551	Cc1cc(N2C[C@H](C)N[C@H](C)C2)ncc1-c1ccc(-c2nc3c(ccn3C)c(=O)[nH]2)cc1	1	Train
TNKS1_ML_1552	Nc1cc(Cl)ccc1C(=O)N1CCO[C@@H]2CCCC[C@@H]21	0	Train
TNKS1_ML_1553	Cc1c(C#N)c(NC(=O)COC(=O)c2ccc(S(C)(=O)=O)cc2)n(-c2ccccc2)c1C	0	Train
TNKS1_ML_1554	CC1(C)COCCN1[C@@H]1CCCCC[C@@H]1C#N	0	Train
TNKS1_ML_1555	CC(C)C(=O)N1CCN(c2[nH+]c3c(s2)C[C@H](C(=O)NC2CC[NH+](C)CC2)CC3)CC1	0	Train
TNKS1_ML_1556	CCn1cc(S(=O)(=O)N2CCN(C(=O)CCS(=O)(=O)c3ccc(C)cc3)CC2)c(C)n1	0	Train
TNKS1_ML_1557	Cc1cccc2c(=O)[nH]c(CCC(=O)Nc3ccc(C(=O)Nc4cccc5cccnc45)cc3)nc12	1	Train
TNKS1_ML_1558	CCOc1ccc2nc(Sc3ccc([C@@H]4NC(=O)[C@H]5CCCC[C@@H]5N4)cc3[N+](=O)[O-])sc2c1	0	Train
TNKS1_ML_1559	O=c1[nH]c(-c2ccccc2)cc2ccncc12	1	Train
TNKS1_ML_1560	CC(C)(O)c1ccc(-c2nc3ccccc3c(=O)[nH]2)cc1	1	Train
TNKS1_ML_1561	COc1ccc(N2CCN(c3nc4c(c(=O)[nH]3)CCCC4)CC2)cc1	1	Train
TNKS1_ML_1562	CC[C@H](C)NC(=O)CN1C(=O)CS[C@@H](c2ccc3c(c2)OCO3)c2c(-c3ccccc3)nn(-c3cccc(C)c3)c21	0	Train
TNKS1_ML_1563	Cc1noc(C)c1S(=O)(=O)N1CCC(C(=O)Nc2nc(CN3C[C@H](C)O[C@H](C)C3)cs2)CC1	0	Train
TNKS1_ML_1564	CCn1nc(C)c(N)c1Oc1cc(C)ccc1C(C)C	0	Train
TNKS1_ML_1565	CS(=O)(=O)Nc1cn[nH]c1[C@@H]1CCCN(Cc2cn3cc(Cl)ccc3n2)C1	0	Train
TNKS1_ML_1566	Cc1nn(-c2ccc(C#N)c(N)c2)c(C)c1Cl	0	Train
TNKS1_ML_1567	CCCCNC(=O)NC1CCC([NH3+])CC1	0	Train
TNKS1_ML_1568	Cc1ccc(C)c2c1[C@@H](NC(C)C)[C@@H](C)[C@H](C)S2(=O)=O	0	Train
TNKS1_ML_1569	CC1(C)CCC[C@@](C#N)(N2CCNCC2)CC1	0	Train
TNKS1_ML_1570	O=C(CSc1nc(O)c2nc[nH]c2n1)c1ccc(Br)cc1	0	Train
TNKS1_ML_1571	COc1cccc([C@H]2C3=C(C[C@@H](c4ccc(O)cc4)CC3=O)Nc3onc(C)c32)c1	0	Train
TNKS1_ML_1572	Cc1cc([N-]S(=O)(=O)c2ccc(N3C(=O)CCS3(=O)=O)cc2C)ccc1Br	0	Train
TNKS1_ML_1573	CN1c2ccc(Cl)cc2-c2nc(SCC(=O)Nc3nccs3)ncc2S1(=O)=O	0	Train

TNKS1_ML_1574	O=C(CCCc1nc2ccccc2s1)OCC(=O)N1CCN(S(=O)(=O)c2ccc(Cl)cc2)CC1	0	Train
TNKS1_ML_1575	Cc1ccc(/C=C2/SC(=O)N(CCNC(=O)[C@@H]3CS[C@@]4(C)CCC(=O)N34)C2=O)cc1	0	Train
TNKS1_ML_1576	Cc1csc(NC(=O)c2noc3c2CCc2sccc2-3)n1	0	Train
TNKS1_ML_1577	O=c1ccc(S(=O)(=O)[N-]c2c(Cl)cccc2Cl)c[nH]1	0	Train
TNKS1_ML_1578	Cc1cnc(Cl)nc1N[C@@H]1CCCCC[C@H]1C	0	Train
TNKS1_ML_1579	Cc1ccc(-c2cc(C(=O)N3CCc4c([nH]c5ccccc45)[C@H]3c3cccc[nH+]3)[nH]n2)s1	0	Train
TNKS1_ML_1580	CC(C)(C)c1cc(C(=O)N2CCC(O)(C(F)(F)F)CC2)n[nH]1	0	Train
TNKS1_ML_1581	O=C(c1ccn[nH]1)N1CCC[C@@H](c2cncc(-c3cccc(C(F)(F)F)c3)n2)C1	0	Train
TNKS1_ML_1582	O=C(CN1CCC(c2nnc(-c3ccc(F)cc3)o2)CC1)NCc1nc2c(c(=O)[nH]1)COCC2	1	Train
TNKS1_ML_1583	Cn1c(=O)oc2cc(S(=O)(=O)N3CCC[C@H](C(=O)Nc4ccc(Cl)cc4)C3)ccc21	0	Train
TNKS1_ML_1584	COc1ccc(C(=O)Nc2ccc(C(=O)N(c3ccncn3)c3ccccc3Cl)cc2)cc1	1	Train
TNKS1_ML_1585	Cc1ccccc1NC(=O)c1ccc(NC(=O)N2CCN(C(=O)c3cc(Cc4n[nH]c(=O)c5ccccc45)ccc3F)CC2)cc1	1	Train
TNKS1_ML_1586	CN1CCCc2c1nc(N1CCC3(CC1)C(=O)N(CS(C)(=O)=O)c1cc(F)cc(F)c13)[nH]c2=O	1	Train
TNKS1_ML_1587	C[C@@H]1CC[C@H](c2nc(N)c3c(n2)CCCC3)C1	0	Train
TNKS1_ML_1588	CN1CCCc2c1cc(-c1ccccc1)[nH]c2=O	1	Train
TNKS1_ML_1589	C[C@@H]([NH3+])Cc1noc(-c2ccc([NH+](C)C)cc2)n1	0	Train
TNKS1_ML_1590	O=C(CCCS(=O)(=O)c1ccc(Cl)cc1)Nc1nnc(CCS(=O)(=O)c2ccccc2)o1	0	Train
TNKS1_ML_1591	C[C@@]1(c2ccc3c(c2)OCO3)NC(=O)N(Cc2ccc(F)cc2Br)C1=O	0	Train
TNKS1_ML_1592	C[C@@H](NC(=O)CSc1nnc(CN(c2ccccc2)S(C)(=O)=O)n1C)c1ccccc1	0	Train
TNKS1_ML_1593	Cc1c(NC(=O)N(C)c2ccccc2)cccc1C(=O)[O-]	0	Train
TNKS1_ML_1594	Cc1cc(-c2nnc3ccc(N)cn23)sc1C	0	Train
TNKS1_ML_1595	Cc1nc2sccn2c1CNc1c(Cl)ccc2nsnc12	0	Train
TNKS1_ML_1596	CN1C(=O)[C@@H](CC(=O)Nc2ccc(-c3ccccc3)cc2)N(Cc2cccc[nH+]2)C1=S	0	Train
TNKS1_ML_1597	O=C(Nc1nccs1)c1sc2c([N+](=O)[O-])cccc2c1Cl	0	Train
TNKS1_ML_1598	O=C(CCc1cccc(F)c1)N(Cc1nc2c(c(=O)[nH]1)COCC2)Cc1cccs1	1	Train
TNKS1_ML_1599	COc1ccccc1N1CCN(c2nc3c(c(=O)[nH]2)CNCC3)CC1	1	Train
TNKS1_ML_1600	[O-]c1nc(CNc2ccc(F)cc2Cl)nc2ccsc12	0	Train
TNKS1_ML_1601	FC(F)(F)c1cccc2nc(Cl)cnc12	0	Train
TNKS1_ML_1602	Cc1ccc[nH]/c1=N\S(=O)(=O)c1ccc2c(c1)CCO2	0	Train
TNKS1_ML_1603	C1CCC2(CC1)CN([C@@H]1CCOC1)CCCN2	0	Train
TNKS1_ML_1604	O=C(CNS(=O)(=O)c1ccc2c(c1)OCCCO2)N1CCN(Cc2cccc3ccccc23)CC1	0	Train
TNKS1_ML_1605	Cc1ncsc1[C@H](C)Nc1cc2c(cc1Cl)NC(=O)CO2	0	Train
TNKS1_ML_1606	O=[N+]([O-])c1ccc(O[C@@H]2O[C@H](CO)[C@@H](O[C@@H]3O[C@H](CO)[C@@H](O)[C@H](O)[C@H]3O)[C@H](O)[C@H]2O)c(Cl)c1	0	Train
TNKS1_ML_1607	Cc1[nH+]n([C@H]2CCS(=O)(=O)C2)c(C)c1CN(C)Cn1nc(Nc2ccc(F)cc2)sc1=S	0	Train
TNKS1_ML_1608	O=C(O)CCCCN1C(=O)C[C@@H](N2C(=O)/C(=C/c3ccc(Cl)cc3)SC2=S)C1=O	0	Train
TNKS1_ML_1609	COc1ccc(C(=O)C2CCN(C3CCN(CC(=O)Nc4nnnn4C)C3=O)CC2)cc1	1	Train

TNKS1_ML_1610	COCCCN1C(=O)/C(=C/c2c(N3CC[NH+](Cc4ccc5c(c4)OCO5)CC3)nc3ccccn3c2=O)SC1=S	0	Train
TNKS1_ML_1611	CC(C)CC1=C[C@@H]([C@@H]2Nn3c(nnc3-c3ccccn3)S2)N=N1	0	Train
TNKS1_ML_1612	Cc1ccc2c(=O)[nH]c(CCCC(=O)N3CCC(Oc4ccc(C#N)cc4)CC3)nn12	1	Train
TNKS1_ML_1613	Cc1cccc(N2CCC(NS(=O)(=O)c3ccccc3OC(F)(F)F)CC2)n1	0	Train
TNKS1_ML_1614	N#C[C@]1(NC(=O)c2ccn(-c3ccccc3Cl)n2)CCSC1	0	Train
TNKS1_ML_1615	NC(=O)c1cccc2cn(-c3ccc([C@@H]4CCCNC4)cc3)nc12	1	Train
TNKS1_ML_1616	COc1ccc(N(CC(=O)N/N=C\c2cc(C)n(-c3ccc(C)cc3)c2C)S(C)(=O)=O)cc1OC	0	Train
TNKS1_ML_1617	O=C1CCS(=O)(=O)N1c1ccc(S(=O)(=O)Nc2ccc(Br)cc2)cc1	0	Train
TNKS1_ML_1618	CC(C)c1[nH]c(S(=O)(=O)N2C[C@@H](C)Sc3ccccc32)c[nH+]1	0	Train
TNKS1_ML_1619	COc1c2c(cc3c1[C@@H](c1c([O-])n(-c4ccccc4)c(=S)n(-c4ccccc4)c1=O)[NH+](C)CC3)OCO2	0	Train
TNKS1_ML_1620	COc1ccc(S(=O)(=O)N(C)C)cc1NC(=O)c1cc(S(=O)(=O)N2CCOCC2)cn1C	0	Train
TNKS1_ML_1621	Cc1cc(N)cc(S(=O)(=O)N[C@@H]2COc3ccccc32)c1	0	Train
TNKS1_ML_1622	COCCN1C(=O)/C(=C\c2cccn2-c2ccc(O)cc2)C(=O)N(c2ccccc2)C1=O	0	Train
TNKS1_ML_1623	CC(C)c1nc(-c2cc(N)c(F)cc2F)n[nH]1	0	Train
TNKS1_ML_1624	O=c1[nH]nc2c3c(cccc13)NC1=C2CCNC1	1	Train
TNKS1_ML_1625	Cn1c(=O)oc2cc(S(=O)(=O)NCc3cccc(Cn4ccnc4)c3)ccc21	0	Train
TNKS1_ML_1626	CC[C@]1(C(=O)[O-])CCCN(C(=O)N[C@@H]2CCC[NH+](C)C2)C1	0	Train
TNKS1_ML_1627	Cc1cc(SCC(=O)Nc2ccc3c(c2)OCO3)nc2ccc(S(=O)(=O)N(C)C)cc12	0	Train
TNKS1_ML_1628	[H]/N=C1C(=C/c2cc(C)n(-c3cc(Cl)c(OC)cc3OC)c2C)/C(=O)N=C2SC(CCCC)=NN2/1	0	Train
TNKS1_ML_1629	N#Cc1ccc2c(c1)oc(=O)n2[C@H]1C[C@H](c2nnc(-c3ccncn3)n2-c2ccccc2Cl)C1	1	Train
TNKS1_ML_1630	C[C@H](NC(=O)NCc1ccnc(N2CC[NH+](C)CC2)c1)c1ccc(Cl)cc1Cl	0	Train
TNKS1_ML_1631	O=C(C[C@@H]1CCN2C[C@@H]1CCCOc1ccccc1CN(C(=O)C1CCCC1)CC2=O)N1CCOCC1	0	Train
TNKS1_ML_1632	CSc1nsc(S[C@H](C(=O)Nc2cc(C)on2)c2ccccc2)n1	0	Train
TNKS1_ML_1633	O=C1/C(=C/c2c(NCCCn3ccnc3)nc3ccccn3c2=O)SC(=S)N1C1CCCC1	0	Train
TNKS1_ML_1634	C/C([O-])=N/S(=O)(=O)c1ccc(NC(=O)c2ccc(-n3ccnc3C)nn2)cc1	0	Train
TNKS1_ML_1635	CN(C)CCn1c2c3c4c(cc(F)cc41)C(=O)NN=C3CC(C)(C)C2	1	Train
TNKS1_ML_1636	CN(C)S(=O)(=O)c1cccc(NC(=O)NC[C@H](c2cccs2)[NH+](C)C)c1	0	Train
TNKS1_ML_1637	CCO[C@@H]1O[C@H](CO)[C@H]2OC(C)(C)O[C@@H]12	0	Train
TNKS1_ML_1638	COc1ccccc1[C@H](NC(=O)c1cc(C)no1)c1ccccn1	0	Train
TNKS1_ML_1639	CC(C)(C)C1Cc2[nH]c(=O)c3ccccc3c2CO1	1	Train
TNKS1_ML_1640	Cc1ccc(C2=NN(C(=O)CSc3nnc(CCC(N)=O)n3C)[C@H](c3ccco3)C2)cc1	0	Train
TNKS1_ML_1641	Cc1c(N)nnn1-c1ccc(Br)cc1F	0	Train
TNKS1_ML_1642	COc1cc([C@H](O)C(F)(F)F)cc(Br)c1[O-]	0	Train
TNKS1_ML_1643	C[C@H](NC(=O)NCCCN1CC[NH+](C)C[C@@H]1c1ccccc1)c1ccc(-n2cncn2)cc1	0	Train
TNKS1_ML_1644	N#Cc1cc(N)ccc1-n1cc(-c2ccccc2)cn1	0	Train
TNKS1_ML_1645	Cc1ccc([N+](=O)[O-])cc1NC(=O)C1CCN(S(=O)(=O)c2ccc(F)cc2)CC1	0	Train
TNKS1_ML_1646	Nc1nc(C2CCCCCC2)ncc1Br	0	Train

TNKS1_ML_1647	N#CC1=C(N)Oc2c(ccc3c(N)cccc23)[C@@H]1c1cccnc1	0	Train
TNKS1_ML_1648	Cc1cn2c([nH+]1)CC[C@H](N[C@@H](C)c1nc3cc(Cl)ccc3n1C)C2	0	Train
TNKS1_ML_1649	Cc1c(F)ccc2c1c(=O)n(C[C@H]1CC[C@H](C(=O)Nc3ccncc3)CC1)c(=O)n2Cc1cccc(C(F)(F)F)c1C#N	1	Train
TNKS1_ML_1650	COc1cc2c(cc1OC)[C@@H](C(=O)Nc1cc(OC)c(OC)c(OC)c1)[C@@H]1c3ccccc3CCN1C2=O	0	Train
TNKS1_ML_1651	CN[C@@H]1COC[C@H]1C(=O)Nc1nc2c(s1)CCC2	0	Train
TNKS1_ML_1652	CC1=C2C(=O)N=C(CSc3ncnc4sc5c(c34)CC[C@@H](C)C5)N=C2S[C@@H]1C(=O)[O-]	0	Train
TNKS1_ML_1653	[NH3+][C@H]1CCCC[C@H]1C(=O)NC[C@@H]1CCC[C@@H]1O	0	Train
TNKS1_ML_1654	COc1cc(/C=C/C(=O)[O-])ccc1OS(=O)(=O)c1c(C)c(C)cc(C)c1C	0	Train
TNKS1_ML_1655	COc1cc(S(=O)(=O)N2CCN(c3cc(N4CCCCC4)nc(C)n3)CC2)c(C)cc1C	0	Train
TNKS1_ML_1656	Cc1ccc2nc(N3CCN(C(=O)Cc4ccc(S(=O)(=O)C(C)C)cc4)CC3)sc2c1	0	Train
TNKS1_ML_1657	Cc1cc(C)c2c3c(sc2n1)[C@@H](c1ccncc1)C(C#N)=C(N)O3	0	Train
TNKS1_ML_1658	Cc1ccc(S(=O)(=O)NCc2cc(-c3ccccn3)n(C)n2)cc1	0	Train
TNKS1_ML_1659	O=c1[nH]c(N2CCC3(CC2)CNc2cc(Cl)ccc23)nc2c1CCCC2	1	Train
TNKS1_ML_1660	CC1(C)CN([C@H]2CCOC2)CCCN1	0	Train
TNKS1_ML_1661	N#Cc1ccc(S(=O)(=O)N[C@@H]2CCCC[C@@H]2O)cc1	0	Train
TNKS1_ML_1662	CC(C)(C)Nc1nn(C[NH+]2CCN(S(=O)(=O)c3ccc(Cl)cc3)CC2)c(=S)s1	0	Train
TNKS1_ML_1663	Cc1sc2[nH]c(=S)n([C@@H]3CC[C@H](C)C[C@@H]3C)c(=O)c2c1C	0	Train
TNKS1_ML_1664	C[C@H]1CCC[C@@H]([C@H](O)c2cn[n-]n2)C1	0	Train
TNKS1_ML_1665	Cc1cc(C)c2c(c1)sc1nncn12	1	Train
TNKS1_ML_1666	CCN(CC)S(=O)(=O)c1ccc(N2C[C@@H](C(=O)Nc3ccccc3C(=O)NC3CC3)CC2=O)cc1	0	Train
TNKS1_ML_1667	CC(=O)O[C@H]1[C@H](C)O[C@@H](Oc2ccc3ccc(=O)oc3c2-c2c(O)ccc3cc(Oc4ccc5ccc(=O)oc5c4)c(=O)oc23)[C@H](O)[C@@H]1O	0	Train
TNKS1_ML_1668	CCOc1ccc(-c2n[nH]c(=S)n2CCC(=O)N2CCCc3ccccc32)cc1	0	Train
TNKS1_ML_1669	O=C(CCc1ccccc1)N(Cc1nc2c(c(=O)[nH]1)COCC2)Cc1cncs1	1	Train
TNKS1_ML_1670	C[C@@H](Cl)C(=O)NC(=O)N[C@H]1CCc2ccccc2C1	0	Train
TNKS1_ML_1671	CN1c2ncccc2C(=O)NC1c1ccc(Cl)cc1Cl	1	Train
TNKS1_ML_1672	C/C(=N\NC(=O)CN(c1cccc(C)c1)S(=O)(=O)c1ccc(C)cc1)c1ccc(I)cc1	0	Train
TNKS1_ML_1673	CCc1c(C(=O)NCCS(=O)(=O)N2C[C@H](C)O[C@@H](C)C2)cnn1-c1ccc(C)cc1	0	Train
TNKS1_ML_1674	O=C(CN1CCC2(CC1)Cc1cc(F)ccc1C2=O)NCc1nc2c(c(=O)[nH]1)COCC2	1	Train
TNKS1_ML_1675	[H]/N=C1\O[C@@]2(C)O[C@@H](c3ccccc3)[C@]1(C#N)C(C#N)(C#N)[C@H]2c1ccccc1	0	Train
TNKS1_ML_1676	C[C@@H]1CC=C2C(C#N)=C(N)C(C#N)(C#N)[C@H](c3ccccc3OC(F)F)[C@@H]2C1	0	Train
TNKS1_ML_1677	Cn1cc(Br)cc1C(=O)NNS(=O)(=O)c1cccc(Cl)c1Cl	0	Train
TNKS1_ML_1678	C=C1C(=O)[C@]23[C@@H](OC(C)=O)C[C@H]4[C@@](C)(COC(C)=O)CCC[C@@]4(C)[C@H]2C[C@H](OC(C)=O)[C@H]1[C@@H]3OC(C)=O	0	Train
TNKS1_ML_1679	Cc1cc(C)c(S(N)(=O)=O)cc1C	0	Train
TNKS1_ML_1680	CCOc1ccc([C@H]2C(C(=O)c3sc(C)nc3C)=C(O)C(=O)N2CCc2ccccc2)cc1OCC	0	Train
TNKS1_ML_1681	C=C1C[C@@]2(C)CC[C@@H]1[C@](O)([S@](=O)(=NC)	0	Train

	c1ccccc1)C2		
TNKS1_ML_1682	O=C(c1cc(F)ccc1O)N1CCC[NH+]2CCC[C@@H]2C1	0	Train
TNKS1_ML_1683	Cc1ccc(NC(=O)[C@H](C)Sc2nnc3n2CCCCC3)cc1S(=O)(=O)N1CCOCC1	0	Train
TNKS1_ML_1684	CSCC[C@@H](NC(=O)c1ccccc1)C(=O)N1CCN(S(=O)(=O)c2cc(C)ccc2C)CC1	0	Train
TNKS1_ML_1685	CCCc1nn(C)c2c1NCC1(CCCC1)N2	0	Train
TNKS1_ML_1686	CCc1nn(-c2ccc(F)cc2F)c(O)c1Br	0	Train
TNKS1_ML_1687	O=C(NCCC1=c2ccccc2=[NH+]C1)C(=O)NC[C@@H](c1cccnc1)[NH+]1CCN(c2ccccc2)CC1	0	Train
TNKS1_ML_1688	CC(C)NC(=O)CN1CCN([C@@H](C)C(=O)Nc2sc3c(c2C#N)CCCCC3)CC1	0	Train
TNKS1_ML_1689	COc1ccc(-c2nn(Cn3ccc([N+](=O)[O-])n3)c(-c3ccc(OC)c(OC)c3)c2Br)cc1OC	0	Train
TNKS1_ML_1690	COc1ccccc1C1=CCN(c2nc3c(c(=O)[nH]2)CCCC3)CC1	1	Train
TNKS1_ML_1691	O=S(=O)(N[C@](c1ccccc1)(c1cc2ccccc2[nH]1)c1cc2ccccc2n1S(=O)(=O)c1ccccc1)c1ccccc1	0	Train
TNKS1_ML_1692	N#Cc1ccc(C(=O)NNC(=O)c2ccc3ccccc3c2)cc1	0	Train
TNKS1_ML_1693	CCOc1ccccc1N1C[C@@H](C(=O)Nc2ccccc2N2CCCC2)CC1=O	0	Train
TNKS1_ML_1694	CC1(C)[C@@](C)(C(=O)O)CC[C@]1(C)C(=O)N1CCCCCC1	0	Train
TNKS1_ML_1695	CC(C)N1CC2=NNC(=O)c3cccc4[nH]c(c2c34)C1	1	Train
TNKS1_ML_1696	Cc1cc(C)cc(Nc2ccc(Br)cc2C=O)c1	0	Train
TNKS1_ML_1697	Cc1nnc(SCC(=O)Nc2ccc(C(F)(F)F)cc2N2CCOCC2)o1	0	Train
TNKS1_ML_1698	Cn1ccnc1C(=O)N1CCC[C@@H]1c1nc(N)ncc1-c1ccc(C(F)(F)F)cc1	0	Train
TNKS1_ML_1699	C[C@@H]1Cc2ccccc2N1C(=O)NCc1cn2cc(Cl)ccc2[nH+]1	0	Train
TNKS1_ML_1700	CC[C@H]1CO[C@H](C)CN1C(=O)c1cc(N)c(Cl)c(Cl)c1	0	Train
TNKS1_ML_1701	COc1ccc([C@H]2c3ccc4ccccc4c3Oc3nc[n+](CCc4ccc(OC)c(OC)c4)c(N)c32)cc1	0	Train
TNKS1_ML_1702	CCOC(=O)c1ccc(NC(=O)c2cc(=O)n(Cc3cccnc3)/c(=N/Cc3ccc[nH+]c3)s2)cc1	0	Train
TNKS1_ML_1703	Cc1ccc(NC(=O)[C@@H](C)[NH+](C)Cc2cccc(C#N)c2)cc1S(=O)(=O)N1CCOCC1	0	Train
TNKS1_ML_1704	CC(C)c1cc(-c2n[nH]c(=S)o2)no1	0	Train
TNKS1_ML_1705	COc1cc(OC)c(OC)cc1/C=C1\Oc2c(CN3CC[NH+](C)CC3)c([O-])cc(C)c2C1=O	0	Train
TNKS1_ML_1706	COC(=O)c1c(NC(=O)/C=C/c2cc(OC)c(OC)c(OC)c2)sc2c1CCN(C(C)=O)C2	0	Train
TNKS1_ML_1707	Cc1oc2c(c1C(=O)N1CC[C@H](c3nn(CC[NH3+])c4nccnc34)C1)CCCC2	0	Train
TNKS1_ML_1708	C[C@H]1CCC[C@@H](c2noc(C=O)n2)C1	0	Train
TNKS1_ML_1709	C[C@H]1CN(C(=O)CC[NH+]2CCc3sccc3[C@@H]2c2ccccc2)CCN1C(=O)c1cccc([N+](=O)[O-])c1	0	Train
TNKS1_ML_1710	O=C(C1=C([O-])C(=O)N(CCCn2cc[nH+]c2)[C@H]1c1cccc(Cl)c1)c1ccncc1	0	Train
TNKS1_ML_1711	C[C@@H](C1CC1)N(Cc1nc2c(c(=O)[nH]1)COCC2)C(=O)CN1CCC(C(=O)c2ccc(F)cc2)CC1	1	Train
TNKS1_ML_1712	CCOc1ccc(NC(C)=O)cc1S(=O)(=O)Nc1nc2ccc(Cl)cc2s1	0	Train
TNKS1_ML_1713	Cn1c(C[NH+]2CCN(C(=O)c3ccccc3)CC2)nc2cc(NC(=O)c3ccc(Cl)cc3)ccc21	0	Train
TNKS1_ML_1714	O[C@H]1CCOC[C@@H]1Oc1ccc2c(c1)OCO2	0	Train
TNKS1_ML_1715	CC(C)N1CCN(C(=O)Nc2sccc2C(=O)[O-])CC1	0	Train
TNKS1_ML_1716	CC(=O)N1c2ccc(S(=O)(=O)Nc3cccc(-c4ccc(N5CCOCC5)nn4)c3)cc2C[C@H]1C	0	Train
TNKS1_ML_1717	C=C[C@H](CC(=O)NCc1[nH]c(-c2ccccc2)c[nH+]1)c1ccccc1	0	Train

TNKS1_ML_1718	C[C@@H]1CN(C(=O)[C@@H]2CCC[C@H]([NH3+])[C@@H]2C)CC(C)(C)O1	0	Train
TNKS1_ML_1719	O=C(Nc1nnc(-c2ccc3c(c2)CCCC3)o1)[C@@H]1CCCCN1S(=O)(=O)c1cccs1	0	Train
TNKS1_ML_1720	COC(=O)c1sc(N2C(=O)c3oc4ccc(C)cc4c(=O)c3[C@@H]2c2ccc(O)cc2)nc1C	0	Train
TNKS1_ML_1721	O=S(=O)([N-]c1cn[nH]c1)c1c(Cl)cc(Cl)cc1Cl	0	Train
TNKS1_ML_1722	O=C(CSc1ncc(-c2ccccc2)n1-c1ccccc1)Nc1ncc(S(=O)(=O)c2ccc([N+](=O)[O-])cc2)s1	0	Train
TNKS1_ML_1723	O=C(C[C@H]1S/C(=N/N=C\c2ccco2)NC1=O)Nc1cccc(C(F)(F)F)c1	0	Train
TNKS1_ML_1724	O=C(CCc1ccccc1)N(Cc1nc2c(c(=O)[nH]1)COCC2)Cc1cccs1	1	Train
TNKS1_ML_1725	C[C@H]1Cc2cc(N)ccc2N1C(=O)c1ccncc1Cl	0	Train
TNKS1_ML_1726	C[C@H]1[C@@H](C(=O)[O-])CCN1C(=O)[C@@H]1CC[C@@H]2CCCC[C@@H]2N1	0	Train
TNKS1_ML_1727	Cc1cc([O-])c(C[NH+]2C[C@@H]3C[C@@H](Cn4c3cccc4=O)C2)c2c1C(=O)/C(=C/c1ccc3c(c1)OCO3)O2	0	Train
TNKS1_ML_1728	C[C@H](CC(=O)NCc1c[nH]nc1N)C(C)(C)C	0	Train
TNKS1_ML_1729	CCOC(=O)c1ccc(C[C@@H]2[C@H](O)c3ccccc3S(=O)(=O)N2Cc2ccc[nH+]c2)cc1	0	Train
TNKS1_ML_1730	CCN1CCN(Cc2ccc(NC(=O)c3nn(-c4ccc(C)cc4)c(=O)n(C)c3=O)cc2)CC1	0	Train
TNKS1_ML_1731	C[C@H]1CCCCCN1c1nc(NN)ncc1Cl	0	Train
TNKS1_ML_1732	C[C@H]1CCC[C@@]2(CN[C@H](C)[C@H](c3ccco3)O2)C1	0	Train
TNKS1_ML_1733	Cc1cccc(-c2nnc([C@H]3C[C@H](NC(=O)c4cc(F)cc5nccnc45)C3)n2-c2ccccc2F)n1	1	Train
TNKS1_ML_1734	COc1ccc(-c2nn(CCC#N)cc2C(=O)Nc2ccccc2Cl)cc1	0	Train
TNKS1_ML_1735	Cc1c(N)nnn1Cc1csc(-c2ccccn2)n1	0	Train
TNKS1_ML_1736	C[C@H](C(=O)[O-])C1(O)CCC(C)(C)CC1	0	Train
TNKS1_ML_1737	O=C(Nc1ccc2c(c1)OCO2)[C@H]1COc2ccccc2C1	0	Train
TNKS1_ML_1738	Cc1nn(-c2ccc(C(=O)N[C@@H](C)c3nc4ccccc4[nH]3)cc2)c(C)c1Cl	0	Train
TNKS1_ML_1739	[H]/N=C1\OC2=C(C(=O)CC(C)(C)C2)[C@H](c2c[nH]c3ccccc23)[C@@H]1C#N	0	Train
TNKS1_ML_1740	CNc1nc([C@H]2CCCc3ccccc32)nc2sccc12	0	Train
TNKS1_ML_1741	COc1ccc(C(=O)C2CCN(C3CCN(Cc4nc(C)c(C)c(=O)[nH]4)C3=O)CC2)cc1	1	Train
TNKS1_ML_1742	O=C(CCn1cc(Cl)cn1)Nc1ccc(S(=O)(=O)Nc2nccs2)cc1	0	Train
TNKS1_ML_1743	Cc1cccn2c(=O)c(/C=C3\SC(=S)N(C(C)C)C3=O)c(NCCCn3ccnc3)nc12	0	Train
TNKS1_ML_1744	C[C@H](N(C)C(=O)N(C)c1ccccc1C(=O)[O-])C(C)(C)C	0	Train
TNKS1_ML_1745	COc1cccc(NC(=O)[C@H](C)NC(=O)N2CCN(C(=O)CCC3=c4ccccc4=[NH+]C3)CC2)c1	0	Train
TNKS1_ML_1746	O=C(NCc1ccccc1)Nc1nnc2n1CCS2	1	Train
TNKS1_ML_1747	Cc1nc(NC(=O)c2cc(Cl)ccc2[N+](=O)[O-])ccc1Br	0	Train
TNKS1_ML_1748	CC[C@H](CO)Nc1nc(C)c(C)s1	0	Train
TNKS1_ML_1749	Cc1nn([C@H](C)C2CCCC2)c(N)c1Cl	0	Train
TNKS1_ML_1750	COc1ccc(-c2nc(O)c3ccccc3n2)cc1	1	Train
TNKS1_ML_1751	Cc1ccc2c(c1)sc1nncn12	1	Train
TNKS1_ML_1752	OC1([C@@H]2[NH2+]CCc3ccccc32)CCC2(CC1)OCCO2	0	Train
TNKS1_ML_1753	COC(=O)C[C@H](c1ccc(OC)c(OC)c1)c1c(O)cc(O)c2c(=O)cc(-c3ccccc3)oc12	0	Train
TNKS1_ML_1754	COC(=O)c1sc(N2C(=O)C(=O)[C@@H](C(=O)c3cc(C)ccc3	0	Train

	C)[C@@H]2c2cccc([N+](=O)[O-])c2)nc1C		
TNKS1_ML_1755	Cc1cc(C)n(-c2nc(CCNC(=O)C(=O)c3c(C)[nH]c4ccccc34)cs2)n1	0	Train
TNKS1_ML_1756	O=C(N[C@H]1C[C@H](c2nnc(-c3ccccc3)n2-c2ccccc2F)C1)c1ccccn1	1	Train
TNKS1_ML_1757	C[C@H](c1ccccc1)N(Cc1nc2c(c(=O)[nH]1)COCC2)C(=O)CN1CCC(C(=O)c2ccc(F)cc2)CC1	1	Train
TNKS1_ML_1758	Cc1nn(C)c2ncc(N[C@H]3CCCSC3)cc12	0	Train
TNKS1_ML_1759	Cn1cc(NC(=O)[C@@H]2C[C@H]3CCCC[C@@H]3N2)ccc1=O	0	Train
TNKS1_ML_1760	CCN1C(=O)N[C@H](c2ccc(C)cc2)C2=C1CN(C(C)(C)C(=O)NCc1cccc[nH+]1)C2=O	0	Train
TNKS1_ML_1761	CCc1c(N)nnn1-c1cc(Cl)ccc1F	0	Train
TNKS1_ML_1762	Cc1cc(C)cc(N2C[C@@H](c3nnc(NC(=O)[C@@H]4CC(=O)N(C5CCCCC5)C4)s3)CC2=O)c1	0	Train
TNKS1_ML_1763	Cc1cc(NC(=O)c2ccc([N+](=O)[O-])c(F)c2)ncc1Br	0	Train
TNKS1_ML_1764	Cc1cc(Cl)ccc1S(=O)(=O)[N-]c1ccc2c(c1)CCN2	0	Train
TNKS1_ML_1765	CCOC(=O)c1c(C)[nH]c(C(=O)C(=O)NCc2cccc[nH+]2)c1-c1ccc(OC)cc1	0	Train
TNKS1_ML_1766	O=C([O-])c1c[nH]c(-c2cc(F)c(F)c(F)c2)n1	0	Train
TNKS1_ML_1767	Cc1nn(-c2ccccc2)c(Cl)c1/C=N/NC(=O)CNC(=O)c1ccccc1Cl	0	Train
TNKS1_ML_1768	C[C@@H](Sc1ccc(S(=O)(=O)N2CCN(C)CC2)cn1)C(=O)Nc1ncc(Cl)cc1Cl	0	Train
TNKS1_ML_1769	Cc1ccc(C)c([C@H](NC(=O)Cc2c(C)[nH]c3ncnn3c2=O)c2cc[nH+]cc2)c1	0	Train
TNKS1_ML_1770	O=C(CC1(CC(=O)N2CCn3c(nnc3C(F)(F)F)C2)CCCC1)NCCCn1ccnc1	0	Train
TNKS1_ML_1771	Cc1nn(C)c(C)c1S(=O)(=O)N1CCCN(C(=O)Nc2ccc(C(C)(C)C)cc2)CC1	0	Train
TNKS1_ML_1772	Cc1ccc([C@@H](C)Nc2nc(Cl)nc(Cl)n2)cc1F	0	Train
TNKS1_ML_1773	O[C@@H]1CCCc2sc(N3CCC[C@H](C(F)(F)F)C3)nc21	0	Train
TNKS1_ML_1774	O=c1[nH]c(-c2ccc(C(F)(F)F)cc2)nc2ccccc12	1	Train
TNKS1_ML_1775	Cc1ccc(-n2nnc(C(=O)Nc3cccc(Cl)c3)c2C)cc1	0	Train
TNKS1_ML_1776	O=C(NCCCn1ccnc1)[C@@H]1CCCN(S(=O)(=O)c2ccc(Cl)cc2)C1	0	Train
TNKS1_ML_1777	CN1CCN([C@@H](CNC(=O)[C@H]2CC(=O)N(c3ccccc3)C2)c2cccn2C)CC1	0	Train
TNKS1_ML_1778	C[C@H](NC(=O)CSc1ccc(S(=O)(=O)N2CCCC2)c[nH+]1)c1ccc2c(c1)OCCO2	0	Train
TNKS1_ML_1779	CCOc1ccc(-c2nnc([C@H]3C[C@H](NC(=O)c4ccnc5cc(F)cnc45)C3)n2-c2cccnc2)nc1	1	Train
TNKS1_ML_1780	C[C@H]1C[C@H]2C[NH2+]C[C@@H]2N1S(=O)(=O)c1cncc(Br)c1	0	Train
TNKS1_ML_1781	COC(=O)[C@H]1C(=O)C2=C(C[C@@H]1C)N=C(C)/C(=C(/[O-])OC1CCCCC1)[C@H]2c1ccc(N(C)C)cc1	0	Train
TNKS1_ML_1782	CSc1ccc(N2C[C@@H](c3nnc(/N=C(\[O-])c4cccc(F)c4)o3)CC2=O)cc1	0	Train
TNKS1_ML_1783	CNC(=O)NC(=O)[C@H](Sc1nnc([C@H](C)[NH+](C)C)n1-c1ccc(Cl)cc1)c1ccccc1	0	Train
TNKS1_ML_1784	O=C(CN1C(=O)CCc2cc(S(=O)(=O)N3CCCC3)ccc21)NCCc1nc2cc(F)ccc2[nH]1	0	Train
TNKS1_ML_1785	C[C@@H](CC(=O)[O-])NC(=O)N1C[C@H](C)CC[C@@H]1C	0	Train
TNKS1_ML_1786	CCC1CCC([NH3+])(c2noc([C@@H]3CCCOC3)n2)CC1	0	Train
TNKS1_ML_1787	CCOc1ccc([C@@H]2C(C(=O)c3ccc(Br)cc3)=C(O)C(=O)N2CC[NH+](C)C)cc1OCC	0	Train
TNKS1_ML_1788	Cc1ccc([C@H](O)c2ccc3c(c2)OCCCO3)cc1Cl	0	Train

TNKS1_ML_1789	O=C(c1ccc(Cl)cc1)C1CCN(CC(=O)N(Cc2nc3c(c(=O)[nH]2)COCC3)CC(F)F)CC1	1	Train
TNKS1_ML_1790	CC(C)(C)Oc1nc2ccc(N)cc2s1	0	Train
TNKS1_ML_1791	Clc1ccc([C@H]2Cc3n[nH]cc3[C@@H](Cl)C2)cc1	0	Train
TNKS1_ML_1792	COc1ccc2c(c1)C1=Nc3[nH+]cnn3[C@@H](c3ccc(F)cc3)[C@@H]1[C@H](c1ccc(OC)c(OC)c1)O2	0	Train
TNKS1_ML_1793	Cc1c(C(C)C)c(=O)n(-c2ccc(S(=O)(=O)Nc3ccc4c(c3)n(C)c(=O)n4C)cc2)n1C	0	Train
TNKS1_ML_1794	O=C(Nc1cccc(C(F)(F)F)c1)O[C@@H]1CO[C@H]2[C@@H]1OC[C@@H]2n1nnnc1SC1CCCC1	0	Train
TNKS1_ML_1795	C[C@@H]1C[S@@](=O)CC[NH+]1CC(=O)Nc1cccc(S(=O)(=O)/N=C2/CCCCCN2)c1	0	Train
TNKS1_ML_1796	Cc1n[nH]c(=S)n1-c1c(Cl)cc(Cl)cc1Cl	0	Train
TNKS1_ML_1797	CCN(CC)C(=O)c1ccc2c(c1)N(CC(=O)Nc1ccc3c(c1)OCO3)C(=O)[C@@H]1CCCCN21	0	Train
TNKS1_ML_1798	N#Cc1nc(-c2ccco2)oc1N1CCCC1	0	Train
TNKS1_ML_1799	O=C(CS[C@H]1NN=C(C2=c3ccccc3=[NH+]C2)N1C[C@@H]1CCCO1)N[C@@H]1CCS(=O)(=O)C1	0	Train
TNKS1_ML_1800	C[C@H](Sc1nc2sc3c(c2c(=O)n1CCC[NH+](C)C)CCCC3)C(=O)Nc1cccc(C#N)c1	0	Train
TNKS1_ML_1801	[NH3+]C1(C(F)(F)F)CCC2(CC1)OCCO2	0	Train
TNKS1_ML_1802	CCc1ccc(C(=O)Nc2ccc(Cl)c(C(N)=O)c2)cc1S(=O)(=O)N1CCOCC1	0	Train
TNKS1_ML_1803	C[C@@H]1CCCC[C@@]12NC(=O)N(CC(=O)N1N=C(c3ccc(Cl)cc3)C[C@H]1c1ccco1)C2=O	0	Train
TNKS1_ML_1804	Cc1cccn2c(=O)cc(CN3CCNC[C@@H]3C#N)nc12	0	Train
TNKS1_ML_1805	O=C([C@H](Cc1ccccc1)NS(=O)(=O)c1cccc2nsnc12)N1CCN(c2cccc[nH+]2)CC1	0	Train
TNKS1_ML_1806	COc1ccc(/N=C2\S[C@@H](C(=O)Nc3ccc(F)cc3)CC(=O)N2Cc2ccc(OC)c(OC)c2)cc1	0	Train
TNKS1_ML_1807	O=C1C[C@@H](c2[nH]cc[nH+]2)C2=C([O-])N[NH+](C3CCCCC3)[C@H]2N1	0	Train
TNKS1_ML_1808	Cc1ncc(/C(O)=C2\C(=O)C(=O)N(CCCn3ccnc3)[C@H]2c2cccc(F)c2)c(C)n1	0	Train
TNKS1_ML_1809	CCC[C@@H](c1nnnn1C1CCCC1)N(Cc1ccc(F)cc1)Cc1cc2cc3c(cc2[nH]c1=O)OCCO3	0	Train
TNKS1_ML_1810	CC(C)c1ccc([C@H]2C(=C(O)c3ccc(S(=O)(=O)N(C)C)cc3)C(=O)C(=O)N2Cc2ccc[nH+]c2)cc1	0	Train
TNKS1_ML_1811	COc1cc([C@@H]2SCC(O)=Nc3[nH]nc(C)c32)ccc1OC1CCCC1	0	Train
TNKS1_ML_1812	Nc1ccc(-c2nn3cnnc3s2)c(Br)c1	0	Train
TNKS1_ML_1813	CCc1ccc(-n2c(C)nnc2SCc2nc(-c3ccc(OC)cc3)no2)cc1	1	Train
TNKS1_ML_1814	C[C@@H](Nc1nc2ccc(N)cc2[nH]1)c1cccnc1	0	Train
TNKS1_ML_1815	N#Cc1ccc(Oc2cc(N)ccc2Cl)cc1Cl	0	Train
TNKS1_ML_1816	Clc1ccsc1-c1nc(C2CC2)n[nH]1	0	Train
TNKS1_ML_1817	CCn1ccc2cc(S(=O)(=O)N3CCC(C(=O)NCc4cccc[nH+]4)CC3)ccc21	0	Train
TNKS1_ML_1818	Cc1nn(-c2ccccc2)c(C)c1S(=O)(=O)Nc1nc2c(s1)C[C@H](C)CC2	0	Train
TNKS1_ML_1819	C[C@H](Sc1ccc(S(=O)(=O)N2CCN(C)CC2)cn1)C(=O)NC1(C#N)CCCCC1	0	Train
TNKS1_ML_1820	Cc1ccc(S(=O)(=O)NCC(=O)NCC(=O)Nc2nc3c(s2)CCCCC3)cc1	0	Train
TNKS1_ML_1821	Cc1nc(Cl)c2c(n1)CCCC2	0	Train
TNKS1_ML_1822	O=c1[nH]c(SCCN2CCN(CCSc3nc4ccccc4c(=O)[nH]3)CC2)nc2ccccc12	1	Train
TNKS1_ML_1823	Cc1nc(N)sc1S(=O)(=O)N1CC[C@H]([NH+](C)C)C1	0	Train
TNKS1_ML_1824	C[C@H](Sc1nnc(-	0	Train

	c2ccncc2)n1C)C(=O)Nc1ccc(S(=O)(=O)N2CCCC2)cc1		
TNKS1_ML_1825	N#Cc1cnn2c1N=C(c1cccc(Cl)c1)[C@H]2NC1CCCCC1	0	Train
TNKS1_ML_1826	Cc1ccccc1OC[C@H](O)Cn1c(N/N=C2/C(=O)Nc3ccc(Br)cc32)[nH+]c2c1c(=O)n(C)c(=O)n2C	0	Train
TNKS1_ML_1827	Cc1ccc(C)n1NC(=O)c1ccc(F)cc1Br	0	Train
TNKS1_ML_1828	Cc1c(C(=O)Nc2cc3c(cc2Br)OCCO3)cnn1-c1cccc(Cl)c1	0	Train
TNKS1_ML_1829	CC1(NC(=O)N2CC[C@@](C)(C(=O)[O-])C2)CCCCC1	0	Train
TNKS1_ML_1830	CNC(=O)c1ccc(CN(C)C(=O)/C=C\c2ccc(S(=O)(=O)NCc3ccco3)cc2)cc1	0	Train
TNKS1_ML_1831	CCNC(=O)[C@@H]1Cc2ccccc2N2CCN(c3cccc[nH+]3)C[C@H]12	0	Train
TNKS1_ML_1832	Cc1ccccc1NCc1nc2nc3c(c([O-])n2n1)CCC3	0	Train
TNKS1_ML_1833	Cc1nnc(N[C@@H](C)c2ccccc2)c(C#N)c1C	0	Train
TNKS1_ML_1834	COc1ccc(C=O)c2[nH]c3c(Cl)ncnc3c12	0	Train
TNKS1_ML_1835	CC[C@H]1CC[C@H](c2nc(N)c(Br)c(C3CC3)n2)C1	0	Train
TNKS1_ML_1836	C[C@H](C(=O)Nc1c(Cl)cc(Cl)cc1Cl)n1cnc2c1c(=O)n(C)c(=O)n2C	0	Train
TNKS1_ML_1837	O=S(=O)([N-]c1ccc(O)cc1)c1cccnc1Cl	0	Train
TNKS1_ML_1838	O=C(Nc1ccc(Cl)nc1)c1ccc(Cl)cc1Cl	0	Train
TNKS1_ML_1839	Cc1nc(C(C)(C)NC(=O)NCc2cnn(C)c2)sc1C	0	Train
TNKS1_ML_1840	Cc1nc(NS(=O)(=O)c2c(Cl)cc(Cl)cc2Cl)no1	0	Train
TNKS1_ML_1841	CN(c1ccc(-c2csc(NC(=O)Cc3ccc4c(c3)OCCO4)n2)cc1)S(C)(=O)=O	0	Train
TNKS1_ML_1842	COc1cccc2c(=O)[nH]c(-c3ccc(Cl)cc3)nc12	1	Train
TNKS1_ML_1843	CCCNc1nsnc1-c1ccc2c(c1)OCCO2	0	Train
TNKS1_ML_1844	O=C(Nc1ccc2c(c1)CCC2)c1ccc([N+](=O)[O-])cc1Cl	0	Train
TNKS1_ML_1845	CC1(C)C(=O)Nc2ccc(S(=O)(=O)N3CCCc4ccc(F)c(F)c43)cc21	0	Train
TNKS1_ML_1846	CC(=O)OC[C@H]1O[C@@H](O[C@H]2CC[C@]3(C)[C@@H](CC[C@@H]4[C@@H]3CC[C@@]3(C)C(=O)CC[C@H]43)C2)[C@H](OC(C)=O)[C@@H](OC(C)=O)[C@@H]1OC(C)=O	0	Train
TNKS1_ML_1847	Nc1nc(Cl)cc(SCc2ccccc2)n1	0	Train
TNKS1_ML_1848	Cc1c(/C=C2\SC(=S)N(CCc3ccccc3)C2=O)c(N2CCC(C(N)=O)CC2)n(C)c(=O)c1C#N	0	Train
TNKS1_ML_1849	CCN(CC)S(=O)(=O)c1ccc(S(=O)(=O)[N-]c2cccc(C#Cc3cccs3)c2)cc1	0	Train
TNKS1_ML_1850	Cc1nn(-c2ccccc2)c2nc(Cc3ccccc3)nc(N3CCCN(C(=O)C4CCCC4)CC3)c12	0	Train
TNKS1_ML_1851	CC(=O)O[C@H]1CC[C@@]2(C=O)[C@@H]3CC[C@]4(C)[C@H](C5=CC(=O)OC5)CC[C@]4(O)[C@@H]3CC[C@]2(O)C1	0	Train
TNKS1_ML_1852	COc1cc(Br)c(NC(=O)N[C@H]2CCC[C@H](C)C2)cc1OC	0	Train
TNKS1_ML_1853	CC1=C2C(=O)N=C(CS[C@H](C)C(=O)O[C@@H](C)C(N)=O)N=C2S[C@H]1C	0	Train
TNKS1_ML_1854	O=C(c1ccccn1)N1C[C@@H]2C[C@H](C1)c1ccc(NS(=O)(=O)c3cccs3)c(=O)n1C2	0	Train
TNKS1_ML_1855	COc1cc([C@@H]2CN3C(=O)CN(CCCOC(C)C)C(=O)[C@]3(C)[C@H]3[NH+]=c4ccccc4=C23)ccc1OC(C)C	0	Train
TNKS1_ML_1856	CC(C)(O)CC[C@@H](O)[C@](C)(O)[C@H]1CC[C@@]2(O)C3=CC(=O)[C@]4(O)C[C@H](O)[C@H](O)C[C@]4(C)[C@@H]3CC[C@]12C	0	Train
TNKS1_ML_1857	Cc1sc2ncnc(Nc3ccc4c(c3)OCCCO4)c2c1C	0	Train
TNKS1_ML_1858	CC1=NN(c2nc(-c3cc4ccccc4[nH]3)cs2)C(=O)[C@H]1C	0	Train
TNKS1_ML_1859	COc1ccc2c(c1)CC1(CCN([C@H]3CCN(Cc4nc5c(c(=O)[nH]4)COCC5)C3=O)CC1)C2=O	1	Train
TNKS1_ML_1860	O=C(NCc1cccs1)c1ccc[nH+]c1N1CCN(S(=O)(=O)c2ccc(C(	0	Train

	F)(F)F)cc2)CC1		
TNKS1_ML_1861	CCNC(=O)c1[nH]c(C)c(C(=O)[C@H]2C(=O)C(=O)N(c3cc(C)on3)[C@@H]2c2ccccc2F)c1C	0	Train
TNKS1_ML_1862	CC(C)NC(=O)Nc1ccccc1C(=O)OCc1cc(=O)n2cc(Br)ccc2n1	0	Train
TNKS1_ML_1863	Cc1ccc(C[n+]2c(N)c(C(=O)NC(C)(C)C)cc3c(=O)n4cccc(C)c4nc32)cc1	0	Train
TNKS1_ML_1864	CCN1CCN(c2ccc([N-]S(=O)(=O)c3c(C)[nH+]c4sccn4c3=O)cc2Cl)CC1	0	Train
TNKS1_ML_1865	CN[C@@H]1CCCc2nc(N3CC[C@@H]4CCCC[C@@H]4C3)sc21	0	Train
TNKS1_ML_1866	CN1CCCc2c1nc(N1CCC3(CC1)CN(CCO)c1cc(F)cc(F)c13)[nH]c2=O	1	Train
TNKS1_ML_1867	Cc1cccc(-c2ccc(N3CCC[C@H](O)C3)nn2)c1	0	Train
TNKS1_ML_1868	COc1ccc(S(=O)(=O)[C@@H](CNS(=O)(=O)c2ccc(OC)cc2OC)c2ccc[nH+]c2)cc1	0	Train
TNKS1_ML_1869	Cc1[nH+]c(SCC(=O)Nc2c(C#N)c3c(n2C[C@H]2CCCO2)CCCC3)n(C2CCCCC2)c1C	0	Train
TNKS1_ML_1870	Oc1nc(N2CCN(c3ccccc3)CC2)nc2c1CCCC2	1	Train
TNKS1_ML_1871	Cc1cnc(-c2nnc(C3CCC(CNC(=O)c4ccc(N(C)C)cc4)CC3)o2)cn1	0	Train
TNKS1_ML_1872	O=C(CN1CCC2(CC1)Cc1cc(F)ccc1C2=O)N(Cc1nc2c(c(=O)[nH]1)COCC2)CC1CC1	1	Train
TNKS1_ML_1873	CCN(CC)S(=O)(=O)c1ccc(C)c([N-]S(=O)(=O)c2ccc(Br)cc2Cl)c1	0	Train
TNKS1_ML_1874	CC(=O)Nc1ccc(S(=O)(=O)NC[C@@H](c2ccc[nH+]c2)S(=O)(=O)c2cccs2)cc1	0	Train
TNKS1_ML_1875	Cc1cc(C(=O)Nc2nc3c(s2)CNCC3)sc1C	0	Train
TNKS1_ML_1876	Cn1c(CC[NH+]2CCN(c3ccccn3)CC2)nc2cc(NC(=O)COc3ccc([N+](=O)[O-])cc3)ccc21	0	Train
TNKS1_ML_1877	N#C[C@H]1C(=O)N=C(N2CCN(C(c3ccccc3)c3ccccc3)CC2)NC12CCCC2	0	Train
TNKS1_ML_1878	O=S(=O)([C@H]1C=NC([C@@H]2NN=C(c3ccccc3)O2)=C1)N1CCN(c2cccc(Cl)c2)CC1	0	Train
TNKS1_ML_1879	Cc1c2cnn(-c3ccccc3)c(=O)c2c(C)n1[C@H](C)C(=O)NCCCN1CCN(C)CC1	0	Train
TNKS1_ML_1880	O=C(c1ccc(F)cc1)C1CCN(CC(=O)N(Cc2nc3c(c(=O)[nH]2)COCC3)Cc2ccccc2F)CC1	1	Train
TNKS1_ML_1881	Clc1nc(Cl)nc(OC2CCC2)n1	0	Train
TNKS1_ML_1882	Cc1cc(N2CCCC2=O)ccc1S(=O)(=O)Nc1cccc(CN2C(=O)[C@H]3CCCN3C2=O)c1	0	Train
TNKS1_ML_1883	O=c1[nH]c(N2CCC3(CC2)CNc2cccc(Br)c23)nc2c1CCCC2	1	Train
TNKS1_ML_1884	CC(=O)Nc1ccc(NC(=O)CN(c2cccc3ccccc23)S(C)(=O)=O)cc1	0	Train
TNKS1_ML_1885	Cc1c(/C([O-])=N/c2nnns2)oc2ccc(Cl)cc12	0	Train
TNKS1_ML_1886	CCOc1ccc(-n2c(SCc3nc(-c4ccc(OC)cc4)no3)nnc2-c2ccncc2)cc1	1	Train
TNKS1_ML_1887	Cc1cc2c(=O)n(NC(=O)c3cnn4c(C(F)(F)F)cc(-c5ccco5)nc34)c(C)nc2s1	0	Train
TNKS1_ML_1888	N#CC1=CC[C@H]2C(C#N)=CC[C@@H]12	0	Train
TNKS1_ML_1889	COc1ccc(-c2cnn(C3CCN(CC(=O)NCc4nc5c(c(=O)[nH]4)COCC5)CC3)c2)cc1	1	Train
TNKS1_ML_1890	C/C(=C/C(=O)Nc1ccc2c(c1)OCCO2)NNC(=O)COc1cc(C)c(Br)cc1Br	0	Train
TNKS1_ML_1891	CC[C@@H]1O[C@@]2(CCC[C@H](C)C2)CN[C@@H]1C	0	Train
TNKS1_ML_1892	CCOc1ccc(-c2nnc([C@H]3C[C@H](NC(=O)c4ccccn4)C3)n2-	1	Train

	c2ccc(Cl)s2)nc1		
TNKS1_ML_1893	COC(=O)[C@]12C(=O)[C@](C)(O)C(=O)[C@]1(C)C(C)=C[C@H]1[C@@]3(C=O)CC[C@H](OC(C)=O)C(C)(C)[C@@H]3CC[C@]12C	0	Train
TNKS1_ML_1894	O=C(Nc1ccc(F)cc1Br)c1ccc(Cl)cc1[O-]	0	Train
TNKS1_ML_1895	Cc1nc(Cl)cc(Nc2ccc3c(c2)OCO3)n1	0	Train
TNKS1_ML_1896	O=C(CN1CCC(C(=O)c2ccc(Cl)cc2)CC1)NCc1nc2c(c(=O)[nH]1)COCC2	1	Train
TNKS1_ML_1897	COc1ccc(-c2nc(O)c3cccc(OC)c3n2)cc1	1	Train
TNKS1_ML_1898	O=C(c1ccc(F)cc1)C1CCN(CC(=O)N(Cc2nc3c(c(=O)[nH]2)COCC3)Cc2cccs2)CC1	1	Train
TNKS1_ML_1899	O=C(CN1CCC(C(=O)c2ccc(F)cc2)CC1)NCc1nc2c(c(=O)[nH]1)COCC2	1	Train
TNKS1_ML_1900	Cc1sc2[nH]c(=S)n(C3(C)CCCCC3)c(=O)c2c1C	0	Train
TNKS1_ML_1901	[H]/N=C1C(=C/c2cn(CCOc3ccccc3)c3ccccc23)\C(=O)N=C2C=C(C)ON2\1	0	Train
TNKS1_ML_1902	Cc1ccc(C(=O)C2CCN(CC(=O)NCc3nc4c(c(=O)[nH]3)COCC4)CC2)cc1	1	Train
TNKS1_ML_1903	CC(C)c1ccc2c(c1)sc1nncn12	1	Train
TNKS1_ML_1904	CC(=O)N1CCc2cc(C(=O)[C@H](C)Sc3nnc(N)s3)ccc21	0	Train
TNKS1_ML_1905	CCOc1ccc([C@@H]2C(C(=O)c3sc(C)nc3C)=C(O)C(=O)N2CC[NH+](CC)CC)cc1OCC	0	Train
TNKS1_ML_1906	Nc1ccc2ncnc(Oc3ccccc3Cl)c2c1	0	Train
TNKS1_ML_1907	Cc1cc([O-])nc2nnc(SCCC(C)C)n12	0	Train
TNKS1_ML_1908	CN(C)[C@H](CNC(=O)C1=c2cc(S(=O)(=O)N3CCOCC3)ccc2=[NH+]C(=O)C1)c1cccs1	0	Train
TNKS1_ML_1909	CSCC[C@@H](NS(=O)(=O)c1ccc2c(c1)OCCO2)C(=O)OCC(=O)c1ccc(Cl)s1	0	Train
TNKS1_ML_1910	CC(=O)c1cccc(Nc2c(C(=O)N3CCCC[C@H]3C)cnc3nc(C)ccc23)c1	0	Train
TNKS1_ML_1911	Cc1nc(COc2ccccc2/C=C/C(=O)NCc2ccc(N3CCOCC3)[nH+]c2)cs1	0	Train
TNKS1_ML_1912	Cc1cc(CN2C(=O)[C@@H](C(C)C)NC(=O)[C@H]2C)no1	0	Train
TNKS1_ML_1913	CCn1cc(C(=O)NC2CCCCCC2)c(=O)c2cc(S(=O)(=O)N3CCOCC3)ccc21	0	Train
TNKS1_ML_1914	FC(F)(F)c1ccc(-c2noc([C@@H]3Cc4[nH]c[nH+]c4C[NH+]3Cc3ccccn3)n2)cc1	0	Train
TNKS1_ML_1915	COc1ccccc1OCCCCn1c([C@@H](C)NC(=O)c2cc(OC)c(OC)c(OC)c2)[nH+]c2ccccc21	0	Train
TNKS1_ML_1916	CNc1cncc(N2C[C@@H](C)O[C@H](C)C2)n1	0	Train
TNKS1_ML_1917	CC(C)c1ccc(C2NC(=O)c3ccccc3N2)cc1	1	Train
TNKS1_ML_1918	C=CC(C)(C)[C@@]12CC(=O)C(=O)N3/C(=C/c4c[nH+]c[nH]4)C(=O)N[C@]31N(OC)c1ccccc12	0	Train
TNKS1_ML_1919	Clc1nc2c(Cl)c3ccccc3nc2s1	0	Train
TNKS1_ML_1920	Cc1ccsc1[C@@H]1CNC[C@@]2(CCC[C@H](C)C2)O1	0	Train
TNKS1_ML_1921	CC(C)[C@@H]1CCC[C@]2(CC1)C[C@H](Cl)CCO2	0	Train
TNKS1_ML_1922	COc1ccc(-c2cc3c(c(=O)[nH]2)CCCN3C)cc1	1	Train
TNKS1_ML_1923	COc1ccccc1NC(=O)c1ccc(NC(=O)CCc2nc3ccccc3c(=O)[nH]2)cc1	1	Train
TNKS1_ML_1924	Cc1nn(C)c(O[C@H]2C[C@@H](C)CC(C)(C)C2)c1N	0	Train
TNKS1_ML_1925	NC(=O)c1cccc2c1O/C(=C\c1ccc(C(=O)N3CCN(c4nc5ccccc5[nH]4)CC3)cc1)C2=O	1	Train
TNKS1_ML_1926	Nc1ccc2nc(Oc3cc(F)ccc3F)ccc2c1	0	Train
TNKS1_ML_1927	O=C(Nc1ccc(N2CCCS2(=O)=O)cc1)[C@@H]1Cc2cc(Cl)ccc2O1	0	Train
TNKS1_ML_1928	Cc1noc(C=CN(C)C)c1S(=O)(=O)NCC[NH+]1CCN(c2ccccc2F)CC1	0	Train

TNKS1_ML_1929	COc1cccc(C(=O)C2CCN(CC(=O)N(Cc3nc4c(c(=O)[nH]3)COCC4)CC(F)F)CC2)c1	1	Train
TNKS1_ML_1930	CN1CCCc2cc([C@H](CNC(=O)Cn3c(=O)oc4ccccc43)N3CC[NH+](C)CC3)ccc21	0	Train
TNKS1_ML_1931	Cc1cccc(NS(=O)(=O)c2c(N)ccc(C)c2C)n1	0	Train
TNKS1_ML_1932	Cc1nc(N)nc(N2CC([NH+]3CCCCC3)C2)c1C	0	Train
TNKS1_ML_1933	COc1ccccc1[C@H](C)NS(=O)(=O)N1C[C@H](C)O[C@H](C)C1	0	Train
TNKS1_ML_1934	CN1CCCc2c1nc(N1CCC3(CC1)C(=O)N(C)c1cc(N4CCOCC4)cc(F)c13)[nH]c2=O	1	Train
TNKS1_ML_1935	Cc1ccc(O)c(C(=O)Nc2cnc3ccccc3c2)c1	0	Train
TNKS1_ML_1936	O=C(NCCN1CCOCC1)c1coc(C[NH+]2CCN(c3cccc(C(F)(F)F)c3)CC2)n1	0	Train
TNKS1_ML_1937	O=C(Nc1nnc2n1CCCS2)c1ccccc1	1	Train
TNKS1_ML_1938	[H]/N=C1C(=C/c2ccc(OCCCOc3ccccc3Cl)cc2)/C(=O)N=C2SC(c3cccnc3)=NN2/1	0	Train
TNKS1_ML_1939	O=c1cc(-c2ccc(O)c(O)c2)oc2cc(O)ccc12	1	Train
TNKS1_ML_1940	O=C(c1cc(-c2cccc(Br)c2)[nH]n1)N1N=C(C(F)F)C[C@@]1(O)C(F)F	0	Train
TNKS1_ML_1941	O=C(C1=C([O-])C(=O)N(CCCn2cc[nH+]c2)[C@@H]1c1cccc(OCc2ccccc2)c1)c1ccco1	0	Train
TNKS1_ML_1942	COC(=O)c1c(NC(=O)c2ccc(S(=O)(=O)N3C[C@H](C)O[C@@H](C)C3)cc2)sc2c1CC[NH+](Cc1ccccc1)C2	0	Train
TNKS1_ML_1943	COc1ccc2c(c1)CC1(CCN(CC(=O)N(Cc3nc4c(c(=O)[nH]3)COCC4)CC(F)F)CC1)C2=O	1	Train
TNKS1_ML_1944	CCc1ccc(CNc2c[nH+]c3c(c2)c(NC(=O)c2ccco2)c(C(=O)OC)n3CCOC)o1	0	Train
TNKS1_ML_1945	CC[C@H]1CNC[C@@]2(CCCC2(C)C)O1	0	Train
TNKS1_ML_1946	CC(C)n1nccc1NC(=O)[C@H](C)Sc1ccc(S(=O)(=O)N2CCCC2)cn1	0	Train
TNKS1_ML_1947	O=c1cc(-c2ccc(O)c(O)c2)oc2cc(O)cc(O)c12	1	Train
TNKS1_ML_1948	O=C(c1ccc(F)cc1)C1CCN(C2CCN(Cc3nc4c(c(=O)[nH]3)COCC4)C2=O)CC1	1	Train
TNKS1_ML_1949	CN(c1ccccc1)c1ccc(N)cc1C#N	0	Train
TNKS1_ML_1950	O[C@H]1CCCN(c2nc(Cl)nc(Cl)n2)C1	0	Train
TNKS1_ML_1951	Cc1ccc(C(=O)[O-])cc1N[C@H](C)c1ccc(Br)s1	0	Train
TNKS1_ML_1952	N#Cc1ccc(-c2ccccn2)cc1	0	Train
TNKS1_ML_1953	CNc1nc(-c2ccc(C#N)cc2)ccc1C#N	0	Train
TNKS1_ML_1954	[H]/N=C1\OC2=C(C(=O)C[C@@H](C)C2)[C@H](c2cccs2)[C@@H]1C#N	0	Train
TNKS1_ML_1955	C=CCn1c(SCC(=O)N2CC[NH+](C)CC2)nnc1[C@@H](C)NC(=O)c1ccccc1	0	Train
TNKS1_ML_1956	OCc1c[nH]c(-c2ccccc2)[nH+]1	0	Train
TNKS1_ML_1957	Cc1[nH+]ccn1-c1ccc(NC(=O)c2sccc2S(=O)(=O)N2CCCCC2)cn1	0	Train
TNKS1_ML_1958	Nc1ncccc1[C@@H](O)c1cc(Cl)c2c(c1)OCO2	0	Train
TNKS1_ML_1959	CCc1nn(C)c(C[C@H]([NH3+])C(CC)(CC)N(C)C)c1Br	0	Train
TNKS1_ML_1960	O=C(CCc1ccccc1)N(Cc1nc2c(c(=O)[nH]1)COCC2)Cc1nccs1	1	Train
TNKS1_ML_1961	CC(=O)O[C@@H]1C[C@H](O)[C@@]2(C)[C@@H](O)[C@H]3[C@@H](C[C@@H](C)[C@@H]12)OC(=O)[C@H]3CN1CCCCC1	0	Train
TNKS1_ML_1962	Oc1c(Br)cccc1/C=N\c1nnc[nH]1	0	Train
TNKS1_ML_1963	COc1ccccc1NC(=O)C(=O)NC[C@H](c1ccc(N(C)C)cc1)N1CC[NH+](C)CC1	0	Train
TNKS1_ML_1964	CC[NH+]1CCC[C@H]1CN1C[C@H](C(=O)NNc2cc(C#N)cc(Cl)n2)CC1=O	0	Train

TNKS1_ML_1965	COc1ccc(CCN(C(=S)Nc2ccccc2)[C@H]2CC(=O)N(c3cccc(Cl)c3)C2=O)cc1OC	0	Train
TNKS1_ML_1966	COc1ccc(-c2noc(CSc3nnc(-c4ccncc4)n3-c3ccc(C)c(C)c3)n2)cc1	1	Train
TNKS1_ML_1967	CNS(=O)(=O)c1cc(C(=O)N2CCC[C@@H]2c2ccc3c(c2)OCCCO3)cc(C)c1C	0	Train
TNKS1_ML_1968	Cc1ccc([C@@H](C(=O)NCc2ccccc2)N(C(=O)c2csnn2)c2ccccc2Br)o1	0	Train
TNKS1_ML_1969	NCc1ccn([C@@H]2CCOC3(CCCCC3)C2)n1	0	Train
TNKS1_ML_1970	Cc1ccc2c(c1)NC(C)(C)c1ss/c(=N\c3cccc(F)c3)c1-2	0	Train
TNKS1_ML_1971	O=C(CCn1cncn1)Nc1nnc2n1CCS2	1	Train
TNKS1_ML_1972	O=C1Oc2ccccc2C12CCN(c1nc3c(c(=O)[nH]1)CCCC3)CC2	1	Train
TNKS1_ML_1973	Cc1ccc(Nc2nc(N/N=C/c3cc(Br)ccc3O)nc(N3CCOCC3)n2)c(Br)c1	0	Train
TNKS1_ML_1974	C[NH+](C)c1ccc([C@@H]2CC(c3ccccc3Cl)=NN2C(=O)CSc2nnc(N)s2)cc1	0	Train
TNKS1_ML_1975	COc1ccc(-c2cc(-c3ccccc3)nc(N/N=C/c3cccc(OC)c3[O-])n2)cc1OC	0	Train
TNKS1_ML_1976	Cc1nn(C)c(N2C[C@H](C)S[C@@H](C)C2)c1N	0	Train
TNKS1_ML_1977	CCc1cccc2sc(N(CC[NH+](C)C)C(=O)[C@@H]3CCCN(S(=O)(=O)c4cccs4)C3)nc12	0	Train
TNKS1_ML_1978	CN1CCN(c2ccc(-c3nc4ccc(F)cc4c(=O)[nH]3)cc2)CC1	1	Train
TNKS1_ML_1979	O=C([O-])[C@H]1CCC[C@@H](NC(=O)N2CC[NH+]3CCC[C@@H]3C2)C1	0	Train
TNKS1_ML_1980	N#Cc1ccc2c(c1)[nH]c(=O)n2[C@H]1C[C@H](c2nnc(-c3ccncn3)n2-c2ccccc2Cl)C1	1	Train
TNKS1_ML_1981	C=CCn1c(N2CC[NH+](Cc3ccc(Br)cc3)CC2)nc2c1c(=O)[nH]c(=O)n2C	0	Train
TNKS1_ML_1982	CCOC(=O)c1nn(-c2ccc(OC(F)(F)F)cc2)c(=O)c2c(NC(C)=O)scc12	0	Train
TNKS1_ML_1983	Cc1nc([C@@H]2CCC[C@@H](C)C2)nc(Cl)c1C	0	Train
TNKS1_ML_1984	Cc1noc2c1N=C(c1ccc(O)cc1)C[C@@H](c1ccccc1)C2	0	Train
TNKS1_ML_1985	C[C@@H]1CCC[C@@H](NC(=O)c2cc(S(=O)(=O)N3CCCCC3)c[nH]2)[C@@H]1C	0	Train
TNKS1_ML_1986	COc1ccc(OC)c2c1sc1nnc(N)n12	1	Train
TNKS1_ML_1987	CC(=O)Nc1ccc(F)c(NCc2cc(Cl)cc3c2OCOC3)c1	0	Train
TNKS1_ML_1988	COc1ccccc1N1CC[NH+](CC[C@@H]2CC[C@H](O)[C@@H](C(=O)N3CCN(c4ccc(F)cc4)CC3)O2)CC1	0	Train
TNKS1_ML_1989	Brc1cc(CNc2cccc3cn[nH]c23)cc2c1OCO2	0	Train
TNKS1_ML_1990	COc1nc(N2CCc3sccc3[C@@H]2C)ccc1N	0	Train
TNKS1_ML_1991	Cc1cc(OCC(=O)N2CCc3c(sc(N)c3C#N)C2)ccc1C(C)C	0	Train
TNKS1_ML_1992	CC1(C)OC(=O)N([C@H]2CC[C@H](NC(=O)c3cccc4cccnc34)CC2)[C@H]1c1ccccc1	1	Train
TNKS1_ML_1993	Cc1cccc(-c2nc3ncc(Br)cc3[nH]2)c1C	0	Train
TNKS1_ML_1994	C[C@@H]1CCC[C@H](N2C(=O)C3(CCCC3)NC(=O)[C@H]2C)[C@@H]1C	0	Train
TNKS1_ML_1995	CC(C)c1nc2c(n1-c1cnn(C)c1)CCNC2	0	Train
TNKS1_ML_1996	C[C@@H](c1ccccn1)N(C)C(=O)c1cc(NCc2ccsc2)cc(S(=O)(=O)N(C)C)c1	0	Train
TNKS1_ML_1997	N#C/C(=C/c1ccc([O-])c(Cl)c1)C(=O)Nc1nccs1	0	Train
TNKS1_ML_1998	Cc1cc(CNc2ncnc3sc(C)c(C)c23)on1	0	Train
TNKS1_ML_1999	COc1ccc2nc([C@H]3CSc4ccccc43)[nH]c2n1	0	Train
TNKS1_ML_2000	Cc1ccc([C@H](C)NS(=O)(=O)c2c[nH+]ccc2N)cc1F	0	Train
TNKS1_ML_2001	CCOc1cc([C@@H]2C(=C(O)c3ccc4c(c3)OCCO4)C(=O)C(=O)N2CCCn2ccnc2)ccc1O	0	Train
TNKS1_ML_2002	Cc1ccc(C2=NO[C@@H]([C@@H]3C=C(c4ccc(Cl)cc4)N=N3)N2)cc1	0	Train

TNKS1_ML_2003	CC1=C[C@@H](NC(=O)CN2C(=O)N(CCC(=O)NCCc3ccc(Cl)cc3)C(=O)[C@H]3SC=C[C@@H]32)N=N1	0	Train
TNKS1_ML_2004	CCS(=O)(=O)N1CCC(C(=O)N[C@@H](C)c2ccc(C#N)cc2)(c2ccccc2)CC1	0	Train
TNKS1_ML_2005	C=C1CCC[C@@]2(C)C[C@@H]3OC(=O)C(C)=C3C[C@@H]12	0	Train
TNKS1_ML_2006	C[C@@]12CC[C@@H](O)[C@@](C)(CO)[C@H]1Cc1sc(NC(=O)C3CCCCC3)nc1[C@H]2CC(=O)NCCN1CCOCC1	0	Train
TNKS1_ML_2007	COc1nccnc1[N-]S(=O)(=O)c1ccc(Nc2cc(C)[nH+]c3cc(C(F)(F)F)ccc23)cc1	0	Train
TNKS1_ML_2008	CC(C)Cc1nnc(NC(=O)C2CCN(S(=O)(=O)c3cc(C(N)=O)n(C)c3)CC2)s1	0	Train
TNKS1_ML_2009	Cc1nnc(SCc2nc(-c3ccc(Cl)cc3)no2)n1-c1ccc(F)cc1	1	Train
TNKS1_ML_2010	COc1cccc([C@H]2C(C(=O)c3sc(C)nc3C)=C(O)C(=O)N2c2nc(C)c(C(C)=O)s2)c1OC	0	Train
TNKS1_ML_2011	O=C(CCc1ccccc1)N(Cc1nc2ccccc2c(=O)[nH]1)Cc1cccs1	1	Train
TNKS1_ML_2012	Cc1ccc2cc(C(=O)Nc3ccc4c(c3)n(C)c(=O)n4C)c(C)nc2c1	0	Train
TNKS1_ML_2013	CC(C)(C)[C@H]1CCCN1C(=O)O	0	Train
TNKS1_ML_2014	Cc1ccc(C)c(C(=O)NC[C@H](c2ccc(C)o2)N2CCOCC2)c1	0	Train
TNKS1_ML_2015	Cc1nc(N(C)C)cc([C@@H](Cc2c[nH]c3ccccc23)NC(=O)c2cccc(F)c2)[nH+]1	0	Train
TNKS1_ML_2016	C[C@@]1(C(=O)[O-])CC[C@H]2C(Br)=C[C@@]21C	0	Train
TNKS1_ML_2017	CC(C)(O)c1ccc(-c2nc3c(F)ccc(F)c3c(=O)[nH]2)cc1	1	Train
TNKS1_ML_2018	C[C@H]1CCCCN1S(=O)(=O)c1ccc(C(=O)N(Cc2cccc[nH+]2)c2nc3ccc(F)cc3s2)cc1	0	Train
TNKS1_ML_2019	CC(=O)Oc1cc(C)c2c(c1C)[C@@H]1OC(=O)[C@@H](C)[C@H]1CC2	0	Train
TNKS1_ML_2020	Cc1cccc2c1c(=O)n(C[C@H]1CC[C@H](C(=O)Nc3ccncc3)CC1)c(=O)n2Cc1cccc(C(F)(F)F)c1C#N	1	Train
TNKS1_ML_2021	Cc1c(Br)cccc1NC(=O)N1CCCC[C@H]1c1[nH]cc[nH+]1	0	Train
TNKS1_ML_2022	COc1cccc(/C=N/c2cc(S(=O)(=O)N(C)C(C)C)ccc2C)c1O	0	Train
TNKS1_ML_2023	CC(=O)C1=CC[C@@H]2[C@H]3C[C@@H]4O[C@@]45C[C@@H](O)CC[C@]5(C)[C@@H]3CC[C@]12C	0	Train
TNKS1_ML_2024	Cc1ccccc1C(=O)N1[C@H](C(=O)NCC(C)C)COC12CCN(C(=O)c1ccco1)CC2	0	Train
TNKS1_ML_2025	Cc1cc(C)c2nc(N(CCC[NH+](C)C)C(=O)C3CCN(S(=O)(=O)c4cccs4)CC3)sc2c1	0	Train
TNKS1_ML_2026	C[C@@H]1CCC[C@H](c2noc(-c3ccc(N)s3)n2)C1	0	Train
TNKS1_ML_2027	CC(C)[C@@H]1CCOC(=O)CC1	0	Train
TNKS1_ML_2028	O=C1N(CCO)c2ccccc2C12CCN(c1nc3c(c(=O)[nH]1)CCCC3)CC2	1	Train
TNKS1_ML_2029	N#Cc1cc(NC(=O)N2CCC[C@@H]2c2ccc3c(c2)OCO3)ccc1F	0	Train
TNKS1_ML_2030	COC(=O)c1[nH]c(C)c(/C(O)=C2/C(=O)C(=O)N(CCCn3cc[nH+]c3)[C@H]2c2cccs2)c1C	0	Train
TNKS1_ML_2031	O=C1COc2ccc(C(=O)CSc3[nH]c(-c4ccc(Br)cc4)c[nH+]3)cc2N1	0	Train
TNKS1_ML_2032	N#Cc1c(N)nc2c(c1-c1cc(CN3CCCCC3)cs1)COCC2	0	Train
TNKS1_ML_2033	Cc1ccc(-c2csc3ncnc(OCC(=O)NC4CC4)c23)cc1	0	Train
TNKS1_ML_2034	CC[C@H](C)N(Cc1nc2c(c(=O)[nH]1)COCC2)C(=O)CN1CCC(C(=O)c2ccc(F)cc2)CC1	1	Train
TNKS1_ML_2035	C[NH+](C)CCN(C1CCN(Cc2ccccc2S(C)(=O)=O)CC1)S(=O)(=O)c1cccc2cccnc12	0	Train
TNKS1_ML_2036	Cc1nn(-c2ccccc2)c2sc(C(=O)Nc3ccncc3)cc12	0	Train
TNKS1_ML_2037	CC(C)C1Cc2[nH]c(=O)c3cccnc3c2CO1	1	Train
TNKS1_ML_2038	Clc1nsnc1O[C@@H]1CCOC1	0	Train
TNKS1_ML_2039	Nc1nn([C@@H]2CCO[C@@]3(CCSC3)C2)c[nH+]1	0	Train
TNKS1_ML_2040	COC(=O)[C@]1(CO)[NH2+][C@H](c2ccccc2F)[C@H]2C(	0	Train

	=O)N(c3ccccc3)C(=O)[C@@H]21		
TNKS1_ML_2041	O=C([O-])[C@H]1c2ccsc2CCN1C(=O)NC[C@H]1CCCO1	0	Train
TNKS1_ML_2042	CN1C(=O)N/C(=C\c2ccc(-c3ccc(Cl)cc3Cl)o2)C1=O	0	Train
TNKS1_ML_2043	CC(C)(C)c1cc(/C=N/NC(=O)CN2CCN(S(=O)(=O)c3ccc(Br)cc3)CC2)cc(C(C)(C)C)c1O	0	Train
TNKS1_ML_2044	CC(=O)Nc1ccc2c(c1)[nH+]c1n2CCN([C@@H](C)c2ccccc2)C1	0	Train
TNKS1_ML_2045	CCN(Cc1ccc(C#N)cc1F)C(=O)Nc1cccc([N+](=O)[O-])c1	0	Train
TNKS1_ML_2046	COc1ccc(C(=O)C2CCN(C(=O)CCCc3cn4cc(F)cc4c(=O)[nH]3)CC2)cc1	1	Train
TNKS1_ML_2047	CCC(=O)N1CCc2cc(S(=O)(=O)CCC(=O)Nc3c(C)n(C)n(-c4ccccc4)c3=O)ccc21	0	Train
TNKS1_ML_2048	C#CCN1CCC(C)(C)CCC1=O	0	Train
TNKS1_ML_2049	COc1cc(/C=C2/N=C(c3cc(I)ccc3Cl)N=C2[O-])cc(OC)c1OC	0	Train
TNKS1_ML_2050	CCN(C(=O)N1CCC(C(=O)c2ccc3cc(C(=O)O)n(C)c3c2)CC1)c1ccc(OC)cc1	1	Train
TNKS1_ML_2051	CCOc1ccc(S(=O)(=O)N2CCC3(CC2)OCCN3S(=O)(=O)c2ccc(F)c(C)c2)cc1F	0	Train
TNKS1_ML_2052	Cc1cc(Nc2ccc(Br)cc2F)n2nnnc2n1	0	Train
TNKS1_ML_2053	Cc1ccc(N[C@@H]2COC3(CCN(C(=O)c4ccc5ccccc5n4)CC3)C2)nn1	0	Train
TNKS1_ML_2054	Cc1cc(C)c(C#N)c(N[C@@H]2CCC[C@H](C)C2)n1	0	Train
TNKS1_ML_2055	Cc1nc(NC[C@@H]2CCCO2)c2c(C)nn(-c3ccccc3Cl)c2n1	0	Train
TNKS1_ML_2056	Cc1nnc(N2CCC(c3ccc(O)cc3)CC2)c(C#N)c1C	0	Train
TNKS1_ML_2057	O=C(CCc1cccnc1)Nc1nnc2n1CCS2	1	Train
TNKS1_ML_2058	COc1ccccc1[C@@H](NC(=O)NCc1coc(-c2cccs2)n1)c1[nH+]ccn1C	0	Train
TNKS1_ML_2059	O=C(c1ccc(F)cc1)C1CCN(CC(=O)N(Cc2nc3c(c(=O)[nH]2)COCC3)CC(F)(F)F)CC1	1	Train
TNKS1_ML_2060	N#CC(C#N)=C(N)/C(C#N)=C\c1ccc(-c2cccc(Cl)c2Cl)o1	0	Train
TNKS1_ML_2061	N#Cc1ccc(Oc2ccc(NC(=O)CCSc3nc4ccccc4c(=O)[nH]3)cc2)cc1	1	Train
TNKS1_ML_2062	O=C1Nc2ccccc2C12CCN(c1nc3c(c(=O)[nH]1)CCCC3)CC2	1	Train
TNKS1_ML_2063	Cn1ncnc1[C@@H]1c2n[nH]c(=O)c3cc(F)cc(c23)N[C@H]1c1ccc(F)cc1	1	Train
TNKS1_ML_2064	O=C(NCc1ccccc1)[C@@H](c1ccccc1)N(Cc1ccc[nH+]c1)C(=O)c1csnn1	0	Train
TNKS1_ML_2065	COC(=O)[C@H](CC1=c2ccccc2=[NH+]C1)NC(=O)[C@H]1CC(=O)N(c2ccccc2F)C1	0	Train
TNKS1_ML_2066	O=C(CSc1ccccc1F)NNC(=O)Cn1c(-c2cscn2)nc2ccccc21	0	Train
TNKS1_ML_2067	C[C@H](c1ccccc1)N(Cc1nc2c(c(=O)[nH]1)COCC2)S(=O)(=O)Cc1ccccc1	1	Train
TNKS1_ML_2068	CC(C)Sc1nc2sc3c(c2c2nnc(SCC(=O)Nc4nccs4)n12)CC(C)(C)OC3	0	Train
TNKS1_ML_2069	CCCn1c(N2CCCCC2)c(/C=C2/SC(=S)N(CCCCCC(=O)O)C2=O)c(C)c(C#N)c1=O	0	Train
TNKS1_ML_2070	COc1ccc(NC(=O)C2CCN(S(=O)(=O)c3cc(-c4noc(C)n4)cs3)CC2)cc1Cl	0	Train
TNKS1_ML_2071	CCOc1ccc(C(=O)OCC(=O)N[C@@H](c2ccc(F)cc2)c2cccs2)cc1[N+](=O)[O-]	0	Train
TNKS1_ML_2072	O=C1Nc2cc(Cl)cc(Cl)c2C12CCN(c1nc3c(c(=O)[nH]1)CCCC3)CC2	1	Train
TNKS1_ML_2073	CC(C)c1ccccc1-n1cc(O)c(C(=O)[O-])n1	0	Train
TNKS1_ML_2074	C[C@H]1CN(c2ccc(CNC(=O)c3ccc(Cn4cncn4)cc3)c[nH+]2)C[C@@H](C)O1	0	Train
TNKS1_ML_2075	C=CCNS(=O)(=O)c1ccc(C(=O)Nc2c(C)n(C)n(-c3ccccc3)c2=O)cc1	0	Train
TNKS1_ML_2076	C=C(C)[C@H]1Cc2c(C)nn(-c3ccccc3)c2C1	0	Train

TNKS1_ML_2077	CC1(C)CS(=O)(=O)N(c2ccc(S(=O)(=O)NC3CCCCC3)cc2)C1=O	0	Train
TNKS1_ML_2078	COc1ccc(-n2c(C)c(C(=O)c3cc(OC)ccc3OC)c3c(C[NH+](C)C)c(O)ccc32)cc1	0	Train
TNKS1_ML_2079	CC[C@@H]1CCCN(c2nc(C)cc(C)n2)C1	0	Train
TNKS1_ML_2080	C[C@@H]1Cc2nc(NC3CC3)ncc2[C@@H](C)C1	0	Train
TNKS1_ML_2081	C[C@@H]1[C@@H](C(=O)[O-])CCN1C(=O)N1CCC(C(C)(C)C)CC1	0	Train
TNKS1_ML_2082	CCS(=O)(=O)C1=NN2C(=[NH2+])/C(=C\c3cc(C)n(-c4ccc(C)c(C)c4)c3C)C(=O)N=C2S1	0	Train
TNKS1_ML_2083	Cc1ccc(/C=C/c2onc(C)c2S(=O)(=O)N2CCC(C(=O)Nc3c(C)cc(C)cc3C)CC2)cc1	0	Train
TNKS1_ML_2084	Cc1cc(NC(=O)NC2CCCCC2)ncc1Br	0	Train
TNKS1_ML_2085	Cc1cnc(NN)nc1NCc1sccc1C	0	Train
TNKS1_ML_2086	CC(C)(C)NC(=O)[C@H](c1cccs1)N(C(=O)C(=O)NCc1ccc2c(c1)OCO2)c1ccc[nH+]c1	0	Train
TNKS1_ML_2087	O=C([O-])[C@@H]1C[NH2+]C[C@]12CCc1ccccc12	0	Train
TNKS1_ML_2088	C[C@H](O)c1ccc(N2CCCC(C)(C)CC2)cc1	0	Train
TNKS1_ML_2089	O=c1[nH]c(-c2ccccc2)cc2c1sc1ccccc12	1	Train
TNKS1_ML_2090	C[C@](N)(c1nc(C2Cc3ccccc3C2)no1)C(F)(F)F	0	Train
TNKS1_ML_2091	Cc1ccccc1N1C(=O)N=C([O-])/C(=C/c2ccc(-c3ccc(F)cc3)o2)C1=O	0	Train
TNKS1_ML_2092	O=C(CN1CCC(c2n[nH]c3cc(F)ccc23)CC1)NCc1nc2c(c(=O)[nH]1)COCC2	1	Train
TNKS1_ML_2093	C[C@@H](Sc1nc(N)c2ccsc2n1)C(N)=O	0	Train
TNKS1_ML_2094	CC[C@@H](C)N(C)C(=O)Nc1cc(C)c(C)c(C(=O)[O-])c1	0	Train
TNKS1_ML_2095	CS(=O)(=O)CC[C@H](NC(=O)c1cc(Br)nn1-c1ncccc1Cl)C(=O)[O-]	0	Train
TNKS1_ML_2096	C[C@H](Cc1cccs1)NC(=O)c1ccc(Cl)cc1[O-]	0	Train
TNKS1_ML_2097	CCOC(=O)C1=C(N)N2C(=O)[C@H](C)SC2=C(C(=O)OC)[C@@H]1c1cc(OC)c(OC)c(OC)c1	0	Train
TNKS1_ML_2098	O=c1[nH]c(N2CCC3(CC2)CNc2cc(F)ccc23)nc2c1CCCC2	1	Train
TNKS1_ML_2099	Cn1cc([C@H]2SCC(O)=Nc3c2cnn3-c2cc(F)ccc2Cl)cn1	0	Train
TNKS1_ML_2100	COc1ccc(-c2noc(CSc3nnc(-c4ccncc4)n3-c3ccc(C)cc3)n2)cc1	1	Train
TNKS1_ML_2101	CN1CC[C@@H](Nc2nc(Cl)nc(N3CCCC3)n2)C1	0	Train
TNKS1_ML_2102	O=c1[nH]c(-c2ccc(C(F)(F)F)cc2)nc2c1CCOC2	1	Train
TNKS1_ML_2103	COc1ccccc1[C@H]1[C@H](C(=O)Nc2ccccc2C)C(=O)C[C@](C)(O)[C@H]1C(=O)Nc1ccccc1C	0	Train
TNKS1_ML_2104	CCCCOc1ccc(C(=O)[C@@H]2C(=O)C(=O)N(Cc3ccc[nH+]c3)[C@@H]2c2ccc(O)c(OCC)c2)c(C)c1	0	Train
TNKS1_ML_2105	O=c1[nH]c2cc([N-]S(=O)(=O)c3ccc(F)cc3)ccc2o1	0	Train
TNKS1_ML_2106	CC(C)CN1C(=O)N(C2Cc3ccccc3C2)C(=O)C12CCN(C[C@H]1C=NN=C1c1ccccc1)CC2	0	Train
TNKS1_ML_2107	CCOc1cc([C@H]2C(C(=O)OCCOC)=C(C)N=C3C[C@@H](c4ccc(OC)c(OC)c4)CC(=O)C32)ccc1OC(C)=O	0	Train
TNKS1_ML_2108	O=C(Nc1ccc(Cl)c([O-])c1)c1ccc(Cl)s1	0	Train
TNKS1_ML_2109	O=C(Nc1ccc(Cl)cc1OCC(F)(F)F)N1CCC[C@@H](O)C1	0	Train
TNKS1_ML_2110	O=c1[nH]c(N2CCC3(CC2)NCc2ccccc23)nc2c1CCCC2	1	Train
TNKS1_ML_2111	COc1cccc(C(=O)/C=C\Nc2ccc(S(=O)(=O)Nc3cnc4ccccc4n3)cc2)c1	0	Train
TNKS1_ML_2112	CC(=O)N1C=Cc2ccccc2[C@H]1CC(=O)Nc1cc(Cl)ccc1-n1cncn1	0	Train
TNKS1_ML_2113	Cc1ccc2c(c1C)c(=O)n(C[C@H]1CC[C@H](C(=O)Nc3ccncc3)CC1)c(=O)n2Cc1cccc(C(F)(F)F)c1C#N	1	Train
TNKS1_ML_2114	CCNC(=O)c1c(NC(=O)[C@@]2(C)Oc3ccc(C)cc3N(C)C2=O)sc2c1CCCC2	0	Train

TNKS1_ML_2115	O=[N+]([O-])c1ccc2nc(SCc3nc4ccccn4c3Br)[nH]c2c1	0	Train
TNKS1_ML_2116	CC(C)(C)c1ccc(-c2cn3cncc3c(=O)[nH]2)cc1	1	Train
TNKS1_ML_2117	CS(=O)(=O)NCc1cc2n(n1)CCC[NH+](Cc1ccc(C(=O)[O-])cc1)C2	0	Train
TNKS1_ML_2118	C[NH+](C)CCN1C(=O)C(O)=C(C(=O)c2ccc3c(c2)OCCO3)[C@H]1c1ccc(Br)cc1	0	Train
TNKS1_ML_2119	CCc1ccc([C@@H]2C(C(=O)c3cccc(S(=O)(=O)N(C)C)c3)=C([O-])C(=O)N2CCC[NH+](C)C)cc1	0	Train
TNKS1_ML_2120	C[C@H]1CCC[NH+]([C@@H]2CCCCC[C@@H]2C(=O)[O-])C1	0	Train
TNKS1_ML_2121	CC(C)C1Cc2nc(O)c(C#N)cc2CO1	1	Train
TNKS1_ML_2122	CN1C(=O)[C@@]2(C(C(=O)c3ccc(S(=O)(=O)N(C)C)cc3)=C(O)C(=O)N2CCC[NH+](C)C)c2ccccc21	0	Train
TNKS1_ML_2123	Cc1cc(C)c2c(NC(=O)c3ccc(F)cc3)c(C(=O)N3CCOCC3)sc2[nH+]1	0	Train
TNKS1_ML_2124	CC(C)[C@@H](NS(=O)(=O)c1cccc2nsnc12)C(=O)N1CCN(c2cccc[nH+]2)CC1	0	Train
TNKS1_ML_2125	C=C[C@]1(C)C=C2C(=O)C[C@@H]3C(C)(C)CCC[C@@]3(C(=O)[O-])[C@@]2(O)CC1	0	Train
TNKS1_ML_2126	Cc1ccc(C)c(S(=O)(=O)N(CCC(=O)N2CCNCC2)CC[NH+]2C[C@@H](C)C[C@@H](C)C2)c1	0	Train
TNKS1_ML_2127	O=C(Nc1ccc([N-]S(=O)(=O)c2ccc3c(c2)OCCCO3)cc1)[C@@H]1COc2ccccc2O1	0	Train
TNKS1_ML_2128	CC1(C)CCC[C@@]2(CC(=O)NC2(C)C)C1	0	Train
TNKS1_ML_2129	CCOc1ccc(C(=O)C2=C(O)C(=O)N(CCCn3cc[nH+]c3)[C@H]2c2cc(OC)c(OC)c(OC)c2)cc1Cl	0	Train
TNKS1_ML_2130	NC(=O)c1cccc2c1O/C(=C\c1ccc(C(=O)N3CCn4c(nnc4C(F)(F)F)C3)cc1)C2=O	1	Train
TNKS1_ML_2131	COc1cc(CN(C(=O)CCn2ccc(C)n2)[C@H]2CCCCNC2=O)ccc1OCCc1cccc[nH+]1	0	Train
TNKS1_ML_2132	Nc1ccc(Br)c(F)c1[N+](=O)[O-]	0	Train
TNKS1_ML_2133	CC1CCN(c2ncnc(NC34CC5CC(CC(C5)C3)C4)c2[N+](=O)[O-])CC1	0	Train
TNKS1_ML_2134	COc1ccc([C@@H]2C3=C(CC(C)(C)CC3=O)Oc3ncn4nc(CSc5nc6ccccc6o5)nc4c32)cc1OC	0	Train
TNKS1_ML_2135	O=c1cc(-c2ccc(O)c(O)c2)oc2ccccc12	1	Train
TNKS1_ML_2136	COc1cc(C(=O)N/N=C/c2c(F)c(F)c(F)c(F)c2F)cc(OC)c1OC	0	Train
TNKS1_ML_2137	C[C@H]1[C@H](C)S(=O)(=O)CCN1C(=O)NC1CCC1	0	Train
TNKS1_ML_2138	Cc1cc(C)[nH+]c(N2CCC(C)(C(=O)[O-])CC2)n1	0	Train
TNKS1_ML_2139	C[C@H](NS(=O)(=O)c1c[nH]c(C(=O)N2CCCC2)c1)c1ccc(F)c(Cl)c1	0	Train
TNKS1_ML_2140	COc1ccc(-c2c3c(=O)n(C)c(=O)n(C)c3c3n2CCO[C@@H]3c2c(O)ccc3ccccc23)cc1	0	Train
TNKS1_ML_2141	O=C(c1cccc(Br)c1)N1N=C(C(F)(F)F)C[C@]1(O)c1ccc([N+](=O)[O-])cc1	0	Train
TNKS1_ML_2142	C[NH+](C)CC1CCN(C(=O)N2CC[C@](C)(C(=O)[O-])C2)CC1	0	Train
TNKS1_ML_2143	O=C(NNC(=O)C1CC1)c1cc(-c2ccccc2)nc2c1cnn2Cc1cccc(F)c1	0	Train
TNKS1_ML_2144	COc1cc(/C=N\N=C2\NC(=O)CS2)ccc1OS(=O)(=O)c1ccc(Cl)cc1	0	Train
TNKS1_ML_2145	Cn1ccc2c(=O)[nH]c(N3CCC4(CC3)C(=O)N(CCO)c3cc(F)cc(F)c34)nc21	1	Train
TNKS1_ML_2146	COc1cc2nc(-c3cc4c(s3)CCC4)[nH]c2cc1OC	0	Train
TNKS1_ML_2147	CC1=C(C(=O)Nc2nc3ccc(Br)cc3s2)[C@H](c2ccc(Cl)c([N+](=O)[O-])c2)C2=C(CC(C)(C)CC2=O)N1	0	Train

TNKS1_ML_2148	COc1ccc(C(=O)C2CCN(C3CCN(CC(=O)Nc4nnc5n4CCS5)C3=O)CC2)cc1	1	Train
TNKS1_ML_2149	C[C@@H]1C[S@](=O)[C@@H]2CCCCCCCCCC[C@@H]2N1	0	Train
TNKS1_ML_2150	[O-]/C(=N\c1nnc(N2CCCCCC2)s1)c1cc(-c2ccc(Br)cc2)on1	0	Train
TNKS1_ML_2151	O=c1cc(-c2cccc(O)c2)oc2ccccc12	1	Train
TNKS1_ML_2152	O=C(NCc1nc(-c2ccncc2)no1)[C@@H]1CC=CC[C@@H]1c1nc2ccccc2s1	0	Train
TNKS1_ML_2153	COc1cc(/C=C2\SC(=S)N(CCC(=O)Nc3ccc(C(C)=O)cc3)C2=O)cc(OC)c1OC	0	Train
TNKS1_ML_2154	CCOc1ccc(C(=O)C2CCN(CC(=O)NCc3nc4c(c(=O)[nH]3)COCC4)CC2)cc1	1	Train
TNKS1_ML_2155	COc1ccccc1NC(=O)/C=C/c1ccc2c(c1)OCO2	0	Train
TNKS1_ML_2156	CCn1c(SCc2nc3c4c(C)c(C(=O)OC)sc4ncn3n2)nnc1-c1cc(C(F)(F)F)n(C)n1	0	Train
TNKS1_ML_2157	COc1ccc2nc([C@@H](C)Cl)[nH]c2n1	0	Train
TNKS1_ML_2158	C[C@H](C(=O)Nc1ccc2c(c1)OCCO2)[NH+]1CCc2sccc2C1	0	Train
TNKS1_ML_2159	CN1CCO[C@H](c2nc(N)c(Br)c(C3CC3)n2)C1	0	Train
TNKS1_ML_2160	O=C(CSc1oc(-c2ccc(F)cc2)nc1S(=O)(=O)c1ccc(Br)cc1)NC[C@@H]1CCCO1	0	Train
TNKS1_ML_2161	COc1ccc([C@@H]2c3cc(OC)c(OC)cc3CC(=O)N2NC(=O)c2ccc([N+](=O)[O-])cc2)cc1OC	0	Train
TNKS1_ML_2162	CC[C@H](C)N1C(=O)/C(=C/c2c(N3CCN(CCO)CC3)nc3c(C)cccn3c2=O)SC1=S	0	Train
TNKS1_ML_2163	CCCOc1cc2nc(C)sc2cc1N	0	Train
TNKS1_ML_2164	CCC(C)(C)C1CCC2(CC1)NC(=O)N(CC(=O)N1C[C@@H](C)C[C@@H](C)C1)C2=O	0	Train
TNKS1_ML_2165	C[C@@H]1[C@@H](NC2=[NH+]N=C(C3=CC(C(=O)N4CCCC4)=NC3)CS2)C(=O)N(c2ccccc2)N1C	0	Train
TNKS1_ML_2166	CC[C@@H]1CCCCN1C(=O)NCC(=O)[O-]	0	Train
TNKS1_ML_2167	NCc1ccc2[nH]c(-c3ccc4nccnc4c3)nc2c1	0	Train
TNKS1_ML_2168	Nc1nc(/C=C/c2ccc3c(c2)OCCO3)cs1	0	Train
TNKS1_ML_2169	O=C(Cc1ccsc1)Nc1ccc2c(c1)OC1(CCCC1)O2	0	Train
TNKS1_ML_2170	Cc1cc(C(=O)Nc2ccc3nc(C)oc3c2)c2ccc(F)cc2n1	0	Train
TNKS1_ML_2171	C[C@H]1CCC[C@](C#N)(NC2CCCC2)CC1	0	Train
TNKS1_ML_2172	C[C@H](Oc1cccc(Br)c1)C(=O)Nc1cccc(S(=O)(=O)N(C)C)c1	0	Train
TNKS1_ML_2173	C[C@H](NC(=O)C(=O)NCC[NH+]1CCN(C(=O)c2ccc([N+](=O)[O-])cc2)CC1)c1ccccc1	0	Train
TNKS1_ML_2174	O=c1[nH]c2ccccc2n1[C@H]1C[C@H](c2nnc(-c3ccncn3)n2-c2ccccc2Cl)C1	1	Train
TNKS1_ML_2175	Cc1ccc(NC(=O)c2ccc(-n3cncn3)cc2)cc1S(=O)(=O)N1CCCCCC1	0	Train
TNKS1_ML_2176	Oc1ccccc1/C=N\n1c([S-])nnc1C(F)(F)F	0	Train
TNKS1_ML_2177	O=C1C(=O)N(c2nccs2)[C@H](c2ccc(F)cc2)C1=C(O)c1ccc(S(=O)(=O)N2CCCC2)cc1	0	Train
TNKS1_ML_2178	O=C(N[C@H]1C[C@H](c2nnc(-c3ccccn3)n2-c2ccccc2F)C1)c1ccnc2cc(F)cnc12	1	Train
TNKS1_ML_2179	O=S(=O)(/N=c1\[nH]ccs1)c1ccc(Cl)s1	0	Train
TNKS1_ML_2180	COc1ccc(C(=O)C2CCN([C@H]3CCN(Cc4nc5ccccc(=O)c5[nH]4)C3=O)CC2)cc1C	1	Train
TNKS1_ML_2181	Cc1cc(N2CCN(C(=O)[C@H](C)NC3=NS(=O)(=O)c4ccccc43)CC2)nc(C(C)C)n1	0	Train
TNKS1_ML_2182	COc1cc(C[C@]2(CCc3cccc[nH+]3)C(=O)NC(=O)N(c3ccccc3F)C2=O)cc(OC)c1OC	0	Train
TNKS1_ML_2183	Cc1ccc(N2C(=O)C[C@H](C(=O)NNC(=O)c3cc(C)oc3C)[C@@H]2c2cccs2)cc1	0	Train

TNKS1_ML_2184	Nc1ccc(I)cc1-c1nc(-c2ccco2)no1	0	Train
TNKS1_ML_2185	O=C(Nc1ccc(Cl)cn1)c1cnn2c1N[C@@H](c1ccco1)C[C@@H]2C(F)(F)F	0	Train
TNKS1_ML_2186	CC(C)(C)c1cc(C[NH+]2CC[C@@]3(O)CCN(S(C)(=O)=O)C[C@H]3C2)n[nH]1	0	Train
TNKS1_ML_2187	CC(C)c1ccc(NCc2cn(C)nn2)c(C(C)C)c1	0	Train
TNKS1_ML_2188	O=C(CCc1ccccc1Cl)Nc1nnc2n1CCS2	1	Train
TNKS1_ML_2189	C[C@]12CC[C@@H]3[C@H](CCC4=C/C(=N\OCC(=O)NCCc5c[nH]cn5)CC[C@@]43C)[C@@H]1CC[C@@H]2O	0	Train
TNKS1_ML_2190	OC1=Nc2[nH]ncc2[C@H](c2ccccc2Cl)SC1	0	Train
TNKS1_ML_2191	COc1ccc(C(=O)C2CCN(CC(=O)NCc3nc4c(c(=O)[nH]3)CCCC4)CC2)cc1	1	Train
TNKS1_ML_2192	C=C1C[C@H]2CC(C)(C)N(C1)N2	0	Train
TNKS1_ML_2193	C[C@H]1CCCCCN1C(=O)[C@@H]1CCCC[C@@H]1C(=O)O	0	Train
TNKS1_ML_2194	O[C@H]1[C@H]([NH2+]Cc2ccccn2)[C@H]2CO[C@H](O2)[C@@H]1N1CCN(c2ccccc2)CC1	0	Train
TNKS1_ML_2195	Cc1oc2c(c(C)cc3o/c(=N/N)c4c(c32)CCC4)c1C	0	Train
TNKS1_ML_2196	COc1ccccc1-c1ccc2c(C)cc(=O)[nH]c2c1	1	Train
TNKS1_ML_2197	O=c1[nH]c2ccccc2n1[C@H]1CC[C@H](c2nnc(-c3ccncn3)n2-c2ccccc2Cl)CC1	1	Train
TNKS1_ML_2198	CCOc1ccccc1NC(=O)N1CCN(Cc2nnnn2-c2ccc(F)c(F)c2)CC1	0	Train
TNKS1_ML_2199	O=[N+]([O-])c1cc(F)cc(Cl)c1F	0	Train
TNKS1_ML_2200	Cc1cc(/C=N/Nc2cccc[nH+]2)c(C)n1-c1cccc(S(=O)(=O)N2CCOCC2)c1	0	Train
TNKS1_ML_2201	CCCn1nc(N)cc1C(F)(F)F	0	Train
TNKS1_ML_2202	Cc1cc(C)c2cc([C@H](c3nnnn3Cc3ccco3)N3CCN(Cc4ccccc4)CC3)c(=O)[nH]c2c1	0	Train
TNKS1_ML_2203	COc1cccc(S(=O)(=O)NC[C@@H](O)Cn2ccccc2=O)c1	0	Train
TNKS1_ML_2204	C[C@@H](COCCC(=O)N1CCN(c2ncc(C(F)(F)F)cn2)CC1)Nc1cn[nH]c(=O)c1C(F)(F)F	1	Train
TNKS1_ML_2205	CC(C)CC(=O)Nc1nnc2n1CCS2	1	Train
TNKS1_ML_2206	C1=NC=C2CC1=c1cc3c(cc12)=[NH+]C=N3	0	Train
TNKS1_ML_2207	Nc1c2ccccc2nc(=O)n1C1CCCCC1	0	Train
TNKS1_ML_2208	CS(=O)(=O)c1cc(C(=O)NCCCN2CCCCCC2=O)c(Cl)cc1Cl	0	Train
TNKS1_ML_2209	O=c1[nH]c(SCCN2CCN(CCCOc3ccc(-c4ccccc4)cc3)CC2)nc2ccccc12	1	Train
TNKS1_ML_2210	CCc1ccc(S(=O)(=O)Nc2ccccc2C#CC[NH3+])s1	0	Train
TNKS1_ML_2211	CC1(C)OC(=O)N([C@H]2CC[C@H](NC(=O)c3cccc4cccnc34)CC2)[C@@H]1c1ccccc1	1	Train
TNKS1_ML_2212	Cc1[nH+]ccn1CC1(O)CCN(C(=O)[C@@H]2CC(=O)N(c3ccccc3C(C)C)C2)CC1	0	Train
TNKS1_ML_2213	COc1cccc2sc(NC(=O)c3ccc(S(=O)(=O)N(CCC#N)CCC#N)cc3)nc12	0	Train
TNKS1_ML_2214	COc1ccccc1N1CCCN(c2nc3c(c(=O)[nH]2)CCCC3)CC1	1	Train
TNKS1_ML_2215	CC(C)[C@H](NC(=O)N1CCN(S(=O)(=O)c2cccc(F)c2)CC1)c1ccc2c(c1)OCCO2	0	Train
TNKS1_ML_2216	Cc1ccc([C@H](C)NC(=O)N2CCN(c3[nH+]ccs3)CC2)cc1	0	Train
TNKS1_ML_2217	CCOc1ccccc1[C@H]1c2[nH]c3ccccc3c2C[C@H]2C(=O)N(CCC[NH+](CC)CC)CC(=O)N21	0	Train
TNKS1_ML_2218	O[C@@H](c1cc(Cl)c2c(c1)OCO2)[C@@H]1CCCCCN1	0	Train
TNKS1_ML_2219	COc1cc(Cl)c(C)cc1NC(=O)c1cnc2c(-c3ccccc3)c(C)nn2c1C	0	Train
TNKS1_ML_2220	C[C@H](Sc1nnc(-c2cccnc2)o1)c1nc([O-])c2c(-c3cccs3)csc2n1	0	Train
TNKS1_ML_2221	O=C1NC2(CCCC2)C(=O)N1CC(=O)N1N=C2/C(=C/c3cccs3)CCC[C@H]2[C@@H]1c1cccs1	0	Train

TNKS1_ML_2222	Cc1ccc2oc3c(c(=O)c2c1)[C@H](c1ccc(OCC(N)=O)cc1)N([C@H]1CCS(=O)(=O)C1)C3=O	0	Train
TNKS1_ML_2223	CC(C)(C)c1cc(C(F)(F)F)c(Cl)nn1	0	Train
TNKS1_ML_2224	Nc1ccc2ncnc(Sc3nc4ccccc4o3)c2c1	0	Train
TNKS1_ML_2225	C[C@@H]1CCC[C@@H]1NC(=O)c1cc(Cl)c(Cl)c([N+](=O)[O-])c1	0	Train
TNKS1_ML_2226	O=C(Nc1ccc(F)cc1)N1CCC2(CC1)O[C@@H](C(=O)N1CCOCC1)Cn1cc[nH+]c12	0	Train
TNKS1_ML_2227	O=C(COC(=O)CN1C(=O)CSc2ccccc21)Nc1cccc(Cl)c1	0	Train
TNKS1_ML_2228	COc1ccc(C(=O)C2=C([O-])C(=O)N(CCCn3cc[nH+]c3)[C@H]2c2cccs2)c(OC)c1	0	Train
TNKS1_ML_2229	COc1cccc(C2=C[C@@H](c3ccc(SC)cc3)N3NN=NC3=[NH+]2)c1	0	Train
TNKS1_ML_2230	Cc1ccc(C(C)C)c(OCc2nnc(S[C@@H](C)C(=O)N(C)[C@H]3CCS(=O)(=O)C3)n2N)c1	0	Train
TNKS1_ML_2231	COc1ccc(CNS(=O)(=O)c2cc(N3C(=O)CCS3(=O)=O)ccc2OC)cc1	0	Train
TNKS1_ML_2232	Cc1sc2nc(CSC(N)=[NH2+])nc([O-])c2c1C	0	Train
TNKS1_ML_2233	CC[C@@H](C)N(Cc1nc2c(c(=O)[nH]1)COCC2)C(=O)CN1CCC(C(=O)c2ccc(F)cc2)CC1	1	Train
TNKS1_ML_2234	O=C([O-])c1c(F)cccc1Oc1ccc2c(c1)OCO2	0	Train
TNKS1_ML_2235	COc1cccc(/C=N/NC(=O)c2cccc(Br)c2)c1OS(=O)(=O)c1ccccc1	0	Train
TNKS1_ML_2236	Nc1ccc2nn(-c3ccccc3)nc2c1Br	0	Train
TNKS1_ML_2237	CC1(C)CCc2c(cc(OCC(=O)NCCCn3cc[nH+]c3)c3c4c(c(=O)oc23)CCC4)O1	0	Train
TNKS1_ML_2238	Cc1ccc(F)c2c1sc1nncn12	1	Train
TNKS1_ML_2239	O=C(Nc1ccc2c(c1)OCCO2)[C@H]1CCCN(c2ccc3cc(S(=O)(=O)N4CCCC4)ccc3n2)C1	0	Train
TNKS1_ML_2240	COc1ccc(CC/[NH+]=C(\O)c2ccc(N3C[C@@H]4C[C@H](C3)Cn3c4cccc3=O)c([N+](=O)[O-])c2)cc1OC	0	Train
TNKS1_ML_2241	COc1ccccc1C(=O)C1CCN(CC(=O)N(Cc2nc3c(c(=O)[nH]2)COCC3)CC(F)F)CC1	1	Train
TNKS1_ML_2242	CNc1ccc(Cl)cc1C(=O)N[C@@H](C)c1ccc(C)s1	0	Train
TNKS1_ML_2243	Cc1nc2oc3c(NCc4ccccc4)ncnc3c2c2c1COC(C)(C)C2	0	Train
TNKS1_ML_2244	C=C1N=C(SCC(=O)Nc2cc(Cl)ccc2OC)[C@@H](C#N)[C@H](c2ccco2)C1C(N)=O	0	Train
TNKS1_ML_2245	Cc1ccc2c(=O)[nH]c(-c3ccc(C4(O)CC4)cc3)cn12	1	Train
TNKS1_ML_2246	Cc1ccc(NC(=O)[C@H](C)OC(=O)Cc2c(C)noc2C)cc1S(=O)(=O)N1CCOCC1	0	Train
TNKS1_ML_2247	CCOc1ccc(-c2nnc([C@H]3C[C@H](NC(=O)c4ccccn4)C3)n2-c2cccc(Cl)c2)nc1	1	Train
TNKS1_ML_2248	Nc1cc(C(=O)c2ccc3c(c2)OCCCO3)ccc1Br	0	Train
TNKS1_ML_2249	C[C@@H](OC(=O)c1cccc(S(=O)(=O)Nc2ccc(F)cc2)c1)C(=O)Nc1ncc(Cl)cc1Cl	0	Train
TNKS1_ML_2250	[H]/N=C1C(=C\c2ccc(OCCOc3ccc(C)c(C)c3)c(OC)c2)/C(=O)N=C2SC(CC)=NN2/1	0	Train
TNKS1_ML_2251	C[C@H](c1cccc(Cl)c1)N1CCN(c2ccc([O-])nn2)CC1	0	Train
TNKS1_ML_2252	Cc1nn(CC(=O)Nc2ccc3c(c2)OCCO3)c(C)c1S(=O)(=O)N(C)Cc1ccco1	0	Train
TNKS1_ML_2253	O=Cc1sc(Sc2ncn[nH]2)nc1Cl	0	Train
TNKS1_ML_2254	O=C(Cc1ccccc1[N+](=O)[O-])N1CCc2[nH]nc(C(=O)N3CCN(Cc4ccccc4)CC3)c2C1	0	Train
TNKS1_ML_2255	Cn1cc(N[C@@H]2CCc3cc(Br)ccc32)ccc1=O	0	Train
TNKS1_ML_2256	CC1(C)Cc2cccc(OCC(=O)Nc3cccc(S(=O)(=O)/N=C4/CCCN4)c3)c2O1	0	Train
TNKS1_ML_2257	CC(C)(C)OC(=O)N1CCC2(CC1)CCN(C(=O)c1cc(Cc3n[nH]	1	Train

	c(=O)c4ccccc34)ccc1F)CC2		
TNKS1_ML_2258	Cc1ccc(-n2ncc3c2C[C@H](c2ccccc2O)CC3=O)cc1	0	Train
TNKS1_ML_2259	Cc1ccccc1-n1nc(-c2cccc(C(F)(F)F)c2)c(C#N)c1N	0	Train
TNKS1_ML_2260	C[C@H]1CC[C@@H](C)C[C@H]1Nc1ccc(-n2cncn2)nc1	0	Train
TNKS1_ML_2261	Cc1nc2ncnc(N)c2c(-c2cccs2)c1C	0	Train
TNKS1_ML_2262	O=c1[nH]nc2c3c(cccc13)NC1=C2CCCCCC1	1	Train
TNKS1_ML_2263	C[C@H]1CC[C@H](C#N)[C@H](N2CCCCC[C@@H]2C)C1	0	Train
TNKS1_ML_2264	COc1ccccc1C1CCN(c2nc3c(c(=O)[nH]2)CCCC3)CC1	1	Train
TNKS1_ML_2265	CC(C)(C)C(=O)CN(Cc1nc2c(c(=O)[nH]1)COCC2)C(=O)CCc1ccccc1	1	Train
TNKS1_ML_2266	O=C(CS[C@@H]1NN=C(C2=c3ccccc3=[NH+]C2)O1)Nc1ccc(S(=O)(=O)N2CCCC2)cc1	0	Train
TNKS1_ML_2267	COc1ccc(Br)cc1S(=O)(=O)[N-]c1cccc(-c2ccc(N3CCCC3)nn2)c1	0	Train
TNKS1_ML_2268	Cc1ccc([C@H]2C(=C(O)c3ccc(S(=O)(=O)N(C)C)cc3)C(=O)C(=O)N2Cc2ccc[nH+]c2)o1	0	Train
TNKS1_ML_2269	CN1CCCc2c1nc(N1CCC3(CC1)C(=O)N(CC(F)(F)F)c1cc(F)cc(F)c13)[nH]c2=O	1	Train
TNKS1_ML_2270	COc1ccc(C(=O)C2CCN(C3CCN(CC(=O)Nc4nnc5ccccn45)C3=O)CC2)cc1	1	Train
TNKS1_ML_2271	O=C(Oc1ccc([N+](=O)[O-])cc1)c1cc(S(=O)(=O)N2CCCCC2)c(Cl)cc1Cl	0	Train
TNKS1_ML_2272	Cc1ccc2cc([C@H]3C[NH+]=C(N)N3C(C)C)ccc2n1	0	Train
TNKS1_ML_2273	COc1ccc(CCn2c(-c3cc(OC)c(OC)c(OC)c3)nc3c(c(=O)c4ccccc4n3C)c2=O)cc1	0	Train
TNKS1_ML_2274	O=C(c1cc2c(cc1[N+](=O)[O-])OCO2)N1CCN(Cc2ccc(C(F)(F)F)cc2)CC1	0	Train
TNKS1_ML_2275	COC(=O)c1sccc1NC(=O)CN1CCCN(Cc2ccccc2Cl)S1(=O)=O	0	Train
TNKS1_ML_2276	Oc1cncc(-c2nc([C@H]3Cc4ccccc43)no2)c1	0	Train
TNKS1_ML_2277	Cc1cc(/C=N/O)cnc1N1CCc2ccccc2C1	0	Train
TNKS1_ML_2278	Cn1c(CN2CCCCC2)cc(=O)c(O)c1CN1CCN(CCOc2ccccc2)CC1	0	Train
TNKS1_ML_2279	Cc1nc2c(ccc3nc(NC(=O)c4ccn(C(C)C)n4)sc32)s1	0	Train
TNKS1_ML_2280	Cc1cccc2c1c(=O)n(C[C@H]1CC[C@@H](C(=O)O)CC1)c(=O)n2Cc1cccc(C(F)(F)F)c1C#N	1	Train
TNKS1_ML_2281	CC(=O)Nc1ccc2nc(NC(=O)C[C@H]3c4ccccc4C=CN3C(C)=O)sc2c1	0	Train
TNKS1_ML_2282	C[C@H]1CC(C)(C)C[C@@]2(C1)NC(=O)N(CC(=O)N(Cc1ccco1)[C@@H]1CCS(=O)(=O)C1)C2=O	0	Train
TNKS1_ML_2283	O=c1[nH]c(N2CCC3(CC2)CNc2cc(F)cc(F)c23)nc2c1CCCC2	1	Train
TNKS1_ML_2284	c1ccc2c(c1)CCC1=C2OCO1	0	Train
TNKS1_ML_2285	N#CC1=c2ccc(cc2)=CC(C#N)=c2ccc(cc2)=C1	0	Train
TNKS1_ML_2286	Cc1ccn(-c2cc(Cl)c(N)cc2C)n1	0	Train
TNKS1_ML_2287	CC(=O)O[C@H]1[C@H]2[C@H](C[C@H](C)[C@H]3[C@H](OC(C)=O)C[C@H](O)[C@@]13C)OC(=O)[C@H]2C[NH+]1CCCC1	0	Train
TNKS1_ML_2288	COc1ccc([C@H]2C(C#N)=C([NH3+])Oc3c2c(C)nn3-c2ccccc2)cc1Cn1nc(C)c([N+](=O)[O-])c1C	0	Train
TNKS1_ML_2289	O=C(NCc1cc[nH+]c(N2CCCC2)c1)c1ccc(NS(=O)(=O)c2cccc([N+](=O)[O-])c2)cc1	0	Train
TNKS1_ML_2290	Cc1cc(N2CC[NH+](C)[C@H](C)C2)cc(C(=O)[O-])c1N	0	Train
TNKS1_ML_2291	Cc1cc([C@H](N)c2ccc(S(C)(=O)=O)cc2)c(C)cc1Cl	0	Train
TNKS1_ML_2292	CC(=O)N[C@H]1CCCN(C(=O)N[C@@H]2CCC[C@H](C)C2)C1	0	Train
TNKS1_ML_2293	Cc1cccc(CN2CCOc3c(O)cc(-c4ccc(C)s4)cc3C2)[nH+]1	0	Train

TNKS1_ML_2294	Cn1c(=O)oc2cc(S(=O)(=O)N3CCC[C@@H](C(=O)Nc4ccc(Cl)cc4)C3)ccc21	0	Train
TNKS1_ML_2295	COc1cc2nc(C[NH+](CCO)[C@H]3CS(=O)(=O)C[C@@H]3O)nc(N3CCCC3)c2cc1OC	0	Train
TNKS1_ML_2296	COc1ccc(C(=O)C2CCN(CC(=O)NCc3nc4c(c(=O)[nH]3)COCC4)CC2)cc1Cl	1	Train
TNKS1_ML_2297	Cc1cc(=O)n2nc([C@@H]3CCCN(C(=O)CCN4Cc5ccccc5C4=O)C3)cc2[nH]1	0	Train
TNKS1_ML_2298	CCOC(=O)c1sc(NC(=O)CSc2[nH+]c3c(c(=O)n(C)c(=O)n3C)n2C)nc1C	0	Train
TNKS1_ML_2299	CSc1nc(C(=O)N2CCc3c(c(C(=O)N4CCNC(=O)C4)nn3Cc3ccccc3)C2)cs1	0	Train
TNKS1_ML_2300	c1ccc2c(c1)CCN2c1ncnc2c1oc1ccccc12	0	Train
TNKS1_ML_2301	C[C@@H]1C[NH+](C)CC[C@@H]1N(C)C(=O)N[C@@H]1CCC(C)(C)c2ccccc21	0	Train
TNKS1_ML_2302	COc1ccc(CCN(Cc2cc3ccc(C)cc3[nH]c2=O)Cc2nnnn2CCc2ccccc2)cc1OC	0	Train
TNKS1_ML_2303	O=C1CCN(c2ccccc2C(=O)NCCc2ccc(Br)cc2)C(=O)N1	1	Train
TNKS1_ML_2304	Cc1ccc2c(=O)[nH]c(-c3ccc([C@@](C)(O)CO)cc3)cn12	1	Train
TNKS1_ML_2305	CC(C)[C@@H](CO)Nc1ncnc2ccc(N)cc12	0	Train
TNKS1_ML_2306	C[C@@H]1C[C@@H]2CCCC[C@@H]2N1C(=O)C1CC1	0	Train
TNKS1_ML_2307	Nc1nc(-c2cc(Cl)c3c(c2)OCCCO3)ncc1I	0	Train
TNKS1_ML_2308	C[C@@H]1C[NH+]2CCC[C@@H]2CN1c1ncc(C(=O)[O-])s1	0	Train
TNKS1_ML_2309	Cc1cn(CC(=O)N2CC[C@](Cc3cccc(-c4ccc[nH+]c4)c3)(C(=O)NC(C)C)C2)nn1	0	Train
TNKS1_ML_2310	C[NH+](C)CCCn1c(S)nnc1-c1cc2n(n1)[C@H](C(F)(F)F)C[C@@H](c1ccc(Cl)cc1)N2	0	Train
TNKS1_ML_2311	Cc1sc2nc(C[NH+]3C[C@H]4CC[C@@H]3C4)nc(NN)c2c1C	0	Train
TNKS1_ML_2312	N#C[C@H]1CCC[C@H]1N1CC2(CCCCC2)C1	0	Train
TNKS1_ML_2313	O=C(NCCCn1cc[nH+]c1)c1cc(S(=O)(=O)N2CCOCC2)c(Cl)cc1Cl	0	Train
TNKS1_ML_2314	Cc1c/c(=N/S(=O)(=O)c2cc(F)ccc2Cl)cc[nH]1	0	Train
TNKS1_ML_2315	Cc1cnc(CNC(=O)c2nn(C)cc2Cl)cn1	0	Train
TNKS1_ML_2316	Cc1ccc(C)c2c(C(=O)[O-])cc(-c3cnccn3)nc12	0	Train
TNKS1_ML_2317	CNS(=O)(=O)c1cc(C(=O)O[C@@H](C)c2nc3sc(C)c(C)c3c(=O)[nH]2)ccc1OC	0	Train
TNKS1_ML_2318	CCCCNC(=O)[C@@H]1Cc2cc(S(=O)(=O)N3CCCC3)ccc2N1C(C)=O	0	Train
TNKS1_ML_2319	Cc1c(C(=O)N2CCC(c3c[nH]c4ccccc34)CC2)sc2ncn(C)c(=O)c12	0	Train
TNKS1_ML_2320	Cc1nc2cc(N[C@H]3CS[C@H](C)C3)ccc2o1	0	Train
TNKS1_ML_2321	CC(=O)N1CCN(S(=O)(=O)c2cccc(C(=O)Nc3ccnn3Cc3ccccc3)c2)CC1	0	Train
TNKS1_ML_2322	Cc1nn(-c2nc3ccccc3s2)c2c1[C@H](c1cccc(OC3CCCC3)c1)CC(=O)N2	0	Train
TNKS1_ML_2323	CC1(C)OC(=O)N([C@H]2CC[C@H](c3cnc(N)c(-c4ncccn4)c3)CC2)[C@H]1c1ccccc1	1	Train
TNKS1_ML_2324	Nc1cc(Br)ccc1Oc1cnc2ccccc2n1	0	Train
TNKS1_ML_2325	[H]/N=C1/[C@H](C2CCC(C)CC2)[NH+]=C(C)N1CC#C	0	Train
TNKS1_ML_2326	C[C@@H]1CCCCN1C(=O)c1cccc(S(=O)(=O)NCc2ccc3c(c2)OCO3)c1	0	Train
TNKS1_ML_2327	Cc1ccc2c(=O)[nH]c(-c3ccc([C@](C)(O)CO)cc3)cn12	1	Train
TNKS1_ML_2328	CCOC(=O)c1ccc(-n2c(C)cc(/C=C3/C(=O)NC(=O)N(c4ccc(F)cc4)C3=O)c2C)cc1	0	Train

TNKS1_ML_2329	Oc1ccccc1/C=N/Nc1ncc(Cl)cc1Cl	0	Train
TNKS1_ML_2330	CCN(C(=O)N1CCC(C(=O)c2ccc3c(c2)cc(C(=O)O)n3C)CC1)c1ccc(OC)cc1	1	Train
TNKS1_ML_2331	COc1ccc(N2C(=O)CCCC2=O)cc1S(=O)(=O)Nc1ccc(C(F)(F)F)cc1	0	Train
TNKS1_ML_2332	CCn1nc(C)c(Br)c1C[NH2+]CC1(O)CCC(C)(C)CC1	0	Train
TNKS1_ML_2333	O=C(CCN1C(=O)S/C(=C\c2cccc([N+](=O)[O-])c2)C1=O)Nc1ccc(Br)cc1	0	Train
TNKS1_ML_2334	C[C@H]1CN[C@@H](c2cc(Br)c3c(c2)OCCO3)SC1	0	Train
TNKS1_ML_2335	COc1ccc(C(=O)C2CCN(CC(=O)NCc3nc4c(c(=O)[nH]3)COCC4)CC2)cc1C	1	Train
TNKS1_ML_2336	Cc1cccc2c1c(=O)n(C[C@H]1CC[C@H](C(=O)Nc3cnn(C)c3)CC1)c(=O)n2Cc1cccc(C(F)(F)F)c1C#N	1	Train
TNKS1_ML_2337	O[C@@H](C1CCCCCC1)[C@H]1CCO[C@]2(CCOC2)C1	0	Train
TNKS1_ML_2338	Oc1cnnc(SCc2nccc3ccccc23)n1	0	Train
TNKS1_ML_2339	COc1ccc(-n2c(SCc3nc(-c4ccc(C)cc4)no3)nnc2-c2ccncc2)cc1	1	Train
TNKS1_ML_2340	CCn1c(=O)c(=O)n(C/C(O)=N/CCN2CCN(c3cccc(Cl)c3)CC2)c2ccccc21	0	Train
TNKS1_ML_2341	C[C@@H]1CC[C@H](C(C)(C)[C@@H]2CCO[C@]3(CCOC3)C2)[C@H](O)C1	0	Train
TNKS1_ML_2342	COc1ccc(-c2csc(-n3nc(C)c([C@@H](c4ccccc4)[C@@H](C#N)C(N)=O)c3O)n2)cc1	0	Train
TNKS1_ML_2343	Cn1c(SCC(=O)NCC(C)(C)[NH+]2CCOCC2)nnc1[C@H]1COc2ccccc2O1	0	Train
TNKS1_ML_2344	CC(C)NC(=O)CCNC(=O)NC[C@H](c1ccc(F)cc1)[NH+](C)C	0	Train
TNKS1_ML_2345	Cc1ccc(NC(=O)Cn2cnc3c(oc4ccccc43)c2=O)cc1C	0	Train
TNKS1_ML_2346	CC1(C)OC(=O)N([C@H]2CC[C@H](C(=O)Nc3cccc4cccnc34)CC2)[C@H]1c1ccccc1	1	Train
TNKS1_ML_2347	CC1(C)CC2=NNC(=O)c3cccc4c3c2c(n4CCN2CCCCC2)C1	1	Train
TNKS1_ML_2348	Cn1c(=O)oc2cc(S(=O)(=O)Nc3ccc4c(c3)CCCC4)c(Cl)cc21	0	Train
TNKS1_ML_2349	Cc1nn(C)c2ncc(N[C@H]3CC[C@H](C)C[C@@H]3C)cc12	0	Train
TNKS1_ML_2350	Cc1nn(Cc2ccccc2Cl)c(C)c1NC(=O)c1cc(-c2ccc(F)cc2)on1	0	Train
TNKS1_ML_2351	O=C(CCc1c(Cl)cccc1Cl)Nc1nnc2n1CCS2	1	Train
TNKS1_ML_2352	CC[NH+]1CCC[C@H](NC(=O)N2CCC(C)(C(=O)[O-])CC2)C1	0	Train
TNKS1_ML_2353	COc1ccc(C(=O)C2CCN(C3CCN(Cc4nccc(=O)[nH]4)C3=O)CC2)cc1	1	Train
TNKS1_ML_2354	Cc1cccc2[nH+]c(CS[C@@H]3N=C4N=NC=C4C(=O)N3c3ccc(Br)cc3)cc(=O)n12	0	Train
TNKS1_ML_2355	Cc1ccccc1COc1ccc(/C=C2\C(=O)N(c3ccc(Br)cc3)C(=O)N=C2[O-])cc1	0	Train
TNKS1_ML_2356	Cc1nc(-c2cccs2)sc1-c1ccc(=O)n(CCNS(=O)(=O)c2cccc(Cl)c2)n1	0	Train
TNKS1_ML_2357	CC[C@@H](C)N1C(=O)[C@H]2[C@@H](C(=O)Nc3ccc(C(C)C)cc3)[C@H]3C=C[C@@]2(O3)[C@H]1C(=O)NC1CCCCC1	0	Train
TNKS1_ML_2358	Cc1ccc(C(=O)Nc2cccc(Br)c2)c([O-])c1	0	Train
TNKS1_ML_2359	Cc1ccc(NC(=O)C(=O)Nc2ccccc2C)c(C)c1	0	Train
TNKS1_ML_2360	O=C(CCc1ccncc1)Nc1nnc2n1CCS2	1	Train
TNKS1_ML_2361	O=S1(=O)C(/C([O-])=N/Cc2ccco2)=NN(c2ccc(F)cc2)c2ccc(F)cc21	0	Train
TNKS1_ML_2362	Cc1cc(NC(=O)c2cc(S(=O)(=O)N3CCOCC3)cn2C)ccc1Br	0	Train
TNKS1_ML_2363	C[C@@H](NC(=O)CN1C(=O)CCn2nc(-c3cn(C)c4ccccc34)cc21)c1ccc2c(c1)OCCO2	0	Train
TNKS1_ML_2364	CCOC(=O)[C@@H](Cc1ccc(/N=C/c2ccc(O)cc2[O-])cc1)N1C(=O)c2ccccc2C1=O	0	Train

TNKS1_ML_2365	Cc1c(CC(=O)N[C@@H]2CS(=O)(=O)C[C@H]2O)c(=O)oc2cc3c(cc12)CCC1(CCCCC1)O3	0	Train
TNKS1_ML_2366	CCCS(=O)(=O)Nc1ccc2n(c1=O)C[C@H]1C[C@@H]2CN(C(=O)c2ccc(N(C)C)cc2)C1	0	Train
TNKS1_ML_2367	Nc1ccc(F)cc1SC[C@@H]1COc2ccccc2O1	0	Train
TNKS1_ML_2368	N#C[C@@H]1Cc2cc([N+](=O)[O-])ccc2-c2cc(Cl)cc(Cl)c21	0	Train
TNKS1_ML_2369	C=CCN(CC=C)S(=O)(=O)c1ccc(C(=O)Nc2nnc(-c3ccc(S(=O)(=O)CC)cc3)o2)cc1	0	Train
TNKS1_ML_2370	Clc1ccc2c(c1)sc1nncn12	1	Train
TNKS1_ML_2371	CC(C)n1c(-c2cccc(N)c2)nc2ccc(F)cc21	0	Train
TNKS1_ML_2372	Cc1cc(=O)[nH]c2cc(-c3ccccc3Cl)ccc12	1	Train
TNKS1_ML_2373	O=C1C[C@@H](c2ccc(Br)cc2)c2cc3c(cc2N1)OCO3	0	Train
TNKS1_ML_2374	COc1cc(C(=O)NN[C@H](C(=O)C2=c3ccccc3=[NH+]C2)c2ccccc2)cc(OC)c1OC	0	Train
TNKS1_ML_2375	Cc1cc(Br)ccc1NC1(C(=O)[O-])CCCCC1	0	Train
TNKS1_ML_2376	CC[NH+](CC)CCN1C(=O)C(=O)[C@H](C(=O)c2ccc3c(c2)C[C@H](C)O3)[C@@H]1c1cccc(O)c1	0	Train
TNKS1_ML_2377	O=c1[nH]c2ccccc2c2ccccc12	1	Train
TNKS1_ML_2378	C[C@H]1CN(c2c(Cl)ccc3nsnc23)[C@@H](C)CN1	0	Train
TNKS1_ML_2379	N#Cc1ccc(O[C@H]2CC[C@H](NC(=O)CCSc3nc4ccccc4c(=O)[nH]3)CC2)cc1	1	Train
TNKS1_ML_2380	CC[C@H]1CO[C@@H](C)CN1S(=O)(=O)c1cc(N)c(C)c(Cl)c1	0	Train
TNKS1_ML_2381	CCCN1CCN(CCCNC(=O)c2cc3n(n2)C[C@@](C)(C(=O)NCc2ccccc2)N(C)C3=O)CC1	0	Train
TNKS1_ML_2382	Cc1cc(-c2c[nH]c([C@H]3CCCCN3)n2)c(C)o1	0	Train
TNKS1_ML_2383	COc1cccc(C[NH+](Cc2ccc3c(c2)OCO3)Cc2nc(C(=O)N3CCN(C)CC3)co2)c1OC	0	Train
TNKS1_ML_2384	Cc1nnc2sc(C(=O)N(C)C[C@H](C)C#N)c(N)c2c1C	0	Train
TNKS1_ML_2385	N#Cc1cccc(Nc2cccc(Cl)c2F)n1	0	Train
TNKS1_ML_2386	COc1ccc(C(=O)C2CCN(C3CCN(Cc4nc5c(cnn5C)c(=O)[nH]4)C3=O)CC2)cc1	1	Train
TNKS1_ML_2387	Oc1nc(-c2ccccc2)nc2ccccc12	1	Train
TNKS1_ML_2388	CCN1CCN(CCCN2CC3(CCN(Cc4ncc[nH]4)CC3)OC2=O)CC1	0	Train
TNKS1_ML_2389	O=C(N[C@H](C(=O)N/N=C/c1cc(Br)c(O)c(Br)c1)c1n[nH]c(=O)c2ccccc12)c1ccccc1	0	Train
TNKS1_ML_2390	O=C(c1ccc(F)cc1)C1CCN(CC(=O)N(Cc2cccc(F)c2)Cc2nc3c(c(=O)[nH]2)COCC3)CC1	1	Train
TNKS1_ML_2391	CC(C)C(=O)N/N=C1/C(=O)N(C)c2ccc(Br)cc21	0	Train
TNKS1_ML_2392	COc1ccc(S(=O)(=O)N(CC(=O)Nc2c(F)cccc2F)c2cc(Cl)ccc2OC)cc1OC	0	Train
TNKS1_ML_2393	CC(C)c1n[nH]c(=S)n1C1(C)CCCC1	0	Train
TNKS1_ML_2394	CC(C)(C)c1ccc(C(=O)Nc2nnc3n2CCCS3)cc1	1	Train
TNKS1_ML_2395	COC(=O)[C@]1(C)N[C@@H](c2ccc(F)cc2)[C@H]2C(=O)N(c3ccc4c(c3)OCCO4)C(=O)[C@H]21	0	Train
TNKS1_ML_2396	[O-][C@@H]1CN(C[C@H]2C=NN=C2c2ccc(Cl)cc2)CC[C@H]1[NH+]1CCCCCC1	0	Train
TNKS1_ML_2397	COC(=O)[C@@H]1O[C@@]12CC(C)(C)OC2(C)C	0	Train
TNKS1_ML_2398	O=C(/C=C/c1ccc(O)cc1)OC[C@H]1O[C@@H](OC(=O)c2cc([O-])c(O)c(O)c2)[C@H](O)[C@@H](O)[C@H]1O	0	Train
TNKS1_ML_2399	Cn1nc(C(=O)NCCCN2CCOCC2)c2c1CCN(C(=O)[C@H]1C=c3ccccc3=[NH+]1)C2	0	Train
TNKS1_ML_2400	CCSC1=[NH+]SC2=NC(=O)/C(=C\c3ccc(OCCOc4ccccc4)cc3)C(=[NH2+])N21	0	Train
TNKS1_ML_2401	O=C(N[C@H]1C[C@H](c2nnc(-c3ccccn3)n2-c2ccccc2F)C1)c1ccnc2cccnc12	1	Train

TNKS1_ML_2402	O=C(NCCCS(=O)(=O)N1CCN(c2ncccn2)CC1)C(c1ccccc1)c1ccccc1	0	Train
TNKS1_ML_2403	CC(C)c1ncc(N2CCC[C@@H]2C(C)C)c(CN)n1	0	Train
TNKS1_ML_2404	C[C@H](C(=O)N1CCN(C(=O)c2ccco2)CC1)N1C(=O)N[C@@](C)(c2ccc3c(c2)CCC3)C1=O	0	Train
TNKS1_ML_2405	Cc1nc([C@@]2(N)CSCC(C)(C)C2)no1	0	Train
TNKS1_ML_2406	CC1(C)CCC[C@H]1n1nnnc1-c1ccc(N)cc1Cl	0	Train
TNKS1_ML_2407	Nc1ncnc2c1ncn2C[C@H]1COc2ccccc21	0	Train
TNKS1_ML_2408	C[C@H]1CC[C@]2(C[C@@H]1C)C[C@@H](O)c1ccc(F)cc1O2	0	Train
TNKS1_ML_2409	Cc1ccc2cc([C@@H](c3nnnn3Cc3ccccc3)N3CC[NH+](Cc4ccccc4)CC3)c(=O)[nH]c2c1	0	Train
TNKS1_ML_2410	CC(=O)c1cccc(NC(=O)c2ccc(C/N=c3/c(N4CCCC4)c(O)c3=O)cc2)c1	0	Train
TNKS1_ML_2411	Cc1cc(NC(=O)/C=C\c2ccc(C)o2)no1	0	Train
TNKS1_ML_2412	CCCOC(=O)C1=C(C)N=C2C[C@@H](c3ccc(OC)c(OC)c3)CC(=O)C2[C@H]1c1cc(Br)c(O)c(OC)c1	0	Train
TNKS1_ML_2413	C[C@H]1CN(Cc2csc(NC(=O)c3cnn(-c4ccc(Cl)cc4)c3)n2)C[C@H](C)O1	0	Train
TNKS1_ML_2414	CC(C)=CC/C=C1\CC[C@@H]2C(=O)NNC(=O)[C@H]2C1	0	Train
TNKS1_ML_2415	COc1cc([C@@H]2Oc3ccc(C)cc3C3=Nc4ncnn4[C@H](c4ccc[nH+]c4)[C@@H]32)cc(OC)c1OC	0	Train
TNKS1_ML_2416	O=S(=O)([N-]c1ncc(C#CCO)s1)c1cccs1	0	Train
TNKS1_ML_2417	Cc1nc2sccn2c(=O)c1S(=O)(=O)NCCC[NH+]1CCN(c2ccccc2)CC1	0	Train
TNKS1_ML_2418	Cc1[nH]nc(C(=O)N2c3ccccc3CC[C@@H]2C)c1N	0	Train
TNKS1_ML_2419	O=C(CN1CCC(C(=O)c2ccc(OCC3CC3)cc2)CC1)NCc1nc2c(c(=O)[nH]1)COCC2	1	Train
TNKS1_ML_2420	O=C(Nc1cccc2cccnc12)c1ccc(N2C(=O)[C@H]3[C@H]4CC[C@H](C4)[C@H]3C2=O)cc1	1	Train
TNKS1_ML_2421	CC(C)C(=O)N1CCC(NC(=O)C2CCN(S(=O)(=O)c3cc(C(N)=O)n(C)c3)CC2)CC1	0	Train
TNKS1_ML_2422	COc1ccc(N2C(=O)/C(=C/c3ccc(OC(=O)c4ccccc4I)c(OC)c3)SC2=S)cc1	0	Train
TNKS1_ML_2423	Cc1ccc(-n2nnc(/C(O)=N/C(C)C)c2C)cc1Cl	0	Train
TNKS1_ML_2424	c1cc(N[C@H]2CCN(C3CC3)C2)cc(-c2nnn[n-]2)c1	0	Train
TNKS1_ML_2425	Cc1ccc([C@]2(O)CC[C@H]3C[NH+](Cc4cc5c(cc4C)OCO5)C[C@H]32)nc1	0	Train
TNKS1_ML_2426	[H]/N=C1C(=C\c2cc(Cl)c(OC(=O)c3ccco3)c(OC)c2)/C(=O)N=C2SC(CC)=NN2/1	0	Train
TNKS1_ML_2427	Cc1nn(C)c(C)c1N[C@@H]1CCCC[C@H]1C	0	Train
TNKS1_ML_2428	C[C@@H]1CCCCCN1c1cc(Cl)nc(N)n1	0	Train
TNKS1_ML_2429	COc1cccc(N2C[C@H](C(=O)NCCN3C(=O)S/C(=C\c4ccc(C)cc4)C3=O)CC2=O)c1	0	Train
TNKS1_ML_2430	CCCOC(=O)CCc1cc2c(C)cc(=O)oc2c(C[NH+]2C[C@@H]3C[C@@H](Cn4c3cccc4=O)C2)c1[O-]	0	Train
TNKS1_ML_2431	O=C(c1ccc(F)cc1)C1CCN(CC(=O)N(Cc2ccccn2)Cc2nc3c(c(=O)[nH]2)COCC3)CC1	1	Train
TNKS1_ML_2432	COc1ccc(S(=O)(=O)Nc2cc3c(cc2Br)OCCO3)cc1OC	0	Train
TNKS1_ML_2433	CCOc1ccc(Nc2scc(-c3ccc(S(=O)(=O)N4CCCC4)cc3)[n+]2Cc2ccco2)cc1	0	Train
TNKS1_ML_2434	Cn1cc(/C=C(\C#N)C(=O)Nc2cccc(O)c2)c2ccccc21	0	Train
TNKS1_ML_2435	Cc1cc(C)c(-c2cnc(N)cn2)cc1C	0	Train
TNKS1_ML_2436	CN1CCN(CCNC(=O)CCc2nnc3ccc(NCc4ccccc4Cl)nn23)CC1	0	Train
TNKS1_ML_2437	O=C(CSc1nnnn1-c1ccc(O)cc1)N1CCN(S(=O)(=O)/C=C\c2ccccc2)CC1	0	Train
TNKS1_ML_2438	CC(C)(C)CN(Cc1nc2c(c(=O)[nH]1)COCC2)C(=O)CN1CCC	1	Train

	(C(=O)c2ccc(F)cc2)CC1		
TNKS1_ML_2439	O=c1[nH]c(N2CCC3(CC2)CN(CCO)c2cccc(Cl)c23)nc2c1CCCC2	1	Train
TNKS1_ML_2440	N#Cc1ccc(-n2nc(C3CC3)c(Br)c2O)cc1	0	Train
TNKS1_ML_2441	C[C@@H](c1ccccc1)N(Cc1nc2c(c(=O)[nH]1)COCC2)S(=O)(=O)Cc1ccccc1	1	Train
TNKS1_ML_2442	CC(C)(CO)C1Cc2[nH]c(=O)c3ccccc3c2CO1	1	Train
TNKS1_ML_2443	O=C(Nc1ccc2c(c1)COC2)N[C@H]1CCOC2(CCCC2)C1	0	Train
TNKS1_ML_2444	Cc1cc(C)c(S(=O)(=O)Nc2cc(C(=O)[O-])cnc2N2CCC[C@@H](C[NH+]3CCCC3)C2)cc1C	0	Train
TNKS1_ML_2445	CC[C@@H]1CCCCN1c1nc2c(s1)[C@H](O)CC(C)(C)C2	0	Train
TNKS1_ML_2446	CC[C@H](C)CN(Cc1nc2c(c(=O)[nH]1)COCC2)C(=O)CN1CCC(C(=O)c2ccc(F)cc2)CC1	1	Train
TNKS1_ML_2447	Cc1ccc(N2C(=O)[C@@H]3[C@H](CC4=c5ccccc5=[NH+]C4)N[C@@]4(C(=O)Nc5c(C)cccc54)[C@@H]3C2=O)cc1	0	Train
TNKS1_ML_2448	CC[C@H]1C(=O)N[C@@H](C)C(=O)N1[C@@H](C)c1c(C)noc1C	0	Train
TNKS1_ML_2449	Cc1ncc2c(=O)[nH]c(-c3ccc(C(C)(C)O)cc3)cn12	1	Train
TNKS1_ML_2450	CC1(C)Cc2c(c(N3CCOCC3)nc3sc4c(N(Cc5ccccc5)Cc5ccccc5)ncnc4c23)CO1	0	Train
TNKS1_ML_2451	CC(C)N1C(=O)[C@H](C2CCCCC2)NC(=O)C[C@@H]1C	0	Train
TNKS1_ML_2452	COc1ccc(NC(=O)c2nnc([C@H]3CCCN(S(=O)(=O)c4ccc(Cl)s4)C3)s2)cc1	0	Train
TNKS1_ML_2453	CC(=O)N1c2ccc(S(=O)(=O)N3CCC[C@@H]3C(=O)Nc3cccc[nH+]3)cc2C[C@H]1C	0	Train
TNKS1_ML_2454	CCOC(=O)[C@H]1[NH+]=c2cc(OC)c(OC)cc2=C1NC(=O)CCN1CCN(c2ccccc2OC)CC1	0	Train
TNKS1_ML_2455	CC1(C)CCCC[C@H]1Nc1[nH+]cc(C(=O)[O-])cc1Cl	0	Train
TNKS1_ML_2456	Cc1ccc(-n2nc(C)c(CC(=O)[O-])c2C)nc1	0	Train
TNKS1_ML_2457	Clc1cccc2nc([C@H]3CCCCS3)[nH]c12	0	Train
TNKS1_ML_2458	CC(=O)c1ccc(NS(=O)(=O)c2cccc(S(=O)(=O)[C@@H]3CCS(=O)(=O)C3)c2)cc1	0	Train
TNKS1_ML_2459	COc1ccc([C@H]2c3c(ncn(CCCN4CCOCC4)c3=[NH2+])Oc3ccc4ccccc4c32)cc1OC	0	Train
TNKS1_ML_2460	Cc1nn(Cc2ccccc2Cl)c(C)c1C[NH+](C)Cn1nc2n(c1=S)CCCCC2	0	Train
TNKS1_ML_2461	C[C@@H]1CCC[NH+](CCCNC(=O)CN2C(=O)CCc3cc(S(=O)(=O)N4CCCCC4)ccc32)C1	0	Train
TNKS1_ML_2462	O=C1c2n[nH]c(-c3ccccc3O)c2[C@@H](c2cccc(Cl)c2)N1C[C@H]1CCCO1	0	Train
TNKS1_ML_2463	COc1ncc(-c2c(C#N)c(N)nc3c2SCCSC3)cn1	0	Train
TNKS1_ML_2464	O=C(Nc1ccccc1C(=O)N/N=C/c1c(Cl)cccc1Cl)c1ccco1	0	Train
TNKS1_ML_2465	COC(=O)Nc1sc2c(c1C(=O)[O-])CCCC2	0	Train
TNKS1_ML_2466	CC(C)(C)c1ccc(-c2nc3ccccc3c(=O)[nH]2)cc1	1	Train
TNKS1_ML_2467	O[C@@H](c1ccoc1)c1cnccn1	0	Train
TNKS1_ML_2468	CCc1c(N)nnn1[C@@H]1CCO[C@@]2(CCSC2)C1	0	Train
TNKS1_ML_2469	O=C(COc1ccc(NC(=O)[C@H]2[C@@H]3C[C@@H]4OC(=O)[C@@H]2[C@@H]4C3)cc1)c1ccccc1	0	Train
TNKS1_ML_2470	Cc1ncc(CNC(=O)Nc2cc(S(=O)(=O)N(C)C(C)C)ccc2C)s1	0	Train
TNKS1_ML_2471	C[C@H](C[NH3+])c1noc(-c2ccc(Br)cc2Cl)n1	0	Train
TNKS1_ML_2472	C[C@@H](C1CCCC1)N1CCCN2CCCC[C@@H]2C1	0	Train
TNKS1_ML_2473	Nc1cncc(C2CC2)n1	0	Train
TNKS1_ML_2474	O=C(c1ccc([N-]S(=O)(=O)c2ccc3c(c2)OCCCO3)cc1)N1CCN(c2cccc[nH+]2)CC1	0	Train
TNKS1_ML_2475	NC(=O)c1nn(-c2ccccc2)nc1NC(=O)Cn1nnc(-c2ccccc2F)n1	0	Train
TNKS1_ML_2476	O=S(=O)(c1ccc(Sc2nc(-	0	Train

	c3ccncc3)n[nH]2)nc1)N1CCC(Cc2ccccc2)CC1		
TNKS1_ML_2477	Cc1cc(=O)[nH]c2cc(-c3ccccc3)ccc12	1	Train
TNKS1_ML_2478	Cc1nn([C@@H]2CCCCC[C@@H]2O)c(C)c1O	0	Train
TNKS1_ML_2479	C[C@H]1CCc2sc(/N=C/c3cccc(O)c3[O-])c(C#N)c2C1	0	Train
TNKS1_ML_2480	CC(C)[C@@H](NC(=O)c1ccc(Cl)cc1)C(=O)Nc1ccc(N2CC[NH+](C)CC2)nc1	0	Train
TNKS1_ML_2481	Cc1c[nH+]c(N(C)[C@@H](C)C/C(N)=N/O)c(Br)c1	0	Train
TNKS1_ML_2482	Cc1sc(-c2noc(C3CCC3)n2)cc1S(=O)(=O)N1CCC(C(=O)NC2CCCCCC2)CC1	0	Train
TNKS1_ML_2483	Cc1cccc2c(=O)[nH]c(-c3ccc(F)cc3)nc12	1	Train
TNKS1_ML_2484	Cc1ccc2nc(CCn3cc[nH+]c3-c3ccoc3)[nH]c2c1C	0	Train
TNKS1_ML_2485	Cc1ccc2nc(Sc3ccc(C(=O)[O-])o3)[nH]c2c1	0	Train
TNKS1_ML_2486	Cc1ccccc1S(=O)(=O)[N-][C@@H]1OC(=O)C(Oc2ccccc2)=C1Br	0	Train
TNKS1_ML_2487	Cc1cc(C)c([C@@H](Cc2c(C)nn(C)c2Cl)[NH2+]N)c(C)c1	0	Train
TNKS1_ML_2488	COc1ccc(-c2nc3c(s2)C[NH+]([C@@H](C(=O)[O-])c2c[nH]c4cc(NC(C)=O)ccc24)CC3)cc1	0	Train
TNKS1_ML_2489	COC(=O)[C@@H]1CCCN1C(=O)c1c(OC)cc(=O)n2c1CCN(Cc1c(C)[nH]c3ccccc13)CC2	0	Train
TNKS1_ML_2490	COc1cccc([C@H]2c3[nH]c4ccccc4c3C[C@H]3C(=O)N(CCc4ccc(OC)c(OC)c4)CC(=O)N32)c1	0	Train
TNKS1_ML_2491	CC1(C)Oc2ccc(NC(=O)c3ccc(-n4ccnn4)cc3)cc2O1	0	Train
TNKS1_ML_2492	Cc1cc(C(=O)Nc2ccc(Oc3ccc(Cl)cc3)nc2)n(C)n1	0	Train
TNKS1_ML_2493	COc1cccc(NC(=O)c2nc(-c3cccs3)cc3c2nc(C)n3[C@@H]2CCS(=O)(=O)C2)c1	0	Train
TNKS1_ML_2494	Cc1cc(N)c(S(=O)(=O)NC[C@@H]2CCCC[NH+]2C)cc1C	0	Train
TNKS1_ML_2495	C[C@]1(C(=O)Nc2nc(-c3ccc(Cl)cc3)cs2)Oc2ccccc2NC1=O	0	Train
TNKS1_ML_2496	CC(=O)Nc1ccc(S(=O)(=O)NCC(=O)N2CCCN(Cc3ccc(C#N)cc3)CC2)cc1	0	Train
TNKS1_ML_2497	C[C@H](NC(=O)CN1CN(c2ccccc2)C2(CCN(C(=O)N(C)C)CC2)C1=O)c1ccccc1	0	Train
TNKS1_ML_2498	CCS(=O)(=O)c1ccc(-c2ccc(NS(=O)(=O)c3cc(Br)ccc3OC)cc2)nn1	0	Train
TNKS1_ML_2499	COc1ccc(S(=O)(=O)N2CCOCC2)cc1NC(=O)CSc1ccc(C(F)(F)F)cn1	0	Train
TNKS1_ML_2500	CCCCn1nnnc1CSc1nc2sc3c(c2c(=O)n1-c1cccc(OC)c1)CCC3	0	Train
TNKS1_ML_2501	Cc1ccc(-n2c(=O)[nH]c([O-])c([C@@H]3[C@H]4[NH+]=c5ccccc5=C4CCN3C)c2=O)cc1	0	Train
TNKS1_ML_2502	C[C@@H]1C[C@H](C(F)(F)F)n2nc([C@@H]3CCCN3C(=O)c3cc[nH]n3)cc2N1	0	Train
TNKS1_ML_2503	CCN(Cc1nc2c(c(=O)[nH]1)COCC2)C(=O)CCc1ccccc1	1	Train
TNKS1_ML_2504	CC12C[C@@H]3C[C@H](C1)CC(C(/C=N/O)=N/O)(C3)C2	0	Train
TNKS1_ML_2505	COC[C@H]1CCCN1C(=O)CC[C@H]1CNC(=O)[C@@H]2[C@@H](CCN2Cc2cccc(Cl)c2)[NH+]1C	0	Train
TNKS1_ML_2506	COC(=O)[C@@H]1CS[C@H]2CCCCCCCCCC[C@@H]2N1	0	Train
TNKS1_ML_2507	CCNc1ccc(Cl)c(CSc2nccc(C)n2)n1	0	Train
TNKS1_ML_2508	CS(=O)(=O)c1ccc(-c2nnc(/C=C/c3nnc(-c4ccc(C#N)cc4)o3)n2-c2ccccc2Cl)nc1	1	Train
TNKS1_ML_2509	Cc1ccc([C@@H](C)NC(=O)c2cc(C)nc3onc(C)c23)c(C)c1	0	Train
TNKS1_ML_2510	C[C@@H](C(=O)Nc1ccc([N+](=O)[O-])cc1Cl)N1CCN(S(=O)(=O)c2ccc3c(c2)OCCO3)CC1	0	Train
TNKS1_ML_2511	CC[C@@H]1C[C@@H](NCc2nc3ccccc3[nH]2)CCO1	0	Train
TNKS1_ML_2512	Nc1c(Br)cnn1Cc1cc(Cl)c2c(c1)OCO2	0	Train
TNKS1_ML_2513	Cc1noc2nc(C3CC3)cc(C(=O)Nc3ccc4c(c3)OCCO4)c12	0	Train

TNKS1_ML_2514	CC(C)[C@H](C#N)c1nc2ccccc2s1	0	Train
TNKS1_ML_2515	O=c1[nH]c(-c2ccc(Br)cc2)nc2c(O)cccc12	1	Train
TNKS1_ML_2516	CCc1nnc(NC(=O)c2ccc3c(c2)S(=O)(=O)c2ccccc2C3=O)s1	0	Train
TNKS1_ML_2517	O=C(NCc1ccc[nH+]c1)c1nnn(-c2cc(Cl)cc(Cl)c2)c1CSc1ncccn1	0	Train
TNKS1_ML_2518	N#C/C(=C\Nc1cccc([N+](=O)[O-])c1)c1nc(-c2cc3c(ccc4ccccc43)oc2=O)cs1	0	Train
TNKS1_ML_2519	Cc1ccc(C(=O)Nc2cc(C#N)ccc2C)s1	0	Train
TNKS1_ML_2520	COc1cc([N-]S(=O)(=O)c2cc3[nH]c4c(c3cc2C)C(=O)CC(C)(C)C4)cc(OC)c1OC	0	Train
TNKS1_ML_2521	O=C1Nc2cc(Cl)ccc2C12CCN(c1nc3c(c(=O)[nH]1)CCCC3)CC2	1	Train
TNKS1_ML_2522	COc1ccc(C(=O)C2CCN([C@@H]3CCN(Cc4nc5ccccc(=O)c5[nH]4)C3=O)CC2)cc1	1	Train
TNKS1_ML_2523	O=S(=O)(c1cccc(/C(O)=N/c2cccc3c[nH+]ccc23)c1)N1CCCCCC1	0	Train
TNKS1_ML_2524	CCn1c(CNC(=O)c2cc(-c3cc(C)oc3C)nc3onc(C)c23)nc2ccccc21	0	Train
TNKS1_ML_2525	CCC(O)(CC)CNC(=O)NC1CC[NH+](C[C@@H]2CCOC2)CC1	0	Train
TNKS1_ML_2526	Cc1cc(-c2nc(C3CCCC3)n[nH]2)sc1Br	0	Train
TNKS1_ML_2527	Cc1ccc(S(=O)(=O)N2CC(c3nc4ccccc4[nH]3)C2)cc1C	0	Train
TNKS1_ML_2528	C[C@H]1CCC[C@@H](NS(=O)(=O)c2cccc3nsnc23)[C@@H]1C	0	Train
TNKS1_ML_2529	CCN(C(=O)N1CCC(C(=O)c2ccc3cnn(C)c3c2)CC1)c1ccc(C(=O)O)cc1	1	Train
TNKS1_ML_2530	O=C(NCc1ccccn1)c1nnsc1NC(=O)N1C[C@@H]2C[C@H](C1)c1cccc(=O)n1C2	0	Train
TNKS1_ML_2531	O=C(Nc1ccc(-n2cc[nH+]c2)nc1)[C@@H]1CCCN(C(=O)c2ccc(Cl)c(Cl)c2)C1	0	Train
TNKS1_ML_2532	O=C(N[C@H]1CS(=O)(=O)C[C@H]1[NH+]1CCCC1)C1CCCCCCC1	0	Train
TNKS1_ML_2533	C[NH+]1CCN(S(=O)(=O)c2cccc(C(=O)Nc3ccccc3C(=O)N3CCCCC3)c2)CC1	0	Train
TNKS1_ML_2534	Cn1cccc1C(=O)NC(=O)CSc1nnc(-c2ccncc2)n1Cc1ccccc1	0	Train
TNKS1_ML_2535	CCOC(=O)c1c(NC(=O)CSC2=N/C(=C/c3cn(C)nc3C)C(=O)N2CC)sc2c1CC[C@H](C)C2	0	Train
TNKS1_ML_2536	Cc1cc2c(cc1S(=O)(=O)N1CCN(C(=O)c3ccc(Cl)cc3)CC1)O[C@@H](C)C(=O)N2	0	Train
TNKS1_ML_2537	O=C(COc1ncnc2ccc(Br)cc12)Nc1ccc(S(=O)(=O)N2CCCC2)cc1	0	Train
TNKS1_ML_2538	O=C(CN1CCC(C(=O)c2ccc(OCC3CCC3)cc2)CC1)NCc1nc2c(c(=O)[nH]1)COCC2	1	Train
TNKS1_ML_2539	COc1ccc(C2(CNC(=O)c3ccc(NC(=O)c4ccco4)cc3)CCOCC2)cc1	1	Train
TNKS1_ML_2540	Cn1cc(N)cc1C(=O)N1CCC[C@@H]1C1CCCCC1	0	Train
TNKS1_ML_2541	O=C(c1ccccc1)C1CCN(CC(=O)N(Cc2nc3c(c(=O)[nH]2)COCC3)CC(F)F)CC1	1	Train
TNKS1_ML_2542	[H]/N=C1\[C@@H](C#N)C2=CCN(C(=O)OCC)C[C@@H]2[C@@H](c2ccc(CC)cc2)C1(C#N)C#N	0	Train
TNKS1_ML_2543	O=C1C[C@@H](c2nnc(/N=C(\[O-])C3c4ccccc4Oc4ccccc43)s2)CN1c1ccc(F)cc1	0	Train
TNKS1_ML_2544	CNc1nc(C2CCN(C(C)=O)CC2)[nH+]c2c1CCN(C(=O)[C@H]1CCC(C)=C(C)C1)C2	0	Train
TNKS1_ML_2545	N#Cc1ccc2c(c1)[nH]c(=O)n2C12CC(c3nnc(-c4ccncn4)n3-c3ccccc3Cl)(C1)C2	1	Train

TNKS1_ML_2546	CC(C)Cn1nc(C(=O)NCc2ccc[nH+]c2)c2c1CCN(C(=O)C1=C[C@H]3N=CC=C3C=C1)C2	0	Train
TNKS1_ML_2547	Cc1oncc1NC1CCN(c2ccc(N(C)C)cc2)CC1	0	Train
TNKS1_ML_2548	OC(=C\C1=NCCN1)/C=C/c1cccc(Cl)c1	0	Train
TNKS1_ML_2549	[H]/N=C1C(=C\c2cc(Cl)c(OCCOc3ccc(C)cc3)c(OC)c2)/C(=O)N=C2SC(CC)=NN2/1	0	Train
TNKS1_ML_2550	COc1ccc(-c2nnc(C3CCN(CC(=O)NCc4nc5c(c(=O)[nH]4)COCC5)CC3)o2)cc1	1	Train
TNKS1_ML_2551	N#CC1(C2(C#N)CCCCC2)CCCCC1	0	Train
TNKS1_ML_2552	C[C@@H]1C[NH+]([C@@H]2CCC(C)(C)c3ccccc32)C[C@@H]1C(=O)[O-]	0	Train
TNKS1_ML_2553	C[C@H](Sc1nc(-c2ccccc2)cc(C(F)(F)F)n1)C(=O)NN1CC[NH+](C)CC1	0	Train
TNKS1_ML_2554	CN(C)c1ccc(-c2c(C#N)c(N)nc3c2CCC3)cc1	0	Train
TNKS1_ML_2555	Cc1sc2ncn(CC3(O)CCN(C(=O)[C@@H](C)CN4CCOCC4)CC3)c(=O)c2c1C	0	Train
TNKS1_ML_2556	[H]/N=C1C(=C/c2ccc(OCCOc3ccccc3)c(OCC)c2)\C(=O)N=C2SN=C(S(C)(=O)=O)N2\1	0	Train
TNKS1_ML_2557	CCC[C@H](Nc1nc2ccc(F)cn2n1)c1cccs1	0	Train
TNKS1_ML_2558	C[C@H]1CCCCCN1c1ncc(Br)cc1CO	0	Train
TNKS1_ML_2559	COc1ccc(N2CCN(c3nc4c(c(=O)[nH]3)CSCC4)CC2)cc1	1	Train
TNKS1_ML_2560	Cc1ccc2c(=O)[nH]c(-c3ccc(C(C)(C)O)cc3)nn12	1	Train
TNKS1_ML_2561	O=C(COC(=O)c1cc(-c2ccccc2)nn1-c1ccccc1)N[C@@H]1CCS(=O)(=O)C1	0	Train
TNKS1_ML_2562	CC(=O)Nc1cccc(NC(=O)C2CCN(c3nc4ccc(F)cc4s3)CC2)c1	0	Train
TNKS1_ML_2563	COc1ccc(CN(C)C(=O)Nc2ccc(C)c3[nH+]cccc23)cc1	0	Train
TNKS1_ML_2564	C[C@H]1C[C@@H](C)CN(S(=O)(=O)Nc2ccccc2C(=O)[O-])C1	0	Train
TNKS1_ML_2565	CN1C(=O)N(C[C@@H](C(=O)NO)[C@H](CC2CCCC2)C(=O)N2CCCCC2)C(=O)C1(C)C	0	Train
TNKS1_ML_2566	COc1cc(C(c2c(C)n(C)n(-c3ccccc3)c2=O)c2c(C)n(C)n(-c3ccccc3)c2=O)ccc1O	0	Train
TNKS1_ML_2567	O=C(CCc1cccc(O)c1)N(Cc1nc2c(c(=O)[nH]1)COCC2)Cc1cccs1	1	Train
TNKS1_ML_2568	CCOc1ccc(N2C(=O)NC(=O)[C@@](Cc3ccc(OC)cc3)(CN3C[C@@H]4C[C@H](C3)Cn3c4cccc3=O)C2=O)cc1	0	Train
TNKS1_ML_2569	COc1cc(/C=C2/C(=O)NC(=O)N(c3ccccc3)C2=O)cc(Br)c1OCc1cccc2ccccc12	0	Train
TNKS1_ML_2570	O=c1[nH]nc2c3c(cccc13)NC1=C2CCN(Cc2ccccc2)C1	1	Train
TNKS1_ML_2571	CC1(C)CC(=O)C2=C(C1)OC(N)=C(C#N)[C@@H]2c1ccc(Br)s1	0	Train
TNKS1_ML_2572	CCc1[nH]c(CNc2cc(C(=O)N(C)[C@H](C)c3ccccn3)cc(S(=O)(=O)N(C)C)c2)c(C)[nH+]1	0	Train
TNKS1_ML_2573	C[C@@H](C(=O)NCCCn1cc[nH+]c1)N1CC[C@@]2(CCC1=O)CC(=O)c1ccccc1O2	0	Train
TNKS1_ML_2574	O=C(CC1CCOCC1)Nc1nnc2n1CCS2	1	Train
TNKS1_ML_2575	CC1(C)Cc2c(c(N3CCOCC3)nc3sc4c(SCCO)ncnc4c23)CO1	0	Train
TNKS1_ML_2576	Cc1ccc(-c2nc3c(C)cccc3c(=O)[nH]2)cc1	1	Train
TNKS1_ML_2577	C[C@@H]1CC(C)(C)O[C@H]([C@H]2CC=CCC2)O1	0	Train
TNKS1_ML_2578	COCCN(CC(=O)Nc1ccc(N2CCN(C(=O)c3ccccc3F)CC2)cc1)C(C)=O	0	Train
TNKS1_ML_2579	C[NH+](CC(=O)NCCNc1ccc(C(F)(F)F)cn1)[C@H]1CCS(=O)(=O)C1	0	Train
TNKS1_ML_2580	O=C([O-])c1cccc(S(=O)(=O)Oc2ccc3c(=O)c(Oc4ccc(F)cc4)coc3c2)c1	0	Train
TNKS1_ML_2581	N#CCCn1cc(-c2cn3cccc(O)c3n2)c2ccccc21	0	Train

TNKS1_ML_2582	CC(C)(O)c1ccc(-c2nc3ccc(F)c(F)c3c(=O)[nH]2)cc1	1	Train
TNKS1_ML_2583	CN(C(=O)c1n[nH]c2ccccc12)c1ccc(C#N)cc1	0	Train
TNKS1_ML_2584	O=C(CCC1CCCCC1)Nc1nnc2n1CCS2	1	Train
TNKS1_ML_2585	COc1ccc(C)cc1NC(=O)[C@H]1c2cc(OC)c(OC)cc2C(=O)N(C)[C@@H]1c1ccc(OC)c(OC)c1	0	Train
TNKS1_ML_2586	C[C@H]1CCCCN1c1n[nH]c(=S)n1C1CCCC1	0	Train
TNKS1_ML_2587	Cc1c[nH+]c(N2CCN(C(=O)c3ccc(S(=O)(=O)Nc4ccncc4)cc3)CC2)s1	0	Train
TNKS1_ML_2588	Cc1cccc(Cn2c(=O)n(C[C@H]3CC[C@H](C(=O)Nc4ccncc4)CC3)c(=O)c3c(C)cccc32)c1C#N	1	Train
TNKS1_ML_2589	Cc1ccc([C@@]2(C)NC(=O)N(CC(=O)OCc3cc(=O)oc4cc(Br)ccc34)C2=O)cc1	0	Train
TNKS1_ML_2590	COc1ccc(OCCCCn2c([C@@H](C)NC(=O)c3cc(OC)c(OC)c(OC)c3)[nH+]c3ccccc32)cc1	0	Train
TNKS1_ML_2591	O=C(N[C@H]1C[C@H](c2nnc(-c3ccc(OC(F)F)cn3)n2-c2ccccc2F)C1)c1ccnc2cc(F)cnc12	1	Train
TNKS1_ML_2592	Cc1ccc([C@@H](N)c2ccc3c(c2)oc(=O)n3C)c(C)c1	0	Train
TNKS1_ML_2593	Cc1c(C(=O)NCCC[NH+]2CCCC[C@@H]2C)cnc2c(-c3ccc4c(c3)OCO4)cnn12	0	Train
TNKS1_ML_2594	Cc1cccn2c(C)c(C(=O)C3=C(O)C(=O)N(CCC[NH+](C)C)[C@@H]3c3cccc(Br)c3)nc12	0	Train
TNKS1_ML_2595	COc1ccc(N[C@@H](C)C(=O)N2CCc3ccccc32)cn1	0	Train
TNKS1_ML_2596	CCOc1ccc(-c2nnc([C@H]3C[C@H](NC(=O)c4ccnc5cc(F)cnc45)C3)n2-c2ccc(C)s2)nc1	1	Train
TNKS1_ML_2597	O=c1occ(C[NH+]2CCSCC2)c([O-])c1C[NH+]1CCN(CCOc2ccc(F)cc2)CC1	0	Train
TNKS1_ML_2598	O=[N+]([O-])c1ccc(-c2nc(O)c3cccc(O)c3n2)cc1	1	Train
TNKS1_ML_2599	C[C@H](c1ccc(F)cc1)N(C)C(=O)C[NH+]1CCC(NC(=O)Nc2ccc(F)cc2)CC1	0	Train
TNKS1_ML_2600	COC(=O)[C@@H](C)N1C(=O)S/C(=C/c2ccc(OCC(=O)Nc3ccc(C)cc3)c(Cl)c2)C1=O	0	Train
TNKS1_ML_2601	C[C@H]1CCC[C@H](C)C12NC(=O)N(CN1CCCCCCC1)C2=O	0	Train
TNKS1_ML_2602	CO[C@H](C)C[C@](C)(N)C#N	0	Train
TNKS1_ML_2603	Cc1[nH]c2ccccc2c1C(=O)C(=O)Nc1ccc2ccccc2n1	0	Train
TNKS1_ML_2604	CNc1snc(C)c1-c1nc(C2CCCCCC2)no1	0	Train
TNKS1_ML_2605	Cc1cc(C(=O)COC(=O)c2ccccc2)c(C)n1-c1ccc(S(N)(=O)=O)cc1	0	Train
TNKS1_ML_2606	CC1(C)OC(=O)N(c2ccc(C(=O)Nc3ccnc4cccnc34)cc2)[C@H]1c1ccccc1	1	Train
TNKS1_ML_2607	CSc1ncc(C[NH+]2CC[C@@H](N3CCN(c4ccccn4)CC3)[C@H](O)C2)cn1	0	Train
TNKS1_ML_2608	Cc1c(C(=O)NCc2ccc(Cl)nc2)sc2nc3n(c(=O)c12)CCCCC3	0	Train
TNKS1_ML_2609	C[C@H](Nc1nn2nnnc2c2ccccc12)c1ccccc1	0	Train
TNKS1_ML_2610	COCCN1C(=O)/C(=C\c2c(NC[C@@H](O)c3ccccc3)nc3c(C)cccn3c2=O)SC1=S	0	Train
TNKS1_ML_2611	CO/C([O-])=C1/C(C)=NC(O)=[NH+][C@H]1c1cn(-c2ccccc2)nc1-c1cccs1	0	Train
TNKS1_ML_2612	Cc1sc2ncn(CC(=O)NC[C@@H]3COc4ccccc4O3)c(=O)c2c1S(=O)(=O)N1C[C@@H](C)C[C@H](C)C1	0	Train
TNKS1_ML_2613	COc1cc(-c2cc3n(C)c(C)c(CCC(=O)Nc4ccc(C)cc4)c(=O)n3n2)cc(OC)c1OC	0	Train
TNKS1_ML_2614	CCn1ncnc1CN1CCc2nc[nH]c2[C@@H]1c1cn[nH]c1-c1ccccc1	0	Train
TNKS1_ML_2615	O=C(N/N=C/c1cc(/N=N/c2cccc(Br)c2)ccc1O)c1ccncc1	0	Train
TNKS1_ML_2616	Oc1c(Br)cc(Br)cc1/C=N/n1c(-c2cccs2)n[nH]c1=S	0	Train

TNKS1_ML_2617	O=C(CCc1ccccc1)N(CCO)Cc1nc2c(c(=O)[nH]1)COCC2	1	Train
TNKS1_ML_2618	Nc1noc([C@@H]2C[C@H]3CC[C@@H]2C3)c1-c1cc(F)cc(F)c1	0	Train
TNKS1_ML_2619	Cc1sc2ncnc(N[C@@H]3CCCCC[C@H]3C)c2c1C	0	Train
TNKS1_ML_2620	Cc1ccc(C2NC(=O)c3ccccc3N2)cc1	1	Train
TNKS1_ML_2621	COc1ccc(-c2ccc(C#N)c(SCC(=O)Nc3ccc4c(c3)OCCO4)n2)cc1OC	0	Train
TNKS1_ML_2622	Cc1c/c(=N\S(=O)(=O)CC#N)c2ccccc2[nH]1	0	Train
TNKS1_ML_2623	CC(C)Cc1nc(Cl)c2ccsc2n1	0	Train
TNKS1_ML_2624	O=C(c1ccc(F)cc1)C1CCN(CC(=O)N(Cc2nc3c(c(=O)[nH]2)COCC3)CC2CC2)CC1	1	Train
TNKS1_ML_2625	Cc1cc(C)c2[nH+]c3c(c(C(=O)[O-])c2c1)[C@@H](C)CC[C@@H]3C	0	Train
TNKS1_ML_2626	c1ccc(C2=NC3=C(CCSC3)CN2)cc1	1	Train
TNKS1_ML_2627	CC(C)(C)OC(=O)N1CCC2=C(C1)Nc1cccc3c(=O)[nH]nc2c13	1	Train
TNKS1_ML_2628	O=C([O-])[C@H]1C[C@@H]2CCCC[C@H]2N1C(=O)NC1CCCC1	0	Train
TNKS1_ML_2629	CC/C1=C\C(=O)O[C@H]2C[C@@H](CC[C@@H](CC)/C=C/C=C\CC1)O[C@@](O)([C@@H]1CSC(=O)N1)C2	0	Train
TNKS1_ML_2630	Cc1ccc(Sc2cc(Cl)nc(N)n2)cc1	0	Train
TNKS1_ML_2631	CC[C@H](C)NC(=O)CN1C(=O)CS[C@@H](c2ccc3c(c2)OCO3)c2c(C(C)(C)C)nn(-c3ccc(OC)cc3)c21	0	Train
TNKS1_ML_2632	CN1C(=O)c2ccccc2N2C(=O)CC[C@@]12C(=O)OCC(=O)c1ccc(NC(=O)c2ccccc2)cc1	0	Train
TNKS1_ML_2633	Cc1ccc(NC(=O)[C@H](C)Sc2ncnc3c2sc2nc(N4CCOCC4)c4c(c23)CC(C)(C)OC4)cc1	0	Train
TNKS1_ML_2634	O=c1[nH]c(-c2ccc(C(F)(F)F)cc2)nc2c1COCC2	1	Train
TNKS1_ML_2635	O=C1/C(=C/c2cccc([N+](=O)[O-])c2)Oc2c1ccc([O-])c2C[NH+]1CCN(c2ccccc2F)CC1	0	Train
TNKS1_ML_2636	CCOC(=O)c1sc(NC(=O)[C@@H]2CCCCN2S(C)(=O)=O)c(C#N)c1C	0	Train
TNKS1_ML_2637	O=S(=O)(/N=c1\[nH]cc(Br)s1)c1cncc(Br)c1	0	Train
TNKS1_ML_2638	O=C1N=C2C=C/C(=[NH+]\C(=O)C(c3ccccc3)c3ccccc3)C=C2N1	0	Train
TNKS1_ML_2639	O=C(Nc1cccc(C#Cc2cccc[nH+]2)c1)[C@H]1COc2ccccc2O1	0	Train
TNKS1_ML_2640	Cc1nn(-c2ccccc2)c(C)c1C(=O)Nc1cccc(S(=O)(=O)/N=C2/CCCN2)c1	0	Train
TNKS1_ML_2641	Cc1cccc(/C=N/NC(=O)c2ccccc2NS(=O)(=O)c2cccs2)c1	0	Train
TNKS1_ML_2642	ClC1=C[C@H](C2CC2)N=N1	0	Train
TNKS1_ML_2643	C[C@@]1(c2ccc(F)cc2)NC(=O)N(CC(=O)N2CCN(S(=O)(=O)Cc3ccccc3)CC2)C1=O	0	Train
TNKS1_ML_2644	C[C@@H](c1ccccc1)n1c(CSc2nnc(-c3ccn(C)n3)n2C[C@H]2CCCO2)n[nH]c1=S	0	Train
TNKS1_ML_2645	CC(C)COc1ccc(/C(O)=C2/C(=O)C(=O)N(Cc3ccc[nH+]c3)[C@H]2c2cccs2)cc1	0	Train
TNKS1_ML_2646	CC(=O)N1CCc2c(sc(NC(=O)c3ccc([N+](=O)[O-])s3)c2-c2nc3ccccc3s2)C1	0	Train
TNKS1_ML_2647	CC(C)NC(=O)C(=O)N1CCN(c2nc(-c3ccco3)c3c(c2C#N)CC(C)(C)OC3)CC1	0	Train
TNKS1_ML_2648	Cc1cc(Br)c(O)c(/N=N\c2ccccc2)c1	0	Train
TNKS1_ML_2649	C/C=C/C1OCC2(CO1)[C@@H](C)C=C(C)C[C@@H]2C	0	Train
TNKS1_ML_2650	CC#CCOc1ccc(C(=O)C2CCN(CC(=O)N(Cc3nc4c(c(=O)[nH]3)COCC4)CC3CC3)CC2)cc1	1	Train
TNKS1_ML_2651	CN1C(=O)C(=Cc2ccc(O)c(Cl)c2)C(=O)N(C)C1=O	0	Train
TNKS1_ML_2652	C[C@@H]1CCC[C@@](C#N)([C@]2(O)CCCOCC2)C1	0	Train

TNKS1_ML_2653	C[C@@H](Sc1nnnn1Cc1cccs1)C(=O)Nc1ccc(Br)cc1	0	Train
TNKS1_ML_2654	COP(=O)(OC)[C@H](NC(=O)c1ccccc1)c1nnc(-c2ccccc2)o1	0	Train
TNKS1_ML_2655	C[C@H](CCO)CNS(=O)(=O)c1ccc(C[NH2+]C(C)(C)C)o1	0	Train
TNKS1_ML_2656	CCCCC12C[NH+]3CC(C)(C[NH+](C1)C31C(=O)Nc3ccc(Br)cc31)C2=O	0	Train
TNKS1_ML_2657	Cc1sc2ncnc(N[C@H]3CC[C@H](C)[C@H](C)C3)c2c1C	0	Train
TNKS1_ML_2658	CC[C@H](C)[C@H](NC(=O)[C@H](C(C)C)N1C(=O)N2CCC3=c4ccccc4=[NH+][C@@H]3[C@@]2(C)C1=O)C(=O)[O-]	0	Train
TNKS1_ML_2659	Cc1ccc(NC(=O)[C@H]2CC[C@H](Cn3c(=O)c4c(C)cccc4n(Cc4cccc(C(F)(F)F)c4C#N)c3=O)CC2)cn1	1	Train
TNKS1_ML_2660	Cc1ccc2[nH]nc(C(=O)N[C@@H]3CCC[C@@H](C)C3)c2c1	0	Train
TNKS1_ML_2661	Cc1cccn2cc(-c3ccc(NC(=O)C(=O)N[C@H]4CC(=O)N(C(C)C)C4=O)cc3)[nH+]c12	0	Train
TNKS1_ML_2662	Cc1nnc(-c2cc(C)n(-c3cccc(C(=O)NCCc4cccc(Cl)c4)c3)n2)o1	0	Train
TNKS1_ML_2663	O=CNc1c(Br)cc(Br)cc1C(=O)[O-]	0	Train
TNKS1_ML_2664	O=c1[nH]c(N2CCC3(CC2)CNc2cc(Cl)cc(Cl)c23)nc2c1CCCC2	1	Train
TNKS1_ML_2665	O=c1[nH]nc2c3c(cccc13)N=C(CN1Cc3ccccc3C1)N2	1	Train
TNKS1_ML_2666	O=C(c1cc(-c2cccs2)[nH]n1)N1CCN(S(=O)(=O)c2ccc(Cl)s2)CC1	0	Train
TNKS1_ML_2667	CC[C@H]1CCC[C@@]2(CC1)C[NH2+]C[C@@H]2C(=O)[O-]	0	Train
TNKS1_ML_2668	O[C@H](C1CCCCC1)[C@@H]1CCOC2(CCC2)C1	0	Train
TNKS1_ML_2669	COc1ccc(CN(Cc2cc3cc(OC)c(OC)cc3[nH]c2=O)S(=O)(=O)c2ccc(Cl)cc2)cc1	0	Train
TNKS1_ML_2670	CCCc1cc2c(=O)n(NC(=O)c3nn(C)cc3Br)c(C)nc2s1	0	Train
TNKS1_ML_2671	CN(C(=O)NC1CCC(C(=O)[O-])CC1)[C@H]1CCC[NH+](C)C1	0	Train
TNKS1_ML_2672	Cc1c(NC(=O)[C@H](C)Sc2nnc(C(F)(F)F)n2C)c(=O)n(-c2ccccc2)n1C	0	Train
TNKS1_ML_2673	CCOC(=O)N1CCN(C(=O)c2ccccc2NS(=O)(=O)c2ccc(F)c(C)c2)CC1	0	Train
TNKS1_ML_2674	Nc1c(-c2nc(-c3ccc(Cl)cc3)no2)nnn1-c1ccc(C(F)(F)F)c(Cl)c1	0	Train
TNKS1_ML_2675	Cc1cc([C@H](C(C)C)[C@H](C)O)n2ncnc2n1	0	Train
TNKS1_ML_2676	C[C@H]1CC(Nc2ccc3nccnc3n2)C[C@H](C)C1	0	Train
TNKS1_ML_2677	c1ccc2c(c1)CCN[C@H]2c1nc(-c2ccoc2)no1	0	Train
TNKS1_ML_2678	COc1ccc(NC(=O)c2ccc(NC(=O)N3CCN(C(=O)c4cc(Cc5n[nH]c(=O)c6ccccc56)ccc4F)CC3)cc2)cc1	1	Train
TNKS1_ML_2679	COCCN1C(=O)C2(CCN(c3nc4c(c(=O)[nH]3)CCCN4C)CC2)c2c(F)cc(F)cc21	1	Train
TNKS1_ML_2680	Cc1ccc(CNC(=O)CSc2nc(-c3ccccc3)nc3c2c(=O)n(C)c(=O)n3C)cc1	0	Train
TNKS1_ML_2681	CCOc1ccc(S(=O)(=O)N2CCN(c3cc(=O)n4nc(-c5ccccc5)sc4n3)CC2)cc1	0	Train
TNKS1_ML_2682	CC(C)c1ccc(-n2nc(C(=O)NCc3cccc[nH+]3)c(=O)n(Cc3ccccc3)c2=O)cc1	0	Train
TNKS1_ML_2683	CC(=O)N(c1ccccc1)c1nc(/C=C/C(=O)N[C@H](c2ccccc2F)c2[nH+]ccn2C)cs1	0	Train
TNKS1_ML_2684	CN(C(=O)N[C@H](C(=O)[O-])C(C)(C)C)C1CC1	0	Train
TNKS1_ML_2685	O=C(c1cccc(OC(F)(F)F)c1)C1CCN(CC(=O)N(Cc2nc3c(c(=O)[nH]2)COCC3)CC(F)F)CC1	1	Train
TNKS1_ML_2686	O=C(/C=C\c1ccc(C(=O)NC2CC2)cc1)N[C@@H]1CS(=O)(=O)C[C@@H]1[NH+]1CCCCC1	0	Train
TNKS1_ML_2687	Cn1ncc2c(N)nc(COc3c(Br)cccc3Br)nc21	0	Train

TNKS1_ML_2688	CCn1c(-c2ccc3c(c2)OCCO3)c[nH+]c1SCC(=O)Nc1cc2c(cc1Cl)OCCO2	0	Train
TNKS1_ML_2689	Cc1ccc(F)cc1N[C@H]1CCO[C@]2(CCOC2)C1	0	Train
TNKS1_ML_2690	O=C(Cn1cnc2sc3c(c2c1=O)CCCC3)N/N=C/c1c(F)c(F)c(F)c1F	0	Train
TNKS1_ML_2691	COc1cc(OC)c(NC(=O)N2C[C@@H](C)CC[C@@H]2C)cc1F	0	Train
TNKS1_ML_2692	CCOC(=O)c1nc(Nc2cccc(F)c2)c2c(C)noc2n1	0	Train
TNKS1_ML_2693	O=C(NC1CC1)[C@@H](c1ccccc1)N1CCN(S(=O)(=O)c2ccccc2Cl)CC1	0	Train
TNKS1_ML_2694	CCn1c(CCC(=O)N[C@H](C#N)c2ccc(F)cc2)nc2cc(S(=O)(=O)N(C)C)ccc21	0	Train
TNKS1_ML_2695	CCOc1ccc(-c2nnc([C@H]3C[C@H](NC(=O)c4cccc5ncc(C)nc45)C3)n2-c2ccccc2F)nc1	1	Train
TNKS1_ML_2696	CCN(CC)S(=O)(=O)c1ccc(C)c(NC(=O)CSc2nnc(C(F)(F)F)n2C)c1	0	Train
TNKS1_ML_2697	Cc1nc(CNC(=O)N2C[C@@H](C)CC[C@@H]2C)sc1C(=O)[O-]	0	Train
TNKS1_ML_2698	CC1(C)CCC(Nc2cc(Cl)ncn2)CC1	0	Train
TNKS1_ML_2699	Clc1ccc(-c2c[nH]c3ncc(Br)cc23)cn1	0	Train
TNKS1_ML_2700	Cc1nc(-c2ccc(CNC(=O)[C@@H]3CCCN(S(=O)(=O)c4c(C)noc4C)C3)cc2)cs1	0	Train
TNKS1_ML_2701	COc1ccc(C[C@@]2(CCC(=O)N3CCNC(=O)CC3)CCC(=O)N2)c2ccccc12	0	Train
TNKS1_ML_2702	O=C(c1ccccc1)[C@@H]1[C@@H]2C(=O)N(c3ccc([N+](=O)[O-])cc3)C(=O)[C@@H]2[C@H]2c3ccccc3C=C[NH+]21	0	Train
TNKS1_ML_2703	O=C([O-])[C@@H]1C[NH2+]CC12CCCCCCC2	0	Train
TNKS1_ML_2704	CCOC(=O)N1CCN(Cc2ccc(OC[C@H]3CCCN(C(=O)c4cccc5c(C)c(C)[nH]c45)C3)cc2)CC1	0	Train
TNKS1_ML_2705	CCCc1nc(-c2nc[n-]n2)nc(NC)c1Br	0	Train
TNKS1_ML_2706	C[C@@H]1c2[nH]c3ccccc3c2[C@@H](c2ccccc2)CN1[C@@H]1CC(=O)N(c2cccc([N+](=O)[O-])c2)C1=O	0	Train
TNKS1_ML_2707	C[C@]1(c2ccccc2Cl)NC(=O)N(CC(=O)N(C2CC2)[C@@H]2CCS(=O)(=O)C2)C1=O	0	Train
TNKS1_ML_2708	CC[C@H](C)c1nc(C)c(Cc2ccc([N+](=O)[O-])cc2)c(N2CCN(C(=O)C(C)C)CC2)n1	0	Train
TNKS1_ML_2709	Cc1ncsc1CCCn1ccnc1-c1cc2n(n1)CCNC2	0	Train
TNKS1_ML_2710	CCC(=O)N1CCN(C(=O)[C@@H](NC(=O)c2cccs2)S(=O)(=O)c2ccc(C)cc2)CC1	0	Train
TNKS1_ML_2711	Cc1coc2cc3c(cc12)[C@@H](C)[C@H](CCC(=O)NC1=CC2=CC=N[C@@H]2C=C1)C(=O)O3	0	Train
TNKS1_ML_2712	O[C@H]1CCC2(C[C@@H]1n1ncc3ccccc31)OCCO2	0	Train
TNKS1_ML_2713	Cc1ccc(S(=O)(=O)[C@H](NC(=O)c2ccco2)C(=O)N2CCN(C(=O)c3ccc(F)cc3)CC2)cc1	0	Train
TNKS1_ML_2714	CN1C(=O)N(C)[C@@](O)(C(F)(F)F)[C@H](C(=O)c2ccccc2)[C@@H]1c1ccccc1[N+](=O)[O-]	0	Train
TNKS1_ML_2715	COc1cccc2c1O[C@H](c1cccc(OC)c1OC)N1N=C(c3ccccc3[O-])C[C@H]21	0	Train
TNKS1_ML_2716	O=C(N[C@H]1C[C@H](c2nnc(-c3nccs3)n2-c2ccccc2F)C1)c1ccccn1	1	Train
TNKS1_ML_2717	COc1cc([C@H]2C(C(=O)c3cccs3)=C(O)C(=O)N2CCCn2cc[nH+]c2)cc(OC)c1OC	0	Train
TNKS1_ML_2718	CN(C)CCNC=C1C(=O)N(C)C(=O)N(C)C1=O	0	Train
TNKS1_ML_2719	CC(C)CNC(=O)c1ccc2c(=O)n(C(C)C)c3nn(CC(=O)c4ccc(Cl)cc4)c(=O)n3c2c1	0	Train

TNKS1_ML_2720	O[C@@H](c1ccc(Cl)c(Cl)c1)[C@H]1CCO[C@]2(CCOC2)C1	0	Train
TNKS1_ML_2721	Cc1nc(C)c(C(=O)C2=C(O)C(=O)N(CCCn3cc[nH+]c3)[C@@H]2c2ccccc2Cl)s1	0	Train
TNKS1_ML_2722	Cc1cccc2c1c(=O)n(C[C@H]1CC[C@H](C(=O)Nc3ccncc3)CC1)c(=O)n2Cc1cccc(Cl)c1C#N	1	Train
TNKS1_ML_2723	O=C1Nc2cccc(F)c2C12CCN(c1nc3c(c(=O)[nH]1)CCCC3)CC2	1	Train
TNKS1_ML_2724	O=c1c(O[C@@H]2O[C@@H](CO)[C@H](O)[C@H]2O)c(-c2cc(O)c(O)c(O)c2)oc2cc(O)cc([O-])c12	0	Train
TNKS1_ML_2725	C[C@H]1CN(S(=O)(=O)CCNC(=O)Cc2ccc3c(c2)CCCC3)C[C@H](C)O1	0	Train
TNKS1_ML_2726	CC(C)(C)[C@H]1CC[C@H](O)[C@H]([C@@H]2CCO[C@]3(CCOC3)C2)C1	0	Train
TNKS1_ML_2727	Cc1noc(C)c1CN1CC(O)C1	0	Train
TNKS1_ML_2728	Nc1nc2cc(Br)cnc2n1-c1ccc(Cl)cc1Cl	0	Train
TNKS1_ML_2729	C=CCNC(=O)N1CCc2[nH]c3ccc(Cl)cc3c2C1	0	Train
TNKS1_ML_2730	CC(=O)O[C@H]1C[C@@](O)(C(C)C)[C@H]2[C@@H](OC(=O)c3ccc(O)cc3)C[C@]3(C)O[C@H]3C[C@]12C	0	Train
TNKS1_ML_2731	C[C@@H](c1ccc(OC(F)(F)F)cc1)n1nccc1N	0	Train
TNKS1_ML_2732	Clc1cc(Nc2cc(Cl)ncn2)ncn1	0	Train
TNKS1_ML_2733	C[C@H](C(=O)Nc1ccc(N2CCOCC2)cc1)N1C(=O)N[C@@H](CC2=c3ccccc3=[NH+]C2)C1=O	0	Train
TNKS1_ML_2734	CN(CCCC(=O)Nc1cccc(S(=O)(=O)N2CCCCC2)c1)S(=O)(=O)c1cccs1	0	Train
TNKS1_ML_2735	Cc1cccc(C(=O)N[C@H](C(=O)N2CCN(C(=O)c3cccs3)CC2)S(=O)(=O)c2ccccc2)c1	0	Train
TNKS1_ML_2736	CCc1nc(C(=O)N(C)[C@H]2CCc3ccccc32)n[n-]1	0	Train
TNKS1_ML_2737	CC[C@H]1CO[C@@H](C)CN1c1[nH+]cc(C(=O)[O-])s1	0	Train
TNKS1_ML_2738	Cc1cscc1[C@H](O)c1cc2c(cc1Cl)OCCCO2	0	Train
TNKS1_ML_2739	C=CCN1C(=O)N[C@H](c2cccc(C)c2)C2=C1CN([C@@H](Cc1ccccc1)C(=O)N1CCOCC1)C2=O	0	Train
TNKS1_ML_2740	CN1CCCc2c1nc(N1CCC3(CC1)C(=O)N(C)c1cc(OCCO)cc(F)c13)[nH]c2=O	1	Train
TNKS1_ML_2741	CC1(C)CC2=NNC(=O)c3cc(F)cc4[nH]c(c2c34)C1	1	Train
TNKS1_ML_2742	Cc1c2cnn(CN3CCN(S(=O)(=O)c4ccc5c(c4)OCCCO5)CC3)c(=O)c2c(C)n1C	0	Train
TNKS1_ML_2743	O=C1Nc2cc(F)cc(F)c2C12CCN(c1nc3c(c(=O)[nH]1)CCCC3)CC2	1	Train
TNKS1_ML_2744	C[C@@H]1Nc2cc(Br)ccc2O[C@H]1C	0	Train
TNKS1_ML_2745	Cc1ccc(NC(=O)c2ccc(NC(=O)N3CCN(C(=O)c4cc(Cc5n[nH]c(=O)c6ccccc56)ccc4F)CC3)cc2)cc1	1	Train
TNKS1_ML_2746	COc1ccc(C(=O)C2CCN(C3CCN(Cc4nc(C)cc(=O)[nH]4)C3=O)CC2)cc1	1	Train
TNKS1_ML_2747	O=c1[nH]c2ccc(S(=O)(=O)N3CCc4sccc4[C@H]3c3cccs3)cc2[nH]1	0	Train
TNKS1_ML_2748	O=C1N(CCO)c2cccc(F)c2C12CCN(c1nc3c(c(=O)[nH]1)CCCC3)CC2	1	Train
TNKS1_ML_2749	COc1cccc2c(O)nc(-c3ccc(N)cc3)nc12	1	Train
TNKS1_ML_2750	O=C(CN1CCN(S(=O)(=O)c2ccc(Br)s2)CC1)Nc1ccc2c(c1)OCO2	0	Train
TNKS1_ML_2751	CC(C)c1nc(C(=O)N2CC[C@H](C)[C@H]3CCCC[C@@H]32)n[n-]1	0	Train
TNKS1_ML_2752	COc1ccc2c(c1)C(CC(=O)N1CCN(C(=O)c3ccccc3N3CCC(=O)NC3=O)CC1)CC2=O	1	Train
TNKS1_ML_2753	C[C@@H]1[NH2+]CCn2c(-c3c(F)cc(F)cc3F)nnc21	0	Train
TNKS1_ML_2754	CCNS(=O)(=O)c1sc2c(c1C(=O)OC)CCN(C(=O)c1cc(C)nn1CC)C2	0	Train

TNKS1_ML_2755	O=C(N[C@H]1C[C@H](c2nnc(-c3ccc(OC(F)F)cn3)n2-c2ccccc2F)C1)c1ccnc2cccnc12	1	Train
TNKS1_ML_2756	CC(C)(OCCO)c1ccc(-c2nc3ccc(F)cc3c(=O)[nH]2)cc1	1	Train
TNKS1_ML_2757	CCOc1ccc(S(=O)(=O)NC[C@H](c2ccc3c(c2)CCN3C)N2CC[NH+](C)CC2)cc1F	0	Train
TNKS1_ML_2758	Cc1nsc(Nc2ccc(F)c(Cl)c2)c1C#N	0	Train
TNKS1_ML_2759	CN1C(=O)Cc2cc(S(=O)(=O)CCC(=O)Nc3ccc(-c4ccno4)cc3)ccc21	0	Train
TNKS1_ML_2760	Cc1cc(I)cn(CC(=O)Nc2ccc3c(c2)OCO3)c1=O	0	Train
TNKS1_ML_2761	Cc1ccc(-n2c(SCc3nc(-c4ccccc4)no3)nnc2-c2ccncc2)cc1	1	Train
TNKS1_ML_2762	CCOC(=O)C1=C(C)[C@@H](c2ccccc2)N2C(=O)[C@@H](Cc3cccc([N+](=O)[O-])c3)SC2=N1	0	Train
TNKS1_ML_2763	O=S(=O)(c1ccc2c(c1)OCCCO2)N1CC[NH+](C[C@@H](O)COc2ccccc2)CC1	0	Train
TNKS1_ML_2764	CCn1nc(C)c2[nH]c(=S)n([C@H]3CCC[C@H](C)[C@@H]3C)c21	0	Train
TNKS1_ML_2765	CCc1ccc([N-]S(=O)(=O)c2c[nH]cn2)cc1	0	Train
TNKS1_ML_2766	CC(C)[C@@H]1NC(=O)C[C@H](C)N(C[C@@H]2CCOC2)C1=O	0	Train
TNKS1_ML_2767	COCCN(Cc1nc2c(c(=O)[nH]1)COCC2)C(=O)CN1CCC(C(=O)c2ccc(OC)cc2)CC1	1	Train
TNKS1_ML_2768	Cc1cc(C)c(C(=O)[O-])c(N2C[C@@H](C)CC[C@@H]2C)[nH+]1	0	Train
TNKS1_ML_2769	Cc1nn(-c2ccccc2)c2c1C=C1C(=O)N[C@H](c3cccs3)[NH+]=C1N2	0	Train
TNKS1_ML_2770	CS(=O)(=O)C1=[NH+]SC2=NC(=O)/C(=C\c3ccc(OC(=O)c4ccccc4)cc3)C(=[NH2+])N21	0	Train
TNKS1_ML_2771	C[C@H]1CN(C(=O)Nc2ccc(N3CCCC3)c(F)c2)C[C@H](C)O1	0	Train
TNKS1_ML_2772	Nc1cc(S(=O)(=O)N[C@@H]2CCN3CCC[C@H]23)ccc1O	0	Train
TNKS1_ML_2773	C[C@H]1CN(C(=O)C[C@@H]2COCCN2)C[C@@H]1C	0	Train
TNKS1_ML_2774	Clc1ccc([C@@H]2C[C@H](c3cccs3)Nc3nnnn32)cc1	0	Train
TNKS1_ML_2775	CC1(C)CCCC[C@@H]1Nc1nsnc1Cl	0	Train
TNKS1_ML_2776	Cc1nc(-c2cccc(NC(=O)c3csc(Nc4ncccn4)n3)c2)cs1	0	Train
TNKS1_ML_2777	O=C(Nc1ccc(S(=O)(=O)Nc2ncccn2)cc1)[C@H]1CCCN(c2ccc(-c3cccs3)n[nH+]2)C1	0	Train
TNKS1_ML_2778	O=C1NN=C2CC(c3ccccc3)Cc3[nH]c4cccc1c4c32	1	Train
TNKS1_ML_2779	Cc1ccc(C[n+]2c(N)c(S(=O)(=O)c3ccc(Cl)cc3)cc3c(=O)n4cc(C)ccc4nc32)cc1	0	Train
TNKS1_ML_2780	O=C(NCC[NH+]1CCN(C(=O)Nc2ccc(F)cc2)CC1)c1ccsc1	0	Train
TNKS1_ML_2781	COC(=O)c1sc2c(cnc3cc(F)ccc32)c1O	0	Train
TNKS1_ML_2782	CNc1nc(C)cc(-n2cc(Cl)cn2)n1	0	Train
TNKS1_ML_2783	N#Cc1ccc(Cl)cc1Nc1cccc(F)n1	0	Train
TNKS1_ML_2784	C[C@@H](c1ccc(F)cc1)N(Cc1nc2c(c(=O)[nH]1)COCC2)C(=O)CN1CCC(C(=O)c2ccc(F)cc2)CC1	1	Train
TNKS1_ML_2785	CCOc1cccc(N2C(=O)NC(=O)/C(=C/c3ccc(I)o3)C2=O)c1	0	Train
TNKS1_ML_2786	C=CCOc1ccc([C@H]2C(C(=O)c3nc4c(C)cccn4c3C)=C([O-])C(=O)N2c2nccs2)cc1	0	Train
TNKS1_ML_2787	CC(=O)N[C@@H](CC1=c2ccccc2=[NH+]C1)C(=O)N1CCN(Cc2c(C)nc3ccccn23)CC1	0	Train
TNKS1_ML_2788	CCC(=O)N(Cc1ccc(C)o1)[C@@H](C(=O)NCS(=O)(=O)c1ccc(C)cc1)c1ccc(O)c(OC)c1	0	Train
TNKS1_ML_2789	COc1cc(/C=C/C(=O)c2ccc3c(c2)NC(=O)CO3)cc(Br)c1O	0	Train
TNKS1_ML_2790	Cc1nc([O-])cc(C2CC[NH+](Cc3ccc4nonc4c3)CC2)n1	0	Train
TNKS1_ML_2791	O=C(NCc1ccco1)C1=C(Cl)C2=[NH+][C@H](c3ccc(F)cc3)C[C@@H](C(F)(F)F)N2N1	0	Train
TNKS1_ML_2792	CC(C)C1(N)CN(C(=O)c2cccc3c2OCCO3)C1	0	Train

TNKS1_ML_2793	O=S(=O)(Nc1cccc(S(=O)(=O)N2CCCCC2)c1)c1ccc2c(c1)OCCCO2	0	Train
TNKS1_ML_2794	CC(=O)O[C@H]1C[C@H](O)[C@]2(C)[C@@H]1[C@H](C)C[C@@H]1OC(=O)[C@@H](C[NH+]3CCCC3)[C@H]1[C@@H]2OC(C)=O	0	Train
TNKS1_ML_2795	Nc1c(C(=O)NCC(F)(F)F)sc2[nH+]c3c(cc12)CCC3	0	Train
TNKS1_ML_2796	O=C1NC(c2ccccc2)=C(c2nc3ccccc3s2)[C@H](c2ccccc2[N+](=O)[O-])N1	0	Train
TNKS1_ML_2797	Cc1nnsc1C(=O)Nc1nc(CCl)cs1	0	Train
TNKS1_ML_2798	C[C@@H](Cn1cccn1)n1cncc1-c1c[nH+]ccc1N	0	Train
TNKS1_ML_2799	Cn1ccc(NC(=O)c2cc(Br)cc(S(=O)(=O)N3CCOCC3)c2F)n1	0	Train
TNKS1_ML_2800	Cc1ccccc1N1C[C@H](C(=O)Nc2ccc(S(=O)(=O)NC(C)(C)C)cc2)CC1=O	0	Train
TNKS1_ML_2801	Cc1cccc2c(=O)[nH]c3c(c12)COC(C(C)(C)CO)C3	1	Train
TNKS1_ML_2802	Cc1cccc(OCC(=O)NNC(=O)c2cc(S(=O)(=O)N(C)C)ccc2N2CCCC2)c1	0	Train
TNKS1_ML_2803	O=C([O-])c1ccc(Sc2ncns2)o1	0	Train
TNKS1_ML_2804	O=C(COC(=O)Cn1c(=O)oc2ccccc21)Nc1nc(-c2ccc(F)c(F)c2)cs1	0	Train
TNKS1_ML_2805	C#CC[C@H](CC)NC(=O)N1CCN([C@H](C)C(=O)N2CCCC2)CC1	0	Train
TNKS1_ML_2806	COc1ccc(C(=O)C2CCN(CC(=O)NCc3nc4c(c(=O)[nH]3)COCC4)CC2)cc1OC	1	Train
TNKS1_ML_2807	COc1ccc2c3c1O[C@@H]1C[C@@H](O)C=C[C@@]31CCN(c1ccnc(NCc3ccccc3)n1)C2	0	Train
TNKS1_ML_2808	COc1ccc2nc(-c3ccc4c(c3)CCN4)[nH]c2n1	0	Train
TNKS1_ML_2809	Cc1cc(C)nc(Nc2cc(C)[nH+]c([C@H]3CCCN(C(=O)c4c(C(C)C)noc4C)C3)c2)n1	0	Train
TNKS1_ML_2810	O=C(CN1CCC(n2cc(-c3ccccc3)cn2)CC1)NCc1nc2c(c(=O)[nH]1)COCC2	1	Train
TNKS1_ML_2811	Nc1cc(Cl)cc(Cl)c1S(=O)(=O)N1CC[C@@H](O)C1	0	Train
TNKS1_ML_2812	O=C1CCCN1CCCNc1nc(-c2ccccn2)nc2c1CCNC2	0	Train
TNKS1_ML_2813	N#Cc1c(Cl)nc2c(c1C#N)CCCC2	0	Train
TNKS1_ML_2814	Cc1ccc2nc[nH]c2c1Br	0	Train
TNKS1_ML_2815	O=C(c1ccc(F)cc1)C1CCN(CC(=O)N(Cc2nc3c(c(=O)[nH]2)COCC3)C[C@H]2CCCO2)CC1	1	Train
TNKS1_ML_2816	Cc1cc(C)cc(N2C[C@@H](C(=O)Nc3nc4ccc(S(C)(=O)=O)cc4s3)CC2=O)c1	0	Train
TNKS1_ML_2817	Cn1c(=O)n(C)n(-c2ccc(C(=O)N[C@H](Cc3ccccc3)c3ccccc3)cc2)c1=O	0	Train
TNKS1_ML_2818	C/C(=N/NC(=O)c1cc(Br)c[nH]1)[C@H]1C[C@H]1c1ccccc1	0	Train
TNKS1_ML_2819	Cc1ccc(NC2CCC3(CC2)OCCO3)cc1Cl	0	Train
TNKS1_ML_2820	C1=Cc2nc3n(c2=CC1)C=C1C=CC=3C1	0	Train
TNKS1_ML_2821	COc1cnn(C)c1[C@H](NN)c1cc2c(s1)CCCC2	0	Train
TNKS1_ML_2822	Cc1cc(N)ccc1C(=O)c1ccc2c(c1)CC(C)(C)O2	0	Train
TNKS1_ML_2823	CC1(C)Cc2nc(-c3ccncc3)ncc2[C@H](NC(=O)[C@@H]2CC(=O)N(C3CCCCC3)C2)C1	0	Train
TNKS1_ML_2824	O=C(Nc1cccc(N2CCCCS2(=O)=O)c1)c1c[nH]nc1-c1ccccc1	0	Train
TNKS1_ML_2825	O=C(c1ccc(F)cc1)C1CCN(CC(=O)N(Cc2ccccc2)Cc2nc3c(c(=O)[nH]2)COCC3)CC1	1	Train
TNKS1_ML_2826	Cn1c(=O)sc2cc(S(=O)(=O)NC3CCCCCC3)ccc21	0	Train
TNKS1_ML_2827	Cc1ccoc1C(=O)Nc1cnc(Br)cn1	0	Train
TNKS1_ML_2828	O=C(NC1CCCCCC1)[C@@H]1c2cccn2-c2c(cnn2-c2ccccc2)C(=O)N1C1CCCCC1	0	Train
TNKS1_ML_2829	C=CCOC(=O)C1=C(C)Nc2c(c(=O)n(C)c(=O)n2C)[C@H]1c1cc(OC)c(OC)c(OC)c1	0	Train

TNKS1_ML_2830	CC1(C)CCCC[C@@H]1n1ccc2c1CCCC2=O	0	Train
TNKS1_ML_2831	CCOc1ccc(/C(C)=N/NC(=O)c2cc(-c3c(C)nn(-c4ccccc4)c3C)n[nH]2)cc1	0	Train
TNKS1_ML_2832	CCOC(=O)C1=C(N)Oc2c(c(C)nn2-c2ccccc2)[C@H]1c1ccc(N(C)C)cc1	0	Train
TNKS1_ML_2833	O=C(CSc1nnc(SCC(=O)[C@H]2C=C3C=CC=C[C@H]3OC2=O)s1)c1cc2ccccc2oc1=O	0	Train
TNKS1_ML_2834	O=C(CN1CCC(C(=O)c2ccc(OCCC3CC3)cc2)CC1)NCc1nc2c(c(=O)[nH]1)COCC2	1	Train
TNKS1_ML_2835	C[C@H]1CN(c2cc(F)c3c(c2)N(C)C(=O)C32CCN(c3nc4c(c(=O)[nH]3)CCCN4C)CC2)C[C@@H](C)N1	1	Train
TNKS1_ML_2836	O=C1CCN(c2ccccc2C(=O)NCc2ccc(-n3cccn3)cc2)C(=O)N1	1	Train
TNKS1_ML_2837	Cc1ccc(-c2nc3c(c(=O)[nH]2)COCC3)cc1	1	Train
TNKS1_ML_2838	Cc1cc(Oc2ccc(Cl)cc2C#N)ccc1N	0	Train
TNKS1_ML_2839	O=C(Nc1cccc2c(=O)[nH]ccc12)[C@H]1CCCNC1	1	Train
TNKS1_ML_2840	COc1ccc(Cl)cc1NC(=O)N1CCN(c2c(C)[nH+]n([C@@H]3CCS(=O)(=O)C3)c2C)CC1	0	Train
TNKS1_ML_2841	O=C1N=C(CSC2=NN[C@H](CN3CCCC3=O)O2)N=C2S[C@@H](c3ccccc3)C=C12	0	Train
TNKS1_ML_2842	COCCOc1ccc(C(=O)C2CCN(CC(=O)NCc3nc4c(c(=O)[nH]3)COCC4)CC2)cc1	1	Train
TNKS1_ML_2843	Cc1c[nH+]ccc1N1CCN(C(=O)Nc2cccc([N-]S(C)(=O)=O)c2)CC1	0	Train
TNKS1_ML_2844	O=C(C[C@H]1CCC[NH2+]1)N1C[C@@H]2CC[C@H](C1)O2	0	Train
TNKS1_ML_2845	CCOc1cc(C(F)(F)F)nc(N2CCNCC2)n1	0	Train
TNKS1_ML_2846	C[C@H]1CC[C@@H](NC(=O)[C@@H]2CCCC[C@@H]2C(=O)O)C[C@@H]1C	0	Train
TNKS1_ML_2847	Oc1ccc2ccccc2c1/C=N\Nc1ncnc2sc3c(c12)CCCC3	0	Train
TNKS1_ML_2848	Cc1ccc(C(=O)NCC#Cc2ccc(N(C)C)cc2)cc1[N+](=O)[O-]	0	Train
TNKS1_ML_2849	CC(C)C1=NO[C@H](c2nc(-c3ccc(F)cc3)no2)C1	0	Train
TNKS1_ML_2850	CN(C)[C@@H](CNC(=O)Nc1ccc(-n2cncn2)cc1C(F)(F)F)c1ccsc1	0	Train
TNKS1_ML_2851	CNc1nc([C@H]2CCCN2S(=O)(=O)Cc2cc(F)cc(F)c2)nc2c1C[NH+](C)CC2	0	Train
TNKS1_ML_2852	Cn1c(=O)c(C(=O)COC(=O)c2cncc(Br)c2)c(N)n(Cc2ccccc2)c1=O	0	Train
TNKS1_ML_2853	CC[NH+](CCCNS(=O)(=O)c1ccc2c(c1)n(C)c(=O)c(=O)n2C)Cc1ccccc1	0	Train
TNKS1_ML_2854	C[C@]12CCCN1CC1=NNC(=O)c3cc(F)cc4[nH]c2c1c34	1	Train
TNKS1_ML_2855	O=C(NCCn1nc(-c2ccc(F)cc2)ccc1=O)C1CCN(c2cc(-n3cncn3)ncn2)CC1	0	Train
TNKS1_ML_2856	Cc1cc(=O)[nH]c2cc(-c3cccc(F)c3)ccc12	1	Train
TNKS1_ML_2857	COc1ccc(-n2c(SCc3nc(-c4ccccc4)no3)nnc2-c2ccncc2)cc1	1	Train
TNKS1_ML_2858	COc1cc(/C=C2/SC(=S)N(c3c(C)n(C)n(-c4ccccc4)c3=O)C2=O)ccc1OC(=O)c1ccccc1	0	Train
TNKS1_ML_2859	Cc1cc2c(=O)n(CC(=O)NC(=O)N[C@@H]3CCCC[C@@H]3C)cnc2s1	0	Train
TNKS1_ML_2860	CNc1ncccc1C[NH+]1CCC[C@@H](c2cncc(-c3cccc(Cl)c3)n2)C1	0	Train
TNKS1_ML_2861	CC1(C)CN(c2ccc(C#N)cc2)C1	0	Train
TNKS1_ML_2862	O=C(c1ccc(OCc2ccccc2)cc1)C1CCN(CC(=O)N(Cc2nc3c(c(=O)[nH]2)COCC3)CC2CC2)CC1	1	Train
TNKS1_ML_2863	CCn1ccc(C(=O)Nc2sc3c(c2C(=O)NCc2ccc[nH+]c2)CCCC3)n1	0	Train
TNKS1_ML_2864	CN1CCCc2c1nc(N1CCC3(CC1)C(=O)N(C)c1cc(N4CCCCC4)cc(F)c13)[nH]c2=O	1	Train
TNKS1_ML_2865	CCOc1cccc([C@H]2C(C(=O)c3c(C)[nH]c(C(=O)OC)c3C)=	0	Train

	C([O-])C(=O)N2CC[NH+](C)C)c1		
TNKS1_ML_2866	Cc1[nH]c[nH+]c1CN1CCc2[nH]cnc2[C@H]1c1ccc(Cl)c(Cl)c1	0	Train
TNKS1_ML_2867	COc1ccc(NS(=O)(=O)c2cc(-c3nc(C)cs3)n(CC(=O)N3CCCC3)c2)cc1Cl	0	Train
TNKS1_ML_2868	CC1(C)OC(=O)N([C@H]2CC[C@H](n3c(=O)[nH]c4cc(Cl)ccc43)CC2)[C@H]1c1ccccc1	1	Train
TNKS1_ML_2869	COc1ccccc1N1CCN(c2nc3c(c(=O)[nH]2)CSCC3)CC1	1	Train
TNKS1_ML_2870	Cc1nc2c(c(N(C)C[C@@H]3COc4ccccc4O3)n1)CC[NH2+]CC2	0	Train
TNKS1_ML_2871	O=c1cc(-c2ccc(O)cc2)oc2cc(O)cc(O)c12	1	Train
TNKS1_ML_2872	COc1ccc(Cl)c2sc(N(CCCn3cc[nH+]c3)C(=O)[C@H]3Oc4ccccc4O[C@H]3C)nc12	0	Train
TNKS1_ML_2873	C[C@@H](NC(=O)[C@@H]1CCCN(S(=O)(=O)N2CCOCC2)C1)C12CC3CC(CC(C3)C1)C2	0	Train
TNKS1_ML_2874	Cc1cccn2c(=O)c(/C=C3\SC(=S)N(CCCCCC(=O)[O-])C3=O)c(N3CC[NH+](C)CC3)nc12	0	Train
TNKS1_ML_2875	N#Cc1cccc(Nc2ccc3ncccc3c2)n1	0	Train
TNKS1_ML_2876	COc1cccc([C@H]2OCCn3c(-c4cccc(C)c4)c4c(=O)n(C)c(=O)n(C)c4c32)c1O	0	Train
TNKS1_ML_2877	Nc1nnc(-c2ccc(Br)c(F)c2)s1	0	Train
TNKS1_ML_2878	N#Cc1cccc(-n2nc(-c3cccc(Cl)c3)cc2N)c1	0	Train
TNKS1_ML_2879	Cc1[nH+]n(C)c(O[C@H]2CC[C@H](C)[C@H](C)C2)c1C(=O)[O-]	0	Train
TNKS1_ML_2880	C[C@H]1CCC[C@](C#N)(N2CCNCC2)C1	0	Train
TNKS1_ML_2881	CC(=O)C1=C(C)C(C(=O)Nc2cc(S(=O)(=O)N3CCCCC3)ccc2Oc2ccccc2)=N[C@@H]1C	0	Train
TNKS1_ML_2882	Cc1ccc(C(=O)N(CCCn2ccnc2)c2nc3c(C)c(C)ccc3s2)cc1[N+](=O)[O-]	0	Train
TNKS1_ML_2883	Nc1c(-c2nc(-c3ccc(F)cc3)cs2)nnn1-c1cccc(F)c1	0	Train
TNKS1_ML_2884	O=C(NC[C@@H]1CN2CCCC[C@H]2CO1)N1CC[C@]2(CCOC2)C1	0	Train
TNKS1_ML_2885	O=C(c1cc2scc(-c3ccccc3)c2[nH]1)N1CCC(C(=O)N2CCOCC2)CC1	0	Train
TNKS1_ML_2886	c1cc(C[NH2+][C@H]2CCCc3cn[nH]c32)c2c(c1)OCCCO2	0	Train
TNKS1_ML_2887	Cc1cc(Br)ccc1-n1c(CO)[nH+]c2cc(N)ccc21	0	Train
TNKS1_ML_2888	Cc1ccc(C)c(S(=O)(=O)C2(C(=O)NCc3ccc[nH+]c3N3CCOCC3)CCCC2)c1	0	Train
TNKS1_ML_2889	Cc1cc(F)cc2c(=O)[nH]c(-c3ccc(C(C)(C)OCCO)cc3)nc12	1	Train
TNKS1_ML_2890	Cc1cc2nc(CSc3nnc(-c4ccncc4)n3-c3ccccc3Cl)cc(=O)n2cc1C	1	Train
TNKS1_ML_2891	Cc1nnc(NC(=O)CSc2nnc3c4c(-c5ccc(Cl)cc5)csc4ncn23)s1	0	Train
TNKS1_ML_2892	CCc1nc2sc([C@@H](c3ccc(F)cc3)N3C[C@H](C)O[C@H](C)C3)c(O)n2n1	0	Train
TNKS1_ML_2893	COCC(=O)N[C@@H](CC(C)C)c1nnc2n1CC[NH+](Cc1ccc3c(c1)OCCO3)CC2	0	Train
TNKS1_ML_2894	Cc1ccc2c(=O)[nH]c(-c3ccc([C@@](C)(O)CCO)cc3)cn12	1	Train
TNKS1_ML_2895	C[C@H](NC(=O)Cn1c(=O)oc2ccc(Cl)cc21)c1ccc(N2CCOCC2)cc1	0	Train
TNKS1_ML_2896	Cc1cc(C(=O)O[C@@H](C)C(=O)NC23CC4CC(CC(C4)C2)C3)c(C)n1C1CC1	0	Train
TNKS1_ML_2897	CC(=O)Nc1ccc(-n2ccc3nc4ccn(Cc5ccc[nH+]c5)c(=O)c4cc3c2=O)cc1	0	Train
TNKS1_ML_2898	Cc1ccc(C(=O)[C@@H](C)n2nc3n(C(C)C)c(=O)c4ccc(C(=O)NC(C)C)cc4n3c2=O)cc1C	0	Train
TNKS1_ML_2899	O=C(CN1CCC(C(=O)c2ccc(F)cc2)CC1)NCc1nc2c(c(=O)[nH]1)CCCC2	1	Train
TNKS1_ML_2900	C[C@@]1(c2ccco2)NC(=O)N(C[C@@H](O)COc2ccc(Br)cc2)C1=O	0	Train

TNKS1_ML_2901	C[C@H](O)[C@H](C)C1(C(=O)O)CCCCCC1	0	Train
TNKS1_ML_2902	COC(=O)NC(=O)c1c(NC(=O)CCCS(=O)(=O)c2ccc(Cl)cc2)sc(C)c1C	0	Train
TNKS1_ML_2903	Cc1ccc(-n2nc(C(=O)NCc3ccc[nH+]c3)c(=O)n(Cc3ccccc3C)c2=O)cc1	0	Train
TNKS1_ML_2904	CCn1cc(C(=O)NCc2ccc(Cl)cc2)c(=O)c2cc(F)c(N3CCN(C(=O)c4ccco4)CC3)cc21	0	Train
TNKS1_ML_2905	c1cc(CNc2nc(C3CC3)ns2)c2c(c1)OCO2	0	Train
TNKS1_ML_2906	Cc1ccc(-n2nc(C)c3c(C)c(CCC(=O)NCCN4CC[NH+](C)CC4)c(C)[nH+]c32)cc1	0	Train
TNKS1_ML_2907	CCOc1ccc([C@@H]2CC(=O)C3=C(C2)NC(=O)C[C@H]3c2cc3c(cc2Br)OCO3)cc1OC	0	Train
TNKS1_ML_2908	CCc1cccc(C)c1-n1c(C)c(C)c2c(N)ncnc21	0	Train
TNKS1_ML_2909	Cc1ccc(-c2nc(C[NH+]3CCN(c4ccccc4O)CC3)co2)c(C)c1	0	Train
TNKS1_ML_2910	COc1ccc([C@@H]2C[C@]3(CC(C)(C)NC(=S)N3)Oc3cc(OC(C)=O)cc(C)c32)cc1	0	Train
TNKS1_ML_2911	Nc1ccc(Oc2ncncc2I)cc1	0	Train
TNKS1_ML_2912	C[C@H]1Cn2c(nnc2C(C)(F)F)CN1C(=O)c1cccc(C(F)(F)c2n[nH]c(=O)c3ccccc23)c1	1	Train
TNKS1_ML_2913	CC(=O)N[C@H](C)c1nc2ccccc2n1CC(=O)Nc1ccc(Cl)c(S(=O)(=O)N(C)C)c1	0	Train
TNKS1_ML_2914	COc1ccc2cc(C[NH+]3CCc4c([O-])nc(-c5ccc[nH+]c5)nc4C3)c(OC)nc2c1	0	Train
TNKS1_ML_2915	Cc1nc2cc(S(=O)(=O)c3ccc4nc(C)[nH]c4c3)ccc2[nH]1	0	Train
TNKS1_ML_2916	Cn1cc(NS(=O)(=O)c2ccnc(Cl)c2)cn1	0	Train
TNKS1_ML_2917	Cc1ccc(Br)cc1N[C@@H]1[C@H]2CCO[C@@H]2C1(C)C	0	Train
TNKS1_ML_2918	Nc1cccc(-c2nc3cccc(Cl)c3o2)c1	0	Train
TNKS1_ML_2919	Cc1ncc2c(=O)[nH]c(-c3ccc(C(C)(C)C)cc3)cn12	1	Train
TNKS1_ML_2920	Cc1cc2ncn(-c3nc(Cl)ncc3F)c2cc1C	0	Train
TNKS1_ML_2921	O=C(NCCc1cccc[nH+]1)[C@H]1CC12CCN(C(=O)C1(N3CCOCC3)CCCCC1)CC2	0	Train
TNKS1_ML_2922	Cc1ccc(NC(=O)NCc2ccc(N3CCO[C@@H](C)C3)nc2)c(F)c1	0	Train
TNKS1_ML_2923	COc1ccc(C(=O)Nc2c(C)n(C)n(-c3ccccc3)c2=O)cc1S(=O)(=O)NC(C)C	0	Train
TNKS1_ML_2924	COc1ccccc1[C@@H]1C(C(=O)c2sc(C)nc2C)=C([O-])C(=O)N1CC[NH+](C)C	0	Train
TNKS1_ML_2925	O=S(=O)([N-]c1ccc2c(c1)OC(F)(F)O2)[C@H]1CCNC1	0	Train
TNKS1_ML_2926	Cc1ccc2oc(C(=O)NCc3ccc4c(c3)n(C)c(=O)n4C)c(C)c2c1	0	Train
TNKS1_ML_2927	C[C@]1(c2nc3cc(F)c(Br)cc3[nH]2)CCCO1	0	Train
TNKS1_ML_2928	Cc1cc(C(=O)Nc2nc(-c3ccc(Cl)cc3)c(C)s2)c2cnn(C)c2n1	0	Train
TNKS1_ML_2929	O=C(Nc1nn2cnnc2s1)C1CCN(c2ncc(C(F)(F)F)cc2Cl)CC1	0	Train
TNKS1_ML_2930	C[C@H]1[C@H]2C[NH2+]C[C@@H]2CN1C(=O)c1cc(Cl)c(Cl)[n-]1	0	Train
TNKS1_ML_2931	N#Cc1cccc(S(=O)(=O)[N-]c2ccc3c(c2)N=[NH+]C3)c1	0	Train
TNKS1_ML_2932	CCOC(=O)c1c(NC(=O)C(=O)N/N=C/c2cn(-c3ccccc3)nc2-c2ccc(C)cc2)sc2c1CC[C@@H](C)C2	0	Train
TNKS1_ML_2933	COc1ccc(NC(=S)N2CCN(Cc3c(C)nn(C)c3C)CC2)cc1OC	0	Train
TNKS1_ML_2934	Cc1nc2c(c(SCC(=O)c3ccc(O)cc3O)n1)CCC2	0	Train
TNKS1_ML_2935	CC1(C)CCN([C@H]2CCCCC[C@@H]2C#N)CC1	0	Train
TNKS1_ML_2936	Cc1cccc2c(O)nc(-c3ccc(C(F)(F)F)cc3)nc12	1	Train
TNKS1_ML_2937	O=C(NC1CCCC1)[C@H](c1cccs1)N(C(=O)c1cnccn1)c1ccc(O)cc1	0	Train
TNKS1_ML_2938	N#Cc1c(Cn2c(=O)n(C[C@H]3CC[C@H](C(=O)O)CC3)c(=O)c3c(F)cccc32)cccc1C(F)(F)F	1	Train

TNKS1_ML_2939	O=[N+]([O-])c1cc(Cl)ccc1CN1CCC[C@@H](O)C1	0	Train
TNKS1_ML_2940	Cc1n[nH]c(C)c1S(=O)(=O)N1CCCN(C(=O)c2cc[nH]c(=O)c2)CC1	0	Train
TNKS1_ML_2941	CCCCn1c(N2CC[NH+](C)CC2)c(/C=C2/SC(=S)N(Cc3ccccc3)C2=O)c(C)c(C#N)c1=O	0	Train
TNKS1_ML_2942	O=C1/C(=C/c2cccc([N+](=O)[O-])c2)Oc2c1ccc(O)c2C[NH+]1C[C@@H]2C[C@@H](Cn3c2cccc3=O)C1	0	Train
TNKS1_ML_2943	CN1CCc2cc([C@@H](CNC(=O)C(=O)Nc3ccc(F)c([N+](=O)[O-])c3)[NH+](C)C)ccc21	0	Train
TNKS1_ML_2944	C#CCn1/c(=N\C(=O)Cc2ccc(SC)cc2)sc2cc(S(N)(=O)=O)ccc21	0	Train
TNKS1_ML_2945	Cc1cccc(NC(=O)c2cnn3c2[NH+]=C(C(=O)[O-])C[C@H]3c2cccc(Br)c2)c1	0	Train
TNKS1_ML_2946	CCc1nc2n(n1)C[C@H](Nc1ncnc3ccccc13)CC2	0	Train
TNKS1_ML_2947	CCOC(=O)c1ccc2c(c1)s/c(=N/C(=O)c1ccc(Br)o1)n2CC(=O)OC	0	Train
TNKS1_ML_2948	O=C(c1ccccn1)C1CCN(CC(=O)N(Cc2nc3c(c(=O)[nH]2)COCC3)CC2CC2)CC1	1	Train
TNKS1_ML_2949	N#Cc1c(Cn2c(=O)n(C[C@H]3CC[C@H](C(=O)O)CC3)c(=O)c3c(Cl)cccc32)cccc1C(F)(F)F	1	Train
TNKS1_ML_2950	C=CCn1c(CCc2ccc(I)cc2)nnc1SCc1[nH]c2cc(C)ccc2[nH+]1	0	Train
TNKS1_ML_2951	COc1ccc([C@H]2C3=C(CC(C)(C)CC3=O)Nc3n[nH]c(C)c32)cc1	0	Train
TNKS1_ML_2952	CCOC(=O)c1c(-c2cccs2)csc1NC(=O)c1c(S)n(-c2ccccc2C)c2ccc(C)c[n+]12	0	Train
TNKS1_ML_2953	O=C(CCc1ccc(F)cc1)N(Cc1nc2c(c(=O)[nH]1)COCC2)Cc1cccs1	1	Train
TNKS1_ML_2954	CCOc1ccc(-c2nnc([C@H]3C[C@H](NC(=O)c4ccccn4)C3)n2-c2ccccc2)nc1	1	Train
TNKS1_ML_2955	O=C(CCc1ccccc1F)N(Cc1nc2c(c(=O)[nH]1)COCC2)Cc1cccs1	1	Train
TNKS1_ML_2956	COc1ccc(-c2ccc3c(c2)C[C@@H](CNC(=O)c2cc(C)nc4ccc(C)cc24)O3)nn1	0	Train
TNKS1_ML_2957	Cc1cc(C)cc(NC=C2C(=O)N(C)C(=O)N(C)C2=O)c1	0	Train
TNKS1_ML_2958	COc1cc([N+](=O)[O-])ccc1NC(=S)NC(=O)c1cc2ccccc2c(Br)c1OC	0	Train
TNKS1_ML_2959	CCOC(=O)C1=C(CSc2nc(-c3cccs3)cc(-c3ccc(Cl)cc3)c2C#N)OC(N)=C(C#N)[C@H]1c1cccnc1	0	Train
TNKS1_ML_2960	Oc1ccc2c(c1)OC[C@H]2N1C[C@H]2C[NH2+]C[C@@H]2C1	0	Train
TNKS1_ML_2961	c1nc2c([nH]1)C[C@@H](c1nc(C3CCCCC3)no1)NC2	0	Train
TNKS1_ML_2962	COCCNC(=O)c1ccc(-c2ccc3c(C)cc(=O)[nH]c3c2)c(Cl)c1	1	Train
TNKS1_ML_2963	Cc1c(CN2CCc3[nH]c4ccc(Cl)cc4c3C2)cc(C#N)n1C	0	Train
TNKS1_ML_2964	Cc1noc(C)c1CSc1ncccc1C(=O)Nc1nc2c(s1)CN(C)CC2	0	Train
TNKS1_ML_2965	O=C(CC1CCCCC1)Nc1nnc2n1CCS2	1	Train
TNKS1_ML_2966	C=CCOc1cccc([C@@H]2C(C(=O)c3nc4c(C)cccn4c3C)=C(O)C(=O)N2CCCn2cc[nH+]c2)c1	0	Train
TNKS1_ML_2967	COCc1c(C(=O)N[C@@H](c2ccc[nH+]c2)C(F)(F)F)cnn1-c1nccc(-c2cccs2)n1	0	Train
TNKS1_ML_2968	CCN(CC)S(=O)(=O)c1ccc(NC(=O)c2ccc(S(=O)(=O)N(C)Cc3ccccc3)cc2)cc1	0	Train
TNKS1_ML_2969	Cc1cccc2c(O)nc(-c3ccc(N)cc3)nc12	1	Train
TNKS1_ML_2970	COC(=O)c1sc(NC(=O)CCN2C(=O)/C(=C\c3ccc(OC)cc3)SC2=S)nc1C	0	Train
TNKS1_ML_2971	COc1cc(F)c2c(c1)N(C)C(=O)C21CCN(c2nc3c(c(=O)[nH]2)	1	Train

	CCCN3C)CC1		
TNKS1_ML_2972	Nc1sc2c(c1-c1nc([C@H]3CCOC3)no1)CCCC2	0	Train
TNKS1_ML_2973	N#Cc1ccc2c(c1)[nH]c(=O)n2[C@H]1C[C@H](c2nnc(-c3ccncn3)n2-c2ccccc2F)C1	1	Train
TNKS1_ML_2974	O=C(N[C@H]1C[C@H](c2nnc(-c3nccs3)n2-c2ccccc2F)C1)c1ccnc2cccnc12	1	Train
TNKS1_ML_2975	Cc1cc(Br)ccc1NC(=O)c1cccn1C	0	Train
TNKS1_ML_2976	CC(C)[C@H]1CC[C@H]2NCCCS(=O)(=O)[C@@H]2C1	0	Train
TNKS1_ML_2977	O=C(NCCc1ccncc1)c1nc2c(NCc3ccccn3)nc3ccccc3n2n1	0	Train
TNKS1_ML_2978	COC(=O)c1ccc(CSc2nc(N3CC[NH2+]CC3)c3c(c2C#N)CC(C)(C)OC3)o1	0	Train
TNKS1_ML_2979	Cc1ccc(S(=O)(=O)/C(C#N)=C/c2c(Oc3ccc(C)cc3C)nc3ccccn3c2=O)cc1	0	Train
TNKS1_ML_2980	COc1cc(/C=N/NC(=O)[C@@H]2C=C(C)N=N2)c(Br)c(Br)c1O	0	Train
TNKS1_ML_2981	Oc1nc(-c2ccc(F)cc2)nc2ccccc12	1	Train
TNKS1_ML_2982	O=S(=O)(NC[C@H](c1ccc2c(c1)OCO2)N1CCN(c2ccc(F)cc2)CC1)c1cccs1	0	Train
TNKS1_ML_2983	Cc1cnc2nc([C@@H]3[NH2+]CCc4sccc43)[n-]c2c1	0	Train
TNKS1_ML_2984	CC(C)c1cc(C(=O)N2CC(=O)N(Cc3ccc[nH+]c3)C[C@@H](OCc3ccccc3)C2)n[nH]1	0	Train
TNKS1_ML_2985	C[C@@H]1C[C@@H]1n1c(N)nc2cc(Br)ccc21	0	Train
TNKS1_ML_2986	COc1cccc(-c2cnnc(-n3nc(C)c4c3NC(=O)C[C@H]4c3cc(C)ccc3C)n2)c1	0	Train
TNKS1_ML_2987	Cc1cnn(-c2cnc(C(C)C)nc2CO)c1	0	Train
TNKS1_ML_2988	CCOc1cc(/C=C(/C#N)c2nc3ccc(OC)cc3[n-]2)cc(I)c1OCC	0	Train
TNKS1_ML_2989	COc1cc2c(cc1OC)C(C[C@H]1CN(C(=O)N3CCOCC3)CC[C@H]1CC(=O)[O-])=[NH+]CC2	0	Train
TNKS1_ML_2990	c1cncc([C@@H](c2nc(-c3ccc4[nH]cnc4c3)no2)[NH+]2CCCC2)c1	0	Train
TNKS1_ML_2991	CCCn1c(N2CCC(C(=O)OCC)CC2)c(/C=C2/SC(=S)N([C@H](C)CC)C2=O)c(C)c(C#N)c1=O	0	Train
TNKS1_ML_2992	O=C1/C(=C\c2ccc(-c3cccc([N+](=O)[O-])c3)o2)C([O-])=NC(=S)N1c1ccc(Cl)cc1	0	Train
TNKS1_ML_2993	C[C@@]1(c2ccc(S(C)(=O)=O)cc2)NC(=O)N(Cc2sc3ccccc3c2Cl)C1=O	0	Train
TNKS1_ML_2994	C[C@@H]1CCC[NH+]([C@@H]2CC3(CC[C@@H]2O)OCCO3)[C@@H]1C	0	Train
TNKS1_ML_2995	CCCN(CCC)C(=O)c1cc(NC(=O)c2cccnc2SC)c(=O)n(C)c1	0	Train
TNKS1_ML_2996	c1coc(-c2noc([C@@H]3Cc4ccccc4N3)n2)c1	0	Train
TNKS1_ML_2997	Cc1ccc(C(=O)NCC(=O)N2CCN(C/C=C/c3ccccc3)CC2)cc1C	0	Train
TNKS1_ML_2998	CN(C)c1ccc2nc(-c3ccc(F)cc3)[nH]c2n1	0	Train
TNKS1_ML_2999	CCOc1cc([C@H]2C(C(=O)c3c(C)nc4ccccn34)=C([O-])C(=O)N2Cc2ccco2)ccc1O	0	Train
TNKS1_ML_3000	Cc1ccc(N2C[C@H](C(=O)NC[C@@H](c3cccn3C)N3CC[NH+](C)CC3)CC2=O)cc1C	0	Train
TNKS1_ML_3001	COc1cc(-c2cc(C(=O)NN/C=C3/C=C(Br)C=CC3=O)[nH]n2)ccc1OC(C)C	0	Train
TNKS1_ML_3002	O=C(c1ccc(F)cc1)C1CCN(CC(=O)N(Cc2ccc(F)cc2)Cc2nc3c(c(=O)[nH]2)COCC3)CC1	1	Train
TNKS1_ML_3003	O=C(Nc1cc(F)c(F)cc1F)c1ccc(Br)cc1[O-]	0	Train
TNKS1_ML_3004	Oc1ccc2ccccc2c1/C=N\c1ccc(Br)cn1	0	Train
TNKS1_ML_3005	O=C(Nc1ccc2[nH]ccc2c1)[C@H]1CC=CC[C@H]1C(=O)O	0	Train
TNKS1_ML_3006	O=C(CN1CCC(C(=O)c2ccc3c(c2)OCO3)CC1)NCc1nc2c(c(=O)[nH]1)COCC2	1	Train
TNKS1_ML_3007	COc1ccc(C(=O)C2CCN(CC(=O)NCc3cc4c(c(=O)[nH]3)CO	1	Train

	CC4)CC2)cc1		
TNKS1_ML_3008	C[C@H]1CCC[C@@]1(C#N)N1CCCC1	0	Train
TNKS1_ML_3009	C[C@H]1C[C@H](C)[C@@H]2[C@@H](C1)NC[C@H](C)S2(=O)=O	0	Train
TNKS1_ML_3010	C/C(=N/O)c1ccccc1O[C@@H]1CCO[C@]2(CCOC2)C1	0	Train
TNKS1_ML_3011	Cc1ccc(-n2nnc(N)c2C)cc1C(F)(F)F	0	Train
TNKS1_ML_3012	COc1ccccc1N(C)C(=O)c1cc(N)c(Cl)c(Cl)c1	0	Train
TNKS1_ML_3013	Cc1ccc(C#N)c(Oc2c(C)cc(Br)cc2N)n1	0	Train
TNKS1_ML_3014	C[C@@]1(C(=O)[O-])CCCN(C(=O)[C@H]2C[C@@H]3CCCC[C@@H]3N2)C1	0	Train
TNKS1_ML_3015	CNC(=O)c1nn(C)cc1NC(=O)Cn1nc(C(F)(F)F)c(Br)c1C1CC1	0	Train
TNKS1_ML_3016	O=C(Nc1cccc2cccnc12)c1ccc(N2C(=O)[C@H]3[C@@H]4C=C[C@@H](C4)[C@H]3C2=O)cc1	1	Train
TNKS1_ML_3017	Cc1nn(-c2nc3ccccc3s2)c(O)c1[C@H]1CC(=O)N(c2ccccc2F)C1=O	0	Train
TNKS1_ML_3018	COc1cc(OC)c(N2/C(=[NH+]/C(=O)CCC(=O)[O-])S[C@@H]3CS(=O)(=O)C[C@@H]32)cc1Cl	0	Train
TNKS1_ML_3019	CC(=O)Nc1ccc(-c2csc(NC(=O)[C@H]3COc4ccc(Cl)cc4C3)n2)cc1	0	Train
TNKS1_ML_3020	O=C(NCCN1CCCc2ccccc21)c1n[nH]c2c1COc1ccccc1-2	0	Train
TNKS1_ML_3021	Cc1nsc(Nc2ccc3c(c2)C(=O)N(C)C3=O)c1C#N	0	Train
TNKS1_ML_3022	Cc1nn(C)c(Sc2nccc([O-])n2)c1N	0	Train
TNKS1_ML_3023	COc1ccc([C@H]2S[N-]O[C@@H]2O)cc1	0	Train
TNKS1_ML_3024	N#Cc1cccc2c1C1(CCN(c3nc4c(c(=O)[nH]3)CCCC4)CC1)C(=O)N2	1	Train
TNKS1_ML_3025	Cc1cccc(CN2CCS(=O)(=O)[C@@H]3CCN(C(=O)NCC(C)C)CC[C@@H]32)[nH+]1	0	Train
TNKS1_ML_3026	O=C1CCCc2cc([C@@H](Cl)[C@@H]3CCCCO3)ccc2N1	0	Train
TNKS1_ML_3027	CN(C)c1ccc(-c2nc3c(c(=O)[nH]2)COCC3)cc1	1	Train
TNKS1_ML_3028	CCOC(=O)C1=C(C)N=c2s/c(=C/c3ccc(OC(C)=O)c(OC)c3)c(=O)n2[C@H]1c1ccc(OC(C)=O)c(OC)c1	0	Train
TNKS1_ML_3029	Cc1cc(C)c2cc(C[NH2+]C3CCCC3)c3nn(C)c(=O)n3c2c1	0	Train
TNKS1_ML_3030	Cc1nn(C)c(Sc2cc3ccccc3[nH]2)c1C#N	0	Train
TNKS1_ML_3031	O=C1[C@H](C2CCN(S(=O)(=O)c3cn[nH]c3)CC2)CCCCN1Cc1ccccc1	0	Train
TNKS1_ML_3032	C[C@H]1c2ccsc2CCN1C(=O)c1cc(Br)c[nH]1	0	Train
TNKS1_ML_3033	C[C@H]1CCc2c(sc3nc(CN4CCOCC4)nc(NCc4ccc(-n5ccnc5)nc4)c23)C1	0	Train
TNKS1_ML_3034	Cc1n[nH]c(C)c1C(=O)NC12CC3CC(CC(C3)C1)C2	0	Train
TNKS1_ML_3035	Cc1nn(C)c2ncc(CN[C@@H]3CCC[C@H](O)C3)cc12	0	Train
TNKS1_ML_3036	COc1ccc([C@@H](Nc2cc(C)cc[nH+]2)c2ccc3ccc(C)nc3c2O)cc1OC	0	Train
TNKS1_ML_3037	Cc1[nH]nc(C(C)C)c1Br	0	Train
TNKS1_ML_3038	C[C@@H]1CNC[C@H](C2CCCC2)O1	0	Train
TNKS1_ML_3039	N[C@H]1C=C[C@H](c2nc(C3CCCCCC3)no2)C1	0	Train
TNKS1_ML_3040	C/C=C\[C@H](C(=O)NCS(=O)(=O)c1ccc(C)cc1)N(Cc1ccc(C)o1)C(=O)COC	0	Train
TNKS1_ML_3041	Cc1ccc(-c2cc3cnccc3c(=O)[nH]2)cc1	1	Train
TNKS1_ML_3042	CC(C)(C)OC(=O)N1CCC2(CC1)CCN(C(=O)c1cc(Cc3n[nH]c(=O)c4ccccc34)ccc1F)C2	1	Train
TNKS1_ML_3043	Cc1nc2ccccn2c1C(=O)C1=C(O)C(=O)N(CC[NH+](C)C)[C@@H]1c1cccc(O)c1	0	Train
TNKS1_ML_3044	Nc1cncc(-c2nc3cc(Cl)ccc3[nH]2)c1	0	Train
TNKS1_ML_3045	CCOc1ccc([C@@H]2c3[nH]c4ccccc4c3C[C@@H]3C(=O)N(CCCOC(C)C)CC(=O)N32)cc1OC	0	Train
TNKS1_ML_3046	COc1ccc2c(c1)CC1(CCN(CC(=O)N(Cc3nc4c(c(=O)[nH]3)C	1	Train

	OCC4)CC3CC3)CC1)C2=O		
TNKS1_ML_3047	COC(=O)[C@@H](Cc1ccc[nH+]c1)NC(=O)[C@H]1CN(C(=O)Nc2ccccc2)CCN1S(C)(=O)=O	0	Train
TNKS1_ML_3048	[C-]#[N+]c1c(Cl)cccc1CN1CCC(N2CCC(C(=O)c3ccc(OC)cc3)CC2)C1=O	1	Train
TNKS1_ML_3049	COc1ccc(C(=O)C2CCN(CC(=O)NCc3nc4c(c(=O)[nH]3)CCCCC4)CC2)cc1	1	Train
TNKS1_ML_3050	CC[C@@H](C)Oc1cccc(C(=O)NC(=S)Nc2ccc(S(=O)(=O)N3CC[NH+](C)CC3)cc2)c1	0	Train
TNKS1_ML_3051	CC1(C)C(C(=O)N2CCCC[C@@H]2[C@H]2CCC[C@@H]2O)C1(C)C	0	Train
TNKS1_ML_3052	CCCN1C(=O)[C@]2(C(C(=O)c3ccc(S(=O)(=O)N4CCOCC4)cc3)=C(O)C(=O)N2CC[NH+](C)C)c2ccccc21	0	Train
TNKS1_ML_3053	COc1ccc(C(=O)C2CCN(CC(=O)N(Cc3nc4c(c(=O)[nH]3)CCCC4)CC3CC3)CC2)cc1	1	Train
TNKS1_ML_3054	C#CC(C)(C)NC(=O)N1[C@H]2CC[C@@H]1[C@H](C(=O)[O-])C2	0	Validation
TNKS1_ML_3055	Cc1cccc(-c2nnc([C@H]3C[C@H](NC(=O)c4ccnc5cccnc45)C3)n2-c2ccccc2F)n1	1	Validation
TNKS1_ML_3056	C[C@H](NC(=O)c1ccncc1[O-])c1ccc(Cl)s1	0	Validation
TNKS1_ML_3057	CN(C)c1ccc(NC(=O)Cc2ccc[nH]2)cc1Cl	0	Validation
TNKS1_ML_3058	O=C(Nc1ccccc1C(=O)N1CCN(c2ccccc2)CC1)c1cccc(S(=O)(=O)N2CCCCC2)c1	0	Validation
TNKS1_ML_3059	COC(=O)c1c(C)[nH]c(C(=O)[C@H](C)N2CCC(c3nc4ccccc4s3)CC2)c1C	0	Validation
TNKS1_ML_3060	Cc1nc2ccc(C(=O)N3CCC[C@@H](CNC(=O)c4ccc(F)cc4)C3)cc2nc1C	0	Validation
TNKS1_ML_3061	CCc1nc2sc([C@@H](c3cc(OC)c(OC)c(OC)c3)[NH+]3CCN(c4ccc(F)cc4)CC3)c([O-])n2n1	0	Validation
TNKS1_ML_3062	COc1cc2ncnc(N3CCC(CN4Cc5ccccc5NC4=O)CC3)c2cc1OC	1	Validation
TNKS1_ML_3063	C[C@@H](Oc1ccccc1Cl)C(=O)Nc1nc(-c2ccc(S(=O)(=O)N3CCOCC3)cc2)cs1	0	Validation
TNKS1_ML_3064	COC(=O)c1sc(C(C)(C)C)cc1NC(=O)NC(=O)c1ccccc1Cl	0	Validation
TNKS1_ML_3065	Cc1ccc2c(c1)CCCN2C(=O)c1c(C)n[nH]c1N	0	Validation
TNKS1_ML_3066	C[C@H]1CN(c2cc(F)ccc2[N+](=O)[O-])C[C@@H](C)N1	0	Validation
TNKS1_ML_3067	CC(C)n1cncc1Cn1ccc2cc(C(=O)[O-])ccc21	0	Validation
TNKS1_ML_3068	CC1=NN(C(=O)[C@H](C)n2ccc(C)n2)[C@](O)(C(F)F)C1	0	Validation
TNKS1_ML_3069	Cc1noc(C)c1[C@H](C)/[NH+]=C/c1cc(Cl)cc(Cl)c1[O-]	0	Validation
TNKS1_ML_3070	CC(=O)N1CCc2c(sc(NC(=O)c3ccc(S(=O)(=O)N(C)C[C@@H]4CCCO4)cc3)c2C#N)C1	0	Validation
TNKS1_ML_3071	Cc1cccn2c(C)c(C(=O)C3=C(O)C(=O)N(CCC[NH+]4CCOCC4)[C@@H]3c3cccc(Cl)c3)nc12	0	Validation
TNKS1_ML_3072	C#CCN(CC(=O)Nc1cc(-c2ccc3c(c2)CCC3)nn1-c1ccccc1)[C@H]1CCS(=O)(=O)C1	0	Validation
TNKS1_ML_3073	O=C(NCCNS(=O)(=O)c1ccc(Cl)c2nonc12)C1=C[C@H]2N=CC=C2C=C1	0	Validation
TNKS1_ML_3074	O=[N+]([O-])c1cc(-c2ccc(F)cc2O)ccc1[O-]	0	Validation
TNKS1_ML_3075	CC1CN(C(=O)c2cc(Cc3n[nH]c(=O)c4ccccc34)ccc2F)Cc2nnc(C(F)(F)F)n21	1	Validation
TNKS1_ML_3076	O=C(N[C@H]1C[C@H](c2nnc(-c3ccccn3)n2-c2ccccc2F)C1)c1cccc2nccnc12	1	Validation
TNKS1_ML_3077	COC(=O)C1=C(N)N2C(=O)CCSC2=C(C#N)[C@H]1c1cccc([N+](=O)[O-])c1	0	Validation
TNKS1_ML_3078	CNc1nc(Cl)nc2ncn(C)c12	0	Validation
TNKS1_ML_3079	CC[C@@H]1NC(=O)C[C@@H](C)N(Cc2cnn(C)c2)C1=O	0	Validation

TNKS1_ML_3080	C[C@H](C#N)C1(O)CCC(C)(C)CC1	0	Validation
TNKS1_ML_3081	C[C@@H](C(=O)O[C@H](C)C(=O)Nc1ccccc1C#N)N1C(=O)[C@H]2CC=CC[C@H]2C1=O	0	Validation
TNKS1_ML_3082	Cc1ccc(-c2[nH]nc(NC(=O)[C@@H]3CCn4c[nH+]cc4C3)c2C)o1	0	Validation
TNKS1_ML_3083	O=c1[nH]c(N2CCC3(CC2)CN(CCO)c2cccc(F)c23)nc2c1CCCC2	1	Validation
TNKS1_ML_3084	CCC[C@@H](C)NC(=O)[C@H]1CCCN(C(=O)C(=O)Nc2cccc3c[nH+]ccc23)C1	0	Validation
TNKS1_ML_3085	[O-]/C(=N\c1c(Cl)ccc2nsnc12)c1ccc(Cl)nn1	0	Validation
TNKS1_ML_3086	CC(C)CC(=O)Nc1nnc2n1CCCS2	1	Validation
TNKS1_ML_3087	C[C@@H](NC(=O)N1CCC(CO)CC1)C12CC3CC(CC(C3)C1)C2	0	Validation
TNKS1_ML_3088	O=C(c1cc(-c2ccccc2)n[nH]1)N1CC[NH+](Cc2cc(=O)n3ccsc3n2)CC1	0	Validation
TNKS1_ML_3089	Cc1cnc(C(=O)Nc2sc(C)c(C)c2C#N)cn1	0	Validation
TNKS1_ML_3090	CC(C)(C)c1ccc(C2NC(=O)c3ccccc3N2)cc1	1	Validation
TNKS1_ML_3091	O=c1[nH]nc([S-])n1C[C@H]1CCCOC1	0	Validation
TNKS1_ML_3092	O=C(Nc1cccc(C(F)(F)F)c1)N[C@@H]1[C@H]2CO[C@H](O2)[C@H](N2CCC(Cc3ccccc3)CC2)[C@H]1O	0	Validation
TNKS1_ML_3093	c1ccc(-c2ccc(CN[C@H]3CO[C@@H]4[C@@H](n5nnnc5Oc5ccc(-n6ccnc6)cc5)CO[C@H]34)cc2)cc1	0	Validation
TNKS1_ML_3094	C[C@H]1C[C@H](Nc2ccc3c(c2)CCO3)CCO1	0	Validation
TNKS1_ML_3095	CN(C)S(=O)(=O)c1ccc(SCC(=O)c2c(N)n(Cc3ccccc3)c(=O)n(C)c2=O)nc1	0	Validation
TNKS1_ML_3096	CC(C)(O)c1ccc(-c2nc3ccc(Cl)cc3c(=O)[nH]2)cc1	1	Validation
TNKS1_ML_3097	O=S(=O)(/C=C/c1ccc(Cl)cc1)N1CCN(S(=O)(=O)c2cccc(F)c2)CC1	0	Validation
TNKS1_ML_3098	C1CCCC(c2n[nH]c(C3CCCC3)n2)CCC1	0	Validation
TNKS1_ML_3099	Cc1nc(N)c2c(C)c(C)n([C@H](C)C(C)C)c2n1	0	Validation
TNKS1_ML_3100	COc1ccc(F)cc1S(=O)(=O)N1CCC2(CC1)OCCN2S(=O)(=O)c1ccc(F)c(C)c1	0	Validation
TNKS1_ML_3101	CC(C)NC(=O)[C@@H](C)NC(=O)[C@H]1CCCN(S(=O)(=O)c2cccc3nsnc23)C1	0	Validation
TNKS1_ML_3102	CN(c1ccc(C(=O)Nc2ccccc2C(=O)N2CCOCC2)c(Cl)c1)S(C)(=O)=O	0	Validation
TNKS1_ML_3103	CO[C@H]1CCC[C@H]1N1CC(=O)NC1=O	0	Validation
TNKS1_ML_3104	[NH3+][C@H](Cc1ccc(Br)cc1)Cc1cccc([N+](=O)[O-])c1	0	Validation
TNKS1_ML_3105	O=C(Nc1ccc(F)cc1)c1ccc(NC(=O)N2CCN(C(=O)c3cc(Cc4n[nH]c(=O)c5ccccc45)ccc3F)CC2)cc1	1	Validation
TNKS1_ML_3106	Cc1cc(N)ccc1-c1nnnn1-c1cc(Br)ccc1F	0	Validation
TNKS1_ML_3107	COc1cc(/C=N/NC(=O)c2ccn(Cc3ccc(Br)cc3)n2)ccc1O	0	Validation
TNKS1_ML_3108	CCCCN1C(=O)[C@]2(C(C(=O)c3cccc(S(=O)(=O)N(C)C)c3)=C(O)C(=O)N2CC[NH+]2CCOCC2)c2ccccc21	0	Validation
TNKS1_ML_3109	Cc1[nH+]n(C)c(C)c1CCN(C)c1[nH+]cc(NC(=O)c2ccc(Br)cc2)cc1C(=O)[O-]	0	Validation
TNKS1_ML_3110	Cc1ccc([O-])c(C[NH+]2CCN([C@H](C)C(=O)N3CCOCC3)CC2)[nH+]1	0	Validation
TNKS1_ML_3111	[H]/N=C1\S[C@@H](C(=O)Nc2ccc(Br)cc2)CC(=O)N1/N=C\c1ccccc1	0	Validation
TNKS1_ML_3112	O=[N+]([O-])c1cnc(N[C@H]2CCNC2)c(Br)c1	0	Validation
TNKS1_ML_3113	CC1=C(C)C[C@@H](C(=O)Nc2sc3c(c2C(=O)N2CCOCC2)CCCC3)[C@@H](C(=O)O)C1	0	Validation
TNKS1_ML_3114	CCc1c[nH+]c2c(c1NOCC(=O)N1CCN(c3ccccc3)CC1)c(=O)n(C)c(=O)n2C	0	Validation
TNKS1_ML_3115	C[C@]1(c2ccc3ccccc3c2)NC(=O)N(CC(=O)C2=CC(C(=O)	0	Validation

	N3CCCC3)=NC2)C1=O		
TNKS1_ML_3116	O=C(CS(=O)(=O)c1cn(CC(=O)N2CCOCC2)c2ccccc12)Nc1cccc(Cl)c1	0	Validation
TNKS1_ML_3117	COC(=O)c1cc(N)c(N2CCCC[C@H]2C)c(Cl)c1	0	Validation
TNKS1_ML_3118	CCc1cccc(N2C[C@@H](C(=O)NNC(=O)c3sc(N4CCOCC4)nc3C)CC2=O)c1	0	Validation
TNKS1_ML_3119	c1cncc(-c2nnc(C3CCCC3)o2)c1	0	Validation
TNKS1_ML_3120	Cc1nc(C(=O)Nc2ccccc2C(=O)NCc2ccco2)nn1-c1ccc(F)cc1	0	Validation
TNKS1_ML_3121	O=C1c2nccnc2N[C@H](SCc2ccc(F)cc2)N1CCC1=c2ccccc2=[NH+]C1	0	Validation
TNKS1_ML_3122	N#Cc1cc(N)cnc1NCC1(O)CCCCC1	0	Validation
TNKS1_ML_3123	CNC(=O)[C@H](C1CCCC1)N1CCN(C(=O)Nc2cccc(F)c2)CC1	0	Validation
TNKS1_ML_3124	Cc1ccc(NC(=S)NCc2ccc(Cl)cc2)c([N+](=O)[O-])c1	0	Validation
TNKS1_ML_3125	CC(C)(C)C1CCN(c2nc3c(s2)[C@@H](O)CCC3)CC1	0	Validation
TNKS1_ML_3126	COc1cc(NS(=O)(=O)c2ccc(F)cc2)cc2sc(NC(=O)c3ccccc3)nc12	0	Validation
TNKS1_ML_3127	COC(=O)C[C@H](N[C@H]1C2=C(CCCC2)C(=O)N1Cc1ccc2c(c1)OCO2)c1ccc(F)c(Oc2ccccc2)c1	0	Validation
TNKS1_ML_3128	Cc1nonc1C[NH+]1[C@H]2CCCC[C@@H]2C[C@@H]1C(=O)[O-]	0	Validation
TNKS1_ML_3129	[H]/N=C1C(=C/c2ccc(OCc3ccc(C)cc3)c(Cl)c2)/C(=O)N=C2SC(S(C)(=O)=O)=NN2/1	0	Validation
TNKS1_ML_3130	N[C@]1(c2nc(-c3cc(F)cc(F)c3)no2)CCOC1	0	Validation
TNKS1_ML_3131	CN(C)CCn1c2c3c4c(cccc41)C(=O)NN=C3CC(C)(C)C2	1	Validation
TNKS1_ML_3132	N#C[C@@]1(N2CCCCC2)CCC[C@@H](C2CC2)C1	0	Validation
TNKS1_ML_3133	CCC[C@H]1CC(=O)O[C@]1(C)C(=O)C[n+]1cc(Br)cc(Br)c1	0	Validation
TNKS1_ML_3134	Cc1cc(NCCc2ccc(O)cc2)c2nncn2n1	1	Validation
TNKS1_ML_3135	CCOC(=O)N1CCN(C(=O)Cc2csc(N)n2)CC1	0	Validation
TNKS1_ML_3136	C[C@H](NC(=O)c1cc(F)ccc1[N+](=O)[O-])c1cccs1	0	Validation
TNKS1_ML_3137	O=C(NCC[NH+]1CCN(C(=O)CCS(=O)(=O)c2ccccc2)CC1)c1ccc2c(c1)OCO2	0	Validation
TNKS1_ML_3138	Cc1cc2nc(C)c(CCC(=O)Nc3cccc(C(=O)N4CCOCC4)c3C)c(C)n2n1	0	Validation
TNKS1_ML_3139	COc1cc2c(=O)c(-c3ccc4c(c3)OCO4)coc2cc1O[C@@H]1O[C@H](CO[C@H]2OC[C@](O)(CO)[C@H]2O)[C@@H](O)[C@H](O)[C@H]1O	0	Validation
TNKS1_ML_3140	C[C@@H](Sc1ccc(S(=O)(=O)N2CCCC2)cn1)C(=O)Nc1cccc(S(N)(=O)=O)c1	0	Validation
TNKS1_ML_3141	Nc1nc(Cl)nc2c1ncn2Cc1ccccn1	0	Validation
TNKS1_ML_3142	C[C@H]1CCCCCN1c1ccc(S(N)(=O)=O)c(N)c1	0	Validation
TNKS1_ML_3143	COc1ccc(NC(=O)CS[C@H]2N=C3N=NC=C3C(=O)N2c2ccc(F)c(Cl)c2)cc1OC	0	Validation
TNKS1_ML_3144	CCOc1cccc2cc(C(=O)N3CCCc4c(C)cccc43)c(=O)oc12	0	Validation
TNKS1_ML_3145	COc1ccc(-n2c(NCc3nc(-c4ccc(C)cc4)no3)nnc2-c2ccncc2)cc1	1	Validation
TNKS1_ML_3146	COCCCNc1nc2c(C)cccn2c(=O)c1/C=C1\SC(=S)N(Cc2ccc3c(c2)OCO3)C1=O	0	Validation
TNKS1_ML_3147	CSc1ccc(NC(=O)C2CCN(S(=O)(=O)[C@@H]3C=C[C@H]([C@@H]4C=CN=N4)O3)CC2)cc1	0	Validation
TNKS1_ML_3148	C[C@@H]1CN(C(=O)c2ccc(Cl)c(S(=O)(=O)NCc3ccco3)c2)C[C@@H](C)O1	0	Validation
TNKS1_ML_3149	CCOc1cc2[nH]c(=S)n(CCC[NH+]3CCN(c4cccc(Cl)c4)CC3)c(=O)c2cc1OCC	0	Validation
TNKS1_ML_3150	Cc1ccc(NC(=O)C2CCN(S(=O)(=O)c3ccc4c(c3)CCC4)CC2)c(O)c1	0	Validation

TNKS1_ML_3151	Cc1cc(C)c(NC(=O)c2cnc(N3CCCCC3)nc2C)c(C)c1	0	Validation
TNKS1_ML_3152	COc1ccc(OC)c(NC(=O)[C@H]2c3cc(OC)c(OC)cc3C(=O)N(C)[C@H]2c2ccc[nH+]c2)c1	0	Validation
TNKS1_ML_3153	CC/C=c1\[nH]n2c(=[NH2+])/c(=C/c3ccc(OS(=O)(=O)c4ccccc4)c(OC)c3)c(=O)nc-2s1	0	Validation
TNKS1_ML_3154	COc1ccc(C(=O)C2CCN([C@@H]3CCN(CC(=O)Nc4nnc5n4CCS5)C3=O)CC2)cc1	1	Validation
TNKS1_ML_3155	COC(=O)c1c(NC(=O)CSc2cc(C)nc(C)n2)sc2c1CC[C@@H](C)C2	0	Validation
TNKS1_ML_3156	Cc1cc(F)cc2c(=O)[nH]c(-c3ccc(C(C)(C)O)cc3)nc12	1	Validation
TNKS1_ML_3157	Cc1cc(NC(=O)c2cc3[nH+]c(N(C)C)n(-c4ccc(Br)cc4)c3s2)n(C)n1	0	Validation
TNKS1_ML_3158	CC[C@]12C=CCN3CCc4c(n(c5ccccc45)[C@H](O)C1)[C@@H]32	0	Validation
TNKS1_ML_3159	C[C@H]1O[C@H](C)[C@H](C(=O)NC(C)(C)C)[C@H]1C	0	Validation
TNKS1_ML_3160	O=C(Nc1c(C(=O)Nc2ccccc2F)oc2ccc[nH+]c12)c1ccc(F)c(F)c1	0	Validation
TNKS1_ML_3161	CCOc1ccc(-c2nnc(C34CC(NC(=O)c5ccnc6cccnc56)(C3)C4)n2-c2cccnc2)nc1	1	Validation
TNKS1_ML_3162	C[C@H](C(=O)Nc1ccc(N2CCOCC2)cc1)N1CCN(Cc2ccc3c(c2)OCO3)CC1	0	Validation
TNKS1_ML_3163	N#Cc1cc(CCl)nc2ccccc12	0	Validation
TNKS1_ML_3164	Cc1cc(Cl)cc(-c2nnc(CN)s2)c1	0	Validation
TNKS1_ML_3165	Cc1cccc(OCCn2cc(/C=C3/C(=O)N=C([O-])N(Cc4ccco4)C3=O)c3ccccc32)c1	0	Validation
TNKS1_ML_3166	c1ccc2c(c1)CN[C@@H](c1nncn1C1CCCCC1)C2	0	Validation
TNKS1_ML_3167	Cc1cnc2nc(-c3snnc3C)[nH]c2c1	0	Validation
TNKS1_ML_3168	Cn1c(-c2cc(Cl)ccc2F)[nH+]c2cc(C(=O)[O-])ccc21	0	Validation
TNKS1_ML_3169	COC(=O)c1sc2nc(CN3CCN(c4cccc[nH+]4)CC3)ccc2c1NC(=O)c1ccsc1	0	Validation
TNKS1_ML_3170	COc1ccccc1/C=C(C(\[O-])=N\S(=O)(=O)c1ccc[nH+]c1)/c1ccccc1	0	Validation
TNKS1_ML_3171	Cc1ccc2nsnc2c1NC(=O)c1ccc(C#N)cc1F	0	Validation
TNKS1_ML_3172	Cc1cc(N)sc1-c1nnc2n1CCCCC2	0	Validation
TNKS1_ML_3173	O=C(NC1CCCC1)c1cc2c(ccc3ccccc32)o1	0	Validation
TNKS1_ML_3174	COc1cc2c(cc1F)[nH]c(=S)n2[C@@H]1CCCCC[C@H]1C	0	Validation
TNKS1_ML_3175	C[C@H]1[C@@H](C2CC2)[NH2+]CC[C@@H](C)S1(=O)=O	0	Validation
TNKS1_ML_3176	N#Cc1ccccc1S(=O)(=O)N1CCC(C(=O)NNC(=O)c2ccccc2-n2cccc2)CC1	0	Validation
TNKS1_ML_3177	O=C(c1ccc(F)cc1)C1CCN(CC(=O)N(Cc2nc3c(c(=O)[nH]2)COCC3)Cc2ccc(S(=O)(=O)N3CCOCC3)s2)CC1	1	Validation
TNKS1_ML_3178	Cc1nc(Cc2nnc(SCc3nnc(-c4cc5c(s4)CC[C@H](C(C)(C)C)C5)o3)o2)cs1	0	Validation
TNKS1_ML_3179	Cc1ccc(C(=O)N2CCC[C@@H]([C@H]3CCC[NH2+]3)C2)c([O-])c1	0	Validation
TNKS1_ML_3180	CC1(C)C(Nc2nccn3cnnc23)C1(C)C	0	Validation
TNKS1_ML_3181	Nc1sc2c(c1-c1nc([C@@H]3CCCOC3)no1)CCCC2	0	Validation
TNKS1_ML_3182	CC/C=C(\C)C(=O)N1CCC[C@H](S(N)(=O)=O)C1	0	Validation
TNKS1_ML_3183	O=[N+]([O-])c1ccc(/N=C(/C#Cc2ccccc2)c2ccccc2)cc1	0	Validation
TNKS1_ML_3184	O=C1SCCc2nc(-c3ccc(C(F)(F)F)cc3)[nH]c(=O)c21	1	Validation
TNKS1_ML_3185	Cc1cc(N2CC(=O)NC3(CCCC3)C2=O)ccc1Br	0	Validation
TNKS1_ML_3186	Cc1ccoc1[C@@H](Br)c1ccc2c(c1)CC(=O)N2	0	Validation
TNKS1_ML_3187	O=C1CC2(CCN(C(=O)CN3CCN(CCSc4nc5ccccc5c(=O)[nH]4)CC3)CC2)Oc2ccccc21	1	Validation
TNKS1_ML_3188	C/C(=N\O)c1c([O-])cc(C(C)C)n(-c2ccccc2)c1=O	0	Validation

TNKS1_ML_3189	C[C@H](C#N)NC1C[C@@H]2CCC[C@H](C1)[NH+]2C	0	Validation
TNKS1_ML_3190	CC[C@@H](C#N)Nc1c(Cl)cc(Br)cc1Cl	0	Validation
TNKS1_ML_3191	CC(C)(C)CC(=O)N1CCC2(CC1)OC[C@H](C(=O)NCc1ccc[nH+]c1)N2C(=O)c1ccccc1	0	Validation
TNKS1_ML_3192	O=c1[nH]c(-c2ccc(Br)cc2)nc2ccccc12	1	Validation
TNKS1_ML_3193	C[C@H](C1CC1)N(Cc1nc2c(c(=O)[nH]1)COCC2)C(=O)CN1CCC(C(=O)c2ccc(F)cc2)CC1	1	Validation
TNKS1_ML_3194	C=C1C[C@@H]2[C@]3(C)C(=C(O)C(=O)[C@@]2(C)[C@]2(C(=O)OC)C(=O)[C@H](C)OC(=O)[C@@]12C)C(C)(C)C(=O)C[C@H]3O	0	Validation
TNKS1_ML_3195	Cc1sc2ncn(CC(=O)NCCC[NH+]3C[C@@H](C)C[C@H](C)C3)c(=O)c2c1S(=O)(=O)N1CCC(C)CC1	0	Validation
TNKS1_ML_3196	Cc1ccc2c(c1)C1=Nc3[nH+]cnn3[C@H](c3cccnc3)[C@@H]1[C@H](c1cccs1)O2	0	Validation
TNKS1_ML_3197	O=C(CN(c1ccc(Br)cc1)S(=O)(=O)c1ccccc1)Nc1ccccc1NS(=O)(=O)c1ccccc1	0	Validation
TNKS1_ML_3198	COc1cc(S(=O)(=O)[N-]c2cccc(C)c2)ccc1N/C=C1\C(=O)N(c2ccccc2)N=C1C	0	Validation
TNKS1_ML_3199	CC(=O)c1cc2c(cc1NC(=O)c1nc(-c3cccs3)n(-c3ccccc3)n1)OCO2	0	Validation
TNKS1_ML_3200	C[C@]1(NC(=O)c2cc(-c3ccco3)n(-c3ccc(Cl)cc3)n2)CCS(=O)(=O)C1	0	Validation
TNKS1_ML_3201	O=S(=O)(c1ccc2[nH]cnc2c1)C(F)(F)F	0	Validation
TNKS1_ML_3202	Cc1nc(-c2ccc(NC3CCC(C)(C)CC3)cc2)co1	0	Validation
TNKS1_ML_3203	CC1(C)O[C@@H]2O[C@@H](C(=O)NCc3nc4ccccc4n3Cc3ccccc3)[C@@H]3OC(C)(C)O[C@@H]3[C@@H]2O1	0	Validation
TNKS1_ML_3204	CCCOc1ccc(S(=O)(=O)N2CCC3(CC2)OCCN3S(=O)(=O)c2ccc(Cl)cc2)cc1C	0	Validation
TNKS1_ML_3205	C[C@H]1CC[C@H](NC(=O)NC2CCC(C(=O)[O-])CC2)[C@@H]1C	0	Validation
TNKS1_ML_3206	O=C([C@@H]1CN(CCc2c[nH]c3ccccc23)CCN1C(=O)c1ccco1)N1CCOCC1	0	Validation
TNKS1_ML_3207	CC[NH+](CCNC(=O)c1sc2ncnc(N3CCN(C)CC3)c2c1C)Cc1ccccc1	0	Validation
TNKS1_ML_3208	CO[C@H]1O[C@H]2CC[C@H](c3ccccc3)O[C@@H]2[C@H](OS(=O)(=O)c2ccc(C)cc2)[C@H]1OS(=O)(=O)c1ccc(C)cc1	0	Validation
TNKS1_ML_3209	O=C(c1ccc(F)cc1)C1CCN(CC(=O)N(Cc2cccnc2)Cc2nc3c(c(=O)[nH]2)COCC3)CC1	1	Validation
TNKS1_ML_3210	[H]/N=C1C(=C/c2ccc(OCc3ccc([N+](=O)[O-])cc3)c(OC)c2)/C(=O)N=C2SC(C)=NN2/1	0	Validation
TNKS1_ML_3211	CC1(C)[C@H]2C[C@@](C)(C(=O)O)CC[C@H]21	0	Validation
TNKS1_ML_3212	Cc1noc(C)c1CCCNC(=O)N1C[C@H]2CC[C@@H]1C2	0	Validation
TNKS1_ML_3213	Cc1cc(S(=O)(=O)N2CC(c3nc(-c4ccc[nH]c4=O)no3)C2)c(Cl)cc1Cl	0	Validation
TNKS1_ML_3214	O=c1[nH]c(-c2ccc(C(F)(F)F)cc2)nc2c1CCCC2	1	Validation
TNKS1_ML_3215	COCCn1nc(C(=O)NCc2ccc[nH+]c2)c2c1CCN(C(=O)CCc1ccccc1Cl)C2	0	Validation
TNKS1_ML_3216	Cc1ccc(S(=O)(=O)N2CC[NH+](Cc3nc4cc(C(=O)N5CCCCC5)ccc4o3)CC2)cc1C	0	Validation
TNKS1_ML_3217	O=Cc1c(Oc2ccccc2Cl)nc2ccccn2c1=O	0	Validation
TNKS1_ML_3218	Cc1cc2c(cc1C)S(=O)(=O)[C@H](C)[C@@H](C)[C@@H]2NC(C)C	0	Validation
TNKS1_ML_3219	Cc1cc(NC(=O)[C@H](c2ccccc2)N2CCC(C(=O)c3ccc4c(c3)OCCO4)CC2)no1	0	Validation
TNKS1_ML_3220	CC(C)C1(O)CN(C(=O)Nc2cccc3ccccc23)C1	0	Validation
TNKS1_ML_3221	NCc1c(Oc2cc(Cl)ccc2Cl)nc2ccccn12	0	Validation
TNKS1_ML_3222	C[C@@H]1CCc2ccccc2N1c1ncc(N)cn1	0	Validation

TNKS1_ML_3223	COc1cc2c(cc1F)[nH]c(=S)n2[C@@H]1CCCC1(C)C	0	Validation
TNKS1_ML_3224	Cc1cc(C)c(-c2nnc3n2C[C@@H](CO)CC3)c(C)c1	0	Validation
TNKS1_ML_3225	COc1cccc(C/N=C(\O)[C@@H]2Cc3cc(S(=O)(=O)N4CCCC4)ccc3N2C(C)=O)c1OC	0	Validation
TNKS1_ML_3226	CCOc1ccc(-c2nnc([C@H]3C[C@H](NC(=O)c4ccnc5cc(F)cnc45)C3)n2-c2ccccn2)nc1	1	Validation
TNKS1_ML_3227	CN1CCN(c2cc(F)c3c(c2)N(C)C(=O)C32CCN(c3nc4c(c(=O)[nH]3)CCCN4C)CC2)CC1	1	Validation
TNKS1_ML_3228	Cn1c(=O)c2nc(SCCC(=O)Nc3ccc(Cl)cc3F)n(C)c2n(C)c1=O	0	Validation
TNKS1_ML_3229	CC(C)(C)[C@H]1CCc2onc(CO)c2C1	0	Validation
TNKS1_ML_3230	CCc1c(C)nc(N2CCN(C)CC2)nc1NC	0	Validation
TNKS1_ML_3231	CN1CCN(CCOc2cc(F)c3c(c2)N(C)C(=O)C32CCN(c3nc4c(c(=O)[nH]3)CCCN4C)CC2)CC1	1	Validation
TNKS1_ML_3232	O=C(C1=C([O-])C(=O)N(c2nc3ccc(F)cc3s2)[C@@H]1c1cccnc1)c1cc2ccccc2o1	0	Validation
TNKS1_ML_3233	CC(C)N(Cc1nc2c(c(=O)[nH]1)COCC2)C(=O)CCc1ccccc1	1	Validation
TNKS1_ML_3234	Cc1ccc2[nH]c3c(N4CCSCC4)ncnc3c2c1	0	Validation
TNKS1_ML_3235	C[C@H]1CCCc2sc3ncn4nc(-c5cccc(O)c5)nc4c3c21	0	Validation
TNKS1_ML_3236	CCOc1ccc(-c2nnc(C34CC(NC(=O)c5ccnc6cccnc56)(C3)C4)n2-c2ccncc2)nc1	1	Validation
TNKS1_ML_3237	O=C(Cc1ccc(NC2=[NH+][C@H]3CS(=O)(=O)C[C@H]3S2)cc1)N1CC[NH+](Cc2ccccc2)CC1	0	Validation
TNKS1_ML_3238	N#C[C@H]1CCO[C@H]1c1cc2ccccc2o1	0	Validation
TNKS1_ML_3239	CC(=O)N[C@H](Cc1ccccc1F)C(=O)N1CC[NH+](Cc2ccc(-c3noc(-c4ccco4)n3)cc2)CC1	0	Validation
TNKS1_ML_3240	COc1ccc(-c2cc(=O)c3ccc(O)cc3o2)cc1	1	Validation
TNKS1_ML_3241	O=C(N=c1[nH]cc[nH]1)c1ccc(CCl)cc1	0	Validation
TNKS1_ML_3242	C[C@@H]1CO[C@@](C)(c2ccccc2)O1	0	Validation
TNKS1_ML_3243	Oc1nc(-c2ccc(C(F)(F)F)cc2)nc2c1CSCC2	1	Validation
TNKS1_ML_3244	Cc1ccc2sc3nncn3c2c1	1	Validation
TNKS1_ML_3245	C[C@H]1CNC[C@H](c2ccc(C(C)(C)C)cc2)O1	0	Validation
TNKS1_ML_3246	CC1(C)[C@H]2CC[C@](C)(C2)[C@@H]1Nc1ccc(C(=O)[O-])nn1	0	Validation
TNKS1_ML_3247	C[C@@H](c1ccccc1)N(Cc1nc2c(c(=O)[nH]1)COCC2)C(=O)CN1CCC(C(=O)c2ccc(F)cc2)CC1	1	Validation
TNKS1_ML_3248	CC1(C)CCC[C@H]1NC(=O)c1cc(F)c(F)cc1[N+](=O)[O-]	0	Validation
TNKS1_ML_3249	COc1cc(/C=C2\SC([N-]c3ccc(O)cc3)=NC2=O)ccc1OCc1ccc(Cl)cc1	0	Validation
TNKS1_ML_3250	Cc1nnc(SCCn2c(N3CCC(C(N)=O)CC3)[nH+]c3c2c(=O)n(C)c(=O)n3C)s1	0	Validation
TNKS1_ML_3251	CCC[C@@H]1CCc2nc(NC(=O)C3CCN(C(=O)N4CCCC4)CC3)sc2C1	0	Validation
TNKS1_ML_3252	CC[C@@H](CNC(=O)N1CCC[C@@H](C)[C@@H]1C)C(=O)[O-]	0	Validation
TNKS1_ML_3253	Cn1c(CN2C[C@@H]3C[C@H](C2)Cn2c3cccc2=O)cc(=O)c(O)c1CN1CCN(CCOc2ccccc2Cl)CC1	0	Validation
TNKS1_ML_3254	CN1C(=O)C[C@H](C(=O)O)C12CC[NH+](Cc1ccc(Cl)s1)CC2	0	Validation
TNKS1_ML_3255	Cc1ccc2c(CSc3nc4sc(C)c(C)c4c(=O)n3C)cc(=O)oc2c1	0	Validation
TNKS1_ML_3256	O[C@@H]1CCC[C@@H]1Nc1ccc2c(c1)OCCO2	0	Validation
TNKS1_ML_3257	O=C1N[C@H](CC2=c3ccccc3=[NH+]C2)C(=O)N1CC(=O)N(C1CCCC1)[C@@H]1CCS(=O)(=O)C1	0	Validation
TNKS1_ML_3258	C[C@@H](CN1CC(C(=O)N2CCN(c3ncc(C(F)(F)F)cn3)CC2)C1)Nc1cn[nH]c(=O)c1C(F)(F)F	1	Validation
TNKS1_ML_3259	N#Cc1ccccc1S(=O)(=O)N1CCN(CC(=O)N[C@H]2CC[NH+	0	Validation

	](Cc3ccccc3)C2)CC1		
TNKS1_ML_3260	COc1cccc2c(=O)[nH]c(-c3ccc(C(C)(C)O)cc3)nc12	1	Validation
TNKS1_ML_3261	CCOC(=O)c1c(NC(=O)c2cc3n(n2)[C@H](C(F)(F)F)C[C@@H](c2ccc4c(c2)OCO4)N3)sc2c1CCCC2	0	Validation
TNKS1_ML_3262	Oc1ccc2oc(-c3ccccc3Br)nc2c1	0	Validation
TNKS1_ML_3263	CC(C)(C)OC(=O)NCC(=O)/[NH+]=C1/S[C@H]2CS(=O)(=O)C[C@H]2N1CCc1ccc(F)cc1	0	Validation
TNKS1_ML_3264	C[C@H]1CN(c2[nH]c(=O)n(C)c(=O)c2Cc2ccc(Br)cc2)C[C@H](C)O1	0	Validation
TNKS1_ML_3265	COC(=O)c1c(OCCCc2ccc[nH+]c2)cc(=O)n2c1CCN(Cc1c[nH]c3ccccc13)CC2	0	Validation
TNKS1_ML_3266	Cc1cccc2c(=O)[nH]c(-c3ccc(Cl)cc3)nc12	1	Validation
TNKS1_ML_3267	COc1cc(/C=C2\SC(=S)N(CCC(=O)Nc3ccc(F)cc3)C2=O)cc(OC)c1OC	0	Validation
TNKS1_ML_3268	O=C(NCCN1C(=O)S/C(=C\c2cccs2)C1=O)c1ccc(N2CCOCC2)c([N+](=O)[O-])c1	0	Validation
TNKS1_ML_3269	CN1CCCc2c1cc(-c1ccc(Cl)cc1)[nH]c2=O	1	Validation
TNKS1_ML_3270	CC[NH+]1CCN(c2nc3ccccn3c(=O)c2/C=C(/C#N)c2nc3ccccc3[nH]2)CC1	0	Validation
TNKS1_ML_3271	C[C@@H](Sc1nnc(C/[NH+]=C(\O)c2ccccc2F)n1C)/C(O)=N/c1nccs1	0	Validation
TNKS1_ML_3272	COC[C@@H](C)NC(=O)c1oc2c(c1C)/C(=N/NC(=O)c1ccc(Cl)cc1)CCC2	0	Validation
TNKS1_ML_3273	Cc1cccc([N+](=O)[O-])c1NC(=O)COC(=O)c1cc2c(C)nn(-c3ccccc3)c2s1	0	Validation
TNKS1_ML_3274	Cn1cc[nH+]c1[C@@H](NC(=O)C1CCN(S(=O)(=O)c2ccccc2)CC1)c1ccccc1F	0	Validation
TNKS1_ML_3275	Cc1ccc2c(CN3C(=O)N[C@](C)(c4ccc(C#N)cc4)C3=O)cc(=O)oc2c1C	0	Validation
TNKS1_ML_3276	Cc1cccc2sc(-c3csc(N)n3)nc12	0	Validation
TNKS1_ML_3277	CCn1c2ccccc2c2cc([C@H]3Nc4c(O)ccc([N+](=O)[O-])c4[C@@H]4C=CC[C@@H]34)ccc21	0	Validation
TNKS1_ML_3278	Cc1c(/C=C2\SC(=S)N([C@H](C)c3ccccc3)C2=O)c(N2CC[NH+](CCO)CC2)n(C)c(=O)c1C#N	0	Validation
TNKS1_ML_3279	O=C(c1cccc(N2CC[NH2+][C@@H](c3ccccc3)C2)n1)N1CCN(c2cccc[nH+]2)CC1	0	Validation
TNKS1_ML_3280	Cc1ccc(S(=O)(=O)N2CCCC[C@@H]2C(=O)Nc2c[nH+]c3ccccc3c2)cc1	0	Validation
TNKS1_ML_3281	Cc1nn(C)c2nc(-c3ccco3)cc(C(=O)Nc3cc(Cl)ccc3N3CCOCC3)c12	0	Validation
TNKS1_ML_3282	Cc1nc(N)sc1-c1cc([C@@H]2CC=CCC2)c(C#N)c(N)n1	0	Validation
TNKS1_ML_3283	O=C(Nc1ccc(-n2cc[nH+]c2)nc1)c1ccc(Cl)cc1Cl	0	Validation
TNKS1_ML_3284	C[C@@]1(Cn2nnnc2-c2ccccc2N)CCCO1	0	Validation
TNKS1_ML_3285	O=C1NC(=O)[C@](Cc2cccc3ccccc23)(CN2C[C@@H]3C[C@H](C2)Cn2c3cccc2=O)C(=O)N1Cc1ccccc1	0	Validation
TNKS1_ML_3286	COc1ccc(C(=O)C2CCN(C3CCN(Cc4nc5sc(C)cc5c(=O)[nH]4)C3=O)CC2)cc1	1	Validation
TNKS1_ML_3287	Cc1cc(C(=O)Nc2ccc3nc(N4CCOCC4)sc3c2)c2nc3n(c2n1)CCCCC3	0	Validation
TNKS1_ML_3288	Cc1c(C(=O)N[C@@H](C)c2cccc(N3CCCC3=O)c2)nnn1-c1cccc2[nH+]cccc12	0	Validation
TNKS1_ML_3289	Fc1ccc(-c2[nH]ncc2Cn2ccnc2-c2cc3n(n2)CCNC3)cc1	0	Validation
TNKS1_ML_3290	C[C@H]1[C@@H](Cl)CC[C@@H]1C[C@@H]1Cc2cc(F)ccc2O1	0	Validation
TNKS1_ML_3291	COC(CNC(=O)c1ccc2c(c1)N(Cc1ccc(Cl)cc1)C(=O)c1ccccc1S2(=O)=O)OC	0	Validation
TNKS1_ML_3292	COc1ccc(C2NC(=O)c3ccccc3N2)cc1	1	Validation
TNKS1_ML_3293	CC(C)(C)c1ccc(C(=O)Nc2nnc3n2CCS3)cc1	1	Validation

TNKS1_ML_3294	CC[C@H]1CC[C@@H](O)[C@H](Oc2ccc3c(c2)OCO3)C1	0	Validation
TNKS1_ML_3295	C[C@@H]1CCC[C@@](C#N)(C2(O)CCC(C(C)(C)C)CC2)C1	0	Validation
TNKS1_ML_3296	O=C([O-])[C@@H]1C[C@H]2CC[C@@H]1[NH+]2c1c(F)c(F)c(F)c(F)c1F	0	Validation
TNKS1_ML_3297	O=S(=O)(c1ccc(Br)cc1)c1nc(-c2ccc(F)cc2)oc1NCCCn1cc[nH+]c1	0	Validation
TNKS1_ML_3298	CCC[C@H](NC(=O)NC1(c2ccccc2F)CC1)c1nnc2ccccn12	0	Validation
TNKS1_ML_3299	CCc1nc(-c2cnn(-c3ccccc3)c2)nc(N)c1Br	0	Validation
TNKS1_ML_3300	C[C@H]1CC[C@H](C)N([C@@H]2CCC[C@@H]2C#N)C1	0	Validation
TNKS1_ML_3301	O=c1[nH]c(N2CCC3(CC2)CNc2ccccc23)nc2c1CCCC2	1	Validation
TNKS1_ML_3302	C[C@@]1(CCc2ccc3c(c2)OCO3)NC(=O)NC1=O	0	Validation
TNKS1_ML_3303	CCOC(=O)c1c(C)sc(C2=C3C(=O)N(C)C(=O)N(C)[C@@H]3N=C2c2ccc(OC)c(F)c2)c1O	0	Validation
TNKS1_ML_3304	Cc1cccc(C(=O)N[C@@H](C(=O)N2CCN(C(=O)c3ccccc3)CC2)S(=O)(=O)c2ccccc2)c1	0	Validation
TNKS1_ML_3305	Cc1ccc2c(=O)[nH]c(-c3ccc(C(C)(C)O)cc3)cn12	1	Validation
TNKS1_ML_3306	O=C(CN1CCC2(CCc3ccccc3C2=O)CC1)NCc1nc2c(c(=O)[nH]1)COCC2	1	Validation
TNKS1_ML_3307	CCC(Br)(CC)C(=O)NC(=O)NC(C)=O	0	Validation
TNKS1_ML_3308	ClCc1coc(-c2cc3c(s2)CCC3)n1	0	Validation
TNKS1_ML_3309	CN[C@@H](C)c1nnn(-c2cccc(C#N)c2)c1C	0	Validation
TNKS1_ML_3310	C[C@H]1CCCCC[C@@H]1N1CCCN2CCC[C@@H]2C1	0	Validation
TNKS1_ML_3311	O=C(Nc1c[nH]c2ccccc12)[C@H]1COc2ccccc2C1	0	Validation
TNKS1_ML_3312	CN(C(=O)N[C@@H]1CC[C@H]([NH+](C)C)C1)[C@@H]1CCCc2c1cnn2C	0	Validation
TNKS1_ML_3313	Cc1ccc(-c2ccc(-n3nc(C)c(S(=O)(=O)NCC4CC4)c3C)nn2)cc1C	0	Validation
TNKS1_ML_3314	CC(C)[C@H]1CC(O)=Nc2c1c(=O)[nH]n2C1CCCCC1	0	Validation
TNKS1_ML_3315	Cc1cc2c(N)c3ccc(N(C)C)cc3nc2cn1	0	Validation
TNKS1_ML_3316	CCc1ccc(-c2cc(C(=O)NCc3cccc[nH+]3)c3c(C)nn([C@@H]4CCS(=O)(=O)C4)c3n2)cc1	0	Validation
TNKS1_ML_3317	C[C@H]1C[C@@H](C)CC(O)(C2(C#N)CCCCC2)C1	0	Validation
TNKS1_ML_3318	CN[C@H]1CCCN(c2c(Cl)ccc3nsnc23)C1	0	Validation
TNKS1_ML_3319	Cc1cnc2[nH]c([C@@H]3CC[C@H](C)O3)nc2c1	0	Validation
TNKS1_ML_3320	C[C@@H]1CCC[NH+]([C@H](c2cc3cc4c(cc3[nH]c2=O)OCCO4)c2nnnn2C2CCCC2)C1	0	Validation
TNKS1_ML_3321	C[C@@H]1C[C@@H](C)CN(S(=O)(=O)c2ccc([N-]S(=O)(=O)c3cccc(Cl)c3Cl)cc2)C1	0	Validation
TNKS1_ML_3322	Cc1ccc(-c2nnc(CCc3nnc(-c4ccncc4)n3-c3ccccc3Cl)o2)cc1	1	Validation
TNKS1_ML_3323	CC[C@H]1CCCN1C(=O)[C@@]1(N)CCC[C@H](C)C1	0	Validation
TNKS1_ML_3324	O=c1c(Oc2ccc(F)cc2)coc2c(O)c([O-])ccc12	0	Validation
TNKS1_ML_3325	Cc1c(C(=O)C2=C([O-])C(=O)N(CC[NH+]3CCOCC3)[C@@H]2c2cccc(Br)c2)cnn1-c1ccccc1	0	Validation
TNKS1_ML_3326	CCOC(=O)c1nn(Cc2cccc[nH+]2)c2c1CN(C(=O)Cc1c[nH]c3ccccc13)CC2	0	Validation
TNKS1_ML_3327	Cc1csc(NC(=O)c2cc(O)nc3ccc(S(=O)(=O)Nc4ccc(F)cc4)cc23)n1	0	Validation
TNKS1_ML_3328	C[C@H](OC(=O)c1cccc2c1O[C@H](C)[C@@H]2C)C(=O)NCCc1ccc(S(N)(=O)=O)cc1	0	Validation
TNKS1_ML_3329	O=C(COC(=O)c1cc(S(=O)(=O)N2CCOCC2)ccc1Cl)NC1CCCCC1	0	Validation
TNKS1_ML_3330	Cc1nn(C)c(C)c1C[C@@H](C)NC(=O)Cn1cccc(C(=O)NC2CCCCC2)c1=O	0	Validation

TNKS1_ML_3331	O=C(N[C@H]1C[C@H](c2nnc(-c3ccc(OC(F)F)cn3)n2-c2ccccc2F)C1)c1ccccn1	1	Validation
TNKS1_ML_3332	CN(C)CC[NH+]1CCC(NC(=O)/C([O-])=N/c2cccc(C#N)c2)CC1	0	Validation
TNKS1_ML_3333	CC(=O)c1ccc(NC(=O)N2CCCN(C(=O)c3cccc(S(C)(=O)=O)c3)CC2)cc1	0	Validation
TNKS1_ML_3334	O=C1COc2ccc(S(=O)(=O)N3CCN(c4nc(-c5ccc(Cl)cc5)cs4)CC3)cc2N1	0	Validation
TNKS1_ML_3335	Cc1cccc(-c2nnc([C@H]3C[C@H](NC(=O)c4cccc5nccnc45)C3)n2-c2ccccc2F)n1	1	Validation
TNKS1_ML_3336	O=C(Nc1ccc2c(c1)OCCCO2)c1ccc(Br)o1	0	Validation
TNKS1_ML_3337	COc1ccc(-c2nc(S(=O)(=O)c3ccc4c(c3)OCCO4)c(N[C@H](C)c3ccccc3)o2)cc1OC	0	Validation
TNKS1_ML_3338	CCC[C@H]1O[C@@H]2C[C@@H]3[C@@H]4CCC5=CC(=O)C=C[C@]5(C)[C@@H]4[C@@H](O)C[C@]3(C)[C@]2(C(=O)CO)O1	0	Validation
TNKS1_ML_3339	OCCN(CCO)c1nc(N2CCCCC2)c2nc(N(CCO)CCO)nc(N3CCCCC3)c2n1	0	Validation
TNKS1_ML_3340	COc1cc(Cl)c(C#N)cc1C(=O)NCCN1CCC(C(=O)c2ccc(F)cc2)CC1	1	Validation
TNKS1_ML_3341	CNS(=O)(=O)c1ccc(N2CCC(N3c4ccccc4C[C@H]3C)CC2)c([N+](=O)[O-])c1	0	Validation
TNKS1_ML_3342	CC(=O)c1cccc([N-]S(=O)(=O)c2ccc(N3C(=O)C(C)(C)CS3(=O)=O)cc2)c1	0	Validation
TNKS1_ML_3343	Nc1nc(-c2ccc(F)cc2)nc2ccc(F)cc12	0	Validation
TNKS1_ML_3344	CCN(CC)C(=O)C(=O)N/N=C\c1cc(C)n(-c2cccc(Br)c2)c1C	0	Validation
TNKS1_ML_3345	Cc1cc2nc(CSc3ccccc3[N-]S(=O)(=O)c3ccc(F)c(F)c3)cc(=O)n2o1	0	Validation
TNKS1_ML_3346	O=S(=O)(N=C1NCN(C2CC2)CN1)c1ccc(Cl)cc1	0	Validation
TNKS1_ML_3347	CC1=C[C@@H](C)[C@H]([C@@H]2OCCC(C)(C)O2)[C@H](C)C1	0	Validation
TNKS1_ML_3348	Cc1nnc(N[C@@H]2CCC[C@@H](C)C2)c(C#N)c1C	0	Validation
TNKS1_ML_3349	Cc1cccc(C(=O)N(CCC2=CCCCC2)c2c(N)n(Cc3ccccc3)c(=O)[nH]c2=O)n1	0	Validation
TNKS1_ML_3350	COc1cc(C(=O)N2CSC[C@@H]2C(=O)NCCc2cscn2)cc(OC)c1OC	0	Validation
TNKS1_ML_3351	CC(C)C[C@@H]1NC(=O)N(C[NH+]2CCN(S(=O)(=O)c3ccc(Cl)c(C(F)(F)F)c3)CC2)C1=O	0	Validation
TNKS1_ML_3352	COc1c2c(c(S(=O)(=O)N3CCC(O)(c4ccc(Cl)cc4)CC3)c3c1OCO3)CCN(C)C2	0	Validation
TNKS1_ML_3353	CC[C@@H]1CO[C@@H](C)CN1C(=O)C(C)(C)C#N	0	Validation
TNKS1_ML_3354	CC(C)C(=O)N1CC(C)(C)c2cc(S(=O)(=O)[N-]c3ccc4c(c3)OCO4)ccc21	0	Validation
TNKS1_ML_3355	C1CCC(c2n[nH]c([C@H]3CC[C@@H]4CCCC[C@@H]4N3)n2)CC1	0	Validation
TNKS1_ML_3356	Cc1nc(-c2ccc(Br)cc2)nc(N)c1C	0	Validation
TNKS1_ML_3357	O=c1[nH]c(-c2ccc(OC(F)(F)F)cc2)nc2c1COCC2	1	Validation
TNKS1_ML_3358	CC1(C)C[C@@](O)([C@H]2CCCC[NH2+]2)CCO1	0	Validation
TNKS1_ML_3359	COCCNC(=O)[C@@H](c1cc[nH+]cc1)N(Cc1ccccc1Cl)C(=O)Cn1nnc2ccccc21	0	Validation
TNKS1_ML_3360	Cc1cccn2c(=O)c(/C=C3\SC(=S)N(Cc4ccccc4)C3=O)c(NC[C@H](O)c3ccccc3)nc12	0	Validation
TNKS1_ML_3361	Cc1c(N)ncnc1-c1ccc(Br)cc1	0	Validation
TNKS1_ML_3362	CC[C@@H]1CCCC[C@H]1NC(=O)N1CC[C@H](C(=O)[O-])C1	0	Validation
TNKS1_ML_3363	COc1ccc2c(c1)CC1(CCN(C3CCN(Cc4nc5c(c(=O)[nH]4)CO	1	Validation

	CC5)C3=O)CC1)C2=O		
TNKS1_ML_3364	C[C@H](CC(=O)N[C@@H]1CCCc2c1cnn2-c1ccc(F)cc1)n1nnc2ccccc21	0	Validation
TNKS1_ML_3365	CCS(=O)(=O)N1CCc2nc(NC(=O)Cc3ccc(S(=O)(=O)C(C)C)cc3)sc2C1	0	Validation
TNKS1_ML_3366	Nc1cc(Cl)c(Cl)cc1Sc1nccc([O-])n1	0	Validation
TNKS1_ML_3367	Cc1cccc(N(C(=O)Cn2nnc(-c3ccccc3F)n2)[C@@H](C(=O)NC(C)(C)C)c2cc[nH+]cc2)c1	0	Validation
TNKS1_ML_3368	Nc1ncnc2c1ccn2Cc1c(Cl)cccc1Cl	0	Validation
TNKS1_ML_3369	COc1ccc(-c2nonc2NC(=O)COc2c(F)c(F)c(F)c(F)c2F)cc1OC	0	Validation
TNKS1_ML_3370	O=C(Nc1cc(C(=O)N2CCN(c3ccc([N+](=O)[O-])cc3)CC2)ccc1N1C[C@@H]2C[C@H](C1)Cn1c2cccc1=O)c1cccnc1	0	Validation
TNKS1_ML_3371	CCOc1cc(/C=C2/C(=O)NC(=O)N(c3ccc(Cl)cc3)C2=O)cc(Br)c1OCCOc1cc(C)cc(CC)c1	0	Validation
TNKS1_ML_3372	CCn1cc(C(=O)Nc2nc(-c3ccc(C#N)cc3)cs2)c(=O)c2ccc(C)nc21	0	Validation
TNKS1_ML_3373	Cc1cc(NC(=O)N[C@H](C)c2cc(C)oc2C)n(C)n1	0	Validation
TNKS1_ML_3374	CC1=C(C(=O)N(C)C)[C@H](c2ccc(F)cc2)NC(=S)N1c1ccc2c(c1)OCCO2	0	Validation
TNKS1_ML_3375	N#Cc1cc2c(nc1Sc1ncn[n-]1)CCC2	0	Validation
TNKS1_ML_3376	CC(C)C[C@H]1NC(=O)[C@@H](C(C)C)NC(=O)[C@@H]2CCCN2C(=O)[C@@H](CO)NC(=O)[C@H](Cc2c[nH]c3ccccc23)NC1=O	0	Validation
TNKS1_ML_3377	CC(C)c1ccc(Nc2ncc(Br)cn2)cc1	0	Validation
TNKS1_ML_3378	[O-]c1c(Cl)nnc2cc3c(cc12)CCCC3	0	Validation
TNKS1_ML_3379	O=C(CCc1nc2ccccc2c(=O)[nH]1)N[C@H]1CC[C@H](c2nnc(-c3ccccc3)o2)CC1	1	Validation
TNKS1_ML_3380	CCc1cc(=O)[nH]c(CN2CCC(N3CCC(C(=O)c4ccc(OC)cc4)CC3)C2=O)n1	1	Validation
TNKS1_ML_3381	Cc1cc(NC(=O)C2CC[NH+](Cc3nc4c(F)cccc4[nH]3)CC2)no1	0	Validation
TNKS1_ML_3382	Cc1ccc2c(c1)sc1nnc(N)n12	1	Validation
TNKS1_ML_3383	Cc1ccccc1S(=O)(=O)Nc1ccc(N2CC[NH2+]CC2)c(C(=O)N(C)C)c1	0	Validation
TNKS1_ML_3384	CC(=O)Oc1ccc(/C(C)=N/NC(=O)CSc2nc3ccccc3c(=O)n2-c2ccc(Cl)cc2)cc1	0	Validation
TNKS1_ML_3385	C=CCOc1cccc([C@H]2C(C(=O)c3ccncc3)=C(O)C(=O)N2CCCn2cc[nH+]c2)c1	0	Validation
TNKS1_ML_3386	Nc1nc2cc(Cl)c(Cl)cc2n1[C@@H]1CCN2CCCC[C@H]12	0	Validation
TNKS1_ML_3387	Cc1cc(=O)[nH]c2cc(-c3ccc(F)cc3)ccc12	1	Validation
TNKS1_ML_3388	O=C(N[C@H]1C[C@H](c2nnc(-c3ccncn3)n2-c2ccccc2Cl)C1)c1ccnc2cccnc12	1	Validation
TNKS1_ML_3389	Cc1cc2oc(=O)cc(CN3C(=O)NC(C)(C)C3=O)c2cc1C(C)C	0	Validation
TNKS1_ML_3390	O=C(N/N=C\c1cn(C(=O)N2CCOCC2)c2ccccc12)c1cc2cc(Br)ccc2o1	0	Validation
TNKS1_ML_3391	[H]/N=C1C(=C/c2cc(C)n(-c3ccc(OC)cc3OC)c2C)/C(=O)N=C2SC(CCC)=NN2/1	0	Validation
TNKS1_ML_3392	Nc1nc2ccccc2n1-c1cc(Cl)c(Cl)cc1Cl	0	Validation
TNKS1_ML_3393	O=C(c1cc(-c2ccc3c(c2)OCO3)n[nH]1)N1CC[C@@H](S(=O)(=O)c2ccc(Cl)cc2)C1	0	Validation
TNKS1_ML_3394	O=S(=O)(N1CCC[C@@H]1[C@H]1CCCN1)C(F)(F)F	0	Validation
TNKS1_ML_3395	CCn1nc(C)c(N)c1NC1(CO)CCCCC1	0	Validation
TNKS1_ML_3396	Cc1cccn2c(C)c(C(=O)C3=C(O)C(=O)N(CCCn4cc[nH+]c4)[C@H]3c3ccccc3F)nc12	0	Validation
TNKS1_ML_3397	CC(C)CN1C(=O)[C@H]2[C@@H](C(=O)NC3CC3)[C@H]3C=C[C@@]2(O3)[C@@H]1C(=O)NC1CCCCC1	0	Validation

TNKS1_ML_3398	COc1ccc(NC(=O)[C@@H]2c3cc(OC)c(OC)cc3C(=O)N(C)[C@@H]2c2ccc(SC)cc2)c(OC)c1	0	Validation
TNKS1_ML_3399	Cc1nc2ccc(S(=O)(=O)N3CCN(c4ccc([N+](=O)[O-])c(NCc5ccccc5)c4)CC3)cc2s1	0	Validation
TNKS1_ML_3400	O=C(Cc1ccc(F)c(Br)c1)Nc1cc[nH]n1	0	Validation
TNKS1_ML_3401	CC[C@@H]1C[C@H](C(F)(F)F)n2[nH+]c([C@@H]3CCCN(C(=O)c4ccc(=O)[nH]c4)C3)cc2N1	0	Validation
TNKS1_ML_3402	COc1ccc([C@H]2C[C@@H](c3ccc(Br)cc3)Nc3[nH+]cnn32)c(OC)c1	0	Validation
TNKS1_ML_3403	Cc1ccc(C2=NN(C(=O)CN3C(=O)N[C@@H](CC4=c5ccccc5=[NH+]C4)C3=O)[C@H](c3ccco3)C2)cc1	0	Validation
TNKS1_ML_3404	Cc1cc(F)cc2c(=O)[nH]c(-c3ccc(C4CCN(C)CC4)cc3)nc12	1	Validation
TNKS1_ML_3405	CC(C)(C)C1CCN(c2cc(NN)ncn2)CC1	0	Validation
TNKS1_ML_3406	CCOc1ccccc1NC(=O)[C@@H](CC)Sc1nc2ccsc2c(=O)n1Cc1cccc[nH+]1	0	Validation
TNKS1_ML_3407	Cc1ccc2[nH]c(Sc3cccc(C#N)n3)nc2c1	0	Validation
TNKS1_ML_3408	Cc1ccc(C)c(CC(=O)N2CCC[C@@H](/C(N)=N/O)C2)c1	0	Validation
TNKS1_ML_3409	COc1cc(O)c2c(=O)c(-c3ccc(O[C@@H]4O[C@@H](CO[C@H]5OC[C@@](O)(CO)[C@H]5O)[C@@H](O)[C@@H](O)[C@H]4O)c(OC)c3)coc2c1	0	Validation
TNKS1_ML_3410	COC(=O)c1ccc(S(=O)(=O)N2c3ccc(S(N)(=O)=O)cc3C[C@@H]2C)cc1	0	Validation
TNKS1_ML_3411	C[C@@H]([C@@H]1C=C(c2ccccc2)N=N1)N(C)C(=O)[C@@H]1CN(C2CC2)C=C(C(=O)NCc2ccccc2)C1=O	0	Validation
TNKS1_ML_3412	C[C@H]1Cc2cc(S(=O)(=O)N3CCC[C@H](C(=O)NCc4ccco4)C3)ccc2N1C(=O)C1CC1	0	Validation
TNKS1_ML_3413	CN(C)c1ncc(Br)cc1C(=O)NNc1cc(C#N)cc(Cl)n1	0	Validation
TNKS1_ML_3414	CC(=O)c1ccc(N2CCN([C@H](C)C(=O)Nc3c(C)n(C)n(-c4ccccc4)c3=O)CC2)c(F)c1	0	Validation
TNKS1_ML_3415	CCOc1ccc(-c2nnc(C34CC(NC(=O)c5ccnc6cccnc56)(C3)C4)n2-c2ccccn2)nc1	1	Validation
TNKS1_ML_3416	O=C(C1=C(O)C(=O)N(c2nnc(SCc3ccc(Cl)cc3)s2)[C@H]1c1cccc(Cl)c1)c1ccc2c(c1)OCCO2	0	Validation
TNKS1_ML_3417	Cn1cc(CNc2nsnc2Cl)cn1	0	Validation
TNKS1_ML_3418	CS(=O)(=O)c1ccc(-c2nnc([C@H]3C[C@H](NC(=O)c4ccccn4)C3)n2-c2ccccc2F)nc1	1	Validation
TNKS1_ML_3419	C[C@H]1CN[C@H](C)CN1c1cc(F)c(Br)cc1[N+](=O)[O-]	0	Validation
TNKS1_ML_3420	O=C(CCCN1C(=O)NC2(CCCC2)C1=O)N1CCN(S(=O)(=O)c2ccc(Cl)s2)CC1	0	Validation
TNKS1_ML_3421	CCCn1c2c(c(=O)n(C)c1=O)C(=O)/C(=C/c1cc3c(cc1Br)OCO3)N2	0	Validation
TNKS1_ML_3422	[H]/N=C1C(=C\c2cccc3ccccc23)\C(=O)N=C2SN=C(S(=O)(=O)CC(C)C)N2\1	0	Validation
TNKS1_ML_3423	O=C(c1cc(Cc2n[nH]c(=O)c3ccccc23)ccc1F)N1CCn2c(nnc2-c2ccc(Br)o2)C1	1	Validation
TNKS1_ML_3424	CNC(=O)c1nn(C[NH+]2CCN(Cc3ccc(Br)s3)CC2)c(=O)c2ccccc12	0	Validation
TNKS1_ML_3425	Cc1cc(NS(=O)(=O)c2ccccc2)ccc1C#CCO	0	Validation
TNKS1_ML_3426	CCN(CC)C(=O)[C@H]1CCCN(c2ccc([N+](=O)[O-])cc2/C=N/n2cnnc2SCc2ccccc2)C1	0	Validation
TNKS1_ML_3427	COc1ccc(CN2C(=O)/C(=C\c3c(NCc4ccco4)nc4ccc(C)cn4c3=O)SC2=S)cc1	0	Validation
TNKS1_ML_3428	Cn1cc(C[NH+]2CC[C@@H]3O[C@@H](C(=O)NCC4CCOCC4)C[C@@H]32)cn1	0	Validation
TNKS1_ML_3429	Cn1c(CC(=O)Nc2ccccc2Cl)nnc1SCC(=O)/[NH+]=C1/NCCS	0	Validation

	1		
TNKS1_ML_3430	COc1cc(NC(=O)/C=C/c2ccc(Cl)cc2)ccc1NC(=O)c1ccco1	0	Validation
TNKS1_ML_3431	OC1=Nc2[nH]ncc2[C@@H](c2c(F)cccc2F)C1	0	Validation
TNKS1_ML_3432	Cc1cc(C)n(CCN(C(=O)c2cccc([N+](=O)[O-])c2)c2nc3c(C(C)C)cccc3s2)n1	0	Validation
TNKS1_ML_3433	CCN(C(=O)CC#N)C(=O)NC(C)C	0	Validation
TNKS1_ML_3434	COc1cccc(N2CCN(c3nc4c(c(=O)[nH]3)CCCC4)CC2)c1	1	Validation
TNKS1_ML_3435	N#Cc1cc(Br)c2c[nH]nc2c1	0	Validation
TNKS1_ML_3436	COc1c(Br)cc(Cl)cc1N[C@@H]1C[C@@H]2CC=C[C@@H]21	0	Validation
TNKS1_ML_3437	CN1CCc2c1nc(N1CCC3(CC1)CN(CCO)c1cc(F)cc(F)c13)[nH]c2=O	1	Validation
TNKS1_ML_3438	C[C@H]1CC[C@H](C(C)(C)c2ccc3c(c2)OCCO3)[C@H]([NH3+])C1	0	Validation
TNKS1_ML_3439	CC(C)(O)c1ccc(-c2nc3c(c(=O)[nH]2)COCC3)cc1	1	Validation
TNKS1_ML_3440	OCc1n[nH]c(-c2cccc(Br)c2)n1	0	Validation
TNKS1_ML_3441	Cc1cc([N-]S(=O)(=O)c2ccc(N)nc2)ccc1F	0	Validation
TNKS1_ML_3442	O=C(c1cc(Cl)c(Cl)[nH]1)N1CCCCCCC1	0	Validation
TNKS1_ML_3443	Cc1ccc(-c2cn3c4c(=O)n(C)c(=O)n(C)c4[nH+]c3n2-c2c(C)n(C)n(-c3ccccc3)c2=O)cc1	0	Validation
TNKS1_ML_3444	CCCCc1nc(C)c(C(=O)N2CCN(Cc3nc(N)c4ccccc4[nH+]3)CC2)s1	0	Validation
TNKS1_ML_3445	OCc1nc(-c2ccc(Br)cc2)n2ccccc12	0	Validation
TNKS1_ML_3446	C[C@@H]1CN(Cc2c[nH]nc2-c2ccc3c(c2)OCCO3)[C@H](c2ccccc2)CO1	0	Validation
TNKS1_ML_3447	CCOc1ccc2c(=O)[nH]c3sccc3c2c1	1	Validation
TNKS1_ML_3448	Cc1nc(N)ccc1-c1cn(C(C)C)nc1C	0	Validation
TNKS1_ML_3449	CC1(C)[C@@H](Nc2ccc3cn[nH]c3c2)[C@@H]2CCCO[C@@H]21	0	Validation
TNKS1_ML_3450	COc1ccc(Cl)cc1/C=C/C(=O)Nc1ccc(NC(=O)[C@H](C)n2cncn2)cc1	0	Validation
TNKS1_ML_3451	O=C(N[C@H]1C[C@H](c2nnc(-c3ccc(OC4CC4)cn3)n2-c2ccccc2F)C1)c1ccccn1	1	Validation
TNKS1_ML_3452	CN(C)S(=O)(=O)c1ccc(S(=O)(=O)Nc2ccccc2C(=O)N2CCOCC2)cc1	0	Validation
TNKS1_ML_3453	COc1ccc(-c2nc(O)c3cccc(C)c3n2)cc1	1	Validation
TNKS1_ML_3454	O=c1[nH]c(CNCc2cccs2)nc2c1COCC2	1	Validation
TNKS1_ML_3455	CN1CCN([C@]2(C#N)CCCNC2)CC1	0	Validation
TNKS1_ML_3456	O=C([O-])c1ccc2[nH+]c(C3=CC=CC3)[nH]c2c1	0	Validation
TNKS1_ML_3457	Cc1cccc(CNC(=O)[C@H]2CC23CCN(C(=O)c2cc(-c4ccccc4)no2)CC3)[nH+]1	0	Validation
TNKS1_ML_3458	CC(C)Cc1nnc(NC(=O)CCC(=O)N2CCC3(CC2)OCCO3)s1	0	Validation
TNKS1_ML_3459	C[C@@H](OC(=O)CCNC1=NS(=O)(=O)c2ccccc21)C(=O)Nc1ccc(N(C)C)cc1	0	Validation
TNKS1_ML_3460	CCOc1ccc([C@@H]2C(C#N)=C(N)S/C2=N/[O-])cc1	0	Validation
TNKS1_ML_3461	Cc1ccccc1-n1c(/C=C/c2nnc(-c3ccc(C#N)cc3)o2)nnc1-c1ccc(S(C)(=O)=O)cn1	1	Validation
TNKS1_ML_3462	COC(=O)[C@@H]1Cc2c([nH]c3ccccc23)CN1C(=O)COc1cc(C)cc2oc(=O)c(Cc3ccccc3)c(C)c12	0	Validation
TNKS1_ML_3463	O=[N+]([O-])c1ccc(-c2nnc(SC[C@@H](O)c3ccc(Br)cc3)o2)cc1	0	Validation
TNKS1_ML_3464	C[C@H](NS(=O)(=O)c1ccc2c(c1)OCCO2)c1cc(=O)[nH]c(N2CCCC2)n1	0	Validation
TNKS1_ML_3465	Cc1ccc(C)c(Oc2ncnc(Cl)c2N)c1C	0	Validation
TNKS1_ML_3466	C[C@H](NC(=O)c1ccc(=O)n(CC(=O)N(C)c2ccccc2)c1)c1[nH]c2ccccc2[nH+]1	0	Validation
TNKS1_ML_3467	O=C1NN=C2CCCCc3[nH]c4cccc1c4c32	1	Validation

TNKS1_ML_3468	O=C(c1ccc(Cl)s1)c1coc2ccc(O)cc12	0	Validation
TNKS1_ML_3469	Cc1ccc(/C=C2\SC(=O)N(CCC(=O)Nc3c(C)n(C)n(-c4ccccc4)c3=O)C2=O)cc1	0	Validation
TNKS1_ML_3470	COc1cc(C[C@@H]2[C@H](O)c3ccccc3S(=O)(=O)N2C(C)C)cc(OC)c1OC	0	Validation
TNKS1_ML_3471	O=C(CNC(=O)c1ccc(Br)s1)OCC(=O)N1CCc2ccccc21	0	Validation
TNKS1_ML_3472	O=C(CCC1CCCC1)Nc1nnc2n1CCS2	1	Validation
TNKS1_ML_3473	O=c1[nH]c2cc(Br)c(NCc3c[nH+]c[nH]3)cc2[nH]1	0	Validation
TNKS1_ML_3474	O=C([O-])[C@H]1CCCC[C@@H]1[NH+]1CCCCCC1	0	Validation
TNKS1_ML_3475	CC(C)[C@@H](NC(=O)c1ccoc1)c1nnc2n1CCN(Cc1ccccc1Cl)CC2	0	Validation
TNKS1_ML_3476	O=C1NC(c2ccccc2)Nc2ccccc21	1	Validation
TNKS1_ML_3477	CC1=C(C(=O)CSc2nnc(NC[C@H]3CCCO3)s2)C[C@@H](C)N1c1ncn[nH]1	0	Validation
TNKS1_ML_3478	CC(C)c1nc(N2CCC(NC(=O)[C@H]3CC(=O)OC3(C)C)CC2)ncc1-c1cc[nH+]cc1	0	Validation
TNKS1_ML_3479	CCOc1ccc(-c2nnc([C@H]3C[C@H](n4c(=O)[nH]c5cc(C#N)ccc54)C3)n2-c2ccccc2Cl)nc1	1	Validation
TNKS1_ML_3480	CN1CCN(c2ccc(NC(=O)c3ccc4c(c3)OCO4)c[nH+]2)CC1	0	Validation
TNKS1_ML_3481	Cc1ccccc1[C@H]1C[C@H](c2ccccc2OC(F)F)[NH+]=C2N=CNN21	0	Validation
TNKS1_ML_3482	CCOc1cc([C@@H]2c3c(-c4c(C)cc(C)cc4O)n[nH]c3C(=O)N2CCOC)ccc1O	0	Validation
TNKS1_ML_3483	Cc1ccc(S(=O)(=O)NC2CC(C)(C)N(O)C(C)(C)C2)cc1	0	Validation
TNKS1_ML_3484	O=C(NC1CCCCCCC1)c1cn(CCc2cccc[nH+]2)cc(C(=O)N2C[C@@H]3CC[C@H]2C3)c1=O	0	Validation
TNKS1_ML_3485	CC(=O)Nc1nnc(SCc2cc(=O)c(OC(=O)[C@H]3CC(=O)N(c4ccccc4)C3)co2)s1	0	Validation
TNKS1_ML_3486	Cc1cc2ncc(-c3ccc(F)cc3)c(N)n2n1	0	Validation
TNKS1_ML_3487	Cc1ccc(S(=O)(=O)Oc2ccc(/C=C3/C(=O)N(c4ccccc4)N=C3[O-])cc2)cc1	0	Validation
TNKS1_ML_3488	CCOc1ccc(-c2nnc([C@H]3C[C@H](NC(=O)c4ccnc5cccnc45)C3)n2-c2ccncc2)nc1	1	Validation
TNKS1_ML_3489	CC1(C(=O)N2CCC([C@@]3(C)NC(=O)N(C4Cc5ccccc5C4)C3=O)CC2)CCCCC1	0	Validation
TNKS1_ML_3490	[H]/N=C1C(=C/c2cccn2CCOc2ccccc2)/C(=O)N=C2SC(N3CCC(C)CC3)=NN2/1	0	Validation
TNKS1_ML_3491	Cc1c(NC(=O)[C@H](C)Sc2ccc(S(=O)(=O)N(C)C)cn2)c(=O)n(-c2ccccc2)n1C	0	Validation
TNKS1_ML_3492	C[C@@H](C(=O)NCc1ccc2c(c1)OCO2)N1CCN(S(=O)(=O)c2ccc3ccccc3c2)CC1	0	Validation
TNKS1_ML_3493	Nc1cc(C2CCN(S(=O)(=O)c3ccc(Cl)cc3)CC2)no1	0	Validation
TNKS1_ML_3494	C#CCN(Cc1nc2c(c(=O)[nH]1)COCC2)C(=O)CN1CCC(C(=O)c2ccc(F)cc2)CC1	1	Validation
TNKS1_ML_3495	COc1ccc(C#N)cc1NC(=O)N1CCc2sccc2[C@@H]1C	0	Validation
TNKS1_ML_3496	Cc1ccc(S(=O)(=O)/N=C(/CN2CCCCC2)N2CCN(S(=O)(=O)c3ccccc3)[C@@H]2c2ccccc2)cc1	0	Validation
TNKS1_ML_3497	Cc1nn(C)c(N[C@@H]2CCC[NH2+]C2)c1C#N	0	Validation
TNKS1_ML_3498	Cc1ccc([C@H]2c3ccsc3CCN2CCC(=O)N2CCN(C(=O)COCc3ccccc3)[C@H](C)C2)cc1	0	Validation
TNKS1_ML_3499	CC(C)(C)OC(=O)N1CCC2(CCN(C(=O)c3cc(Cc4n[nH]c(=O)c5ccccc45)ccc3F)C2)C1	1	Validation
TNKS1_ML_3500	CSC1=[NH+]SC2=NC(=O)/C(=C\c3ccc(OCc4ccc(C)cc4)cc3)C(=[NH2+])N21	0	Validation
TNKS1_ML_3501	Cc1ccc(-c2cc(C(=O)NCc3ccc[nH+]c3)c3c(C)nn([C@H]4CCS(=O)(=	0	Validation

	O)C4)c3n2)cc1		
TNKS1_ML_3502	c1ccc2c(c1)SCC[C@H]2Nc1ccc2c(c1)OCCO2	0	Validation
TNKS1_ML_3503	N#Cc1ccccc1N[C@@H]1CCCc2cccnc21	0	Validation
TNKS1_ML_3504	C[C@H]1CC[C@@H](n2c(C(F)(F)F)nc3c2CCNC3)[C@H](C)C1	0	Validation
TNKS1_ML_3505	Cc1ccc([C@H]2Nc3c(C)ccc(C(=O)[O-])c3[C@@H]3C=CC[C@@H]23)cc1	0	Validation
TNKS1_ML_3506	N#CC1=C(N)OC2=C(C(=O)CCC2)[C@@H]1[C@H]1CC=CCC1	0	Validation
TNKS1_ML_3507	O=c1cc(-c2ccc([N+](=O)[O-])cc2)oc2ccccc12	1	Validation
TNKS1_ML_3508	O=C(Nc1c(F)cc(F)cc1F)c1ccc2cn[nH]c2c1	0	Validation
TNKS1_ML_3509	CN(CC(=O)N1C[C@@H](C(N)=O)Oc2ccccc21)S(=O)(=O)/C=C/c1ccccc1	0	Validation
TNKS1_ML_3510	Nc1cc(F)ccc1Oc1cnc2ccccc2n1	0	Validation
TNKS1_ML_3511	COCC(=O)N1CCN(C(=O)[C@H]2CC[C@H](Cn3c(=O)c4cc(OC)ccc4n(C)c3=O)CC2)CC1	1	Validation
TNKS1_ML_3512	Cc1cc2ccc3cccnc3c2nc1N	0	Validation
TNKS1_ML_3513	O=c1[nH]c(-c2ccc(Cl)cc2)nc2c1COCC2	1	Validation
TNKS1_ML_3514	O=C(CN1CCC(c2noc3cc(F)ccc23)CC1)NCc1nc2c(c(=O)[nH]1)COCC2	1	Validation
TNKS1_ML_3515	CN(C)S(=O)(=O)c1ccc(N2C[C@H](C(=O)NCCC3=c4ccccc4=[NH+]C3)CC2=O)cc1	0	Validation
TNKS1_ML_3516	C[C@H]1C[C@H]([NH+]2[C@@H](C(=O)[O-])C[C@H]3CCCC[C@H]32)CC(C)(C)C1	0	Validation
TNKS1_ML_3517	O=C(NCc1nccs1)c1nn(Cc2ccccc2)c2c1CN(C(=O)c1cscn1)CC2	0	Validation
TNKS1_ML_3518	Cc1[nH+]ccn1Cc1cccc(CNC(=O)Nc2ccc(CN3CCCC3=O)cc2)c1	0	Validation
TNKS1_ML_3519	Cc1nc2cc(N)ccc2n1-c1cc(Cl)ccc1F	0	Validation
TNKS1_ML_3520	O=C1CN(Cc2[nH]cc[nH+]2)C[C@@H](O)CN1C1CCCCC1	0	Validation
TNKS1_ML_3521	C=CCc1cc(/C=C2/C(=O)NC(=O)N(c3ccc(Br)cc3)C2=O)cc(OC)c1OCc1ccc(Cl)cc1	0	Validation
TNKS1_ML_3522	CSc1ccc2nc(N(CC[NH+](C)C)C(=O)[C@H]3CCCN3S(=O)(=O)c3cccs3)sc2c1	0	Validation
TNKS1_ML_3523	Cc1c(C(=O)NC[C@](C)(O)c2cc3ccccc3s2)cccc1[N+](=O)[O-]	0	Validation
TNKS1_ML_3524	COc1ccc(C(=O)Nc2ccc(C(=O)NCC3(c4ccc(OC)cc4)CCOCC3)c(Cl)c2)cc1	1	Validation
TNKS1_ML_3525	CC(C)C(=O)Nc1ccc(C2NC(=O)c3cccnc3N2C)cc1	1	Validation
TNKS1_ML_3526	O=C=Nc1cc(Cl)nc(Cl)c1	0	Validation
TNKS1_ML_3527	Fc1cc(Nc2nccnc2Cl)cc(F)c1F	0	Validation
TNKS1_ML_3528	COCCOc1cc(F)c2c(c1)N(C)C(=O)C21CCN(c2nc3c(c(=O)[nH]2)CCCN3C)CC1	1	Validation
TNKS1_ML_3529	Cc1ccc(C(=O)Nc2c(C)ccc([N+](=O)[O-])c2C)cc1C	0	Validation
TNKS1_ML_3530	COc1cccc(Oc2c(C)oc3c4c(ccc3c2=O)OCN([C@H]2CCS(=O)(=O)C2)C4)c1	0	Validation
TNKS1_ML_3531	CC(C)c1nc(Cl)nc(N(C)C)n1	0	Validation
TNKS1_ML_3532	CC(C)N1C(N)=[NH+]C[C@H]1c1cc(Cl)c2c(c1)OCCCO2	0	Validation
TNKS1_ML_3533	O=C1NC(c2ccc(OCc3ccccc3)cc2)Nc2ncccc21	1	Validation
TNKS1_ML_3534	Cc1cnc(CNC(=O)c2cc(-c3cn(Cc4cccnc4)c(C)n3)n3ccccc23)cn1	0	Validation
TNKS1_ML_3535	CN1CCCc2c1nc(N1CCC3(CC1)C(=O)N(C)c1cc(OCC(=O)O)cc(F)c13)[nH]c2=O	1	Validation
TNKS1_ML_3536	CC(C)(C)c1c(C(=O)[O-])nnn1C[C@H]1C[C@H]2CC[C@@H]1C2	0	Validation
TNKS1_ML_3537	CCc1ccc([C@@H]2Nc3ccccc3S(=O)(=O)N2)cc1	0	Validation
TNKS1_ML_3538	CCN(C(C)=O)c1cccc(-c2ccnc3c(C(=O)NCCCC(=O)[O-])cnn23)c1	0	Validation

TNKS1_ML_3539	COC(=O)[C@H](Cc1c[nH]c[nH+]1)NC(=O)CO/N=C1\C=C2CC[C@@H]3[C@H]4CC[C@@H](C(C)=O)[C@@]4(C)CC[C@H]3[C@@]2(C)CC1	0	Validation
TNKS1_ML_3540	C[C@H](Cn1cncn1)NC(=O)c1coc(C[NH+]2CCN(C/C=C/c3ccccc3)CC2)n1	0	Validation
TNKS1_ML_3541	CCc1ccc2[nH]c(=O)c([C@H](c3nnnn3CCOC)[NH+](Cc3cccnc3)Cc3ccco3)cc2c1	0	Validation
TNKS1_ML_3542	COc1cccc2c(O)nc(-c3ccccc3)nc12	1	Validation
TNKS1_ML_3543	CC1=C(S(=O)(=O)Nc2ccc(C)c(C)c2)[C@@H](C)N=N1	0	Validation
TNKS1_ML_3544	Cc1ccc(C(=O)N[C@@H](c2ccccc2F)c2[nH+]ccn2C)cc1NS(C)(=O)=O	0	Validation
TNKS1_ML_3545	C[C@]12C(=O)N(/N=C/c3ccc(Cl)cc3)CC(=O)N1CCc1c2[nH]c2ccccc12	0	Validation
TNKS1_ML_3546	COC(=O)c1cnc(NC(=O)C2(S(=O)(=O)c3ccc(C)c(C)c3)CCCC2)s1	0	Validation
TNKS1_ML_3547	COc1cccc2c1c(=O)n(C[C@H]1CC[C@H](C(=O)O)CC1)c(=O)n2Cc1cccc(C(F)(F)F)c1C#N	1	Validation
TNKS1_ML_3548	Cc1noc(C)c1-c1nc(=O)c(I)c([O-])[nH]1	0	Validation
TNKS1_ML_3549	CS(=O)(=O)c1ccc2nc(N(CC[NH+]3CCOCC3)C(=O)c3c[nH]c4ccccc34)sc2c1	0	Validation
TNKS1_ML_3550	CCS(=O)(=O)[N-]c1cccc(C2=NN(S(=O)(=O)c3ccccc3)[C@H](c3ccccc3F)C2)c1	0	Validation
TNKS1_ML_3551	N#Cc1ccc(-n2ncc3ccc(N)cc32)c(Cl)c1	0	Validation
TNKS1_ML_3552	Oc1ccc2c(c1)sc1nncn12	1	Validation
TNKS1_ML_3553	CC(=O)Nc1ccc(S(=O)(=O)N(C)CC(=O)OCC(=O)C2=c3ccccc3=[NH+][C@H]2C)cc1	0	Validation
TNKS1_ML_3554	Cn1cccc1/C=N/n1c([S-])nnc1-c1cccs1	0	Validation
TNKS1_ML_3555	[H]/N=C1C(=C/c2ccc([NH+](CC)CC)cc2)/C(=O)N=C2SC(C3CCCCC3)=NN2/1	0	Validation
TNKS1_ML_3556	Cc1ccc(S(=O)(=O)[N-]c2ccc(F)c(N)c2)s1	0	Validation
TNKS1_ML_3557	Cc1cc(C)cc(-c2nnc3cc(Cl)nc(C)n23)c1	0	Validation
TNKS1_ML_3558	Cc1ccnc2nc(C(=O)Nc3ccc(NC(=O)c4ccc(F)cc4)cc3C(F)(F)F)nn12	0	Validation
TNKS1_ML_3559	COc1cccc2sc3nncn3c12	1	Validation
TNKS1_ML_3560	N#CCCN(Cc1cccnc1)S(=O)(=O)c1ccc(S(=O)(=O)NC2CCCCC2)cc1	0	Validation
TNKS1_ML_3561	CC[C@H]1CC[C@@H](O)[C@H](n2cnc3cc(C)c(C)cc32)C1	0	Validation
TNKS1_ML_3562	C[C@@H]1CN(C(=O)NCC(C)(C)c2cc[nH+]cc2)C[C@@H](c2ccc(F)cc2)O1	0	Validation
TNKS1_ML_3563	Cc1cc(S(=O)(=O)N2CCCN(S(=O)(=O)c3c[nH]cn3)CC2)c(Cl)cc1Cl	0	Validation
TNKS1_ML_3564	COc1ccc(-n2nnnc2SCC(=O)N(Cc2cccs2)c2ccc3c(c2)OCCO3)cc1	0	Validation
TNKS1_ML_3565	C[C@@H](Sc1ccc(S(=O)(=O)N2CCCCCC2)c[nH+]1)C(=O)N1CCNC1=O	0	Validation
TNKS1_ML_3566	O=S(=O)(c1c[nH]c2ncccc12)N1CCC2(CC1)C[C@@H]2c1nnc(-c2ccccc2)o1	0	Validation
TNKS1_ML_3567	C[C@@H]1CCC[C@@]2(CS/C(=N\c3ccc(CC#N)cc3)N2)C1	0	Validation
TNKS1_ML_3568	C[C@]1(C(=O)O)CCC[C@@]2(C)[C@H]3CC[C@H]4C[C@]3(CC[C@@H]12)CC4=O	0	Validation
TNKS1_ML_3569	COc1ccc(OC)c([C@@H]2C(C(=O)c3sc(C)nc3C)=C([O-])C(=O)N2CCC[NH+](C)C)c1	0	Validation
TNKS1_ML_3570	Cc1cccn2c(C)c(C(=O)C3=C([O-])C(=O)N(CC[NH+]4CCOCC4)[C@H]3c3ccc(F)cc3)nc12	0	Validation
TNKS1_ML_3571	Cc1nc([S-])sc1CC(=O)NC1CCCCCCC1	0	Validation

TNKS1_ML_3572	Cc1cc2c(=O)[nH]c(-c3ccc(C(C)(C)O)cc3)cn2n1	1	Validation
TNKS1_ML_3573	O=C1NC(c2ccc(C(F)(F)F)cc2)=NC2CSCC12	1	Validation
TNKS1_ML_3574	Cc1cccc2c(=O)[nH]c(-c3ccc(C(C)(C)O)cc3)nc12	1	Validation
TNKS1_ML_3575	CC[C@@H](C)N(CC)C(=O)c1cc2cc(N)ccc2o1	0	Validation
TNKS1_ML_3576	[NH3+]C1(CC(=O)N2CCCN3CCC[C@H]3C2)CCCC1	0	Validation
TNKS1_ML_3577	Cc1c(C(=O)Nc2ccc(Cl)c(S(=O)(=O)N3CCCC3)c2)cnn1[C@@H]1CCS(=O)(=O)C1	0	Validation
TNKS1_ML_3578	[NH3+][C@H]1CCCC[C@@H]1C(=O)N[C@@H]1CCCC[C@@H]1O	0	Validation
TNKS1_ML_3579	CC(C)(C)NC(=O)N1CCC(CNS(=O)(=O)c2cc(F)cc(F)c2)CC1	0	Validation
TNKS1_ML_3580	C#Cc1cccc([N-]S(=O)(=O)c2cc(C)ccc2O)c1	0	Validation
TNKS1_ML_3581	Clc1ncc(Br)c(NC2CCCCCC2)n1	0	Validation
TNKS1_ML_3582	COc1ccc(N2C(=O)C(C)(C)CS2(=O)=O)cc1S(=O)(=O)Nc1ccc(C)c(Cl)c1	0	Validation
TNKS1_ML_3583	CC1(C)CC2=NNC(=O)c3cccc4c3c2c(n4CCN2CCCC2)C1	1	Validation
TNKS1_ML_3584	COc1ccc(C(=O)C2CCN(C3CCCN(Cc4nc5c(c(=O)[nH]4)COCC5)C3=O)CC2)cc1	1	Validation
TNKS1_ML_3585	COc1ccc2[nH]c([S@](=O)Cc3ncc(C)c(OC)c3C)nc2c1	0	Validation
TNKS1_ML_3586	C[C@H](Nc1ccc2c(c1)OC(F)(F)O2)c1nccn1C	0	Validation
TNKS1_ML_3587	COc1ccc(C(=O)C2CCN(CC(=O)N(Cc3nc4c(c(=O)[nH]3)COCC4)CC(F)F)CC2)cc1	1	Validation
TNKS1_ML_3588	CC[C@@H](C)NC(=O)[C@@H](C)NC(=O)c1cc2n(n1)CCCN(Cc1cccc(C)c1)C2=O	0	Validation
TNKS1_ML_3589	COC(=O)c1c(C)sc(C2=C3C(=O)N(C)C(=O)N(C)[C@H]3N=C2c2c(OC)cccc2OC)c1O	0	Validation
TNKS1_ML_3590	CC(C)(C)OC(=O)N1C[C@@H]2C[C@H]3CNC[C@@]3(C1)O2	0	Validation
TNKS1_ML_3591	C[C@H]1CC(=O)NC2(CCCCC2)C(=O)N1C[C@H]1CCCO1	0	Validation
TNKS1_ML_3592	Cl.O=c1[nH]c(-c2ccc(C3CCNCC3)cc2)nc2c(F)cc(F)cc12	1	Validation
TNKS1_ML_3593	Cc1cccc(-n2c(=C(C#N)C#N)s/c(=C\c3ccc([C@H]4C[C@H]4C)o3)c2=O)c1	0	Validation
TNKS1_ML_3594	Cc1ccc(-c2noc(CSc3nnc(C)n3-c3ccc(C)c(C)c3)n2)cc1	1	Validation
TNKS1_ML_3595	O=C(CSc1nnc(-c2ccncc2)n1-c1ccc(Br)cc1)N/N=C/c1ccccn1	0	Validation
TNKS1_ML_3596	CC1(C)[C@H]2CC[C@@]13CNS(=O)(=O)[C@@H]3C2	0	Validation
TNKS1_ML_3597	CC(C)(C)NC(=O)N(CCN1CCOCC1)CC(=O)N1N=C(c2ccc(F)cc2)C[C@H]1c1ccco1	0	Validation
TNKS1_ML_3598	CCOc1ccc(-c2nnc([C@H]3C[C@H](NC(=O)c4cccc5nc(C)c(C)nc45)C3)n2-c2ccccc2F)nc1	1	Validation
TNKS1_ML_3599	CN1CCCc2c1nc(N1CCC3(CC1)C(=O)N(CC(=O)O)c1cc(F)cc(F)c13)[nH]c2=O	1	Validation
TNKS1_ML_3600	CC(C)(C)c1ccc(S(=O)(=O)N2CCN(CN3C(=O)[C@]4(C[NH+]5CCC4CC5)N=C3[O-])CC2)cc1	0	Validation
TNKS1_ML_3601	O=S(=O)(NC[C@H]1C[C@@H]2CC[NH+]1C[C@@H]2CN1CCN(c2ccccn2)CC1)c1ccccc1	0	Validation
TNKS1_ML_3602	CCOC(=O)c1sc2[nH+]c(N3CCOCC3)c3c(c2c1N)CC(C)(C)SC3	0	Validation
TNKS1_ML_3603	N#Cc1ccnc(Nc2ccc(F)cc2Br)c1	0	Validation
TNKS1_ML_3604	O=C(Nc1ccc2c(c1)OCO2)c1cc(S(=O)(=O)N2CCc3ccccc3C2)ccc1Cl	0	Validation
TNKS1_ML_3605	COc1cc(OC)c2c(=O)c(-c3ccc(O[C@H]4O[C@@H](CO)[C@@H](O)[C@@H](O)[C@@H]4O)cc3)coc2c1	0	Validation
TNKS1_ML_3606	Cc1ccc(-c2nnn(CC(=O)N(c3ccc4c(c3)OCCO4)[C@@H](C(=O)NC3	0	Validation

	CCCC3)c3ccc(F)cc3)n2)o1		
TNKS1_ML_3607	Cc1nn(-c2ccccc2)c2c1[C@@H](c1ccccc1)c1c(ncn3nc(CSc4nc5ccccc5s4)nc13)O2	0	Validation
TNKS1_ML_3608	Cc1nc(-c2cc3ccccc3oc2=O)sc1C(=O)[O-]	0	Validation
TNKS1_ML_3609	CN(C)[C@@H](CNS(=O)(=O)c1ccc(N2CCCCS2(=O)=O)cc1)c1cn(C)c2ccccc12	0	Validation
TNKS1_ML_3610	COc1ccc(C)cc1NC(=O)[C@@H](Sc1cc(C)nc(C)n1)c1ccccc1	0	Validation
TNKS1_ML_3611	COc1ccc(C(=O)C2CCN(C3CCN(Cc4ncc(C)c(=O)[nH]4)C3=O)CC2)cc1	1	Validation
TNKS1_ML_3612	Cc1ccc([N-]S(=O)(=O)c2ccc(Cl)c(C(=O)N3CCN(Cc4ccccc4)CC3)c2)cc1C	0	Validation
TNKS1_ML_3613	C/C(=N/NC(=O)c1cc(C)n(-c2ccccc2)c1C)c1ccc2c(c1)OCCO2	0	Validation
TNKS1_ML_3614	N[C@@]1(c2noc(C(F)(F)F)n2)CCCc2ccccc21	0	Validation
TNKS1_ML_3615	COc1ccc(C(=O)C2=C(O)C(=O)N(CCCn3cc[nH+]c3)[C@H]2c2cc(OC)c3c(c2)OCO3)cc1OC	0	Validation
TNKS1_ML_3616	[O-]c1nnc(-c2ccc(Cl)cc2)cc1O	0	Validation
TNKS1_ML_3617	CC(=[NH2+])C(C#N)C(=O)COC(=O)c1cccc(S(=O)(=O)N2CCc3ccccc3C2)c1	0	Validation
TNKS1_ML_3618	COc1cc(OC)c2sc3nncn3c2c1	1	Validation
TNKS1_ML_3619	C[C@H]1C[NH+](C(C)(C)CNc2nc3ccccc3o2)C[C@@H](C)O1	0	Validation
TNKS1_ML_3620	C[C@@H](c1ccc(O)cc1O)[NH+]1CCC[C@H](N)C1	0	Validation
TNKS1_ML_3621	C[C@H]1CCC[C@H](N(C)C(=O)c2nc(N)ccc2Cl)C1	0	Validation
TNKS1_ML_3622	O=C(CSc1ccc(S(=O)(=O)N2CCCCCC2)cn1)N1CCN(c2ccc(O)cc2)CC1	0	Validation
TNKS1_ML_3623	C#CCOc1nc(Cl)nnc1Cl	0	Validation
TNKS1_ML_3624	COc1ccc(C(=O)C2CCN(C(=O)CCCc3cn4cc(F)cc4c(=O)[nH]3)CC2)cn1	1	Validation
TNKS1_ML_3625	CC[NH+]1CCCC[C@H]1C(=O)N1CCc2c(c(C(=O)N3CCC(O)CC3)nn2CCc2ccccc2)C1	0	Validation
TNKS1_ML_3626	CC[C@@H]1CO[C@H](C)CN1Cc1c(N)cccc1Br	0	Validation
TNKS1_ML_3627	CCc1cc2c(=O)c(-n3ccccc3=O)coc2cc1O	0	Validation
TNKS1_ML_3628	Cc1cc(C)c(/N=C2/NC[C@@H](C)CS2)c(C)c1	0	Validation
TNKS1_ML_3629	[H]/N=C1C(=C/c2cc(Br)ccc2OC)/C(=O)N=C2SC(c3ccccc3)=NN2/1	0	Validation
TNKS1_ML_3630	C[C@H]1CN([C@@H]2CCCCC[C@@H]2O)C[C@H](C)N1	0	Validation
TNKS1_ML_3631	Cc1nc(-c2nc(-c3c(C(=O)NNS(=O)(=O)c4ccc(Cl)cc4)noc3C)cs2)cs1	0	Validation
TNKS1_ML_3632	CC(=O)OCC(=O)[C@@]12OC(C)(C)O[C@@H]1C[C@H]1[C@H]3CC(C=O)=C4C=C(OCCCl)CC[C@]4(C)[C@@]3(F)[C@@H](O)C[C@@]12C	0	Validation
TNKS1_ML_3633	COc1cc(/C=N/Nc2ncc(C(F)(F)F)cc2Cl)ccc1OCC(=O)N1C[C@@H](C)O[C@H](C)C1	0	Validation
TNKS1_ML_3634	CN1CCN(S(=O)(=O)c2ccc(SCC(=O)Nc3ccc(N4CCCC4)cc3)nc2)CC1	0	Validation
TNKS1_ML_3635	CN(C(=O)N1CCCCCC1)c1ccc(CN)cc1	0	Validation
TNKS1_ML_3636	[H]/N=C1\CCCN1c1cc(C#N)ccc1Cl	0	Validation
TNKS1_ML_3637	CC1(C)OC(=O)N(c2ccc(-c3cc(-c4ncccn4)c[nH]c3=O)cc2)[C@H]1c1ccccc1	1	Validation
TNKS1_ML_3638	CC(C)c1ccc([C@H]2C(C(=O)c3ccc4c(c3)OCCO4)=C([O-])C(=O)N2CC[NH+]2CCOCC2)cc1	0	Validation
TNKS1_ML_3639	N#Cc1ccc(N2CCN(Cc3ccc4c(c3)NC(=O)C3CCCNC43)CC2)nc1	1	Validation

TNKS1_ML_3640	C[C@@H]1CCc2c(sc(NC(=O)CN3CCN(S(=O)(=O)c4ccc5c(c4)OCCO5)CC3)c2C#N)C1	0	Validation
TNKS1_ML_3641	Cc1cc(NC(=O)c2ccc3c(c2)COCO3)n(-c2ccc(Cl)cc2)n1	0	Validation
TNKS1_ML_3642	Cc1cc(N2CCC(C(=O)[O-])(n3c[nH+]c4ccccc43)CC2)cc[nH+]1	0	Validation
TNKS1_ML_3643	COc1cccc(-c2nn(Cc3ccccc3)cc2C(=O)NCc2nnc3n2CCC3)c1	0	Validation
TNKS1_ML_3644	Cc1cccc(NC(=O)N2CCC[C@]3(C(=O)N(C)C)C[NH+](Cc4cnn(C)c4)C[C@@H]3C2)c1	0	Validation
TNKS1_ML_3645	Cc1ccc(F)cc1/[NH+]=c1\oc2cc(O)ccc2cc1C(=O)Nc1nccs1	0	Validation
TNKS1_ML_3646	CSc1ccc(NS(=O)(=O)c2cc(-c3nc(C)cs3)n(CC(=O)N3CCOCC3)c2)cc1	0	Validation
TNKS1_ML_3647	Cc1ccc(Nc2ccnc(Cl)n2)c(Br)c1	0	Validation
TNKS1_ML_3648	Cc1nn(C)c(C(=O)NNC(=O)c2ccc(Br)s2)c1[N+](=O)[O-]	0	Validation
TNKS1_ML_3649	COc1ccc(CC(=O)N(CCCn2cc[nH+]c2)c2nc3c(OC)ccc(OC)c3s2)cc1OC	0	Validation
TNKS1_ML_3650	CC[NH+](CC)CCNC(=O)C(=O)Nc1c2c(nn1-c1cccc(Cl)c1)CS(=O)(=O)C2	0	Validation
TNKS1_ML_3651	CN(C)c1ccc([C@@H]2CC(=O)Nc3cc4c(cc32)OCO4)cc1	0	Validation
TNKS1_ML_3652	O=C([O-])[C@@H]1CC[C@H](Nc2c([N+](=O)[O-])ccc(F)c2F)C1	0	Validation
TNKS1_ML_3653	CC(C)c1ccc([C@]2(O)CC(C)(C)OC2(C)C)cc1	0	Validation
TNKS1_ML_3654	COc1cc(/C=N/NC(=O)[C@@H](C)Oc2ccc(-c3ccccc3)cc2)ccc1OCc1ccccc1C	0	Validation
TNKS1_ML_3655	Cc1nc2sc([C@@H](c3ccco3)N3CCN(C(c4ccccc4)c4ccccc4)CC3)c([O-])n2n1	0	Validation
TNKS1_ML_3656	CC(C)c1ccc(-c2cc(=O)c3ccccc3o2)cc1	1	Validation
TNKS1_ML_3657	O=C(NCc1ccc(-c2nc3ccccc3c(=O)[nH]2)cc1)OCc1ccccc1	1	Validation
TNKS1_ML_3658	Cc1nc(NC(=O)c2cccc(S(=O)(=O)N3CCCCCC3)c2)sc1-c1nccn1C	0	Validation
TNKS1_ML_3659	Cn1c(CN2CCOC[C@H]2c2[nH]cc(-c3ccc(S(C)(=O)=O)cc3)[nH+]2)nc2ccccc21	0	Validation
TNKS1_ML_3660	Cc1nn(C)c(Cl)c1C[NH+]1C[C@@H](C(=O)NC(C)(C)C)[C@@]2(Cc3ccccc3C(=O)N2)C1	0	Validation
TNKS1_ML_3661	N#Cc1c(NC(=O)COC(=O)CCCNC2=NS(=O)(=O)c3ccccc32)sc2c1CCC2	0	Validation
TNKS1_ML_3662	COc1cccc(CN2CCN(c3ccccc3[C@H](O)c3nccn3C)CC2)c1	0	Validation
TNKS1_ML_3663	O=C(CSc1nnc(-c2ccc3c(c2)OCO3)o1)N1CCN(Cc2nc3ccccc3s2)CC1	0	Validation
TNKS1_ML_3664	Cc1ccc(C(=O)C2=C(O)C(=O)N(c3nnc(SCc4ccc(F)cc4)s3)[C@@H]2c2ccco2)o1	0	Validation
TNKS1_ML_3665	C/C=C1/CN2CC[C@@]34C(=C(C=O)[C@H]1C[C@H]23)Nc1c(O)cccc14	0	Validation
TNKS1_ML_3666	Cc1cccc(C)c1OCCCCn1c(CCCNC(=O)c2ccc(Cl)cc2)[nH+]c2ccccc21	0	Validation
TNKS1_ML_3667	COc1cc(/C=N/NC(=O)[C@@H]2COc3ccccc3O2)ccc1OCC(=O)N1[C@H](C)CCC[C@H]1C	0	Validation
TNKS1_ML_3668	Cc1ccc(NC(=O)c2noc3c2CCc2sccc2-3)cc1C	0	Validation
TNKS1_ML_3669	CN(C)c1ccc(CNC(=O)Cn2c(=O)oc3cc(S(=O)(=O)N4CCCCCC4)ccc32)cc1	0	Validation
TNKS1_ML_3670	COc1ccc(C(=O)C2CCN(CC(=O)NCc3cc4c(c(=O)o3)COCC4)CC2)cc1	1	Validation
TNKS1_ML_3671	C[C@@H](CC(=O)[O-])NC(=O)N1C[C@H](C)C[C@@H](C)C1	0	Validation
TNKS1_ML_3672	COc1cc([C@@H](O[C@@H]2O[C@H](CO)[C@@H](O)[C@H](O)[C@H]2O)[C@@H]2CO[C@H](c3ccc(O)c(OC)c3)[C@H]2CO)ccc1O	0	Validation
TNKS1_ML_3673	Cc1nn(CC(=O)Nc2ccc3c(c2)nc(CN2CCN(C)CC2)n3C)c(=O)c2ccccc12	0	Validation

TNKS1_ML_3674	Cc1nc(C(=O)Nc2ccc(/N=N/c3ccccc3)cc2)ccc1C#N	0	Validation
TNKS1_ML_3675	CC[NH+]1CCN([C@@H](C)CNc2nc(CN3CCOCC3)nc3scc(-c4ccccc4)c23)CC1	0	Validation
TNKS1_ML_3676	O=C1c2ccc(Br)cc2C(=O)N1C[C@H]1CCC[C@H]1O	0	Validation
TNKS1_ML_3677	O=c1c2c3c(sc2[nH]c(=S)n1-c1ccsc1)CCCC3	0	Validation
TNKS1_ML_3678	Cc1ccc(S(=O)(=O)[N-]c2ccc(N3CC[NH2+]CC3)c(C(=O)NC(C)C)c2)cc1Cl	0	Validation
TNKS1_ML_3679	Cc1ccc(NC(=O)CSc2nnc3n(C)c(=O)c4c5c(sc4n23)COC(C)(C)C5)c(C)c1	0	Validation
TNKS1_ML_3680	CCc1nsc(NCC2(O)CCC(C)(C)CC2)n1	0	Validation
TNKS1_ML_3681	Cn1c(=O)c2cc(S(=O)(=O)N[C@@H](C(=O)N3CCc4ccccc4C3)c3ccccc3)ccc2n(C)c1=O	0	Validation
TNKS1_ML_3682	COc1ccccc1CN1C[C@@H]2C[C@@H](c3ccc(C#CC(C)(C)O)cc3)[NH+]3CCC[C@@]23C1=O	0	Validation
TNKS1_ML_3683	Cn1c(N)c(C(=O)COC(=O)c2ncc(Cl)c(Cl)c2Cl)c(=O)n(C)c1=O	0	Validation
TNKS1_ML_3684	C[NH+]1CCC(N2C[C@@H]3C[C@H](C2)c2ccc(NC(=O)COc4ccccc4)c(=O)n2C3)CC1	0	Validation
TNKS1_ML_3685	CC(C)N1CCN(C(=O)N[C@H]2CCC[C@@H](C(=O)[O-])C2)CC1	0	Validation
TNKS1_ML_3686	O=S(=O)(c1cnc[nH]1)N1CCCC[C@@H]1[C@@H]1CCCC[C@@H]1O	0	Validation
TNKS1_ML_3687	CSc1ccc(Nc2nnc(C)c(C)c2C#N)cc1	0	Validation
TNKS1_ML_3688	C[C@H]1CN2CCC[C@@H]2CN1C(=O)Nc1ccc(F)c(F)c1	0	Validation
TNKS1_ML_3689	C[C@@H]1CCC[C@@H](NC(=O)[C@@H]2CCCC[C@@H]2C(=O)[O-])[C@@H]1C	0	Validation
TNKS1_ML_3690	CS(=O)(=O)c1ccc(-c2nc3c(c(=O)[nH]2)COCC3)cc1	1	Validation
TNKS1_ML_3691	Oc1ccc(-c2nc(O)c3cccc(O)c3n2)cc1	1	Validation
TNKS1_ML_3692	CCN(CC)CCn1c2c3c4c(cccc41)C(=O)NN=C3CC(C)(C)C2	1	Validation
TNKS1_ML_3693	CCc1ccc([C@@H]2C(C(=O)c3ccc4c(c3)C[C@H](C)O4)=C([O-])C(=O)N2CCCN2CCOCC2)cc1	0	Validation
TNKS1_ML_3694	C[C@@H]1CCC[C@@H](CNC(=O)N2CCC3(CC2)OCCO3)C1	0	Validation
TNKS1_ML_3695	CCOc1ccc([C@@H]2C(C(=O)c3sc(C)nc3C)=C([O-])C(=O)N2CCCn2cc[nH+]c2)cc1OCC	0	Validation
TNKS1_ML_3696	Cc1c(/C=C2/SC(=S)N(C)C2=O)c(NCc2ccc3c(c2)OCO3)n(C)c(=O)c1C#N	0	Validation
TNKS1_ML_3697	O=C(c1ccc(F)cc1)C1CCN(CC(=O)N(Cc2nc3c(c(=O)[nH]2)COCC3)Cc2ccc(F)cc2F)CC1	1	Validation
TNKS1_ML_3698	N#Cc1ccc(OC(F)F)c(NC(=O)N2CCC[C@H](O)C2)c1	0	Validation
TNKS1_ML_3699	CS(=O)(=O)c1ccc(NC(=O)[C@H]2CCCC[C@H]2c2nc3ccc(Cl)cc3[nH]2)cn1	0	Validation
TNKS1_ML_3700	O=C(N[C@H]1C=CCCC1)[C@H]1CC=CC[C@H]1C(=O)N[C@H]1C=CCCC1	0	Validation
TNKS1_ML_3701	O=C(Nc1nc2c(s1)CN(C(=O)CCC(=O)c1ccccc1)CC2)NC1CCCC1	0	Validation
TNKS1_ML_3702	COc1ccc(C(=O)C2CCN([C@@H]3CCN(Cc4nc5c(c(=O)[nH]4)COCC5)C3=O)CC2)cc1C	1	Validation
TNKS1_ML_3703	COc1ccccc1N1CCN(c2nc3c(c(=O)[nH]2)C[S+]([O-])CC3)CC1	1	Validation
TNKS1_ML_3704	O=C(c1ccc(F)cc1)C1CCN(CC(=O)N(Cc2nc3c(c(=O)[nH]2)COCC3)C[C@@H]2CCCO2)CC1	1	Validation
TNKS1_ML_3705	CSc1ccc(N2C[C@H](C(=O)Nc3cccc(OCCn4ccnc4)c3)CC2=O)cc1	0	Validation
TNKS1_ML_3706	Cc1ccc(S(=O)(=O)Nc2ccc(C[NH+]3CCN(c4ccccn4)CC3)cc2)cc1C	0	Validation
TNKS1_ML_3707	Cc1cc2ccccc2nc1N[C@@H](C)c1nncn1C	0	Validation
TNKS1_ML_3708	Cc1ccc(C)c(-n2nc3c(c2N)C(C)(C)CC3)c1	0	Test

TNKS1_ML_3709	Cc1cccc2c1sc1nncn12	1	Test
TNKS1_ML_3710	Cc1nc(-c2ccc(Cl)c(S(=O)(=O)N3CCC[C@@H](C(=O)NCc4ccc(Cl)cc4)C3)c2)no1	0	Test
TNKS1_ML_3711	Cc1nn(-c2ccccc2)c2ncc(C(=O)Nc3ccc4c(c3)OCCO4)c(Cl)c12	0	Test
TNKS1_ML_3712	Cn1c(=O)n([C@H]2C[C@H](c3nnc(-c4ccncn4)n3-c3ccccc3Cl)C2)c2ccc(C#N)cc21	1	Test
TNKS1_ML_3713	C[C@@H]1O[C@H](C)[C@H](c2n[nH]c(C3CCCCC3)n2)[C@H]1C	0	Test
TNKS1_ML_3714	CCCCN1C(=O)C2=NN=C(c3cc(C)cc(C)c3[O-])[C@H]2[C@@H]1c1ccc(O)c(OCC)c1	0	Test
TNKS1_ML_3715	C[C@@H]1CCCN2C(=[NH2+])[C@@H](c3ccc4c(c3)CCO4)N=C12	0	Test
TNKS1_ML_3716	CCOC(=O)c1nn(Cc2cccc[nH+]2)c2c1CN(C(=O)c1ccc3[nH]ccc3c1)CC2	0	Test
TNKS1_ML_3717	Cc1nc([C@@H](C(C)C)[C@H](C)Cl)cc2n[nH]c(=O)n12	0	Test
TNKS1_ML_3718	Cc1sc2ncn(CCC(=O)N[C@H](C)c3ccc(-n4cc[nH+]c4)cc3)c(=O)c2c1C	0	Test
TNKS1_ML_3719	Clc1ccccc1/N=N/Nc1ccccc1Cl	0	Test
TNKS1_ML_3720	Nc1ccncc1[C@@H]1CCCc2cccnc21	0	Test
TNKS1_ML_3721	COC(=O)NC(=O)c1ccsc1NC(=O)c1ccc(S(=O)(=O)N2C[C@H](C)O[C@@H](C)C2)cc1	0	Test
TNKS1_ML_3722	CC(=O)N1CCc2cc(S(=O)(=O)Nc3ccc(Cl)c(S(=O)(=O)N4CCCCC4)c3)ccc21	0	Test
TNKS1_ML_3723	COc1ccc(-n2c(C)nnc2SCc2nc(-c3ccc(OC)c(OC)c3)no2)cc1	1	Test
TNKS1_ML_3724	Cc1cccc(N(CC(=O)N/N=C\c2cc(C)n(-c3ccc(Br)cc3)c2C)S(C)(=O)=O)c1	0	Test
TNKS1_ML_3725	N/C(=N/O)[C@H]1CCCN1C(=O)C1CCCCCCC1	0	Test
TNKS1_ML_3726	CCn1c(CSc2nc3ccccc3s2)nnc1S[C@H](C(=O)NC(N)=O)C(C)C	0	Test
TNKS1_ML_3727	C[C@@H]1CC[C@](C#N)(C2(O)CCCCC2)C1	0	Test
TNKS1_ML_3728	C[NH2+][C@H]1COC[C@H]1/C([O-])=N/c1c(Cl)cc(Cl)c2nsnc12	0	Test
TNKS1_ML_3729	CCCCn1cc(S(=O)(=O)c2ccc(C)cc2)c(=O)c2cc(F)c(N3CC[NH+](CCC)CC3)cc21	0	Test
TNKS1_ML_3730	CC[C@H](Sc1cc(C)ncn1)C(=O)Nc1ccc(S(=O)(=O)N2CCCC2)cc1	0	Test
TNKS1_ML_3731	C[C@H]1CCC[C@@H](C)N1C(=O)CSc1nnc2c(n1)[nH]c1ccc(Br)cc12	0	Test
TNKS1_ML_3732	C=CCOc1ccc(C(O)=C2C(=O)C(=O)N(CCCn3cc[nH+]c3)[C@@H]2c2cccc(Cl)c2)cc1	0	Test
TNKS1_ML_3733	C[C@H]1CCCC[C@@H]1N(C)C(=O)c1ccc(S([NH-])(=O)=O)o1	0	Test
TNKS1_ML_3734	Cc1c(Cl)cccc1N(CC(=O)Nc1ccc(S(=O)(=O)N2CCOCC2)cc1)S(C)(=O)=O	0	Test
TNKS1_ML_3735	CNC(=O)NC(=O)[C@H](C)Sc1nc2sc(C)c(C)c2c(=O)n1C1CCCC1	0	Test
TNKS1_ML_3736	Cn1c(CN2CCN(S(=O)(=O)c3ccc(C(N)=O)o3)CC2)nc2cc(Cl)ccc21	0	Test
TNKS1_ML_3737	Cc1[nH]c2ccccc2c1C(=O)COC(=O)c1ccc(F)c(S(=O)(=O)N2CCOCC2)c1	0	Test
TNKS1_ML_3738	CS(=O)(=O)c1cccc(S(=O)(=O)NCC[NH+]2CCN(c3[nH+]ccc4c3NCCC4)CC2)c1	0	Test
TNKS1_ML_3739	CN[C@]1(C#N)CCCC1(C)C	0	Test
TNKS1_ML_3740	CCOc1ccc(-c2nnc([C@H]3C[C@H](NC(=O)c4ccnc5cc(F)cnc45)C3)n2-c2ccncc2)nc1	1	Test

TNKS1_ML_3741	CCCCCc1nc2sc3c(c2c(=O)n1N)CCC3	0	Test
TNKS1_ML_3742	O=C(Cn1cc(S(=O)(=O)c2ccccc2)c2ccccc21)NCCCN1CCCC1=O	0	Test
TNKS1_ML_3743	N#Cc1cccc(S(=O)(=O)N2CCN(Cc3ccco3)C[C@@H]2C(=O)NCc2ccccc2)c1	0	Test
TNKS1_ML_3744	O=C(CNC(=O)c1ccccn1)N/N=C/c1cc(Br)ccc1OC(=O)/C=C/c1ccco1	0	Test
TNKS1_ML_3745	C=CCn1c(=O)n(CC=C)c(=O)n(CC(=O)N[C@H](c2ccc(C)cc2)c2cccs2)c1=O	0	Test
TNKS1_ML_3746	Cc1c(NS(=O)(=O)c2ccc(CN3C(=O)c4ccccc4C3=O)cc2)c(=O)n(-c2ccccc2)n1C	0	Test
TNKS1_ML_3747	Brc1cccn2nc(N[C@@H]3C[C@H]4CC[C@@H]3C4)nc12	0	Test
TNKS1_ML_3748	C[C@@H]1CC(Nc2ncc(N)cc2Br)C[C@@H](C)C1	0	Test
TNKS1_ML_3749	COc1ccc([C@@H]([NH3+])CC(=O)N2CCN(c3nc(-c4ccccc4)c4cc(Br)ccc4n3)CC2)cc1OC	0	Test
TNKS1_ML_3750	COc1ccc([C@H](C#N)N2CCN(C(=O)C[C@H](NC(C)=O)c3ccccc3)CC2)cc1	0	Test
TNKS1_ML_3751	CC[C@@H](C)c1cnc2c(c1SCC(=O)Nc1nc3ccc(C)cc3s1)c(=O)n(C)c(=O)n2C	0	Test
TNKS1_ML_3752	CC(C)C(=O)N1CCc2c(nc3cc(C4CC[NH+](C[C@@H]5CC=CCC5)CC4)nn3c2[O-])C1	0	Test
TNKS1_ML_3753	CC1CC[NH+]([C@H](c2cc3cc4c(cc3nc2O)OCO4)c2nnnn2C[C@H]2CCCO2)CC1	0	Test
TNKS1_ML_3754	Cc1cc(C(=O)NCc2ccc(Cn3ccnc3)cc2)c(C)n1-c1nccs1	0	Test
TNKS1_ML_3755	O=C1Nc2cccc(Br)c2C12CCN(c1nc3c(c(=O)[nH]1)CCCC3)CC2	1	Test
TNKS1_ML_3756	O=C(Nc1nnc2n1CCS2)C1CCC1	1	Test
TNKS1_ML_3757	Cc1ccc(S(=O)(=O)N2CCC[C@H]2C(=O)NCc2ccccc2Cn2cc[nH+]c2)s1	0	Test
TNKS1_ML_3758	CC[C@H](N[C@H](C)c1cc(C)oc1C)/C(N)=N/O	0	Test
TNKS1_ML_3759	O=C(Nc1ccc(Br)cn1)c1cc([N+](=O)[O-])ccc1N1CCCCC1	0	Test
TNKS1_ML_3760	CC(C)n1c[nH+]cc1-c1nc2ccc(Cl)cn2c1N	0	Test
TNKS1_ML_3761	O=c1cc(-c2ccccc2)oc2ccccc12	1	Test
TNKS1_ML_3762	Cc1nn(C(C)C)c(N(C)c2cccc(C#N)c2)c1N	0	Test
TNKS1_ML_3763	CCc1nnc(NC(=O)[C@H](C)Sc2nnc(CNC(=O)c3ccc4c(c3)OCO4)n2CC)s1	0	Test
TNKS1_ML_3764	COc1ccccc1N1CCC(c2nc3c(c(=O)[nH]2)CCCC3)CC1	1	Test
TNKS1_ML_3765	COc1ccc(-n2c(SCCCN3C(=O)c4cccc5cccc(c45)C3=O)nnc2-c2ccncc2)cc1	1	Test
TNKS1_ML_3766	O=C(N[C@H]1C[C@H](c2nnc(-c3ccccn3)n2-c2ccccc2F)C1)c1ccc(F)cn1	1	Test
TNKS1_ML_3767	Cc1ccc([C@@H]2CC(c3cccs3)=NN2C(=O)CSc2nnc(CNC(=O)c3ccco3)n2C)cc1	0	Test
TNKS1_ML_3768	COc1cc([C@@H]2c3c(oc4ccc(F)cc4c3=O)C(=O)N2c2nncs2)cc(Br)c1O	0	Test
TNKS1_ML_3769	CC(=O)Nc1ccc(S(=O)(=O)N[C@@H](C)C(=O)N2CCN(Cc3cccc(C)c3)CC2)cc1	0	Test
TNKS1_ML_3770	N#C[C@H]1CCCCC[C@@H]1Nn1cccc1	0	Test
TNKS1_ML_3771	CC(C)OCCCNC(=O)[C@@]1(C)Cn2cnc(C(=O)Nc3cc(F)ccc3F)c2C(=O)N1C1CC1	0	Test
TNKS1_ML_3772	Cc1ccc(C)c(OC(=O)c2cc3ccccc3oc2=O)c1C	0	Test
TNKS1_ML_3773	CCCNC(=O)[C@H]1CCCN1C(=O)c1cccc(NS(=O)(=O)c2ccc(C)cc2)c1	0	Test
TNKS1_ML_3774	C[NH+]1CCN([C@H](CNS(=O)(=O)c2ccccc2Br)c2ccc3c(c2)OCO3)CC1	0	Test
TNKS1_ML_3775	C[C@H]1Cc2cc(-c3cc(C(=O)Nc4ccc(S(=O)(=O)N5CCOCC5)cc4)no3)ccc2O1	0	Test
TNKS1_ML_3776	COc1ccccc1C(=O)NNC(=O)CCN1C(=O)/C(=C\c2ccccc2)S	0	Test

	C1=S		
TNKS1_ML_3777	CN(C)S(=O)(=O)c1ccc(-c2csc(NC(=O)/C=C\c3ccsc3)n2)cc1	0	Test
TNKS1_ML_3778	CCCn1c(CCC(=O)Nn2c[nH+]c3ccccc32)[nH+]c2cc(S(=O)(=O)N3CCCC3)ccc21	0	Test
TNKS1_ML_3779	Cc1ccccc1Cn1ccc(NC(=O)CCS(=O)(=O)c2nc(-c3ccc4c(c3)OCO4)cc(C(F)(F)F)n2)n1	0	Test
TNKS1_ML_3780	CCCOc1ccccc1[C@H]1SCC(O)=Nc2c1c(O)nn2C1CCCCC1	0	Test
TNKS1_ML_3781	CCO[C@H](C[C@@](Oc1ccccc1)(C(=S)Nc1ccccc1)n1nnc2ccccc21)c1ccccc1	0	Test
TNKS1_ML_3782	Cc1cc(C)n2nc(C(=O)Nc3cc(Cl)c(C)cc3Br)nc2n1	0	Test
TNKS1_ML_3783	Nc1ccccc1-c1cnn(-c2ncncc2Cl)c1	0	Test
TNKS1_ML_3784	CC(C)Cc1ccc(S(=O)(=O)N(CCN2CCCCC2)CC(=O)N2CCNCC2)cc1	0	Test
TNKS1_ML_3785	COc1ccc2c(=O)[nH]c3sccc3c2c1	1	Test
TNKS1_ML_3786	COC(=O)c1ccc([C@@H]2C(C(=O)c3ccc4c(c3)C[C@H](C)O4)=C(O)C(=O)N2c2nc(C)c(C(C)=O)s2)cc1	0	Test
TNKS1_ML_3787	Cc1cc(C(=O)Nc2ccc(Br)cn2)sc1C	0	Test
TNKS1_ML_3788	CCn1c(CNc2ccc(N3CCCC3=O)c(C)c2)[nH+]c2cc(S(=O)(=O)N3CCCCC3)ccc21	0	Test
TNKS1_ML_3789	CS(=O)(=O)[N-]c1cccc(C(N)=S)c1	0	Test
TNKS1_ML_3790	C[C@H]1CSCCN1c1nc2ccc(N)cc2o1	0	Test
TNKS1_ML_3791	c1ccc2c3c([nH]c2c1)[C@@H](c1nc(C2CC2)n[nH]1)CCC3	0	Test
TNKS1_ML_3792	Cc1cc(Oc2ccccc2I)nc(N)n1	0	Test
TNKS1_ML_3793	CC(C)(C)OC(=O)N1CCC[C@]2(C)[C@@H](O)CCC[C@@H]12	0	Test
TNKS1_ML_3794	C#CCN1C(=O)[C@@]2(CC(O)=Nc3c2cnn3Cc2ccccc2Cl)c2ccccc21	0	Test
TNKS1_ML_3795	NC(=O)Cn1c(CSc2nc(C(F)(F)F)nc3ccccc23)nc2ccccc2c1=O	0	Test
TNKS1_ML_3796	CN(C)CC(=O)Nc1ccc2[nH]c(=O)c3ccccc3c2c1	1	Test
TNKS1_ML_3797	Cc1oc(NC(=O)[C@H](C)[NH+]2CCc3cc(F)ccc3C2)c(C#N)c1C	0	Test
TNKS1_ML_3798	Cc1ccc(C(=O)C2=C([O-])C(=O)N(CCCn3cc[nH+]c3)[C@@H]2c2ccncc2)cc1	0	Test
TNKS1_ML_3799	O=C(Nc1nnc2n1CCS2)C1CC1	1	Test
TNKS1_ML_3800	Cc1nn(C)c(C)c1S(=O)(=O)NCc1ccc(C(=O)c2cccs2)s1	0	Test
TNKS1_ML_3801	O=C(CNS(=O)(=O)c1ccc(Cl)c(Cl)c1)N1CCNCC1	0	Test
TNKS1_ML_3802	O=C(Nc1cc(S(=O)(=O)N2CCOCC2)ccc1N1CCCCC1)c1cc(Cl)ccc1Cl	0	Test
TNKS1_ML_3803	CCCC[NH+]1CCN(C(=O)c2cn(CC)nc2S(=O)(=O)N2CCN(c3cccc(Cl)c3)CC2)CC1	0	Test
TNKS1_ML_3804	Cc1oc(C)c(-c2nc3cc(N)ccc3o2)c1C	0	Test
TNKS1_ML_3805	Cc1ccc(-c2nc(C)sc2CC(=O)NCCC[NH+]2CCN(c3ncccn3)CC2)cc1	0	Test
TNKS1_ML_3806	CO[C@@H]1C[C@H](O[C@@H]2CC[C@@]3(C)[C@H]4CC[C@@]5(C)[C@@H](C6=CC(=O)OC6)[C@@H](OC(C)=O)C[C@]5(O)[C@@H]4CC[C@@H]3C2)O[C@@H](C)[C@@H]1O	0	Test
TNKS1_ML_3807	Cc1csc([C@H](C)NC(=O)N2CC[C@](C)(C(=O)[O-])C2)n1	0	Test
TNKS1_ML_3808	CCN(C(=O)N1CCC(C(=O)c2ccc3c(cnn3C)c2)CC1)c1ccc(C(=O)O)cc1	1	Test
TNKS1_ML_3809	CC[C@@H]1CC[C@@H](c2noc(-c3sc(N)cc3C)n2)C1	0	Test
TNKS1_ML_3810	C[C@]12CC[C@@H]3c4ccc(O)cc4CC[C@H]3[C@@H]1CC/C2=N\NC(=O)CC#N	0	Test
TNKS1_ML_3811	COc1ccc(/C=C\c2onc(C)c2NC(C)=O)cc1S(=O)(=O)[N-]c1ccc2c(c1)OCCO2	0	Test
TNKS1_ML_3812	O=C(N[C@H]1C[C@H](c2nnc(-c3cnccn3)n2-c2ccccc2F)C1)c1ccccn1	1	Test
TNKS1_ML_3813	Cc1ccc(N)cc1N1CCCCS1(=O)=O	0	Test

TNKS1_ML_3814	CCCc1cnc2c(c1NOCC(=O)Nc1c(C)cc(C)cc1C)c(=O)n(C)c(=O)n2C	0	Test
TNKS1_ML_3815	CSc1cc2c(cc1NC(=O)N[C@@H]1CCCC[C@@H]1C)OCCO2	0	Test
TNKS1_ML_3816	Cc1cc(C)n2ncc(C(=O)N[C@@H]3CCCc4c3cnn4Cc3ccc(Cl)cc3)c2n1	0	Test
TNKS1_ML_3817	O=C1CCCc2ccc([N+](=O)[O-])cc21	0	Test
TNKS1_ML_3818	COc1cccc([C@H](CNC(=O)c2cc(S(=O)(=O)NC(C)(C)C)ccc2C)[NH+]2CCCC2)c1	0	Test
TNKS1_ML_3819	O=C(CCCc1ccccc1)Nc1nnc2n1CCS2	1	Test
TNKS1_ML_3820	CSc1nnc2c(n1)O[C@@H](c1ccc(N(C)C)cc1)Nc1ccc(Br)cc1-2	0	Test
TNKS1_ML_3821	Nc1c(I)cnn1Cc1cc2ccccc2o1	0	Test
TNKS1_ML_3822	O=C(CCc1ccc(O)cc1)N(Cc1nc2c(c(=O)[nH]1)COCC2)Cc1cccs1	1	Test
TNKS1_ML_3823	O=c1[nH]c(N2CCC3(CC2)CN(CCO)c2cc(F)cc(F)c23)nc2c1CCCC2	1	Test
TNKS1_ML_3824	O=C(c1cc(NS(=O)(=O)c2ccc(F)cc2)cc(C(=O)N2CCOCC2)c1)N1CCOCC1	0	Test
TNKS1_ML_3825	CC(C)n1ccc(Cn2ccc3ccc(C(=O)[O-])cc32)n1	0	Test
TNKS1_ML_3826	O=C1CCC[C@H]1[C@@H]1CCCCC[C@@H]1O	0	Test
TNKS1_ML_3827	Nc1noc(-c2ccc(Cl)c(Cl)c2)c1Br	0	Test
TNKS1_ML_3828	CN(C(=O)c1cc(N)cn1C)C1CCCCCC1	0	Test
TNKS1_ML_3829	COc1cccc(N2C(=O)CS[C@H]2c2cccc([N-]S(=O)(=O)c3ccc(Br)cc3)c2)c1	0	Test
TNKS1_ML_3830	Cc1n[nH]c(C)c1CCC(=O)Nc1nnc2n1CCS2	1	Test
TNKS1_ML_3831	COC(=O)c1ccc([C@@H]2[C@H](C(=O)c3c(C)[nH]c(C(=O)OC)c3C)C(=O)C(=O)N2CC[NH+](C)C)cc1	0	Test
TNKS1_ML_3832	Cc1ncccc1[N-]S(=O)(=O)c1[nH+]cn(C)c1Cl	0	Test
TNKS1_ML_3833	C[C@H]1CC(C)(C)C[C@@](Nc2ccccc2Br)(C(=O)[O-])C1	0	Test
TNKS1_ML_3834	Cn1c(O)c(C(=O)C[C@H](Nc2ccc[nH+]c2)c2ccc3c(c2)OCO3)c(=O)c2ccccc21	0	Test
TNKS1_ML_3835	COc1ccc(-c2nc3c(c(=O)[nH]2)COCC3)cc1	1	Test
TNKS1_ML_3836	COc1cc([C@@H]2C(C(=O)OC(C)C)=C(C)NC3=C2C(=O)CCC3)cc(Cl)c1O	0	Test
TNKS1_ML_3837	COc1cc(NC(=O)C2=Cc3cc(F)ccc3OC2)ccc1NC(C)=O	0	Test
TNKS1_ML_3838	COc1ccc(NC(=O)[C@@H]2c3cc(OC)c(OC)cc3C(=O)N(C)[C@@H]2c2cccs2)cc1OC	0	Test
TNKS1_ML_3839	Cc1cc(C)n(-c2cc(N3CCC[C@@H](C(=O)NCCn4nc(-c5ccc(F)cc5)ccc4=O)C3)ncn2)n1	0	Test
TNKS1_ML_3840	O=C([O-])[C@H]1CCCN(c2cc(-c3cc[nH+]cc3)ncn2)C1	0	Test
TNKS1_ML_3841	CN(C[C@H]1CC=C(Cl)S1)C(=O)CSc1nnc(C2=c3ccccc3=[NH+]C2)n1C1CC1	0	Test
TNKS1_ML_3842	Cc1[nH+]ccn1-c1ccc(CNC(=O)/C=C/c2cn(-c3ccccc3)nc2-c2ccc[nH+]c2)cc1F	0	Test
TNKS1_ML_3843	O=C(NCCCCc1ccccc1)c1cc(S(=O)(=O)N2CCOCC2)ccc1N1CCOCC1	0	Test
TNKS1_ML_3844	COc1ccc([C@H](c2nnnn2C(C)(C)C)N2CCN(c3nc4ccccc4s3)CC2)cc1OC	0	Test
TNKS1_ML_3845	N#Cc1ccccc1Cn1c(=O)n(C[C@H]2CC[C@H](C(=O)Nc3ccncc3)CC2)c(=O)c2ccccc21	1	Test
TNKS1_ML_3846	Cc1nn(C)c(Nc2c(Cl)cc(Cl)c3nsnc23)c1C#N	0	Test
TNKS1_ML_3847	CCc1nn(C)c2c1[nH]c(=S)n2[C@@H]1CCC[C@@H](C)C1	0	Test
TNKS1_ML_3848	Cc1ccc(-c2nnc(SCC(=O)Nc3ncc(C(F)(F)F)cc3Cl)n2N)cc1	0	Test
TNKS1_ML_3849	Cc1cccc(N2C(=O)NC(=O)C(CCc3cc[nH+]cc3)(CCc3cc[nH+]cc3)C2=O)c1C	0	Test
TNKS1_ML_3850	Cn1cc(Cn2c(N)[nH+]c3cc(Cl)ccc32)cn1	0	Test
TNKS1_ML_3851	Cn1c[nH+]c(S(=O)(=O)[N-	0	Test

	]c2ccc([NH+]3CCCCC3)cc2)c1Cl		
TNKS1_ML_3852	CC1COC(C)(C(C)C)OC1	0	Test
TNKS1_ML_3853	O/N=C/c1ccc(N2CCCCC2)s1	0	Test
TNKS1_ML_3854	C[C@@H]1CN(c2ncnc3c2cnn3CCNC(=O)c2cc3c4c(c2)CCN4CCC3)C[C@@H](C)O1	0	Test
TNKS1_ML_3855	C[C@H]1C(=O)N[C@@](C)(C2CC2)C(=O)N1C1CCCCCC1	0	Test
TNKS1_ML_3856	O=C1NN=C2CCCc3[nH]c4cccc1c4c32	1	Test
TNKS1_ML_3857	Cn1cc(Cc2sc(N)nc2C2CC2)cn1	0	Test
TNKS1_ML_3858	CC(C)Nc1cc(-c2ccc(F)cc2Cl)[nH]n1	0	Test
TNKS1_ML_3859	CCOC(=O)c1nn(Cc2cccc[nH+]2)c2c1CN(C(=O)Cc1c(C)[nH]c3c(F)cccc13)CC2	0	Test
TNKS1_ML_3860	O=C1N/C(=N\c2ccc(F)cc2)S/C1=C\c1ccc(S[C@H]2N=C3C=CC=C[C@H]3N2)o1	0	Test
TNKS1_ML_3861	CCn1/c(=N/C(=O)c2ccc(S(=O)(=O)N3C[C@H](C)O[C@H](C)C3)cc2)sc2cc3c(cc21)OCCO3	0	Test
TNKS1_ML_3862	Cc1ccc([C@@H](C)N2CC(=O)N[C@@H](C(C)(C)C)C2=O)o1	0	Test
TNKS1_ML_3863	Cc1noc(/C=C/c2ccco2)c1S(=O)(=O)N1CCC(C(=O)N[C@H]2CCCc3ccccc32)CC1	0	Test
TNKS1_ML_3864	C[C@]1(CNc2ccnc(NN)n2)CCCS1	0	Test
TNKS1_ML_3865	Cc1nn(C)c(C)c1NC(=O)[C@@H](C)Sc1[nH]c(-c2cccs2)c(-c2cccs2)[nH+]1	0	Test
TNKS1_ML_3866	CNc1cc(S(N)(=O)=O)ccc1Cl	0	Test
TNKS1_ML_3867	C[C@H]1C[C@@H](OC[C@H](O)C[NH+]2CCN(CCN3C(=O)c4cccc5cccc(c45)C3=O)CC2)CC(C)(C)C1	0	Test
TNKS1_ML_3868	C[C@@H]1C[C@@H](C)CN(C(=O)C(=O)Nc2c3c(nn2-c2ccc(Cl)cc2)CS(=O)(=O)C3)C1	0	Test
TNKS1_ML_3869	CC(C)c1ccc(NC(=O)O[C@@H]2CO[C@@H]3[C@@H](NS(=O)(=O)c4ccc(Cl)cc4)CO[C@H]23)cc1	0	Test
TNKS1_ML_3870	O=C(CN1CCC(C(=O)c2ccc(OC3CCOCC3)cc2)CC1)NCc1nc2c(c(=O)[nH]1)COCC2	1	Test
TNKS1_ML_3871	COc1ccc(C(=O)C2CCN([C@H]3CCN(Cc4nc5ccccc(=O)c5[nH]4)C3=O)CC2)cc1	1	Test
TNKS1_ML_3872	COc1cccc([C@H]2Nc3ccc(N(C)C(C)=O)cc3[C@H]3C=CC[C@H]32)c1O	0	Test
TNKS1_ML_3873	CN(C)C(=O)[C@@]1(Cc2cccc(-c3ccccc3)c2)CCCN(C(=O)Cc2c[nH]nc2O)C1	0	Test
TNKS1_ML_3874	CN(C)c1cc(F)c2c(c1)N(C)C(=O)C21CCN(c2nc3c(c(=O)[nH]2)CCCN3C)CC1	1	Test
TNKS1_ML_3875	Cc1sc2nc(SCC(=O)NCCN3C(=O)CSC3=O)n(C(C)C)c(=O)c2c1C	0	Test
TNKS1_ML_3876	Cc1ccc2ccc([C@@H](Nc3ccc(Cl)cn3)c3cccc[nH+]3)c(O)c2n1	0	Test
TNKS1_ML_3877	CCCn1cc(C(=O)N2CCCC[C@@H]2c2nc([O-])c3c(n2)CC[NH+](C(C)C)C3)c(C)n1	0	Test
TNKS1_ML_3878	CC(C)n1ncc2c(C(=O)NC(C)(C)C#N)cc(-c3ccco3)nc21	0	Test
TNKS1_ML_3879	CCn1cc(Cl)c(C(=O)N2N=C(c3ccc4c(c3)OCCO4)C[C@@H]2c2ccccc2O)n1	0	Test
TNKS1_ML_3880	CCN(CC)C(=O)c1cc(C)nc2c1c(=O)n(CC(=O)Nc1ccc(C)c(C)c1)c(=O)n2C	0	Test
TNKS1_ML_3881	O=C(c1ccc(OC(F)(F)F)cc1)C1CCN(CC(=O)N(Cc2nc3c(c(=O)[nH]2)COCC3)CC(F)F)CC1	1	Test
TNKS1_ML_3882	COc1cc(-c2nnc(/C=C/c3nnc(-c4ccc(S(C)(=O)=O)cn4)n3-c3ccccc3Cl)o2)ccn1	1	Test
TNKS1_ML_3883	Cc1ccsc1[C@@H](C)Nc1ccc(Cl)c(C(=O)[O-])c1	0	Test
TNKS1_ML_3884	COc1ccc(CC/N=C2/C(=O)C(=O)[C@H]2N2CCN(c3cccc[nH+]3)CC2)cc1	0	Test

TNKS1_ML_3885	C[C@H](C(=O)Nc1cccc(S(N)(=O)=O)c1)N1CCN(S(=O)(=O)c2cccs2)CC1	0	Test
TNKS1_ML_3886	CCc1ccccc1NC(=O)[C@@]12CC[C@@H](c3nc(C#N)c(C#N)nc31)C2(C)C	0	Test
TNKS1_ML_3887	Cc1sc(NC(=O)[C@]2(C)CC[NH2+]C2)c(C#N)c1C	0	Test
TNKS1_ML_3888	Cc1nc(C)c(C(=O)N(c2ccc3c(c2)OCCO3)[C@@H](C(=O)NCc2ccco2)c2ccccc2)s1	0	Test
TNKS1_ML_3889	O=c1c2c3c(sc2ncn1CCn1ccnc1)C[C@@H](NCc1ccc(F)cc1)CC3	0	Test
TNKS1_ML_3890	Cc1ccc(-c2nc3ccccc3c(=O)[nH]2)cc1	1	Test
TNKS1_ML_3891	COc1ccc([C@H]2[NH2+]C[C@@]3(C)CN2C[C@@H](C)[C@H]3O)cc1OC	0	Test
TNKS1_ML_3892	Cc1sc2nc(C(C)C)n([C@@H]3CCCN(C(=O)c4[nH]nc5c4CCC5)C3)c(=O)c2c1C	0	Test
TNKS1_ML_3893	Cn1ncc2c(Sc3ccc(O)cc3)ncnc21	0	Test
TNKS1_ML_3894	COc1cc([C@H]2C(C#N)=C(SCC(=O)Nc3cc(Cl)cc(Cl)c3)NC3=C2C(=O)CCC3)cc(OC)c1OC	0	Test
TNKS1_ML_3895	O=c1cc(-c2cccc(O)c2)oc2cc(O)ccc12	1	Test
TNKS1_ML_3896	O=C(Nc1ccc2c(c1)CCC2)c1ccc(Cl)cc1[N+](=O)[O-]	0	Test
TNKS1_ML_3897	CCCCSc1nc(SCC(=O)Nc2nccs2)c2c3c(sc2n1)CO[C@@](C)(CC)C3	0	Test
TNKS1_ML_3898	O=C(NCC#Cc1ccccc1)N[C@@H]1CCOC2(CCCCC2)C1	0	Test
TNKS1_ML_3899	O=C(NCC1CCN(C(=O)c2cnccn2)CC1)c1ccc(S(=O)(=O)N2CCCCC2)cc1	0	Test
TNKS1_ML_3900	COc1ccc(C2(CNC(=O)c3ccc(NC(=O)c4ccco4)cc3Cl)CCOCC2)cc1	1	Test
TNKS1_ML_3901	[H]/N=c1/c(S(=O)(=O)c2ccccc2)cc2c(=O)n3cccc(C)c3nc2n1CCc1ccc(OC)c(OC)c1	0	Test
TNKS1_ML_3902	O=C(CCc1nc2cc(Br)cnc2[nH]1)NC[C@@H]1CCC[NH+]2CCCC[C@@H]12	0	Test
TNKS1_ML_3903	O=C1Nc2cc(F)ccc2C12CCN(c1nc3c(c(=O)[nH]1)CCCC3)CC2	1	Test
TNKS1_ML_3904	CCn1c(=O)c(=O)n(C[C@@H](O)COCc2ccccc2Cl)c2cc(S(=O)(=O)N(C)C)ccc21	0	Test
TNKS1_ML_3905	COc1c(C)cccc1C(=O)NC(=S)Nc1cc2nn(-c3ccc(C)cc3)nc2cc1Cl	0	Test
TNKS1_ML_3906	COc1ccc([C@@H]2C3=C(C[C@H](c4cccs4)CC3=O)N[C@@H]3N=NC(C)=C23)cc1O	0	Test
TNKS1_ML_3907	Cn1c(SCc2nnc(C(=O)NCc3ccc(F)cc3)s2)n[nH+]c1[C@H]1CCS(=O)(=O)C1	0	Test
TNKS1_ML_3908	O=C(C[C@H]1CSc2nc3c(c(=O)n21)CCC3)Nc1ccccc1Cc1nc(-c2cc[nH+]cc2)no1	0	Test
TNKS1_ML_3909	CCOC(=O)[C@H]1CCCN(C(=O)CN(C)C(=O)[C@H](C)NC(=O)c2cccc(Cl)c2Cl)C1	0	Test
TNKS1_ML_3910	COc1ccc2c(c1)sc1nncn12	1	Test
TNKS1_ML_3911	O=C(CCCn1nnc2cc(S(=O)(=O)N3CCCCC3)ccc21)NCCc1c[nH]c[nH+]1	0	Test
TNKS1_ML_3912	COc1cc(N[C@H](C)c2c(C)noc2C)ncn1	0	Test
TNKS1_ML_3913	CCc1cc2c(c(C(C)(C)C)c1)OCCNC2	0	Test
TNKS1_ML_3914	O=c1[nH]c(-c2ccc(Cl)cc2)nc2ccccc12	1	Test
TNKS1_ML_3915	C[C@H]1CCCC[C@@H]1NC(=O)[C@]1(C)Cn2c(cc3occc32)C(=O)N1CC[NH+]1CCOCC1	0	Test
TNKS1_ML_3916	O=C(Nc1nc(-c2ccc[nH+]c2)cs1)[C@H]1CCCN(S(=O)(=O)c2ccc(F)cc2)C1	0	Test
TNKS1_ML_3917	Cc1nc(C[NH3+])sc1-c1nc(C2CCC(C)(C)CC2)no1	0	Test
TNKS1_ML_3918	Cc1c(N2C(=O)CS[C@H]2c2ccccc2O)c(=O)n(-c2ccccc2)n1C	0	Test

TNKS1_ML_3919	C[C@@H](NC(=O)c1ccc(Br)cc1)C(=O)Nc1ccc2c(c1)OC(F)(F)O2	0	Test
TNKS1_ML_3920	COc1nc2ccccc2cc1[C@H]1CC(=O)Nc2n[nH]c(C3CCC3)c21	0	Test
TNKS1_ML_3921	CCn1c(=O)c(-c2noc(C(=O)N[C@H](C)CCc3ccccc3)n2)nn(-c2ccc(F)cc2)c1=O	0	Test
TNKS1_ML_3922	COc1cc(NC(=O)C2CCN(c3nc(Cl)nc4[nH+]c[nH]c34)CC2)cc(OC)c1OC	0	Test
TNKS1_ML_3923	C[NH2+][C@H]1CCCc2nc(-c3ncn[nH]3)sc21	0	Test
TNKS1_ML_3924	O=C(NC[C@H]1CCCO1)C1CCN(c2[nH+]cccc2-c2nc(-c3cccc(F)c3)no2)CC1	0	Test
TNKS1_ML_3925	C[C@@H]1CCC[C@@H](Nc2ccc(-n3nccn3)cc2)C1	0	Test
TNKS1_ML_3926	O=C(CCc1ccco1)Nc1nnc2n1CCS2	1	Test
TNKS1_ML_3927	CS(=O)(=O)c1ccc(-c2nnc([C@H]3C[C@H](NC(=O)c4cccc5cccnc45)C3)n2-c2ccccc2F)nc1	1	Test
TNKS1_ML_3928	C[C@H]1CC[C@H](Nc2nc3ccc(Cl)cn3n2)[C@@H]1C	0	Test
TNKS1_ML_3929	Cc1ccc(C)c(/C(O)=C2\C(=O)C(=O)N(c3nnc(C)s3)[C@H]2c2ccc(O)cc2)c1	0	Test
TNKS1_ML_3930	COc1cc(/C=N/N=C2\NC(=O)[C@H](CC(=O)Nc3ccccc3)S2)cc(Cl)c1OC	0	Test
TNKS1_ML_3931	COC(=O)[C@@H]1[NH+]=c2ccc(Cl)cc2=C1NC(=O)[C@H](C)N1CCN(c2cccc(C(F)(F)F)c2)CC1	0	Test
TNKS1_ML_3932	Cn1nc(C(=O)NCCn2c[nH+]c3ccccc32)cc1NC(=O)Cc1ccccc1	0	Test
TNKS1_ML_3933	O=C(C1COC1)N1CCC(C2Cc3[nH]c(=O)c4ccccc4c3CO2)CC1	1	Test
TNKS1_ML_3934	Nc1nc(O)c(C2=c3cc(Cl)ccc3=NC2=O)s1	0	Test
TNKS1_ML_3935	CCC[C@@H](NC(=O)CCc1c(C)c2cc3c(C)c(C)oc3cc2oc1=O)C(=O)N[C@H](Cc1ccc(Cl)cc1)C(=O)[O-]	0	Test
TNKS1_ML_3936	[H]/N=C1\C(C#N)C2=CC[NH+](C)C[C@H]2[C@H](CCc2ccccc2)C1(C#N)C#N	0	Test
TNKS1_ML_3937	COc1ccc([C@H]2C(C(=O)c3cc4cccc(OC)c4o3)=C([O-])C(=O)N2CCCn2cc[nH+]c2)cc1OC	0	Test
TNKS1_ML_3938	Cc1ccc(-n2nc(-c3ccccc3)c(C#N)c2N)cc1	0	Test
TNKS1_ML_3939	CC(=O)Nc1c(-c2ccc(C)cc2)c(C)nn1-c1ccc(C(=O)Nc2cccc(C)n2)cc1	0	Test
TNKS1_ML_3940	O=C1CCN(c2ccccc2C(=O)NCCc2nc(-c3ccccn3)no2)C(=O)N1	1	Test
TNKS1_ML_3941	Cc1noc2nc(-c3ccccc3)cc(C(=O)NO)c12	0	Test
TNKS1_ML_3942	C=CCNC(=O)N1[C@H]2CCCC[C@@H]2S[C@H]2CCCC[C@@H]21	0	Test
TNKS1_ML_3943	O=[N+]([O-])c1ccc(Br)cc1N1CCC[C@@H]1CO	0	Test
TNKS1_ML_3944	Cc1ccc(NC(=O)N[C@@H]2CCS(=O)(=O)C2)c(Br)c1	0	Test
TNKS1_ML_3945	Oc1ccc(O)c2c1sc1nncn12	1	Test
TNKS1_ML_3946	O=C(COc1ccc([N+](=O)[O-])cc1Br)N/N=C/C1=c2ccccc2=[NH+]C1	0	Test
TNKS1_ML_3947	Cc1ccc(C[NH2+][C@@H](C)c2cnc(N3CCCCCCC3)nc2C)o1	0	Test
TNKS1_ML_3948	Cc1ccc2cc(C[NH+](Cc3ccc4c(c3)OCO4)Cc3nnnn3CCc3ccccc3)c(=O)[nH]c2c1C	0	Test
TNKS1_ML_3949	CC(C)(C)c1csc([C@H](N)c2cc(Cl)c3c(c2)OCO3)n1	0	Test
TNKS1_ML_3950	Cc1csc(CNCc2cccc(Br)c2[O-])n1	0	Test
TNKS1_ML_3951	Cc1cc(NS(=O)(=O)c2ccc(NC(=O)CCCC(=O)Nc3ccc(S(=O)(=O)Nc4cc(C)on4)cc3)cc2)no1	0	Test
TNKS1_ML_3952	CCCc1noc2nc(-c3cccnc3)cc(/C([O-])=N/c3nccc4sccc34)c12	0	Test
TNKS1_ML_3953	CCn1cnnc1CNC(=O)c1cnn(-c2cccc(C(F)(F)F)c2)c1C1CC1	0	Test
TNKS1_ML_3954	Cc1ccc(S(=O)(=O)N2CCN(CC(=O)Nc3ccc4c(c3)OC(F)(F)O	0	Test

	4)CC2)cc1		
TNKS1_ML_3955	CC(C)(O)C#CC(C)(C)C#CC(C)(C)C#CC(=O)[O-]	0	Test
TNKS1_ML_3956	O[C@@H]1C[C@H](N2CCOCC2)[C@H]2O[C@@H]12	0	Test
TNKS1_ML_3957	O=[N+]([O-])c1cccc(/C=N/c2ccc(S(=O)(=O)[N-]c3ccc(I)cc3)cc2)c1	0	Test
TNKS1_ML_3958	CN1c2ncccc2C(=O)NC1c1ccc(NC(=O)C2CCC2)cc1	1	Test
TNKS1_ML_3959	Cc1ccnc(-n2nc(C(F)(F)F)c(C)c2O)c1	0	Test
TNKS1_ML_3960	COc1ccccc1N1CCN(c2nc3c(c(=O)[nH]2)CCCC3)CC1	1	Test
TNKS1_ML_3961	[H]/N=C1/NC(=O)N(C2CC2)[C@]12CCCCC2(C)C	0	Test
TNKS1_ML_3962	O=C([O-])[C@@H](Nc1ccc(Cl)c(Br)c1)C1CCCC1	0	Test
TNKS1_ML_3963	C=CCN1C(=O)[C@]2(C(C#N)=C(N)Oc3cc(C)n(CC=C)c(=O)c32)c2ccccc21	0	Test
TNKS1_ML_3964	CC(C)C1CC2=NNC(=O)c3cccc4[nH]c(c2c34)C1	1	Test
TNKS1_ML_3965	O=S(=O)(c1ccc(NC2=NN=C(c3ccc4c(c3)OCCCO4)CS2)cc1)N1CCOCC1	0	Test
TNKS1_ML_3966	CCO[C@]1(C)OCC(=O)[C@]2(CC[C@H]3[C@H]4CCC5=CC(=O)C=C[C@@]5(C)[C@@H]4[C@@H](O)C[C@@]32C)O1	0	Test
TNKS1_ML_3967	C[C@H](Sc1nnnn1-c1ccc([O-])cc1)C(=O)N[C@@H](C)c1ccc(Cl)cc1Cl	0	Test
TNKS1_ML_3968	Cc1cc(NC(=O)C(=O)c2c(C)nn(-c3cccc(C(F)(F)F)c3)c2C)ccc1N1CC[NH+](C)CC1	0	Test
TNKS1_ML_3969	O=c1[nH]c(N2CCC3(CC2)COc2ccccc23)nc2c1CCCC2	1	Test
TNKS1_ML_3970	CC(C)(O)c1ccc(-c2nc3c(F)cccc3c(=O)[nH]2)cc1	1	Test
TNKS1_ML_3971	O=C(c1ccc(Cl)cc1)C1CCN(C2CCN(Cc3nc4c(c(=O)[nH]3)COCC4)C2=O)CC1	1	Test
TNKS1_ML_3972	C[C@@H]1CN(S(=O)(=O)N2CCc3ccccc3C2)CCN1S(=O)(=O)N1CCc2ccccc2C1	0	Test
TNKS1_ML_3973	Cc1ccc(-c2cc(/C([O-])=N/c3nnc(Cc4ccccc4)s3)c3c(C)noc3n2)cc1	0	Test
TNKS1_ML_3974	COc1ccc(S(=O)(=O)N(CC(=O)Nc2ccc(S(=O)(=O)/N=c3\[nH]ccs3)cc2)c2ccccc2)cc1OC	0	Test
TNKS1_ML_3975	CN(C)CCOc1cc(F)c2c(c1)N(C)C(=O)C21CCN(c2nc3c(c(=O)[nH]2)CCCN3C)CC1	1	Test
TNKS1_ML_3976	CCc1ccc2c(c1)sc1nncn12	1	Test
TNKS1_ML_3977	C/C(=C\c1cc(Cl)c2c(c1)OCCO2)C(=O)O	0	Test
TNKS1_ML_3978	COCCn1c(C)cc2c(c1=O)[C@]1(C(=O)N(Cc3ccccc3)c3ccccc31)C(C(=O)OC)=C(N)O2	0	Test
TNKS1_ML_3979	O=c1[nH]c(-c2ccc3c(c2)OCO3)nc2ccccc12	1	Test
TNKS1_ML_3980	CCC[C@@H](C)c1nc(N)ns1	0	Test
TNKS1_ML_3981	C/N=C1\C=C(NC)CC(C)(C)C1	0	Test
TNKS1_ML_3982	CN(Cc1ccc2[nH+]cccc2c1)C(=O)c1ccc(=O)n(CC(=O)NC2CCCCC2)c1	0	Test
TNKS1_ML_3983	Cc1cc(N[C@H]2CC[C@H](C)[C@H](C)C2)n2ncnc2n1	0	Test
TNKS1_ML_3984	C=CCOC(=O)c1sc(N2C(=O)C(=O)C(=C(O)c3ccc4c(c3)C[C@@H](C)O4)[C@H]2c2ccc[nH+]c2)nc1C	0	Test
TNKS1_ML_3985	CC/N=C1\S[C@H](CC(=O)Nc2ccc(N3CCOCC3)cc2)C(=O)N1CC	0	Test
TNKS1_ML_3986	OC1(CN[C@@H]2CCOC2)CCCCCC1	0	Test
TNKS1_ML_3987	N#Cc1ccc(NC(=O)Nc2ccc(Br)cn2)cc1	0	Test
TNKS1_ML_3988	CC(C)(C)C(=O)N1CCN(C(=O)Nc2cccc(F)c2F)CC1	0	Test
TNKS1_ML_3989	CCc1nnc(N2C(=O)c3oc4cc(C)c(Cl)cc4c(=O)c3[C@H]2c2cccc(O)c2)s1	0	Test
TNKS1_ML_3990	Cc1cc(C(=O)[C@@H]2CC(=O)Nc3c2c(C)nn3-c2ccc(C)c(Cl)c2)c(C)s1	0	Test
TNKS1_ML_3991	CS(=O)(=O)c1ccc(-c2nnc([C@H]3C[C@H](NC(=O)c4ccnc5cccnc45)C3)n2-c2ccccc2F)nc1	1	Test

TNKS1_ML_3992	CC(c1ccc(F)cc1F)N(Cc1nc2c(c(=O)[nH]1)COCC2)C(=O)CN1CCC(C(=O)c2ccc(F)cc2)CC1	1	Test
TNKS1_ML_3993	C[C@@H]1CC(C)(C)C[C@@]2(CNC(=O)C2)C1	0	Test
TNKS1_ML_3994	CCO[C@H]1[C@H](NC(=O)c2cccnc2)c2ccccc2C12CCN(C(=O)C1=CN=NC1)CC2	0	Test
TNKS1_ML_3995	CC(=O)N1CCc2ccc(NC(=O)c3ccc(S(=O)(=O)N4CCCCC4)cc3)cc21	0	Test
TNKS1_ML_3996	O=C(COC(=O)c1cc(S(=O)(=O)N2CCOCC2)ccc1Cl)Nc1ccc(F)c(Cl)c1	0	Test
TNKS1_ML_3997	CN(C)S(=O)(=O)c1ccc(C(O)=C2C(=O)C(=O)N(Cc3ccc[nH+]c3)[C@@H]2c2ccc(Cl)cc2)cc1	0	Test
TNKS1_ML_3998	C[C@@H]1CN(c2cc(C(=O)[O-])c([NH3+])cc2F)CCCO1	0	Test
TNKS1_ML_3999	CC#Cc1cc(CC)nc(S(C)(=O)=O)n1	0	Test
TNKS1_ML_4000	CCCc1c(-c2cccs2)nn([C@@H]2CCS(=O)(=O)C2)c1N	0	Test
TNKS1_ML_4001	COC(=O)c1cccc(NC(=O)c2cccc(S(=O)(=O)[C@H]3CCS(=O)(=O)C3)c2)c1	0	Test
TNKS1_ML_4002	CC(C)[C@H](NS(=O)(=O)c1cccc2nsnc12)C(=O)NCC[NH+](C(C)C)C(C)C	0	Test
TNKS1_ML_4003	Cc1ccn(CC(=O)N2CCOc3c(O)cc(-c4cccc(Cl)c4)cc3C2)n1	0	Test
TNKS1_ML_4004	CC(C)CN(Cc1nc2c(c(=O)[nH]1)COCC2)C(=O)CN1CCC(C(=O)c2ccc(F)cc2)CC1	1	Test
TNKS1_ML_4005	O=C(N[C@H]1C[C@H](c2nnc(-c3ccccn3)n2-c2ccccc2F)C1)c1cc(F)cc2ncncl2	1	Test
TNKS1_ML_4006	S=c1[nH]nc(C2CC2)n1-c1ccc2c(c1)OCO2	0	Test
TNKS1_ML_4007	NC(=O)c1ccc2sc(Br)nc2c1	0	Test
TNKS1_ML_4008	O=C([O-])C[C@@H]1CCCN(C(=O)N[C@H]2CC[NH+](C3CC3)C2)C1	0	Test
TNKS1_ML_4009	O=C([O-])C1(Nc2ccc(Br)cc2)C2CC3CC(C2)CC1C3	0	Test
TNKS1_ML_4010	Cc1cccc(OCCCCn2c(CCCNC(=O)c3cccc(Br)c3)[nH+]c3ccccc32)c1	0	Test
TNKS1_ML_4011	CCn1c[nH+]c2c1c(=O)n(CC(=O)Nc1cccc(C(C)=O)c1)c(=O)n2Cc1ccccc1	0	Test
TNKS1_ML_4012	Cc1ccc([N-]S(=O)(=O)c2ccc3c(c2)CCN3C(=O)CCC(=O)N2CCCCCC2)c(C)c1	0	Test
TNKS1_ML_4013	O=C(NCC1(c2ccccc2)CCOCC1)[C@H]1CC12CCN(C(=O)c1ccc3c(c1)no[n+]3[O-])CC2	0	Test
TNKS1_ML_4014	Cc1cccc(C(=O)Nc2ccc(-c3nccs3)cc2)c1[N+](=O)[O-]	0	Test
TNKS1_ML_4015	Cn1ccc2c(=O)[nH]c(N3CCC4(CC3)CN(CCO)c3cc(F)cc(F)c34)nc21	1	Test
TNKS1_ML_4016	CCn1cc(C(=O)Nc2cccc(C#N)c2)c(S(=O)(=O)N2CC[NH+](c3cccc(C)c3C)CC2)n1	0	Test
TNKS1_ML_4017	Cc1cnc(CNc2cc3c(cc2[NH3+])NC(=O)CC3)cn1	0	Test
TNKS1_ML_4018	[O-]c1ccccc1/C=N\c1cc(F)cc(F)c1	0	Test
TNKS1_ML_4019	CC[C@]1(C)C[C@@H](n2ncc(C)c2N)CCO1	0	Test
TNKS1_ML_4020	CCc1[nH+]c(-c2cc(Cl)c3c(c2)OCCO3)nc(N)c1Br	0	Test
TNKS1_ML_4021	Cc1ccc(CNc2c(/C=C3\SC(=S)N(C[C@H]4CCCO4)C3=O)c(C)c(C#N)c(=O)n2C)cc1	0	Test
TNKS1_ML_4022	CC(C)c1nc(C2CCC(O)CC2)n(-c2cccc3cccnc23)n1	0	Test
TNKS1_ML_4023	C[C@H]1CN(C(=O)CCNC(=O)c2cccs2)C(=Nc2cccc(S(=O)(=O)N(C)C)c2)S1	0	Test
TNKS1_ML_4024	O=C(NNC(=O)[C@H]1CCCC[C@@H]1c1nc2ccccc2s1)[C@@H]1COc2ccccc2O1	0	Test
TNKS1_ML_4025	O=C1/C(=C/c2c(NCCCn3ccnc3)nc3ccccn3c2=O)SC(=S)N1C[C@H]1CCCO1	0	Test
TNKS1_ML_4026	Cc1n[nH]c(C)c1S(=O)(=O)N1CCC[C@@H](C(=O)N2CCC(CC(N)=O)CC2)C1	0	Test

TNKS1_ML_4027	[H]/N=C1/S[C@@H](C)C(C)=C1c1nc2cc(F)ccc2n1C(C)C	0	Test
TNKS1_ML_4028	O=C(COC(=O)C1=c2ccccc2=[NH+]C(=O)C1)Nc1ccc(S(=O)(=O)N2CCCC2)cc1	0	Test
TNKS1_ML_4029	Nc1ccc(C(=O)N2CCC[C@@H]2c2cccs2)cc1[N+](=O)[O-]	0	Test
TNKS1_ML_4030	Cc1ccc(-n2nc(C)c3c(=O)c(C(=O)NCc4ccc(Cl)cc4)c[nH]c32)cc1	0	Test
TNKS1_ML_4031	CN(C)C[C@H]1O[C@@H]2OC(C)(C)O[C@@H]2[C@H]1O	0	Test
TNKS1_ML_4032	CSc1nsc(NC(=O)N2CC[C@@H]3CCCC[C@H]32)n1	0	Test
TNKS1_ML_4033	CC[NH+](CC)CCN1C(=O)C(O)=C(C(=O)c2c(C)nc3ccccn23)[C@H]1c1cccc(Oc2ccccc2)c1	0	Test
TNKS1_ML_4034	Cc1ccc(-n2c(C)cc(/C([O-])=C(/C#N)C(=O)c3cccc(NC(=O)c4ccco4)c3)c2C)cc1C	0	Test
TNKS1_ML_4035	C[C@H]1CCN(c2[nH+]cc(C(=O)[O-])s2)[C@H](C)C1	0	Test
TNKS1_ML_4036	CC(=O)N1CCc2nc3cc([C@H]4CCCCN4C(=O)CCC4CCCC4)nn3c(O)c2C1	0	Test
TNKS1_ML_4037	CC(=O)N1CCN(S(=O)(=O)c2cccc(C(=O)Nc3cccc(N4CCCC4=O)c3)c2)CC1	0	Test
TNKS1_ML_4038	CSc1ccccc1NC(=O)c1cc(-c2ccco2)n([C@H]2CCS(=O)(=O)C2)n1	0	Test
TNKS1_ML_4039	CC(c1cccs1)N(Cc1nc2c(c(=O)[nH]1)COCC2)C(=O)CN1CCC(C(=O)c2ccc(F)cc2)CC1	1	Test
TNKS1_ML_4040	Oc1nc(-c2ccc(C(F)(F)F)cc2)nc2c(O)cccc12	1	Test
TNKS1_ML_4041	COC(=O)c1ccc(S(=O)(=O)N2c3ccc(S(N)(=O)=O)cc3C[C@H]2C)cc1	0	Test
TNKS1_ML_4042	Cc1cc(NS(=O)(=O)c2ccc(NC(=O)CCCCC(=O)Nc3ccc(S(=O)(=O)Nc4cc(C)on4)cc3)cc2)no1	0	Test
TNKS1_ML_4043	COc1ccc(-c2noc(CSc3nnc(C)n3-c3ccc(F)cc3)n2)cc1	1	Test
TNKS1_ML_4044	CCc1nn2c(nnc3c(=O)n(NC(=O)c4ccc5c(c4)OCCO5)ccc32)c1-c1cccc(Cl)c1	0	Test
TNKS1_ML_4045	CNC(=O)[C@@H]1CCCCN1c1nc(C)c(C)nc1Cl	0	Test
TNKS1_ML_4046	Nc1n[nH]cc1[C@H](O)[C@H]1CCOC2(CCCC2)C1	0	Test
TNKS1_ML_4047	N#Cc1ccccc1Cn1c(=O)n(C[C@H]2CC[C@H](C(=O)Nc3ccncc3)CC2)c(=O)c2nc[nH]c21	1	Test
TNKS1_ML_4048	CC1(C)Cc2c(c(N3CCOCC3)[nH+]c3sc4c(N(CCO)Cc5ccccc5)ncnc4c23)CO1	0	Test
TNKS1_ML_4049	Cc1ccc(S(=O)(=O)N2CCC[C@@H]2C(=O)NCc2ccc(N(C)C)cc2)s1	0	Test
TNKS1_ML_4050	Clc1ccc(Cl)c2nocc12	0	Test
TNKS1_ML_4051	O=C([O-])C[C@H]1SC(N2N=C(c3ccc(F)cc3)C[C@H]2c2ccc(Cl)cc2)=NC1=O	0	Test
TNKS1_ML_4052	C[C@@H]1CCCN(c2ccc(C#N)c(Cl)c2)CC1	0	Test
TNKS1_ML_4053	CC1(C)CCCN(c2cc3nc[nH]c(=O)c3cc2N)C1	0	Test
TNKS1_ML_4054	CCN(c1nc2c(s1)[C@@H]([NH2+]C)CCC2)c1ccccc1C	0	Test
TNKS1_ML_4055	O=S(=O)(NCC1CC[NH+](Cc2ccco2)CC1)c1ccc(-c2cc(C(F)(F)F)on2)s1	0	Test
TNKS1_ML_4056	CC[C@@]1(O)CCN(Cc2cc(C3CCCCC3)no2)C[C@H]1O	0	Test
TNKS1_ML_4057	O=C(Nc1cccc(-c2c[nH+]c3n2CCC3)c1)c1ccc(Cl)s1	0	Test
TNKS1_ML_4058	Cc1cc(C(=O)Nc2ccccc2N2CCCC2=O)cc(S(=O)(=O)N(C)C)c1C	0	Test
TNKS1_ML_4059	S[C@H]1C=C2OC=CCC2=C2SC=N[C@H]21	0	Test
TNKS1_ML_4060	O=C1NC(c2ccc(Cl)cc2)Nc2ccccc21	1	Test
TNKS1_ML_4061	Cc1occc1C(=O)N/N=C1\CCCc2oc(C(=O)N3CCN(c4ccccc4)CC3)c(C)c21	0	Test
TNKS1_ML_4062	CC(=O)Nc1cc(NC(=O)c2cccc(S(=O)(=O)N[C@H]3CCS(=O)(=O)C3)c2)ccc1F	0	Test
TNKS1_ML_4063	Cc1ccc(C(=O)N2[C@H](C(=O)NC[C@@H]3CCCO3)COC	0	Test

	23CCN(C(=O)c2cccc([N+](=O)[O-])c2)CC3)cc1		
TNKS1_ML_4064	Cc1cc(=O)[nH]c2cc(-c3ccc(N)cc3Cl)ccc12	1	Test
TNKS1_ML_4065	O=C([O-])[C@H](Nc1ccccc1Br)[C@@H]1CC=CCC1	0	Test
TNKS1_ML_4066	Cc1cccc2c1c(=O)n(C[C@H]1CC[C@H](C(=O)Nc3ccnnc3)CC1)c(=O)n2Cc1cccc(C(F)(F)F)c1C#N	1	Test
TNKS1_ML_4067	COc1ccc2c(c1)C(CC(=O)NCCNC(=O)c1ccccc1N1CCC(=O)NC1=O)CC2=O	1	Test
TNKS1_ML_4068	CN(c1cccc(NC(=O)CN(c2ccc3c(c2)OCCO3)S(C)(=O)=O)c1)S(C)(=O)=O	0	Test
TNKS1_ML_4069	Cc1cc(C)n(-c2ccc(CNCc3c(C)nn(C)c3C)cn2)n1	0	Test
TNKS1_ML_4070	Cc1nc(-c2cc(Br)c3c(c2)OCCO3)nc(N)c1Br	0	Test
TNKS1_ML_4071	Cc1ccc(N2C(=O)CCS2(=O)=O)cc1S(=O)(=O)NC[C@H](c1ccc(Cl)cc1)[NH+](C)C	0	Test
TNKS1_ML_4072	Cc1nn(C)c2c1[nH+]c(N)n2C[C@H]1Cc2ccccc21	0	Test
TNKS1_ML_4073	CN1CCCc2c1nc(N1CCC3(CC1)C(=O)N(C)c1cc(OCCN4CCCCC4)cc(F)c13)[nH]c2=O	1	Test
TNKS1_ML_4074	CCOc1ccc(-c2nnc([C@H]3C[C@H](NC(=O)c4cccc5cccnc45)C3)n2-c2ccc(C)s2)nc1	1	Test
TNKS1_ML_4075	O=c1[nH]nc2c3c(cccc13)NC1=C2CCCC1	1	Test
TNKS1_ML_4076	Cc1cc(N[C@@H]2CCC[C@@H](C)C2)nc(Cl)n1	0	Test
TNKS1_ML_4077	CCOc1ccc(-c2nnc([C@H]3C[C@H](NC(=O)c4ccnc5cccnc45)C3)n2-c2cccnc2)nc1	1	Test
TNKS1_ML_4078	Cc1csc(-c2cc(S(=O)(=O)Nc3ccccc3F)cn2CC(=O)N2CCOCC2)n1	0	Test
TNKS1_ML_4079	Cc1cccc2c(=O)[nH]c(N3CCC4(CC3)CN(CCO)c3cc(F)cc(F)c34)nc12	1	Test
TNKS1_ML_4080	O=C(C[C@H](C[NH+]1CCN(Cc2cccc[nH+]2)CC1)C(=O)[O-])Nc1ccc2c(c1)OCO2	0	Test
TNKS1_ML_4081	CCOc1ccc(C(=O)[C@@H]2C(=O)C(=O)N(CCCn3cc[nH+]c3)[C@@H]2c2ccc([N+](=O)[O-])cc2)cc1Cl	0	Test
TNKS1_ML_4082	CC[C@H](C)NS(=O)(=O)c1ccc(C(=O)N(C)Cc2cnn(-c3ccccc3)c2)cc1	0	Test
TNKS1_ML_4083	Cc1sc2nc(SCCNS(C)(=O)=O)n(C3CCCC3)c(=O)c2c1C	0	Test
TNKS1_ML_4084	Cc1nn(C(C)C)c(NC2(CO)CCCC2)c1N	0	Test
TNKS1_ML_4085	Nc1nc(Cl)cc(N2CCN(Cc3ccon3)CC2)n1	0	Test
TNKS1_ML_4086	Cc1sc2nc(CN3CCSCC3)nc(NN)c2c1C	0	Test
TNKS1_ML_4087	Cc1sc2nc(CN(C)c3ccccc3)nc(N)c2c1C	0	Test
TNKS1_ML_4088	C=CCOc1ccc(C(=O)C2=C([O-])C(=O)N(CCC[NH+]3CCOCC3)[C@H]2c2ccc(Br)cc2)cc1C	0	Test
TNKS1_ML_4089	COc1ccc(F)cc1C(=O)C1=C([O-])C(=O)N(CCCn2cc[nH+]c2)[C@@H]1c1cccc(F)c1	0	Test
TNKS1_ML_4090	CC1(C)OC(=O)N(c2ccc(C(=O)Nc3cccc4cccnc34)cc2)[C@H]1c1ccccc1	1	Test
TNKS1_ML_4091	N#Cc1c(Cn2c(=O)n(C[C@H]3CC[C@H](C(=O)O)CC3)c(=O)c3ccccc32)cccc1C(F)(F)F	1	Test
TNKS1_ML_4092	CNC(=O)c1ccccc1NC(=O)c1nn(-c2ccccc2C(F)(F)F)c(C)cc1=O	0	Test
TNKS1_ML_4093	Cc1ccc(Cl)cc1NC1CCC2(CC1)OCCO2	0	Test
TNKS1_ML_4094	CC(C)c1nc2cc(C(=O)[O-])cc(Br)c2[nH]1	0	Test
TNKS1_ML_4095	CCOc1ccc(C(=O)C2CCN(CC(=O)NCc3nc4c(c(=O)[nH]3)COCC4)CC2)cc1F	1	Test
TNKS1_ML_4096	CN1CCN(c2nc3c(c(=O)[nH]2)CCCC3)CC1	1	Test
TNKS1_ML_4097	C[C@H]1CN(c2ncc(N)cc2C#N)C[C@@H]1C	0	Test
TNKS1_ML_4098	O=C(CCc1ccccc1O)N(Cc1nc2c(c(=O)[nH]1)COCC2)Cc1cccs1	1	Test

TNKS1_ML_4099	Cc1ccc(C)c(-c2cc(N)n(C)n2)c1	0	Test
TNKS1_ML_4100	Cc1ccc([NH+]2C[C@@H](C)OC[C@@H]2C)c(C(=O)[O-])c1	0	Test
TNKS1_ML_4101	CC(C)n1cnc([C@H]2CN(S(C)(=O)=O)c3ccc(C(=O)NCCc4cccc(F)c4)cc32)n1	0	Test
TNKS1_ML_4102	O[C@@H](c1cccs1)c1cc2c(cc1Cl)OCCCO2	0	Test
TNKS1_ML_4103	Cc1cc([N+](=O)[O-])nn1C[C@@H](C)c1nnc([S-])n1CCCN1CCOCC1	0	Test
TNKS1_ML_4104	N#C/C(=C\c1cn(CC(=O)NC[C@H]2CCCO2)c2ccccc12)C(=O)N1CCN(c2ccccc2)CC1	0	Test
TNKS1_ML_4105	Cc1noc(C)c1S(=O)(=O)N1CCC[NH+](CCC(=O)Nc2sc3c(c2C#N)CCCC3)CC1	0	Test
TNKS1_ML_4106	Cc1ccccc1NC(=O)c1cnc2c(c1)c(C)nn2C	0	Test
TNKS1_ML_4107	Cc1ccc(C)c(-c2cc3c(N4CC[C@H](O)C4)nccn3n2)c1	0	Test
TNKS1_ML_4108	COC(=O)c1c(NC(=O)c2ccc3ncsc3c2)sc2c1CCN(C(C)=O)C2	0	Test
TNKS1_ML_4109	Cc1nc(C[NH+]2[C@H]3CC[C@@H]2[C@H](C(=O)[O-])C3)oc1C	0	Test
TNKS1_ML_4110	CCOC(=O)C1=C(C)N=c2s/c(=C\c3cn(CC)nc3C)c(=O)n2[C@@H]1c1ccc(OC)cc1	0	Test
TNKS1_ML_4111	[H]/N=C1C(=C/c2ccc(-c3cccc(Cl)c3)o2)/C(=O)N=C2SC(c3ccncc3)=NN2/1	0	Test
TNKS1_ML_4112	Nc1ncc2c(n1)CN(S(=O)(=O)c1ccc(Oc3ccccc3Cl)cc1)CC2	0	Test
TNKS1_ML_4113	Cc1ccc2[nH]c3c(c2c1)CN(C(=O)C[C@@]1(c2ccccc2)CC(=O)N(Cc2ccc[nH+]c2)C1=O)CC3	0	Test
TNKS1_ML_4114	CCc1nnc(NC(=O)[C@H](C)Sc2nnc(CCNC(=O)c3ccccc3F)n2C)s1	0	Test
TNKS1_ML_4115	C[C@H](Cn1cncn1)Nc1nc(CN(C)C)nc2scc(-c3ccccc3)c12	0	Test
TNKS1_ML_4116	Cc1nonc1COc1c(Cl)cc(Cl)cc1C(=O)[O-]	0	Test
TNKS1_ML_4117	O=C(NC1CCC2(CC1)OCCO2)C1C2CC3CC(C2)CC1C3	0	Test
TNKS1_ML_4118	CC(C)(Oc1ccc(Cl)cc1)C(=O)NCCS(=O)(=O)N1CCN(c2cccc[nH+]2)CC1	0	Test
TNKS1_ML_4119	CNc1nc(-c2ccccc2)ncc1I	0	Test
TNKS1_ML_4120	CC[NH2+][C@H]1c2cc(C(C)(C)C)ccc2S(=O)(=O)[C@H](C)[C@@H]1C	0	Test
TNKS1_ML_4121	C[C@H]1CCCN(S(=O)(=O)c2cccc(-c3cs/c(=N\N=C\c4ccco4)[nH]3)c2)C1	0	Test
TNKS1_ML_4122	CN(Cc1ccccc1Br)c1ccc(C#N)c(N)c1	0	Test
TNKS1_ML_4123	O=C(c1ccc(C(F)(F)F)cc1)C1CCN(CC(=O)N(Cc2nc3c(c(=O)[nH]2)COCC3)CC(F)F)CC1	1	Test
TNKS1_ML_4124	Cc1c(C)n(Cc2ccc(Cl)s2)c2ncnc(N)c12	0	Test
TNKS1_ML_4125	CCNC(=O)[C@@]1(Cc2ccc(-c3ccc[nH+]c3)cc2)CCN(C(=O)Cn2nc(C)nc2C)C1	0	Test
TNKS1_ML_4126	C[C@]12CCCN1CC1=NNC(=O)c3cccc4[nH]c2c1c34	1	Test
TNKS1_ML_4127	O=C(CCc1ccccc1)N(Cc1nc2c(c(=O)[nH]1)COCC2)Cc1ncco1	1	Test
TNKS1_ML_4128	COc1ccc(C2NC(=O)c3cccnc3N2)cc1	1	Test
TNKS1_ML_4129	COC(=O)c1ccc2c(c1)s/c(=N\C(=O)c1ccc(S(=O)(=O)N(C)C)cc1)n2C	0	Test
TNKS1_ML_4130	[O-]/C(=N\N=C\C1=c2ccccc2=[NH+]C1)[C@@H]1Cc2ccccc2O1	0	Test
TNKS1_ML_4131	Cc1noc(C)c1[C@@H](C)N1CCCNC2(CCCCC2)C1	0	Test
TNKS1_ML_4132	C/C(CC(C)(C)c1ccccc1)=N\NC(=O)CN(c1cccc(Cl)c1)S(=O)(=O)c1ccc(C)cc1	0	Test
TNKS1_ML_4133	O=C1NC2(CCCCCCC2)C(=O)N1CN1CCCC1	0	Test
TNKS1_ML_4134	Cn1cnc(N)c1S(=O)(=O)N1CCC[C@H]1C1CCCC1	0	Test
TNKS1_ML_4135	CCC(=O)Nc1ccc(S(=O)(=O)NC[C@H](c2ccco2)[NH+]2CC	0	Test

	OCC2)c(C)c1		
TNKS1_ML_4136	Br.Cc1cccc2c(=O)[nH]c(-c3ccc(CN)cc3)nc12	1	Test
TNKS1_ML_4137	COc1ccc(OC)c([C@H]2Nc3ccc(Br)cc3-c3nnc(SC)nc3O2)c1	0	Test
TNKS1_ML_4138	CC(=O)N1CCCc2cc(S(=O)(=O)Nc3ccccc3-c3nnc4n3CCCCC4)ccc21	0	Test
TNKS1_ML_4139	Cc1ccc(N(Cc2ccc(C(=O)NCCCN3CC[NH+](C)CC3)cc2)S(C)(=O)=O)cc1C	0	Test
TNKS1_ML_4140	CCCCCCN1C(=O)/C(=C/c2c(N3CCN(CCO)CC3)nc3c(C)ccn3c2=O)SC1=S	0	Test
TNKS1_ML_4141	O=c1[nH]c(-c2ccc(O)cc2)nc2ccccc12	1	Test
TNKS1_ML_4142	C[C@H]1CCCC[C@H]1Oc1nc(N)ncc1Cl	0	Test
TNKS1_ML_4143	O=C(NNS(=O)(=O)c1cnc(Cl)c(Cl)c1)c1ccc(Cl)cc1Cl	0	Test
TNKS1_ML_4144	CC1(C)[C@]2(C(=O)NCCN(CCNC(=O)[C@]34CC[C@](C)(C(=O)C3)C4(C)C)CCNC(=O)[C@]34CC[C@](C)(C(=O)C3)C4(C)C)CC[C@@]1(C)C(=O)C2	0	Test
TNKS1_ML_4145	O=C(c1ccnc(N2CC[NH2+][C@H](c3ccccc3)C2)c1)N1CCN(c2cccc[nH+]2)CC1	0	Test
TNKS1_ML_4146	C[C@@H](C(=O)N[C@H](C)c1ccccc1)[NH+]1CCN(C(=O)[C@@H]2COc3ccccc3O2)CC1	0	Test
TNKS1_ML_4147	O=C(Nc1ccc(Br)cc1F)c1ccc(F)nc1	0	Test
TNKS1_ML_4148	O=C(CN1N=N[C@@H]2C(=O)N(c3cccc(Br)c3)C(=O)[C@H]21)Nc1ccc(Br)cc1	0	Test
TNKS1_ML_4149	COc1cccc([C@H]2C[C@@H](c3cccc(F)c3)n3ncnc3N2)c1	0	Test
TNKS1_ML_4150	O=CN[C@H]1[C@@H]2CCC[C@H]1[C@]13CCCC[C@@]1(C2)O3	0	Test
TNKS1_ML_4151	NCc1ncoc1-c1cc2c(s1)CCCCC2	0	Test
TNKS1_ML_4152	C[C@H]1CCCC[C@H]1Nc1ncnc2sccc12	0	Test
TNKS1_ML_4153	Cc1cc(Br)cc(N)c1Oc1ncncc1Cl	0	Test
TNKS1_ML_4154	CC(C)(O)c1ccc(-c2nc3cccc(F)c3c(=O)[nH]2)cc1	1	Test
TNKS1_ML_4155	CC(C)Oc1ccc(S(=O)(=O)N2CCOCC2)cc1NC(=O)/C=C\c1cccc2cccnc12	0	Test
TNKS1_ML_4156	CSc1ccc(CCNC(=O)CCc2c(C)nn(-c3cc(N4CCOCC4)ncn3)c2C)cc1	0	Test
TNKS1_ML_4157	COc1ccc(/C=N/N2CC(=O)N3[C@@H](Cc4c([nH]c5ccccc45)C3(C)C)C2=O)c(OC)c1	0	Test
TNKS1_ML_4158	N#C/C(=C/c1cccc(O)c1)c1nc2ncc(Br)cc2[n-]1	0	Test
TNKS1_ML_4159	O=c1[nH]c(N2CCC3(CC2)CN(CCO)c2cc(Cl)cc(Cl)c23)nc2c1CCCC2	1	Test
TNKS1_ML_4160	c1ccc(-c2ncc3c(n2)CCSC3)cc1	1	Test
TNKS1_ML_4161	Cc1cc(NC(=O)/C=C/c2cn(-c3ccccc3)nc2-c2ccccc2)n([C@@H]2CCS(=O)(=O)C2)n1	0	Test
TNKS1_ML_4162	CCCCCN1C(=O)/C(=C\c2c(N3CCN(C(=O)OCC)CC3)nc3ccccn3c2=O)SC1=S	0	Test
TNKS1_ML_4163	CC(=O)Nc1ccc(NC(=O)c2ccc(S(=O)(=O)N3CCN(C=O)CC3)cc2)cc1	0	Test
TNKS1_ML_4164	O=C(CCc1ccc(Cl)cc1Cl)Nc1nnc2n1CCS2	1	Test
TNKS1_ML_4165	O=C(CN1CCC(C(=O)N2CCc3ccccc3C2)CC1)NCc1nc2c(c(=O)[nH]1)COCC2	1	Test
TNKS1_ML_4166	Cc1cc2c(=O)[nH]c(-c3ccc(C(C)(C)O)cc3)cn2c1	1	Test
TNKS1_ML_4167	CC(=O)Nc1ccc(C[NH+]2CCc3c(c(C(=O)NCc4nccs4)nn3CC3CC3)C2)cc1	0	Test
TNKS1_ML_4168	C[C@H]1C(F)(F)[C@](F)(Cl)[C@@]1(C)C(=O)[O-]	0	Test
TNKS1_ML_4169	N#CC1=C(N)S[C@@H](c2ccccc2)[C@@H]2C(=O)Oc3ccc(Br)cc3[C@@H]12	0	Test
TNKS1_ML_4170	CCOC(=O)C1CCN(C(=O)[C@@H]2CCCN(S(=O)(=O)c3ccc4c(c3)CCCC(=O)N4)C2)CC1	0	Test
TNKS1_ML_4171	CCc1cc(C(=O)N2CCOC(C)(C)C2)n[nH]1	0	Test
TNKS1_ML_4172	Cc1cc(C(=O)Nc2nc3c(s2)CN(S(=O)(=O)c2cc(Cl)ccc2C)CC	0	Test

	3)no1		
TNKS1_ML_4173	O=C(/N=c1\sn(-c2ccc(Cl)cc2)nc1C(=O)N1CCCC1)c1nnn(-c2ccccc2)c1-c1ccccc1	0	Test
TNKS1_ML_4174	CC(C)[C@@H]1CCC[C@H](C)C(=O)O1	0	Test
TNKS1_ML_4175	C[C@H]1C[C@H](n2ncc(Br)c2N)CC[C@H]1C	0	Test
TNKS1_ML_4176	CCn1c(CCC(=O)Nc2cnn(Cc3ccccc3)c2)[nH+]c2cc(S(=O)(=O)N(C)C)ccc21	0	Test
TNKS1_ML_4177	CC1CC2=NNC(=O)c3cccc4[nH]c(c2c34)C1	1	Test
TNKS1_ML_4178	CCOc1ccc2nc(N3C(=O)CNC3=S)sc2c1	0	Test
TNKS1_ML_4179	O=C(C[C@@H]1SC(N2CCCC2)=NC1=O)Nc1ccc(C(=O)NCc2cn3ccccc3[nH+]2)cc1	0	Test
TNKS1_ML_4180	C/C(=N/NC(=O)C[NH+]1CCN(S(=O)(=O)c2ccc(Br)cc2)CC1)c1ccccc1	0	Test
TNKS1_ML_4181	CC(C)n1cc2c(n1)CN(C(=O)c1cc(Cc3n[nH]c(=O)c4ccccc34)ccc1F)C2	1	Test
TNKS1_ML_4182	COc1ccc2cc(C(=O)Nc3ccc4c(c3)CCC4)[nH]c2c1	0	Test
TNKS1_ML_4183	COc1ccc(C(=O)C2CCN(CC(=O)NCc3nc4c(c(=O)[nH]3)CCC4)CC2)cc1	1	Test
TNKS1_ML_4184	C[C@H]1C[C@H](NC(=O)N(C)C[C@@H](C)C(=O)[O-])CC[C@H]1C	0	Test
TNKS1_ML_4185	Cn1cc(S(=O)(=O)N2CCC(C(=O)Nc3ccc(Br)cc3)CC2)cc1C(N)=O	0	Test
TNKS1_ML_4186	C#CCN1C(=O)[C@@]2(C(C(=O)OCCOC)=C1C)c1ccccc1-c1oc(N)nc12	0	Test
TNKS1_ML_4187	Cc1cnc(C)c(N2CCNCC23CCCCC3)n1	0	Test
TNKS1_ML_4188	O=C(COc1cc(Cl)c(Cl)cc1Cl)N=c1[nH]cc[nH]1	0	Test
TNKS1_ML_4189	Cc1cc(C)c(N)c(-c2nc3cc(Cl)ccc3o2)c1	0	Test
TNKS1_ML_4190	CC(=O)Nc1ccc(C(=O)CSc2nc(C(C)(C)C)nc3c2c(=O)n(C)c(=O)n3C)cc1	0	Test
TNKS1_ML_4191	CC[C@H](C)Nc1nc2ccccn2c(=O)c1/C=C1\SC(=S)N(Cc2ccc3c(c2)OCO3)C1=O	0	Test
TNKS1_ML_4192	CC[C@H]1CCC[C@@]2(CC(=O)NC(=O)[C@@H]2C(C)C)C1	0	Test
TNKS1_ML_4193	C[C@]1(c2ccc3c(c2)CCC3)NC(=O)N(CC(=O)N2N=C(c3ccco3)C[C@H]2c2cccs2)C1=O	0	Test
TNKS1_ML_4194	CCCN1C(=O)/C(=C\c2c(N[C@@H](CC)CO)nc3c(C)cccn3c2=O)SC1=S	0	Test
TNKS1_ML_4195	CC[NH+](CC)c1ccc(/C=C2\C(=O)NC(=O)N(c3ccc(Br)c(C)c3)C2=O)c(O)c1	0	Test
TNKS1_ML_4196	Cc1sc2[nH]c(=S)n([C@H]3CC[C@H](C)[C@@H]3C)c(=O)c2c1C	0	Test
TNKS1_ML_4197	OC1=Nc2[nH]ncc2[C@H](c2cccs2)SC1	0	Test
TNKS1_ML_4198	O=C1NC(c2cccc(OCc3ccccc3)c2)Nc2ccccc21	1	Test
TNKS1_ML_4199	O=C1NC(=N/c2ccccc2)/C(=C/c2ccc(O)cc2)S1	0	Test
TNKS1_ML_4200	COCCc1ncc(C[NH+]2CCC[C@H]2c2cc(Nc3ncc(C)s3)nc(C)n2)cn1	0	Test
TNKS1_ML_4201	COC(=O)[C@H]1Cc2c([nH]c3ccccc23)CN1C(=O)COc1ccc2c(C)cc(=O)oc2c1	0	Test
TNKS1_ML_4202	Cc1ccc(F)cc1NC(=O)N[C@@H](c1ccccc1)c1ccco1	0	Test
TNKS1_ML_4203	CCOc1ccc(-c2nnc([C@H]3C[C@H](NC(=O)c4cccc5nccnc45)C3)n2-c2ccccc2F)nc1	1	Test
TNKS1_ML_4204	O=C(CN(c1ccc(Cl)c(Cl)c1)S(=O)(=O)c1ccccc1)Nc1ccc(S(=O)(=O)N2CCOCC2)cc1	0	Test
TNKS1_ML_4205	O=C(Nc1cccc(-c2ccc(N3CCCC3)[nH+]n2)c1)c1ccc(S(=O)(=O)N2CCOCC2)cc1	0	Test
TNKS1_ML_4206	C[C@H]1OCC[C@H]1[C@H](Cl)c1ccc2c(c1)CCCC(=O)N	0	Test

	2		
TNKS1_ML_4207	N#CC1=CCC2=NC=C(CCCC[NH+]3CCN(c4ccc5oc(C(N)=O)cc5c4)CC3)C2=C1	0	Test
TNKS1_ML_4208	O=C(NCc1ccsc1)N1CCC[C@@H](C[NH+]2CCOCC2)C1	0	Test
TNKS1_ML_4209	Nc1sc2c(c1-c1nnc3n1CCCCC3)CCC2	0	Test
TNKS1_ML_4210	CC(=O)O[C@@H]1CC[C@]2(C=O)[C@@H]3CC[C@]4(C)[C@H](C5=CC(=O)OC5)CC[C@]4(O)[C@@H]3CC[C@]2(O)C1	0	Test
TNKS1_ML_4211	Cc1ccc(-n2nc(C(=O)NCc3cccnc3)c(=O)n(Cc3cccc(C)c3)c2=O)cc1	0	Test
TNKS1_ML_4212	[H]/N=C1\C(=Cc2ccc(-c3ccc(OC)c(Cl)c3)o2)C(=O)N=C2SC(COc3ccccc3)=NN21	0	Test
TNKS1_ML_4213	Cc1ncsc1Cc1nc2c([nH]1)C(=O)NCC(C)(C)C2	0	Test
TNKS1_ML_4214	Cc1ccc(/C=C\C(=O)Nc2ccc3c(c2)NC(=O)[C@H](C)O3)cc1	0	Test
TNKS1_ML_4215	Cc1nc(N(C)[C@@H](C)CC#N)cc2n[nH]c(=O)n12	0	Test
TNKS1_ML_4216	Cc1ccc2oc(-c3nnc(C(=O)NCCN4CCN(c5cc(Cl)ccc5C)CC4)o3)c(C)c2c1	0	Test
TNKS1_ML_4217	CCN(CC)c1ccc(C[NH+]2CCc3nnc([C@H](C)NC(=O)COc4ccccc4)n3CC2)cc1	0	Test
TNKS1_ML_4218	N#Cc1ccccc1N[C@H]1CCc2ccccc21	0	Test
TNKS1_ML_4219	CN(C)C(=O)c1cc[nH+]cc1[O-]	0	Test
TNKS1_ML_4220	C=CCC[C@@H](O)C[NH+]1CCN(Cc2nc(C(=O)N3CCC[C@@H](C(=O)N(CC)CC)C3)co2)CC1	0	Test
TNKS1_ML_4221	Cc1cc(N)sc1-c1nc(C2(C)CC2)no1	0	Test
TNKS1_ML_4222	Cc1cc(C)n2nc(C(=O)Nc3ccc(S(=O)(=O)N4C[C@@H](C)C[C@@H](C)C4)cc3)nc2n1	0	Test
TNKS1_ML_4223	Cc1cc(F)cc2c(=O)[nH]c(-c3ccc(C4CCNCC4)cc3)nc12.Cl	1	Test
TNKS1_ML_4224	Cc1ccc(-c2cc(=O)c3ccccc3o2)cc1	1	Test
TNKS1_ML_4225	COc1cc(C(=O)[O-])c(NS(=O)(=O)c2ccc3c(c2)CCC3)cc1OC(F)F	0	Test
TNKS1_ML_4226	CC[C@H](CO)NC(=O)C(=O)Nc1c2c(nn1-c1cccc(Cl)c1)CS(=O)(=O)C2	0	Test
TNKS1_ML_4227	O=C([O-])/C(=C\c1ccc(-c2ccccc2Cl)o1)c1ccc(S(=O)(=O)N2CCOCC2)s1	0	Test
TNKS1_ML_4228	C[C@]12CCC[C@]3(CO3)[C@@H]1C[C@@H]1[C@@H](C[NH2+]Cc3ccc[nH+]c3)C(=O)O[C@@H]1C2	0	Test
TNKS1_ML_4229	Cc1nc2nc(NCc3ccc(F)cc3)nn2c([O-])c1Cl	0	Test
TNKS1_ML_4230	CC(C)(O)c1ccc(-c2cn3c(F)ccc3c(=O)[nH]2)cc1	1	Test
TNKS1_ML_4231	O=C(CSc1ncnc2nc(N3CCOCC3)sc12)Nc1ccc2c(c1)CCC2	0	Test
TNKS1_ML_4232	O=C([O-])C1([C@@H]2CCCCC[C@@H]2O)CCCCC1	0	Test
TNKS1_ML_4233	[H]/N=C1\NC[C@]2(CCCOC2)N1c1ccc(Cl)cc1	0	Test
TNKS1_ML_4234	Cc1ccc(N([C@H](C)C(=O)Nc2ccc(S(=O)(=O)N3CCOCC3)cc2)S(C)(=O)=O)cc1	0	Test
TNKS1_ML_4235	Cc1cccc(N2C[C@H](C(=O)Nc3ccc(S(=O)(=O)Nc4ncccn4)cc3)CC2=O)c1C	0	Test
TNKS1_ML_4236	CC1(C)OC(=O)N(c2ccc(-c3cnc(N)c(-c4ncc(F)cn4)c3)cc2)[C@H]1c1ccccc1	1	Test
TNKS1_ML_4237	Cc1cc(C)cc(C(=O)Nc2ccc(-c3ccc(=O)n(C)n3)cc2)c1	0	Test
TNKS1_ML_4238	Cc1c(F)ccc2c1c(=O)n(C[C@H]1CC[C@H](C(=O)O)CC1)c(=O)n2Cc1cccc(C(F)(F)F)c1C#N	1	Test
TNKS1_ML_4239	C[C@H]1CC[C@@H](C)CN1C(=O)c1cc(N)nc(Cl)c1	0	Test
TNKS1_ML_4240	[H]/N=C1C(=C/c2ccc(OCCOc3ccccc3CC=C)c(OCC)c2)/C(=O)N=C2SC(C(C)C)=NN2/1	0	Test
TNKS1_ML_4241	O=S(=O)(c1ccc(Cl)s1)N1CC[NH2+]CC12CCCCC2	0	Test
TNKS1_ML_4242	CNc1cc(Nc2nccc([C@H]3CCCN(Cc4ccc[nH+]c4NC)C3)n2)ncn1	0	Test
TNKS1_ML_4243	N#Cc1ccc(CN(Cc2nc3c(c(=O)[nH]2)COCC3)C(=O)CN2CCC(C(=O)c3ccc(F)cc3)CC2)s1	1	Test

TNKS1_ML_4244	CC1(C)C[C@@H]2C[C@@](C)(CN2C(=O)[C@]2(C)COC[C@@H]2[NH3+])C1	0	Test
TNKS1_ML_4245	C/C(=C/c1ccco1)CN1CCc2[nH]nc(-c3ccc4c(c3)OCO4)c2C1	0	Test
TNKS1_ML_4246	Cc1nn(-c2ccccc2)c(C)c1NC(=O)CCc1nc2cc(S(=O)(=O)N(C)C)ccc2n1C	0	Test
TNKS1_ML_4247	COc1ccc(C(=O)N[C@@H](C)c2ccccc2NS(C)(=O)=O)cc1S(=O)(=O)N(C)C	0	Test
TNKS1_ML_4248	O=C(Cc1ccccc1)Nc1nnc2n1CCS2	1	Test
TNKS1_ML_4249	Cc1ccc(NC(=O)CN2C(=O)C(C)(C)c3cc(S(=O)(=O)N4CCOCC4)ccc32)cc1Cl	0	Test
TNKS1_ML_4250	CC(=O)O[C@H]1[C@H]([NH+]2CCOCC2)c2c(ccc(/C=C\C(=O)N3CCOCC3)c2O)OC1(C)C	0	Test
TNKS1_ML_4251	COc1ccccc1N1CCN(c2nc3c(c(=O)[nH]2)CC(F)(F)CC3)CC1	1	Test
TNKS1_ML_4252	Cc1ccc([N+](=O)[O-])cc1Oc1ccc([C@H](C)N)cc1Br	0	Test
TNKS1_ML_4253	O=C(Nc1ccc(N2CCCN(C(=O)c3ccccc3)CC2)[nH+]c1)C1CCCCC1	0	Test
TNKS1_ML_4254	CCCc1nn(C)c2c1[nH]c(=S)n2[C@@H]1CCCCC1(C)C	0	Test
TNKS1_ML_4255	Nc1c(C2CC2)nc2c(Br)cccn12	0	Test
TNKS1_ML_4256	Cc1cc(C)cc(NS(=O)(=O)c2cc(-c3noc(C(=O)N4CCCC4)n3)sc2C)c1	0	Test
TNKS1_ML_4257	C/C(=N/N=C1\NC(=O)C[C@@H](C(=O)Nc2cccc(Cl)c2)S1)c1cccs1	0	Test
TNKS1_ML_4258	NC(=O)c1cccc2c1O/C(=C\c1ccc(C(=O)NCCc3nc4c(C(N)=O)cccc4[nH]3)cc1)C2=O	1	Test
TNKS1_ML_4259	O=C(N[C@H]1C[C@H](c2nnc(-c3ccc(OC(F)F)cn3)n2-c2ccccc2F)C1)c1cc(F)cc2nccnc12	1	Test
TNKS1_ML_4260	CC(C)(C)OC(=O)NCCC(=O)/N=C1/S[C@H]2CS(=O)(=O)C[C@H]2N1c1cc(Cl)ccc1Cl	0	Test
TNKS1_ML_4261	O=C(CN1CCC(C(=O)N2CCc3c(Cl)cccc3C2)CC1)NCc1nc2c(c(=O)[nH]1)COCC2	1	Test
TNKS1_ML_4262	Cc1cc(S(=O)(=O)N2C[C@H](C)CC[C@@H]2C)sc1C[NH3+]	0	Test
TNKS1_ML_4263	Cc1cc(C)nc(NS(=O)(=O)c2ccc(/N=C/c3c(O)ccc4ccccc34)cc2)n1	0	Test
TNKS1_ML_4264	COc1ccc(-c2noc(CSc3nnc(-c4ccncc4)n3-c3ccccc3)n2)cc1	1	Test
TNKS1_ML_4265	CCOc1cc([C@H]2C(C(=O)c3ccc(C)o3)=C(O)C(=O)N2CC[NH+](C)C)ccc1OCc1ccccc1	0	Test
TNKS1_ML_4266	Cc1cc(NC(=O)[C@H](C)OC(=O)CCNS(=O)(=O)c2c(Cl)cccc2Cl)no1	0	Test
TNKS1_ML_4267	CC(C)(CO)NC(=O)[C@@H]1CCCC[C@@H]1[NH3+]	0	Test
TNKS1_ML_4268	COc1cc(C[NH3+])cc(Br)c1O[C@H]1CCCC[C@H]1O	0	Test
TNKS1_ML_4269	O=C(CCCN1C(=O)c2ccccc2C1=O)NCCS(=O)(=O)N1CCN(Cc2ccccc2)CC1	0	Test
TNKS1_ML_4270	CCc1c(C)nc(N2CCN(c3ccccc3F)CC2)nc1O	1	Test
TNKS1_ML_4271	COc1ccc(F)cc1NC(=O)N[C@H]1CCOC2(CCCC2)C1	0	Test
TNKS1_ML_4272	CC(=O)c1oc2ccccc2c1NC(=O)[C@@H](C)N1CCN(Cc2cn3ccccc3[nH+]2)CC1	0	Test
TNKS1_ML_4273	CC(=O)N[C@H]1[C@H](O)[C@@H]2OC(C)(C)OC[C@@H]2O[C@@H]1Oc1ccc(C)cc1	0	Test
TNKS1_ML_4274	Cc1ccc(S(=O)(=O)NC2CC2)cc1C(=O)N[C@@H](c1ccccc1)c1[nH+]ccn1C	0	Test
TNKS1_ML_4275	O=C(N[C@H]1CCC[C@H]1C(=O)[O-])c1csc2ccccc12	0	Test
TNKS1_ML_4276	O=C(Nc1nc2ccc(F)cc2s1)[C@@H]1CCCN(S(=O)(=O)c2ccc(Cl)cc2)C1	0	Test
TNKS1_ML_4277	CCOC(=O)C1=C(CN2CCN(C(=O)C(C)C)CC2)N(C)C(=O)N[C@@H]1c1ccccc1Cl	0	Test
TNKS1_ML_4278	CCS(=O)(=O)N1CCc2ccc(S(=O)(=O)NCCc3ccccn3)cc2C1	0	Test

TNKS1_ML_4279	CCN1C(=O)/C(=C/c2c(F)cccc2Cl)S/C1=N\c1nnc(C)s1	0	Test
TNKS1_ML_4280	COc1ccc2cc(C(=O)N3CCc4nc(NC(=O)C5CC5)sc4C3)[nH]c2c1	0	Test
TNKS1_ML_4281	N#Cc1ccc(Cl)cc1NC(=O)c1sccc1C#CCO	0	Test
TNKS1_ML_4282	COc1ccc([C@H](NC(=O)N2CCO[C@@H](C)[C@H]2C)C2CCOCC2)cc1	0	Test
TNKS1_ML_4283	Nc1ncc(F)c(-c2ccc(Cl)cc2)n1	0	Test
TNKS1_ML_4284	CC1(C)CCN(c2ccc(Cl)cc2C#N)C1	0	Test
TNKS1_ML_4285	Cc1noc2nc(-c3ccccc3)cc(C(=O)N[C@@H]3CC[C@H]([NH+](C)C)C3)c12	0	Test
TNKS1_ML_4286	C[C@@H]1[C@@H](O)CC[C@H]1Cc1nn(C)c2ccccc12	0	Test
TNKS1_ML_4287	Cc1cccc(Nc2nnc(SCCCn3c(=O)c4c(ncn4C)n(C)c3=O)s2)c1	0	Test
TNKS1_ML_4288	C[C@@H]1CCCC[C@@H]1Nc1nsnc1Cl	0	Test
TNKS1_ML_4289	CC1(C)[C@H]2CC=C(/C=N\N3CCCCC3)[C@@H]1C2	0	Test
TNKS1_ML_4290	C[C@H]1C[C@H](C)C[NH+](Cc2[nH+]c3c(c(=O)[nH]c(=O)n3C)n2Cc2ccc(Cl)c(Cl)c2)C1	0	Test
TNKS1_ML_4291	CCc1c(C(=O)[O-])nnn1-c1ccc(C(C)C)cc1	0	Test
TNKS1_ML_4292	Cc1nc([S-])sc1CC(=O)Nc1ccc(Br)cc1	0	Test
TNKS1_ML_4293	Cc1ccc2[nH]c3nc(SC(C)C)nnc3c2c1	0	Test
TNKS1_ML_4294	CN1CCCc2c1nc(N1CCC3(CC1)C(=O)N(CC(O)CO)c1cc(F)cc(F)c13)[nH]c2=O	1	Test
TNKS1_ML_4295	C[C@H]1c2ccsc2CCN1c1nc2c(s1)CCC[C@@H]2O	0	Test
TNKS1_ML_4296	CN1CCCc2c1nc(N1CCC3(CC1)C(=O)N(CCO)c1cc(F)cc(F)c13)[nH]c2=O	1	Test
TNKS1_ML_4297	O=c1[nH]c(-c2ccc(F)cc2)nc2c(O)cccc12	1	Test
TNKS1_ML_4298	Oc1nc(-c2ccccc2)nc2c(O)cccc12	1	Test
TNKS1_ML_4299	CC(=O)c1ccc(-n2c(C)cc(/C=C3/C(=O)NC(=O)N(c4ccc(C)cc4)C3=O)c2C)cc1	0	Test
TNKS1_ML_4300	Cc1nnsc1C(=O)Nc1sc2c(c1C(=O)NCc1cccnc1)CCCC2	0	Test
TNKS1_ML_4301	Cc1nn(C)c(C)c1NCc1c(C)nc2ccccn12	0	Test
TNKS1_ML_4302	C[NH+]1CCN(c2ccc(-c3ccc(NS(=O)(=O)c4ccc(Br)cc4)cc3)nn2)CC1	0	Test
TNKS1_ML_4303	Cc1ccc([C@@]2(O)CC[C@@H]3C[NH+](Cc4ccc5c(c4)C[C@H](C)O5)C[C@@H]32)nc1	0	Test
TNKS1_ML_4304	CCc1nc([C@H]2COc3ccccc3O2)nc(NC)c1I	0	Test
TNKS1_ML_4305	Cc1cc(/C=C2/C(=[NH2+])N3C(=NC2=O)SN=C3S(=O)(=O)CC(C)C)c(C)n1-c1cccc(F)c1	0	Test
TNKS1_ML_4306	CN1CCCc2c1nc(N1CCC3(CC1)C(=O)N(C)c1cc(OCCN4CCCC4)cc(F)c13)[nH]c2=O	1	Test
TNKS1_ML_4307	CN1CCc2nc(NCc3ccco3)ncc2C1	0	Test
TNKS1_ML_4308	Cc1cccc2c(=O)[nH]c3c(c12)COC(C(C)C)C3	1	Test
TNKS1_ML_4309	CCOC(=O)CCc1cc2c(C)cc(=O)oc2c(C[NH+]2C[C@@H]3C[C@@H](Cn4c3cccc4=O)C2)c1O	0	Test
TNKS1_ML_4310	C[C@H]1[NH2+]CCN(C(=O)OC(C)(C)C)[C@@H]1C	0	Test
TNKS1_ML_4311	Cc1nc2c(Cl)cc(Cl)cn2c1N	0	Test
TNKS1_ML_4312	O=C(N[C@H]1CCC[C@H](C(F)(F)F)C1)N1CCC2(CC1)OCCO2	0	Test
TNKS1_ML_4313	Cc1cc(C)c2c(n1)SCCN2C(=O)Nc1cccc(C(=O)NCc2cccs2)c1	0	Test
TNKS1_ML_4314	O=C(CCc1nc2ccccc2c(=O)[nH]1)Nc1ccc(-c2nnc(-c3ccccc3)o2)cc1	1	Test
TNKS1_ML_4315	COCCN1Cc2cccc(C(=O)N[C@H](C)c3ccc4c(c3)OCCO4)c2C1=O	0	Test
TNKS1_ML_4316	COc1ccc(C(=O)C2CCN([C@H]3CCN(Cc4nc5c(c(=O)[nH]4)COCC5)C3=O)CC2)cc1C	1	Test

TNKS1_ML_4317	[H]/N=c1/c(S(=O)(=O)c2ccc(Br)cc2)cc2c(=O)n3cccc(C)c3nc2n1CCc1ccc(OC)c(OC)c1	0	Test
TNKS1_ML_4318	N#C/C(c1nnc2n1CCCCC2)=c1/s/c(=C\c2ccccc2[N+](=O)[O-])c(=O)n1-c1ccccc1	0	Test
TNKS1_ML_4319	C[C@H]1CC[NH+]1c1cc(F)c(F)cc1C(=O)[O-]	0	Test
TNKS1_ML_4320	CC(=O)Nc1ccc(/C=C\C(=O)NS(=O)(=O)c2ccc3c(c2)CCCC3)cc1	0	Test
TNKS1_ML_4321	Cc1cccc(-c2nnc([C@H]3C[C@H](NC(=O)c4ccccn4)C3)n2-c2ccccc2F)n1	1	Test
TNKS1_ML_4322	Cn1c(=O)c2c(ncn2C[C@H](O)CN2CCN(Cc3ccccc3Cl)CC2)n(C)c1=O	0	Test
TNKS1_ML_4323	COCCNC(=O)[C@H](c1ccc[nH+]c1)N(Cc1ccccc1Cl)C(=O)Cn1nnc2ccccc21	0	Test
TNKS1_ML_4324	Nc1ccc(Cl)c(C[NH+]2CCN(c3nccs3)CC2)n1	0	Test
TNKS1_ML_4325	O=C(Nc1cc[nH]n1)c1cc(F)cc(Br)c1	0	Test
TNKS1_ML_4326	CCOC(=O)C1=c2s/c(=C\c3ccncc3)c(=O)n2C(N)=C(C(=O)OC)[C@H]1c1ccncc1	0	Test
TNKS1_ML_4327	CNC(=O)NCC(=O)N[C@@H]1CCc2cc(Cl)ccc21	0	Test
TNKS1_ML_4328	Cc1c(C(c2cc3c(cc2Br)OCO3)c2c(C)n(C)n(-c3ccccc3)c2=O)c(=O)n(-c2ccccc2)n1C	0	Test
TNKS1_ML_4329	N#CC(/C([O-])=N/c1ccccc1)=C1/[NH+]=C(N)c2ccccc21	0	Test
TNKS1_ML_4330	[H]/N=C1/NC[C@@H](C2CCOCC2)N1c1ccc(Cl)c(Br)c1	0	Test
TNKS1_ML_4331	CS(=O)(=O)[C@H]1CCC[C@@H]1Nc1ccc2c(c1)OCO2	0	Test
TNKS1_ML_4332	CCc1nnc(S)n1/N=C/c1ccc(O)c(O)c1	0	Test
TNKS1_ML_4333	CC(=O)O[C@H]1[C@@H](C)[C@H]2CC[C@@]13CC=C(C)C[C@H]23	0	Test
TNKS1_ML_4334	COCCOC(=O)C1=C(C)N=C2SCC(CC(=O)NC(C)C)=[N+]2[C@@H]1c1cccc(Oc2ccccc2)c1	0	Test
TNKS1_ML_4335	CC(=O)c1ccc(NC(=O)Cn2c(=O)n(-c3cccc(Cl)c3)c(=O)c3c4c(sc32)COC(C)(C)C4)cc1	0	Test
TNKS1_ML_4336	Cc1cc(N2CCO[C@H](CO)C2)n2ncnc2n1	0	Test
TNKS1_ML_4337	CC1(C)C[C@@H](Nc2ccc3c(c2)OCO3)CCO1	0	Test
TNKS1_ML_4338	Cc1nn(C)c(C)c1N[C@H]1CCCC1(C)C	0	Test
TNKS1_ML_4339	Cc1ccccc1-n1ncc2c(O)nc(SCC(=O)N3CCN(c4ccccc4)CC3)nc21	0	Test
TNKS1_ML_4340	O=[N+]([O-])c1cc(S(=O)(=O)N2CCOCC2)ccc1NCc1cccc(C(F)(F)F)c1	0	Test
TNKS1_ML_4341	Cc1ccc(C(=O)Nc2nn3c(-c4ccccc4)nnc3s2)cc1	0	Test
TNKS1_ML_4342	Cc1c(C(=O)OCC(=O)Nc2sc3c(c2C(N)=O)CCC3)oc2ccc(Br)cc12	0	Test
TNKS1_ML_4343	COc1ccc(-n2nc3c(c2NC(=O)C(=O)NCCCc2ccccc2)CS(=O)(=O)C3)cc1	0	Test
TNKS1_ML_4344	O=C(NC[C@@H](c1ccccc1Cl)[NH+]1CCOCC1)c1ccc(S(=O)(=O)c2ccccc2)o1	0	Test
TNKS1_ML_4345	CC1(C)CCC(NC(=O)N2CCC[C@@](C)(C(=O)[O-])C2)CC1	0	Test
TNKS1_ML_4346	Cc1nc(Cn2cc(-c3nc(C(=O)NCCc4ccncc4)c4ccccn34)[nH+]c2C)cs1	0	Test
TNKS1_ML_4347	C[C@@H](c1ccc(Cl)cc1)N(C)c1ncc(CN)cc1Cl	0	Test
TNKS1_ML_4348	CN(CC(=O)Nc1cccc2ccccc12)C(=O)c1cc(S(=O)(=O)N2CCOCC2)c[nH]1	0	Test
TNKS1_ML_4349	[H]/N=C1\[C@H](C2CC2)[NH+]=C(C2CC2)N1CC#C	0	Test
TNKS1_ML_4350	CC(=O)c1sc(N2C(=O)C([O-])=C(C(=O)c3sc(C)nc3C)[C@@H]2c2ccc(C(C)C)cc2)nc1C	0	Test
TNKS1_ML_4351	CCCn1nc(C(=O)N2CCOCC2)c2c1CC[C@@H]([NH2+]CC1(N3CCOCC3)CCCC1)C2	0	Test
TNKS1_ML_4352	COc1cc(/C=C2\C(=O)N(c3cccc(C(F)(F)F)c3)N=C2C)ccc1O	0	Test

	CC(N)=O		
TNKS1_ML_4353	COC(=O)[C@@H]1O[C@H](OCC(=O)[C@H]2CC[C@@H]3[C@@H]4CC[C@@H]5C[C@H](O)CC[C@]5(C)[C@H]4C(=O)C[C@@]23C)[C@@H](O)[C@H](O)[C@H]1O	0	Test
TNKS1_ML_4354	C[C@H]1C[C@@H]1C(=O)NC1(C#N)CCCCCC1	0	Test
TNKS1_ML_4355	Cc1nn(CCCC(=O)NCc2ccc(Cl)cc2)c(=O)c2cc3occc3n12	0	Test
TNKS1_ML_4356	Cc1nc(Cl)cc(O[C@H]2CCCC[C@@H]2C)n1	0	Test
TNKS1_ML_4357	CCc1ccc(S(=O)(=O)Nc2cc(-c3ccco3)nn2[C@@H]2CCS(=O)(=O)C2)cc1	0	Test
TNKS1_ML_4358	Cc1ccc(Cl)cc1S(=O)(=O)/N=c1/cc(Cl)cc[nH]1	0	Test
TNKS1_ML_4359	CCS[C@H]1CC(=O)N(c2ccc(S(=O)(=O)Nc3nnc(CC)s3)cc2)C1=O	0	Test
TNKS1_ML_4360	Cc1cc/c(=N/S(=O)(=O)c2cc(-c3nc(C)c(C(=O)N4CCCCCC4)s3)n(C)c2C)[nH]c1	0	Test
TNKS1_ML_4361	C[C@]1(C(=O)[O-])CCC[C@@]2(C)[C@H]3CC[C@H]4C[C@]3(CC[C@@H]12)C[C@H]4CO	0	Test
TNKS1_ML_4362	CC1=C[C@H]([C@H]2N=N[C@H](S)N2/N=C/c2cc(Br)cc(Br)c2[O-])N=N1	0	Test

Table S8. Complete machine learning dataset used for model development and evaluation. The dataset includes the curated experimentally validated TNKS1 active compounds and the selected property-matched DUD-E decoys, together with the corresponding assignment of each compound to the training, validation, or independent test set.

SMILES	***docking score***	***glide ecoul***	***glide emodel***	***glide energy***	***glide evdw***	***glide hbond***	***glide ligand efficiency***	***glide lipo***	***glide rewards***

COc1cccc(-c2ccc(C(=O)[O-])c(=O)[nH]2)c1	-10.573	-8.795	-77.857	-46.696	-37.901	-733	-589	-3.488	-3.377
COc1ccc(-c2ccc(C(=O)[O-])c(=O)[nH]2)cc1	-10.554	-9.483	-80.5	-46.539	-37.056	-766	-587	-3.395	-3.326
O=C([O-])c1ccc(-c2ccc(Cl)cc2)[nH]c1=O	-10.411	-9.428	-81.492	-46.758	-37.33	-793	-626	-3.411	-3.355
O=C([O-])c1ccc(-c2ccccc2)[nH]c1=O	-10.234	-9.190	-75.303	-43.998	-34.808	-754	-653	-3.261	-3.575
O=c1cc(-c2ccccc2)oc2ccccc12	-10.021	-7.422	-74.752	-46.732	-39.31	-0.52	-608	-4.544	-2.527
O=C(c1ccc2c(c1)OCCO2)N1CCc2c(nc(N3CCCC3)[nH]c2=O)C1	-10.014	-5.747	-80.214	-60.243	-54.496	-128	-358	-4.277	-2.421
O=C(CN1CCC(Nc2nc3c(c(=O)[nH]2)CCC3)CC1)Nc1ccccc1C(F)(F)F	-10.001	-12.239	-87.416	-59.323	-47.084	-696	-323	-3.276	-2.386
CC(=O)c1ccc(NC(=O)[C@@H](C)Sc2nc3c(c(=O)[nH]2)CCCC3)cc1	-9.976	-2.890	-80.616	-55.386	-52.496	-197	-384	-4.818	-2.461
O=C(Cc1n[nH]c(=O)c2ccccc12)N1CCC(c2[nH]ncc2-c2ccncc2)CC1	-9.953	-4.820	-88.095	-56.591	-51.771	-486	-321	-4.521	-2.58
COc1ccc(CCNC(=O)[C@H]2CCCC[C@@H]2c2nc3ccccc3c(=O)[nH]2)cc1	-9.915	-6.025	-75.692	-57.181	-51.156	-449	-0.33	-4.419	-2.268
O=C(NCc1ccccc1)N1CCc2nc(N3CCCC3)[nH]c(=O)c2C1	-9.906	-6.632	-74.288	-47.478	-40.846	-0.32	-381	-3.683	-3.421
O=c1nc(-c2cccnc2)[nH]c2c1CCN(Cc1ccc(C(F)(F)F)cc1)C2	-9.856	2.492	-77.566	-50.47	-52.963	0.0	-352	-5.239	-2.734
O=C(CN1CCc2nc(N3CCCC3)[nH]c(=O)c2C1)N1CCCCC1	-9.849	-9.028	-72.291	-51.65	-42.622	-529	-394	-3.138	-3.326
O=C(c1nc2ccccc2s1)C1CCN(C(=O)Cc2n[nH]c(=O)c3ccccc23)CC1	-9.833	-3.863	-88.654	-58.114	-54.251	-481	-317	-4.444	-2.143
CC(C)n1cc(CN2CCc3c(nc(N4CCCC4)[nH]c3=O)C2)c2ccccc21	-9.800	-5.874	-65.025	-42.59	-36.716	-0.32	-338	-5.052	-2.555
O=c1[n-]c(-c2ccccn2)nc2c1CC[N@H+](Cc1cccc(Cl)c1)C2	-9.766	-2.072	-75.094	-48.19	-46.118	0.0	-391	-5.224	-2.55
O=C(Cn1nnc2ccccc21)N1CCc2c(nc(N3CCCCC3)[nH]c2=O)C1	-9.740	-5.627	-93.662	-61.942	-56.315	0.0	-336	-3.935	-2.65
COc1ccc([C@]2(O)CC[N@H+](CC(=O)Nc3ccccc3C(N)=O)CC2)cc1	-9.700	-12.097	-74.891	-54.953	-42.855	-0.89	-346	-3.828	-2.197
COc1cccc(NC(=O)N(Cc2ccccc2)Cc2nc3ccccc3c(=O)[nH]2)c1	-9.697	-4.608	-85.869	-59.749	-55.141	-425	-313	-4.290	-2.076
O=c1cc(-c2ccc(Cl)cc2)nc(N2CCN(c3ccccc3F)CC2)[nH]1	-9.630	-1.763	-79.274	-52.927	-51.164	0.0	-357	-4.713	-2.488
O=C(CN1CCc2nc(N3CCCC3)[nH]c(=O)c2C1)N1CCCCCC1	-9.606	-4.518	-65.757	-47.997	-43.48	-282	-369	-3.692	-3.404
CC(=O)c1cccc(NC(=O)N2CCc3nc(N4CCCC4)[nH]c(=O)c3C2)c1	-9.586	-5.623	-70.665	-45.499	-39.876	-0.52	-342	-3.820	-2.941
O=C(N1CCOCC1)N1CCc2nc(N3CCOCC3)[nH]c(=O)c2C1	-9.578	-2.938	-75.409	-49.943	-47.005	-0.32	-383	-3.013	-3.893
COc1cccc(C[NH2+]Cc2cc3c(C)cc(C)cc3[nH]c2=O)c1	-9.563	-3.557	-72.413	-51.178	-47.621	-0.32	-398	-4.823	-2.387
O=c1[n-]c(N2CCOCC2)nc2c1CC[N@H+](CC1CCCCC1)C2	-9.530	-781	-58.372	-42.235	-41.454	0.0	-397	-4.857	-2.994

C[C@H]1CC[C@H]([N@H+]2CCc3nc(-c4ccccc4)[nH]c(=O)c3C2)CC1	-9.510	-5.716	-63.669	-45.435	-39.719	-0.32	-396	-4.146	-2.494
O=c1[nH]c(-c2ccncc2)nc2c1CCN(Cc1cnc(N3CCCCC3)nc1)C2	-9.498	-5.880	-66.539	-47.511	-41.631	-0.55	-317	-3.105	-3.622
COc1ccccc1CNCc1cc2c(C)cc(C)cc2[nH]c1=O	-9.466	-3.665	-71.858	-51.389	-47.724	-0.32	-394	-4.632	-2.424
O=C(C1CCCC1)N1CCc2nc(-c3ccccc3)[nH]c(=O)c2C1	-9.438	-3.218	-65.843	-44.263	-41.045	-0.32	-393	-3.511	-3.590
O=C1N[C@]2(CC[N@@H+](Cc3ccccc3)CC2)Nc2ccccc21	-9.414	-4.181	-66.157	-46.015	-41.834	-581	-409	-3.742	-3.271
Cc1ccccc1NC(=O)CSc1nnc(-c2ccccn2)n1-c1ccccc1Cl	-9.401	-5.150	-82.630	-56.420	-51.27	-525	-313	-3.034	-3.240
O=C(Nc1ccccc1C(F)(F)F)N1CCc2nc(N3CCOCC3)[nH]c(=O)c2C1	-9.375	-1.892	-65.647	-42.144	-40.252	-0.32	-312	-3.978	-3.167
O=c1cc(-c2ccccc2)c2cc3c(cc2[nH]1)OCCO3	-9.369	-6.008	-58.753	-39.723	-33.715	-0.32	-446	-3.831	-2.794
Cc1cccc(C[N@H+]2CCc3c(nc(N4CCC(C)CC4)[nH]c3=O)C2)c1	-9.357	-2.873	-50.033	-38.816	-35.943	0.0	-0.36	-4.014	-3.566
COc1ccc2[nH]c(=O)c(C[NH2+]Cc3ccccc3)cc2c1	-9.341	-4.799	-70.314	-47.362	-42.563	-125	-425	-4.524	-2.712
CC(=O)c1cccc(NC(=O)N2CCc3nc(N4CCCCC4)[nH]c(=O)c3C2)c1	-9.328	-2.512	-83.624	-56.584	-54.072	0.0	-322	-3.547	-3.200
CCOc1ccc2[nH]c(=O)c(C[NH2+]Cc3ccc(F)cc3)cc2c1	-9.317	-4.700	-69.250	-48.363	-43.664	-116	-388	-4.396	-2.866
C[C@@H]1CN(c2nc(=O)c3c([nH]2)CN(Cc2ccc(C#N)cc2)CC3)C[C@@H](C)O1	-9.307	-4.972	-81.030	-55.066	-50.094	-13	-332	-3.804	-2.644
CCOc1ccc2[nH]c(=O)c(CN(Cc3cccnc3)C(=O)c3ccccc3F)cc2c1	-9.299	-2.136	-75.882	-54.521	-52.385	-0.53	-291	-4.669	-1.695
O=C([C@@H]1CCCO1)N1CCc2nc(N3CCCC3)[nH]c(=O)c2C1	-9.289	-4.658	-68.194	-47.205	-42.547	-0.32	-404	-2.947	-3.714
CC(C)c1ccc(CN2CCc3c([nH]c(N4C[C@@H](C)O[C@H](C)C4)nc3=O)C2)cc1	-9.275	-4.832	-72.914	-52.265	-47.433	-0.08	-0.32	-4.699	-2.113
C[C@H]1CN(c2nc3c(c(=O)[nH]2)CCN(C(=O)c2ccccc2F)C3)C[C@H](C)O1	-9.245	-2.501	-59.535	-42.216	-39.715	-0.32	-0.33	-3.289	-3.619
O=C([C@@H]1CCCO1)N1CCc2nc(-c3ccccc3)[nH]c(=O)c2C1	-9.240	-4.337	-70.225	-48.530	-44.193	-0.32	-385	-3.091	-3.458
COc1ccc(CN(Cc2nc3ccccc3c(=O)[nH]2)C(=O)c2ccc(C)cc2)cc1	-9.232	-1.628	-53.784	-42.589	-40.961	-204	-298	-5.446	-1.866
CC(=O)c1cccc(NC(=O)N2CCc3nc(N4CCOCC4)[nH]c(=O)c3C2)c1	-9.222	-2.142	-82.108	-56.027	-53.885	0.0	-318	-3.547	-3.155
NC(=O)c1ccc(Oc2nccnc2[C@H]2CC[N@@H+](CC3CC3)CC2)cc1	-9.206	-6.530	-65.607	-48.671	-42.141	-458	-354	-4.464	-1.938
COc1cccc(C[NH2+]Cc2cc3c(C)ccc(C)c3[nH]c2=O)c1	-9.196	-4.173	-74.788	-51.805	-47.632	-0.24	-383	-4.798	-2.061
O=c1[n-]c(-c2ccccn2)nc2c1CC[N@H+](Cc1cccs1)C2	-9.177	1.951	-69.988	-46.196	-48.147	0.0	-399	-5.05	-2.738
CCOc1ccc(CN2CCc3c([nH]c(-c4cccnc4)nc3=O)C2)cc1	-9.152	-3.432	-66.109	-48.507	-45.076	-475	-339	-4.579	-1.914

CCN(CC)c1ccc(CN2CCc3c(nc(N4CCCCC4)[nH]c3=O)C2)c(O)c1	-9.149	-8.833	-50.331	-37.59	-28.758	-731	-305	-3.829	-2.152
COc1ccc(CN(Cc2nc3ccccc3c(=O)[nH]2)C(=O)c2cccc(OC)c2)cc1	-9.126	-6.118	-82.487	-56.742	-50.624	-449	-285	-4.411	-1.357

Table S9. Raw molecular docking data for the 52 compounds retained following structure-based virtual screening against the TNKS1 catalytic binding site. Reported parameters include docking score, Coulomb energy (Glide ecoul), Glide model energy (Glide emodel), Glide energy, van der Waals contribution (Glide evdw), hydrogen-bond contribution (Glide hbond), ligand efficiency, lipophilic contribution (Glide lipo), and Glide rewards.

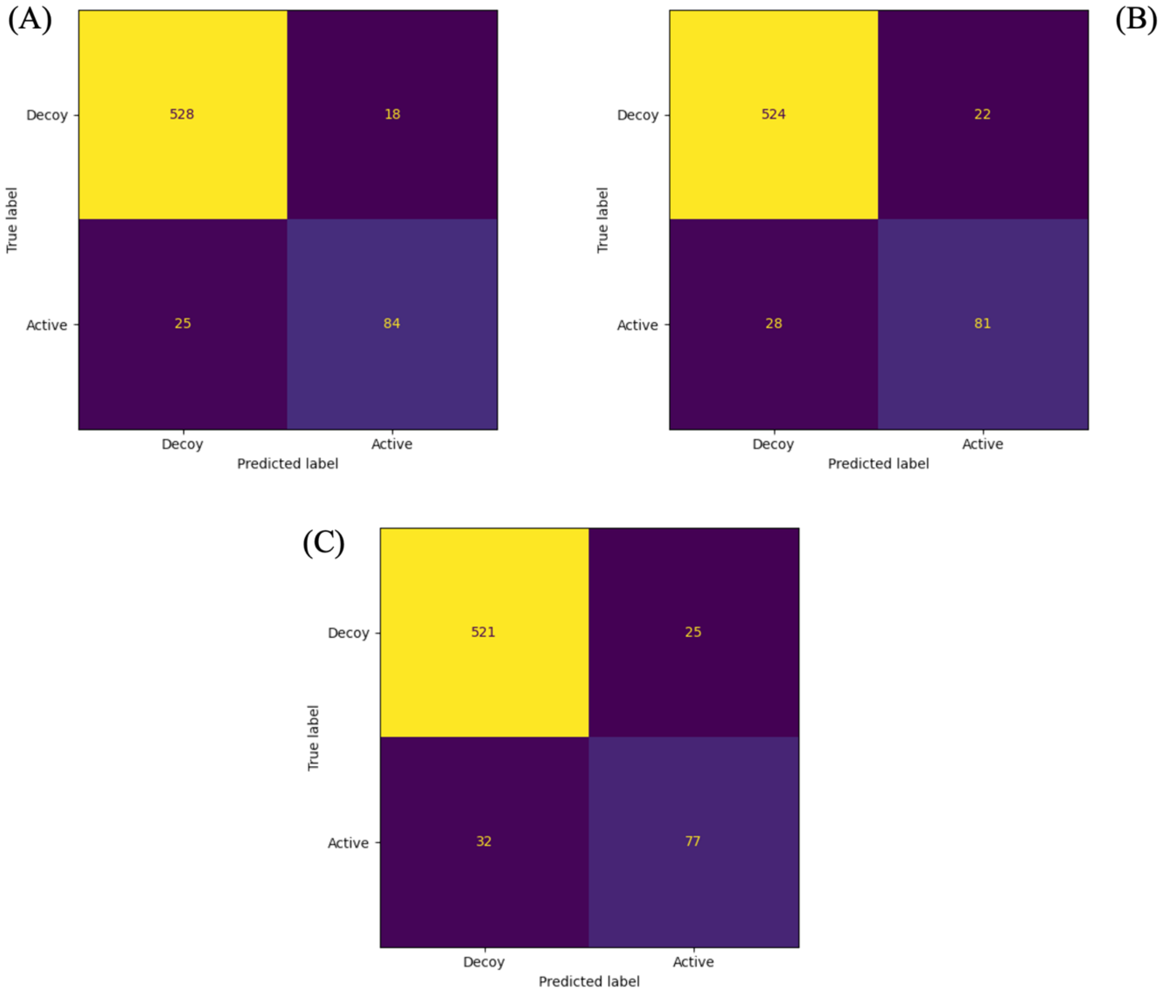

Figure S5 Confusion matrices of the machine-learning classifiers on the independent test set. Confusion matrices obtained for (A) XGBoost, (B) SVM-RBF, and (C) Logistic Regression. Model performance was evaluated on the independent test set comprising 655 compounds, including 109 experimentally validated TNKS1 inhibitors (actives) and 546 decoys. Rows indicate the true class labels, whereas columns indicate the predicted class labels.

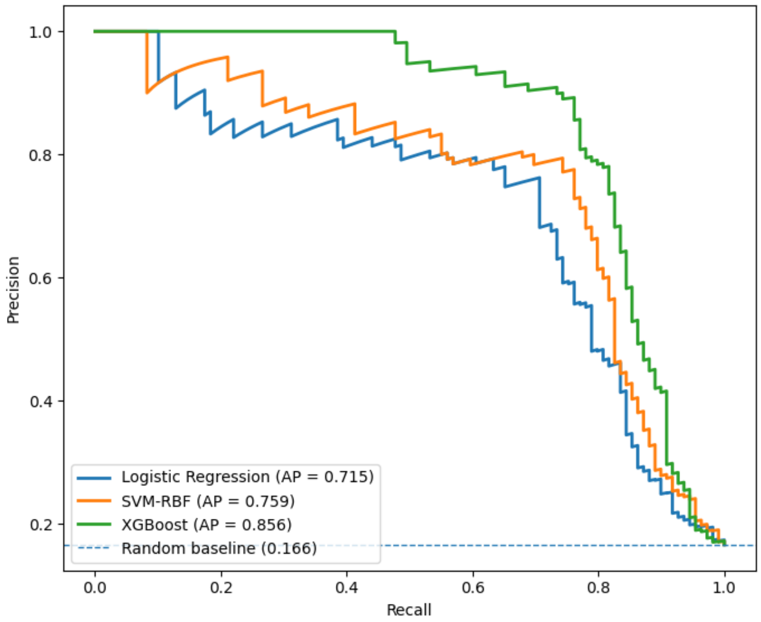

Figure S6. Precision–recall curves of the machine-learning classifiers on the independent test set. Precision–recall curves for Logistic Regression, SVM-RBF, and XGBoost evaluated on the independent test set comprising 655 compounds (109 experimentally validated TNKS1 inhibitors and 546 decoys). Average precision (AP) values are reported in the legend for each classifier. The dashed horizontal line indicates the random-classification baseline corresponding to the prevalence of the positive class in the test set.

Compound	Reference inhibitor	2D structure	Canonical SMILES	Tanimoto similarity
Compound 3	XAV939	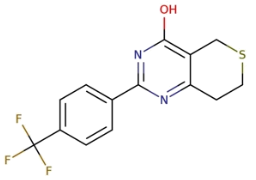	Oc1nc(-c2ccc(C(F)(F	0.067
Compound 3	IWR-1	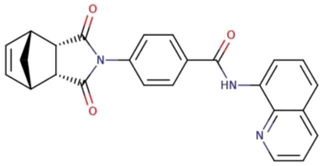	O=C(Nc1cccc2cccnc12)c1ccc(N2C(=O)[C@H]3[C@H]4C=C[C@H](C4)[C@H]3C2=O)cc1	0.087
Compound 3	JW55	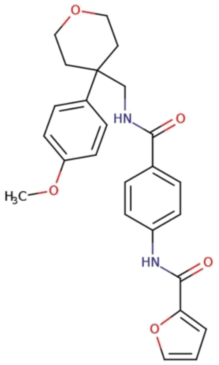	COc1ccc(C2(CNC(=O)c3ccc(NC(=O)c4ccco4)cc3)CCOCC2)cc1	0.081

Table S10. Structural similarity analysis of Compound 3 against representative tankyrase inhibitors. Structural similarity between Compound 3 and the representative tankyrase inhibitors XAV939, IWR-1, and JW55 was evaluated using Morgan fingerprints with a radius of 2 and a fingerprint size of 2048 bits. Pairwise molecular similarity was quantified using the Tanimoto coefficient. The low similarity values obtained for all three reference inhibitors indicate limited structural overlap with Compound 3.

	Replicate 1 (RLU)	Replicate 2 (RLU)	Average (RLU)
Blank	3.1×10^4	3.3×10^4	3.2×10^4
CTR +	2.5×10^6	2.2×10^6	2.35×10^6
Compound 1	1.6×10^6	1.8×10^6	1.7×10^6
Compound 2	1.4×10^6	1.7×10^6	1.55×10^6
Compound 3	5.1×10^5	4.8×10^5	4.95×10^5
Compound 4	2.3×10^6	2.5×10^6	2.4×10^6
Compound 5	2.5×10^6	2.1×10^6	2.3×10^6

Table S11 Luminescence signal (RLU) of the tested compounds. Raw RLU values obtained for individual replicates (Replicate 1 and Replicate 2) and their corresponding mean values for the blank control, positive control (CTR+), and test compounds (Compounds 1–5).
